# Modular synthesis of 3,3-diarylpropylamines *via* the photo- and copper-catalyzed 1,3-aminoarylation of cyclopropanes

**DOI:** 10.1039/d6sc06246f

**Published:** 2026-10-01

**Authors:** Carlo Baldassari, Carolin N. Veras Rodriguez, Tin V. T. Nguyen, Jerome Waser

**Affiliations:** a Laboratory of Catalysis and Organic Synthesis, Institute of Chemical Sciences and Engineering, Ecole Polytechnique Fédérale de Lausanne Ch-1015 Lausanne Switzerland jerome.waser@epfl.ch; b IBM Research Saumerstrasse 4 8803 Rüschlikon Switzerland; c NCCR Catalysis Zurich Switzerland

## Abstract

3,3-Diarylpropylamines are privileged pharmacophores in drugs such as fendiline and tolterodine, but their synthesis has been limited by the use of stepwise, single-bond-forming routes. We report the first 1,3-aminoarylation of the C–C bond in aryl cyclopropanes, achieved through two mechanistically distinct, complementary catalytic transformations. The photoactivation of *N*-chloro-*N*-(phenylsulfonyl)benzenesulfonamide (NCBSI) enables regioselective 1,3-aminochlorination; the resulting chloroamine is further elaborated by Friedel–Crafts coupling with electron-rich arenes or telescoped reductive coupling with aryl iodides to afford 1,3-aminoarylation products. A copper-catalyzed process instead couples electron-poor aryl boronic acids, *N*-fluoro-*N*-(phenylsulfonyl)benzenesulfonamide (NFSI) and aryl cyclopropanes in a single step. Collectively, these approaches give access to aryl partners spanning the full electronic spectrum, enabling the modular and convergent synthesis of structurally diverse 3,3-diarylpropylamines from a common cyclopropane precursor.

## Abstract 2

The 1,3-aminoarylation of aryl cyclopropanes is reported using photo- and copper-catalyzed reactions. Together, these complementary pathways enable modular access to pharmaceutically relevant 3,3-diarylpropylamines with diverse electronic properties.

## Graphical abstract

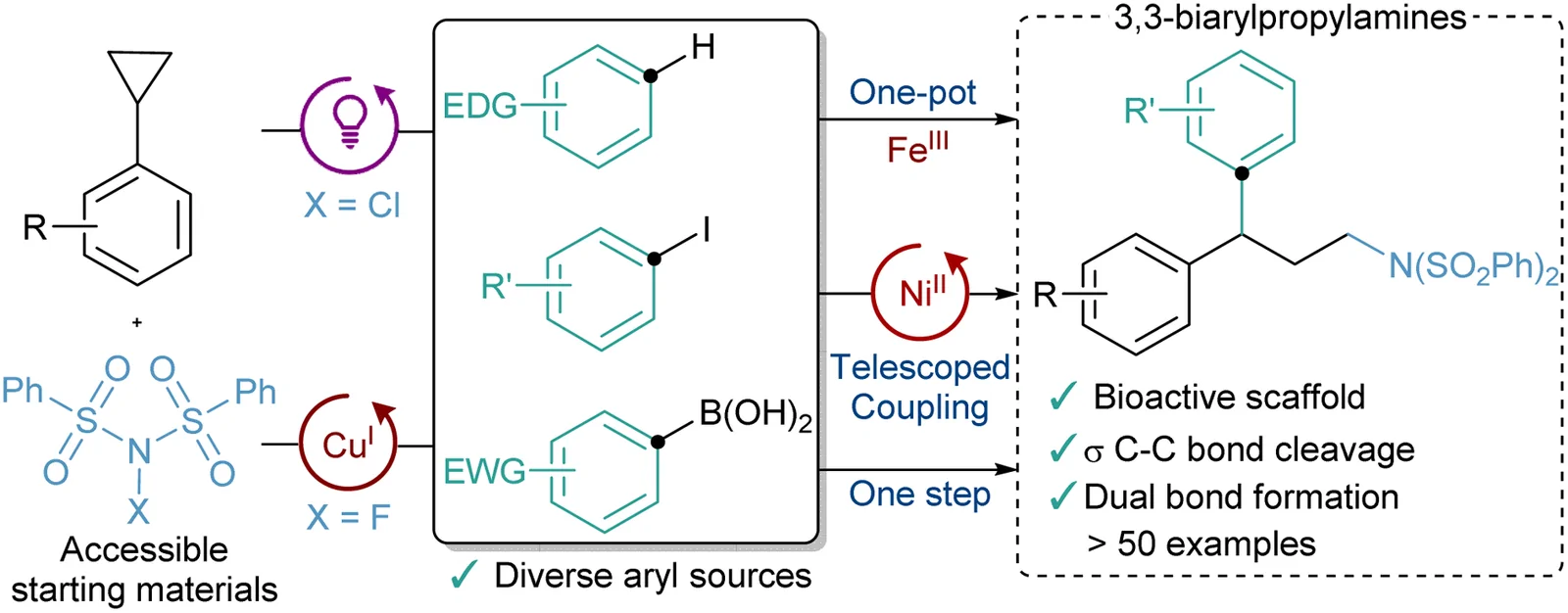

## Introduction

3,3-Diarylpropylamines constitute a broad and pharmaceutically privileged class of bioactive molecules, including drugs such as diisopromine (1), fendiline (2), the antiallergic tolpropamine (3), and the antimuscarinic Detrol (4) (Scheme 1A).^1–4^ Their synthesis has traditionally relied on stepwise strategies including cyanide or olefin reductions,^5,6^ azetidine ring opening,^7^ and base-promoted additions to 1,1-biarylethylenes,^8^ each approach forming only a single bond and requiring a relatively complex starting material. These strategies inherently limit convergency and restrict the rapid diversification of these pharmaceutically important scaffolds. Methods capable of forging multiple bonds, simultaneously or sequentially, from a common intermediate would therefore offer a more powerful entry point to structurally diverse diarylpropylamines, provided that key C–N and C–C bond-forming events could be achieved with high selectivity.

**Scheme 1:**
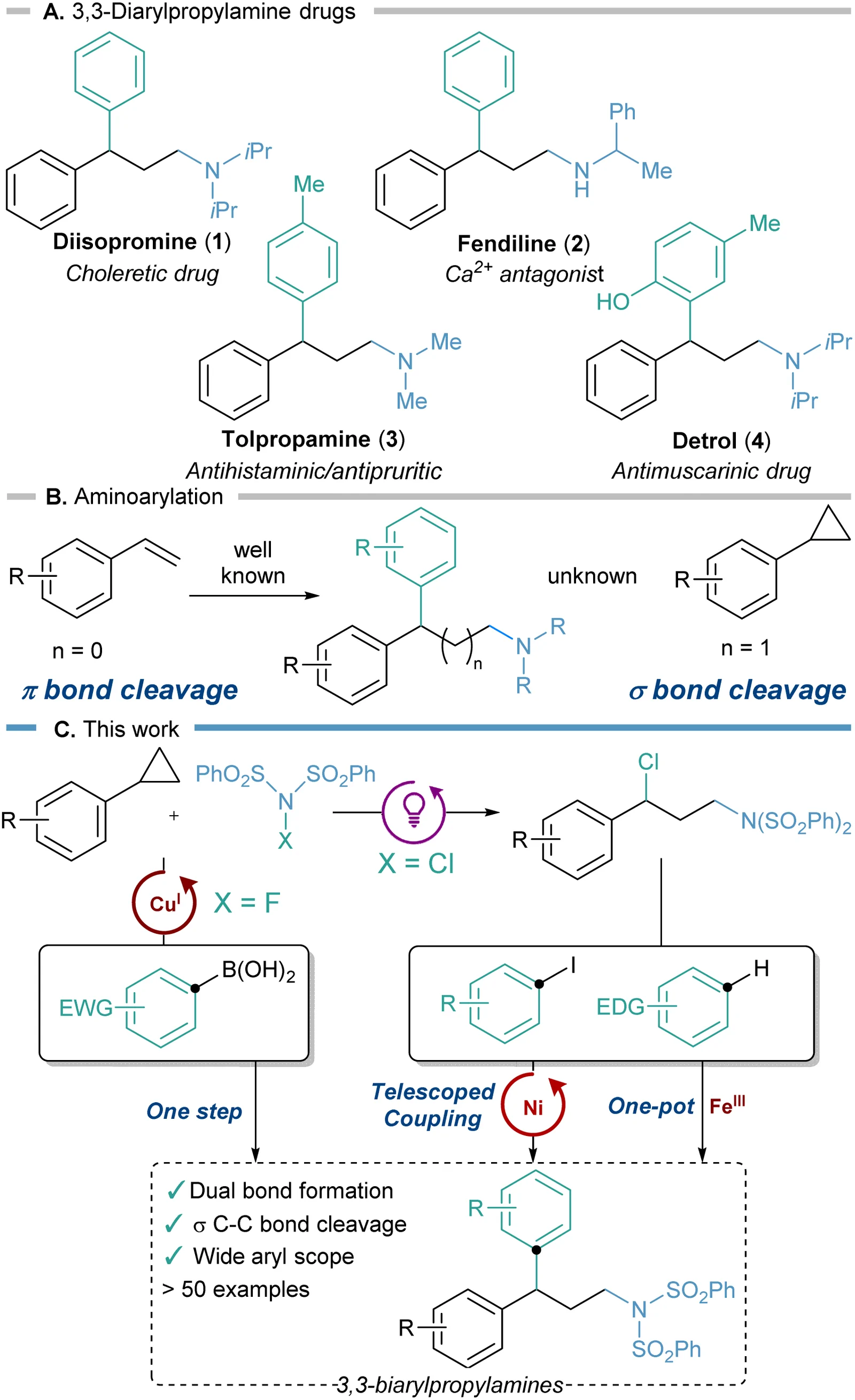
(A) Commercial drugs containing the 3,3-diarylpropylamine scaffold. (B) State of the art on aminoarylation of π and σ bonds. (C) 1,3-Aminoarylation strategies presented in the current work.

Aminoarylation reactions of alkenes that simultaneously form C–C and C–N bonds in a 1,2-relationship *via* π bond functionalization have been extensively explored employing diverse aryl and amine coupling partners (Scheme 1B).^9–17^ To complement this well-established approach, forming a C–C and a C–N bond in a 1,3-relationship would be highly attractive. This could be achieved by the more challenging cleavage of the C–C σ-bond of cyclopropanes. Although elegant strategies for unstrained C–C cleavage have been reported,^18,19^ such transformations remain generally difficult due to the high bond strength and challenging selectivity. While strain-release favours this strategy in cyclopropanes,^20,21^ its application in the context of 1,3-aminoarylation of cyclopropanes has not been reported so far.

As an approach to go beyond classical donor–acceptor cyclopropane systems,^22–24^ the oxidative C–C cleavage of aryl cyclopropanes has recently emerged as a versatile platform for constructing 1,3-bisfunctionalized 3-phenylpropane frameworks.^25–28^ Following early demonstrations under strongly oxidizing conditions,^29,30^ subsequent work extended this cleavage strategy to install heteroatom-based functional groups.^31,32^ More recently, an expanding set of oxidative methods, including inorganic oxidants,^33,34^ electrochemical activation,^35–37^ photocatalysis,^38–48^ and copper catalysis^49–56^ has considerably broadened the landscape of accessible transformations. Our group reported a photocatalytic 1,3-bischlorination of cyclopropanes *via* photolysis of Willgerodt-type reagents.^57^ While this method allowed subsequent arylation at the benzylic chloride, only chloride installation was possible at the primary position and it still required additional steps to reach diarylpropylamine targets. Therefore, despite this important progress, a general platform for the 1,3-aminoarylation of aryl cyclopropanes encompassing a broad range of aryl partners is still missing.

Herein, we address this gap by establishing, to our knowledge, the first examples of 1,3-aminoarylation of aryl cyclopropanes. Photochemical and copper-mediated catalysis operate as complementary, mechanistically distinct approaches that together accommodate three fundamentally different classes of aryl partners (electron-rich arenes, aryl iodides and electron-poor aryl boronic acids) to collectively allow the synthesis of 3,3-diarylpropylamines with a broad arene scope from simple and readily accessible starting materials (Scheme 1C). In the first approach, fully regioselective 1,3-aminochlorination was first achieved by simple but unprecedented photolysis of *N*-chloro-*N*-(phenylsulfonyl)benzenesulfonamide (NCBSI). Although the aminochlorination of olefins is well precedented,^58–60^ the corresponding 1,3-aminochlorination of cyclopropanes has not yet been realized. This intermediate functions as a common diversification handle, enabling two complementary arylation modes: a Friedel–Crafts-type coupling with electron-rich arenes and a telescoped, nickel-catalyzed reductive coupling with aryl iodides. In the second approach, we leveraged the well-established use of copper in multicomponent arylation reactions with aryl boronic acids and esters,^61–68^ to develop a complementary multicomponent copper-catalyzed 1,3-aminoarylation with aryl boronic acids proceeding in a single step. A main challenge in this case was to overcome inherently favored two-component reactions, such as arene amination and homocoupling. This could be realized successfully in the case of electron-poor boronic acids, making it complementary to the aminochlorination/Friedel–Crafts sequence.

## Results and discussion

In order to implement our Friedel–Crafts strategy, a selective 1,3-aminochlorination of aryl cyclopropanes first had to be developed. Despite its straightforward and well-established synthesis from dibenzenesulfonimide, the use of the chlorinated analogue of NFSI, NCBSI, has been mostly limited to the preparation of functionalized sulfonimide derivatives,^69,70^ with only a single report describing its application as a chlorinating reagent in the oxychlorination of alkynes.^71^ Leveraging the known lability of the N–Cl bond,^72,73^ we started our investigation by irradiating NCBSI in the presence of phenyl cyclopropane (5a) (Scheme 2). By tuning the reaction conditions (see the SI for details), after 16 h of irradiation (440 nm, 2 × 21 W lamps, and 0.05 M concentration) the 1,3-aminochlorination product was obtained in 97% isolated yield. Alkyl-substituted cyclopropanes showed no ring-opening under these photochemical conditions, in accordance with their reactivity (see SI, Section S11). Scaling up to 5 mmol produced a lower 78% yield of 6a, probably due to less efficient irradiation with higher quantities. In the dark, product 6a is not formed and partial chlorination of the aromatic ring is observed, similarly to that reported with the Willgerodt reagent.^53^

**Scheme 2:**
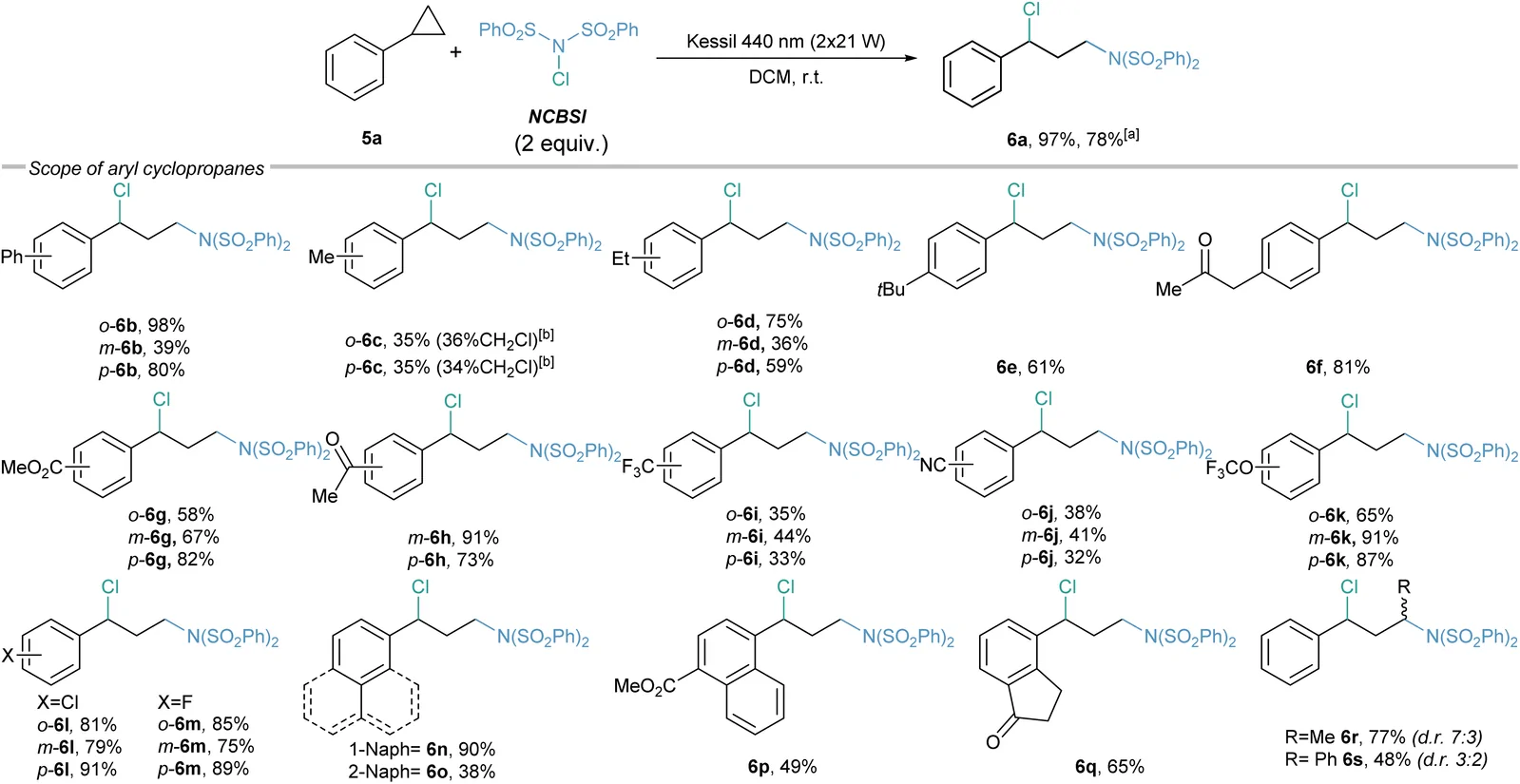
Scope of the photochemical 1,3-aminochlorination of aryl cyclopropanes 5. Reaction conditions: 0.3 mmol scale and 2.0 equiv. NCBSI in anhydrous DCM (0.5 M). Isolated yield is reported. ^*a*^5.0 mmol scale. ^*b*1^H NMR yield was determined with CH_2_Br_2_ as the internal standard.

The scope of aryl cyclopropanes was then studied, starting with variation of substituents on the benzene ring. A phenyl substituent led to the formation of products *o*-6b and *p*-6b in 98% and 80%, respectively. Product *m*-6b was obtained in only 39% yield due to competitive aromatic chlorination, favored by the synergistic donating effect of the substituents, a trend observed for all electron-donating groups. *Para*- and *ortho*-substituted tolyl cyclopropanes 6c gave a 35% yield, together with 34–36% of the products resulting from benzylic chlorination, most probably *via* a hydrogen atom transfer (HAT) pathway.^57^*m*-Tolyl cyclopropane was not successful; besides the chloromethylated product, only chlorination of the aromatic ring was observed. In contrast, with a larger ethyl group, *o*-6d and *p*-6d were formed in good yields and the formation of *m*-6d was possible, even if less efficient. Product 6e, bearing a *tert*-butyl substituent in the *para* position, was formed in 61% yield, while *para* methyl benzyl ketone-substituted 6f was formed in 81% yield. The competition with electrophilic chlorination prevents the use of more electron-donating functional groups. Notably, substrates bearing electron-withdrawing groups performed well (6g–j), underscoring the strong oxidative power of the reagent. The modest yields observed for the trifluoromethyl-(6i) and cyano-(6j) substituted substrates are attributed to incomplete conversion, consistent with their electronic properties disfavoring the initial oxidative step while favoring competing NCBSI degradation. Trifluoromethoxy-substituted (6k) and halogenated arenes (6l–m) delivered yields between 65% and 91% across different substitution patterns. Polycyclic systems such as naphthyl derivatives (6n–p) and an indanone (6q) exhibited variable efficiency, with lower yields observed for the 2-substituted naphthalene derivative, consistent with its lower capacity to stabilize radical cation intermediates.^74^ Finally, substrates with a second alkyl or aryl substituent on the cyclopropane ring gave products 6r and 6s in good yields. The reactions proceeded with electronic control on the position that better stabilizes the positive charge, and no diastereoselectivity was observed.

Subsequently, the functionalization of the benzyl chlorides was explored in a one-pot sequence with the Friedel–Crafts reaction using FeCl_3_ as a Lewis acid in the presence of K_3_PO_4_ as a base (Scheme 3).^57^ Using biphenyl as the arene partner, the reaction with model substrate 6a gave an 86% isolated yield of *p*-7a. Different electron-rich arenes such as toluene, anisole and mesitylene gave respectively 77% (*p*-7b, a potential precursor of 3), 86% (*p*-7c), and 90% (*p*-7d) yields. The reaction with *p*-cresol afforded diarylated product 7e in 47% yield, a potential precursor of Detrol (4). Selected cyclopropanes with a substituted benzene ring yielded the arylation products 7f–j in 55–72% yields. In addition, the 1,3-aminochlorinated product 6 can react as an electrophile in substitution reactions with heteroatom nucleophiles (Scheme 3). Under the same conditions as the Friedel–Crafts reaction, both benzyl ether 8a and sulfide 8b can be obtained in 69% and 68% yields, respectively (Scheme 4A). The substitution with benzylamine (9) was achieved in the presence of catalytic TBAI and K_2_CO_3_ in acetonitrile, giving 10 in 81% yield (Scheme 4B). Lastly, the formation of an aryl ether was attempted with *o*-cresol (11) using an ethanolic NaOH solution. Product 12 bearing only one benzenesulfonyl group was obtained in 37% yield. Chloride 6a was subjected to reported reductive cross electrophile coupling conditions with aryl iodides as partners (Scheme 4C).^57^ This method allowed introduction of aromatic rings with electronics not compatible with the Friedel–Crafts conditions or better control of the regioselectivity of the arylation. The reaction with iodobenzenes 13 gave product 7k in 46% yield, *m*-7l in 34% yield and the previously unobserved isomer *o*-7c in 22% yield. No attempt was made to optimize the reaction conditions beyond what had been reported in the literature.

**Scheme 3:**
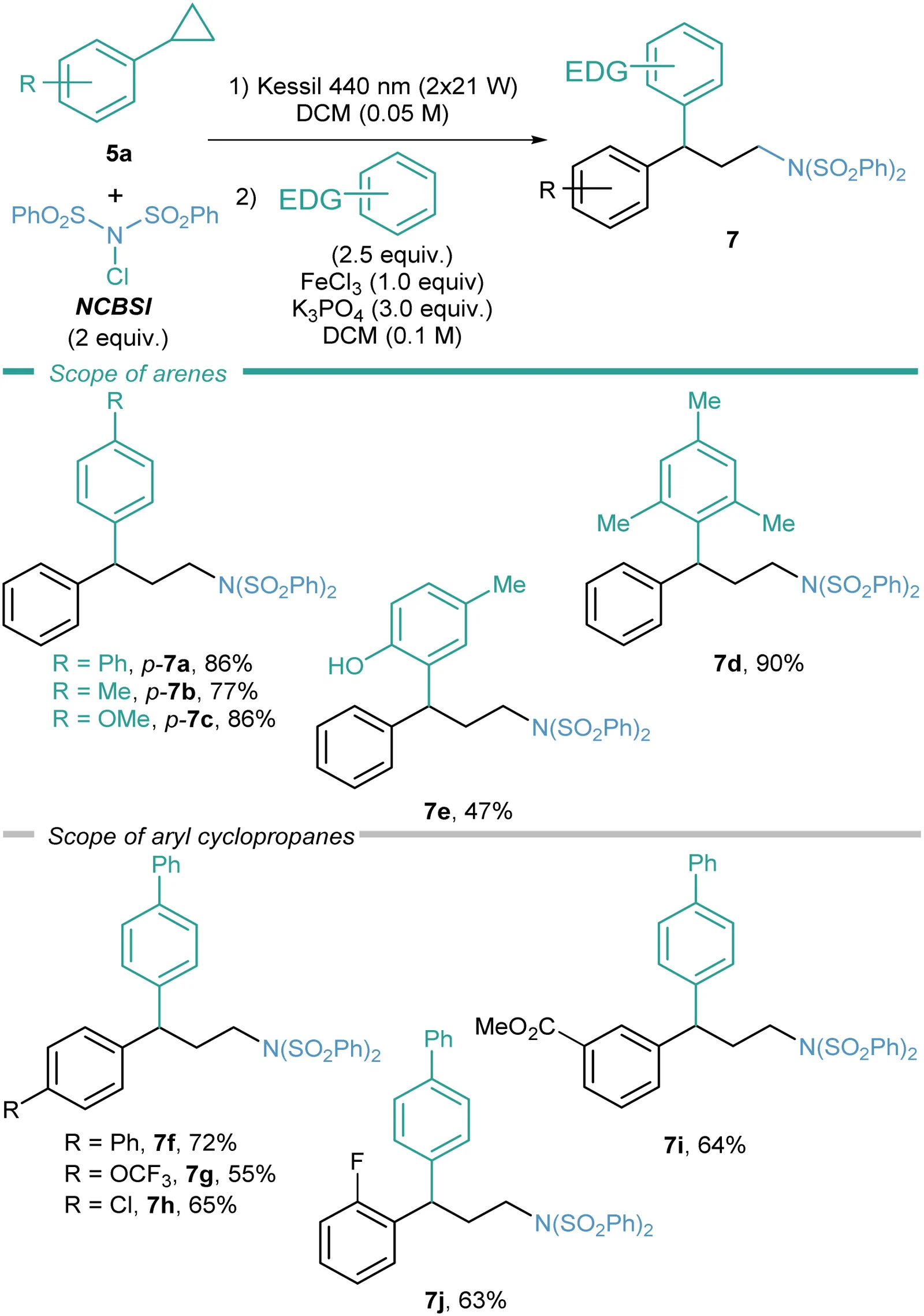
Scope of the one-pot photochemical 1,3-aminochlorination/Friedel–Crafts arylation. Reaction conditions: 0.3 mmol scale, 2.0 equiv. NCBSI in anhydrous DCM (0.5 M) and then 1.0 equiv. FeCl_3_, 2.0 equiv. arene and 3.0 equiv. K_3_PO_4_ in anhydrous DCM (1 M). Isolated yield is reported.

**Scheme 4:**
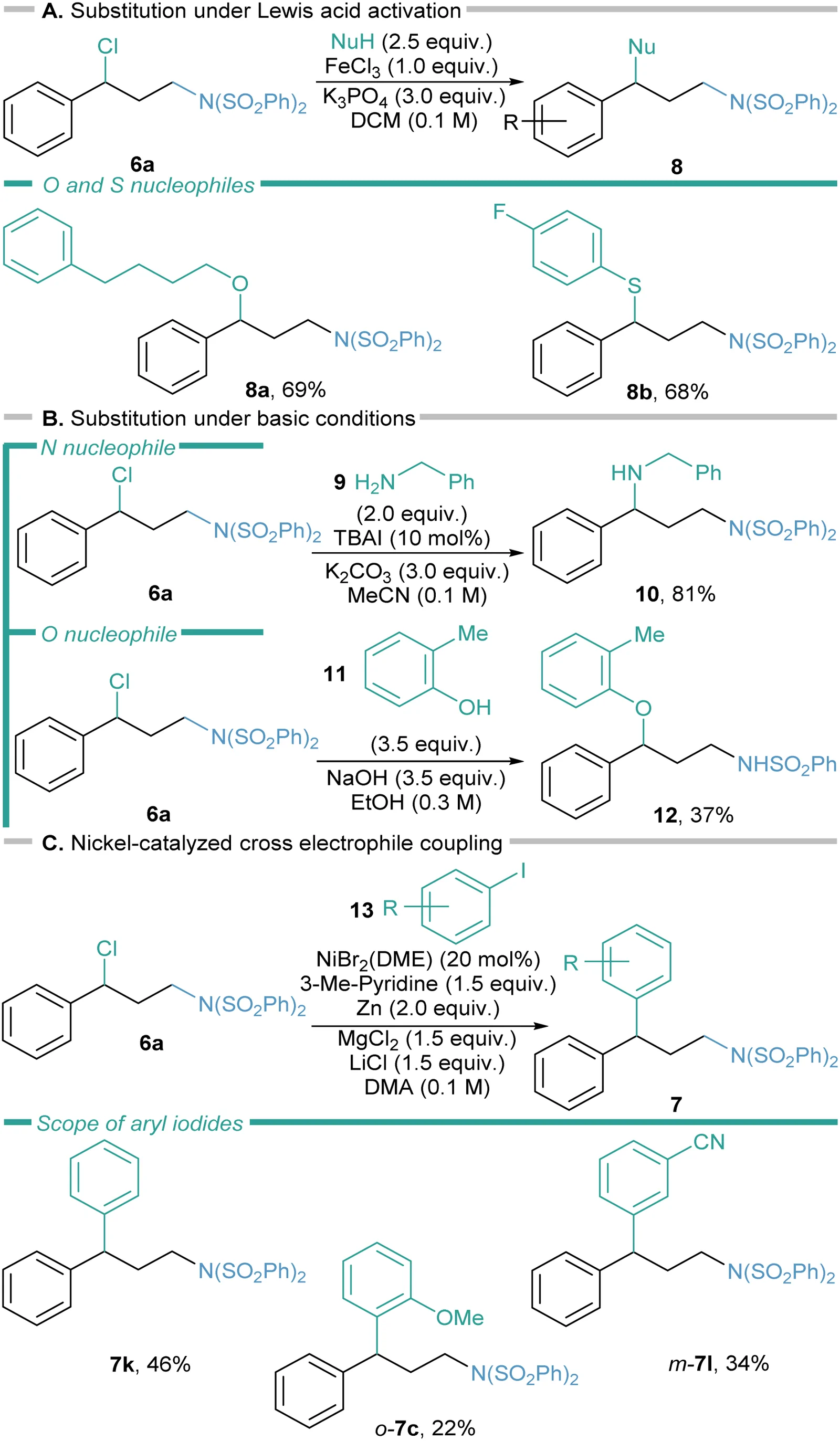
Post-functionalization of the aminochlorinated product 6a: (A) benzylic substitution with O and S nucleophiles under Lewis acid activation. (B) Benzylic substitution with N and O nucleophiles under basic conditions. (C) Nickel-catalyzed cross electrophile coupling. Reactions were performed at a 0.3 mmol scale. Isolated yields are reported.

Overall, this first method represents a direct way to access different 3,3-diarylpropylamines in moderate to excellent yield with wide functional group tolerance. However, the Friedel–Crafts step limits the method to the introduction of electron-rich arenes. The combination of 1,3-aminochlorination with cross-electrophile coupling provides a way to overcome this limitation, but this approach is low yielding and requires a multistep procedure. We therefore considered a more direct approach through the combination of NFSI and boronic acids under copper catalysis to enable a direct 1,3-aminoarylation of the aryl cyclopropanes, based on Zhang's previous work on other amination processes^49–51^ and reported copper-catalyzed arylation methods.^15^

The investigation started by using phenyl cyclopropane 5a, NFSI and phenylboronic acid 14a. The product 7k was observed in trace amounts using CuI as the copper source and phenanthroline L1 as the ligand in DCM (Table 1, entry 1). A 17% yield was obtained by changing the source to Cu(MeCN)_4_OTf (Table 1, entry 2). Switching the counterion to BF_4_ and PF_6_ led to a progressive enhancement of the yield, first to 24% and then to 29% (Table 1, entries 3 and 4).

**Table 1:** Optimization of copper-catalyzed 1,3-aminoarylation

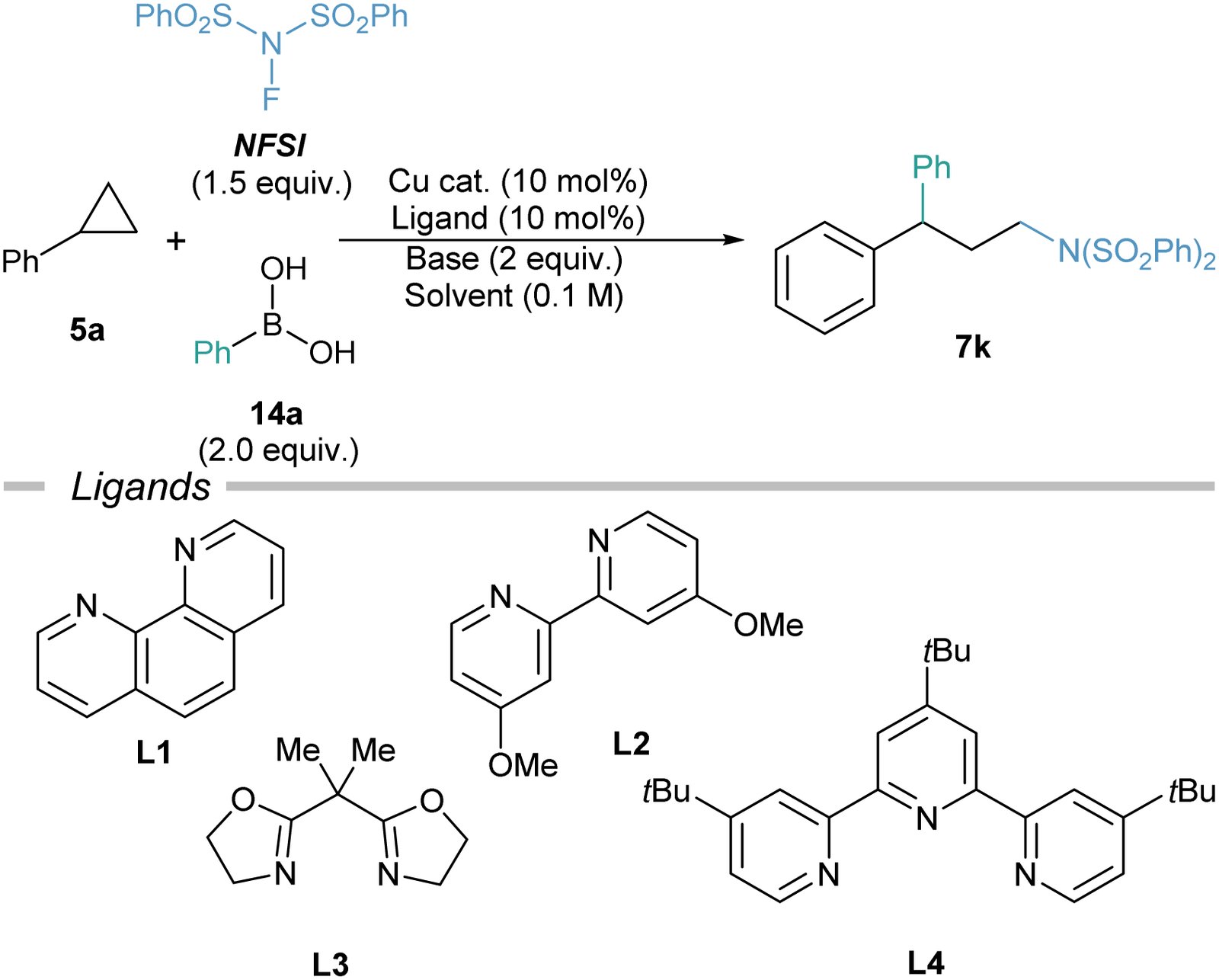					
Entry	Cu cat.	Ligand	Solvent	Temp.	Yield^a^
1	CuI	L1	DCM	25 °C	Trace
2	Cu(MeCN)_4_OTf	L1	DCM	25 °C	17%
3	Cu(MeCN)_4_BF_4_	L1	DCM	25 °C	24%
4	Cu(MeCN)_4_PF_6_	L1	DCM	25 °C	29%
5	Cu(MeCN)_4_PF_6_	L2	DCM	25 °C	15%
6	Cu(MeCN)_4_PF_6_	L3	DCM	25 °C	20%
7	Cu(MeCN)_4_PF_6_	L4	DCM	25 °C	—
8	Cu(MeCN)_4_PF_6_	L1	DCM	0 °C	26%
9	Cu(MeCN)_4_PF_6_	L1	DCM	−20 °C	17%
10^b^	Cu(MeCN)_4_PF_6_	L1	DCM	40 °C	41%
11^c^	Cu(MeCN)_4_PF_6_	L1	DCE	70 °C	45%
12^c^	Cu(MeCN)_4_PF_6_	L1(5%)	DCE	70 °C	57%^d^

a ^1^H NMR yield was determined with CH_2_Br_2_ as the internal standard.

b Reaction time: 1 h.

c Reaction time: 10 min.

d Isolated yield.

The effect of the ligand was explored next; functionalized bipyridine L2 resulted in a lower 15% yield, while box L3 gave a 20% yield (Table 1, entries 5 and 6). Finally, a tridentate terpyridine L4 resulted in complete inhibition of the reaction (Table 1, entry 7); therefore, ligand L1 remained the best. The optimization continued by scanning different temperatures. At 0 °C, a similar yield of 26% was obtained and a further decrease of the temperature to −20 °C gave a 17% yield (Table 1, entries 8 and 9). In contrast, refluxing in DCM or DCE resulted in about 41% and 45% yields (Table 1, entries 10 and 11) and the reaction was faster, reaching completion after 1 h and 10 min, respectively. The optimization campaign ended by reducing the amount of L1 to 5%, obtaining an optimal 57% isolated yield (Table 1, entry 12). (For the full optimization process, see SI, Section 5). This direct method allowed us to access product 7k with enhanced efficiency and better overall yield.

The study proceeded to investigate the scope of aryl cyclopropanes (Scheme 5). As with the previous method, alkyl-substituted cyclopropanes showed no ring-opening under the optimized conditions (see SI, Section S11). Overall, a variety of substituted substrates were compatible with the reaction, delivering the corresponding products in 21–67% yields. A range of functional groups, including aryl (7a), alkyl (7b, 7m–n), *para* methyl benzyl ketone (7o), halogen (7p–r), trifluoromethyl (7s) and carbonyl derivatives (7t), were tolerated. An additional advantage of this method is the absence of halogenation side products for methyl-substituted substrates, allowing the synthesis of polymethylated product 7u in 40% yield. As with the previous method, *trans*-1,2-diphenylcyclopropane formed product 7v in 53% yield and 1 : 1 dr. Various functional groups, including nitrile (7l), halogens (7p and 7q), trifluoromethyl (7s), ester (7w), trifluoromethoxy (7x) and pyridine (7y), were tolerated. The reaction of unfunctionalized aryl cyclopropanes with functionalized aryl boronic acids generally gives better results than the reverse combination. A benzyl bromide (7z) and mixed substitution patterns (7aa and 7ab) were also accommodated, further demonstrating the broad applicability of the transformation. This method does not work with electron-rich boronic acids (see SI, Section S6); therefore, the arylation scope is electronically complementary to the two-step, one-pot protocol presented previously (*vide supra*). While yields typically remained in the low-to-good range (21–70%), the strength of this approach lies in direct three component synthesis of 3,3-diarylpropylamines with wide functional group tolerance across diverse substitution patterns. Both rapid synthesis and chemical diversity are essential for medicinal chemistry applications. The moderate yields originated from multiple side reactions possible in such three component processes, resulting in particular from other undesired two- or three-component combinations (see mechanism discussion, *vide infra*). This protocol was also scalable, producing 7k in 46% yield on a 5 mmol scale (Scheme 6A).

**Scheme 5:**
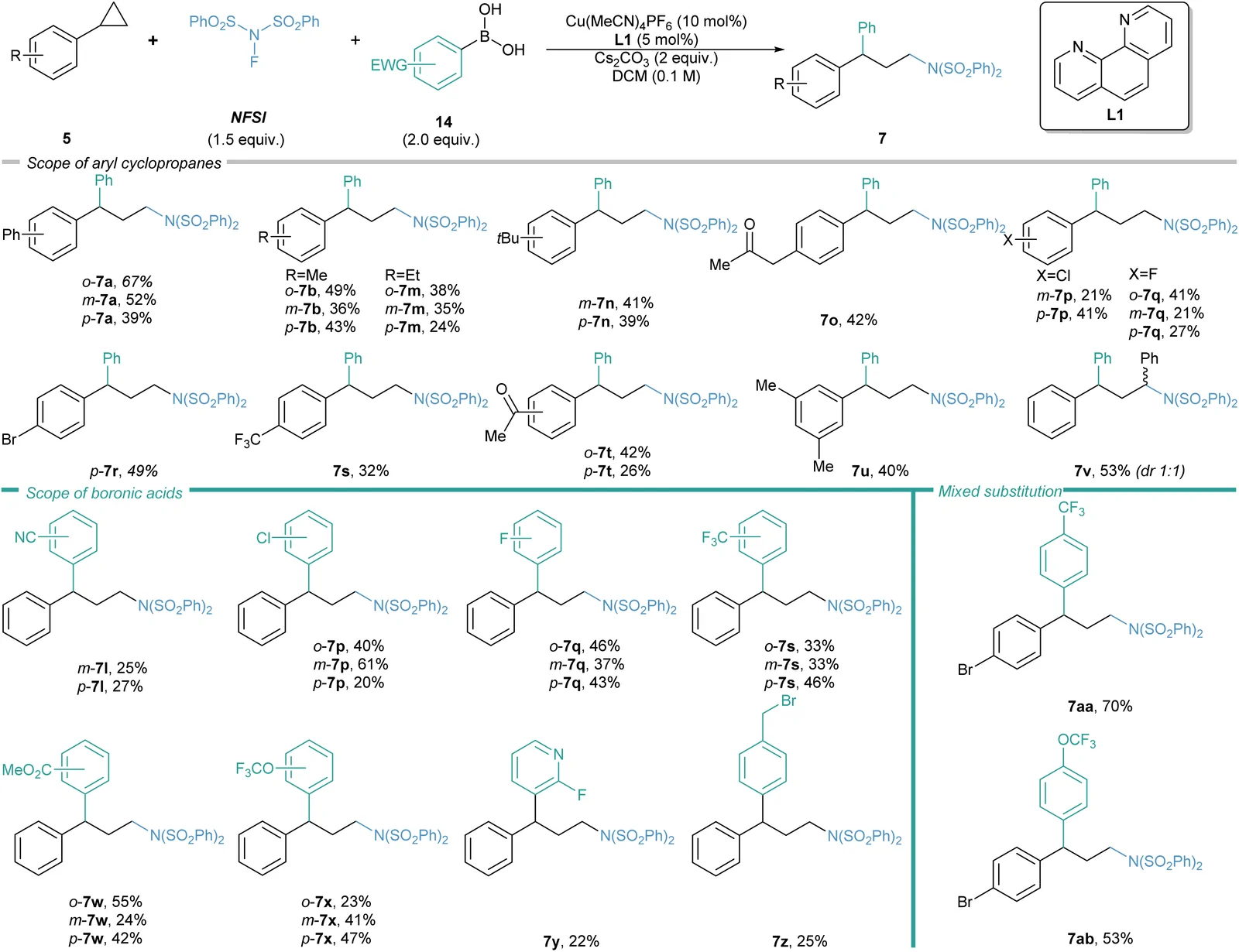
Scope of the copper-catalyzed 1,3-aminoarylation of aryl cyclopropanes 5. Reaction conditions: 0.3 mmol scale, 2.0 equiv. boronic acid 14, 1.5 equiv. NFSI, Cu(MeCN)_4_PF_6_ (10 mol%), L1 (5 mol%), Cs_2_CO_3_ (2.0 equiv.), DCE (0.1 M), 70 °C, and 10 min.

**Scheme 6:**
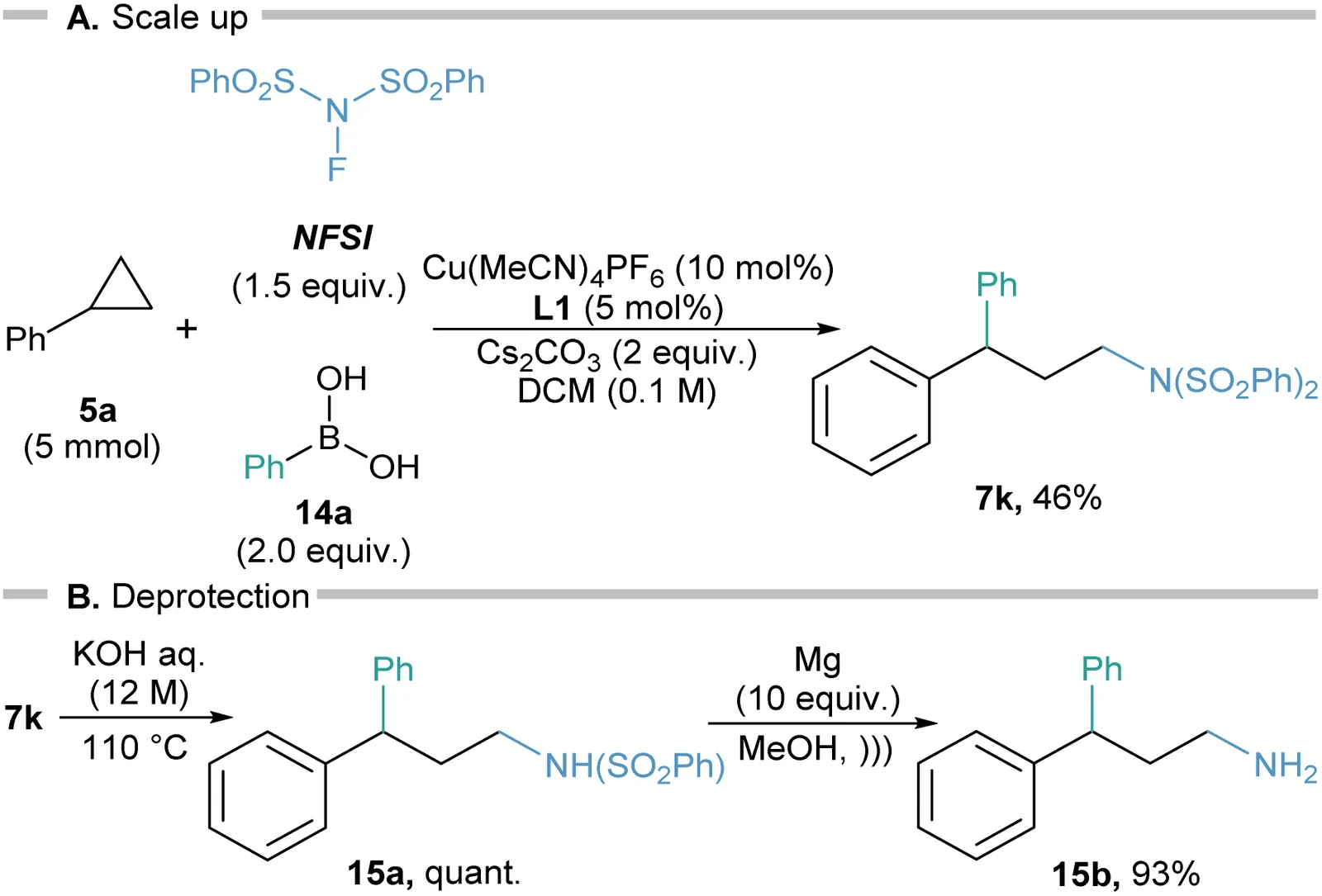
(A) Scale-up of copper-catalyzed 1,3-aminoarylation. (B) Deprotection of 7k.

Furthermore, mono-deprotection of the benzenesulfonimide was achieved under basic conditions to afford 15 quantitatively and the free amine was obtained in 93% yield following a reported method (Scheme 6B).^55^

To better understand the developed methods and the observed selectivity and limitations, mechanistic investigations were performed, first on the light-mediated aminochlorination. The formation of the product is inhibited by different types of radical quenchers (Scheme 7A), clearly indicating radical pathways as already proposed in the literature for other oxidative ring-opening reactions of aryl cyclopropanes.^49–51,57^ In particular, TEMPO adducts 16 and 17 were detected by mass spectrometry, supporting the presence of chloride and benzylic radicals. The use of 1,1-diphenylethylene led to the isolation of alkenes 18, 19, and 20 in 14%, 36% and 54% yields, respectively, supporting the presence of the bisulfonimide radical in addition to the chloride radical. In the presence of BHT, HAT products 21 (23% yield) and 22 (35% yield) were isolated, confirming again the presence of the radical species. Finally, when 1,1-diphenylcyclopropane (5x) was used, alkene 23 was obtained in 52% yield. To address the possibility of a radical chain for the photocatalyzed method, a light on–off experiment was performed (Scheme 7B). The experiment showed product formation even in the absence of irradiation, confirming the presence of a radical chain. Based on the experimental results and previous reports,^49–51,57^ a plausible mechanism can be proposed (Scheme 7C). Given the lability of the N–Cl bond, homolytic cleavage of NCBSI is proposed as an initiation step, generating radicals Ia and IIa, which could be trapped in the form of adducts 16 and 18–22. This initiation pathway is further supported by radical trapping experiments using toluene as a chlorine radical scavenger, consistent with a chlorine radical-mediated initiation step (see SI, Section S7).^75^ As the first propagation step, oxidative C–C cleavage of 5 proceeds through oxidation with Ia*via* single electron transfer (SET) and subsequent nucleophilic attack of the imide on the cyclopropyl radical cation to form benzylic radical IV, which could be trapped as TEMPO adduct 17. Both chloride radical Ia (*E*^°^(Cl˙/Cl^−^) = + 1.84 V *vs.* SCE in MeCN)^38^ and imide radical IIa (*E*^°^(N˙/N^−^) ≈ +2.2 V *vs.* SCE in MeCN)^76^ are strong oxidants known to be able to oxidize aryl cyclopropanes (between +1.4 and +2.2 V *vs.* SCE in MeCN).^38,76^ Nevertheless, selective amination of the terminal position is observed exclusively, which could originate from the higher oxidation potential of radical IIa.

**Scheme 7:**
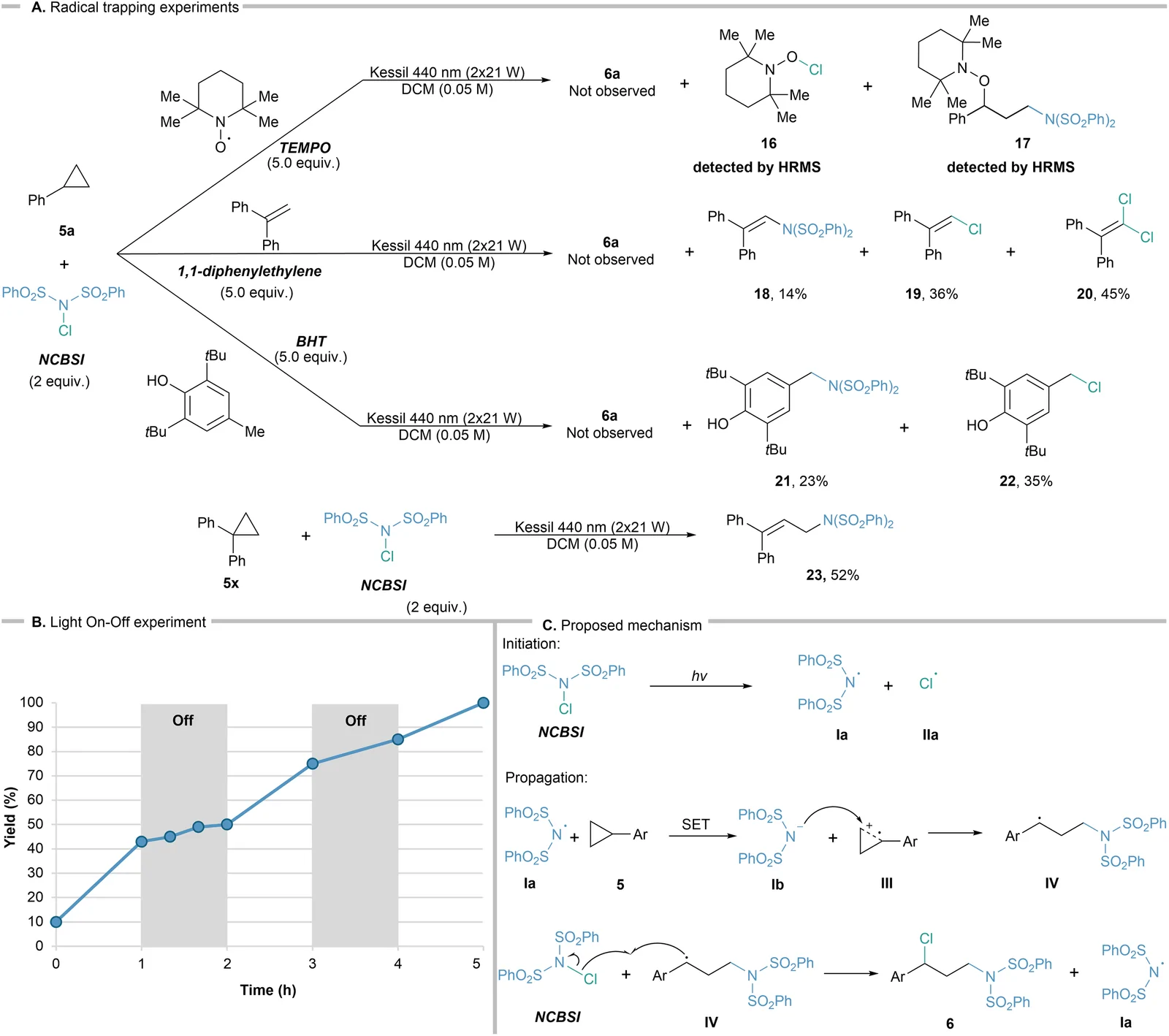
(A) Radical trapping experiments. (B) Light on–off experiment. (C) Proposed radical chain mechanism.

Finally, in the second propagation step, chloroamination product 6 can be formed by the reaction of benzylic radical IV with NCBSI to regenerate the sulfonimide radical Ia. As this process is expected to proceed *via* chloride extraction, only the N-centered radical Ia would be generated, which could also rationalize the selective terminal amination. The copper-catalyzed 1,3-aminoarylation was also inhibited by radical traps (Scheme 8A). The detection of TEMPO adduct 17 by HRMS, along with trapping products obtained with 1,1-diphenylethylene (18) and BHT (21), confirmed the involvement of the sulfonimide radical. The use of cyclopropane 5x also led to the isolation of 23 in 52% yield. Interestingly, when NCBSI was employed, selective formation of halogenated product 6a was observed in an 86% NMR yield, with no trace of product 7k (Scheme 8B). This suggests that the halogenation process is faster than the C–C bond formation, highlighting the different reactivity of N–Cl and N–F reagents.

**Scheme 8:**
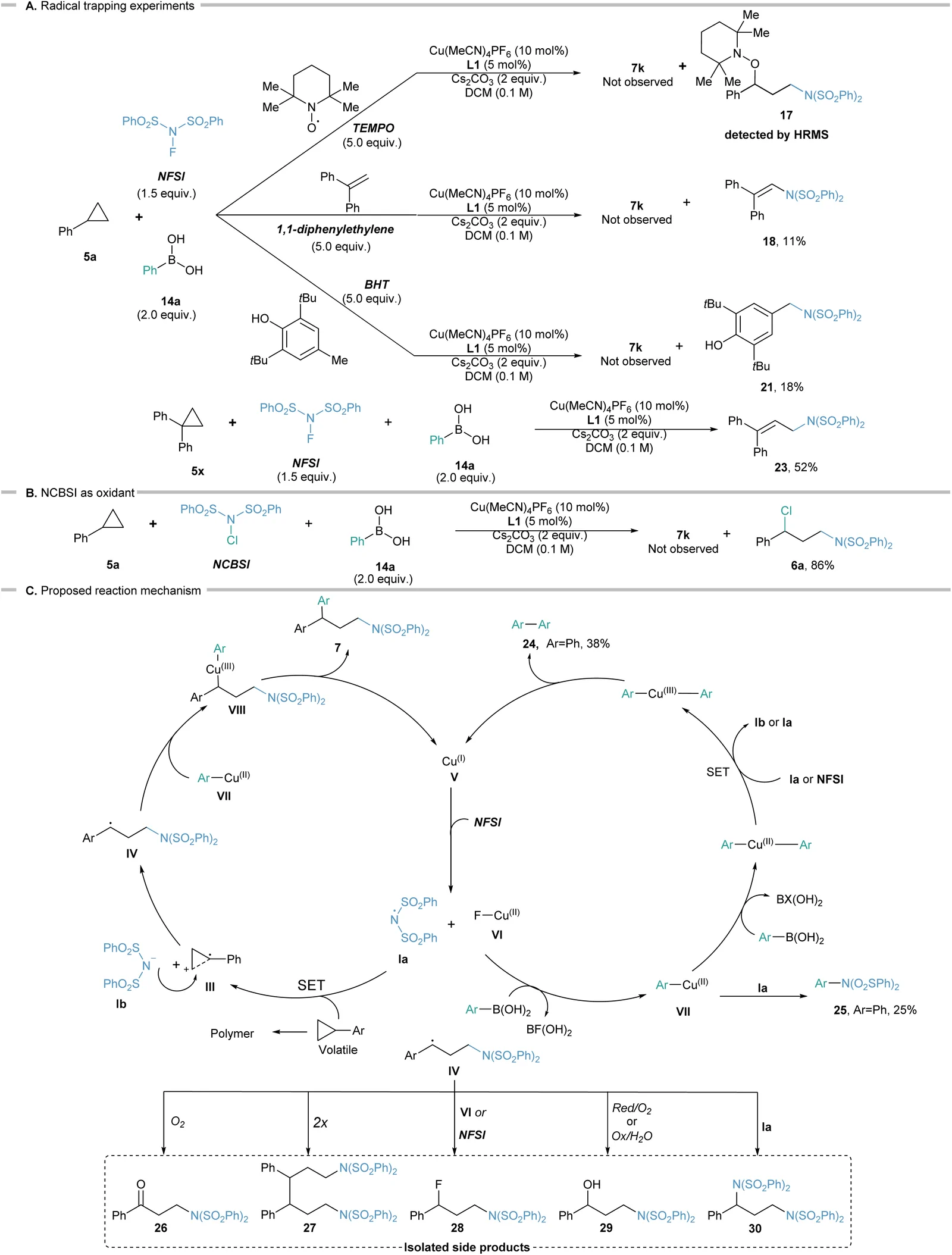
(A) Radical trapping experiments. (B) Use of NCBSI as the oxidant. (C) Proposed reaction mechanism.

Based on the experimental results and on the previous studies,^49–51,57^ the following reaction mechanism can tentatively be proposed (Scheme 8C). A Cu(i) species (V) would first be oxidized by NFSI to form Cu(ii) intermediate VI and radical Ia.^77,78^ The former would undergo transmetallation with the boronic acid to generate aryl Cu(ii) complex VII, whereas the latter reacts with the cyclopropane, forming IV after C–C cleavage of radical cation III. The benzyl radical (IV) is captured by VII, forming a transient Cu(iii) species VIII that undergoes reductive elimination with formation of product 7 and regeneration of catalyst V. To understand the low mass balance of the reaction for some substrates, a careful analysis of side products was performed. First, full consumption of the aryl cyclopropane starting material was observed. The formation of biphenyl (24) and the aniline derivative 25 was observed in 38% and 25% yields, respectively, together with small amounts of phenol and biarylether, indicating the presence of secondary two-component arylation catalytic cycles consuming NFSI and boronic acid (Scheme 8C, right catalytic cycle). Indeed, a Cu-catalyzed homocoupling reaction of boronic acids has been reported with the same catalytic system using oxygen as the oxidant.^79^ As the transmetallation of electron-rich boronic acids is faster, homocoupling would be favored, rationalizing why these substrates were not successful.^80^ These fast direct C–C and C–N couplings make aminoarylation inherently more challenging to develop than other transformations involving the introduction of a second heteroatom, alkynes or cyanide,^50,54^ for which the direct coupling process is slower. Concerning side products originating from the aryl cyclopropane, no single major pathway could be identified. Ketone 26, dimer 27, fluoride 28, alcohol 29, diamine 30 and a polymeric material were all formed in less than 5% yield. Side product 27 resulted from dimerization of radical IV, and 26, 28 and 30 from its reaction with trace amounts of oxygen, NFSI, or radical Ia. The formation of alcohol 29 could originate either from formation of a peroxide with oxygen, followed by reduction, or from over-oxidation to the carbocation followed by reaction with water. This indicates that low efficiency of the final radical recombination-reductive elimination process may be at the origin of the moderate yields obtained.

## Conclusions

In summary, we have established a unified, reagent-controlled platform for assembling 3,3-diarylpropylamines from aryl cyclopropanes through two mechanistically complementary activation modes. The photoactivation of NCBSI enables rapid and regioselective 1,3-aminative chlorination of cyclopropanes, providing versatile intermediates that can be reacted in a one-pot sequence with electron-rich arenes and heteroatom nucleophiles or arylated through a-nickel catalyzed telescoped coupling. In parallel, a copper-catalyzed direct 1,3-aminoarylation engages electron-poor aryl boronic acids, extending the arylation space to partners inaccessible under the photochemical conditions. Mechanistic investigations clarified the divergent catalytic principles and rationalized the scope of each method. Our work underscores the complementary power of photochemistry and transition-metal catalysis in expanding chemical space and provides a general foundation for the discovery-oriented synthesis of bioactive amine building blocks.

## Author contributions

C. B. planned the research, performed the reaction optimization experiments, scope exploration, scale-up reactions, control experiments, and mechanism studies, and prepared the material for the manuscript and the SI. C. N. V. R. contributed to the synthesis of the starting materials and to the execution of the scope exploration. T. V. T. N. performed preliminary studies on the copper catalyzed aminoarylation method and helped in the preparation of the manuscript. J. W. supervised the research and participated in the writing and editing of the manuscript, as well as proofreading the SI. All authors have given approval to the final version of the manuscript.

## Conflicts of interest

There are no conflicts to declare.

## Supplementary Material

SC-OLF-D6SC06246F-s001

## Data Availability

Experimental procedures and analytical data for all new compounds are provided in the supplementary information (SI). See DOI: https://doi.org/10.1039/d6sc06246f. Raw NMR, IR and MS data will be made freely accessible at zenodo.org (https://doi.org/10.5281/zenodo.20659665). Supplementary information is available.

## References

[cit1] Belal Md., Li Z., Lu X., Yin G. (2021). Sci. China:Chem..

[cit2] Moe S. T., Smith D. L., DelMar E. G., Shimizu S. M., Van Wagenen B. C., Balandrin M. F., (Eric) Chien Y., Raszkiewicz J. L., Artman L. D., White H. S., Mueller A. L. (2000). Bioorg. Med. Chem. Lett..

[cit3] Maryanoff B. E., Nortey S. O., Gardocki J. F. (1984). J. Med. Chem..

[cit4] Claudino T. S., Scholten J. D., Monteiro A. L. (2017). Catal. Commun..

[cit5] Yoo K., Kim H., Yun J. (2009). J. Org. Chem..

[cit6] Hu R., Pan F., You C., Ding X., Li X., Zhang X. (2025). Org. Lett..

[cit7] Goswami G., Chauhan N., Mal A., Das S., Das M., Ghorai M. K. (2018). ACS Omega.

[cit8] Luo Z.-B., Wang Y.-W., Peng Y. (2020). Org. Biomol. Chem..

[cit9] Jiang H., Studer A. (2020). Chem. Soc. Rev..

[cit10] Chen X., Xiao F., He W.-M. (2021). Org. Chem. Front..

[cit11] Wu Z., Hu M., Li J., Wu W., Jiang H. (2021). Org. Biomol. Chem..

[cit12] Kwon Y., Wang Q. (2022). Chem.–Asian J..

[cit13] Brenzovich W. E., Benitez D., Lackner A. D., Shunatona H. P., Tkatchouk E., Goddard W. A., Toste F. D. (2010). Angew. Chem., Int. Ed..

[cit14] Sahoo B., Hopkinson M. N., Glorius F. (2013). J. Am. Chem. Soc..

[cit15] Wang D., Wu L., Wang F., Wan X., Chen P., Lin Z., Liu G. (2017). J. Am. Chem. Soc..

[cit16] Monos T. M., McAtee R. C., Stephenson C. R. J. (2018). Science.

[cit17] Yuan Q., Wu C., Bao Y., Huang Z., Wang H., Wei Y., Wang S. (2025). Chem. Commun..

[cit18] Wang Y., Zhang J.-X., Shu W. (2020). ACS Catal..

[cit19] Wang Y., Meng H., Li S., Shu W. (2024). ACS Catal..

[cit20] Sanderson R. T. , Chemical bonds and bond energy, Academic Press, New York, 2nd edn, 1976

[cit21] De Meijere A. (1979). Angew. Chem., Int. Ed. Engl..

[cit22] Reissig H.-U., Zimmer R. (2003). Chem. Rev..

[cit23] Schneider T. F., Kaschel J., Werz D. B. (2014). Angew. Chem., Int. Ed..

[cit24] Pirenne V., Muriel B., Waser J. (2021). Chem. Rev..

[cit25] Liu Y., Wang Q.-L., Chen Z., Zhou C.-S., Xiong B.-Q., Zhang P.-L., Yang C.-A., Zhou Q. (2019). Beilstein J. Org. Chem..

[cit26] Taily I. M., Saha D., Banerjee P. (2021). Org. Biomol. Chem..

[cit27] Chen K., Li Z.-Q., Zhu C., Feng C. (2023). Trends Chem..

[cit28] Manod M., Jabbar H. A., Noushad A., Vijayakumar A., Mohan C. (2025). ChemistrySelect.

[cit29] Shono T., Matsumura Y. (1970). J. Org. Chem..

[cit30] Wang Y., Tanko J. M. (1998). J. Chem. Soc., Perkin Trans. 2.

[cit31] Pitts C. R., Ling B., Snyder J. A., Bragg A. E., Lectka T. (2016). J. Am. Chem. Soc..

[cit32] Banik S. M., Mennie K. M., Jacobsen E. N. (2017). J. Am. Chem. Soc..

[cit33] Wei W., Ai J., Lu Z., Guang T., Dong W., Song C. (2025). J. Org. Chem..

[cit34] Zhou F., Fang L., Si Y., Huang Q., Tang P. (2025). Org. Lett..

[cit35] Zhou W., Chen P., Li Z.-Q., Xiao L.-T., Bai J., Song X.-R., Luo M.-J., Xiao Q. (2023). Org. Lett..

[cit36] Chen Z., Zhao S., Wang T., Xue F., Zhu C., Yue Y., Feng C. (2024). J. Org. Chem..

[cit37] Kim Y., Jang J., Singh M., Kim T. H., Kim D. Y. (2026). Asian J. Org. Chem..

[cit38] Petzold D., Singh P., Almqvist F., König B. (2019). Angew. Chem., Int. Ed..

[cit39] Ge L., Wang D.-X., Xing R., Ma D., Walsh P. J., Feng C. (2019). Nat. Commun..

[cit40] Zuo Z., Daniliuc C. G., Studer A. (2021). Angew. Chem., Int. Ed..

[cit41] Ge L., Zhang C., Pan C., Wang D.-X., Liu D.-Y., Li Z.-Q., Shen P., Tian L., Feng C. (2022). Nat. Commun..

[cit42] Nguyen T. V. T., Wodrich M. D., Waser J. (2022). Chem. Sci..

[cit43] Zuo Z., Studer A. (2022). Org. Lett..

[cit44] Xu Y., Gao H., Pan C., Shi Y., Zhang C., Huang G., Feng C. (2023). Angew. Chem., Int. Ed..

[cit45] Li M., Wu Y., Song X., Sun J., Zhang Z., Zheng G., Zhang Q. (2024). Nat. Commun..

[cit46] Ren L., Wei J., Yu Y., Huang L., Yang L., Wang J., Hao N., Fu Q., Yi D., Wei S., Lu J. (2024). ACS Catal..

[cit47] Kim D., Ju H., Lee W., Hong S. (2024). Chem. Sci..

[cit48] Pan J., Chen S., Lv L., Li Z. (2025). Org. Lett..

[cit49] Wang L., Wang X., Zhang G., Yang S., Li Y., Zhang Q. (2019). Org. Chem. Front..

[cit50] Yang S., Wang L., Zhang H., Liu C., Zhang L., Wang X., Zhang G., Li Y., Zhang Q. (2019). ACS Catal..

[cit51] Wang X., Wang L., Yang S., Zhang L., Li Y., Zhang Q. (2020). Org. Biomol. Chem..

[cit52] Zhang H., Xiao H., Jiang F., Fang Y., Zhu L., Li C. (2021). Org. Lett..

[cit53] Yang S., Liu C., Shangguan X., Li Y., Zhang Q. (2022). Chem. Sci..

[cit54] Bu Y., Zhang Y., Shangguan X., Sun S., Li Y., Zhang Q. (2024). Tetrahedron Lett..

[cit55] Nguyen A. L., Zhang J., Huang S.-H., Wang Q. (2024). Org. Lett..

[cit56] Wu X., Song X., Xia Y. (2024). Adv. Sci..

[cit57] Nguyen T. V. T., Nguyen T. V. Q., Tran T. T. H., Nguyen Q.-H., Waser J. (2026). JACS Au.

[cit58] Legnani L., Prina-Cerai G., Delcaillau T., Willems S., Morandi B. (2018). Science.

[cit59] Kwon Y.-D., Joaquin D., Davenport M. T., Olsen J., Carpio M., Yousufuddin M., Ess D. H., Kürti L. (2026). JACS Au.

[cit60] Wang W., Liu L., Chang W., Li J. (2018). Chem.–Eur. J..

[cit61] Wang F., Wang D., Mu X., Chen P., Liu G. (2014). J. Am. Chem. Soc..

[cit62] Yu X., Zhao Q., Chen J., Chen J., Xiao W. (2018). Angew. Chem., Int. Ed..

[cit63] Lv X.-L., Wang C., Wang Q.-L., Shu W. (2019). Org. Lett..

[cit64] Liang Q., Walsh P. J., Jia T. (2020). ACS Catal..

[cit65] Yang S., Gao P., Suo M.-T., Gao S.-X., Duan X.-H., Guo L.-N. (2020). Chem. Commun..

[cit66] Zhu X., Su M., Zhang Q., Li Y., Bao H. (2020). Org. Lett..

[cit67] Lee S., Yu J. D., Monterde A. L., Tung S. E., Wang Y.-N., Gay B. L., Hull K. L. (2024). ACS Catal..

[cit68] Luo Q., Yao J.-S., Gao Z., Liu X.-Y., Li Z.-L. (2026). Synthesis.

[cit69] Liu X., An R., Zhang X., Luo J., Zhao X. (2016). Angew. Chem., Int. Ed..

[cit70] Li C., Long P., Wu H., Yin H., Chen F.-X. (2019). Org. Biomol. Chem..

[cit71] Sun J., Guo Y., Xia J., Zheng G., Zhang Q. (2023). Molecules.

[cit72] Stilinović V., Horvat G., Hrenar T., Nemec V., Cinčić D. (2017). Chem.–Eur. J..

[cit73] Misal B., Palav A., Ganwir P., Chaturbhuj G. (2021). Tetrahedron Lett..

[cit74] Milne P. H., Wayner D. D. M., DeCosta D. P., Pincock J. A. (1992). Can. J. Chem..

[cit75] Xiang M., Zhou C., Yang X.-L., Chen B., Tung C.-H., Wu L.-Z. (2020). J. Org. Chem..

[cit76] Sheng W., Huang X., Cai J., Zheng Y., Wen Y., Song C., Li J. (2023). Org. Lett..

[cit77] Li J., Zhang Z., Wu L., Zhang W., Chen P., Lin Z., Liu G. (2019). Nature.

[cit78] Mandal M., Buss J. A., Chen S.-J., Cramer C. J., Stahl S. S. (2024). Chem. Sci..

[cit79] Kirai N., Yamamoto Y. (2009). Eur. J. Org. Chem..

[cit80] Salamé A., Rio J., Ciofini I., Perrin L., Grimaud L., Payard P.-A. (2022). Molecules.

