# Supplementary material for: Modular synthesis of 3,3-diarylpropylamines via the photo- and copper-catalyzed 1,3-aminoarylation of cyclopropanes
Source: Chem Sci. 2026 Oct 1. Online ahead of print. doi: 10.1039/d6sc06246f (PMC13669858; doi:10.1039/d6sc06246f)

Supplementary Information (SI) for Chemical Science.
This journal is © The Royal Society of Chemistry

Modular Synthesis of 3,3-Diarylpropylamines via the Photo- and Copper-Catalyzed 1,3-Aminoarylation of Cyclopropanes

Carlo Baldassari,[a,b,c] Carolin N. Veras Rodriguez,[a] Tin V. T. Nguyen[a] and Jerome Waser*[a,c]

[a]Laboratory of Catalysis and Organic Synthesis, Institute of Chemical Sciences and Engineering, Ecole Polytechnique Fédérale de Lausanne, EPFL SB ISIC LCSO, BCH 4306, 1015 Lausanne, Switzerland.

[b]IBM Research, Saumerstrasse 4, 8803, Rüschlikon, Switzerland.

[c]NCCR Catalysis, Zurich, Switzerland.

jerome.waser@epfl.ch

Table of contents

S1. General methods 4

S2. Photochemical experiments setup 5

S3. Synthesis of *N*-chloro-*N*-(phenylsulfonyl) benzenesulfonamide (NCBSI) 6

S4. Synthesis of cyclopropanes 5 6

General procedure A 6

General procedure B 7

General procedure C 7

General procedure D 8

S5. Optimization tables for 1,3-aminochlorination[a] 29

NCBSI loading 29

Solvent amount 29

Light intensity 29

Light wavelength 30

S6. Optimization tables and control experiments for the 1,3-aminoarylation[a] ... 31

Copper source 31

Catalyst loading 31

Ligands 32

Base 33

Solvent 33

Ligand loading 33

Nucleophiles 34

Stoichiometry 34

Temperature 34

Water effect 35

Others 35

Control experiments 35

S7. Radical trapping experiments 37

1,3-Aminochlorination method 38

1,3-Aminoarylation method 38

S8. 1,3-Aminoarylation Side Products analysis 41

Light On-Off experiment 45

S9. 1,3-Aminochlorination of aryl cyclopropanes 46

General procedure E 46
S10. One-pot amino-chlorination/Friedel-Crafts reaction. **Error! Bookmark not defined.**
General procedure F 66
General procedure G 66
General procedure H 67
S11. Product modifications 96
General procedure I 96
S12. References 101
S13. NMR spectra 104
Aryl cyclopropanes **5** 105
1,3-aminochlorinated products **6** 163
1,3-aminoarylated products **7** 209
Other products 271

S1. General methods

For quantitative flash chromatography, distilled technical grade solvents were used. THF, Et_2O, toluene, Acetonitrile and CH_2Cl_2 were dried by passage over activated alumina under nitrogen atmosphere (H2O content < 7 ppm, Karl-Fischer titration). All chemicals were purchased and used as received unless stated otherwise. Chromatographic purification was performed as flash chromatography using Macherey-Nagel silica 40-63, 60 Å, using the solvents indicated as eluent with 0.1-0.5 bar pressure. TLC was performed on Merck silica gel 60 F254 TLC glass plates and visualized with UV light. Reverse phase chromatography was performed as flash chromatography through BUCHI C-810 Flash using Aquarius C18AQ 15μm columns. Preparative RP-HPLC were performed on an Agilent 1260 HPLC system with a G2260A 1260 Prep ALS Autosampler, a G1361a 1260 Prep Pump, a G1365C 1260 MWD detector and a G1364B 1260 FC-PS collector, coupled with a Waters XBridge semi-preparative C18 column (19 x 150 mm, 5 μm). Water (solvent A) and water:acetonitrile 5:95 (solvent B), each containing 0.1% formic acid, were used as the mobile phase at a flow rate of 20 $mL.min^{-1}$. ^{1}H-NMR spectra were recorded at room temperature on a Brucker DPX-400 400 MHz spectrometer in $CDCl_3$, Acetone-d_6, CD_3CN or CD_3OD, all signals are reported in ppm with the internal chloroform signal at 7.26 ppm, the internal acetone signal at 2.09 ppm, the internal acetonitrile signal at 1.94 ppm and the internal methanol signal at 3.34 ppm as standard. The data is reported as (s = singlet, d = doublet, t = triplet, q = quadruplet, p = quintet, m = multiplet or unresolved, br = broad signal, integration, coupling constant(s) in Hz, interpretation). ^{13}C-NMR spectra were recorded with ^{1}H-decoupling on a Brucker DPX-400 101 MHz spectrometer in $CDCl_3$, Acetone-d_6, CD_3CN or CD_3OD, all signals are reported in ppm with the internal chloroform signal at 77.0 ppm, Acetone-d_6 signal at 29.8 ppm, CD_3CN signal at 1.3 ppm or CD_3OD signal at 49.0 ppm as standard. Infrared spectra were recorded on a JASCO FT-IR B4100 or a Bruker Alpha-P spectrophotometer with an ATR device and a ZnSe prism and are reported as cm-1 (w = weak, m = medium, s = strong). High resolution mass spectrometric measurements were performed by the mass spectrometry service of ISIC at the EPFL on a MICROMASS (ESI) Q-TOF Ultima API. GC-FID analysis were performed on an Agilent Intuvo 9000 GC System using N_2 as carrier and FID system as detector. All photocatalyzed reactions were carried out in oven dried glassware and under inert atmosphere unless specified otherwise. They were placed on a stirring plate with Kessil lamps (440 nm, 40 W) irradiating from both sides. The distance between the Kessil lamps and the vials was approximately 10 cm. Long irradiation resulted in temperature increasing up to 50 °C during overnight reactions unless a fan was used in which case the temperature rose to 30-35°C.

S2. Photochemical experiments setup

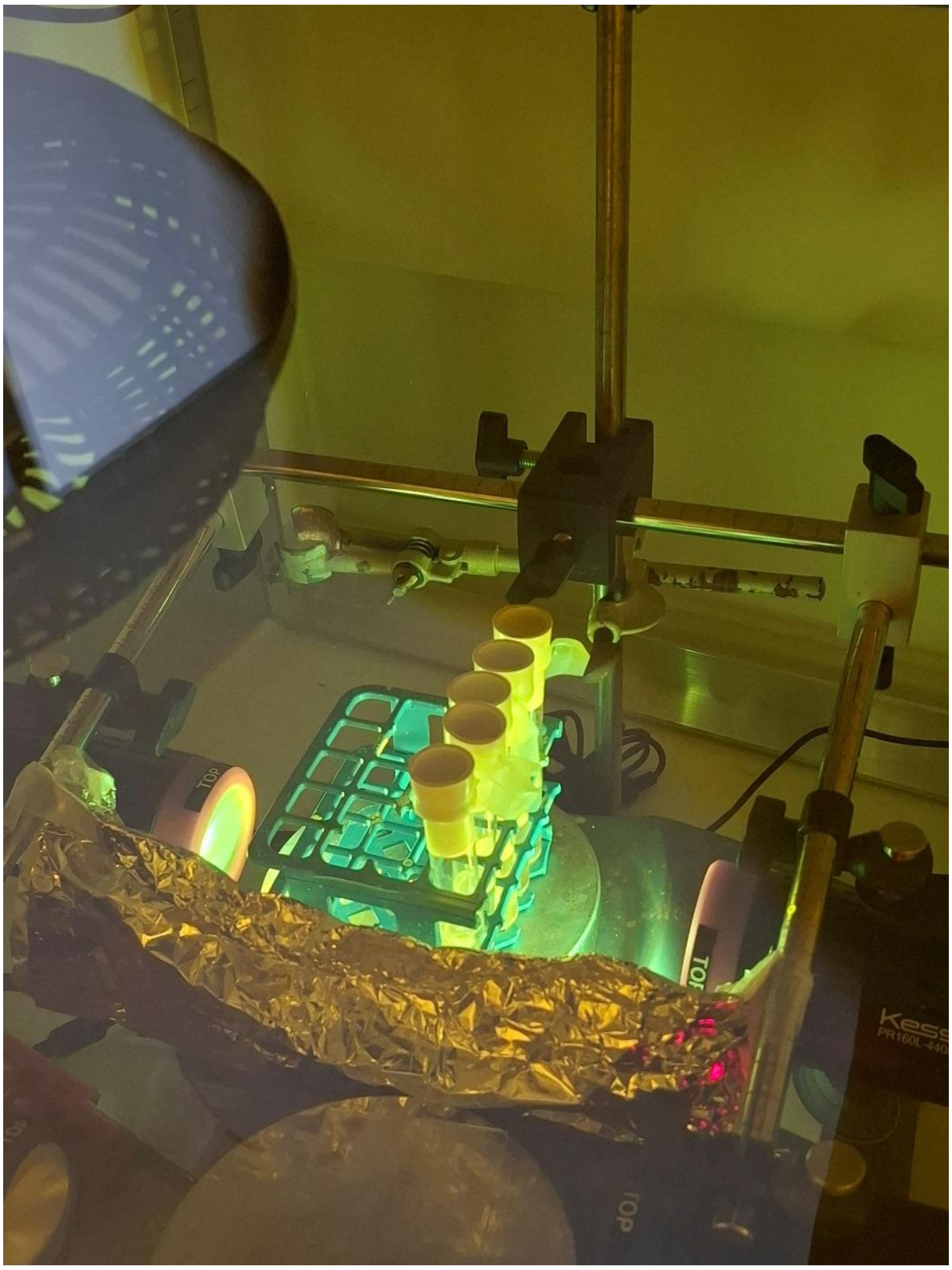

S3. Synthesis of *N*-chloro-*N*-(phenylsulfonyl) benzenesulfonamide (NCBSI)

*t*BuOCl was prepared fresh accordingly with procedure reported.[1]

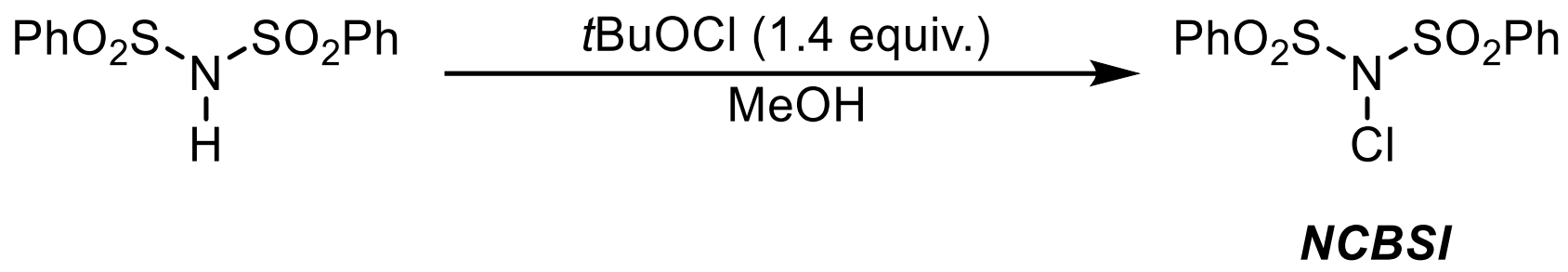

Following a reported procedure,[2] to a saturated solution of *N*-(phenylsulfonyl) benzenesulfonamide (900 mg, 3.00 mmol, 1.0 equiv.) in MeOH (1 mL) was added *t*BuOCl (0.490 mL, 4.33 mmol, 1.4 equiv.) quickly. A large amount of white precipitate was formed after a few seconds. The precipitate was filtered, washed using only H_2O and dried under vacuum to give *N*-chloro-*N*-(phenylsulfonyl) benzenesulfonamide (**NCBSI**) as a white powder (908 mg, 2.74 mmol, 90% yield).

^{1}H NMR (400 MHz, $CDCl_3$) δ 8.09 – 8.00 (m, 4H, Ar*H*), 7.73 (t, *J* = 7.5 Hz, 2H, Ar*H*), 7.60 (t, *J* = 7.9 Hz, 4H, Ar*H*).

^{13}C NMR (101 MHz, $CDCl_3$) δ 136.7, 135.2, 129.4, 129.3.

HRMS (ESI/QTOF) m/z: $[M + Cl]^-$ Calcd for $C_{12}H_{10}NO_4S_2^-$ 296.0057; Found 296.0054. NMR data consistent with reported value.[1]

S4. Synthesis of cyclopropanes 5

Substrates **5a** (phenylcyclopropane) and **5aa** (4-bromo-1-cyclopropylbenzene) are commercially available.

General procedure A

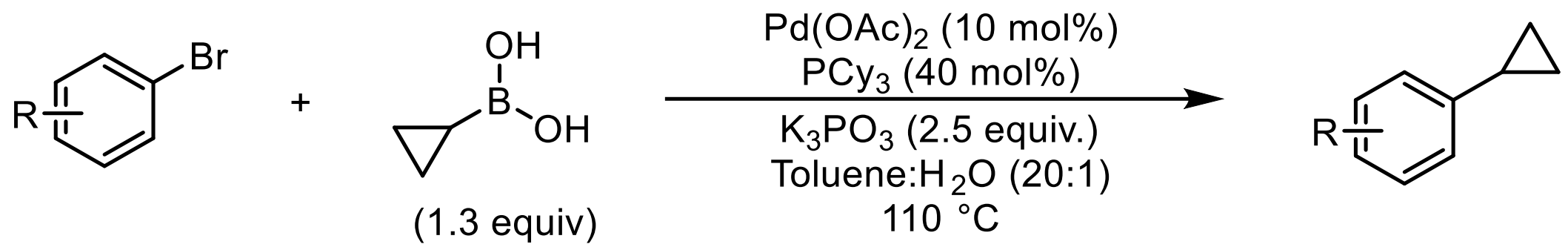

Following a reported procedure,[3] to a solution of aryl bromide (4.00 mmol, 1.0 equiv.) in 20:1 toluene:water (28 mL) was added potassium phosphate tribasic potassium (2.1 g, 10 mmol, 2.5 equiv.), cyclopropylboronic acid (445 mg, 5.20 mmol, 1.3 equiv.), tricyclohexylphosphine (0.70 ml, 1.6 mmol, 0.4 equiv., 2M solution in toluene) and palladium(II) diacetate (91 mg, 0.40 mmol, 0.1 equiv.). The resulting mixture was heated to 110 °C and was stirred at this temperature overnight. The reaction mixture was then cooled to room temperature, diluted with Et_2O (30 mL) and washed with water (30 mL). The aqueous layer was extracted with Et_2O (3 × 30 mL). The combined organic layers were washed with brine (50 mL), dried over Na_2SO_4, filtered and concentrated under reduced pressure. The crude mixture was purified by column

chromatography on Biotage (Büchi flashpure cartridge 25 g, Pentane: Et_2O from 0 to 40%) to obtain the product.

General procedure B

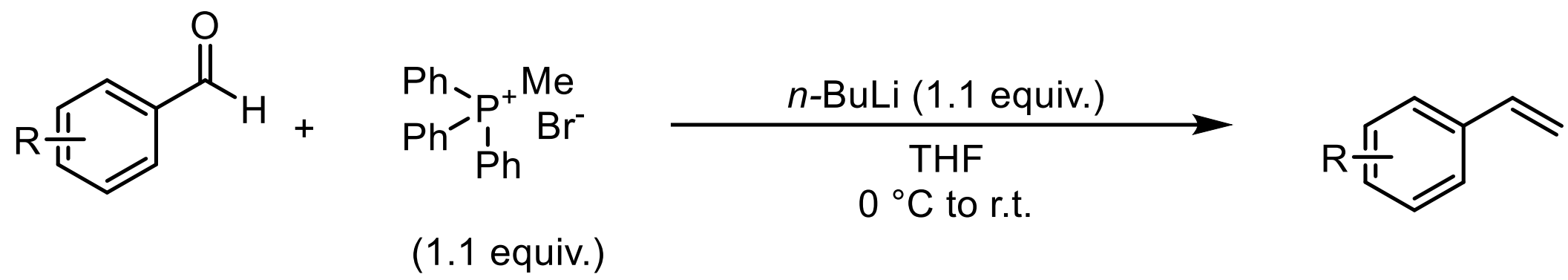

Following a reported procedure,[4] To a solution of methyltriphenylphosphonium bromide (3.93 g, 11.0 mmol, 1.1 equiv.) in anhydrous THF (40 mL) was added dropwise n-BuLi (4.4 mL, 2.5 M in hexanes, 11 mmol, 1.1 equiv.) at 0 °C under a N_2 atmosphere. The solution was stirred at room temperature for 2 hours and then a solution of the aldehyde (10 mmol, 1.0 equiv.) in anhydrous THF (10 mL) was added dropwise at 0 °C. The reaction was stirred for 16 hours at room temperature and then quenched by addition of saturated aqueous NH_4Cl solution. The aqueous layer was extracted with Et_2O and the combined organic layers were washed with brine then dried over Na_2SO_4. After filtration, the solvents were removed under reduced pressure, and the residue was purified by flash column chromatography on Biotage (Büchi flashpure cartridge 25 g, Pentane: Et_2O from 0 to 40%).

General procedure C

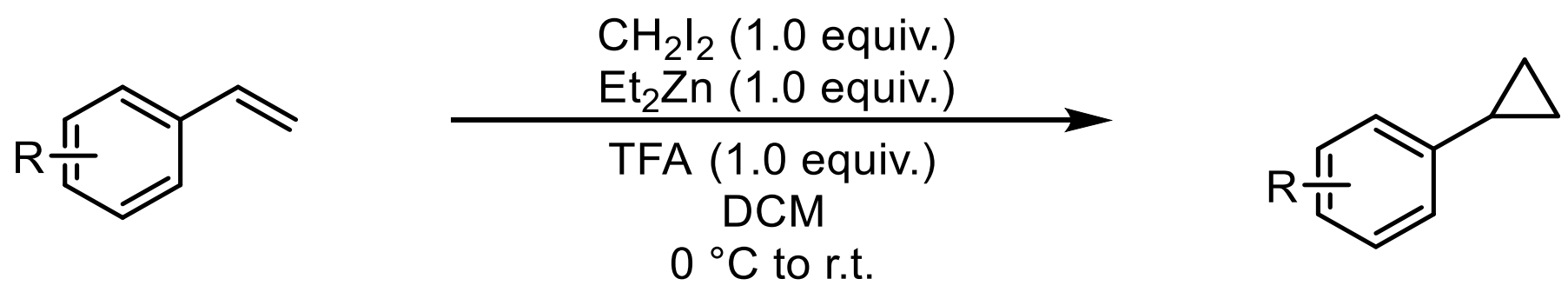

Following a reported procedure,[3] to an oven-dry three-neck flask equipped with a stirring bar, DCM (5 mL) and a 0.8 M solution of Et_2Zn in hexanes (4.8 mL, 6.0 mmol, 1.0 equiv.) were added carefully. The solution was cooled down to 0°C and a solution of TFA (460 μl, 6.00 mmol, 1.0 equiv.) in DCM (5 mL) was added dropwise. The mixture was stirred for 30 min and dropwise addition was repeated first with a solution of CH_2I_2 (485 μl, 6.00 mmol, 1.0 equiv.) in DCM (5 mL) and finally with a solution of a substituted alkene (6.0 mmol, 1.0 equiv.) in DCM (5 mL). The reaction mixture was stirred overnight and let to gradually warm up to room temperature. The reaction was quenched with 1 M HCl at 0 °C, the DCM layer was separated, and the aqueous layer was extracted with pentane. The organic layers were washed with $NaHCO_3$, washed with H_2O, dried with Na_2SO_4, filtered through Celite, and concentrated. The product was purified by column chromatography on silica gel, eluting with pentane. If not removed by column chromatography due to poor separation, CH_2I_2 is removed by dissolving the crude sample in Et_2O, adding an excess of DABCO, and refluxing the mixture for 3 h. Upon cooling, the reaction mixture was filtered through Celite (washing with Et_2O), concentrated, and pushed through a plug of silica gel, eluting with pentane. In case of the presence of unreacted starting material, the crude can be

dissolved in DCM and 1 equiv. of mCPBA was added at 0 °C. The mixture was then allowed to warm up to room temperature overnight, then the product was isolated by column chromatography on Biotage (Büchi flashpure cartridge 25 g, Pentane: Et_2O from 0 to 40%).

General procedure D

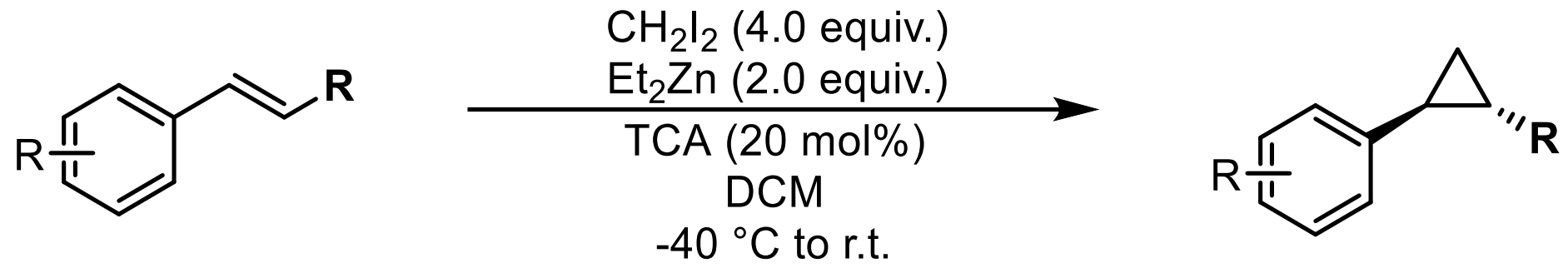

Following a reported procedure,[5] to an oven-dry three-neck flask equipped with a stirring bar, DCM (5 mL) and a 0.8 M solution of Et_2Zn in hexanes (9.6 mL, 10 mmol, 2.0 equiv.) were added carefully. The mixture was cooled down to -40 °C and a solution of CH_2I_2 (5.4 g, 20 mmol, 4.0 equiv.) in DCM (5 mL) was added dropwise and the reaction mixture was stirred for 1h. The same operation was repeated first with a solution of CCl_3COOH (163 mg, 1.0 mmol, 0.2 equiv.) and DME (450 mg, 5.00 mmol, 1.0 equiv.) in DCM (5 mL) and then, after adjusting the temperature to -15°C, with a solution of olefin (5.0 mmol, 1.0 equiv.) in DCM (5 mL). The reaction mixture was stirred overnight and let to gradually warm up to room temperature. The reaction was quenched with 1 M HCl at 0 °C, the DCM layer was separated, and the aqueous layer was extracted with pentane. The organic layers were washed with $NaHCO_3$, washed with H_2O, dried with Na_2SO_4, filtered through Celite, and concentrated. The product was purified by column chromatography on silica gel, eluting with pentane. If not removed by column chromatography due to poor separation, CH_2I_2 was removed by dissolving the crude sample in Et_2O, adding an excess of DABCO, and refluxing the mixture for 3 h. Upon cooling, the reaction mixture was filtered through Celite (washing with Et_2O), concentrated, and pushed through a plug of silica gel, eluting with pentane. In case of the presence of unreacted starting material, the crude was dissolved in DCM and 1 equiv. of mCPBA was added at 0 °C. The mixture was allowed to warm up to room temperature overnight, then the product was isolated by column chromatography on Biotage (Büchi flashpure cartridge 25 g, Pentane: Et_2O from 0 to 40%).

2-Cyclopropyl-1,1'-biphenyl (o-**5b**)

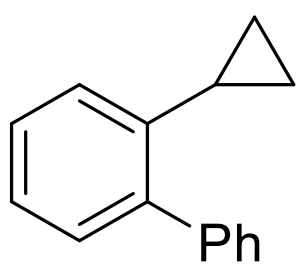

Following the general procedure A using 1-bromo-2-phenylbenzene (932 mg, 4.00 mmol, 1.0 equiv.), yielding 2-cyclopropyl-1,1'-biphenyl (*o*-**5b**) as a clear oil (766 mg, 3.94 mmol, 99%).

R_f (Pentane) = 0.82.
^{1}H NMR (400 MHz, $CDCl_3$) δ 7.48 – 7.40 (m, 4H, Ar*H*), 7.38 – 7.31 (m, 1H, Ar*H*), 7.30 – 7.18 (m, 3H, Ar*H*), 6.94 (dd, *J* = 7.6, 1.3 Hz, 1H, Ar*H*), 1.89 (tt, *J* = 8.5, 5.3 Hz, 1H, C*H*), 0.88 – 0.79 (m, 2H, CH_2), 0.70 (ddd, *J* = 6.7, 5.3, 4.2 Hz, 2H, CH_2).
^{13}C NMR (101 MHz, $CDCl_3$) δ 142.5, 142.0, 140.9, 129.9, 129.8, 128.9, 128.1, 127.6, 127.3, 126.8, 125.3, 123.8, 13.6, 9.8.
HRMS (Sicrit plasma/LTQ-Orbitrap) m/z: $[M + H]^+$ Calcd for $C_{15}H_{15}^+$ 195.1168; Found 195.1168.
NMR data consistent with reported value.[6]

3-Cyclopropyl-1,1'-biphenyl (m-**5b**)

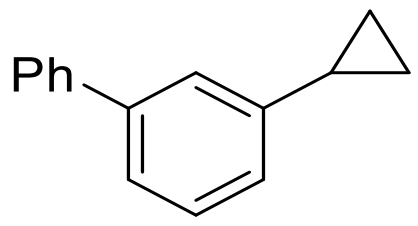

Following the general procedure A using 1-bromo-3-phenylbenzene (932 mg, 4.00 mmol, 1.0 equiv.), yielding 2-cyclopropyl-1,1'-biphenyl (*o*-**5b**) as a clear oil (389 mg, 2.00 mmol, 50%).

R_f (Pentane) = 0.82.
^{1}H NMR (400 MHz, $CDCl_3$) δ 7.59 (dt, *J* = 7.9, 1.2 Hz, 2H, Ar*H*), 7.48 – 7.41 (m, 2H, Ar*H*), 7.41 – 7.29 (m, 4H, Ar*H*), 7.07 (dq, *J* = 7.3, 1.5 Hz, 1H, Ar*H*), 2.07 – 1.90 (m, 1H, C*H*), 1.05 – 0.93 (m, 2H, CH_2), 0.77 (dtd, *J* = 6.2, 4.7, 1.1 Hz, 2H, CH_2).
^{13}C NMR (101 MHz, $CDCl_3$) δ 144.6, 141.6, 141.4, 128.8, 127.4, 127.3, 124.9, 124.7, 124.5, 15.6, 9.4.
One carbon not resolved.
HRMS (APCI/QTOF) m/z: $[M + H]^+$ Calcd for $C_{15}H_{15}^+$ 195.1168; Found 195.1170.
NMR data consistent with reported value.[6]

4-Cyclopropyl-1,1'-biphenyl (*p*-**5b**)

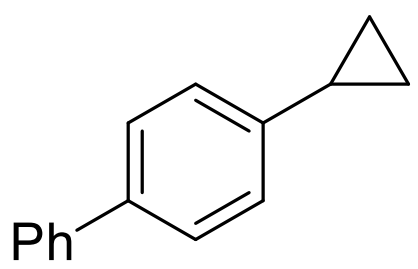

Following the general procedure A using 1-bromo-4-phenylbenzene (932 mg, 2.00 mmol, 1.0 equiv.), yielding 2-cyclopropyl-1,1'-biphenyl (*o*-**5b**) as a clear oil (368 mg, 1.89 mmol, 95%).

R_f (Pentane) = 0.82.
^{1}H NMR (400 MHz, $CDCl_3$) δ 7.61 – 7.54 (m, 2H, Ar*H*), 7.53 – 7.47 (m, 2H, Ar*H*), 7.46 – 7.38 (m, 2H, Ar*H*), 7.36 – 7.28 (m, 1H, Ar*H*), 7.21 – 7.09 (m, 2H, Ar*H*), 1.94 (tt, *J* = 8.4, 5.1 Hz, 1H, C*H*), 1.04 – 0.94 (m, 2H, CH_2), 0.80 – 0.69 (m, 2H, CH_2).
^{13}C NMR (101 MHz, $CDCl_3$) δ 143.2, 141.1, 138.4, 128.7, 127.0, 126.9, 126.0, 15.1, 9.3.
HRMS (Sicrit plasma/LTQ-Orbitrap) m/z: $[M + H]^+$ Calcd for $C_{15}H_{15}^+$ 195.1168; Found

195.1168.
NMR data consistent with reported value.[6]

1-Cyclopropyl-2-methylbenzene (o-**5c**)

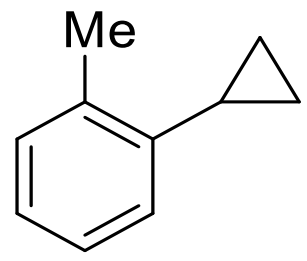

Following the general procedure A using 1-bromo-2-methylbenzene (684 mg, 4.00 mmol, 1.0 equiv.), yielding 1-cyclopropyl-2-methylbenzene (*o*-**5c**) as a clear oil (480 mg, 3.63 mmol, 91%).

R_f (Pentane) = 0.80.
^{1}H NMR (400 MHz, $CDCl_3$) δ 7.18 – 7.05 (m, 3H, Ar*H*), 6.98 (dd, *J* = 6.8, 2.2 Hz, 1H, Ar*H*), 2.43 (s, 3H, C*H_3*), 1.89 (tt, *J* = 8.5, 5.4 Hz, 1H, C*H*), 0.99 – 0.84 (m, 2H, C*H_2*), 0.64 (td, *J* = 6.0, 4.2 Hz, 2H, C*H_2*).
^{13}C NMR (101 MHz, $CDCl_3$) δ 141.3, 137.9, 129.5, 125.8, 125.6, 125.4, 19.6, 13.4, 6.7.
HRMS (APCI/QTOF) m/z: $[M + H]^+$ Calcd for $C_{10}H_{13}^+$ 133.1012; Found 133.1007.
NMR data consistent with reported value.[6]

1-Cyclopropyl-3-methylbenzene (m-**5c**)

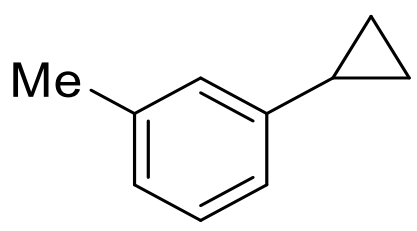

Following the general procedure A using 1-bromo-3-methylbenzene (684 mg, 4.00 mmol, 1.0 equiv.), yielding 1-cyclopropyl-3-methylbenzene (*m*-**5c**) as a clear oil (272 mg, 2.06 mmol, 51%).

R_f (Pentane) = 0.80.
^{1}H NMR (400 MHz, $CDCl_3$) δ 7.14 (dd, *J* = 8.2, 6.8 Hz, 1H, Ar*H*), 6.96 (d, *J* = 7.6 Hz, 1H, Ar*H*), 6.91 – 6.84 (m, 2H, Ar*H*), 2.32 (s, 3H, C*H_3*), 1.86 (tt, *J* = 8.2, 5.3 Hz, 1H, C*H*), 0.94 (ddt, *J* = 10.0, 5.9, 2.7 Hz, 2H, C*H_2*), 0.68 (qd, *J* = 5.4, 1.6 Hz, 2H, C*H_2*).
^{13}C NMR (101 MHz, $CDCl_3$) δ 144.0, 137.9, 128.3, 126.6, 126.3, 122.8, 21.6, 15.4, 9.2.
HRMS (APCI/QTOF) m/z: $[M + H]^+$ Calcd for $C_{10}H_{13}^+$ 133.1012; Found 133.1011.
NMR data consistent with reported value.[6]

1-Cyclopropyl-4-methylbenzene (p-**5c**)

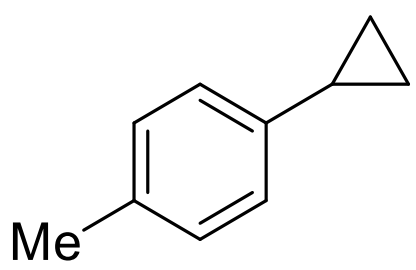

Following the general procedure A using 1-bromo-4-methylbenzene (684 mg, 4.00 mmol, 1.0 equiv.), yielding 1-cyclopropyl-2-methylbenzene (*p*-**5c**) as a clear oil (307 mg, 2.32 mmol, 58%).

R_f (Pentane) = 0.80.
^{1}H NMR (400 MHz, $CDCl_3$) δ 7.07 (d, *J* = 7.8 Hz, 2H, Ar*H*), 6.98 (d, *J* = 8.1 Hz, 2H, Ar*H*), 2.31 (s, 3H, CH_3), 1.87 (tt, *J* = 8.5, 5.1 Hz, 1H, C*H*), 1.00 – 0.86 (m, 2H, CH_2), 0.66 (dt, *J* = 6.5, 4.6 Hz, 2H, CH_2).
^{13}C NMR (101 MHz, $CDCl_3$) δ 141.0, 134.9, 129.1, 125.7, 21.1, 15.1, 9.0.
HRMS (Sicrit plasma/LTQ-Orbitrap) m/z: $[M + H]^+$ Calcd for $C_{10}H_{13}^+$ 133.1012; Found 133.1012.
NMR data consistent with reported value.[6]

1-Cyclopropyl-2-ethylbenzene (o-**5d**)

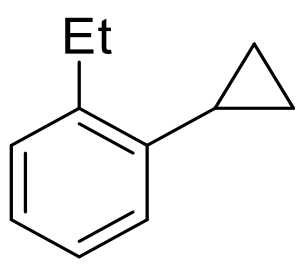

Following the general procedure A using 1-bromo-2-ethylbenzene (740 mg, 4.00 mmol, 1.0 equiv.), yielding 1-cyclopropyl-2-ethylbenzene (*o*-**5d**) as a clear oil (513 mg, 3.51 mmol, 88%).

R_f (Pentane) = 0.79.
^{1}H NMR (400 MHz, $CDCl_3$) δ 7.21 – 7.07 (m, 3H, Ar*H*), 7.01 – 6.93 (m, 1H, Ar*H*), 2.84 (qd, *J* = 7.7, 1.2 Hz, 2H, CH_2), 1.96 (tt, *J* = 8.5, 5.3 Hz, 1H, C*H*), 1.28 (td, *J* = 7.6, 1.3 Hz, 3H, CH_3), 0.99 – 0.88 (m, 2H, CH_2), 0.67 (tdd, *J* = 5.7, 4.2, 1.2 Hz, 2H, CH_2).
^{13}C NMR (101 MHz, $CDCl_3$) δ 143.7, 140.8, 128.0, 125.9, 125.9, 125.4, 26.1, 15.0, 12.9, 7.3.
HRMS (Sicrit plasma/LTQ-Orbitrap) m/z: $[M + H]^+$ Calcd for $C_{11}H_{15}^+$ 147.1168; Found 147.1168.
NMR data consistent with reported value.[6]

1-Cyclopropyl-3-ethylbenzene (m-**5d**)

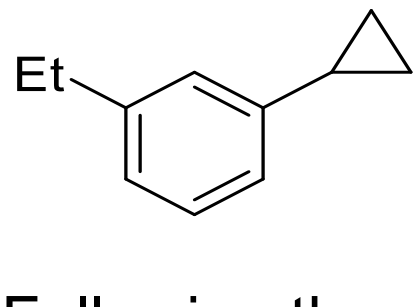

Following the general procedure A using 1-bromo-3-ethylbenzene (740 mg, 4.00 mmol, 1.0 equiv.), yielding 1-cyclopropyl-3-ethylbenzene (*m*-**5d**) as a clear oil (526 mg, 3.60 mmol, 90%).

R_f (Pentane) = 0.79.
^{1}H NMR (400 MHz, $CDCl_3$) δ 7.18 (t, *J* = 7.6 Hz, 1H, Ar*H*), 6.99 (dt, *J* = 7.7, 1.3 Hz, 1H, Ar*H*), 6.93 (d, *J* = 1.9 Hz, 1H, Ar*H*), 6.88 (dt, *J* = 7.6, 1.5 Hz, 1H, Ar*H*), 2.62 (q, *J* = 7.6 Hz, 2H, CH_2), 1.88 (tt, *J* = 8.4, 5.1 Hz, 1H, C*H*), 1.23 (t, *J* = 7.6 Hz, 3H, CH_3),

1.00 – 0.90 (m, 2H, C*H*$_2$), 0.78 – 0.65 (m, 2H, C*H*$_2$).
^{13}C NMR (101 MHz, $CDCl_3$) δ 144.3, 144.1, 128.4, 125.5, 125.1, 122.9, 29.0, 15.8, 15.5, 9.2.
HRMS (Sicrit plasma/LTQ-Orbitrap) m/z: $[M + H]^+$ Calcd for $C_{11}H_{15}^+$ 147.1168; Found 147.1168.
NMR data consistent with reported value. [7]

1-Cyclopropyl-4-ethylbenzene (p-**5d**)

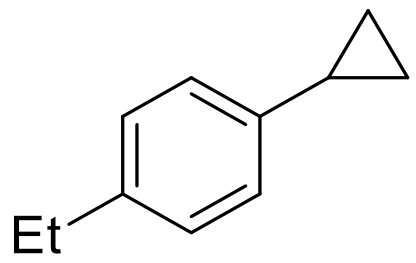

Following the general procedure A using 1-bromo-4-ethylbenzene (740 mg, 4.00 mmol, 1.0 equiv.), yielding 1-cyclopropyl-4-ethylbenzene (*p*-**5d**) as a clear oil (543 mg, 3.71 mmol, 93%).

R_f (Pentane) = 0.79.
^{1}H NMR (400 MHz, $CDCl_3$) δ 7.13 – 7.07 (m, 2H, Ar*H*), 7.04 – 6.97 (m, 2H, Ar*H*), 2.61 (q, *J* = 7.6 Hz, 2H, C*H*$_2$), 1.87 (tt, *J* = 8.4, 5.1 Hz, 1H, C*H*), 1.22 (t, *J* = 7.6 Hz, 3H, C*H*$_3$), 0.96 – 0.88 (m, 2H, C*H*$_2$), 0.67 (dt, *J* = 6.5, 4.5 Hz, 2H, C*H*$_2$).
^{13}C NMR (101 MHz, $CDCl_3$) δ 141.4, 141.2, 127.9, 125.8, 28.5, 15.8, 15.14, 9.1.
HRMS (Sicrit plasma/LTQ-Orbitrap) m/z: $[M + H]^+$ Calcd for $C_{11}H_{15}^+$ 147.1168; Found 147.1169.
NMR data consistent with reported value.[8]

1-(*Tert*-butyl)-3-cyclopropylbenzene (m-**5e**)

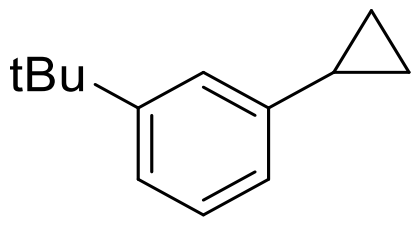

Following the general procedure A using 1-bromo-3-tert-butyl-benzene (852 mg, 4.00 mmol, 1.0 equiv.), yielding 1-(tert-butyl)-3-cyclopropylbenzene (*m*-**5e**) as a clear oil (642 mg, 3.69 mmol, 92%).

R_f (Pentane) = 0.79.
^{1}H NMR (400 MHz, $CDCl_3$) δ 7.23 – 7.10 (m, 3H, Ar*H*), 6.85 (ddd, *J* = 6.1, 2.5, 1.6 Hz, 1H, Ar*H*), 1.95 – 1.85 (m, 1H, C*H*), 1.33 (s, 9H, CC*H*$_3$), 1.02 – 0.89 (m, 2H, C*H*$_2$), 0.78 – 0.65 (m, 2H, C*H*$_2$).
^{13}C NMR (101 MHz, $CDCl_3$) δ 151.1, 143.6, 128.2, 123.3, 122.6, 122.3, 34.7, 31.5, 15.7, 9.3.
HRMS (ESI/QTOF) m/z: $[M + H]^+$ Calcd for $C_{13}H_{19}^+$ 175.1481; Found 175.1480.
NMR data consistent with reported value.[3]

1-(*Tert* -butyl)-4-cyclopropylbenzene (p-**5e**)

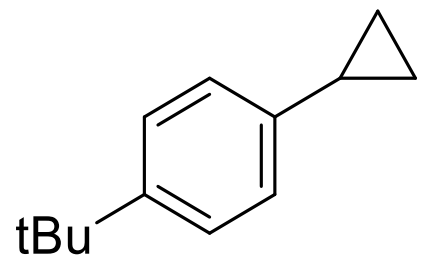

Following the general procedure A using using 1-bromo-4-tert-butyl-benzene (852 mg, 4.00 mmol, 1.0 equiv.), yielding 1-(tert-butyl)-4-cyclopropylbenzene (*p*-**5e**) as a clear oil (607 mg, 3.48 mmol, 87%).

R_f (Pentane) = 0.79.
^{1}H NMR (400 MHz, $CDCl_3$) δ 7.33 – 7.26 (m, 2H, Ar*H*), 7.06 – 6.95 (m, 2H, Ar*H*), 1.87 (tt, *J* = 8.5, 5.1 Hz, 1H, C*H*), 1.30 (s, 9H, CCH_3), 0.99 – 0.85 (m, 2H, CH_2), 0.72 – 0.63 (m, 2H, CH_2).
^{13}C NMR (101 MHz, $CDCl_3$) δ 148.4, 141.0, 125.5, 125.3, 34.5, 31.5, 15.0, 9.1.
HRMS (ESI/QTOF) m/z: $[M + H]^+$ Calcd for $C_{13}H_{19}^+$ 175.1481; Found 175.1483.
NMR data consistent with reported value.[3]

1-(4-Cyclopropylphenyl)propan-2-one (**5f**)

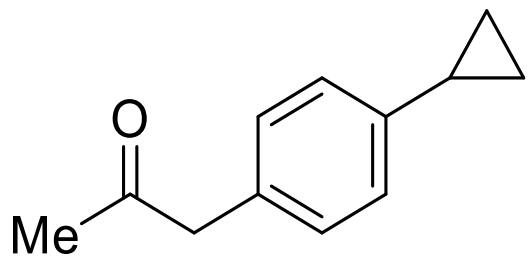

Following the general procedure A using 1-(4-bromophenyl)propan-2-one (852 mg, 4.00 mmol, 1.0 equiv.), yielding 1-(tert-butyl)-4-cyclopropylbenzene (**5f**) as a clear oil (417 mg, 2.39 mmol, 60%).

R_f (Pentane:Et_2O = 95:5) = 0.45.
^{1}H NMR (400 MHz, $CDCl_3$) δ 7.09 (d, *J* = 8.3 Hz, 2H, Ar*H*), 7.04 (d, *J* = 8.2 Hz, 2H, Ar*H*), 3.64 (s, 2H, CH_2), 2.13 (s, 3H, CH_3), 1.88 (tt, *J* = 8.5, 5.1 Hz, 1H, C*H*), 0.99 – 0.89 (m, 2H, CH_2), 0.68 (dt, *J* = 6.6, 4.6 Hz, 2H, CH_2).
^{13}C NMR (101 MHz, $CDCl_3$) δ 206.7, 142.9, 131.2, 129.3, 126.1, 50.7, 29.2, 15.1, 9.2.
IR (ν_{max}, cm^{-1}) 1710 (s), 1516 (m), 1420 (m), 1357 (m), 1227 (m), 1161 (m), 1047 (m), 1019 (m), 897 (m), 815 (m)
HRMS (APCI/QTOF) m/z: $[M + H]^+$ Calcd for $C_{12}H_{15}O^+$ 175.1117; Found 175.1110.

Methyl 2-cyclopropylbenzoate (o-**5g**)

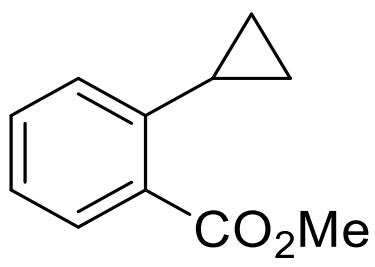

Following the general procedure A using methyl 2-bromobenzoate (860 mg, 4.00 mmol, 1.0 equiv.), yielding methyl 2-cyclopropylbenzoate (*o*-**5g**) as a clear oil (664 mg, 3.86 mmol, 96%).

R_f (Pentane:Et_2O = 8:2) = 0.15.
^{1}H NMR (400 MHz, $CDCl_3$) δ 7.79 (dd, *J* = 7.8, 1.5 Hz, 1H, Ar*H*), 7.39 (td, *J* = 7.6, 1.5 Hz, 1H, Ar*H*), 7.19 (td, *J* = 7.6, 1.3 Hz, 1H, Ar*H*), 7.03 – 6.98 (m, 1H, Ar*H*), 3.92 (s, 3H, CH_3), 2.65 (tt, *J* = 8.5, 5.4 Hz, 1H, C*H*), 1.06 – 0.93 (m, 2H, CH_2), 0.76 – 0.63 (m, 2H, CH_2).
^{13}C NMR (101 MHz, $CDCl_3$) δ 168.8, 144.8, 131.9, 131.4, 130.1, 125.8, 125.4, 52.1, 13.6, 8.9.
HRMS (Sicrit plasma/LTQ-Orbitrap) m/z: $[M + H]^+$ Calcd for $C_{11}H_{13}O_2^+$ 177.0910; Found 177.0909.
NMR data consistent with reported value.[9]

Methyl 3-cyclopropylbenzoate (m-**5g**)

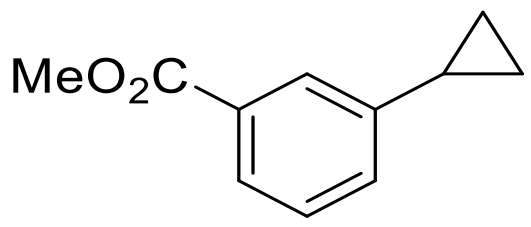

Following the general procedure A methyl 3-bromobenzoate (860 mg, 4.00 mmol, 1.0 equiv.), yielding methyl 3-cyclopropylbenzoate (*m*-**5g**) as a clear oil (680 mg, 1.89 mmol, 96%).

R_f (Pentane:Et_2O = 8:2) = 0.15.
^{1}H NMR (400 MHz, $CDCl_3$) δ 7.81 (dt, *J* = 7.5, 1.6 Hz, 1H, Ar*H*), 7.73 (t, *J* = 1.8 Hz, 1H, Ar*H*), 7.35 – 7.27 (m, 2H, Ar*H*), 3.91 (s, 3H, CH_3), 1.94 (tt, *J* = 8.4, 5.1 Hz, 1H, C*H*), 1.05 – 0.94 (m, 2H, CH_2), 0.78 – 0.70 (m, 2H, CH_2).
^{13}C NMR (101 MHz, $CDCl_3$) δ 167.4, 144.6, 130.6, 130.3, 128.4, 126.8, 126.8, 52.2, 15.4, 9.5.
HRMS (APCI/QTOF) m/z: $[M + H]^+$ Calcd for $C_{11}H_{13}O_2^+$ 177.0910; Found 177.0913.
NMR data consistent with reported value.[10]

Methyl 4-cyclopropylbenzoate (p-**5g**)

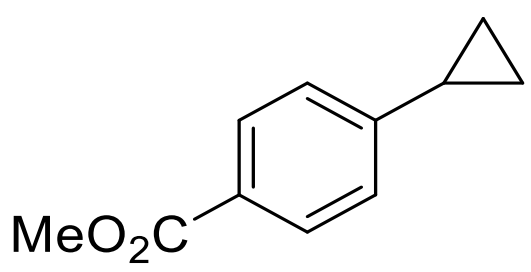

Following the general procedure A using methyl 4-bromobenzoate (860 mg, 4.00 mmol, 1.0 equiv.), yielding methyl 4-cyclopropylbenzoate (*p*-**5g**) as a clear oil (685 mg, 3.89 mmol, 97%).

R_f (Pentane:Et_2O = 8:2) = 0.15.
^{1}H NMR (400 MHz, $CDCl_3$) δ 7.98 – 7.86 (m, 2H, Ar*H*), 7.16 – 7.04 (m, 2H, Ar*H*), 3.89 (s, 3H, CH_3), 1.94 (tt, *J* = 8.4, 5.0 Hz, 1H, C*H*), 1.09 – 1.00 (m, 2H, CH_2), 0.84 – 0.72 (m, 2H, CH_2).
^{13}C NMR (101 MHz, $CDCl_3$) δ 167.3, 150.1, 129.8, 127.4, 125.5, 52.1, 15.8, 10.4.
HRMS (Sicrit plasma/LTQ-Orbitrap) m/z: $[M + H]^+$ Calcd for $C_{11}H_{13}O_2^+$ 177.0910;

Found 177.0909.
NMR data consistent with reported value.[8]

1-(2-Cyclopropylphenyl)ethan-1-one (o-**5h**)

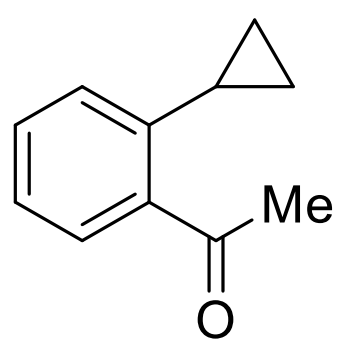

Following the general procedure A using 1-(2-bromophenyl)ethanone (796 mg, 4.00 mmol, 1.0 equiv.), yielding 1-(2-cyclopropylphenyl)ethan-1-one (*o*-**5h**) as a clear oil (575 mg, 3.59 mmol, 90%).

R$_f$ (Pentane:Et$_2$O = 95:5) = 0.22.
^{1}H NMR (400 MHz, CDCl$_3$) δ 7.52 (dd, *J* = 7.7, 1.4 Hz, 1H, Ar*H*), 7.36 (td, *J* = 7.6, 1.4 Hz, 1H, Ar*H*), 7.21 (td, *J* = 7.6, 1.2 Hz, 1H, Ar*H*), 7.04 (dd, *J* = 7.8, 1.2 Hz, 1H, Ar*H*), 2.62 (s, 3H, C*H$_3$*), 2.40 (tt, *J* = 8.5, 5.4 Hz, 1H, C*H*), 1.06 – 0.90 (m, 2H, C*H$_2$*), 0.82 – 0.55 (m, 2H, C*H$_2$*).
^{13}C NMR (101 MHz, CDCl$_3$) δ 203.5, 142.4, 140.5, 131.3, 128.1, 126.3, 125.5, 30.6, 13.6, 9.0.
HRMS (Sicrit plasma/LTQ-Orbitrap) m/z: [M + H]$^+$ Calcd for $C_{11}H_{13}O^+$ 161.0961; Found 161.0955.
NMR data consistent with reported value.[11]

1-(3-Cyclopropylphenyl)ethan-1-one (m-**5h**)

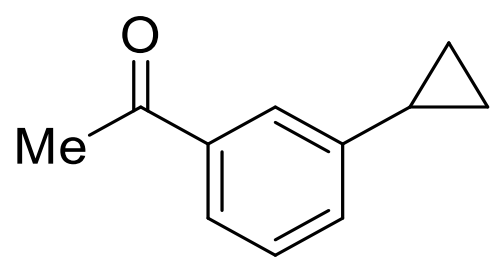

Following the general procedure A using 1-(3-bromophenyl)ethanone (796 mg, 4.00 mmol, 1.0 equiv.), yielding 1-(3-cyclopropylphenyl)ethan-1-one (*m*-**5h**) as a clear oil (614 mg, 3.83 mmol, 96%).

R$_f$ (Pentane:Et$_2$O = 95:5) = 0.22.
^{1}H NMR (400 MHz, CDCl$_3$) δ 7.72 (dt, *J* = 7.6, 1.5 Hz, 1H, Ar*H*), 7.67 (t, *J* = 1.9 Hz, 1H, Ar*H*), 7.34 (t, *J* = 7.6 Hz, 1H, Ar*H*), 7.30 – 7.24 (m, 1H, Ar*H*), 2.59 (s, 3H, C*H$_3$*), 1.95 (tt, *J* = 8.4, 5.1 Hz, 1H, C*H*), 1.06 – 0.97 (m, 2H, C*H$_2$*), 0.78 – 0.70 (m, 2H, C*H$_2$*).
^{13}C NMR (101 MHz, CDCl$_3$) δ 198.5, 144.8, 137.3, 130.6, 128.6, 125.7, 125.55, 26.8, 15.5, 9.5.
HRMS (APCI/QTOF) m/z: [M + H]$^+$ Calcd for $C_{11}H_{13}O^+$ 161.0961; Found 161.0959.
NMR data consistent with reported value.[9]

1-(4-Cyclopropylphenyl)ethan-1-one (p-**5h**)

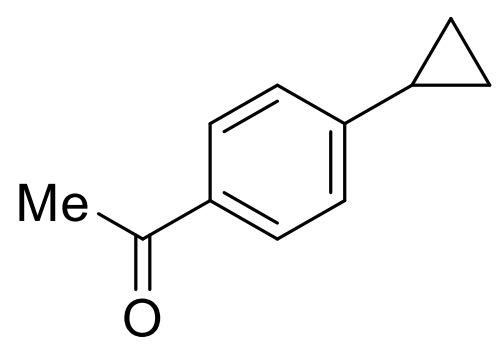

Following the general procedure A using 1-(4-bromophenyl)ethanone (796 mg, 4.00 mmol, 1.0 equiv.), yielding 1-(4-cyclopropylphenyl)ethan-1-one (*p*-**5h**) as a clear oil (593 mg, 3.70 mmol, 93%).

R$_f$ (Pentane:Et_2O = 95:5) = 0.22.
^{1}H NMR (400 MHz, $CDCl_3$) δ 7.89 – 7.80 (m, 2H, Ar*H*), 7.16 – 7.09 (m, 2H, Ar*H*), 2.57 (s, 3H, CH_3), 1.95 (tt, *J* = 8.4, 5.0 Hz, 1H, C*H*), 1.11 – 1.02 (m, 2H, CH_2), 0.82 – 0.74 (m, 2H, CH_2).
^{13}C NMR (101 MHz, $CDCl_3$) δ 197.8, 150.5, 134.7, 128.6, 125.6, 26.6, 15.9, 10.5.
HRMS (APCI/QTOF) m/z: $[M + H]^+$ Calcd for $C_{11}H_{13}O^+$ 161.0961; Found 161.0965.
NMR data consistent with reported value.[6]

1-Cyclopropyl-2-(trifluoromethyl)benzene (o-**5i**)

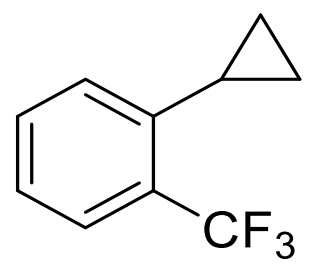

Following the general procedure A using 1-bromo-2-(trifluoromethyl)benzene (964 mg, 4.00 mmol, 1.0 equiv.), yielding 1-cyclopropyl-2-(trifluoromethyl)benzene (*o*-**5i**) as a clear oil (470 mg, 2.52 mmol, 63%).

R$_f$ (Pentane) = 0.64.
H NMR (400 MHz, $CDCl_3$) δ 7.53 – 7.38 (m, 1H, Ar*H*), 7.32 – 7.22 (m, 1H, Ar*H*), 7.08 (tt, *J* = 7.6, 1.2 Hz, 1H, Ar*H*), 6.89 (d, *J* = 7.9 Hz, 1H, Ar*H*), 2.09 (ddtt, *J* = 10.4, 7.0, 3.5, 1.8 Hz, 1H, C*H*), 0.95 – 0.83 (m, 2H, CH_2), 0.63 (dt, *J* = 6.7, 4.7 Hz, 2H, CH_2).
^{19}F NMR (376 MHz, $CDCl_3$) δ -60.2.
^{13}C NMR (101 MHz, $CDCl_3$) δ 142.4 (q, *J* = 1.7 Hz), 132.0 (d, *J* = 1.4 Hz), 129.8 (q, *J* = 29.7 Hz), 125.9, 125.9 (q, *J* = 5.9 Hz), 125.5, 125.0 (q, *J* = 273.8 Hz), 12.4 (q, *J* = 2.5 Hz), 8.9.
IR (v_{max}, cm^{-1}) 1313 (s), 1281 (m), 1154 (s), 1111 (s), 1042 (s), 901 (w), 763 (s), 751 (m).
HRMS (Sicrit plasma/LTQ-Orbitrap) m/z: $[M]^+$ Calcd for $C_{10}H_9F_3^+$ 186.0651; Found 186.0651.

1-Cyclopropyl-3-(trifluoromethyl)benzene (o-**5i**)

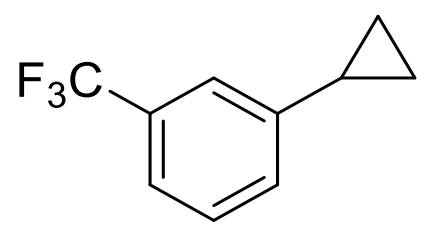

Following the general procedure A using 1-bromo-2-(trifluoromethyl)benzene (964 mg, 4.00 mmol, 1.0 equiv.), yielding 1-cyclopropyl-3-(trifluoromethyl)benzene (*m*-**5i**) as a clear oil (410 mg, 2.20 mmol, 55%).

R$_f$ (Pentane) = 0.64.
^{1}H NMR (400 MHz, $CDCl_3$) δ 7.32 – 7.19 (m, 3H, Ar*H*), 7.15 – 7.10 (m, 1H, Ar*H*), 1.84 (tt, *J* = 8.4, 5.1 Hz, 1H, C*H*), 0.98 – 0.82 (m, 2H, CH_2), 0.62 (dt, *J* = 6.6, 4.8 Hz, 2H, CH_2).
^{19}F NMR (376 MHz, $CDCl_3$) δ -62.6.
^{13}C NMR (101 MHz, $CDCl_3$) δ 145.2, 130.8 (q, *J* = 31.9 Hz), 129.1 (d, *J* = 1.4 Hz), 128.8, 124.5 (q, *J* = 272.2 Hz), 122.7 (q, *J* = 3.8 Hz), 122.3 (q, *J* = 3.9 Hz), 15.5, 9.6.
HRMS (Sicrit plasma/LTQ-Orbitrap) m/z: $[M]^+$ Calcd for $C_{10}H_9F_3^+$ 186.0651; Found 186.0651.
NMR data consistent with reported value.[10]

1-Cyclopropyl-4-(trifluoromethyl)benzene (p-**5i**)

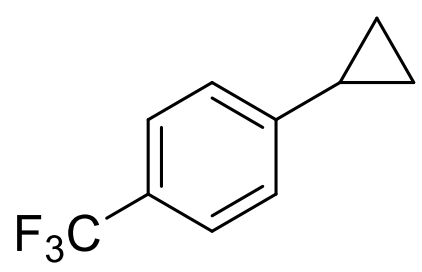

Following the general procedure A using 1-bromo-2-(trifluoromethyl)benzene (964 mg, 4.00 mmol, 1.0 equiv.), yielding 1-cyclopropyl-4-(trifluoromethyl)benzene (*p*-**5i**) as a clear oil (570 mg, 2.82 mmol, 70%).

R$_f$ (Pentane) = 0.64.
^{1}H NMR (400 MHz, $CDCl_3$) δ 7.49 (d, *J* = 8.1 Hz, 2H, Ar*H*), 7.15 (d, *J* = 8.1 Hz, 2H, Ar*H*), 1.95 (tt, *J* = 8.5, 5.0 Hz, 1H, C*H*), 1.08 – 1.01 (m, 2H, CH_2), 0.75 (dt, *J* = 6.8, 4.7 Hz, 2H, CH_2).
^{19}F NMR (376 MHz, $CDCl_3$) δ -58.0.
^{13}C NMR (101 MHz, $CDCl_3$) δ 148.5, 127.7 (q, *J* = 32.3 Hz), 125.9, 125.3 (q, *J* = 3.9 Hz), 124.8 (q, *J* = 271.8 Hz), 15.5, 10.1.
HRMS (Sicrit plasma/LTQ-Orbitrap) m/z: $[M]^-$ Calcd for $C_{10}H_9F_3^-$ 186.0662; Found 186.0654.
NMR data consistent with reported value.[10]

2-Cyclopropylbenzonitrile (o-**5j**)

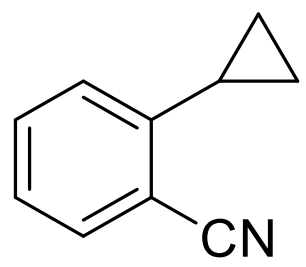

Following the general procedure A using 2-bromobenzonitrile (728 mg, 4.00 mmol, 1.0 equiv.), yielding 2-cyclopropylbenzonitrile(*o*-**5j**) as a clear oil (486 mg, 3.39 mmol, 85%).

R$_f$ (Pentane:Et_2O = 95:5) = 0.25.
^{1}H NMR (400 MHz, $CDCl_3$) δ 7.58 (dd, *J* = 7.7, 1.4 Hz, 1H, Ar*H*), 7.46 (td, *J* = 7.8, 1.4

Hz, 1H, Ar*H*), 7.22 (td, *J* = 7.6, 1.2 Hz, 1H, Ar*H*), 6.94 (d, *J* = 8.0 Hz, 1H, Ar*H*), 2.28 (tt, *J* = 8.4, 5.1 Hz, 1H, C*H*), 1.22 – 1.05 (m, 2H, C*H*$_2$), 0.80 (dt, *J* = 6.8, 4.8 Hz, 2H, C*H*$_2$).
^{13}C NMR (101 MHz, $CDCl_3$) δ 148.0, 132.9, 132.7, 125.9, 124.4, 118.5, 113.2, 14.2, 9.7.
HRMS (Sicrit plasma/LTQ-Orbitrap) m/z: $[M + H]^+$ Calcd for $C_{10}H_{10}N^+$ 144.0808; Found 144.0807.
NMR data consistent with reported value.[12]

3-Cyclopropylbenzonitrile (m-**5j**)

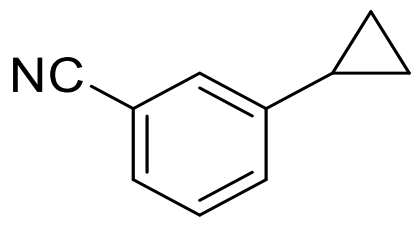

Following the general procedure A using 3-iodobenzonitrile (916 mg, 4.00 mmol, 1.0 equiv.), yielding 3-cyclopropylbenzonitrile(*m*-**5j**) as a clear oil (469 mg, 3.27 mmol, 82%).

R$_f$ (Pentane:Et_2O = 95:5) = 0.25.
^{1}H NMR (400 MHz, $CDCl_3$) δ 7.42 (dt, *J* = 7.7, 1.4 Hz, 1H, Ar*H*), 7.36 – 7.27 (m, 3H, Ar*H*), 1.92 (tt, *J* = 8.4, 5.1 Hz, 1H, C*H*), 1.09 – 1.00 (m, 2H, C*H*$_2$), 0.79 – 0.68 (m, 2H, C*H*$_2$).
^{13}C NMR (101 MHz, $CDCl_3$) δ 145.7, 130.4, 129.4, 129.2, 129.1, 119.2, 112.4, 15.3, 9.8.
HRMS (ESI/QTOF) m/z: $[M + H]^+$ Calcd for $C_{10}H_{10}N^+$ 144.0808; Found 144.0807.
NMR data consistent with reported value.[13]

4-Cyclopropylbenzonitrile (p-**5j**)

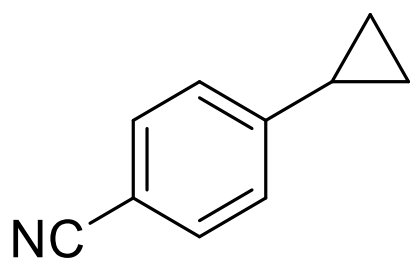

Following the general procedure A using 4-bromobenzonitrile (728 mg, 4.00 mmol, 1.0 equiv.), yielding 4-cyclopropylbenzonitrile(*p*-**5j**) as a clear oil (502 mg, 3.50 mmol, 88%).

R$_f$ (Pentane:Et_2O = 95:5) = 0.25.
^{1}H NMR (400 MHz, $CDCl_3$) δ 7.59 – 7.45 (m, 2H, Ar*H*), 7.16 – 7.03 (m, 2H, Ar*H*), 1.93 (tt, *J* = 8.4, 5.0 Hz, 1H, C*H*), 1.16 – 1.01 (m, 2H, C*H*$_2$), 0.83 – 0.71 (m, 2H, C*H*$_2$).
^{13}C NMR (101 MHz, $CDCl_3$) δ 150.3, 132.2, 126.3, 119.4, 109.0, 16.0, 10.7.
HRMS (APCI/QTOF) m/z: $[M + H]^+$ Calcd for $C_{10}H_{10}N^+$ 144.0808; Found 144.0807.
NMR data consistent with reported value.[11]

1-Cyclopropyl-2-(trifluoromethoxy)benzene (o-**5k**)

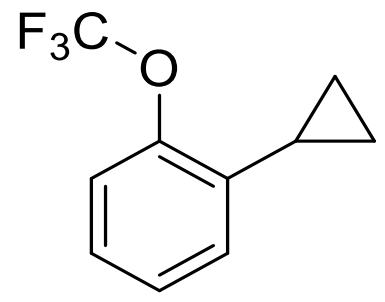

Following the general procedure A using using 1-bromo-2-(trifluoromethoxy)benzene (964 mg, 4.00 mmol, 1.0 equiv.), yielding 1-cyclopropyl-2-(trifluoromethoxy)benzene (*o*-**5k**) as a clear oil (756 mg, 3.74 mmol, 93%).

R_f (Pentane) = 0.52.
^{1}H NMR (400 MHz, $CDCl_3$) δ 7.25 (t, *J* = 8.0 Hz, 1H, Ar*H*), 6.99 (dd, *J* = 7.8, 1.7 Hz, 2H, Ar*H*), 6.90 (s, 1H, Ar*H*), 1.91 (tt, *J* = 8.5, 5.1 Hz, 1H, C*H*), 1.06 – 0.94 (m, 2H, CH_2), 0.71 (dt, *J* = 6.9, 4.8 Hz, 2H, CH_2).
^{19}F NMR (376 MHz, $CDCl_3$) δ -57.7.
^{13}C NMR (101 MHz, $CDCl_3$) δ 149.5, 146.6, 129.4, 124.0, 120.5 (q, *J* = 255.9 Hz), 118.3, 117.7, 15.3, 9.6.
HRMS (APCI/QTOF) m/z: $[M + H]^+$ Calcd for $C_{10}H_{10}F_3O^+$ 203.0678; Found 203.0678.
NMR data consistent with reported value.[14]

1-Cyclopropyl-3-(trifluoromethoxy)benzene (m-**5k**)

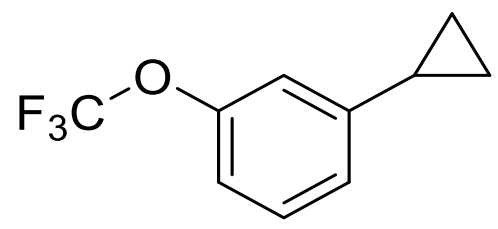

Following the general procedure A using 1-bromo-3-(trifluoromethoxy)benzene (964 mg, 4.00 mmol, 1.0 equiv.), yielding 1-cyclopropyl-3-(trifluoromethoxy)benzene(*m*-**5k**) as a clear oil (657 mg, 3.25 mmol, 81%).

R_f (Pentane) = 0.52.
^{1}H NMR (400 MHz, $CDCl_3$) δ 7.25 (t, *J* = 7.9 Hz, 1H, Ar*H*), 6.99 (ddd, *J* = 8.0, 2.9, 1.4 Hz, 2H, Ar*H*), 6.90 (dq, *J* = 2.2, 1.2 Hz, 1H, Ar*H*), 1.91 (tt, *J* = 8.4, 5.1 Hz, 1H, C*H*), 1.06 – 0.95 (m, 2H, CH_2), 0.75 – 0.67 (m, 2H, CH_2).
^{19}F NMR (376 MHz, $CDCl_3$) δ -57.7.
^{13}C NMR (101 MHz, $CDCl_3$) δ 149.6 (d, *J* = 1.8 Hz), 146.8, 129.6, 124.2, 120.6 (q, *J* = 256.7 Hz), 118.4, 117.8, 15.4, 9.7.
HRMS (APCI/QTOF) m/z: $[M + H]^+$ Calcd for $C_{10}H_{10}F_3O^+$ 203.0678; Found 203.0677.
NMR data consistent with reported value.[14]

1-Cyclopropyl-4-(trifluoromethoxy)benzene (p-**5k**)

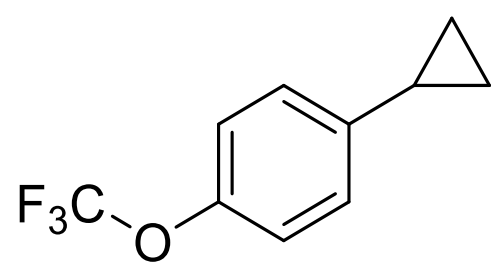

Following the general procedure A 1-bromo-4-(trifluoromethoxy)benzene (964 mg, 4.00 mmol, 1.0 equiv.), yielding 1-cyclopropyl-4-(trifluoromethoxy)benzene(*p*-**5k**) as a clear oil (694 mg, 3.43 mmol, 86%).

R_f (Pentane) = 0.52.
^{1}H NMR (400 MHz, $CDCl_3$) δ 7.13 – 7.05 (m, 4H, Ar*H*), 1.90 (tt, *J* = 8.4, 5.1 Hz, 1H, C*H*), 1.01 – 0.95 (m, 2H, C*H_2*), 0.73 – 0.63 (m, 2H, C*H_2*).
^{19}F NMR (376 MHz, $CDCl_3$) δ -58.0.
^{13}C NMR (101 MHz, $CDCl_3$) δ 147.2 (d, *J* = 1.9 Hz), 142.9, 127.0, 121.0, 120.7 (q, *J* = 256.3 Hz), 15.0, 9.5.
HRMS (Sicrit plasma/LTQ-Orbitrap) m/z: $[M + H]^+$ Calcd for $C_{10}H_{10}F_3O^+$ 203.0678; Found 203.0677.
NMR data consistent with reported value.[6]

1-Chloro-2-cyclopropylbenzene (o-**5l**)

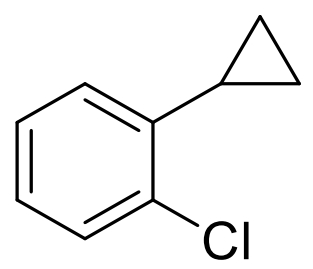

Following the general procedure A using 1-bromo-2-chlorobenzene (766 mg, 2.00 mmol, 1.0 equiv.), yielding 1-chloro-2-cyclopropylbenzene (*o*-**5l**) as a clear oil (490 mg, 3.21 mmol, 80%).

R_f (Pentane) = 0.68.
^{1}H NMR (400 MHz, $CDCl_3$) δ 7.34 (dd, *J* = 7.8, 1.5 Hz, 1H, Ar*H*), 7.16 (td, *J* = 7.6, 1.6 Hz, 1H, Ar*H*), 7.09 (td, *J* = 7.6, 1.8 Hz, 1H, Ar*H*), 6.93 (dd, *J* = 7.6, 1.8 Hz, 1H, Ar*H*), 2.20 (tt, *J* = 8.5, 5.3 Hz, 1H, C*H*), 1.09 – 0.96 (m, 2H, C*H_2*), 0.77 – 0.62 (m, 2H, C*H_2*).
^{13}C NMR (101 MHz, $CDCl_3$) δ 141.0, 135.4, 129.3, 126.8, 126.7, 126.2, 13.4, 8.1.
HRMS (APCI/QTOF) m/z: $[M + H]^+$ Calcd for $C_9H_{10}Cl^+$ 153.0466; Found 153.0463.
NMR data consistent with reported value.[6]

1-Chloro-3-cyclopropylbenzene (m-**5l**)

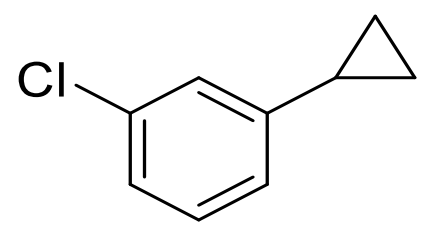

Following the general procedure A 1-bromo-3-chlorobenzene (766 mg, 2.00 mmol, 1.0 equiv.), yielding 1-chloro-3-cyclopropylbenzene (*m*-**5l**) as a clear oil (470 mg, 3.08 mmol, 77%).

R_f (Pentane) = 0.68.
^{1}H NMR (400 MHz, $CDCl_3$) δ 7.17 (t, *J* = 7.7 Hz, 1H, Ar*H*), 7.11 (dt, *J* = 8.0, 1.5 Hz, 1H, Ar*H*), 7.04 (t, *J* = 1.9 Hz, 1H, Ar*H*), 6.95 (dt, *J* = 7.5, 1.5 Hz, 1H, Ar*H*), 1.87 (tt, *J* = 8.5, 5.1 Hz, 1H, C*H*), 1.02 – 0.94 (m, 2H, C*H_2*), 0.70 (dt, *J* = 6.8, 4.8 Hz, 2H, C*H_2*).
^{13}C NMR (101 MHz, $CDCl_3$) δ 146.4, 134.3, 129.6, 125.9, 125.6, 124.1, 15.4, 9.5.
HRMS (APCI/QTOF) m/z: $[M + H]^+$ Calcd for $C_9H_{10}Cl^+$ 153.0466; Found 153.0393.
NMR data consistent with reported value.[6]

1-Chloro-4-cyclopropylbenzene (p-**5l**)

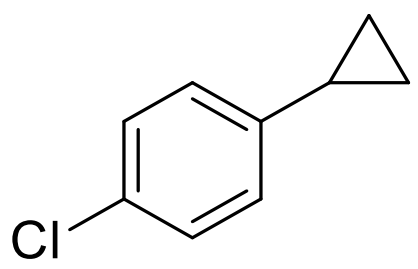

Following the general procedure A using 1-bromo-4-chlorobenzene (766 mg, 2.00 mmol, 1.0 equiv.), yielding 1-chloro-4-cyclopropylbenzene (*p*-**5l**) as a clear oil (584 mg, 3.83 mmol, 96%).

R_f (Pentane) = 0.68.
^{1}H NMR (400 MHz, $CDCl_3$) δ 7.24 – 7.15 (m, 2H, Ar*H*), 7.03 – 6.95 (m, 2H, Ar*H*), 1.86 (tt, *J* = 8.5, 5.0 Hz, 1H, C*H*), 1.02 – 0.93 (m, 2H, CH_2), 0.66 (dt, *J* = 6.7, 4.8 Hz, 2H, CH_2).
^{13}C NMR (101 MHz, $CDCl_3$) δ 142.6, 131.0, 128.4, 127.2, 125.8, 15.1, 9.4.
HRMS (Sicrit plasma/LTQ-Orbitrap) m/z: $[M]^+$ Calcd for $C_9H_9Cl^+$ 152.0387; Found 152.0388.
NMR data consistent with reported value.[6]

1-Ethenyl-2-fluorobenzene (**S1**)

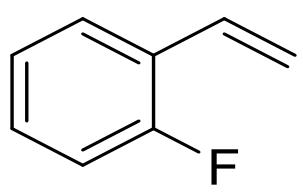

Following the general procedure B using 2-fluorobenzaldehyde (1991 mg, 16.00 mmol, 1.0 equiv.), yielding 1-ethenyl-2-fluorobenzene (**S1**) as a clear oil (1373 mg, 11.20 mmol, 70%).

R_f (Pentane) = 0.72.
^{1}H NMR (400 MHz, $CDCl_3$) δ 7.49 (ddd, *J* = 8.5, 7.1, 1.4 Hz, 1H, Ar*H*), 7.21 (ddd, *J* = 7.8, 5.4, 1.7 Hz, 1H, Ar*H*), 7.10 (t, *J* = 7.5 Hz, 1H, Ar*H*), 7.06 – 6.97 (m, 1H, Ar*H*), 6.88 (dd, *J* = 17.8, 11.2 Hz, 1H, ArC*H*), 5.82 (dd, *J* = 17.8, 1.3 Hz, 1H, CH_2), 5.37 (d, *J* = 11.1 Hz, 1H, CH_2).
^{19}F NMR (376 MHz, $CDCl_3$) δ -118.7.
^{13}C NMR (101 MHz, $CDCl_3$) δ 160.5 (d, *J* = 249.5 Hz), 129.5 (d, *J* = 4.0 Hz), 129.2 (d, *J* = 8.4 Hz), 127.2 (d, *J* = 3.8 Hz), 125.5 (d, *J* = 12.1 Hz), 124.2 (d, *J* = 3.5 Hz), 116.6 (d, *J* = 4.7 Hz), 115.8 (d, *J* = 22.1 Hz).
HRMS (Sicrit plasma/LTQ-Orbitrap) m/z: $[M + H]^+$ Calcd for $C_8H_8F^+$ 123.0605; Found 123.0604.
NMR data consistent with reported value.[15]

1-Cyclopropyl-2-fluorobenzene (o-**5m**)

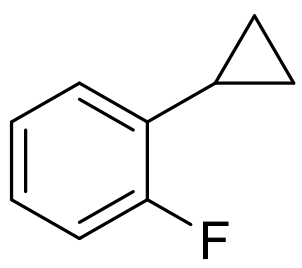

Following the general procedure C using 1-ethenyl-2-fluorobenzene (**S1**, 733 mg, 6.00 mmol, 1.0 equiv.), yielding 1-cyclopropyl-2-fluorobenzene (*o*-**5m**) as a clear oil (565 mg, 4.15 mmol, 69%).

R_f (Pentane) = 0.74.
^{1}H NMR (400 MHz, $CDCl_3$) δ 7.16 – 7.08 (m, 1H, Ar*H*), 7.01 (dddd, *J* = 11.8, 9.4, 7.7, 1.4 Hz, 2H, Ar*H*), 6.90 (td, *J* = 7.6, 1.8 Hz, 1H, Ar*H*), 2.09 (tt, *J* = 8.5, 5.2 Hz, 1H, C*H*), 1.03 – 0.94 (m, 2H, CH_2), 0.84 – 0.65 (m, 2H, CH_2).
^{19}F NMR (376 MHz, $CDCl_3$) δ -120.3.
^{13}C NMR (101 MHz, $CDCl_3$) δ 162.0 (d, *J* = 244.5 Hz), 130.8 (d, *J* = 14.2 Hz), 126.7 (d, *J* = 8.0 Hz), 126.1 (d, *J* = 4.4 Hz), 124.0 (d, *J* = 3.6 Hz), 115.1 (d, *J* = 22.3 Hz), 8.8 (d, *J* = 5.4 Hz), 7.9.
IR (v_{max}, cm^{-1}) 1615 (m), 1587 (s), 1491 (m), 1142 (m), 940 (m), 872 (m), 853 (m), 777 (s)
HRMS (Sicrit plasma/LTQ-Orbitrap) m/z: $[M + H]^+$ Calcd for $C_9H_{10}F^+$ 137.0761; Found 137.0761.

1-Ethenyl-3-fluorobenzene (**S2**)

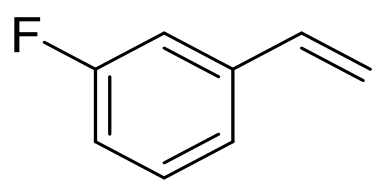

Following the general procedure B using 3-fluorobenzaldehyde (2482 mg, 20.00 mmol, 1.0 equiv.), yielding 1-ethenyl-3-fluorobenzene (**S2**) as a clear oil (904 mg, 7.00 mmol, 37%).

R_f (Pentane) = 0.72.
^{1}H NMR (400 MHz, $CDCl_3$) δ 7.33 – 7.26 (m, 1H, Ar*H*), 7.20 – 7.08 (m, 2H, Ar*H*), 6.99 – 6.90 (m, 1H, Ar*H*), 6.68 (dd, *J* = 17.6, 10.8 Hz, 1H, C*H*), 5.76 (d, *J* = 17.6 Hz, 1H, CH_2), 5.30 (d, *J* = 10.8 Hz, 1H, CH_2).
^{19}F NMR (376 MHz, $CDCl_3$) δ -113.7.
^{13}C NMR (101 MHz, $CDCl_3$) δ 163.3 (d, *J* = 245.2 Hz), 140.1 (d, *J* = 7.5 Hz), 136.0 (d, *J* = 2.7 Hz), 130.1 (d, *J* = 8.3 Hz), 122.3 (d, *J* = 2.7 Hz), 115.3, 114.8 (d, *J* = 21.3 Hz), 112.8 (d, *J* = 21.7 Hz).
HRMS (Sicrit plasma/LTQ-Orbitrap) m/z: $[M + H]^+$ Calcd for $C_8H_8F^+$ 123.0605; Found 123.0604. Found 123.0604.
NMR data consistent with reported value.[16]

1-Cyclopropyl-3-fluorobenzene (m-**5m**)

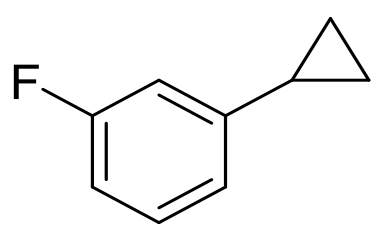

Following the general procedure C using 1-ethenyl-3- fluorobenzene (733 mg, 6.00 mmol, 1.0 equiv.), yielding 1-cyclopropyl-3-fluorobenzene (*m*-**5m**) as a clear oil (344 mg, 2.53 mmol, 42%).

R$_f$ (Pentane) = 0.75.
^{1}H NMR (400 MHz, $CDCl_3$) δ 7.19 (td, *J* = 8.0, 6.1 Hz, 1H, Ar*H*), 6.90 – 6.78 (m, 2H, Ar*H*), 6.78 – 6.67 (m, 1H, Ar*H*), 1.89 (tt, *J* = 8.4, 5.0 Hz, 1H, C*H*), 1.05 – 0.88 (m, 2H, CH_2), 0.77 – 0.58 (m, 2H, CH_2).
^{19}F NMR (376 MHz, $CDCl_3$) δ -114.1.
^{13}C NMR (101 MHz, $CDCl_3$) δ 163.2 (d, *J* = 244.7 Hz), 147.0 (d, *J* = 7.6 Hz), 129.7 (d, *J* = 8.7 Hz), 121.6 (d, *J* = 2.7 Hz), 112.5 (d, *J* = 15.3 Hz), 112.3 (d, *J* = 15.1 Hz), 15.4 (d, *J* = 2.2 Hz), 9.7.
HRMS (Sicrit plasma/LTQ-Orbitrap) m/z: $[M + H]^+$ Calcd for $C_9H_{10}F^+$ 137.0761; Found 137.0761.
NMR data consistent with reported value.[6]

1-Cyclopropyl-4-fluorobenzene (p-**5m**)

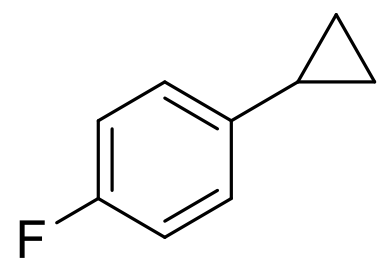

Following the general procedure C using 1-ethenyl-4-fluorobenzene (611 mg, 5.00 mmol, 1.0 equiv.), yielding 1-cyclopropyl-4-fluorobenzene (*p*-**5m**) as a clear oil (286 mg, 2.10 mmol, 42%).

R$_f$ (Pentane) = 0.74.
^{1}H NMR (400 MHz, $CDCl_3$) δ 7.08 – 6.99 (m, 2H, Ar*H*), 6.97 – 6.89 (m, 2H, Ar*H*), 1.88 (tt, *J* = 8.4, 5.1 Hz, 1H, C*H*), 0.97 – 0.91 (m, 2H, CH_2), 0.63 (dt, *J* = 6.6, 4.7 Hz, 2H, CH_2).
^{19}F NMR (376 MHz, $CDCl_3$) δ -118.3.
^{13}C NMR (101 MHz, $CDCl_3$) δ 161.2 (d, *J* = 242.7 Hz), 139.6 (d, *J* = 3.0 Hz), 127.3 (d, *J* = 7.7 Hz), 115.1 (d, *J* = 21.3 Hz), 14.9, 9.0.
HRMS (Sicrit plasma/LTQ-Orbitrap) m/z: $[M + H]^+$ Calcd for $C_9H_{10}F^+$ 137.0761; Found 137.0761.
NMR data consistent with reported value.[6]

1-Cyclopropylnaphthalene (**5n**)

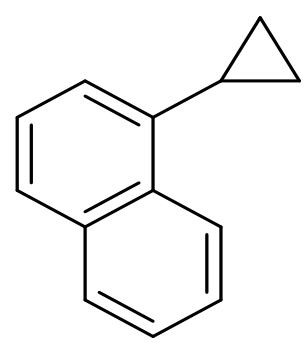

Following the general procedure A using 1-bromonaphthalene (828 mg, 4.00 mmol, 1.0 equiv.), yielding 1-cyclopropylnaphthalene (**5n**) as a clear oil (603 mg, 3.58 mmol, 90%).

R$_f$ (Pentane) = 0.70.
^{1}H NMR (400 MHz, $CDCl_3$) δ 8.42 (dq, *J* = 8.5, 0.9 Hz, 1H, Ar*H*), 7.86 (dd, *J* = 7.9, 1.6 Hz, 1H, Ar*H*), 7.71 (d, *J* = 8.1 Hz, 1H, Ar*H*), 7.52 (dddd, *J* = 21.1, 8.1, 6.8, 1.4 Hz,

2H, Ar*H*), 7.39 (dd, *J* = 8.2, 7.1 Hz, 1H, Ar*H*), 7.29 – 7.26 (m, 1H, Ar*H*), 2.35 (tt, *J* = 8.5, 5.4 Hz, 1H, C*H*), 1.11 – 1.03 (m, 2H, CH_2), 0.78 (td, *J* = 5.9, 4.2 Hz, 2H, CH_2).
^{13}C NMR (101 MHz, $CDCl_3$) δ 139.3, 133.7, 133.7, 128.6, 126.7, 125.8, 125.7, 125.6, 124.6, 123.9, 13.4, 6.5.
HRMS (Sicrit plasma/LTQ-Orbitrap) m/z: $[M + H]^+$ Calcd for $C_{13}H_{13}^+$ 169.1012; Found 169.1011.
NMR data consistent with reported value.[12]

2-Cyclopropylnaphthalene (**5o**)

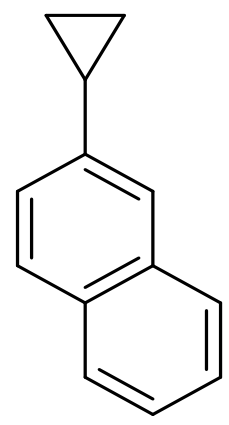

Following the general procedure A using 2-bromonaphthalene (828 mg, 4.00 mmol, 1.0 equiv.), yielding 2-cyclopropylnaphthalene (**5o**) as a clear oil (600 mg, 3.56 mmol, 89%).

R_f (Pentane) = 0.71.
^{1}H NMR (400 MHz, $CDCl_3$) δ 8.01 – 7.90 (m, 3H, Ar*H*), 7.74 (d, *J* = 1.8 Hz, 1H, Ar*H*), 7.62 (dddd, *J* = 19.0, 8.2, 6.8, 1.5 Hz, 2H, Ar*H*), 7.38 (dd, *J* = 8.5, 1.9 Hz, 1H, Ar*H*), 2.24 (tt, *J* = 8.4, 5.1 Hz, 1H, C*H*), 1.26 – 1.18 (m, 2H, CH_2), 1.06 – 1.00 (m, 2H, CH_2).
^{13}C NMR (101 MHz, $CDCl_3$) δ 141.6, 133.7, 132.0, 128.0, 127.7, 127.4, 126.1, 125.0, 124.8, 123.9, 15.8, 9.3.
HRMS (Sicrit plasma/LTQ-Orbitrap) m/z: $[M + H]^+$ Calcd for $C_{13}H_{13}^+$ 169.1012; Found 169.1011.
NMR data consistent with reported value.[13]

Methyl 4-bromo-1-naphthoate (**S3**)

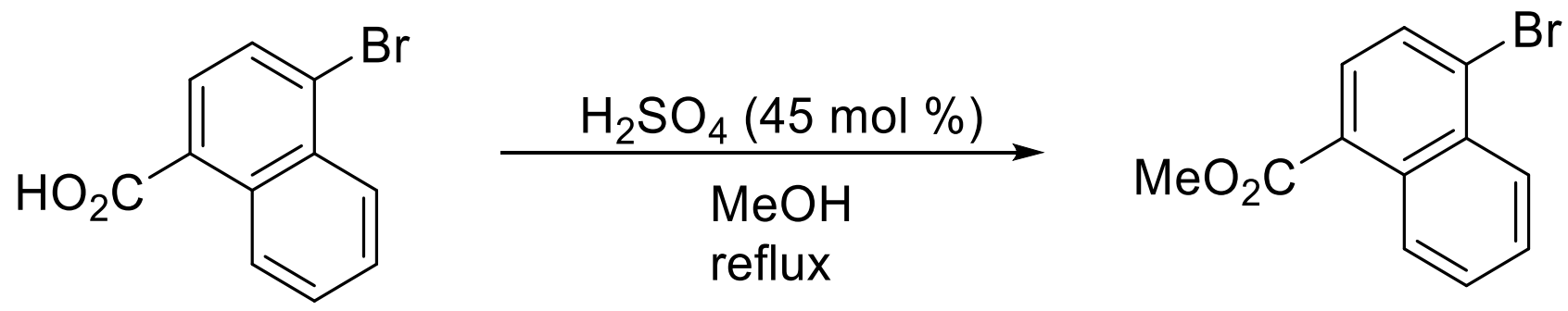

To a solution in MeOH (14.5 mL) of 4-bromo-1-naphthoic acid (1.00 g, 3.98 mmol, 1.0 equiv.), 0.1 mL of H_2SO_4 (1.87 mmol, 0.45 equiv.) were added dropwise. The mixture was refluxed for 23 h and the reaction was monitored by TLC. After complete conversion, the mixture was cooled to room temperature and NaOH aq 4 M was added until basic pH. The resulting mixture was extracted with 20 mL of AcOEt twice and the combined organic layers were washed with brine and dried over Na_2SO_4. The salt was removed by filtration and the solvents of the filtrate were removed under reduced pressure to give pure product **S3** (734 mg, 2.77 mmol, 70% yield).

R$_f$ (Pentane:Et_2O = 7:3) = 0.14.
^{1}H NMR (400 MHz, $CDCl_3$) δ 9.03 – 8.85 (m, 1H, Ar*H*), 8.44 – 8.26 (m, 1H, Ar*H*), 7.99 (d, *J* = 7.9 Hz, 1H, Ar*H*), 7.82 (d, *J* = 7.9 Hz, 1H, Ar*H*), 7.75 – 7.59 (m, 2H, Ar*H*), 4.00 (s, 3H, C*H$_3$*).
^{13}C NMR (101 MHz, $CDCl_3$) δ 167.6, 132.5, 132.3, 130.2, 129.0, 128.9, 128.6, 127.9, 127.8, 127.2, 126.4, 52.5.
HRMS (APCI/QTOF) m/z: $[M + H]^+$ Calcd for $C_{12}H_{10}BrO_2^+$ 264.9859; Found 264.9856.
NMR data consistent with reported value.[17]

Methyl 4-cyclopropyl-1-naphthoate (**5p**)

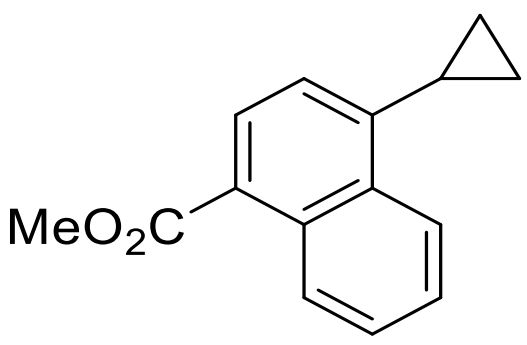

Following the general procedure A using methyl 4-bromonaphthalene-1-carboxylate **S3** (734 mg, 2.77 mmol, 1.0 equiv.), yielding methyl 4-cyclopropyl-1-naphthoate (**5p**) as a clear oil (478 mg, 2.11 mmol, 76%).

R$_f$ (Pentane:Et_2O = 8:2) = 0.19.
^{1}H NMR (400 MHz, $CDCl_3$) δ 9.06 – 8.83 (m, 1H, Ar*H*), 8.49 (dd, *J* = 8.2, 1.7 Hz, 1H, Ar*H*), 8.09 (d, *J* = 7.6 Hz, 1H, Ar*H*), 7.69 – 7.53 (m, 2H, Ar*H*), 7.28 – 7.24 (m, 1H, Ar*H*), 3.99 (d, *J* = 0.9 Hz, 3H, C*H$_3$*), 2.40 (ddd, *J* = 14.0, 8.5, 5.4 Hz, 1H, C*H*), 1.19 – 1.09 (m, 2H, C*H$_2$*), 0.86 – 0.77 (m, 2H, C*H$_2$*).
^{13}C NMR (101 MHz, $CDCl_3$) δ 168.3, 145.2, 133.9, 131.5, 130.2, 127.5, 126.4, 126.2, 125.6, 124.9, 122.5, 52.2, 14.0, 7.1.
IR (ν_{max}, cm^{-1}) 1712 (s), 1586 (m), 1514 (m), 1432 (m), 1279 (s), 1248 (s), 1230 (s), 1196 (s), 1130 (s), 1027 (s), 770 (s)
HRMS (ESI/QTOF) m/z: $[M + H]^+$ Calcd for $C_{15}H_{15}O_2^+$ 227.1067; Found 227.1070.

4-Cyclopropyl-2,3-dihydro-1H-inden-1-one (**5q**)

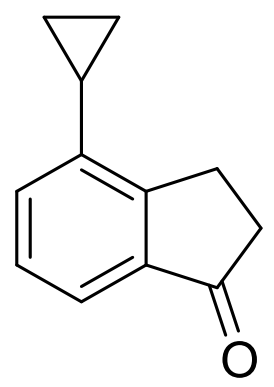

Following the general procedure A using 4-bromoindan-1-one (844 mg, 4.00 mmol, 1.0 equiv.), yielding 4-cyclopropyl-2,3-dihydro-1H-inden-1-one (**5q**) as a clear oil (615 mg, 3.57 mmol, 89%).

R$_f$ (Pentane:Et_2O = 95:5) = 0.28.
^{1}H NMR (400 MHz, $CDCl_3$) δ 7.58 (d, *J* = 7.6 Hz, 1H, Ar*H*), 7.29 (t, *J* = 7.6 Hz, 1H, Ar*H*), 7.13 (d, *J* = 7.5 Hz, 1H, Ar*H*), 3.33 – 3.11 (m, 2H, C*H$_2$*), 2.76 – 2.65 (m, 2H,

C*H*$_2$), 1.96 (tt, *J* = 8.5, 5.2 Hz, 1H, C*H*), 1.09 – 0.99 (m, 2H, C*H*$_2$), 0.79 – 0.66 (m, 2H, C*H*$_2$).
^{13}C NMR (101 MHz, $CDCl_3$) δ 207.7, 154.9, 141.6, 136.9, 129.7, 127.8, 121.0, 36.4, 24.7, 11.9, 7.9.
HRMS (ESI/QTOF) m/z: $[M + H]^+$ Calcd for $C_{12}H_{13}O^+$ 173.0961; Found 173.0960.
NMR data consistent with reported value.[18]

Prop-1-en-1-ylbenzene (**S4**)

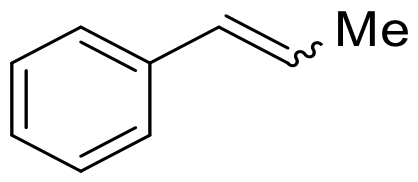

Following the general procedure D using Benzaldehyde (1.9 g, 18 mmol, 1.0 equiv.) ethyltriphenylphosphonium bromide (7.3 g, 20 mmol, 1.1 equiv.), yielding prop-1-en-1-ylbenzene (**S4**) as a clear oil (1.40 g, 11.8 mmol, 65%, mixture of inseparable diastereoisomers d.r. 6:4)

R_f (Pentane) = 0.75.
^{1}H NMR (400 MHz, $CDCl_3$, signals of the minor diastereoisomer (*cis*) are indicated in *italic*) δ 7.32 (dq, *J* = 16.1, 7.9 Hz, 4H, Ar*H*), 7.21 (dt, *J* = 14.1, 7.2 Hz, 1H, Ar*H*), 6.49 – 6.36 (m, 1H, ArC*H*), 6.24 (dq, *J* = 15.8, 6.5 Hz, 1H, C*H*CH_3), *5.80 (dq, J = 11.5, 7.2 Hz, 1H,* C*H*CH_3*)*, 1.90 (td, *J* = 7.7, 1.9 Hz, 3H, C*H*$_3$).
^{13}C NMR (101 MHz, $CDCl_3$, signals of the minor diastereoisomer (cis) are indicated in *italic*)) δ 138.1, *137.8*, 131.2, *130.0*, *129.0*, 128.6, *128.3*, 126.9, *126.9*, *126.5*, 126.0, 125.8, 18.6, *14.8*.
HRMS (Sicrit plasma/LTQ-Orbitrap) m/z: $[M + H]^+$ Calcd for $C_9H_{11}^+$ 119.0855; Found 119.0850.
NMR data consistent with reported value.[19]

(2-Methylcyclopropyl)benzene (**5r**)

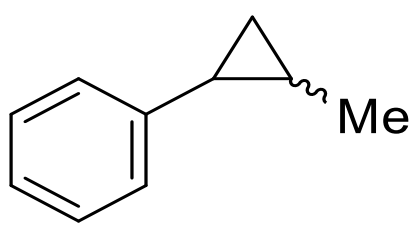

Following the general procedure D using **S4** (591 mg, 5.00 mmol, 1.0 equiv.), yielding (2-methylcyclopropyl)benzene (**5r**) as a clear oil (338 mg, 2.60 mmol, 51%, mixture of inseparable diastereoisomers, d.r. 6:4).

R_f (Pentane) = 0.76.
^{1}H NMR (400 MHz, $CDCl_3$, signals of the minor diastereoisomer (cis) are indicated in *italic*) δ 7.32 – 7.10 (m, 4H, Ar*H*), 7.08 – 7.03 (m, 1H, Ar*H*), *2.11 (td, J = 9.0, 6.1 Hz, 1H,* ArC*H)*, 1.60 (dt, *J* = 9.0, 4.8 Hz, 1H, ArC*H*), 1.21 (d, *J* = 5.9 Hz, 3H), *1.19 – 1.12 (m, 1H,* C*H*CH_3*)*, 1.12 – 1.04 (m, 1H), *1.00 (td, J = 8.4, 4.8 Hz, 1H,* C*H*$_2$*)*, 0.91 (dt, *J* = 8.3, 4.8 Hz, 1H), *0.82 (d, J = 6.2 Hz, 3H),* 0.76 (dt, *J* = 8.4, 5.1 Hz, 1H), *0.61 (q, J = 5.5 Hz, 1H,* C*H*$_2$*)*.
^{13}C NMR (101 MHz, $CDCl_3$, signals of the minor diastereoisomer (cis) are indicated

in *italic*) δ 144.2, *139.7*, *129.4*, 128.4, *128.0*, *125.7*, 125.6, 125.3, 24.5, *21.2*, 19.2, 18.1, 17.8, *13.7*, *12.8*, *11.0*.
HRMS (Sicrit plasma/LTQ-Orbitrap) m/z: $[M + H]^+$ Calcd for $C_{10}H_{13}^+$ 133.1012; Found 133.1012.
NMR data consistent with reported value.[5]

(E)-1,2-Diphenylcyclopropane (**5s**)

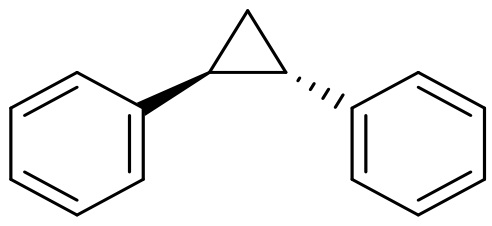

Following the general procedure D using (E)-stilbene (901 mg, 5.00 mmol, 1.0 equiv.), yielding trans-1,2-diphenylcyclopropane (**5s**) as a clear oil (941 mg, 4.84 mmol, 97%).

R_f (Pentane) = 0.75.
^{1}H NMR (400 MHz, $CDCl_3$) δ 7.35 – 7.26 (m, 4H, Ar*H*), 7.22 – 7.12 (m, 6H, Ar*H*), 2.23 – 2.14 (m, 2H, C*H*), 1.49 – 1.41 (m, 2H, C*H₂*).
^{13}C NMR (101 MHz, $CDCl_3$) δ 142.7, 128.5, 125.9, 125.9, 28.2, 18.3.
HRMS (Sicrit plasma/LTQ-Orbitrap) m/z: $[M + H]^+$ Calcd for $C_{15}H_{15}^+$ 195.1168; Found 195.1171.
NMR data consistent with reported value.[5]

1-Cyclopropyl-3,5-dimethylbenzene (**5t**)

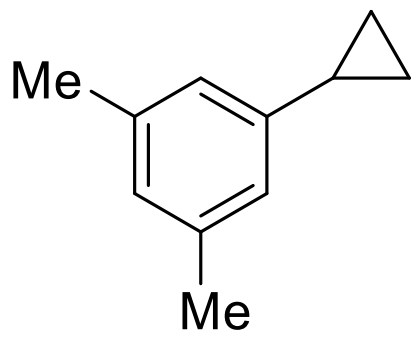

Following the general procedure A using 1-bromo-3,5-dimethylbenzene (740 mg, 4.00 mmol, 1.0 equiv.), yielding 1-cyclopropyl-3,5-dimethylbenzene (**5t**) as a clear oil (544 mg, 3.72 mmol, 93%).

R_f (Pentane) = 0.75.
^{1}H NMR (400 MHz, $CDCl_3$) δ 6.79 (tt, *J* = 1.7, 0.8 Hz, 1H, Ar*H*), 6.70 (d, *J* = 1.8 Hz, 2H, Ar*H*), 2.28 (s, 6H, CH_3), 1.83 (tt, *J* = 8.4, 5.1 Hz, 1H, C*H*), 0.96 – 0.89 (m, 2H, CH_2), 0.68 (dt, *J* = 6.6, 4.5 Hz, 2H, CH_2).
^{13}C NMR (101 MHz, $CDCl_3$) δ 144.0, 137.9, 127.2, 123.6, 21.4, 15.3, 9.1.
HRMS (Sicrit plasma/LTQ-Orbitrap) m/z: $[M + H]^+$ Calcd for $C_{11}H_{15}^+$ 147.1168; Found 147.1168.
NMR data consistent with reported value.[20]

Cyclopropane-1,1-diyldibenzene (**5u**)

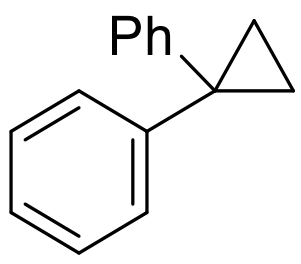

Following the general procedure D using 1,1-diphenylethylene (901 mg, 5.00 mmol, 1.0 equiv.), yielding cyclopropane-1,1-diyldibenzene (**5u**) as a clear oil (970 mg, 4.93 mmol, 100%).

R_f (Pentane) = 0.77.
^{1}H NMR (400 MHz, $CDCl_3$) δ 7.23 – 7.13 (m, 8H, Ar*H*), 7.13 – 7.07 (m, 2H, Ar*H*), 1.23 (d, *J* = 0.6 Hz, 4H, CH_2).
^{13}C NMR (101 MHz, $CDCl_3$) δ 145.9, 128.6, 128.4, 126.1, 30.0, 16.6.
HRMS (Sicrit plasma/LTQ-Orbitrap) m/z: $[M + H]^+$ Calcd for $C_{15}H_{15}^+$ 195.1168; Found 195.1167.
NMR data consistent with reported value.[5]

(2-cyclopropylethyl)benzene (**5v**)

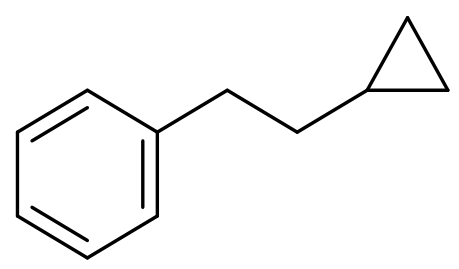

Following the general procedure D using 4-Phenyl-1-butene (661 mg, 5.00 mmol, 1.0 equiv.), yielding (2-cyclopropylethyl)benzene (**5u**) as a clear oil (730 mg, 4.99 mmol, 100%).

R_f (Pentane) = 0.82.

^{1}H NMR (400 MHz, CDCl3) δ 7.30 – 7.24 (m, 2H), 7.22 – 7.14 (m, 3H), 2.77 – 2.66 (m, 2H), 1.55 – 1.48 (m, 3H), 0.77 – 0.64 (m, 1H), 0.45 – 0.38 (m, 2H), 0.08 – 0.01 (m, 2H).
^{13}C NMR (101 MHz, $CDCl_3$) δ 142.8, 128.6, 128.4, 125.7, 36.9, 36.2, 10.9, 4.6.
NMR data consistent with reported value.[21]

S5. Optimization tables for 1,3-aminochlorination[a]

To a 5 mL test tube, NCBSI (68 mg, 0.20 mmol, 2.0 equiv.) was added. The tube was then closed with a rubber septum and sealed off with parafilm. Three cycles of evacuate-refill with nitrogen were performed to remove O_2. DCM (2 mL, 0.05M) was added and the solution was stirred under irradiation of Kessil lamps (440nm, 2x21W) and after few seconds the cyclopropylbenzene **5a** (13 µL, 0.10 mmol,1.0 equiv.). It is important that the addition of starting material happens a few seconds after the irradiation started to ensure reproducibility. The reaction was stirred under irradiation for 8-12 h, then the solvent was removed under reduced pressure.

NCBSI loading

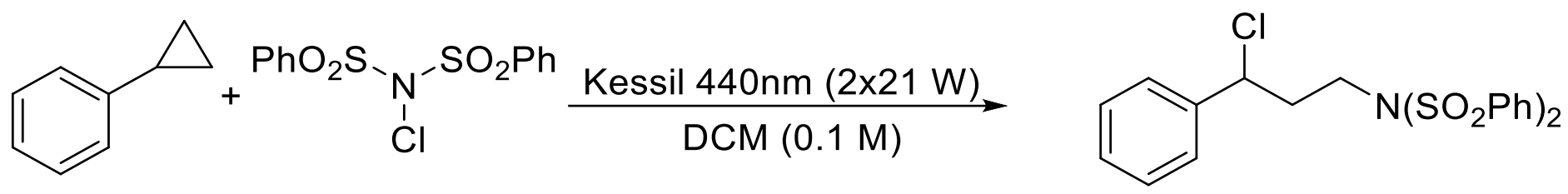

5a ***NCBSI*** (**X** equiv.) **6a**

Entry	**NCBSI (X equiv.)**	NMR conversion (%)[a]	NMR Yield (%)[a]
1	1.0	76	76
2	1.5	87	85
**3**	**2.0**	**90**	**89**

Solvent amount

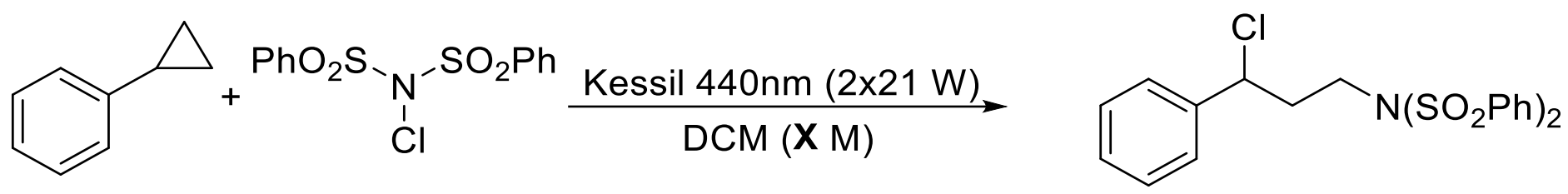

5a ***NCBSI*** (2.0 equiv.) **6a**

Entry	**Conc. (X M)**	NMR conversion (%)[a]	NMR Yield (%)[a]
1	0.1	90	89
**2**	**0.05**	**100**	**quant.**

Light intensity

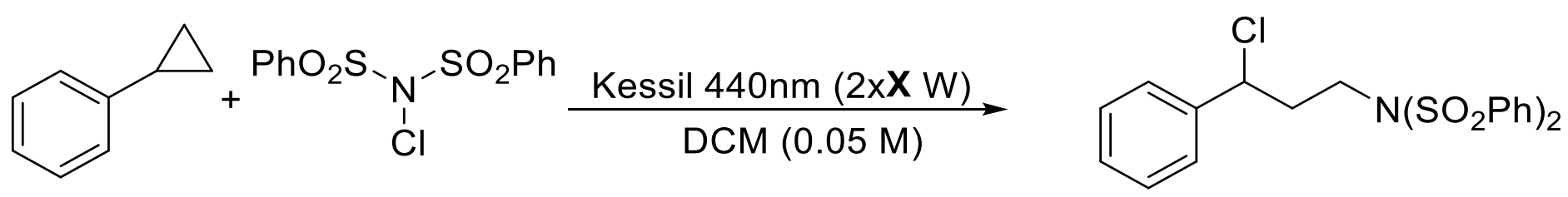

5a ***NCBSI*** (2.0 equiv.) **6a**

Entry	**Light intensity (X W)**	NMR conversion (%)[a]	NMR Yield (%)[a]
1	10.5	92	89
**2**	**21**	**100**	**quant.**
3	31.5	100	quant.

Light wavelength

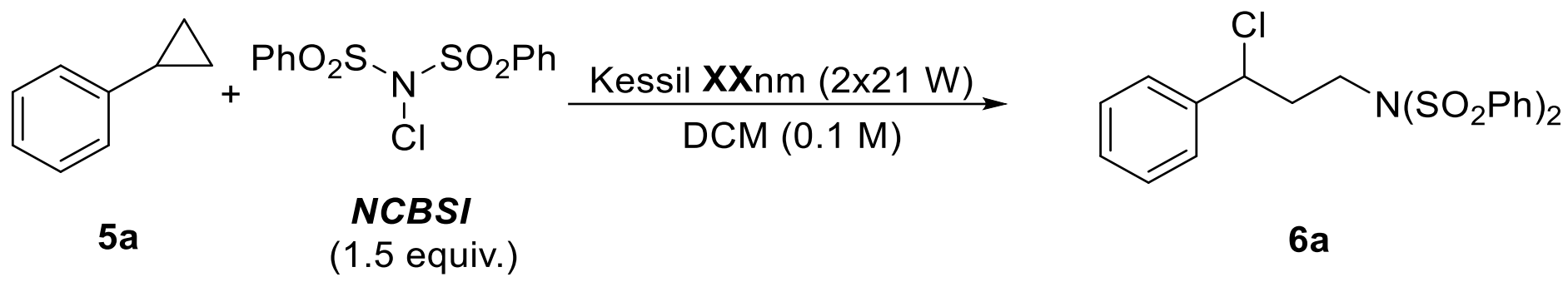

Entry	Light wavelength	NMR conversion (%)[a]	NMR Yield (%)[a]
1	390 nm	86	40
2	427 nm	100	quant.
**3**	**440 nm**	**100**	**quant.**

[a]NMR conversion and yield were determined with CH_2Br_2 as internal standard.

S6. Optimization tables and control experiments for the 1,3-aminoarylation[a]

To an oven dried 5 mL test tube, NFSI (48 mg, 0.15 mmol, 1.5 equiv.), the aryl boronic acid (0.2 mmol, 2.0 equiv.), Cs_2CO_3 (67 mg, 0.20 mmol, 2.0 equiv.), $Cu(MeCN)_4PF_6$ (3.7 mg, 0.010 mmol, 10 mol%), and 1,10-phenanthroline (0.5 mg, 0.005 mmol, 5 mol%) were added. The tube was then closed with a rubber septum and sealed off with parafilm. Three cycles of evacuate-refill with nitrogen were performed to remove O_2. Then cyclopropylbenzene **5a** (13 µL, 0.10 mmol, 1.0 equiv.) was added together with DCE (1 mL, 0.1 M) and the mixture was stirred at 70 °C for 5-10 minutes. The consumption of the oxidant is indicated by a change of color from light blue to brown. The solvents were removed under reduced pressure.

Copper source

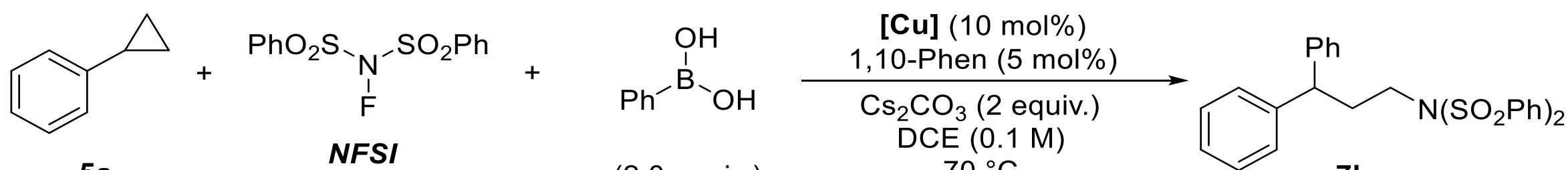

Entry	**[Cu]**	NMR conversion (%)[a]	NMR Yield (%)[a]
1	CuCN	10	-
2	CuBr	17	-
3	CuCl	16	-
4	$CuBr_2$	20	-
5	Cu(OAc)	28	-
6	CuTc	66	28
7	$Cu(OTf)_2$	77	40
8	$Cu(OTf)_2$ Toluene	46	19
9	CuI	72	41
10	$Cu(MeCN)_4OTf$	77	38
11	$Cu(MeCN)_4BF_4$	54	26
12	$Cu(MeCN)_4SbF_6$	78	45
**13**	**$Cu(MeCN)_4PF_6$**	**90**	**57**

Catalyst loading

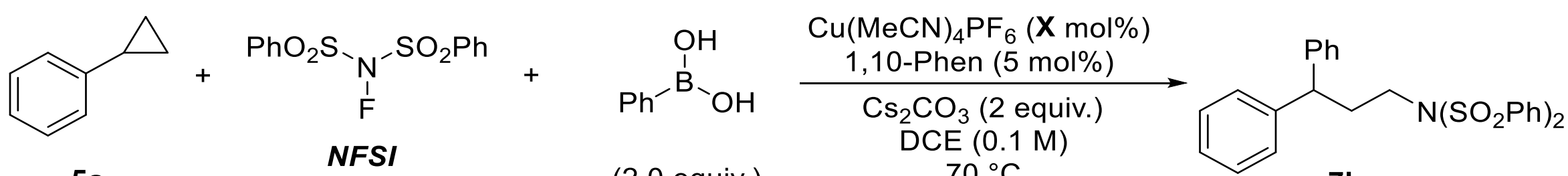

Entry	**Copper X**	NMR conversion (%)[a]	NMR Yield (%)[a]
1	100	100	20
2	5	86	43
3	3	100	41
4	1	96	34
**5**	**10**	**90**	**57**

Ligands

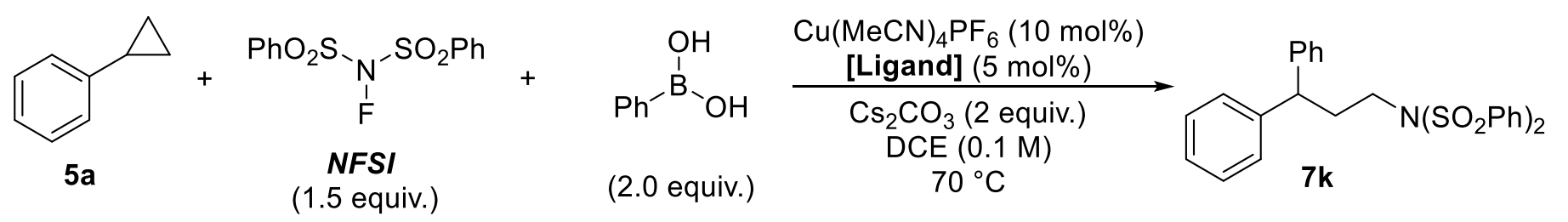

5a + *NFSI* (1.5 equiv.) + (2.0 equiv.) → 7k

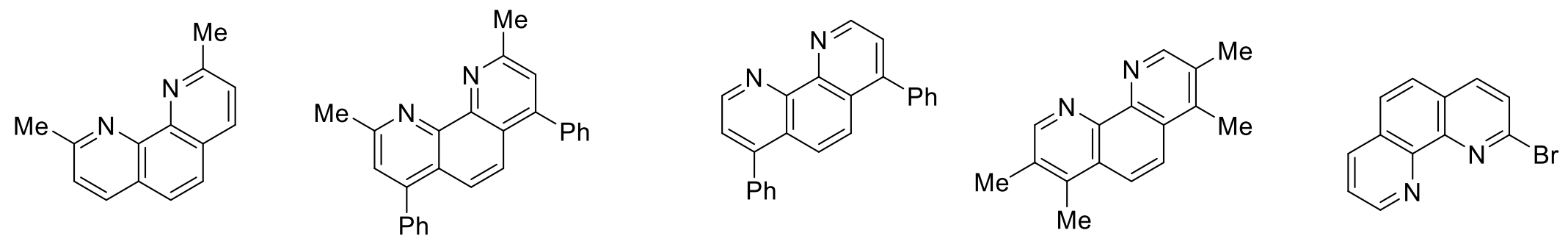

NMR Conversion: 52% NMR Yield: -

NMR Conversion: 44% NMR Yield: 20%

NMR Conversion: 75% NMR Yield: 23%

NMR Conversion: 54% NMR Yield: 35%

NMR Conversion: 77% NMR Yield: 30%

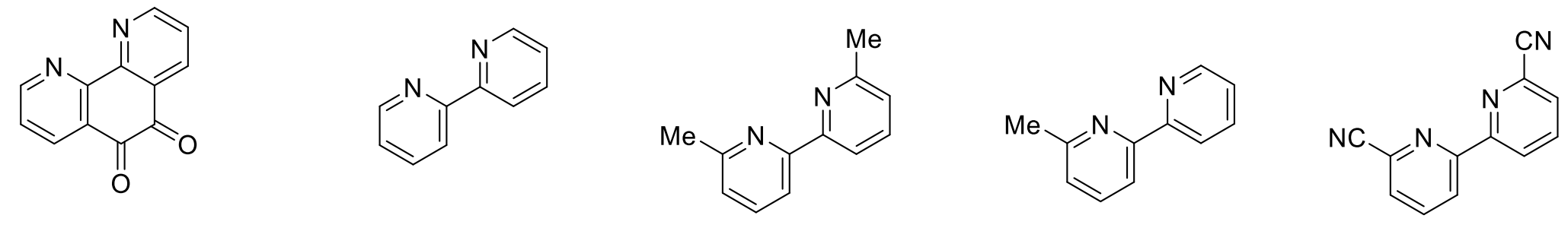

NMR Conversion: 80% NMR Yield: 31%

NMR Conversion: 57% NMR Yield: 27%

NMR Conversion: 62% NMR Yield: -%

NMR Conversion: 72% NMR Yield: 33%

NMR Conversion: -% NMR Yield: -%

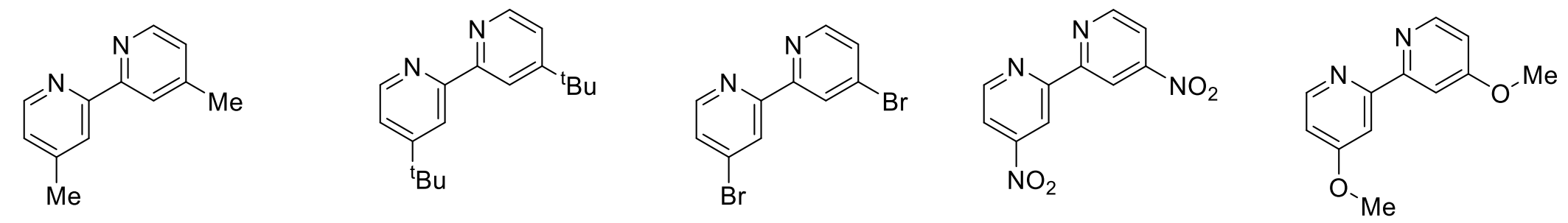

NMR Conversion: 83% NMR Yield: 33%

NMR Conversion: 78% NMR Yield: 29%

NMR Conversion: 78% NMR Yield: 34%

NMR Conversion: 61% NMR Yield: 30%

NMR Conversion: 78% NMR Yield: 42%

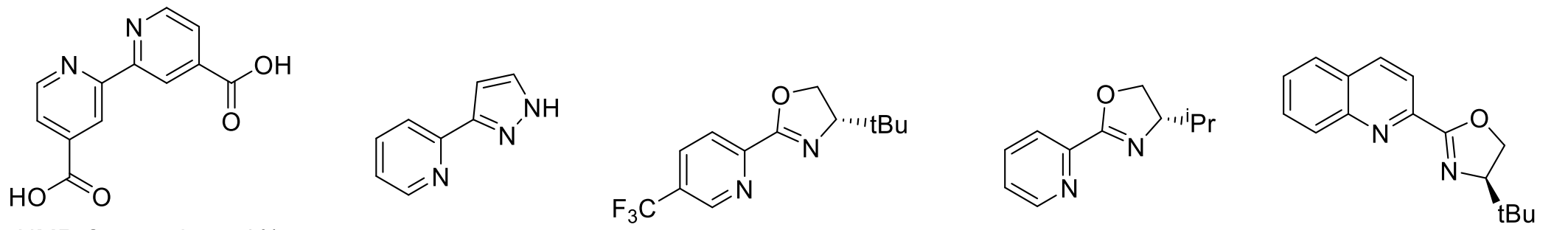

NMR Conversion: 59% NMR Yield: -%

NMR Conversion: 68% NMR Yield: 23%

NMR Conversion: 78% NMR Yield: 19%

NMR Conversion: 53% NMR Yield: 22%

NMR Conversion: 33% NMR Yield: -%

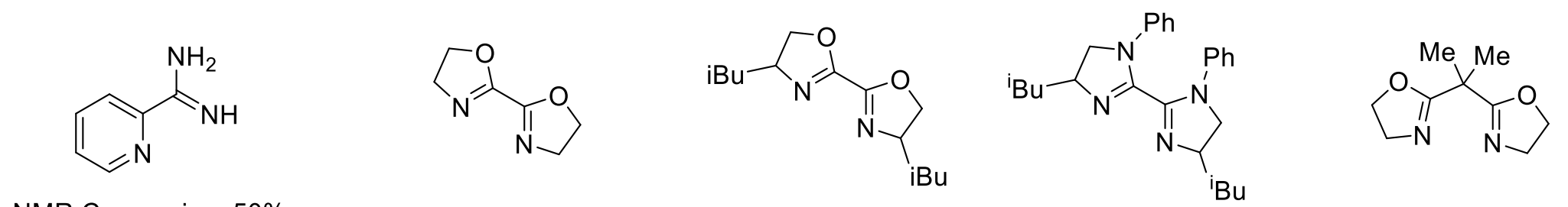

NMR Conversion: 50% NMR Yield: 21%

NMR Conversion: 68% NMR Yield: 26%

NMR Conversion: 69% NMR Yield: 18%

NMR Conversion: 29% NMR Yield: -%

NMR Conversion: 75% NMR Yield: 19%

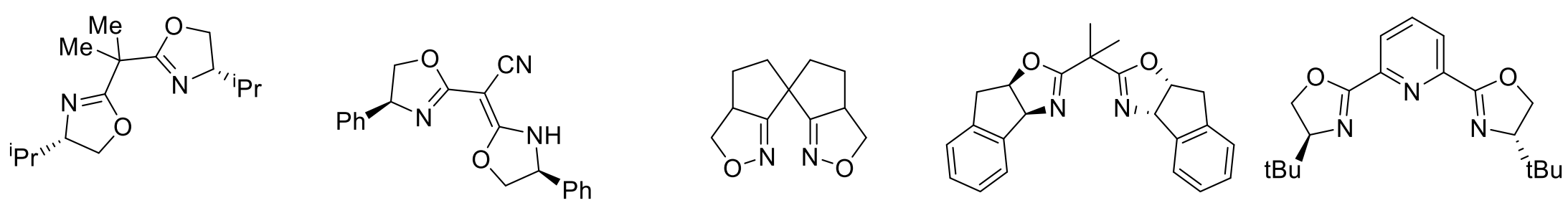

NMR Conversion: 68% NMR Yield: 12%

NMR Conversion: 35% NMR Yield: -%

NMR Conversion: 43% NMR Yield: -%

NMR Conversion: 12% NMR Yield: <5%

NMR Conversion: 42% NMR Yield: 20%

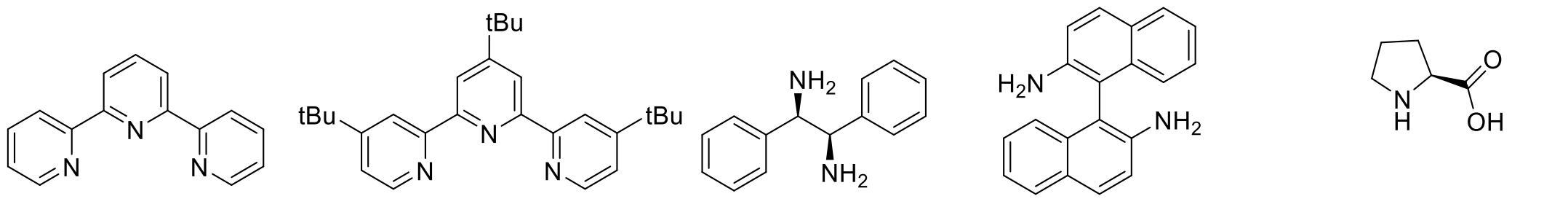

NMR Conversion: 22% NMR Yield: -%

NMR Conversion: 64% NMR Yield: <5%

NMR Conversion: 69% NMR Yield: 9%

NMR Conversion: 34% NMR Yield: -%

NMR Conversion: 25% NMR Yield: 10%

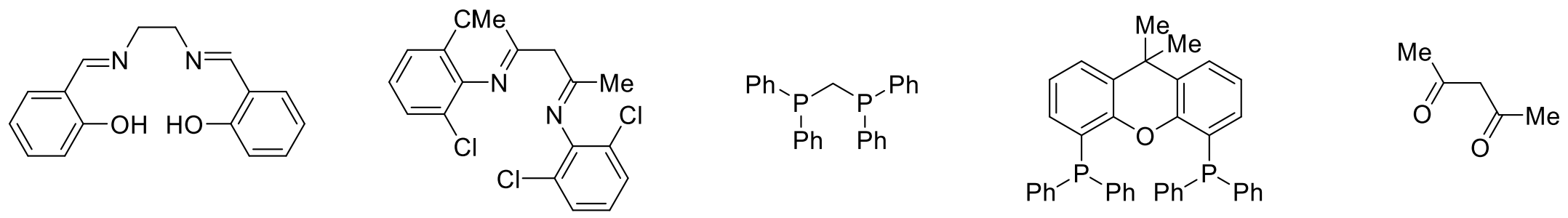

NMR Conversion: 17% NMR Yield: -%

NMR Conversion: 8% NMR Yield: -%

NMR Conversion: 11% NMR Yield: -%

NMR Conversion: 54% NMR Yield: 14%

NMR Conversion: 62% NMR Yield: -%

Base

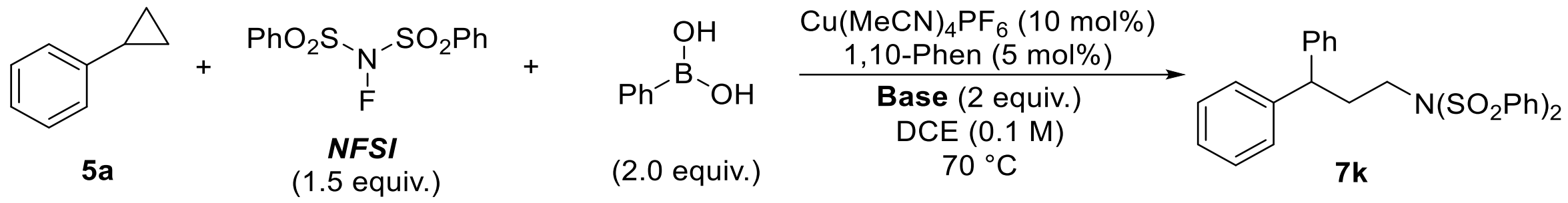

Entry	**Base**	NMR conversion (%)[a]	NMR Yield (%)[a]
1	Triethylamine	70	40
2	DIPEA	45	2
3	2,6-Lutidine	80	-
4	DMAP	-	-
5	Pyridine	88	20
6	CsF	77	30
7	LiOAc	9	-
8	NaOAc	17	-
9	KOAc	19	-
10	CsOAc	17	-
11	K_3PO_4	65	37
12	DBU	<5	-
13	DABCO	<5	-
14	tBuOLi	64	9
15	Li_2CO_3	44	-
16	Na_2CO_3	39	-
17	K_2CO_3	55	32
**18**	**Cs_2CO_3**	**90**	**57**

Solvent

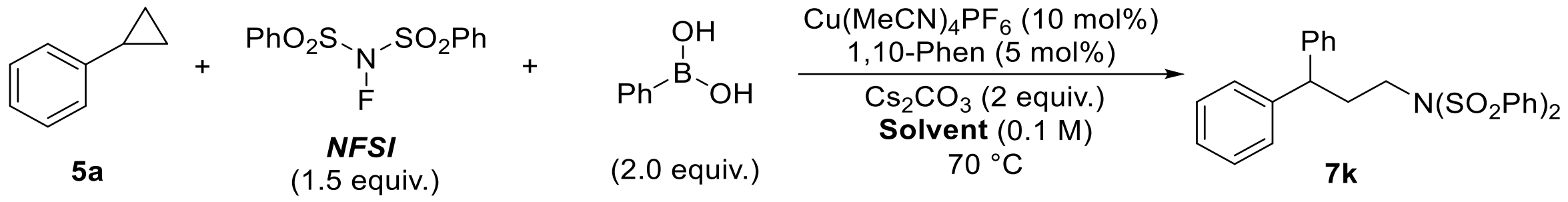

Entry	**Solvent**	NMR conversion (%)[a]	NMR Yield (%)[a]
1	Acetonitrile	38%	16
2	MeOH	15%	-
3	DCE:DMA	10%	-
4	$CHCl_3$	48%	25
5	PhCl	61%	30
6	Acetone	60%	36
7	Toluene	13%	-
8	$PhCF_3$	23%	9
9	THF	9%	-
10	Et_2O	<5%	-
**12**	**DCE**	**90**	**57**

Ligand loading

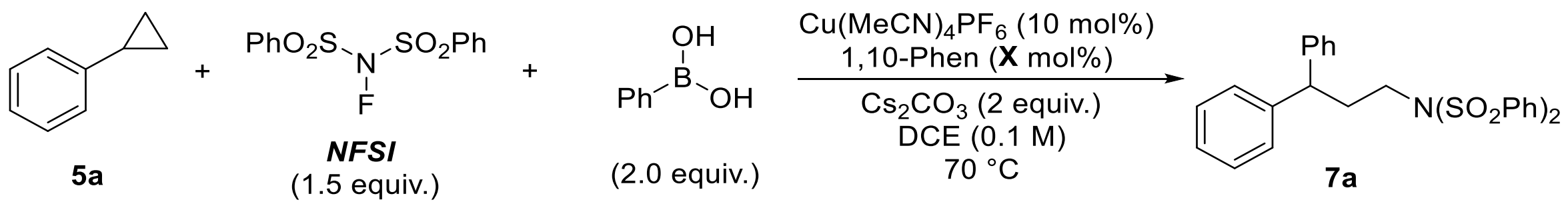

Entry	**Ligand X**	NMR conversion (%)[a]	NMR Yield (%)[a]
1	2	72	57
2	3	70	34
3	4	71	10
**4**	**5**	**90**	**57**
5	15	30	34
6	20	12	10
7	25	9	0

Nucleophiles

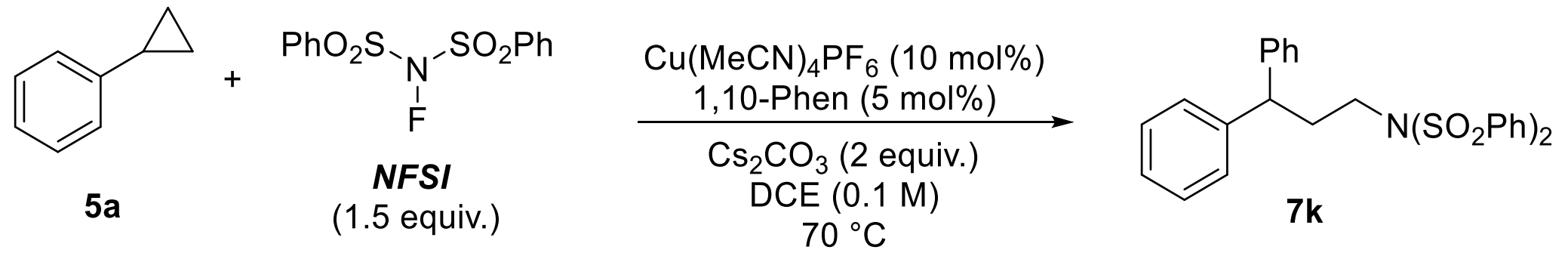

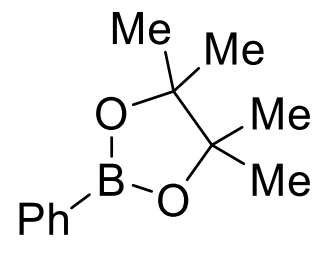

NMR Conversion: 50%
NMR Yield: -

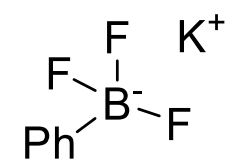

NMR Conversion: 67%
NMR Yield: -

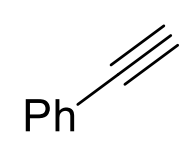

NMR Conversion: 57%
NMR Yield: -

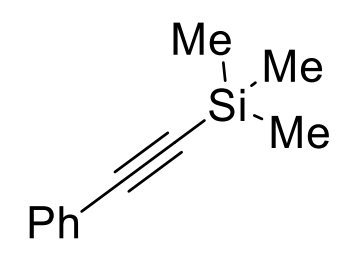

NMR Conversion: 23%
NMR Yield: -

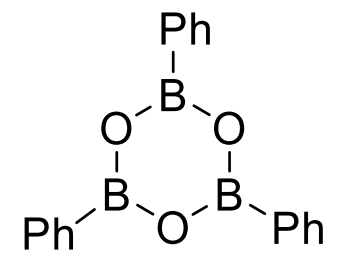

NMR Conversion: 85%
NMR Yield: 44%

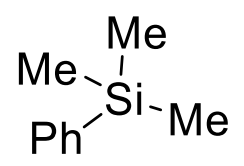

NMR Conversion: 7%
NMR Yield: -

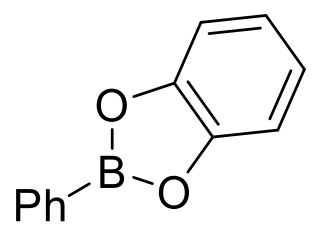

NMR Conversion: 27%
NMR Yield: 4%

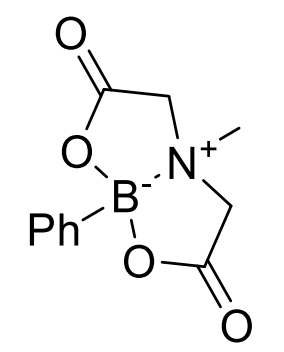

NMR Conversion: 37%
NMR Yield: -%

Stoichiometry

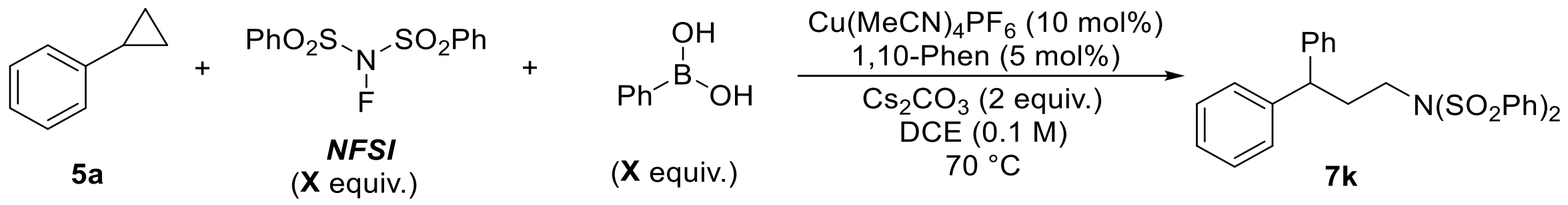

Entry	**NFSI equiv.**	**Boronic acid/Base equiv.**	NMR conversion (%)[a]	NMR Yield (%)[a]
1	0.75	0.55	61	39
2	2.0	2.0	73	20
3	5.0	2.0	85	27
4	1.5	5.0	100	27
5	5.0	5.0	85	41
6	2.0	1.5	82	44
**7**	**1.5**	**2.0**	**90**	**57**

Temperature

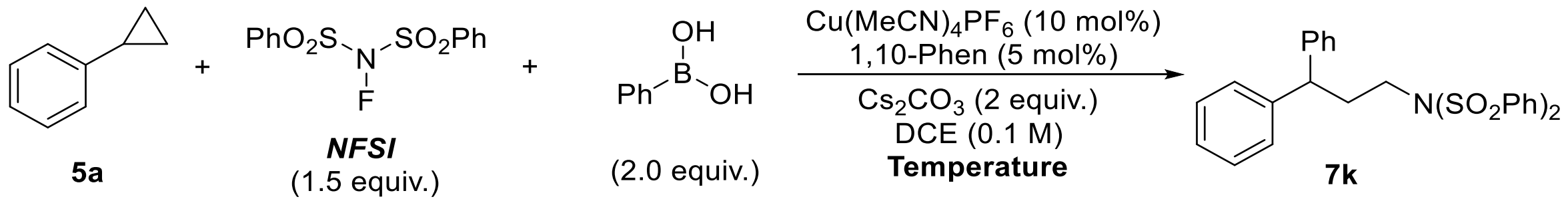

Entry	**Temperature (°C)**	NMR conversion (%)[a]	NMR Yield (%)[a]
1	-40	-	-
2	-20	20	17
3	0	50	40
4	Room temperature	85	27
5	30	64	41
**6**	**70**	**90**	**57**

Water effect

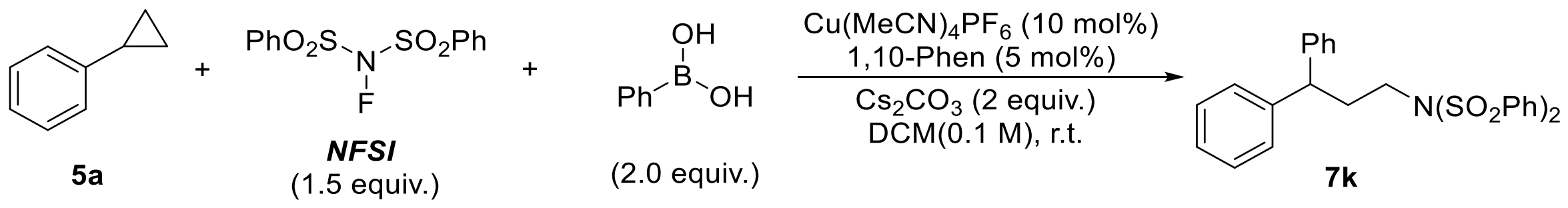

Entry	**Variation**	NMR conversion (%)[a]	NMR Yield (%)[a]
1	Glovebox+4AMS	50	31
2	10 µL H_2O	51	31
3	200 µL H_2O	52	18

Others

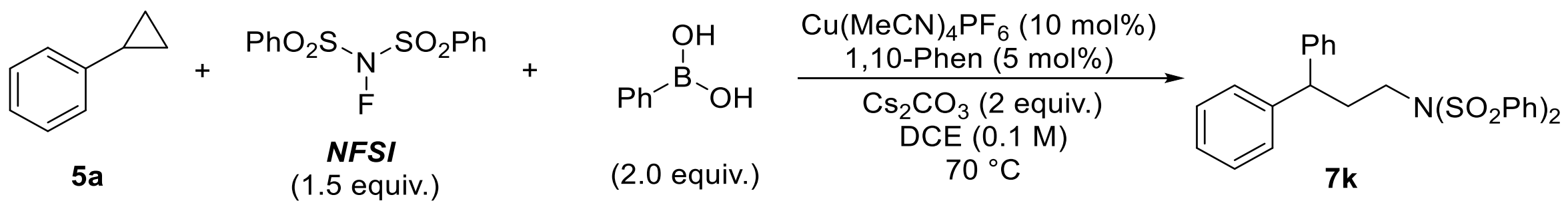

Entry	**Variation**	NMR conversion (%)[a]	NMR Yield (%)[a]
1	Neat	36	10
2	Boronic acid dropwise	14	-
3	NFSI and boronic acid dropwise	-	-
4	5.0 equiv. of NFSI, Boronic acid and base in 0.02M solution	6	41
5	3.0 equiv. Benzensulfonimide in 0.02M	95	28
6	1.5 equiv. of TMSBr	83	31
7	1.5 equiv. of $(TMS)_2O$	87	38
8	1.0 equiv. $CaNO_3$	78	46
9	4.0 equiv.18-C-6	-	-
10	In plastic vessel	70	40
11	MW at 100°C for 5 min	65	30
12	MW at 70°C for 10 min	51	32
13	At r.t. under Kessil 440nm (2x21W)	75	51
14	under Kessil 440nm (2x21W)	71	34

Control experiments

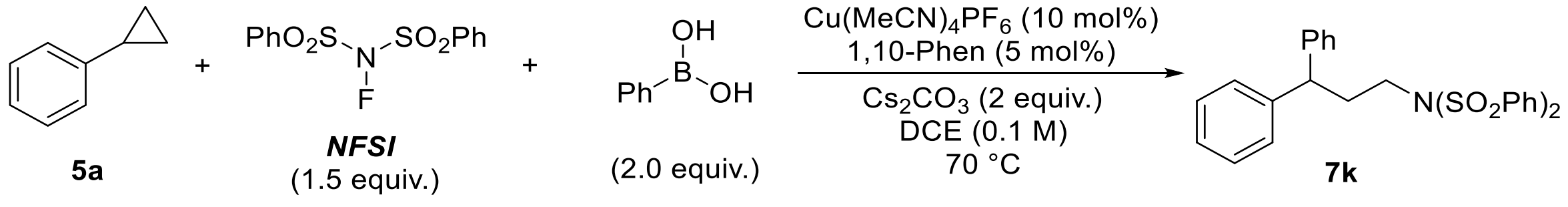

Entry	**Variation**	NMR conversion (%)[a]	NMR Yield (%)[a]
1	No Base	-	-
2	No NFSI	-	-
3	No Boronic acid	-	-
4	No Copper	-	-
5	No ligand	-	-

[a]NMR conversion and yield were determined with CH_2Br_2 as internal standard.

S7. Radical trapping experiments

1,3-Aminochlorination photolysis

To gain insight into the reactivity of NCBSI under the photochemical reaction conditions, control and mechanistic experiments were performed.

Degradation of NCBSI upon irradiation

To a 5 mL test tube, NCBSI (68 mg, 0.20 mmol, 2.0 equiv.) was added. The tube was then closed with a rubber septum and sealed off with parafilm. Three cycles of evacuate-refill with nitrogen were performed to remove O_2. DCM (2 mL, 0.05M) was added and the solution was stirred under irradiation of Kessil lamps (440nm, 2x21W) and after 10 minutes the cyclopropylbenzene **5a** (13 µL, 0.10 mmol,1.0 equiv.). The reaction was stirred under irradiation for 8-12 h, then the solvent was removed under reduced pressure. No conversion and full recovery of the starting material **5a** was observed by NMR analysis.

Generation of chlorine radical upon irradiation

To further probe the involvement of chlorine radicals under the reaction conditions, trapping experiments were conducted using toluene as a chlorine radical scavenger.[22]

A GC-FID calibration line was made using Dodecane (0.1 M) as internal standard, and Benzyl chloride (0.02-0.10 M).

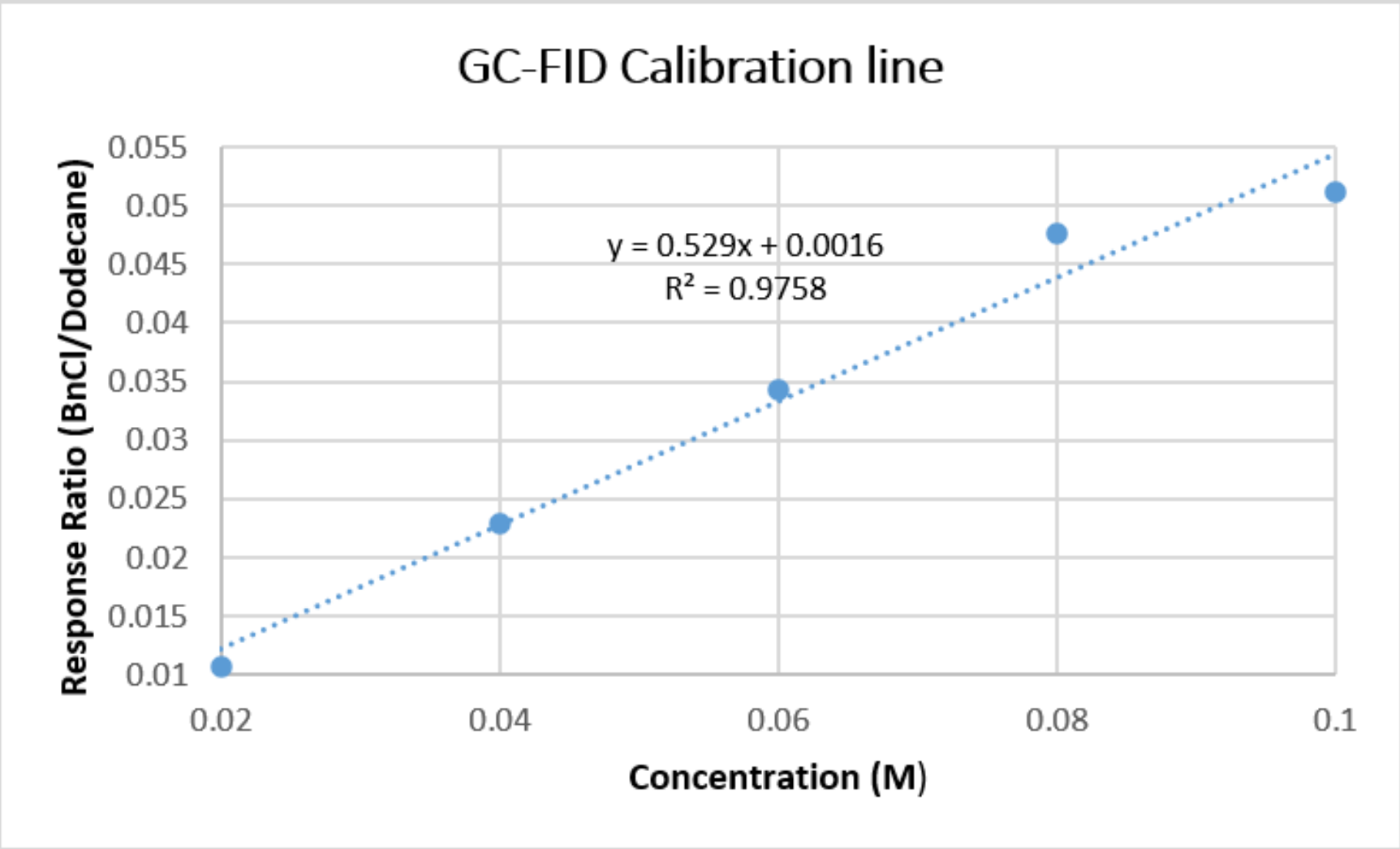

In a 15*100 mm Borosilicate glass tube, NCBSI (101 mg, 0.300 mmol) was added. The tube was then closed with a rubber septum and sealed off with parafilm. Three cycles of evacuate-refill with nitrogen were performed to remove O_2. DCM (3 mL, 0.1 M), Dodecane (68 µL, 0.30 mmol, 1.0 equiv.) and Toluene (318 µL, 0.300 mmol, 1.0 equiv.) were added, then the solution was stirred under irradiation of Kessil lamps (440nm, 2x21W) and aliquots of 0.3 mL were taken every 30 seconds for 2.5 minutes. The samples were analyzed by GC-FID and by interpolation with the calibration line, the quantity of Benzyl chloride formed was obtained.

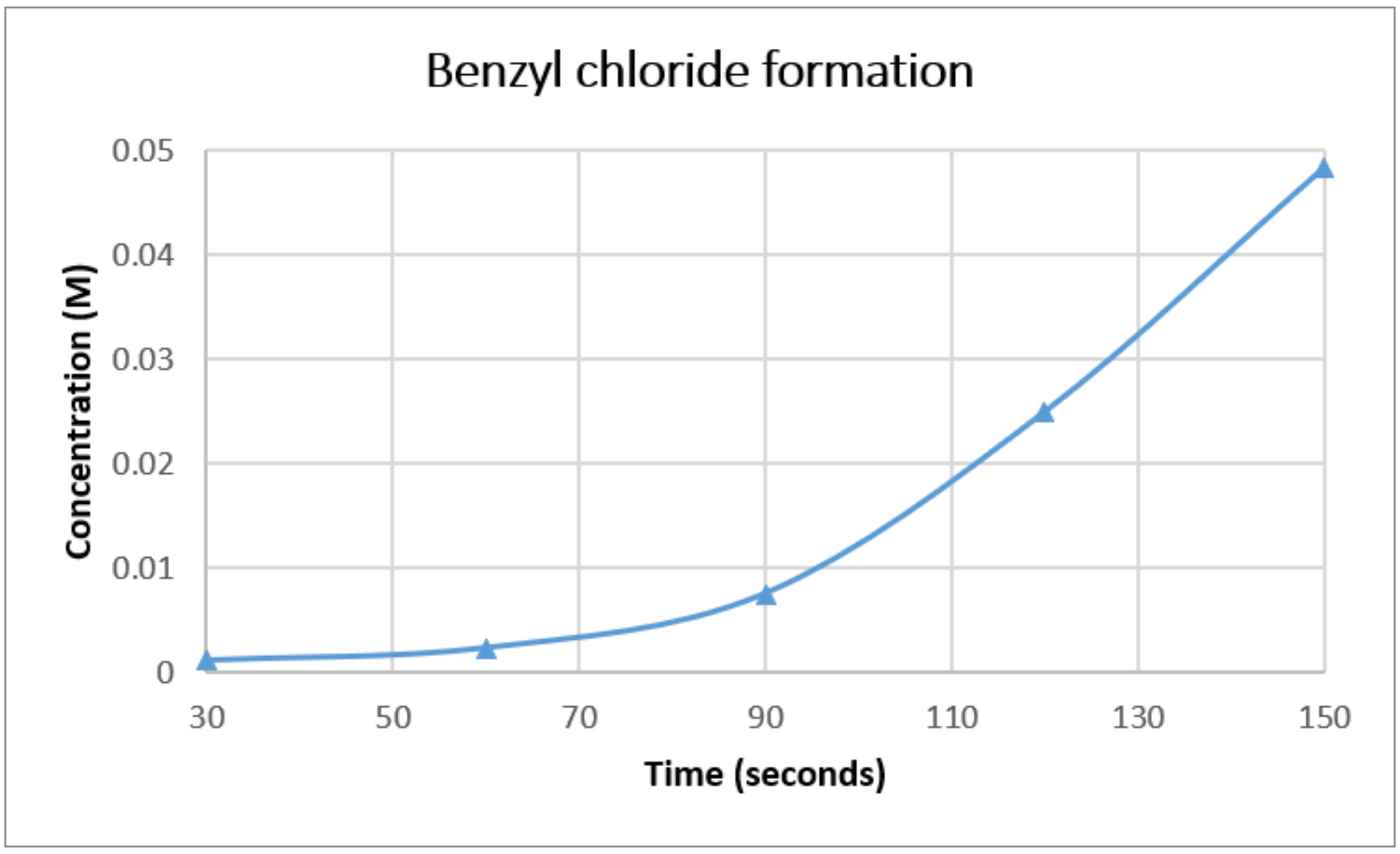

Formation of benzyl chloride was observed upon irradiation, as confirmed by GC-FID analysis, supporting the generation of chlorine radical species by photolysis.

1,3-Aminochlorination method

In a 15*100 mm Borosilicate glass tube, NCBSI (203 mg, 0.600 mmol, 2.0 equiv.) and solid trapping agent (5.0 equiv.) were added. The tube was then closed with a rubber septum and sealed off with parafilm. Three cycle of evacuate-refill with nitrogen were performed to remove O_2. DCM (6 mL, 0.05M) was added and the solution was stirred under irradiation of Kessil lamps (440nm, 2x21W) and after few seconds the cyclopropylbenzene **5a** (39 µL, 0.30 mmol,1.0 equiv.) and liquid trapping agent (5.0 equiv.) were added. It is important that the addition of starting material happens a few seconds after the irradiation started to ensure reproducibility. The reaction was stirred under irradiation for 8-12 h, then the solvent was removed under reduced pressure and the crude was purified by chromatography on Biotage (Büchi flashpure cartridge 25 g, Hexane:AcOEt from 10 to 40%) and by reverse phase chromatography on Büchi (BGB Aquarius C18AQ 40g, 100Ǻ, 15 µm, H_2O (0.1% TFA):MeCN(0.1% TFA) from 0 to 100%) to obtain the products.

1,3-Aminoarylation method

To an oven dried 5 mL microwave vial, NFSI (145 mg, 0.450 mmol, 1.5 equiv.), the phenyl boronic acid (72 mg, 0.60 mmol, 2.0 equiv.), Cs_2CO_3 (198 mg, 0.600 mmol, 2.0 equiv.), $Cu(MeCN)_4PF_6$ (11 mg, 0.030 mmol, 10 mol%), and 1,10-phenanthroline (45.6 mg, 0.250 mmol, 5 mol%) and solid trapping agent (5.0 equiv.) were added. The tube was then closed with a rubber septum and sealed off with parafilm. Three cycles of evacuate-refill with nitrogen were performed to remove O_2. Then cyclopropylbenzene **5a** (39 µL, 0.30 mmol, 1.0 equiv.) and liquid trapping agent (5.0 equiv.) were added together with DCE (3 mL, 0.1 M) and the mixture was stirred at 70 °C for 5-10 minutes. The consumption of the oxidant is indicated by a change of color from light blue to brown. The solvents were removed under reduced pressure and the crude was

purified by chromatography on Biotage (Büchi flashpure cartridge 25 g, Hexane:AcOEt from 10 to 40%) and by reverse phase chromatography on Büchi (BGB Aquarius C18AQ 40g, 100Å, 15 µm, H_2O (0.1% TFA):MeCN(0.1% TFA) from 0 to 100%) to obtain the products.

2,2,6,6-Tetramethylpiperidin-1-yl hypochlorite (**16**)

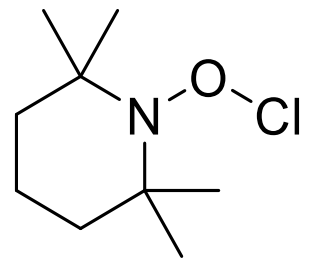

HRMS (ESI/QTOF) m/z: $[M + H]^+$ Calcd for $C_9H_{19}ClNO^+$ 192.1150; Found 192.1142.

N-(3-Phenyl-3-((2,2,6,6-tetramethylpiperidin-1-yl)oxy)propyl)-N-(phenylsulfonyl)benzenesulfonamide (**17**)

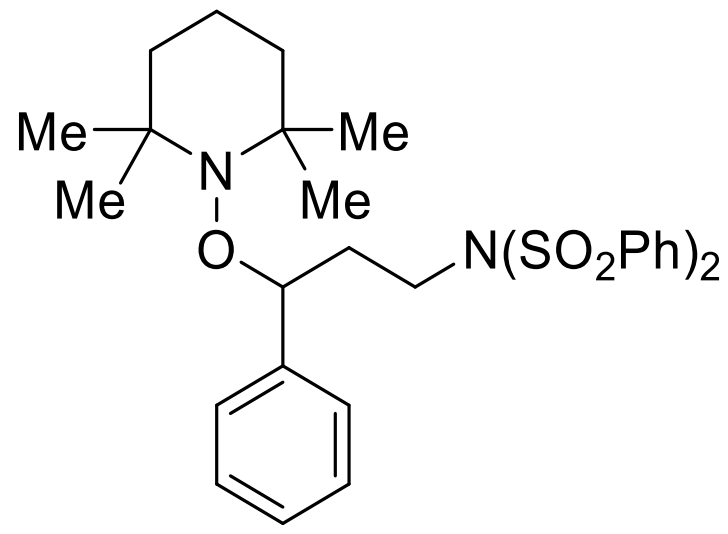

HRMS (ESI/QTOF) m/z: $[M + H]^+$ Calcd for $C_{30}H_{39}N_2O_5S_2^+$ 571.2295; Found 571.2298.

N-(2,2-Diphenylvinyl)-N-(phenylsulfonyl)benzenesulfonamide (**18**), 14%

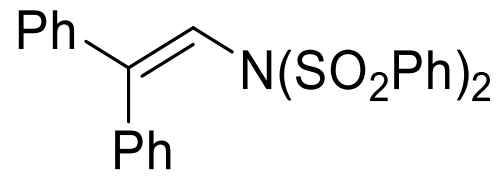

R_f (Hex:AcOEt = 9:1) = 0.28.
^{1}H NMR (400 MHz, $CDCl_3$) δ 7.76 – 7.70 (m, 4H, Ar*H*), 7.63 – 7.56 (m, 2H, Ar*H*), 7.47 – 7.40 (m, 4H, Ar*H*), 7.40 – 7.20 (m, 10H, Ar*H*), 6.15 (s, 1H, C*H*N).
^{13}C NMR (101 MHz, $CDCl_3$) δ 152.4, 140.0, 138.8, 136.9, 134.0, 130.1, 129.2, 128.9, 128.9, 128.8, 128.8, 128.5, 128.4, 128.3, 116.4.
HRMS (nanochip-ESI/LTQ-Orbitrap) m/z: [M + H]+ Calcd for C26H22NO4S2+ 476.0985; Found 476.1028.
NMR data consistent with reported value.[23]

(2-Chloroethene-1,1-diyl)dibenzene (**19**), 36%

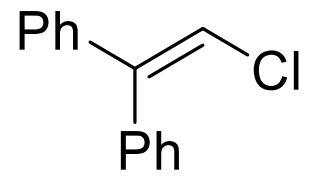

R_f (Hex:AcOEt = 9:1) = 0.68.
^{1}H NMR (400 MHz, $CDCl_3$) δ 7.35 – 7.17 (m, 8H, Ar*H*), 7.17 – 7.08 (m, 2H, Ar*H*), 6.51 (s, 1H, C*H*Cl).

^{13}C NMR (101 MHz, $CDCl_3$) δ 144.0, 140.3, 137.7, 130.0, 128.6, 128.3, 128.2, 128.1, 127.9, 116.0.
HRMS (Sicrit plasma/LTQ-Orbitrap) m/z: $[M]^+$ Calcd for $C_{14}H_{11}Cl^+$ 214.0544; Found 214.0544.
NMR data consistent with reported value.[24]

(2,2-Dichloroethene-1,1-diyl)dibenzene (**20**), 45%

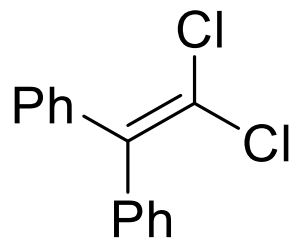

R$_f$ (Hex:AcOEt = 9:1) = 0.68.
^{1}H NMR (400 MHz, $CDCl_3$) δ 7.30 – 7.18 (m, 10H, Ar*H*).
^{13}C NMR (101 MHz, $CDCl_3$) δ 140.6, 139.6, 129.4, 128.4, 128.1.
One carbon not resolved.
NMR data consistent with reported value.[25]
HRMS (Sicrit plasma/LTQ-Orbitrap) m/z: $[M]^+$ Calcd for $C_{14}H_{10}Cl_2^+$ 248.0154; Found 248.0154.

N-(3,5-Di-tert-butyl-4-hydroxybenzyl)-N-(phenylsulfonyl)benzenesulfonamide (**21**), 23%

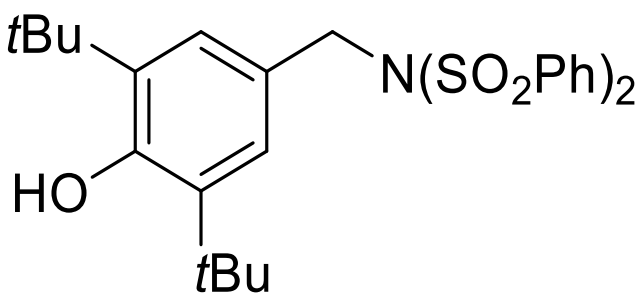

R$_f$ (Hex:AcOEt = 9:1) = 0.32.
^{1}H NMR (400 MHz, $CDCl_3$) δ 7.79 (dt, *J* = 7.3, 1.3 Hz, 4H, Ar*H*), 7.58 – 7.50 (m, 2H, Ar*H*), 7.41 (t, *J* = 7.9 Hz, 4H, Ar*H*), 7.19 (s, 2H, Ar*H*), 5.14 (s, 1H, O*H*), 4.90 (s, 2H, ArC*H$_2$*), 1.36 (s, 18H, C*H$_3$*).
^{13}C NMR (101 MHz, $CDCl_3$) δ 153.8, 140.6, 136.0, 133.5, 128.8, 128.1, 126.4, 125.0, 53.3, 34.4, 30.3.
HRMS (ESI/QTOF) m/z: [M + H]+ Calcd for C27H34NO5S2+ 516.1873; Found 516.1869.
NMR data consistent with reported value.[26]

2,6-Di-tert-butyl-4-(chloromethyl)phenol (**22**), 35%

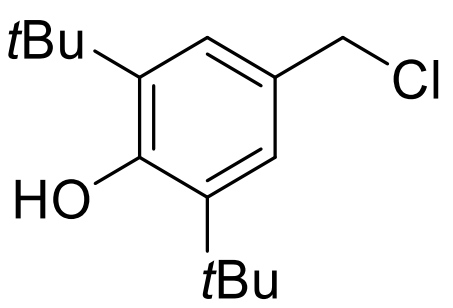

R$_f$ (Hex:AcOEt = 9:1) = 0.54.
^{1}H NMR (400 MHz, $CDCl_3$) δ 7.19 (s, 2H, Ar*H*), 5.22 (s, 1H, O*H*), 4.59 (s, 2H,

ArCH_2), 1.45 (s, 18H, CH_3).
^{13}C NMR (101 MHz, $CDCl_3$) δ 153.7, 136.2, 131.7, 124.6, 66.3, 34.5, 30.4.
HRMS (Sicrit plasma/LTQ-Orbitrap) m/z: $[M + H]^+$ Calcd for C15H24ClO+ 255.1510; Found 255.1511.
NMR data consistent with reported value.[27]

N-(3,3-Diphenylallyl)-N-(phenylsulfonyl)benzenesulfonamide (**23**)

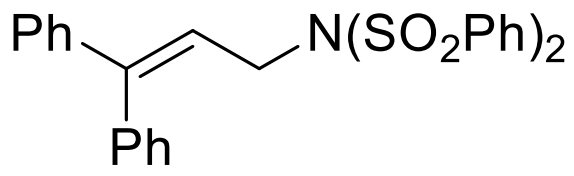

Following procedure E using cyclopropane-1,1-diyldibenzene (**5u**) (60 mg, 0.30 mmol, 1.0 equiv.), yielding N-(3,3-diphenylallyl)-N-(phenylsulfonyl)benzenesulfonamide (**23**) as a colorless wax (76 mg, 0.16 mmol, 52%).

Following procedure H using cyclopropane-1,1-diyldibenzene (**5u**) (60 mg, 0.30 mmol, 1.0 equiv.), yielding N-(3,3-diphenylallyl)-N-(phenylsulfonyl)benzenesulfonamide (**23**) as a colorless wax (76 mg, 0.16 mmol, 52%).

R_f (Hex:AcOEt = 8:2) = 0.52.
^{1}H NMR (400 MHz, $CDCl_3$) δ 7.97 (dt, *J* = 7.4, 1.3 Hz, 4H, Ar*H*), 7.64 – 7.56 (m, 2H, Ar*H*), 7.49 (t, *J* = 7.8 Hz, 4H, Ar*H*), 7.42 – 7.30 (m, 3H, Ar*H*), 7.24 – 7.10 (m, 5H, Ar*H*), 7.02 – 6.88 (m, 2H, Ar*H*), 5.91 (t, *J* = 6.3 Hz, 1H, C*H*), 4.44 (d, *J* = 6.3 Hz, 2H, CHCH_2).
^{13}C NMR (101 MHz, $CDCl_3$) δ 145.0, 141.0, 140.2, 138.4, 133.9, 129.9, 129.2, 129.2, 128.6, 128.6, 128.3, 128.3, 128.0, 127.9, 127.6, 123.6, 48.7.
IR (vmax, cm-1) 1448 (m), 1375 (s), 1169 (s), 1089 (m), 1049 (m), 992 (m), 851 (m), 809 (m), 754 (s), 742 (s), 721 (m).
HRMS (ESI/QTOF) m/z: $[M + Na]^+$ Calcd for $C_{27}H_{23}NNaO_4S_2^+$ 512.0961; Found 512.0963.

S8. 1,3-Aminoarylation Side Products analysis

To an oven dried 50mL round-bottomed flask, NFSI (2.4 g, 7.5 mmol, 1.5 equiv.), phenyl boronic acid (1.2 g, 10.0 mmol, 2.0 equiv.), Cs_2CO_3 (3.3 g, 10.0 mmol, 2.0 equiv.), $Cu(MeCN)_4PF_6$ (186 mg, 0.5 mmol, 10 mol%), and 1,10-phenanthroline (45.6 mg, 0.25 mmol, 5 mol%) were added. The tube was then closed with a rubber septum and sealed off with parafilm. Three cycles of evacuate-refill with nitrogen were performed to remove O_2. Then cyclopropylbenzene **5a** (650 µL, 5.0 mmol, 1.0 equiv.) was added together with DCE (50 mL, 0.1M) and the mixture was stirred at 70°C for 10-30 minutes. The consumption of the oxidant is indicated by a change of color from light blue to brown. The solvents were removed under reduced pressure and the crude was purified by chromatography on Biotage (Büchi flashpure cartridge 120 g, Hexane:AcOEt from 10 to 40%), by reverse phase chromatography on Büchi (BGB Aquarius C18AQ 40g, 100Å, 15 µm, H_2O (0.1% TFA):MeCN(0.1% TFA) from 0 to

100%) and by Preparatory RP-HPLC (H_2O (0.1% TFA):MeCN(0.1% TFA) from 0 to 100%) to obtain the products.

N-Phenyl-N-(phenylsulfonyl)benzenesulfonamide (**25**)

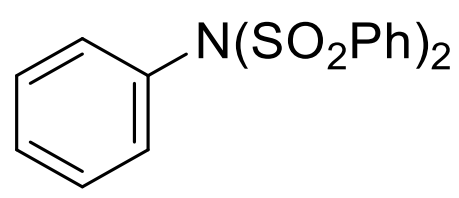

Product **25** obtained as a colorless wax (49.5 mg, 0.125 mmol, 25% yield).

R$_f$ (Hex:AcOEt = 8:2) = 0.48.
^{1}H NMR (400 MHz, $CDCl_3$) δ 7.98 – 7.91 (m, 4H, Ar*H*), 7.71 – 7.63 (m, 2H, Ar*H*), 7.55 (dd, *J* = 8.5, 7.2 Hz, 4H, Ar*H*), 7.48 – 7.42 (m, 1H, Ar*H*), 7.40 – 7.32 (m, 2H, Ar*H*), 7.06 – 6.99 (m, 2H, Ar*H*).
^{13}C NMR (101 MHz, $CDCl_3$) δ 139.6, 134.3, 134.1, 131.7, 130.5, 129.4, 129.1, 128.7.
HRMS (ESI/QTOF) m/z: $[M + Na]^+$ Calcd for $C_{18}H_{15}NNaO_4S_2^+$ 396.0335; Found 396.0319.
NMR data consistent with reported value.[28]

N-(3-Oxo-3-phenylpropyl)-N-(phenylsulfonyl)benzenesulfonamide (**26**)

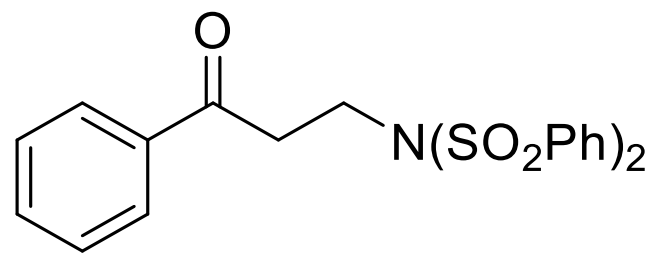

R$_f$ (Hex:AcOEt = 8:2) = 0.32.
^{1}H NMR (400 MHz, $CDCl_3$) δ 8.04 – 7.96 (m, 4H, Ar*H*), 7.90 – 7.85 (m, 2H, Ar*H*), 7.72 – 7.65 (m, 2H, Ar*H*), 7.59 – 7.53 (m, 5H, Ar*H*), 7.50 – 7.42 (m, 2H, Ar*H*), 4.22 – 4.09 (m, 2H, CH_2CH_2), 3.48 – 3.36 (m, 2H, NCH_2).
^{13}C NMR (101 MHz, $CDCl_3$) δ 197.5, 139.6, 136.3, 134.2, 133.7, 129.4, 128.9, 128.4, 128.1, 44.9, 39.2.
HRMS (ESI/QTOF) m/z: $[M + H]^+$ Calcd for $C_{21}H_{20}NO_5S_2^+$ 430.0777; Found 430.0774.
NMR data consistent with reported value.[29]

N,N'-(3,4-Diphenylhexane-1,6-diyl)bis(N-(phenylsulfonyl)benzenesulfonamide) (**27**)

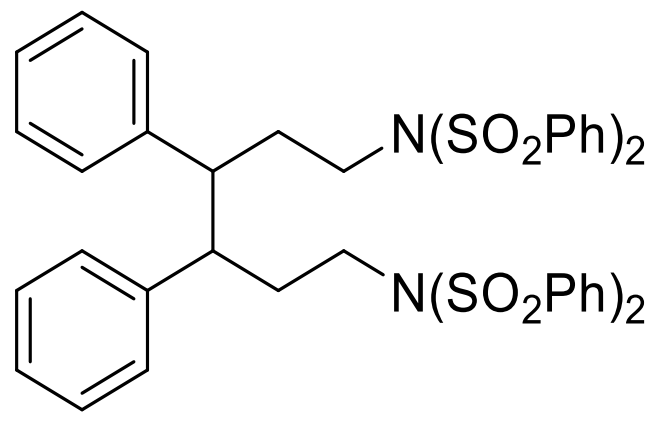

R$_f$ (Hex:AcOEt = 8:2) = 0.26.
^{1}H NMR (400 MHz, $CDCl_3$) δ 7.90 – 7.83 (m, 8H, Ar*H*), 7.62 (t, *J* = 7.6 Hz, 4H, Ar*H*), 7.49 (t, *J* = 7.8 Hz, 8H, Ar*H*), 7.16 (dd, *J* = 5.1, 1.9 Hz, 6H, Ar*H*), 6.78 (dd, *J* = 6.6, 2.9 Hz, 4H, Ar*H*), 3.48 (ddd, *J* = 15.7, 11.5, 4.6 Hz, 2H, NCH_2), 3.23 (ddd, *J* = 15.6,

11.2, 5.1 Hz, 2H, NCH_2), 2.67 (d, *J* = 10.3 Hz, 2H, ArC*H*), 2.15 – 1.91 (m, 4H, CH_2CH_2).
^{13}C NMR (101 MHz, $CDCl_3$) δ 139.9, 134.0, 129.3, 128.9, 128.7, 128.2, 128.2, 126.8, 49.6, 48.4, 33.1.
IR (v_{max}, cm^{-1}) 1489 (m), 1451 (m), 1375 (s), 1169 (s), 1088 (m), 873 (m), 793 (m), 750 (s).
HRMS (ESI/QTOF) m/z: $[M + Na]^+$ Calcd for $C_{42}H_{40}N_2NaO_8S_4^+$ 851.1560; Found 851.1571.

N-(3-Fluoro-3-phenylpropyl)-N-(phenylsulfonyl)benzenesulfonamide (**28**)

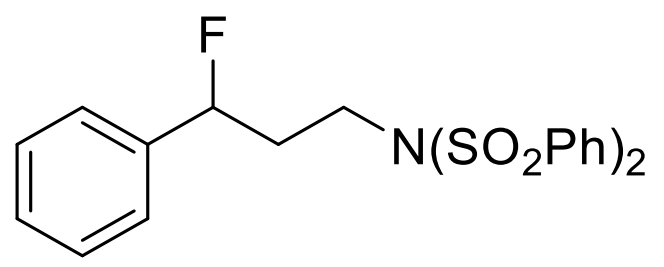

R_f (Hex:AcOEt = 9:1) = 0.44.
^{1}H NMR (400 MHz, $CDCl_3$) δ 8.03 – 7.95 (m, 4H, Ar*H*), 7.73 – 7.62 (m, 2H, Ar*H*), 7.55 (dd, *J* = 8.5, 7.2 Hz, 4H, Ar*H*), 7.42 – 7.31 (m, 3H, Ar*H*), 7.29 – 7.23 (m, 2H, Ar*H*), 5.45 (ddd, *J* = 47.9, 7.9, 4.6 Hz, 1H, ArC*H*), 3.95 – 3.81 (m, 2H, NCH_2), 2.39 – 2.18 (m, 2H, CH_2CH_2).
^{19}F NMR (376 MHz, $CDCl_3$) δ -178.2.
^{13}C NMR (101 MHz, $CDCl_3$) δ 139.7, 139.1, 138.9, 134.1, 129.3, 128.8, 128.4, 125.5 (d, *J* = 6.9 Hz), 91.9 (d, *J* = 171.9 Hz), 45.7 (d, *J* = 4.3 Hz), 37.4 (d, *J* = 23.3 Hz).
HRMS (ESI/QTOF) m/z: [M + Na]+ Calcd for $C_{21}H_{20}FNNaO_4S_2^+$ 456.0710; Found 456.0705.
NMR data consistent with reported value.[3]

N-(3-Hydroxy-3-phenylpropyl)-N-(phenylsulfonyl)benzenesulfonamide (**29**)

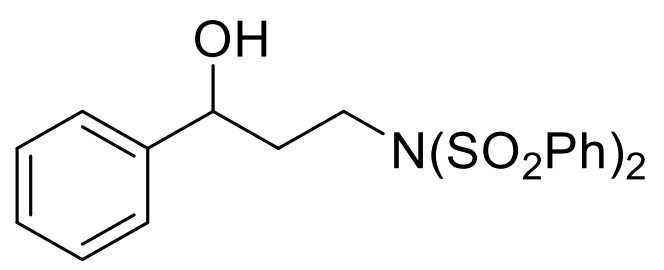

R_f (Hex:AcOEt = 8:2) = 0.26.
^{1}H NMR (400 MHz, $CDCl_3$) δ 8.09 – 7.98 (m, 4H, Ar*H*), 7.70 (t, *J* = 7.4 Hz, 2H, Ar*H*), 7.58 (t, *J* = 7.7 Hz, 4H, Ar*H*), 7.35 (dt, *J* = 15.0, 6.9 Hz, 3H, Ar*H*), 7.27 – 7.17 (m, 2H, Ar*H*), 4.70 (dd, *J* = 9.2, 3.6 Hz, 1H, ArC*H*), 3.92 (q, *J* = 6.7 Hz, 2H , NCH_2), 2.16 (d, *J* = 3.7 Hz, 1H, CH_2CH_2), 2.12 – 1.99 (m, 1H, CH_2CH_2).
^{13}C NMR (101 MHz, $CDCl_3$) δ 143.5, 139.8, 134.1, 129.3, 128.7, 128.4, 127.9, 125.8, 71.1, 46.6, 38.8.
HRMS (ESI/QTOF) m/z: $[M + Na]^+$ Calcd for $C_{21}H_{21}NNaO_5S_2^+$ 454.0753; Found 454.0742.
NMR data consistent with reported value.[30]

N,N'-(1-Phenylpropane-1,3-diyl)bis(N-(phenylsulfonyl)benzenesulfonamide) (**30**)

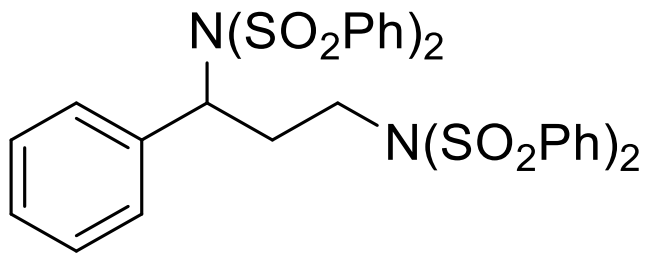

R_f (Hex:AcOEt = 8:2) = 0.24.
^{1}H NMR (400 MHz, $CDCl_3$) δ 7.87 – 7.78 (m, 5H), 7.61 – 7.53 (m, 4H), 7.50 – 7.41 (m, 9H), 7.33 (s, 3H), 7.28 – 7.21 (m, 3H), 7.19 (s, 1H), 5.43 (dd, *J* = 11.2, 4.2 Hz, 1H), 3.44 – 3.30 (m, 2H), 3.23 – 3.05 (m, 1H), 2.29 – 2.13 (m, 1H).m, 2H, NCH_2), 3.23 – 3.05 (m, 1H, CH_2CH_2), 2.29 – 2.13 (m, 1H, CH_2CH_2).
^{13}C NMR (101 MHz, $CDCl_3$) δ 140.5, 139.5, 134.4, 134.1, 133.7, 130.0, 129.3, 129.0, 128.8, 128.7, 128.4, 128.3, 62.1, 46.8, 33.1.
IR (vmax, cm-1) 1451 (w), 1377 (s), 1263 (w), 1171 (s), 1087 (m), 910 (w), 861 (m), 751 (s)
HRMS (ESI/QTOF) m/z: $[M + Na]^+$ Calcd for $C_{33}H_{30}N_2NaO_8S_4^+$ 733.0777; Found 733.0768.

Uncharacterized polymer

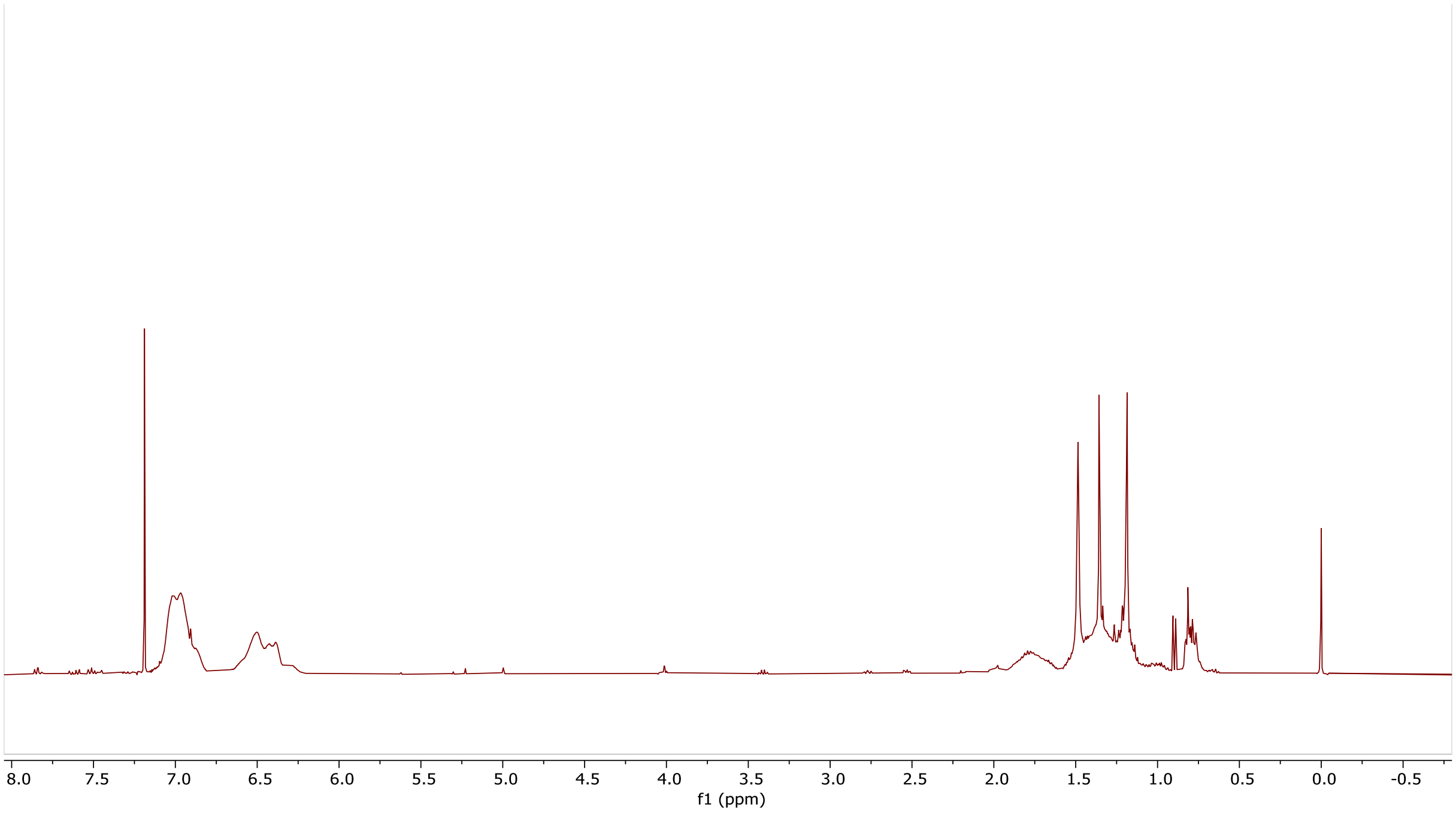

Light On-Off experiment

The experiment was run according to general procedure E using NCBSI (101.0 mg, 0.300 mmol, 2.0 equiv.), cyclopropylbenzene **5a** (20µl, 0.15 mmol, 1.0 equiv.), DCM-d_2 (3 mL) as a solvent and 1,3-dinitrobenzene as internal standard (26 mg, 0.15 mmol, 1.0 equiv.). The samples were taken diluting 0.1 mL of mixture into 0.35 mL of DCM-d_2. The first entry was taken after few seconds the reaction was started. Then serial NMR analysis were performed after the indicated time.

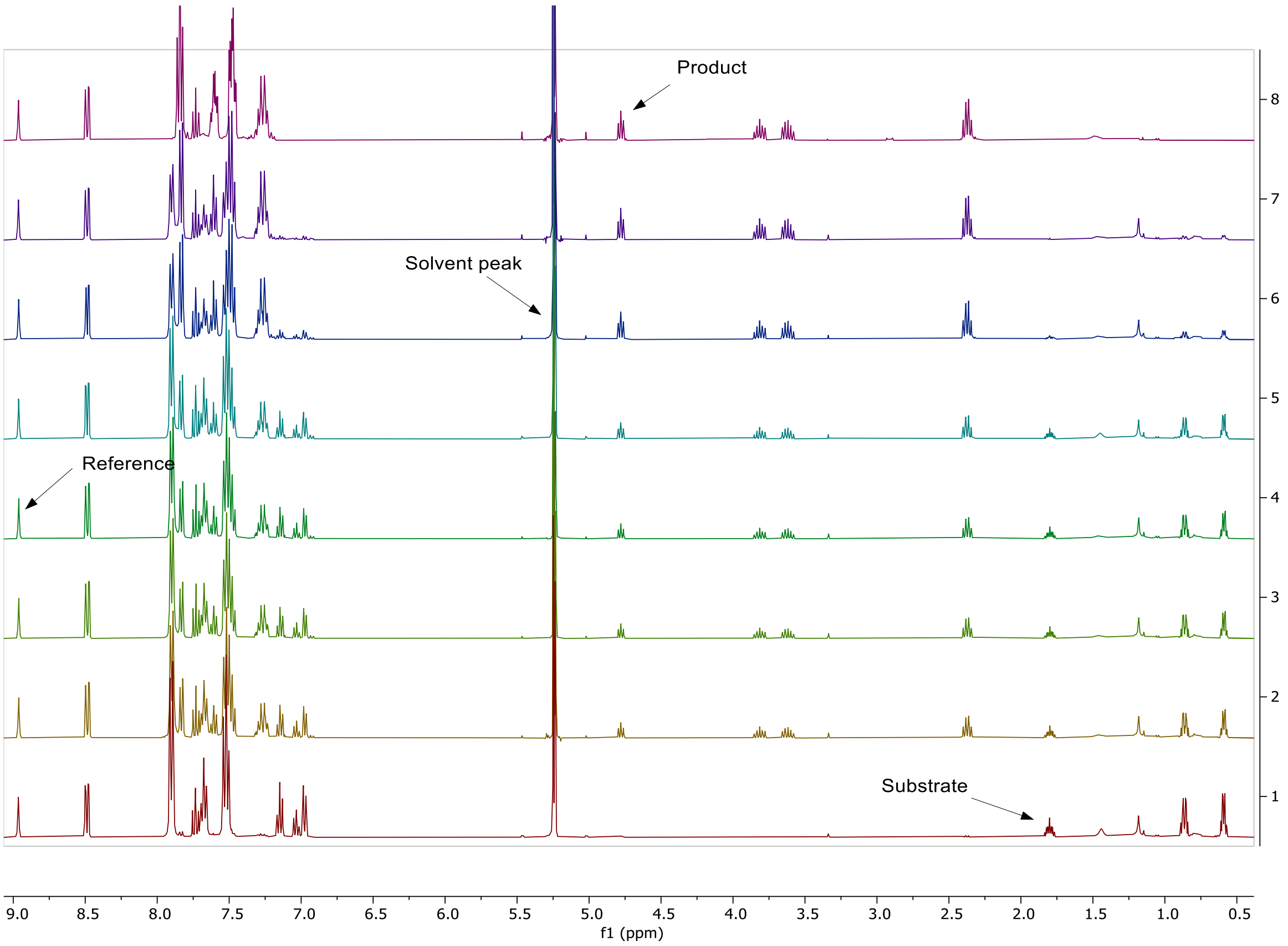

Entry	1	2	3	4	5	6	7	8
Light status	0	On	Off	Off	Off	On	Off	On
Time (h)	0	1	1.3	1.6	2	3	4	5
Product NMR yield (%)	10	43	45	49	50	75	85	100

S9. 1,3-Aminochlorination of aryl cyclopropanes

General procedure E

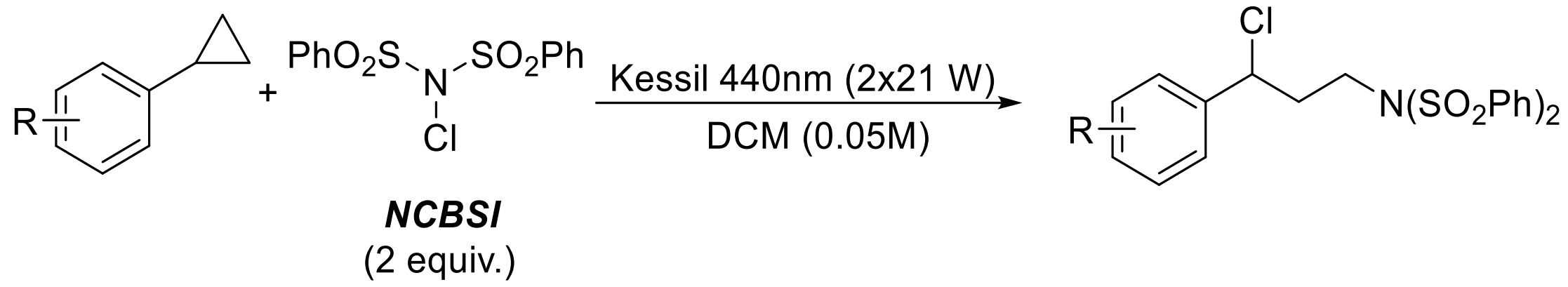

In a 15*100 mm Borosilicate glass tube, NCBSI (203 mg, 0.600 mmol, 2.0 equiv.) was added. The tube was then closed with a rubber septum and sealed off with parafilm. Three cycles of evacuate-refill with nitrogen were performed to remove O_2. DCM (6 mL, 0.05 M) was added and the solution was stirred under irradiation of Kessil lamps (440nm, 2x21W) and after few seconds the arylcyclopropane (1.0 equiv.) was added. It is important that the addition of starting material happens a few seconds after the irradiation started to ensure reproducibility. The reaction was stirred under irradiation for 8-12 h, then the solvent was removed under reduced pressure and the crude was purified by chromatography on Biotage (Büchi flashpure cartridge 25 g, Hexane:AcOEt from 10 to 40%) to obtain the products.

N-(3-Chloro-3-phenylpropyl)-N-(phenylsulfonyl)benzenesulfonamide (**6a**)

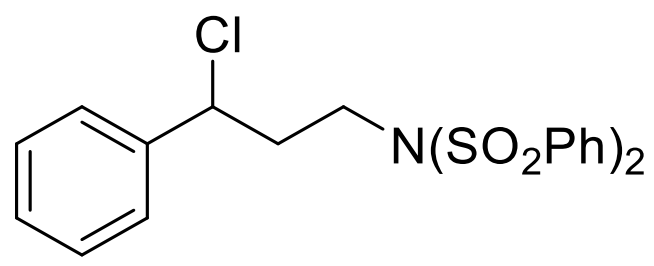

Following the general procedure E using cyclopropylbenzene **5a** (39 µL, 0.30 mmol, 1.0 equiv.), yielding N-(3-chloro-3-phenylpropyl)-N-(phenylsulfonyl)benzenesulfonamide (**6a**) as a colorless wax (131 mg, 0.290 mmol, 97%).

R$_f$ (Hex:AcOEt = 8:2) = 0.38.
^{1}H NMR (400 MHz, $CDCl_3$) δ 8.01 – 7.95 (m, 4H, Ar*H*), 7.70 – 7.62 (m, 2H, Ar*H*), 7.55 (ddt, *J* = 8.0, 6.8, 1.3 Hz, 4H, Ar*H*), 7.41 – 7.29 (m, 5H, Ar*H*), 4.87 – 4.79 (m, 1H, ArC*H*), 3.92 (ddd, *J* = 15.2, 8.3, 6.6 Hz, 1H, NCH_2), 3.83 – 3.66 (m, 1H, NCH_2), 2.52 – 2.39 (m, 2H, CH_2CH_2).
^{13}C NMR (101 MHz, $CDCl_3$) δ 140.5, 139.6, 134.1, 129.3, 128.9, 128.8, 128.3, 127.0, 60.5, 46.9, 39.6.
IR (ν_{max}, cm^{-1}) 1449 (m), 1374 (s), 1167 (s), 1086 (m), 863 (m), 751 (s), 722 (m).
HRMS (ESI/QTOF) m/z: $[M + Na]^+$ Calcd for $C_{21}H_{20}ClNNaO_4S_2^+$ 472.0414; Found 472.0414.

N-(3-([1,1'-Biphenyl]-2-yl)-3-chloropropyl)-N-(phenylsulfonyl)benzenesulfonamide (*o*-**6b**)

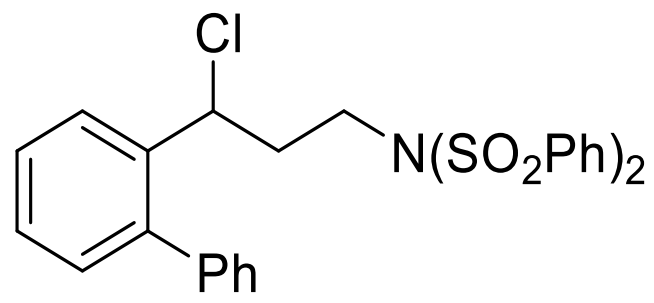

Following the general procedure E using *o*-**5b** (61 mg, 0.30 mmol, 1.0 equiv.), yielding N-(3-([1,1'-biphenyl]-2-yl)-3-chloropropyl)-N-(phenylsulfonyl) benzenesulfonamide (*o*-**6b**) as a colorless wax (155 mg, 0.290 mmol, 98%).

R_f (Hex:AcOEt = 8:2) = 0.35.
^{1}H NMR (400 MHz, $CDCl_3$) δ 7.85 – 7.80 (m, 4H, Ar*H*), 7.59 – 7.47 (m, 3H, Ar*H*), 7.42 (ddt, *J* = 8.0, 6.7, 1.3 Hz, 4H, Ar*H*), 7.38 – 7.24 (m, 5H, Ar*H*), 7.23 – 7.18 (m, 2H, Ar*H*), 7.17 – 7.10 (m, 1H, Ar*H*), 4.83 (dd, *J* = 7.7, 6.7 Hz, 1H, ArC*H*), 3.75 – 3.63 (m, 1H, NCH_2), 3.43 – 3.30 (m, 1H, NCH_2), 2.42 – 2.29 (m, 2H, CH_2CH_2).
^{13}C NMR (101 MHz, $CDCl_3$) δ 140.9, 140.0, 139.5, 138.0, 134.1, 130.3, 129.2, 129.2, 128.6, 128.4, 128.3, 127.7, 127.3, 57.0, 46.8, 39.8.
One carbon not resolved.
IR (v_{max}, cm^{-1}) 1445 (m), 1374 (s), 1352 (m), 1184 (m), 1165 (s), 1098 (m), 1083 (m), 1069 (m), 1027 (m), 871 (m), 868 (m), 857 (m), 835 (m), 799 (m), 782 (m), 778 (m), 774 (m), 759 (s), 750 (s), 745 (s), 736 (s), 723 (s), 717 (s).
HRMS (ESI/QTOF) m/z: $[M + Na]^+$ Calcd for $C_{27}H_{24}ClNNaO_4S_2^+$ 548.0727; Found 548.0737.

N-(3-([1,1'-Biphenyl]-3-yl)-3-chloropropyl)-N-(phenylsulfonyl)benzenesulfonamide (*m*-**6b**)

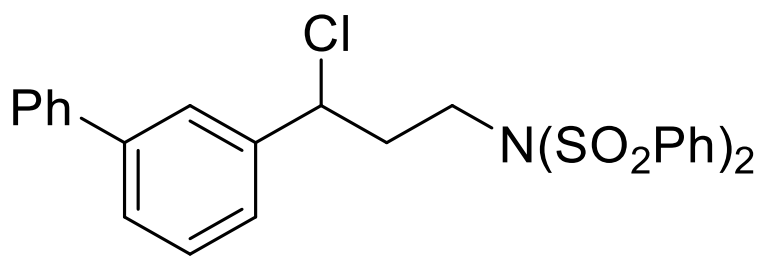

Following the general procedure E using *m*-**5b** (61 mg, 0.30 mmol, 1.0 equiv.), yielding N-(3-([1,1'-biphenyl]-3-yl)-3-chloropropyl)-N-(phenylsulfonyl) benzenesulfonamide (*m*-**6b**) as a colorless wax (62 mg, 0.12 mmol, 39%).

R_f (Hex:AcOEt = 8:2) = 0.35.
^{1}H NMR (400 MHz, $CDCl_3$) δ 8.00 – 7.95 (m, 4H, Ar*H*), 7.67 – 7.49 (m, 10H, Ar*H*), 7.50 – 7.41 (m, 3H, Ar*H*), 7.41 – 7.34 (m, 1H, Ar*H*), 7.31 (dt, *J* = 7.7, 1.5 Hz, 1H, Ar*H*), 4.90 (dd, *J* = 7.9, 6.3 Hz, 1H, ArC*H*), 3.94 (ddd, *J* = 15.1, 8.6, 6.4 Hz, 1H, NCH_2), 3.76 (ddd, *J* = 15.4, 8.8, 6.5 Hz, 1H, NCH_2), 2.58 – 2.47 (m, 2H, CH_2CH_2).
^{13}C NMR (101 MHz, $CDCl_3$) δ 142.0, 141.0, 140.7, 139.6, 134.2, 129.4, 129.3, 129.0, 128.4, 127.8, 127.6, 127.4, 125.9, 125.9, 60.4, 46.9, 39.6.
IR (v_{max}, cm^{-1}) 1449 (m), 1373 (s), 1357 (m), 1166 (s), 1084 (m), 858 (m), 840 (m), 747 (s), 724 (s).
HRMS (ESI/QTOF) m/z: $[M + Na]^+$ Calcd for $C_{27}H_{24}ClNNaO_4S_2^+$ 548.0727; Found 548.0737.

N-(3-([1,1'-Biphenyl]-4-yl)-3-chloropropyl)-N-(phenylsulfonyl)benzenesulfonamide (*p*-**6b**)

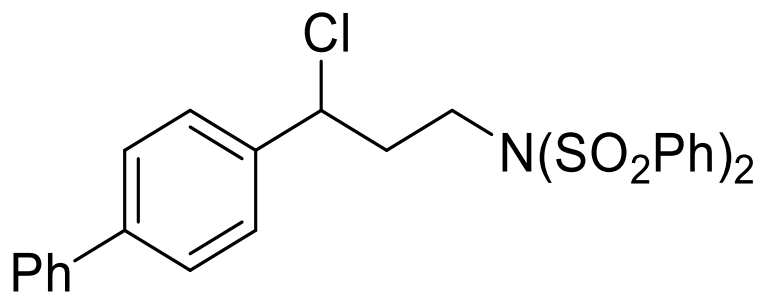

Following the general procedure E using *m*-**5b** (61 mg, 0.30 mmol, 1.0 equiv.), yielding N-(3-([1,1'-biphenyl]-4-yl)-3-chloropropyl)-N-(phenylsulfonyl) benzenesulfonamide (*p*-**6b**) as a colorless wax (84 mg, 0.16 mmol, 80%).

R_f (Hex:AcOEt = 8:2) = 0.35.
^{1}H NMR (400 MHz, $CDCl_3$) δ 8.04 – 7.96 (m, 4H, Ar*H*), 7.72 – 7.62 (m, 2H, Ar*H*), 7.57 (ddd, *J* = 14.1, 8.4, 6.9 Hz, 8H, Ar*H*), 7.50 – 7.43 (m, 2H, Ar*H*), 7.39 (dd, *J* = 7.6, 5.4 Hz, 3H), 4.89 (t, *J* = 7.1 Hz, 1H, ArC*H*), 4.01 – 3.87 (m, 1H, NCH_2), 3.82 – 3.68 (m, 1H, NCH_2), 2.58 – 2.43 (m, 2H, CH_2CH_2).
^{13}C NMR (101 MHz, $CDCl_3$) δ 141.8, 140.5, 139.6, 139.5, 134.2, 129.4, 129.0, 128.4, 127.8, 127.7, 127.5, 127.2, 60.2, 46.9, 39.6.
IR (ν_{max}, cm^{-1}) 1445 (m), 1375 (s), 1350 (m), 1191 (m), 1184 (m), 1168 (s), 1091 (m), 1084 (m), 872 (m), 867 (m), 861 (m), 858 (m), 850 (m), 778 (m), 773 (m), 765 (s), 755 (s), 750 (s), 744 (s), 739 (m), 733 (m), 723 (m), 718 (m).
HRMS (ESI/QTOF) m/z: $[M + Na]^+$ Calcd for $C_{27}H_{24}ClNNaO_4S_2^+$ 548.0727; Found 548.0721.

N-(3-Chloro-3-(o-tolyl)propyl)-N-(phenylsulfonyl)benzenesulfonamide (*o*-**6c**)

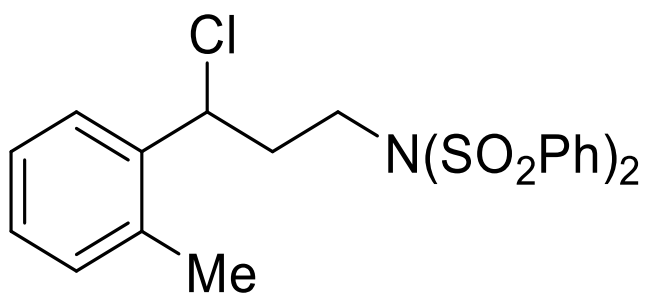

Following the general procedure E using *o*-**5c** (41 mg, 0.30 mmol, 1.0 equiv.), yielding N-(3-chloro-3-(o-tolyl)propyl)-N-(phenylsulfonyl)benzenesulfonamide (*o*-**6c**) as a colorless wax (49 mg, 0.20 mmol, 35%).

R_f (Hex:AcOEt = 8:2) = 0.37.
^{1}H NMR (400 MHz, $CDCl_3$) δ 8.04 – 7.94 (m, 4H, Ar*H*), 7.73 – 7.62 (m, 2H, Ar*H*), 7.60 – 7.51 (m, 4H, Ar*H*), 7.44 – 7.35 (m, 1H, Ar*H*), 7.25 – 7.18 (m, 2H, Ar*H*), 7.18 – 7.11 (m, 1H, Ar*H*), 5.09 (dd, *J* = 8.2, 6.0 Hz, 1H, ArC*H*), 4.03 – 3.91 (m, 1H, NCH_2), 3.79 (ddd, *J* = 15.4, 8.8, 6.6 Hz, 1H, NCH_2), 2.54 – 2.44 (m, 2H, CH_2CH_2), 2.33 (s, 3H, CH_3).
^{13}C NMR (101 MHz, $CDCl_3$) δ 139.6, 138.4, 135.5, 134.2, 130.9, 129.3, 128.6, 128.4, 126.8, 126.4, 56.9, 47.2, 38.4, 19.2.
IR (ν_{max}, cm^{-1}) 1479 (w), 1449 (m), 1375 (s), 1168 (s), 1085 (m), 861 (m), 751 (s).
HRMS (ESI/QTOF) m/z: $[M + Na]^+$ Calcd for $C_{22}H_{22}ClNNaO_4S_2^+$ 486.0571; Found 486.0573.

N-(3-Chloro-3-(p-tolyl)propyl)-N-(phenylsulfonyl)benzenesulfonamide (*p*-**6c**)

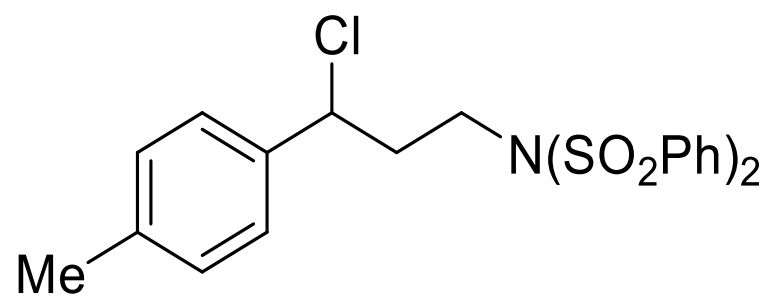

Following the general procedure E using *p*-**5c** (41 mg, 0.30 mmol, 1.0 equiv.), yielding N-(3-chloro-3-(p-tolyl)propyl)-N-(phenylsulfonyl)benzenesulfonamide (*p*-**6c**) as a colorless wax (49 mg, 0.20 mmol, 35%).

R_f (Hex:AcOEt = 8:2) = 0.37.
^{1}H NMR (400 MHz, $CDCl_3$) δ 8.00 – 7.95 (m, 4H, Ar*H*), 7.71 – 7.63 (m, 2H, Ar*H*), 7.55 (dd, *J* = 8.5, 7.2 Hz, 4H, Ar*H*), 7.19 (q, *J* = 8.2 Hz, 4H, Ar*H*), 4.81 (t, *J* = 7.2 Hz, 1H, ArC*H*), 3.97 – 3.84 (m, 1H, NCH_2), 3.72 (dt, *J* = 15.4, 7.5 Hz, 1H, NCH_2), 2.50 – 2.41 (m, 2H, CH_2CH_2), 2.36 (s, 3H, CH_3).
^{13}C NMR (101 MHz, $CDCl_3$) δ 139.7, 138.7, 137.6, 134.1, 129.6, 129.3, 128.4, 126.9, 60.5, 47.0, 39.5, 21.3.
IR (ν_{max}, cm^{-1}) 1449 (m), 1375 (s), 1169 (s), 1087 (m), 863 (m), 755 (s).
HRMS (ESI/QTOF) m/z: $[M + Na]^+$ Calcd for $C_{22}H_{22}ClNNaO_4S_2^+$ 486.0571; Found 486.0573.

N-(3-Chloro-3-(2-ethylphenyl)propyl)-N-(phenylsulfonyl)benzenesulfonamide (*o*-**6d**)

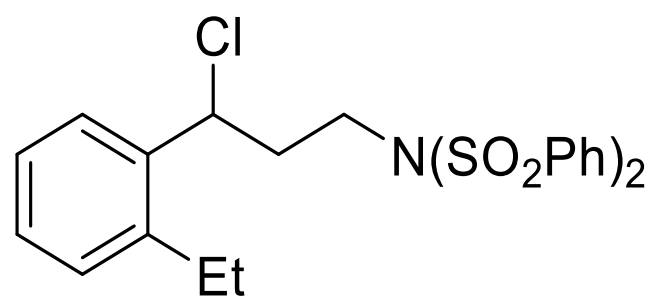

Following the general procedure E using *o*-**5d** (45 mg, 0.30 mmol, 1.0 equiv.), yielding N-(3-chloro-3-(2-ethylphenyl)propyl)-N-(phenylsulfonyl) benzenesulfonamide (*o*-**6d**)) as a colorless wax (107 mg, 0.22 mmol, 75%).

R_f (Hex:AcOEt = 8:2) = 0.37.
^{1}H NMR (400 MHz, $CDCl_3$) δ 7.90 – 7.84 (m, 4H, Ar*H*), 7.57 – 7.48 (m, 2H, Ar*H*), 7.41 (dd, *J* = 8.5, 7.1 Hz, 4H, Ar*H*), 7.32 – 7.27 (m, 1H, Ar*H*), 7.15 – 7.10 (m, 2H, Ar*H*), 7.07 – 7.03 (m, 1H, Ar*H*), 5.03 (dd, *J* = 8.5, 5.8 Hz, 1H, ArC*H*), 3.86 (ddd, *J* = 15.0, 9.2, 5.8 Hz, 1H, NCH_2), 3.65 (ddd, *J* = 15.4, 9.1, 6.5 Hz, 1H, NCH_2), 2.53 (qd, *J* = 7.5, 1.3 Hz, 2H, CH_3CH_2), 2.37 (dtd, *J* = 11.2, 5.7, 3.0 Hz, 2H, CH_2CH_2), 1.08 (t, *J* = 7.6 Hz, 3H, CH_2CH_3).
^{13}C NMR (101 MHz, $CDCl_3$) δ 141.2, 139.6, 138.0, 134.1, 129.3, 129.1, 128.8, 128.3, 126.8, 126.8, 56.2, 47.2, 39.1, 25.6, 15.6.
IR (ν_{max}, cm^{-1}) 1712 (w), 1449 (m), 1374 (s), 1167 (s), 1086 (m), 861 (m), 751 (s)
HRMS (ESI/QTOF) m/z: $[M + Na]^+$ Calcd for $C_{23}H_{24}ClNNaO_4S_2^+$ 500.0727; Found 500.0707.

N-(3-Chloro-3-(3-ethylphenyl)propyl)-N-(phenylsulfonyl)benzenesulfonamide (*m*-**6d**)

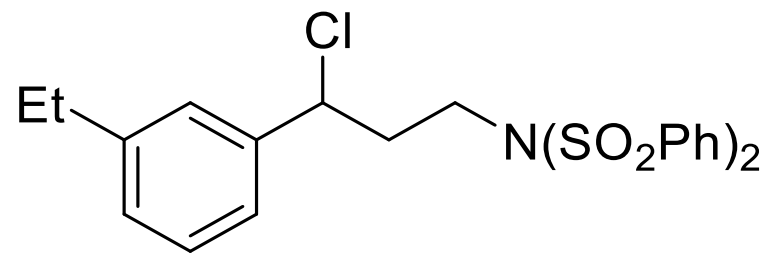

Following the general procedure E using *m*-**5d** (45 mg, 0.30 mmol, 1.0 equiv.), yielding N-(3-chloro-3-(3-ethylphenyl)propyl)-N-(phenylsulfonyl) benzenesulfonamide (*m*-**6d**)) as a colorless wax (52 mg, 0.11 mmol, 36%).

R_f (Hex:AcOEt = 8:2) = 0.37.
^{1}H NMR (400 MHz, $CDCl_3$) δ 8.01 – 7.93 (m, 4H, Ar*H*), 7.71 – 7.62 (m, 2H, Ar*H*), 7.55 (dd, *J* = 8.5, 7.2 Hz, 5H, Ar*H*), 7.32 (d, *J* = 8.2 Hz, 1H, Ar*H*), 7.18 (d, *J* = 2.3 Hz, 1H, Ar*H*), 7.09 (dd, *J* = 8.2, 2.4 Hz, 1H, Ar*H*), 4.79 (t, *J* = 7.1 Hz, 1H, ArC*H*), 3.96 – 3.83 (m, 1H, NCH_2), 3.71 (dt, *J* = 15.4, 7.3 Hz, 1H, NCH_2), 2.76 (q, *J* = 7.5 Hz, 2H, CH_3CH_2), 2.44 (q, *J* = 7.3 Hz, 2H, CH_2CH_2), 1.24 (t, *J* = 7.7 Hz, 3H, CH_2CH_3).
^{13}C NMR (101 MHz, $CDCl_3$) δ 142.3, 139.6, 139.2, 134.2, 129.8, 129.3, 129.3, 128.3, 128.1, 125.6, 59.8, 46.8, 39.5, 26.9, 14.0.
IR (ν_{max}, cm^{-1}) 1478 (m), 1451 (m), 1377 (s), 1171 (s), 1089 (m), 1069 (m), 858 (s), 756 (s).
HRMS (ESI/QTOF) m/z: $[M + Na]^+$ Calcd for $C_{23}H_{24}ClNNaO_4S_2^+$ 500.0727; Found 500.0714.

N-(3-Chloro-3-(4-ethylphenyl)propyl)-N-(phenylsulfonyl)benzenesulfonamide (*p*-**6d**)

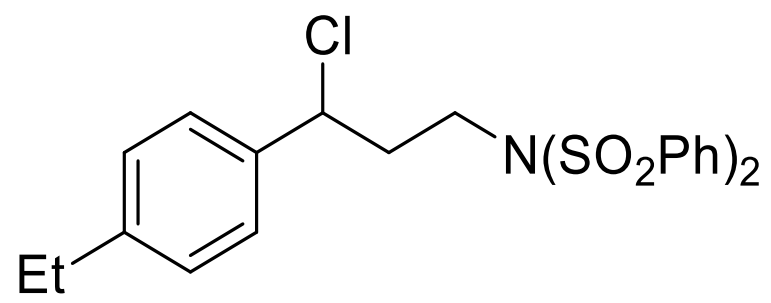

Following the general procedure E using *p*-**5d** (45 mg, 0.30 mmol, 1.0 equiv.), yielding N-(3-chloro-3-(4-ethylphenyl)propyl)-N-(phenylsulfonyl) benzenesulfonamide (*p*-**6d**)) as a colorless wax (85 mg, 0.18 mmol, 59%).

R_f (Hex:AcOEt = 8:2) = 0.37.
^{1}H NMR (400 MHz, $CDCl_3$) δ 7.94 – 7.86 (m, 4H, Ar*H*), 7.61 – 7.53 (m, 2H, Ar*H*), 7.49 – 7.42 (m, 4H, Ar*H*), 7.18 – 7.08 (m, 4H, Ar*H*), 4.74 (t, *J* = 7.2 Hz, 1H, ArC*H*), 3.83 (ddd, *J* = 15.2, 8.1, 6.6 Hz, 1H, NCH_2), 3.64 (dt, *J* = 15.4, 7.4 Hz, 1H, NCH_2), 2.57 (q, *J* = 7.6 Hz, 2H, CH_3CH_2), 2.43 – 2.34 (m, 2H, CH_2CH_2), 1.16 (t, *J* = 7.6 Hz, 3H, CH_2CH_3).
^{13}C NMR (101 MHz, $CDCl_3$) δ 145.0, 139.6, 137.8, 134.1, 129.3, 128.4, 128.3, 127.0, 60.5, 47.0, 39.5, 28.6, 15.6.
IR (ν_{max}, cm^{-1}) 1450 (m), 1373 (s), 1166 (s), 1085 (m), 857 (m), 752 (s).
HRMS (ESI/QTOF) m/z: $[M + Na]^+$ Calcd for $C_{23}H_{24}ClNNaO_4S_2^+$ 500.0727; Found 500.0714.

N-(3-(4-(Tert-butyl)phenyl)-3-chloropropyl)-N-(phenylsulfonyl)benzenesulfonamide (**6e**)

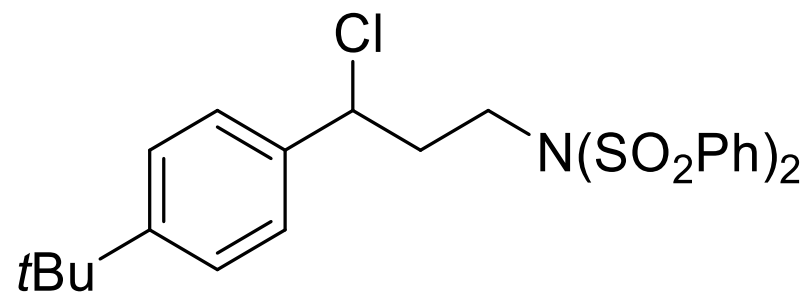

Following the general procedure E using **5e** (54 mg, 0.30 mmol, 1.0 equiv.), yielding N-(3-(4-(tert-butyl)phenyl)-3-chloropropyl)-N-(phenylsulfonyl) benzenesulfonamide (**6e**) as a colorless wax (92 mg, 0.18 mmol, 61%).

R_f (Hex:AcOEt = 8:2) = 0.38.
^{1}H NMR (400 MHz, $CDCl_3$) δ 8.02 – 7.92 (m, 4H, Ar*H*), 7.72 – 7.62 (m, 2H, Ar*H*), 7.61 – 7.50 (m, 4H, Ar*H*), 7.44 – 7.36 (m, 2H, Ar*H*), 7.33 – 7.19 (m, 2H, Ar*H*), 4.83 (dd, *J* = 8.2, 6.1 Hz, 1H, ArC*H*), 4.00 – 3.86 (m, 1H, NC*H₂*), 3.72 (ddd, *J* = 15.4, 9.2, 6.3 Hz, 1H, NC*H₂*), 2.54 – 2.38 (m, 2H, CH₂C*H₂*), 1.33 (s, 9H, CC*H₃*).
^{13}C NMR (101 MHz, $CDCl_3$) δ 151.9, 139.6, 137.5, 134.1, 129.3, 128.4, 126.7, 125.9, 60.4, 47.0, 39.5, 34.8, 31.4.
IR (v_{max}, cm^{-1}) 1449 (m), 1375 (s), 1168 (s), 1086 (m), 862 (m), 755 (s), 737 (s).
HRMS (nanochip-ESI/LTQ-Orbitrap) m/z: $[M + Na]^+$ Calcd for $C_{25}H_{28}ClNNaO_4S_2^+$ 528.1040; Found 528.1058.

N-(3-Chloro-3-(4-(2-oxopropyl)phenyl)propyl)-N-(phenylsulfonyl)benzenesulfonamide (**6f**)

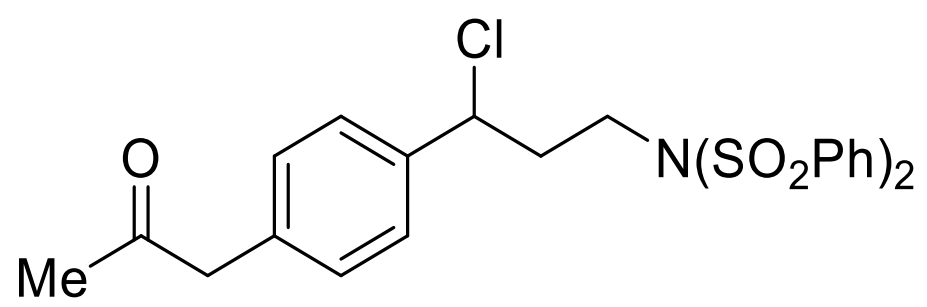

Following the general procedure E using **5f** (54 mg, 0.30 mmol, 1.0 equiv.), yielding N-(3-chloro-3-(4-(2-oxopropyl)phenyl)propyl)-N-(phenylsulfonyl) benzenesulfonamide (**6f**) as a colorless wax (123 mg, 0.240 mmol, 81%).

R_f (Hex:AcOEt = 8:2) = 0.31.
^{1}H NMR (400 MHz, $CDCl_3$) δ 8.01 – 7.90 (m, 4H, Ar*H*), 7.71 – 7.60 (m, 2H, Ar*H*), 7.55 (td, *J* = 7.8, 2.4 Hz, 4H, Ar*H*), 7.33 – 7.24 (m, 2H, Ar*H*), 7.22 – 7.12 (m, 2H, Ar*H*), 4.86 – 4.77 (m, 1H, ArC*H*), 3.98 – 3.85 (m, 1H, NC*H₂*), 3.82 – 3.66 (m, 3H, COC*H₂*, NC*H₂*), 2.53 – 2.39 (m, 2H, CH₂C*H₂*), 2.17 (s, 3H, COC*H₃*).
^{13}C NMR (101 MHz, $CDCl_3$) δ 206.2, 139.9, 139.6, 135.1, 134.5, 130.3, 129.6, 128.6, 127.7, 60.4, 50.8, 47.2, 39.9, 30.0.
IR (v_{max}, cm^{-1}) 1713 (m), 1448 (m), 1371 (s), 1168 (s), 1086 (m), 864 (m), 755 (s).
HRMS (ESI/QTOF) m/z: $[M + Na]^+$ Calcd for $C_{24}H_{24}ClNNaO_5S_2^+$ 528.0677; Found 528.0654.

Methyl 2-(1-chloro-3-(N-(phenylsulfonyl)phenylsulfonamido)propyl)benzoate (*o*-**6g**)

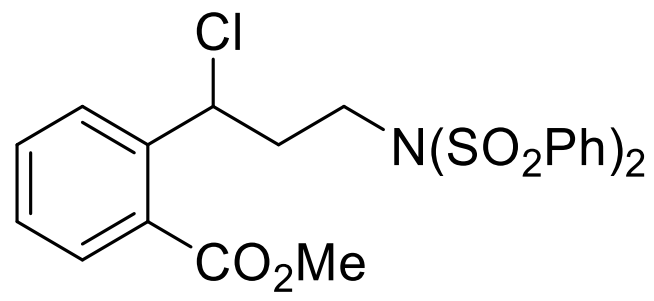

Following the general procedure E using *o*-**5g** (54 mg, 0.30 mmol, 1.0 equiv.), yielding methyl 2-(1-chloro-3-(N-(phenylsulfonyl)phenylsulfonamido)propyl) benzoate (*o*-**6g**) as a colorless wax (89 mg, 0.18 mmol, 58%).

R_f (Hex:AcOEt = 8:2) = 0.28.
^{1}H NMR (400 MHz, $CDCl_3$) δ 8.01 – 7.96 (m, 4H, Ar*H*), 7.90 (dd, *J* = 7.9, 1.4 Hz, 1H, Ar*H*), 7.75 – 7.69 (m, 1H, Ar*H*), 7.68 – 7.61 (m, 2H, Ar*H*), 7.55 (dq, *J* = 8.8, 7.5 Hz, 5H, Ar*H*), 7.37 (td, *J* = 7.6, 1.3 Hz, 1H, Ar*H*), 5.99 (dd, *J* = 9.2, 4.4 Hz, 1H, ArC*H*), 4.04 (ddd, *J* = 15.5, 11.0, 4.6 Hz, 1H, NCH_2), 3.91 (s, 3H, $COOCH_3$), 3.80 (ddd, *J* = 15.6, 11.0, 5.1 Hz, 1H, NCH_2), 2.51 (ddt, *J* = 13.5, 11.0, 4.8 Hz, 1H, CH_2CH_2), 2.39 (dddd, *J* = 13.7, 10.9, 9.2, 4.6 Hz, 1H, CH_2CH_2).
^{13}C NMR (101 MHz, $CDCl_3$) δ 167.3, 142.1, 139.6, 134.0, 132.8, 130.6, 129.2, 128.5, 128.3, 128.2, 56.5, 52.6, 47.0, 40.2.
One carbon not resolved.
IR (v_{max}, cm^{-1}) 1719 (m), 1448 (m), 1376 (m), 1267 (s), 1166 (s), 1086 (s), 1078 (m), 862 (m), 735 (s).
HRMS (nanochip-ESI/LTQ-Orbitrap) m/z: $[M + Na]^+$ Calcd for $C_{23}H_{22}ClNNaO_6S_2^+$ 530.0469; Found 530.0461.

Methyl 3-(1-chloro-3-(N-(phenylsulfonyl)phenylsulfonamido)propyl)benzoate (*m*-**6g**)

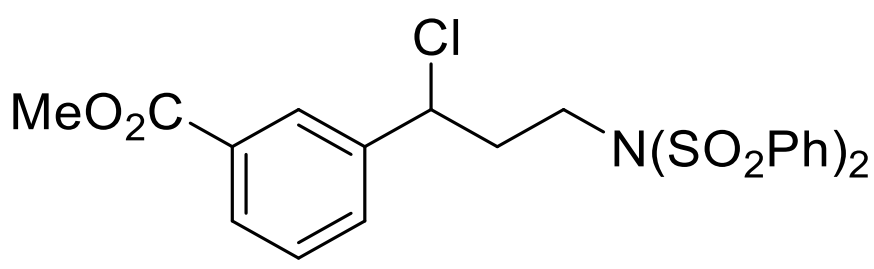

Following the general procedure E using *m*-**5g** (54 mg, 0.30 mmol, 1.0 equiv.), yielding methyl 3-(1-chloro-3-(N-(phenylsulfonyl)phenylsulfonamido)propyl) benzoate (*m*-**6g**) as a colorless wax (102 mg, 0.200 mmol, 67%).

R_f (Hex:AcOEt = 8:2) = 0.28.
^{1}H NMR (400 MHz, $CDCl_3$) δ 8.03 – 7.93 (m, 6H, Ar*H*), 7.69 – 7.62 (m, 2H, Ar*H*), 7.59 – 7.52 (m, 5H, Ar*H*), 7.44 (t, *J* = 7.7 Hz, 1H, Ar*H*), 4.87 (t, *J* = 7.1 Hz, 1H, ArC*H*), 3.99 – 3.87 (m, 4H, $COOCH_3$, NCH_2), 3.82 – 3.68 (m, 1H, NCH_2), 2.45 (q, *J* = 7.7 Hz, 2H, CH_2CH_2).
^{13}C NMR (101 MHz, $CDCl_3$) δ 166.5, 141.0, 139.5, 134.2, 131.4, 130.9, 129.9, 129.3, 129.1, 128.3, 128.1, 59.7, 52.4, 46.8, 39.5.
IR (v_{max}, cm^{-1}) 1721 (s), 1450 (m), 1371 (s), 1288 (m), 1163 (s), 1078 (m), 854 (m), 738 (s).
HRMS (ESI/QTOF) m/z: $[M + Na]^+$ Calcd for $C_{23}H_{22}ClNNaO_6S_2^+$ 530.0469; Found 530.0458.

Methyl 4-(1-chloro-3-(N-(phenylsulfonyl)phenylsulfonamido)propyl)benzoate (*p*-**6g**)

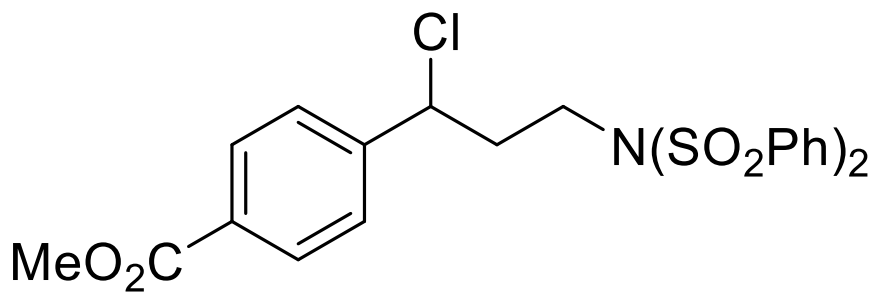

Following the general procedure E using *p*-**5g** (54 mg, 0.30 mmol, 1.0 equiv.), yielding methyl 4-(1-chloro-3-(N-(phenylsulfonyl)phenylsulfonamido)propyl) benzoate (*p*-**6g**) as a colorless wax (125 mg, 0.250 mmol, 82%).

R_f (Hex:AcOEt = 8:2) = 0.28.
^{1}H NMR (400 MHz, $CDCl_3$) δ 8.07 – 8.01 (m, 2H, Ar*H*), 8.01 – 7.91 (m, 4H, Ar*H*), 7.72 – 7.61 (m, 2H, Ar*H*), 7.54 (dd, *J* = 8.4, 7.2 Hz, 4H, Ar*H*), 7.44 – 7.34 (m, 2H, Ar*H*), 4.87 (t, *J* = 7.0 Hz, 1H, ArC*H*), 3.98 – 3.85 (m, 4H, COOC*H*$_3$, NC*H*$_2$), 3.76 (dt, *J* = 15.3, 7.3 Hz, 1H, NC*H*$_2$), 2.44 (q, *J* = 7.5 Hz, 2H, CH_2C*H*$_2$).
^{13}C NMR (101 MHz, $CDCl_3$) δ 166.5, 145.2, 139.5, 134.2, 130.5, 130.2, 129.3, 128.3, 127.0, 59.5, 52.4, 46.7, 39.4.
IR (ν_{max}, cm^{-1}) 1721 (s), 1447 (m), 1374 (s), 1281 (s), 1166 (s), 1086 (s), 864 (m), 753 (s).
HRMS (ESI/QTOF) m/z: [M + Na]$^+$ Calcd for $C_{23}H_{22}ClNNaO_6S_2^+$ 530.0469; Found 530.0458.

N-(3-(3-Acetylphenyl)-3-chloropropyl)-N-(phenylsulfonyl)benzenesulfonamide (*m*-**6h**)

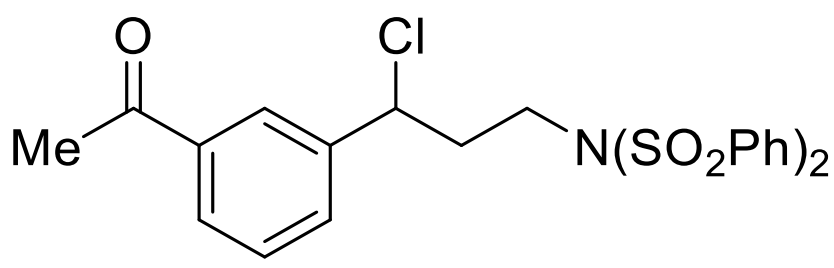

Following the general procedure E using *m*-**5h** (50 mg, 0.30 mmol, 1.0 equiv.), yielding N-(3-(3-acetylphenyl)-3-chloropropyl)-N-(phenylsulfonyl) benzenesulfonamide (*m*-**6h**) as a colorless wax (135 mg, 0.270 mmol, 91%).

R_f (Hex:AcOEt = 8:2) = 0.33.
^{1}H NMR (400 MHz, $CDCl_3$) δ 8.02 – 7.93 (m, 4H, Ar*H*), 7.90 (dd, *J* = 7.4, 1.5 Hz, 2H, Ar*H*), 7.70 – 7.59 (m, 2H, Ar*H*), 7.59 – 7.49 (m, 5H, Ar*H*), 7.46 (td, *J* = 7.4, 1.0 Hz, 1H, Ar*H*), 4.89 (t, *J* = 7.1 Hz, 1H, ArC*H*), 4.00 – 3.86 (m, 1H, NC*H*$_2$), 3.75 (dt, *J* = 15.4, 7.4 Hz, 1H, NC*H*$_2$), 2.61 (s, 3H, COC*H*$_3$), 2.46 (q, *J* = 7.5 Hz, 2H, CH_2C*H*$_2$).
^{13}C NMR (101 MHz, $CDCl_3$) δ 197.5, 141.2, 139.5, 137.6, 134.2, 131.6, 129.3, 128.7, 128.2, 126.6, 59.7, 46.7, 39.5, 26.8.
One carbon not resolved.
IR (ν_{max}, cm^{-1}) 1685 (m), 1445 (m), 1373 (m), 1356 (m), 1279 (m), 1266 (m), 1184 (m), 1165 (s), 1081 (m), 1065 (m), 866 (m), 859 (m), 838 (m), 777 (m), 761 (m), 752 (s), 734 (s), 719 (s).
HRMS (ESI/QTOF) m/z: [M + H]$^+$ Calcd for $C_{23}H_{23}ClNO_5S_2^+$ 492.0701; Found 492.0715.

N-(3-(4-Acetylphenyl)-3-chloropropyl)-N-(phenylsulfonyl)benzenesulfonamide (*p*-**6h**)

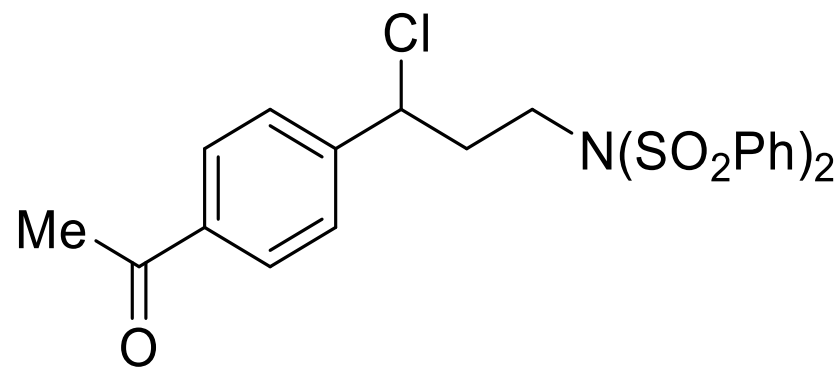

Following the general procedure E using *p*-**5h** (50 mg, 0.30 mmol, 1.0 equiv.), yielding N-(3-(4-acetylphenyl)-3-chloropropyl)-N-(phenylsulfonyl) benzenesulfonamide (*p*-**6h**) as a colorless wax (108 mg, 0.220 mmol, 73%).

R_f (Hex:AcOEt = 8:2) = 0.33.
^{1}H NMR (400 MHz, $CDCl_3$) δ 8.10 – 7.83 (m, 6H, Ar*H*), 7.74 – 7.62 (m, 2H, Ar*H*), 7.55 (ddt, *J* = 8.0, 6.4, 1.3 Hz, 4H, Ar*H*), 7.46 – 7.38 (m, 2H, Ar*H*), 4.92 – 4.79 (m, 1H, ArC*H*), 3.92 (ddd, *J* = 15.0, 8.2, 6.6 Hz, 1H, NCH_2), 3.76 (ddd, *J* = 15.3, 8.3, 6.9 Hz, 1H, NCH_2), 2.61 (s, 3H, COCH_3), 2.54 – 2.36 (m, 2H, CH_2CH_2).
^{13}C NMR (101 MHz, $CDCl_3$) δ 197.5, 145.4, 139.6, 137.4, 134.2, 129.4, 129.0, 128.3, 127.3, 59.5, 46.7, 39.5, 26.8.
IR (ν_{max}, cm^{-1}) 1684 (s), 1609 (w), 1447 (m), 1368 (s), 1267 (s), 1166 (s), 1084 (m), 863 (m), 741 (s).
HRMS (APCI/QTOF) m/z: $[M + Na]^+$ Calcd for $C_{23}H_{22}ClNNaO_5S_2^+$ 514.0520; Found 514.0511.

N-(3-Chloro-3-(2-(trifluoromethyl)phenyl)propyl)-N-(phenylsulfonyl) benzenesulfonamide (*o*-**6i**)

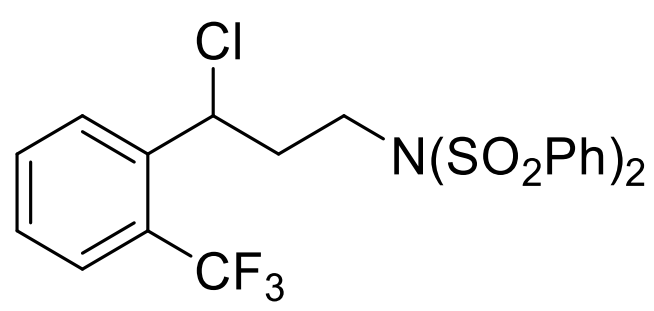

Following the general procedure E using *o*-**5i** (58 mg, 0.30 mmol, 1.0 equiv.), yielding N-(3-chloro-3-(2-(trifluoromethyl)phenyl)propyl)-N-(phenylsulfonyl) benzenesulfonamide (*o*-**6i**)) as a colorless wax (54 mg, 0.10 mmol, 35%).

R_f (Hex:AcOEt = 8:2) = 0.35.
^{1}H NMR (400 MHz, $CDCl_3$) δ 7.97 (dt, *J* = 8.0, 1.0 Hz, 4H, Ar*H*), 7.76 – 7.71 (m, 1H, Ar*H*), 7.69 – 7.58 (m, 4H, Ar*H*), 7.58 – 7.51 (m, 4H, Ar*H*), 7.43 (ddt, *J* = 8.4, 7.5, 1.0 Hz, 1H, Ar*H*), 5.25 – 5.16 (m, 1H, ArC*H*), 4.12 – 3.87 (m, 1H, NCH_2), 3.67 (ddd, *J* = 15.5, 8.7, 6.9 Hz, 1H, NCH_2), 2.50 – 2.38 (m, 2H, CH_2CH_2).
^{19}F NMR (376 MHz, $CDCl_3$) δ -58.3.
^{13}C NMR (101 MHz, $CDCl_3$) δ 139.4, 139.4, 134.07, 132.7, 129.2, 129.2, 128.6, 128.2, 126.9 (q, *J* = 30.2 Hz), 125.6 (q, *J* = 5.8 Hz), 123.9 (q, *J* = 274.0 Hz), 54.7 (d, *J* = 2.7 Hz), 46.7, 40.1.
IR (ν_{max}, cm^{-1}) 1450 (m), 1375 (s), 1315 (s), 1166 (s), 1164 (s), 1120 (s), 1087 (m), 1036 (m), 863 (m), 755 (s).
HRMS (nanochip-ESI/LTQ-Orbitrap) m/z: $[M + H]^+$ Calcd for $C_{22}H_{20}ClF_3NO_4S_2^+$ 518.0469; Found 518.0486.

N-(3-Chloro-3-(3-(trifluoromethyl)phenyl)propyl)-N-(phenylsulfonyl) benzenesulfonamide (*m*-**6i**)

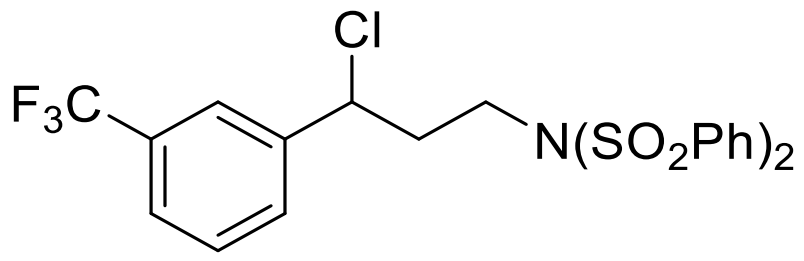

Following the general procedure E using *m*-**5i** (58 mg, 0.30 mmol, 1.0 equiv.), yielding N-(3-chloro-3-(3-(trifluoromethyl)phenyl)propyl)-N-(phenylsulfonyl) benzenesulfonamide (*m*-**6i**) as a colorless wax (69 mg, 0.13 mmol, 44%).

R_f (Hex:AcOEt = 8:2) = 0.35.
^{1}H NMR (400 MHz, $CDCl_3$) δ 8.10 – 7.90 (m, 4H, A*rH*), 7.74 – 7.64 (m, 2H, A*rH*), 7.64 – 7.46 (m, 8H, A*rH*), 4.87 (dd, *J* = 8.2, 5.9 Hz, 1H, ArC*H*), 4.08 – 3.86 (m, 1H, NC*H₂*), 3.78 (ddd, *J* = 15.3, 8.8, 6.5 Hz, 1H, NC*H₂*), 2.50 – 2.37 (m, 2H, CH_2CH_2).
^{19}F NMR (376 MHz, $CDCl_3$) δ -62.6.
^{13}C NMR (101 MHz, $CDCl_3$) δ 141.6, 139.6, 134.3, 131.3 (q, *J* = 32.6 Hz), 130.4 (d, *J* = 1.5 Hz), 129.6, 129.4, 128.3, 125.6 (q, *J* = 3.7 Hz), 123.9 (q, *J* = 272.5 Hz), 123.8 (q, *J* = 3.8 Hz), 59.4, 46.7, 39.6.
IR (ν_{max}, cm^{-1}) 1450 (m), 1379 (m), 1329 (s), 1166 (s), 1127 (s), 1076 (m), 859 (m), 755 (s).
HRMS (ESI/QTOF) m/z: $[M + Na]^+$ Calcd for $C_{22}H_{19}ClF_3NNaO_4S_2^+$ 540.0288; Found 540.0293.

N-(3-Chloro-3-(4-(trifluoromethyl)phenyl)propyl)-N-(phenylsulfonyl) benzenesulfonamide (*p*-**6i**)

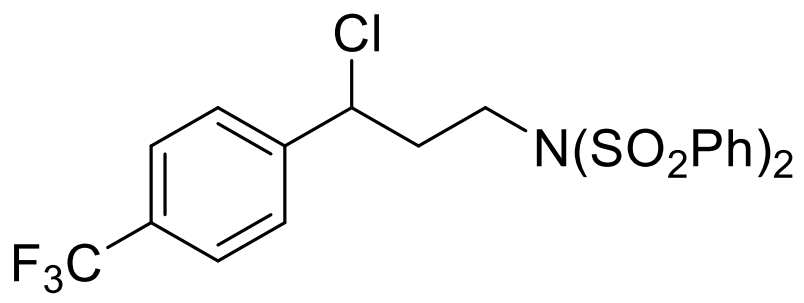

Following the general procedure E using *p*-**5i** (58 mg, 0.30 mmol, 1.0 equiv.), yielding N-(3-chloro-3-(4-(trifluoromethyl)phenyl)propyl)-N-(phenylsulfonyl) benzenesulfonamide (*p*-**6i**) as a colorless wax (52 mg, 0.10 mmol, 33%).

R_f (Hex:AcOEt = 8:2) = 0.35.
^{1}H NMR (400 MHz, $CDCl_3$) δ 7.97 (dt, *J* = 7.3, 1.3 Hz, 4H, A*rH*), 7.72 – 7.59 (m, 4H, A*rH*), 7.55 (dd, *J* = 8.5, 7.2 Hz, 4H, A*rH*), 7.44 (d, *J* = 8.1 Hz, 2H, A*rH*), 4.87 (dd, *J* = 7.9, 6.2 Hz, 1H, ArC*H*), 3.91 (ddd, *J* = 15.0, 8.5, 6.6 Hz, 1H, NC*H₂*), 3.74 (ddd, *J* = 15.4, 8.5, 6.7 Hz, 1H, NC*H₂*), 2.57 – 2.34 (m, 2H, CH_2CH_2).
^{19}F NMR (376 MHz, $CDCl_3$) δ -62.7.
^{13}C NMR (101 MHz, $CDCl_3$) δ 144.4 (d, *J* = 1.6 Hz), 139.5, 134.2, 130.9 (q, *J* = 32.7 Hz), 129.4, 128.3, 127.5, 125.9 (q, *J* = 3.7 Hz), 123.9 (q, *J* = 272.1 Hz), 59.3, 46.6, 39.5.
IR (ν_{max}, cm^{-1}) 1449 (m), 1375 (s), 1325 (s), 1166 (s), 1124 (s), 1086 (m), 1068 (s), 863 (m), 757 (s).

HRMS (nanochip-ESI/LTQ-Orbitrap) m/z: $[M + Na]^+$ Calcd for $C_{22}H_{19}ClF_3NNaO_4S_2^+$ 540.0288; Found 540.0306.

N-(3-Chloro-3-(2-cyanophenyl)propyl)-N-(phenylsulfonyl)benzenesulfonamide (*o*-**6j**)

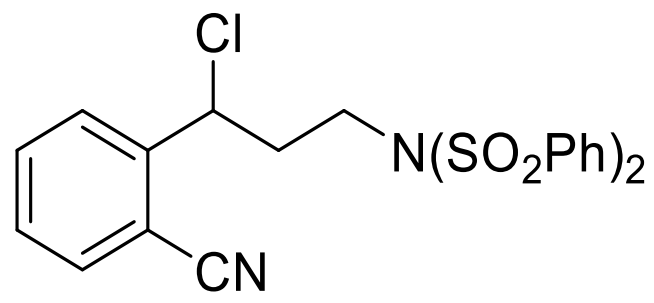

Following the general procedure E using *o*-**5j** (44 mg, 0.30 mmol, 1.0 equiv.), yielding N-(3-chloro-3-(2-cyanophenyl)propyl)-N-(phenylsulfonyl) benzenesulfonamide (*o*-**6j**) as a colorless wax (54 mg, 0.11 mmol, 38%).

$\mathbf{R_f}$ (Hex:AcOEt = 8:2) = 0.24.
^{1}H NMR (400 MHz, $CDCl_3$) δ 7.91 (dd, *J* = 7.6, 1.7 Hz, 4H, Ar*H*), 7.63 – 7.44 (m, 9H, Ar*H*), 7.38 – 7.33 (m, 1H, Ar*H*), 5.13 (dd, *J* = 8.9, 5.3 Hz, 1H, ArC*H*), 3.92 (ddd, *J* = 15.4, 10.3, 5.2 Hz, 1H, NCH_2), 3.68 (ddd, *J* = 15.5, 10.3, 5.6 Hz, 1H, NCH_2), 2.53 – 2.31 (m, 2H, CH_2CH_2).
^{13}C NMR (101 MHz, $CDCl_3$) δ 143.6, 139.5, 134.2, 133.6, 133.2, 129.4, 129.2, 128.3, 127.8, 116.8, 111.3, 56.8, 46.6, 38.9.
IR (ν_{max}, cm^{-1}) 2227 (w), 1685 (w), 1450 (m), 1372 (s), 1165 (s), 1084 (m), 861 (m), 754 (s), 735 (s).
HRMS (ESI/QTOF) m/z: $[M + Na]^+$ Calcd for $C_{22}H_{19}ClN_2NaO_4S_2^+$ 497.0367; Found 497.0363.

N-(3-Chloro-3-(3-cyanophenyl)propyl)-N-(phenylsulfonyl)benzenesulfonamide (*m*-**6j**)

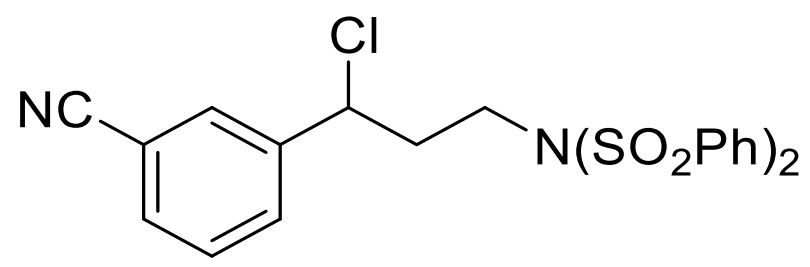

Following the general procedure E using *m*-**5j** (44 mg, 0.30 mmol, 1.0 equiv.), yielding N-(3-chloro-3-(3-cyanophenyl)propyl)-N-(phenylsulfonyl) benzenesulfonamide (*m*-**6j**) as a colorless wax (58 mg, 0.12 mmol, 41%).

$\mathbf{R_f}$ (Hex:AcOEt = 8:2) = 0.24.
^{1}H NMR (400 MHz, $CDCl_3$) δ 8.04 – 7.89 (m, 4H, Ar*H*), 7.76 – 7.66 (m, 2H, Ar*H*), 7.65 – 7.53 (m, 7H, Ar*H*), 7.53 – 7.40 (m, 1H, Ar*H*), 4.83 (dd, *J* = 8.5, 5.6 Hz, 1H, ArC*H*), 3.92 (ddd, *J* = 14.7, 8.5, 5.9 Hz, 1H, NCH_2), 3.78 (ddd, *J* = 15.3, 8.8, 6.5 Hz, 1H, NCH_2), 2.40 (ddd, *J* = 10.8, 8.4, 5.6 Hz, 2H, CH_2CH_2).
^{13}C NMR (101 MHz, $CDCl_3$) δ 142.1, 139.5, 134.3, 132.3, 131.4, 130.6, 129.9, 129.4, 128.3, 118.3, 113.1, 58.8, 46.6, 39.6.
IR (ν_{max}, cm^{-1}) 2232 (w), 1448 (m), 1372 (s), 1166 (s), 1087 (m), 856 (m), 753 (s).
HRMS (ESI/QTOF) m/z: $[M + Na]^+$ Calcd for $C_{22}H_{19}ClN_2NaO_4S_2^+$ 497.0367; Found 497.0375.

N-(3-Chloro-3-(4-cyanophenyl)propyl)-N-(phenylsulfonyl)benzenesulfonamide (*p*-**6j**)

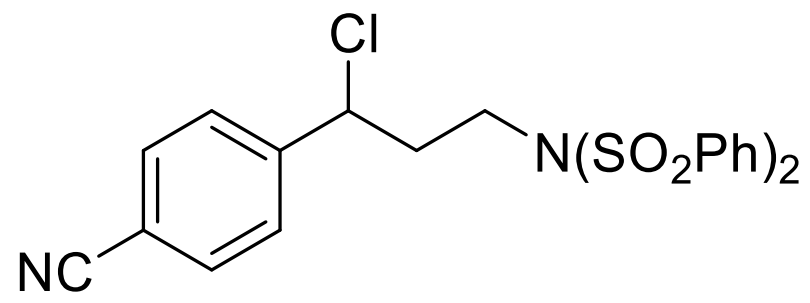

Following the general procedure E using *p*-**5j** (44 mg, 0.30 mmol, 1.0 equiv.), yielding N-(3-chloro-3-(4-cyanophenyl)propyl)-N-(phenylsulfonyl) benzenesulfonamide (*p*-**6j**) as a colorless wax (46 mg, 0.10 mmol, 32%).

R_f (Hex:AcOEt = 8:2) = 0.24.
^{1}H NMR (400 MHz, $CDCl_3$) δ 8.04 – 7.88 (m, 4H, Ar*H*), 7.80 – 7.63 (m, 4H, Ar*H*), 7.56 (t, *J* = 7.8 Hz, 4H, Ar*H*), 7.50 – 7.38 (m, 2H, Ar*H*), 4.85 (dd, *J* = 8.3, 5.8 Hz, 1H, ArC*H*), 3.91 (ddd, *J* = 14.9, 8.5, 6.2 Hz, 1H, NCH_2), 3.77 (ddd, *J* = 15.3, 8.7, 6.5 Hz, 1H, NCH_2), 2.52 – 2.35 (m, 2H, CH_2CH_2).
^{13}C NMR (101 MHz, $CDCl_3$) δ 145.5, 139.4, 134.3, 132.8, 129.4, 128.3, 127.8, 118.4, 112.7, 59.1, 46.6, 39.4.
IR (ν_{max}, cm^{-1}) 2230 (w), 1448 (m), 1376 (s), 1166 (s), 1084 (m), 863 (m), 755 (s).
HRMS (ESI/QTOF) m/z: $[M + Na]^+$ Calcd for $C_{22}H_{19}ClN_2NaO_4S_2^+$ 497.0367; Found 497.0378.

N-(3-Chloro-3-(2-(trifluoromethoxy)phenyl)propyl)-N-(phenylsulfonyl) benzenesulfonamide (*o*-**6k**)

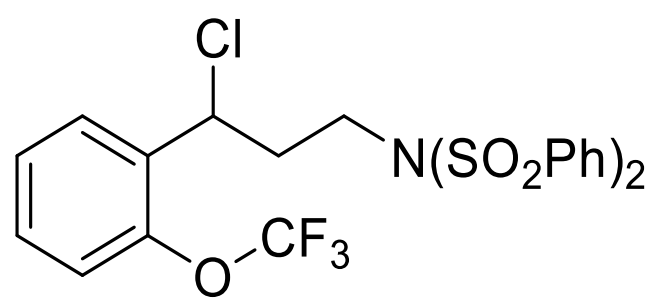

Following the general procedure E using *o*-**5k** (63 mg, 0.30 mmol, 1.0 equiv.), yielding N-(3-chloro-3-(2-(trifluoromethoxy)phenyl)propyl)-N-(phenylsulfonyl) benzenesulfonamide (*o*-**6k**) as a colorless wax (104 mg, 0.190 mmol, 65%).

R_f (Hex:AcOEt = 8:2) = 0.34.
^{1}H NMR (400 MHz, $CDCl_3$) δ 8.03 – 7.94 (m, 4H, Ar*H*), 7.67 (tt, *J* = 7.0, 1.4 Hz, 2H, Ar*H*), 7.56 (tt, *J* = 7.6, 1.2 Hz, 4H, Ar*H*), 7.39 (t, *J* = 7.9 Hz, 1H, Ar*H*), 7.29 – 7.23 (m, 1H, Ar*H*), 7.22 – 7.13 (m, 2H, Ar*H*), 4.83 (dd, *J* = 8.7, 5.4 Hz, 1H, ArC*H*), 3.93 (ddd, *J* = 14.9, 9.1, 5.6 Hz, 1H, NCH_2), 3.77 (ddd, *J* = 15.4, 9.3, 6.2 Hz, 1H, NCH_2), 2.50 – 2.36 (m, 2H, CH_2CH_2).
^{19}F NMR (376 MHz, $CDCl_3$) δ -57.7.
^{13}C NMR (101 MHz, $CDCl_3$) δ 149.5 (d, *J* = 1.9 Hz), 142.9, 139.6, 134.2, 130.4, 129.4, 128.3, 125.4, 121.1, 120.5 (q, *J* = 257.6 Hz), 119.7, 59.3, 46.7, 39.6.
IR (ν_{max}, cm^{-1}) 1449 (m), 1375 (m), 1253 (s), 1215 (s), 1157 (s), 1084 (m), 856 (m), 750 (s).
HRMS (nanochip-ESI/LTQ-Orbitrap) m/z: $[M + Na]^+$ Calcd for $C_{22}H_{19}ClF_3NNaO_5S_2^+$ 556.0237; Found 556.0257.

N-(3-Chloro-3-(3-(trifluoromethoxy)phenyl)propyl)-N-(phenylsulfonyl) benzenesulfonamide (*m*-**6k**)

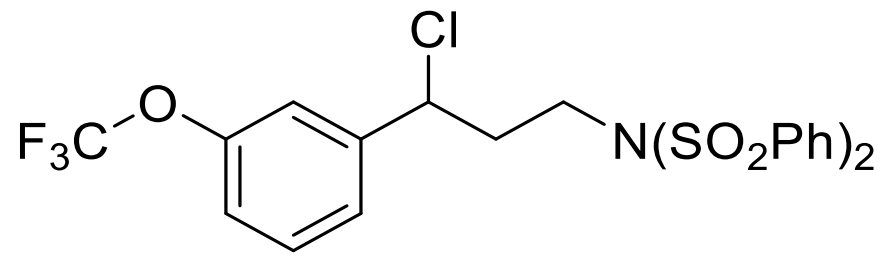

Following the general procedure E using *m*-**5k** (63 mg, 0.30 mmol, 1.0 equiv.), yielding N-(3-chloro-3-(3-(trifluoromethoxy)phenyl)propyl)-N-(phenylsulfonyl) benzenesulfonamide (*m*-**6k**) as a colorless wax (145 mg, 0.270 mmol, 91%).

R_f (Hex:AcOEt = 8:2) = 0.34.
^{1}H NMR (400 MHz, $CDCl_3$) δ 8.09 – 7.88 (m, 4H, Ar*H*), 7.81 – 7.61 (m, 2H, Ar*H*), 7.61 – 7.49 (m, 4H, Ar*H*), 7.39 (t, *J* = 7.9 Hz, 1H, Ar*H*), 7.25 (d, *J* = 7.5 Hz, 1H, Ar*H*), 7.23 – 7.06 (m, 2H, Ar*H*), 4.82 (dd, *J* = 8.7, 5.4 Hz, 1H, ArC*H*), 3.93 (ddd, *J* = 14.9, 9.2, 5.6 Hz, 1H, NCH_2), 3.77 (ddd, *J* = 15.4, 9.3, 6.1 Hz, 1H, NCH_2), 2.42 (dtd, *J* = 14.0, 8.5, 5.2 Hz, 2H, CH_2CH_2).
^{19}F NMR (376 MHz, $CDCl_3$) δ -57.7.
^{13}C NMR (101 MHz, $CDCl_3$) δ 149.5 (d, *J* = 1.9 Hz), 142.9, 139.6, 134.2, 130.4, 129.4, 128.3, 125.3, 121.1, 120.5 (q, *J* = 257.8 Hz), 119.7, 59.3, 46.7, 39.6.
IR ($ν_{max}$, cm^{-1}) 1450 (m), 1376 (m), 1260 (s), 1217 (s), 1168 (s), 1087 (m), 861 (m), 757 (s).
HRMS (ESI/QTOF) m/z: $[M + Na]^+$ Calcd for $C_{22}H_{19}ClF_3NNaO_5S_2^+$ 556.0237; Found 556.0253.

N-(3-Chloro-3-(4-(trifluoromethoxy)phenyl)propyl)-N-(phenylsulfonyl) benzenesulfonamide (*p*-**6k**)

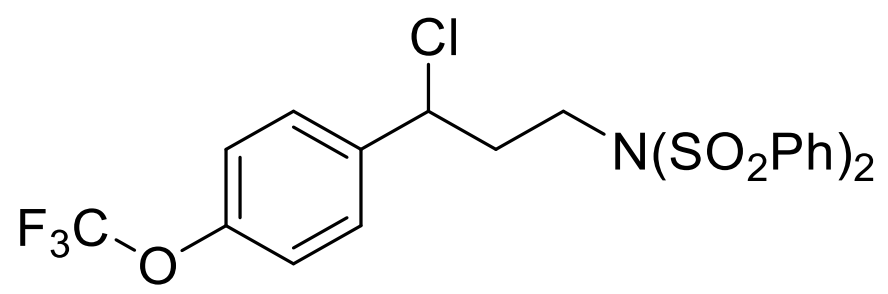

Following the general procedure E using *p*-**5k** (63 mg, 0.30 mmol, 1.0 equiv.), yielding N-(3-chloro-3-(4-(trifluoromethoxy)phenyl)propyl)-N-(phenylsulfonyl) benzenesulfonamide (*p*-**6k**) as a colorless wax (139 mg, 0.260 mmol, 87%).

R_f (Hex:AcOEt = 8:2) = 0.34.
^{1}H NMR (400 MHz, $CDCl_3$) δ 8.03 – 7.92 (m, 4H, Ar*H*), 7.77 – 7.65 (m, 2H, Ar*H*), 7.55 (dd, *J* = 8.6, 7.2 Hz, 4H, Ar*H*), 7.43 – 7.33 (m, 2H, Ar*H*), 7.21 (d, *J* = 8.3 Hz, 2H, Ar*H*), 4.91 – 4.78 (m, 1H, ArC*H*), 3.91 (ddd, *J* = 15.0, 8.7, 6.7 Hz, 1H, NCH_2), 3.73 (ddd, *J* = 15.4, 8.6, 6.6 Hz, 1H, NCH_2), 2.51 – 2.40 (m, 2H, CH_2CH_2).
^{19}F NMR (376 MHz, $CDCl_3$) δ -57.8.
^{13}C NMR (101 MHz, $CDCl_3$) δ 149.2 (d, *J* = 1.9 Hz), 139.4, 139.1, 134.1, 129.2, 128.5, 128.2, 121.2, 120.4 (q, *J* = 257.7 Hz), 59.2, 46.6, 39.5.
IR ($ν_{max}$, cm^{-1}) 1510 (w), 1449 (w), 1375 (m), 1256 (s), 1214 (s), 1163 (s), 1085 (m),

862 (m), 755 (s).
HRMS (nanochip-ESI/LTQ-Orbitrap) m/z: $[M + Na]^+$ Calcd for $C_{22}H_{19}ClF_3NNaO_5S_2^+$ 556.0237; Found 556.0261.

N-(3-Chloro-3-(2-chlorophenyl)propyl)-N-(phenylsulfonyl)benzenesulfonamide (*o*-**6l**)

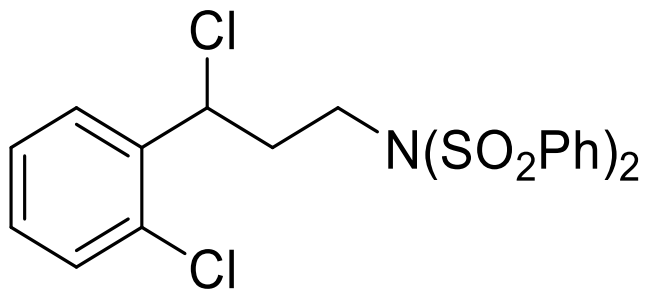

Following the general procedure E using *o*-**5l** (47 mg, 0.30 mmol, 1.0 equiv.), yielding N-(3-chloro-3-(2-chlorophenyl)propyl)-N-(phenylsulfonyl) benzenesulfonamide (*o*-**6l**) as a colorless wax (117 mg, 0.240 mmol, 81%).

R_f (Hex:AcOEt = 8:2) = 0.37.
^{1}H NMR (400 MHz, $CDCl_3$) δ 7.96 – 7.85 (m, 4H, A*rH*), 7.64 – 7.54 (m, 2H, A*rH*), 7.51 – 7.41 (m, 5H, A*rH*), 7.34 – 7.09 (m, 3H, A*rH*), 5.27 (dd, *J* = 8.7, 5.3 Hz, 1H, ArC*H*), 3.89 (ddd, *J* = 15.3, 9.9, 5.4 Hz, 1H, NCH_2), 3.71 (ddd, *J* = 15.5, 10.0, 5.8 Hz, 1H, NCH_2), 2.46 – 2.24 (m, 2H, CH_2CH_2).
^{13}C NMR (101 MHz, $CDCl_3$) δ 139.6, 137.8, 134.1, 132.6, 129.8, 129.3, 128.5, 128.3, 127.6, 56.3, 46.8, 38.6.
One carbon not resolved.
IR (v_{max}, cm^{-1}) 1476 (w), 1447 (m), 1373 (s), 1166 (s), 1085 (s), 1034 (m), 861 (m), 749 (s).
HRMS (ESI/QTOF) m/z: $[M + Na]^+$ Calcd for $C_{21}H_{19}Cl_2NNaO_4S_2^+$ 506.0025; Found 506.0033.

N-(3-Chloro-3-(3-chlorophenyl)propyl)-N-(phenylsulfonyl)benzenesulfonamide (*m*-**6l**)

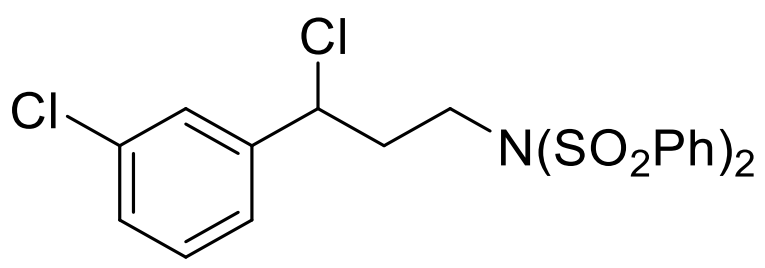

Following the general procedure E using *m*-**6l** (47 mg, 0.30 mmol, 1.0 equiv.), yielding N-(3-chloro-3-(3-chlorophenyl)propyl)-N-(phenylsulfonyl) benzenesulfonamide (*m*-**6j**) as a colorless wax (115 mg, 0.240 mmol, 79%).

R_f (Hex:AcOEt = 8:2) = 0.37.
^{1}H NMR (400 MHz, $CDCl_3$) δ 7.95 – 7.87 (m, 4H, A*rH*), 7.67 – 7.56 (m, 2H, A*rH*), 7.47 (dd, *J* = 8.5, 7.2 Hz, 4H, A*rH*), 7.25 – 7.16 (m, 3H, A*rH*), 7.11 (dt, *J* = 5.3, 2.6 Hz, 1H, A*rH*), 4.70 (dd, *J* = 8.0, 6.2 Hz, 1H, ArC*H*), 3.83 (ddd, *J* = 14.9, 8.5, 6.4 Hz, 1H, NCH_2), 3.66 (ddd, *J* = 15.3, 8.6, 6.7 Hz, 1H, NCH_2), 2.39 – 2.25 (m, 2H, CH_2CH_2).
^{13}C NMR (101 MHz, $CDCl_3$) δ 142.5, 139.5, 134.7, 134.2, 130.2, 129.4, 128.9, 128.3, 127.2, 125.2, 59.4, 46.7, 39.5.
IR (v_{max}, cm^{-1}) 1448 (m), 1373 (s), 1165 (s), 1083 (s), 857 (m), 746 (s).

HRMS (ESI/QTOF) m/z: $[M + Na]^+$ Calcd for $C_{21}H_{19}Cl_2NNaO_4S_2^+$ 506.0025; Found 506.0006.

N-(3-Chloro-3-(4-chlorophenyl)propyl)-N-(phenylsulfonyl)benzenesulfonamide (*p*-**6l**)

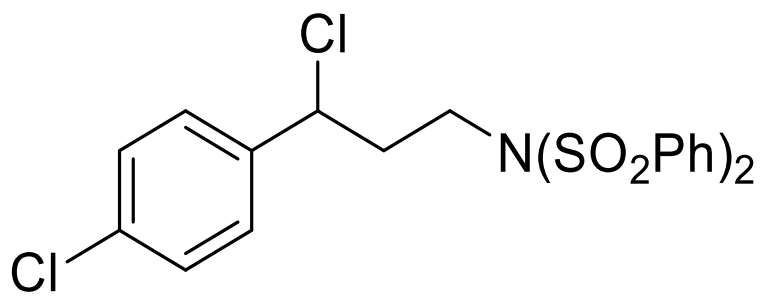

Following the general procedure E using *p*-**5l** (47 mg, 0.30 mmol, 1.0 equiv.), yielding N-(3-chloro-3-(4-chlorophenyl)propyl)-N-(phenylsulfonyl) benzenesulfonamide (*p*-**6l**) as a colorless wax (132 mg, 0.270 mmol, 91%).

$\mathbf{R_f}$ (Hex:AcOEt = 8:2) = 0.37.
^{1}H NMR (400 MHz, $CDCl_3$) δ 8.03 – 7.72 (m, 4H, Ar*H*), 7.67 – 7.51 (m, 2H, Ar*H*), 7.44 (dd, *J* = 8.5, 7.2 Hz, 4H, Ar*H*), 7.33 – 7.18 (m, 2H, Ar*H*), 7.18 – 7.07 (m, 2H, Ar*H*), 4.71 (t, *J* = 7.1 Hz, 1H, ArC*H*), 3.92 – 3.71 (m, 1H, NCH_2), 3.63 (dt, *J* = 15.4, 7.5 Hz, 1H, NCH_2), 2.34 (q, *J* = 7.4 Hz, 2H, CH_2CH_2).
^{13}C NMR (101 MHz, $CDCl_3$) δ 139.5, 139.0, 134.5, 134.2, 129.3, 129.1, 128.4, 128.2, 59.5, 46.7, 39.5.
IR (ν_{max}, cm^{-1}) 1493 (w), 1449 (m), 1374 (s), 1167 (s), 1087 (s), 862 (m), 754 (s).
HRMS (ESI/QTOF) m/z: $[M + Na]^+$ Calcd for $C_{21}H_{19}Cl_2NNaO_4S_2^+$ 506.0025; Found 506.0006.

N-(3-Chloro-3-(2-fluorophenyl)propyl)-N-(phenylsulfonyl)benzenesulfonamide (*o*-**6m**)

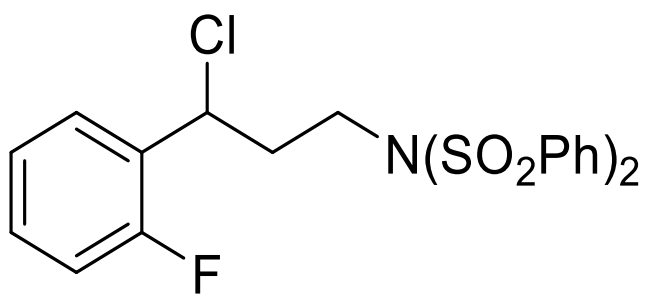

Following the general procedure using *o*-**5m** (42 mg, 0.30 mmol, 1.0 equiv.), yielding N-(3-chloro-3-(2-fluorophenyl)propyl)-N-(phenylsulfonyl) benzenesulfonamide (*o*-**6m**) as a colorless wax (120 mg, 0.260 mmol, 85%).

$\mathbf{R_f}$ (Hex:AcOEt = 8:2) = 0.37.
^{1}H NMR (400 MHz, $CDCl_3$) δ 7.90 – 7.82 (m, 4H, Ar*H*), 7.60 – 7.49 (m, 2H, Ar*H*), 7.42 (dd, *J* = 8.5, 7.2 Hz, 4H, Ar*H*), 7.30 (td, *J* = 7.6, 1.8 Hz, 1H, Ar*H*), 7.23 – 7.08 (m, 1H, Ar*H*), 7.04 (td, *J* = 7.5, 1.2 Hz, 1H, Ar*H*), 6.92 (ddd, *J* = 9.6, 8.3, 1.2 Hz, 1H, Ar*H*), 5.04 (dd, *J* = 8.3, 6.0 Hz, 1H, ArC*H*), 3.83 (ddd, *J* = 15.2, 9.1, 6.0 Hz, 1H, NCH_2), 3.64 (ddd, *J* = 15.5, 9.2, 6.5 Hz, 1H, NCH_2), 2.35 (dtd, *J* = 8.7, 5.7, 3.1 Hz, 2H, CH_2CH_2).
^{19}F NMR (376 MHz, $CDCl_3$) δ -117.6.
^{13}C NMR (101 MHz, $CDCl_3$) δ 159.8 (d, *J* = 248.5 Hz), 139.9, 134.5, 130.7 (d, *J* = 8.4 Hz), 129.6, 128.8 (d, *J* = 6.1 Hz), 128.7 (d, *J* = 7.0 Hz), 128.6, 125.1 (d, *J* = 3.7 Hz), 116.2 (d, *J* = 21.8 Hz), 53.4 (d, *J* = 4.0 Hz), 47.1, 38.8.
IR (ν_{max}, cm^{-1}) 1492 (m), 1450 (m), 1371 (s), 1167 (s), 1086 (m), 1030 (w), 863 (m),

754 (s), 735 (s).
HRMS (nanochip-ESI/LTQ-Orbitrap) m/z: $[M + Na]^+$ Calcd for $C_{21}H_{19}ClFNNaO_4S_2^+$ 490.0320; Found 490.0315.

N-(3-Chloro-3-(3-fluorophenyl)propyl)-N-(phenylsulfonyl)benzenesulfonamide (*m*-**6m**)

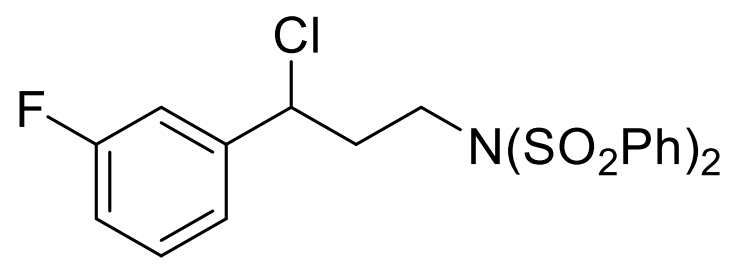

Following the general procedure E using *m*-**5m** (42 mg, 0.30 mmol, 1.0 equiv.), yielding N-(3-chloro-3-(3-fluorophenyl)propyl)-N-(phenylsulfonyl) benzenesulfonamide (*m*-**6m**) as a colorless wax (105 mg, 0.220 mmol, 75%).

R_f (Hex:AcOEt = 8:2) = 0.37.
^{1}H NMR (400 MHz, $CDCl_3$) δ 8.08 – 7.92 (m, 4H, A*rH*), 7.76 – 7.61 (m, 2H, A*rH*), 7.55 (t, *J* = 7.8 Hz, 4H, A*rH*), 7.32 (ddd, *J* = 9.0, 7.8, 5.8 Hz, 1H, A*rH*), 7.18 – 6.93 (m, 3H, A*rH*), 4.81 (dd, *J* = 8.2, 5.9 Hz, 1H, ArC*H*), 3.92 (ddd, *J* = 15.0, 8.8, 6.0 Hz, 1H, NCH_2), 3.76 (ddd, *J* = 15.4, 9.0, 6.5 Hz, 1H, NCH_2), 2.42 (dtd, *J* = 10.2, 5.7, 2.9 Hz, 2H, CH_2CH_2).
^{19}F NMR (376 MHz, $CDCl_3$) δ -111.9 (td, *J* = 9.0, 5.7 Hz).
^{13}C NMR (101 MHz, $CDCl_3$) δ 162.9 (d, *J* = 247.2 Hz), 142.9 (d, *J* = 7.2 Hz), 139.5, 134.2, 130.5 (d, *J* = 8.2 Hz), 129.3, 128.3, 122.6 (d, *J* = 3.0 Hz), 115.7 (d, *J* = 21.1 Hz), 114.1 (d, *J* = 22.5 Hz), 59.4 (d, *J* = 1.9 Hz), 46.7, 39.5.
IR (ν_{max}, cm^{-1}) 3068 (w), 3061 (w), 1449 (m), 1366 (m), 1265 (m), 1167 (s), 1085 (m), 857 (m), 735 (s).
HRMS (nanochip-ESI/LTQ-Orbitrap) m/z: $[M + Na]^+$ Calcd for $C_{21}H_{19}ClFNNaO_4S_2^+$ 490.0320; Found 490.0319.

N-(3-Chloro-3-(4-fluorophenyl)propyl)-N-(phenylsulfonyl)benzenesulfonamide (*p*-**6m**)

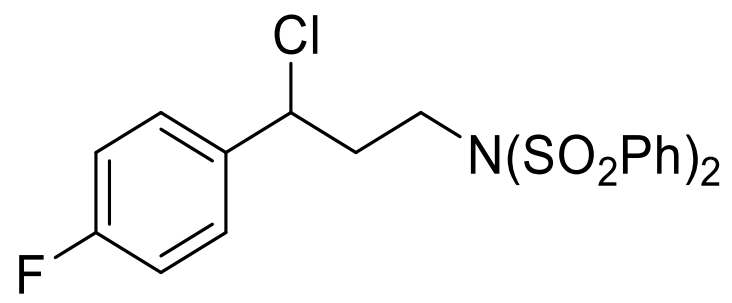

Following the general procedure E using *p*-**5m** (42 mg, 0.30 mmol, 1.0 equiv.), yielding N-(3-chloro-3-(4-fluorophenyl)propyl)-N-(phenylsulfonyl) benzenesulfonamide (*p*-**6m** as a colorless wax (125 mg, 0.270 mmol, 89%).

R_f (Hex:AcOEt = 8:2) = 0.37.
^{1}H NMR (400 MHz, $CDCl_3$) δ 8.03 – 7.88 (m, 4H, A*rH*), 7.74 – 7.61 (m, 2H, A*rH*), 7.55 (dd, *J* = 8.5, 7.2 Hz, 4H, A*rH*), 7.29 (qd, *J* = 6.0, 2.7 Hz, 2H, A*rH*), 7.04 (dd, *J* = 9.5, 7.7 Hz, 2H, A*rH*), 4.82 (t, *J* = 7.1 Hz, 1H, ArC*H*), 4.02 – 3.84 (m, 1H, NCH_2), 3.73 (dt, *J* = 15.4, 7.7 Hz, 1H, NCH_2), 2.44 (q, *J* = 7.5 Hz, 2H, CH_2CH_2).
^{19}F NMR (376 MHz, $CDCl_3$) δ -112.7.

^{13}C NMR (101 MHz, $CDCl_3$) δ 162.7 (d, *J* = 248.0 Hz), 139.5, 136.4 (d, *J* = 3.2 Hz), 134.2, 129.3, 128.8 (d, *J* = 8.4 Hz), 128.3, 115.8 (d, *J* = 21.7 Hz), 59.6, 46.8, 39.7.
IR (ν_{max}, cm^{-1}) 1604 (w), 1511 (m), 1448 (m), 1373 (s), 1227 (m), 1165 (s), 1084 (m), 1024 (w), 910 (w), 859 (s), 754 (s), 734 (s), 724 (s).
HRMS (nanochip-ESI/LTQ-Orbitrap) m/z: $[M + Na]^+$ Calcd for $C_{21}H_{19}ClFNNaO_4S_2^+$ 490.0320; Found 490.0319.

N-(3-Chloro-3-(naphthalen-1-yl)propyl)-N-(phenylsulfonyl) benzenesulfonamide (**6n**)

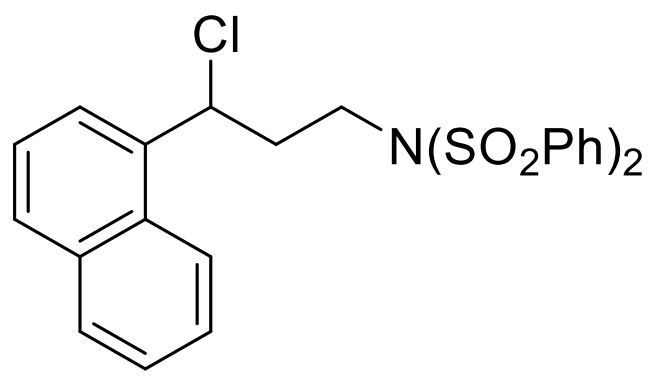

Following the general procedure using **5n** (52 mg, 0.30 mmol, 1.0 equiv.), yielding N-(3-chloro-3-(naphthalen-1-yl)propyl)-N-(phenylsulfonyl) benzenesulfonamide (**6n**) as a colorless wax (135 mg, 0.270 mmol, 90%).

R$_f$ (Hex:AcOEt = 8:2) = 0.38.
^{1}H NMR (400 MHz, DMSO-*d$_6$*) δ 8.28 (ddd, *J* = 13.9, 6.5, 3.3 Hz, 2H, A*rH*), 7.94 – 7.86 (m, 4H, A*rH*), 7.84 – 7.75 (m, 5H, A*rH*), 7.72 – 7.62 (m, 5H, A*rH*), 6.09 (dd, *J* = 9.0, 4.6 Hz, 1H, ArC*H*), 4.00 (qdd, *J* = 15.5, 10.2, 5.3 Hz, 2H, NC*H$_2$*), 2.60 (dtd, *J* = 27.1, 9.9, 5.2 Hz, 2H, CH_2CH_2).
^{13}C NMR (101 MHz, DMSO-*d$_6$*) δ 138.5, 135.5, 134.7, 131.7, 131.0, 130.1, 129.7, 127.8, 127.7, 126.1, 124.9, 124.6, 123.9, 54.9, 46.5, 37.9.
One carbon not resolved.
IR (ν_{max}, cm^{-1}) 1490 (m), 1449 (m), 1369 (s), 1176 (s), 1168 (s), 1163 (s), 1157 (m), 1086 (s), 1029 (m), 877 (s), 871 (s), 801 (m), 772 (s), 762 (s), 753 (s), 745 (s), 741 (s), 737 (s), 732 (s), 723 (s), 717 (s).
HRMS (Sicrit plasma/LTQ-Orbitrap) m/z: $[M + H_{-1}]^+$ Calcd for $C_{25}H_{21}ClNO_4S_2^+$ 498.0595; Found 498.0595.

N-(3-Chloro-3-(naphthalen-2-yl)propyl)-N-(phenylsulfonyl)benzenesulfonamide (**6o**)

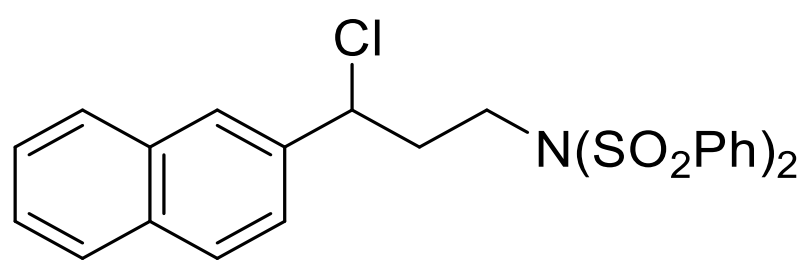

Following the general procedure E using **5o** (52 mg, 0.30 mmol, 1.0 equiv.), yielding N-(3-chloro-3-(naphthalen-2-yl)propyl)-N-(phenylsulfonyl) benzenesulfonamide (**6o** as a colorless wax (57 mg, 0.11 mmol, 38%).

R$_f$ (Hex:AcOEt = 8:2) = 0.38.
^{1}H NMR (400 MHz, $CDCl_3$) δ 8.40 – 8.22 (m, 1H, A*rH*), 8.05 – 7.99 (m, 1H, A*rH*), 7.97 – 7.88 (m, 4H, A*rH*), 7.68 – 7.42 (m, 10H, A*rH*), 7.22 (s, 1H, A*rH*), 5.59 (dd, *J* = 8.8, 5.1 Hz, 1H, ArC*H*), 3.99 (ddd, *J* = 14.9, 9.1, 5.4 Hz, 1H, NC*H$_2$*), 3.85 (ddd, *J* =

15.3, 9.2, 6.1 Hz, 1H, NCH_2), 2.70 – 2.55 (m, 2H, CH_2CH_2).
^{13}C NMR (101 MHz, $CDCl_3$) δ 139.6, 135.0, 134.2, 133.4, 131.5, 131.2, 129.4, 128.3, 127.7, 127.3, 125.8, 125.7, 124.6, 123.3, 56.1, 47.1, 38.4.
IR ($ν_{max}$, cm^{-1}) 1490 (w), 1449 (m), 1367 (m), 1312 (m), 1291 (m), 1181 (m), 1172 (s), 1163 (s), 1087 (s), 1080 (m), 1031 (m), 867 (m), 859 (m), 852 (s), 849 (s), 843 (s), 838 (m), 812 (m), 761 (s), 752 (s), 745 (s), 741 (s), 735 (s), 731 (s), 725 (s), 716 (s).
HRMS (Sicrit plasma/LTQ-Orbitrap) m/z: $[M + H_{-1}]^+$ Calcd for $C_{25}H_{21}ClNO_4S_2^+$ 498.0595; Found 498.0596.

Methyl 4-(1-chloro-3-(N-(phenylsulfonyl)phenylsulfonamido)propyl)-1-naphthoate (**6p**)

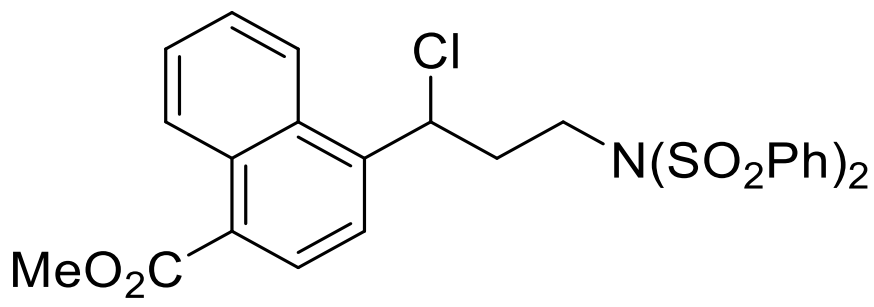

Following the general procedure E using **5p** (70 mg, 0.30 mmol, 1.0 equiv.), yielding methyl 4-(1-chloro-3-(N-(phenylsulfonyl)phenylsulfonamido)propyl)-1-naphthoate (**6p**) as a colorless wax (82 mg, 0.15 mmol, 49%).

R_f (Hex:AcOEt = 8:2) = 0.31.
^{1}H NMR (400 MHz, $CDCl_3$) δ 9.01 – 8.87 (m, 1H, Ar*H*), 8.13 (d, *J* = 7.7 Hz, 1H, Ar*H*), 8.09 – 8.03 (m, 1H, Ar*H*), 8.02 – 7.93 (m, 4H, Ar*H*), 7.72 – 7.57 (m, 5H, Ar*H*), 7.50 (dd, *J* = 8.5, 7.3 Hz, 4H, Ar*H*), 5.70 (dd, *J* = 9.2, 4.7 Hz, 1H, ArC*H*), 4.02 (m, 4H, COOCH_3, NCH_2), 3.92 (ddd, *J* = 15.3, 9.6, 6.0 Hz, 1H, NCH_2), 2.79 – 2.50 (m, 2H, CH_2CH_2).
^{13}C NMR (101 MHz, $CDCl_3$) δ 167.8, 140.5, 139.5, 134.2, 131.7, 130.6, 129.4, 129.3, 128.5, 128.3, 127.8, 127.2, 126.9, 123.4, 123.0, 77.4, 52.5, 47.0, 38.5.
IR ($ν_{max}$, cm^{-1}) 1717 (m), 1448 (m), 1374 (s), 1167 (s), 1086 (m), 863 (m), 775 (s), 754 (s), 737 (s).
HRMS (ESI/QTOF) m/z: $[M + Na]^+$ Calcd for $C_{27}H_{24}ClNNaO_6S_2^+$ 580.0626; Found 580.0629.

N-(3-Chloro-3-(3-oxo-2,3-dihydro-1H-inden-4-yl)propyl)-N-(phenylsulfonyl) benzenesulfonamide (**6q**)

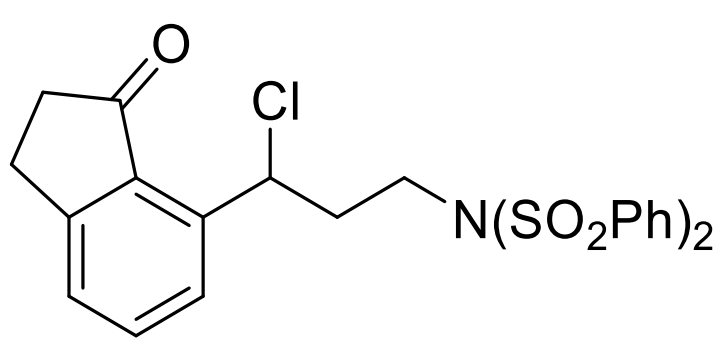

Following the general procedure E using **5q** (53 mg, 0.30 mmol, 1.0 equiv.), yielding N-(3-chloro-3-(3-oxo-2,3-dihydro-1H-inden-4-yl)propyl)-N-(phenylsulfonyl) benzenesulfonamide (**6q**) as a colorless wax (99 mg, 0.20 mmol, 65%).

R$_f$ (Hex:AcOEt = 8:2) = 0.32.
^{1}H NMR (400 MHz, $CDCl_3$) δ 8.03 – 7.93 (m, 4H, Ar*H*), 7.75 (dd, *J* = 7.6, 1.1 Hz, 1H, Ar*H*), 7.73 – 7.61 (m, 3H, Ar*H*), 7.55 (dd, *J* = 8.4, 7.2 Hz, 4H, Ar*H*), 7.44 (t, *J* = 7.6 Hz, 1H, Ar*H*), 5.05 (dd, *J* = 8.5, 5.8 Hz, 1H, ArC*H*), 3.98 (ddd, *J* = 14.8, 8.2, 6.0 Hz, 1H, NC*H$_2$*), 3.81 (ddd, *J* = 15.3, 8.7, 6.5 Hz, 1H, NC*H$_2$*), 3.15 (dt, *J* = 17.3, 5.5 Hz, 1H, ArC*H$_2$*), 3.10 – 2.99 (m, 1H, ArC*H$_2$*), 2.71 (ddd, *J* = 6.7, 4.9, 1.5 Hz, 2H, COC*H$_2$*), 2.59 – 2.46 (m, 2H, CH_2C*H$_2$*).
^{13}C NMR (101 MHz, $CDCl_3$) δ 206.3, 152.6, 139.5, 138.3, 137.9, 134.3, 132.2, 129.4, 128.4, 128.4, 124.3, 56.1, 46.9, 38.2, 36.1, 24.2.
IR (ν_{max}, cm^{-1}) 1712 (s), 1447 (m), 1372 (s), 1273 (m), 1166 (s), 1085 (m), 858 (m), 753 (s), 722 (s).
HRMS (ESI/QTOF) m/z: $[M + H]^+$ Calcd for $C_{24}H_{23}ClNO_5S_2^+$ 504.0701; Found 504.0699.

N-(4-Chloro-4-phenylbutan-2-yl)-N-(phenylsulfonyl)benzenesulfonamide (**6r**)

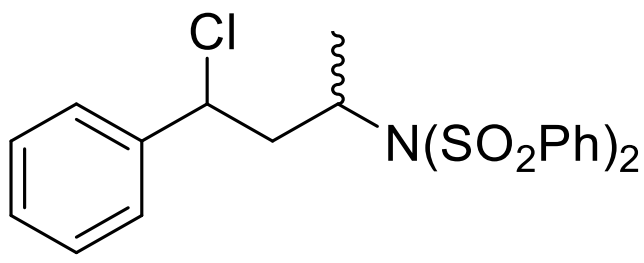

Following the general procedure E using **5r** (41 mg, 0.30 mmol, 1.0 equiv., d.r. 6:4), yielding N-(4-chloro-4-phenylbutan-2-yl)-N-(phenylsulfonyl)benzenesulfonamide (**6r**) as a colorless wax (107 mg, 0.230 mmol, 77% mixture of inseparable diastereoisomers d.r. 7:3 from CH_2C*H$_2$*).

R$_f$ (Hex:AcOEt = 8:2) = 0.38.
^{1}H NMR (400 MHz, Acetone, signals of the minor diastereoisomer are indicated in *italic*) δ 8.12 – 7.61 (m, 10H, Ar*H*), 7.47 – 7.29 (m, 5H, Ar*H*), *5.04 (dd, J = 11.0, 4.0 Hz, 1H*, ArC*H)*, 4.90 (dd, *J* = 8.0, 6.4 Hz, 1H, ArC*H*), *4.57 (dqd, J = 10.5, 6.9, 3.6 Hz, 1H*, CH_3C*H*N*)*, 4.22 (d, *J* = 6.9 Hz, 1H, CH_3C*H*N), *2.86 (s, 1H*, CH_2C*H$_2$)*, 2.81 – 2.75 (m, 2H, CH_2C*H$_2$*), *2.29 (ddd, J = 14.4, 10.9, 3.6 Hz, 1H*, CH_2C*H$_2$)*, *1.53 (d, J = 6.9 Hz, 3H*, CHC*H$_3$)*, 1.41 (d, *J* = 7.0 Hz, 3H, CHC*H$_3$*).
^{13}C NMR (101 MHz, Acetone, signals of the minor diastereoisomer are indicated in *italic*) δ *142.1*, 141.3, *135.1*, *135.1*, 130.2, 130.2, 129.7, *129.5*, *129.1*, 129.0, 127.9, *127.7*, *61.6*, 61.1, *58.4*, 58.3, 45.9, *45.8*, 20.0, *18.4*.
Four carbons not resolved.
IR (ν_{max}, cm^{-1}) 1449 (m), 1366 (s), 1165 (s), 1085 (m), 1069 (m), 854 (m), 754 (m), 733 (s), 721 (s).
HRMS (ESI/QTOF) m/z: $[M + Na]^+$ Calcd for $C_{22}H_{22}ClNNaO_4S_2^+$ 486.0571; Found 486.0556.

N-(3-Chloro-3,3-diphenylpropyl)-N-(phenylsulfonyl)benzenesulfonamide (**6s**)

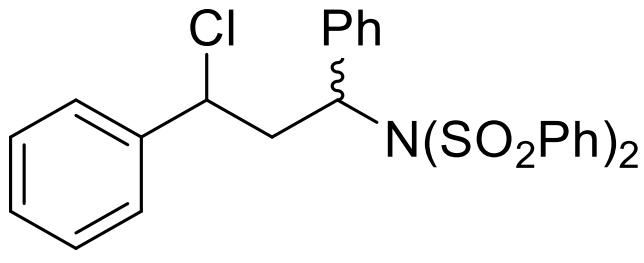

Following the general procedure E using **5s** (61 mg, 0.30 mmol, 1.0 equiv.), yielding N-(3-chloro-3ii,3-diphenylpropyl)-N-(phenylsulfonyl) benzenesulfonamide (**6s**) as a colorless wax (76 mg, 0.14 mmol, 48% mixture of inseparable diastereoisomers d.r. 3:2).

R_f (Hex:AcOEt = 8:2) = 0.34.
^{1}H NMR (400 MHz, $CDCl_3$, signals of the minor diastereoisomer are indicated in *italic*) δ 7.72 – 7.27 (m, 18H, Ar*H*), 7.19 – 7.05 (m, 1H, Ar*H*), 7.03 – 6.92 (m, 1H, Ar*H*), *6.03 (dd, J = 12.5, 2.7 Hz, 1H*, ArC*H)*, 5.24 (dd, *J* = 10.3, 5.0 Hz, 1H, ArC*H*), *4.67 (dd, J = 12.1, 2.0 Hz, 1H*, CH_2C*H*N*)*, 4.58 (dd, *J* = 9.1, 5.7 Hz, 1H, CH_2C*H*N), 3.61 (ddd, *J* = 14.1, 10.3, 5.7 Hz, 1H, CH_2C*H₂*), *3.52 (ddd, J = 14.7, 12.4, 2.1 Hz, 1H*, CH_2C*H₂)*, 2.58 (ddd, *J* = 14.2, 9.2, 5.1 Hz, 1H, CH_2C*H₂*), *2.10 (ddd, J = 14.9, 12.1, 2.7 Hz, 1H*, CH_2C*H₂)*.
^{13}C NMR (101 MHz, $CDCl_3$, signals of the minor diastereoisomer are indicated in *italic*) δ *141.0*, *140.4*, 139.9, 134.7, *134.0*, 133.7, *130.2*, 130.1, *128.9*, 128.9, 128.9, *128.8*, 128.7, *128.4*, 127.2, *126.7*, *62.5*, 62.1, *61.0*, 59.7, 43.4, *41.8*.
Eight carbons not resolved.
IR (ν_{max}, cm^{-1}) 1451 (m), 1373 (s), 1167 (s), 1086 (m), 1001 (m), 969 (m), 884 (m), 773 (m), 721 (s).
HRMS (nanochip-ESI/LTQ-Orbitrap) m/z: $[M + Na]^+$ Calcd for $C_{27}H_{24}ClNNaO_4S_2^+$ 548.0727; Found 548.0722.

S10. 1,3-Aminoarylation of cyclopropanes

General procedure F

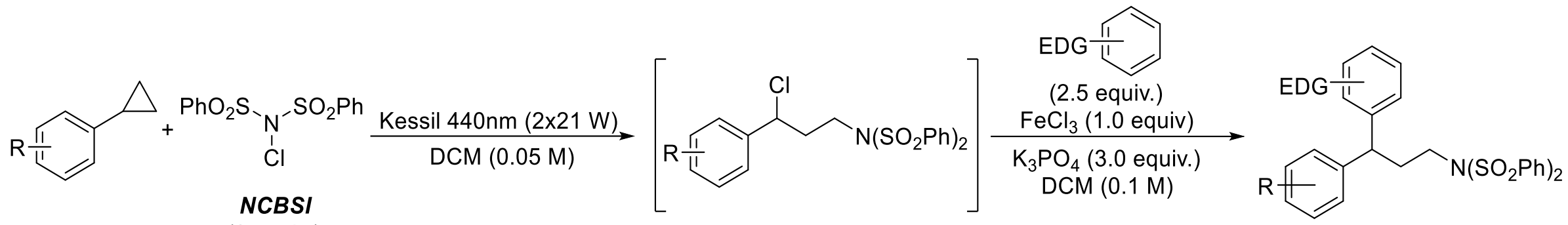

In a 15*100 mm borosilicate glass tube, NCBSI (203 mg, 0.600 mmol, 2.0 equiv.) was added. The tube was then closed with a rubber septum and sealed off with parafilm. Three cycles of evacuate-refill with nitrogen were performed to remove O_2. DCM (6 mL, 0.05 M) was added and the solution was stirred under irradiation of Kessil lamps (440 nm, 2x21 W) and after few seconds the aryl cyclopropane (1.0 equiv.) was added. It is important that the addition of starting material happens few seconds after the irradiation started to ensure reproducibility. The reaction was stirred under irradiation for 8-12 h, then the volume was reduced to half with a nitrogen flush. The resulting solution was added to an oven dry 15*100 mm norosilicate glass tube containing $FeCl_3$ (49 mg, 0.30 mmol, 1.0 equiv.), K_3PO_4 (191 mg, 0.900 mmol, 3.0 equiv.) and arene (2 equiv.). The mixture is stirred at room temperature overnight, then the solvent was removed under reduced pressure and the crude was purified by chromatography on Biotage (Büchi flashpure cartridge 25 g, Hexane:AcOEt from 10 to 40%) and by reverse phase chromatography on Büchi (BGB Aquarius C18AQ 40g, 100Å, 15 μm, H_2O (0.1% TFA):MeCN(0.1% TFA) from 0 to 100%) to obtain the products.

General procedure G

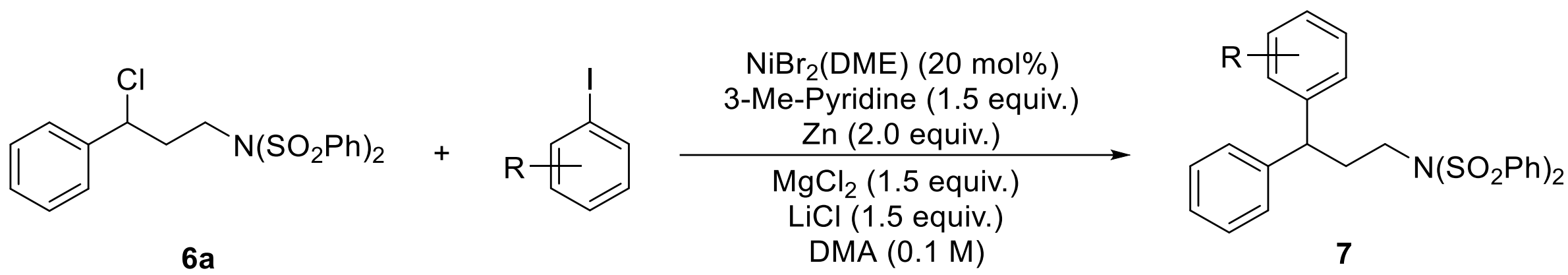

6a **7**

To an oven dried 15*100 mm borosilicate glass tube **6a** (135 mg, 0.300 mmol, 1.0 equiv.) was added, then it was introduced inside a glove-box. The flask was loaded with $NiBr_2(DME)$ (18 mg, 0.060 mmol , 20 mol%), Zn (39 mg, 0.60 mmol, 2.0 equiv.), $MgCl_2$ (43 mg, 0.45 mmol, 1.5 equiv.) and LiCl (19 mg, 0.45 mmol, 1.0 equiv.). The tube was then closed with a rubber septum taken out of the glove-box and sealed off with parafilm. DMA (3 mL, 0.1M) and 3-methylpyridine (42 mg, 0.45 mmol, 1.5 equiv.) were added and the mixture was stirred at room temperature overnight. The mixture was diluted with 10 mL Et_2O and washed twice with 10 mL LiCl 5%. The solvents were removed under reduced pressure and the crude was purified by chromatography on Biotage (Büchi flashpure cartridge 25 g, Hexane:AcOEt from 10 to 40%) and by reverse phase chromatography on Büchi (BGB Aquarius C18AQ 40g, 100Å, 15 μm, H_2O (0.1% TFA):MeCN(0.1% TFA) from 0 to 100%) to obtain the products.

General procedure H

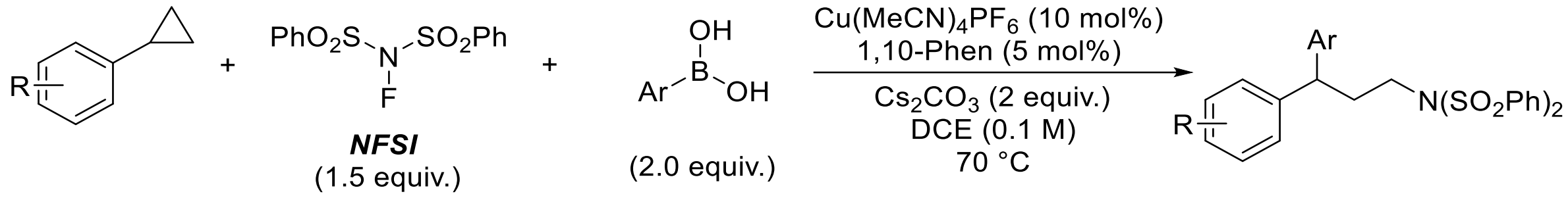

To an oven dried 5 mL microwave vial, NFSI (145 mg, 0.450 mmol, 1.5 equiv.), the aryl boronic acid (0.6 mmol, 2.0 equiv.), Cs_2CO_3 (198 mg, 0.600 mmol, 2.0 equiv.), $Cu(MeCN)_4PF_6$ (11 mg, 0.030 mmol, 10 mol%), and 1,10-phenanthroline (1.3 mg, 0.0050 mmol, 5 mol%) were added. The tube was then closed with a rubber septum and sealed off with parafilm. Three cycles of evacuate-refill with nitrogen were performed to remove O_2. Then arylcyclopropane **5** (0.3 mmol, 1.0 equiv.) was added together with DCE (3 mL, 0.1 M) and the mixture was stirred at 70 °C for 5-10 minutes. The consumption of the oxidant is indicated by a change of color from light blue to brown. The solvents were removed under reduced pressure and the crude was purified by chromatography on Biotage (Büchi flashpure cartridge 25 g, Hexane:AcOEt from 10 to 40%) and by reverse phase chromatography on Büchi (BGB Aquarius C18AQ 40g, 100Å, 15 µm, H_2O (0.1% TFA):MeCN(0.1% TFA) from 0 to 100%) to obtain the products.

N-(3-([1,1'-Biphenyl]-2-yl)-3-phenylpropyl)-N-(phenylsulfonyl)benzenesulfonamide (*o*-**7a**)

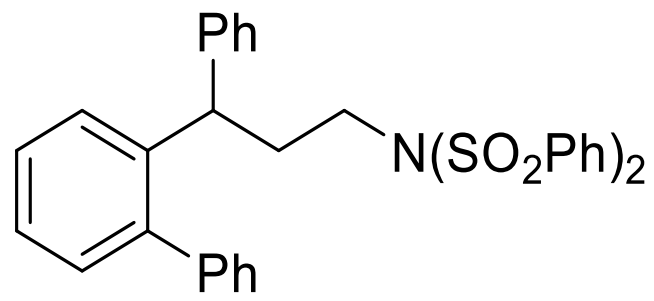

Following the general procedure H using *o*-**5b** (61 mg, 0.30 mmol, 1.0 equiv.) and phenylboronic acid (73 mg, 0.60 mmol, 2.0 equiv.), yielding N-(3-([1,1'-biphenyl]-2-yl)-3-phenylpropyl)-N-(phenylsulfonyl)benzenesulfonamide (*o*-**7a**) as a colorless wax (114 mg, 0.200 mmol, 67%).

$\mathbf{R_f}$ (Hex:AcOEt = 8:2) = 0.48.
^{1}H NMR (400 MHz, $CDCl_3$) δ 7.81 – 7.71 (m, 4H, Ar*H*), 7.60 – 7.49 (m, 2H, Ar*H*), 7.41 (t, *J* = 7.9 Hz, 4H, Ar*H*), 7.35 – 7.26 (m, 5H, Ar*H*), 7.23 – 7.01 (m, 8H, Ar*H*), 6.95 (d, *J* = 7.4 Hz, 2H, Ar*H*), 3.93 (t, *J* = 7.8 Hz, 1H, ArC*H*), 3.33 (ddd, *J* = 10.6, 5.9, 1.9 Hz, 2H, NC*H₂*), 2.36 (q, *J* = 8.0 Hz, 2H, CH_2CH_2).
^{13}C NMR (101 MHz, $CDCl_3$) δ 143.5, 142.4, 141.5, 140.7, 139.6, 133.9, 130.3, 129.5, 129.2, 128.6, 128.3, 128.0, 128.0, 127.4, 127.2, 126.4, 126.3, 47.9, 44.3, 36.4.
One carbon not resolved.
IR (ν_{max}, cm^{-1}) 1449 (m), 1374 (s), 1169 (s), 1086 (m), 871 (m), 802 (m), 742 (s)
HRMS (ESI/QTOF) m/z: $[M + Na]^+$ Calcd for $C_{33}H_{29}NNaO_4S_2^+$ 590.1430; Found 590.1433.

N-(3-([1,1'-Biphenyl]-3-yl)-3-phenylpropyl)-N-(phenylsulfonyl)benzenesulfonamide (*m*-**7a**)

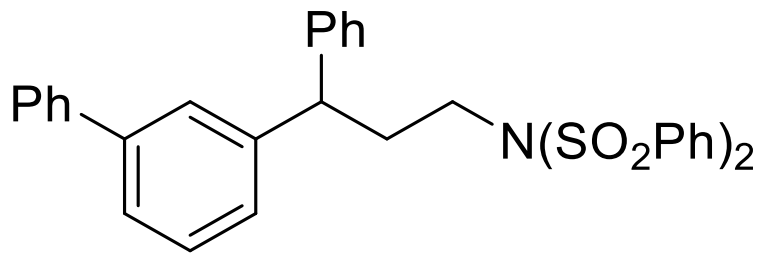

Following the general procedure H using *m*-**5b** (61 mg, 0.30 mmol, 1.0 equiv.) and phenylboronic acid (73 mg, 0.60 mmol, 2.0 equiv.), yielding N-(3-([1,1'-biphenyl]-3-yl)-3-phenylpropyl)-N-(phenylsulfonyl)benzenesulfonamide (*m*-**7a**) as a colorless wax (88 mg, 0.16 mmol, 52%).

R_f (Hex:AcOEt = 8:2) = 0.48.
^{1}H NMR (400 MHz, $CDCl_3$) δ 7.80 (dt, *J* = 7.3, 1.3 Hz, 4H, A*rH*), 7.56 – 7.47 (m, 4H, A*rH*), 7.43 – 7.33 (m, 8H, A*rH*), 7.32 – 7.09 (m, 8H, A*rH*), 3.87 (t, *J* = 8.0 Hz, 1H, ArC*H*), 3.61 – 3.45 (m, 2H, NCH_2), 2.54 – 2.40 (m, 2H, CH_2CH_2).
^{13}C NMR (101 MHz, $CDCl_3$) δ 143.9, 143.4, 141.7, 141.2, 139.7, 133.9, 129.2, 129.2, 128.9, 128.8, 128.3, 127.8, 127.5, 127.3, 126.9, 126.8, 126.7, 125.6, 49.1, 48.1, 35.5.
IR (v_{max}, cm^{-1}) 1450 (m), 1372 (s), 1166 (s), 1083 (m), 881 (m), 739 (s).
HRMS (ESI/QTOF) m/z: $[M + Na]^+$ Calcd for $C_{33}H_{29}NNaO_4S_2^+$ 590.1430; Found 590.1431.

N-(3-([1,1'-Biphenyl]-4-yl)-3-phenylpropyl)-N-(phenylsulfonyl)benzenesulfonamide (*p*-**7a**)

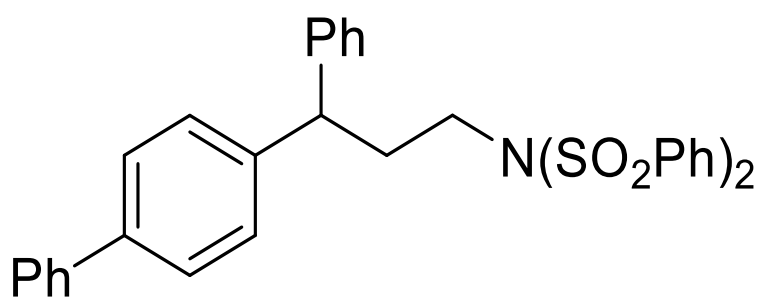

Following the general procedure F using cyclopropylbenzene **5a** (39 µL, 0.30 mmol, 1.0 equiv.) and biphenyl (116 mg, 0.750 mmol, 2.5 equiv.), yielding N-(3-([1,1'-biphenyl]-4-yl)-3-phenylpropyl)-N-(phenylsulfonyl)benzenesulfonamide (*p*-**7a**) as a colorless wax (99 mg, 0.17 mmol, 86%).

Following the general procedure H using *p*-**5b** (61 mg, 0.30 mmol, 1.0 equiv.) and phenylboronic acid (73 mg, 0.60 mmol, 2.0 equiv.), yielding N-(3-([1,1'-biphenyl]-4-yl)-3-phenylpropyl)-N-(phenylsulfonyl)benzenesulfonamide (*p*-**7a**) as a colorless wax (67 mg, 0.12 mmol, 39%).

R_f (Hex:AcOEt = 8:2) = 0.48.
^{1}H NMR (400 MHz, $CDCl_3$) δ 7.81 (d, *J* = 7.8 Hz, 4H, A*rH*), 7.43 (dddt, *J* = 30.7, 22.9, 15.0, 7.6 Hz, 12H, A*rH*), 7.21 (dp, *J* = 23.0, 7.9 Hz, 8H, A*rH*), 3.85 (t, *J* = 7.9 Hz, 1H, ArC*H*), 3.59 – 3.48 (m, 2H, NCH_2), 2.47 (q, *J* = 8.1 Hz, 2H, CH_2CH_2).
^{13}C NMR (101 MHz, $CDCl_3$) δ 143.4, 142.5, 140.9, 139.7, 139.6, 134.0, 129.2, 128.9, 128.8, 128.3, 127.9, 127.5, 127.4, 127.1, 126.8, 48.7, 48.1, 35.5.

IR (ν_{max}, cm^{-1}) 1489 (m), 1453 (m), 1374 (s), 1169 (s), 1087 (m), 874 (s), 797 (m), 738 (s).
HRMS (APCI/QTOF) m/z: $[M + Na]^+$ Calcd for $C_{33}H_{29}NNaO_4S_2^+$ 590.1430; Found 590.1438.

N-(3-Phenyl-3-(o-tolyl)propyl)-N-(phenylsulfonyl)benzenesulfonamide (*o*-**7b**)

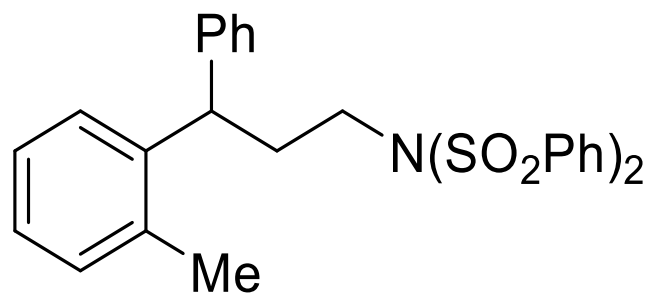

Following the general procedure H using *o*-**5c** (41 mg, 0.30 mmol, 1.0 equiv.) and phenylboronic acid (73 mg, 0.60 mmol, 2.0 equiv.), yielding N-(3-phenyl-3-(o-tolyl)propyl)-N-(phenylsulfonyl)benzenesulfonamide (*o*-**7b**) as a colorless wax (75 mg, 0.15 mmol, 49%).

$\mathbf{R_f}$ (Hex:AcOEt = 8:2) = 0.48.
^{1}H NMR (400 MHz, $CDCl_3$) δ 7.95 – 7.81 (m, 4H, Ar*H*), 7.64 – 7.54 (m, 2H, Ar*H*), 7.45 (t, *J* = 7.8 Hz, 4H, Ar*H*), 7.33 – 7.04 (m, 9H, Ar*H*), 4.03 (t, *J* = 7.8 Hz, 1H, ArC*H*), 3.58 (qdd, *J* = 15.4, 10.9, 5.5 Hz, 2H, NCH_2), 2.56 – 2.32 (m, 2H, CH_2CH_2), 2.19 (s, 3H, CH_3).
^{13}C NMR (101 MHz, $CDCl_3$) δ 143.1, 141.0, 139.7, 136.4, 133.9, 130.8, 129.2, 128.6, 128.2, 128.2, 126.6, 126.5, 126.4, 48.2, 44.7, 35.9, 19.9.
One carbon not resolved.
IR (ν_{max}, cm^{-1}) 1488 (m), 1449 (m), 1372 (s), 1166 (s), 1084 (m), 873 (m), 737 (s).
HRMS (ESI/QTOF) m/z: $[M + Na]^+$ Calcd for $C_{28}H_{27}NNaO_4S_2^+$ 528.1274; Found 528.1284.

N-(3-Phenyl-3-(m-tolyl)propyl)-N-(phenylsulfonyl)benzenesulfonamide (*m*-**7b**)

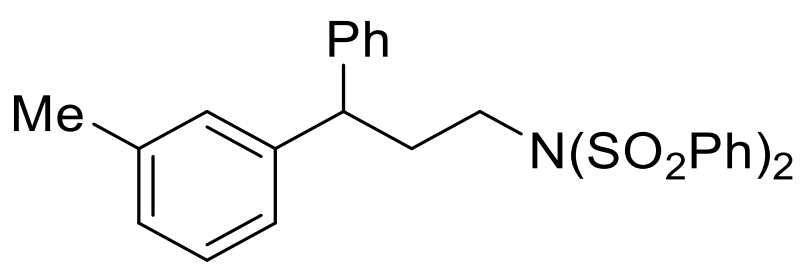

Following the general procedure H using *m*-**5c** (41 mg, 0.30 mmol, 1.0 equiv.) and phenylboronic acid (73 mg, 0.60 mmol, 2.0 equiv.), yielding N-(3-phenyl-3-(m-tolyl)propyl)-N-(phenylsulfonyl)benzenesulfonamide (*m*-**7b**) as a colorless wax (55 mg, 0.11 mmol, 36%).

$\mathbf{R_f}$ (Hex:AcOEt = 8:2) = 0.48.
^{1}H NMR (400 MHz, $CDCl_3$) δ 7.94 – 7.82 (m, 4H, Ar*H*), 7.69 – 7.59 (m, 2H, Ar*H*), 7.50 (dd, *J* = 8.5, 7.3 Hz, 4H, Ar*H*), 7.33 – 7.28 (m, 2H, Ar*H*), 7.25 – 7.16 (m, 4H, Ar*H*), 7.02 (d, *J* = 7.2 Hz, 3H, Ar*H*), 3.84 (t, *J* = 8.0 Hz, 1H, ArC*H*), 3.69 – 3.52 (m, 2H, NCH_2), 2.49 (dt, *J* = 10.2, 8.0 Hz, 2H, CH_2CH_2), 2.32 (s, 3H, CH_3).
^{13}C NMR (101 MHz, $CDCl_3$) δ 143.6, 143.4, 139.7, 138.3, 133.9, 129.2, 128.8, 128.7, 128.7, 128.3, 127.9, 127.5, 126.7, 124.8, 49.0, 48.2, 35.5, 21.6.

IR (ν_{max}, cm^{-1}) 1452 (m), 1373 (s), 1166 (s), 1083 (m), 862 (m), 741 (s).
HRMS (ESI/QTOF) m/z: $[M + Na]^+$ Calcd for $C_{28}H_{27}NNaO_4S_2^+$ 528.1274; Found 528.1279.

N-(3-Phenyl-3-(p-tolyl)propyl)-N-(phenylsulfonyl)benzenesulfonamide (*p*-**7b**)

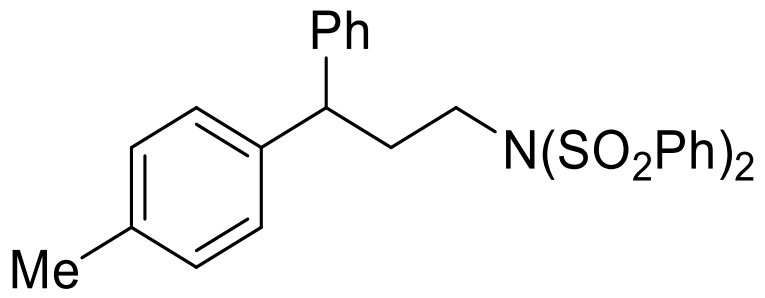

Following the general procedure F using cyclopropylbenzene **5a** (39 µL, 0.30 mmol, 1.0 equiv.) and toluene (69 mg, 0.75 mmol, 2.5 equiv.), yielding N-(3-phenyl-3-(p-tolyl)propyl)-N-(phenylsulfonyl)benzenesulfonamide (*p*-**7b**) as a colorless wax (117 mg, 0.230 mmol, 77%).

Following the general procedure H using *p*-**5c** (41 mg, 0.30 mmol, 1.0 equiv.) and phenylboronic acid (73 mg, 0.60 mmol, 2.0 equiv.), yielding N-(3-phenyl-3-(p-tolyl)propyl)-N-(phenylsulfonyl)benzenesulfonamide (*p*-**7b**) as a colorless wax (65 mg, 0.13 mmol, 43%).

$\mathbf{R_f}$ (Hex:AcOEt = 8:2) = 0.48.
^{1}H NMR (400 MHz, $CDCl_3$) δ 8.02 – 7.86 (m, 4H, Ar*H*), 7.75 – 7.59 (m, 2H, Ar*H*), 7.58 – 7.45 (m, 4H, Ar*H*), 7.31 (ddd, *J* = 7.8, 6.6, 1.8 Hz, 2H, Ar*H*), 7.22 (d, *J* = 7.4 Hz, 3H, Ar*H*), 7.12 (s, 4H, Ar*H*), 3.86 (t, *J* = 7.9 Hz, 1H, ArC*H*), 3.69 – 3.54 (m, 2H, NC*H2*), 2.50 (dt, *J* = 9.0, 7.6 Hz, 2H, CH_2CH_2), 2.33 (s, 3H, C*H3*).
^{13}C NMR (101 MHz, $CDCl_3$) δ 143.7, 140.4, 139.7, 136.2, 133.9, 129.4, 129.2, 129.2, 128.7, 128.3, 128.3, 127.8, 127.7, 126.6, 48.7, 48.2, 35.5, 21.1.
IR (ν_{max}, cm^{-1}) 1449 (m), 1372 (s), 1165 (s), 1085 (m), 872 (s), 734 (s).
HRMS (ESI/QTOF) m/z: $[M + Na]^+$ Calcd for $C_{28}H_{27}NNaO_4S_2^+$ 528.1274; Found 528.1250.

N-(3-(2-Methoxyphenyl)-3-phenylpropyl)-N-(phenylsulfonyl)benzenesulfonamide (*o*-**7c**)

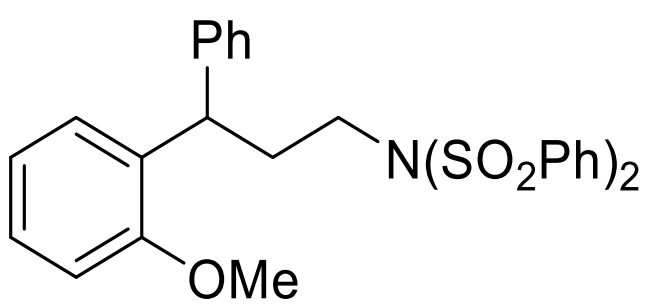

Following the general procedure G using cyclopropylbenzene **5a** (39 µL, 0.30 mmol, 1.0 equiv.) and 1-iodo-2-methoxybenzene (91 mg, 0.36 mmol, 1.2 equiv.), yielding N-(3-(2-methoxyphenyl)-3-phenylpropyl)-N-(phenylsulfonyl)benzenesulfonamide (*o*-**7c**) as a colorless wax (35 mg, 0.07 mmol, 22%).

$\mathbf{R_f}$ (Hex:AcOEt = 8:2) = 0.40.
^{1}H NMR (400 MHz, $CDCl_3$) δ 8.04 – 7.85 (m, 4H, Ar*H*), 7.70 – 7.62 (m, 2H, Ar*H*), 7.55 (dd, *J* = 8.5, 7.3 Hz, 4H, Ar*H*), 7.39 – 7.27 (m, 5H, Ar*H*), 7.26 – 7.19 (m, 2H,

Ar*H*), 6.99 (td, *J* = 7.5, 1.2 Hz, 1H, Ar*H*), 6.92 – 6.81 (m, 1H, Ar*H*), 4.41 (t, *J* = 7.9 Hz, 1H, ArC*H*), 3.82 (s, 3H, OCH_3), 3.76 – 3.58 (m, 2H, NCH_2), 2.74 – 2.36 (m, 2H, CH_2CH_2).
^{13}C NMR (101 MHz, $CDCl_3$) δ 157.0, 143.4, 139.8, 133.9, 131.9, 129.2, 128.5, 128.3, 128.2, 127.8, 127.7, 126.4, 120.8, 110.8, 55.5, 48.3, 41.1, 34.7.
IR (ν_{max}, cm^{-1}) 1493 (m), 1451 (m), 1375 (s), 1244 (m), 1169 (s), 1087 (m), 875 (m), 754 (s).
HRMS (ESI/QTOF) m/z: $[M + Na]^+$ Calcd for $C_{28}H_{27}NNaO_5S_2^+$ 544.1223; Found 544.1219.

N-(3-(4-Methoxyphenyl)-3-phenylpropyl)-N-(phenylsulfonyl)benzenesulfonamide (*p*-**7c**)

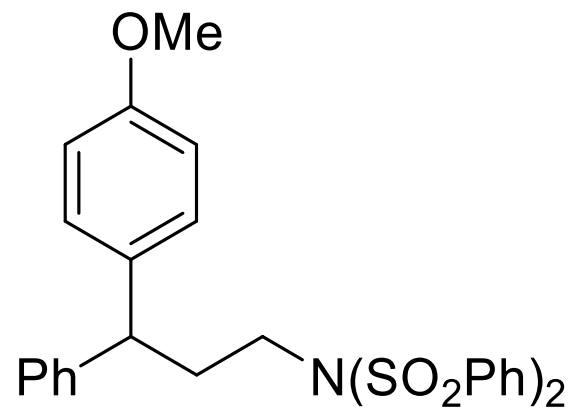

Following the general procedure F using cyclopropylbenzene **5a** (39 µL, 0.30 mmol, 1.0 equiv.) and anisole (65 mg, 0.75 mmol, 2.5 equiv.), yielding N-(3-(4-methoxyphenyl)-3-phenylpropyl)-N-(phenylsulfonyl)benzenesulfonamide (*p*-**7c**) as a colorless wax (134 mg, 0.250 mmol, 86%).

R_f (Hex:AcOEt = 8:2) = 0.40.
^{1}H NMR (400 MHz, $CDCl_3$) δ 7.86 (d, *J* = 7.6 Hz, 4H, Ar*H*), 7.57 (t, *J* = 7.5 Hz, 2H, Ar*H*), 7.45 (t, *J* = 7.7 Hz, 4H, Ar*H*), 7.30 – 7.12 (m, 5H, Ar*H*), 7.09 (d, *J* = 8.4 Hz, 2H, Ar*H*), 6.89 – 6.74 (m, 2H, Ar*H*), 3.80 (t, *J* = 8.0 Hz, 1H, ArC*H*), 3.74 (s, 3H, OCH_3), 3.59 – 3.49 (m, 2H, NCH_2), 2.43 (q, *J* = 8.1 Hz, 2H, CH_2CH_2).
^{13}C NMR (101 MHz, $CDCl_3$) δ 158.3, 143.9, 139.7, 135.5, 133.9, 129.2, 128.8, 128.7, 128.3, 127.7, 126.6, 114.1, 55.4, 48.2, 48.2, 35.6.
IR (ν_{max}, cm^{-1}) 1512 (m), 1450 (m), 1374 (s), 1251 (m), 1169 (s), 1087 (m), 1034 (m), 875 (m), 751 (s).
HRMS (ESI/QTOF) m/z: $[M + Na]^+$ Calcd for $C_{28}H_{27}NNaO_5S_2^+$ 544.1223; Found 544.1218.

N-(3-Mesityl-3-phenylpropyl)-N-(phenylsulfonyl)benzenesulfonamide (**7d**)

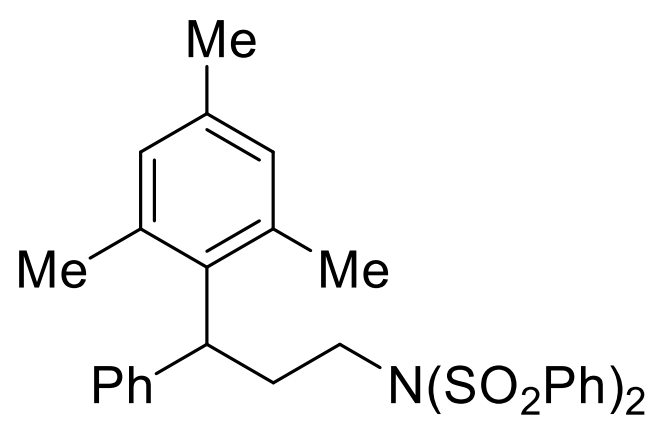

Following the general procedure F using cyclopropylbenzene **5a** (39 µL, 0.30 mmol, 1.0 equiv.) and mesitylene (72 mg, 0.75 mmol, 2.5 equiv.), yielding N-(3-mesityl-3-

phenylpropyl)-N-(phenylsulfonyl)benzenesulfonamide (**7d**) as a colorless wax (144 mg, 0.270 mmol, 90%).

R$_f$ (Hex:AcOEt = 8:2) = 0.52.
^{1}H NMR (400 MHz, $CDCl_3$) δ 7.81 – 7.65 (m, 4H, Ar*H*), 7.56 – 7.46 (m, 2H, Ar*H*), 7.38 (t, *J* = 7.8 Hz, 4H, Ar*H*), 7.20 – 7.08 (m, 2H, Ar*H*), 7.04 (t, *J* = 7.2 Hz, 1H, Ar*H*), 7.00 – 6.93 (m, 2H, Ar*H*), 4.33 (dd, *J* = 9.7, 6.4 Hz, 1H, ArC*H*), 3.66 (ddd, *J* = 15.0, 12.2, 4.6 Hz, 1H, NCH_2), 3.23 (ddd, *J* = 15.1, 12.0, 4.7 Hz, 1H, NCH_2), 2.62 (tdd, *J* = 11.8, 6.4, 4.7 Hz, 1H, CH_2CH_2), 2.38 (tdd, *J* = 12.4, 9.7, 4.7 Hz, 1H, CH_2CH_2), 2.16 (s, 3H, *p*-CH_3), 2.00 (s, 6H, *o*-CH_3).
^{13}C NMR (101 MHz, $CDCl_3$) δ 143.0, 139.7, 137.1, 136.7, 136.1, 134.0, 130.3, 129.2, 128.4, 128.3, 127.0, 125.9, 48.2, 41.3, 31.6, 21.4, 20.9.
IR (ν_{max}, cm^{-1}) 1449 (m), 1375 (s), 1169 (s), 1087 (m), 870 (m), 738 (s).
HRMS (ESI/QTOF) m/z: $[M + Na]^+$ Calcd for $C_{30}H_{31}NNaO_4S_2^+$ 556.1587; Found 556.1578.

N-(3-(2-Hydroxy-5-methylphenyl)-3-phenylpropyl)-N-(phenylsulfonyl)benzenesulfonamide (**7e**)

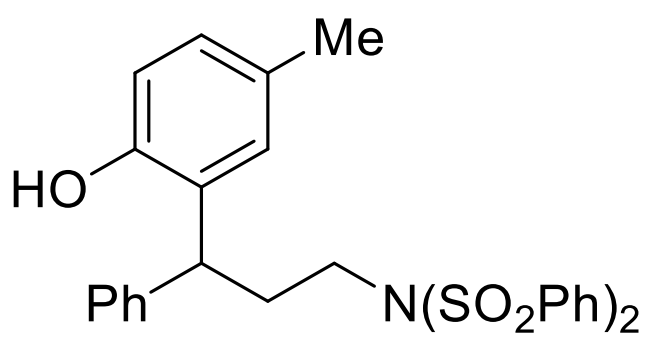

Following the general procedure F using cyclopropylbenzene **5a** (39 µL, 0.30 mmol, 1.0 equiv.) and p-cresol (81 mg, 0.75 mmol, 2.5 equiv.), yielding N-(3-(2-hydroxy-5-methylphenyl)-3-phenylpropyl)-N-(phenylsulfonyl)benzenesulfonamide (**7e**) as a colorless wax (74 mg, 0.14 mmol, 47%).

R$_f$ (Hex:AcOEt = 8:2) = 0.34.
^{1}H NMR (400 MHz, $CDCl_3$) δ 7.93 (d, *J* = 7.8 Hz, 4H, Ar*H*), 7.65 (d, *J* = 7.6 Hz, 2H, Ar*H*), 7.52 (t, *J* = 7.7 Hz, 4H, Ar*H*), 7.28 (ddt, *J* = 19.6, 14.2, 7.4 Hz, 5H, Ar*H*), 6.92 (dd, *J* = 8.1, 2.2 Hz, 1H, Ar*H*), 6.92 (dd, *J* = 8.1, 2.2 Hz, 1H, Ar*H*), 6.64 (d, *J* = 8.0 Hz, 1H, Ar*H*), 4.25 (t, *J* = 7.9 Hz, 1H, ArC*H*), 3.99 (s, 1H, O*H*), 3.65 (pd, *J* = 15.4, 6.0 Hz, 2H, NCH_2), 2.52 (ddq, *J* = 23.5, 11.5, 5.3 Hz, 2H, CH_2CH_2), 2.32 (s, 3H, CH_3).
^{13}C NMR (101 MHz, $CDCl_3$) δ 151.0, 143.1, 139.7, 133.9, 130.4, 129.4, 129.2, 128.7, 128.7, 128.3, 128.2, 128.1, 126.7, 116.0, 48.2, 41.8, 34.7, 20.9.
IR (ν_{max}, cm^{-1}) 1507 (w), 1448 (m), 1371 (s), 1165 (s), 1086 (m), 910 (m), 865 (m), 735 (s).
HRMS (QTOF) m/z: $[M + Na]^+$ Calcd for $C_{28}H_{27}NNaO_5S_2^+$ 544.1223; Found 544.1205.

N-(3,3-Di([1,1'-biphenyl]-4-yl)propyl)-N-(phenylsulfonyl)benzenesulfonamide (**7f**)

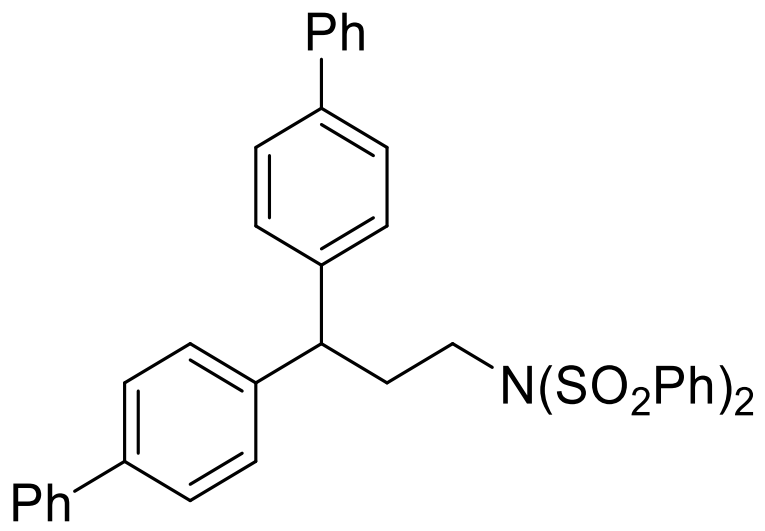

Following the general procedure F using *p*-**5b** (58 mg, 0.30 mmol, 1.0 equiv.) and biphenyl (116 mg, 0.750 mmol, 2.5 equiv.), yielding N-(3,3-di([1,1'-biphenyl]-4-yl)propyl)-N-(phenylsulfonyl)benzenesulfonamide (**7f**) as a colorless wax (140 mg, 0.210 mmol, 72%).

$\mathbf{R_f}$ (Hex:AcOEt = 8:2) = 0.48.
^{1}**H NMR** (400 MHz, $CDCl_3$) δ 7.91 (dd, *J* = 8.4, 1.3 Hz, 4H, Ar*H*), 7.60 (dt, *J* = 17.9, 7.9 Hz, 10H, Ar*H*), 7.47 (dt, *J* = 20.9, 7.7 Hz, 8H, Ar*H*), 7.35 (dd, *J* = 7.7, 5.4 Hz, 6H, Ar*H*), 3.98 (t, *J* = 7.9 Hz, 1H, ArC*H*), 3.72 – 3.52 (m, 2H, NC*H₂*), 2.59 (dt, *J* = 10.9, 7.9 Hz, 2H, CH_2CH_2).
^{13}**C NMR** (101 MHz, $CDCl_3$) δ 142.5, 140.9, 139.7, 139.7, 134.0, 129.2, 128.9, 128.3, 127.6, 127.4, 127.1, 48.5, 48.1, 35.5.
IR (ν_{max}, cm^{-1}) 1486 (m), 1374 (s), 1167 (s), 1083 (m), 875 (m), 749 (s).
HRMS (nanochip-ESI/LTQ-Orbitrap) m/z: $[M + Na]^+$ Calcd for $C_{39}H_{33}NNaO_4S_2^+$ 666.1743; Found 666.1743.

N-(3-([1,1'-Biphenyl]-4-yl)-3-(4-(trifluoromethoxy)phenyl)propyl)-N-(phenylsulfonyl)benzenesulfonamide (**7g**)

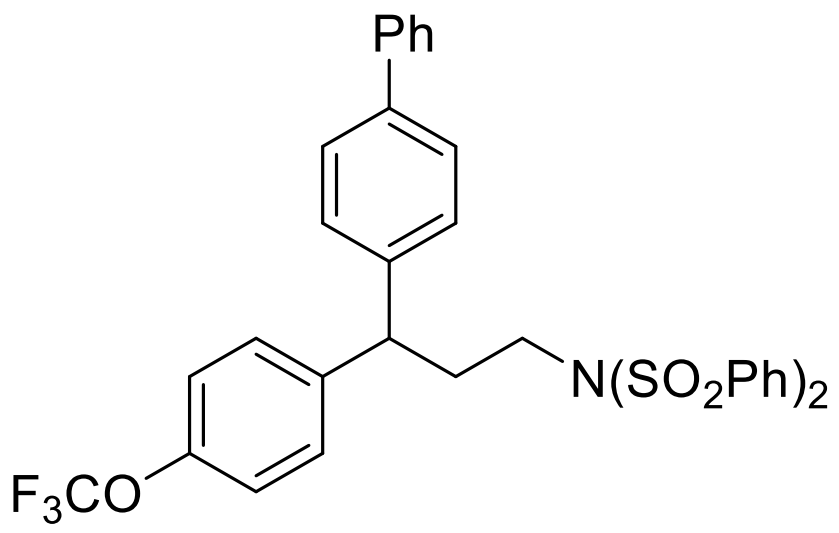

Following the general procedure F using *p*-**5i** (61 mg, 0.30 mmol, 1.0 equiv.) and biphenyl (116 mg, 0.750 mmol, 2.5 equiv.), yielding N-(3-([1,1'-biphenyl]-4-yl)-3-(4-(trifluoromethoxy)phenyl)propyl)-N-(phenylsulfonyl)benzenesulfonamide (**7g**) as a colorless wax (107 mg, 0.160 mmol, 55%).

$\mathbf{R_f}$ (Hex:AcOEt = 8:2) = 0.42.
^{1}**H NMR** (400 MHz, $CDCl_3$) δ 8.01 – 7.85 (m, 4H, Ar*H*), 7.63 (td, *J* = 7.3, 1.2 Hz, 2H, Ar*H*), 7.57 (t, *J* = 7.9 Hz, 4H, Ar*H*), 7.50 (t, *J* = 7.9 Hz, 4H, Ar*H*), 7.44 (dd, *J* = 8.3, 6.9 Hz, 2H, Ar*H*), 7.38 – 7.32 (m, 1H, Ar*H*), 7.31 – 7.25 (m, 4H, Ar*H*), 7.17 (d, *J* = 8.2 Hz, 2H, Ar*H*), 3.95 (t, *J* = 7.9 Hz, 1H, ArC*H*), 3.58 (dt, *J* = 8.6, 5.8 Hz, 2H, NCH_2), 2.54 (ddt, *J* = 14.3, 10.6, 4.6 Hz, 2H, CH_2CH_2).
^{19}**F NMR** (376 MHz, $CDCl_3$) δ -57.9.

^{13}C NMR (101 MHz, $CDCl_3$) δ 148.0 (d, *J* = 2.0 Hz), 142.2, 141.9, 140.7, 140.0, 139.6, 134.1, 129.3, 129.2, 129.0, 128.3, 128.2, 127.6, 127.5, 127.1, 120.6 (q, *J* = 257.1 Hz), 48.1, 47.9, 35.5.
One carbon not resolved.
IR (v_{max}, cm^{-1}) 2925 (w), 1489 (m), 1375 (s), 1259 (s), 1221 (m), 1167 (s), 1087 (m), 874 (m), 750 (s).
HRMS (ESI/QTOF) m/z: $[M + Na]^+$ Calcd for $C_{34}H_{28}F_3NNaO_5S_2^+$ 674.1253; Found 674.1230.

N-(3-([1,1'-Biphenyl]-4-yl)-3-(4-chlorophenyl)propyl)-N-(phenylsulfonyl)benzenesulfonamide (**7h**)

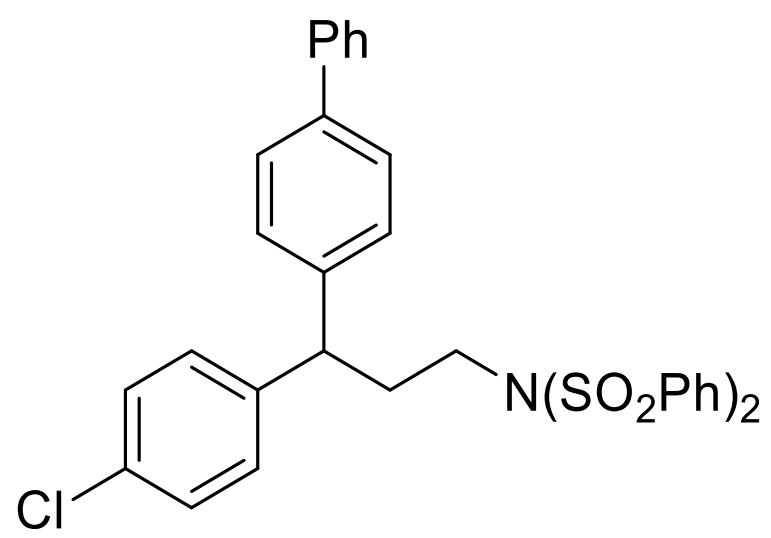

Following the general procedure F using *p*-**5l** (46 mg, 0.30 mmol, 1.0 equiv.) and biphenyl (116 mg, 0.750 mmol, 2.5 equiv.), yielding N-(3-([1,1'-biphenyl]-4-yl)-3-(4-chlorophenyl)propyl)-N-(phenylsulfonyl)benzenesulfonamide (**7h**) as a colorless wax (118 mg, 0.190 mmol, 65%).

R_f (Hex:AcOEt = 8:2) = 0.41.
^{1}H NMR (400 MHz, $CDCl_3$) δ 7.81 (dd, *J* = 8.3, 1.4 Hz, 4H, Ar*H*), 7.57 – 7.38 (m, 10H, Ar*H*), 7.35 (t, *J* = 7.7 Hz, 2H, Ar*H*), 7.29 – 7.22 (m, 1H, Ar*H*), 7.22 – 7.14 (m, 4H, Ar*H*), 7.13 – 7.07 (m, 2H, Ar*H*), 3.82 (t, *J* = 7.9 Hz, 1H, ArC*H*), 3.50 (dd, *J* = 9.5, 6.8 Hz, 2H, NC*H₂*), 2.43 (p, *J* = 7.8 Hz, 2H, CH_2CH_2).
^{13}C NMR (101 MHz, $CDCl_3$) δ 141.9, 140.7, 139.8, 139.6, 134.0, 132.6, 129.2, 129.0, 128.9, 128.3, 128.2, 127.6, 127.5, 127.1, 48.0, 47.9, 35.4.
Two carbon not resolved.
IR (v_{max}, cm^{-1}) 1488 (m), 1449 (m), 1372 (s), 1166 (s), 1087 (s), 1012 (m), 872 (m), 826 (m), 792 (m), 742 (s), 723 (m).
HRMS (ESI/QTOF) m/z: $[M + Na]^+$ Calcd for $C_{33}H_{28}ClNNaO_4S_2^+$ 624.1040; Found 624.1052.

Methyl 3-(1-([1,1'-biphenyl]-4-yl)-3-(N-(phenylsulfonyl)phenylsulfonamido)propyl)benzoate (**7i**)

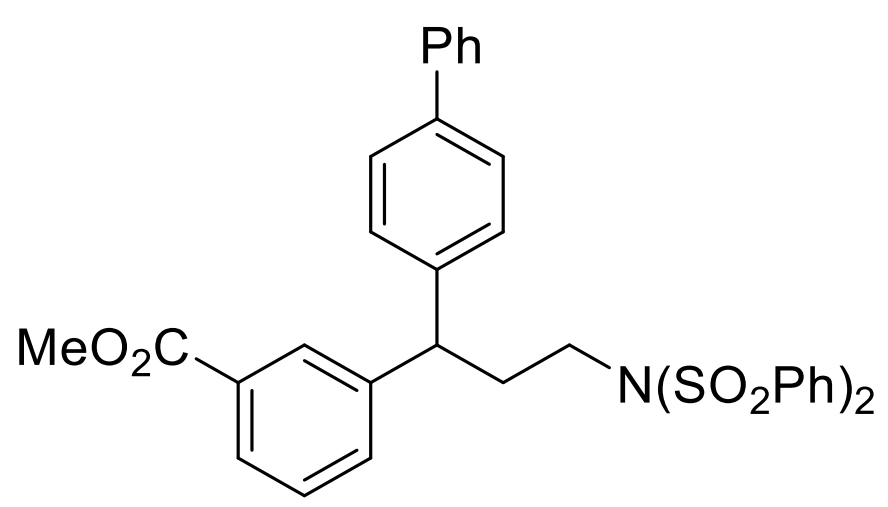

Following the general procedure F using *m*-**5g** (53 mg, 0.30 mmol, 1.0 equiv.) and biphenyl (116 mg, 0.750 mmol, 2.5 equiv.), yielding methyl 3-(1-([1,1'-biphenyl]-4-yl)-3-(N-(phenylsulfonyl)phenylsulfonamido)propyl)benzoate (**7i**) as a colorless wax (120 mg, 0.190 mmol, 64%).

R$_f$ (Hex:AcOEt = 8:2) = 0.30.
^{1}H NMR (400 MHz, $CDCl_3$) δ 7.96 – 7.88 (m, 6H, A*rH*), 7.66 – 7.60 (m, 2H, A*rH*), 7.59 – 7.31 (m, 13H, A*rH*), 7.30 – 7.27 (m, 2H, A*rH*), 3.98 (t, *J* = 7.9 Hz, 1H, ArC*H*), 3.93 (s, 3H, COOC*H$_3$*), 3.60 (t, *J* = 8.1 Hz, 2H, NC*H$_2$*), 2.55 (qt, *J* = 13.3, 6.6 Hz, 2H, CH$_2$C*H$_2$*).
^{13}C NMR (101 MHz, $CDCl_3$) δ 167.1, 143.9, 141.9, 140.8, 139.9, 139.7, 134.0, 132.4, 130.7, 129.3, 129.0, 129.0, 129.0, 128.3, 128.3, 128.1, 127.6, 127.5, 127.1, 52.3, 48.6, 48.0, 35.4.
IR (ν_{max}, cm^{-1}) 1488 (m), 1374 (s), 1166 (s), 1087 (s), 873 (s), 792 (s), 741 (s).
HRMS (ESI/QTOF) m/z: $[M + Na]^+$ Calcd for $C_{35}H_{31}NNaO_6S_2^+$ 648.1485; Found 648.1517.

N-(3-([1,1'-Biphenyl]-4-yl)-3-(2-fluorophenyl)propyl)-N-(phenylsulfonyl)benzenesulfonamide (**7j**)

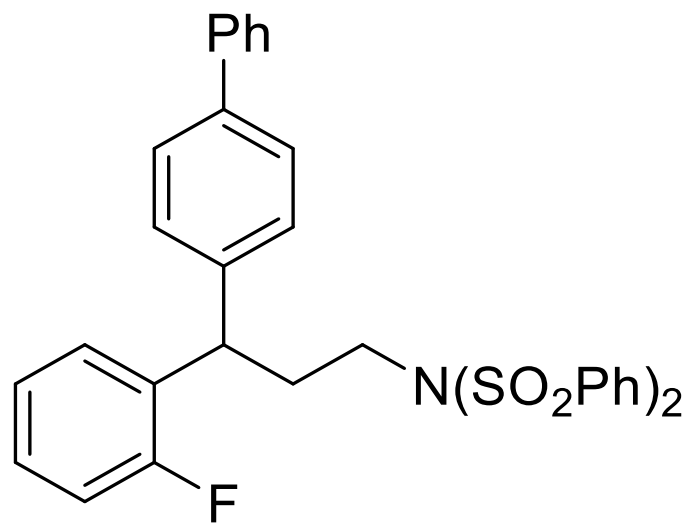

Following the general procedure F using *o*-**5m** (41 mg, 0.30 mmol, 1.0 equiv.) and biphenyl (116 mg, 0.750 mmol, 2.5 equiv.), yielding N-(3-([1,1'-biphenyl]-4-yl)-3-(2-fluorophenyl)propyl)-N-(phenylsulfonyl)benzenesulfonamide (**7j**) as a colorless wax (110 mg, 0.190 mmol, 63%).

R$_f$ (Hex:AcOEt = 8:2) = 0.36.
^{1}H NMR (400 MHz, $CDCl_3$) δ 7.92 (d, *J* = 7.7 Hz, 4H, A*rH*), 7.67 – 7.47 (m, 10H, A*rH*), 7.44 (t, *J* = 7.7 Hz, 2H, A*rH*), 7.34 (td, *J* = 8.2, 1.7 Hz, 4H, A*rH*), 7.26 – 7.10 (m, 2H, A*rH*), 7.03 (ddd, *J* = 9.9, 8.2, 1.4 Hz, 1H, A*rH*), 4.29 (t, *J* = 8.0 Hz, 1H, ArC*H*), 3.64 (t, *J* = 8.3 Hz, 2H, NC*H$_2$*), 2.69 – 2.44 (m, 2H, CH$_2$C*H$_2$*).
^{19}F NMR (376 MHz, $CDCl_3$) δ -117.4.
^{13}C NMR (101 MHz, $CDCl_3$) δ 160.7 (d, *J* = 245.8 Hz), 141.3, 140.8, 139.8, 139.7, 134.0, 130.4 (d, *J* = 14.2 Hz), 129.2, 128.9, 128.5 (d, *J* = 4.1 Hz), 128.4, 128.3, 128.3, 127.5, 127.4, 127.1, 124.5 (d, *J* = 3.5 Hz), 115.8 (d, *J* = 22.6 Hz), 48.0, 41.0 (d, *J* = 2.5 Hz), 34.5.
IR (ν_{max}, cm^{-1}) 1487 (m), 1372 (m), 1166 (s), 1084 (m), 909 (m), 901 (w), 875 (m), 754 (s), 731 (s).

HRMS (ESI/QTOF) m/z: $[M + Na]^+$ Calcd for $C_{33}H_{28}FNNaO_4S_2^+$ 608.1336; Found 608.1366.

N-(3,3-Diphenylpropyl)-N-(phenylsulfonyl)benzenesulfonamide (**7k**)

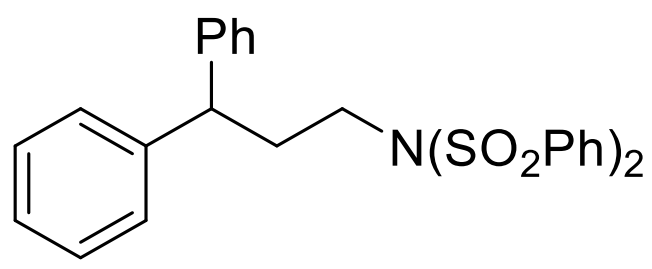

Following the general procedure G using cyclopropylbenzene **5a** (39 μL, 0.30 mmol, 1.0 equiv.) and iodobenzene (73 mg, 0.36 mmol, 1.2 equiv.), yielding N-(3,3-diphenylpropyl)-N-(phenylsulfonyl)benzenesulfonamide (**7k**) as a colorless wax (68 mg, 0.14 mmol, 46%).

Following the general procedure H using cyclopropylbenzene **5a** (39 μL, 0.30 mmol, 1.0 equiv.) and phenylboronic acid (73 mg, 0.60 mmol, 2.0 equiv.), N-(3,3-diphenylpropyl)-N-(phenylsulfonyl)benzenesulfonamide (**7k**) was obtained as a colorless wax (82 mg, 0.17 mmol, 57%).

$\mathbf{R_f}$ (Hex:AcOEt = 8:2) = 0.48.
^{1}H NMR (400 MHz, $CDCl_3$) δ 7.92 – 7.85 (m, 4H, A*rH*), 7.66 – 7.57 (m, 2H, A*rH*), 7.50 (t, *J* = 7.9 Hz, 4H, A*rH*), 7.30 (t, *J* = 7.6 Hz, 4H, A*rH*), 7.21 (d, *J* = 7.0 Hz, 6H, A*rH*), 3.88 (t, *J* = 7.9 Hz, 1H, ArC*H*), 3.66 – 3.47 (m, 2H, NCH_2), 2.50 (q, *J* = 8.0 Hz, 2H, CH_2CH_2).
^{13}C NMR (101 MHz, $CDCl_3$) δ 143.5, 139.7, 134.0, 129.2, 128.8, 128.3, 127.9, 126.7, 49.1, 48.2, 35.5.
IR (ν_{max}, cm^{-1}) 1451 (m), 1374 (s), 1167 (s), 1087 (m), 872 (m), 800 (m), 747 (s).
HRMS (ESI/QTOF) m/z: $[M + Na]^+$ Calcd for $C_{27}H_{25}NNaO_4S_2^+$ 514.1117; Found 514.1109.

N-(3-(3-Cyanophenyl)-3-phenylpropyl)-N-(phenylsulfonyl)benzenesulfonamide (*m*-**7l**)

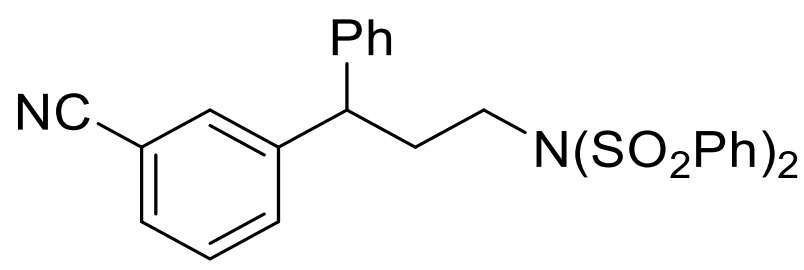

Following the general procedure G using cyclopropylbenzene **5a** (39 μL, 0.30 mmol, 1.0 equiv.) and 3-iodobenzonitrile (82 mg, 0.36 mmol, 1.2 equiv.), yielding N-(3-(3-cyanophenyl)-3-phenylpropyl)-N-(phenylsulfonyl)benzenesulfonamide (*m*-**7l**) as a colorless wax (53 mg, 0.10 mmol, 34%).

Following the general procedure H using cyclopropylbenzene **5a** (39 μL, 0.30 mmol, 1.0 equiv.) and 3-cyanophenylboronic acid (88 mg, 0.6 mmol, 2.0 equiv.), N-(3-(3-cyanophenyl)-3-phenylpropyl)-N-(phenylsulfonyl)benzenesulfonamide (*m*-**7l**) was obtained as a colorless wax (42 mg, 0.080 mmol, 27%).

$\mathbf{R_f}$ (Hex:AcOEt = 8:2) = 0.31.
^{1}H NMR (400 MHz, $CDCl_3$) δ 7.89 – 7.74 (m, 4H, A*rH*), 7.69 – 7.54 (m, 2H, A*rH*),

7.48 – 7.40 (m, 5H, Ar*H*), 7.40 – 7.35 (m, 2H, Ar*H*), 7.33 (td, *J* = 7.5, 0.9 Hz, 1H, Ar*H*), 7.27 (dd, *J* = 8.1, 6.6 Hz, 2H, Ar*H*), 7.22 – 7.13 (m, 1H, Ar*H*), 7.13 – 7.06 (m, 2H, Ar*H*), 3.84 (t, *J* = 7.9 Hz, 1H, ArC*H*), 3.59 – 3.42 (m, 2H, NCH_2), 2.57 – 2.25 (m, 2H, CH_2CH_2).
^{13}C NMR (101 MHz, $CDCl_3$) δ 145.3, 141.7, 139.6, 134.1, 132.4, 131.4, 130.5, 129.6, 129.3, 129.2, 128.3, 127.9, 127.4, 118.9, 112.8, 48.5, 47.8, 35.2.
IR (ν_{max}, cm^{-1}) 2228 (m), 1450 (m), 1372 (s), 1167 (s), 1085 (m), 861 (m), 741 (s).
HRMS (ESI/QTOF) m/z: [M + Na]$^+$ Calcd for $C_{28}H_{24}N_2NaO_4S_2^+$ 539.1070; Found 539.1085.

N-(3-(4-Cyanophenyl)-3-phenylpropyl)-N-(phenylsulfonyl)benzenesulfonamide (*p*-**7l**)

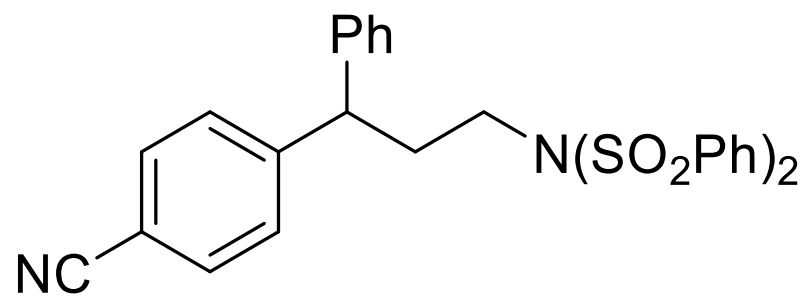

Following the general procedure H using cyclopropylbenzene **5a** (39 µL, 0.30 mmol, 1.0 equiv.) and 4-cyanophenylboronic acid (88 mg, 0.60 mmol, 2.0 equiv.), affording N-(3-(4-cyanophenyl)-3-phenylpropyl)-N-(phenylsulfonyl)benzenesulfonamide (*p*-**7l**) as a colorless wax (39 mg, 0.070 mmol, 25%).

R_f (Hex:AcOEt = 8:2) = 0.31.
^{1}H NMR (400 MHz, $CDCl_3$) δ 7.91 – 7.84 (m, 4H, Ar*H*), 7.68 – 7.61 (m, 2H, Ar*H*), 7.61 – 7.56 (m, 2H, Ar*H*), 7.54 – 7.48 (m, 4H, Ar*H*), 7.37 – 7.29 (m, 4H, Ar*H*), 7.28 – 7.23 (m, 1H, Ar*H*), 7.20 – 7.12 (m, 2H, Ar*H*), 3.94 (t, *J* = 7.9 Hz, 1H, ArC*H*), 3.56 (t, *J* = 8.1 Hz, 2H, NCH_2), 2.67 – 2.40 (m, 2H, CH_2CH_2).
^{13}C NMR (101 MHz, $CDCl_3$) δ 149.1, 141.7, 139.6, 134.1, 132.6, 129.3, 129.1, 128.6, 128.3, 127.9, 127.4, 118.9, 110.7, 49.0, 47.8, 35.1.
IR (ν_{max}, cm^{-1}) 2228 (w), 1450 (m), 1373 (s), 1166 (s), 1087 (m), 874 (m), 741 (s).
HRMS (ESI/QTOF) m/z: [M + Na]$^+$ Calcd for $C_{28}H_{24}N_2NaO_4S_2^+$ 539.1070; Found 539.1076.

N-(3-(2-Ethylphenyl)-3-phenylpropyl)-N-(phenylsulfonyl)benzenesulfonamide (*o*-**7m**)

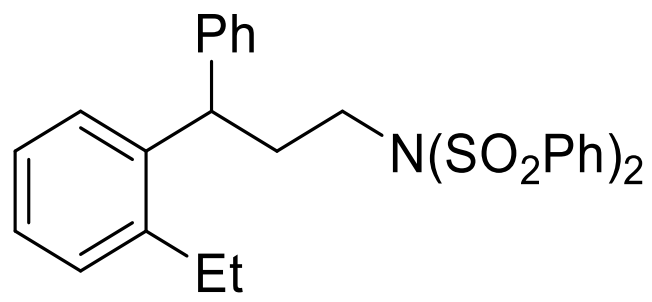

Following the general procedure H using *o*-**5d** (45 mg, 0.30 mmol, 1.0 equiv.) and phenylboronic acid (73 mg, 0.60 mmol, 2.0 equiv.), N-(3-(2-ethylphenyl)-3-phenylpropyl)-N-(phenylsulfonyl)benzenesulfonamide (*o*-**7m**) was obtained as a colorless wax (60 mg, 0.12 mmol, 38%).

R_f (Hex:AcOEt = 8:2) = 0.50.
^{1}H NMR (400 MHz, $CDCl_3$) δ 7.89 – 7.81 (m, 4H, Ar*H*), 7.61 – 7.53 (m, 2H, Ar*H*), 7.45 (dd, *J* = 8.5, 7.2 Hz, 4H, Ar*H*), 7.31 – 7.10 (m, 9H, Ar*H*), 4.12 (t, *J* = 7.8 Hz, 1H,

ArC*H*), 3.70 – 3.49 (m, 2H, NCH_2), 2.63 (dq, *J* = 15.0, 7.5 Hz, 1H, CH_2CH_2), 2.53 (dq, *J* = 14.7, 7.4 Hz, 1H, CH_2CH_2), 2.48 – 2.38 (m, 2H, CH_3CH_2), 1.07 (t, *J* = 7.5 Hz, 3H, CH_2CH_3).
^{13}C NMR (101 MHz, $CDCl_3$) δ 143.5, 142.1, 140.3, 139.7, 133.9, 129.2, 129.0, 128.7, 128.3, 128.1, 127.0, 126.8, 126.5, 126.2, 48.2, 43.9, 36.3, 25.6, 15.3.
IR (ν_{max}, cm^{-1}) 1449 (m), 1373 (s), 1166 (s), 1085 (m), 1029 (w), 871 (m), 738 (s).
HRMS (ESI/QTOF) m/z: $[M + Na]^+$ Calcd for $C_{29}H_{29}NNaO_4S_2^+$ 542.1430; Found 542.1438.

N-(3-(3-ethylphenyl)-3-phenylpropyl)-N-(phenylsulfonyl)benzenesulfonamide (*m*-**7m**)

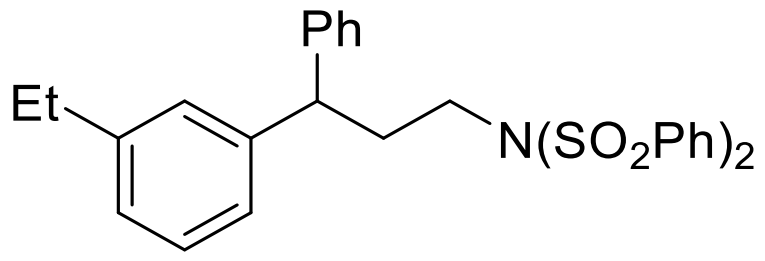

Following the general procedure H using *o*-**5d** (45 mg, 0.30 mmol, 1.0 equiv.) and phenylboronic acid (73 mg, 0.60 mmol, 2.0 equiv.), N-(3-(3-ethylphenyl)-3-phenylpropyl)-N-(phenylsulfonyl)benzenesulfonamide (*m*-**7m**) was obtained as a colorless wax (55 mg, 0.11 mmol, 35%).

$\mathbf{R_f}$ (Hex:AcOEt = 8:2) = 0.50.
^{1}H NMR (400 MHz, $CDCl_3$) δ 7.98 – 7.90 (m, 4H, Ar*H*), 7.65 (t, *J* = 7.5 Hz, 2H, Ar*H*), 7.53 (t, *J* = 7.7 Hz, 4H, Ar*H*), 7.34 (t, *J* = 7.5 Hz, 2H, Ar*H*), 7.30 – 7.21 (m, 4H, Ar*H*), 7.13 – 7.05 (m, 3H, Ar*H*), 3.90 (t, *J* = 7.9 Hz, 1H, ArC*H*), 3.63 (dd, *J* = 10.2, 6.4 Hz, 2H, NCH_2), 2.67 (q, *J* = 7.6 Hz, 2H, CH_3CH_2), 2.55 (dt, *J* = 9.5, 7.3 Hz, 2H, CH_2CH_2), 1.27 (t, *J* = 7.6 Hz, 3H, CH_2CH_3).
^{13}C NMR (101 MHz, $CDCl_3$) δ 144.7, 143.6, 143.3, 139.7, 133.9, 129.2, 128.7, 128.7, 128.3, 127.8, 127.5, 126.6, 126.2, 125.1, 49.1, 48.2, 35.5, 29.0, 15.7.
IR (ν_{max}, cm^{-1}) 1449 (m), 1372 (s), 1166 (s), 1084 (m), 862 (m), 741 (s).
HRMS (ESI/QTOF) m/z: $[M + Na]^+$ Calcd for $C_{29}H_{29}NNaO_4S_2^+$ 542.1430; Found 542.1435.

N-(3-(4-Ethylphenyl)-3-phenylpropyl)-N-(phenylsulfonyl)benzenesulfonamide (*p*-**7m**)

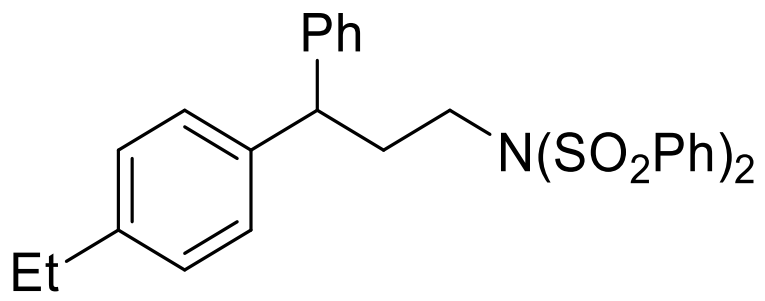

Following the general procedure H using *o*-**5d** (45 mg, 0.30 mmol, 1.0 equiv.) and phenylboronic acid (73 mg, 0.60 mmol, 2.0 equiv.), N-(3-(4-ethylphenyl)-3-phenylpropyl)-N-(phenylsulfonyl)benzenesulfonamide (*p*-**7m**) was obtained as a colorless wax (37 mg, 0.070 mmol, 24%).

$\mathbf{R_f}$ (Hex:AcOEt = 8:2) = 0.50.
^{1}H NMR (400 MHz, $CDCl_3$) δ 7.93 – 7.87 (m, 4H, Ar*H*), 7.62 (t, *J* = 7.4 Hz, 2H, Ar*H*), 7.50 (t, *J* = 7.8 Hz, 4H, Ar*H*), 7.30 (t, *J* = 7.4 Hz, 2H, Ar*H*), 7.22 (dd, *J* = 8.0, 2.1 Hz,

3H, Ar*H*), 7.14 (s, 4H, Ar*H*), 3.86 (t, *J* = 7.9 Hz, 1H, ArC*H*), 3.58 (dd, *J* = 10.4, 6.1 Hz, 2H, NC*H*2), 2.63 (q, *J* = 7.6 Hz, 2H, CH_3CH_2), 2.56 – 2.45 (m, 2H, CH_2CH_2), 1.23 (t, *J* = 7.6 Hz, 3H, CH_2CH_3).
^{13}C NMR (101 MHz, $CDCl_3$) δ 143.7, 142.6, 140.7, 139.7, 133.9, 129.2, 128.7, 128.3, 128.2, 127.8, 127.8, 126.6, 48.7, 48.2, 35.5, 28.5, 15.7.
IR (ν_{max}, cm^{-1}) 1449 (m), 1373 (s), 1166 (s), 1086 (m), 872 (m), 742 (s).
HRMS (ESI/QTOF) m/z: $[M + Na]^+$ Calcd for $C_{29}H_{29}NNaO_4S_2^+$ 542.1430; Found 542.1431.

N-(3-(3-(Tert-butyl)phenyl)-3-phenylpropyl)-N-(phenylsulfonyl)benzenesulfonamide (*m*-**7n**)

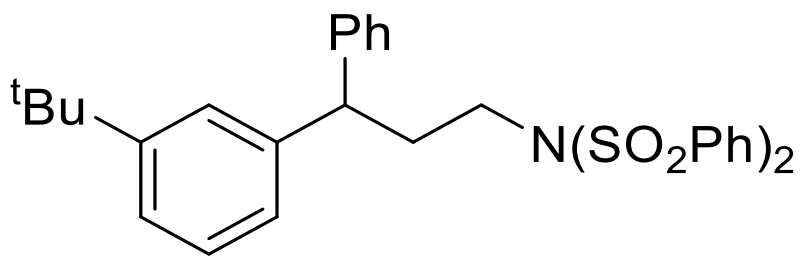

Following the general procedure H using *m*-**5e** (54 mg, 0.30 mmol, 1.0 equiv.) and phenylboronic acid (73 mg, 0.60 mmol, 2.0 equiv.), N-(3-(3-(tert-butyl)phenyl)-3-phenylpropyl)-N-(phenylsulfonyl)benzenesulfonamide (*m*-**7n**) was obtained as a colorless wax (67 mg, 0.12 mmol, 41%).

R_f (Hex:AcOEt = 8:2) = 0.51.
^{1}H NMR (400 MHz, $CDCl_3$) δ 7.89 – 7.70 (m, 4H, Ar*H*), 7.63 – 7.47 (m, 2H, Ar*H*), 7.43 – 7.37 (m, 4H, Ar*H*), 7.26 – 7.08 (m, 8H, Ar*H*), 6.93 (dt, *J* = 6.1, 2.1 Hz, 1H, Ar*H*), 3.79 (t, *J* = 8.0 Hz, 1H, ArC*H*), 3.58 – 3.41 (m, 2H, NC*H*2), 2.50 – 2.35 (m, 2H, CH_2CH_2), 1.22 (s, 9H, CCH_3).
^{13}C NMR (101 MHz, $CDCl_3$) δ 151.6, 143.7, 142.9, 139.7, 133.9, 129.2, 128.7, 128.4, 128.2, 127.8, 126.6, 125.0, 124.8, 123.7, 49.4, 48.3, 35.6, 34.8, 31.5.
IR (ν_{max}, cm^{-1}) 1450 (m), 1375 (s), 1280 (m), 1169 (s), 1134 (m), 1086 (m), 865 (m), 743 (s).
HRMS (ESI/QTOF) m/z: $[M + Na]^+$ Calcd for $C_{31}H_{33}NNaO_4S_2^+$ 570.1743; Found 570.1751.

N-(3-(4-(Tert-butyl)phenyl)-3-phenylpropyl)-N-(phenylsulfonyl)benzenesulfonamide (*p*-**7n**)

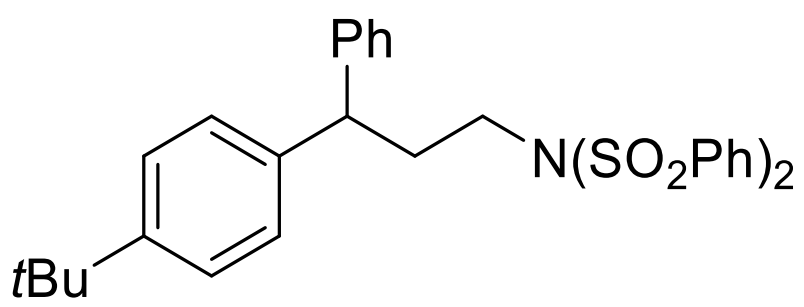

Following the general procedure H using *p*-**5e** (54 mg, 0.30 mmol, 1.0 equiv.) and phenylboronic acid (73 mg, 0.60 mmol, 2.0 equiv.), N-(3-(4-(tert-butyl)phenyl)-3-phenylpropyl)-N-(phenylsulfonyl)benzenesulfonamide (*p*-**7n**) was obtained as a colorless wax (64 mg, 0.12 mmol, 39%).

$\mathbf{R_f}$ (Hex:AcOEt = 8:2) = 0.51.
^{1}H NMR (400 MHz, $CDCl_3$) δ 7.85 – 7.76 (m, 4H, Ar*H*), 7.56 – 7.48 (m, 2H, Ar*H*), 7.40 (dd, *J* = 8.4, 7.2 Hz, 4H, Ar*H*), 7.27 – 7.17 (m, 4H, Ar*H*), 7.17 – 7.03 (m, 5H, Ar*H*), 3.77 (t, *J* = 7.9 Hz, 1H, ArC*H*), 3.52 – 3.44 (m, 2H, NCH_2), 2.47 – 2.36 (m, 2H, CH_2CH_2), 1.21 (s, 9H, CCH_3).
^{13}C NMR (101 MHz, $CDCl_3$) δ 149.4, 143.7, 140.3, 139.7, 133.9, 129.2, 128.7, 128.3, 127.9, 127.4, 126.6, 125.6, 48.6, 48.2, 35.5, 34.5, 31.5.
IR (ν_{max}, cm^{-1}) 1451 (m), 1376 (s), 1169 (s), 1088 (m), 875 (m), 793 (m), 754 (m), 738 (m).
HRMS (ESI/QTOF) m/z: $[M + Na]^+$ Calcd for $C_{31}H_{33}NNaO_4S_2^+$ 570.1743; Found 570.1769.

N-(3-(4-(2-Oxopropyl)phenyl)-3-phenylpropyl)-N-(phenylsulfonyl)benzenesulfonamide (**7o**)

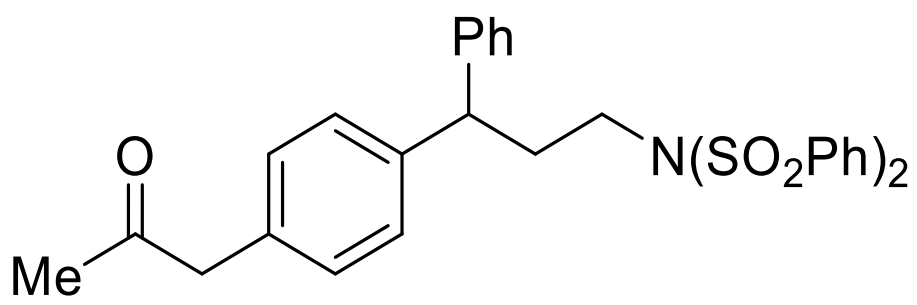

Following the general procedure H using **5f** (54 mg, 0.30 mmol, 1.0 equiv.) and phenylboronic acid (73 mg, 0.60 mmol, 2.0 equiv.), N-(3-(4-(2-oxopropyl)phenyl)-3-phenylpropyl)-N-(phenylsulfonyl)benzenesulfonamide (**7o**) was obtained as a colorless wax (69 mg, 0.13 mmol, 42%).

$\mathbf{R_f}$ (Hex:AcOEt = 8:2) = 0.30.
^{1}H NMR (400 MHz, $CDCl_3$) δ 7.84 – 7.77 (m, 4H, Ar*H*), 7.60 – 7.50 (m, 2H, Ar*H*), 7.43 (dd, *J* = 8.4, 7.2 Hz, 4H, Ar*H*), 7.28 – 7.20 (m, 2H, Ar*H*), 7.18 – 7.03 (m, 7H, Ar*H*), 3.79 (t, *J* = 7.9 Hz, 1H, ArC*H*), 3.59 (s, 2H, COCH_2), 3.50 (ddd, *J* = 10.9, 6.5, 2.4 Hz, 2H, NCH_2), 2.42 (q, *J* = 8.1 Hz, 2H, CH_2CH_2), 2.08 (s, 3H, COCH_3).
^{13}C NMR (101 MHz, $CDCl_3$) δ 206.5, 143.3, 142.3, 139.7, 134.0, 132.6, 129.8, 129.2, 128.8, 128.3, 128.2, 127.9, 126.8, 50.6, 48.7, 48.1, 35.5, 29.6.
IR (ν_{max}, cm^{-1}) 1721 (m), 1449 (m), 1371 (s), 1165 (s), 1085 (m), 872 (m), 738 (s).
HRMS (ESI/QTOF) m/z: $[M + Na]^+$ Calcd for $C_{30}H_{29}NNaO_5S_2^+$ 570.1379; Found 570.1388.

N-(3-(2-Chlorophenyl)-3-phenylpropyl)-N-(phenylsulfonyl)benzenesulfonamide (*o*-**7p**)

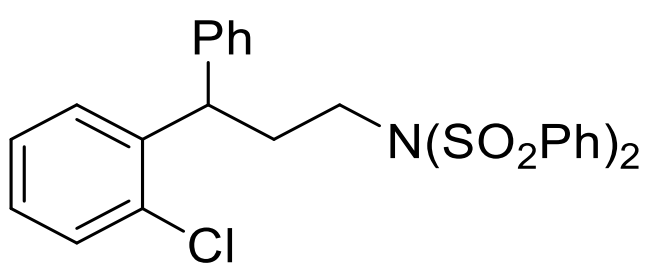

Following the general procedure H using cyclopropylbenzene **5a** (39 μL, 0.30 mmol, 1.0 equiv.) and 2-chlorophenylboronic acid (94 mg, 0.60 mmol, 2.0 equiv.), N-(3-(2-chlorophenyl)-3-phenylpropyl)-N-(phenylsulfonyl)benzenesulfonamide (*o*-**7p**) was obtained as a colorless wax (63 mg, 0.12 mmol, 40%).

R_f (Hex:AcOEt = 8:2) = 0.31.
^{1}H NMR (400 MHz, $CDCl_3$) δ 7.85 – 7.79 (m, 4H, Ar*H*), 7.59 – 7.51 (m, 2H, Ar*H*), 7.43 (dd, *J* = 8.5, 7.2 Hz, 4H, Ar*H*), 7.30 – 7.14 (m, 8H, Ar*H*), 7.08 (ddd, *J* = 7.9, 7.0, 1.9 Hz, 1H, Ar*H*), 4.36 (t, *J* = 7.9 Hz, 1H, ArC*H*), 3.62 – 3.43 (m, 2H, NCH_2), 2.51 – 2.18 (m, 2H, CH_2CH_2).
^{13}C NMR (101 MHz, $CDCl_3$) δ 142.0, 140.8, 139.7, 134.2, 134.0, 129.9, 129.2, 128.7, 128.6, 128.3, 128.3, 127.9, 127.3, 126.9, 47.9, 44.6, 35.2.
IR (ν_{max}, cm^{-1}) 1449 (m), 1375 (s), 1170 (s), 1087 (m), 872 (m), 753 (s).
HRMS (ESI/QTOF) m/z: $[M + Na]^+$ Calcd for $C_{27}H_{24}ClNNaO_4S_2^+$ 548.0727; Found 548.0735.

N-(3-(3-Chlorophenyl)-3-phenylpropyl)-N-(phenylsulfonyl)benzenesulfonamide (*m*-**7p**)

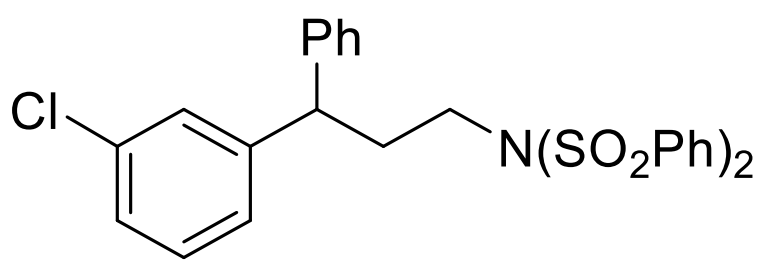

Following the general procedure H using *m*-**5l** (47 mg, 0.30 mmol, 1.0 equiv.) and phenylboronic acid (73 mg, 0.60 mmol, 2.0 equiv.), N-(3-(3-chlorophenyl)-3-phenylpropyl)-N-(phenylsulfonyl)benzenesulfonamide (*m*-**7p**) was obtained as a colorless wax (33 mg, 0.060 mmol, 21%).

Following the general procedure H using cyclopropylbenzene **5a** (39 µL, 0.30 mmol, 1.0 equiv.) and 3-chlorophenylboronic acid (94 mg, 0.60 mmol, 2.0 equiv.), N-(3-(3-chlorophenyl)-3-phenylpropyl)-N-(phenylsulfonyl)benzenesulfonamide (*m*-**7p**) was obtained as a colorless wax (96 mg, 0.18 mmol, 61%).

R_f (Hex:AcOEt = 8:2) = 0.31.
^{1}H NMR (400 MHz, $CDCl_3$) δ 7.98 – 7.74 (m, 4H, Ar*H*), 7.61 – 7.50 (m, 2H, Ar*H*), 7.43 (dd, *J* = 8.5, 7.3 Hz, 4H, Ar*H*), 7.28 – 7.21 (m, 2H, Ar*H*), 7.20 – 7.10 (m, 6H, Ar*H*), 7.02 (dt, *J* = 7.3, 1.6 Hz, 1H, Ar*H*), 3.77 (t, *J* = 7.9 Hz, 1H, ArC*H*), 3.53 – 3.44 (m, 2H, NCH_2), 2.48 – 2.27 (m, 2H, CH_2CH_2).
^{13}C NMR (101 MHz, $CDCl_3$) δ 145.6, 142.6, 139.6, 134.6, 134.0, 130.1, 129.3, 128.9, 128.3, 128.0, 127.8, 127.0, 126.9, 126.1, 48.7, 47.9, 35.3.
IR (ν_{max}, cm^{-1}) 1450 (m), 1374 (s), 1169 (s), 1085 (m), 884 (m), 742 (s).
HRMS (ESI/QTOF) m/z: $[M + Na]^+$ Calcd for $C_{27}H_{24}ClNNaO_4S_2^+$ 548.0727; Found 548.0740.

N-(3-(4-Chlorophenyl)-3-phenylpropyl)-N-(phenylsulfonyl)benzenesulfonamide (*p*-**7p**)

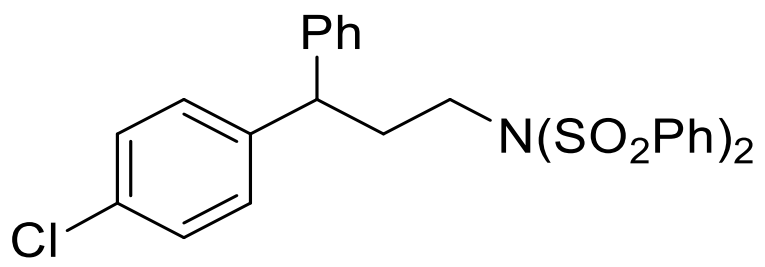

Following the general procedure H using *p*-**5l** (47 mg, 0.30 mmol, 1.0 equiv.) and phenylboronic acid (73 mg, 0.60 mmol, 2.0 equiv.), N-(3-(4-chlorophenyl)-3-

phenylpropyl)-N-(phenylsulfonyl)benzenesulfonamide (*p*-**7p**) was obtained as a colorless wax (64 mg, 0.12 mmol, 41%).

Following the general procedure H using cyclopropylbenzene **5a** (39 μL, 0.30 mmol, 1.0 equiv.) and 4-chlorophenylboronic acid (94 mg, 0.60 mmol, 2.0 equiv.), N-(3-(4-chlorophenyl)-3-phenylpropyl)-N-(phenylsulfonyl)benzenesulfonamide (*p*-**7p**) was obtained as a colorless wax (32 mg, 0.060 mmol, 20%).

R$_f$ (Hex:AcOEt = 8:2) = 0.31.
^{1}H NMR (400 MHz, $CDCl_3$) δ 7.84 – 7.77 (m, 4H, Ar*H*), 7.56 – 7.50 (m, 2H, Ar*H*), 7.41 (dd, *J* = 8.5, 7.2 Hz, 4H, Ar*H*), 7.25 – 7.12 (m, 5H, Ar*H*), 7.11 – 7.02 (m, 4H, Ar*H*), 3.77 (t, *J* = 7.9 Hz, 1H, ArC*H*), 3.54 – 3.41 (m, 2H, NCH_2), 2.40 (dqd, *J* = 11.5, 5.6, 2.6 Hz, 2H, CH_2CH_2).
^{13}C NMR (101 MHz, $CDCl_3$) δ 142.9, 142.0, 139.6, 134.0, 132.4, 129.2, 129.2, 128.9, 128.9, 128.2, 127.8, 126.9, 48.3, 48.0, 35.4.
IR (ν_{max}, cm^{-1}) 1491 (m), 1449 (m), 1374 (s), 1168 (s), 1089 (m), 874 (m), 790 (m), 752 (s).
HRMS (ESI/QTOF) m/z: $[M + Na]^+$ Calcd for $C_{27}H_{24}ClNNaO_4S_2^+$ 548.0727; Found 548.0732.

N-(3-(2-Fluorophenyl)-3-phenylpropyl)-N-(phenylsulfonyl)benzenesulfonamide (*o*-**7q**)

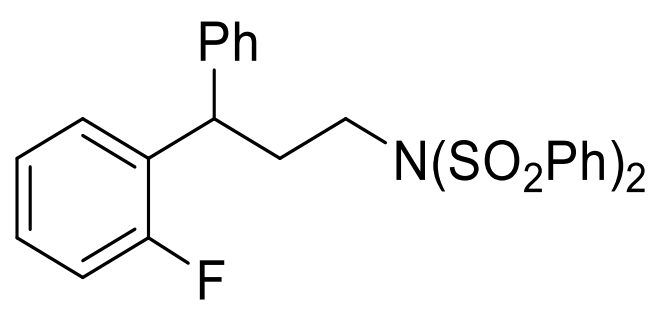

Following the general procedure H using *o*-**5m** (42 mg, 0.30 mmol, 1.0 equiv.) and phenylboronic acid (73 mg, 0.60 mmol, 2.0 equiv.), N-(3-(2-fluorophenyl)-3-phenylpropyl)-N-(phenylsulfonyl)benzenesulfonamide (*o*-**7q**) was obtained as a colorless wax (62 mg, 0.12 mmol, 41%).

Following the general procedure H using cyclopropylbenzene **5a** (39 μL, 0.30 mmol, 1.0 equiv.) and 2-fluorophenylboronic acid (84 mg, 0.60 mmol, 2.0 equiv.), N-(3-(2-fluorophenyl)-3-phenylpropyl)-N-(phenylsulfonyl)benzenesulfonamide (*o*-**7q**) was obtained as a colorless wax (70 mg, 0.14 mmol, 46%).

R$_f$ (Hex:AcOEt = 8:2) = 0.30.
^{1}H NMR (400 MHz, $CDCl_3$) δ 7.94 (d, *J* = 7.8 Hz, 4H, Ar*H*), 7.65 (t, *J* = 7.4 Hz, 2H, Ar*H*), 7.53 (t, *J* = 7.7 Hz, 4H, Ar*H*), 7.30 (dp, *J* = 22.3, 7.5 Hz, 7H, Ar*H*), 7.15 (t, *J* = 7.4 Hz, 1H, Ar*H*), 7.08 – 6.98 (m, 1H, Ar*H*), 4.28 (t, *J* = 8.0 Hz, 1H, ArC*H*), 3.66 (t, *J* = 8.2 Hz, 2H, NCH_2), 2.62 – 2.49 (m, 2H, CH_2CH_2).
^{19}F NMR (376 MHz, $CDCl_3$) δ -117.4 (dt, *J* = 11.6, 6.0 Hz).
^{13}C NMR (101 MHz, $CDCl_3$) δ 160.6 (d, *J* = 245.7 Hz), 142.2, 139.6, 134.0, 130.4 (d, *J* = 14.3 Hz), 129.2, 128.8, 128.5 (d, *J* = 4.2 Hz), 128.3, 128.2, 127.9, 126.9, 124.4 (d, *J* = 3.4 Hz), 115.7 (d, *J* = 22.5 Hz), 48.0, 41.2 (d, *J* = 2.5 Hz), 34.5.
IR (ν_{max}, cm^{-1}) 1491 (m), 1451 (m), 1372 (s), 1227 (m), 1168 (s), 1086 (m), 875 (m),

755 (s).
HRMS (ESI/QTOF) m/z: $[M + Na]^+$ Calcd for $C_{27}H_{24}FNNaO_4S_2^+$ 532.1023; Found 532.1028.

N-(3-(3-fluorophenyl)-3-phenylpropyl)-N-(phenylsulfonyl)benzenesulfonamide (*m*-**7q**)

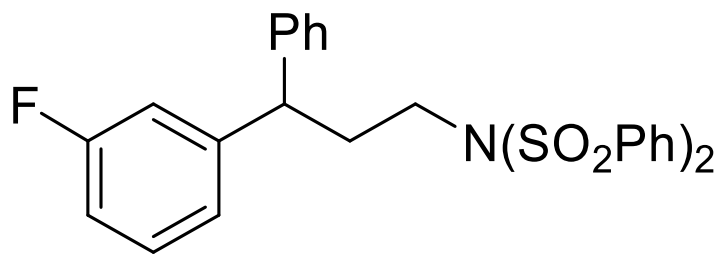

Following the general procedure H using *m*-**5m** (42 mg, 0.30 mmol, 1.0 equiv.) and phenylboronic acid (73 mg, 0.60 mmol, 2.0 equiv.), N-(3-(3-fluorophenyl)-3-phenylpropyl)-N-(phenylsulfonyl)benzenesulfonamide (*m*-**7q**) was obtained as a colorless wax (32 mg, 0.060 mmol, 21%).

Following the general procedure H using cyclopropylbenzene **5a** (39 µL, 0.30 mmol, 1.0 equiv.) and 2-fluorophenylboronic acid (84 mg, 0.60 mmol, 2.0 equiv.), N-(3-(3-fluorophenyl)-3-phenylpropyl)-N-(phenylsulfonyl)benzenesulfonamide (*m*-**7q**) was obtained as a colorless wax (55 mg, 0.11 mmol, 37%).

R_f (Hex:AcOEt = 8:2) = 0.30.
^{1}H NMR (400 MHz, $CDCl_3$) δ 7.81 (d, *J* = 7.9 Hz, 4H, A*rH*), 7.54 (t, *J* = 7.4 Hz, 2H, A*rH*), 7.42 (t, *J* = 7.7 Hz, 4H, A*rH*), 7.30 – 7.06 (m, 6H, A*rH*), 6.92 (d, *J* = 7.7 Hz, 1H, A*rH*), 6.81 (dd, *J* = 8.7, 5.9 Hz, 2H, A*rH*), 3.79 (t, *J* = 7.9 Hz, 1H, ArC*H*), 3.50 (t, *J* = 8.2 Hz, 2H, NCH_2), 2.40 (h, *J* = 6.4 Hz, 2H, CH_2CH_2).
^{19}F NMR (376 MHz, $CDCl_3$) δ -112.7.
^{13}C NMR (101 MHz, $CDCl_3$) δ 163.1 (d, *J* = 246.1 Hz), 146.2 (d, *J* = 6.8 Hz), 142.7, 139.6, 134.0, 130.2 (d, *J* = 8.3 Hz), 129.2, 128.9, 128.3, 127.8, 127.0, 123.5 (d, *J* = 2.7 Hz), 114.8 (d, *J* = 21.4 Hz), 113.6 (d, *J* = 21.0 Hz), 48.7 (d, *J* = 1.7 Hz), 48.0, 35.3.
IR (v_{max}, cm^{-1}) 1590 (w), 1487 (w), 1449 (m), 1374 (s), 1169 (s), 1087 (m), 864 (m), 750 (s).
HRMS (ESI/QTOF) m/z: $[M + Na]^+$ Calcd for $C_{27}H_{24}FNNaO_4S_2^+$ 532.1023; Found 532.1033.

N-(3-(4-Fluorophenyl)-3-phenylpropyl)-N-(phenylsulfonyl)benzenesulfonamide (*p*-**7q**)

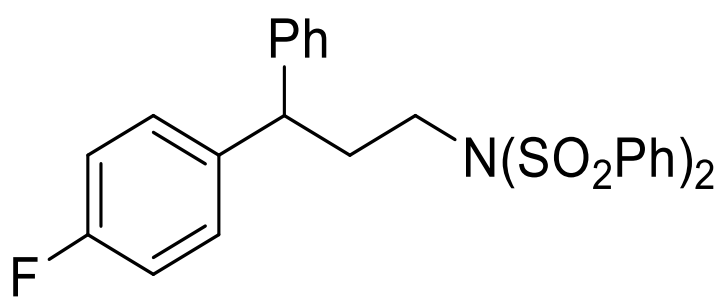

Following the general procedure H using *p*-**5m** (42 mg, 0.30 mmol, 1.0 equiv.) and phenylboronic acid (73 mg, 0.60 mmol, 2.0 equiv.), N-(3-(4-fluorophenyl)-3-phenylpropyl)-N-(phenylsulfonyl)benzenesulfonamide (*p*-**7q**) was obtained as a colorless wax (41 mg, 0.08 mmol, 27%).

Following the general procedure H using cyclopropylbenzene **5a** (39 µL, 0.30 mmol, 1.0 equiv.) and 2-fluorophenylboronic acid (84 mg, 0.60 mmol, 2.0 equiv.), N-(3-(4-fluorophenyl)-3-phenylpropyl)-N-(phenylsulfonyl)benzenesulfonamide (*p*-**7q**) was obtained as a colorless wax (66 mg, 0.13 mmol, 43%).

R_f (Hex:AcOEt = 8:2) = 0.30.
^{1}H NMR (400 MHz, $CDCl_3$) δ 7.92 – 7.87 (m, 4H, A*rH*), 7.66 – 7.59 (m, 2H, A*rH*), 7.51 (dd, *J* = 8.5, 7.2 Hz, 4H, A*rH*), 7.31 (dd, *J* = 8.1, 6.7 Hz, 2H, A*rH*), 7.24 – 7.13 (m, 5H, A*rH*), 7.02 – 6.94 (m, 2H, A*rH*), 3.87 (t, *J* = 8.0 Hz, 1H, ArC*H*), 3.61 – 3.51 (m, 2H, NC*H_2*), 2.47 (qd, *J* = 7.6, 2.8 Hz, 2H, CH_2C*H_2*).
^{19}F NMR (376 MHz, $CDCl_3$) δ -117.0.
^{13}C NMR (101 MHz, $CDCl_3$) δ 161.7 (d, *J* = 245.1 Hz), 143.3, 139.7, 139.2 (d, *J* = 3.3 Hz), 134.0, 129.3, 129.3, 129.2, 128.9, 128.3, 126.9, 115.6 (d, *J* = 21.2 Hz), 48.3, 48.1, 35.6.
IR (ν_{max}, cm^{-1}) 1509 (m), 1450 (m), 1372 (s), 1223 (m), 1167 (s), 1087 (m), 874 (m), 750 (s).
HRMS (ESI/QTOF) m/z: [M + Na]$^+$ Calcd for $C_{27}H_{24}FNNaO_4S_2^+$ 532.1023; Found 532.1032.

N-(3-(4-Bromophenyl)-3-phenylpropyl)-N-(phenylsulfonyl)benzenesulfonamide (**7r**)

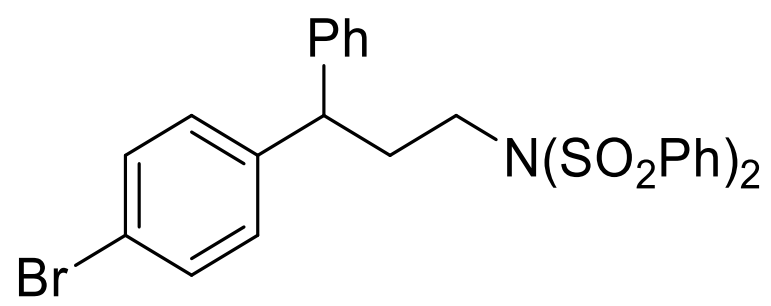

Following the general procedure H using 4-bromo-1-cyclopropylbenzene **5aa** (61 mg, 0.30 mmol, 1.0 equiv.) and phenylboronic acid (73 mg, 0.60 mmol, 2.0 equiv.), N-(3-(4-bromophenyl)-3-phenylpropyl)-N-(phenylsulfonyl)benzenesulfonamide (**7r**) was obtained as a colorless wax (84 mg, 0.15 mmol, 49%).

R_f (Hex:AcOEt = 8:2) = 0.28.
^{1}H NMR (400 MHz, $CDCl_3$) δ 7.88 (dd, *J* = 8.4, 1.3 Hz, 4H, A*rH*), 7.71 – 7.59 (m, 2H, A*rH*), 7.51 (t, *J* = 7.8 Hz, 4H, A*rH*), 7.46 – 7.41 (m, 2H, A*rH*), 7.31 (t, *J* = 7.4 Hz, 2H, A*rH*), 7.25 – 7.15 (m, 3H, A*rH*), 7.12 – 7.05 (m, 2H, A*rH*), 3.84 (t, *J* = 7.9 Hz, 1H, ArC*H*), 3.55 (td, *J* = 6.9, 2.7 Hz, 2H, NC*H_2*), 2.47 (dddd, *J* = 12.6, 10.6, 7.9, 4.4 Hz, 2H, CH_2C*H_2*).
^{13}C NMR (101 MHz, $CDCl_3$) δ 142.8, 142.6, 139.6, 134.0, 131.9, 129.6, 129.2, 128.9, 128.3, 127.8, 127.0, 120.6, 48.4, 47.9, 35.3.
IR (ν_{max}, cm^{-1}) 1486 (m), 1448 (m), 1372 (s), 1165 (s), 1084 (m), 1010 (m), 872 (s), 786 (m), 736 (s).
HRMS (ESI/QTOF) m/z: [M + Na]$^+$ Calcd for $C_{27}H_{24}BrNNaO_4S_2^+$ 592.0222; Found 592.0237.

N-(3-Phenyl-3-(2-(trifluoromethyl)phenyl)propyl)-N-(phenylsulfonyl)benzenesulfonamide (*o*-**7s**)

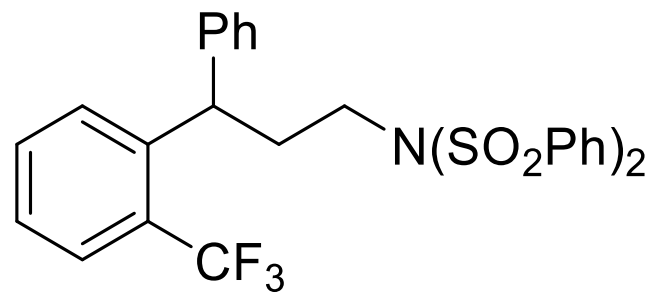

Following the general procedure H using cyclopropylbenzene **5a** (39 µL, 0.30 mmol, 1.0 equiv.) and 2-(trifluoromethyl)phenylboronic acid (114 mg, 0.60 mmol, 2.0 equiv.), N-(3-phenyl-3-(2-(trifluoromethyl)phenyl)propyl)-N-(phenylsulfonyl)benzenesulfonamide (*o*-**7s**) was obtained as a colorless wax (55 mg, 0.10 mmol, 33%).

R_f (Hex:AcOEt = 8:2) = 0.34.
^{1}H NMR (400 MHz, $CDCl_3$) δ 7.92 (d, *J* = 7.8 Hz, 4H, Ar*H*), 7.66 (dd, *J* = 11.8, 7.7 Hz, 3H, Ar*H*), 7.53 (t, *J* = 7.7 Hz, 5H, Ar*H*), 7.44 (d, *J* = 7.9 Hz, 1H, Ar*H*), 7.39 – 7.22 (m, 6H, Ar*H*), 4.40 (t, *J* = 7.8 Hz, 1H, ArC*H*), 3.71 (ddd, *J* = 16.4, 12.1, 4.6 Hz, 1H, NCH_2), 3.50 (ddd, *J* = 15.5, 12.1, 4.6 Hz, 1H, NCH_2), 2.70 – 2.55 (m, 1H, CH_2CH_2), 2.45 (dd, *J* = 12.9, 7.7 Hz, 1H, CH_2CH_2).
^{19}F NMR (376 MHz, $CDCl_3$) δ -57.7.
^{13}C NMR (101 MHz, $CDCl_3$) δ 142.6, 142.0, 139.6, 134.0, 132.4, 129.7, 129.2, 128.8, 128.3, 128.0, 127.0, 126.7, 126.0 (q, *J* = 6.1 Hz), 124.7 (d, *J* = 274.3 Hz), 47.9, 43.3, 36.2.
One carbon not resolved.
IR (v_{max}, cm^{-1}) 1450 (m), 1375 (s), 1312 (s), 1166 (s), 1118 (s), 1087 (m), 1035 (m), 871 (m), 743 (s).
HRMS (nanochip-ESI/LTQ-Orbitrap) m/z: $[M + Na]^+$ Calcd for $C_{28}H_{24}F_3NNaO_4S_2^+$ 582.0991; Found 582.0999.

N-(3-Phenyl-3-(3-(trifluoromethyl)phenyl)propyl)-N-(phenylsulfonyl)benzenesulfonamide (*m*-**7s**)

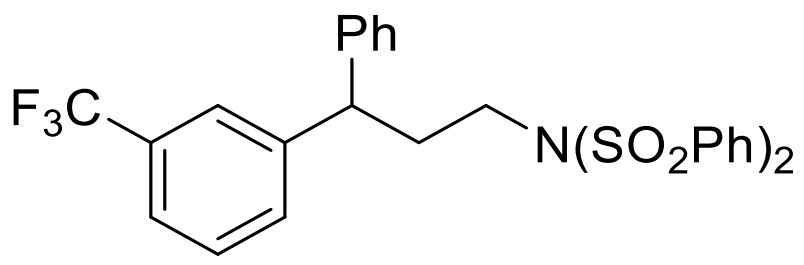

Following the general procedure H using cyclopropylbenzene **5a** (39 µL, 0.30 mmol, 1.0 equiv.) and 3-(trifluoromethyl)phenylboronic acid (114 mg, 0.60 mmol, 2.0 equiv.), N-(3-phenyl-3-(3-(trifluoromethyl)phenyl)propyl)-N-(phenylsulfonyl)benzenesulfonamide (*o*-**7s**) was obtained as a colorless wax (55 mg, 0.10 mmol, 33%).

R_f (Hex:AcOEt = 8:2) = 0.34.
^{1}H NMR (400 MHz, $CDCl_3$) δ 7.84 – 7.75 (m, 4H, Ar*H*), 7.58 – 7.50 (m, 2H, Ar*H*), 7.46 – 7.36 (m, 6H, Ar*H*), 7.32 (dd, *J* = 4.8, 2.0 Hz, 2H, Ar*H*), 7.24 (td, *J* = 7.5, 1.6 Hz, 2H, Ar*H*), 7.19 – 7.09 (m, 3H, Ar*H*), 3.86 (t, *J* = 7.9 Hz, 1H, ArC*H*), 3.50 (ddd, *J* = 8.9, 6.4, 1.5 Hz, 2H, NCH_2), 2.52 – 2.31 (m, 2H, CH_2CH_2).
^{19}F NMR (376 MHz, $CDCl_3$) δ -62.4.

^{13}**C NMR** (101 MHz, $CDCl_3$) δ 144.6, 142.4, 139.6, 134.1, 131.3, 131.0 (q, *J* = 32.0 Hz), 129.3, 129.0, 128.2, 127.8, 127.1, 124.4 (q, *J* = 3.8 Hz), 124.2 (q, *J* = 272.4 Hz), 123.7 (q, *J* = 3.8 Hz), 48.8, 47.9, 35.3.
One carbon not resolved.
IR (v_{max}, cm^{-1}) 1449 (m), 1373 (s), 1327 (s), 1164 (s), 1123 (s), 1074 (s), 866 (m), 739 (s).
HRMS (ESI/QTOF) m/z: $[M + Na]^+$ Calcd for $C_{28}H_{24}F_3NNaO_4S_2^+$ 582.0991; Found 582.0998.

N-(3-Phenyl-3-(4-(trifluoromethyl)phenyl)propyl)-N-(phenylsulfonyl)benzenesulfonamide (*p*-**7s**)

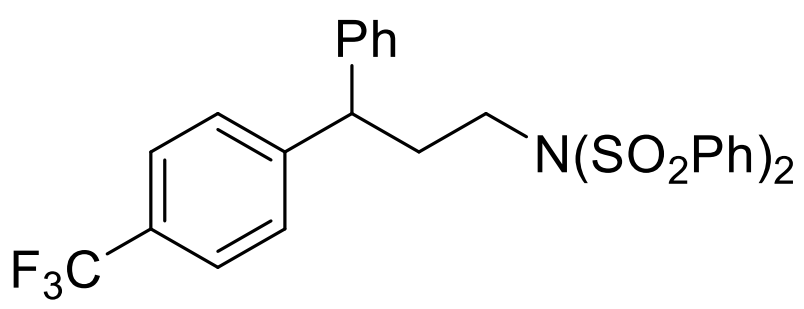

Following the general procedure H using *p*-**5i** (45 mg, 0.30 mmol, 1.0 equiv.) and phenylboronic acid (73 mg, 0.60 mmol, 2.0 equiv.), N-(3-phenyl-3-(4-(trifluoromethyl)phenyl)propyl)-N-(phenylsulfonyl)benzenesulfonamide (*p*-**7s**) was obtained as a colorless wax (53 mg, 0.09 mmol, 32%).

Following the general procedure H using cyclopropylbenzene **5a** (39 µL, 0.30 mmol, 1.0 equiv.) and 4-(trifluoromethyl)phenylboronic acid (114 mg, 0.60 mmol, 2.0 equiv.), N-(3-phenyl-3-(4-(trifluoromethyl)phenyl)propyl)-N-(phenylsulfonyl)benzenesulfonamide (*p*-**7t**) was obtained as a colorless wax (78 mg, 0.14 mmol, 46%).

$\mathbf{R_f}$ (Hex:AcOEt = 8:2) = 0.34.
^{1}**H NMR** (400 MHz, $CDCl_3$) δ 7.96 – 7.76 (m, 4H, Ar*H*), 7.63 – 7.55 (m, 2H, Ar*H*), 7.46 (dd, *J* = 8.5, 7.2 Hz, 4H, Ar*H*), 7.33 – 7.26 (m, 2H, Ar*H*), 7.24 – 7.08 (m, 7H, Ar*H*), 3.86 (t, *J* = 8.0 Hz, 1H, ArC*H*), 3.59 – 3.44 (m, 2H, NCH_2), 2.46 (dddd, *J* = 9.6, 8.3, 6.6, 1.5 Hz, 2H, CH_2CH_2).
^{19}**F NMR** (376 MHz, $CDCl_3$) δ -57.9.
^{13}**C NMR** (101 MHz, $CDCl_3$) δ 147.9 (d, *J* = 1.9 Hz), 142.8, 142.3, 139.6, 134.0, 129.2, 129.2, 129.0, 128.3, 127.8, 127.0, 121.3, 120.6 (q, *J* = 256.8 Hz), 48.4, 47.9, 35.5.
IR (v_{max}, cm^{-1}) 1507 (w), 1450 (m), 1373 (m), 1258 (s), 1221 (s), 1163 (s), 1086 (m), 873 (m), 739 (s).
HRMS (ESI/QTOF) m/z: $[M + Na]^+$ Calcd for $C_{28}H_{24}F_3NNaO_4S_2^+$ 582.0991; Found 582.0987.

N-(3-(2-Acetylphenyl)-3-phenylpropyl)-N-(phenylsulfonyl)benzenesulfonamide (*o*-**7t**)

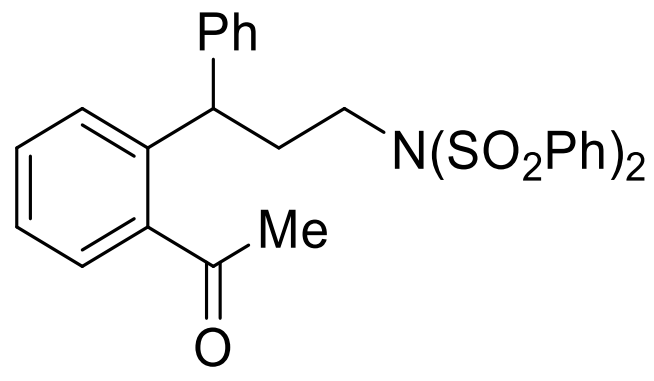

Following the general procedure H using *o*-**5h** (50 mg, 0.30 mmol, 1.0 equiv.) and phenylboronic acid (73 mg, 0.60 mmol, 2.0 equiv.), N-(3-(4-acetylphenyl)-3-phenylpropyl)-N-(phenylsulfonyl)benzenesulfonamide (*o*-**7t**) was obtained as a colorless wax (67 mg, 0.13 mmol, 42%).

R$_f$ (Hex:AcOEt = 8:2) = 0.27.
^{1}H NMR (400 MHz, $CDCl_3$) δ 7.93 – 7.84 (m, 4H, A*rH*), 7.65 – 7.59 (m, 2H, A*rH*), 7.56 – 7.47 (m, 6H, A*rH*), 7.41 (ddd, *J* = 17.9, 7.6, 1.5 Hz, 2H, A*rH*), 7.31 – 7.27 (m, 2H, A*rH*), 7.23 – 7.14 (m, 3H, A*rH*), 4.75 (t, *J* = 7.7 Hz, 1H, ArC*H*), 3.67 – 3.57 (m, 2H, NC*H$_2$*), 2.47 (dt, *J* = 9.9, 7.8 Hz, 2H, CH_2C*H$_2$*), 2.41 (s, 3H, COC*H$_3$*).
^{13}C NMR (101 MHz, $CDCl_3$) δ 203.4, 143.2, 142.7, 139.7, 139.1, 134.0, 133.9, 131.5, 129.2, 129.2, 128.6, 128.5, 128.5, 128.4, 128.3, 126.6, 126.3, 48.1, 42.8, 35.8, 30.4.
IR (ν_{max}, cm^{-1}) 1686 (w), 1509 (w), 1450 (m), 1375 (s), 1169 (s), 1088 (m), 872 (m), 755 (s).
HRMS (ESI/QTOF) m/z: $[M + Na]^+$ Calcd for $C_{29}H_{27}NNaO_5S_2^+$ 556.1223; Found 556.1223.

N-(3-(4-Acetylphenyl)-3-phenylpropyl)-N-(phenylsulfonyl)benzenesulfonamide (*p*-**7t**)

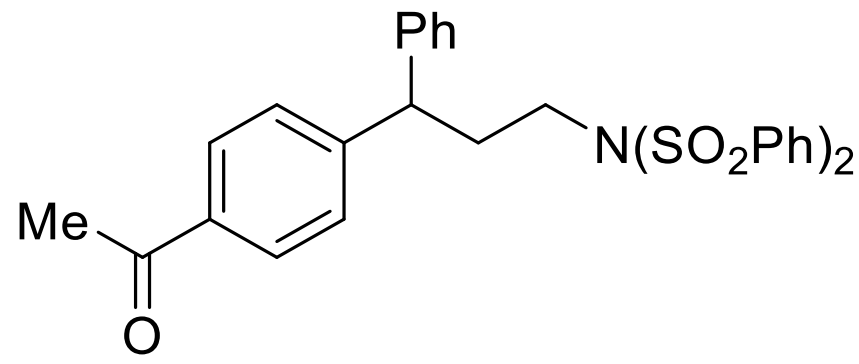

Following the general procedure H using *p*-**5h** (50 mg, 0.30 mmol, 1.0 equiv.) and phenylboronic acid (73 mg, 0.60 mmol, 2.0 equiv.), N-(3-(4-acetylphenyl)-3-phenylpropyl)-N-(phenylsulfonyl)benzenesulfonamide (*p*-**7t**) was obtained as a colorless wax (41 mg, 0.080 mmol, 26%).

R$_f$ (Hex:AcOEt = 8:2) = 0.27.
^{1}H NMR (400 MHz, $CDCl_3$) δ 7.87 – 7.77 (m, 6H, A*rH*), 7.59 – 7.53 (m, 2H, A*rH*), 7.43 (dd, *J* = 8.5, 7.3 Hz, 4H, A*rH*), 7.28 – 7.21 (m, 4H, A*rH*), 7.20 – 7.08 (m, 3H, A*rH*), 3.88 (t, *J* = 7.9 Hz, 1H, ArC*H*), 3.56 – 3.46 (m, 2H, NC*H$_2$*), 2.51 (s, 3H, COC*H$_3$*), 2.44 (tdd, *J* = 8.0, 6.6, 3.5 Hz, 2H, CH_2C*H$_2$*).
^{13}C NMR (101 MHz, $CDCl_3$) δ 198.1, 149.2, 142.4, 139.6, 135.7, 134.1, 129.3, 129.0, 129.0, 128.3, 128.1, 127.9, 127.1, 49.0, 48.0, 35.2, 26.7.
IR (ν_{max}, cm^{-1}) 1681 (m), 1604 (m), 1449 (m), 1370 (s), 1268 (m), 1166 (s), 1085 (m), 872 (m), 790 (m), 735 (s).

HRMS (nanochip-ESI/LTQ-Orbitrap) m/z: [M + Na]$^+$ Calcd for $C_{29}H_{27}NNaO_5S_2^+$ 556.1223; Found 556.1233.

N-(3-(3,5-Dimethylphenyl)-3-phenylpropyl)-N-(phenylsulfonyl)benzenesulfonamide (**7u**)

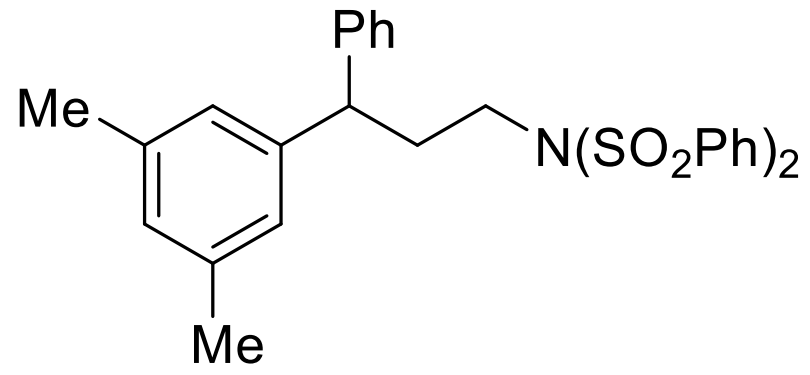

Following the general procedure H using **5t** (45 mg, 0.30 mmol, 1.0 equiv.) and phenylboronic acid (73 mg, 0.60 mmol, 2.0 equiv.), N-(3-(3,5-dimethylphenyl)-3-phenylpropyl)-N-(phenylsulfonyl)benzenesulfonamide (**7u**) was obtained as a colorless wax (63 mg, 0.12 mmol, 40%).

R$_f$ (Hex:AcOEt = 8:2) = 0.45.
^{1}H NMR (400 MHz, $CDCl_3$) δ 7.84 – 7.76 (m, 4H, Ar*H*), 7.52 (t, *J* = 7.5 Hz, 2H, Ar*H*), 7.40 (t, *J* = 7.7 Hz, 4H, Ar*H*), 7.21 (t, *J* = 7.5 Hz, 2H, Ar*H*), 7.12 (dd, *J* = 14.0, 7.3 Hz, 3H, Ar*H*), 6.75 (s, 3H, Ar*H*), 3.71 (t, *J* = 8.0 Hz, 1H, ArC*H*), 3.49 (td, *J* = 7.2, 3.7 Hz, 2H, NCH_2), 2.40 (q, *J* = 8.1 Hz, 2H, CH_2CH_2), 2.20 (s, 6H, CH_3).
^{13}C NMR (101 MHz, $CDCl_3$) δ 143.7, 143.3, 139.7, 138.2, 133.9, 129.2, 128.7, 128.3, 128.3, 127.8, 126.6, 125.7, 49.0, 48.2, 35.5, 21.5.
IR (ν_{max}, cm^{-1}) 1495 (w), 1450 (m), 1371 (s), 1169 (s), 1087 (m), 990 (m), 887 (m), 773 (m), 752 (s), 721 (m).
HRMS (nanochip-ESI/LTQ-Orbitrap) m/z: [M + Na]$^+$ Calcd for $C_{29}H_{29}NNaO_4S_2^+$ 542.1430; Found 542.1447.

N-(Phenylsulfonyl)-N-(1,3,3-triphenylpropyl)benzenesulfonamide (**7v**)

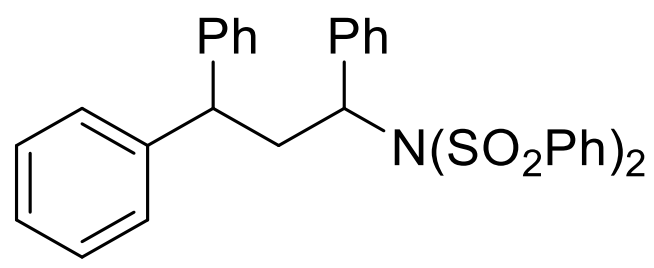

Following the general procedure H using **5s** (60 mg, 0.30 mmol, 1.0 equiv.) and phenylboronic acid (73 mg, 0.60 mmol, 2.0 equiv.), N-(phenylsulfonyl)-N-(1,3,3-triphenylpropyl)benzenesulfonamide (**7v**) was obtained as a colorless wax (90 mg, 0.16 mmol, 53%).

R$_f$ (Hex:AcOEt = 8:2) = 0.42.
^{1}H NMR (400 MHz, $CDCl_3$) δ 7.53 – 7.01 (m, 23H, Ar*H*), 6.80 – 6.61 (m, 2H, Ar*H*), 5.24 (dd, *J* = 12.7, 3.1 Hz, 1H, ArC*H*), 3.76 – 3.55 (m, 2H, CHCH_2, NC*H*), 2.26 – 2.08 (m, 1H, CH_2CH_2).
^{13}C NMR (101 MHz, $CDCl_3$) δ 143.9, 143.3, 134.8, 130.6, 128.9, 128.6, 128.6, 128.5, 128.2, 127.8, 126.9, 126.6, 63.4, 48.1, 38.0.
IR (ν_{max}, cm^{-1}) 1494 (w), 1453 (m), 1369 (s), 1167 (s), 1085 (m), 989 (m), 886 (m),

772 (s), 749 (s).
HRMS (ESI/QTOF) m/z: $[M + Na]^+$ Calcd for $C_{33}H_{29}NNaO_4S_2^+$ 590.1430; Found 590.1432.

Methyl 2-(1-phenyl-3-(N-(phenylsulfonyl)phenylsulfonamido)propyl)benzoate (*o*-**7w**)

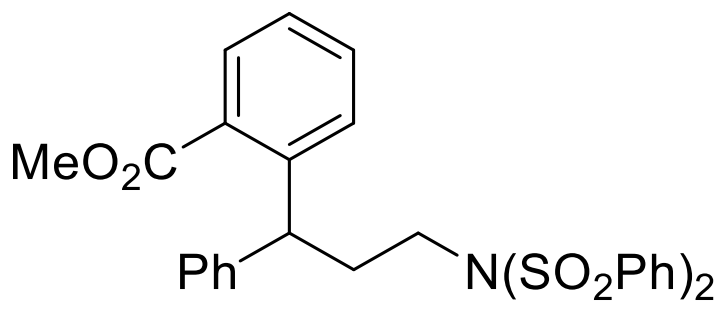

Following the general procedure H using cyclopropylbenzene **5a** (39 µL, 0.30 mmol, 1.0 equiv.) and 2-methoxycarbonylphenylboronic acid (108 mg, 0.60 mmol, 2.0 equiv.), methyl 2-(1-phenyl-3-(N-(phenylsulfonyl)phenylsulfonamido)propyl)benzoate (*o*-**7w**) was obtained as a colorless wax (90 mg, 0.16 mmol, 55%).

R_f (Hex:AcOEt = 8:2) = 0.28.
^{1}H NMR (400 MHz, $CDCl_3$) δ 7.87 – 7.78 (m, 4H, Ar*H*), 7.72 (dd, *J* = 7.8, 1.5 Hz, 1H, Ar*H*), 7.57 – 7.48 (m, 2H, Ar*H*), 7.46 – 7.35 (m, 5H, Ar*H*), 7.28 – 7.10 (m, 7H, Ar*H*), 4.91 (t, *J* = 7.7 Hz, 1H, ArC*H*), 3.81 (s, 3H, COOCH_3), 3.64 – 3.45 (m, 2H, NCH_2), 2.51 – 2.33 (m, 2H, CH_2CH_2).
^{13}C NMR (101 MHz, $CDCl_3$) δ 168.5, 144.6, 143.0, 139.7, 133.9, 132.2, 130.5, 130.3, 129.2, 128.6, 128.5, 128.3, 128.3, 126.6, 126.4, 52.3, 48.1, 43.2, 35.9.
IR (ν_{max}, cm^{-1}) 1720 (s), 1450 (m), 1374 (s), 1266 (m), 1169 (s), 1087 (m), 871 (m), 750 (s).
HRMS (ESI/QTOF) m/z: $[M + Na]^+$ Calcd for $C_{29}H_{27}NNaO_6S_2^+$ 572.1172; Found 572.1172.

Methyl 3-(1-phenyl-3-(N-(phenylsulfonyl)phenylsulfonamido)propyl)benzoate (*m*-**7w**)

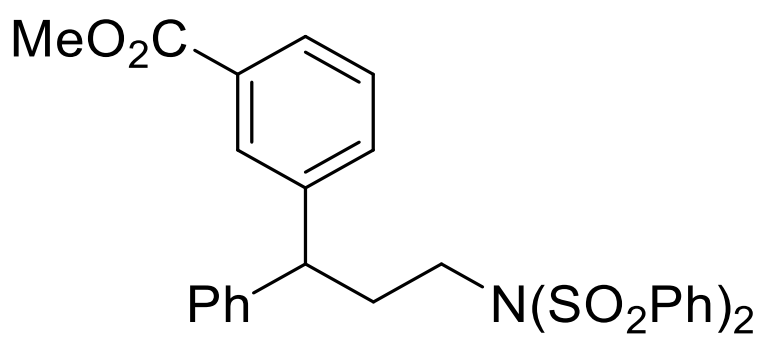

Following the general procedure H using cyclopropylbenzene **5a** (39 µL, 0.30 mmol, 1.0 equiv.) and 2-methoxycarbonylphenylboronic acid (108 mg, 0.60 mmol, 2.0 equiv.), methyl 3-(1-phenyl-3-(N-(phenylsulfonyl)phenylsulfonamido)propyl)benzoate (*m*-**7w**) was obtained as a colorless wax (40 mg, 0.070 mmol, 24%).

R_f (Hex:AcOEt = 8:2) = 0.28.
^{1}H NMR (400 MHz, $CDCl_3$) δ 7.95 – 7.81 (m, 6H, Ar*H*), 7.68 – 7.57 (m, 2H, Ar*H*), 7.50 (t, *J* = 7.7 Hz, 4H, Ar*H*), 7.44 – 7.27 (m, 4H, Ar*H*), 7.25 – 7.17 (m, 3H, Ar*H*), 3.92 (m, 4H, ArC*H*, COOCH_3), 3.58 (t, *J* = 8.1 Hz, 2H, NCH_2), 2.64 – 2.41 (m, 2H, CH_2CH_2).
^{13}C NMR (101 MHz, $CDCl_3$) δ 167.1, 144.0, 142.8, 139.7, 134.0, 132.4, 130.6, 129.2, 128.9, 128.9, 128.3, 128.2, 128.1, 127.8, 127.0, 52.3, 48.9, 48.0, 35.3.
IR (ν_{max}, cm^{-1}) 1721 (w), 1450 (m), 1374 (s), 1168 (s), 1086 (m), 872 (m), 753 (s).

HRMS (ESI/QTOF) m/z: $[M + Na]^+$ Calcd for $C_{29}H_{27}NNaO_6S_2^+$ 572.1172; Found 572.1175.

Methyl 4-(1-phenyl-3-(N-(phenylsulfonyl)phenylsulfonamido)propyl)benzoate (*p*-**7w**)

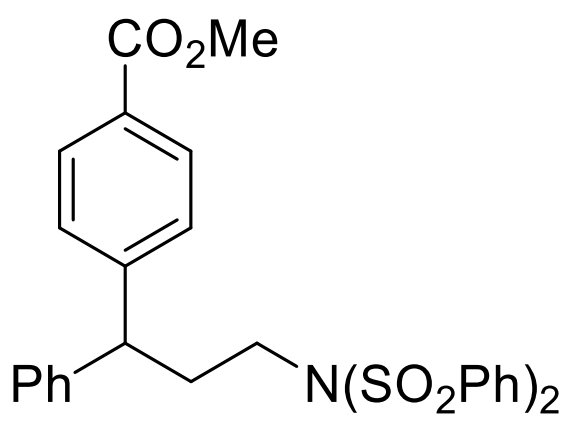

Following the general procedure H using cyclopropylbenzene **5a** (39 µL, 0.30 mmol, 1.0 equiv.) and 4-methoxycarbonylphenylboronic acid (108 mg, 0.60 mmol, 2.0 equiv.), methyl 4-(1-phenyl-3-(N-(phenylsulfonyl)phenylsulfonamido)propyl)benzoate (*p*-**7w**) was obtained as a colorless wax (69 mg, 0.12 mmol, 42%).

$\mathbf{R_f}$ (Hex:AcOEt = 8:2) = 0.28.
^{1}H NMR (400 MHz, $CDCl_3$) δ 7.94 (d, *J* = 8.2 Hz, 2H, Ar*H*), 7.89 – 7.80 (m, 4H, Ar*H*), 7.63 – 7.55 (m, 2H, Ar*H*), 7.47 (t, *J* = 7.8 Hz, 4H, Ar*H*), 7.33 – 7.13 (m, 7H, Ar*H*), 3.91 (t, *J* = 8.0 Hz, 1H, ArC*H*), 3.87 (s, 3H, COOCH_3), 3.55 (dd, *J* = 9.4, 6.9 Hz, 2H, NCH_2), 2.56 – 2.39 (m, 2H, CH_2CH_2).
^{13}C NMR (101 MHz, $CDCl_3$) δ 166.9, 148.7, 142.4, 139.5, 133.9, 130.0, 129.1, 128.8, 128.6, 128.1, 127.8, 127.8, 126.9, 52.1, 48.9, 47.8, 35.1.
IR (ν_{max}, cm^{-1}) 1721 (s), 1449 (m), 1372 (s), 1282 (s), 1169 (s), 1112 (m), 1088 (m), 875 (m), 753 (s).
HRMS (ESI/QTOF) m/z: $[M + Na]^+$ Calcd for $C_{29}H_{27}NNaO_6S_2^+$ 572.1172; Found 572.1176.

N-(3-Phenyl-3-(2-(trifluoromethoxy)phenyl)propyl)-N-(phenylsulfonyl)benzenesulfonamide (*o*-**7x**)

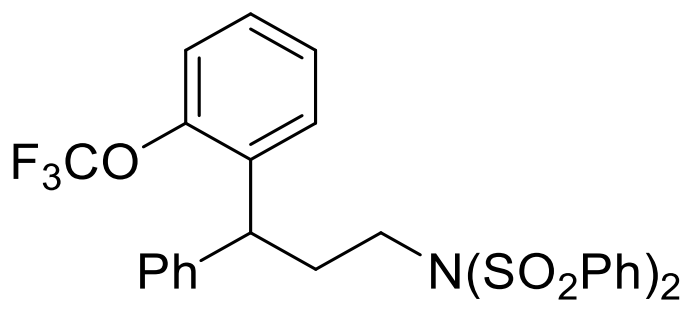

Following the general procedure H using cyclopropylbenzene **5a** (39 µL, 0.30 mmol, 1.0 equiv.) and 2-(trifluoromethoxy)phenylboronic acid (124 mg, 0.60 mmol, 2.0 equiv.), N-(3-phenyl-3-(2-(trifluoromethoxy)phenyl)propyl)-N-(phenylsulfonyl)benzenesulfonamide (*o*-**7x**) was obtained as a colorless wax (39 mg, 0.070 mmol, 23%).

$\mathbf{R_f}$ (Hex:AcOEt = 8:2) = 0.36.
^{1}H NMR (400 MHz, $CDCl_3$) δ 7.89 – 7.73 (m, 4H, Ar*H*), 7.63 – 7.52 (m, 2H, Ar*H*), 7.49 – 7.38 (m, 4H, Ar*H*), 7.31 – 7.26 (m, 1H, Ar*H*), 7.26 – 7.10 (m, 8H, Ar*H*), 4.21 (t, *J* = 7.9 Hz, 1H, ArC*H*), 3.51 (t, *J* = 8.3 Hz, 2H, NCH_2), 2.59 – 2.28 (m, 2H, CH_2CH_2).
^{19}F NMR (376 MHz, $CDCl_3$) δ -56.5.

^{13}C NMR (101 MHz, $CDCl_3$) δ 147.2 (d, J = 1.6 Hz), 142.0, 139.7, 135.7, 134.0, 129.2, 128.8, 128.7, 128.3, 128.0, 127.1, 126.9, 120.7 (q, J = 257.9 Hz), 120.3 (d, J = 1.9 Hz), 47.9, 41.4, 35.0.
One carbon not resolved.
IR (ν_{max}, cm^{-1}) 1451 (m), 1376 (m), 1260 (s), 1216 (s), 1169 (s), 1087 (m), 881 (m), 756 (m).
HRMS (ESI/QTOF) m/z: $[M + Na]^+$ Calcd for $C_{28}H_{24}F_3NNaO_5S_2^+$ 598.0940; Found 598.0939.

N-(3-Phenyl-3-(3-(trifluoromethoxy)phenyl)propyl)-N-(phenylsulfonyl)benzenesulfonamide (*m*-**7x**)

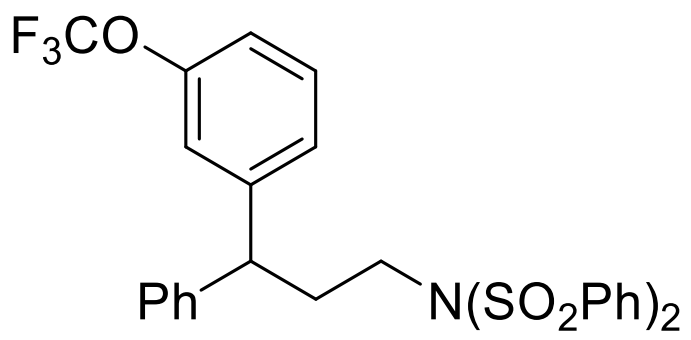

Following the general procedure H using cyclopropylbenzene **5a** (39 µL, 0.30 mmol, 1.0 equiv.) and 3-(trifluoromethoxy)phenylboronic acid (124 mg, 0.60 mmol, 2.0 equiv.), N-(3-phenyl-3-(3-(trifluoromethoxy)phenyl)propyl)-N-(phenylsulfonyl)benzenesulfonamide (*m*-**7x**) was obtained as a colorless wax (71 mg, 0.12 mmol, 41%).

R_f (Hex:AcOEt = 8:2) = 0.36.
^{1}H NMR (400 MHz, $CDCl_3$) δ 7.81 (dt, J = 8.4, 1.2 Hz, 4H, Ar*H*), 7.57 – 7.50 (m, 2H, Ar*H*), 7.41 (dd, J = 8.5, 7.2 Hz, 4H, Ar*H*), 7.23 (tdd, J = 7.9, 3.6, 2.5 Hz, 3H, Ar*H*), 7.18 – 7.08 (m, 3H, Ar*H*), 7.06 (dt, J = 7.9, 1.5 Hz, 1H, Ar*H*), 7.01 – 6.92 (m, 2H, Ar*H*), 3.81 (t, J = 8.0 Hz, 1H, ArC*H*), 3.49 (td, J = 8.2, 2.2 Hz, 2H, NC*H₂*), 2.39 (ttd, J = 11.4, 5.8, 2.9 Hz, 2H, CH_2CH_2).
^{19}F NMR (376 MHz, $CDCl_3$) δ -57.6.
^{13}C NMR (101 MHz, $CDCl_3$) δ 149.6 (d, J = 1.8 Hz), 146.0, 142.4, 139.7, 134.0, 130.1, 129.2, 129.0, 128.3, 127.8, 127.1, 126.2, 120.6 (q, J = 257.2 Hz), 120.4, 119.0, 48.7, 47.9, 35.3.
IR (ν_{max}, cm^{-1}) 1450 (m), 1376 (m), 1260 (s), 1217 (s), 1167 (s), 1087 (m), 877 (m), 752 (m).
HRMS (ESI/QTOF) m/z: $[M + Na]^+$ Calcd for $C_{28}H_{24}F_3NNaO_5S_2^+$ 598.0940; Found 598.0942.

N-(3-Phenyl-3-(4-(trifluoromethoxy)phenyl)propyl)-N-(phenylsulfonyl)benzenesulfonamide (*p*-**7x**)

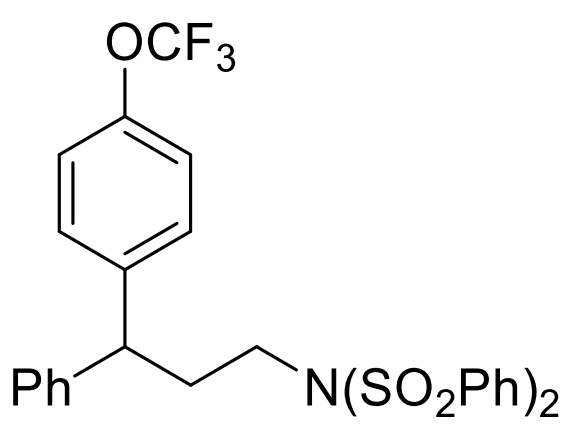

Following the general procedure H using cyclopropylbenzene **5a** (39 µL, 0.30 mmol, 1.0 equiv.) and 4-(trifluoromethoxy)phenylboronic acid (124 mg, 0.60 mmol, 2.0 equiv.), N-(3-phenyl-3-(4-(trifluoromethoxy)phenyl)propyl)-N-(phenylsulfonyl)benzenesulfonamide (*p*-**7x**) was obtained as a colorless wax (81 mg, 0.14 mmol, 47%).

R$_f$ (Hex:AcOEt = 8:2) = 0.36.
^{1}H NMR (400 MHz, $CDCl_3$) δ 7.83 – 7.76 (m, 4H, Ar*H*), 7.57 – 7.50 (m, 2H, Ar*H*), 7.44 – 7.37 (m, 4H, Ar*H*), 7.27 – 7.20 (m, 2H, Ar*H*), 7.18 – 7.09 (m, 5H, Ar*H*), 7.09 – 7.03 (m, 2H, Ar*H*), 3.81 (t, *J* = 7.9 Hz, 1H, ArC*H*), 3.62 – 3.38 (m, 2H, NCH_2), 2.42 (dddd, *J* = 9.7, 8.3, 6.6, 1.5 Hz, 2H, CH_2CH_2).
^{19}F NMR (376 MHz, $CDCl_3$) δ -57.8.
^{13}C NMR (101 MHz, $CDCl_3$) δ 147.9 (q, *J* = 1.9 Hz), 142.8, 142.3, 139.6, 134.0, 129.2, 129.2, 128.9, 128.3, 127.8, 127.0, 121.3, 120.6 (q, *J* = 256.8 Hz), 48.3, 47.9, 35.5.
IR (ν_{max}, cm^{-1}) 1508 (w), 1454 (w), 1376 (m), 1262 (s), 1222 (m), 1166 (s), 1088 (m), 875 (m), 754 (m).
HRMS (ESI/QTOF) m/z: [M + Na]$^+$ Calcd for $C_{28}H_{24}F_3NNaO_5S_2^+$ 598.0940; Found 598.0944.

N-(3-(2-Fluoropyridin-3-yl)-3-phenylpropyl)-N-(phenylsulfonyl)benzenesulfonamide (**7y**)

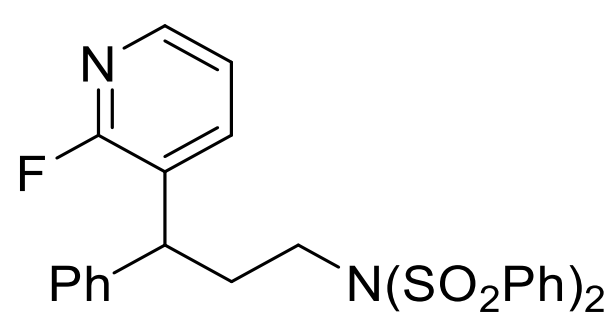

Following the general procedure H using cyclopropylbenzene **5a** (39 µL, 0.30 mmol, 1.0 equiv.) and (2-fluoropyridin-3-yl)boronic acid (84 mg, 0.60 mmol, 2.0 equiv.), N-(3-(2-fluoropyridin-3-yl)-3-phenylpropyl)-N-(phenylsulfonyl)benzenesulfonamide (**7y**) was obtained as a colorless wax (33 mg, 0.060 mmol, 22%).

R$_f$ (Hex:AcOEt = 8:2) = 0.18.
^{1}H NMR (400 MHz, $CDCl_3$) δ 8.10 (dt, *J* = 4.9, 1.5 Hz, 1H, Ar*H*), 7.96 – 7.86 (m, 4H, Ar*H*), 7.74 – 7.61 (m, 3H, Ar*H*), 7.58 – 7.48 (m, 4H, Ar*H*), 7.41 – 7.32 (m, 2H, Ar*H*), 7.32 – 7.23 (m, 4H, Ar*H*), 7.18 (ddd, *J* = 7.4, 4.8, 1.6 Hz, 1H, Ar*H*), 4.17 (t, *J* = 7.9 Hz, 1H, ArC*H*), 3.71 – 3.55 (m, 2H, NCH_2), 2.62 – 2.41 (m, 2H, CH_2CH_2).
^{19}F NMR (376 MHz, $CDCl_3$) δ -71.3.
^{13}C NMR (101 MHz, $CDCl_3$) δ 161.4 (d, *J* = 239.0 Hz), 145.8 (d, *J* = 14.7 Hz), 140.7, 139.6, 139.0 (d, *J* = 5.1 Hz), 134.1, 129.3, 129.1, 128.3, 128.0, 127.4, 125.8 (d, *J* = 29.0 Hz), 121.9 (d, *J* = 4.2 Hz), 47.8, 41.5 (d, *J* = 2.0 Hz), 34.2.
IR (ν_{max}, cm^{-1}) 1444 (m), 1437 (m), 1374 (s), 1170 (s), 1087 (m), 873 (m), 790 (m), 753 (s).
HRMS (nanochip-ESI/LTQ-Orbitrap) m/z: [M + Na]$^+$ Calcd for $C_{26}H_{23}FN_2NaO_4S_2^+$ 533.0975; Found 533.0988.

N-(3-(4-(Bromomethyl)phenyl)-3-phenylpropyl)-N-(phenylsulfonyl)benzenesulfonamide (**7z**)

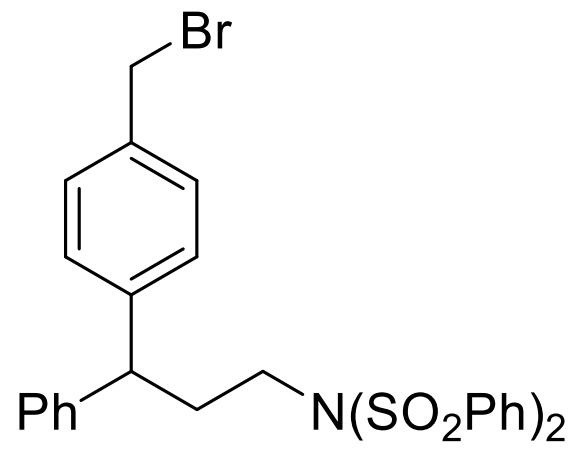

Following the general procedure H using cyclopropylbenzene **5a** (39 µL, 0.30 mmol, 1.0 equiv.) and 4-(bromomethyl)phenylboronic acid (129 mg, 0.60 mmol, 2.0 equiv.), N-(3-(4-(bromomethyl)phenyl)-3-phenylpropyl)-N-(phenylsulfonyl)benzenesulfonamide (**7z**) was obtained as a colorless wax (44 mg, 0.080 mmol, 25%).

R_f (Hex:AcOEt = 8:2) = 0.25.
^{1}H NMR (400 MHz, $CDCl_3$) δ 7.88 – 7.75 (m, 4H, A*rH*), 7.63 – 7.53 (m, 2H, A*rH*), 7.46 – 7.39 (m, 4H, A*rH*), 7.28 – 7.20 (m, 4H, A*rH*), 7.16 – 7.08 (m, 5H, A*rH*), 4.39 (s, 2H), 3.80 (t, *J* = 7.9 Hz, 1H, ArC*H*), 3.48 (td, *J* = 7.4, 3.6 Hz, 2H, NCH_2), 2.42 (dt, *J* = 9.1, 7.6 Hz, 2H, CH_2CH_2).
^{13}C NMR (101 MHz, $CDCl_3$) δ 143.9, 143.0, 139.6, 136.2, 134.0, 129.5, 129.2, 128.9, 128.3, 128.3, 127.8, 126.9, 48.7, 48.0, 35.4, 33.5.
IR (v_{max}, cm^{-1}) 1449 (m), 1372 (s), 1166 (s), 1084 (m), 872 (m), 786 (m), 737 (s).
HRMS (ESI/QTOF) m/z: $[M + Na]^+$ Calcd for $C_{28}H_{26}BrNNaO_4S_2^+$ 606.0379; Found 606.0391.

N-(3-(4-Bromophenyl)-3-(4-(trifluoromethyl)phenyl)propyl)-N-(phenylsulfonyl)benzenesulfonamide (**7aa**),

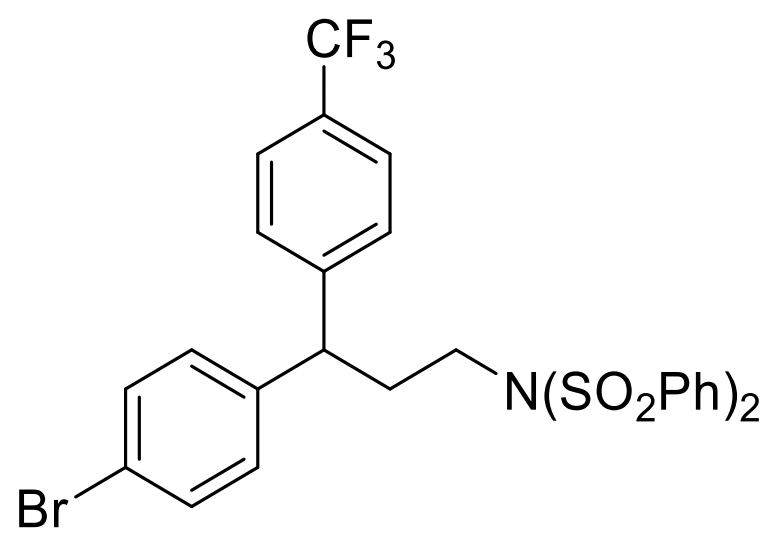

Following the general procedure H using 4-bromo-1-cyclopropylbenzene **5aa** (61 mg, 0.30 mmol, 1.0 equiv.) and phenylboronic acid (73 mg, 0.60 mmol, 2.0 equiv.), N-(3-(4-bromophenyl)-3-phenylpropyl)-N-(phenylsulfonyl)benzenesulfonamide (**7r**) as a colorless wax (135 mg, 0.210 mmol, 70%).

R_f (Hex:AcOEt = 8:2) = 0.48.
^{1}H NMR (400 MHz, $CDCl_3$) δ 7.94 – 7.77 (m, 4H, A*rH*), 7.63 (t, *J* = 7.5 Hz, 2H, A*rH*), 7.57 (d, *J* = 8.0 Hz, 2H, A*rH*), 7.51 (t, *J* = 7.8 Hz, 4H, A*rH*), 7.45 (d, *J* = 8.1 Hz, 2H, A*rH*), 7.31 (d, *J* = 8.0 Hz, 2H, A*rH*), 7.09 (d, *J* = 8.1 Hz, 2H, A*rH*), 3.93 (t, *J* = 7.9 Hz, 1H, ArC*H*), 3.55 (td, *J* = 7.0, 2.3 Hz, 2H, NCH_2), 2.51 (q, *J* = 8.0 Hz, 2H, CH_2CH_2).

^{19}F NMR (376 MHz, $CDCl_3$) δ -62.5.
^{13}C NMR (101 MHz, $CDCl_3$) δ 146.9 (d, *J* = 1.6 Hz), 141.4, 139.4, 134.1, 132.1, 129.6, 129.3, 129.2 (q, *J* = 32.6 Hz), 128.2, 128.2, 125.8 (q, *J* = 3.7 Hz), 123.9 (t, *J* = 272.1 Hz), 121.0, 48.1, 47.6, 35.1.
IR (ν_{max}, cm^{-1}) 1449 (w), 1374 (s), 1325 (s), 1165 (s), 1121 (s), 1069 (s), 1013 (m), 873 (m), 822 (m), 790 (m), 740 (s).
HRMS (ESI/QTOF) m/z: $[M + Na]^+$ Calcd for $C_{28}H_{23}BrF_3NNaO_4S_2^+$ 660.0096; Found 660.0110.

N-(3-(4-Bromophenyl)-3-(4-(trifluoromethoxy)phenyl)propyl)-N-(phenylsulfonyl)benzenesulfonamide (**7ab**),

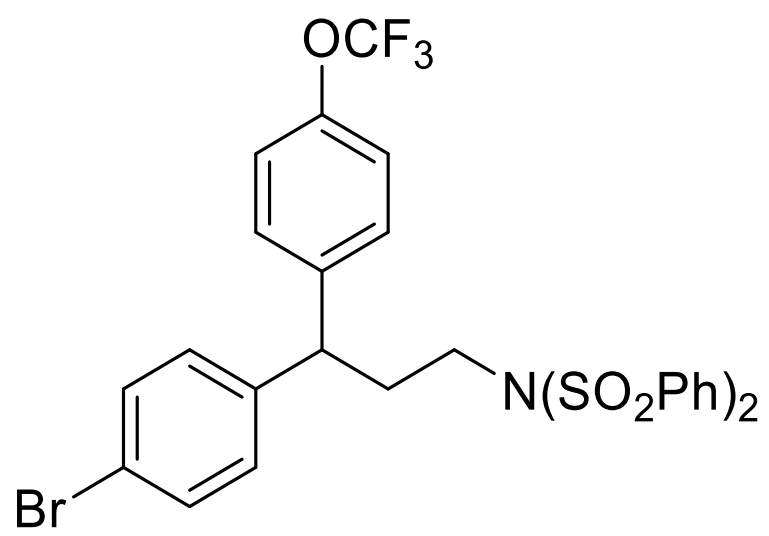

Following the general procedure H using 4-bromo-1-cyclopropylbenzene **5aa** (61 mg, 0.30 mmol, 1.0 equiv.) and phenylboronic acid (73 mg, 0.60 mmol, 2.0 equiv.), N-(3-(4-bromophenyl)-3-phenylpropyl)-N-(phenylsulfonyl)benzenesulfonamide (**7r**) as a colorless wax (105 mg, 0.160 mmol, 53%).

R_f (Hex:AcOEt = 8:2) = 0.48.
^{1}H NMR (400 MHz, $CDCl_3$) δ 7.90 – 7.83 (m, 4H, Ar*H*), 7.67 – 7.58 (m, 2H, Ar*H*), 7.55 – 7.48 (m, 4H, Ar*H*), 7.46 – 7.41 (m, 2H, Ar*H*), 7.23 – 7.17 (m, 2H, Ar*H*), 7.18 – 7.11 (m, 2H, Ar*H*), 7.09 – 7.04 (m, 2H, Ar*H*), 3.86 (t, *J* = 7.9 Hz, 1H, ArC*H*), 3.53 (ddd, *J* = 9.5, 5.8, 1.3 Hz, 2H, NCH_2), 2.46 (q, *J* = 8.1 Hz, 2H, CH_2CH_2).
^{19}F NMR (376 MHz, $CDCl_3$) δ -57.9.
^{13}C NMR (101 MHz, $CDCl_3$) δ 141.8, 141.4, 139.4, 133.9, 131.9, 129.4, 129.1, 128.9, 128.1, 121.3, 120.8, 120.5 (q, *J* = 257.1 Hz), 47.6, 47.6, 35.2.
One carbon not resolved.
IR (ν_{max}, cm^{-1}) 1449 (w), 1375 (m), 1260 (s), 1223 (m), 1167 (s), 1083 (m), 1012 (w), 874 (m), 781 (m), 748 (m).
HRMS (ESI/QTOF) m/z: $[M + Na]^+$ Calcd for $C_{28}H_{23}BrF_3NNaO_5S_2^+$ 676.0045; Found 676.0052.

S11. Ring-Opening of Alkyl-Substituted Cyclopropanes

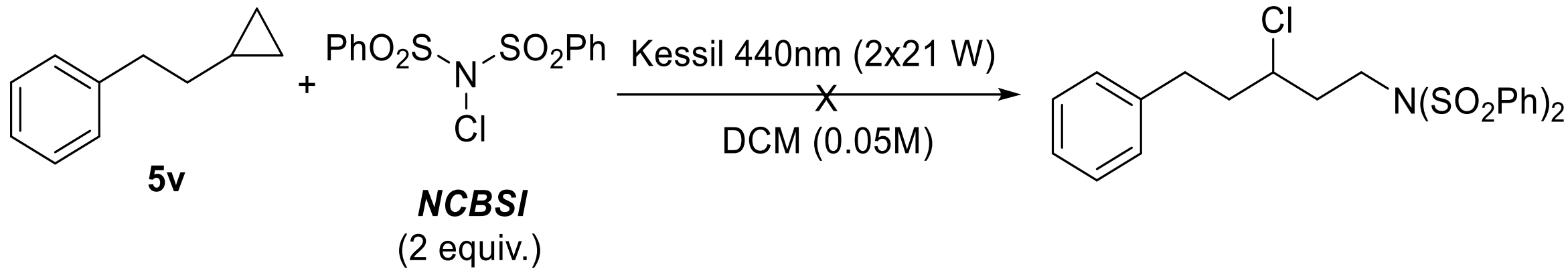

Following the general procedure E using (2-cyclopropylethyl)benzene **5v** (45 mg, 0.30 mmol, 1.0 equiv.), no NMR conversion was observed.

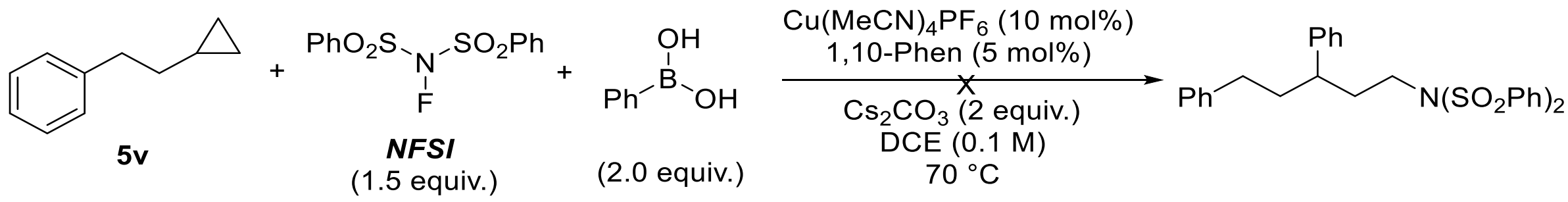

Following the general procedure H using (2-cyclopropylethyl)benzene **5v** (45 mg, 0.30 mmol, 1.0 equiv.), no NMR conversion was observed.

S12. Product modifications

General procedure I

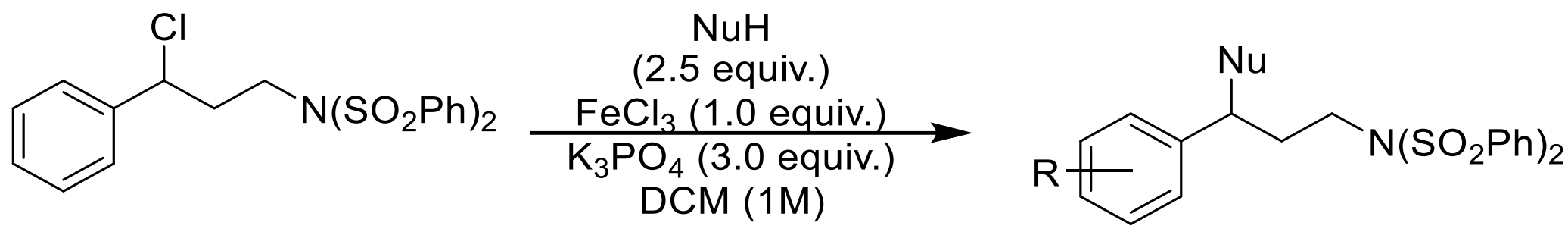

NCBSI (203 mg, 0.600 mmol, 2.0 equiv.) was added into a 15*100 mm borosilicate glass tube. The tube was then closed with a rubber septum and sealed off with parafilm. Three cycles of evacuate-refill with nitrogen were performed to remove O_2. DCM (6 mL, 0.05 M) was added and the solution was stirred under irradiation of (440 nm, 2x21 W) and after few seconds the arylcyclopropane (1.0 equiv.) is added. It is important that the addition of starting material happens few seconds after the irradiation started to ensure reproducibility. The reaction was stirred under irradiation for 8-12 h, then the volume was reduced to half with a nitrogen flush. The resulting solution was added to an oven dry 15*100 mm borosilicate glass tube containing $FeCl_3$ (49 mg, 0.30 mmol, 1.0 equiv.), K_3PO_4 (191 mg, 0.900 mmol, 3.0 equiv.) and the nucleophile (2.5 equiv.). The mixture was stirred at room temperature 16h, then the solvent was removed under reduced pressure and the crude product was purified by chromatography on Biotage (Büchi flashpure cartridge 25 g, Hexane:AcOEt from 10 to 40%) and if needed by reverse phase chromatography on Büchi (BGB Aquarius C18AQ 40g, 100Å, 15 µm, H_2O (0.1% TFA):MeCN(0.1% TFA) from 0 to 100%) to obtain the products.

N-(3-Phenyl-3-(3-phenylpropoxy)propyl)-N-(phenylsulfonyl)benzenesulfonamide (**8a**)

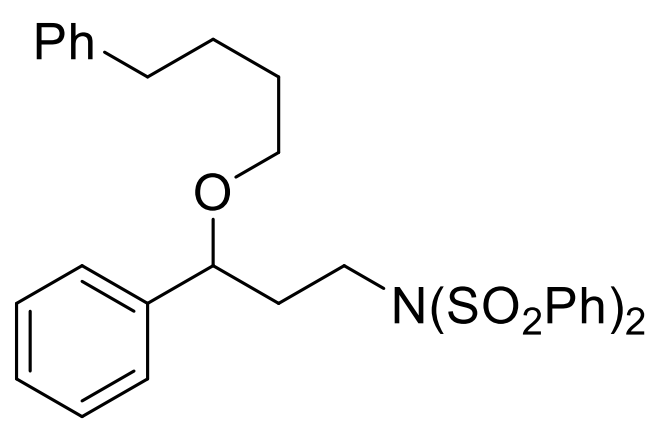

Following the general procedure H using **6a** (135 mg, 0.300 mmol, 1.0 equiv.) and 4-phenylbutan-1-ol (113 mg, 0.750 mmol, 2.5 equiv.), N-(3-phenyl-3-(3-phenylpropoxy)propyl)-N-(phenylsulfonyl)benzenesulfonamide (**8a**) was obtained as a colorless wax (116 mg, 0.200 mmol, 69%).

R_f (Hex:AcOEt = 8:2) = 0.38.
^{1}H NMR (400 MHz, $CDCl_3$) δ 8.02 – 7.95 (m, 4H, A*rH*), 7.70 – 7.61 (m, 2H, A*rH*), 7.52 (t, *J* = 7.9 Hz, 4H, A*rH*), 7.40 – 7.26 (m, 5H, A*rH*), 7.20 (td, *J* = 6.9, 1.8 Hz, 5H, A*rH*), 4.22 (dd, *J* = 7.6, 5.4 Hz, 1H, ArC*H*), 3.90 (ddd, *J* = 14.9, 8.7, 6.0 Hz, 1H, NC*H₂*), 3.78 (ddd, *J* = 15.3, 8.7, 6.7 Hz, 1H, NC*H₂*), 3.41 – 3.20 (m, 2H, OC*H₂*), 2.63 (t, *J* = 7.6 Hz, 2H, CH_2CH_2), 2.19 – 2.02 (m, 2H, ArC*H₂*), 1.79 – 1.67 (m, 2H, $CH_2CH_2CH_2$), 1.67 – 1.55 (m, 2H, $CH_2CH_2CH_2$).
^{13}C NMR (101 MHz, $CDCl_3$) δ 142.6, 141.8, 140.0, 133.9, 129.2, 128.6, 128.6, 128.4,

128.3, 127.8, 126.6, 125.8, 79.4, 68.6, 46.6, 38.2, 35.9, 29.7, 28.3.
IR (vmax, cm-1) 1450 (m), 1374 (s), 1355 (m), 1167 (s), 1088 (s), 1070 (m), 854 (m), 747 (s), 742 (s), 722 (m).
HRMS (ESI/QTOF) m/z: $[M + Na]^+$ Calcd for $C_{31}H_{33}NNaO_5S_2^+$ 586.1692; Found 586.1696.

N-(3-((4-Fluorophenyl)thio)-3-phenylpropyl)-N-(phenylsulfonyl)benzenesulfonamide (**8b**)

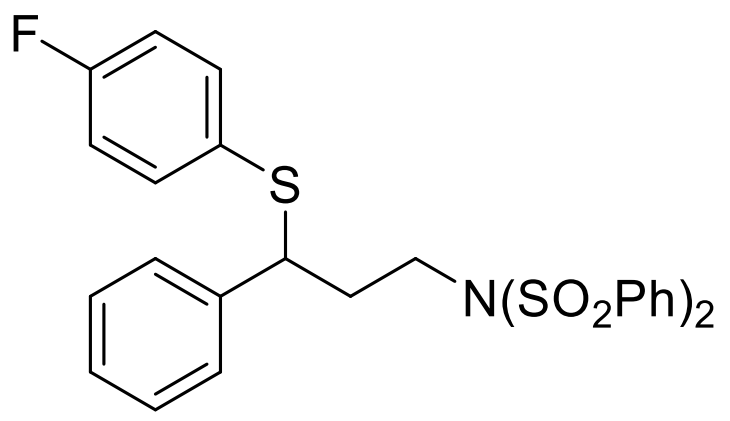

Following the general procedure H using **6a** (135 mg, 0.300 mmol, 1.0 equiv.) and 4-fluorobenzenethiol (96 mg, 0.75 mmol, 2.5 equiv.), N-(3-((4-fluorophenyl)thio)-3-phenylpropyl)-N-(phenylsulfonyl)benzenesulfonamide (**8b**) was obtained as a colorless wax (111 mg, 0.200 mmol, 68%).

R_f (Hex:AcOEt = 8:2) = 0.40.
^{1}H NMR (400 MHz, $CDCl_3$) δ 7.78 (dd, *J* = 8.3, 1.3 Hz, 4H, Ar*H*), 7.58 – 7.45 (m, 2H, Ar*H*), 7.38 (t, *J* = 7.8 Hz, 4H, Ar*H*), 7.03 (s, 5H, Ar*H*), 7.03 – 6.91 (m, 2H, Ar*H*), 6.76 (t, *J* = 8.7 Hz, 2H, Ar*H*), 3.85 (t, *J* = 7.6 Hz, 1H, SC*H*), 3.69 (ddd, *J* = 15.4, 9.8, 5.8 Hz, 1H, NCH_2), 3.44 (ddd, *J* = 15.5, 9.9, 6.1 Hz, 1H, NCH_2), 2.27 – 2.12 (m, 2H, CH_2CH_2).
^{19}F NMR (376 MHz, $CDCl_3$) δ -113.1.
^{13}C NMR (101 MHz, $CDCl_3$) δ 162.9 (d, *J* = 248.3 Hz), 140.3, 139.7, 136.5, 136.4, 134.0, 129.2, 128.6, 128.3, 127.9, 127.7, 115.9 (d, *J* = 21.7 Hz), 51.6 (d, *J* = 1.2 Hz), 47.4, 35.2.
IR (vmax, cm-1) 1588 (w), 1489 (m), 1449 (m), 1373 (s), 1225 (m), 1166 (s), 1086 (m), 1070 (m), 864 (s), 833 (m), 739 (s).
HRMS (ESI/QTOF) m/z: $[M + Na]^+$ Calcd for $C_{27}H_{24}FNNaO_4S_3^+$ 564.0744; Found 564.0743.

N-(3-(Benzylamino)-3-phenylpropyl)-N-(phenylsulfonyl)benzenesulfonamide (**10**)

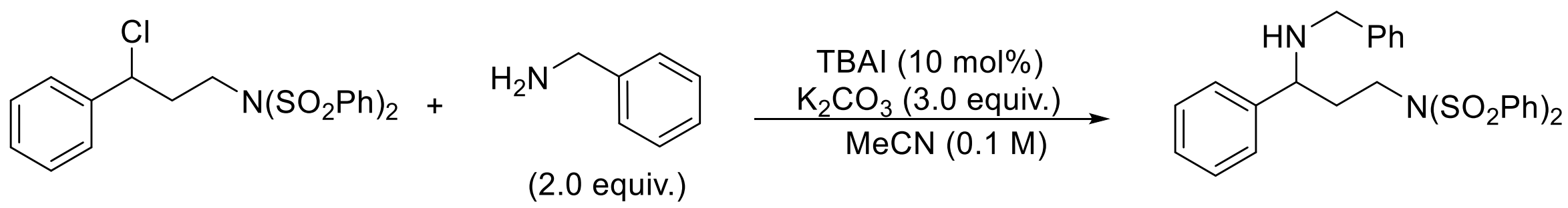

In a 15*100 mm borosilicate glass tube, **6a** (135 mg, 0.300 mmol, 1.0 equiv.), Benzylamine (64 mg, 0.60 mmol, 2.0 equiv.), K_2CO_3 (104 mg, 0.750 mmol, 2.5 equiv.) and TBAI (11 mg, 0.030 mmol, 10 mol%) were dissolved in MeCN (3 mL). The mixture was stirred at room temperature 16h, then the solvents were evaporated under reduced pressure. The crude was purified by chromatography on Biotage (Büchi

flashpure cartridge 25 g, Hexane:AcOEt from 10 to 40%) and N-(3-(benzylamino)-3-phenylpropyl)-N-(phenylsulfonyl)benzenesulfonamide (**10**) was obtained as a colorless wax (127 mg, 0.240 mmol, 81%).

R$_f$ (Hex:AcOEt = 8:2) = 0.22.
^{1}H NMR (400 MHz, $CDCl_3$) δ 7.87 – 7.75 (m, 4H, Ar*H*), 7.59 – 7.49 (m, 2H, Ar*H*), 7.46 – 7.36 (m, 4H, Ar*H*), 7.32 – 7.06 (m, 10H), 3.80 – 3.64 (m, 1H, ArC*H*), 3.59 – 3.32 (m, 4H, NHCH_2, NCH_2), 2.01 (h, *J* = 9.1 Hz, 2H, CH_2CH_2), 1.69 (s, 1H, CH_2N*H*).
^{13}C NMR (101 MHz, $CDCl_3$) δ 139.8, 133.9, 129.2, 129.2, 128.8, 128.5, 128.3, 128.2, 128.2, 127.6, 127.2, 127.1, 59.9, 51.2, 46.7, 37.5.
IR (vmax, cm-1) 1450 (m), 1372 (s), 1357 (m), 1166 (s), 1086 (m), 1067 (m), 854 (m), 732 (s).
HRMS (ESI/QTOF) m/z: [M + H]$^+$ Calcd for $C_{28}H_{29}N_2O_4S_2^+$ 521.1563; Found 521.1561.

N-(3-Phenyl-3-(o-tolyloxy)propyl)benzenesulfonamide (**12**),

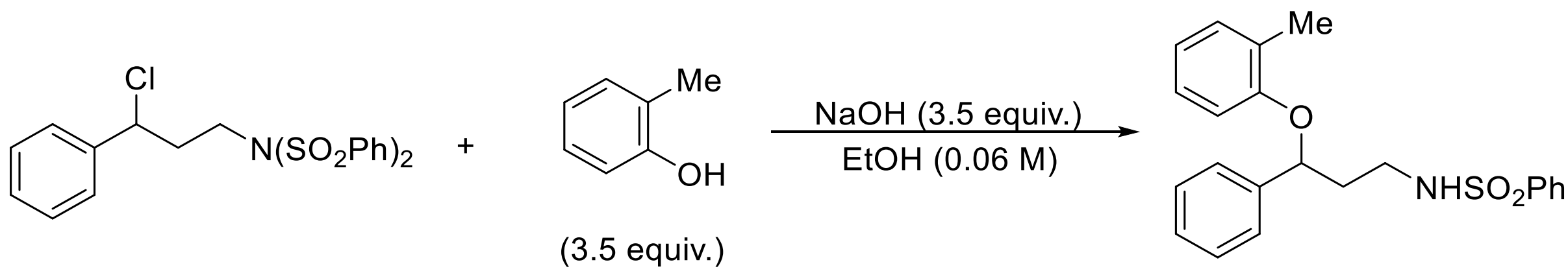

In a 15*100 mm borosilicate glass tube, **6a** (135 mg, 0.300 mmol, 1.0 equiv.), *o*-cresol (114 mg, 1.05 mmol, 3.5 equiv.) and NaOH (42 mg, 1.1 mmol, 3.5 equiv.) were dissolved in EtOH (4.5 mL). The mixture was refluxed 16h, then it was extracted with DCM (20 mL) twice. The combined organic layers were washed with water (40 mL) and with brine (40 mL). The organic layer was dried over Na_2SO_4, filtered and the solvent removed under reduced pressure. The crude was purified by chromatography on Biotage (Büchi flashpure cartridge 25 g, Hexane:AcOEt from 10 to 40%) and N-(3-phenyl-3-(o-tolyloxy)propyl)benzenesulfonamide (**12**) was obtained as a colorless wax (42 mg, 0.11 mmol, 37%).

R$_f$ (Hex:AcOEt = 8:2) = 0.37.
^{1}H NMR (400 MHz, $CDCl_3$) δ 7.90 – 7.81 (m, 2H, Ar*H*), 7.66 – 7.54 (m, 1H, Ar*H*), 7.49 (dd, *J* = 8.4, 7.0 Hz, 2H, Ar*H*), 7.33 – 7.24 (m, 4H, Ar*H*), 7.24 – 7.18 (m, 2H, Ar*H*), 7.17 – 7.09 (m, 1H, Ar*H*), 6.96 (td, *J* = 7.8, 1.8 Hz, 1H, Ar*H*), 6.82 (td, *J* = 7.4, 1.1 Hz, 1H, Ar*H*), 6.52 (d, *J* = 8.2 Hz, 1H, Ar*H*), 5.30 – 5.17 (m, 1H, ArC*H*), 4.93 (s, 1H, CH_2N*H*), 3.21 (q, *J* = 6.1 Hz, 2H, NCH_2), 2.31 (s, 3H, OCH_3), 2.20 – 2.05 (m, 2H, CH_2CH_2).
^{13}C NMR (101 MHz, $CDCl_3$) δ 155.5, 140.7, 139.8, 132.8, 131.0, 129.3, 128.9, 127.9, 127.2, 126.8, 126.8, 125.7, 120.9, 112.8, 78.0, 40.4, 38.0, 16.8.
IR (vmax, cm-1) 1491 (m), 1449 (m), 1373 (m), 1325 (m), 1238 (m), 1164 (s), 1089 (m), 873 (m), 750 (s), 718 (m).

HRMS (ESI/QTOF) m/z: $[M + Na]^+$ Calcd for $C_{22}H_{23}NNaO_3S^+$ 404.1291; Found 404.1283.

N-(3,3-Diphenylpropyl)benzenesulfonamide (**15a**)

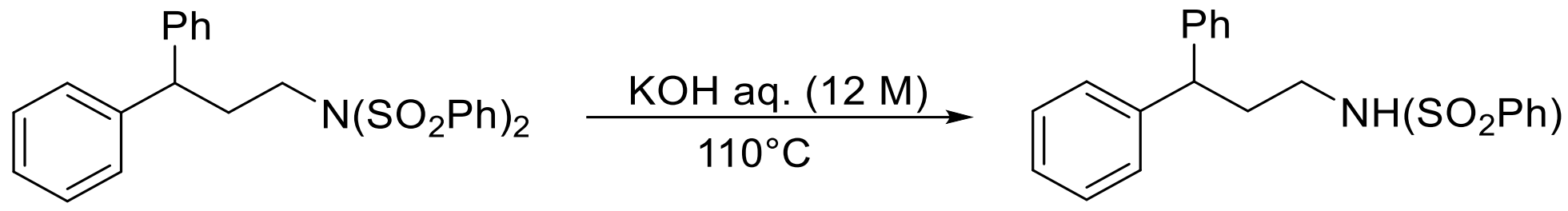

In a 5 mL microwave vial an aqueous solution of KOH (12 M, 1.0 mL, 12 mmol, 4.0 equiv.) was added to **7k** (148 mg, 0.300 mmol, 1.0 equiv.). The mixture was refluxed 16h, then it was acidified with NH_4Cl sat. and extracted with DCM (20 mL). The organic layer was washed with brine, dried over Na_2SO_4 and the solvent was removed under reduced pressure. The crude product was purified by flash column chromatography to give N-(3,3-diphenylpropyl)benzenesulfonamide (**15**) as a yellowish oil (104 mg, 0.300 mmol, quant.).

R_f (Hex:AcOEt = 8:2) = 0.22.
^{1}H NMR (400 MHz, $CDCl_3$) δ 7.90 – 7.76 (m, 2H, Ar*H*), 7.62 – 7.53 (m, 1H, Ar*H*), 7.48 (dd, *J* = 8.3, 6.8 Hz, 2H, Ar*H*), 7.34 – 7.21 (m, 4H, Ar*H*), 7.21 – 7.09 (m, 6H, Ar*H*), 4.66 (t, *J* = 6.2 Hz, 1H, CH_2NH), 3.94 (t, *J* = 7.9 Hz, 1H, ArC*H*), 2.93 (q, *J* = 6.7 Hz, 2H, NCH_2), 2.23 (q, *J* = 7.3 Hz, 2H, CH_2CH_2).
^{13}C NMR (101 MHz, $CDCl_3$) δ 143.7, 140.0, 132.7, 129.2, 128.7, 127.8, 127.2, 126.6, 48.3, 41.9, 35.6.
IR (vmax, cm-1) 1493 (w), 1449 (m), 1325 (s), 1160 (s), 1093 (m), 752 (s).
HRMS (Nanochip-based ESI/LTQ-Orbitrap) m/z: $[M + H]^+$ Calcd for $C_{21}H_{22}NO_2S^+$ 352.1366; Found 352.1366.

3,3-Diphenylpropan-1-amine (**15b**)

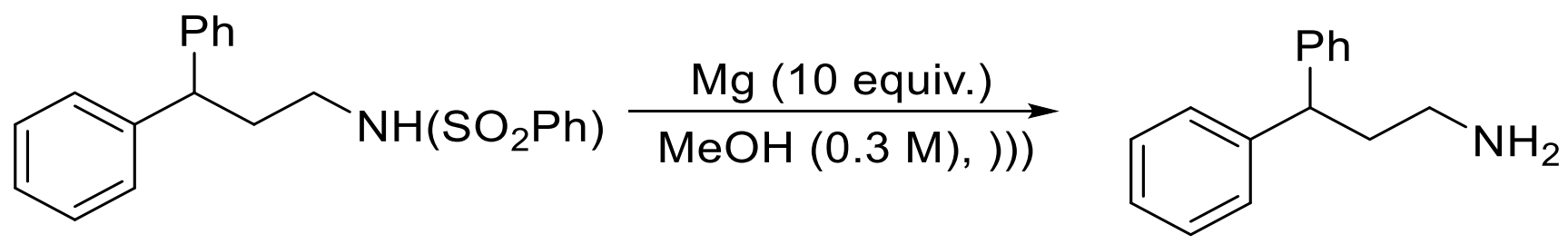

In a 5 mL oven-dried Schlenk tube **15a** (104 mg, 0.300 mmol, 1.0 equiv.) was added. Dry MeOH (1 mL) was added followed by Mg powder (79 mg, 3.0 mmol, 10.0 equiv.). The mixture was sonicated for 5 h. Then the solvent was removed under reduced pressure and the crude was purified by reverse phase chromatography on Büchi (BGB Aquarius C18AQ 20g, 100Å, 15 µm) to obtain 3,3-diphenylpropan-1-amine (**15b**) as a clear oil (59 mg, 0.28 mmol, 93%).

R_f ($CHCl_3$:MeOH = 8:2) = 0.36.
^{1}H NMR (400 MHz, DMSO) δ 7.81 (s, 2H, NH_2), 7.30 (q, *J* = 5.0 Hz, 8H, Ar*H*), 7.20 (tt, *J* = 5.5, 2.4 Hz, 2H, Ar*H*), 4.07 (t, *J* = 7.9 Hz, 1H, ArC*H*), 2.66 (dd, *J* = 9.5, 6.1 Hz, 2H, NCH_2), 2.36 – 2.24 (m, 2H, CH_2CH_2).
^{13}C NMR (101 MHz, DMSO) δ 143.9, 128.6, 127.5, 126.4, 47.3, 37.7, 32.4.

HRMS (ESI/QTOF) m/z: $[M + H]^+$ Calcd for $C_{15}H_{18}N^+$ 212.1434; Found 212.1438.
NMR data consistent with reported value.[31]

S13. References

1. M. Benz, T. M. Klapötke, J. Stierstorfer, M. Voggenreiter, “Synthesis and Characterization of Binary, Highly Endothermic, and Extremely Sensitive 2,2′-Azobis(5-azidotetrazole),” *Journal of the American Chemical Society* 144 (2022): 6143–6147, https://doi.org/10.1021/jacs.2c00995.
2. P. Zhang, M. Li, X.-S. Xue, et al., “*N* -Trifluoromethylthio-dibenzenesulfonimide: A Shelf-Stable, Broadly Applicable Electrophilic Trifluoromethylthiolating Reagent,” *The Journal of Organic Chemistry* 81 (2016): 7486–7509, https://doi.org/10.1021/acs.joc.6b01178.
3. C. R. Pitts, B. Ling, J. A. Snyder, A. E. Bragg, T. Lectka, “Aminofluorination of Cyclopropanes: A Multifold Approach through a Common, Catalytically Generated Intermediate,” *Journal of the American Chemical Society* 138 (2016): 6598–6609, https://doi.org/10.1021/jacs.6b02838.
4. S. Haubenreisser, T. H. Wöste, C. Martínez, K. Ishihara, K. Muñiz, “Structurally Defined Molecular Hypervalent Iodine Catalysts for Intermolecular Enantioselective Reactions,” *Angewandte Chemie International Edition* 55 (2016): 413–417, https://doi.org/10.1002/anie.201507180.
5. D. Cheng, D. Huang, Y. Shi, “Synergistic effect of additives on cyclopropanation of olefins,” *Organic & Biomolecular Chemistry* 11 (2013): 5588, https://doi.org/10.1039/c3ob40751a.
6. L. Wang, X. Wang, G. Zhang, S. Yang, Y. Li, Q. Zhang, “Copper-catalyzed 1,3-aminoazidation of arylcyclopropanes: a facile access to 1,3-diamine derivatives,” *Organic Chemistry Frontiers* 6 (2019): 2934–2938, https://doi.org/10.1039/C9QO00638A.
7. A. Zhou, Y. Shao, F. Chen, P.-C. Qian, J. Cheng, “The copper-catalyzed ring-opening reactions of cyclopropanes by N-fluorobenzenesulfonimide toward N-allylsulfonamides,” *Tetrahedron Letters* 89 (2022): 153597, https://doi.org/10.1016/j.tetlet.2021.153597.
8. H. Zhang, H. Xiao, F. Jiang, Y. Fang, L. Zhu, C. Li, “Copper-Catalyzed Ring-Opening 1,3-Aminotrifluoromethylation of Arylcyclopropanes,” *Organic Letters* 23 (2021): 2268–2272, https://doi.org/10.1021/acs.orglett.1c00390.
9. J. Du, J. Li, L. Qian, X. Luan, Z. Zuo, “Organophotoredox and Cobalt-catalyzed 1,2-Difunctionalization of Mono-Donor Cyclopropanes,” *Angewandte Chemie International Edition* 64 (2025): e202515761, https://doi.org/10.1002/anie.202515761.
10. L. Hu, Y. Xiang, X.-B. Lan, Y. Xie, “An Intermolecular Hydroarylation of Unactivated Arylcyclopropane via Re_2O_7 /HFIP-Mediated Ring Opening,” *Organic Letters* 26 (2024): 2085–2090, https://doi.org/10.1021/acs.orglett.4c00272.
11. A. Gagnon, M. Duplessis, P. Alsabeh, F. Barabé, “Palladium-Catalyzed Cross-Coupling Reaction of Tricyclopropylbismuth with Aryl Halides and Triflates,” *The Journal of Organic Chemistry* 73 (2008): 3604–3607, https://doi.org/10.1021/jo702377h.
12. M. Zhang, X. Cui, X. Chen, et al., “Facile synthesis of aryl(het)cyclopropane catalyzed by palladacycle,” *Tetrahedron* 68 (2012): 900–905, https://doi.org/10.1016/j.tet.2011.11.024.
13. J. Pan, H. Qu, Y. Li, et al., “Switchable Divergent Electrochemical Hydrodehalogenation of *gem* -Dihalocyclopropanes,” *The Journal of Organic Chemistry* 89 (2024): 16929–16935, https://doi.org/10.1021/acs.joc.4c01748.

14. X. Wang, L. Wang, S. Yang, L. Zhang, Y. Li, Q. Zhang, “Copper-catalyzed 1,3-aminothiocyanation of arylcyclopropanes,” *Organic & Biomolecular Chemistry* 18 (2020): 4932–4935, https://doi.org/10.1039/D0OB01060J.
15. S. Khanra, S. Saha, S. Mandal, N. D. Paul, “Metal–Ligand Cooperation for Iron(II)-Catalyzed Direct Wittig-Type Olefination from Alcohols in Air,” *European Journal of Organic Chemistry* 28 (2025): e202500257, https://doi.org/10.1002/ejoc.202500257.
16. J. Hou, X. Han, Y. Zhang, J. Huang, J. Wang, K. Yuan, “Triflic Acid/Silane Promoted Deoxygenative Transformation of Ketones via Carbocations,” *Organic Letters* 25 (2023): 5709–5713, https://doi.org/10.1021/acs.orglett.3c01762.
17. M. Yang, Y. Li, H. Li, et al., “CuCl-Catalyzed Conversion of Aryl Boronic Acids and Carbon Dioxide to Carboxylate Esters,” *European Journal of Organic Chemistry* 2022 (2022): e202200212, https://doi.org/10.1002/ejoc.202200212.
18. M. Umeda, H. Noguchi, T. Nishimura, “Enantioselective Synthesis of Chiral Indane Derivatives by Rhodium-Catalyzed Addition of Arylboron Reagents to Substituted Indenes,” *Organic Letters* 22 (2020): 9597–9602, https://doi.org/10.1021/acs.orglett.0c03651.
19. X.-L. Lu, M. Shannon, X.-S. Peng, H. N. C. Wong, “Stereospecific Iron-Catalyzed Carbon(sp^2)–Carbon(sp^3) Cross-Coupling with Alkyllithium and Alkenyl Iodides,” *Organic Letters* 21 (2019): 2546–2549, https://doi.org/10.1021/acs.orglett.9b00394.
20. L. Ernst, T. Rieck, M. Soliven, “Preferred conformation and barriers to internal rotation of ortho-disubstituted cyclopropylbenzenes,” *Canadian Journal of Chemistry* 77 (1999): 1697–1706.
21. D. Wang, X. Xue, K. N. Houk, Z. Shi, “Mild Ring-Opening 1,3-Hydroborations of Non-Activated Cyclopropanes,” *Angewandte Chemie International Edition* 57 (2018): 16861–16865, https://doi.org/10.1002/anie.201811036.
22. M. Xiang, C. Zhou, X.-L. Yang, B. Chen, C.-H. Tung, L.-Z. Wu, “Visible Light-Catalyzed Benzylic C–H Bond Chlorination by a Combination of Organic Dye (Acr^+ -Mes) and *N* -Chlorosuccinimide,” *The Journal of Organic Chemistry* 85 (2020): 9080–9087, https://doi.org/10.1021/acs.joc.0c01000.
23. F. Wang, J. Chen, X. Jia, et al., “Direct Benzylic C(sp^3)–O Coupling with Alcohol via Site-Selective C(sp^3)–H Cleavage at Room Temperature through a Remote Directing Group-Enabled Radical Relay Strategy,” *The Journal of Organic Chemistry* 87 (2022): 10698–10709, https://doi.org/10.1021/acs.joc.2c00884.
24. P. Lian, W. Long, J. Li, Y. Zheng, X. Wan, “Visible-Light-Induced Vicinal Dichlorination of Alkenes through LMCT Excitation of $CuCl_2$,” *Angewandte Chemie International Edition* 59 (2020): 23603–23608, https://doi.org/10.1002/anie.202010801.
25. S. Braverman, Y. Zafrani, “Ramberg-Bäcklund rearrangement vs. β-Elimination of haloform from trichloro and trifluoromethyl sulfones,” *Tetrahedron* 54 (1998): 1901–1912, https://doi.org/10.1016/S0040-4020(97)10404-5.
26. Z. Fang, Y. Feng, H. Dong, D. Li, T. Tang, “Copper(I)-catalyzed radical decarboxylative imidation of carboxylic acids with N-fluoroarylsulfonimides,” *Chemical Communications* 52 (2016): 11120–11123, https://doi.org/10.1039/C6CC03227C.
27. G. N. Ziakas, E. A. Rekka, A. M. Gavalas, P. T. Eleftheriou, P. N. Kourounakis, “New analogues of butylated hydroxytoluene as anti-inflammatory and antioxidant agents,” *Bioorganic & Medicinal Chemistry* 14 (2006): 5616–5624, https://doi.org/10.1016/j.bmc.2006.04.030.

28. L. Chen, H. Lang, L. Fang, J. Yu, L. Wang, “Nickel-Catalyzed Desulfitative Suzuki–Miyaura Cross-Coupling of *N* , *N* -Disulfonylmethylamines and Arylboronic Acids,” *European Journal of Organic Chemistry* 2014 (2014): 6385–6389, https://doi.org/10.1002/ejoc.201402919.
29. Z. Fan, N. Liang, Y. Wang, “Rhodium(II)-Catalyzed Oxo-Amination of Aryl Cyclopropanes,” *European Journal of Organic Chemistry* 28 (2025): e202500235, https://doi.org/10.1002/ejoc.202500235.
30. W. Sheng, X. Huang, J. Cai, et al., “Electrochemical Oxidation Enables Regioselective 1,3-Hydroxyfunctionalization of Cyclopropanes,” *Organic Letters* 25 (2023): 6178–6183, https://doi.org/10.1021/acs.orglett.3c02309.
31. Y. Gorbunova, D. N. Zakusilo, I. A. Boyarskaya, A. V. Vasilyev, “Reactions of 3-arylpropenenitriles with arenes under superelectrophilic activation conditions: Hydroarylation of the carbon-carbon double bond followed by cyclization into 3-arylindanones,” *Tetrahedron* 76 (2020): 131264, https://doi.org/10.1016/j.tet.2020.131264.

S14. NMR spectra

^{1}H NMR Spectrum of NCBSI (400 MHz, $CDCl_3$)

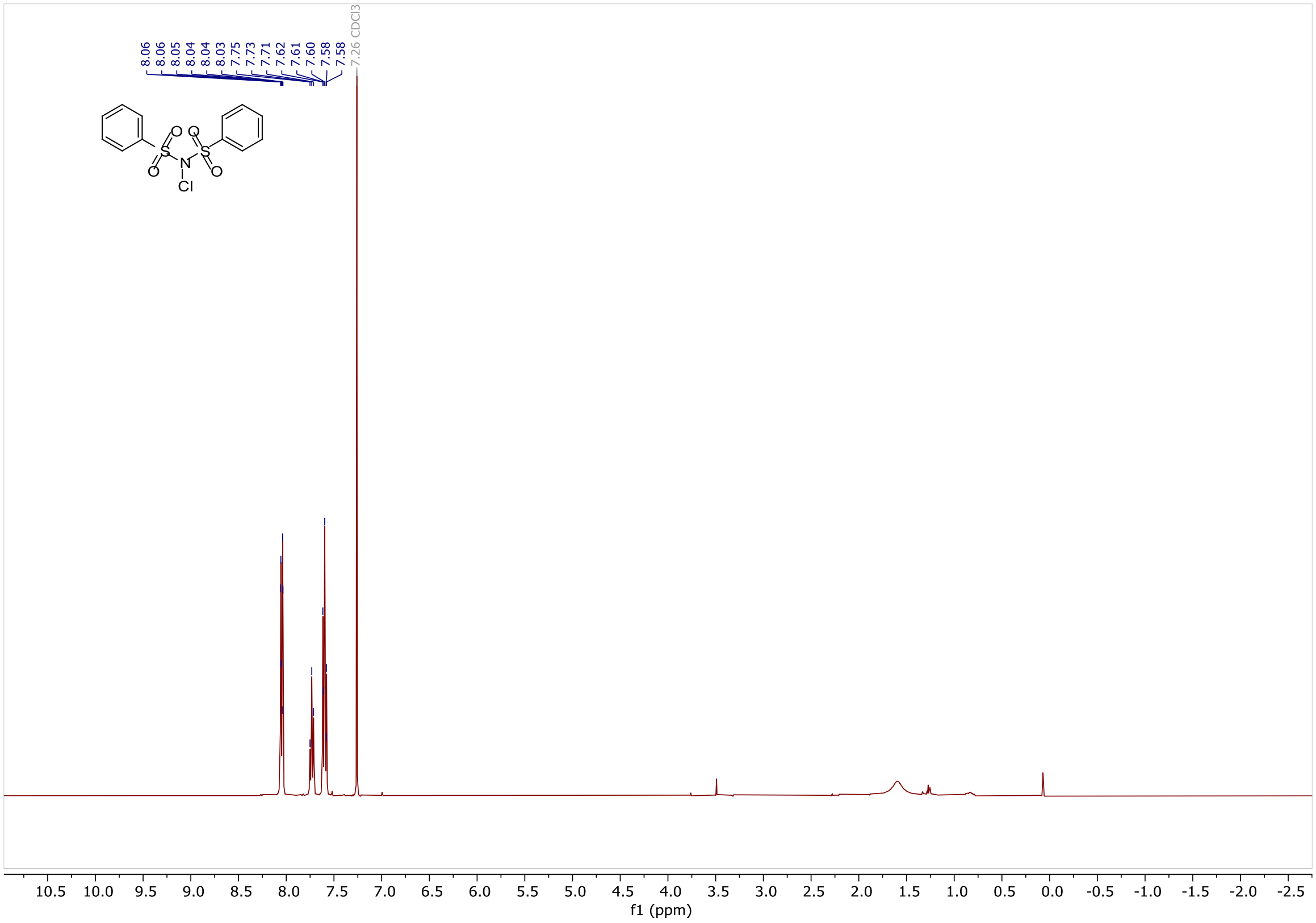

^{13}C NMR Spectrum of NCBSI (101 MHz, $CDCl_3$)

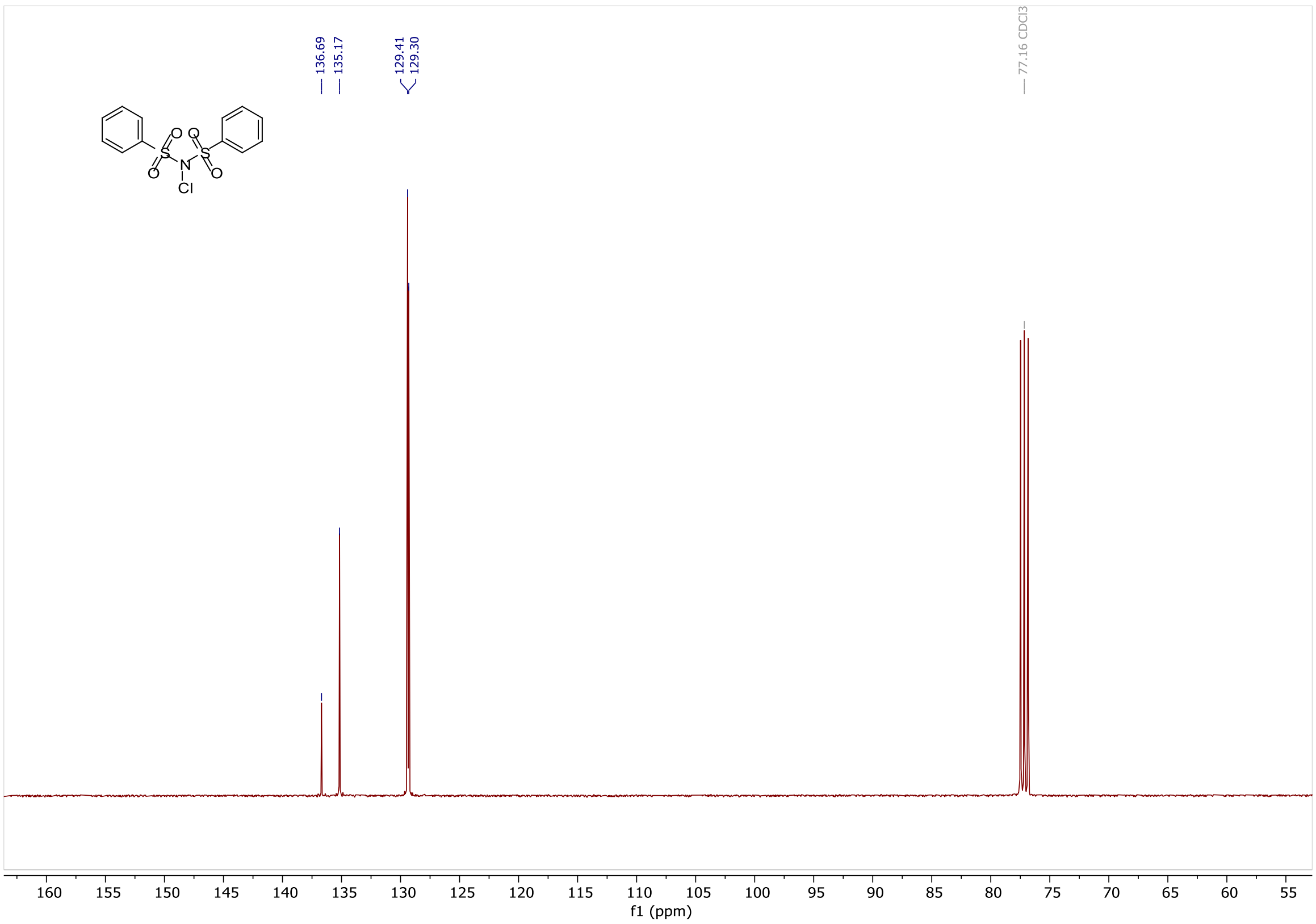

Aryl cyclopropanes **5**

^{1}H NMR Spectrum of *o*-5b (400 MHz, $CDCl_3$)

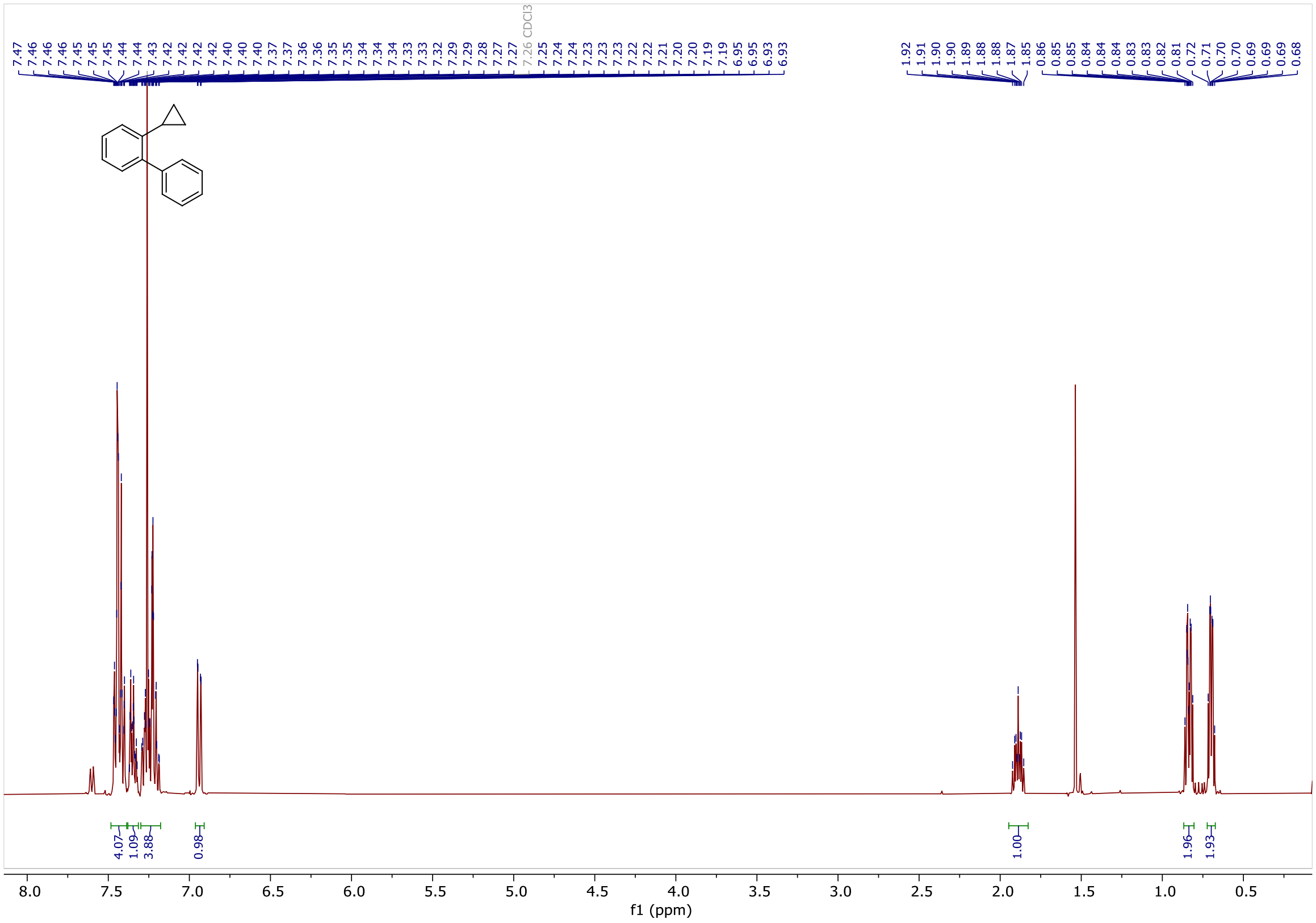

^{13}C NMR Spectrum of *o*-5b (101 MHz, $CDCl_3$)

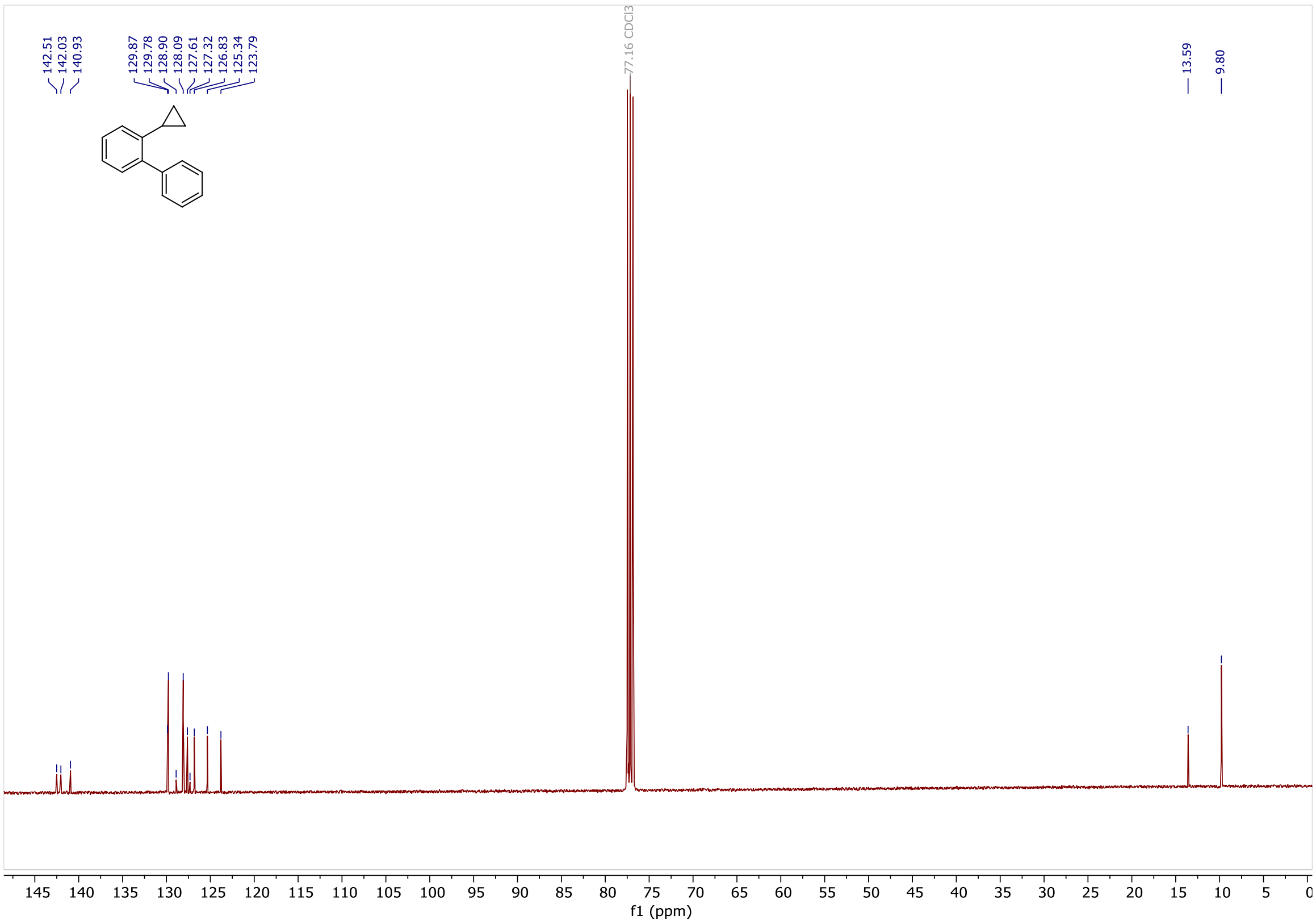

^{1}H NMR Spectrum of *m*-5b (400 MHz, $CDCl_3$)

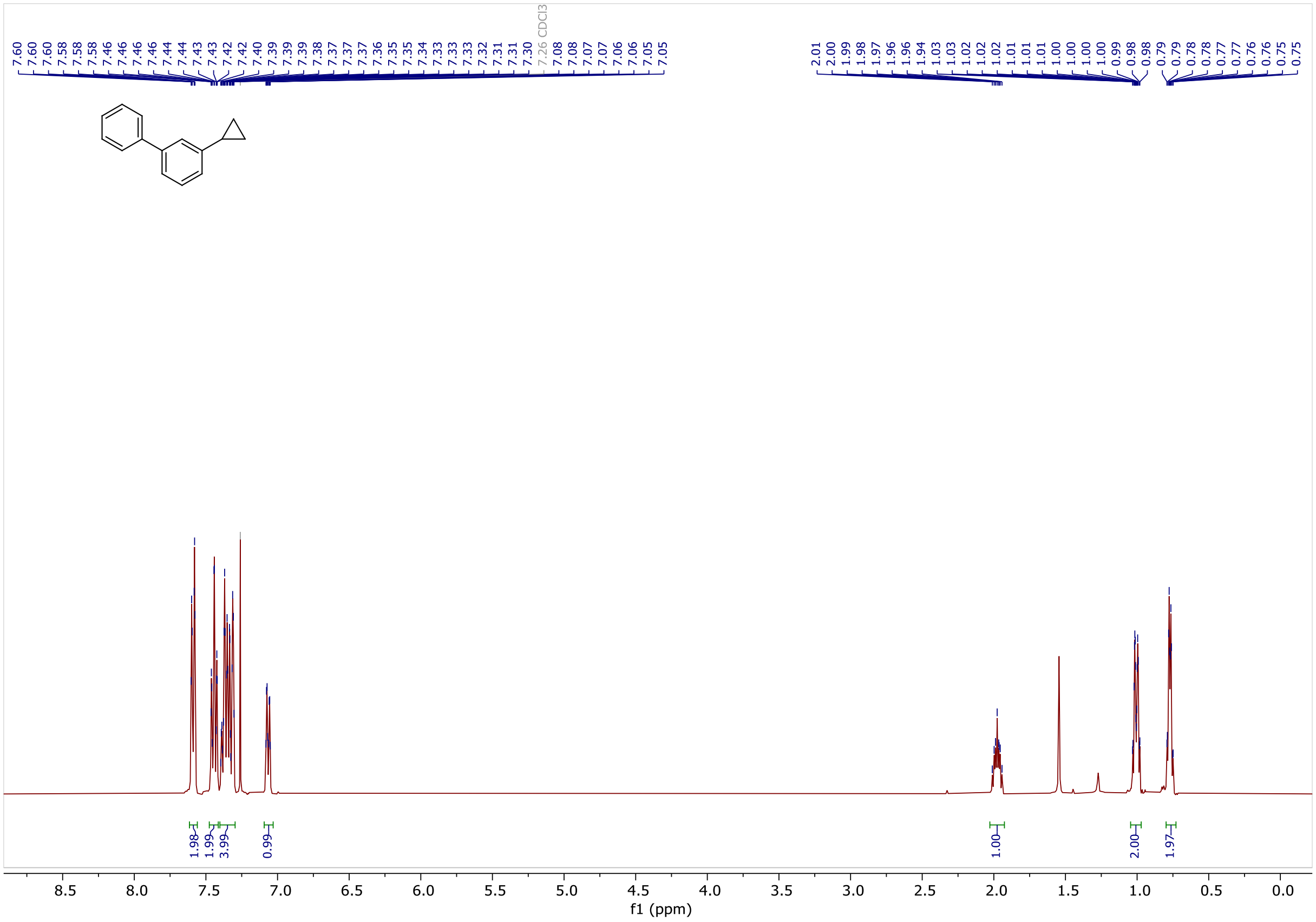

^{13}C NMR Spectrum of *m*-5b (101 MHz, $CDCl_3$)

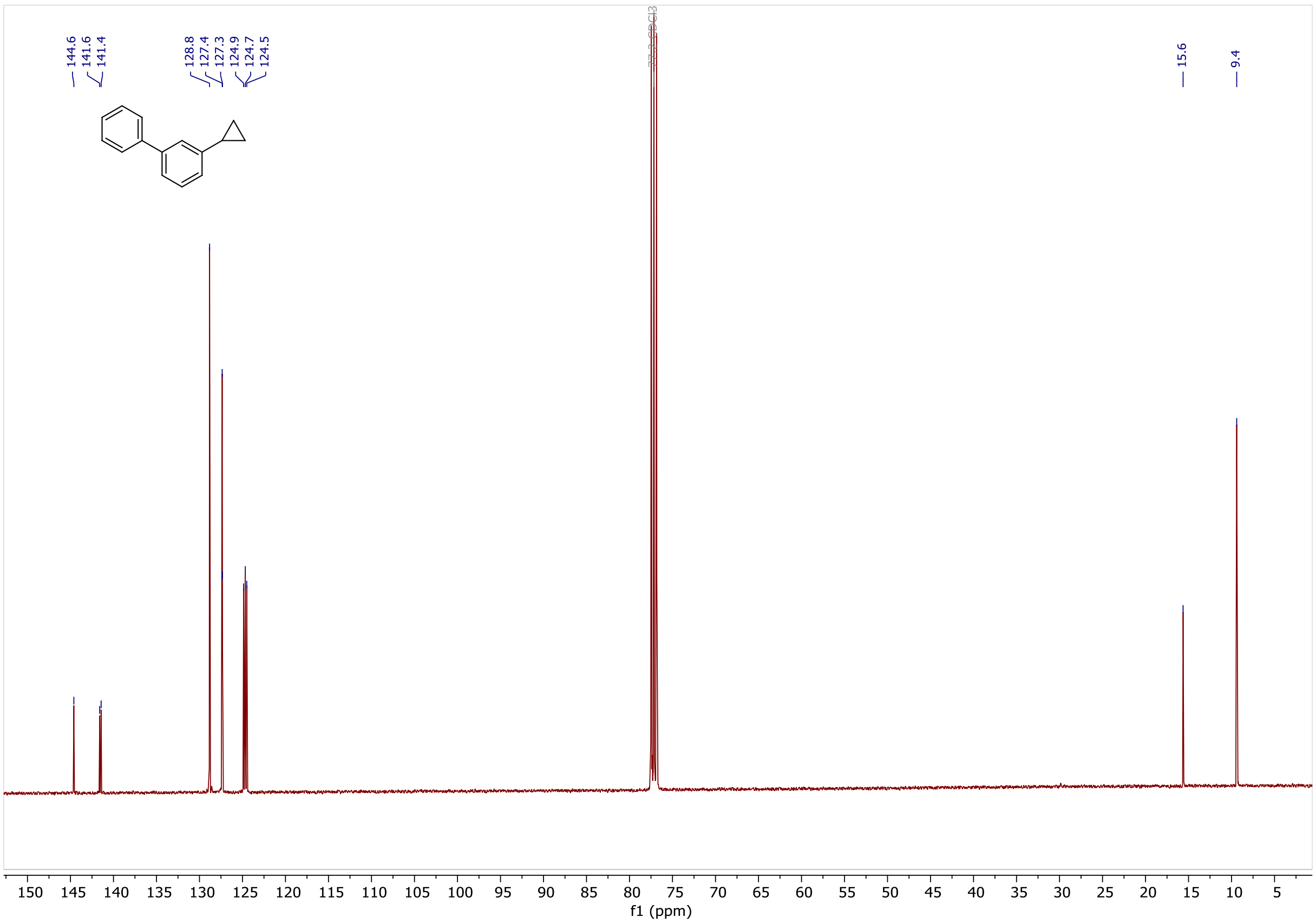

^{1}H NMR Spectrum of *p*-5b (400 MHz, $CDCl_3$)

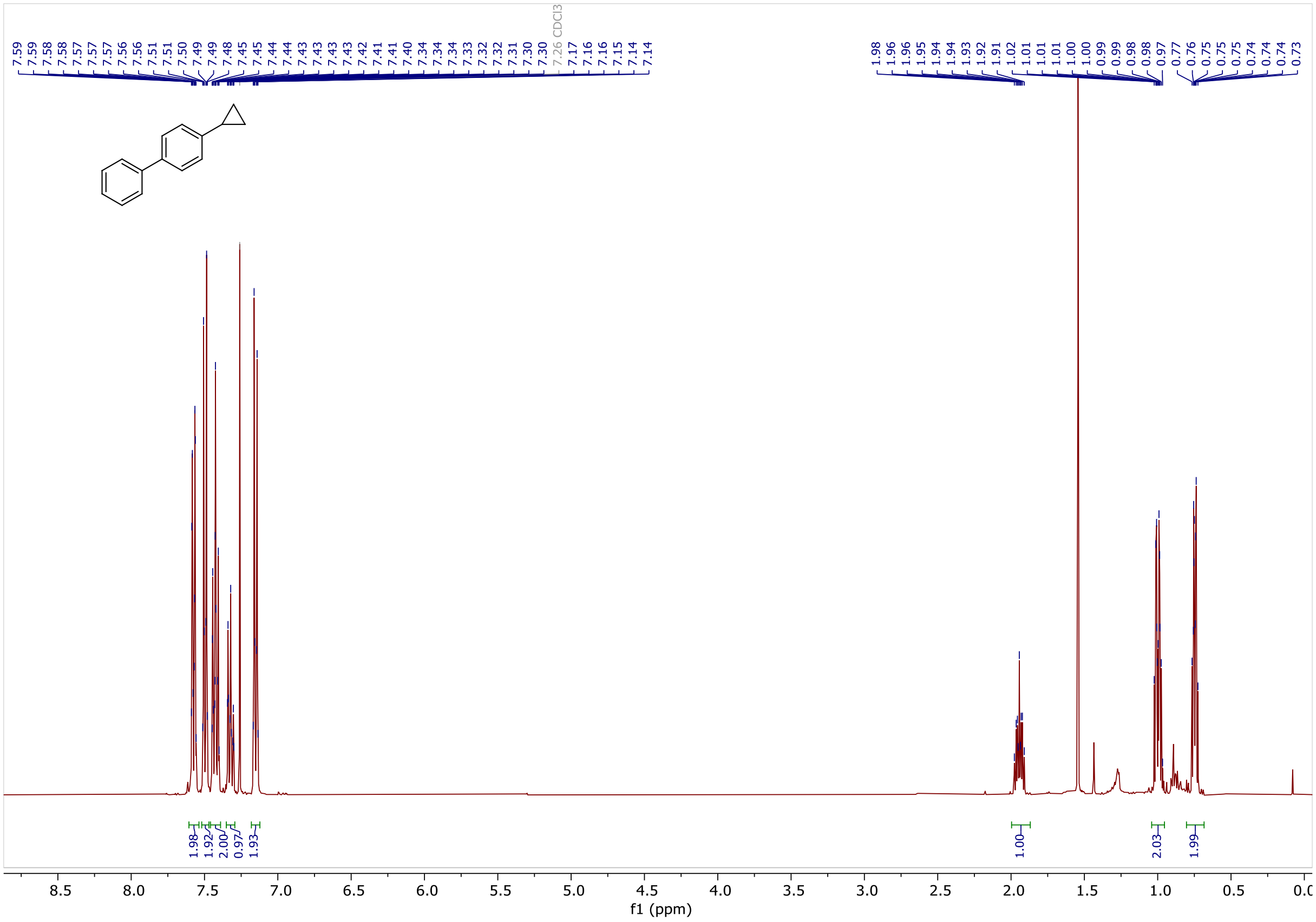

^{13}C NMR Spectrum of *p*-5b (101 MHz, $CDCl_3$)

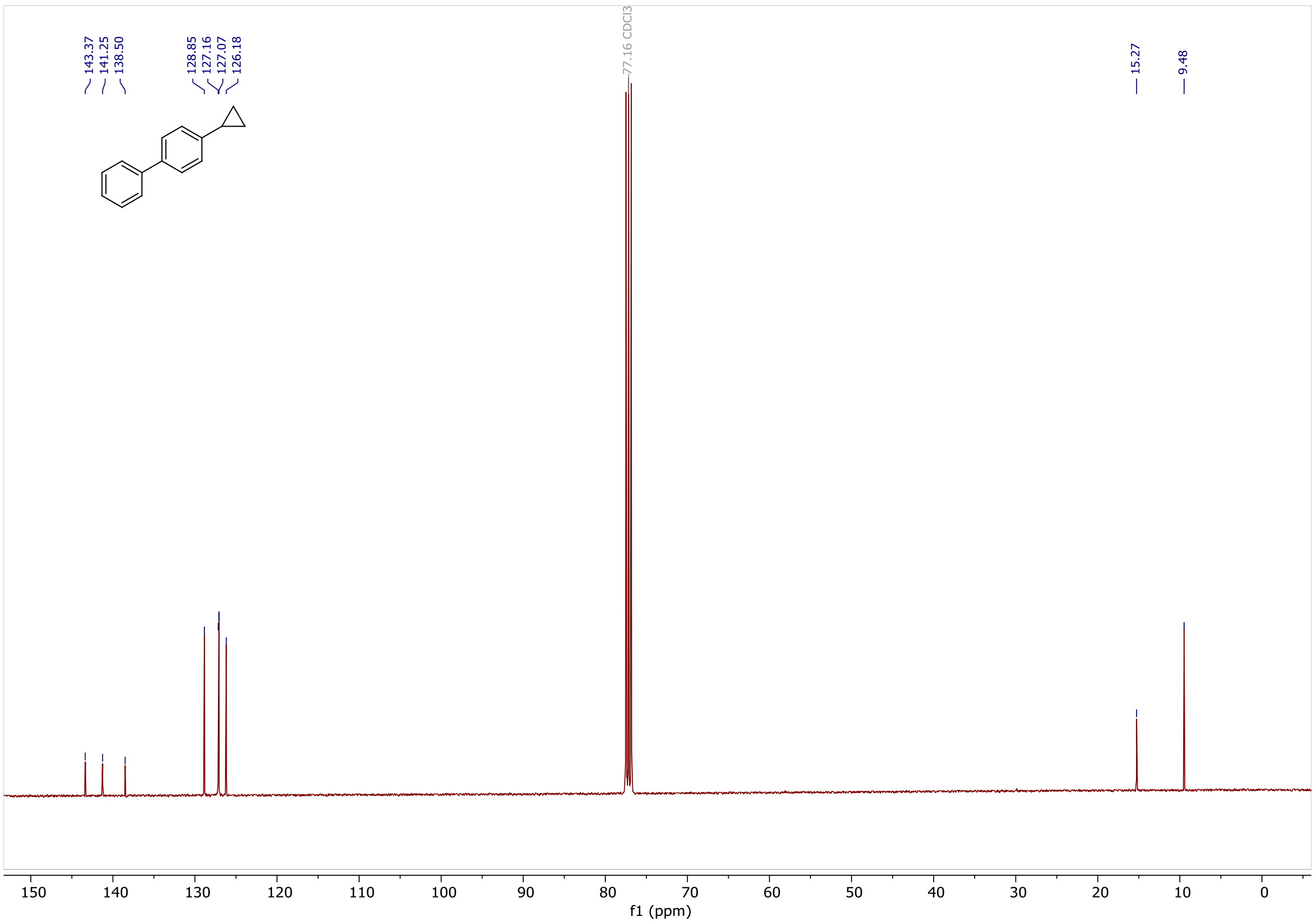

^{1}H NMR Spectrum of *o*-5c (400 MHz, $CDCl_3$)

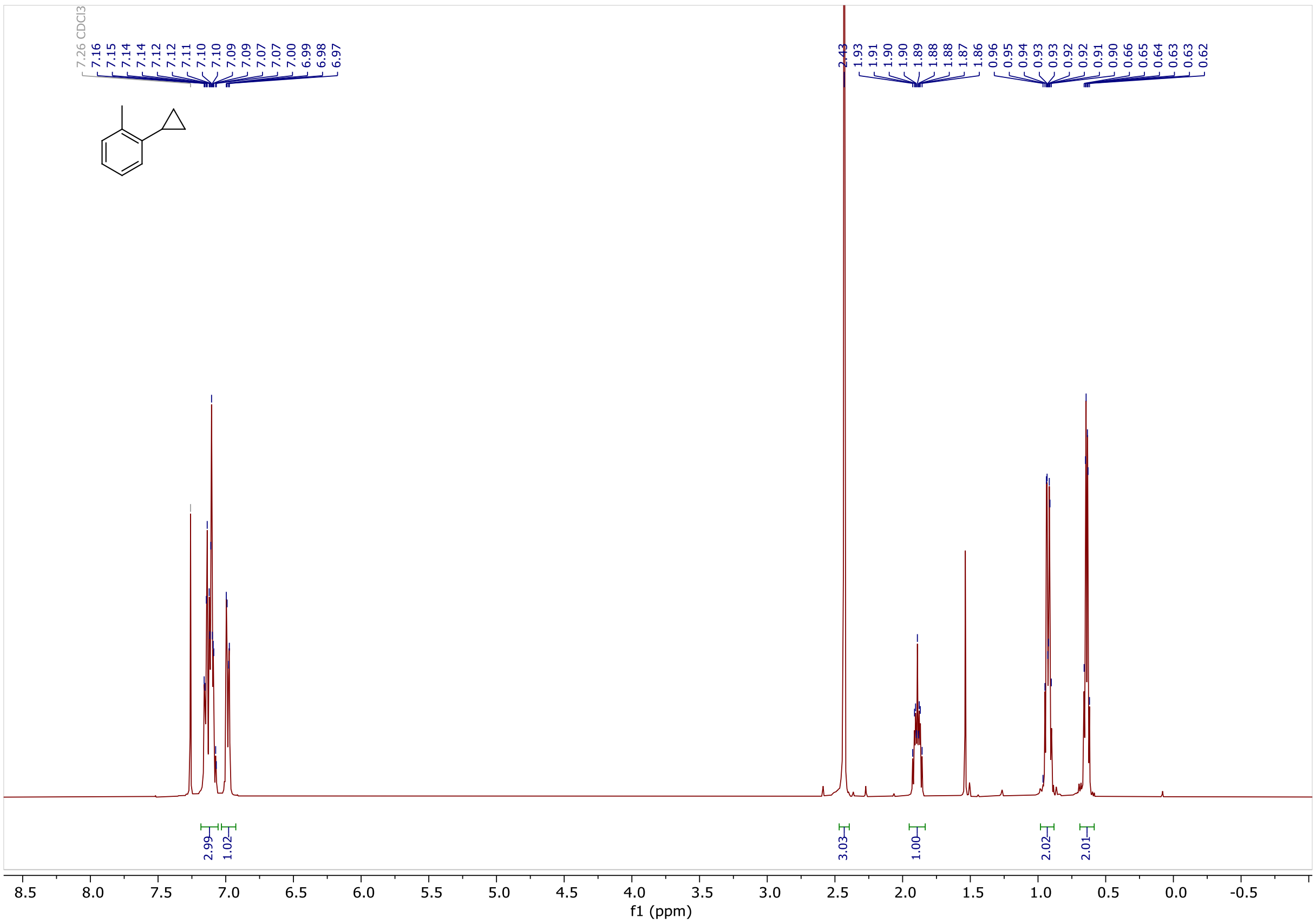

^{13}C NMR Spectrum of *o*-5c (101 MHz, $CDCl_3$)

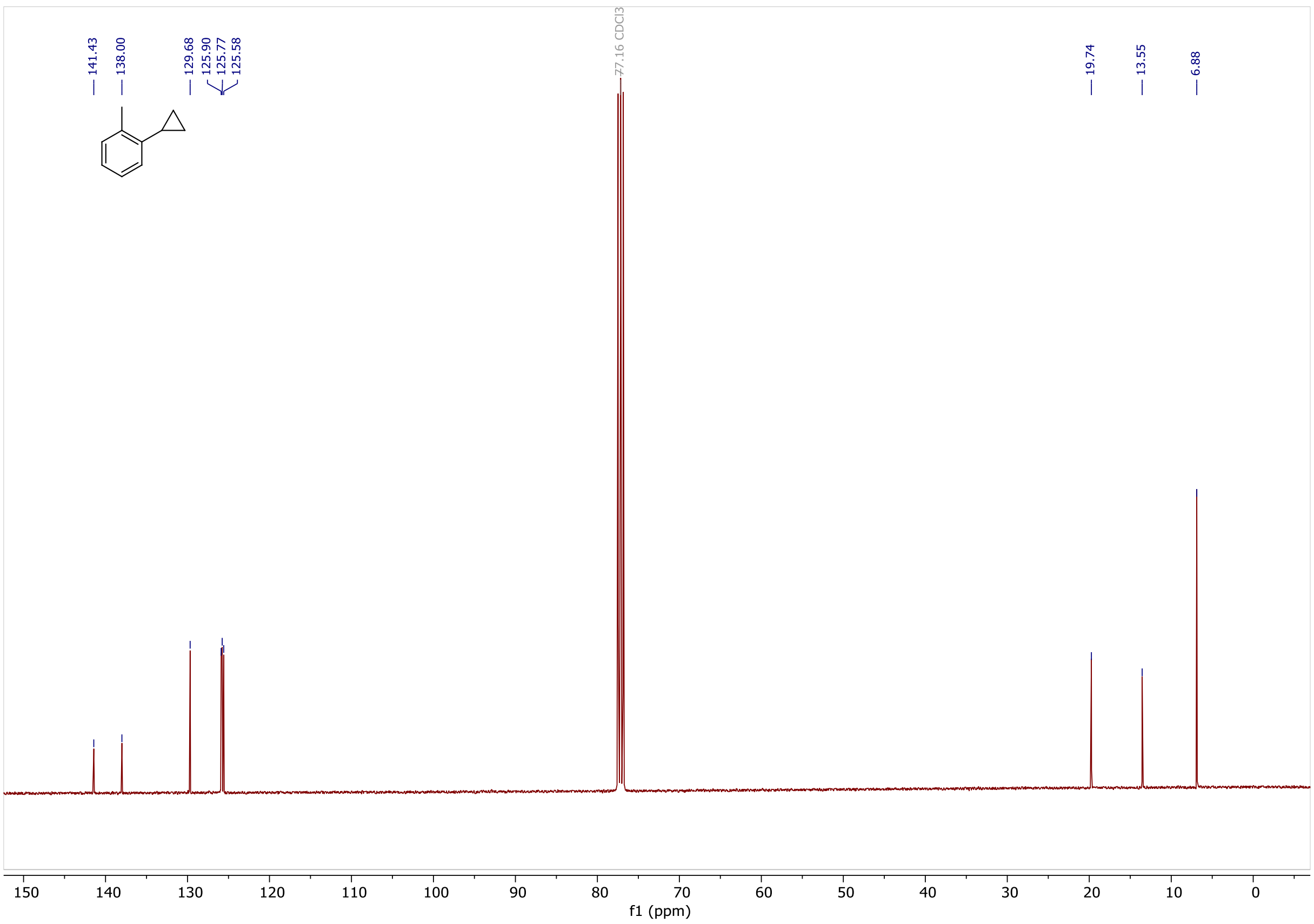

^{1}H NMR Spectrum of *m*-5c (400 MHz, $CDCl_3$)

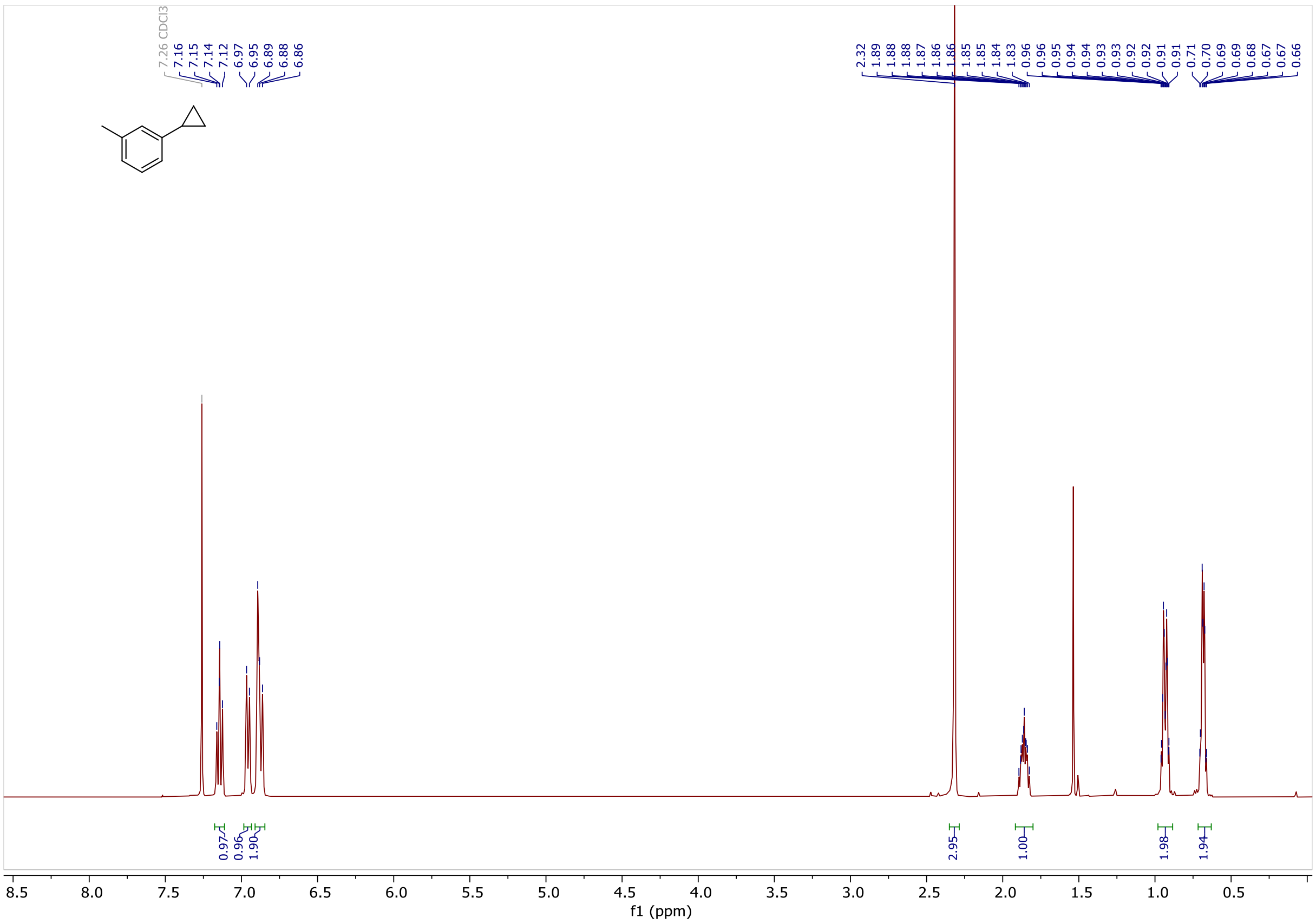

^{13}C NMR Spectrum of *m*-5c (101 MHz, $CDCl_3$)

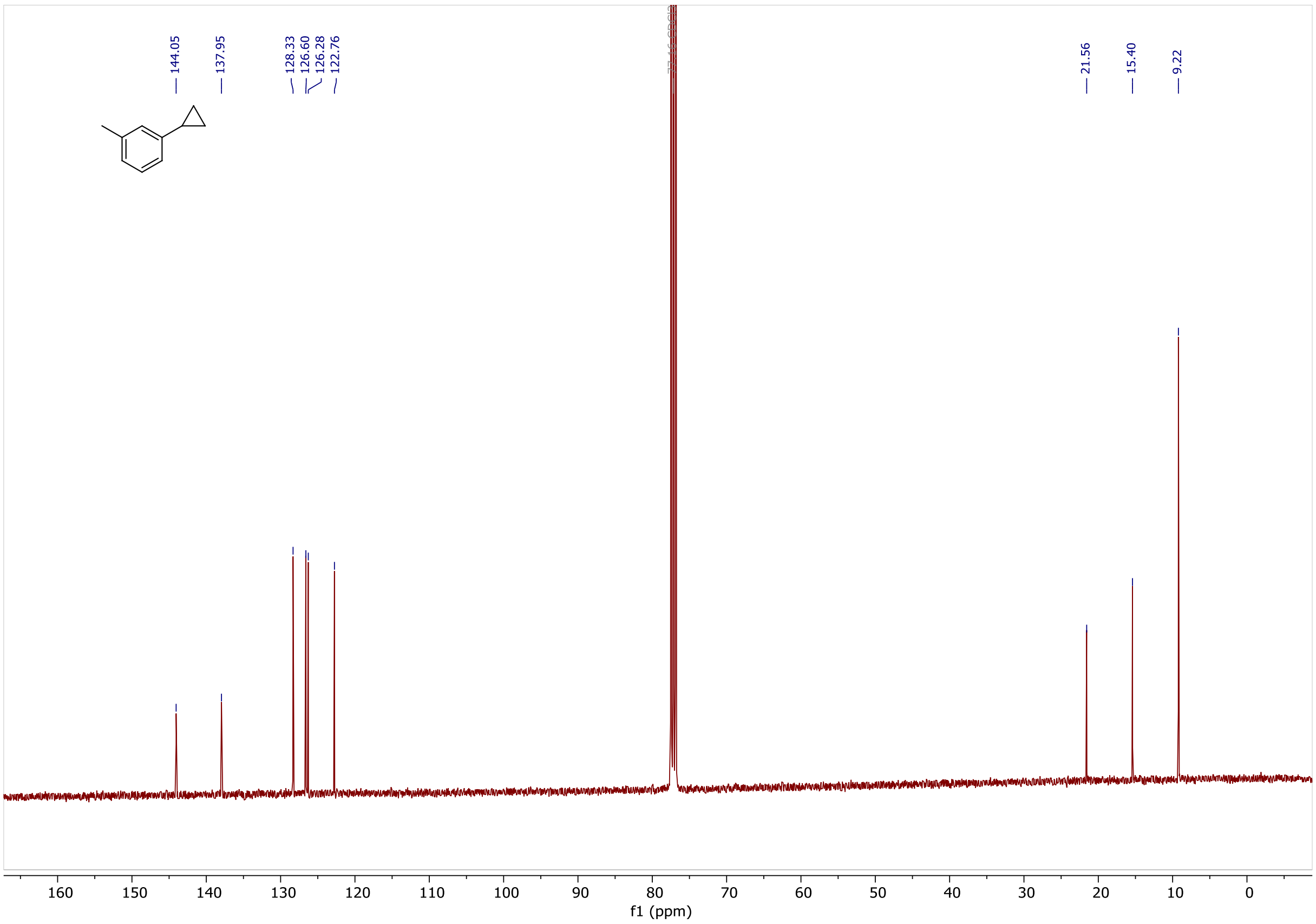

^{1}H NMR Spectrum of *p*-5c (400 MHz, $CDCl_3$)

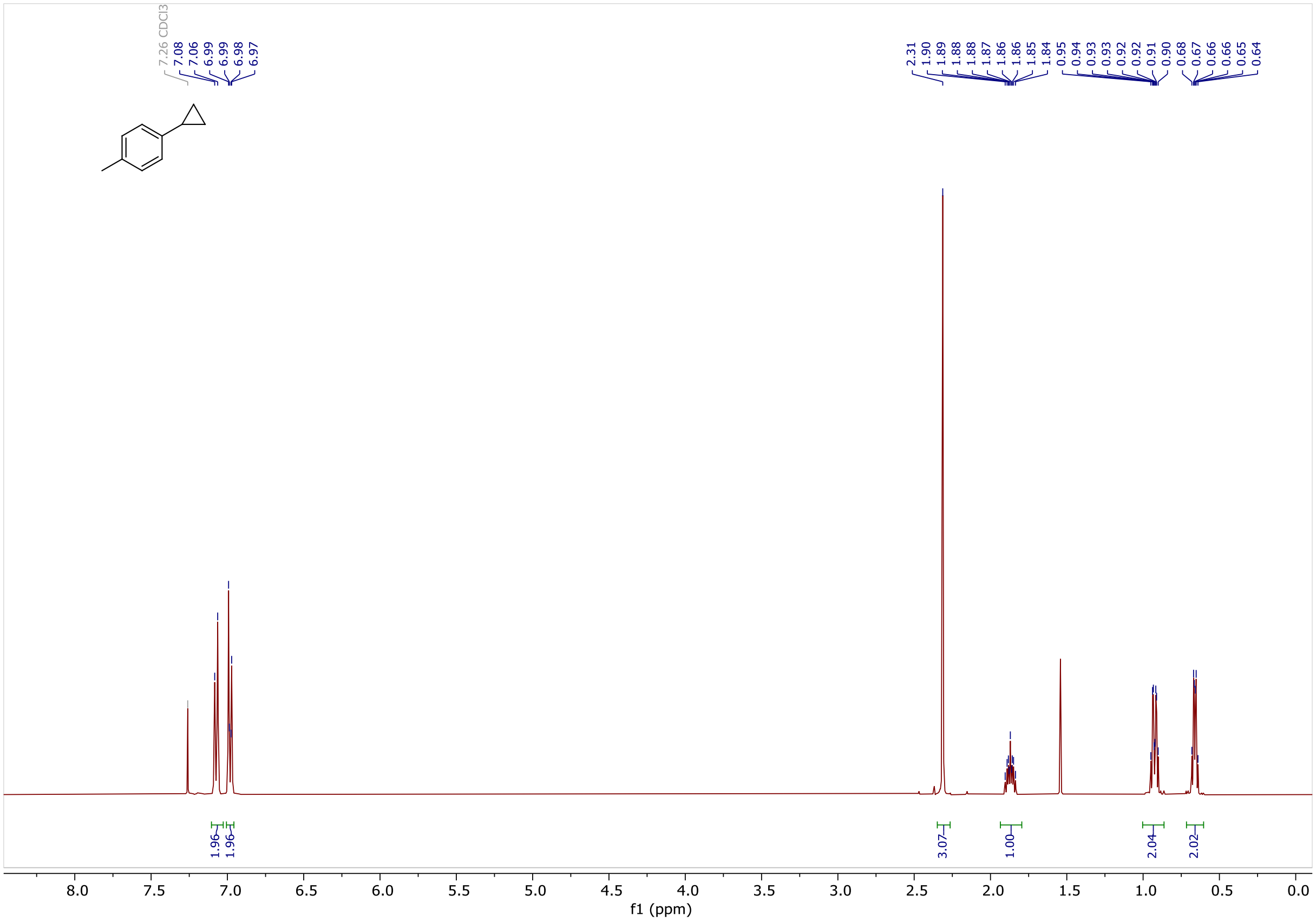

^{13}C NMR Spectrum of *p*-5c (101 MHz, $CDCl_3$)

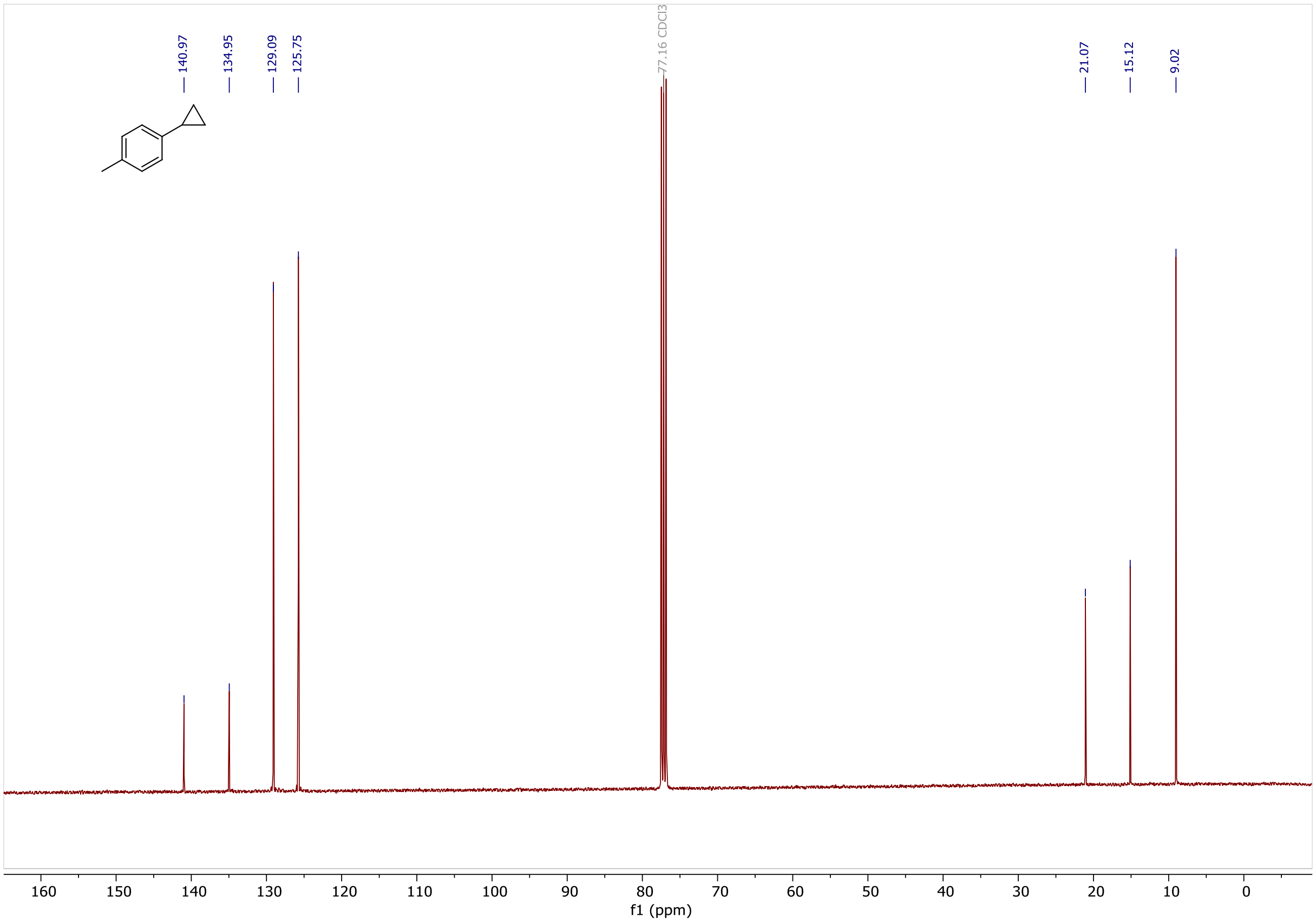

^{1}H NMR Spectrum of *o*-5d (400 MHz, $CDCl_3$)

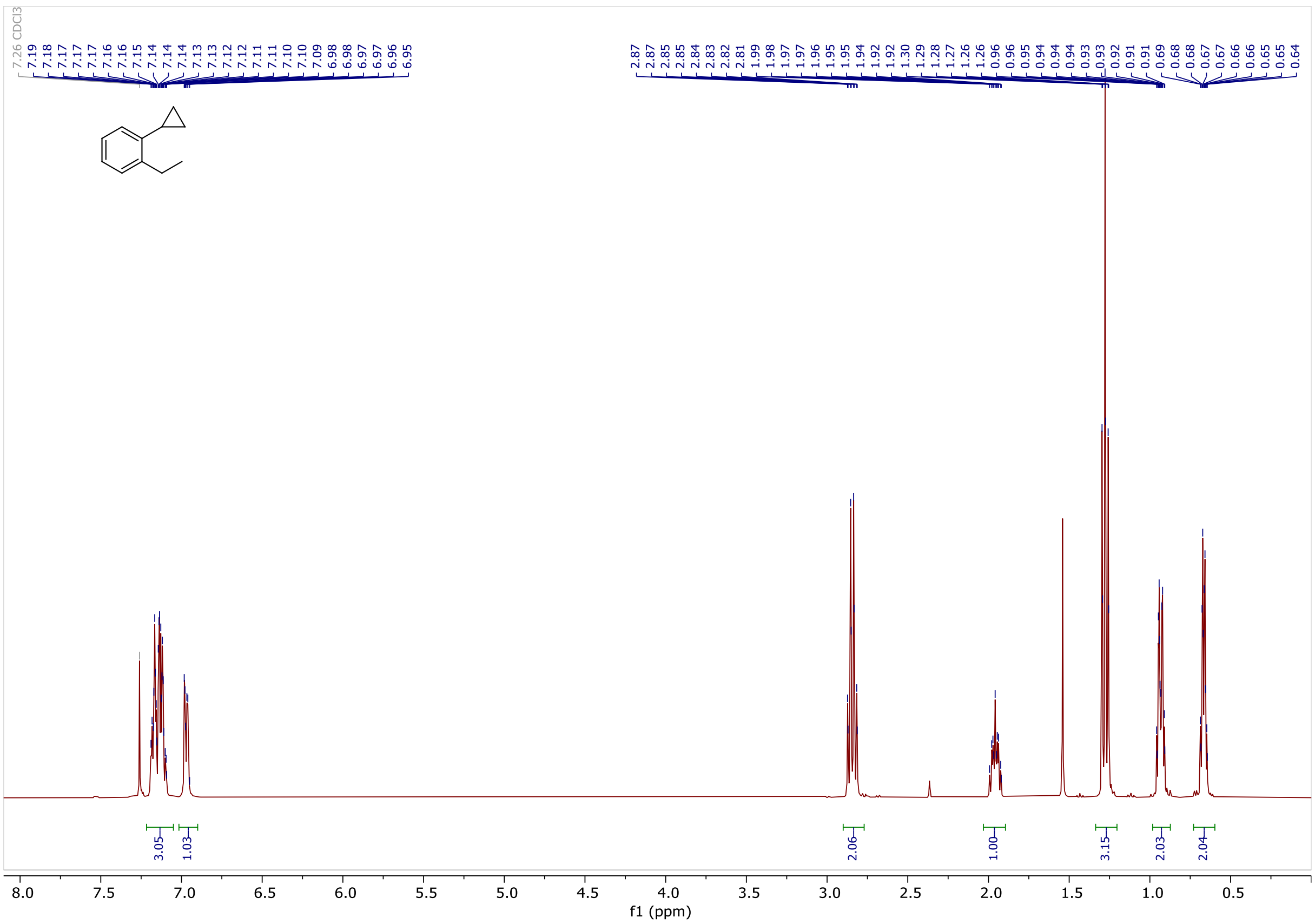

^{13}C NMR Spectrum of *o*-5d (101 MHz, $CDCl_3$)

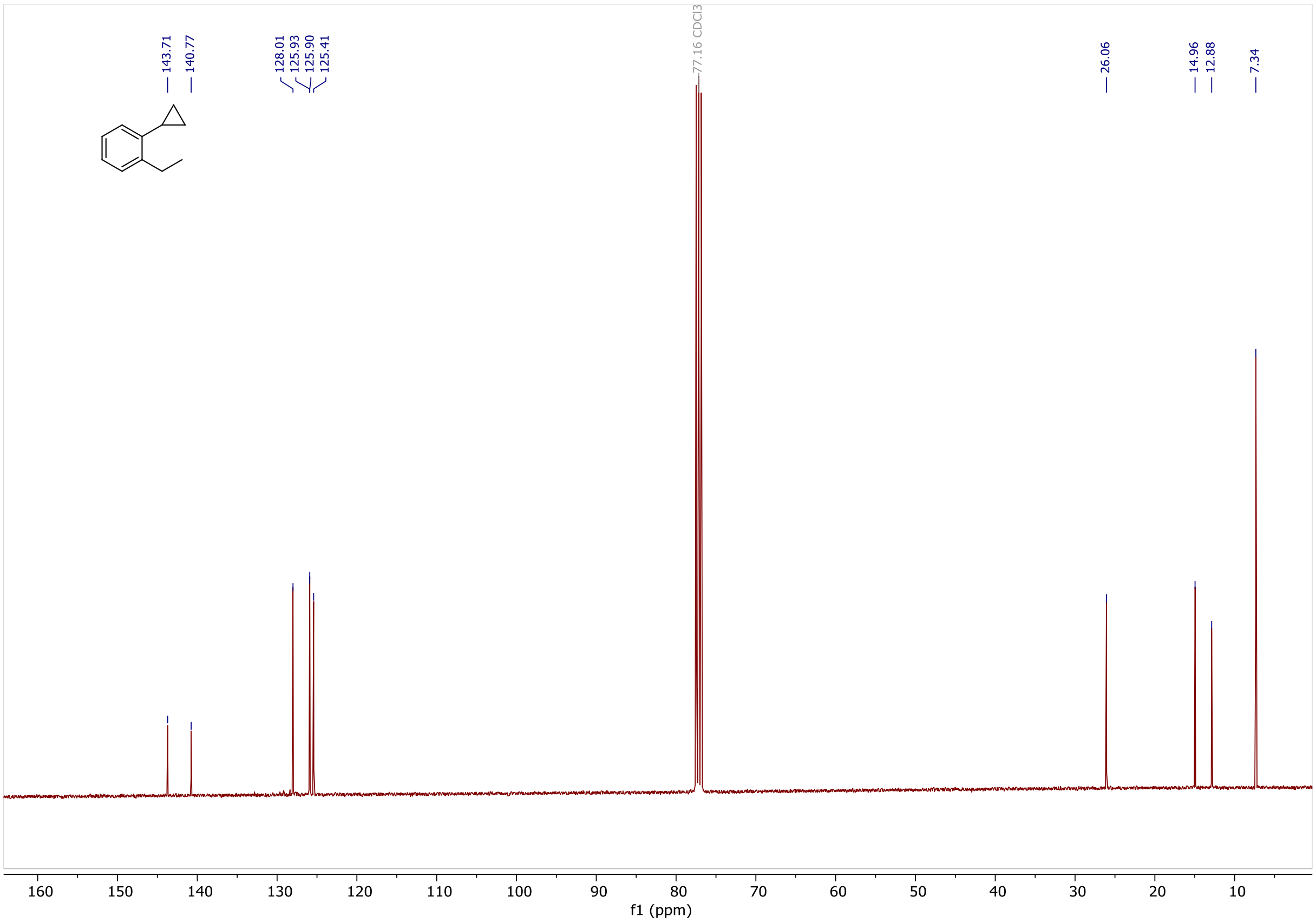

^{1}H NMR Spectrum of *m*-5d (400 MHz, $CDCl_3$)

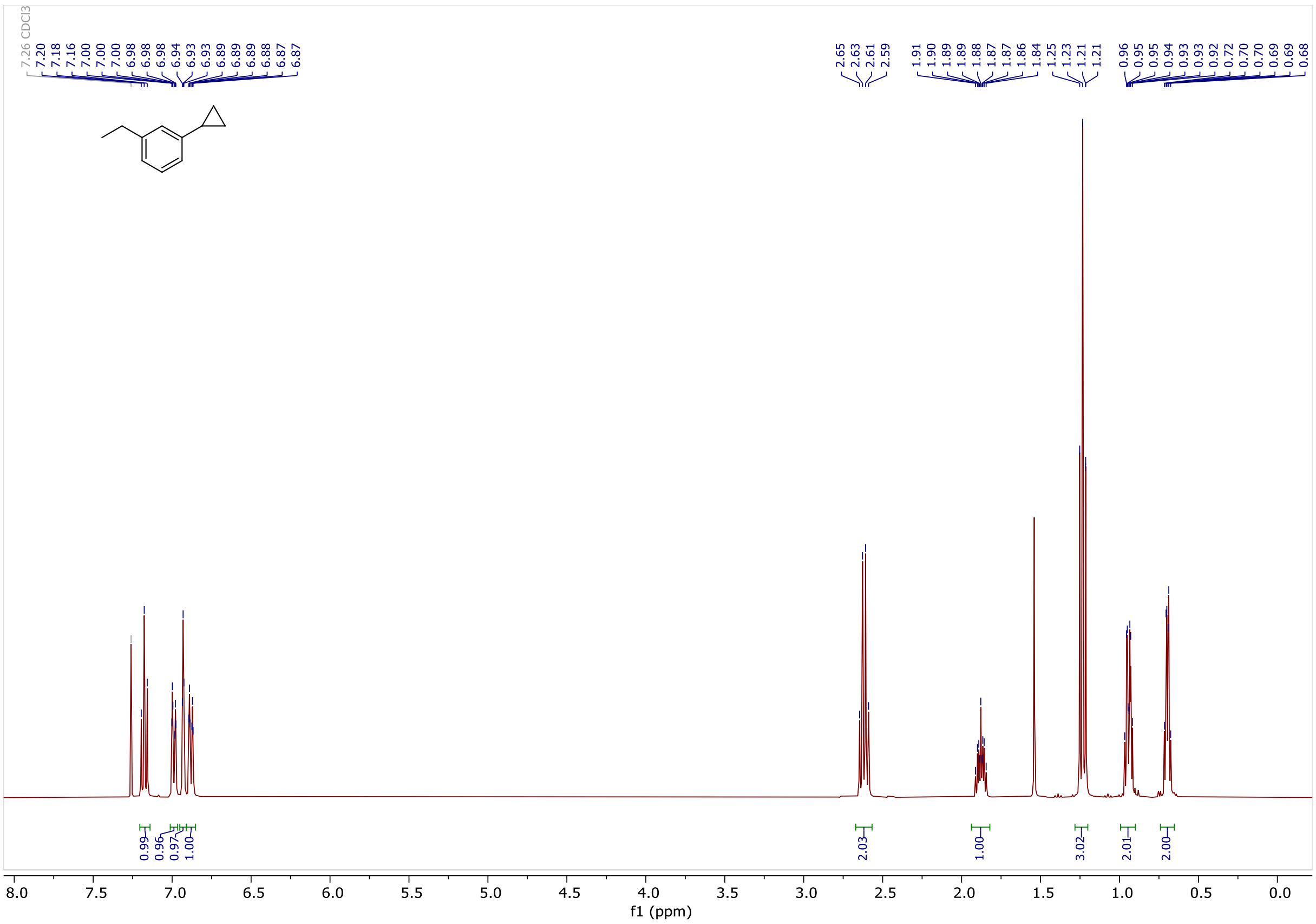

^{13}C NMR Spectrum of *m*-5d (101 MHz, $CDCl_3$)

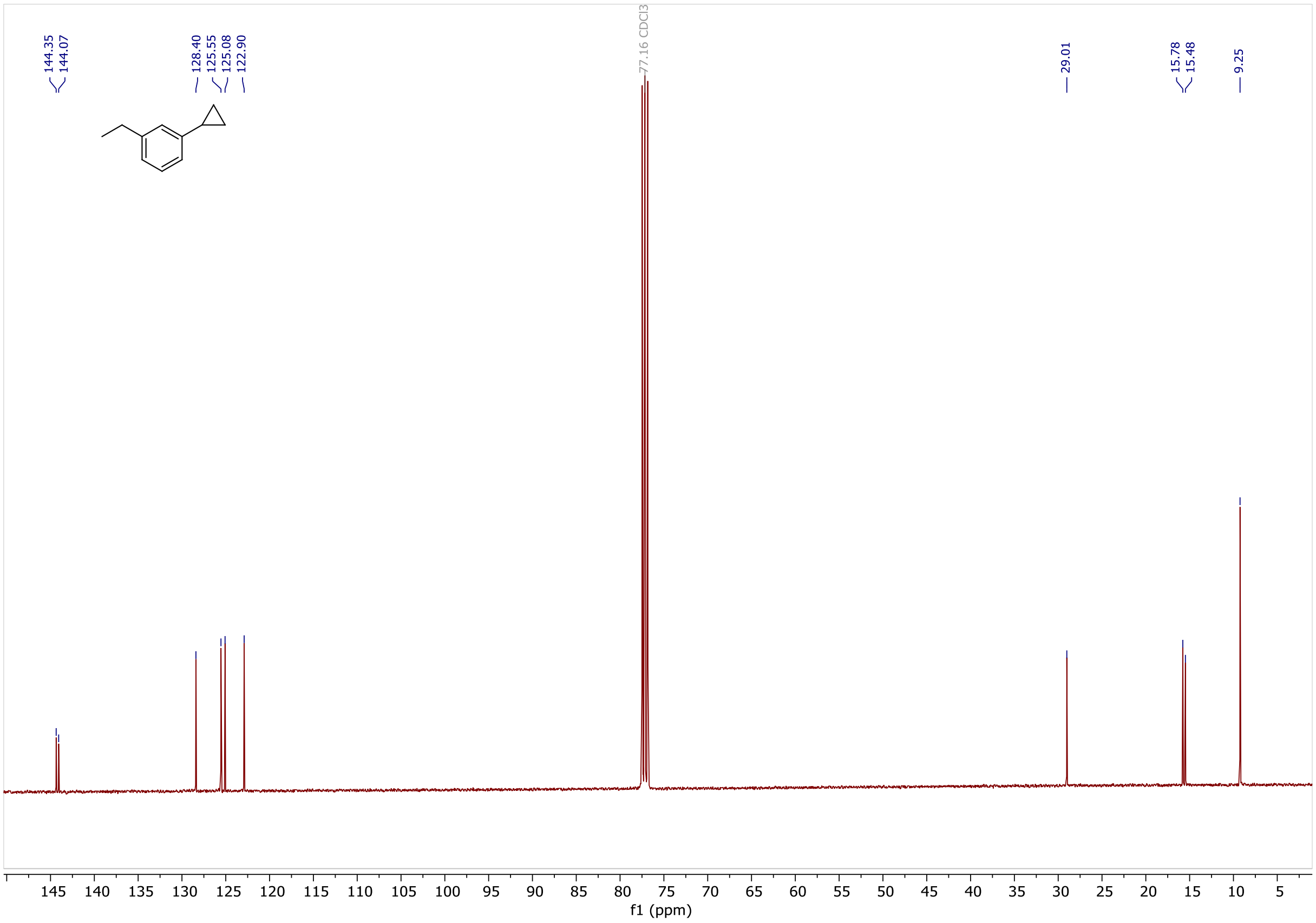
144.35
144.07
128.40
125.55
125.08
122.90
77.16 CDCl3
29.01
15.78
15.48
9.25
145 140 135 130 125 120 115 110 105 100 95 90 85 80 75 70 65 60 55 50 45 40 35 30 25 20 15 10 5
f1 (ppm)

^{1}H NMR Spectrum of *p*-5d (400 MHz, $CDCl_3$)

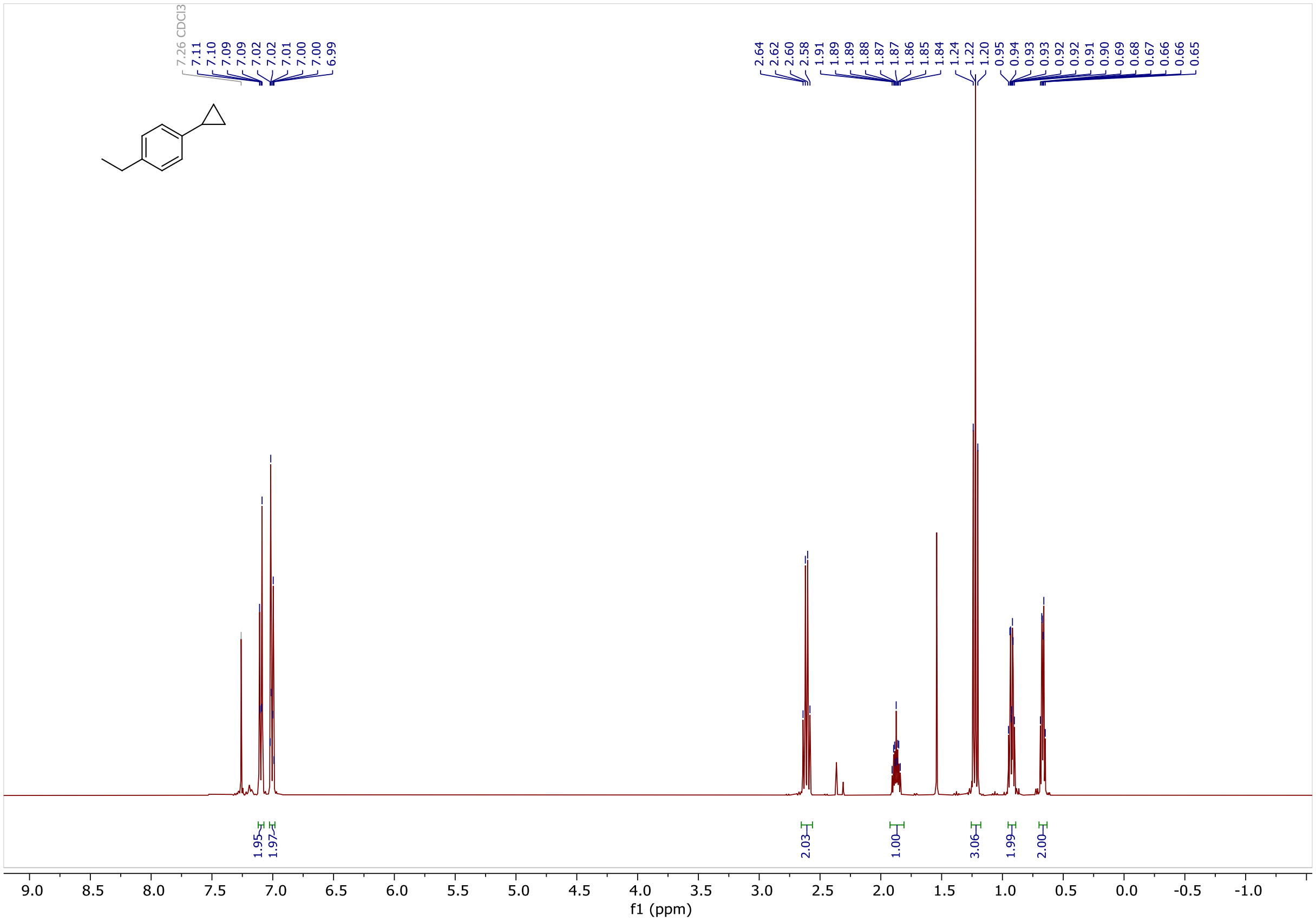

^{13}C NMR Spectrum of *p*-5d (101 MHz, $CDCl_3$)

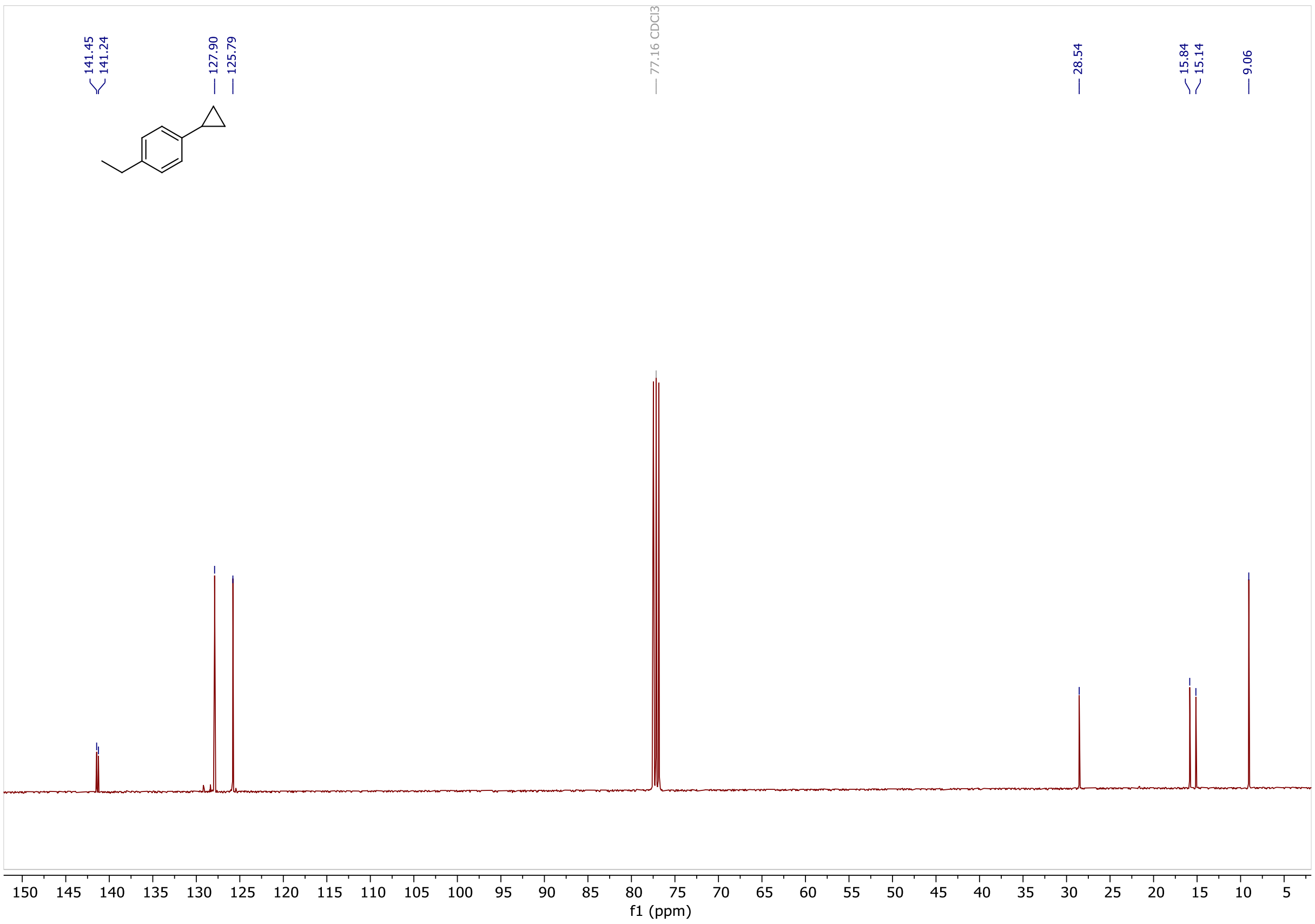

^{1}H NMR Spectrum of *m*-5e (400 MHz, CDCl3)

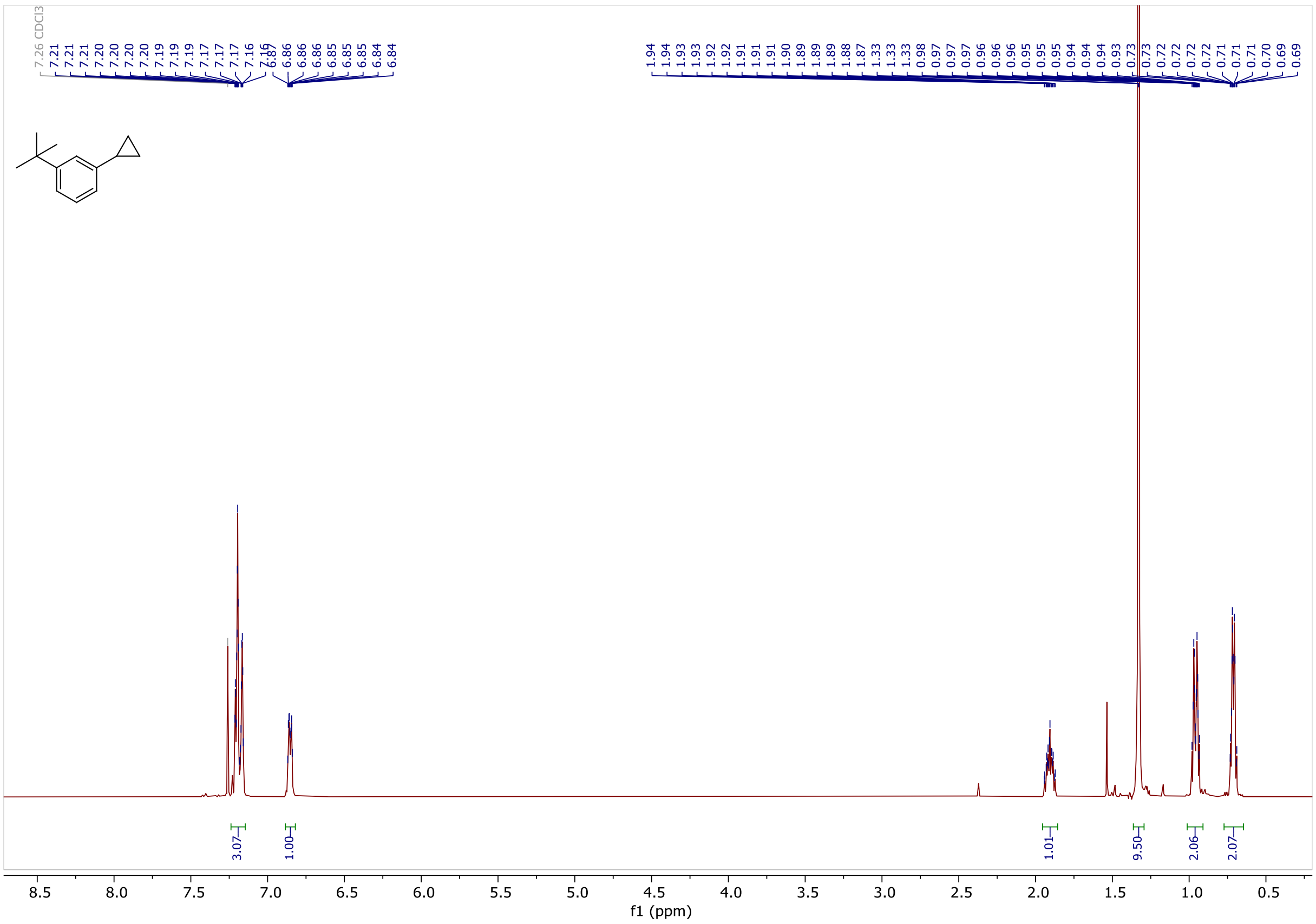

^{13}C NMR Spectrum of *m*-5e (101 MHz, CDCl3)

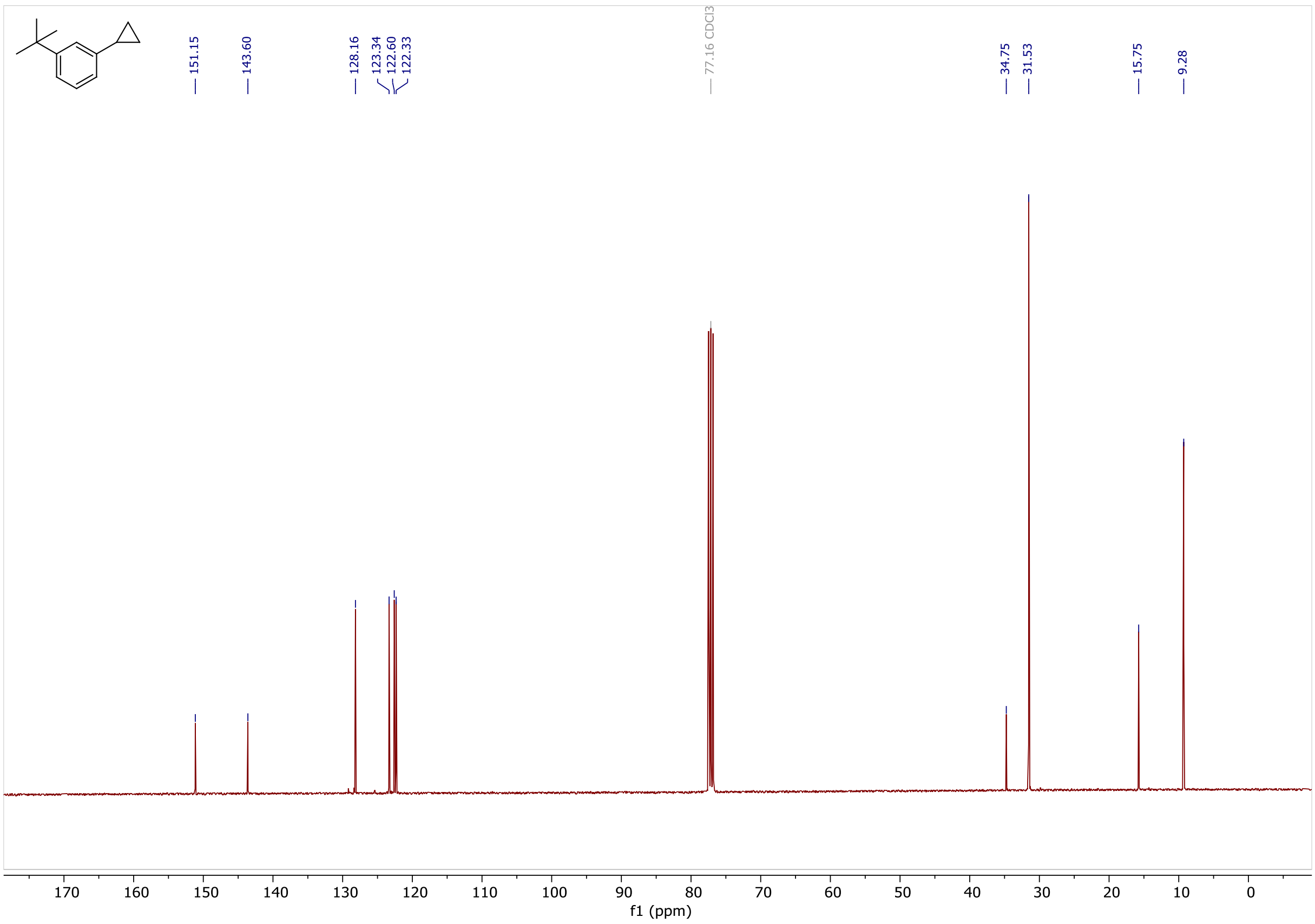

^{1}H NMR Spectrum of *p*-5e (400 MHz, $CDCl_3$)

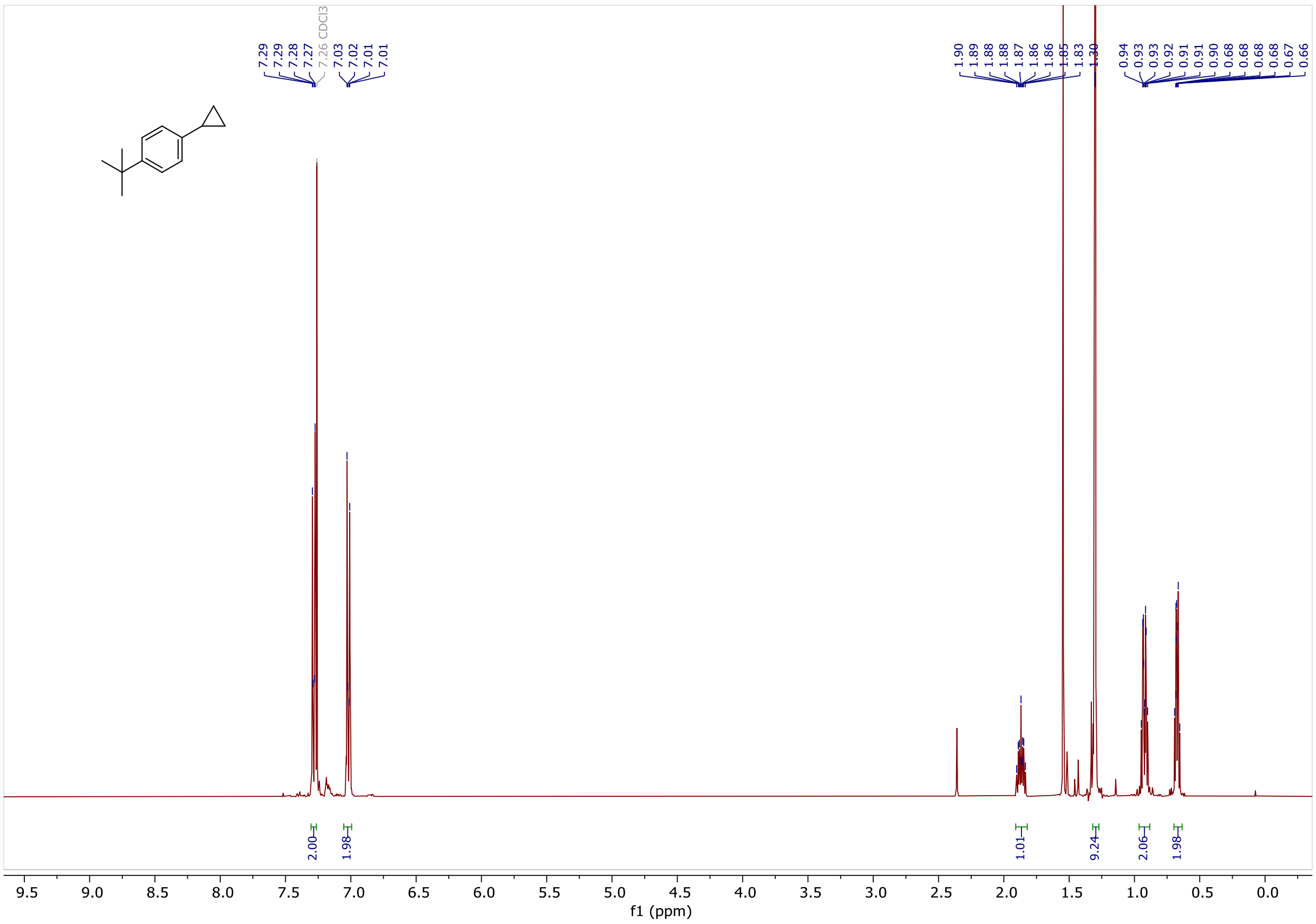

^{13}C NMR Spectrum of *p*-5e (101 MHz, $CDCl_3$)

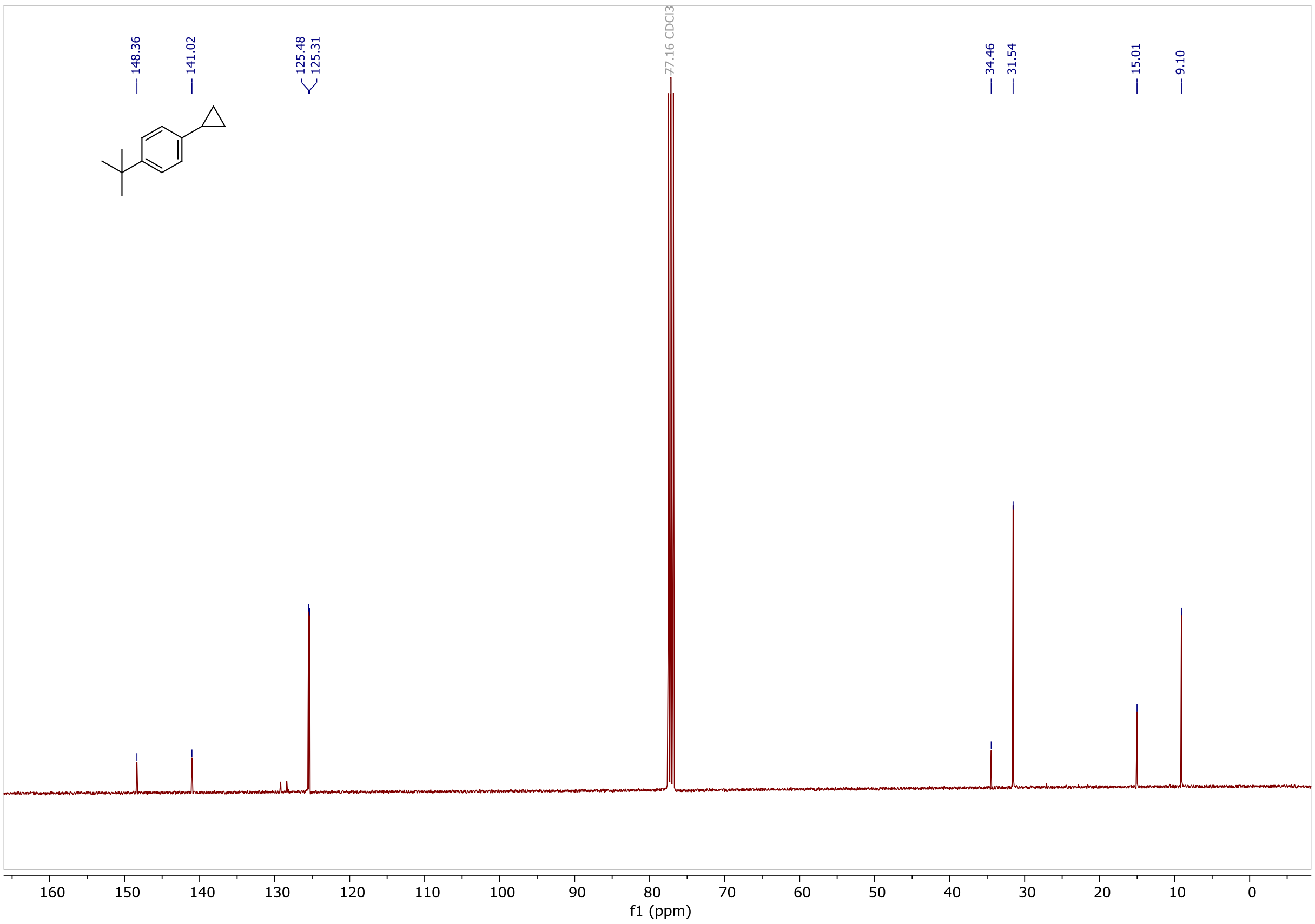

^{1}H NMR Spectrum of 5f (400 MHz, $CDCl_3$)

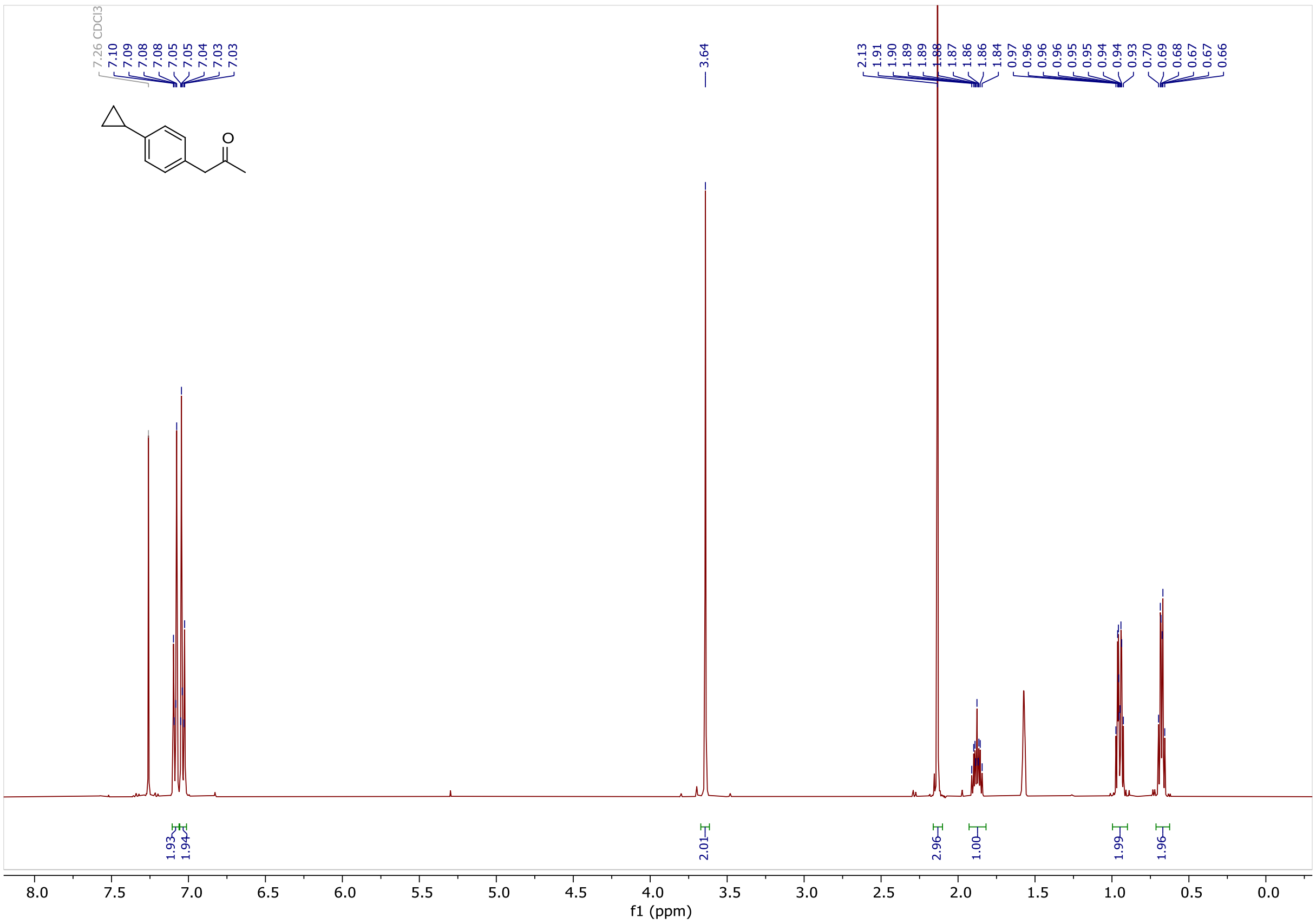

^{13}C NMR Spectrum of 5f (101 MHz, $CDCl_3$)

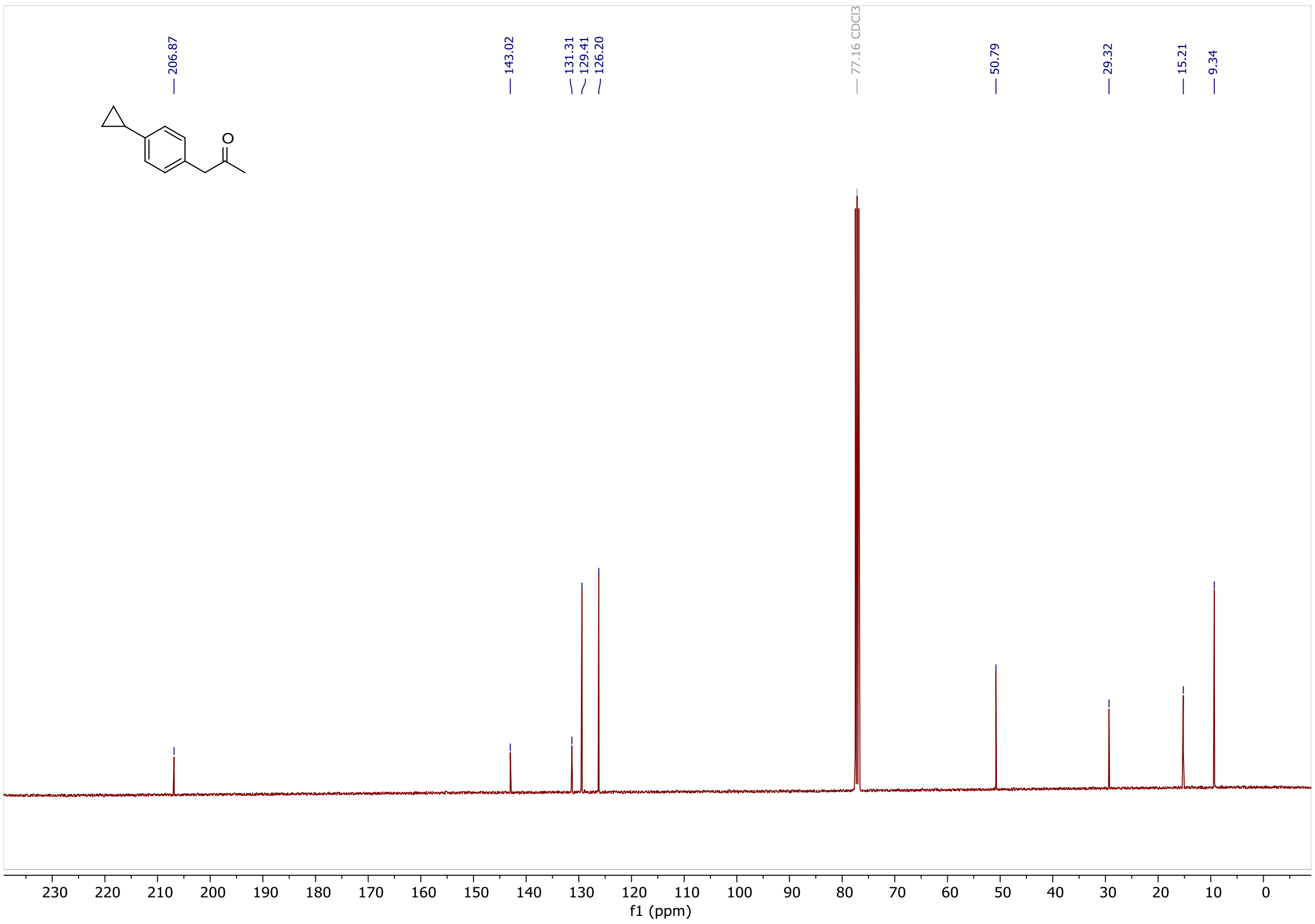

^{1}H NMR Spectrum of *o*-5g (400 MHz, $CDCl_3$)

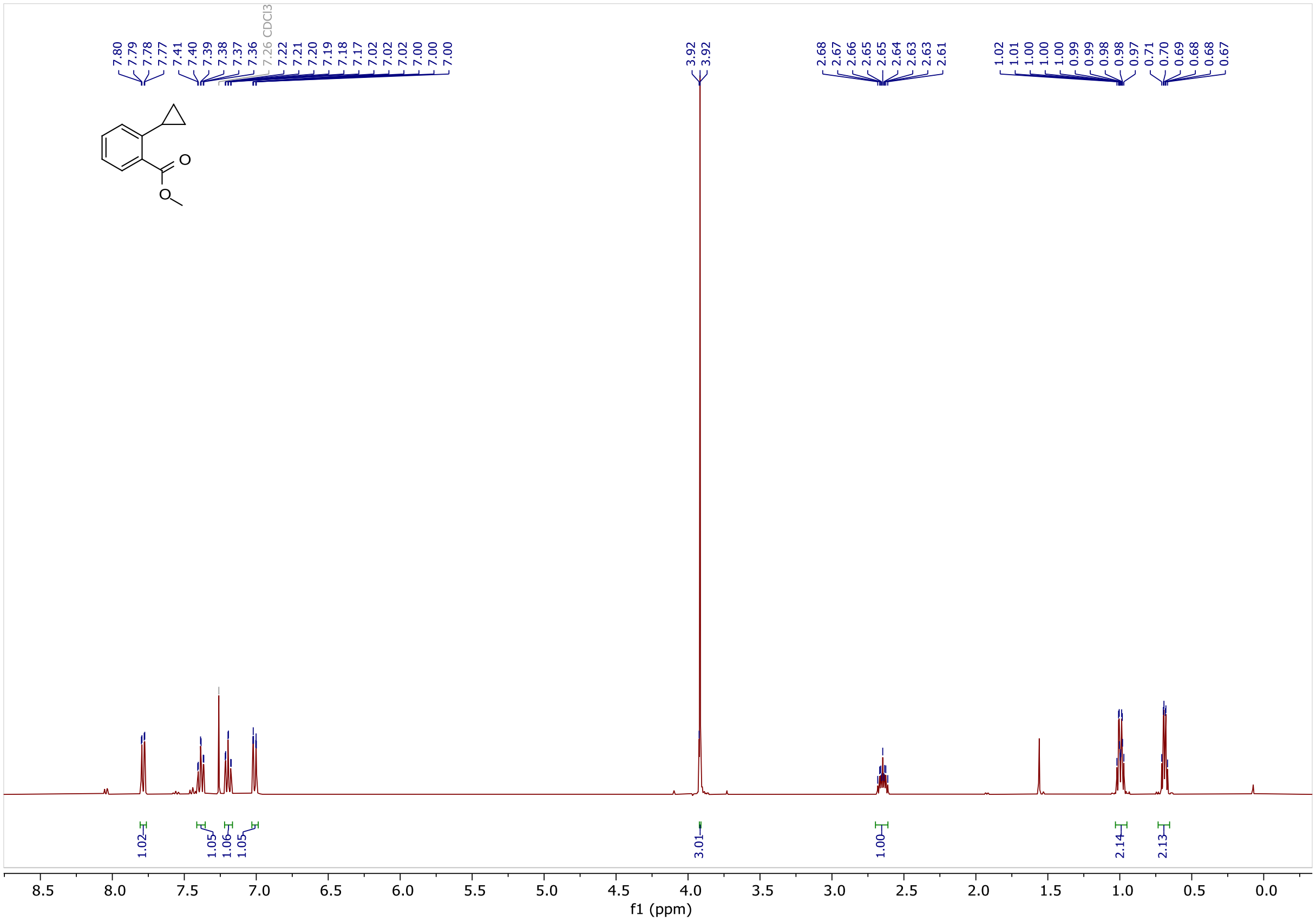

^{13}C NMR Spectrum of *o*-5g (101 MHz, $CDCl_3$)

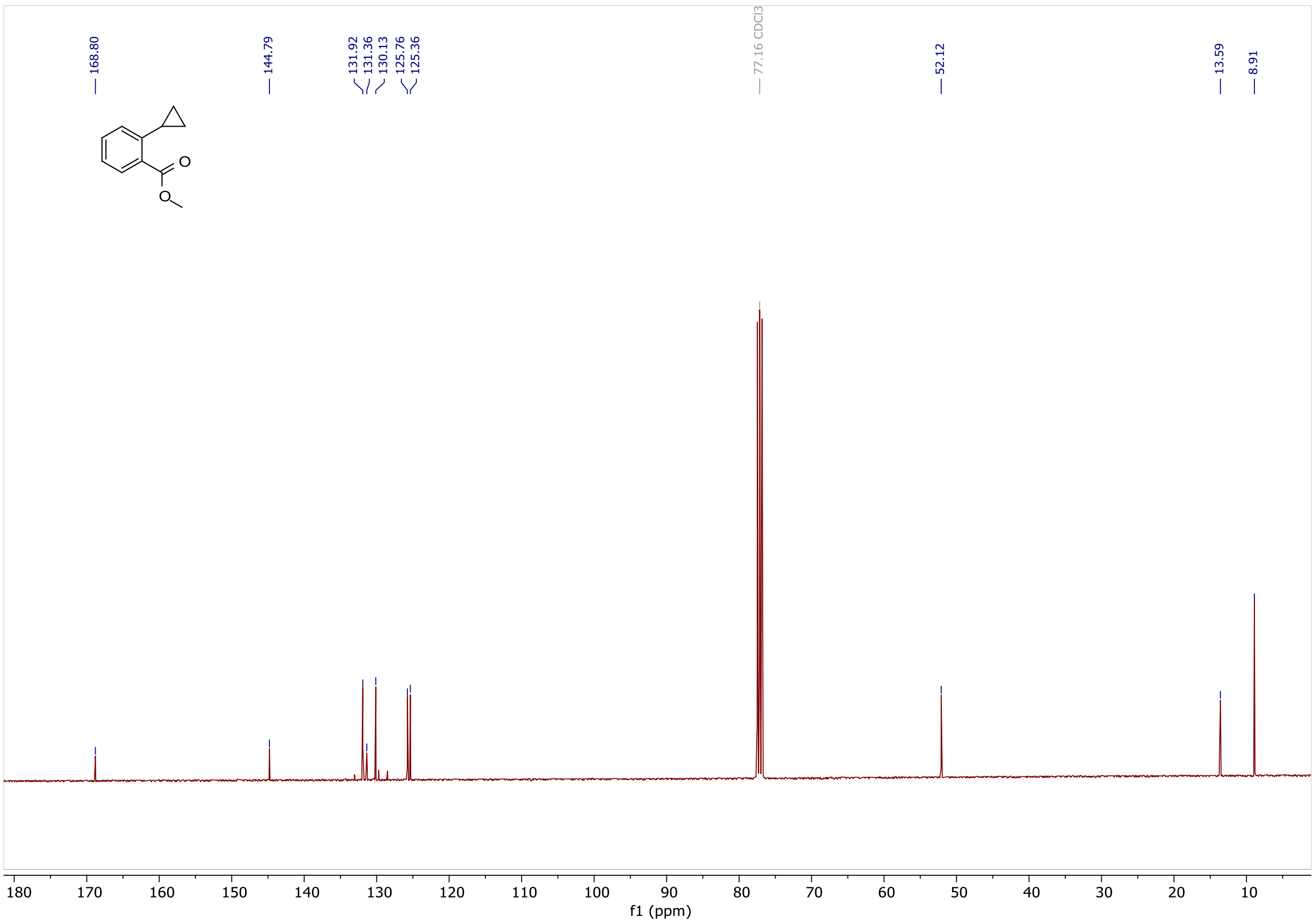

^{1}H NMR Spectrum of *m*-5g (400 MHz, $CDCl_3$)

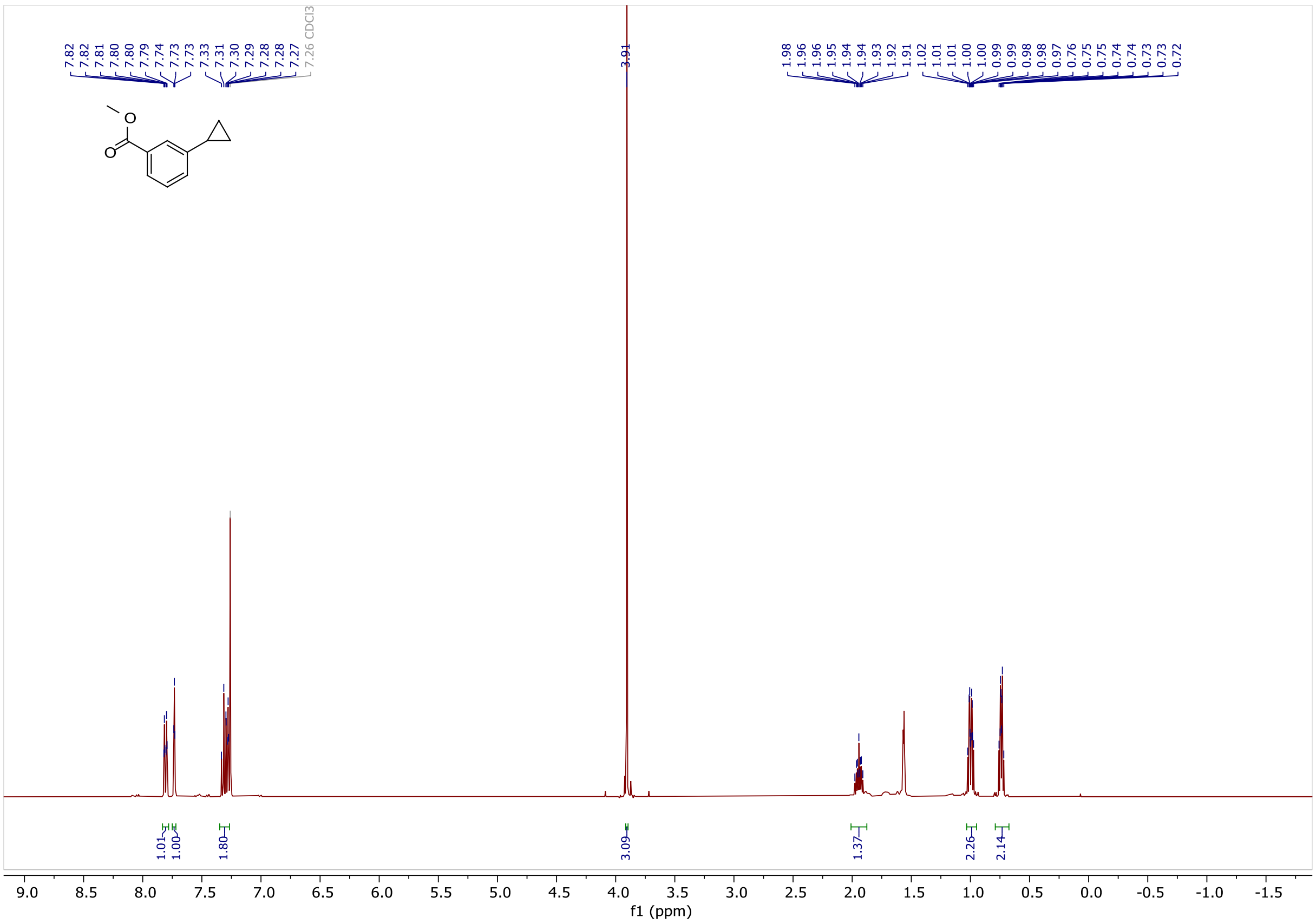

^{13}C NMR Spectrum of *m*-5g (101 MHz, $CDCl_3$)

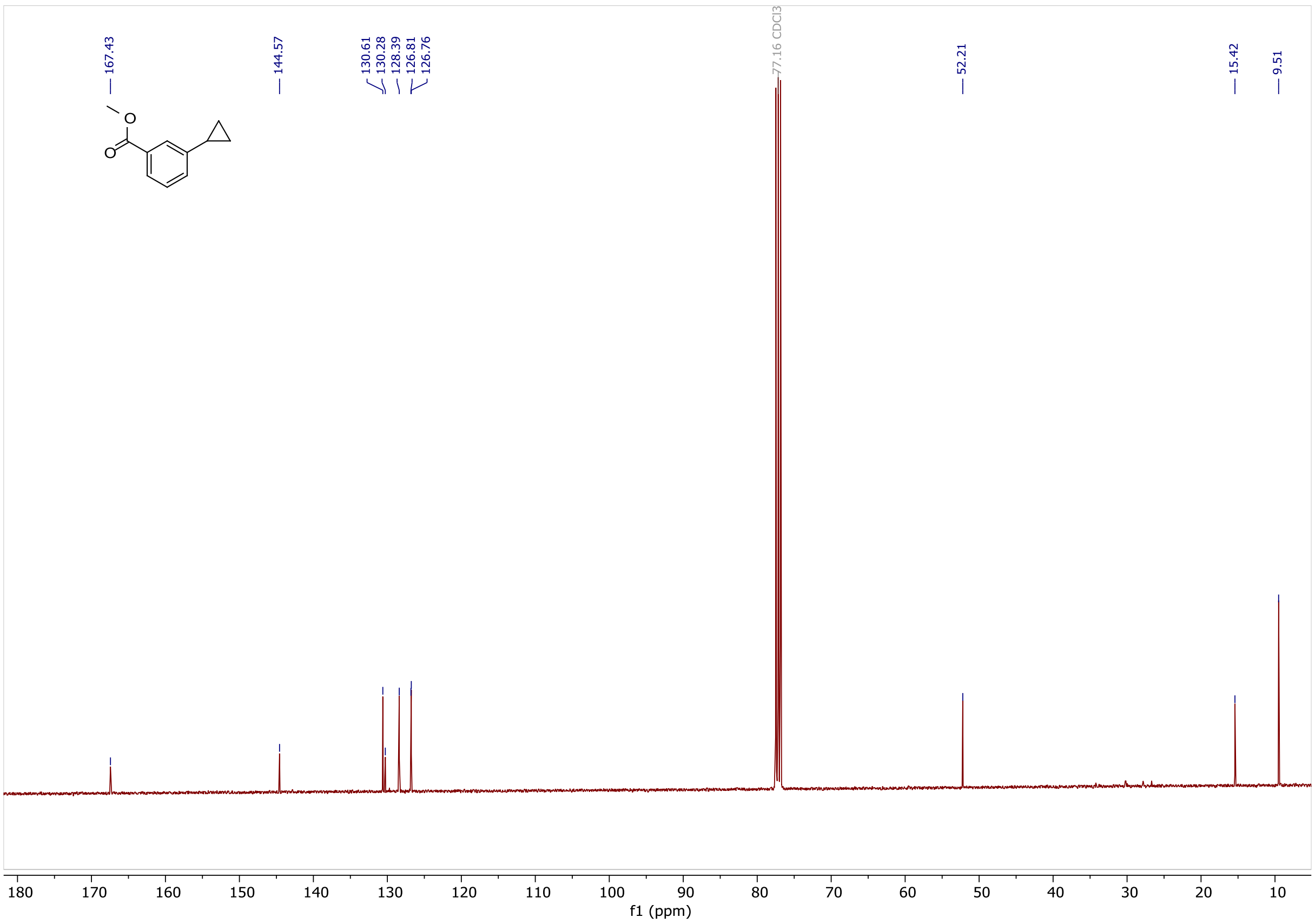

^{1}H NMR Spectrum of *p*-5g (400 MHz, $CDCl_3$)

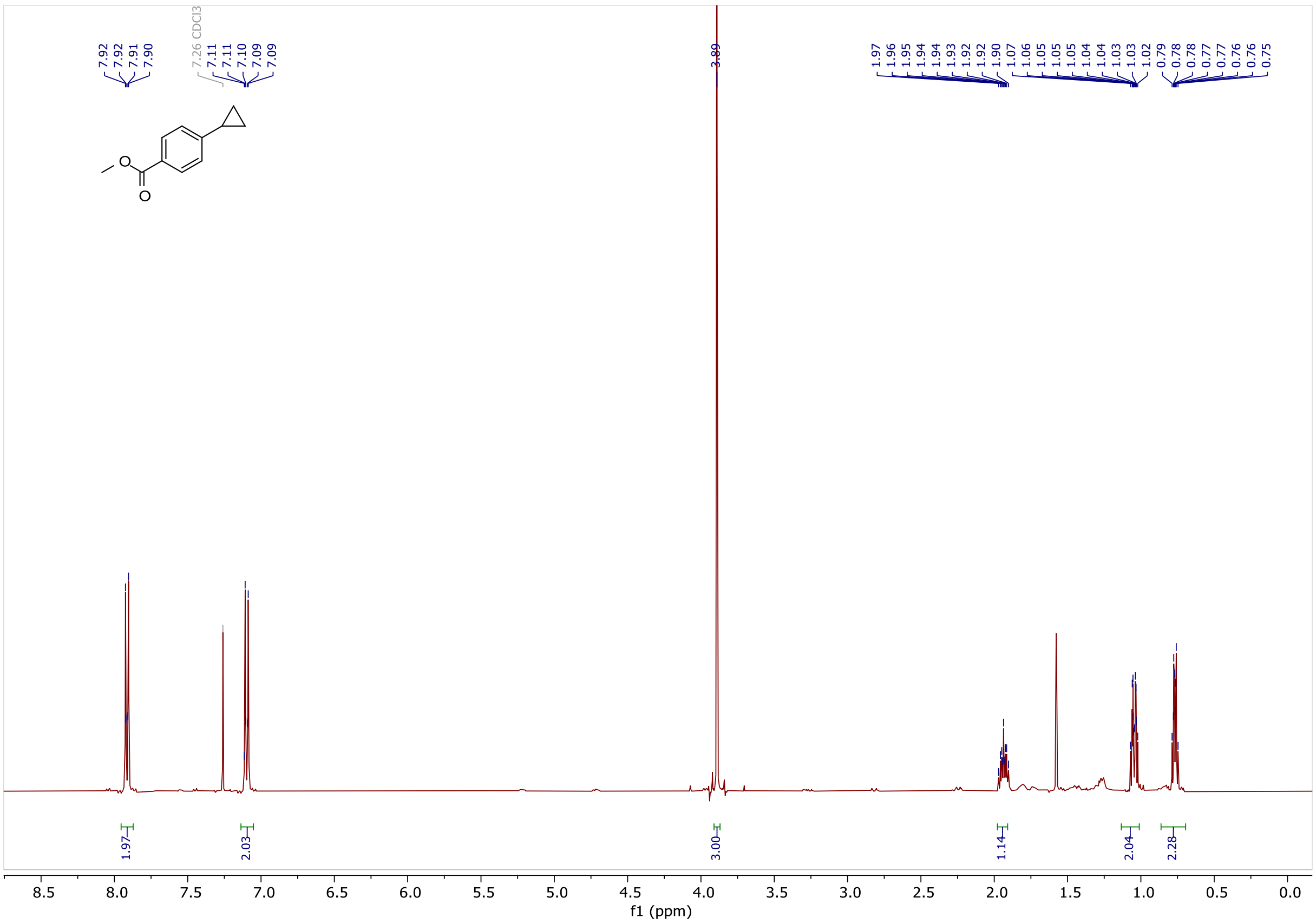

^{13}C NMR Spectrum of *p*-5g (101 MHz, $CDCl_3$)

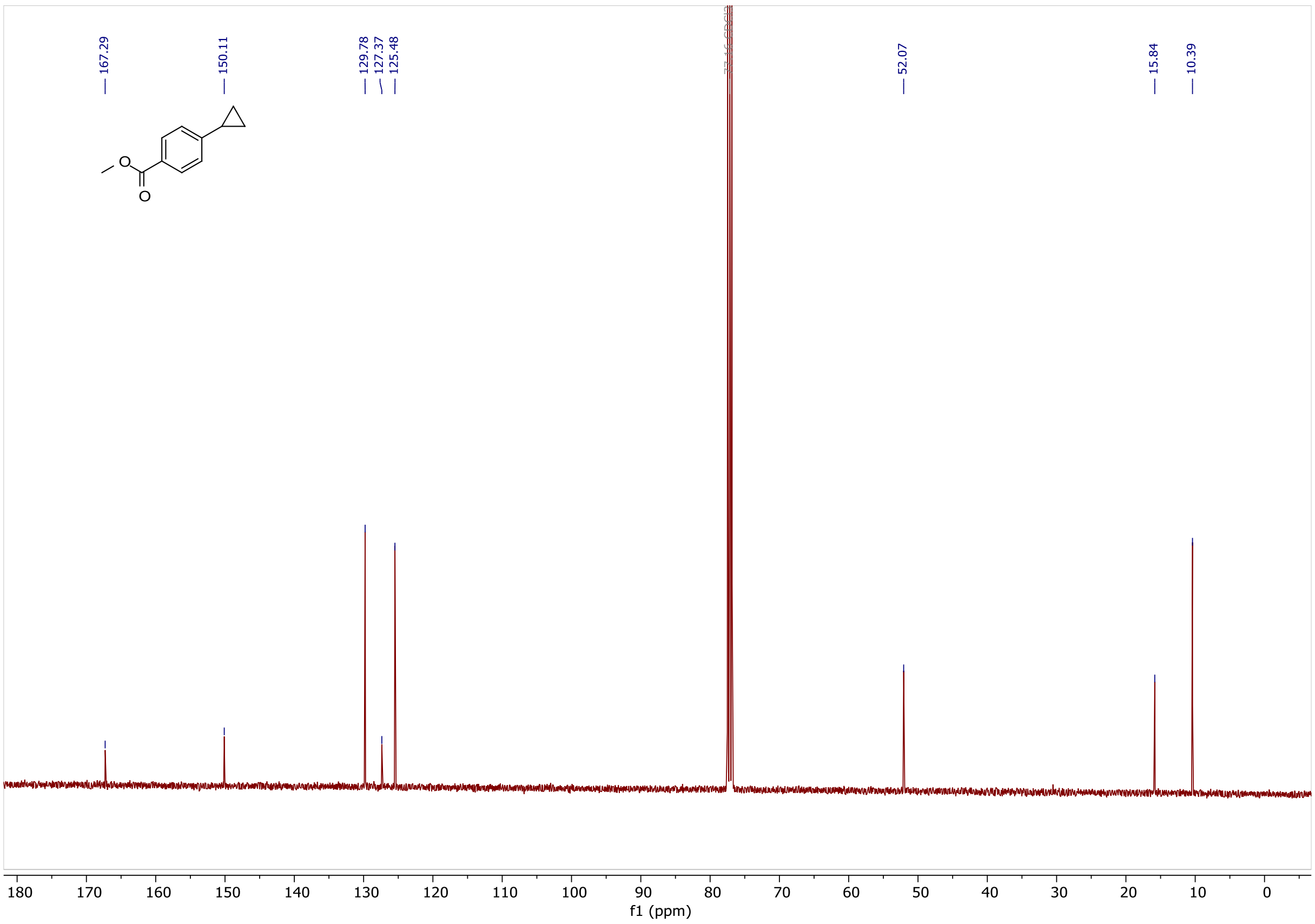

^{1}H NMR Spectrum of *o*-5h (400 MHz, $CDCl_3$)

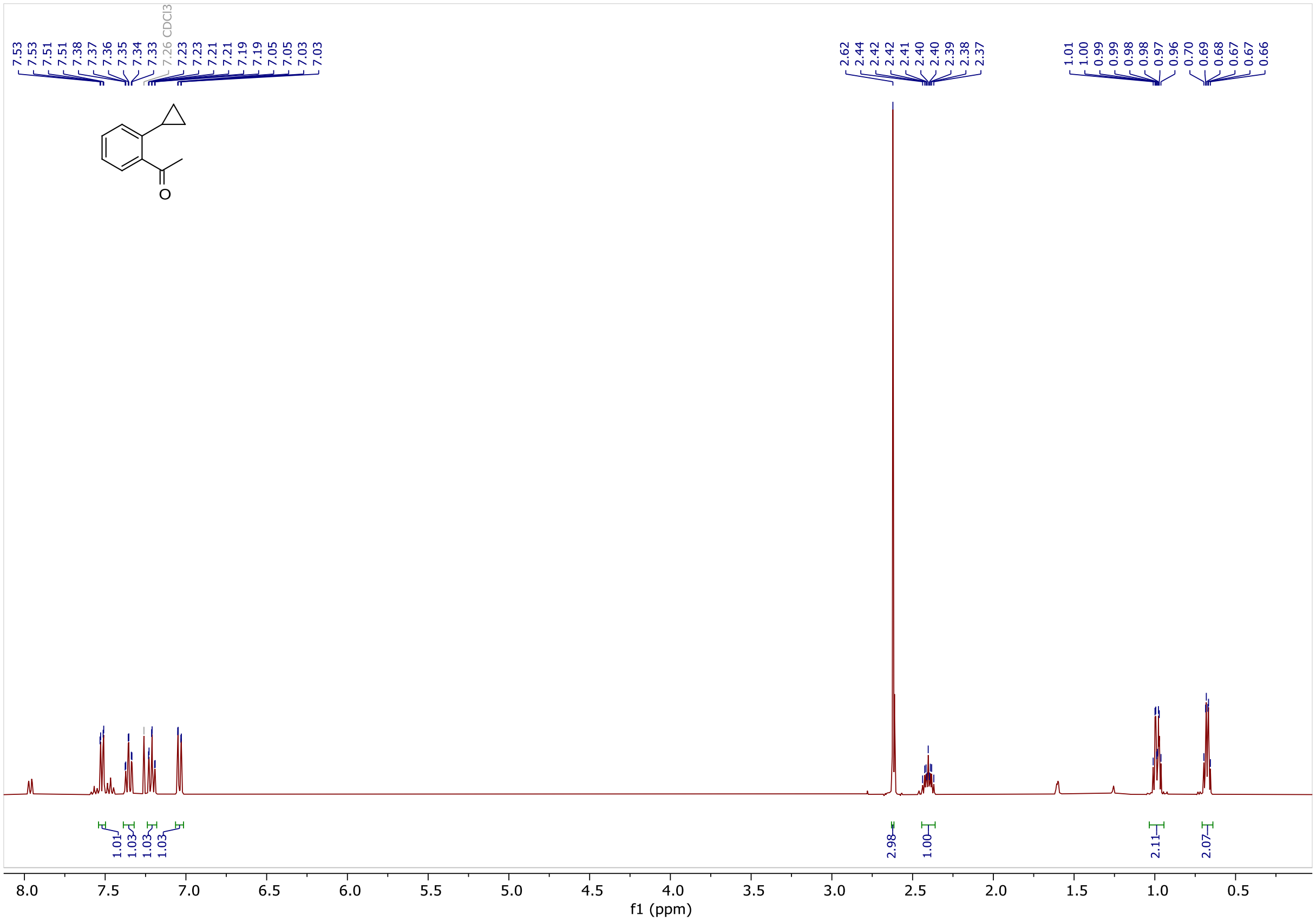

^{13}C NMR Spectrum of *o*-5h (101 MHz, $CDCl_3$)

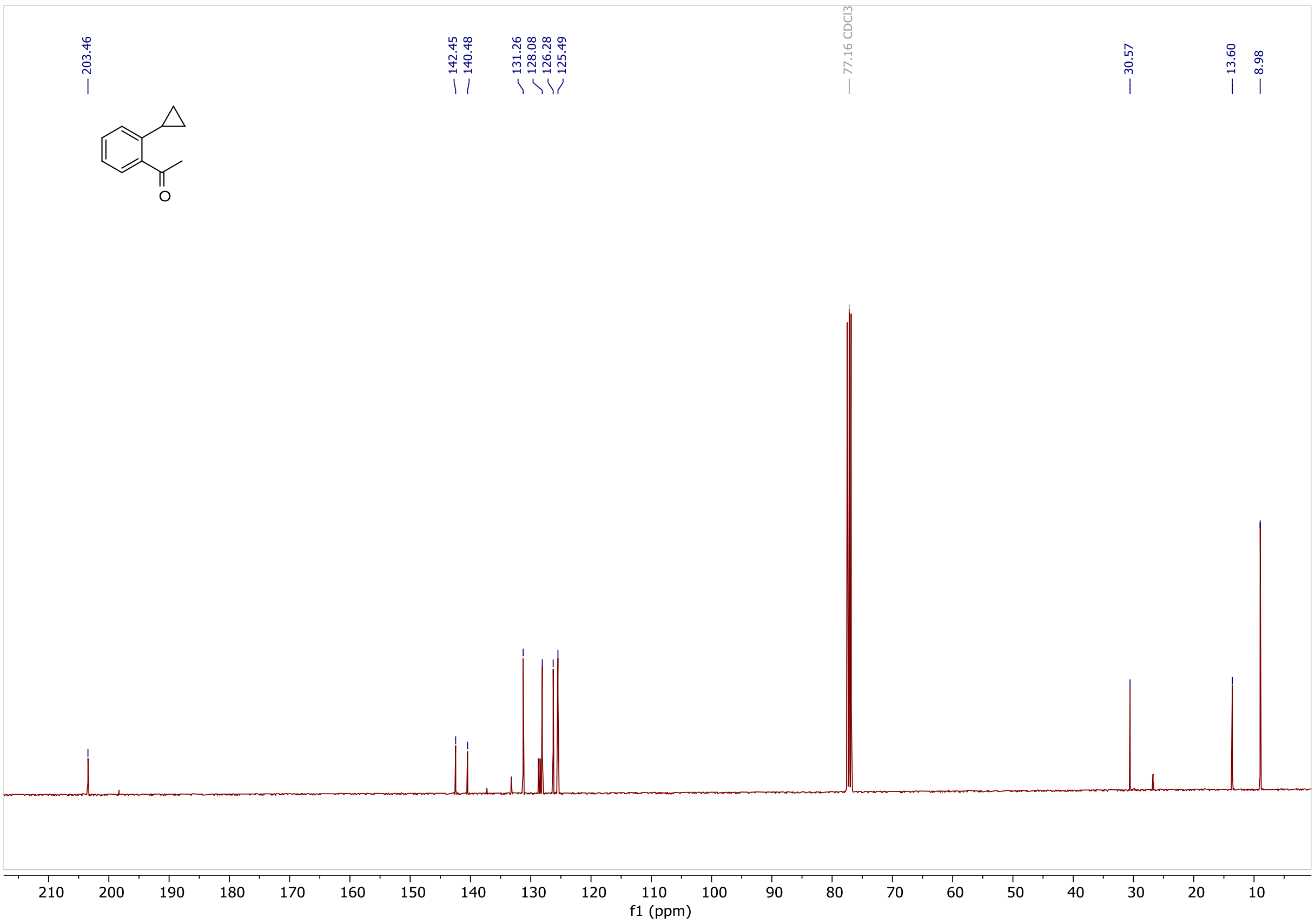

^{1}H NMR Spectrum of *m*-5h (400 MHz, $CDCl_3$)

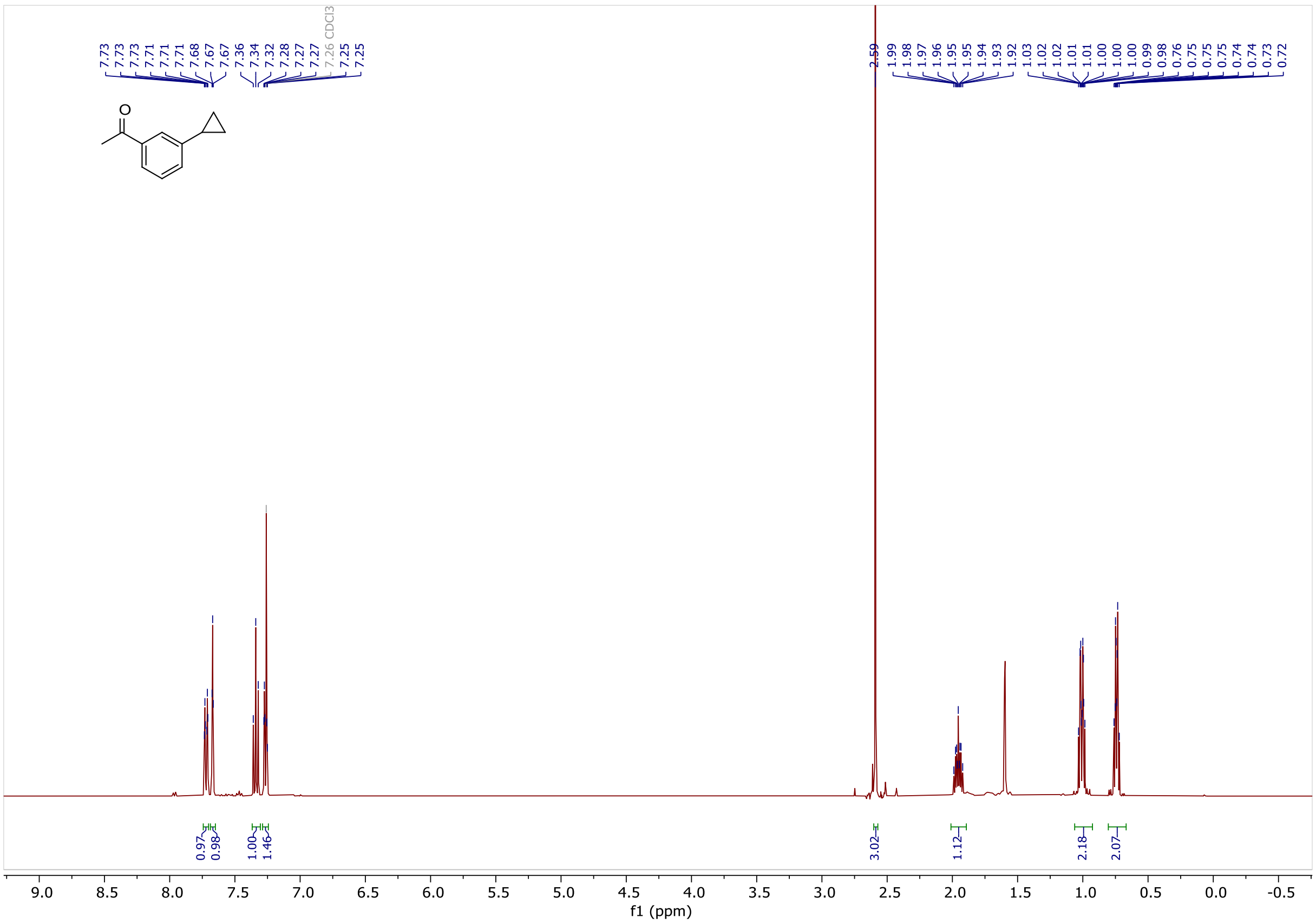

^{13}C NMR Spectrum of *m*-5h (101 MHz, $CDCl_3$)

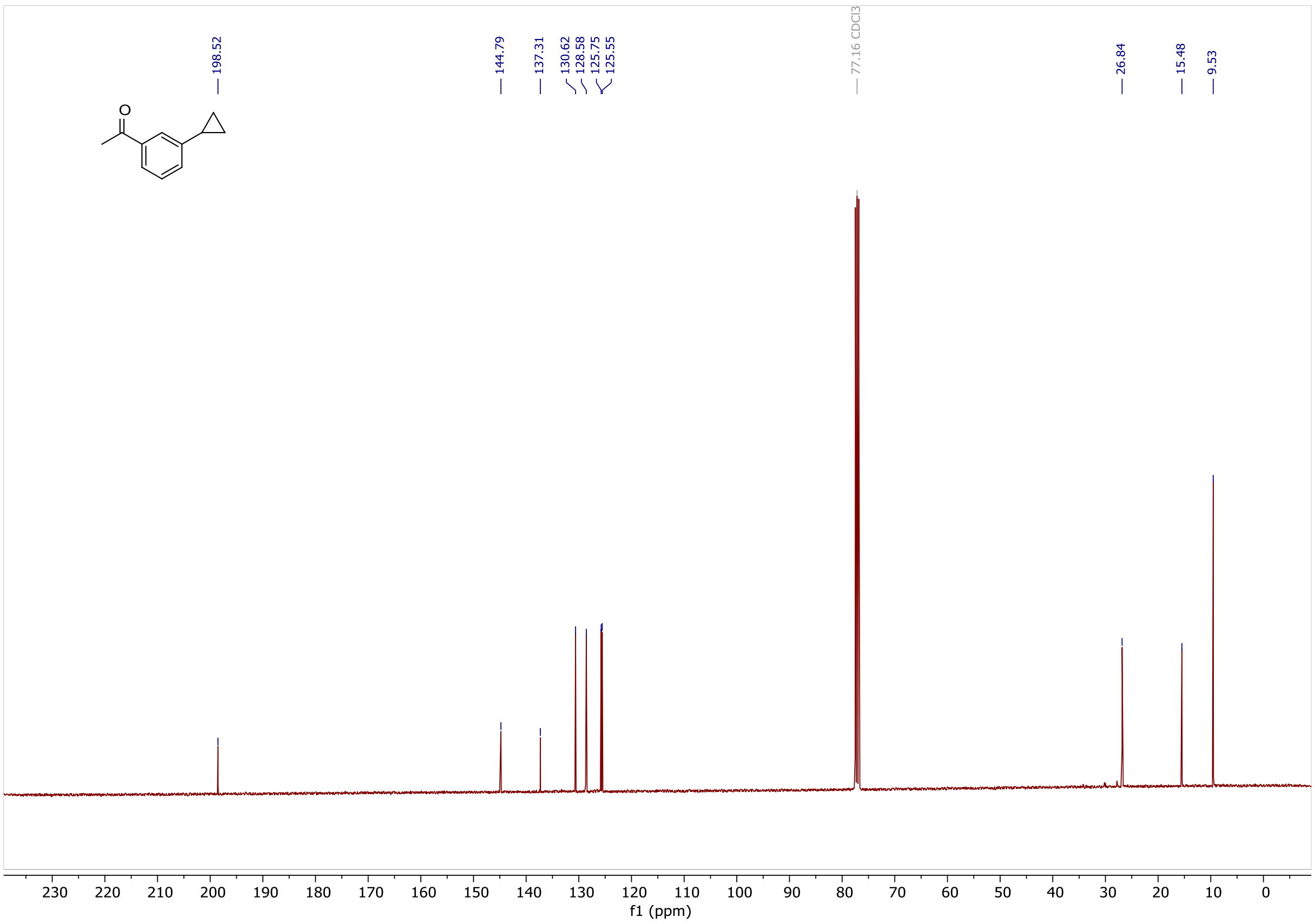

^{1}H NMR Spectrum of *p*-5h (400 MHz, $CDCl_3$)

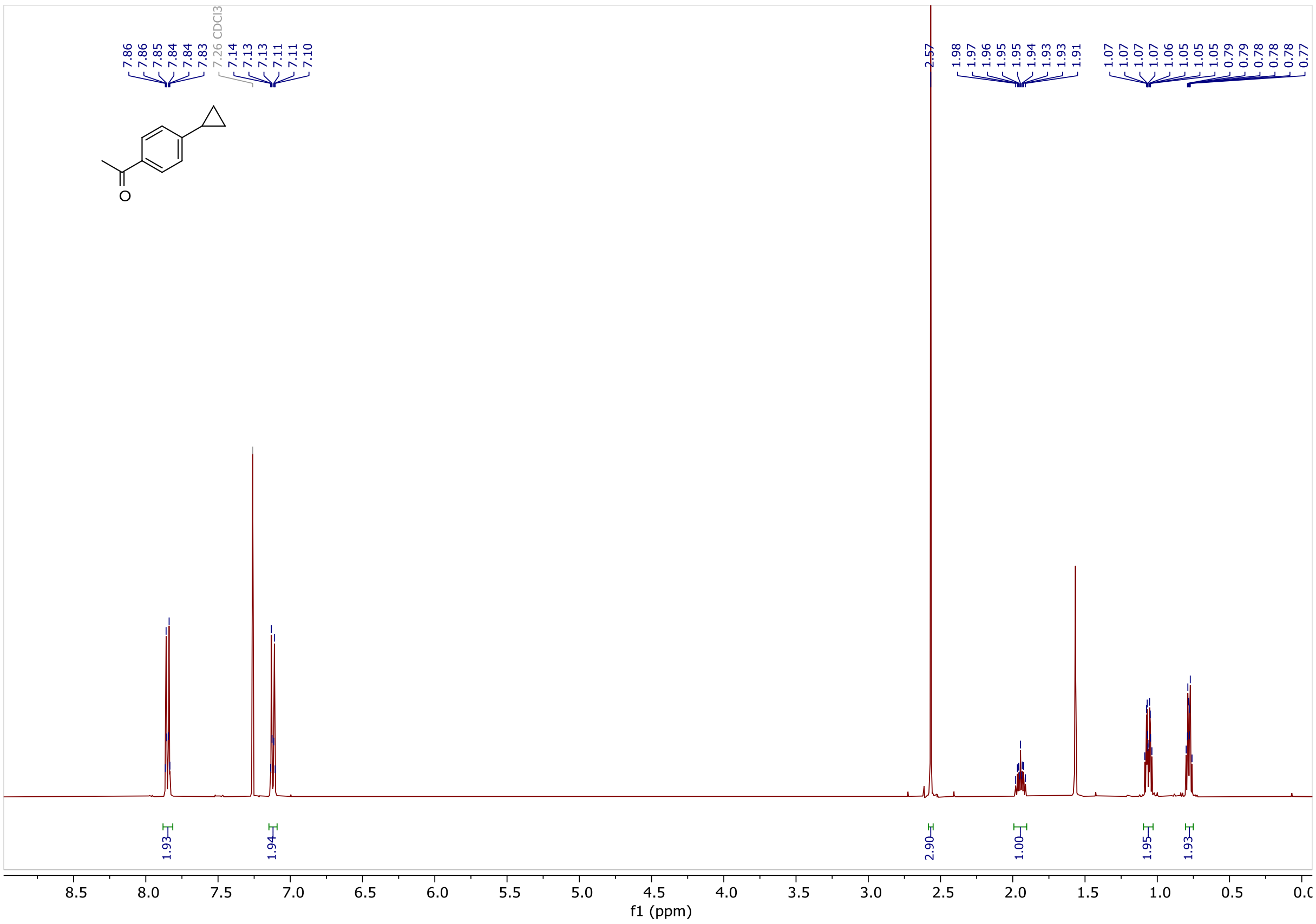

^{13}C NMR Spectrum of *p*-5h (101 MHz, $CDCl_3$)

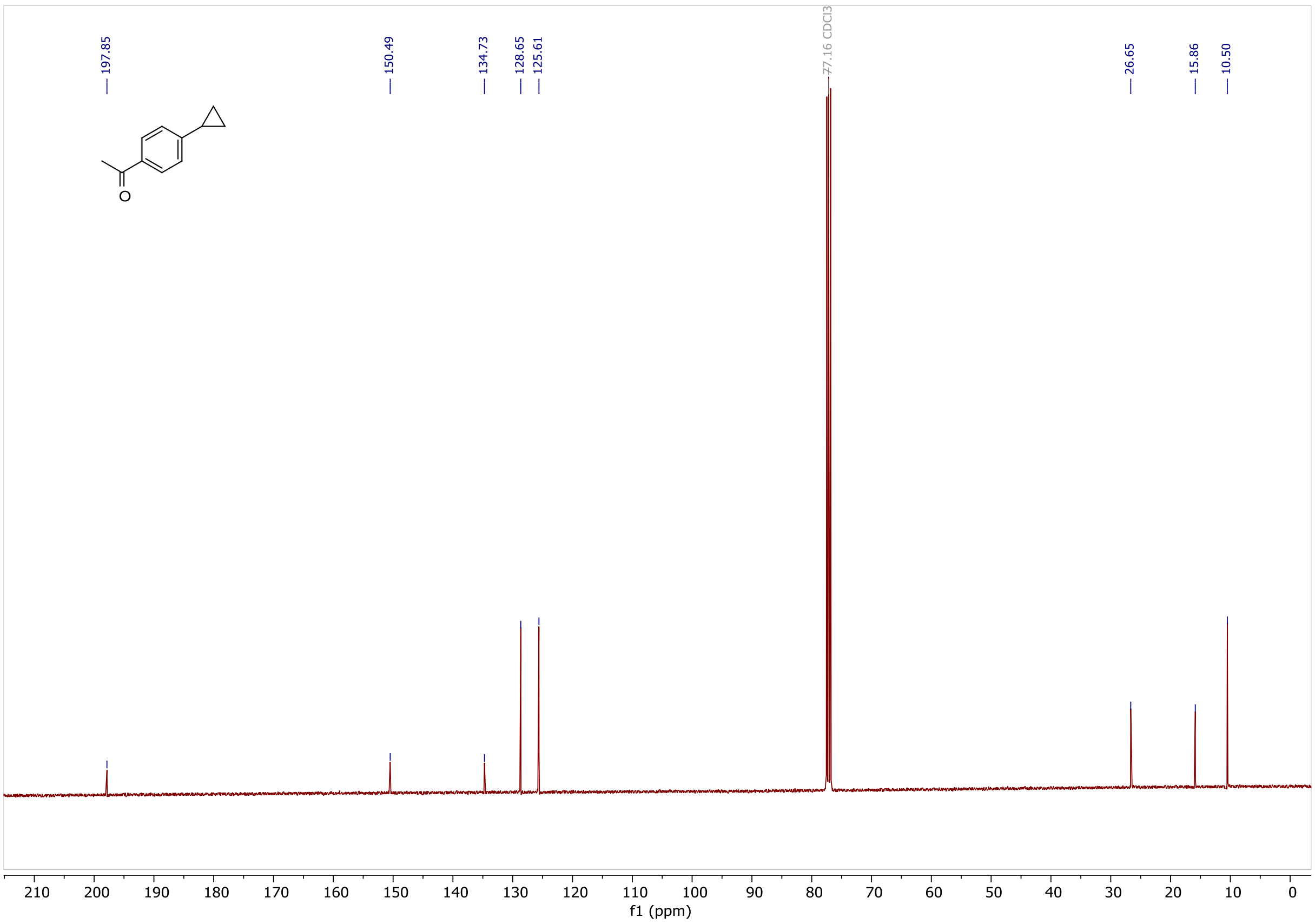

^{1}H NMR Spectrum of *o*-5i (400 MHz, $CDCl_3$)

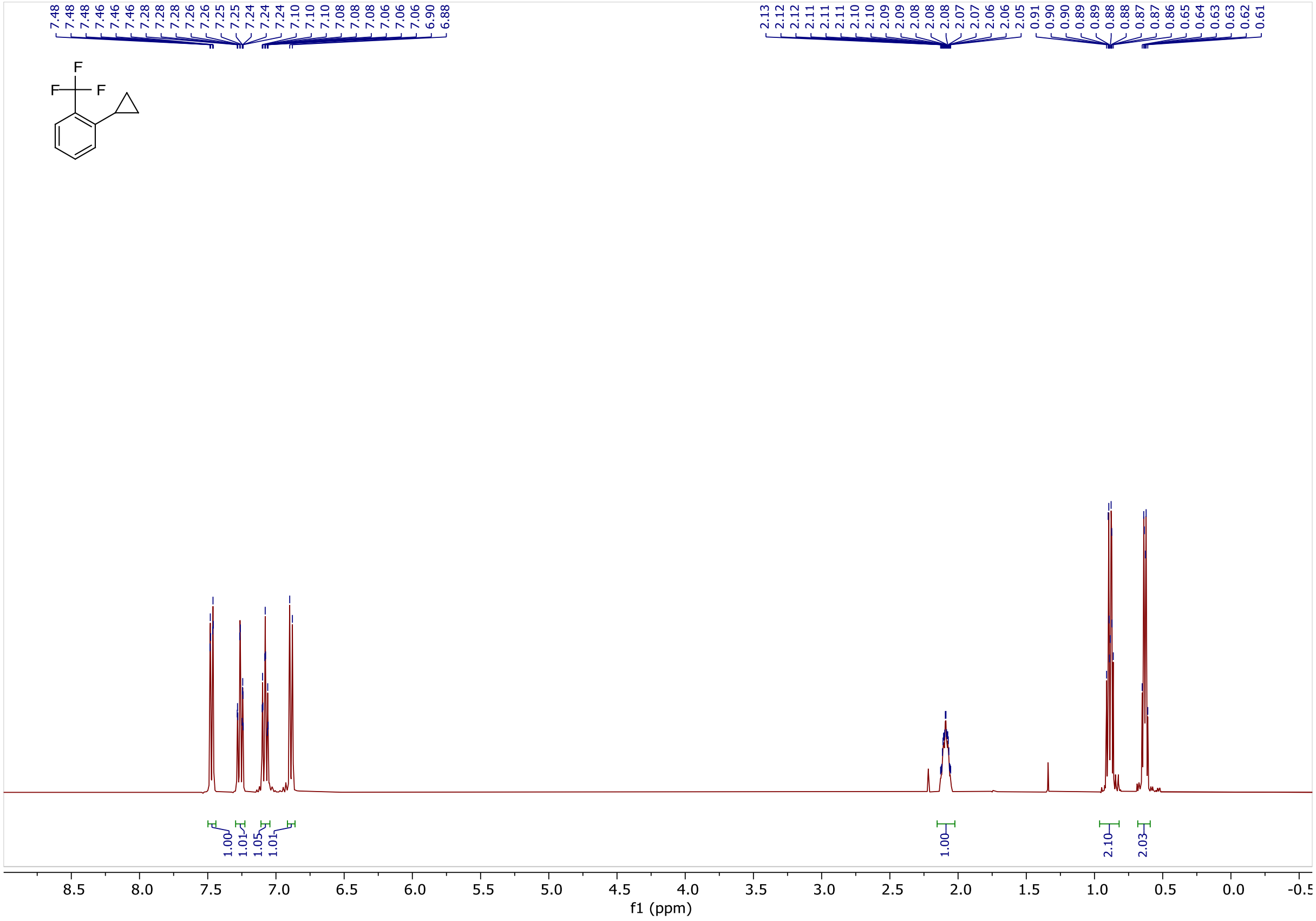

^{19}F NMR Spectrum of *o*-5i (376 MHz, $CDCl_3$)

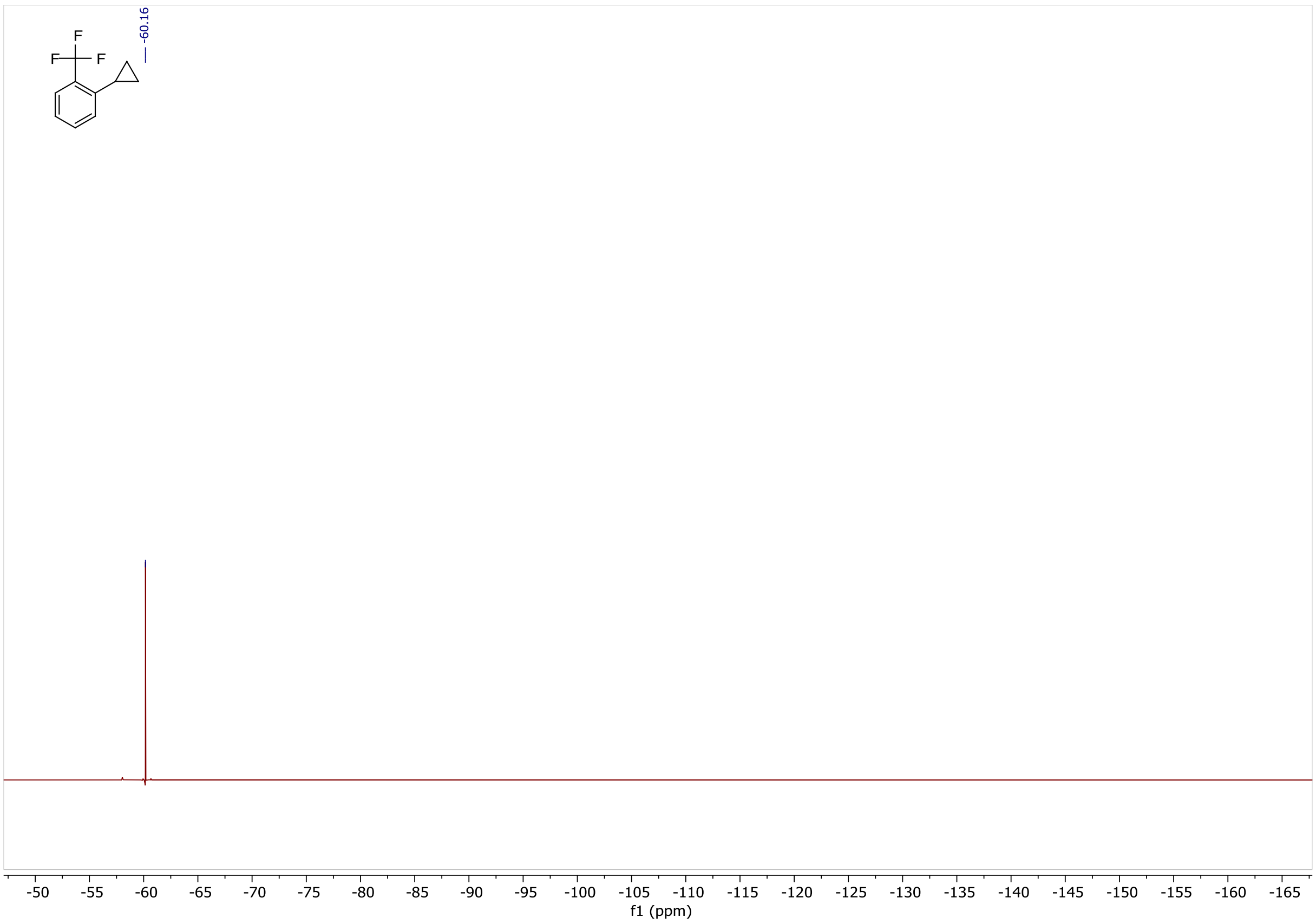

^{13}C NMR Spectrum of *o*-5i (101 MHz, $CDCl_3$)

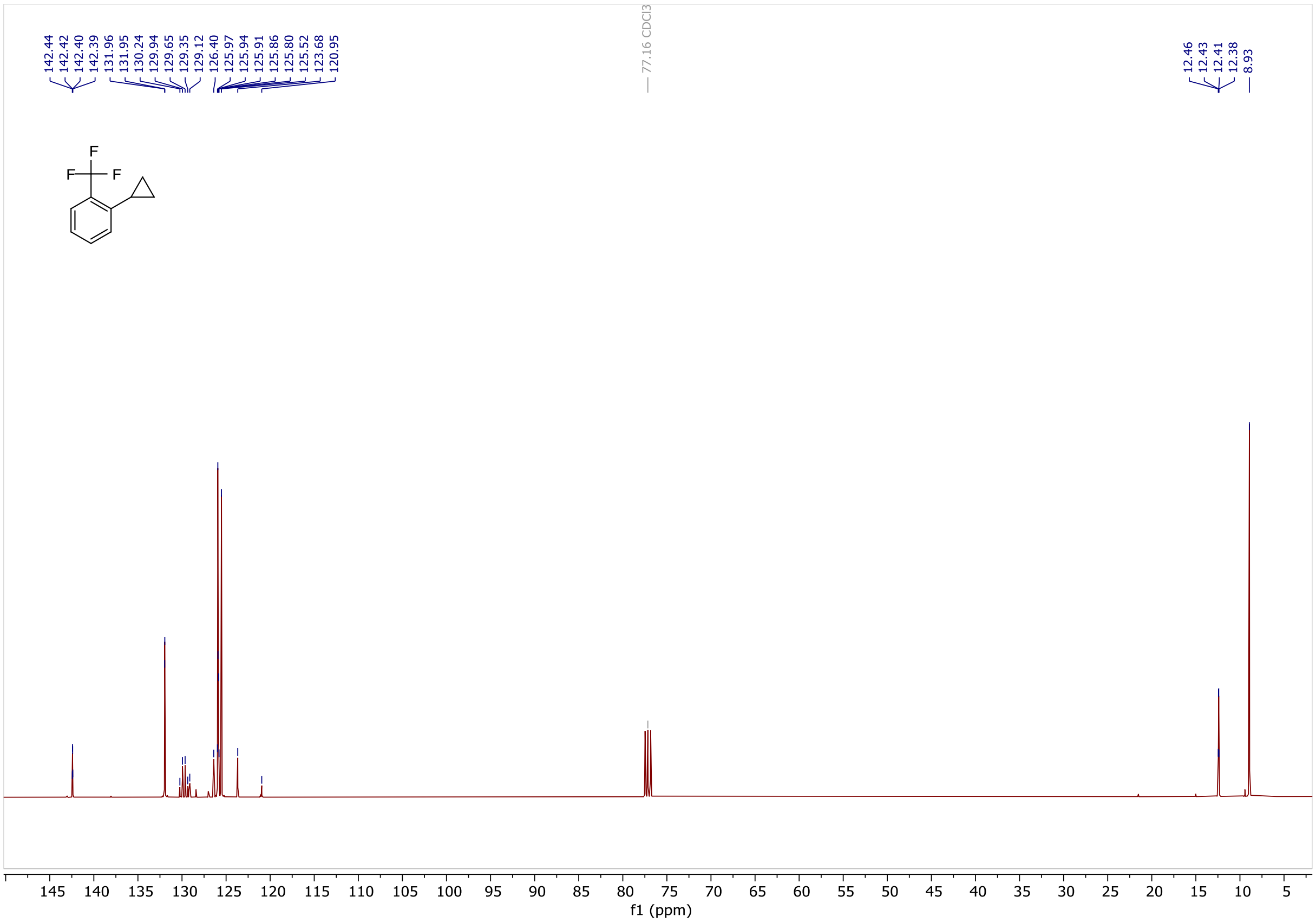

^{1}H NMR Spectrum of *m*-5i (400 MHz, $CDCl_3$)

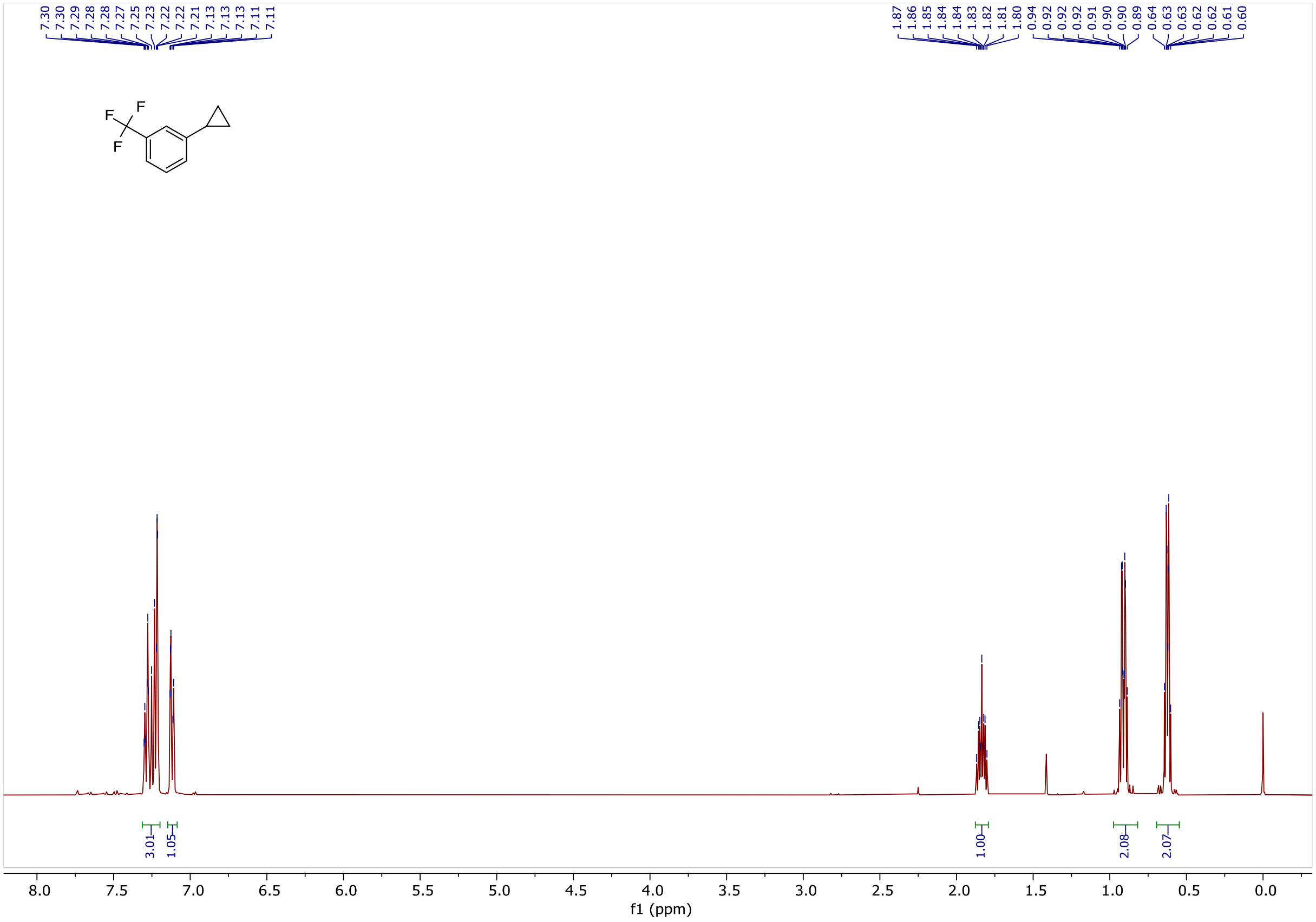

^{19}F NMR Spectrum of *m*-5i (376 MHz, $CDCl_3$)

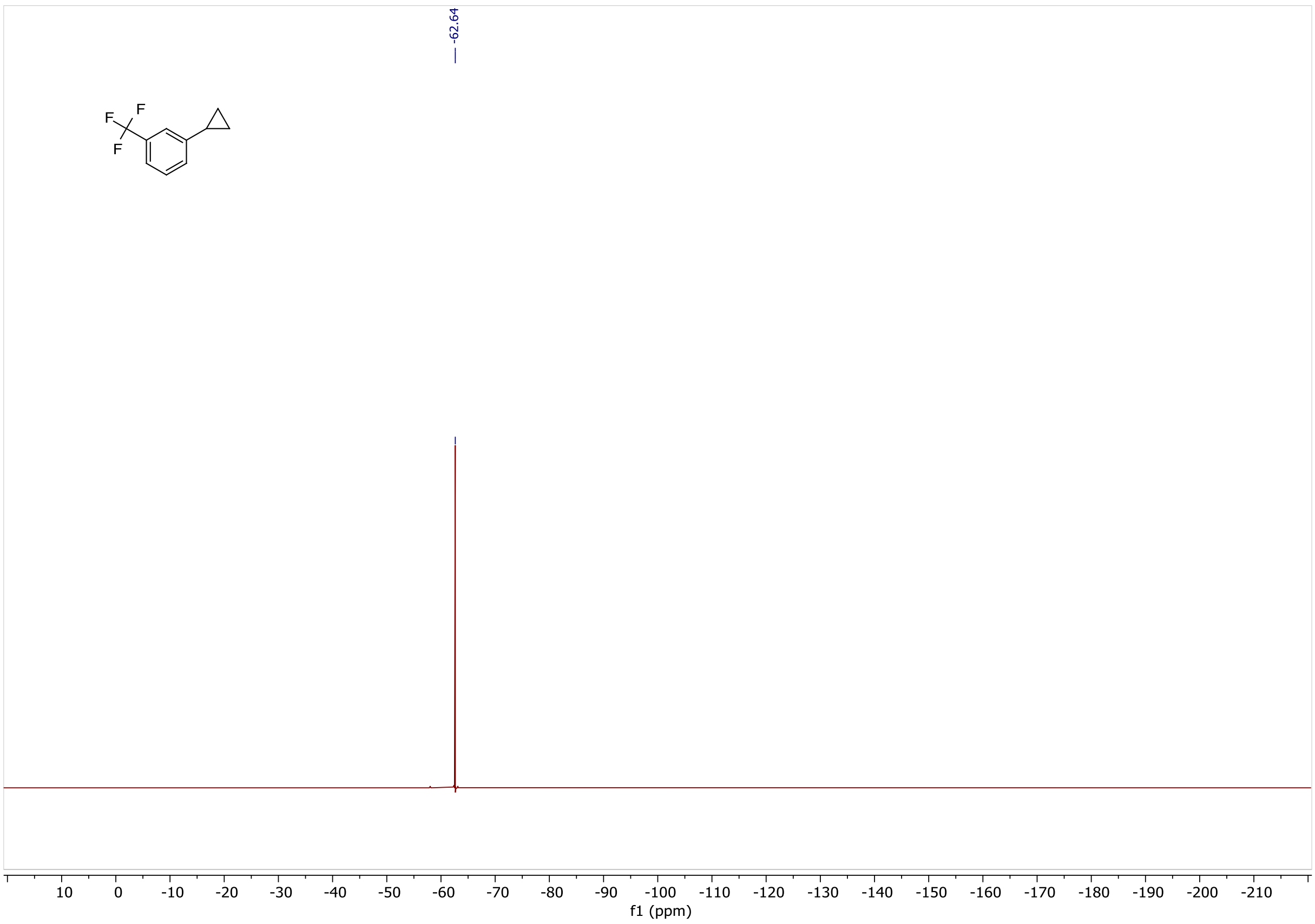

^{13}C NMR Spectrum of *m*-5i (101 MHz, $CDCl_3$)

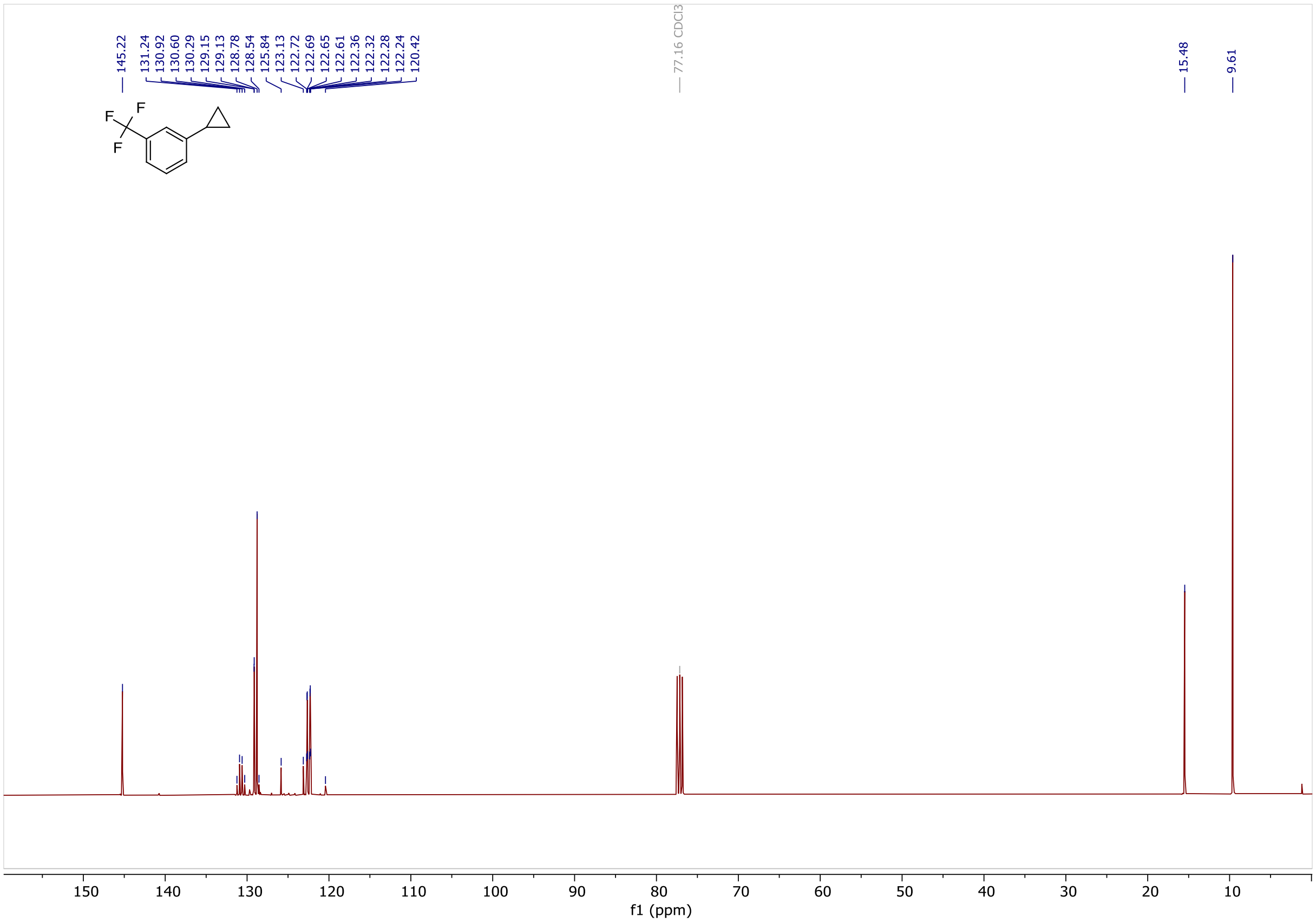

^{1}H NMR Spectrum of *p*-5i (400 MHz, $CDCl_3$)

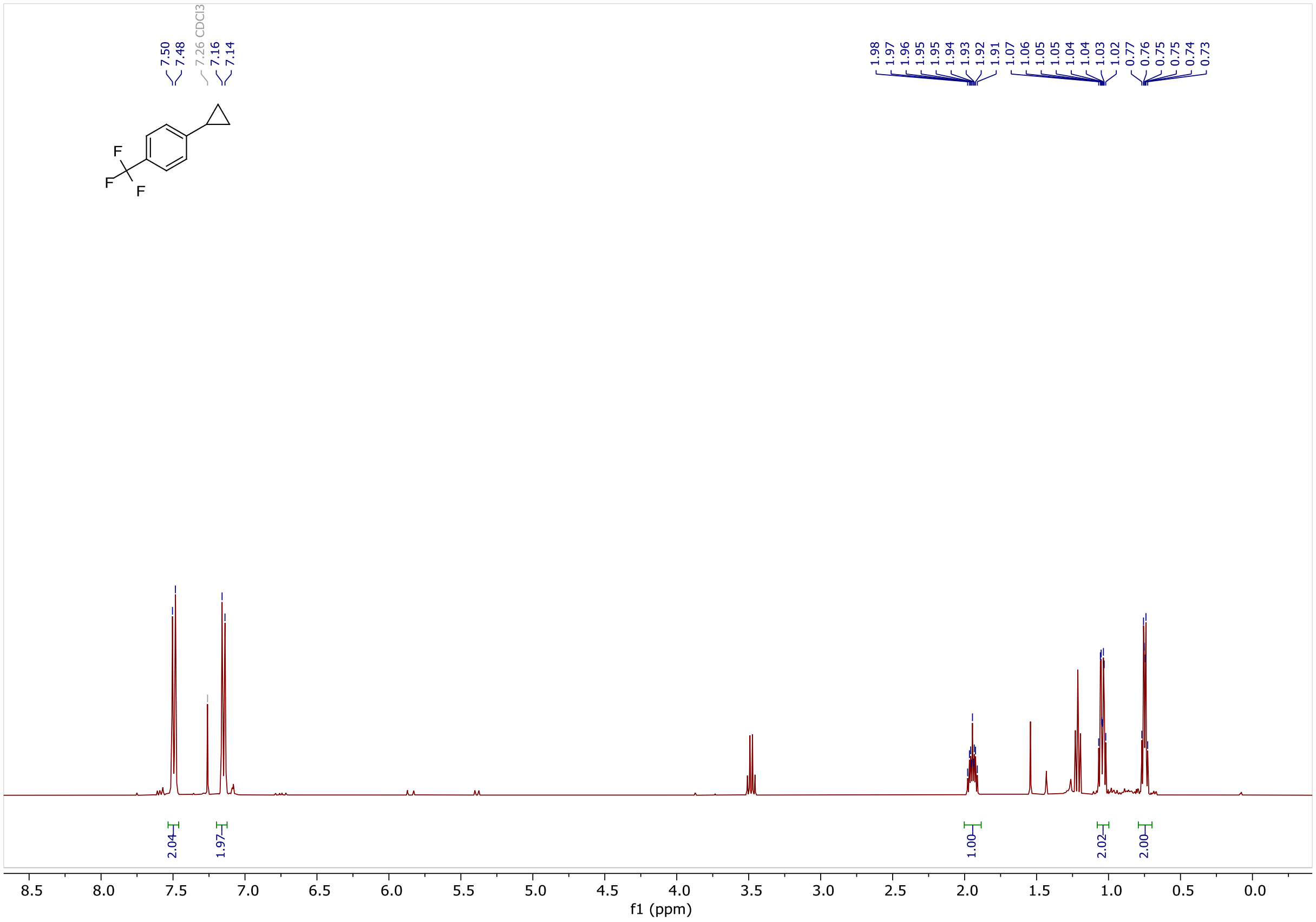

^{19}F NMR Spectrum of *p*-5i (376 MHz, $CDCl_3$)

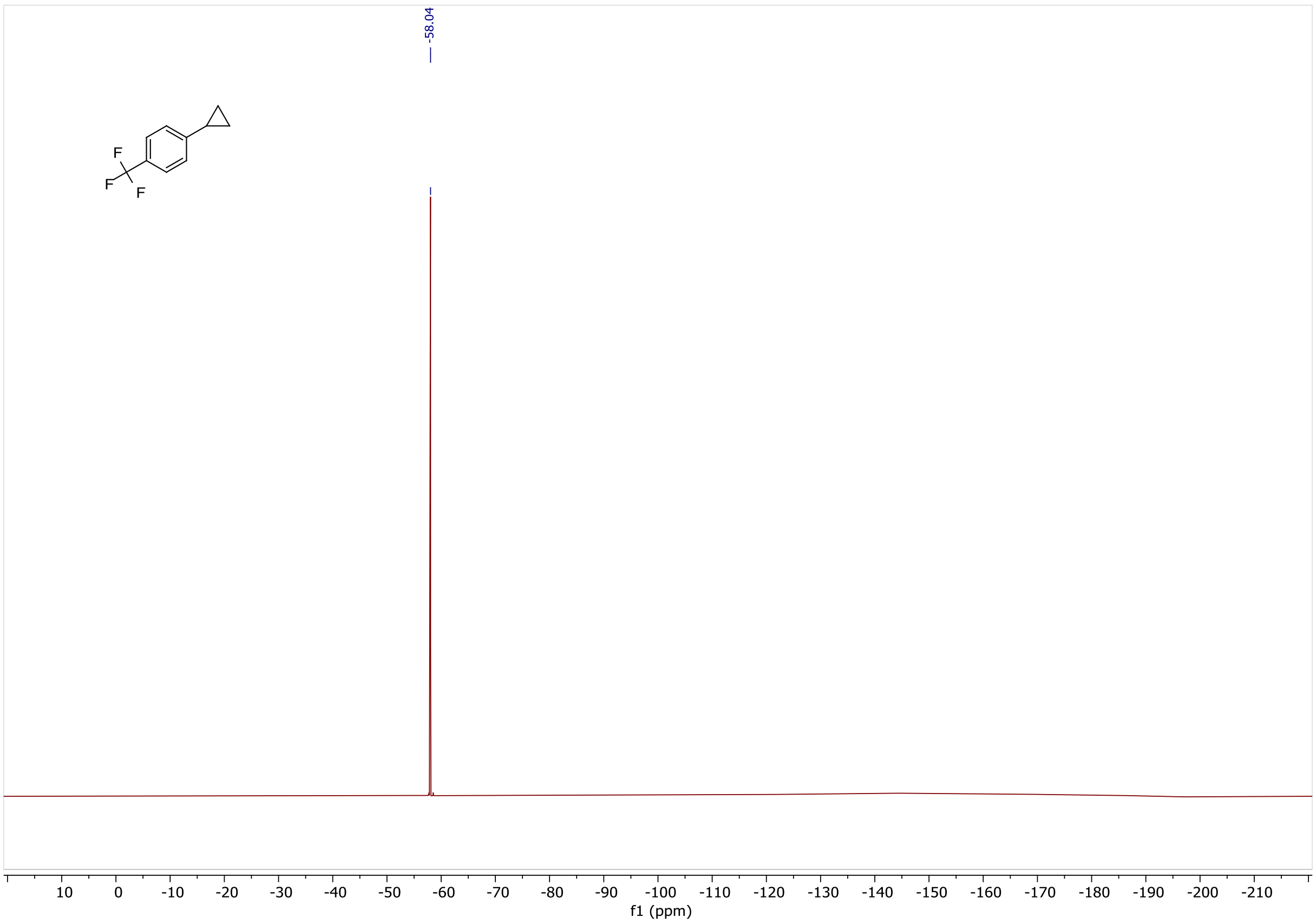

^{13}C NMR Spectrum of *p*-5i (101 MHz, $CDCl_3$)

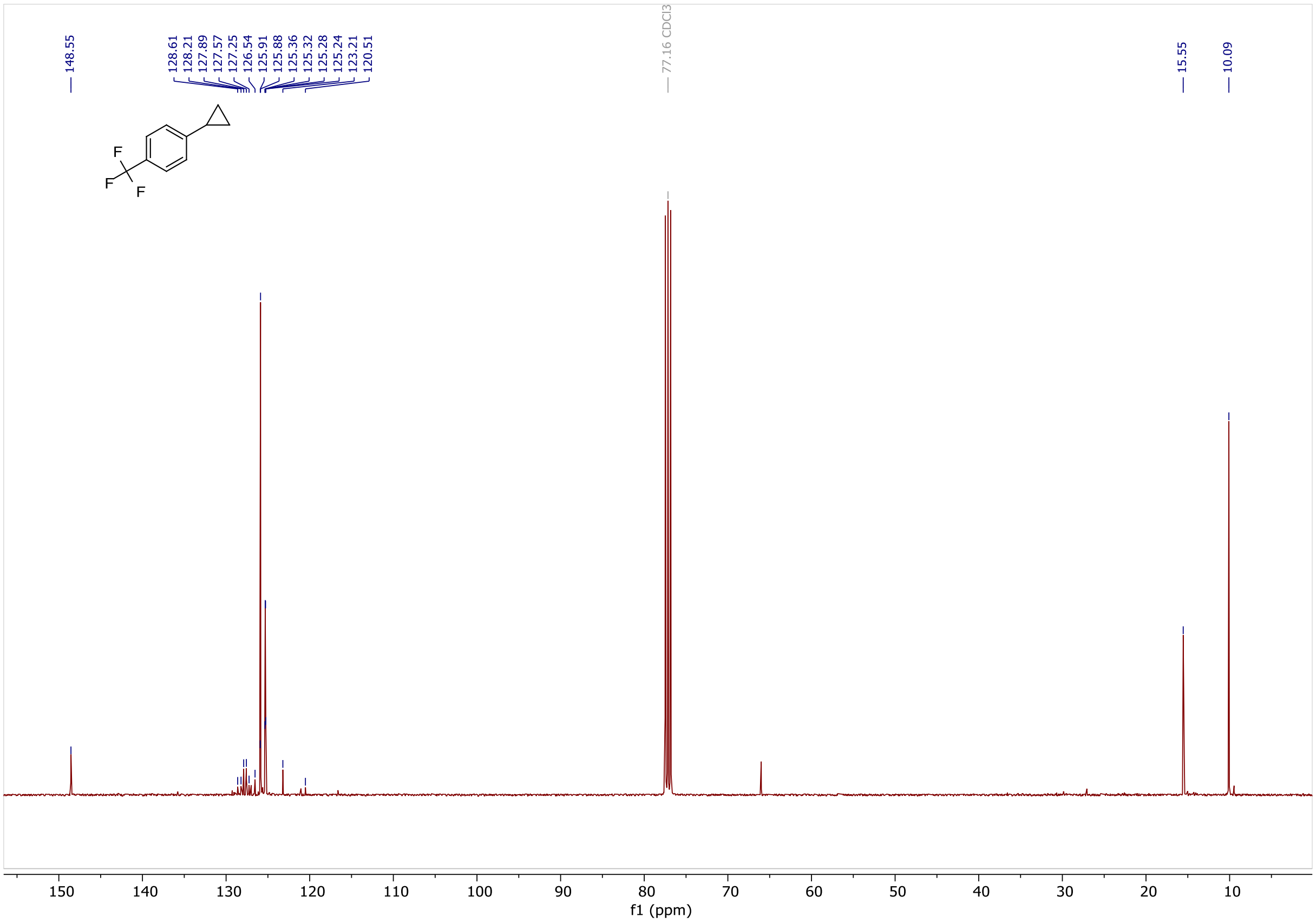

^{1}H NMR Spectrum of *o*-5j (400 MHz, $CDCl_3$)

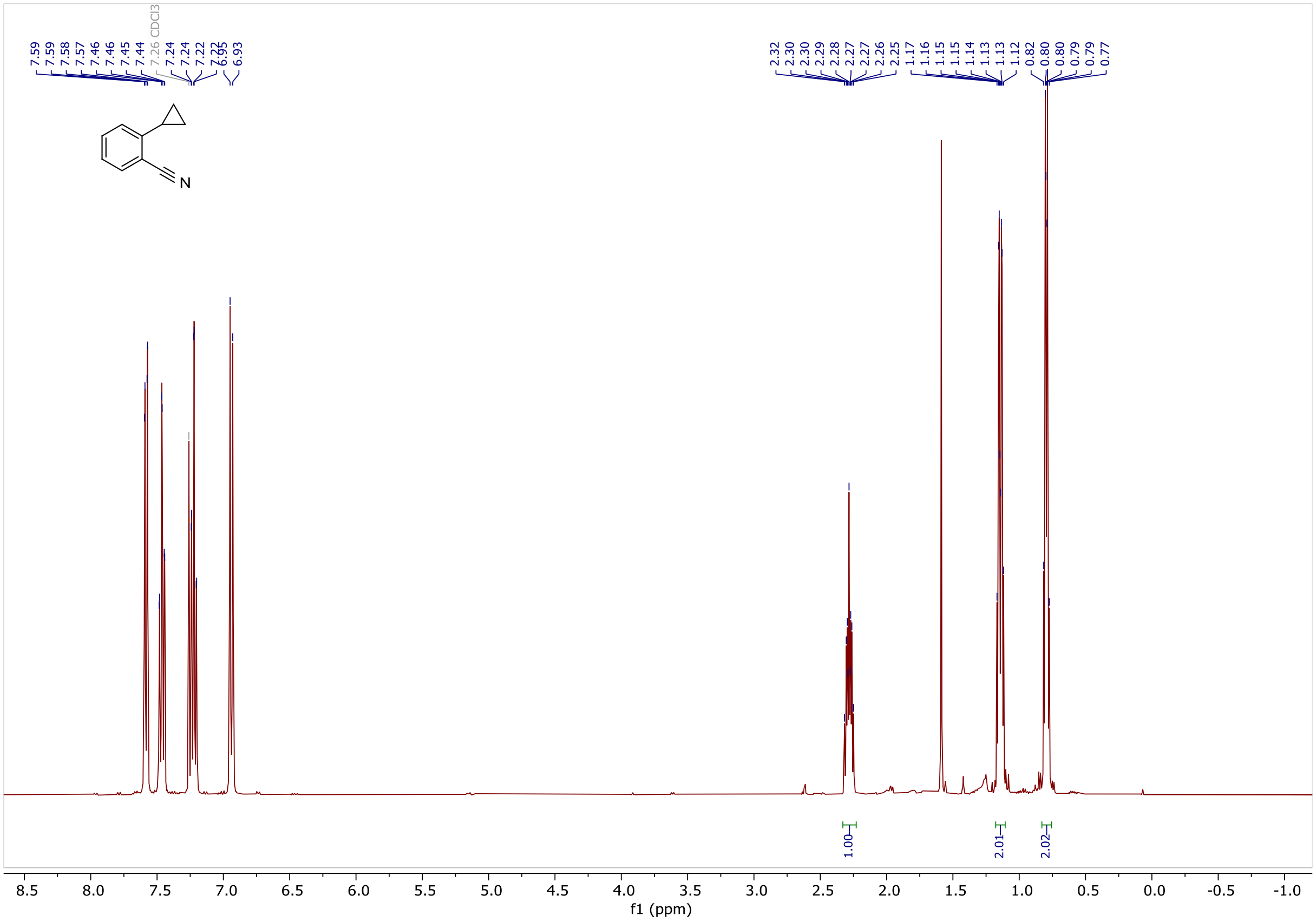

^{13}C NMR Spectrum of *o*-5j (101 MHz, $CDCl_3$)

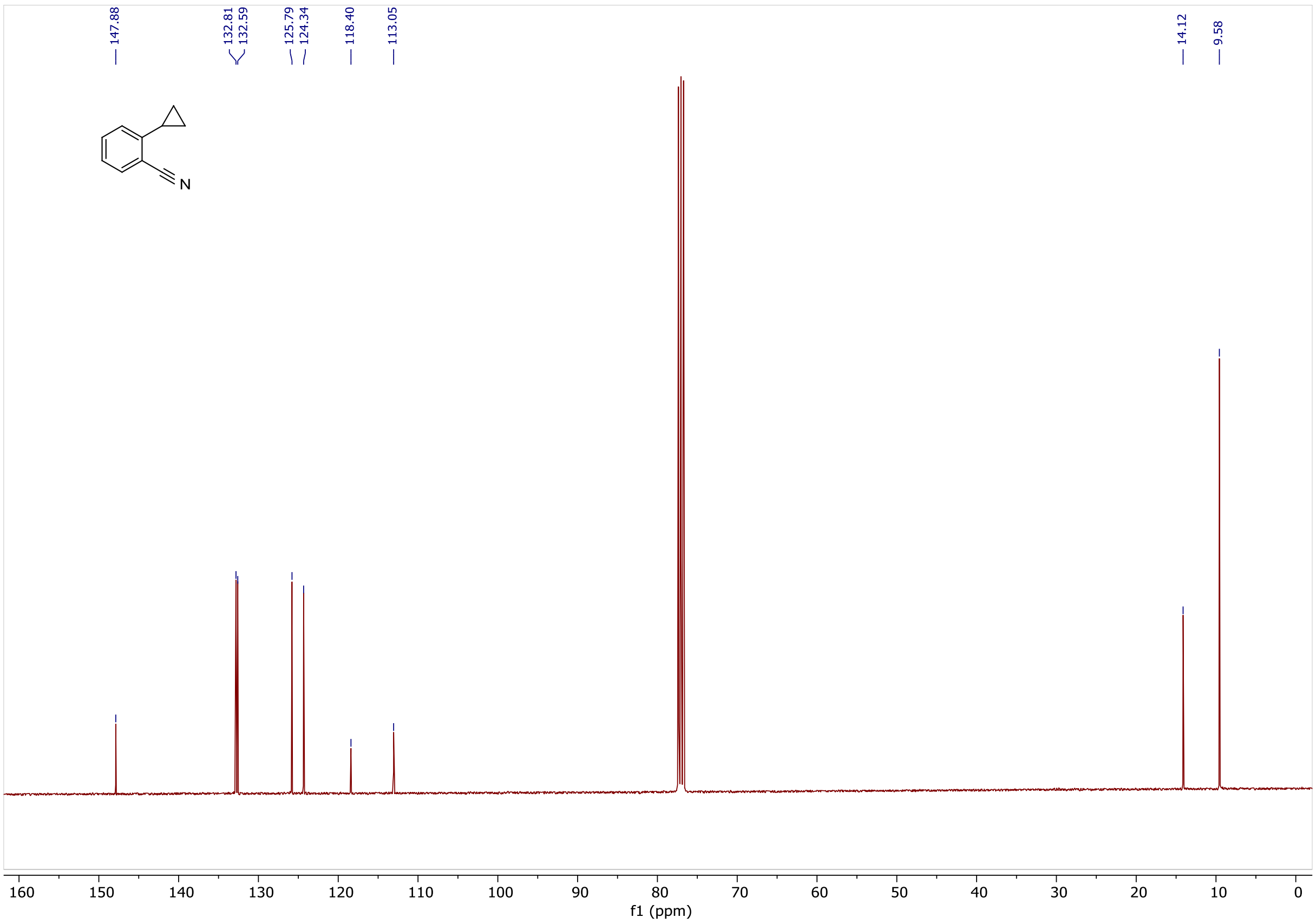

^{1}H NMR Spectrum of *m*-5j (400 MHz, $CDCl_3$)

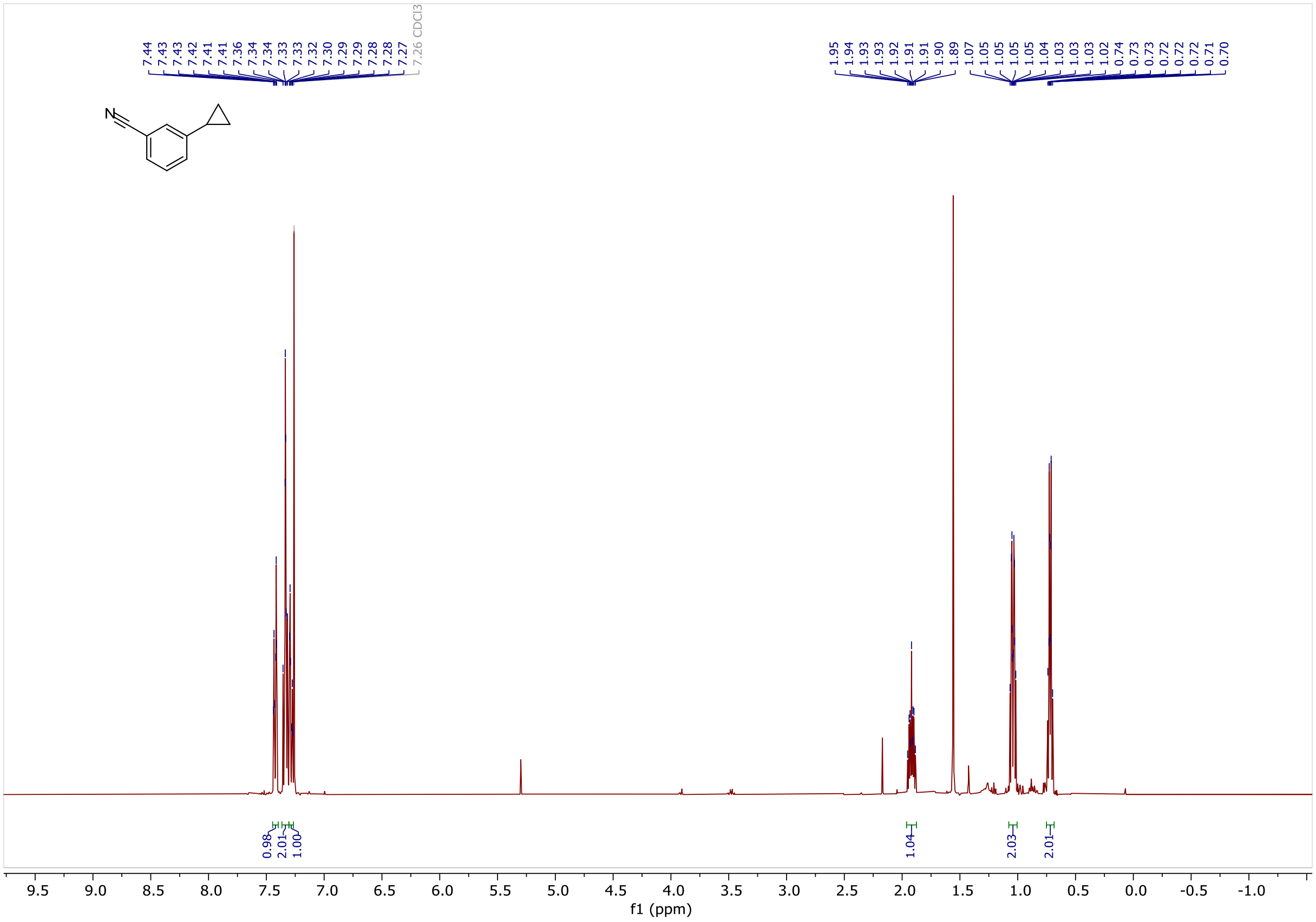

^{13}C NMR Spectrum of *m*-5j (101 MHz, $CDCl_3$)

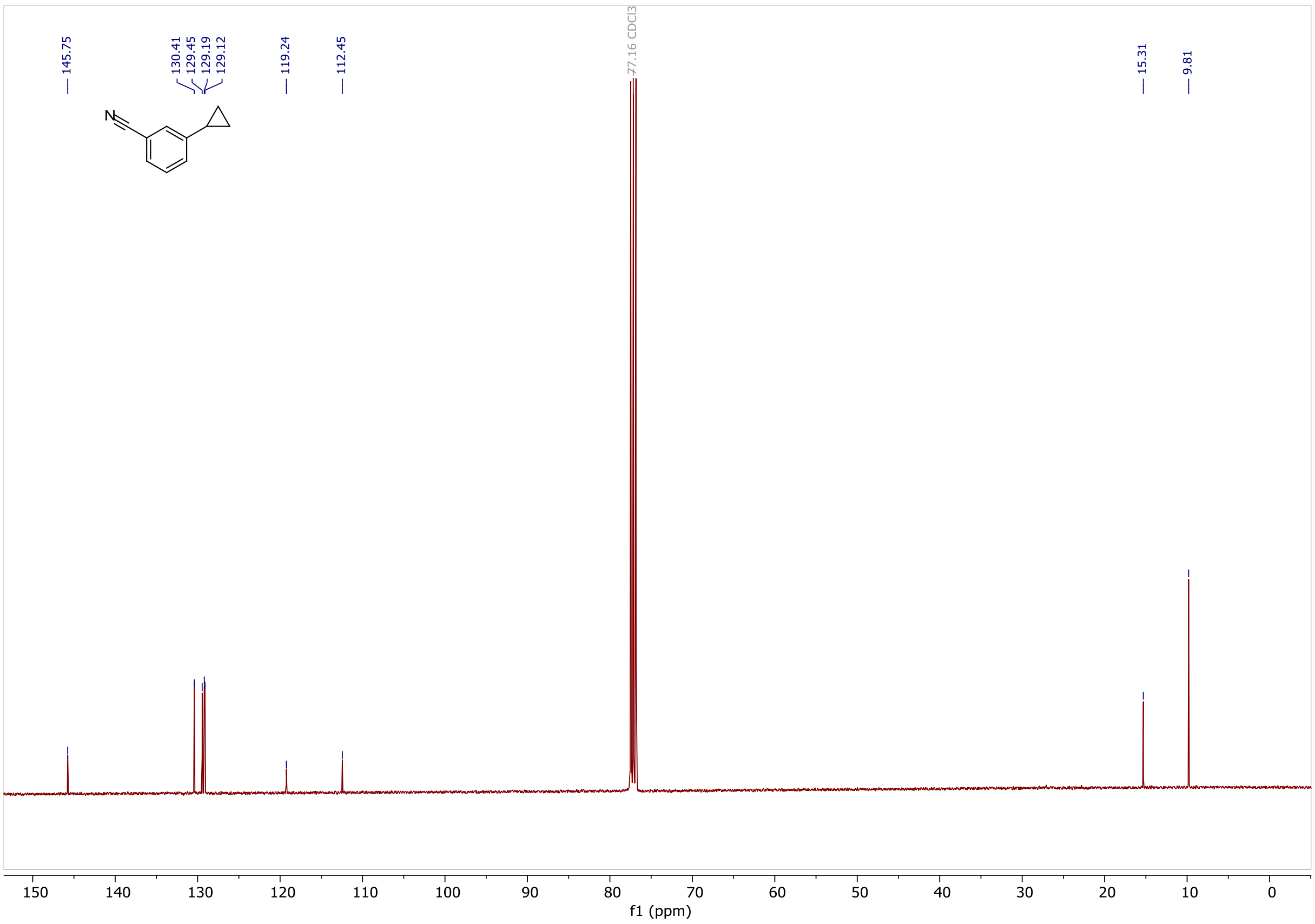

^{1}H NMR Spectrum of *p*-5j (400 MHz, $CDCl_3$)

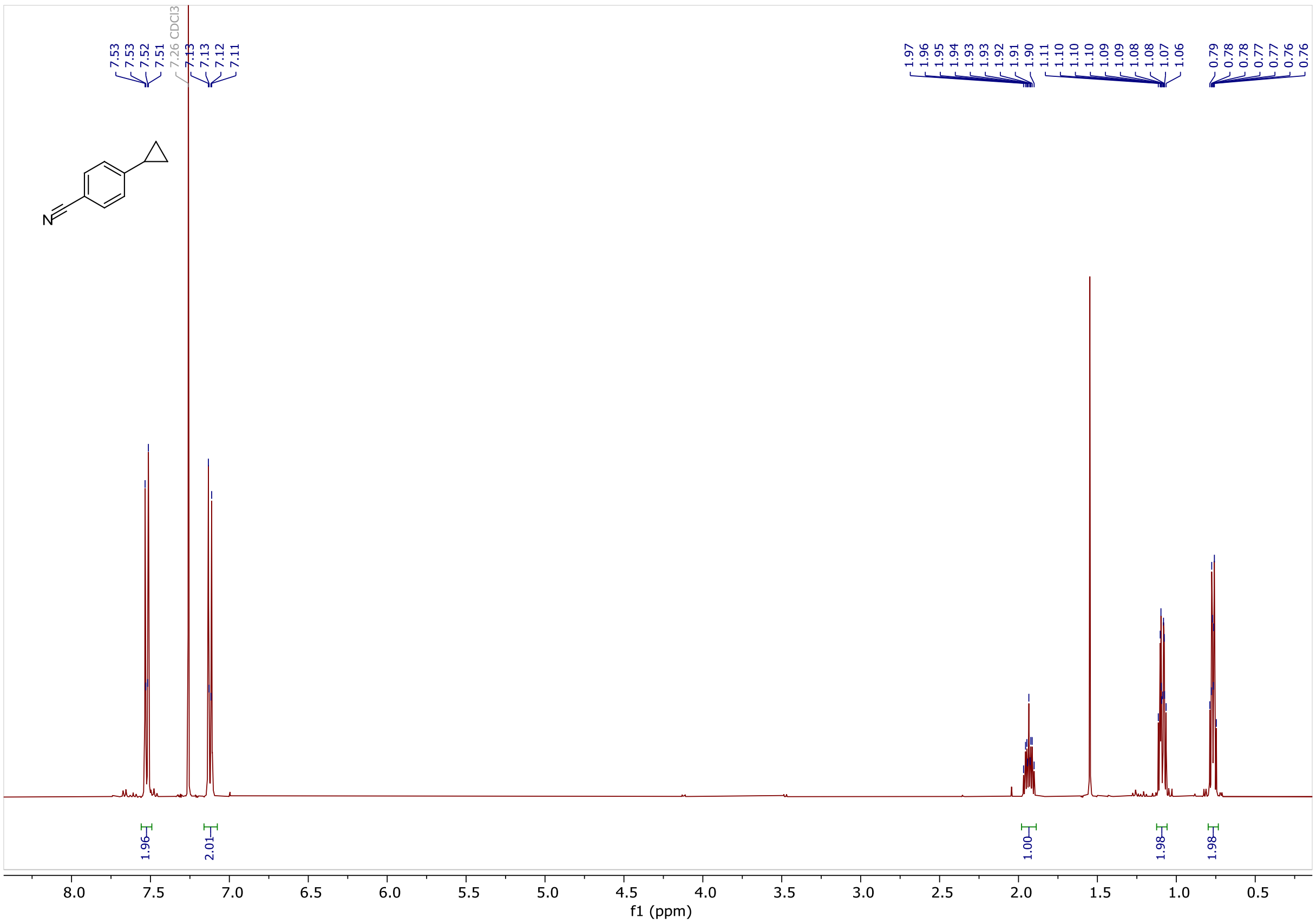

^{13}C NMR Spectrum of *p*-5j (101 MHz, $CDCl_3$)

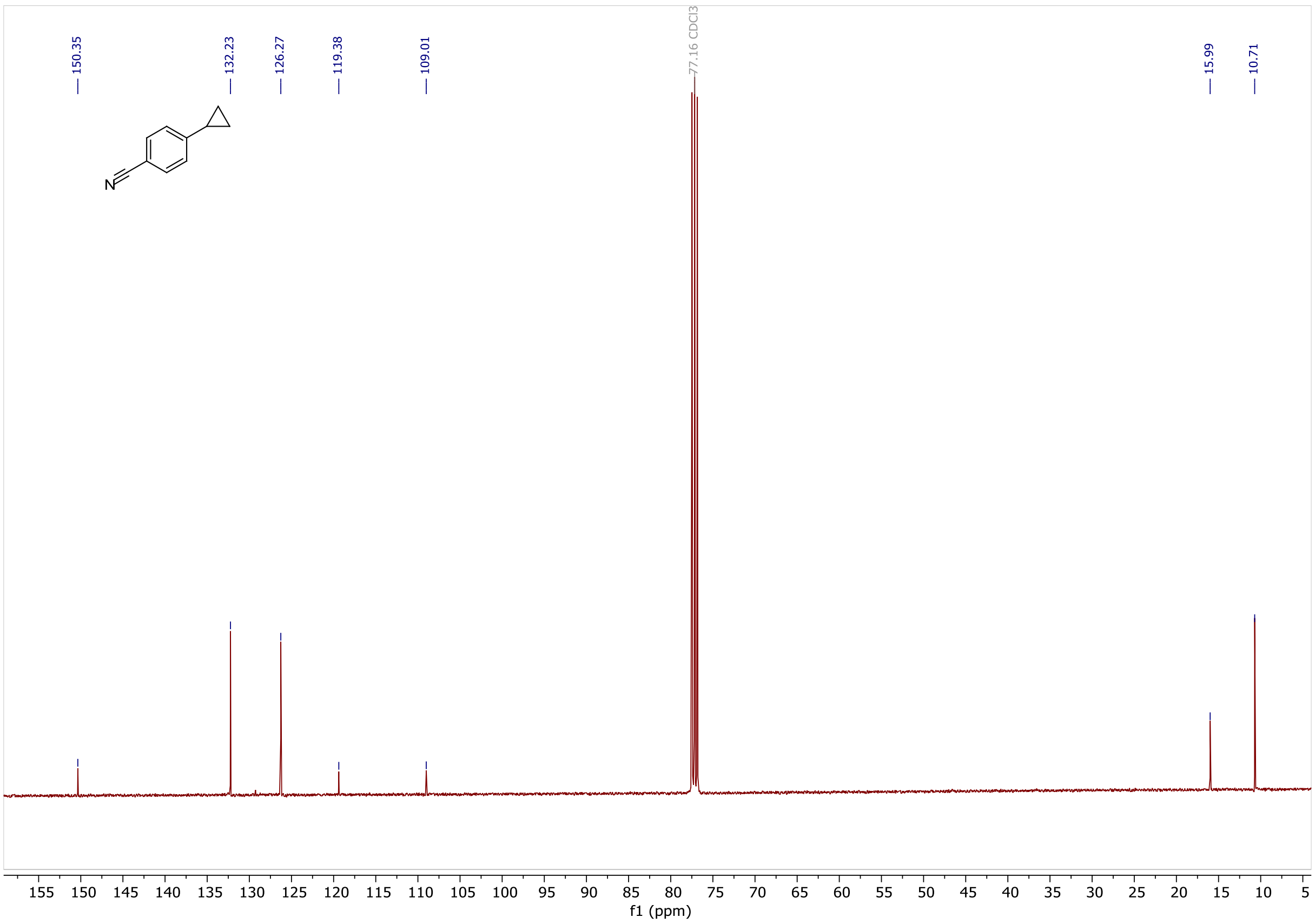

^{1}H NMR Spectrum of *o*-5k (400 MHz, $CDCl_3$)

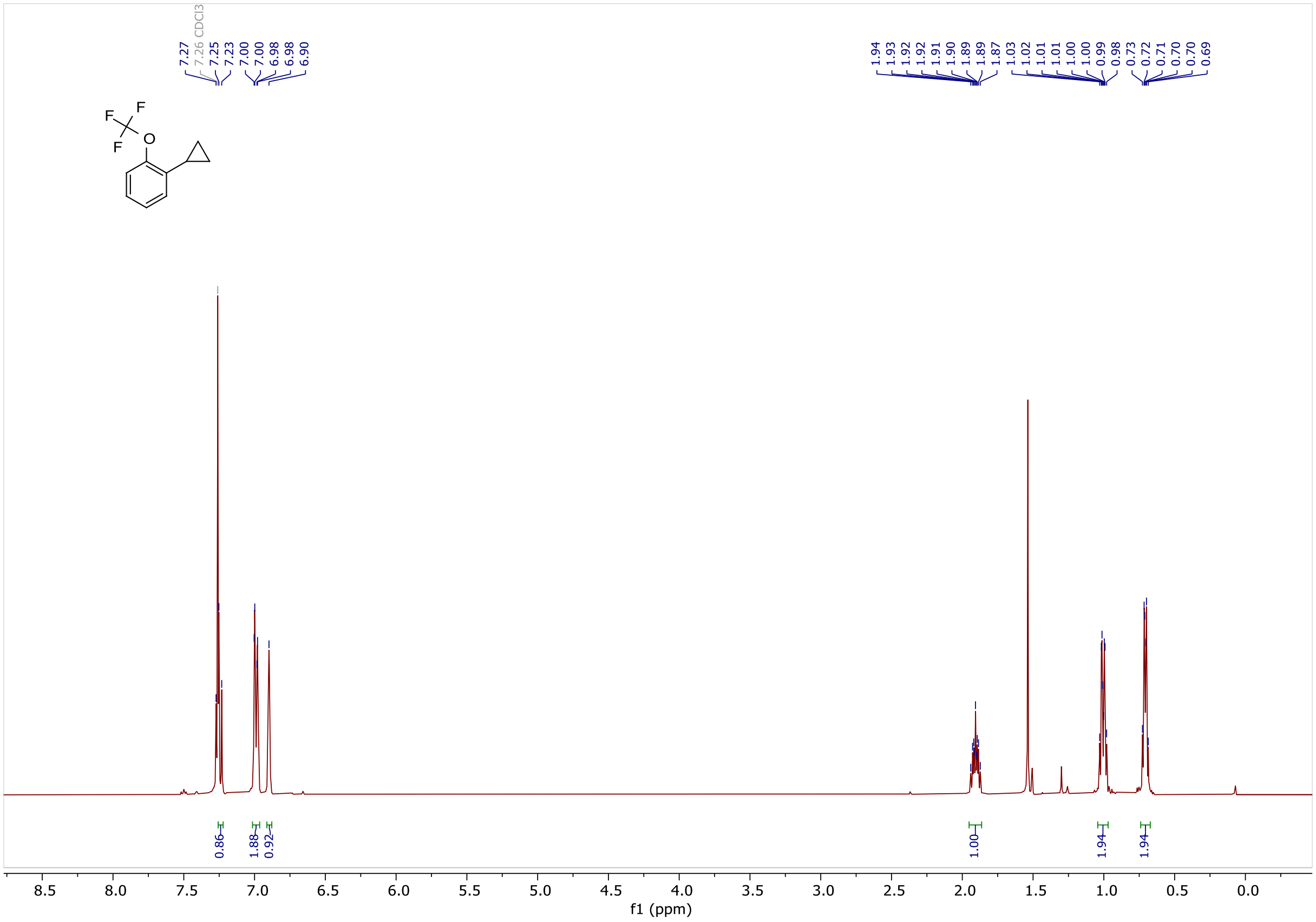

^{19}F NMR Spectrum of *o*-5k (376 MHz, $CDCl_3$)

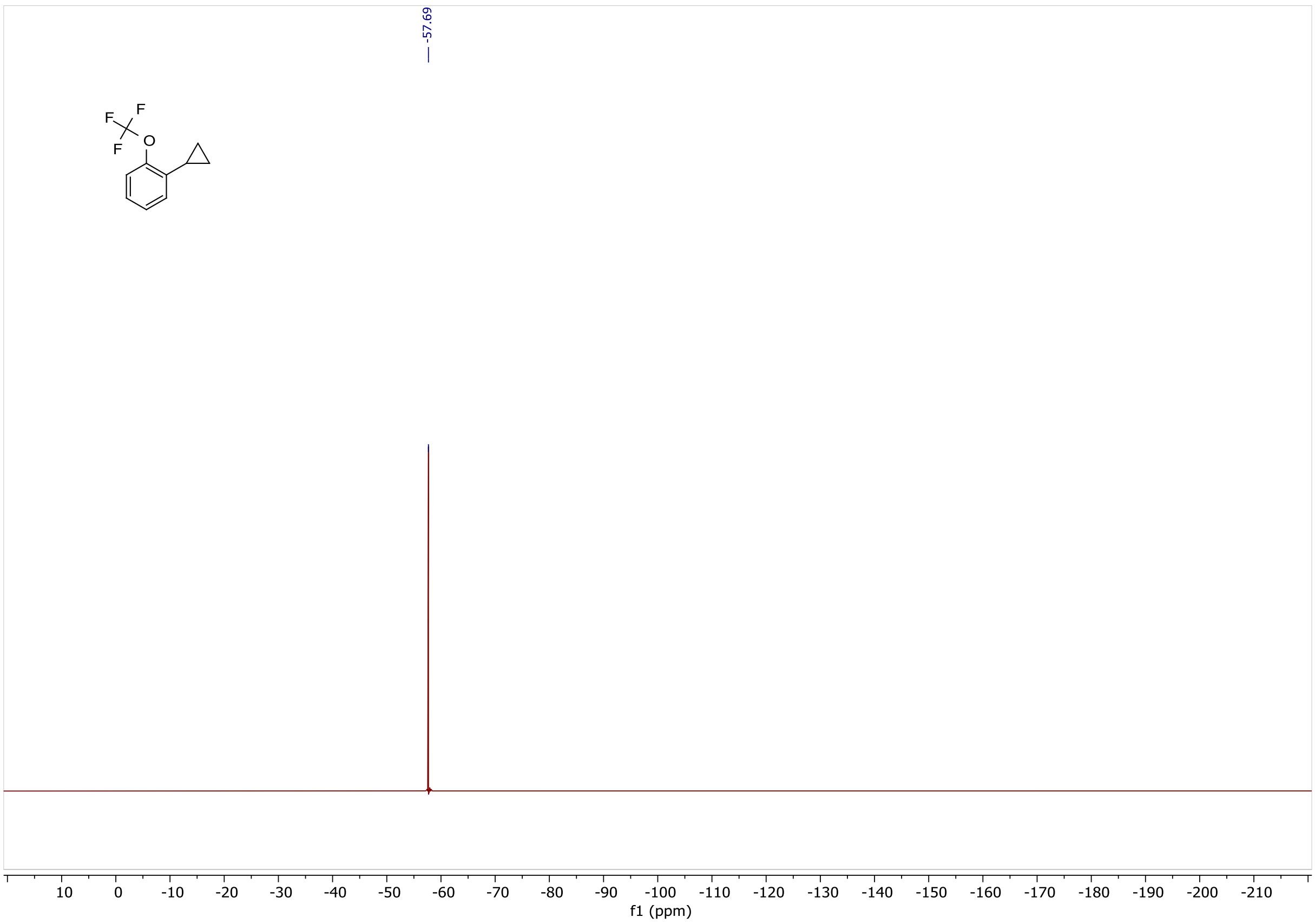

^{13}C NMR Spectrum of *o*-5k (101 MHz, $CDCl_3$)

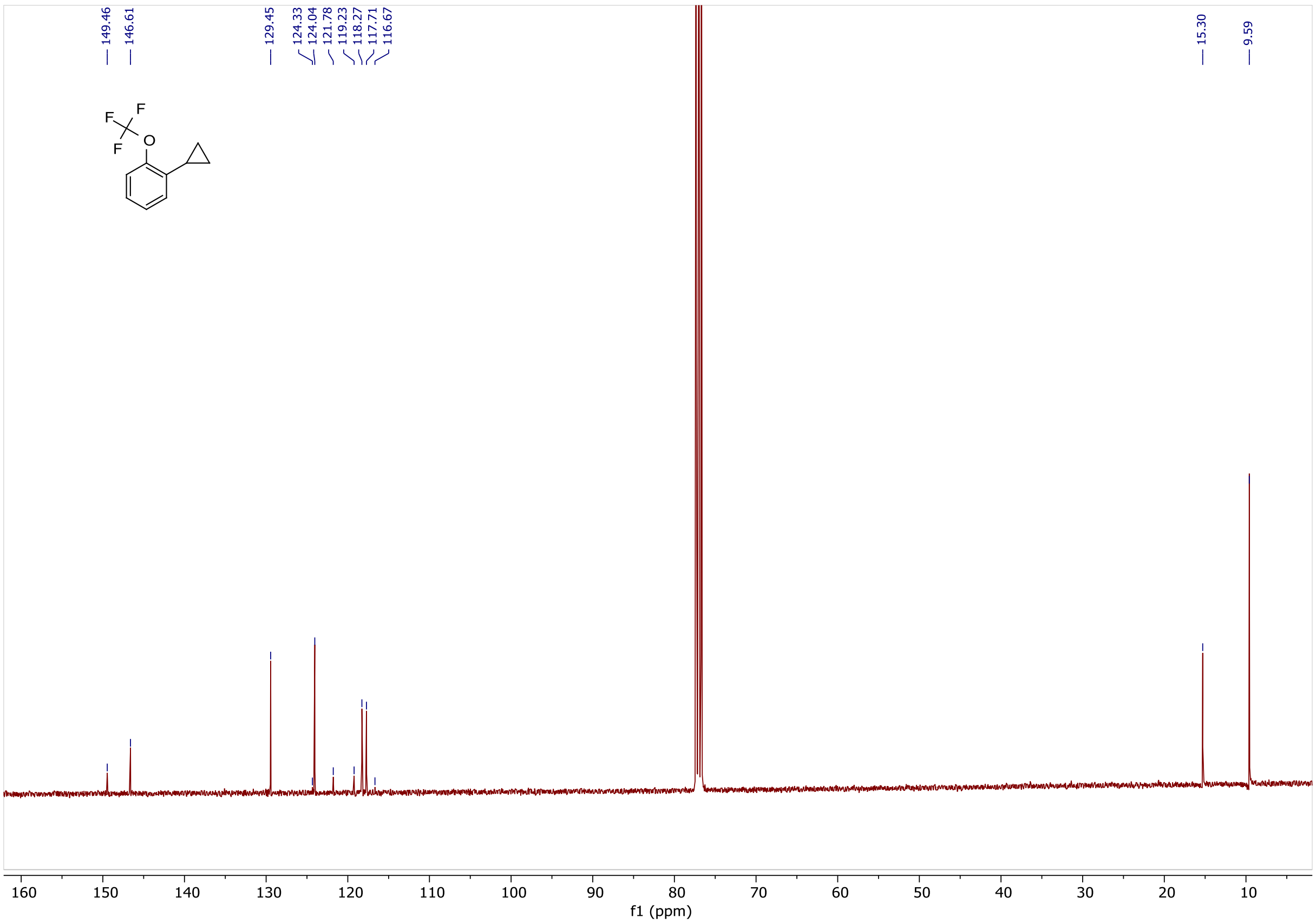

^{1}H NMR Spectrum of *m*-5k (400 MHz, $CDCl_3$)

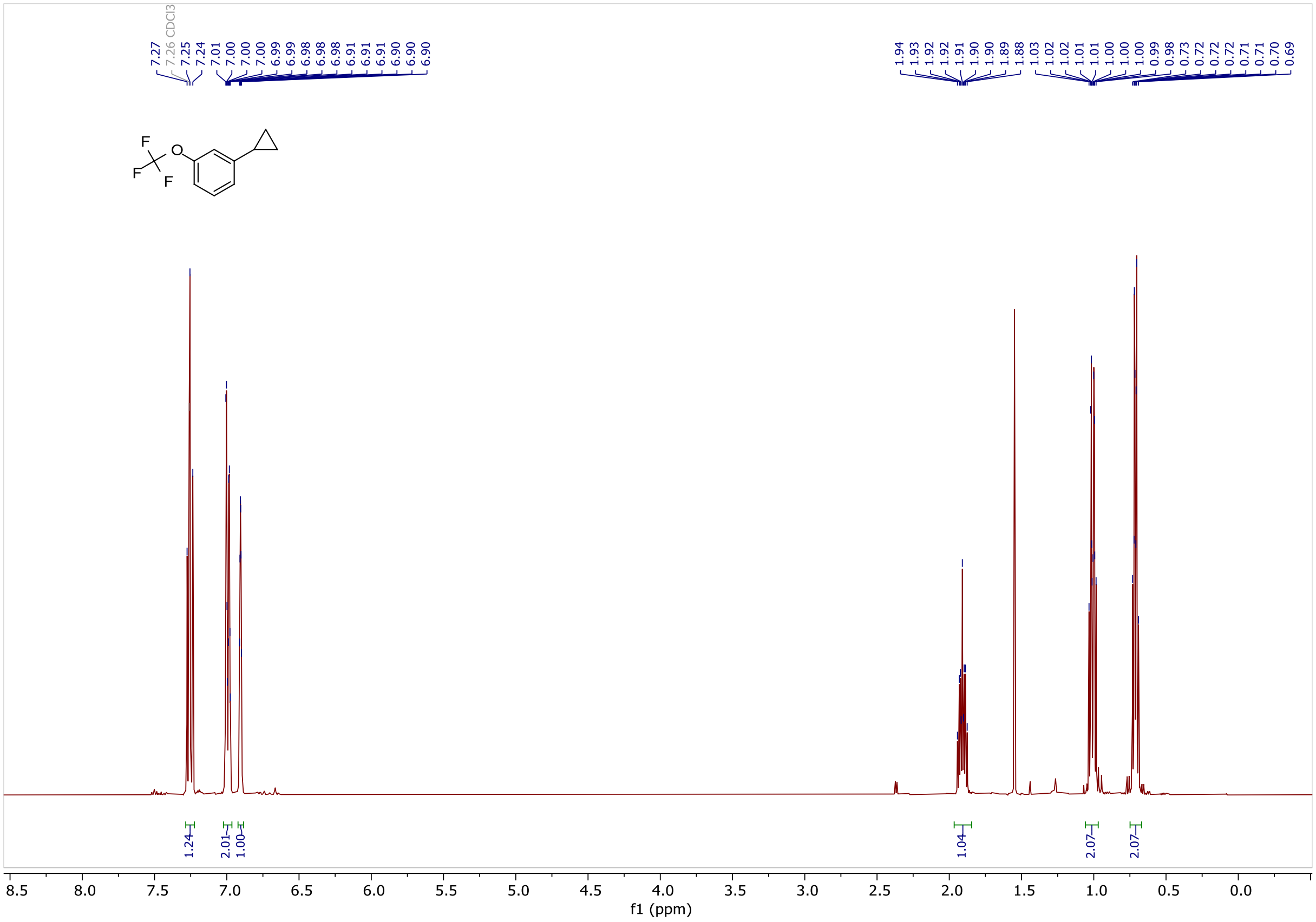

^{19}F NMR Spectrum of *m*-5k (376 MHz, $CDCl_3$)

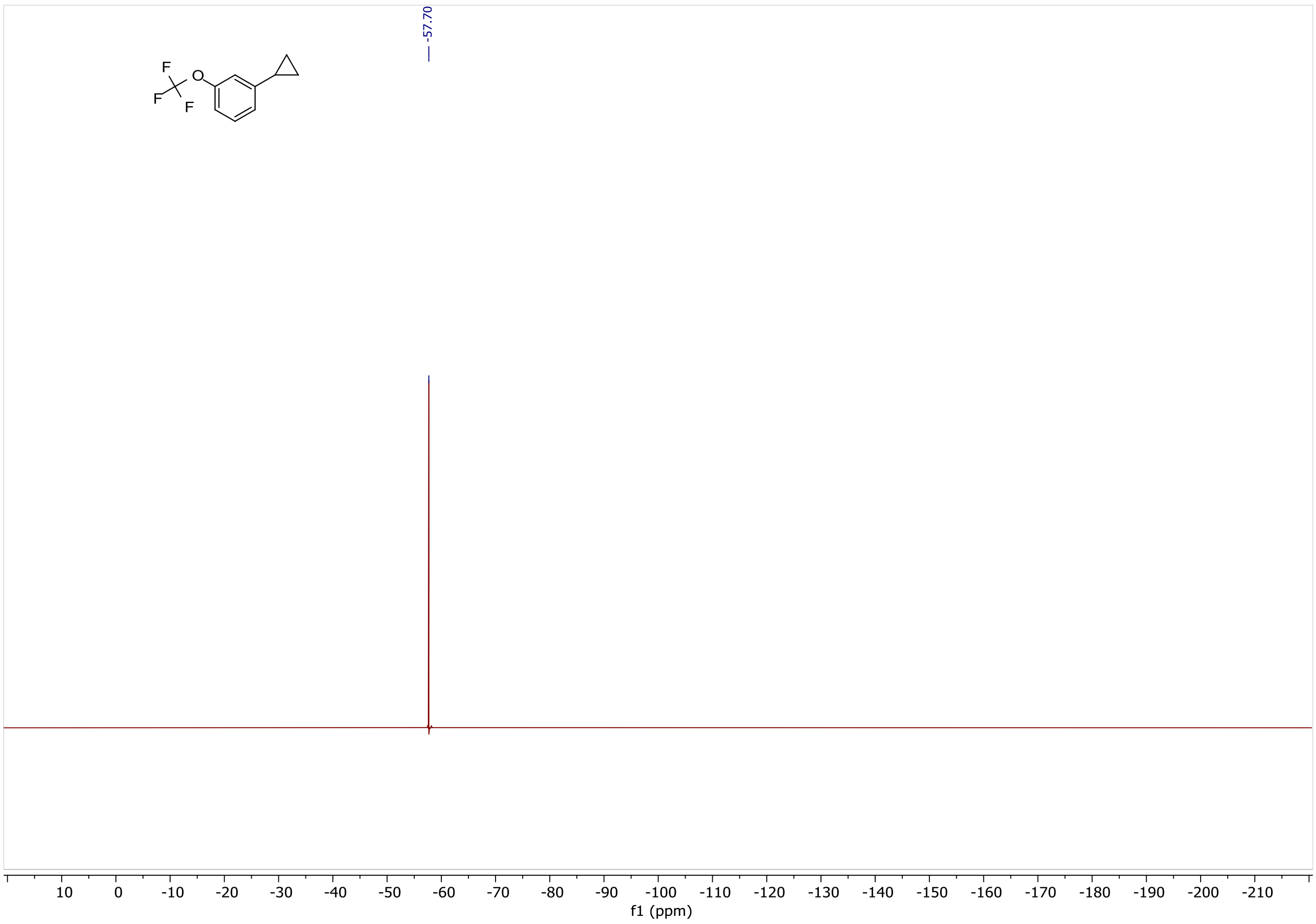

^{13}C NMR Spectrum of *m*-5k (101 MHz, $CDCl_3$)

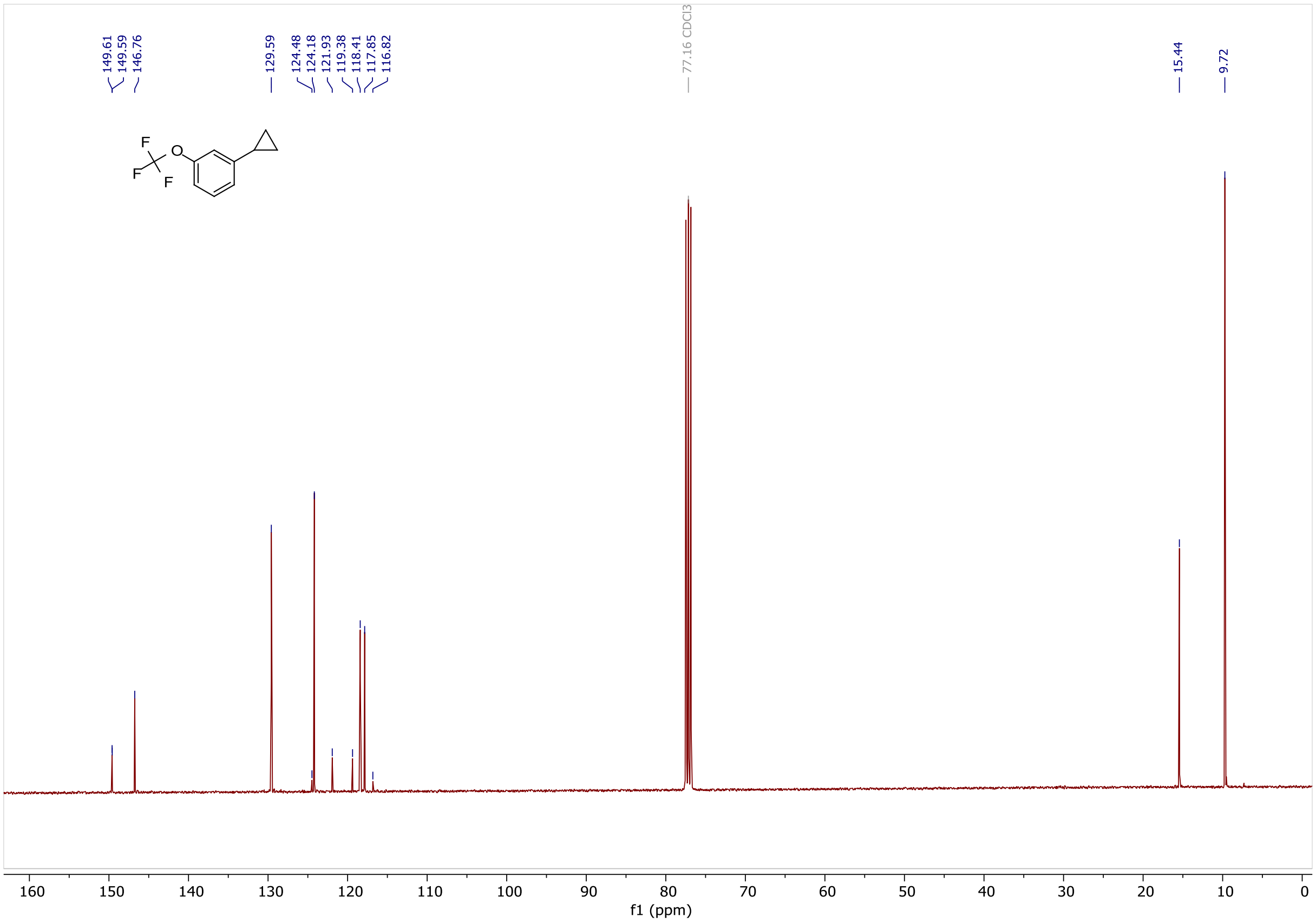

^{1}H NMR Spectrum of *p*-5k (400 MHz, $CDCl_3$)

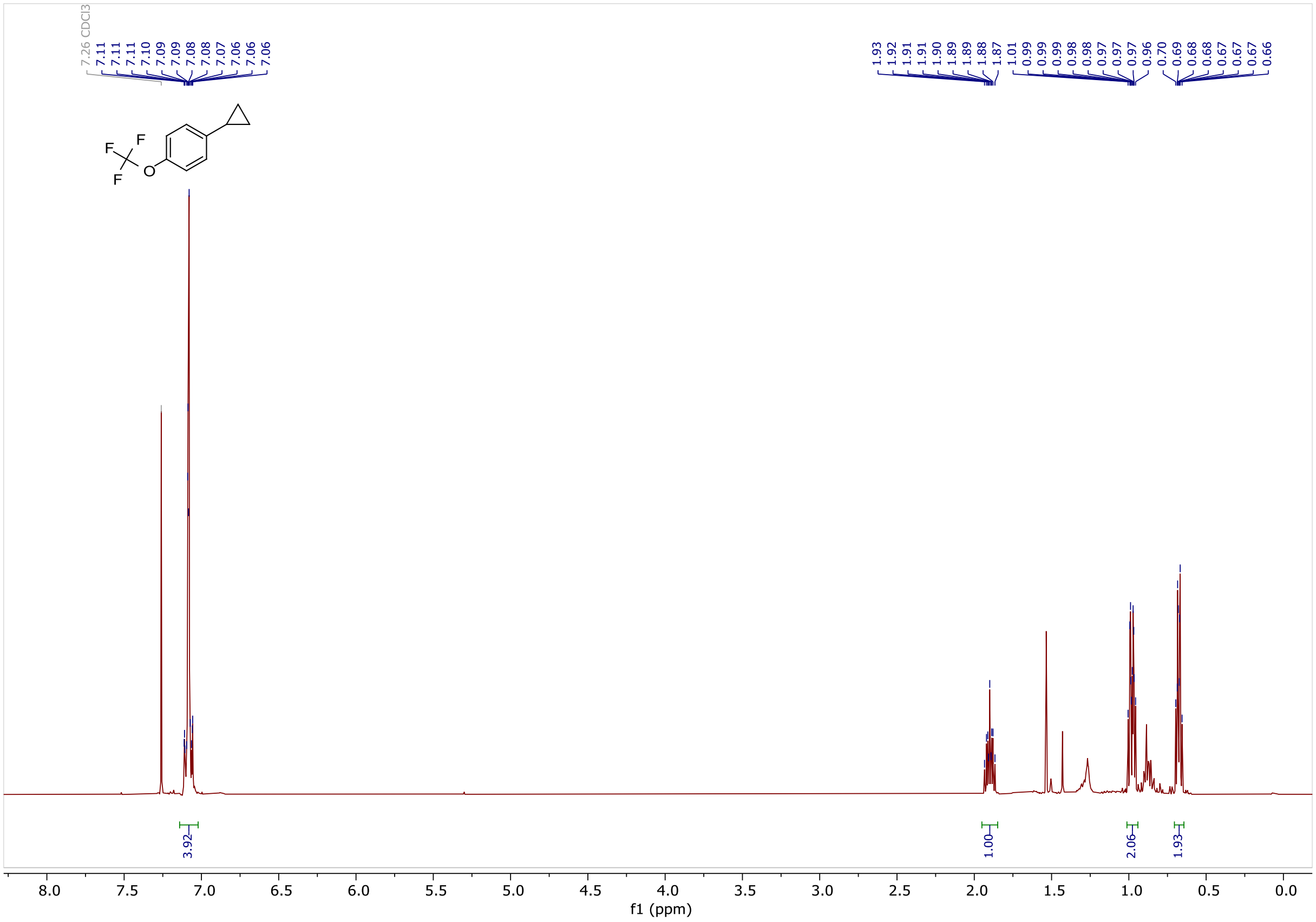

^{19}F NMR Spectrum of *p*-5k (376 MHz, $CDCl_3$)

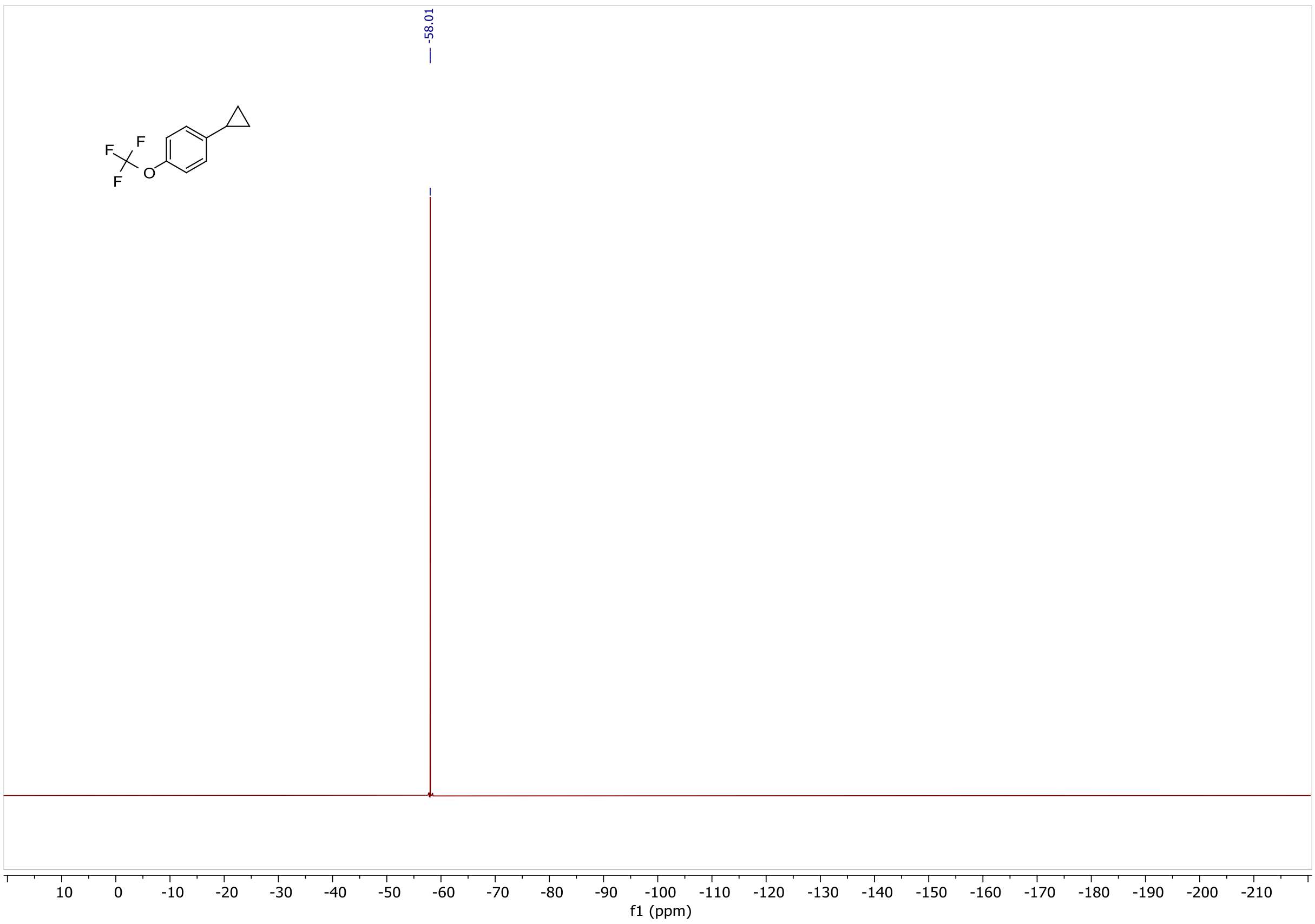

^{13}C NMR Spectrum of *p*-5k (101 MHz, $CDCl_3$)

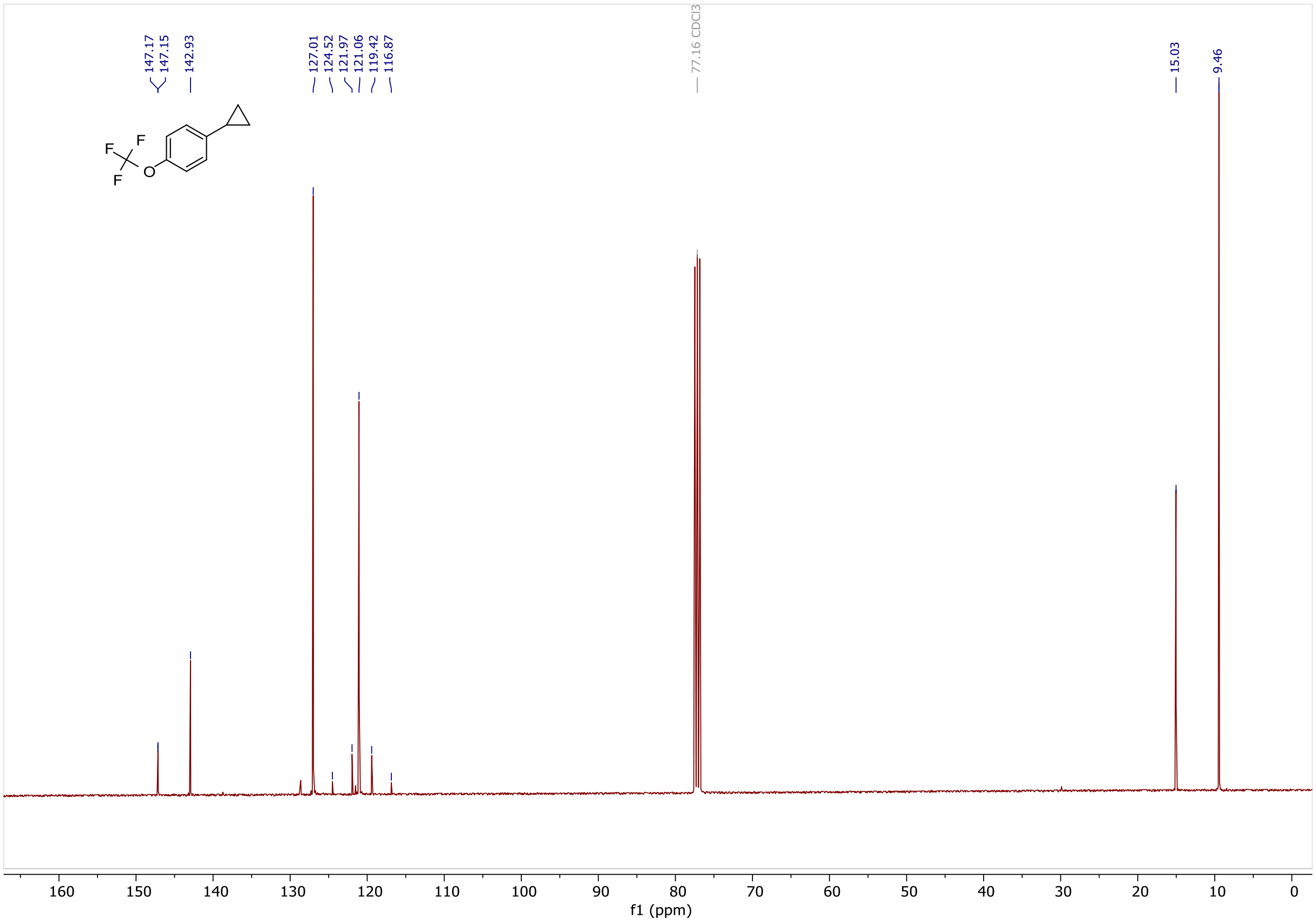

¹H NMR Spectrum of *o*-5l (400 MHz, CDCl3)

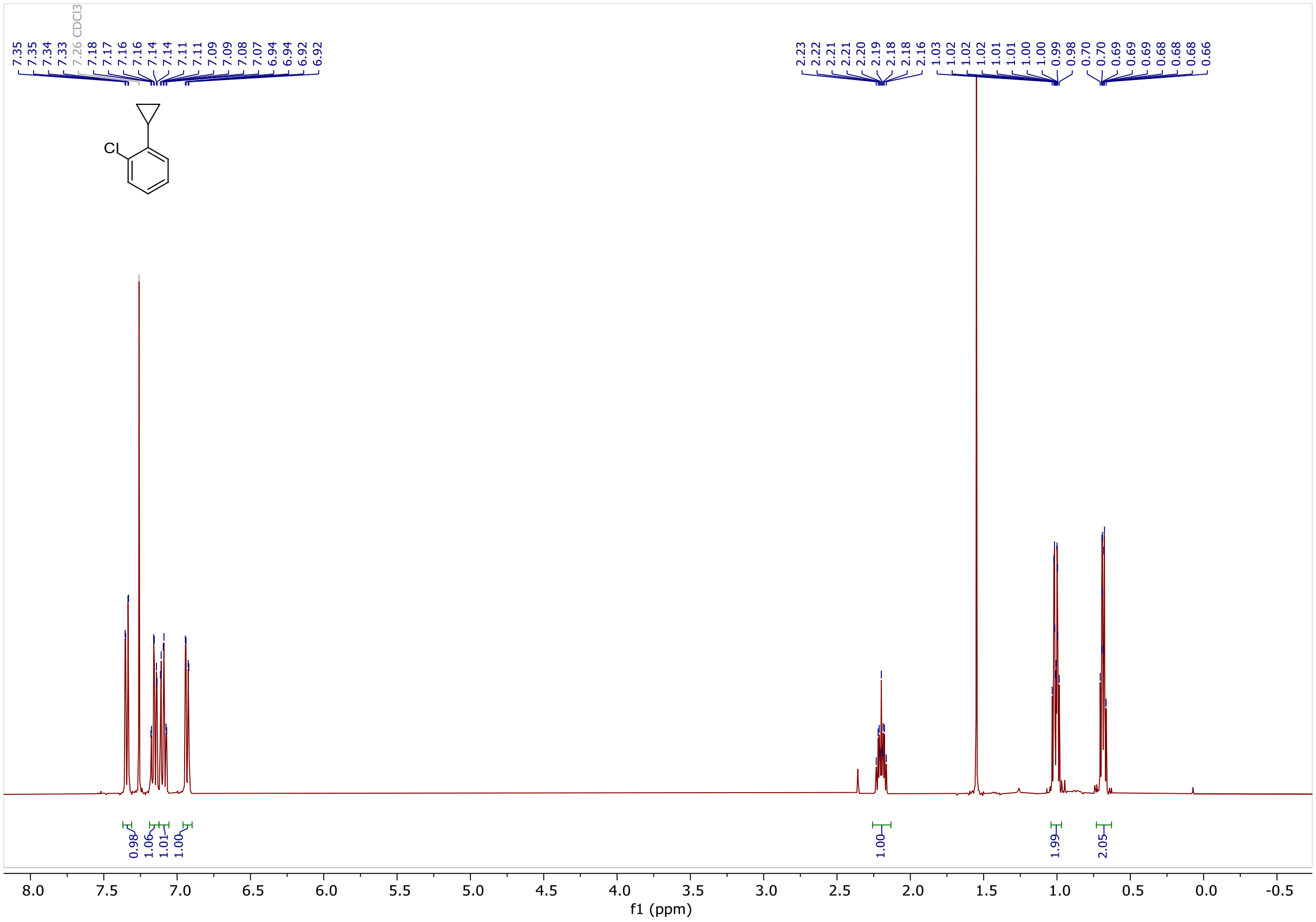

¹³C NMR Spectrum of *o*-5l (101 MHz, CDCl3)

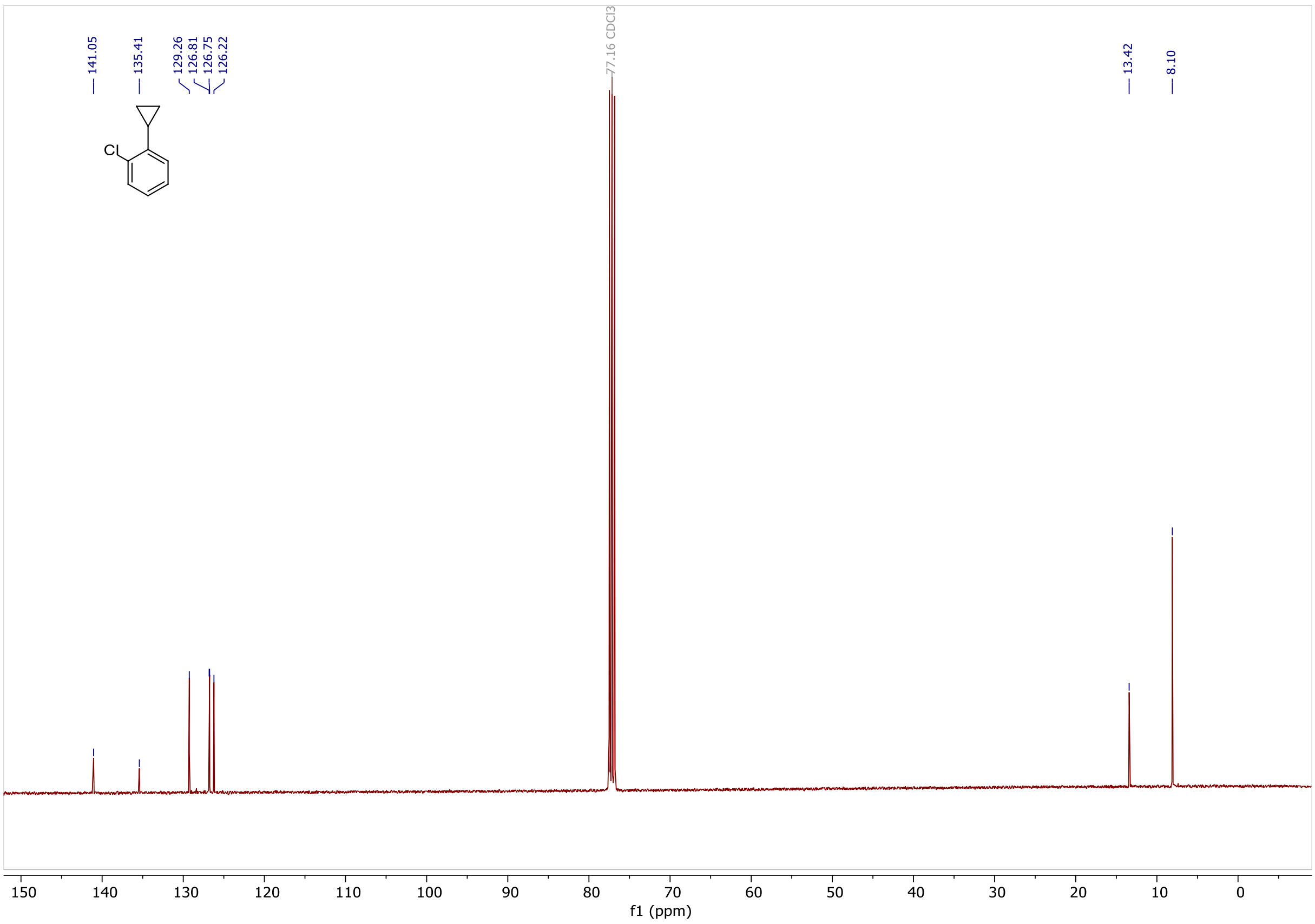

^{1}H NMR Spectrum of *m*-5l (400 MHz, $CDCl_3$)

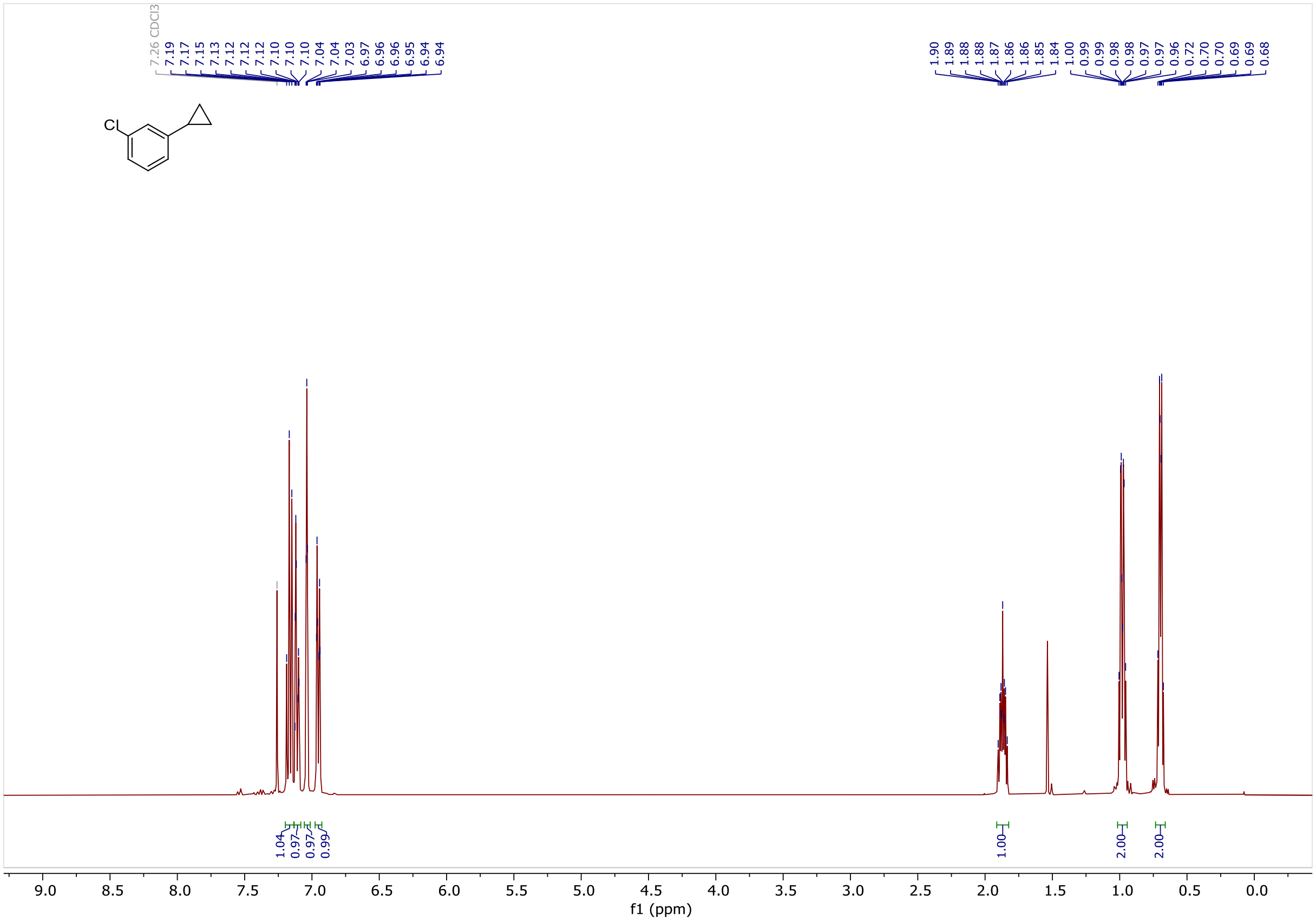

^{13}C NMR Spectrum of *m*-5l (101 MHz, $CDCl_3$)

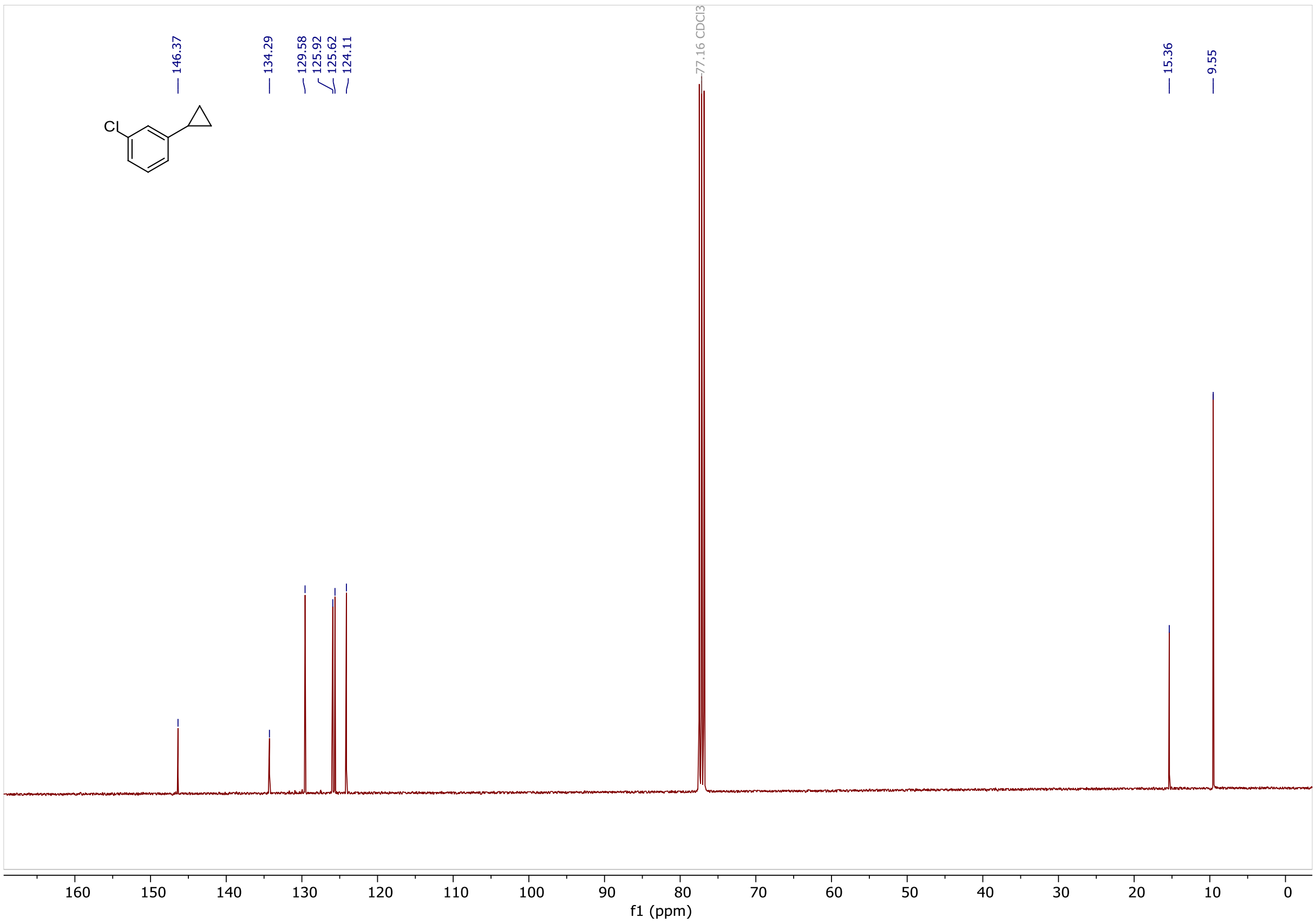

^{1}H NMR Spectrum of *p*-5l (400 MHz, $CDCl_3$)

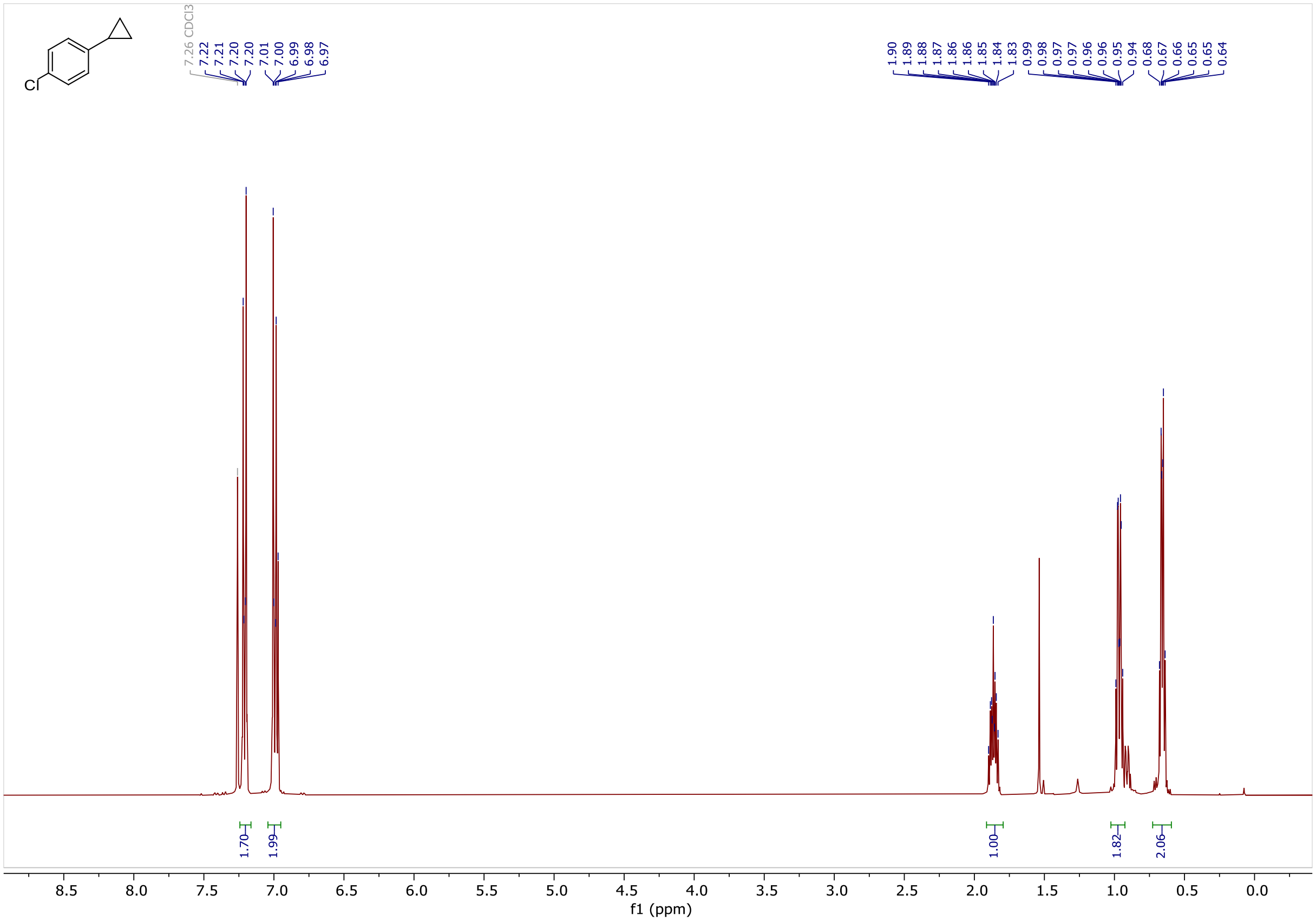

^{13}C NMR Spectrum of *p*-5l (101 MHz, $CDCl_3$)

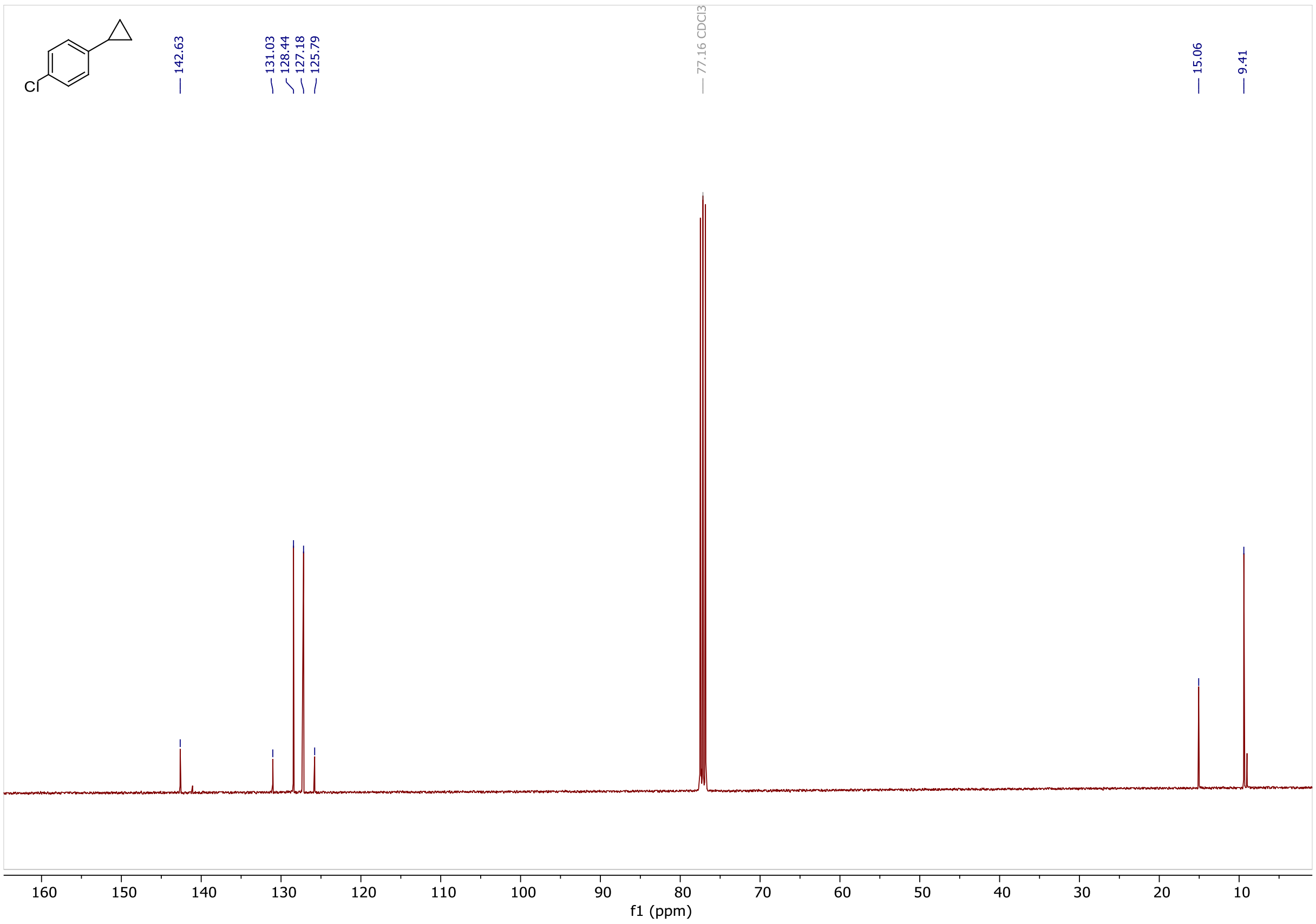

^{1}H NMR Spectrum of S1 (400 MHz, $CDCl_3$)

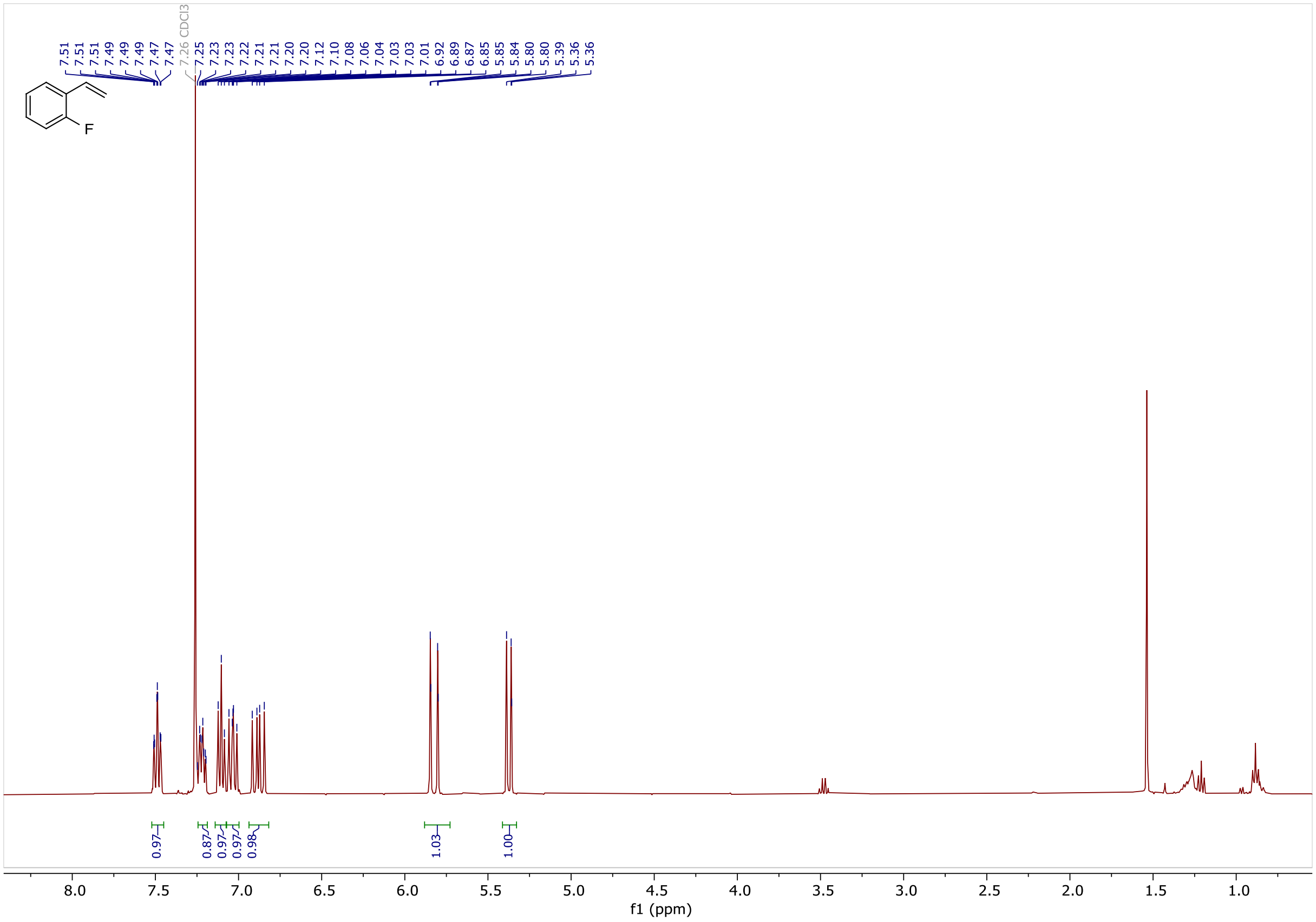

^{19}F NMR Spectrum of S1 (376 MHz, $CDCl_3$)

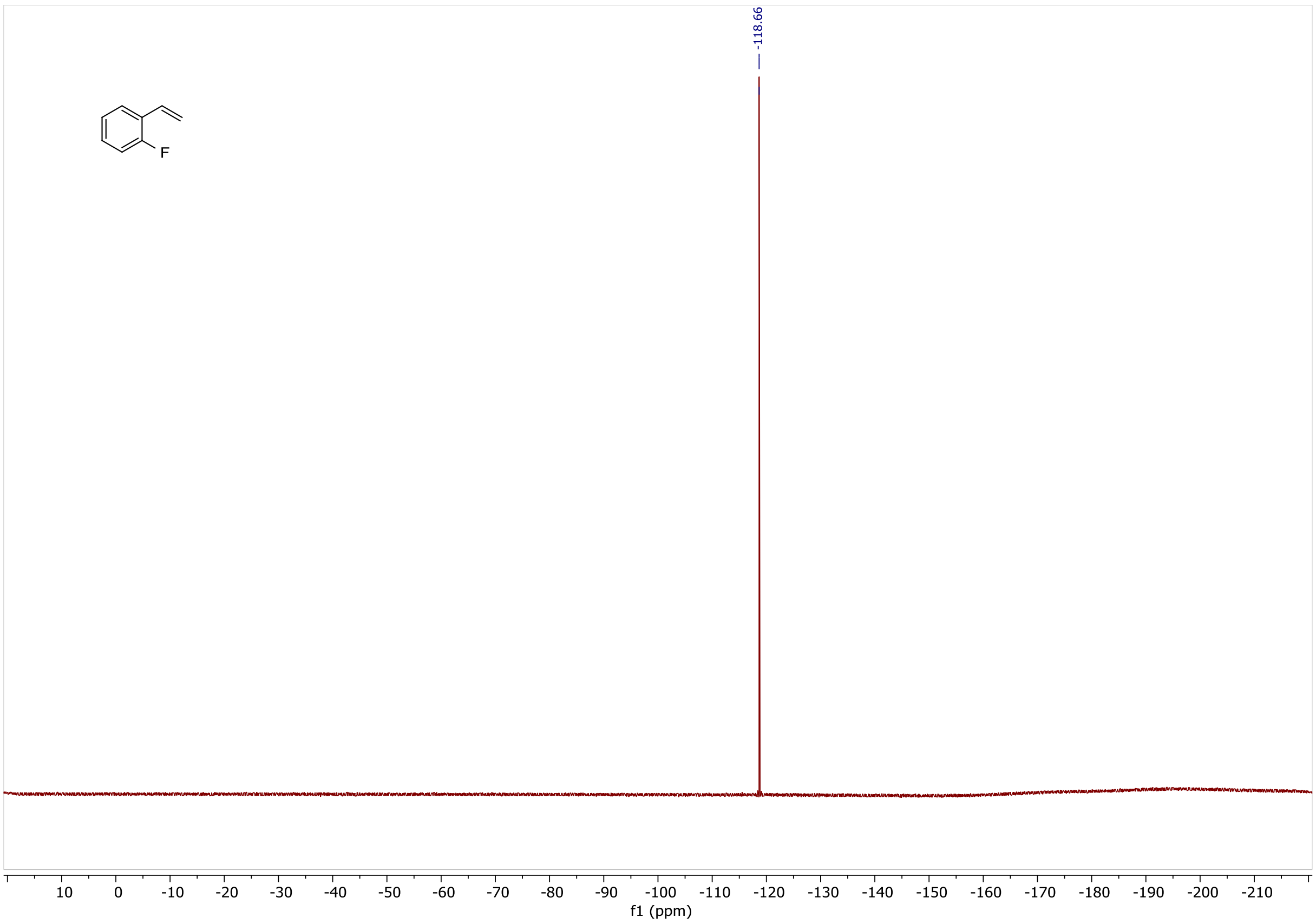

^{13}C NMR Spectrum of S1 (101 MHz, $CDCl_3$)

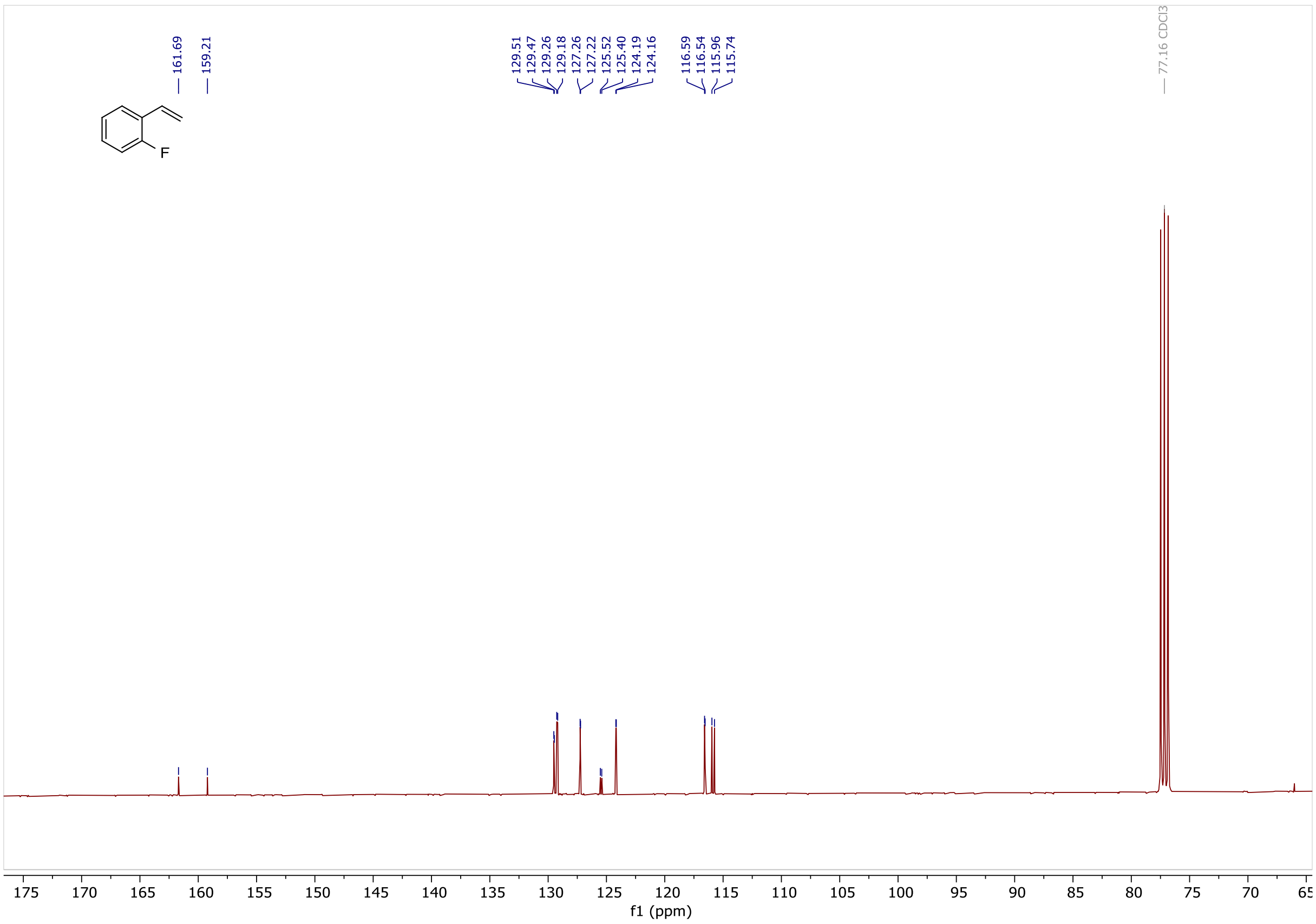

^{1}H NMR Spectrum of *o*-5m (400 MHz, $CDCl_3$)

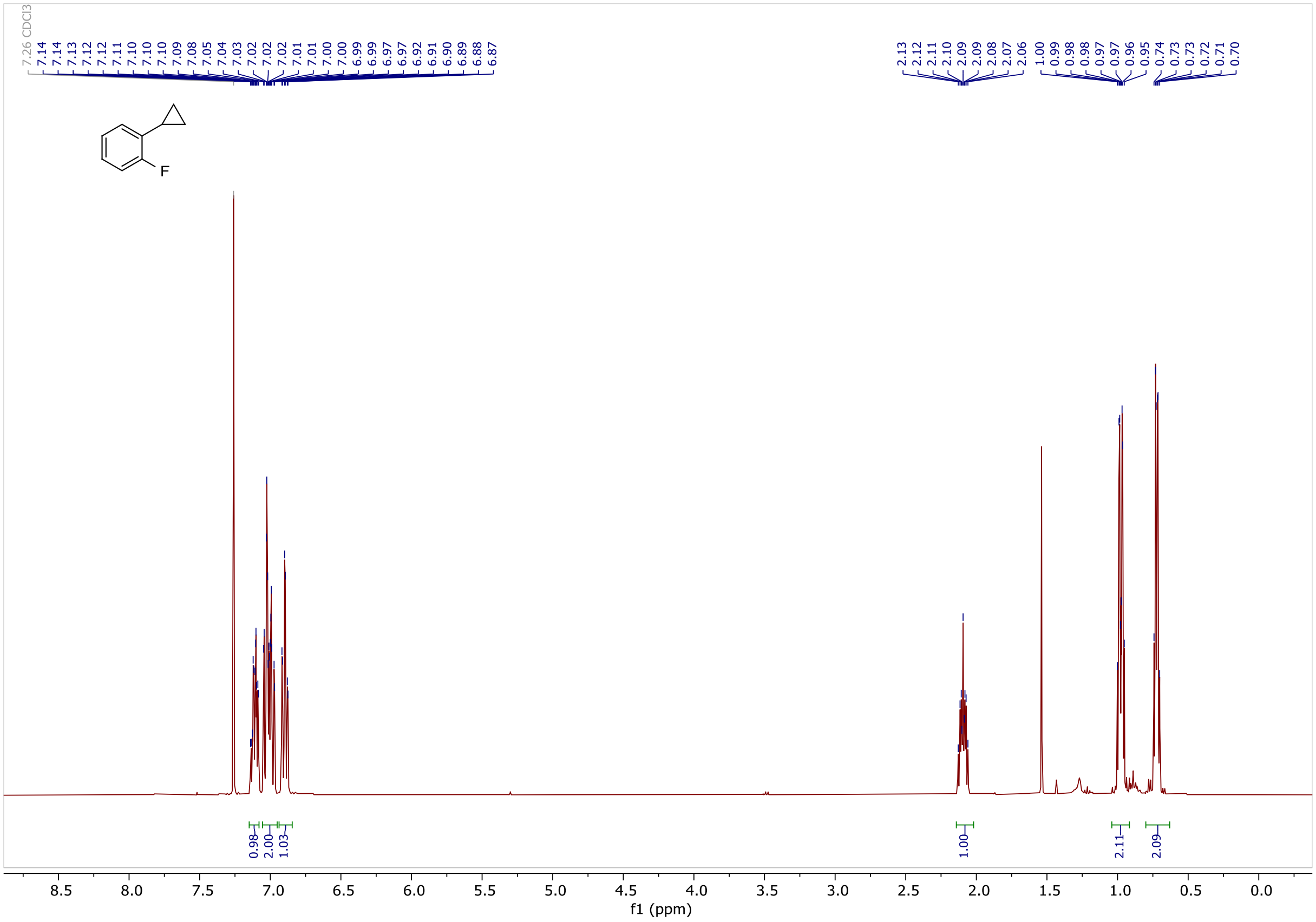

^{19}F NMR Spectrum of *o*-5m (376 MHz, $CDCl_3$)

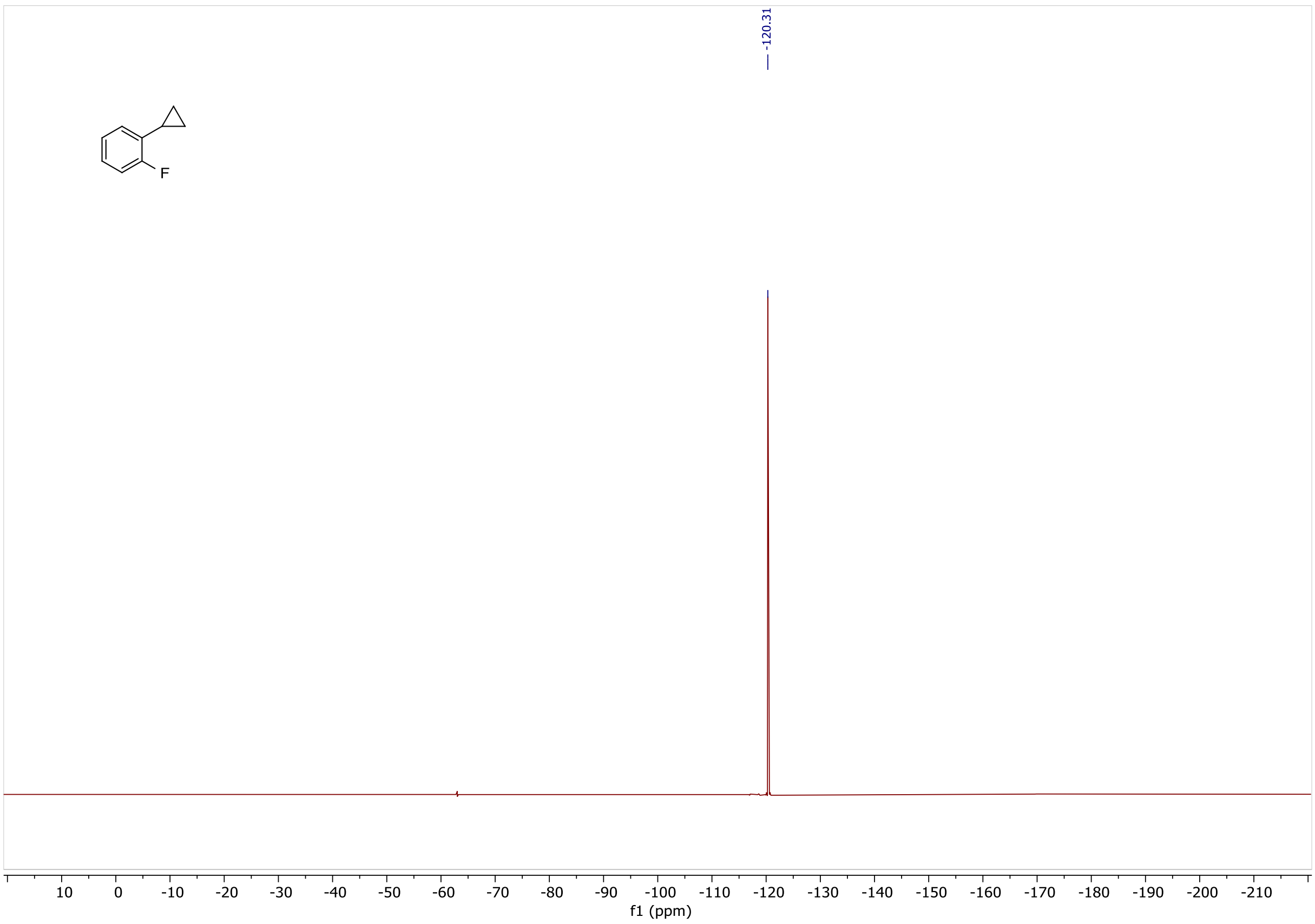

^{13}C NMR Spectrum of *o*-5m (101 MHz, $CDCl_3$)

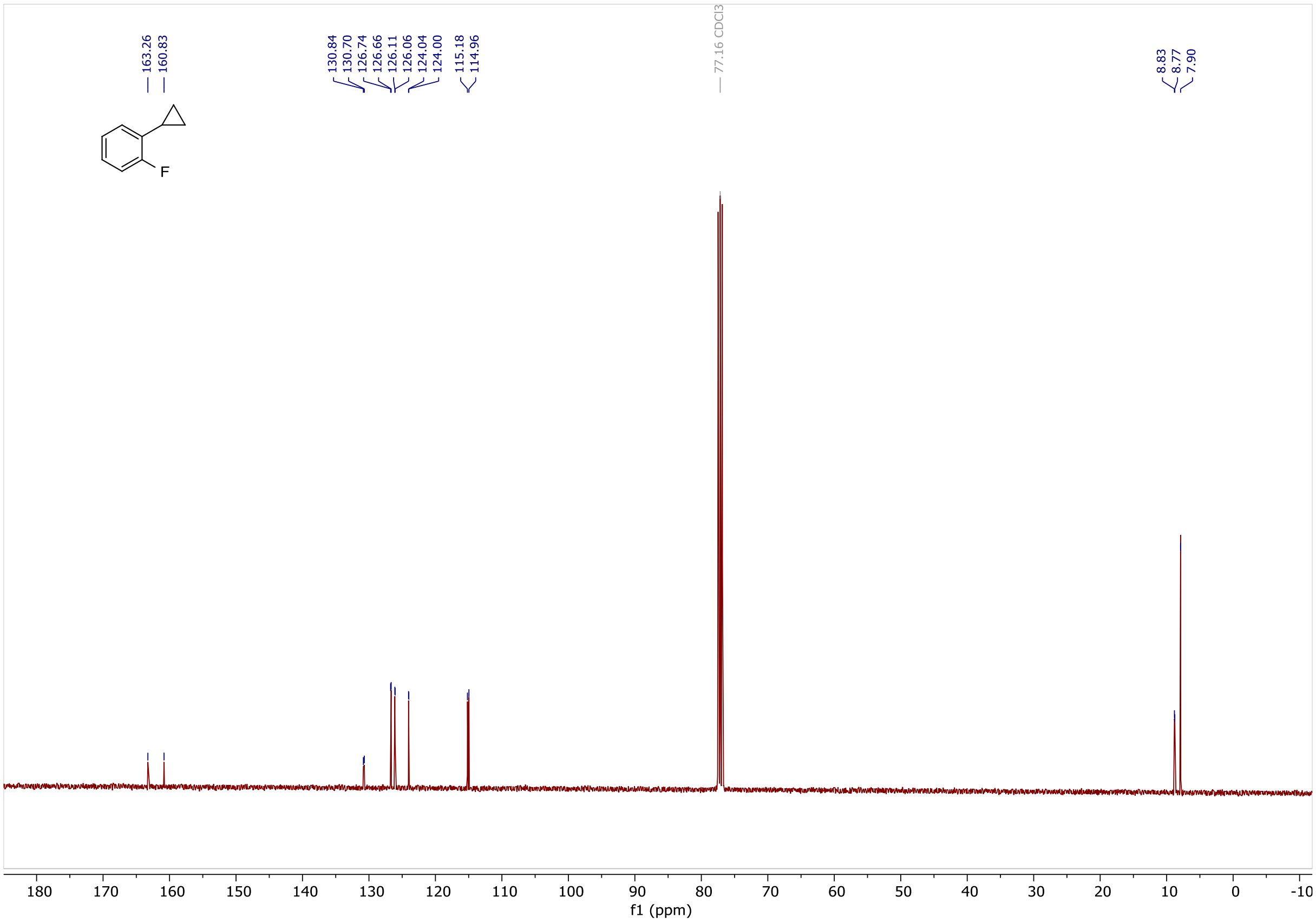

^{1}H NMR Spectrum of S2 (400 MHz, $CDCl_3$)

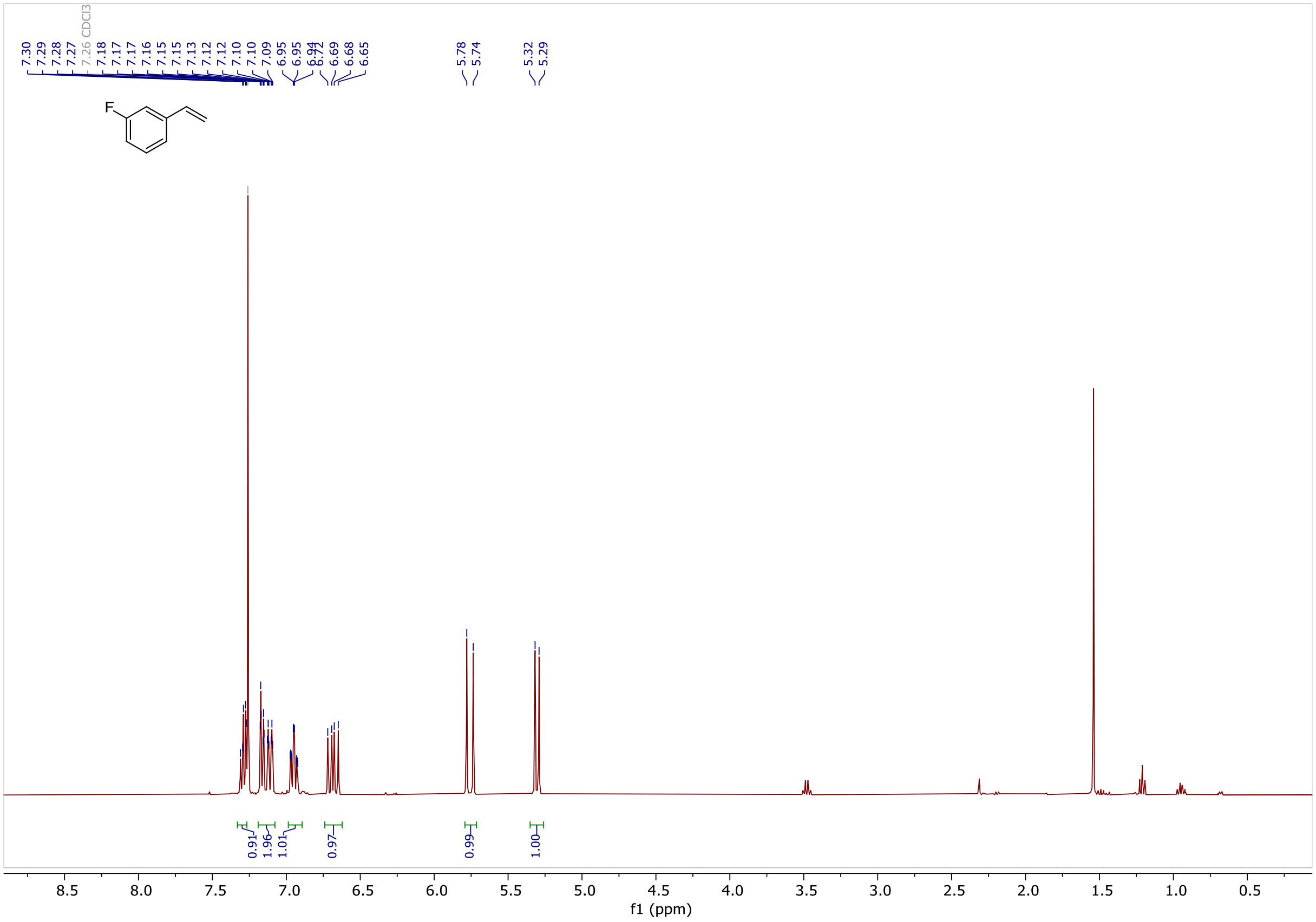

^{19}F NMR Spectrum of S2 (376 MHz, $CDCl_3$)

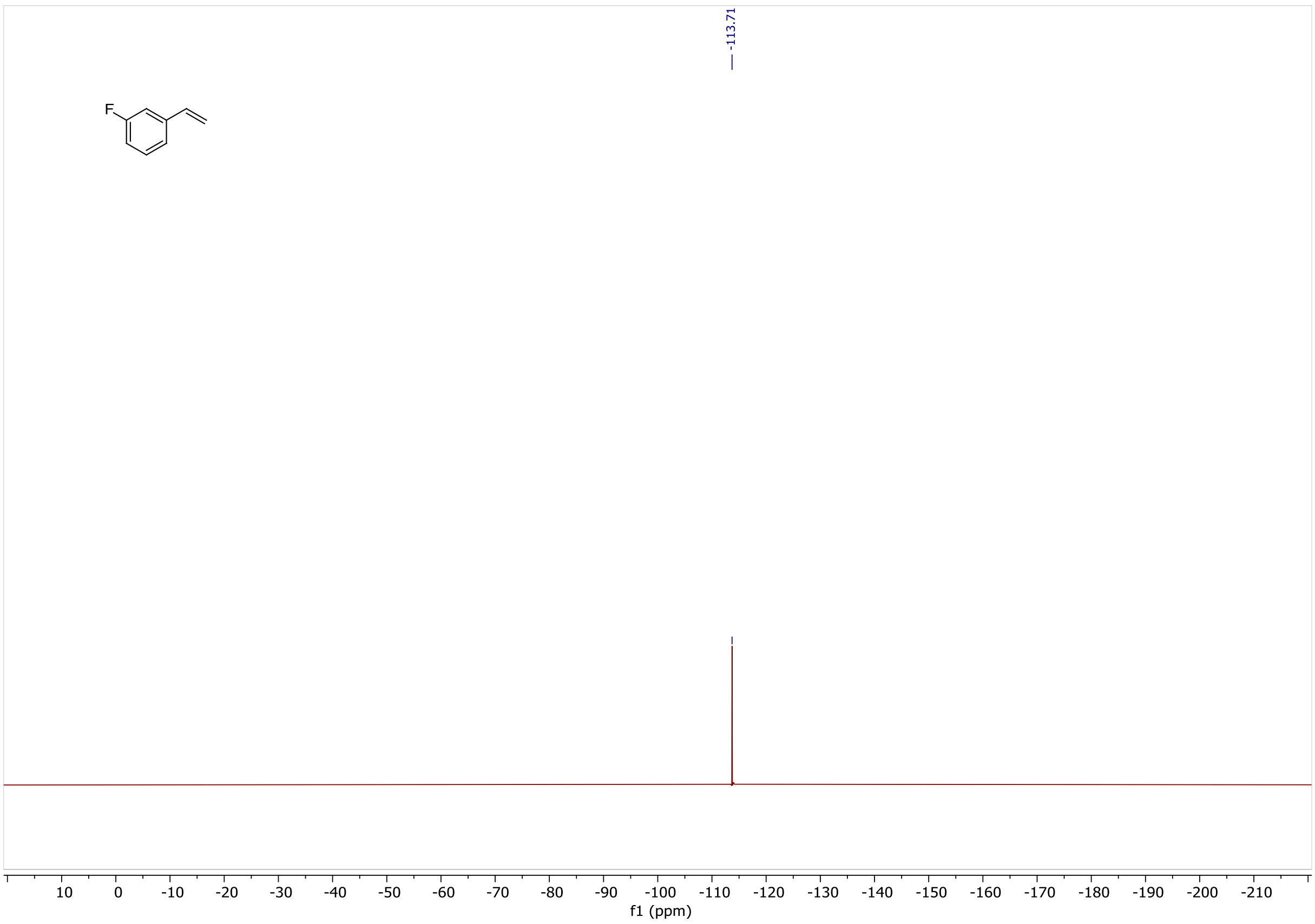

^{13}C NMR Spectrum of S2 (101 MHz, $CDCl_3$)

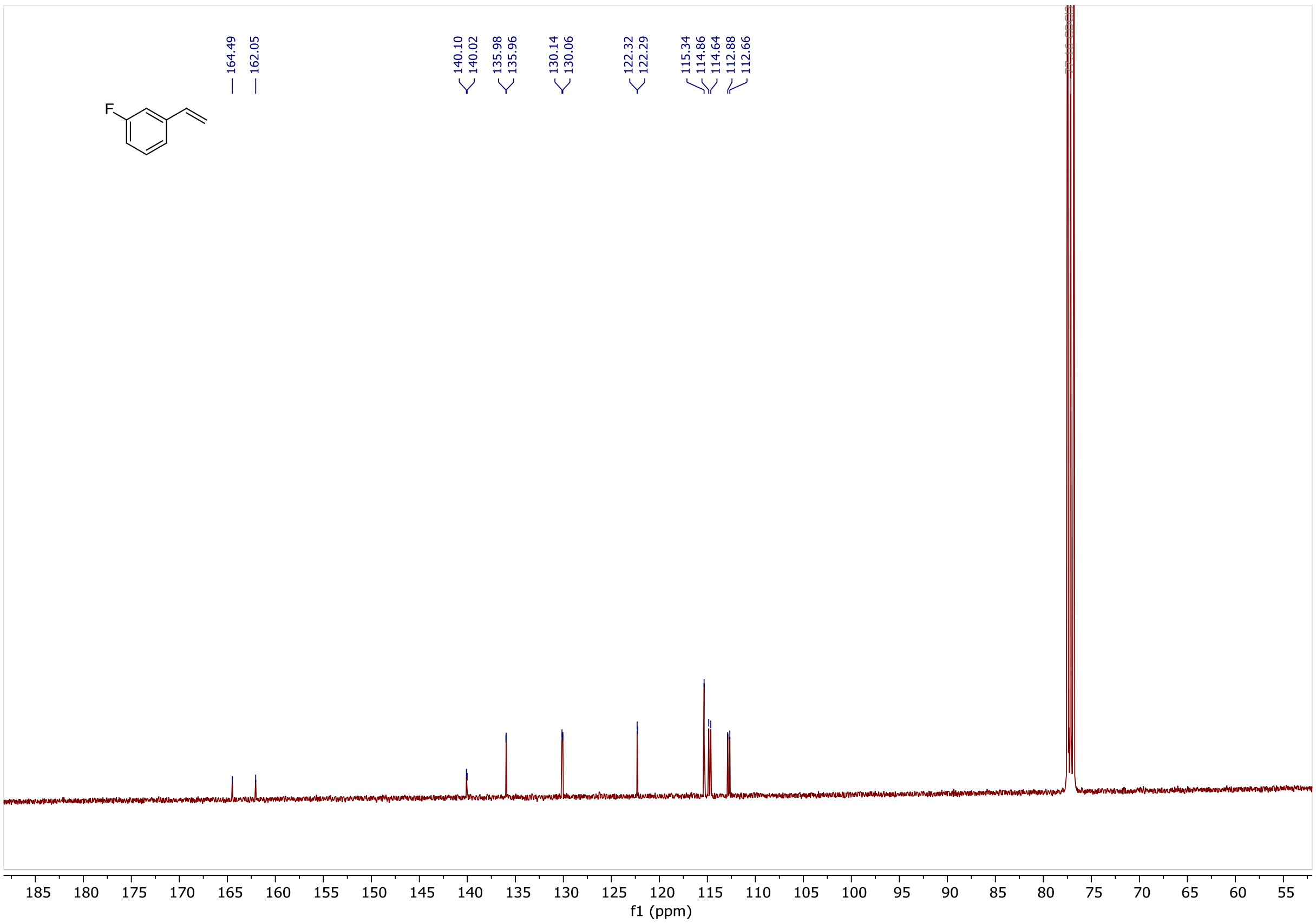

^{1}H NMR Spectrum of *m*-5m (400 MHz, $CDCl_3$)

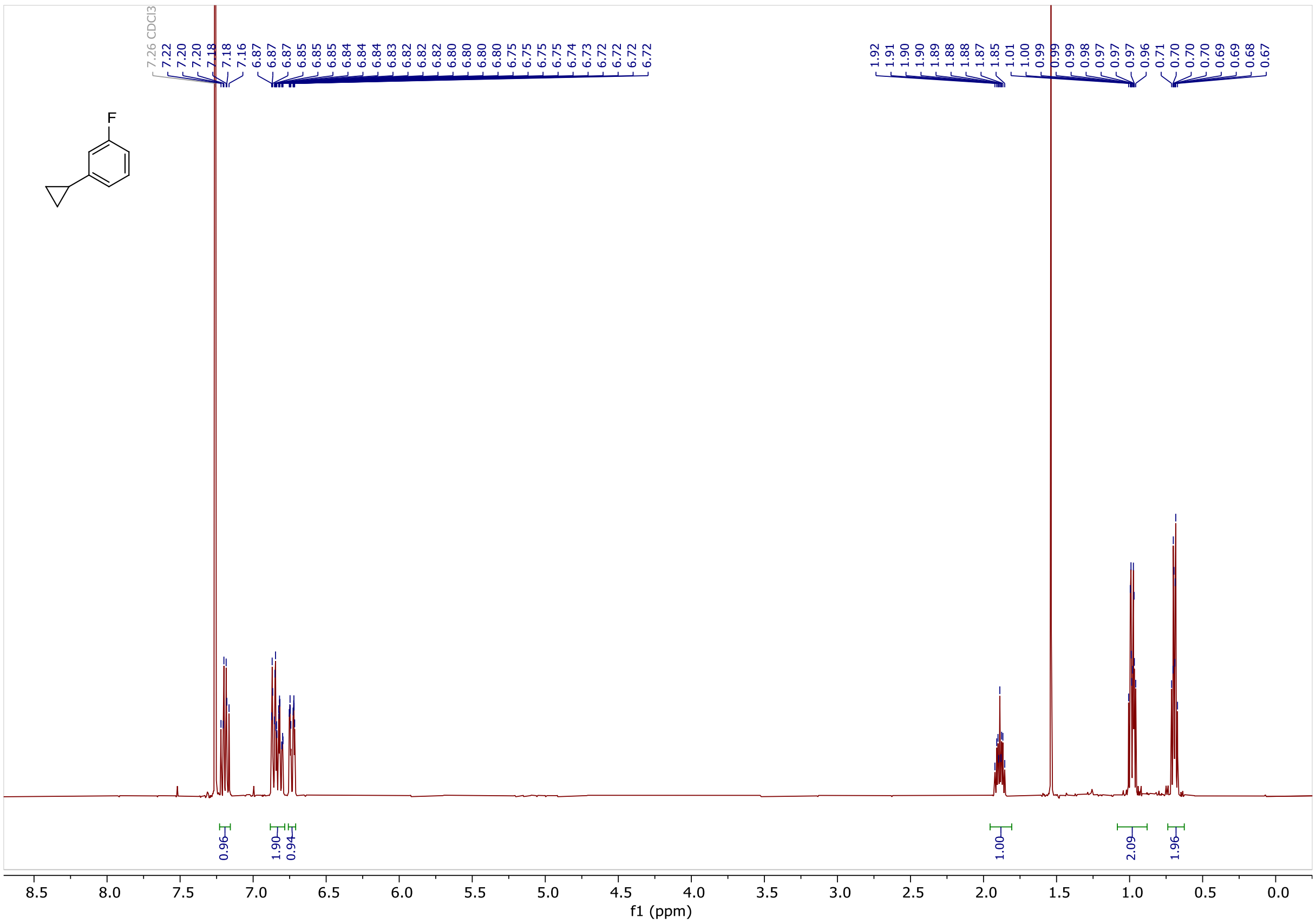

^{19}F NMR Spectrum of *m*-5m (376 MHz, $CDCl_3$)

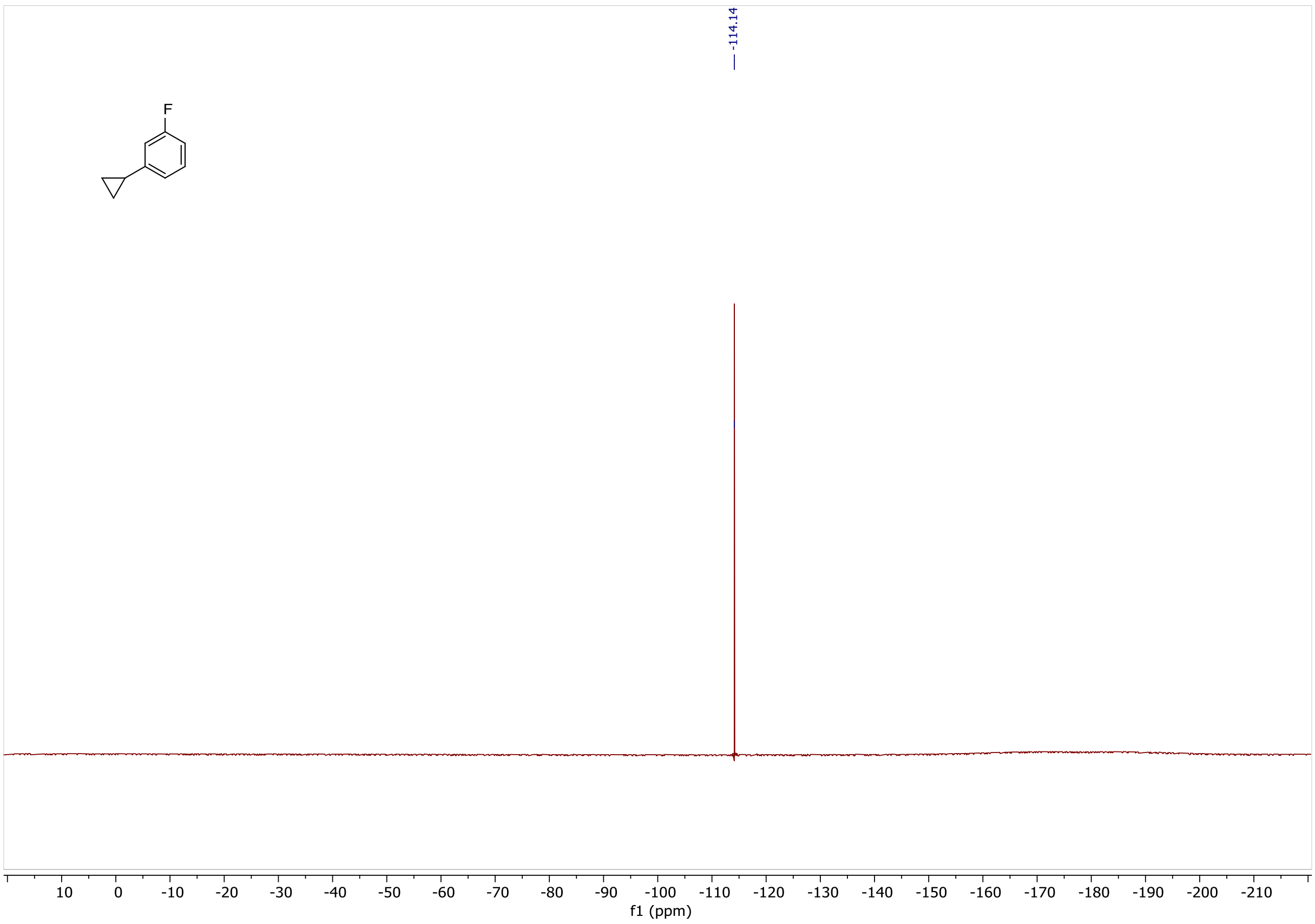

^{13}C NMR Spectrum of *m*-5m (101 MHz, CDCl$_3$)

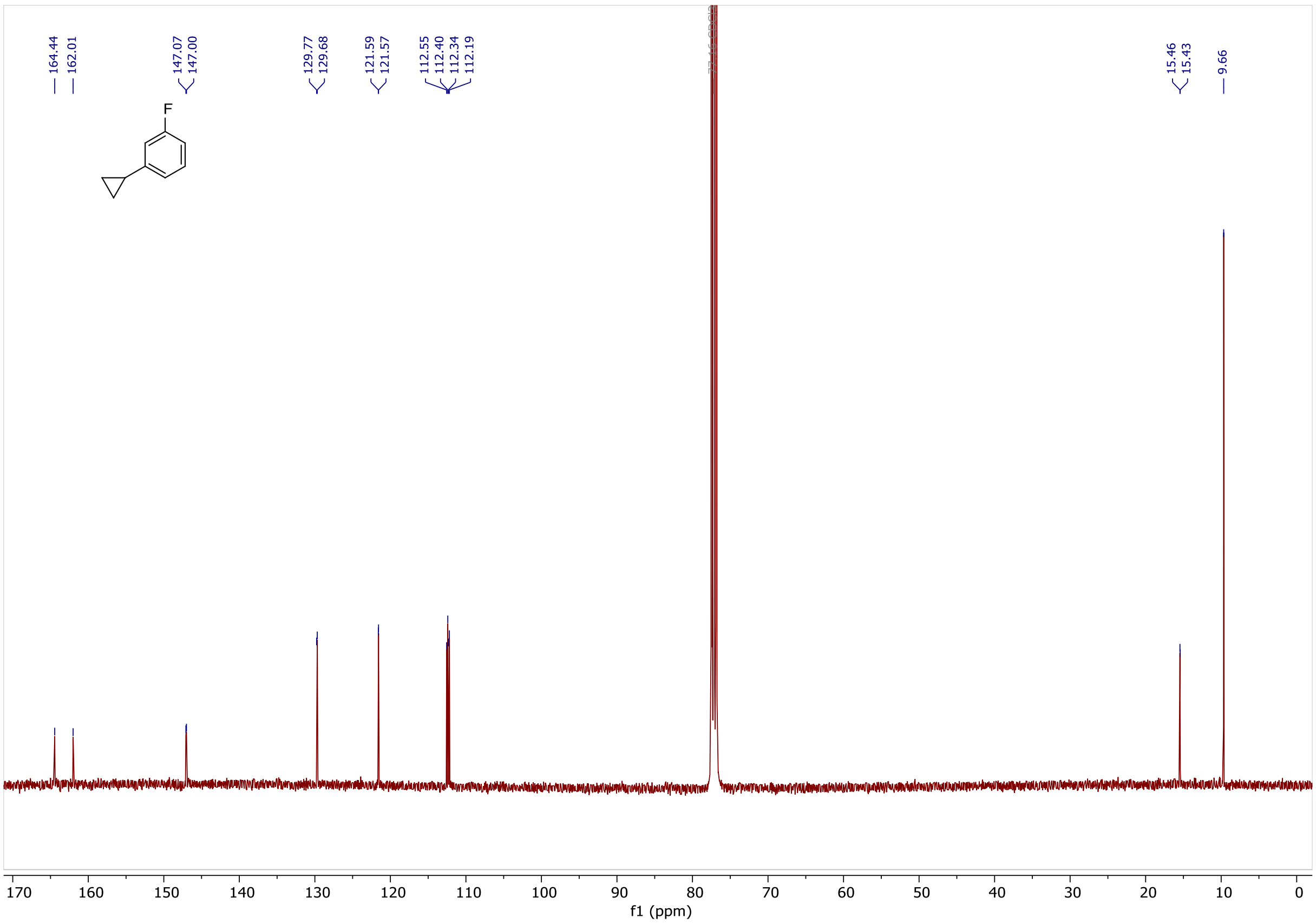

^{1}H NMR Spectrum of *p*-5m (400 MHz, $CDCl_3$)

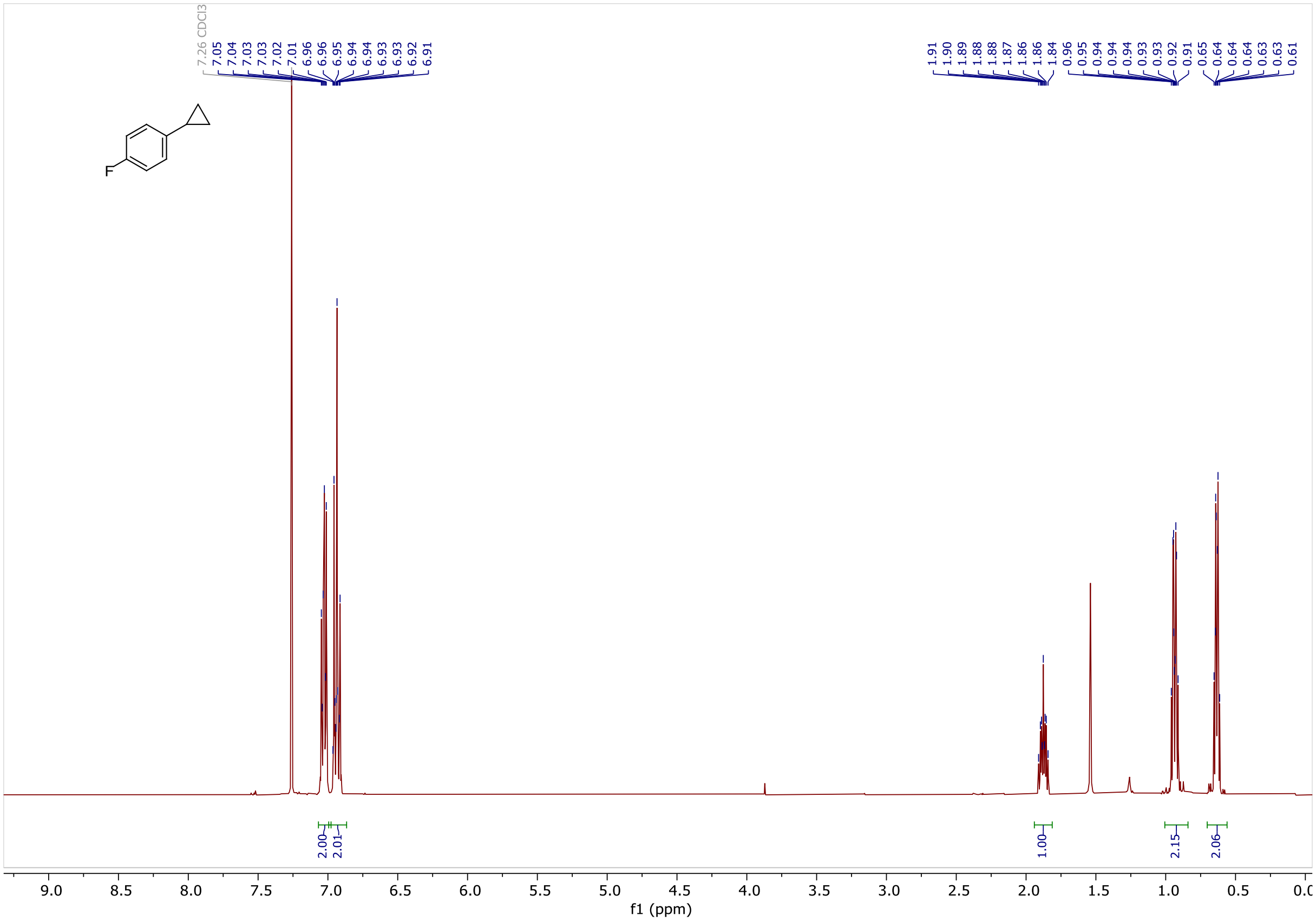

^{19}F NMR Spectrum of *p*-5m (376 MHz, $CDCl_3$)

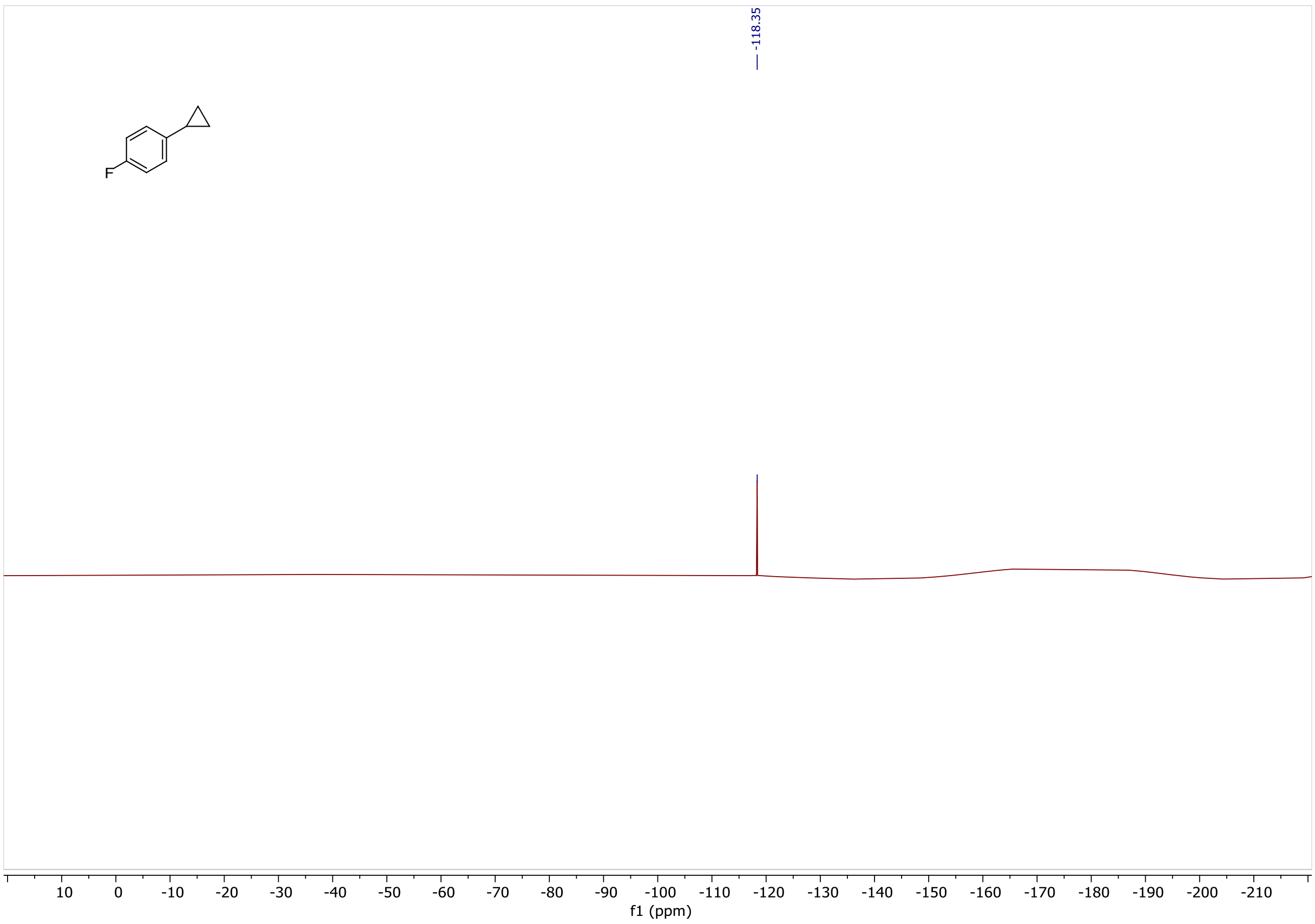

^{13}C NMR Spectrum of *p*-5m (101 MHz, $CDCl_3$)

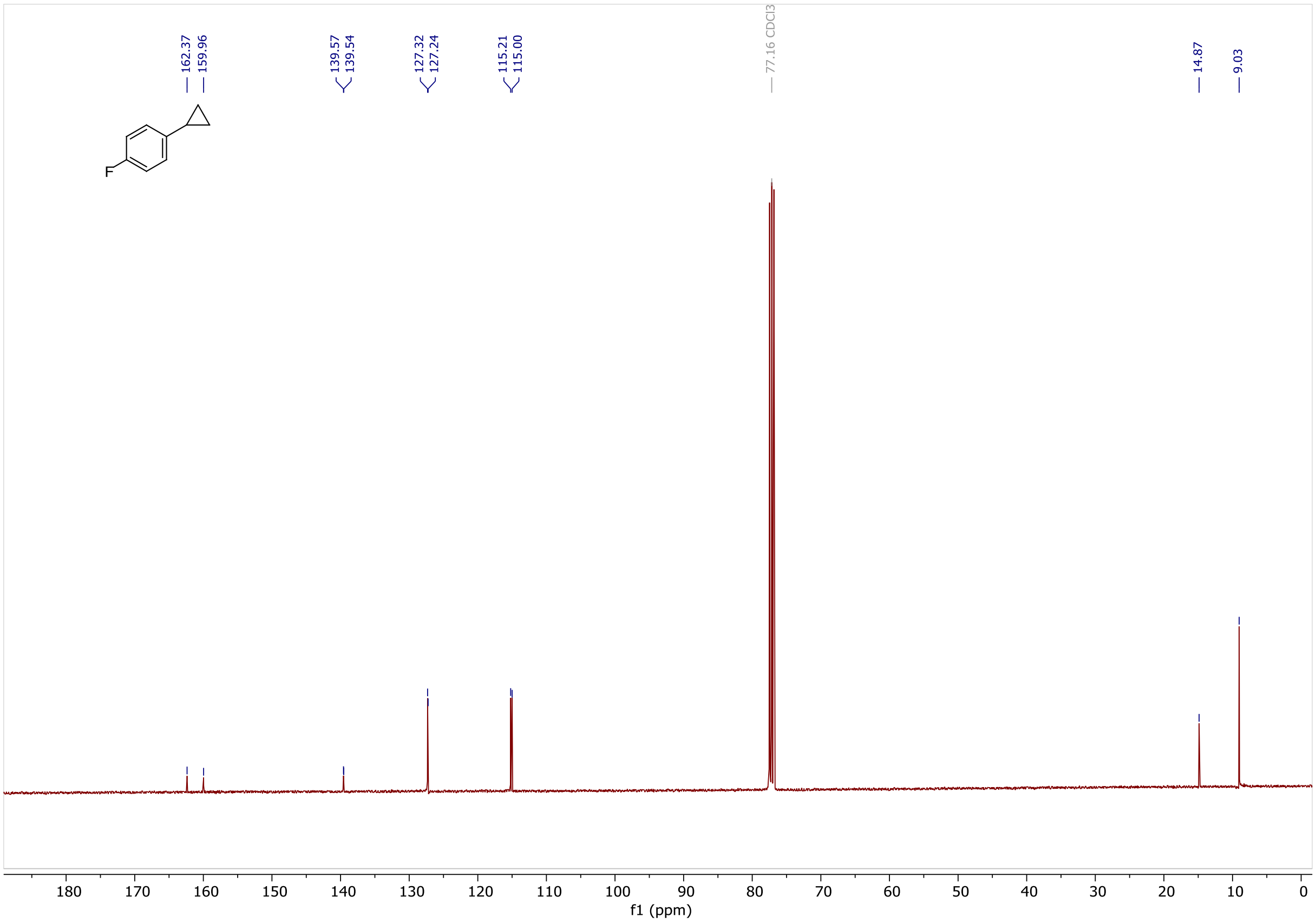

^{1}H NMR Spectrum of 5n (400 MHz, $CDCl_3$)

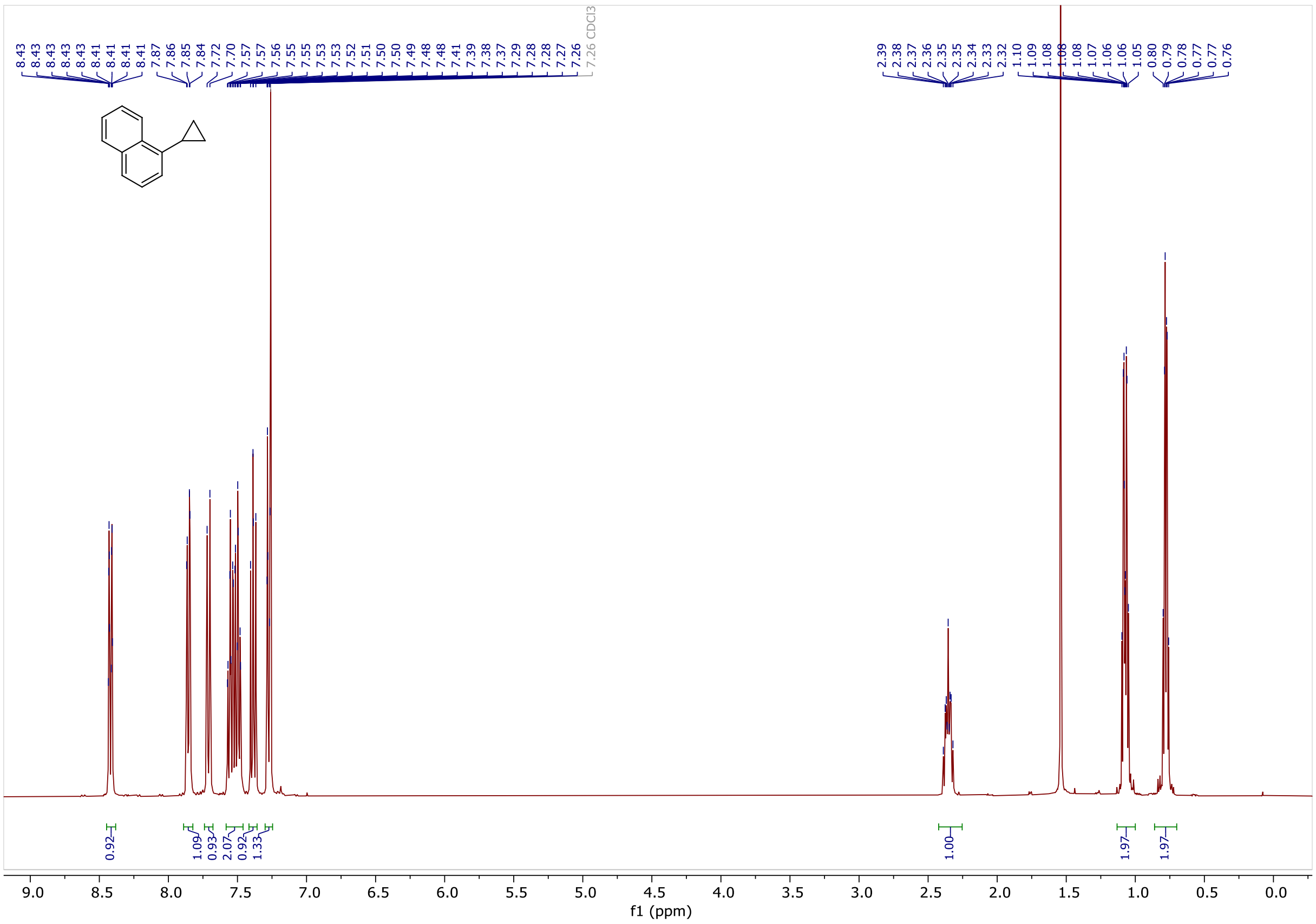

^{13}C NMR Spectrum of 5n (101 MHz, $CDCl_3$)

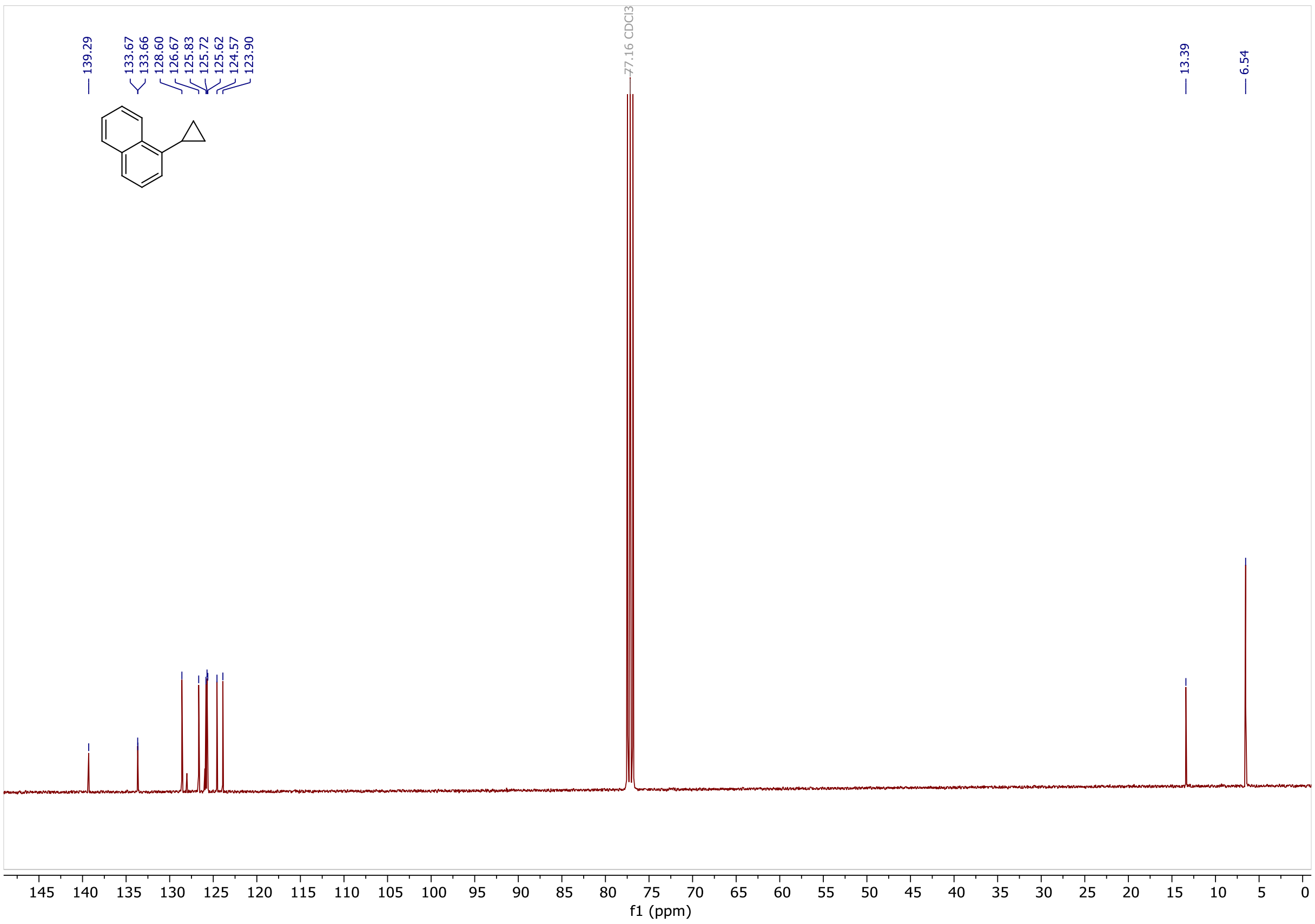

^{1}H NMR Spectrum of 5o (400 MHz, $CDCl_3$)

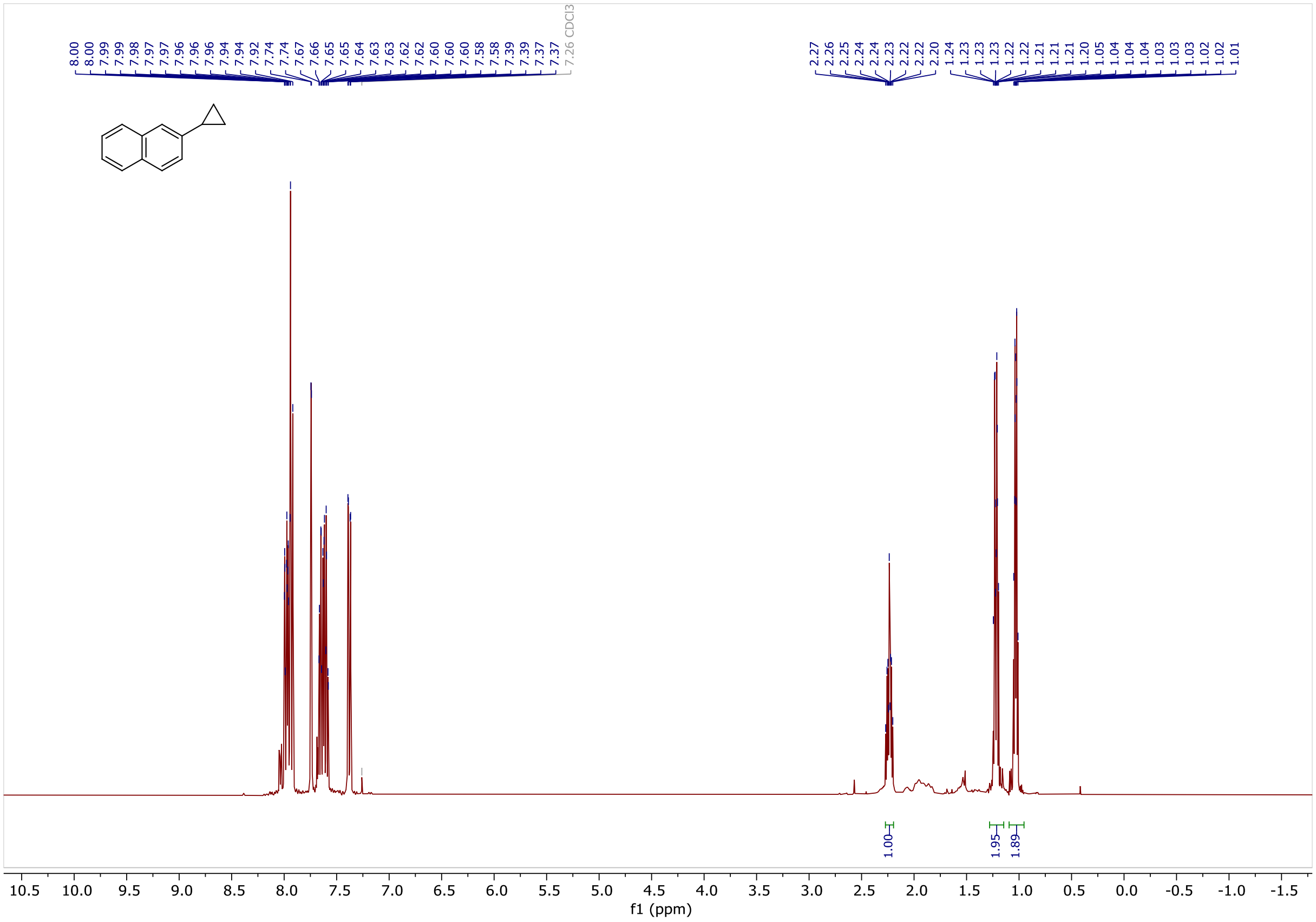

^{13}C NMR Spectrum of 5o (101 MHz, $CDCl_3$)

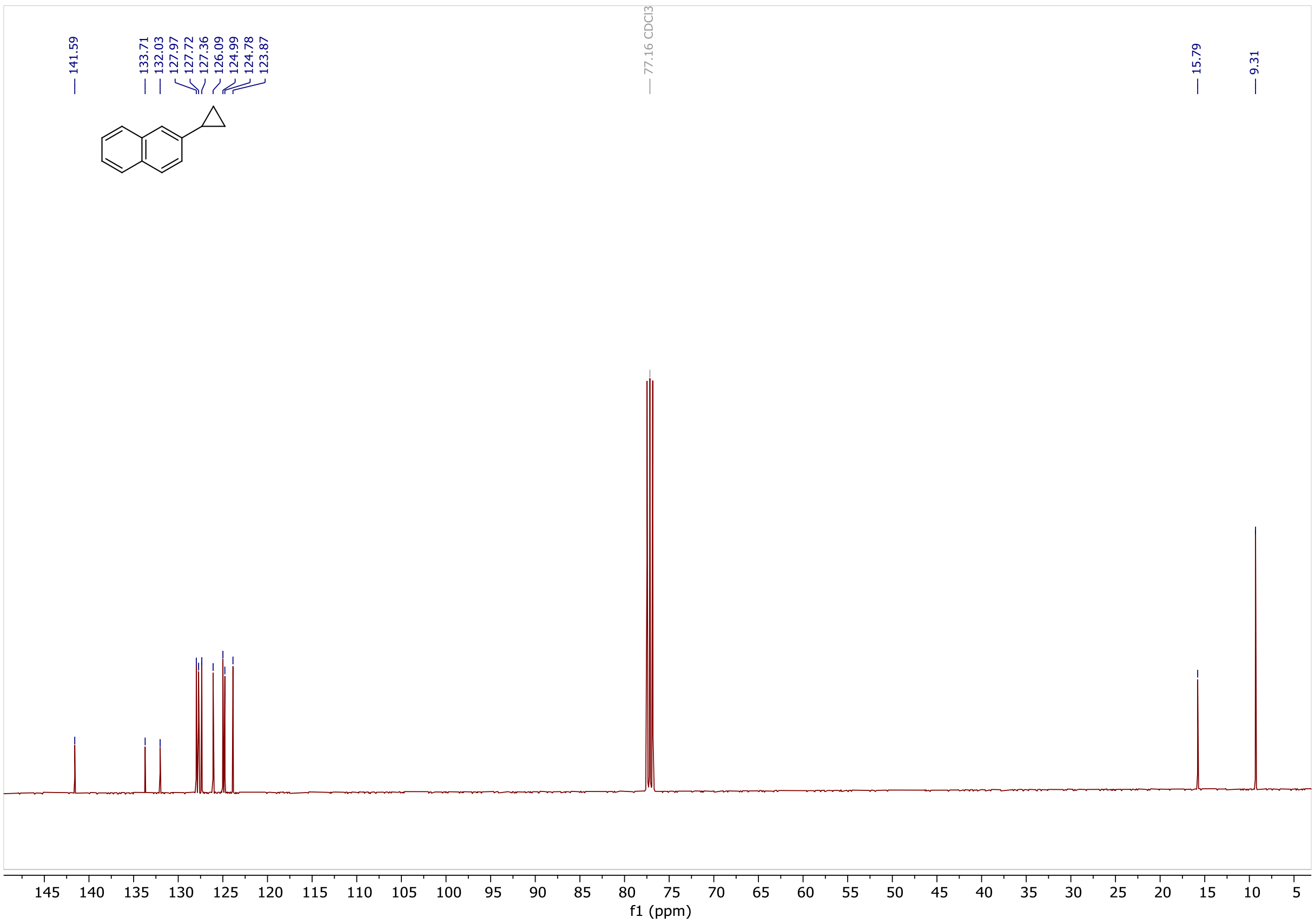

^{1}H NMR Spectrum of S3 (400 MHz, $CDCl_3$)

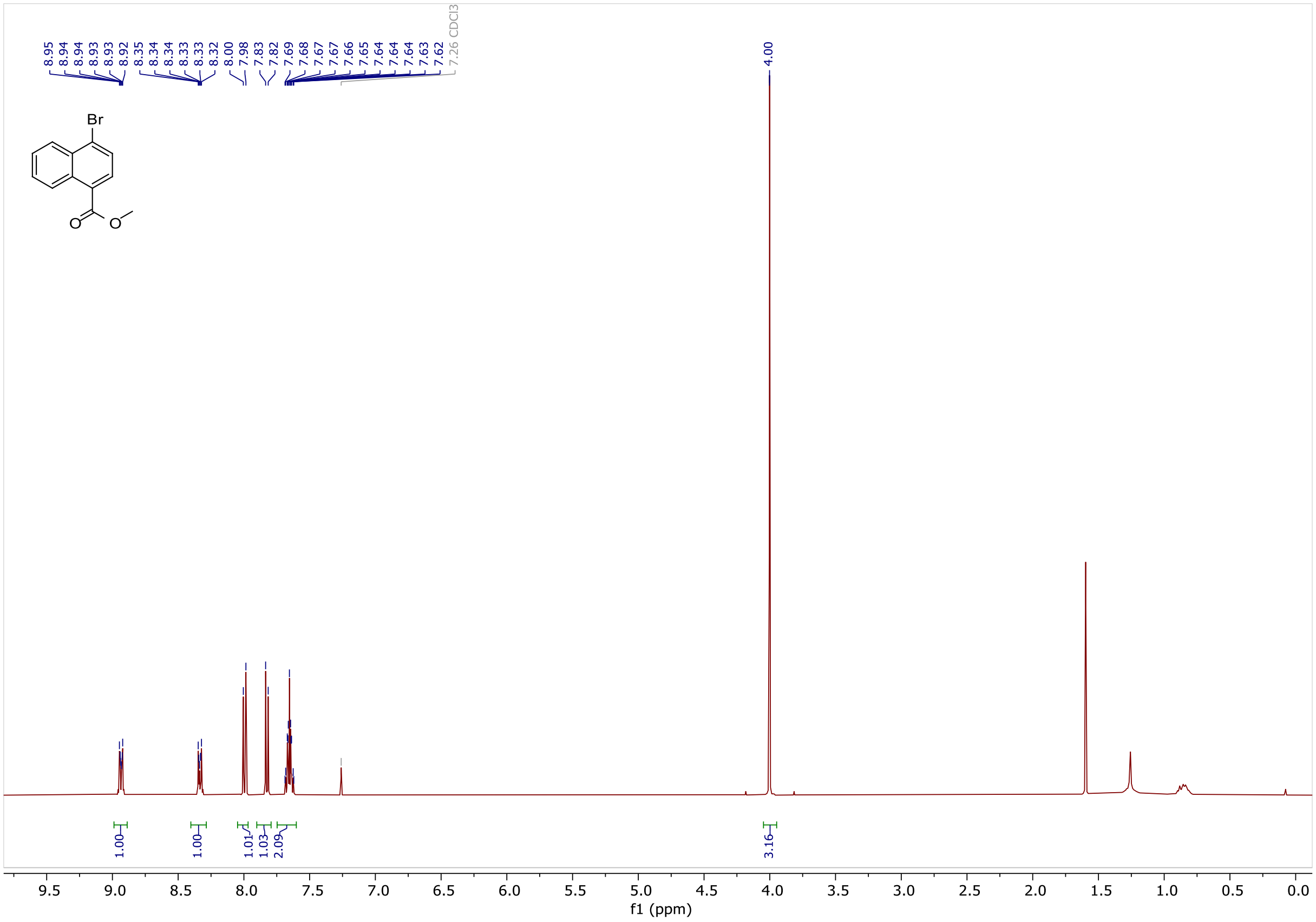

^{13}C NMR Spectrum of S3 (101 MHz, $CDCl_3$)

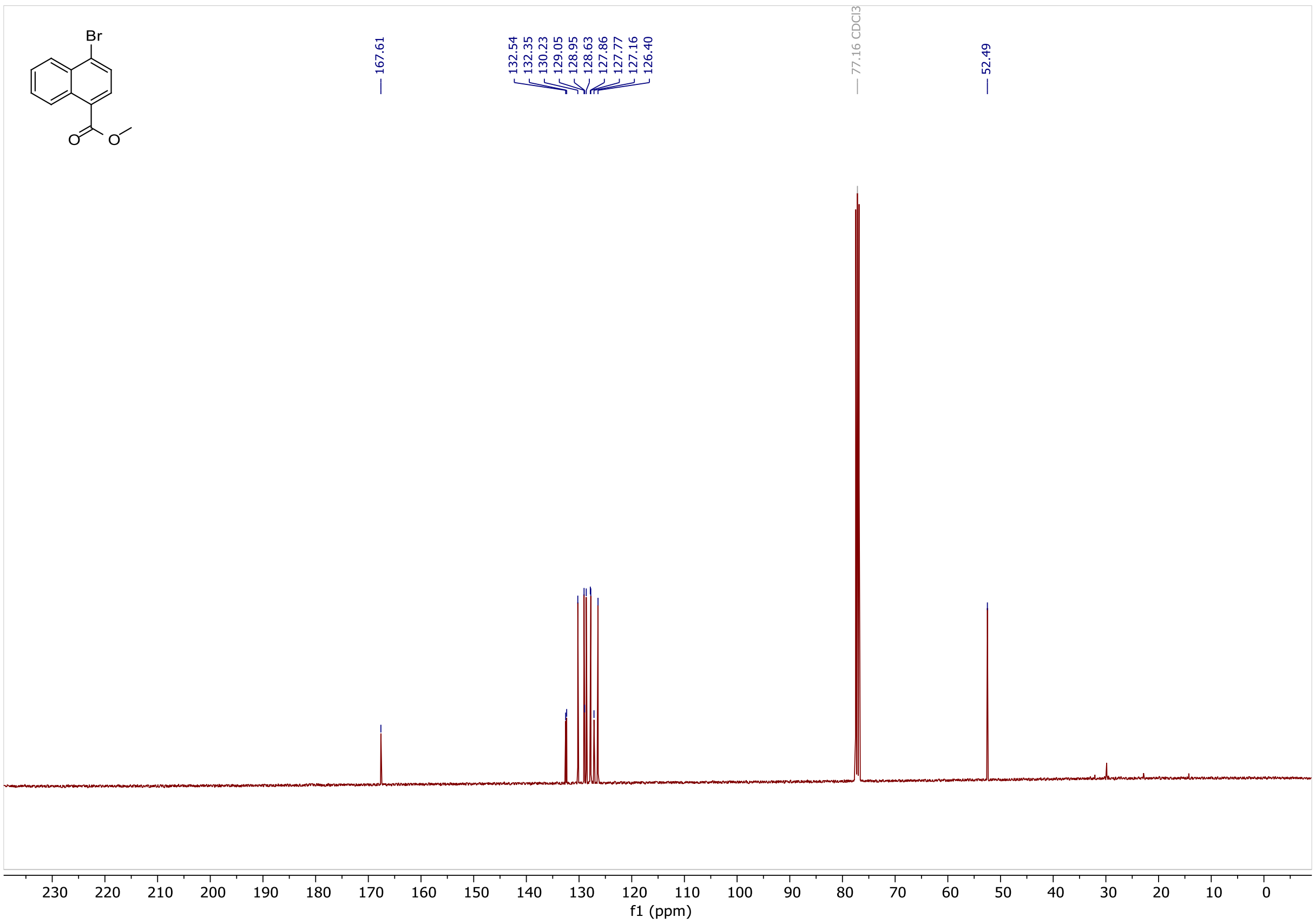

^{1}H NMR Spectrum of 5p (400 MHz, $CDCl_3$)

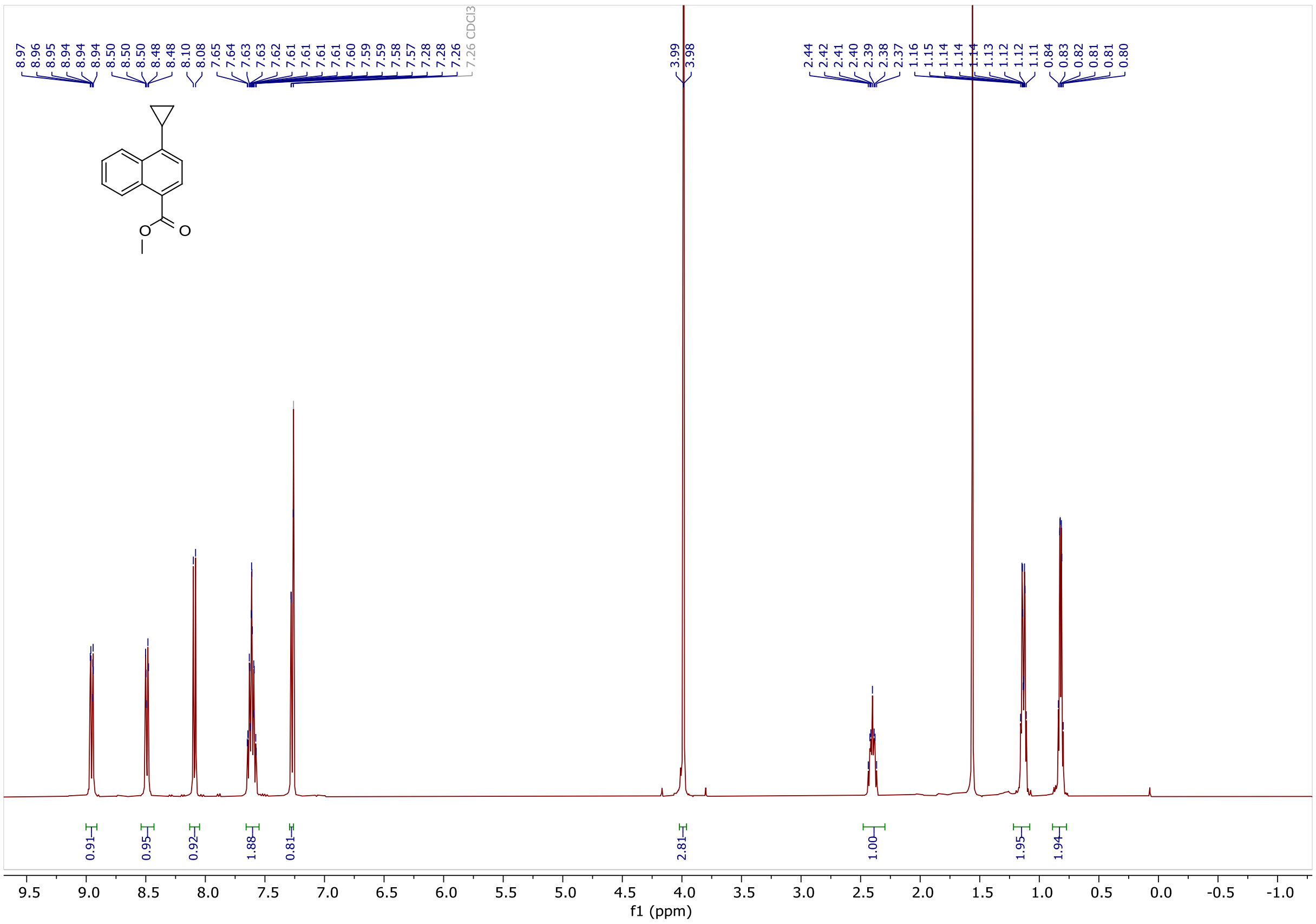

^{13}C NMR Spectrum of 5p (101 MHz, $CDCl_3$)

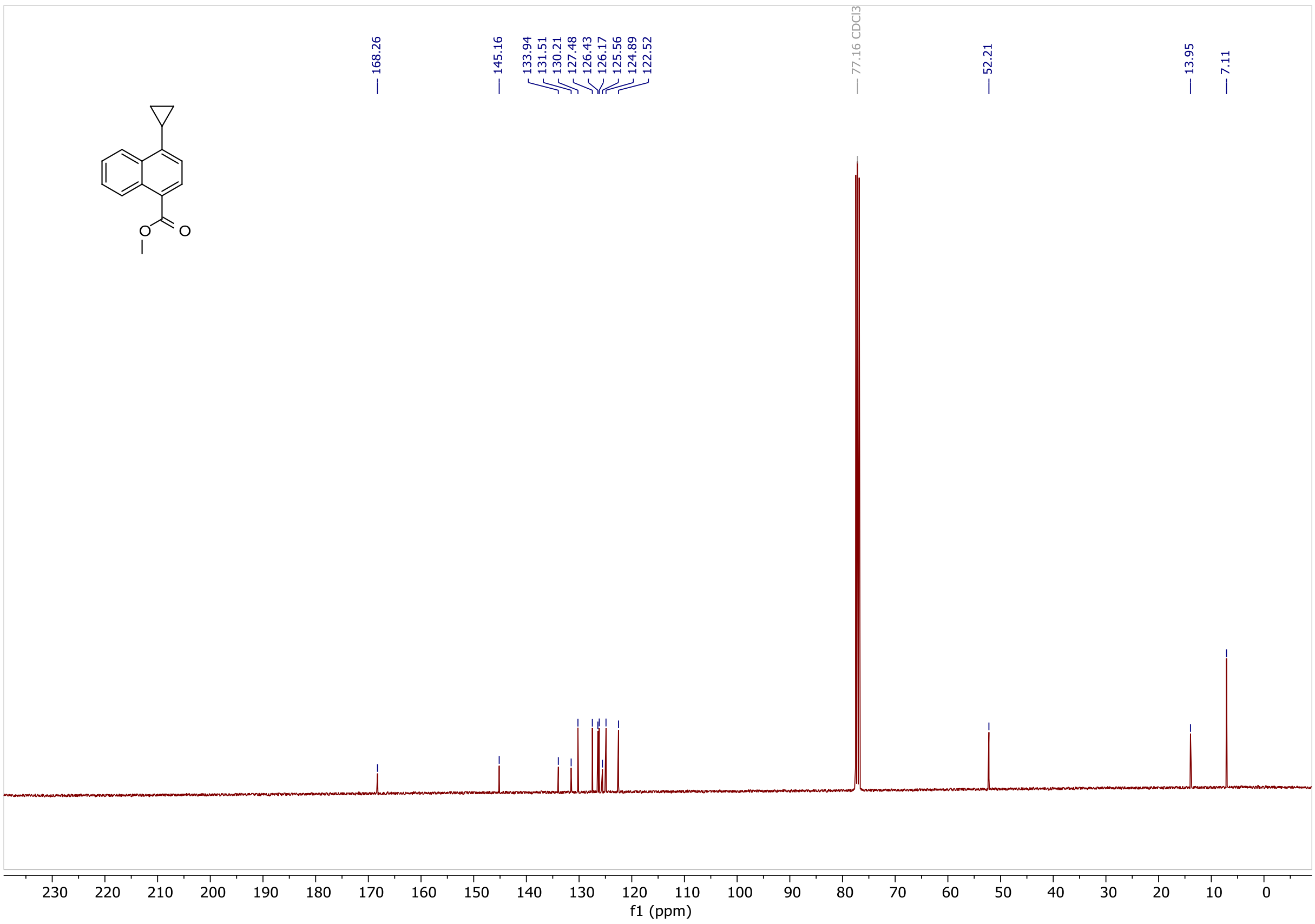

^{1}H NMR Spectrum of 5q (400 MHz, $CDCl_3$)

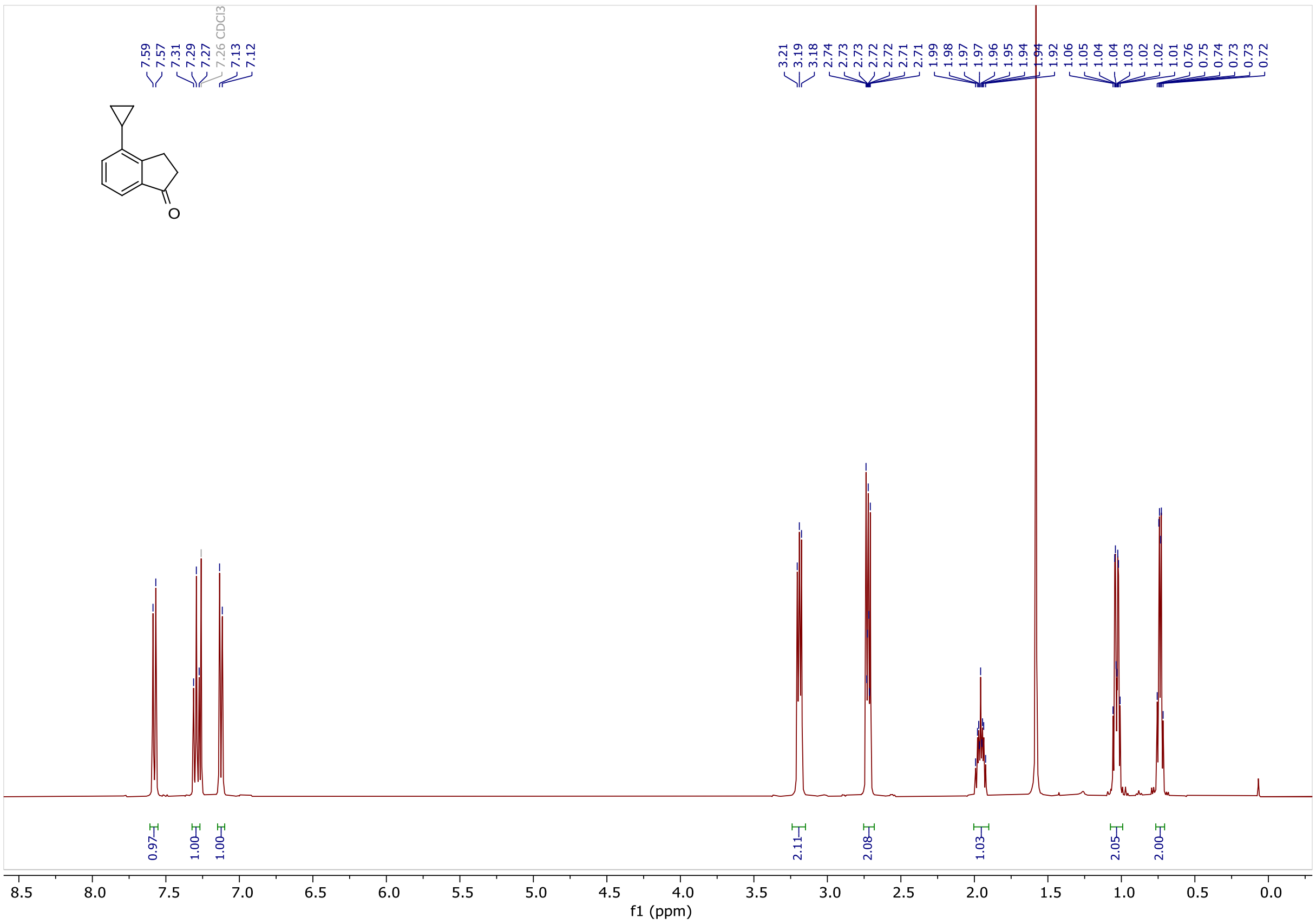

^{13}C NMR Spectrum of 5q (101 MHz, $CDCl_3$)

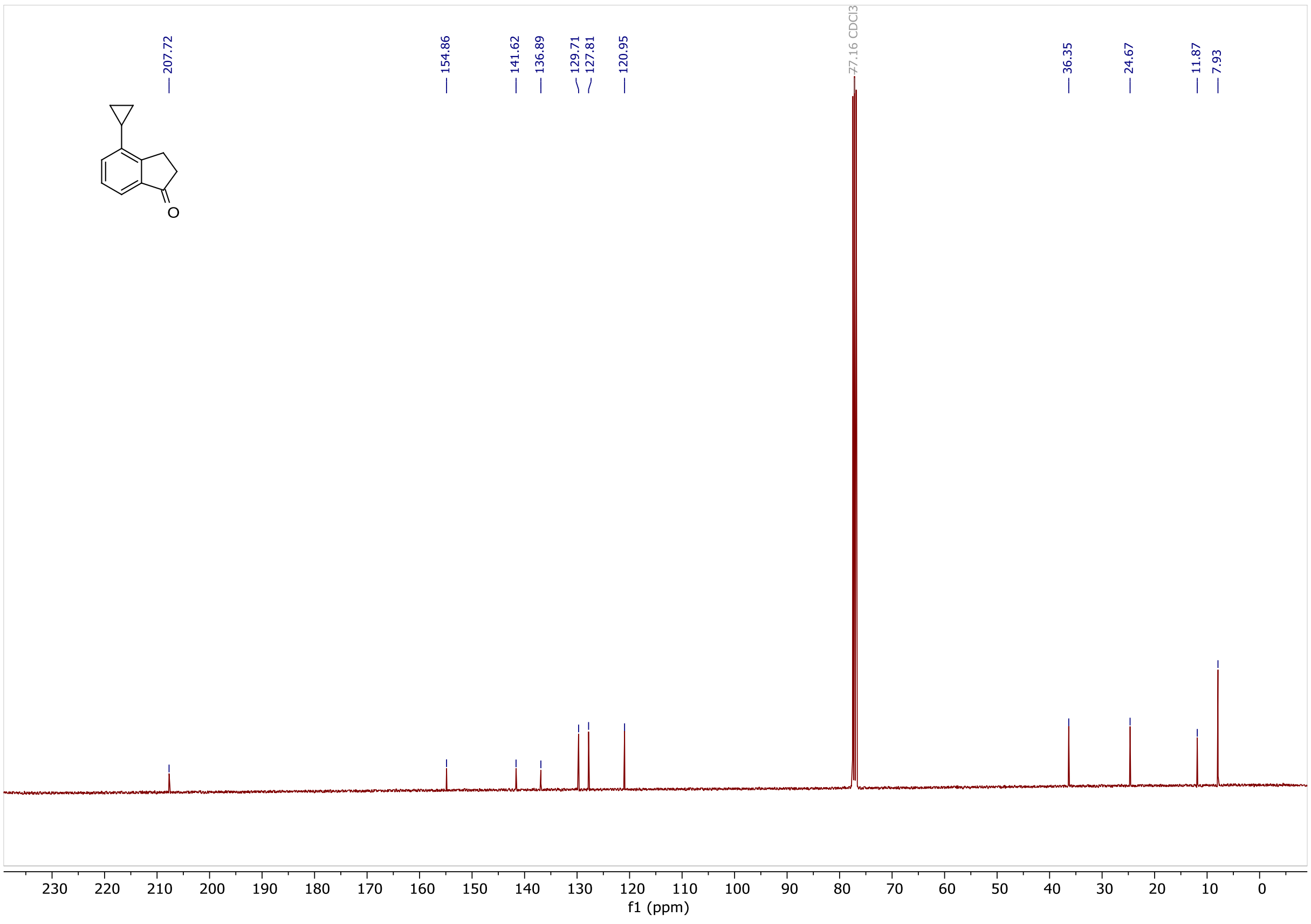

^{1}H NMR Spectrum of S4 (400 MHz, $CDCl_3$)

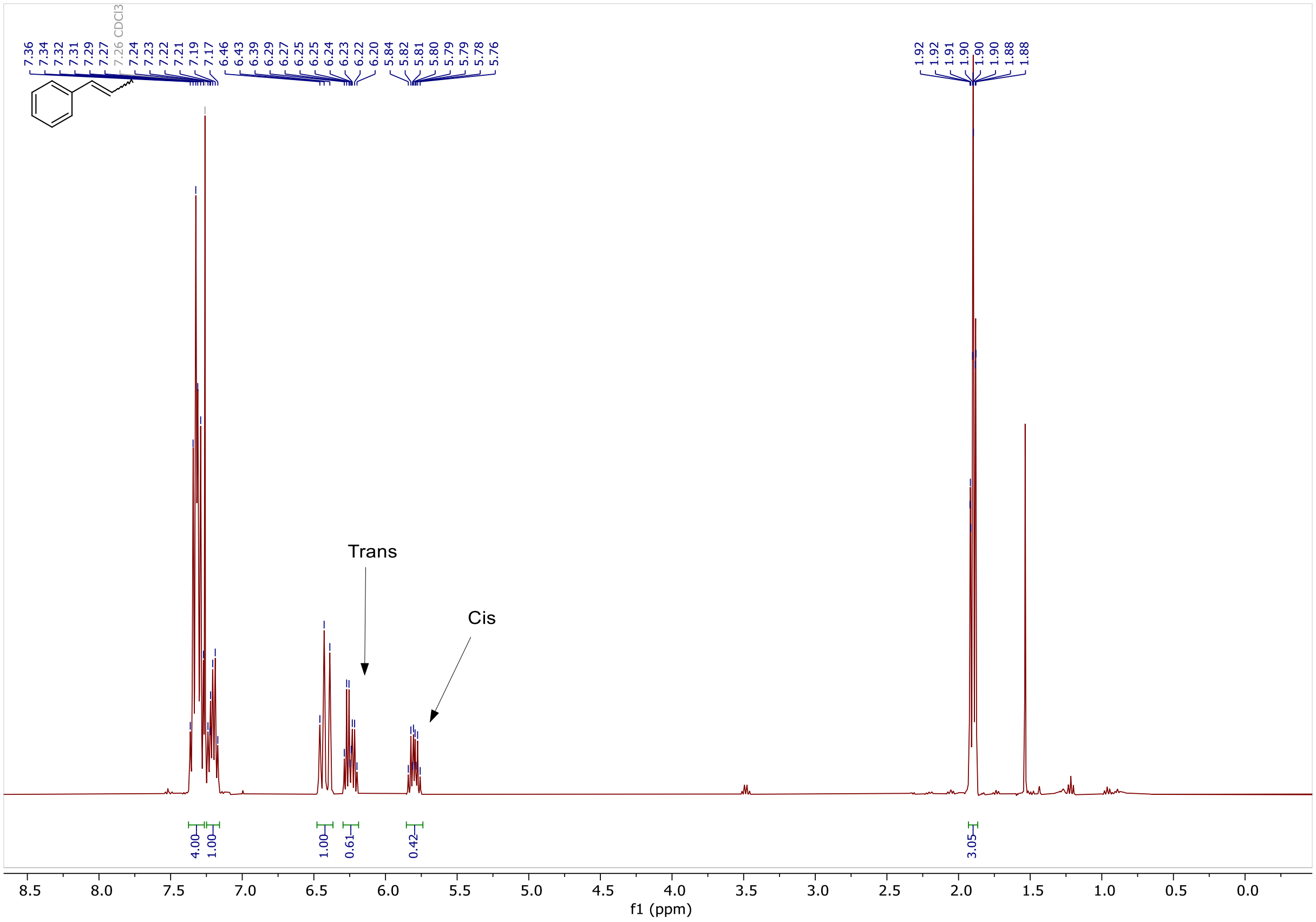

^{13}C NMR Spectrum of S4 (101 MHz, $CDCl_3$)

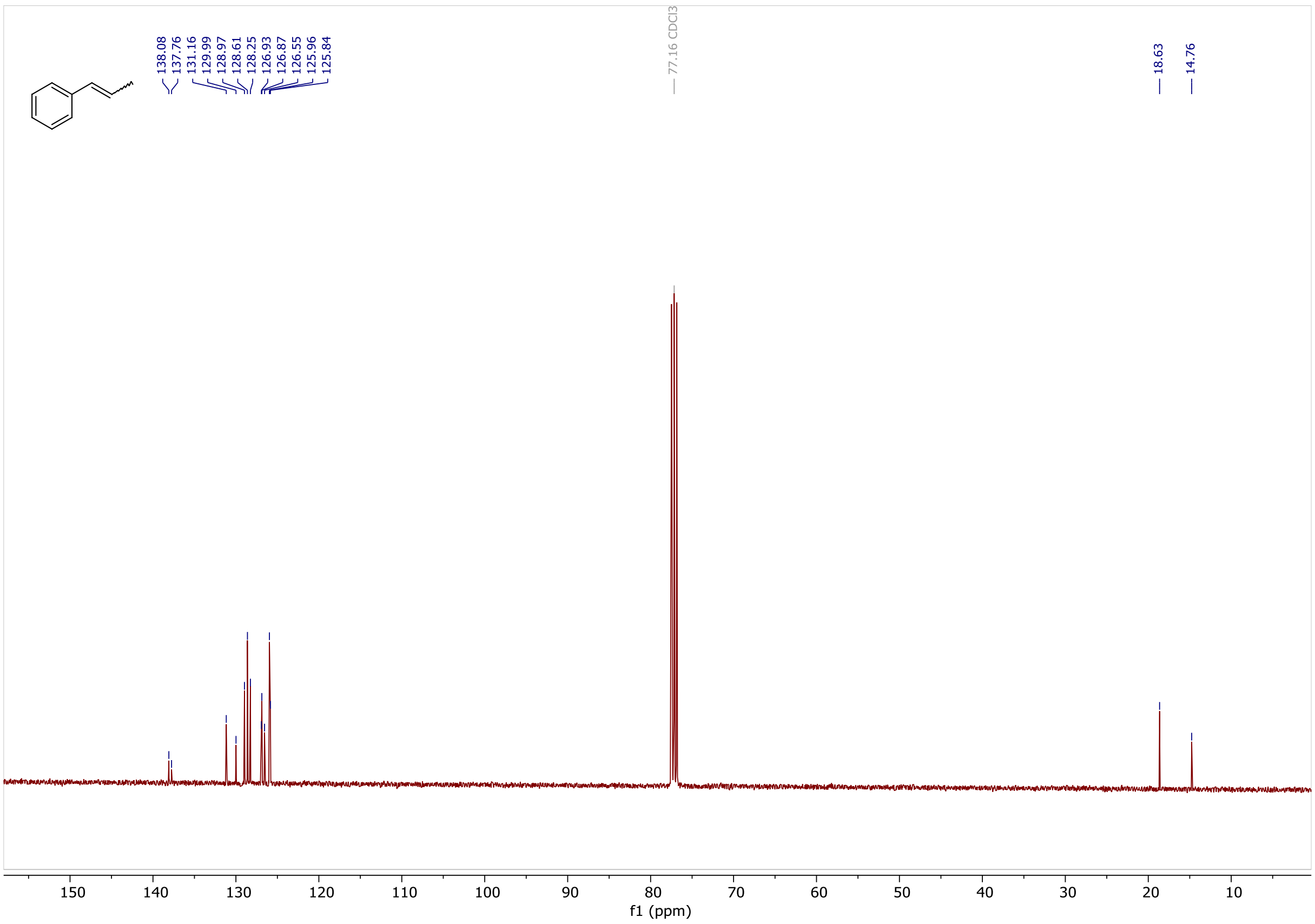

^{1}H NMR Spectrum of 5r (400 MHz, $CDCl_3$)

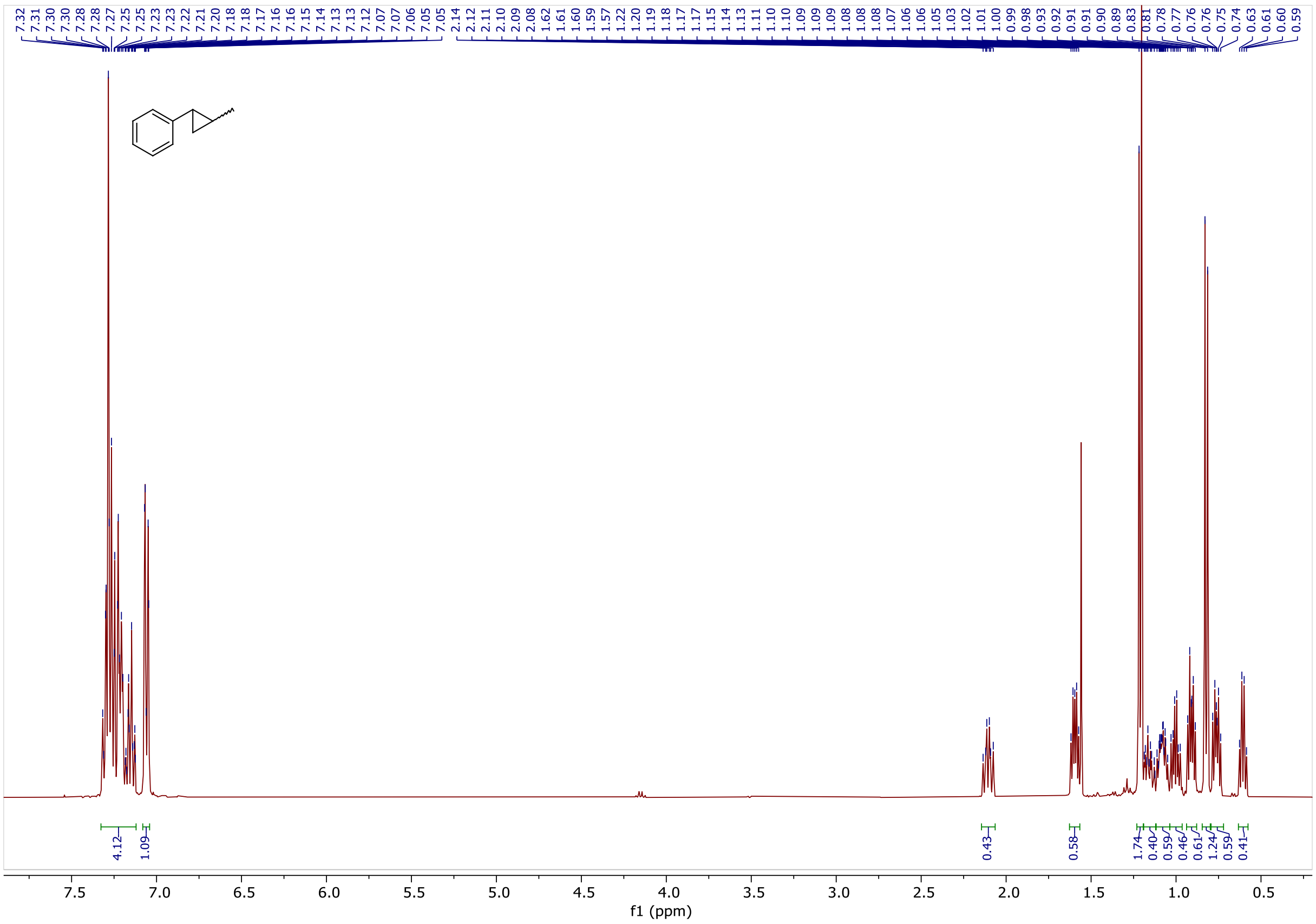

^{13}C NMR Spectrum of 5r (101 MHz, $CDCl_3$)

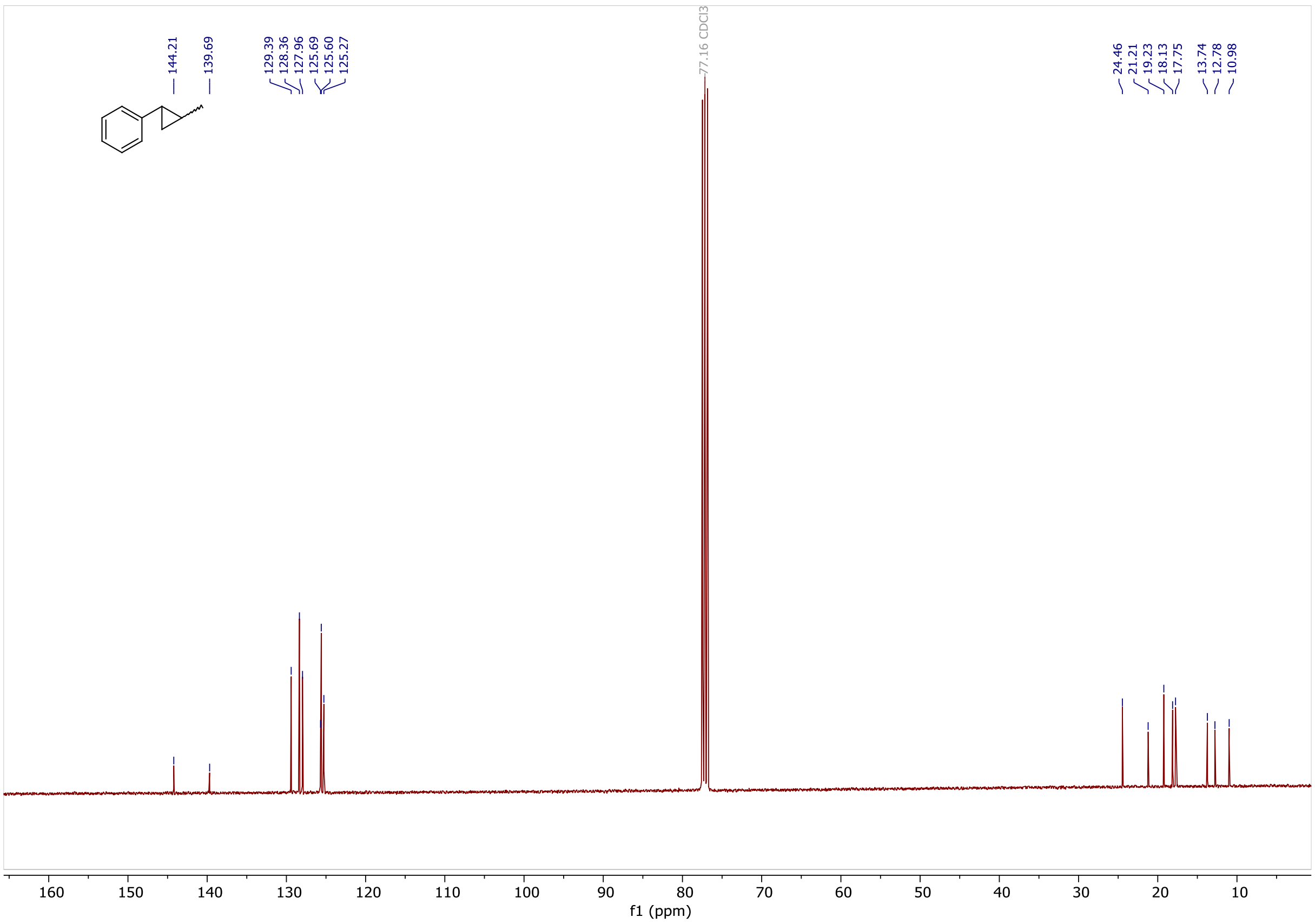

^{1}H NMR Spectrum of 5s (400 MHz, $CDCl_3$)

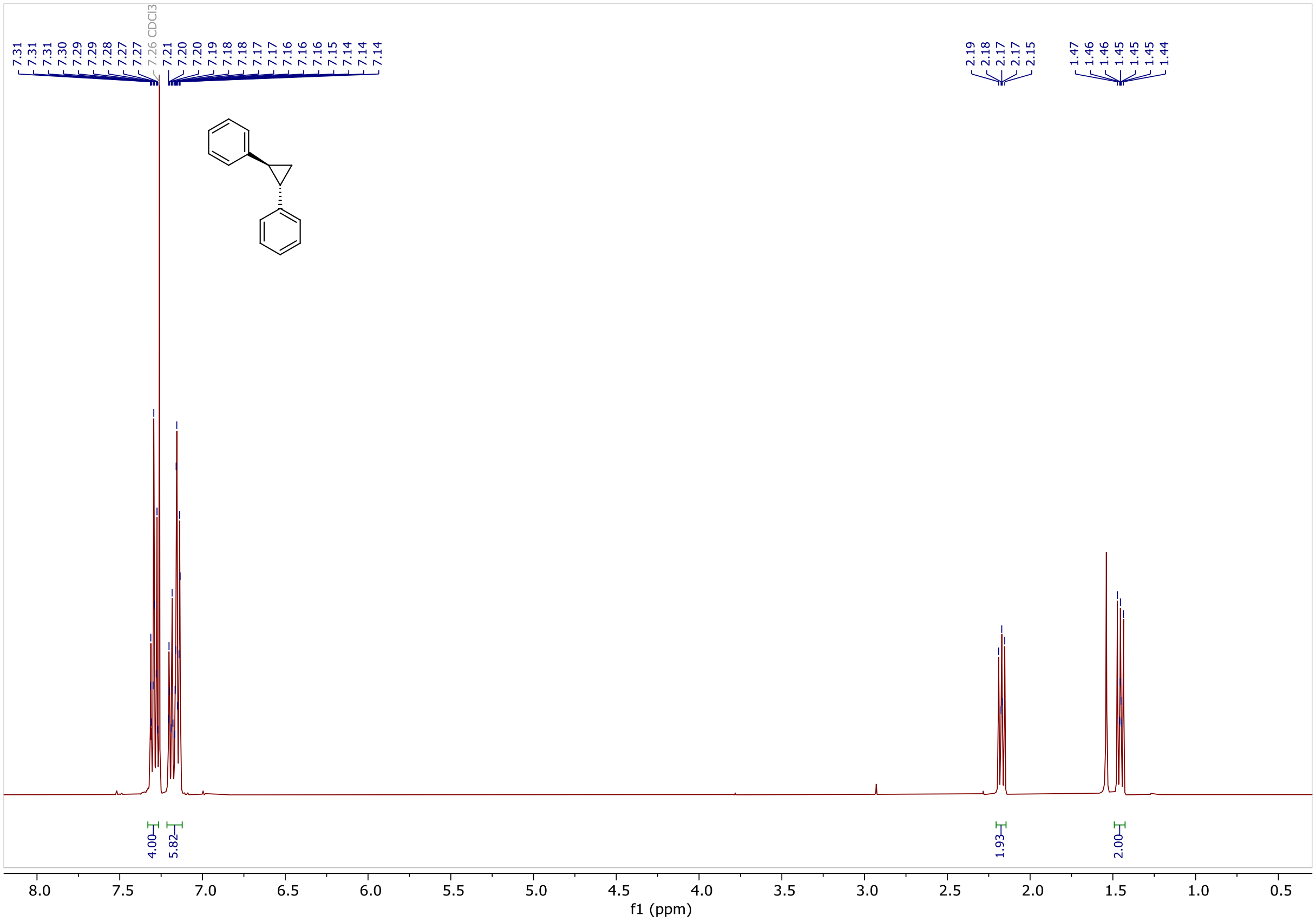

^{13}C NMR Spectrum of 5s (101 MHz, $CDCl_3$)

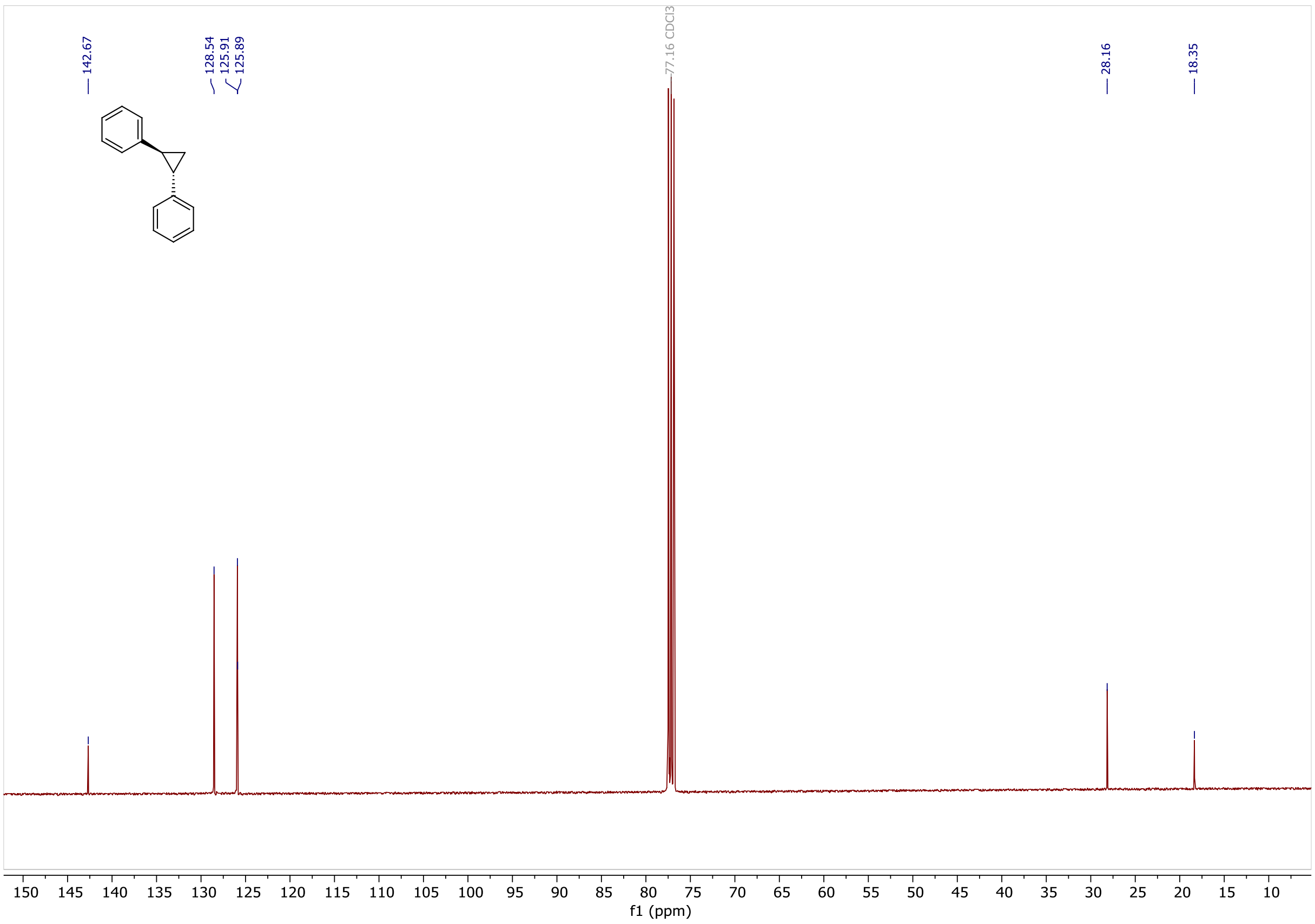

^{1}H NMR Spectrum of 5t (400 MHz, $CDCl_3$)

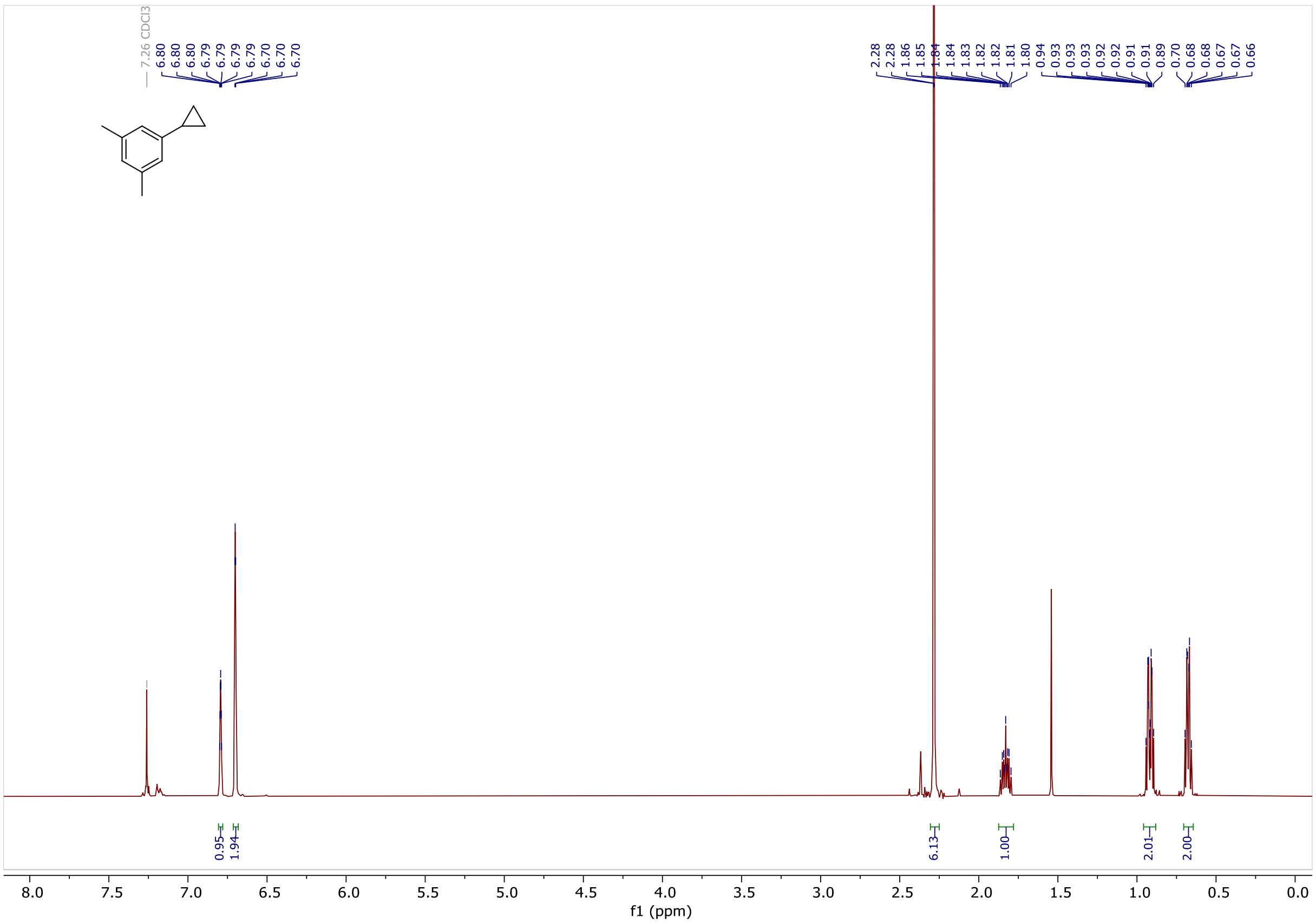

^{13}C NMR Spectrum of 5t (101 MHz, $CDCl_3$)

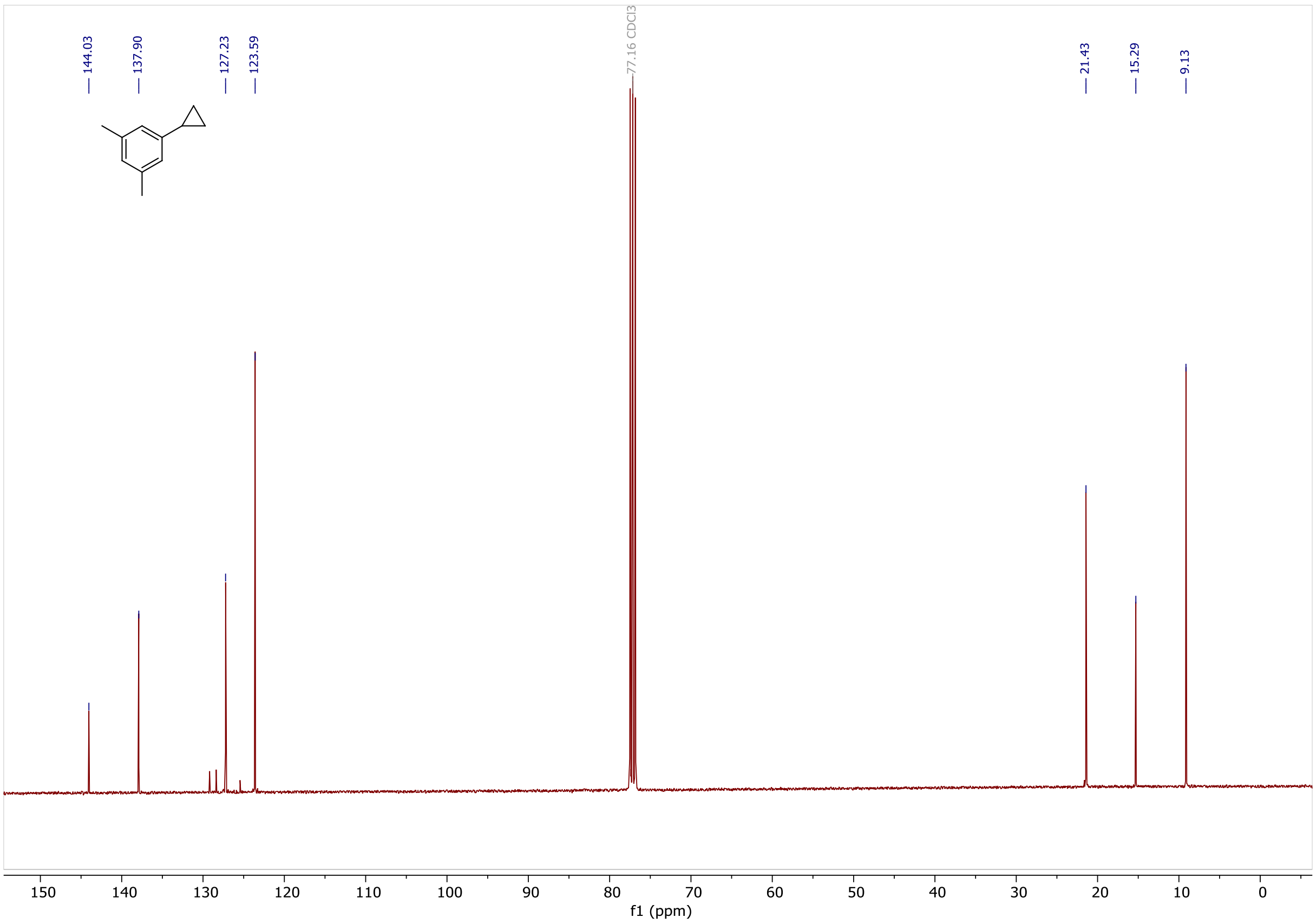

^{1}H NMR Spectrum of 5u (400 MHz, $CDCl_3$)

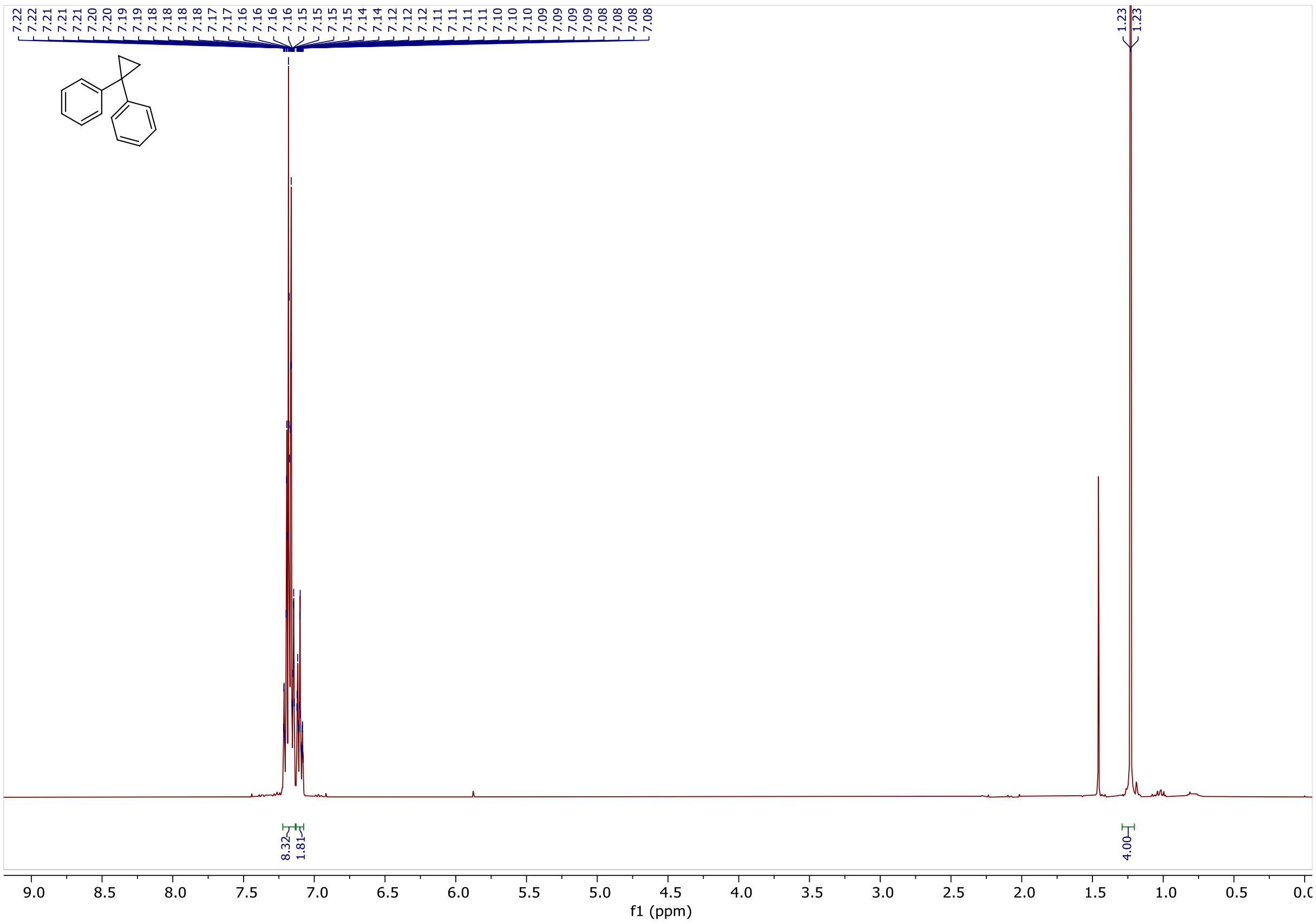

^{13}C NMR Spectrum of 5u (101 MHz, $CDCl_3$)

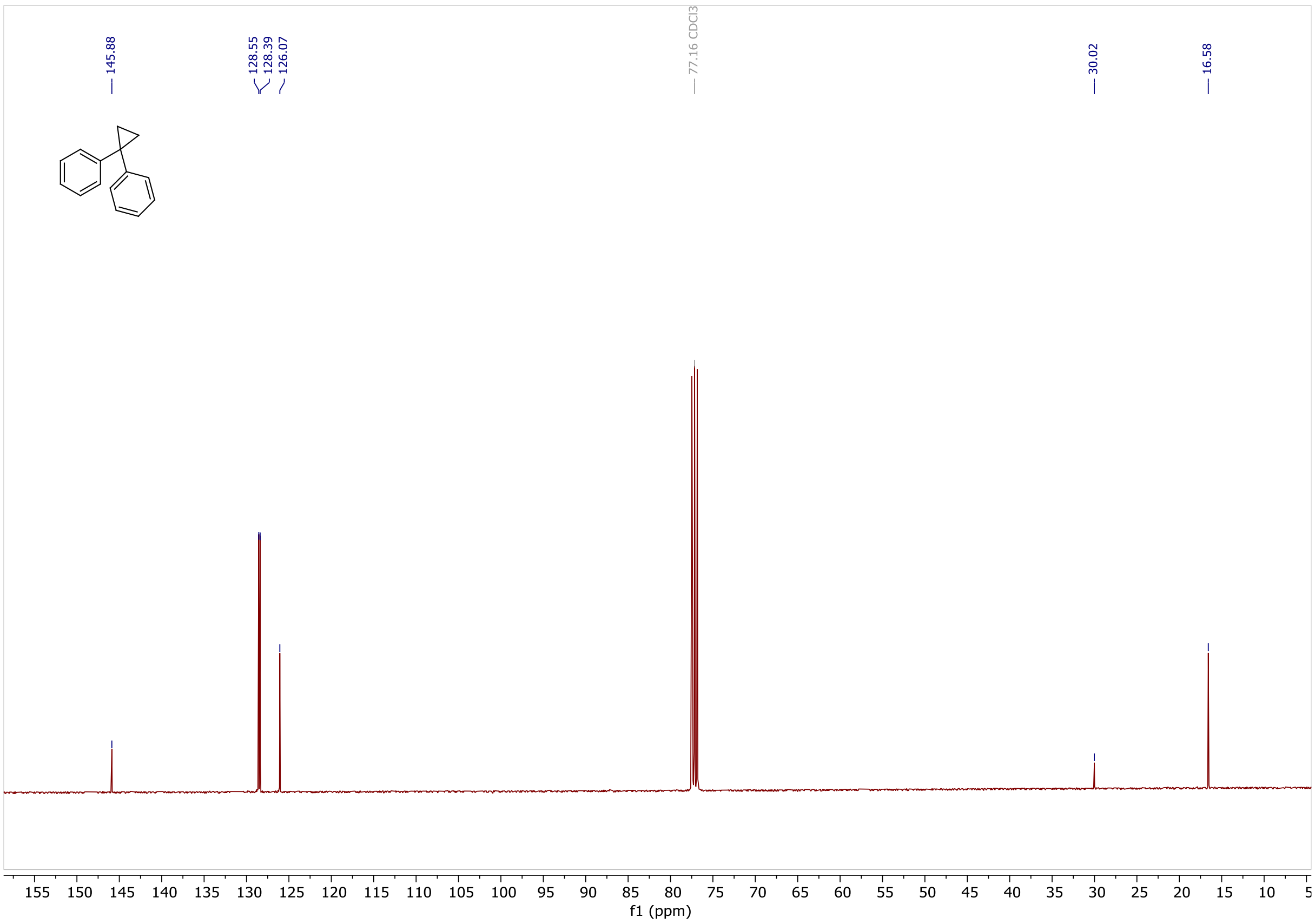

^{1}H NMR Spectrum of 5v (400 MHz, $CDCl_3$)

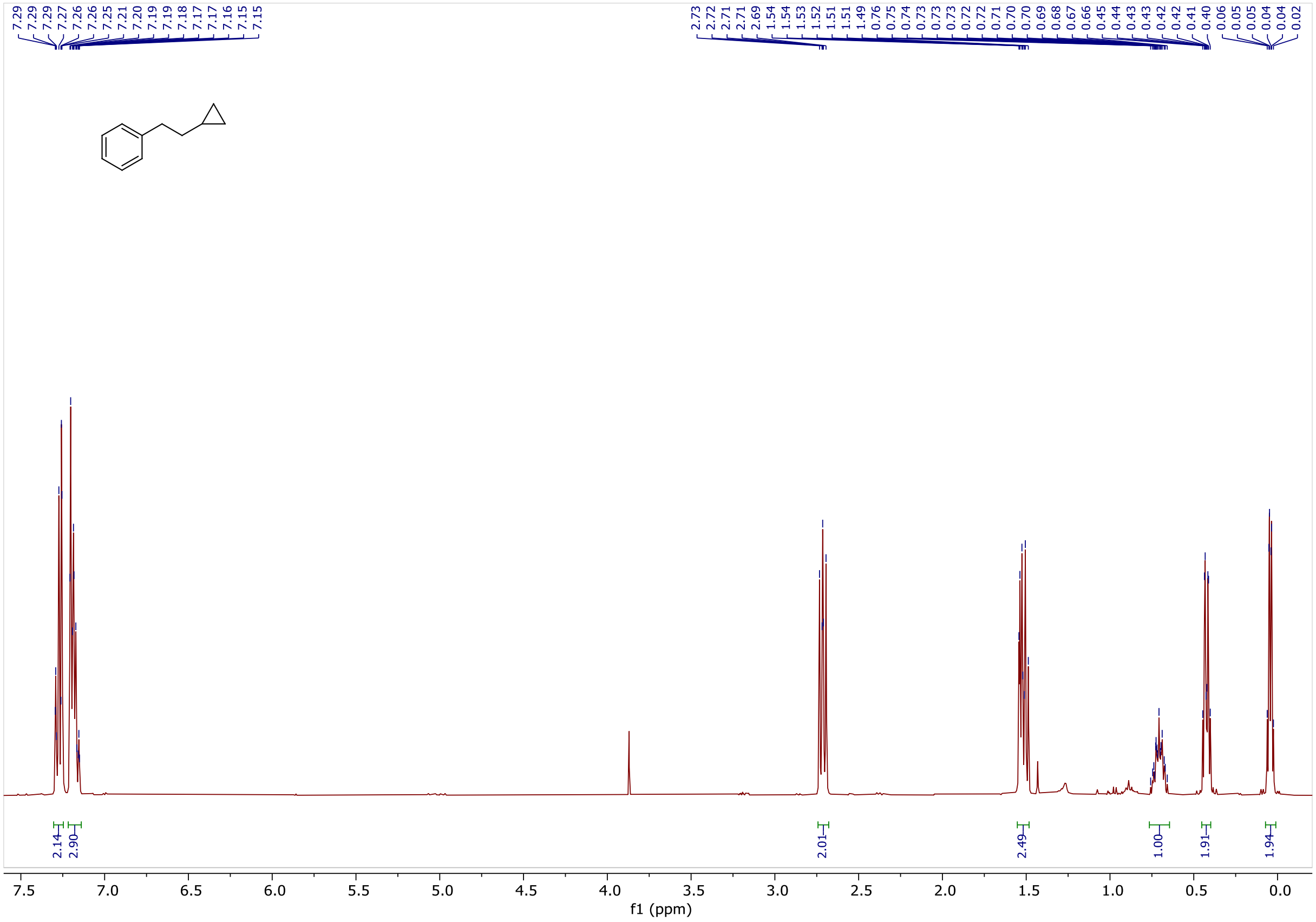

^{13}C NMR Spectrum of 5v (101 MHz, $CDCl_3$)

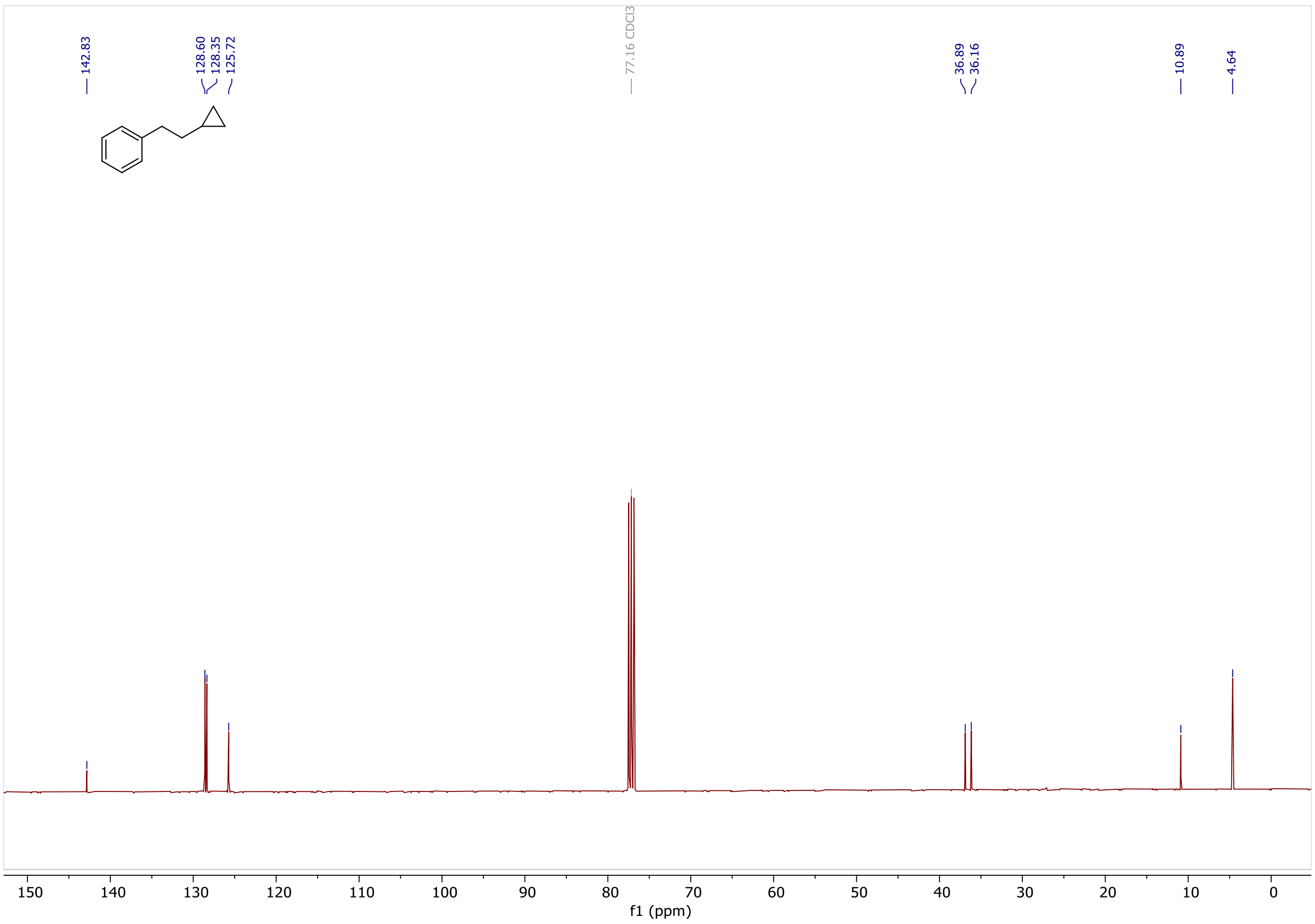

1,3-aminochlorinated products **6**

^{1}H NMR Spectrum of 6a (400 MHz, $CDCl_3$)

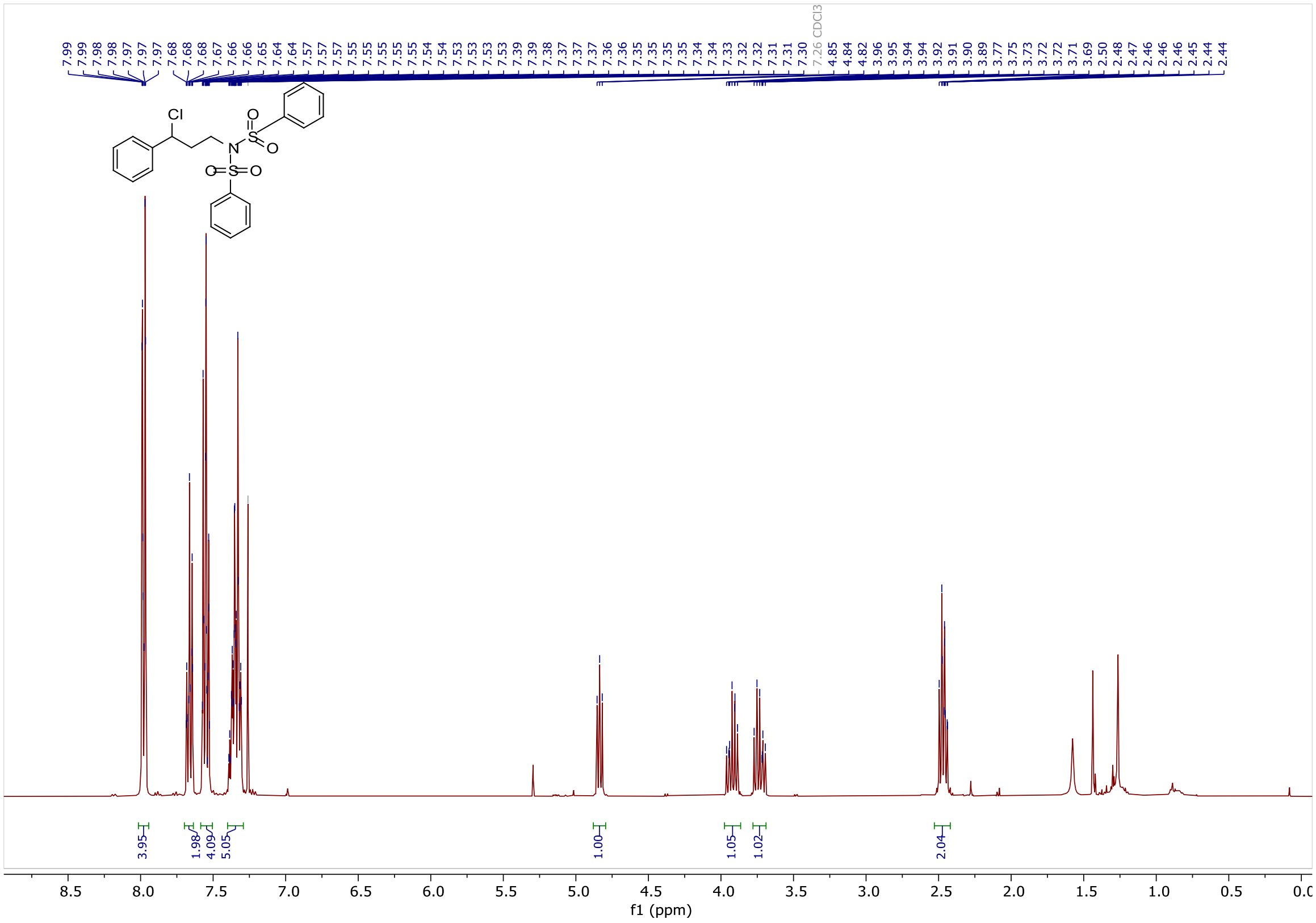

^{13}C NMR Spectrum of 6a (101 MHz, $CDCl_3$)

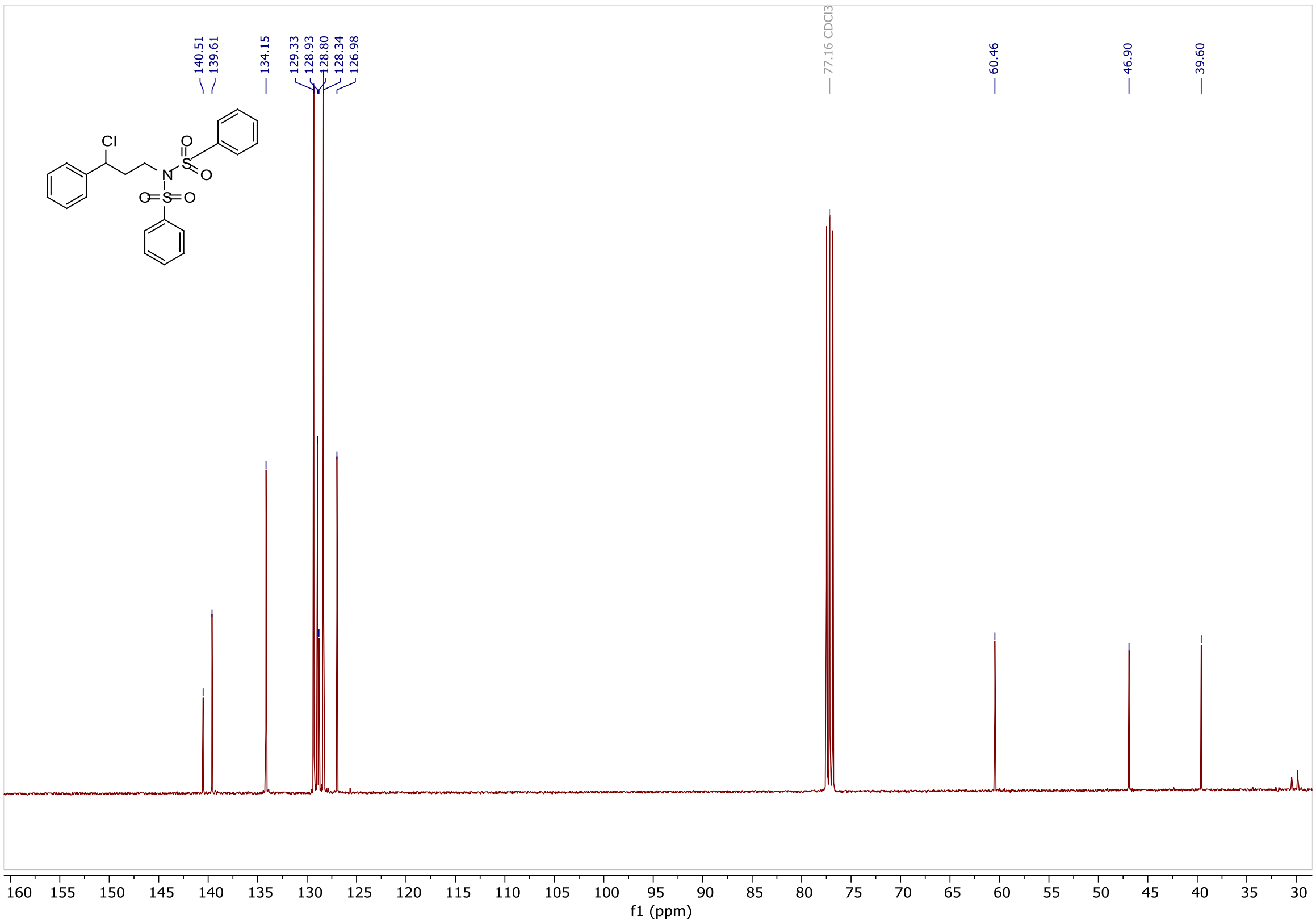

^{1}H NMR Spectrum of *o*-6b (400 MHz, $CDCl_3$)

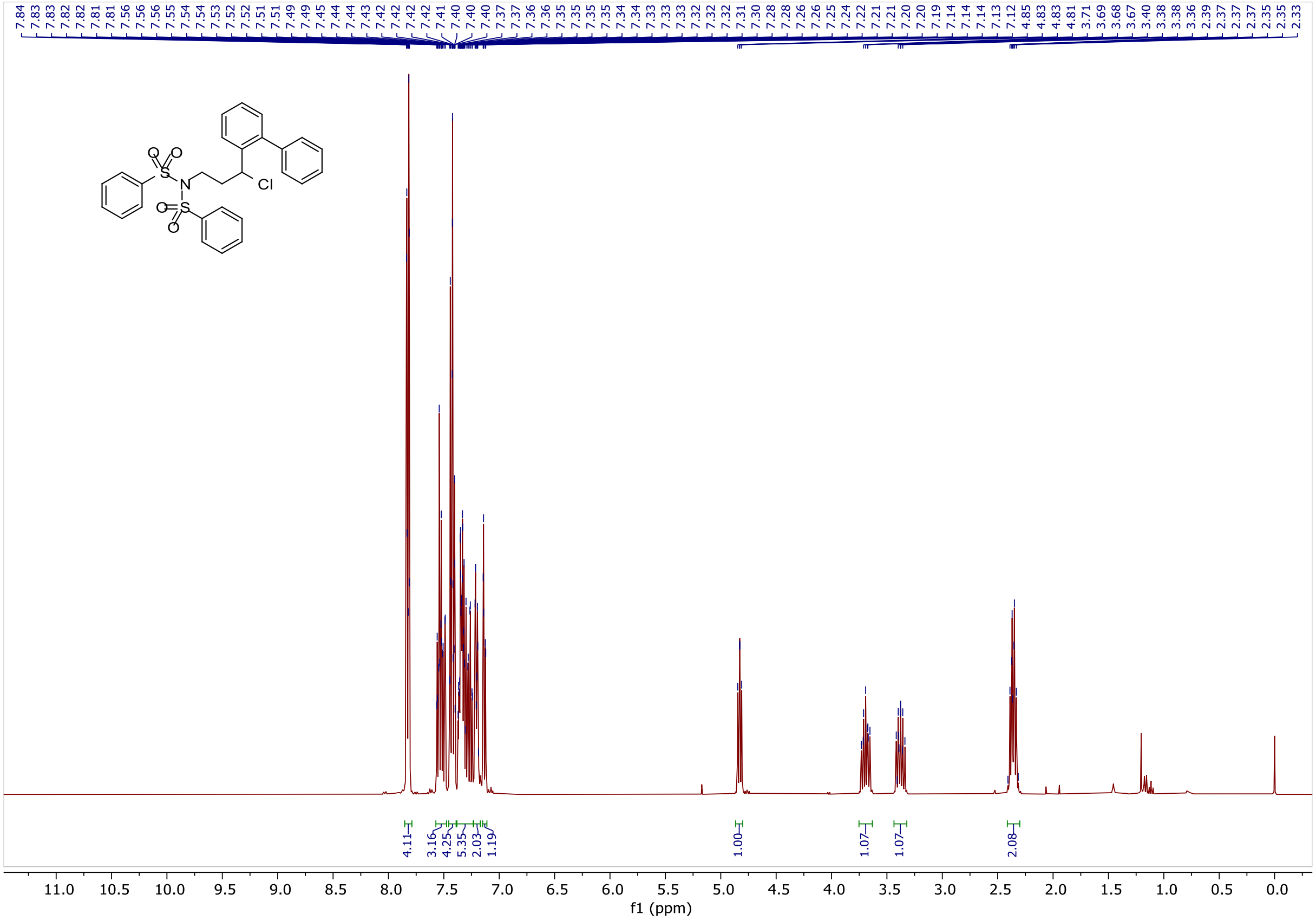

^{13}C NMR Spectrum of *o*-6b (101 MHz, $CDCl_3$)

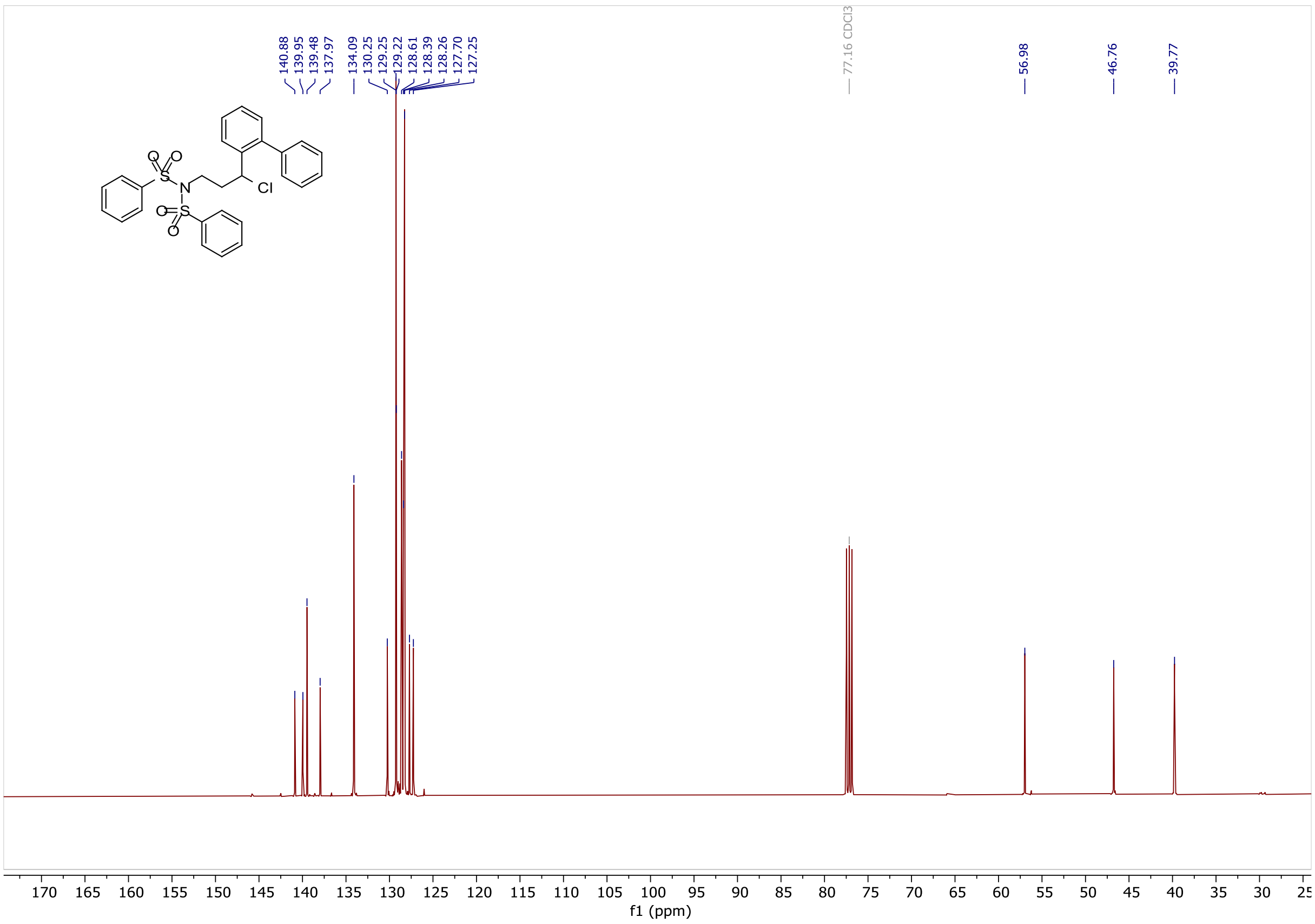

^{1}H NMR Spectrum of *m*-6b (400 MHz, $CDCl_3$)

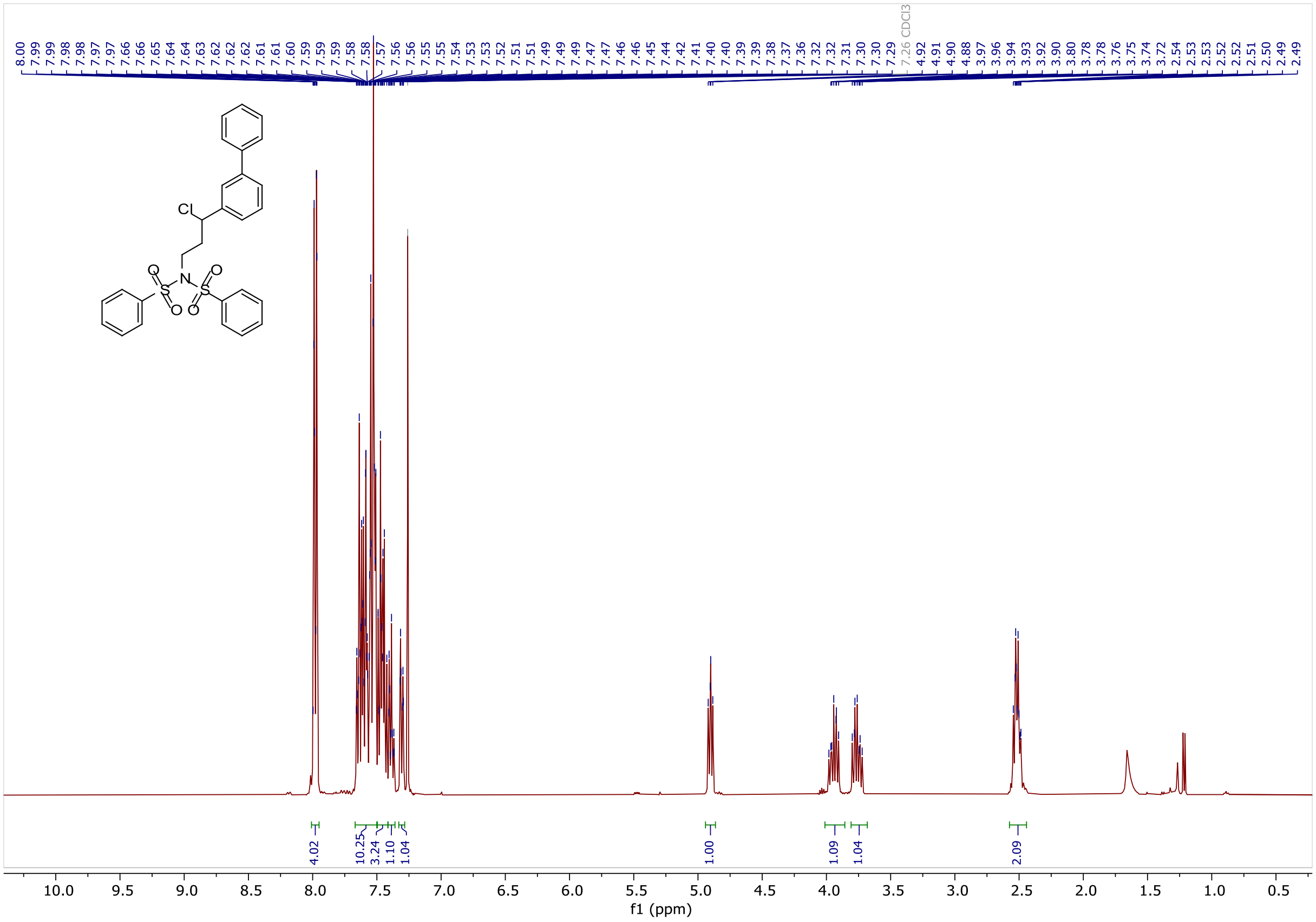

^{13}C NMR Spectrum of *m*-6b (101 MHz, $CDCl_3$)

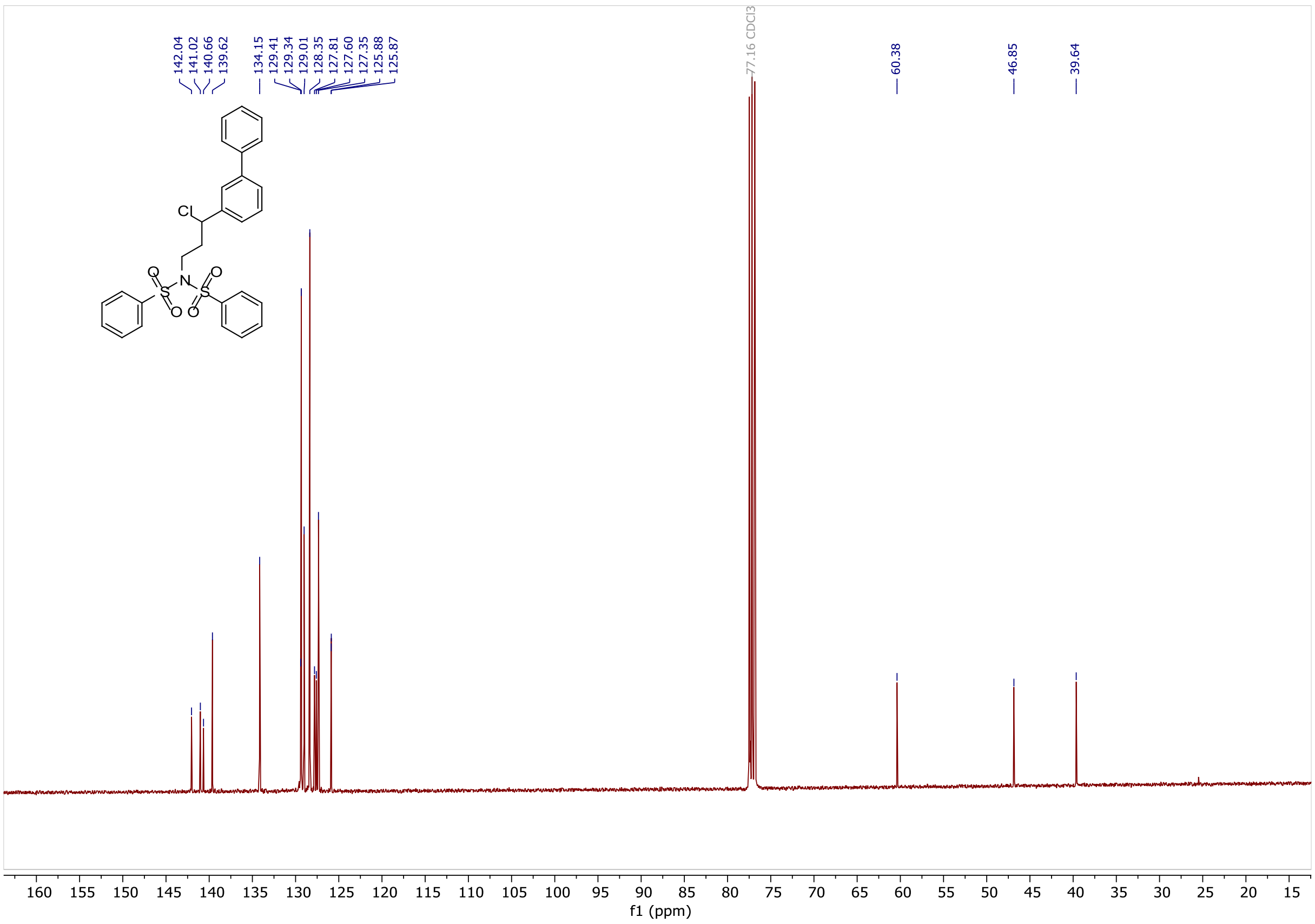

^{1}H NMR Spectrum of *p*-**6b** (400 MHz, CDCl$_3$)

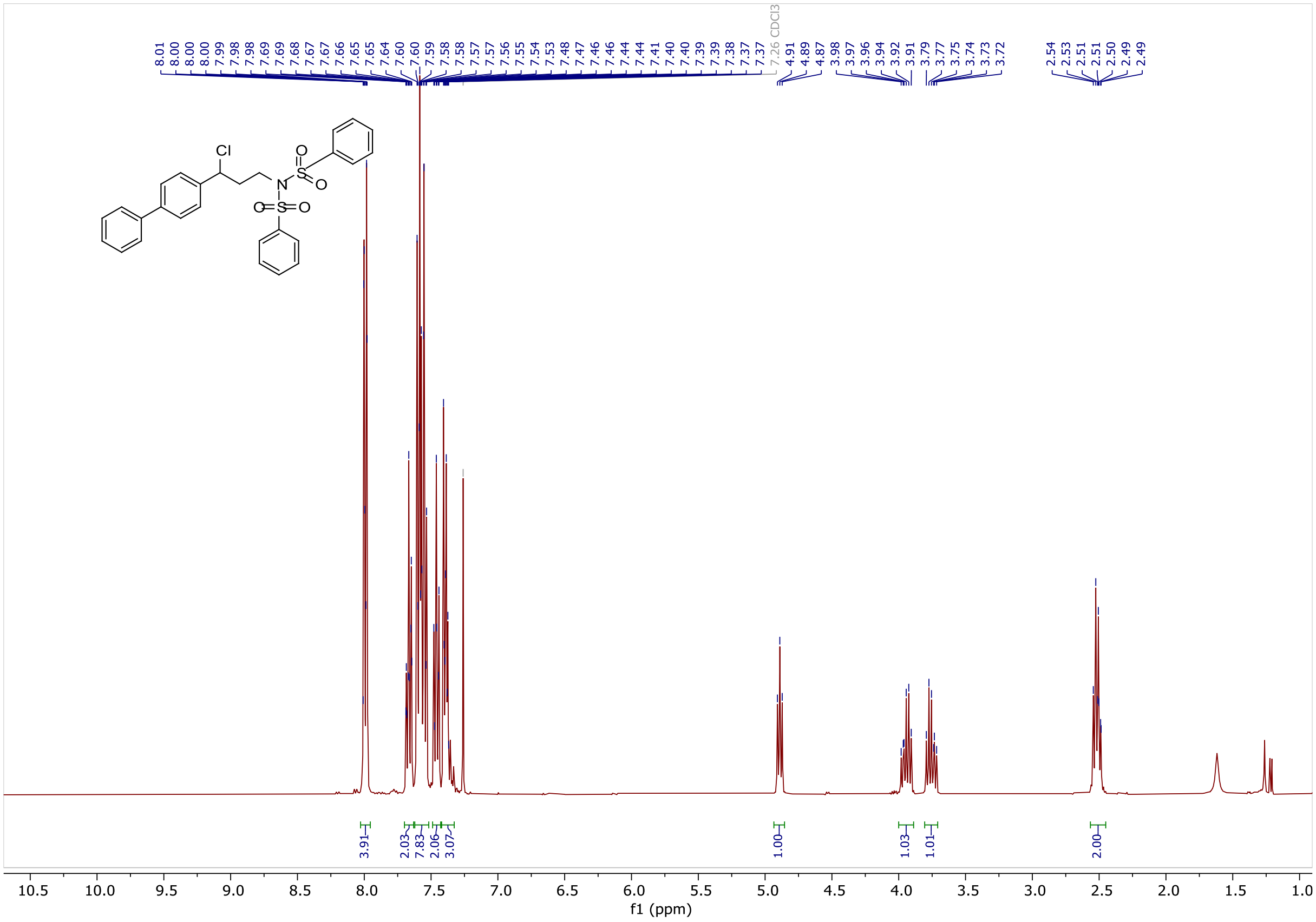

^{13}C NMR Spectrum of *p*-**6b** (101 MHz, CDCl$_3$)

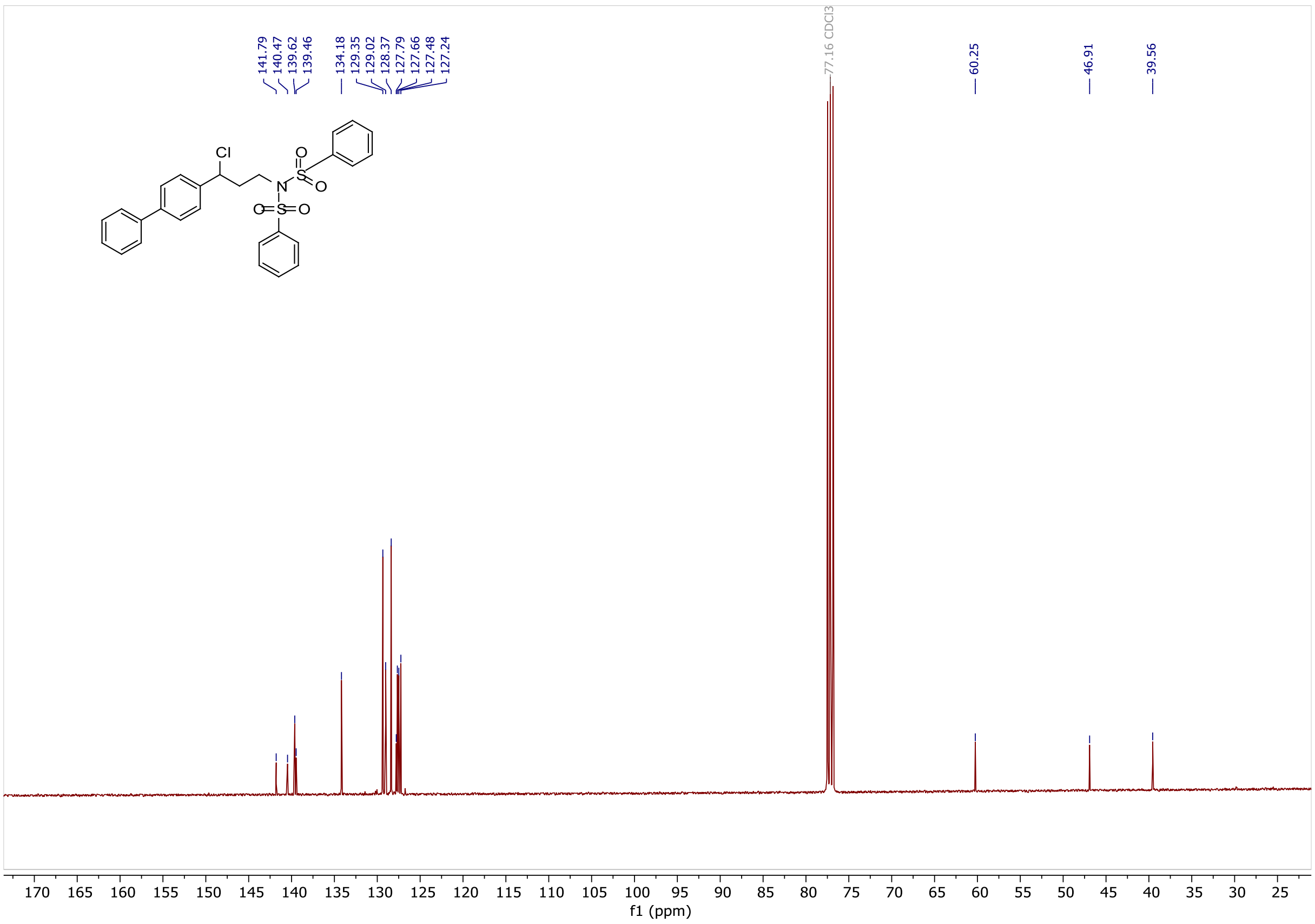

^{1}H NMR Spectrum of *o*-6c (400 MHz, $CDCl_3$)

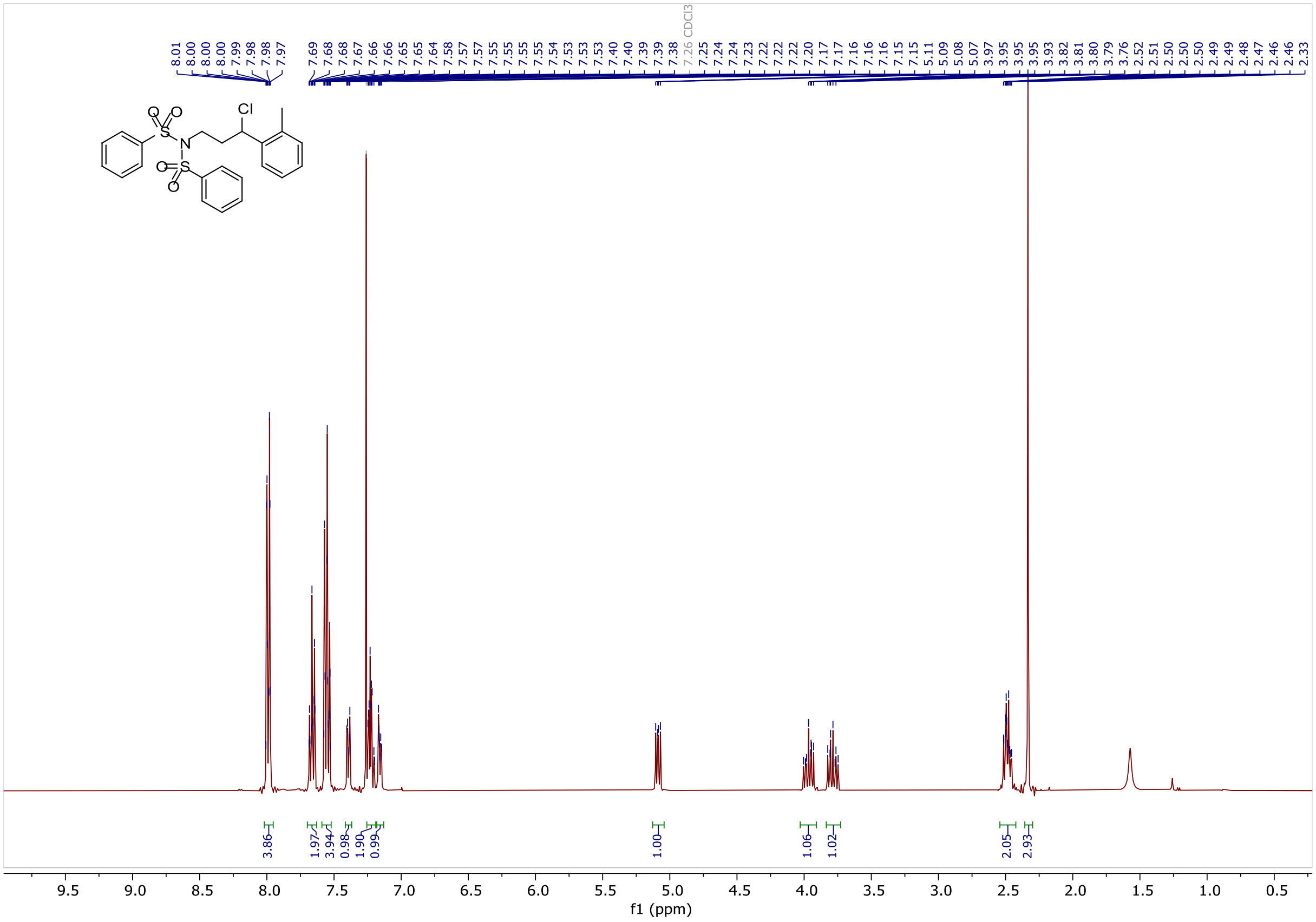

^{13}C NMR Spectrum of *o*-6c (101 MHz, $CDCl_3$)

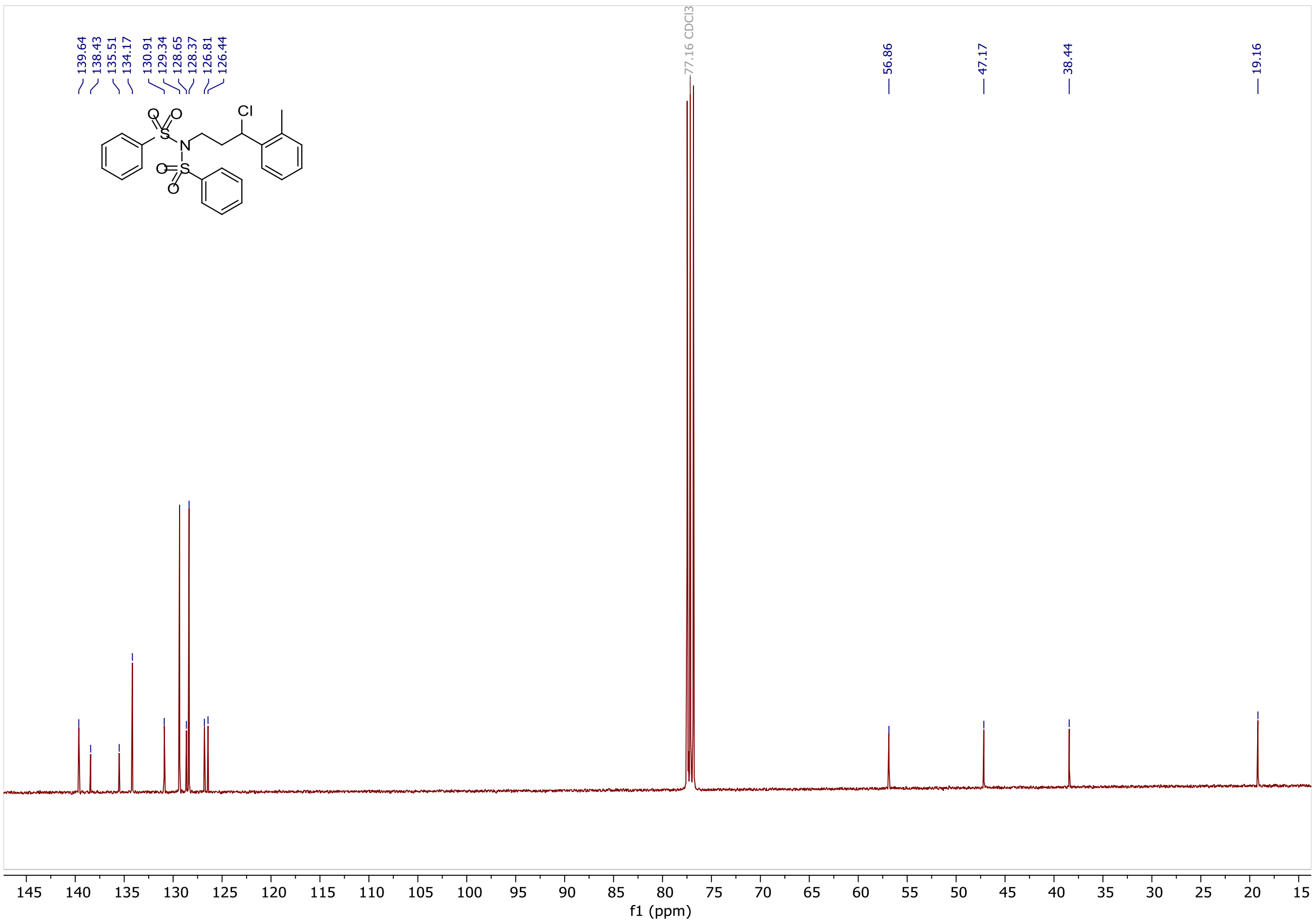

^{1}H NMR Spectrum of *p*-6c (400 MHz, $CDCl_3$)

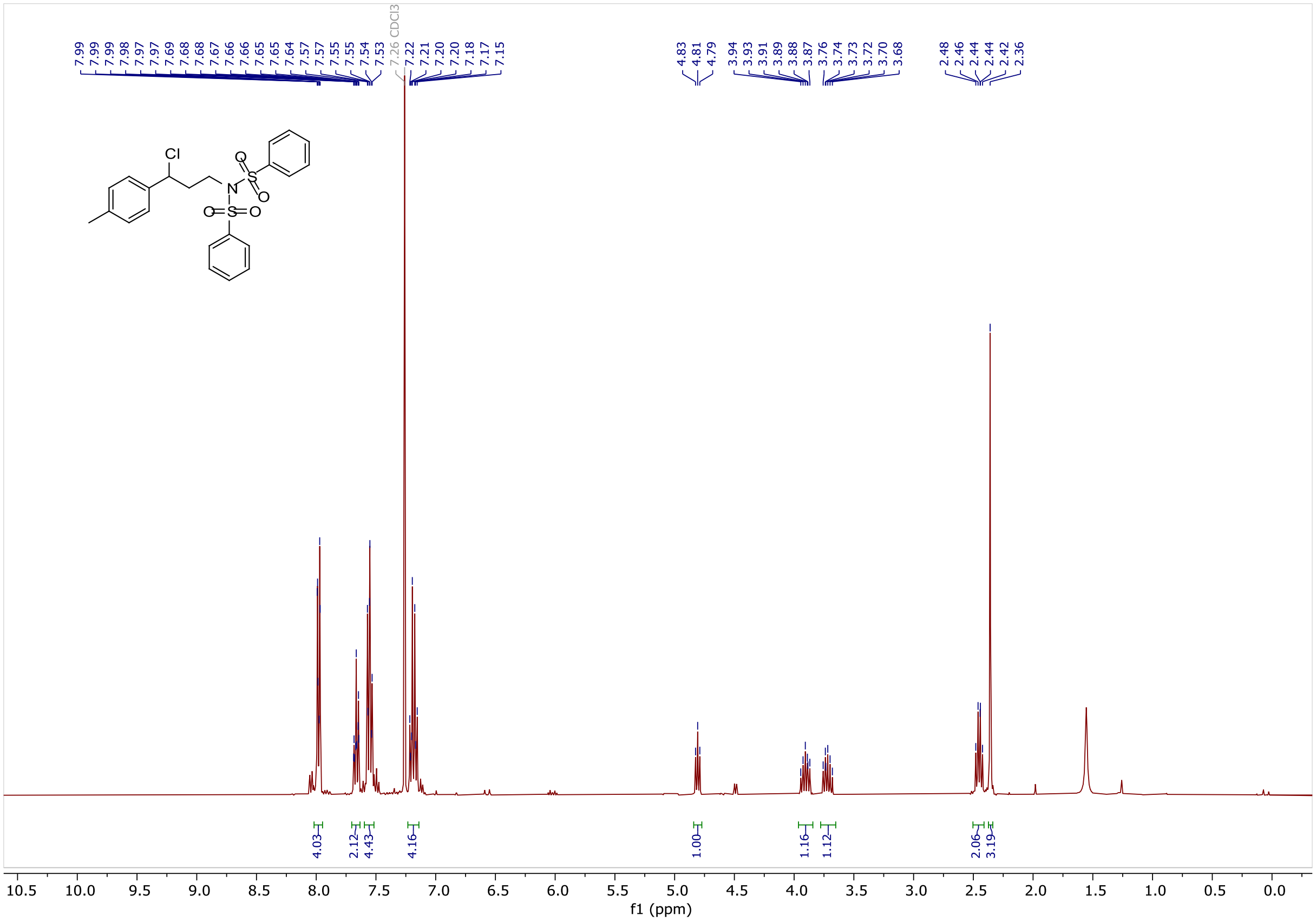

^{13}C NMR Spectrum of *p*-6c (101 MHz, $CDCl_3$)

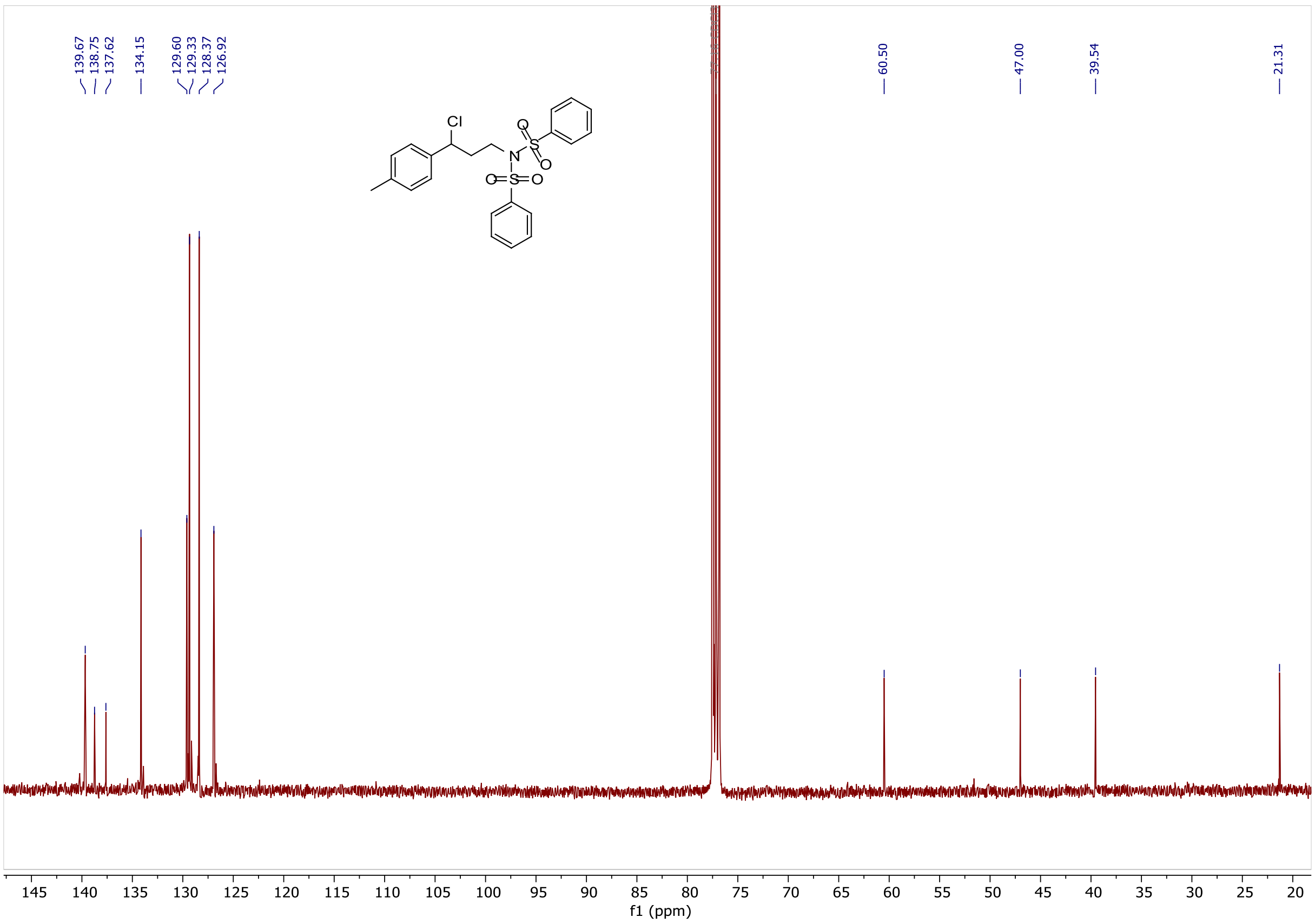

^{1}H NMR Spectrum of *o*-6d (400 MHz, $CDCl_3$)

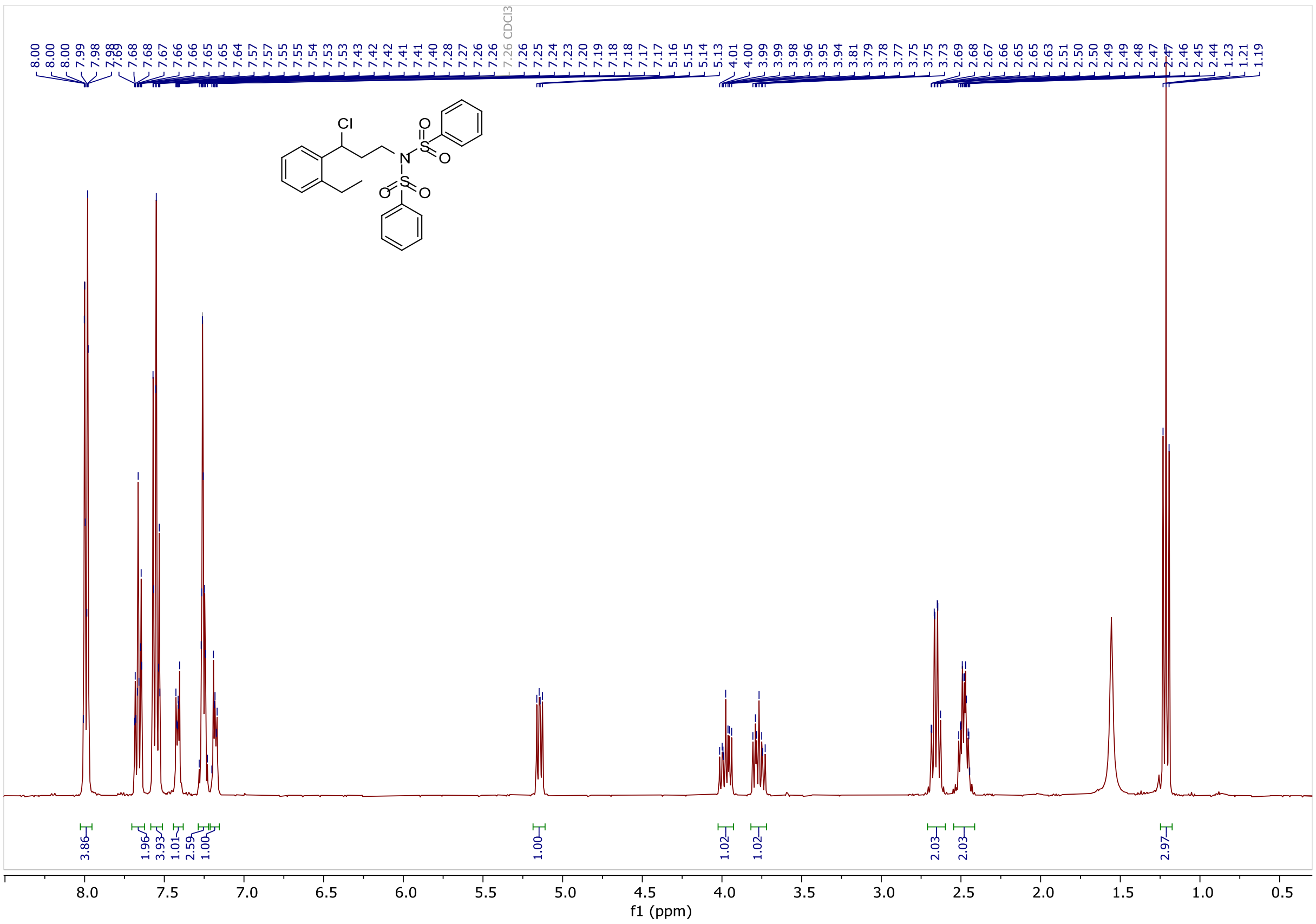

^{13}C NMR Spectrum of *o*-6d (101 MHz, $CDCl_3$)

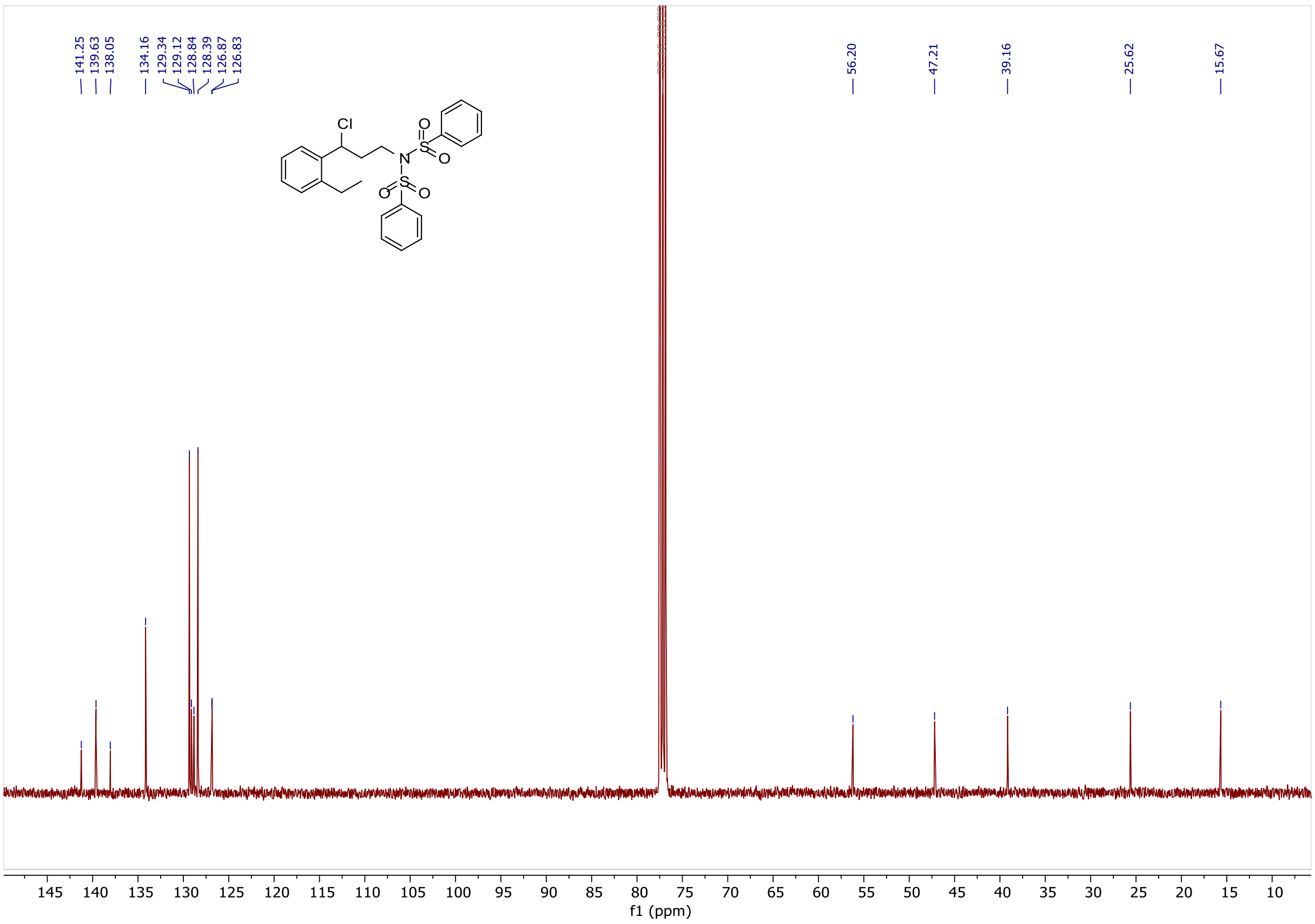

^{1}H NMR Spectrum of *m*-6d (400 MHz, $CDCl_3$)

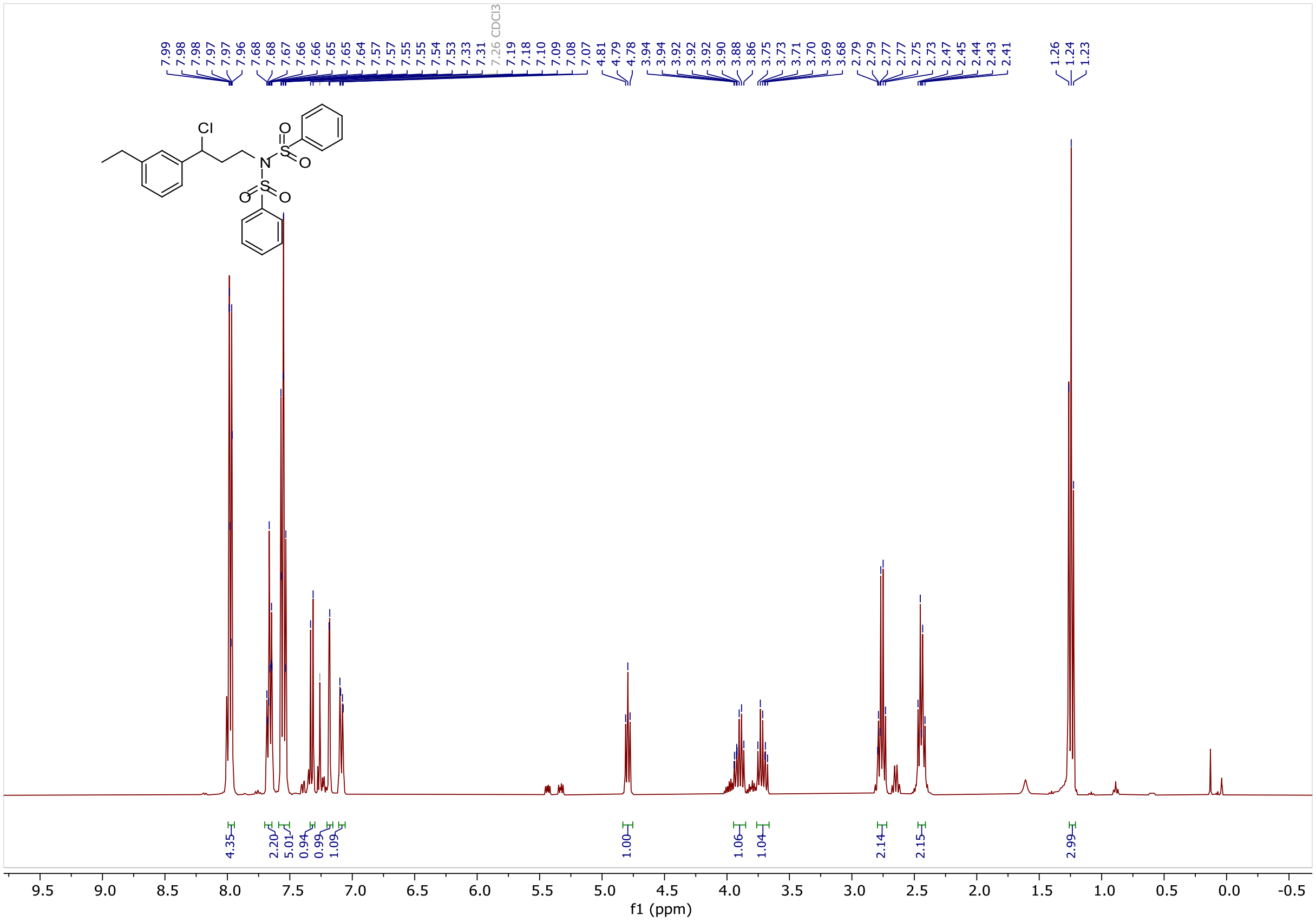

^{13}C NMR Spectrum of *m*-6d (101 MHz, $CDCl_3$)

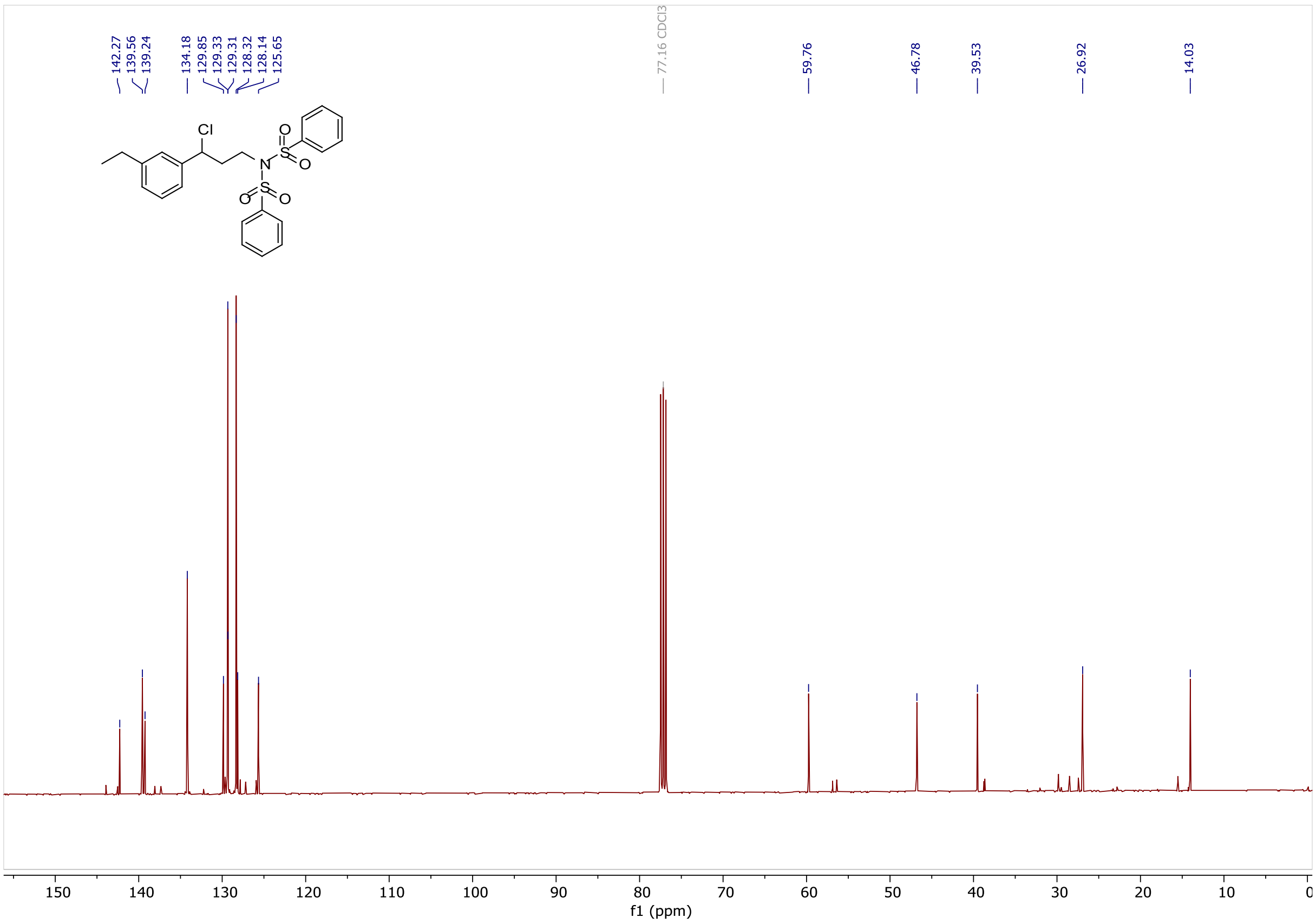

^{1}H NMR Spectrum of *p*-6d (400 MHz, $CDCl_3$)

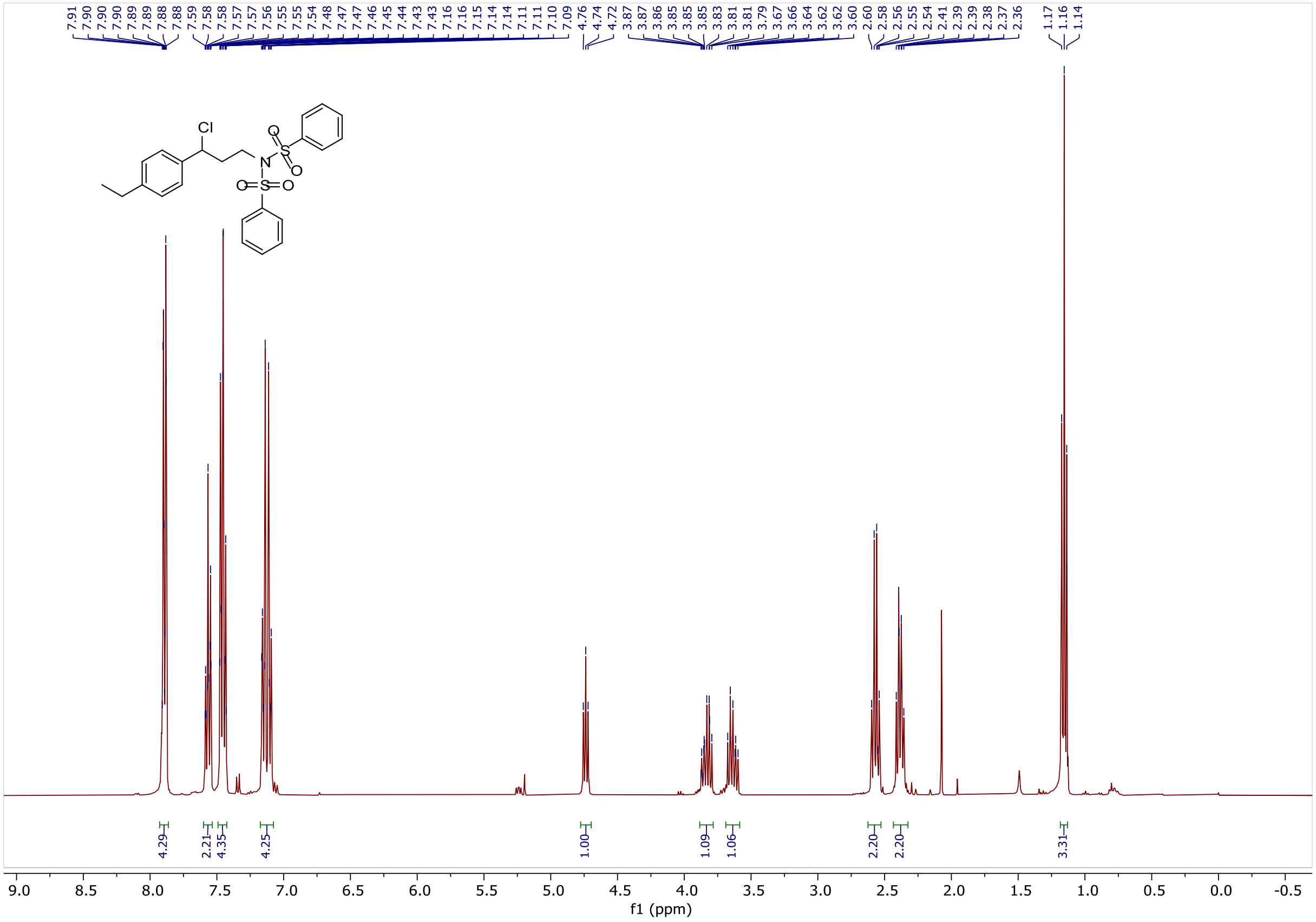

^{13}C NMR Spectrum of *p*-6d (101 MHz, $CDCl_3$)

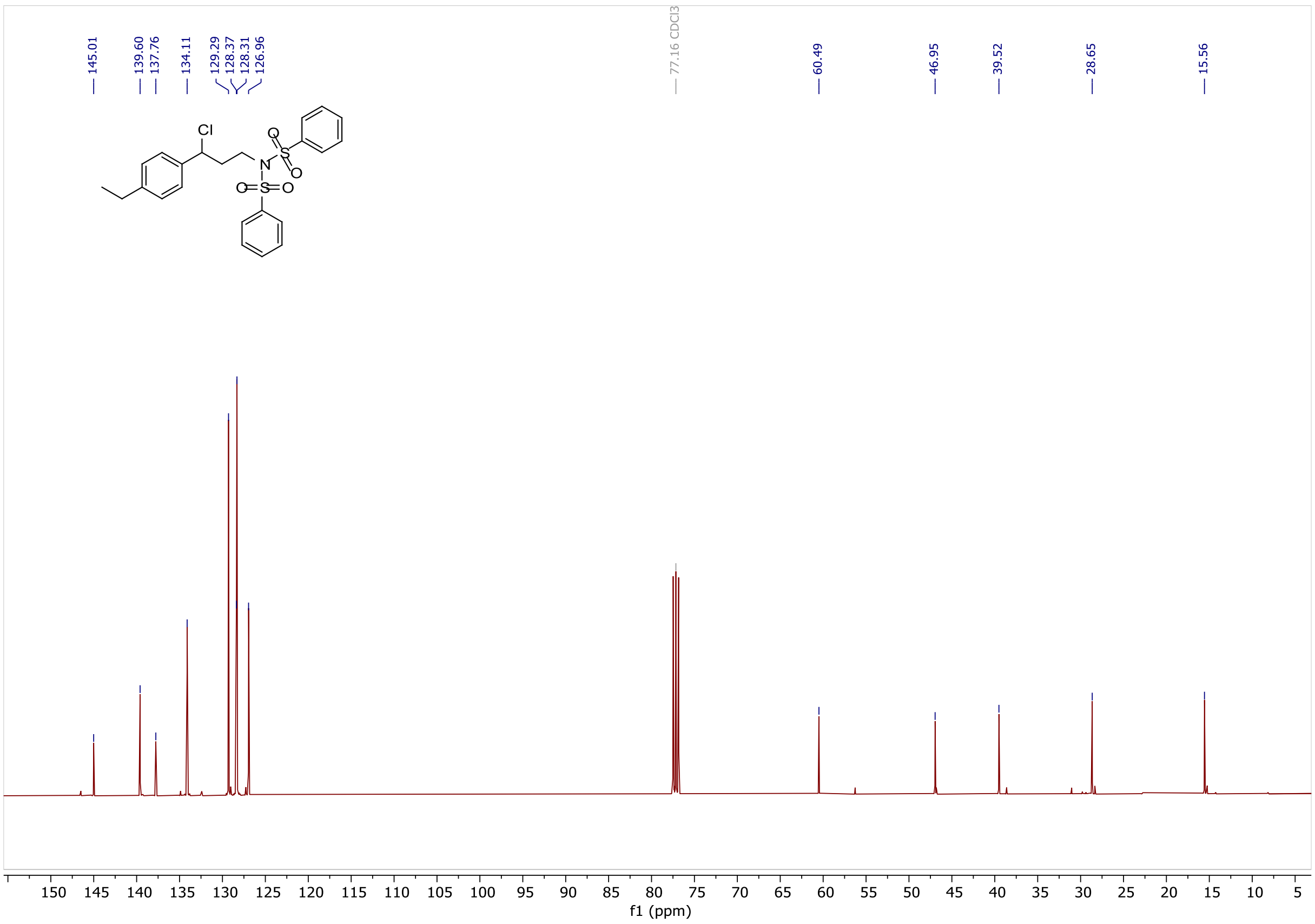

^{1}H NMR Spectrum of 6e (400 MHz, $CDCl_3$)

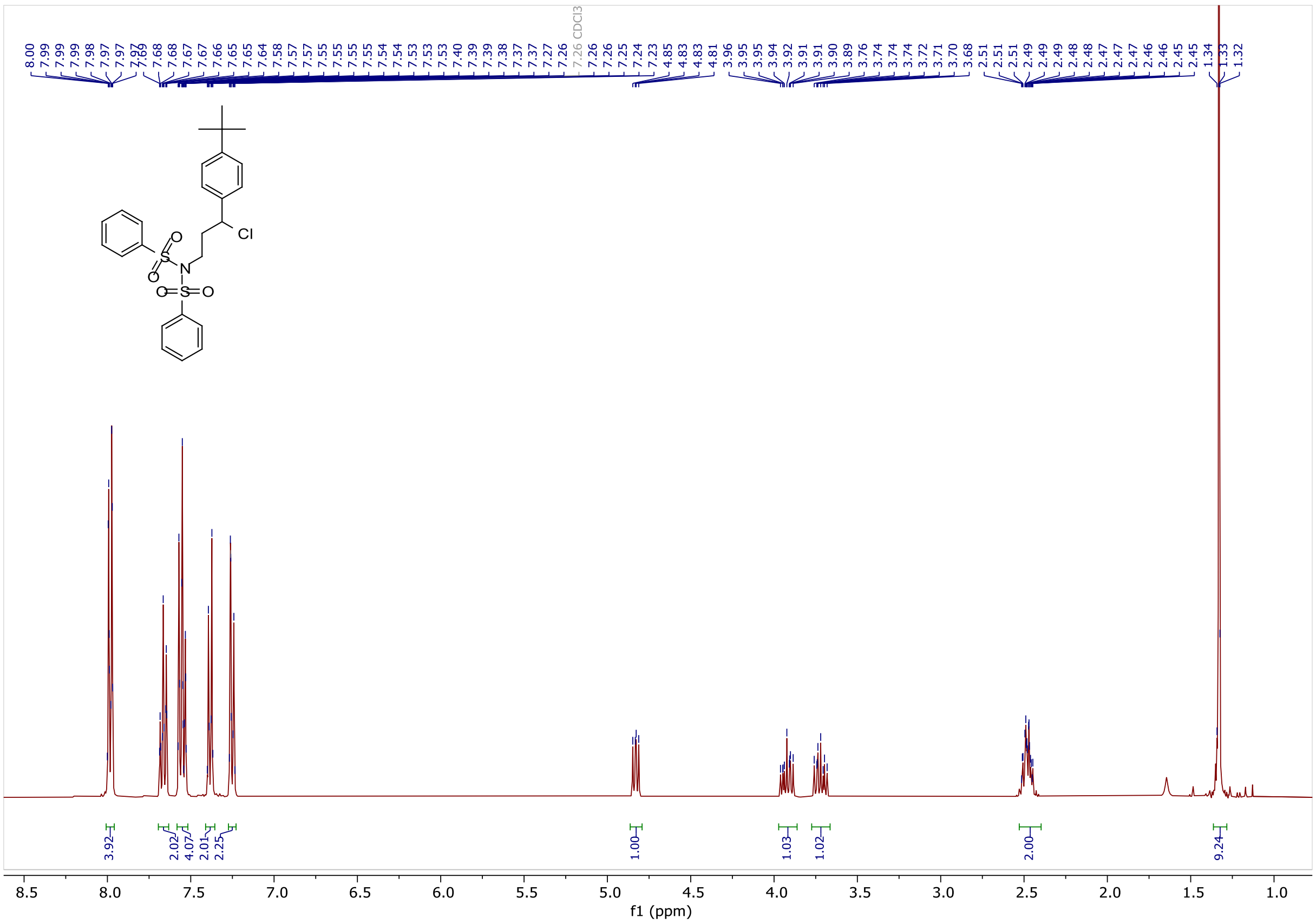

^{13}C NMR Spectrum of 6e (101 MHz, $CDCl_3$)

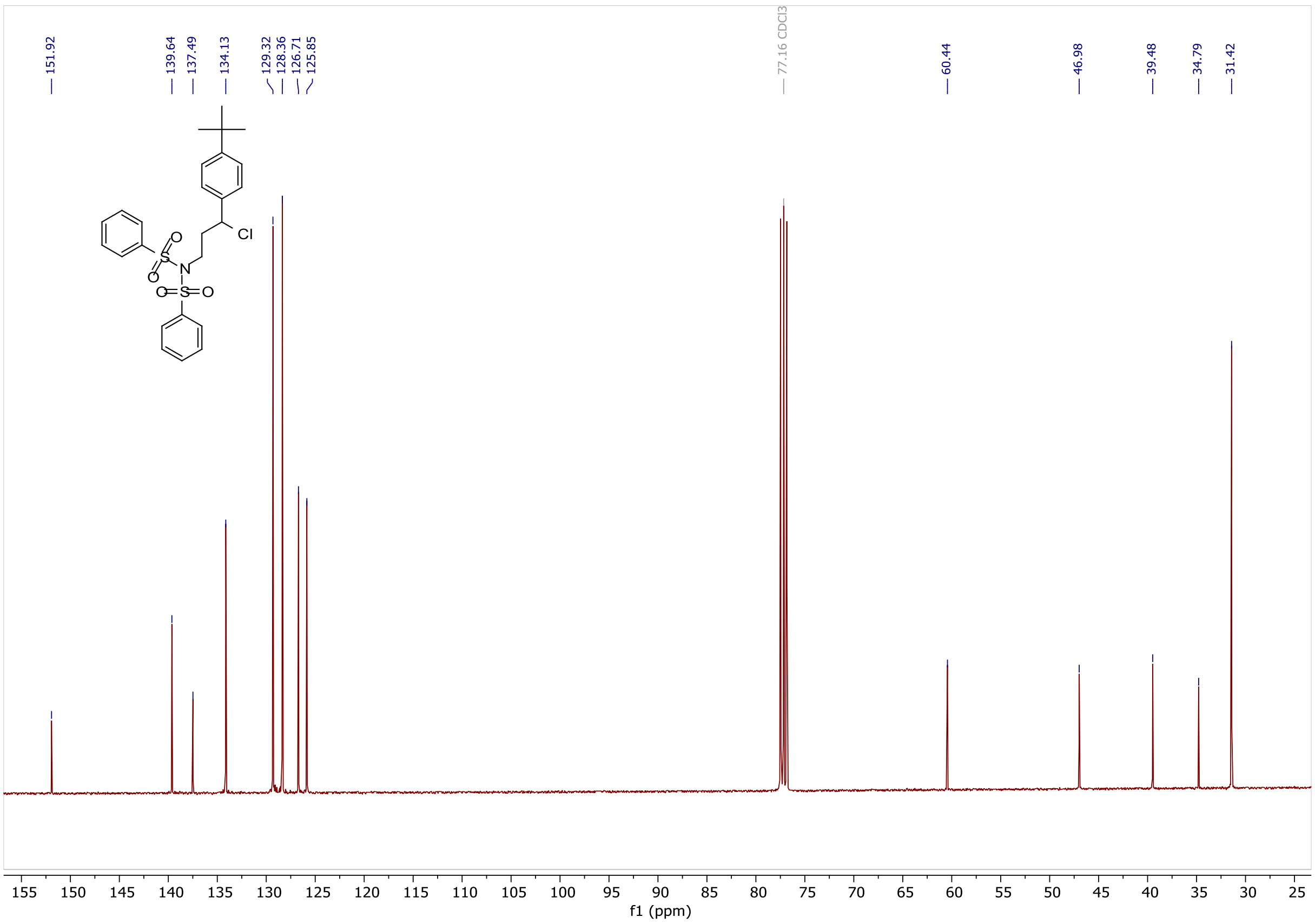

^{1}H NMR Spectrum of 6f (400 MHz, $CDCl_3$)

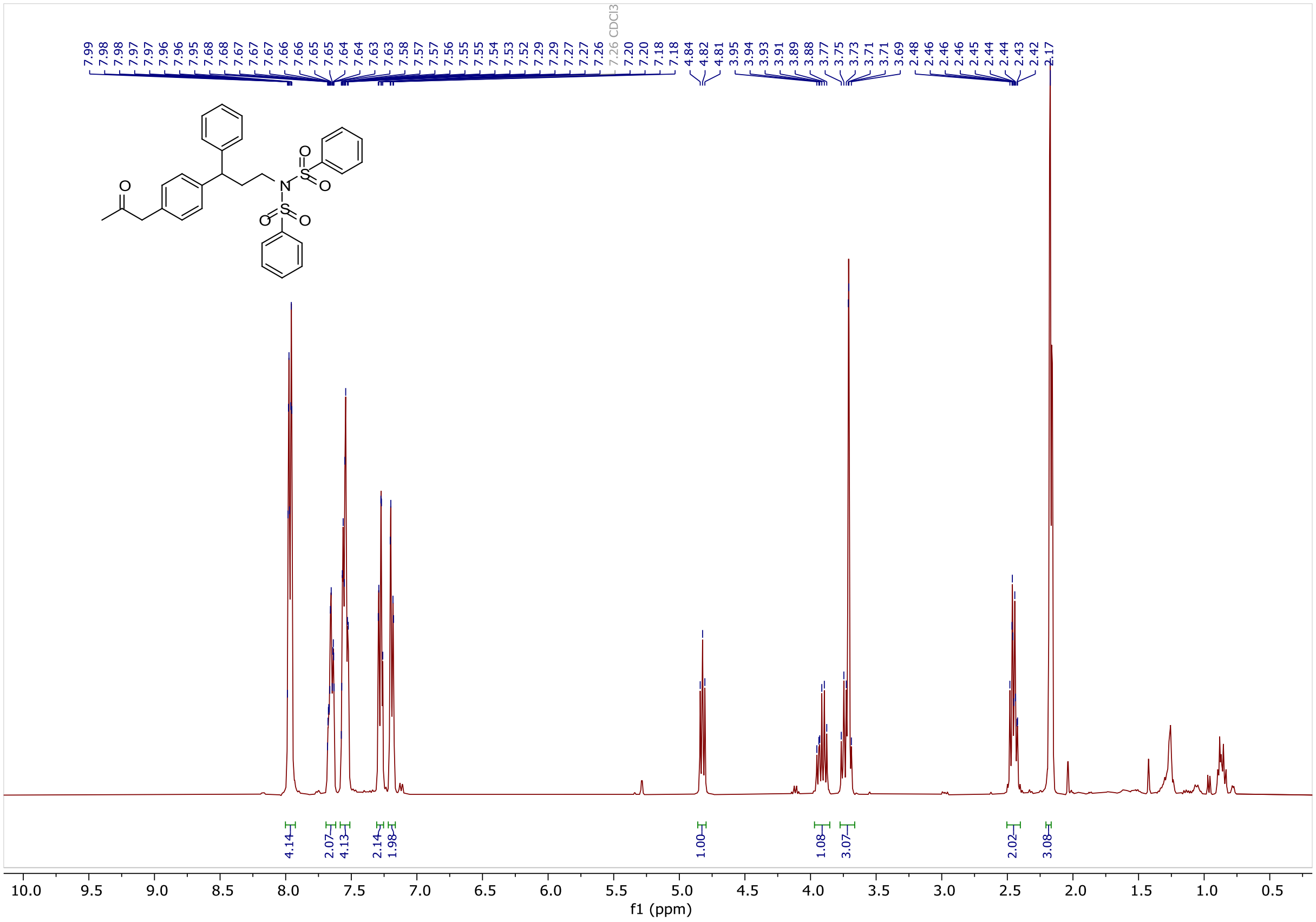

^{13}C NMR Spectrum of f6 (101 MHz, $CDCl_3$)

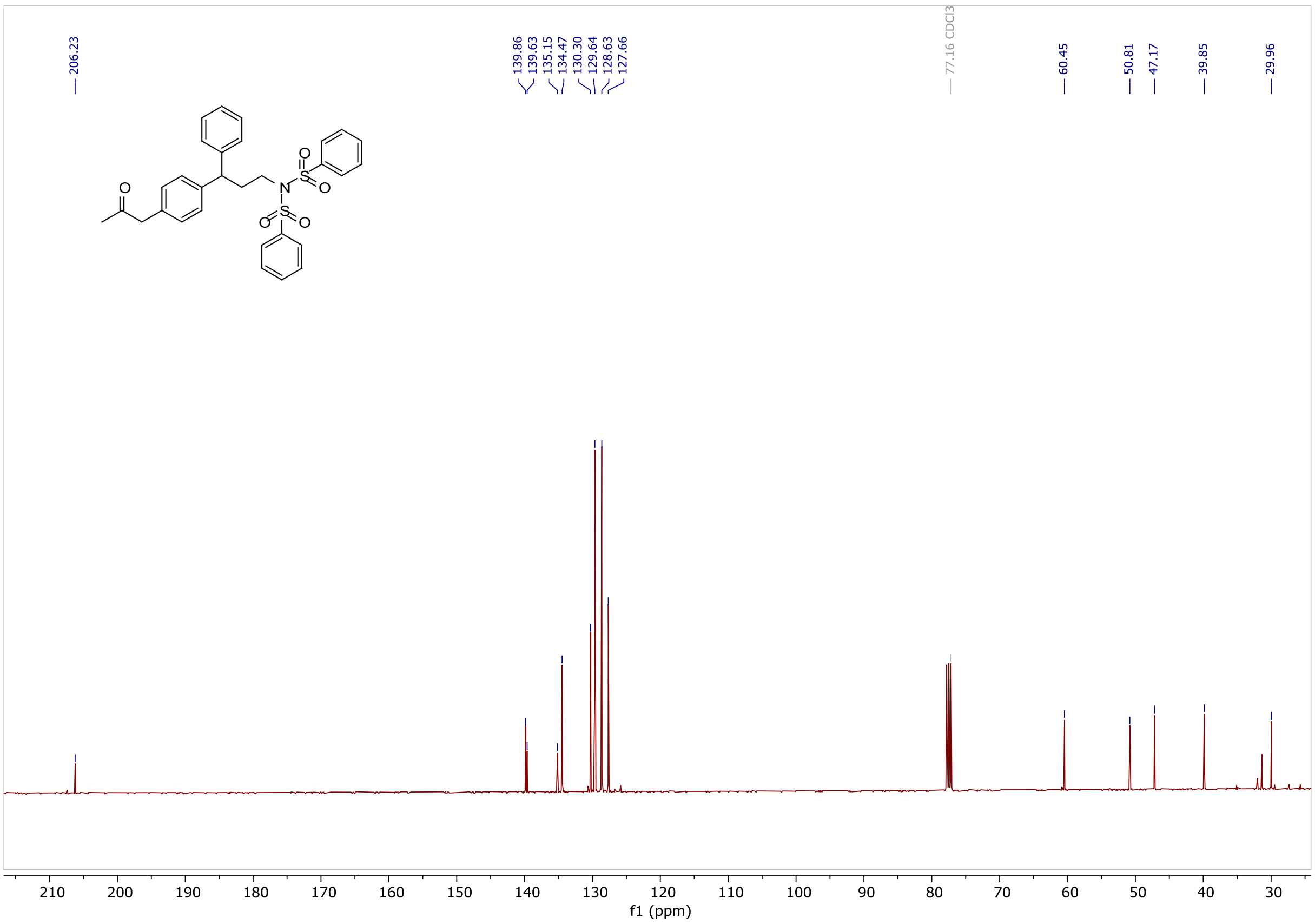

^{1}H NMR Spectrum of *o*-6g (400 MHz, $CDCl_3$)

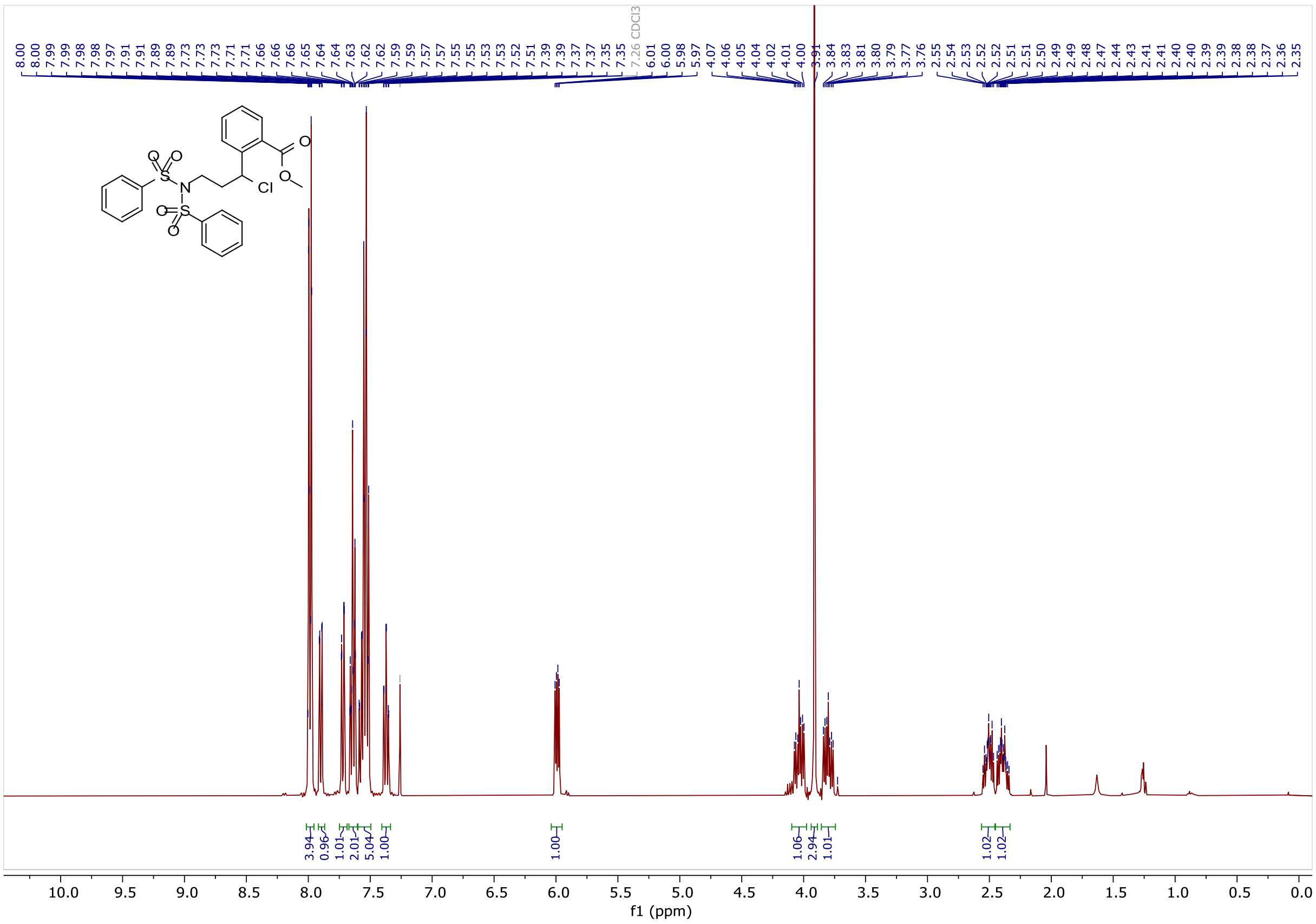

^{13}C NMR Spectrum of *o*-6g (101 MHz, $CDCl_3$)

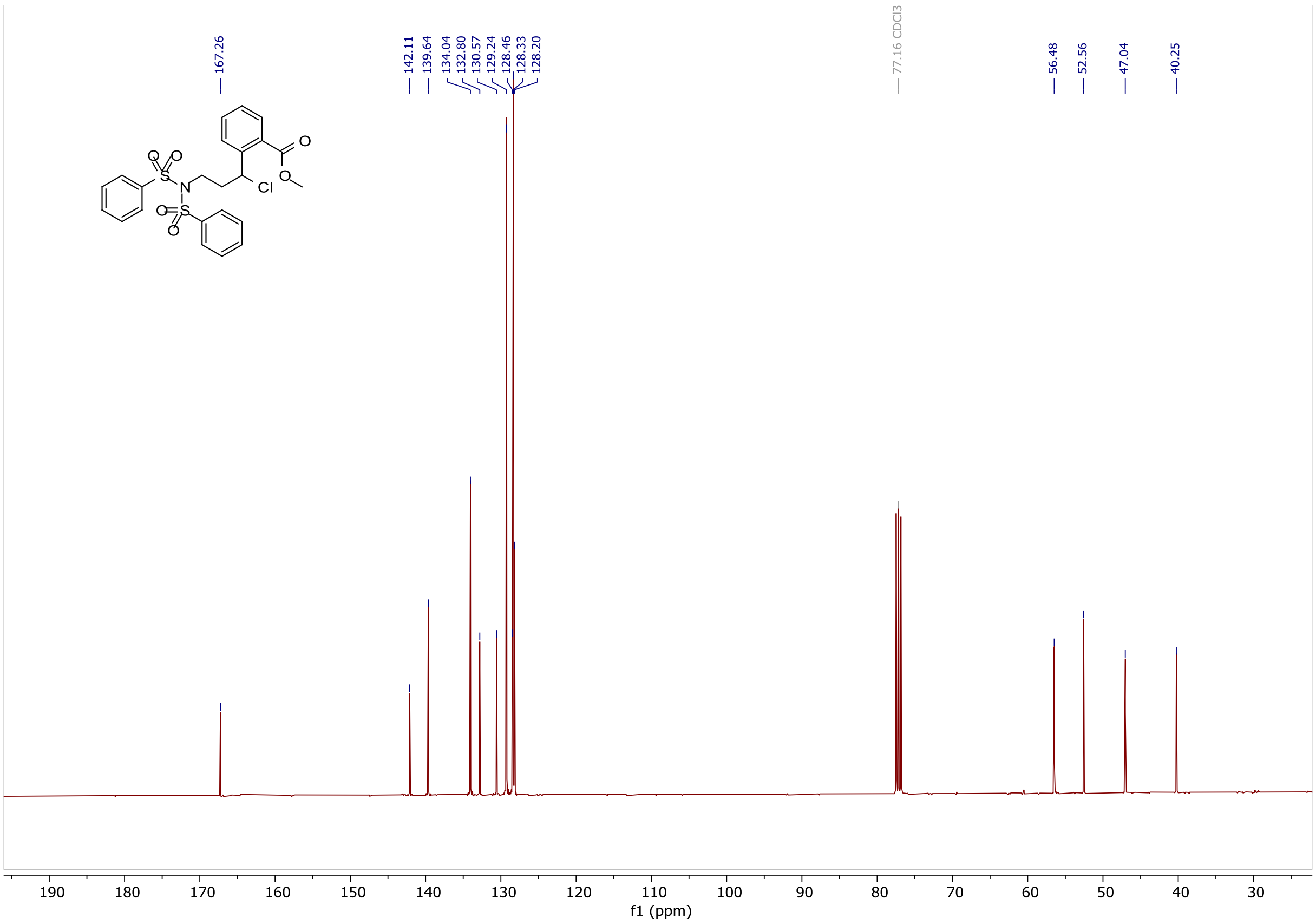

^{1}H NMR Spectrum of *m*-6g (400 MHz, $CDCl_3$)

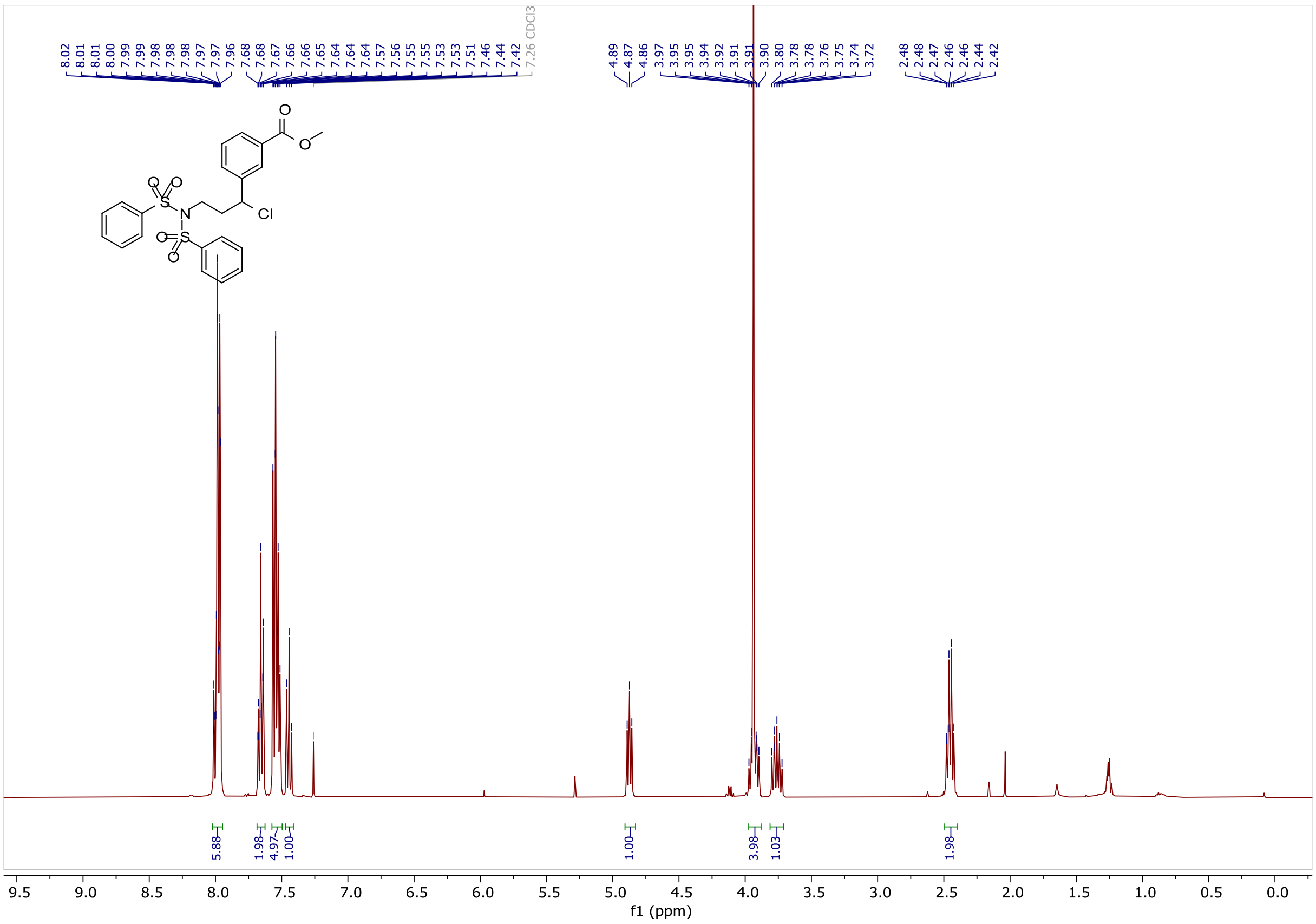

^{13}C NMR Spectrum of *m*-6g (101 MHz, $CDCl_3$)

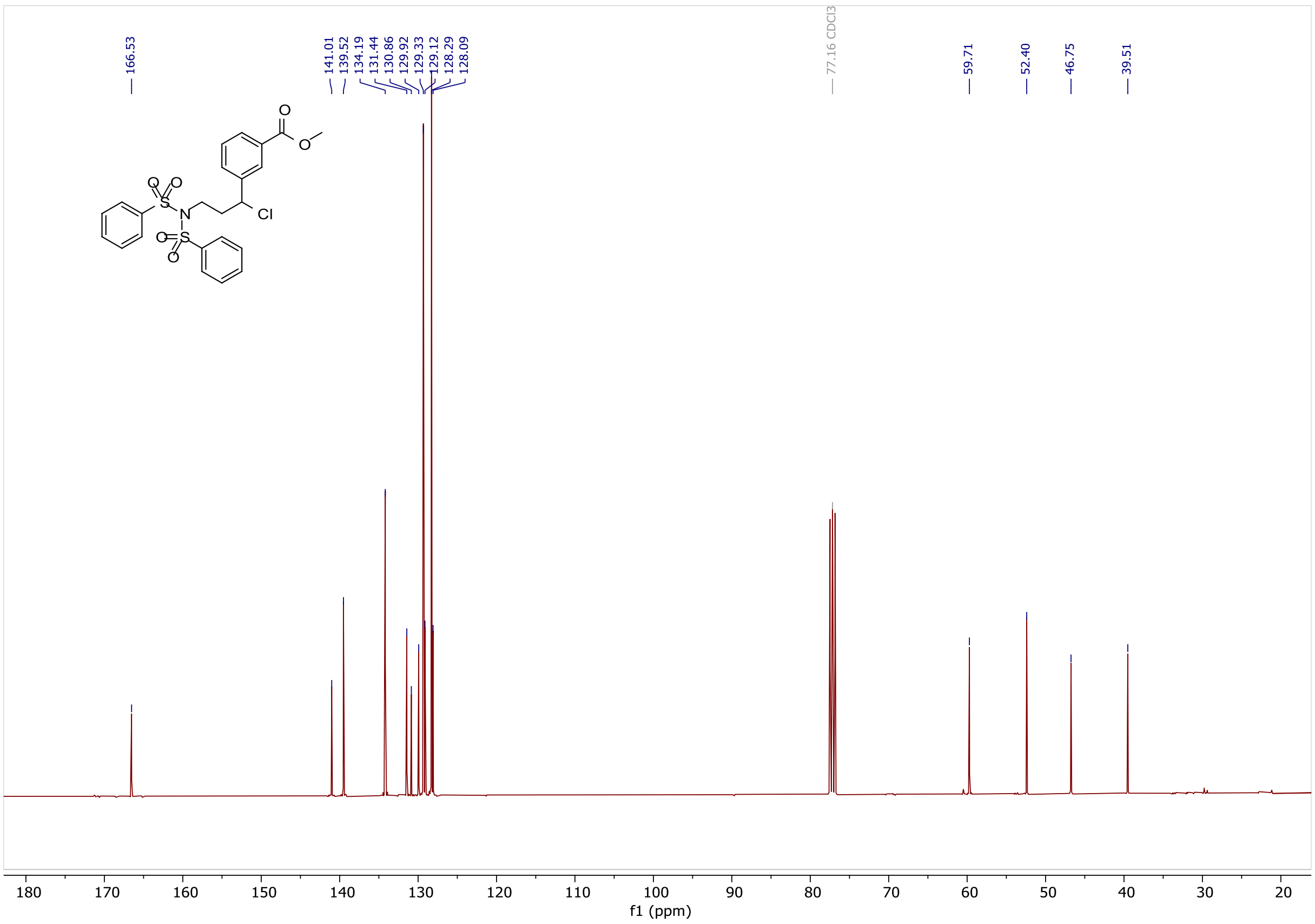

^{1}H NMR Spectrum of *p*-6g (400 MHz, $CDCl_3$)

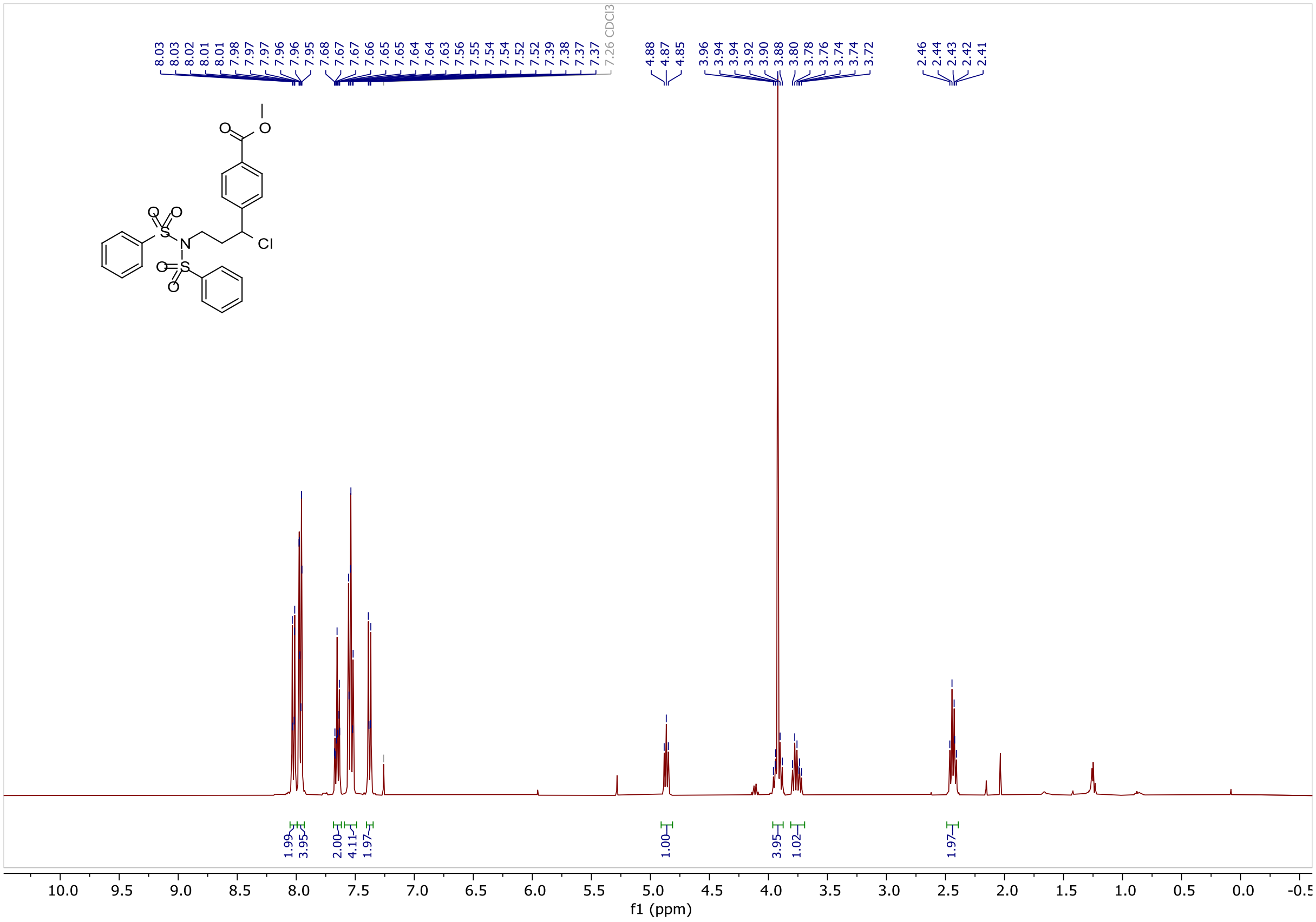

^{13}C NMR Spectrum of *p*-6g (101 MHz, $CDCl_3$)

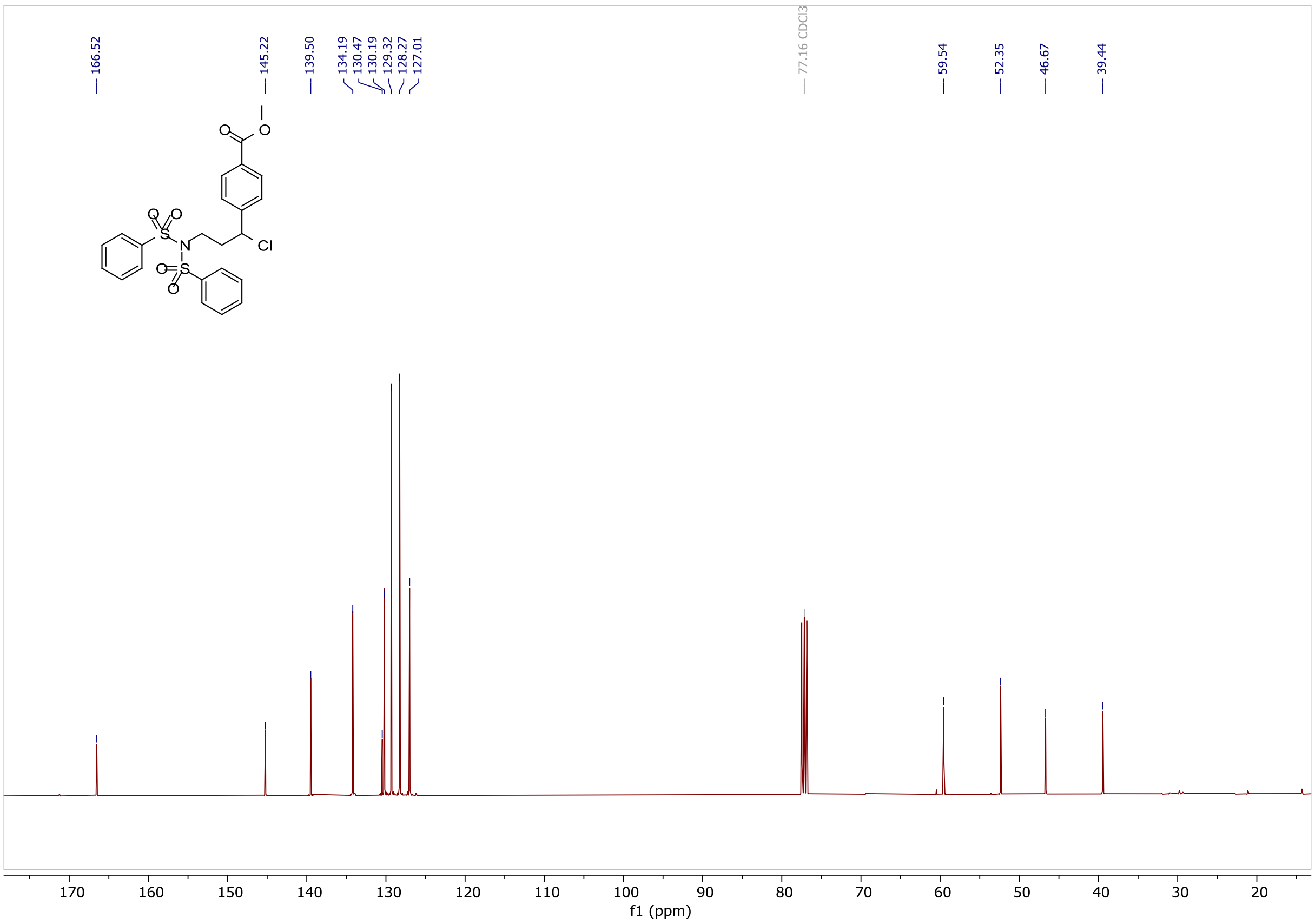

^{1}H NMR Spectrum of *m*-6h (400 MHz, $CDCl_3$)

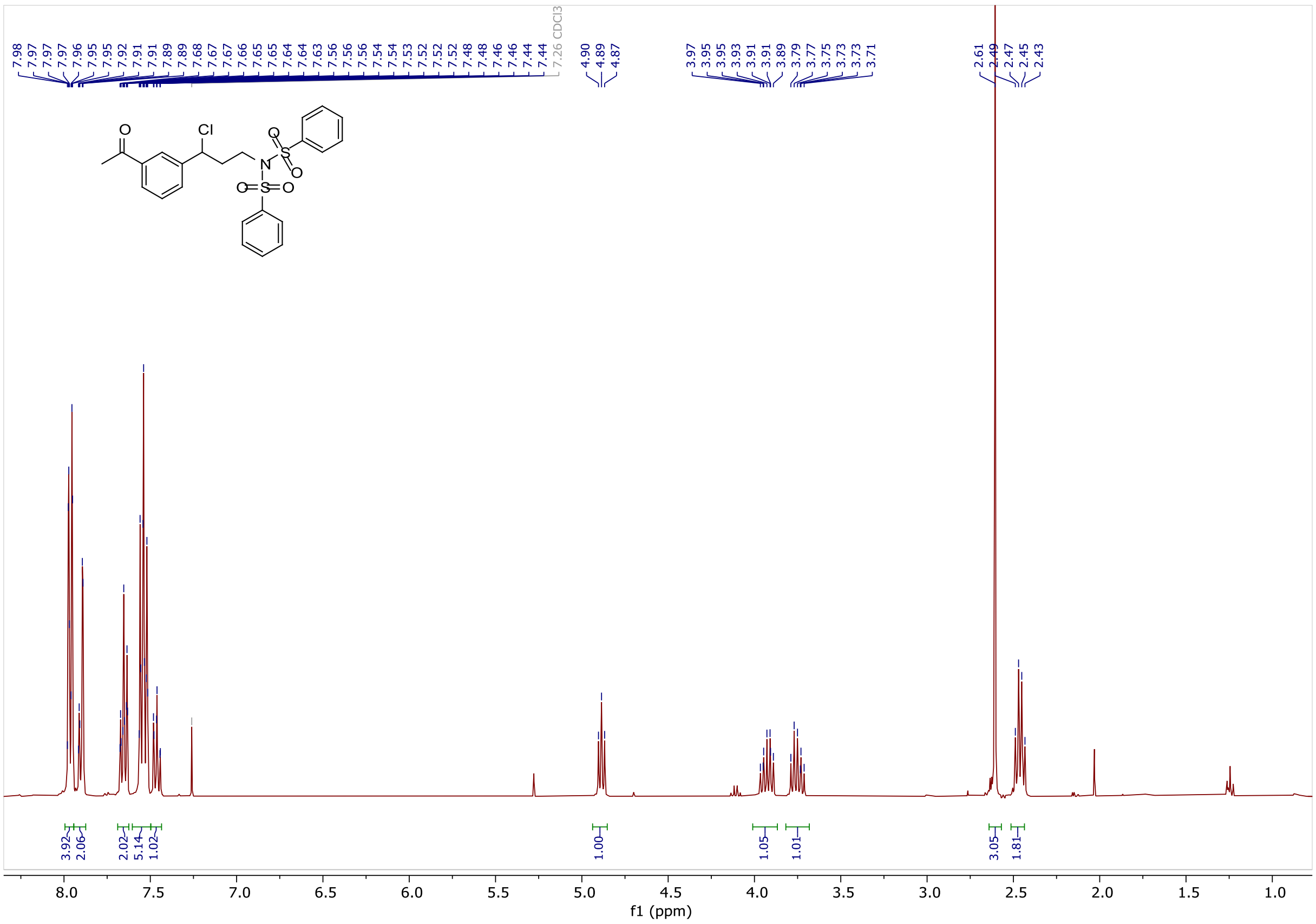

^{13}C NMR Spectrum of *m*-6h (101 MHz, $CDCl_3$)

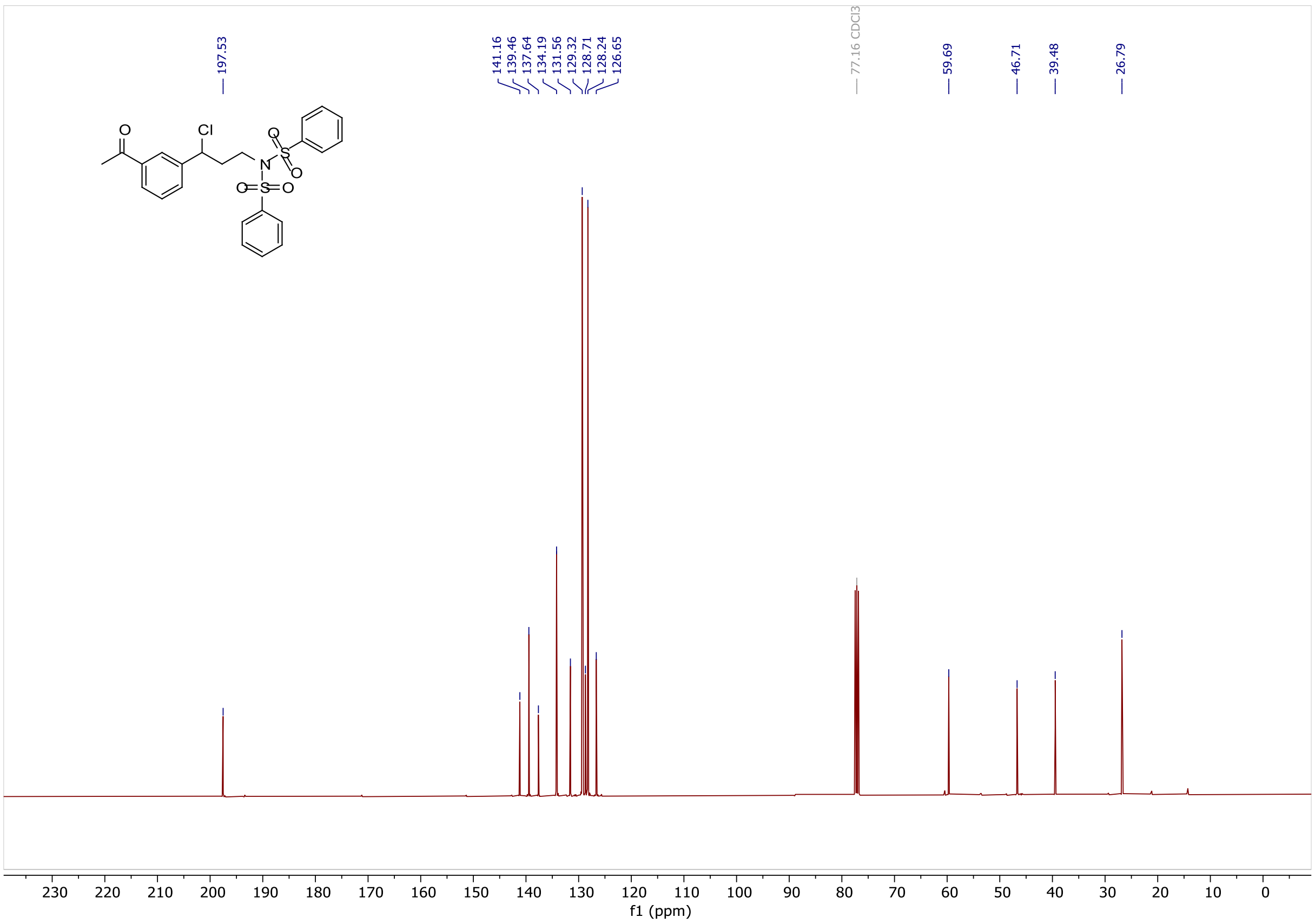

^{1}H NMR Spectrum of *p*-6h (400 MHz, $CDCl_3$)

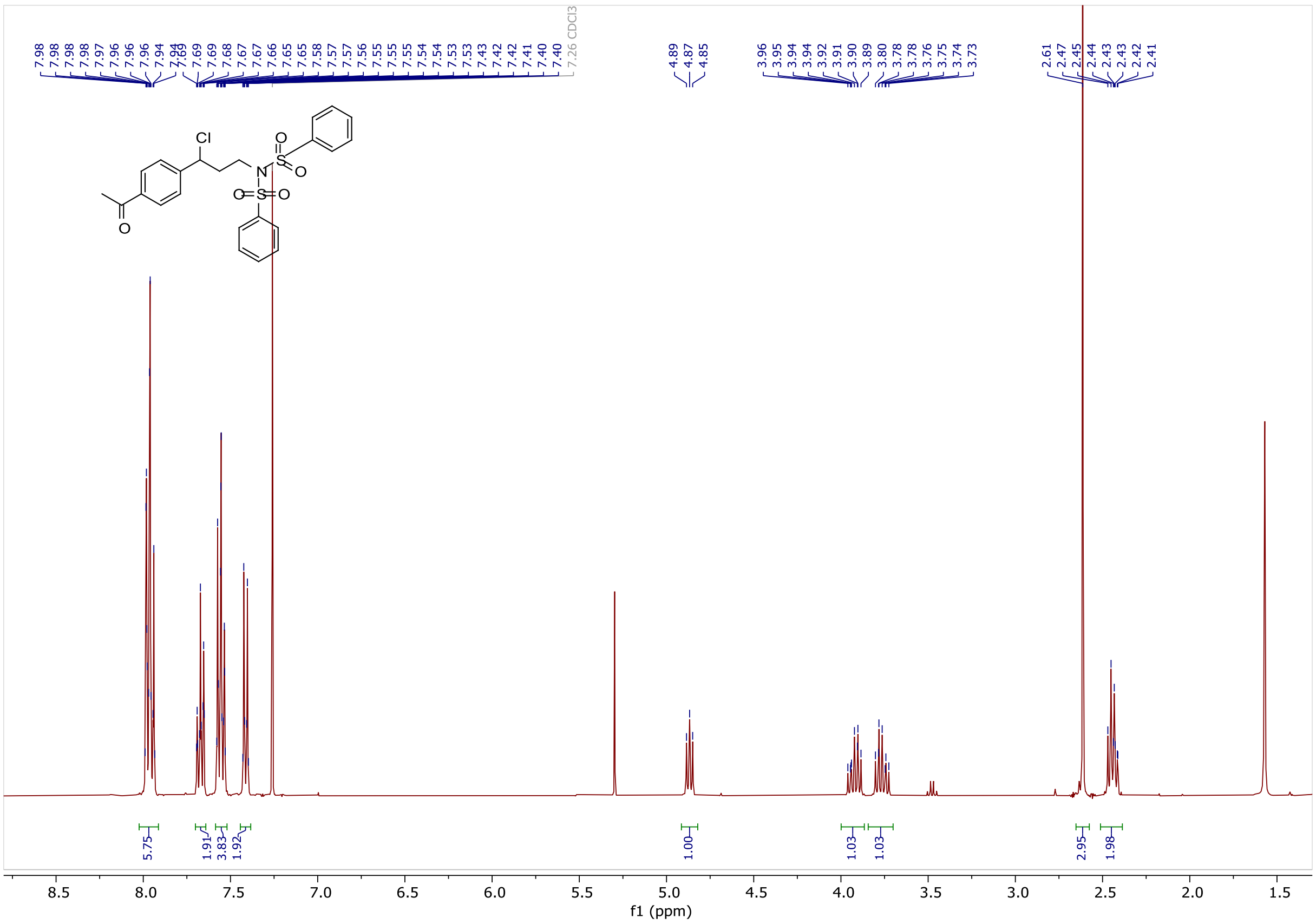

^{13}C NMR Spectrum of *p*-6h (101 MHz, $CDCl_3$)

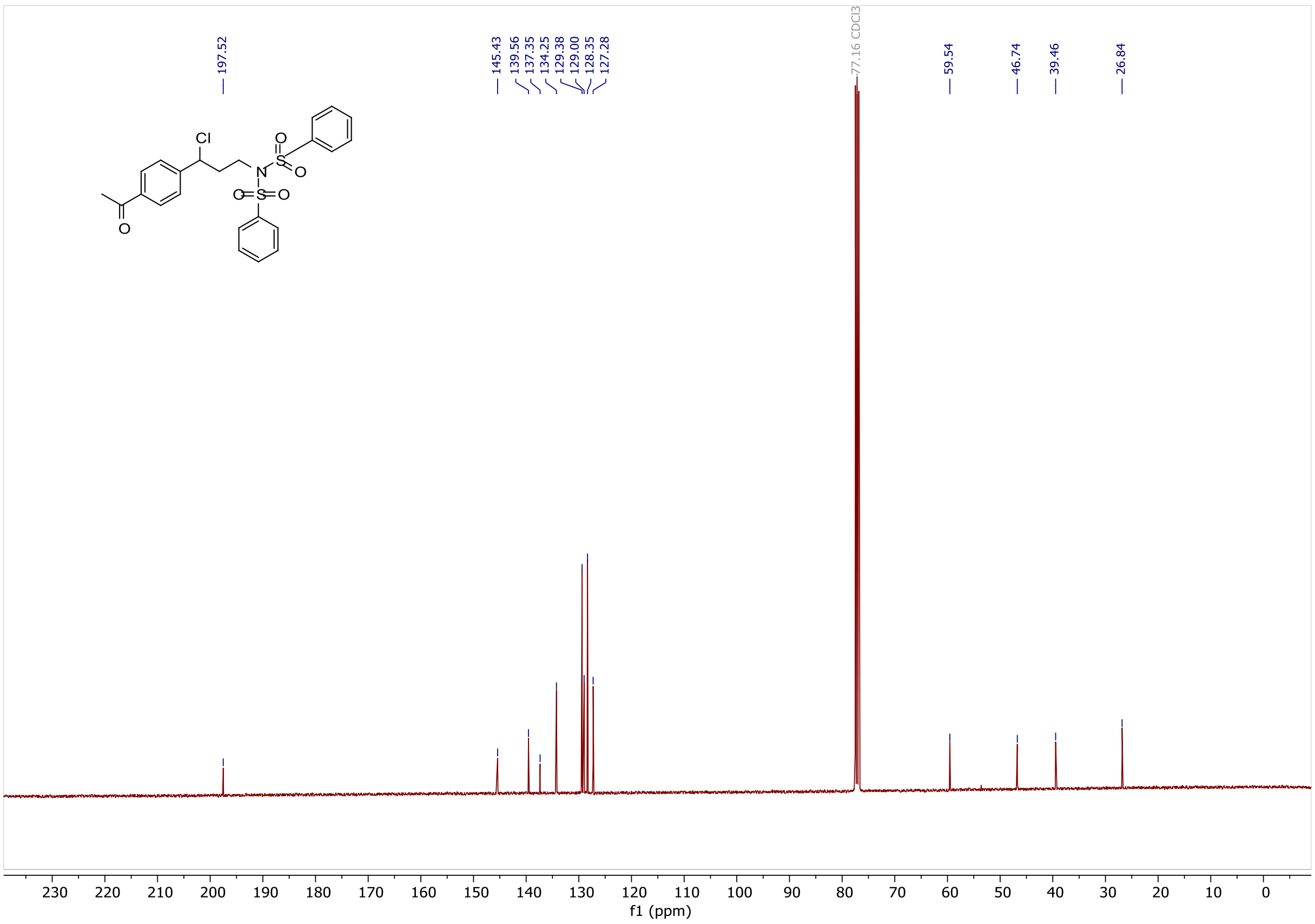

^{1}H NMR Spectrum of *o*-6i (400 MHz, $CDCl_3$)

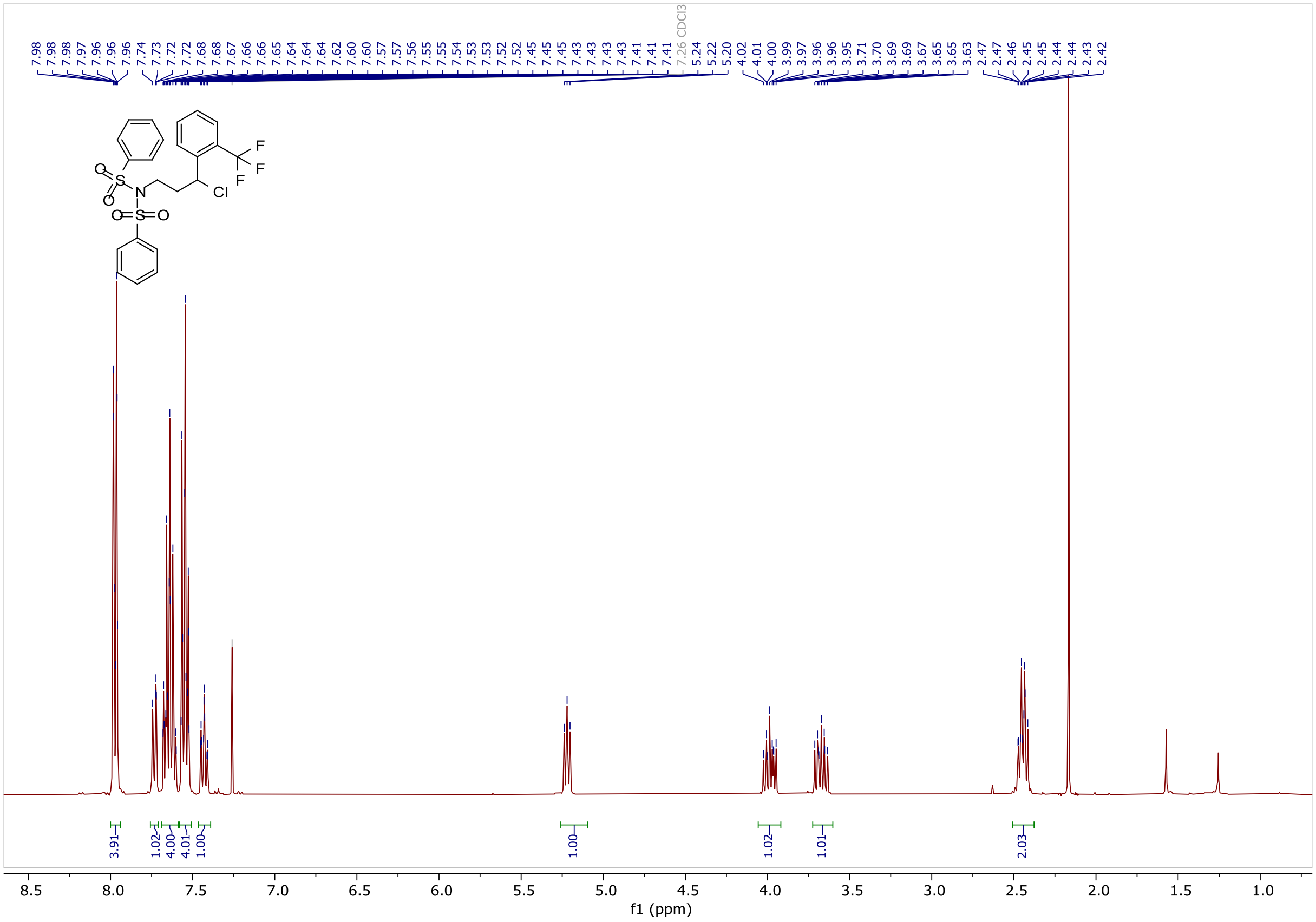

^{19}F NMR Spectrum of *o*-6i (376 MHz, $CDCl_3$)

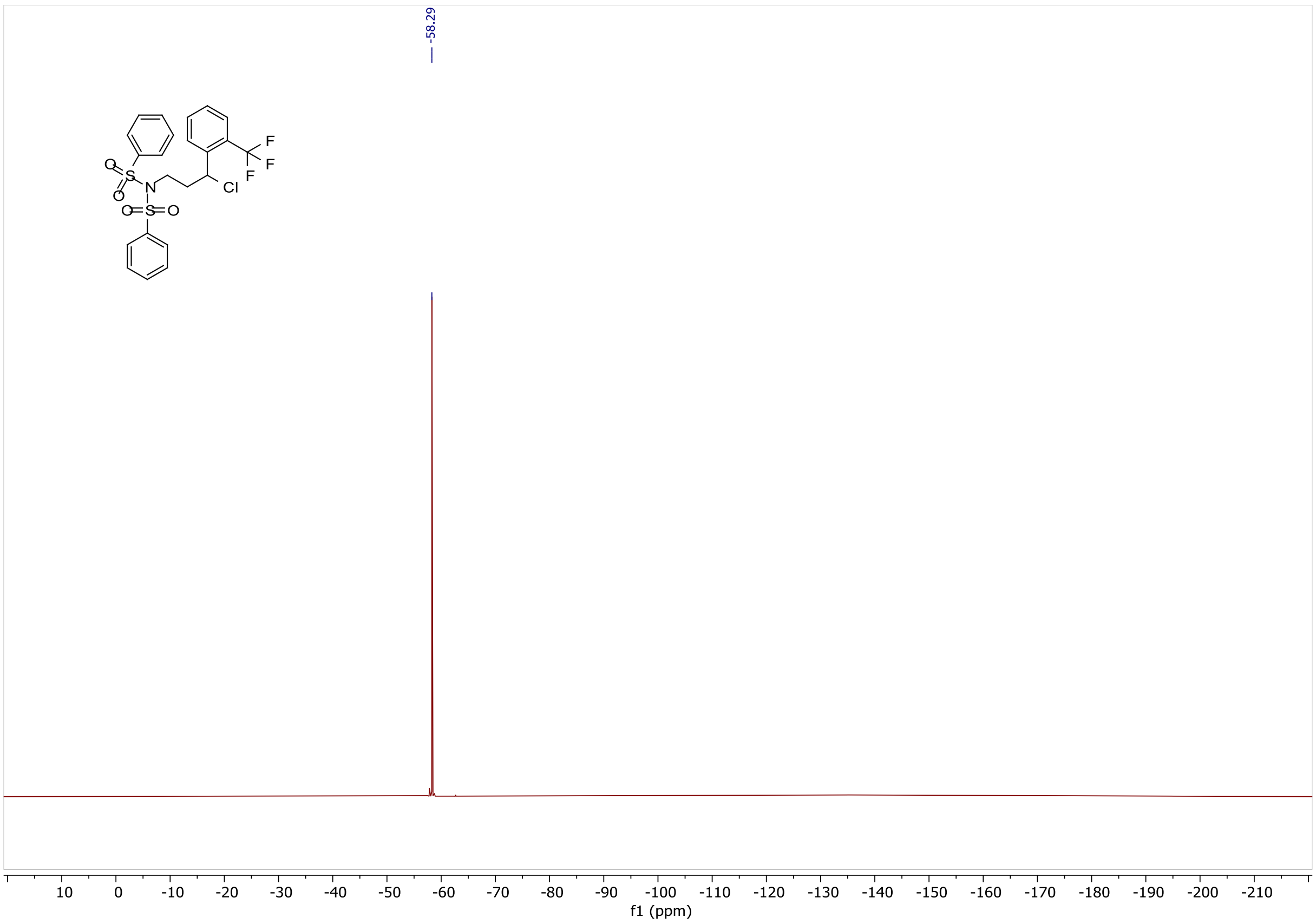

^{13}C NMR Spectrum of *o*-6i (101 MHz, $CDCl_3$)

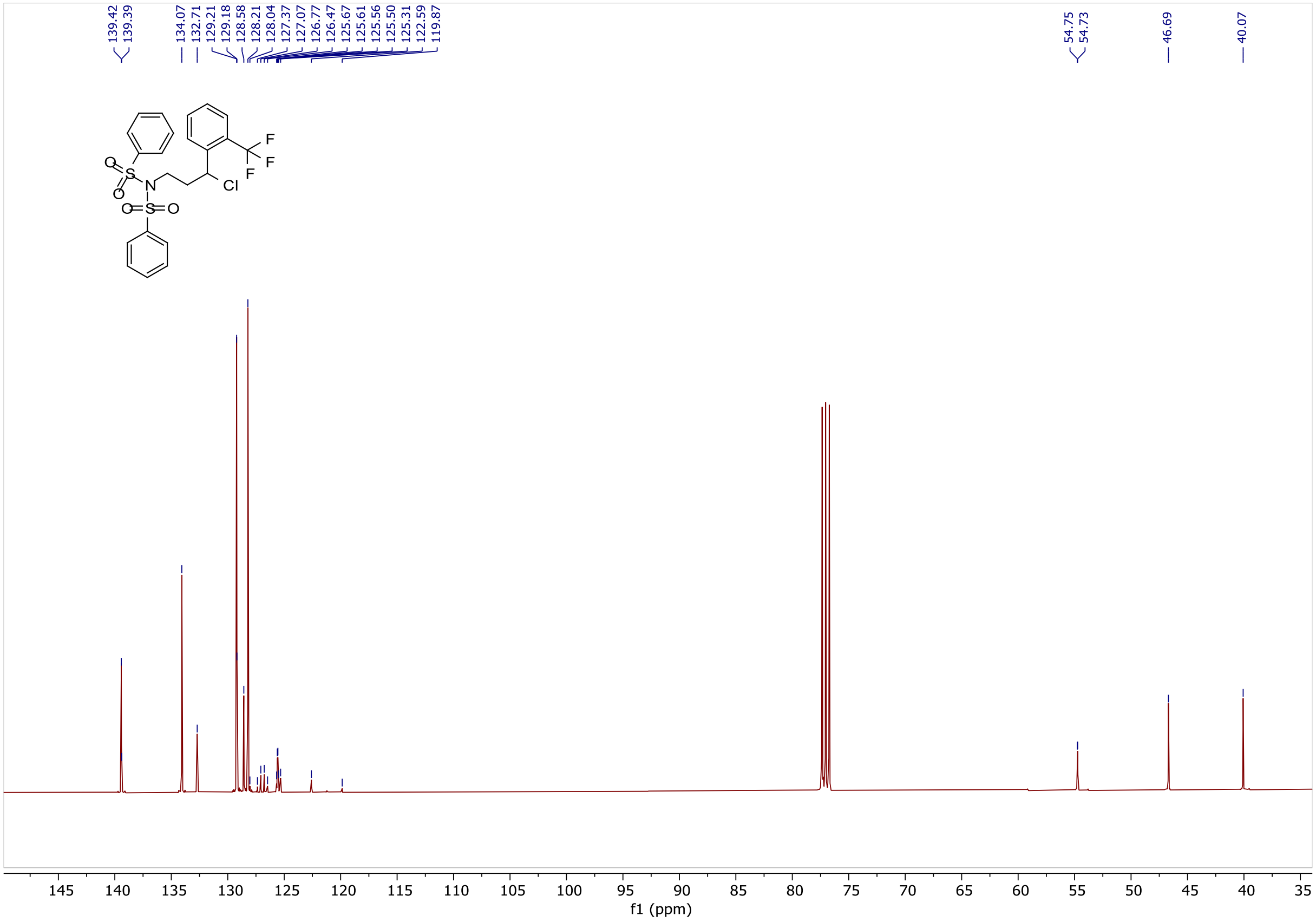

^{1}H NMR Spectrum of *m*-6i (400 MHz, $CDCl_3$)

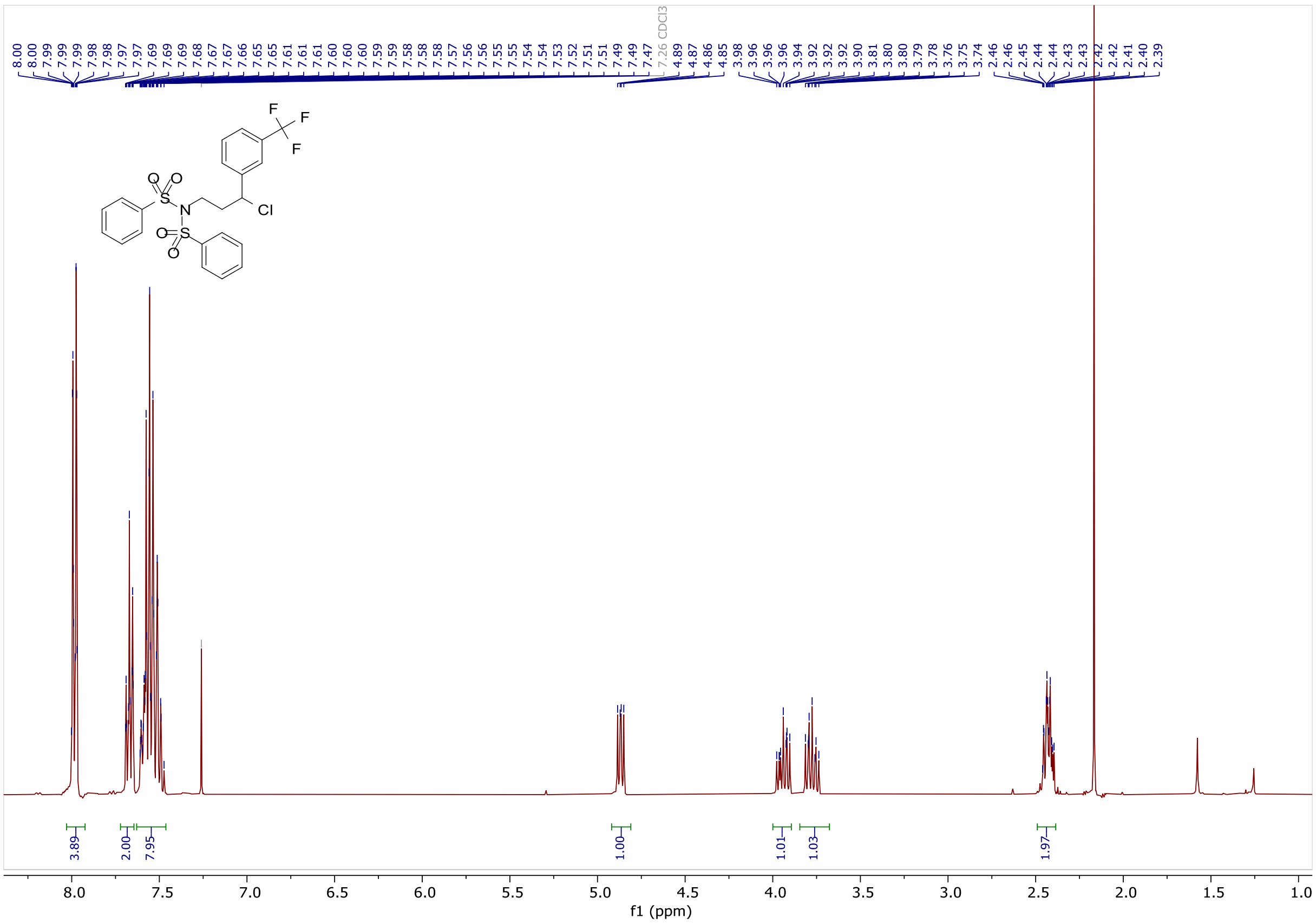

^{19}F NMR Spectrum of *m*-6i (376 MHz, $CDCl_3$)

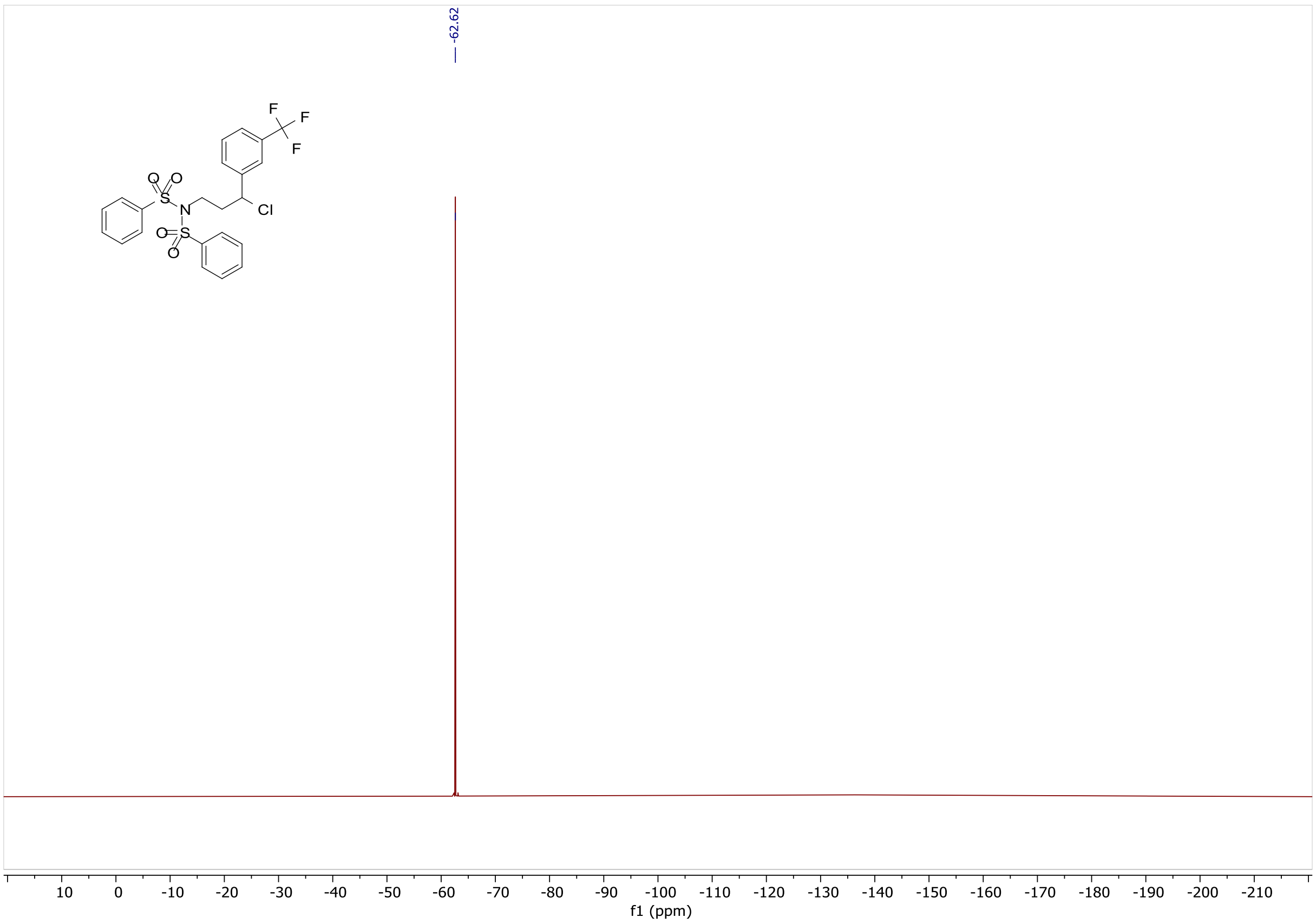

^{13}C NMR Spectrum of *m*-6i (101 MHz, $CDCl_3$)

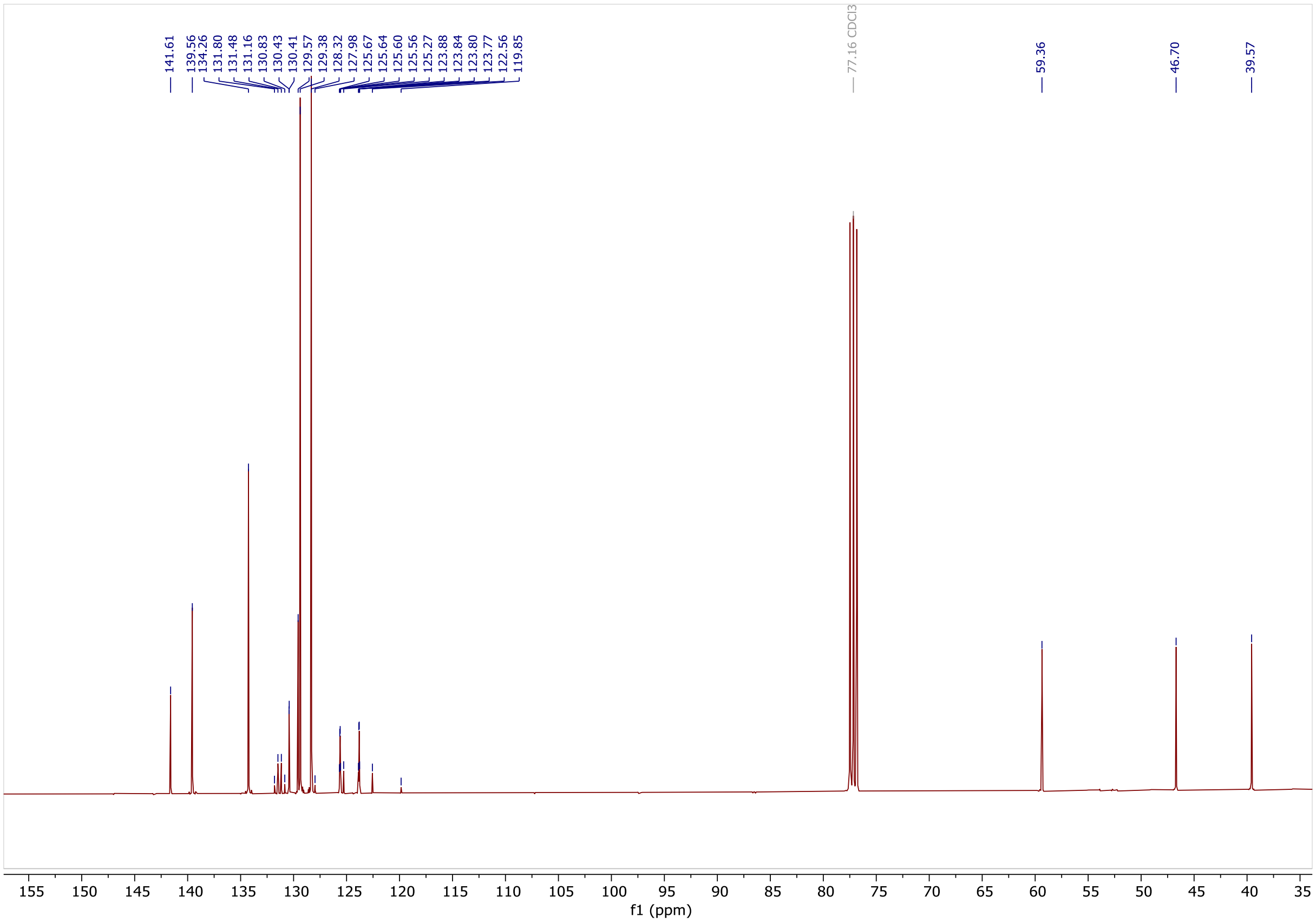

^{1}H NMR Spectrum of *p*-6i (400 MHz, $CDCl_3$)

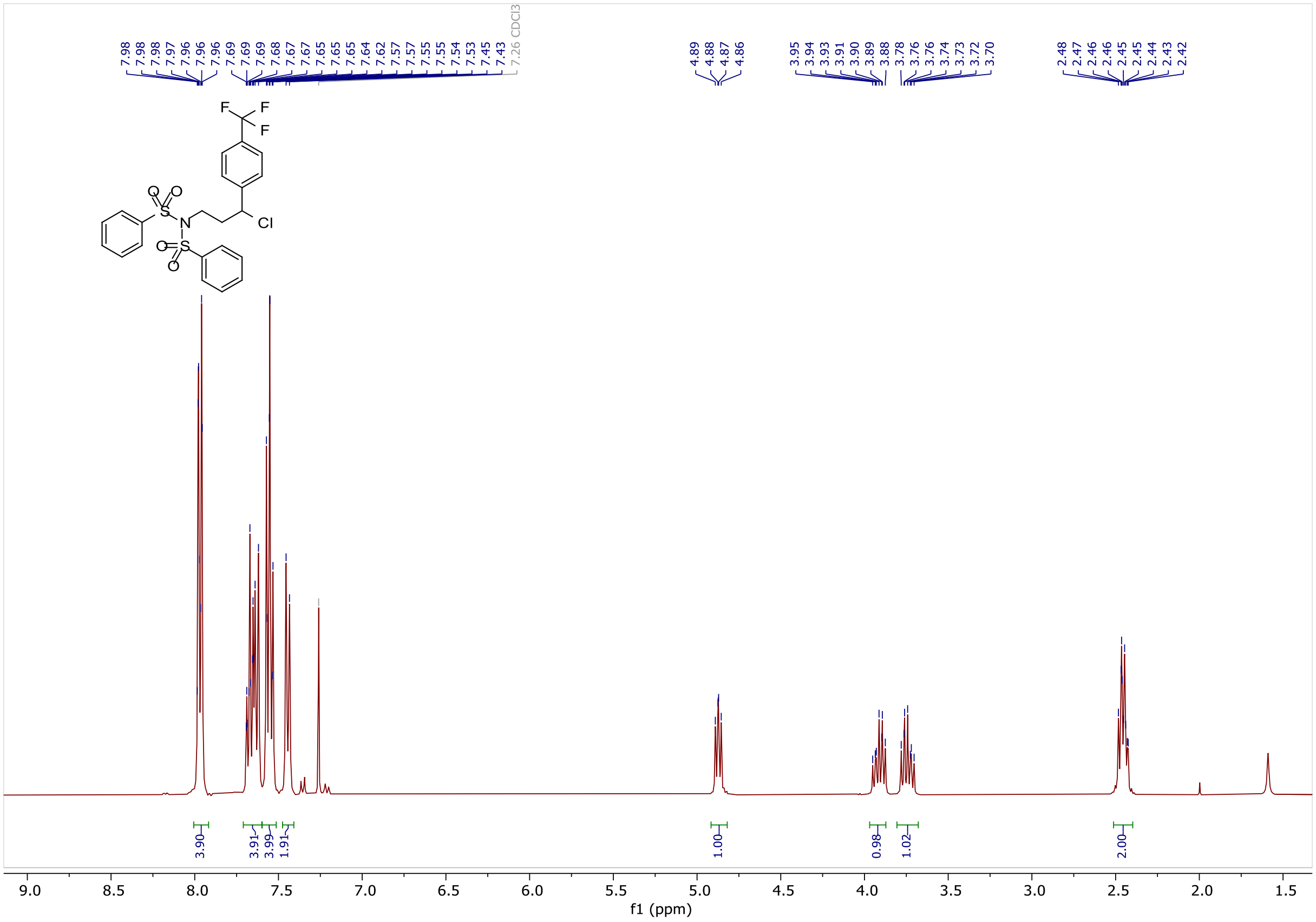

^{19}F NMR Spectrum of *p*-6i (376 MHz, $CDCl_3$)

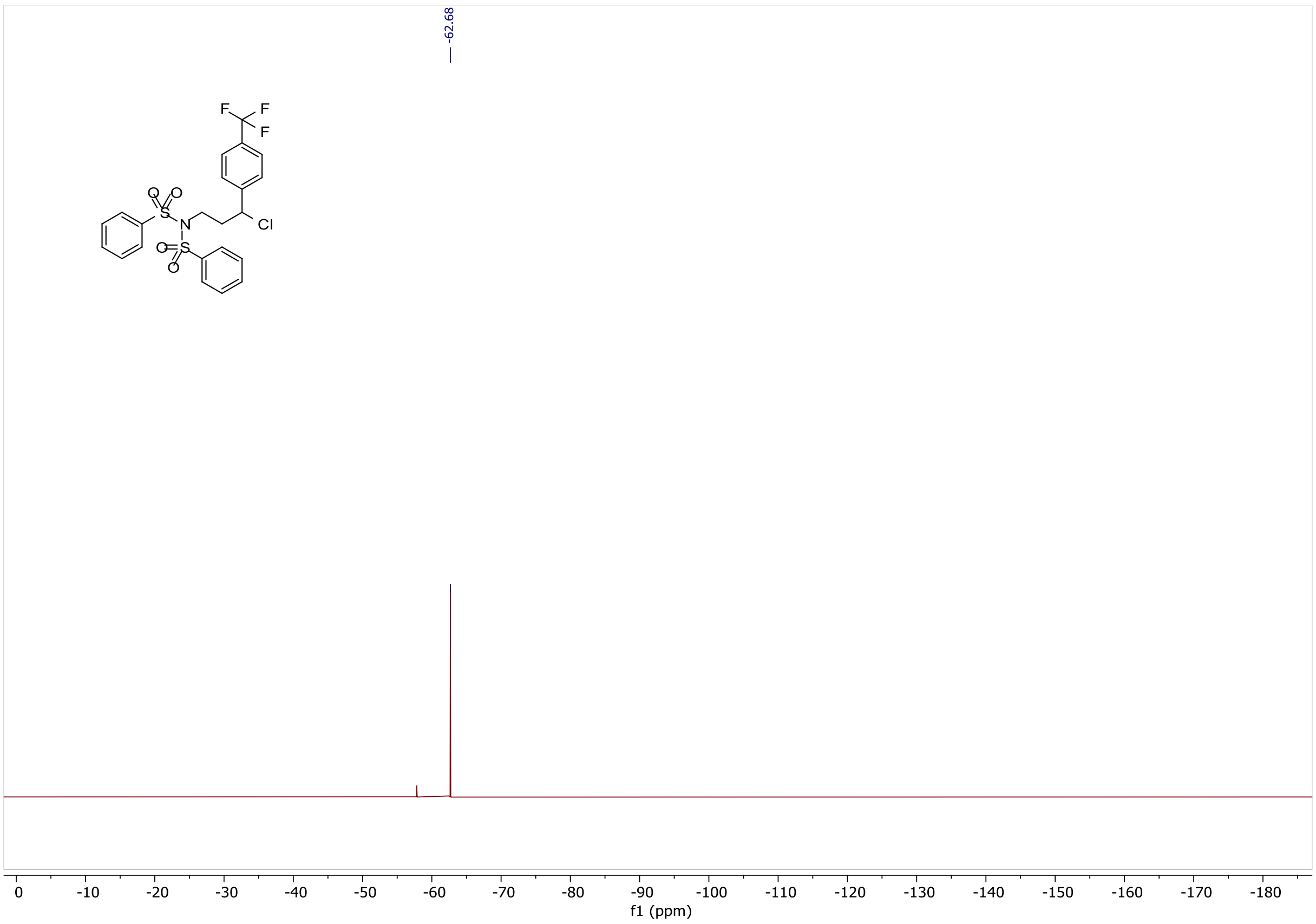

^{13}C NMR Spectrum of *p*-6i (101 MHz, $CDCl_3$)

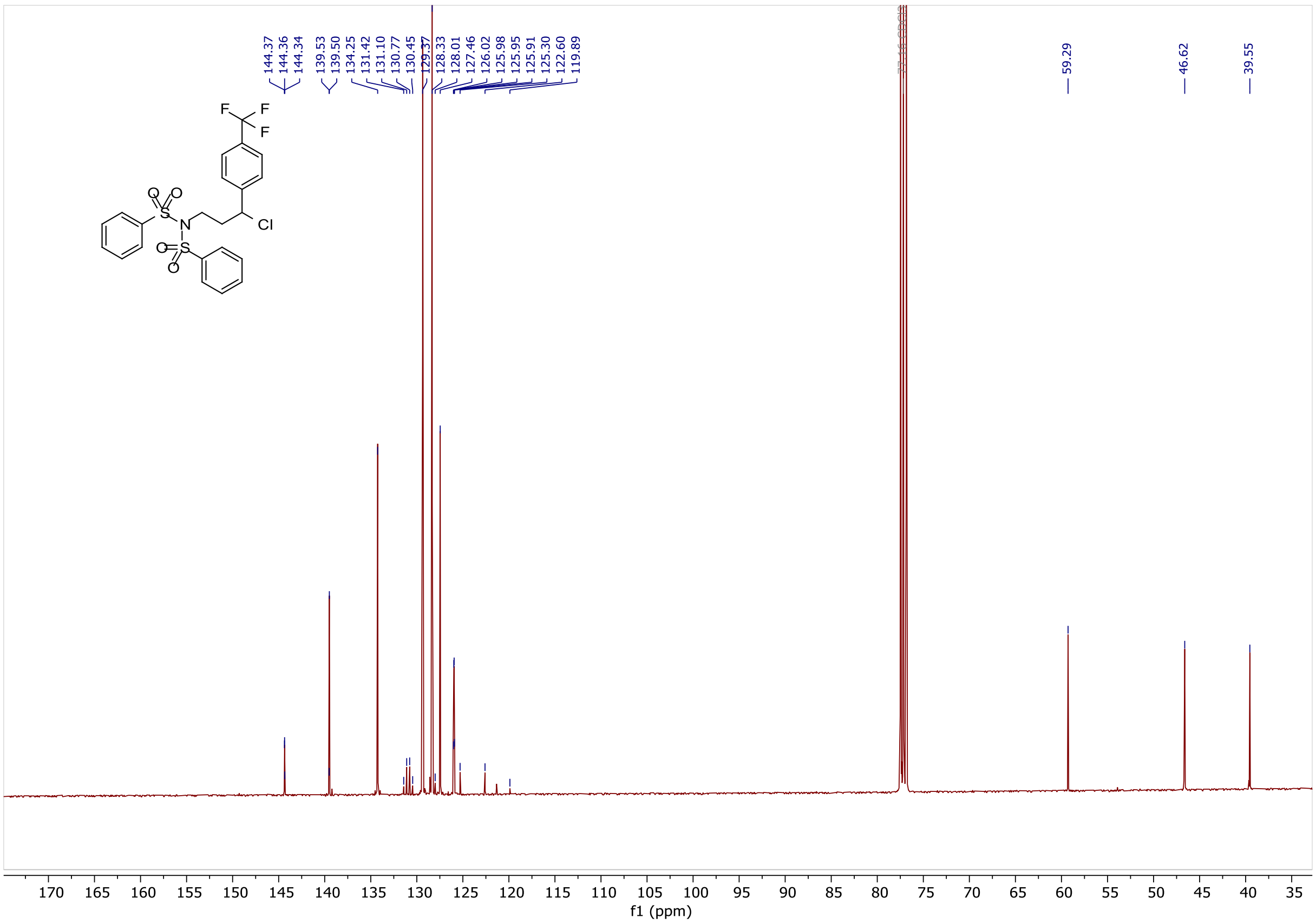

^{1}H NMR Spectrum of *o*-6j (400 MHz, $CDCl_3$)

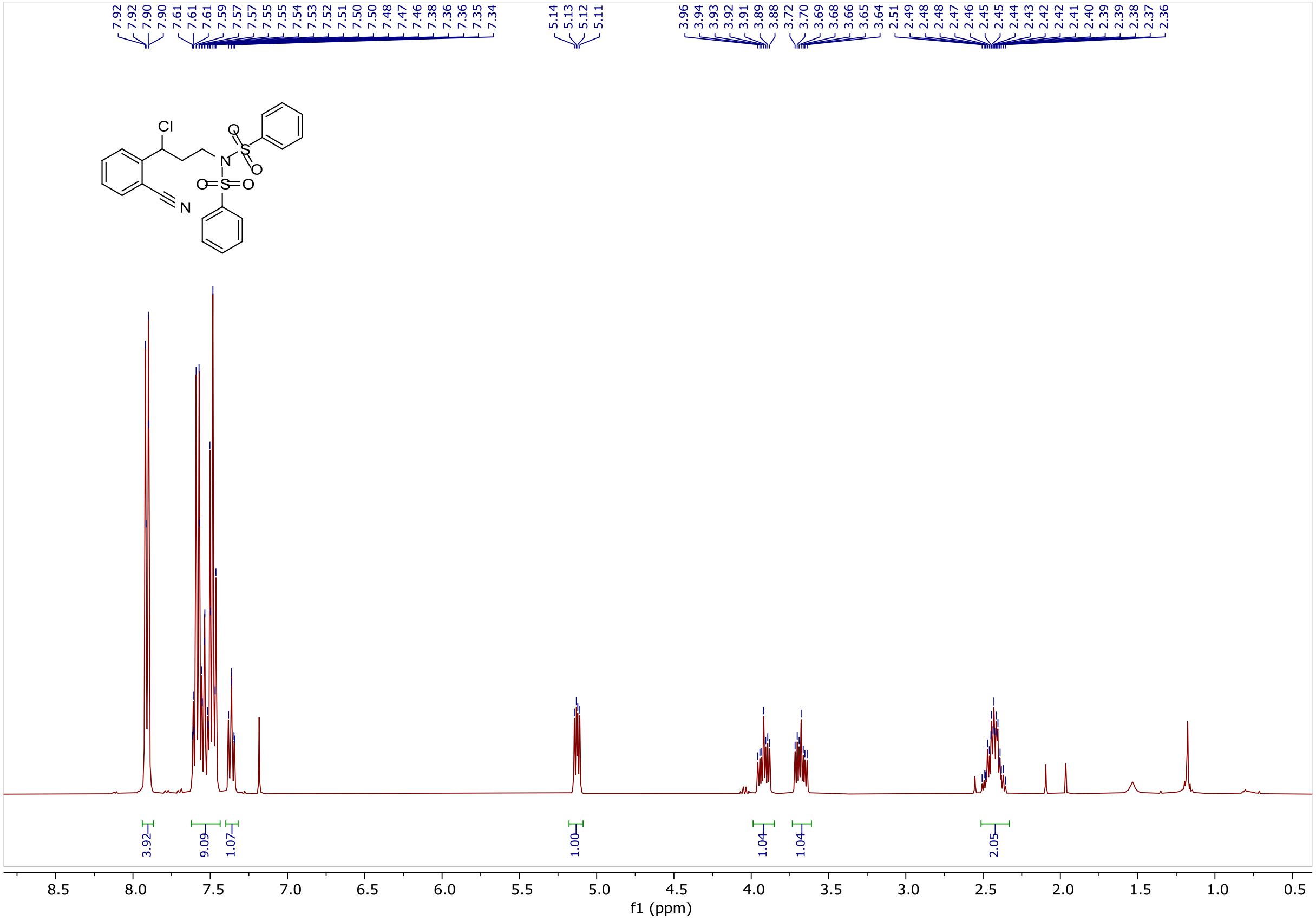

^{13}C NMR Spectrum of *o*-6j (101 MHz, $CDCl_3$)

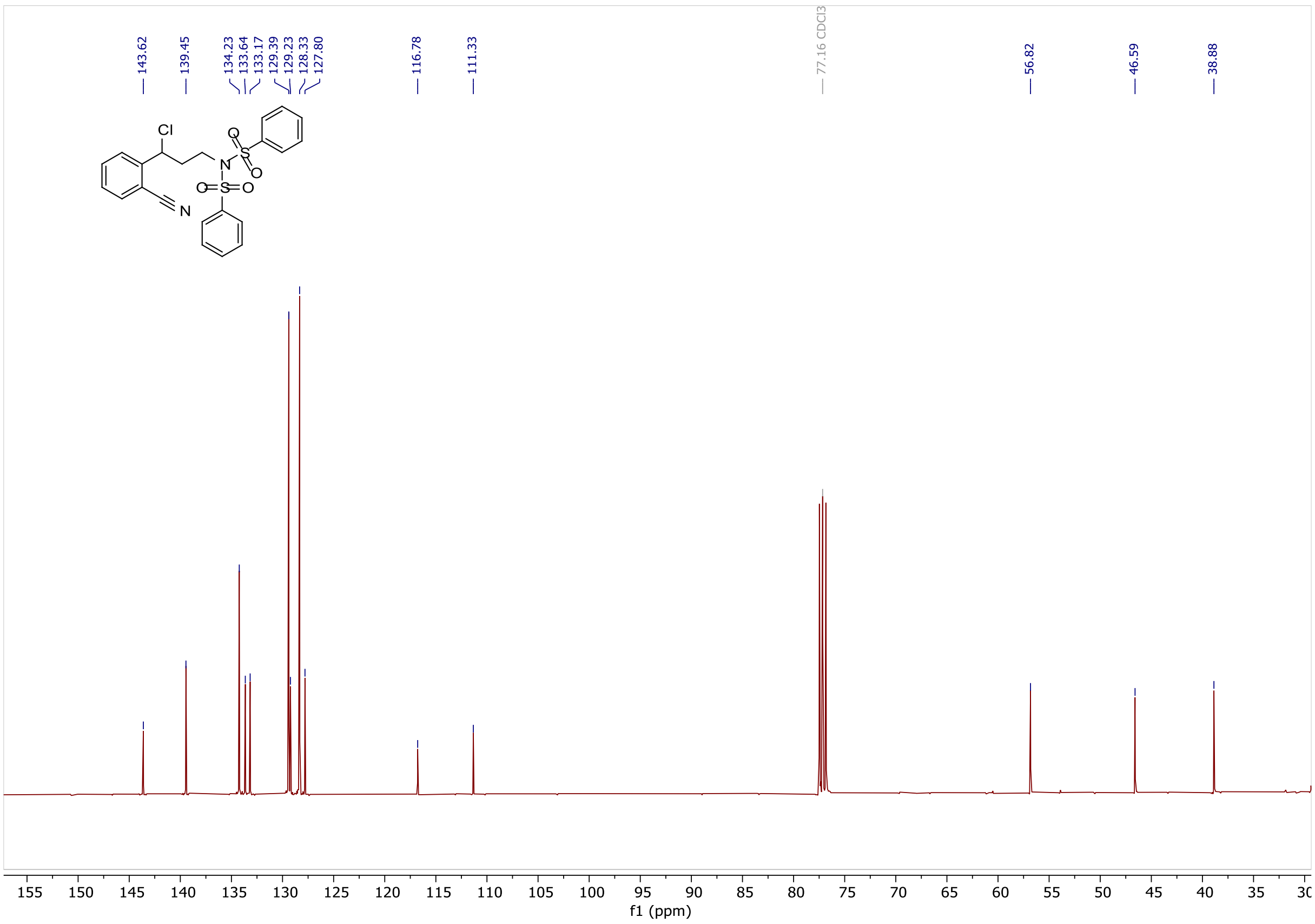

^{1}H NMR Spectrum of *m*-6j (400 MHz, $CDCl_3$)

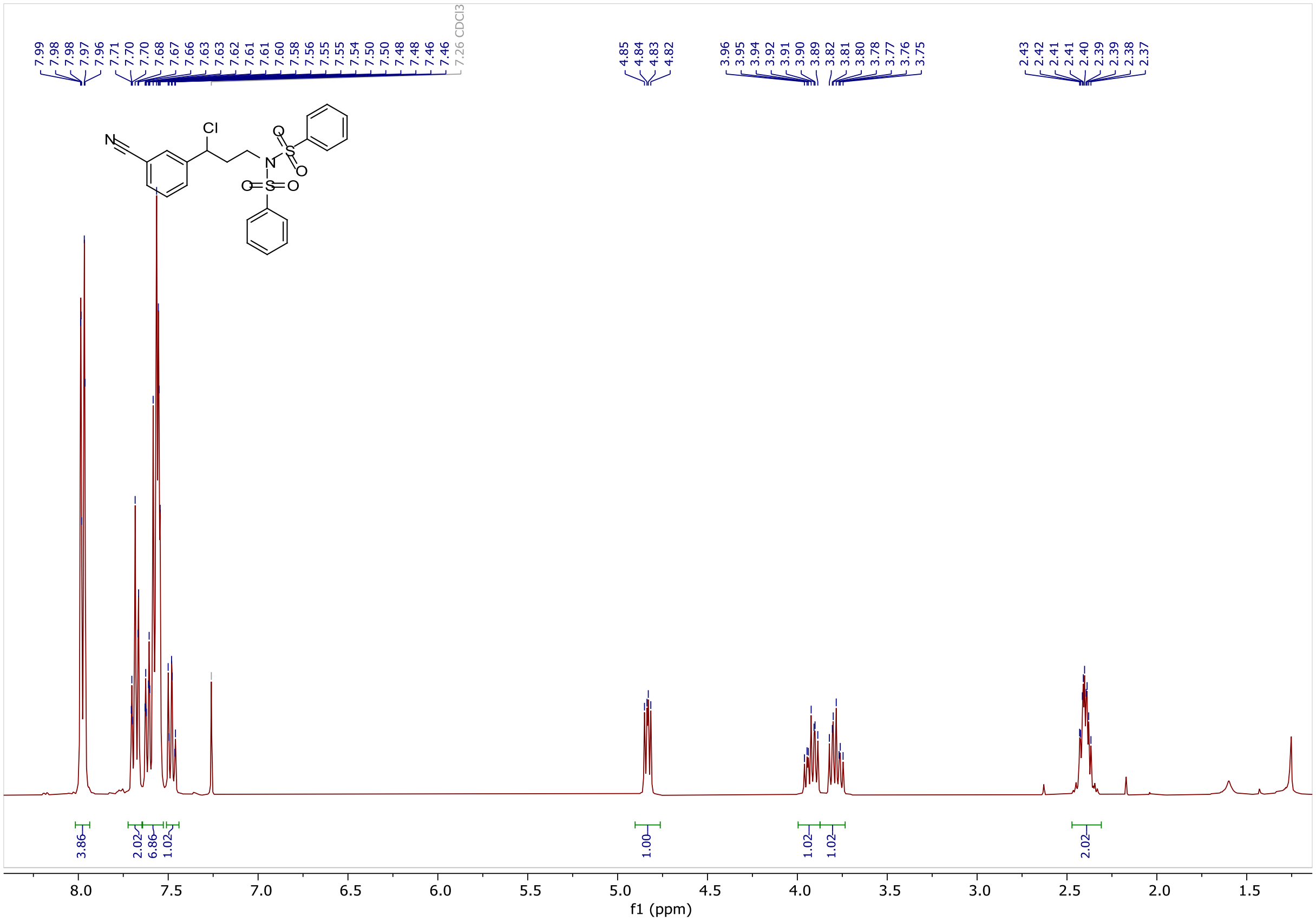

^{13}C NMR Spectrum of *m*-6j (101 MHz, $CDCl_3$)

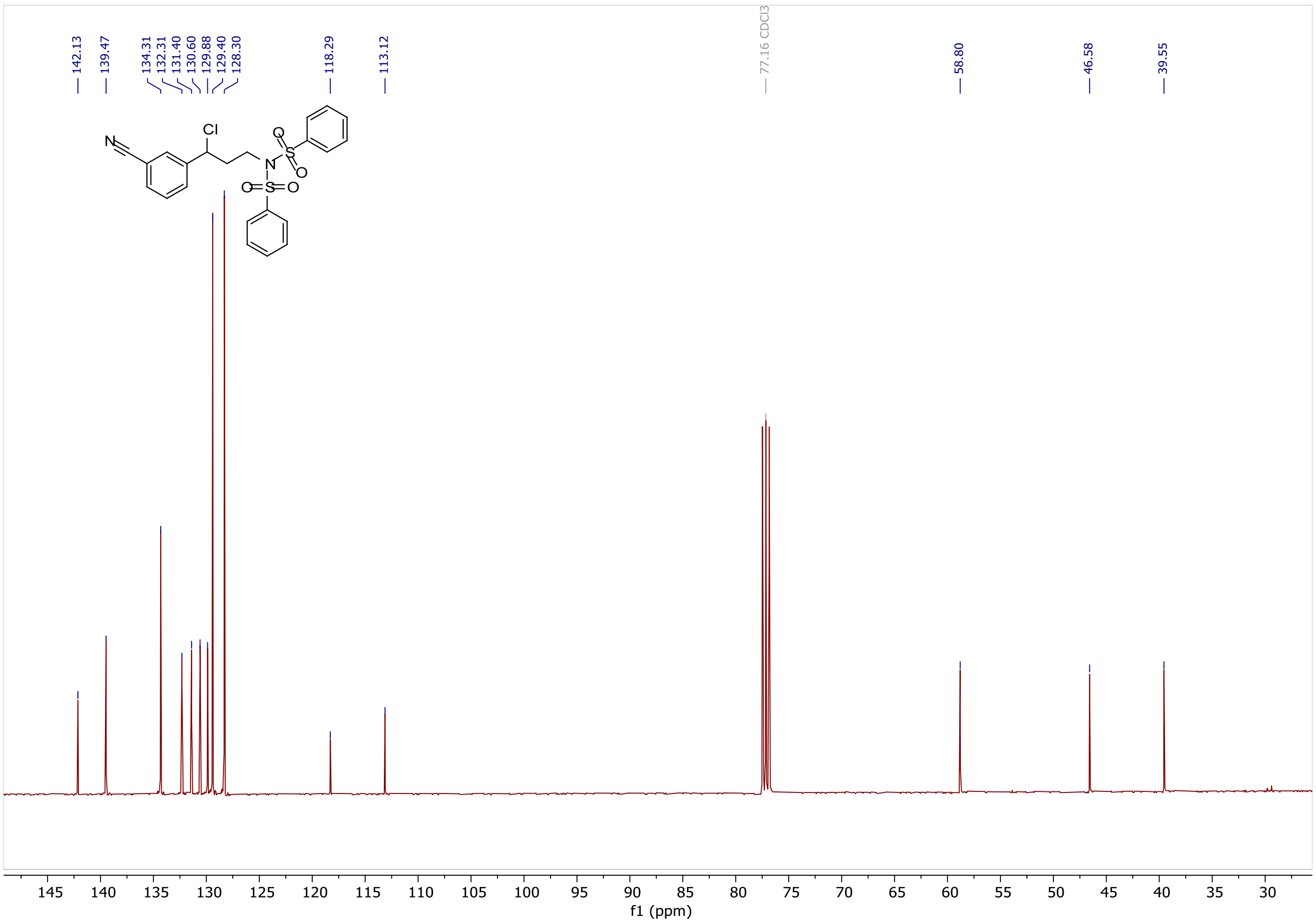

^{1}H NMR Spectrum of *p*-6j (400 MHz, $CDCl_3$)

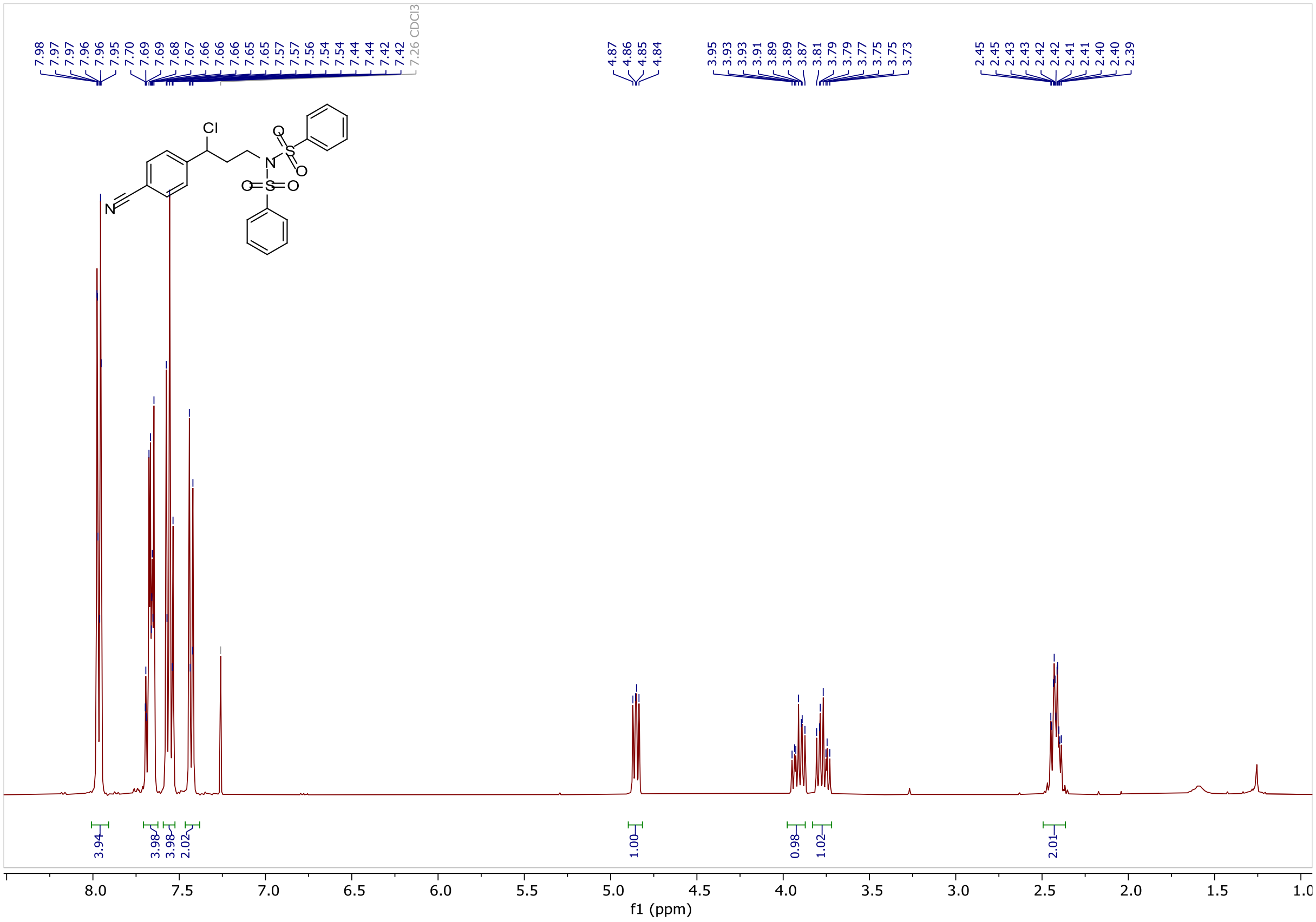

^{13}C NMR Spectrum of *p*-6j (101 MHz, $CDCl_3$)

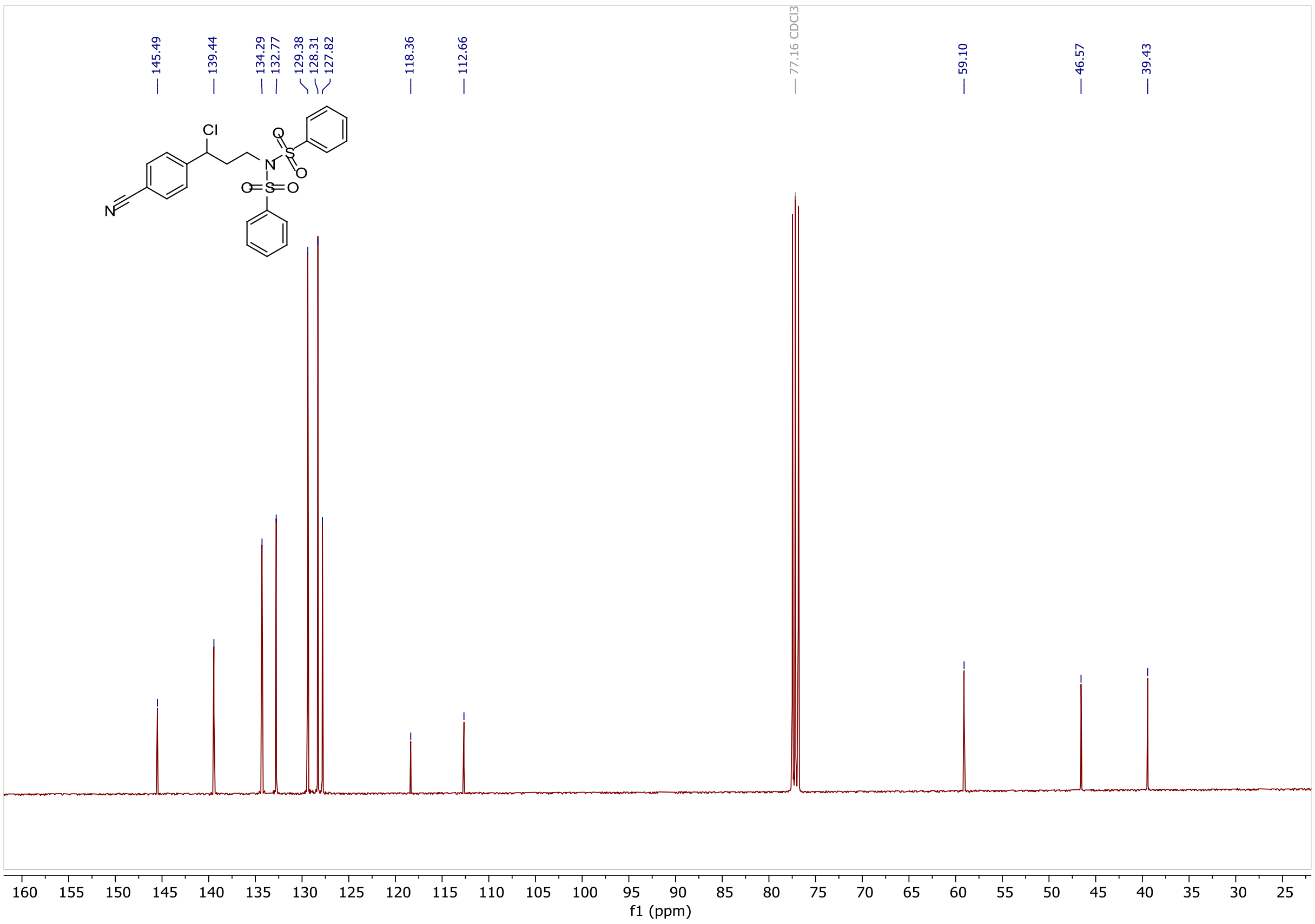

^{1}H NMR Spectrum of *o*-6k (400 MHz, $CDCl_3$)

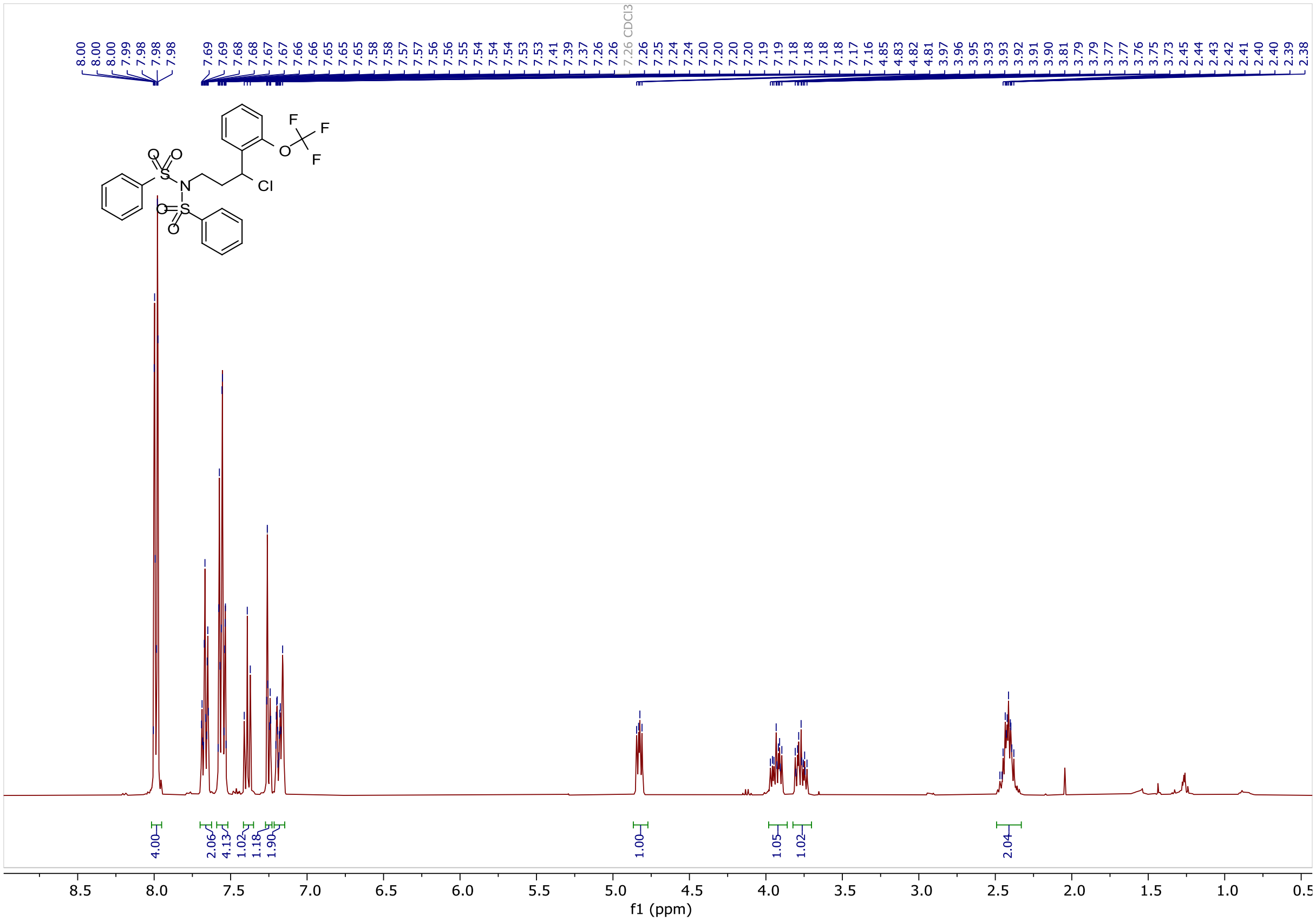

^{19}F NMR Spectrum of *o*-6k (376 MHz, $CDCl_3$)

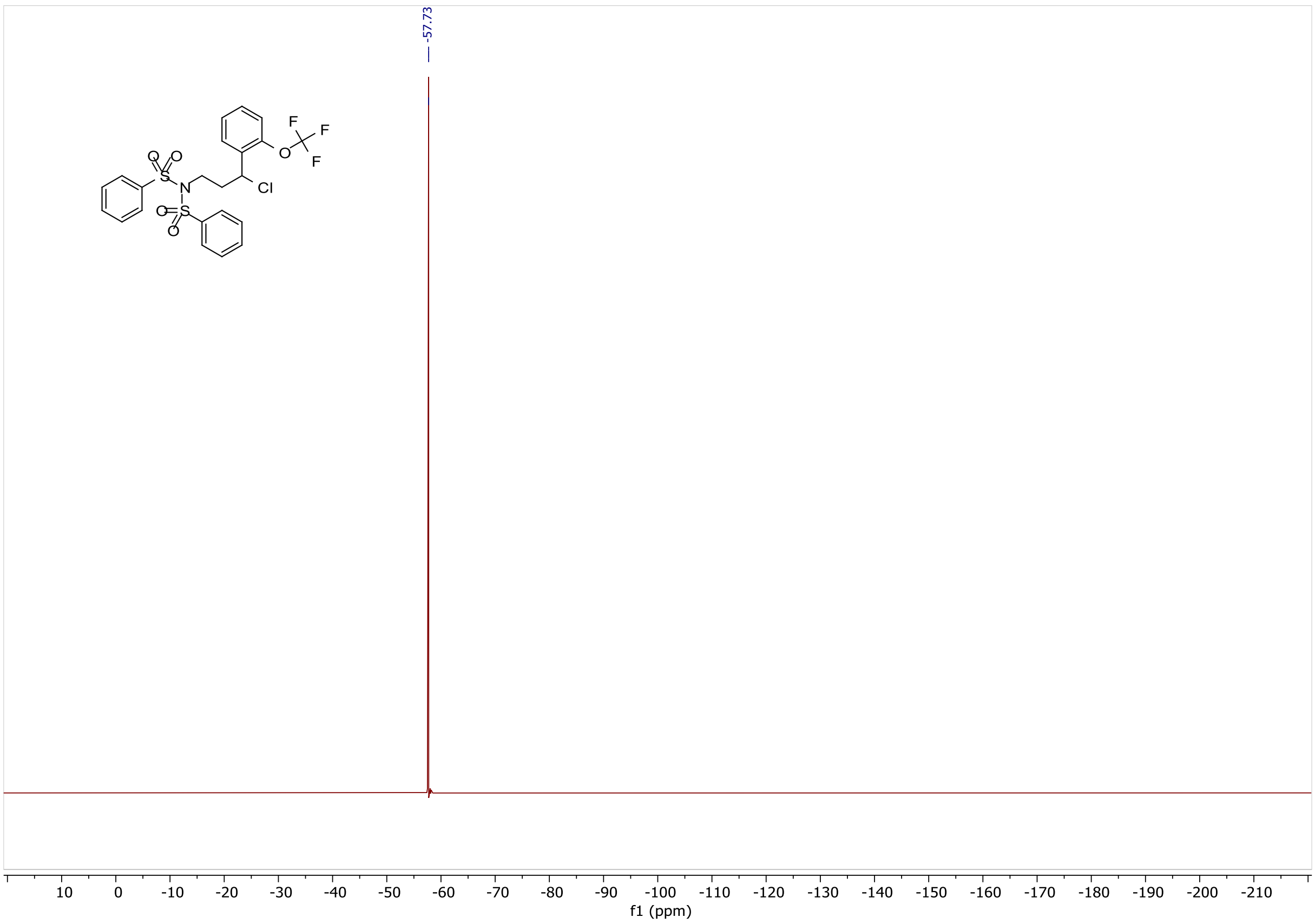

^{13}C NMR Spectrum of *o*-6k (101 MHz, $CDCl_3$)

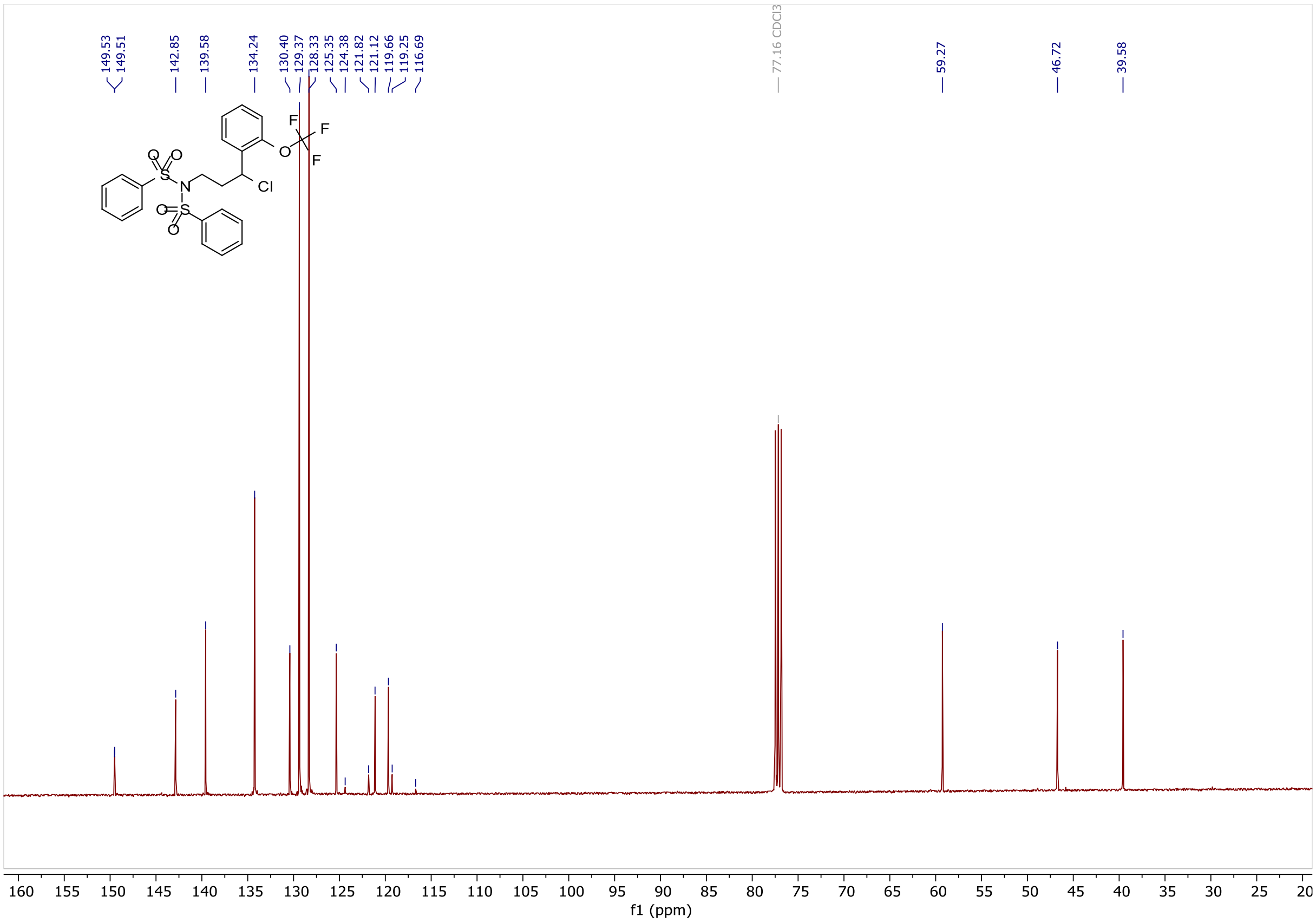

^{1}H NMR Spectrum of *m*-6k (400 MHz, $CDCl_3$)

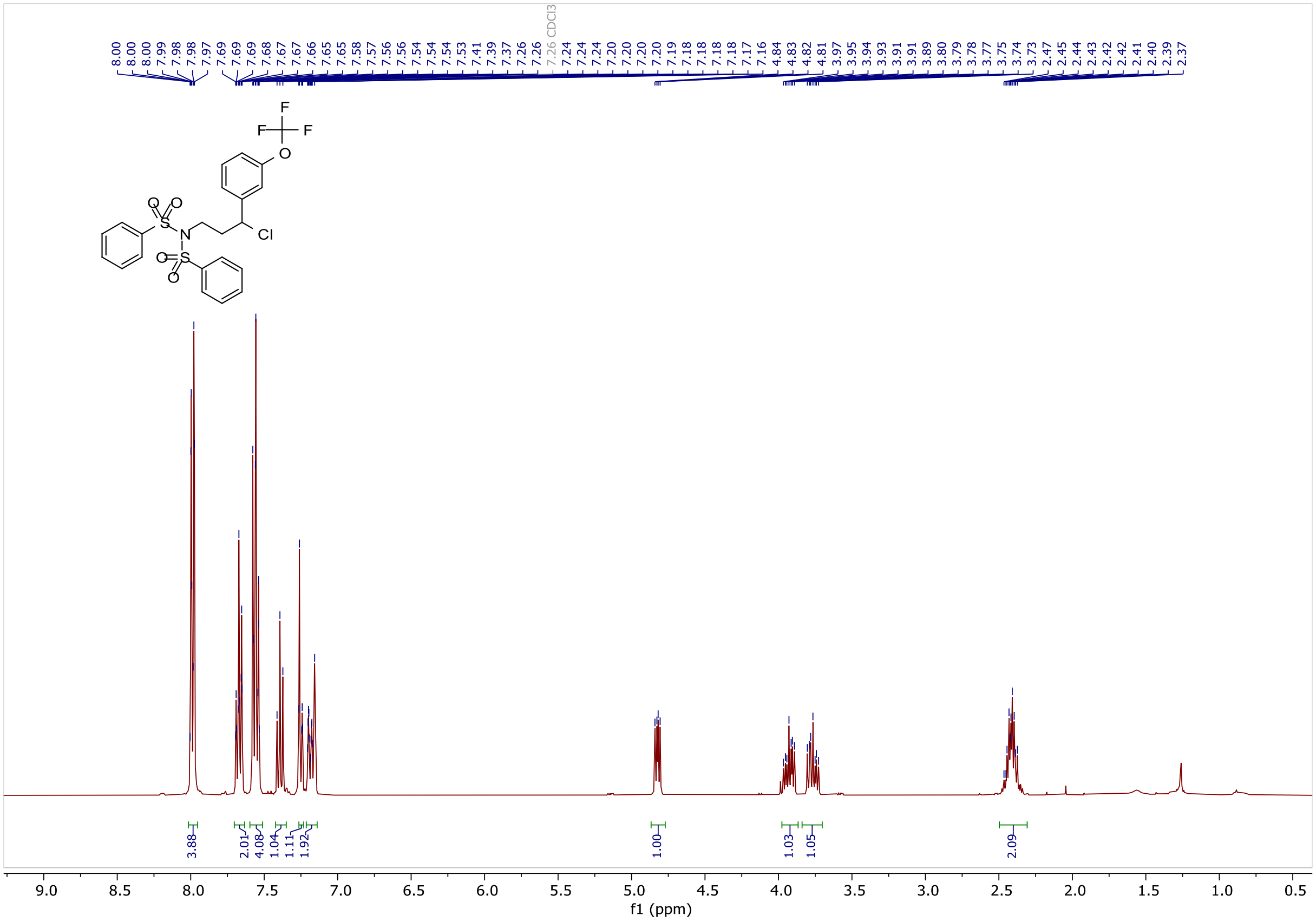

^{19}F NMR Spectrum of *m*-6k (376 MHz, $CDCl_3$)

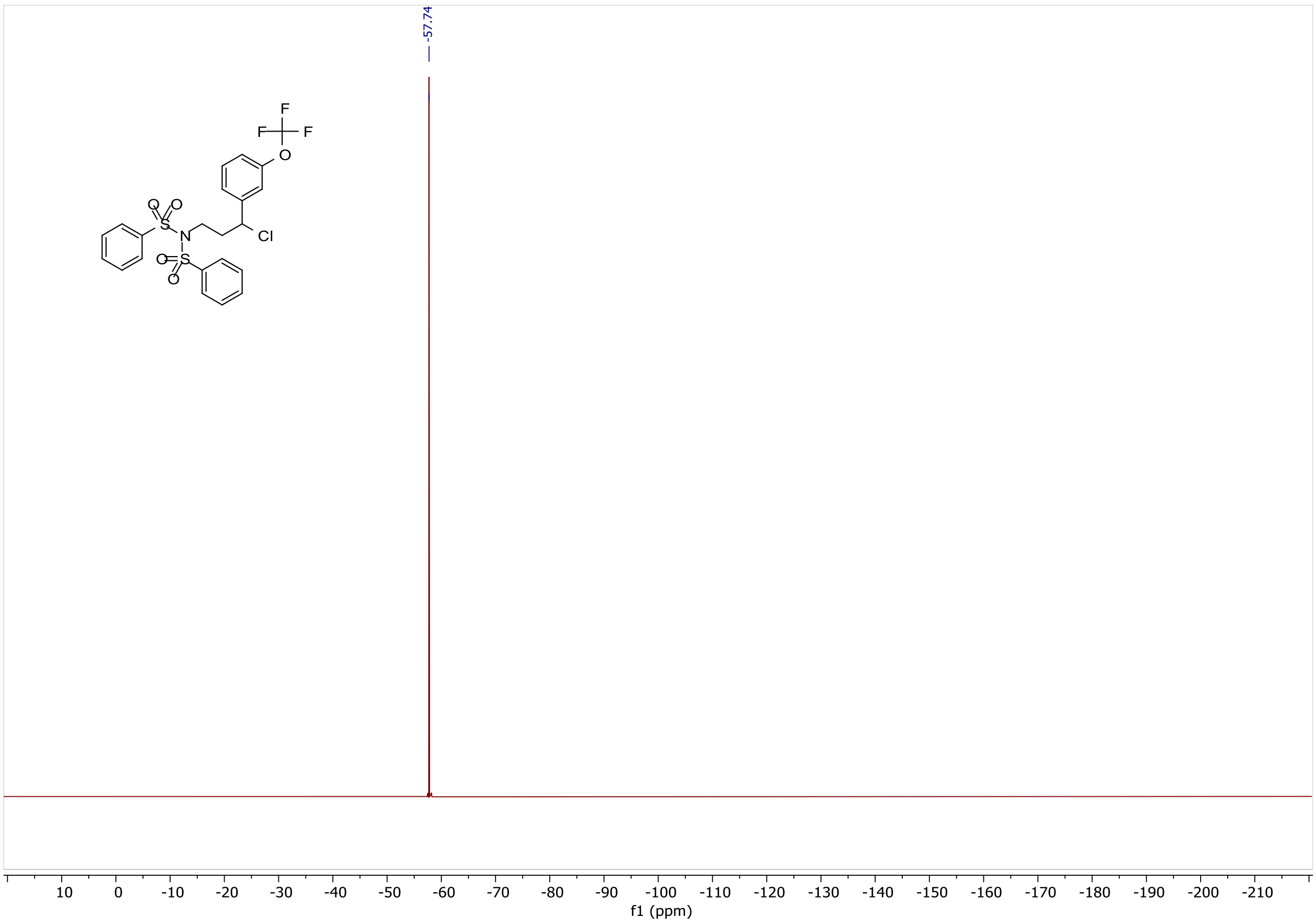

^{13}C NMR Spectrum of *m*-6k (101 MHz, $CDCl_3$)

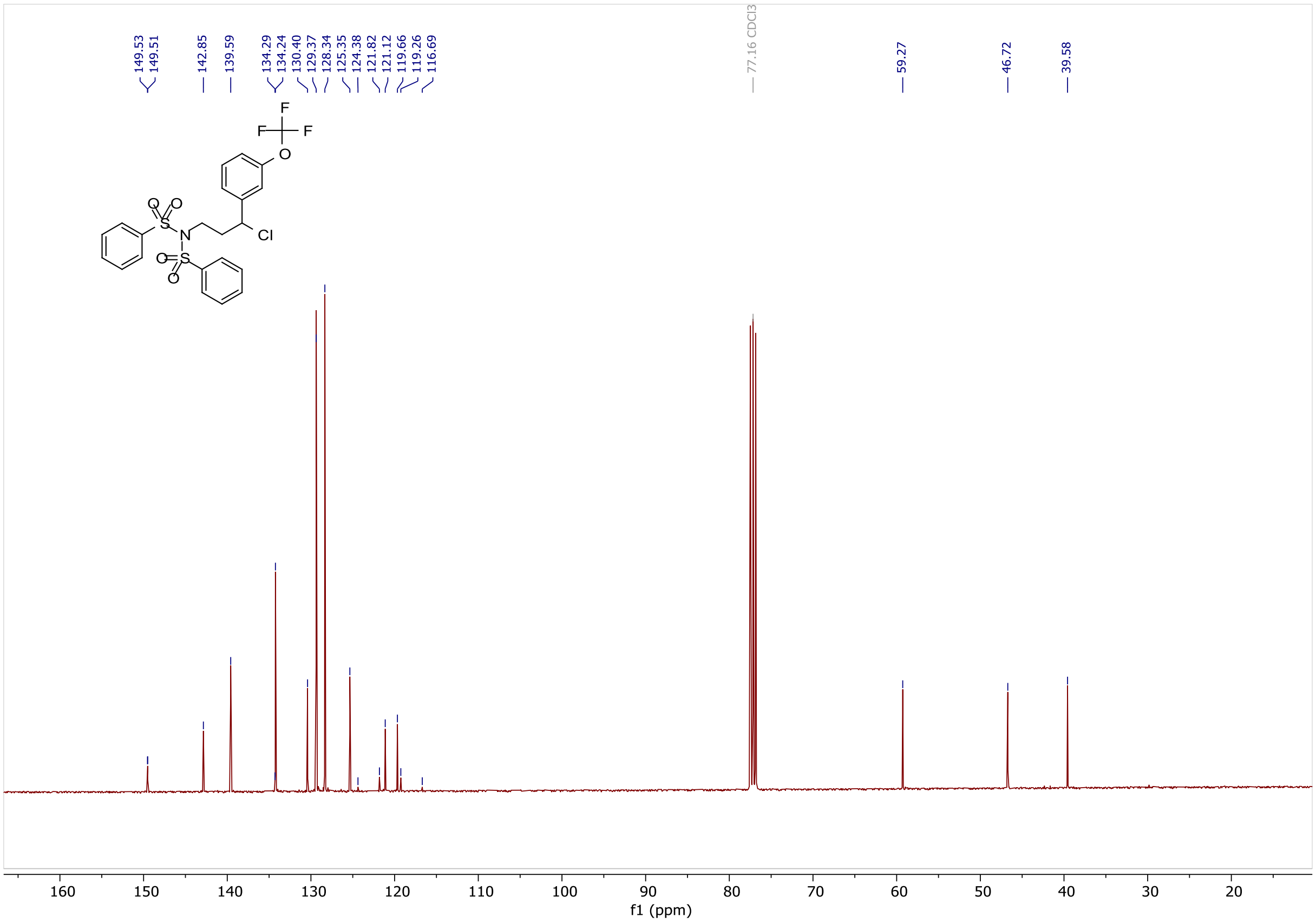

^{1}H NMR Spectrum of *p*-6k (400 MHz, $CDCl_3$)

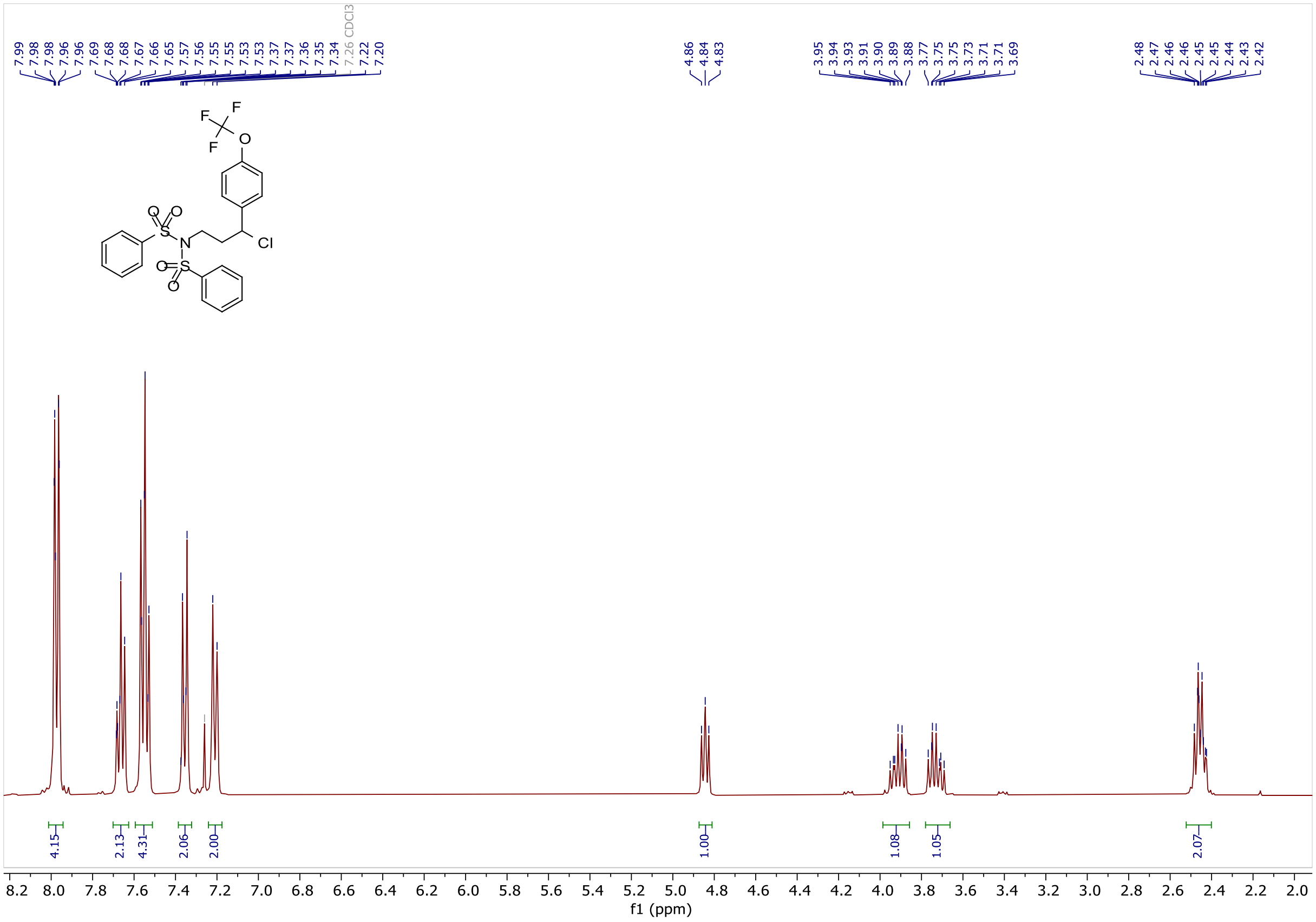

^{19}F NMR Spectrum of *p*-6k (376 MHz, $CDCl_3$)

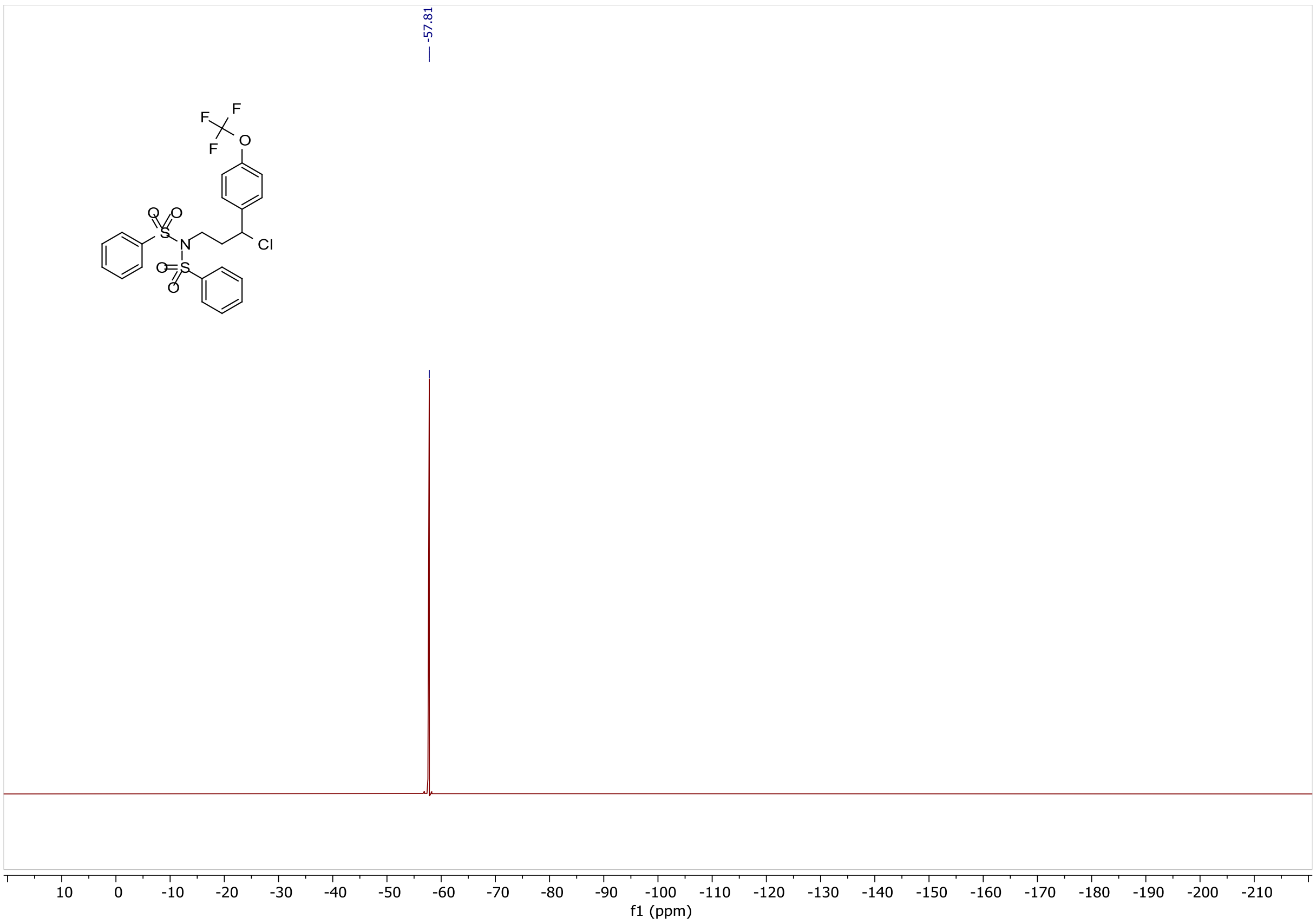

^{13}C NMR Spectrum of *p*-6k (101 MHz, $CDCl_3$)

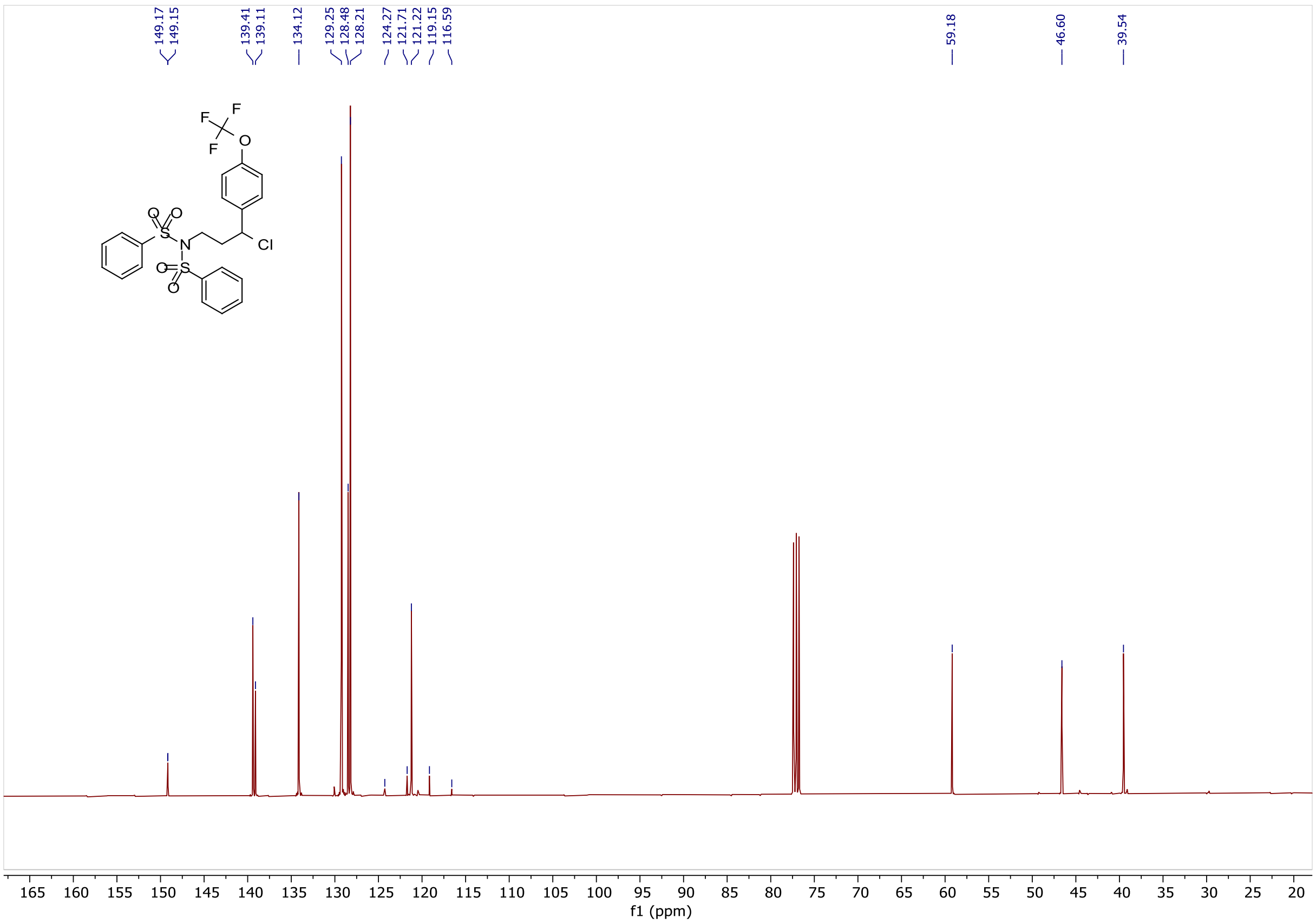

^{1}H NMR Spectrum of *o*-6l (400 MHz, $CDCl_3$)

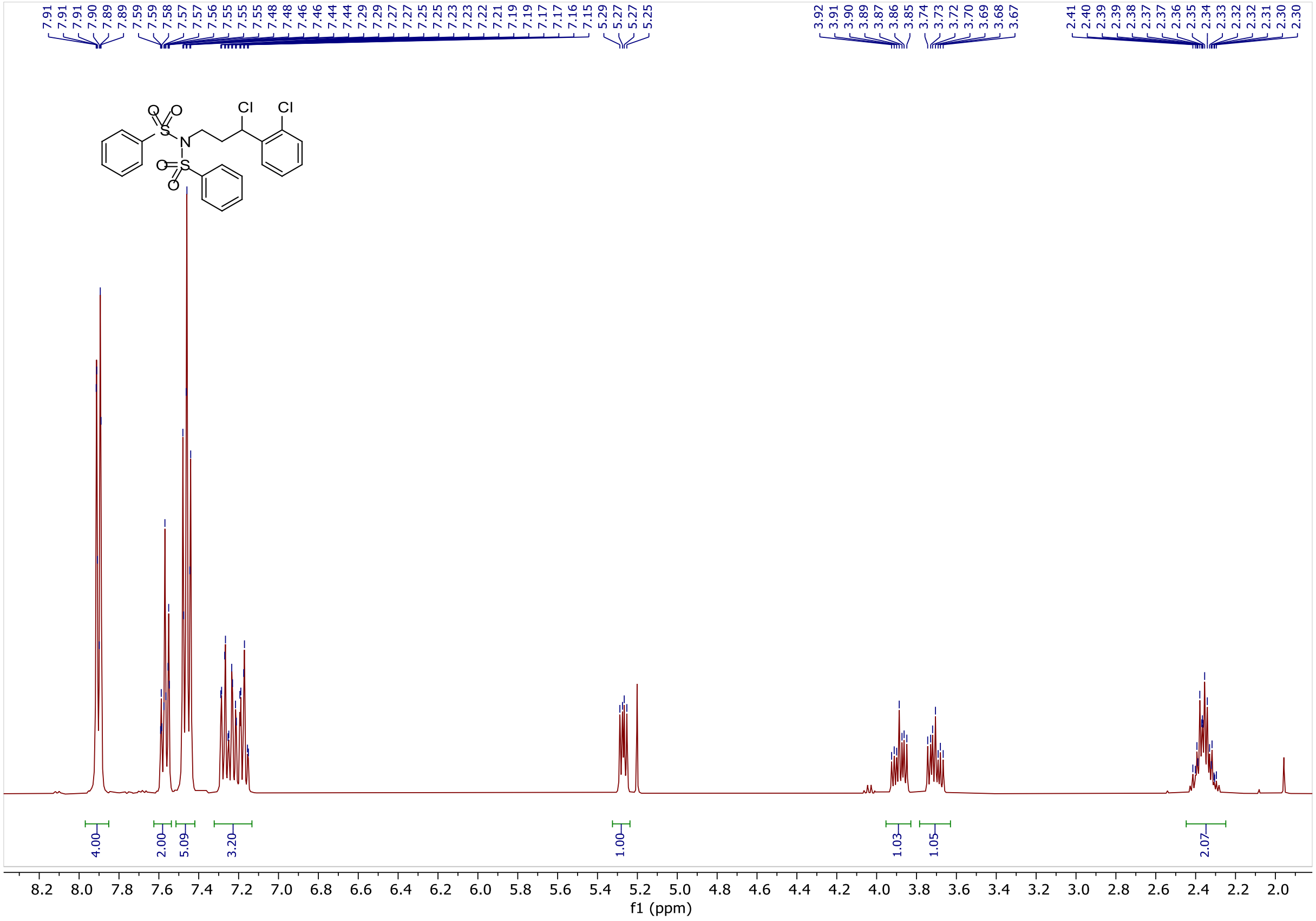

^{13}C NMR Spectrum of *o*-6l (101 MHz, $CDCl_3$)

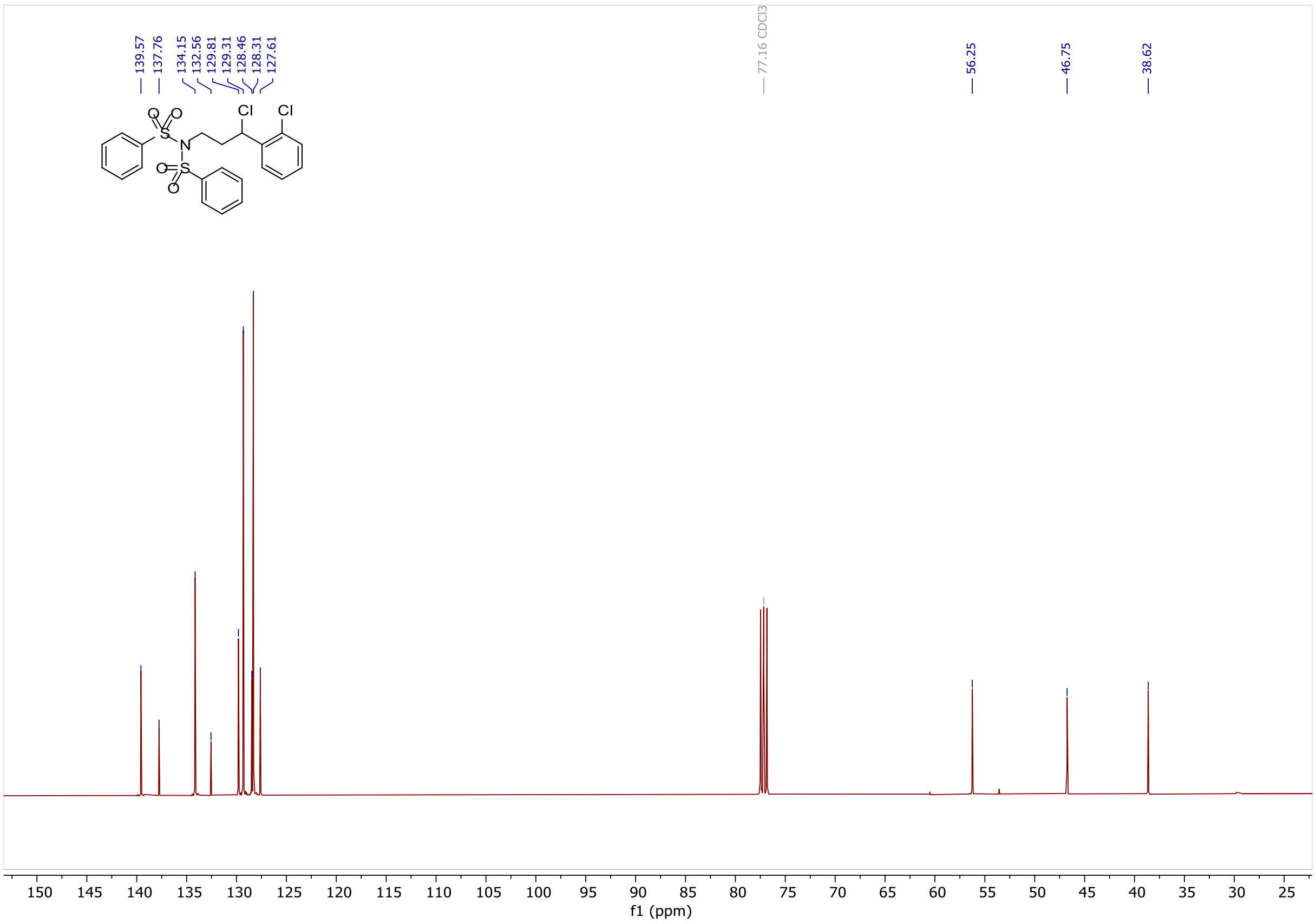

^{1}H NMR Spectrum of *m*-6l (400 MHz, $CDCl_3$)

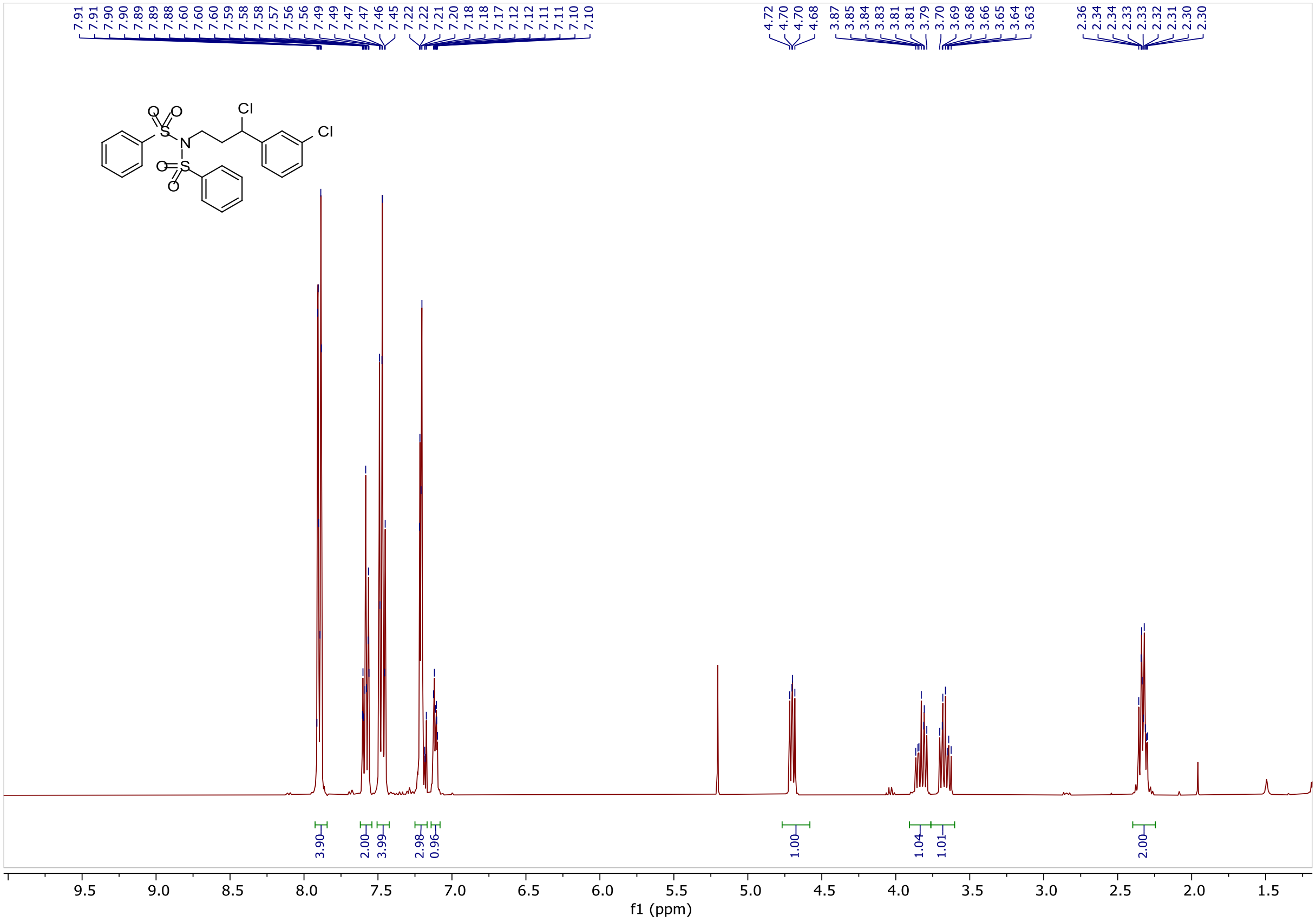

^{13}C NMR Spectrum of *m*-6l (101 MHz, $CDCl_3$)

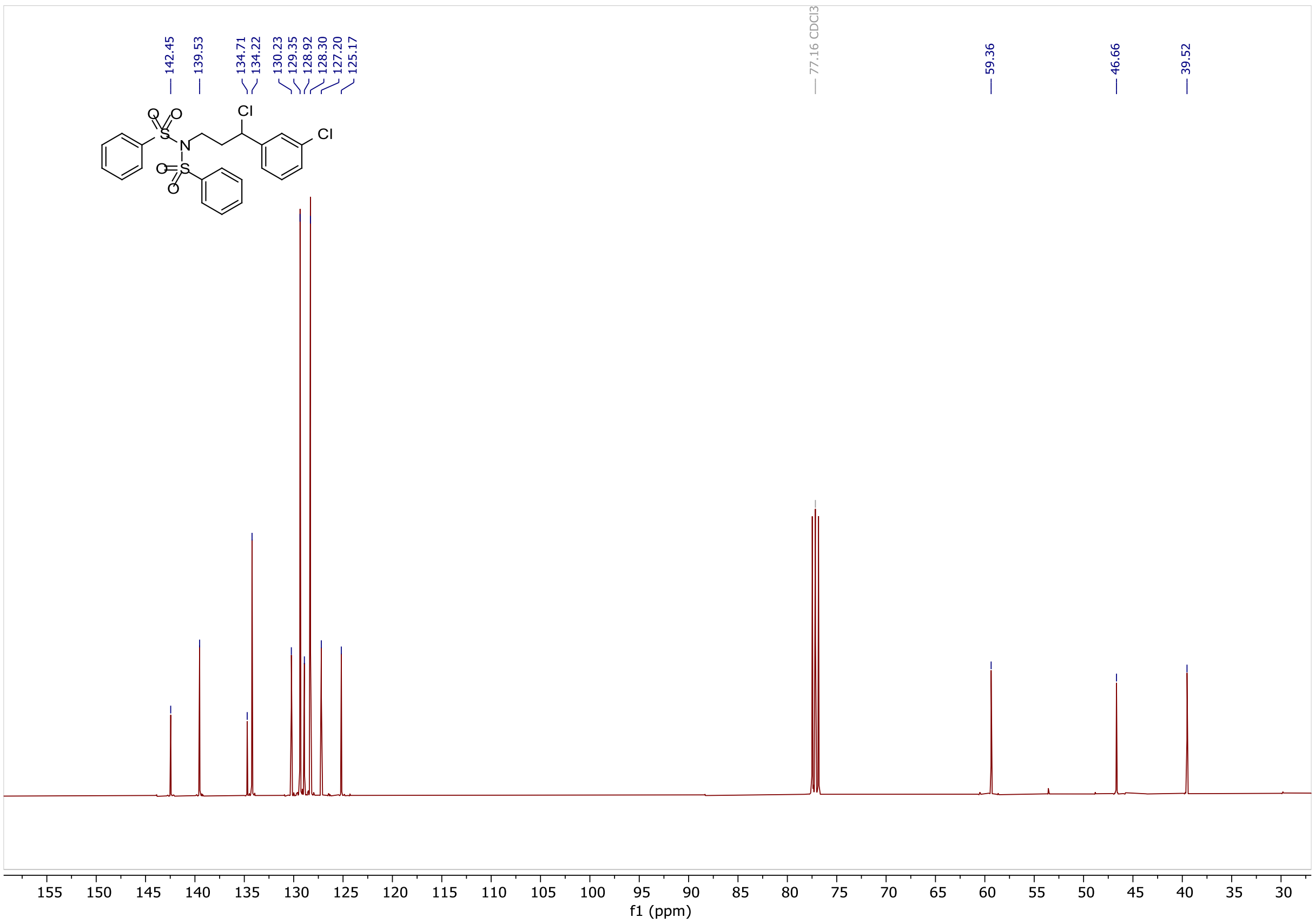

^{1}H NMR Spectrum of *p*-6l (400 MHz, CDCl$_3$)

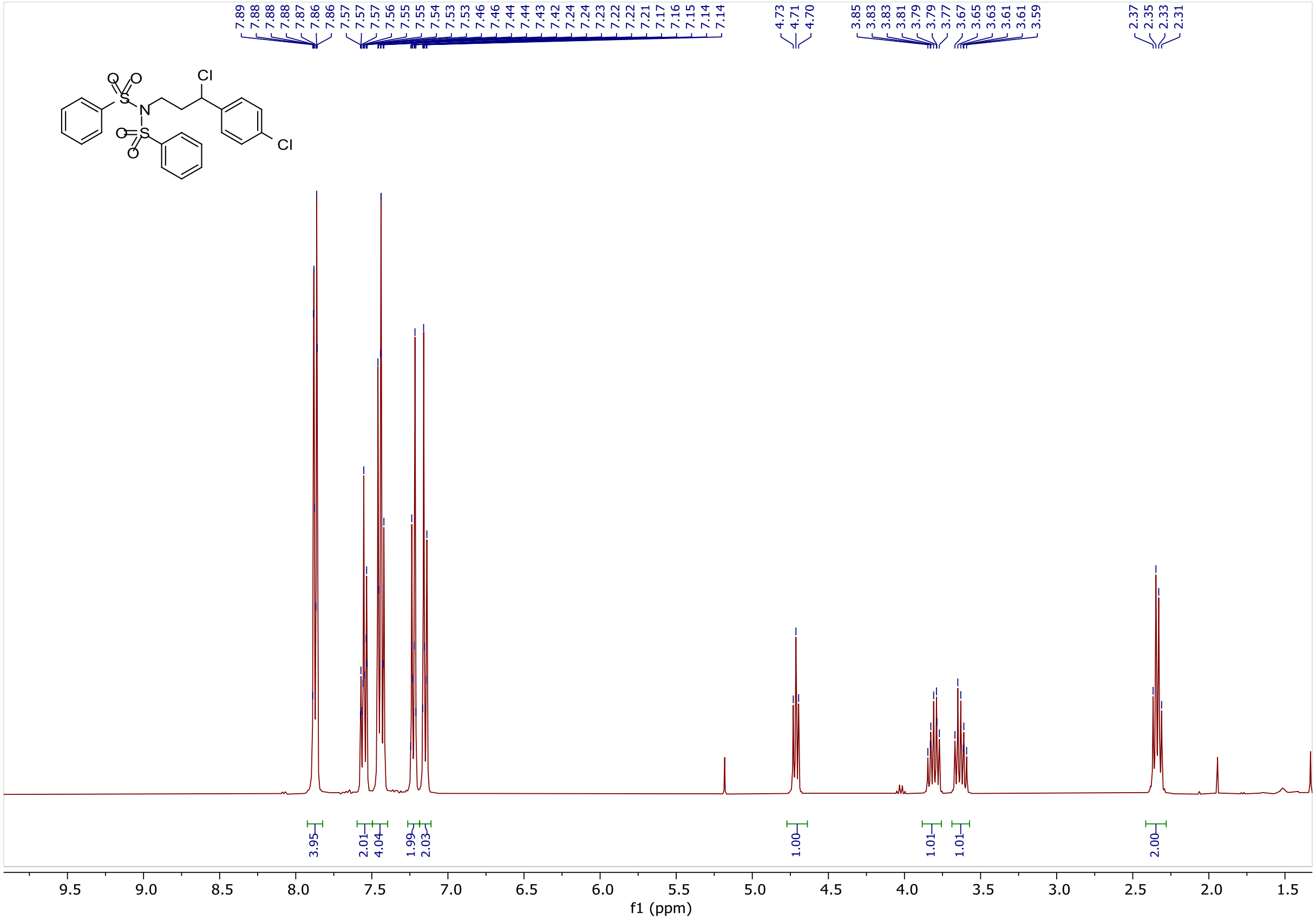

^{13}C NMR Spectrum of *p*-6l (101 MHz, CDCl$_3$)

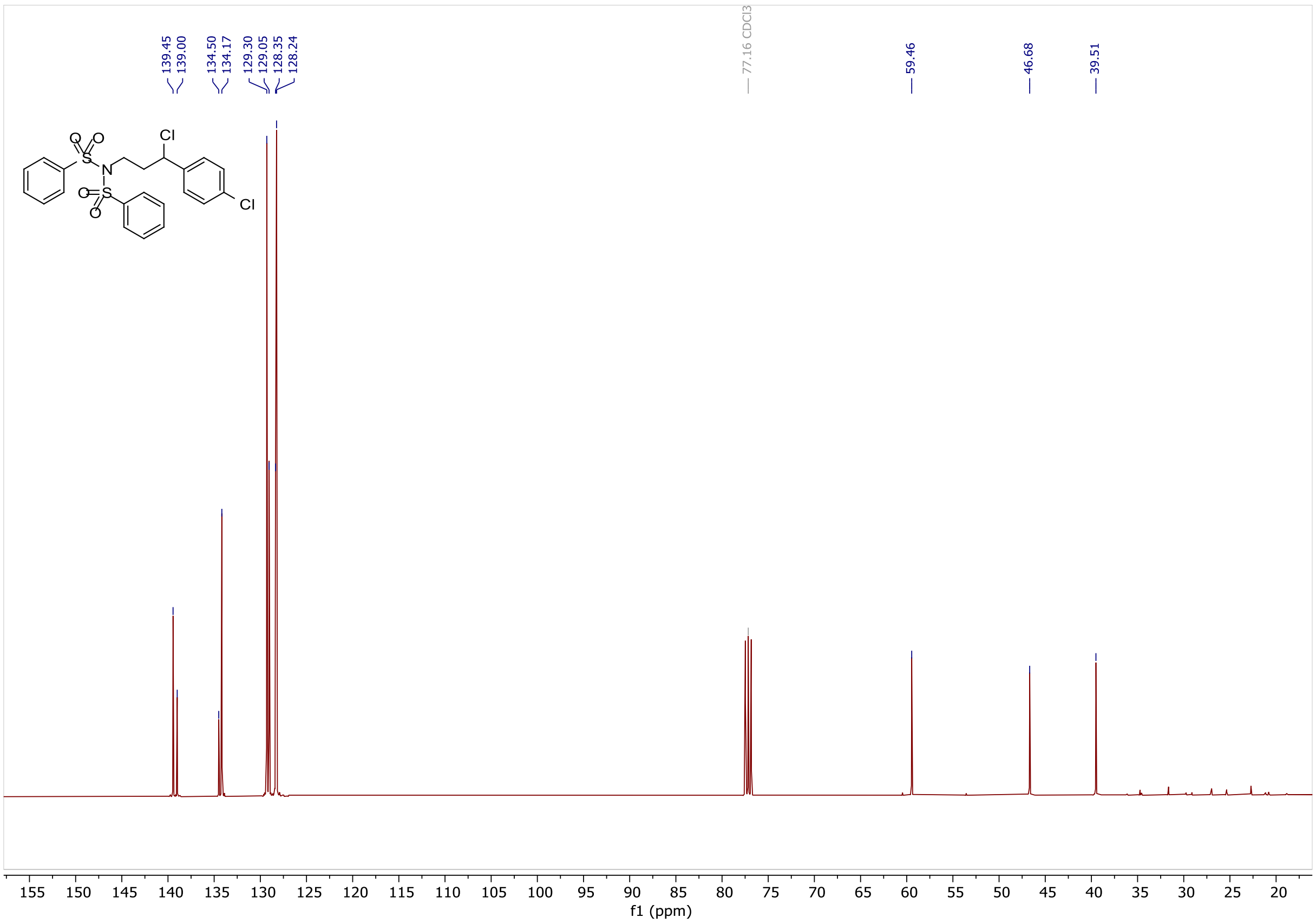

^{1}H NMR Spectrum of *o*-6m (400 MHz, $CDCl_3$)

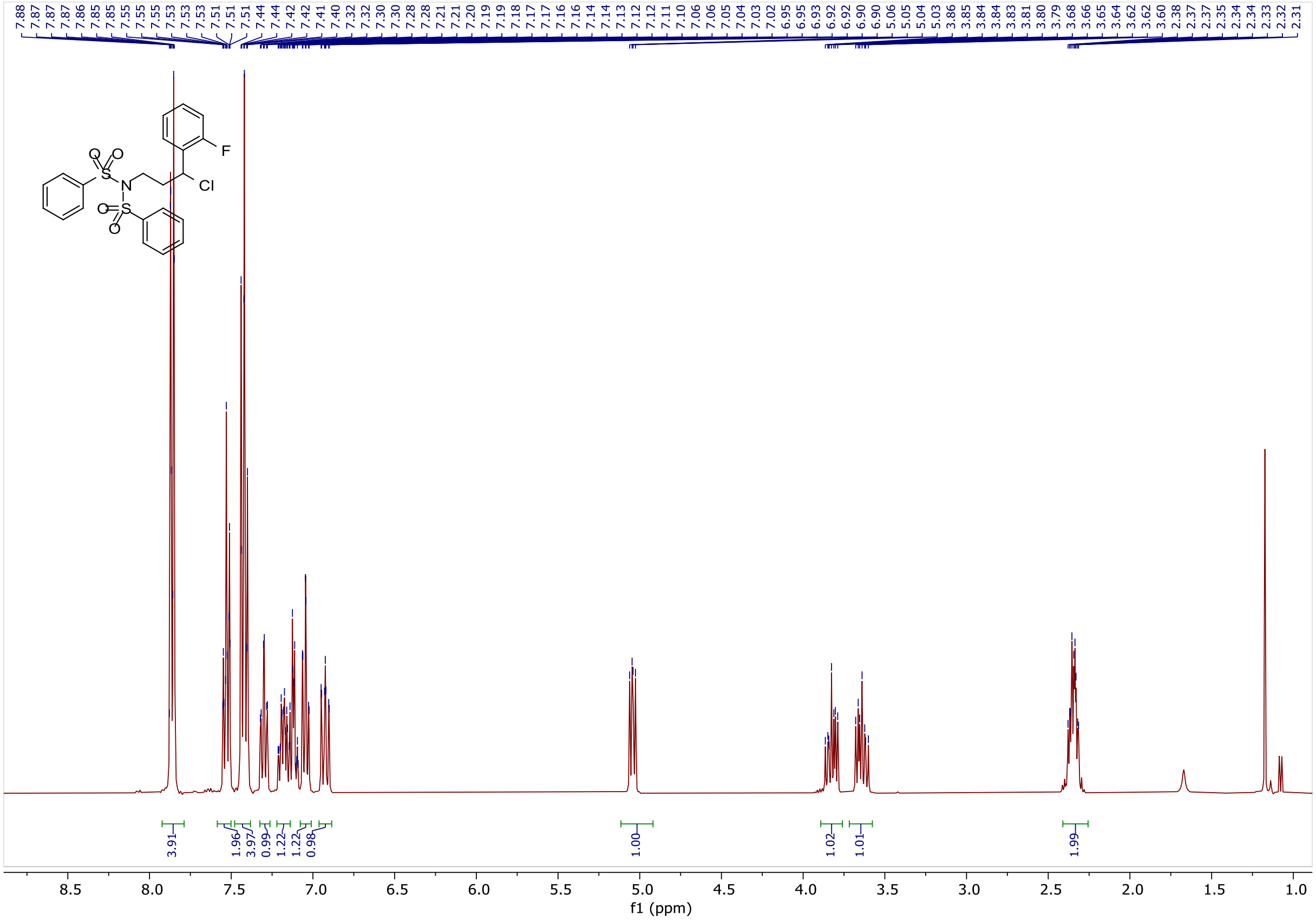

^{19}F NMR Spectrum of *o*-6m (376 MHz, $CDCl_3$)

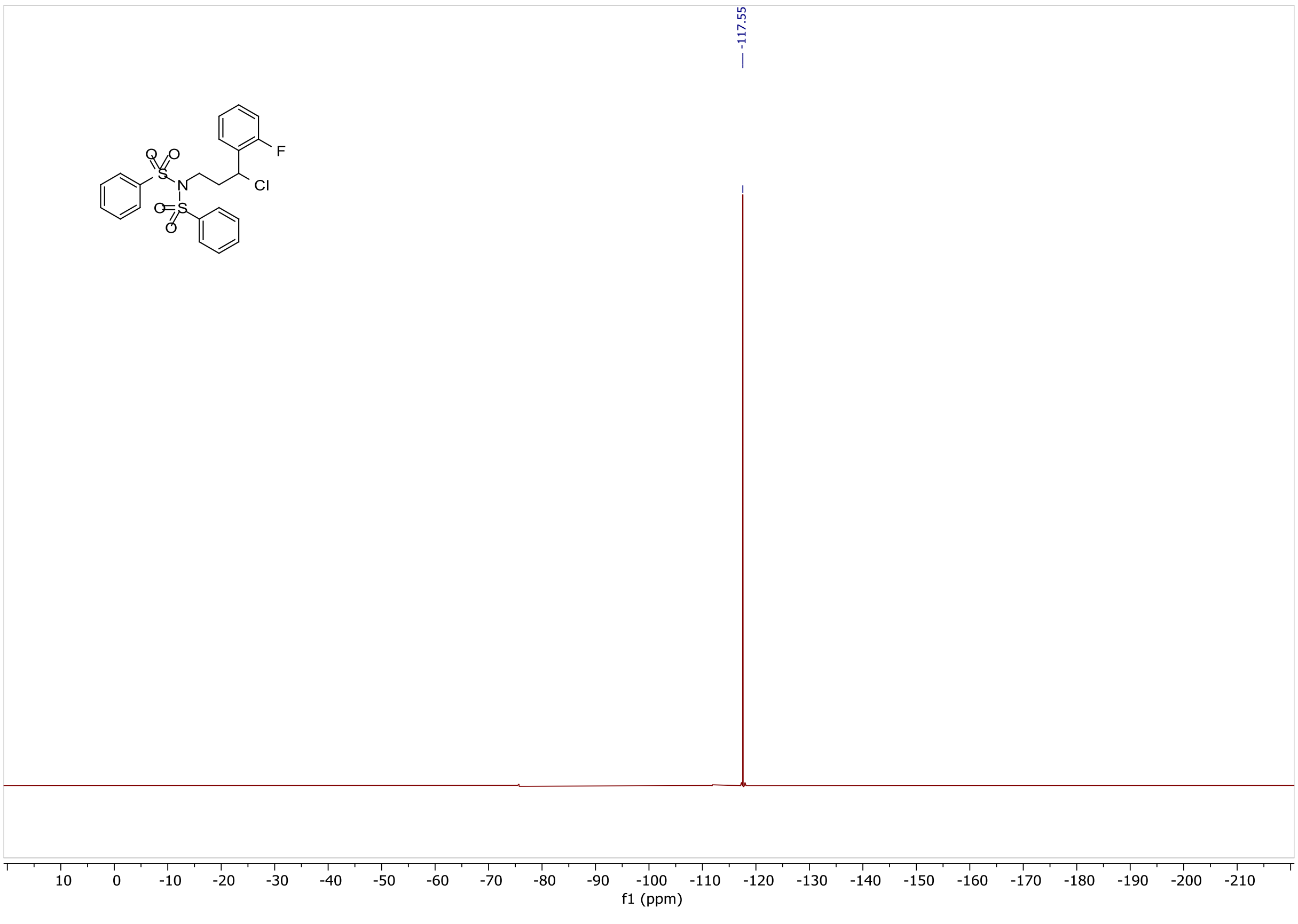

^{13}C NMR Spectrum of *o*-6m (101 MHz, $CDCl_3$)

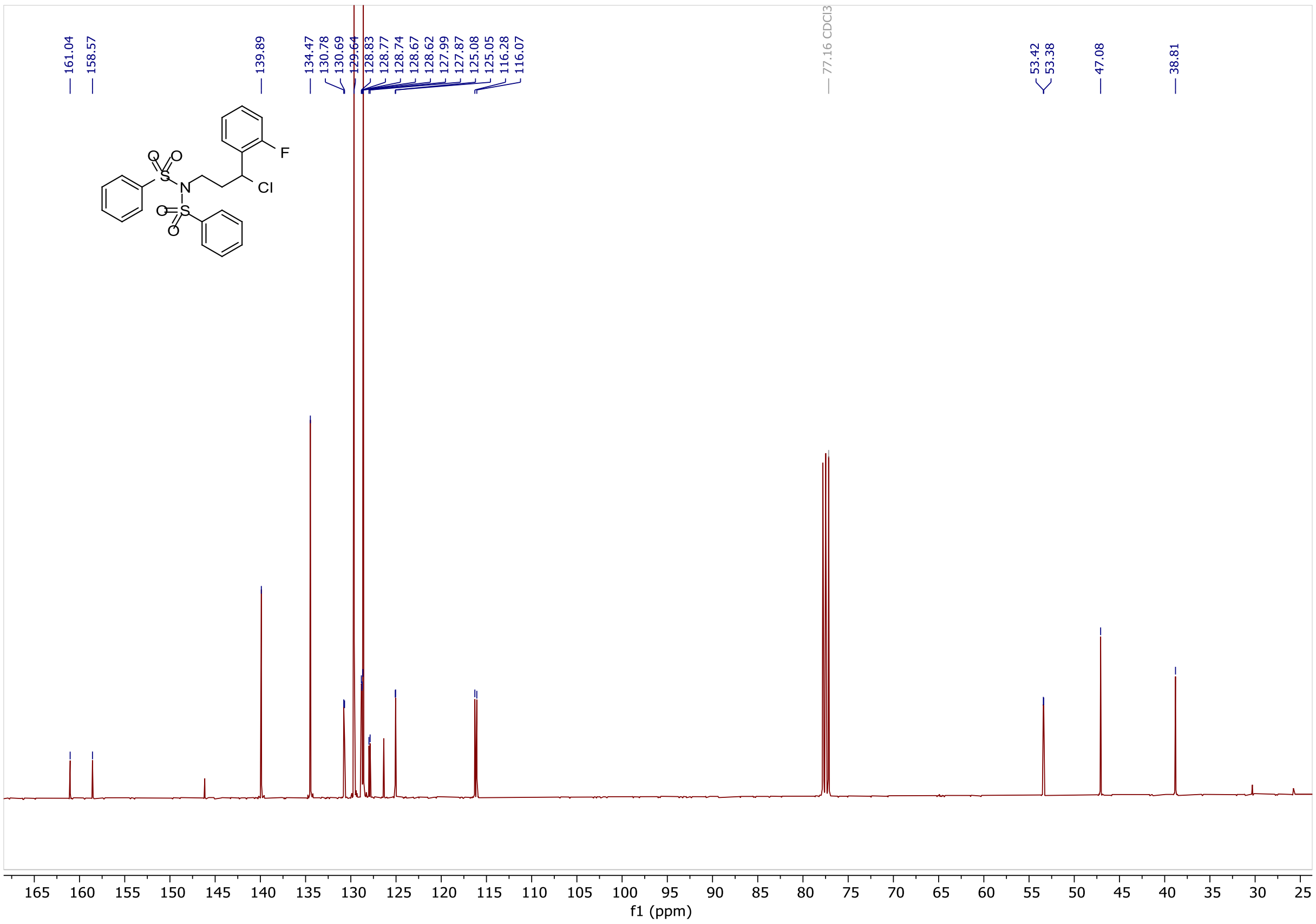

^{1}H NMR Spectrum of *m*-6m (400 MHz, CDCl$_3$)

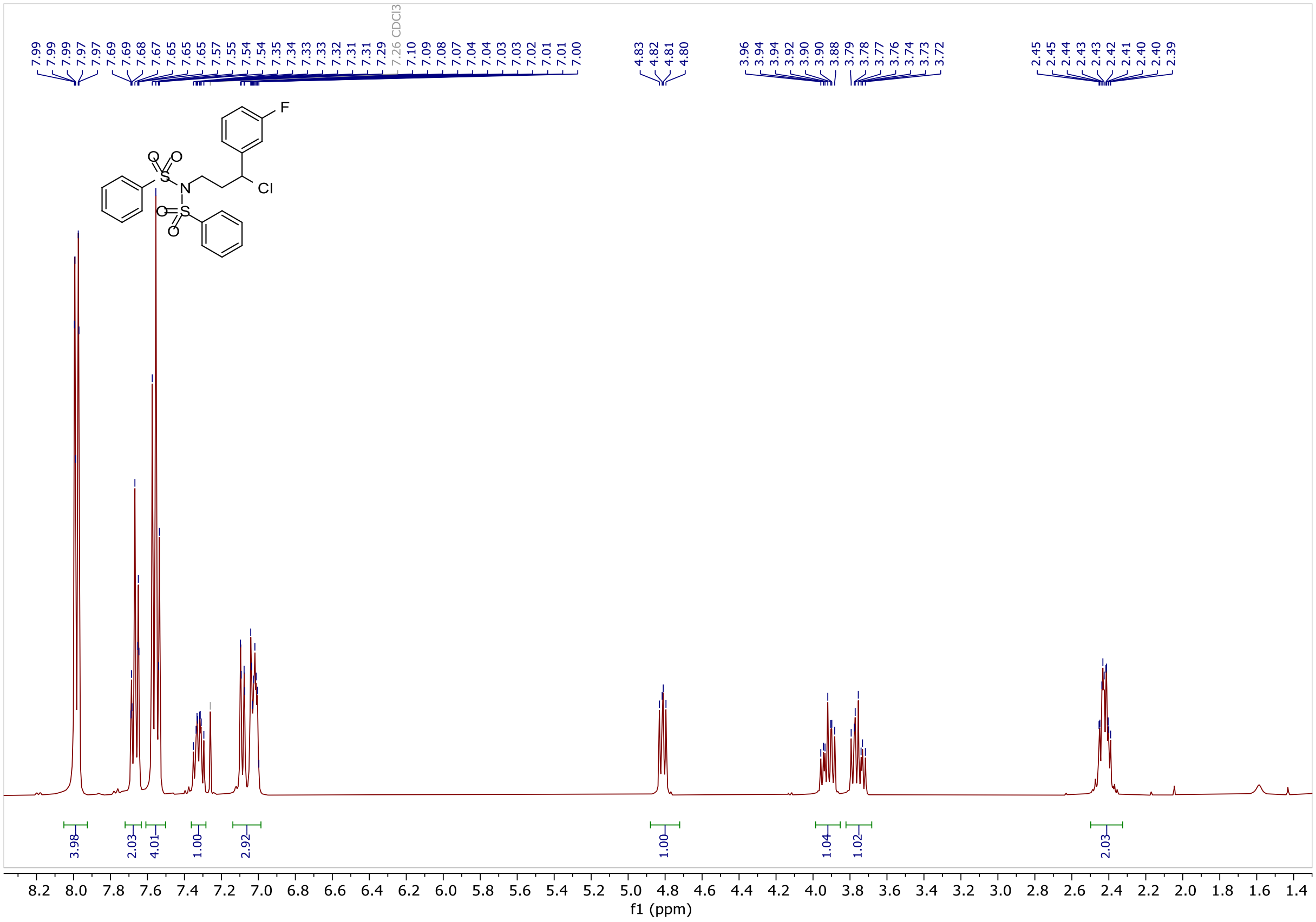

^{19}F NMR Spectrum of *m*-6m (376 MHz, CDCl$_3$)

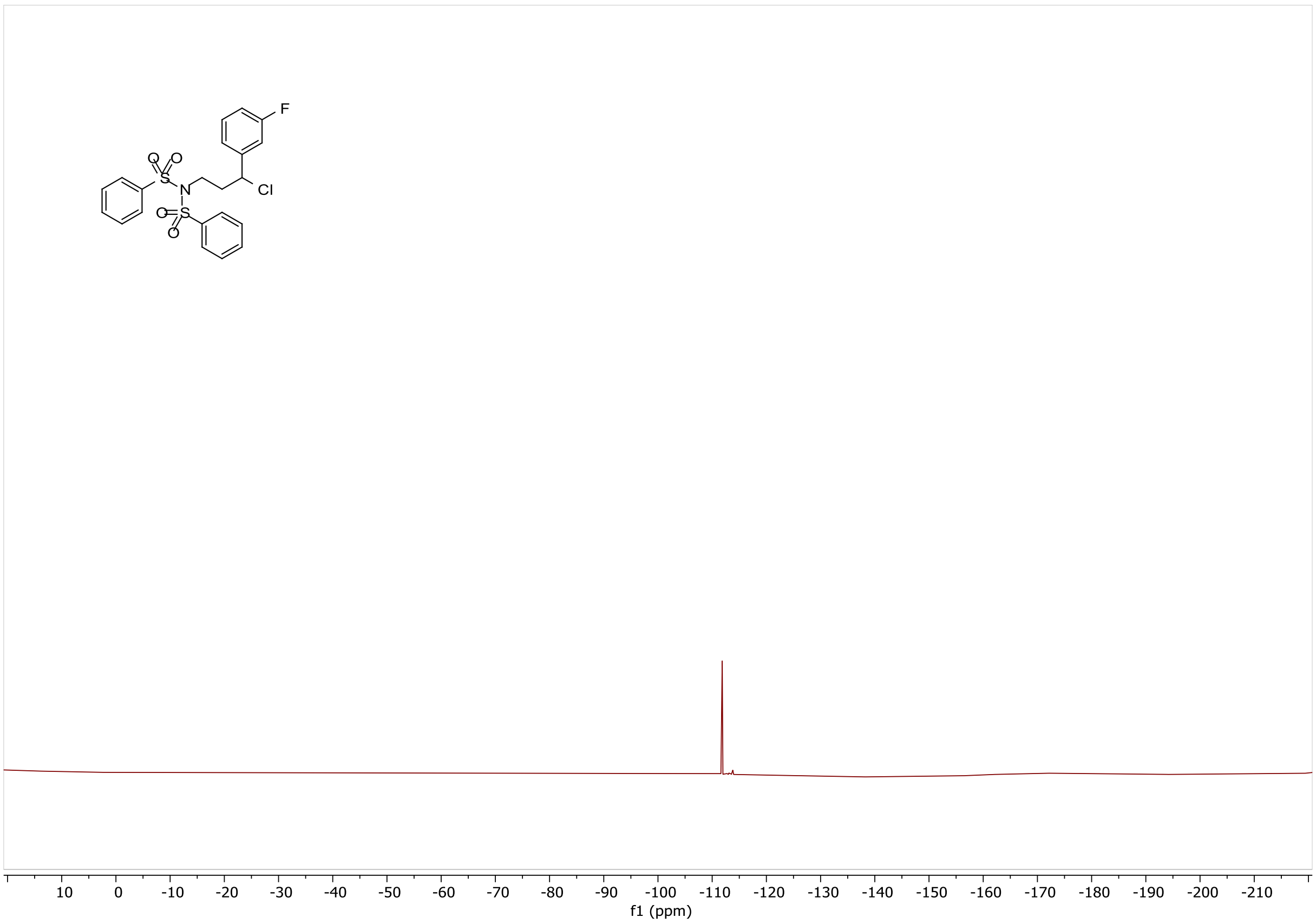

^{13}C NMR Spectrum of *m*-6m (101 MHz, $CDCl_3$)

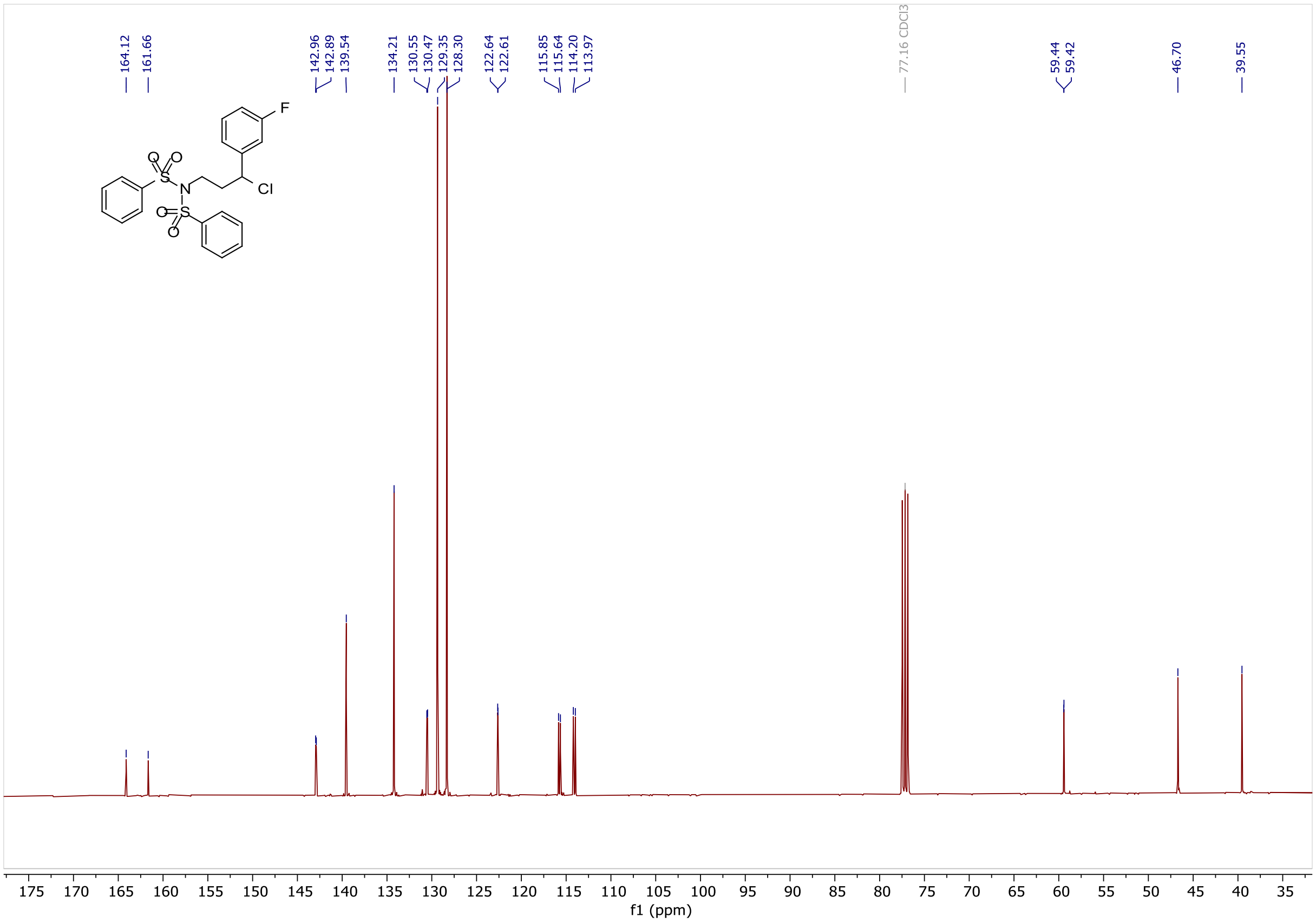

^{1}H NMR Spectrum of *p*-6m (400 MHz, $CDCl_3$)

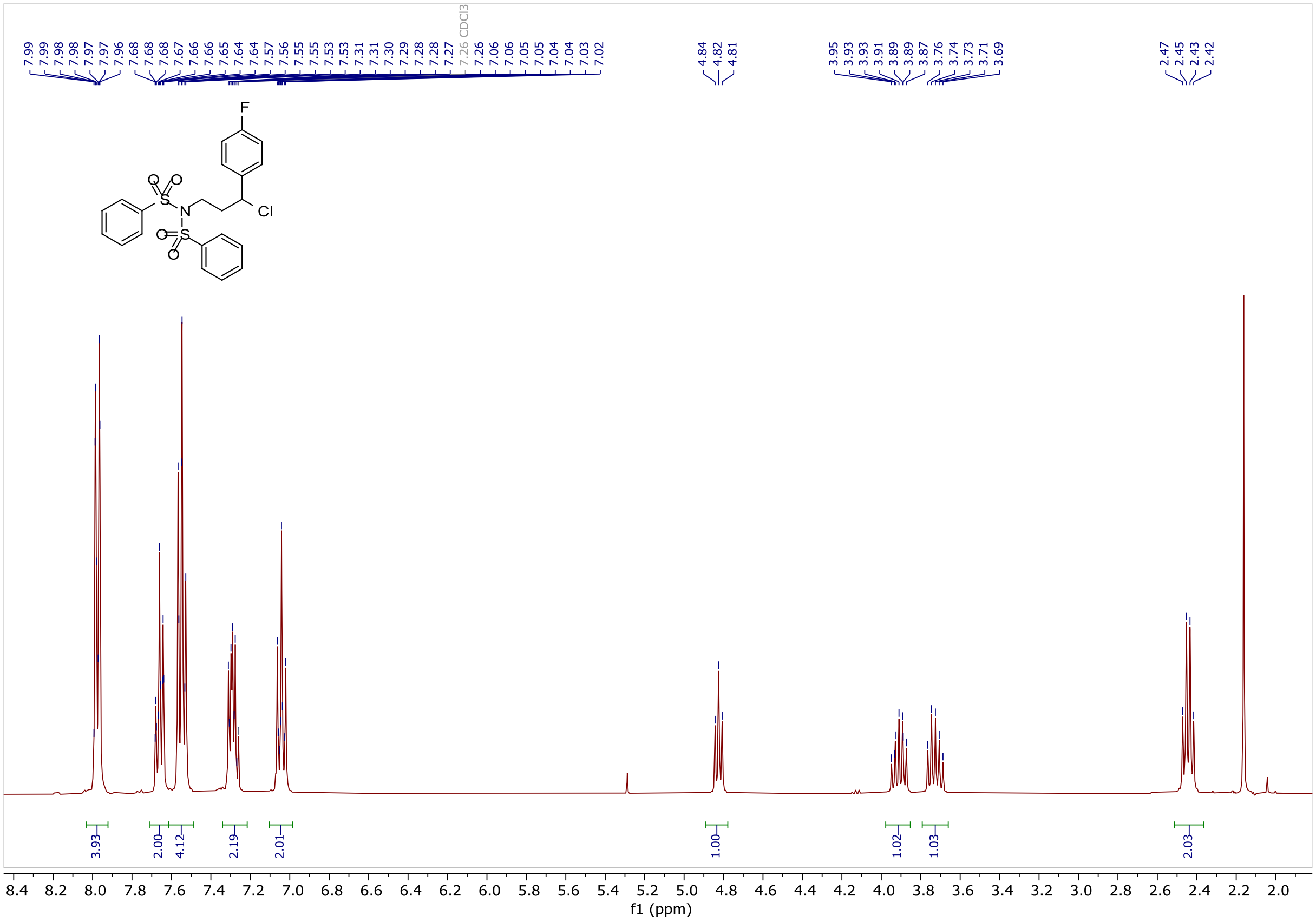

^{19}F NMR Spectrum of *p*-6m (376 MHz, $CDCl_3$)

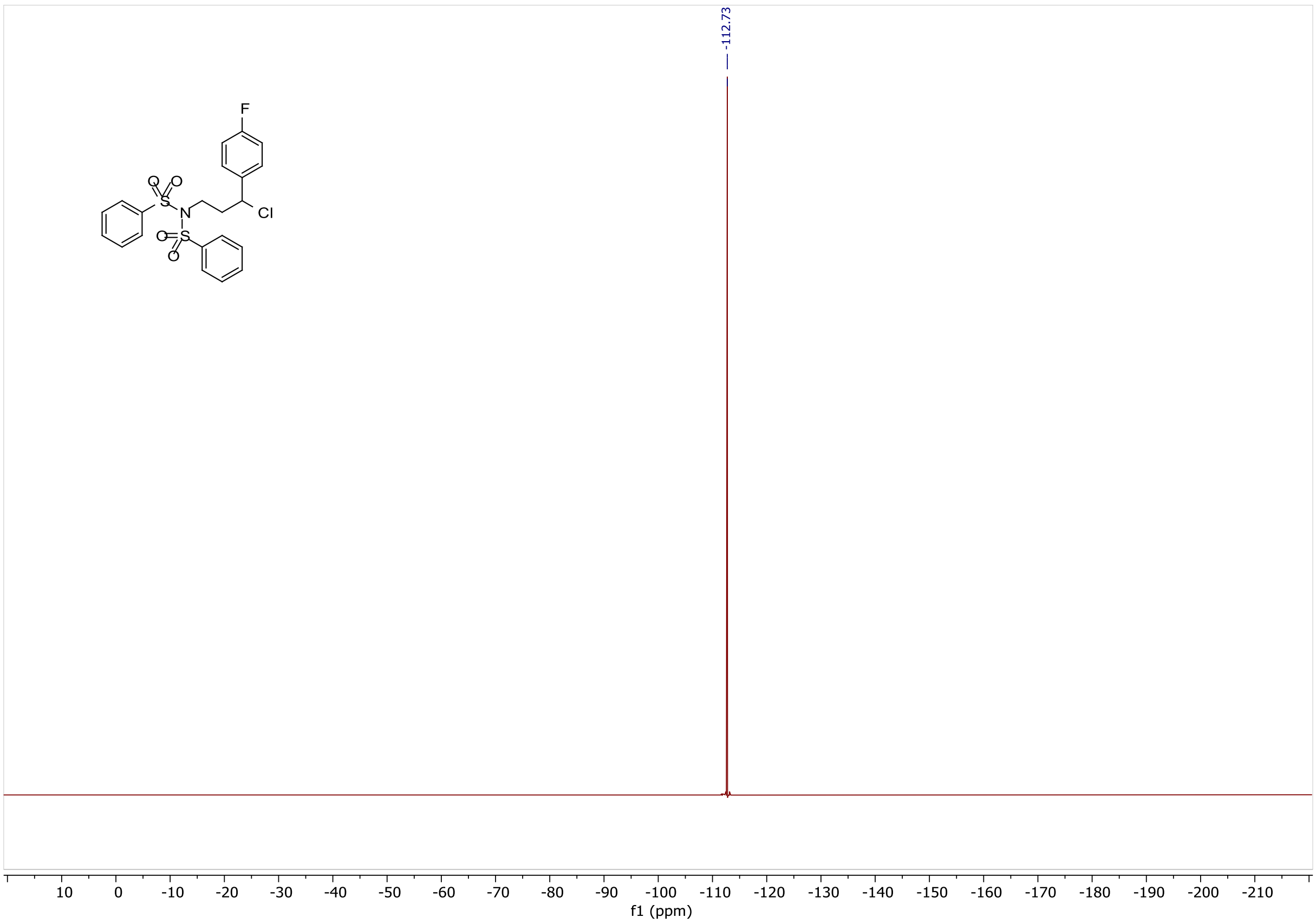

^{13}C NMR Spectrum of *p*-6m (101 MHz, $CDCl_3$)

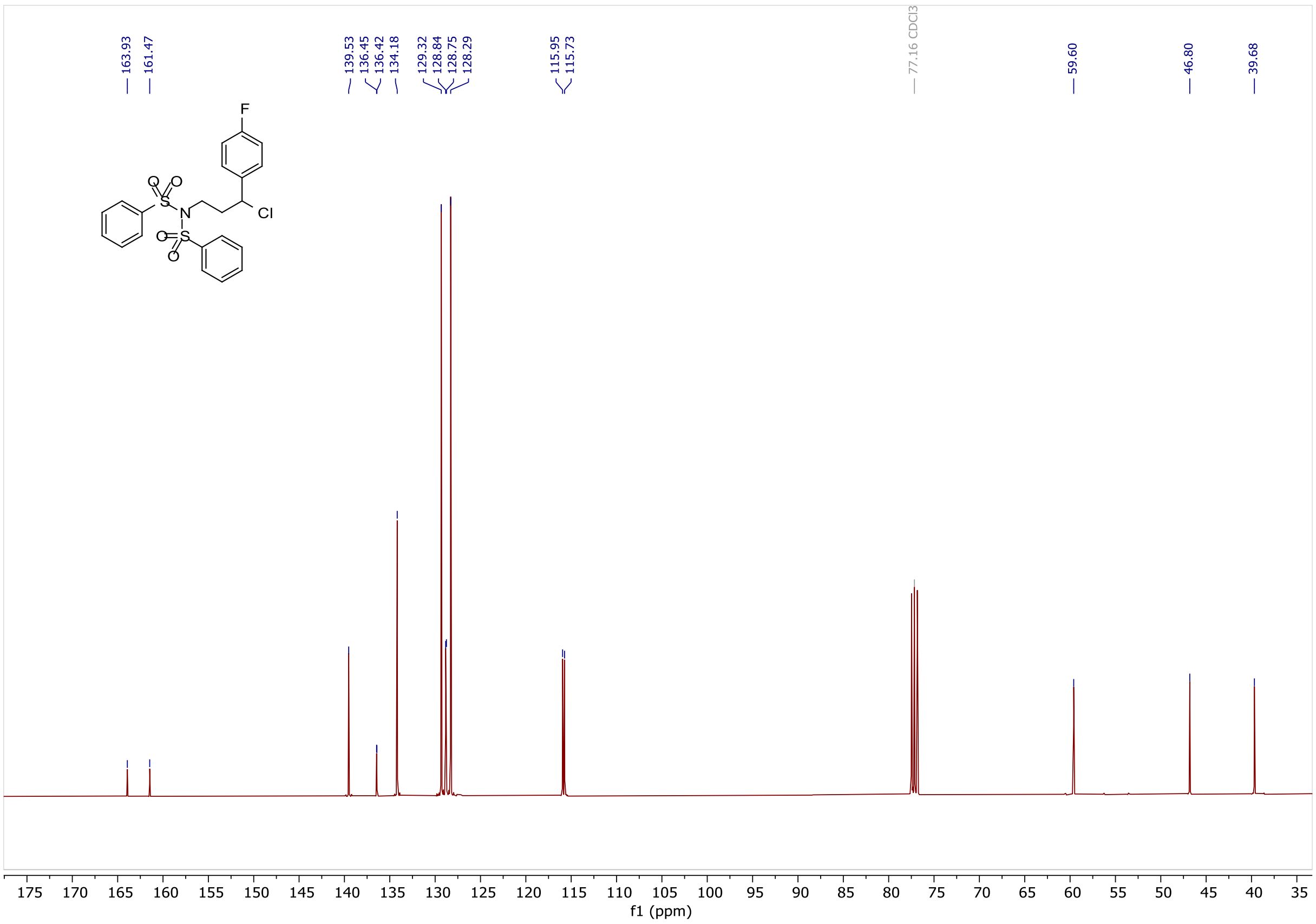

^{1}H NMR Spectrum of 6n (400 MHz, DMSO-d_6)

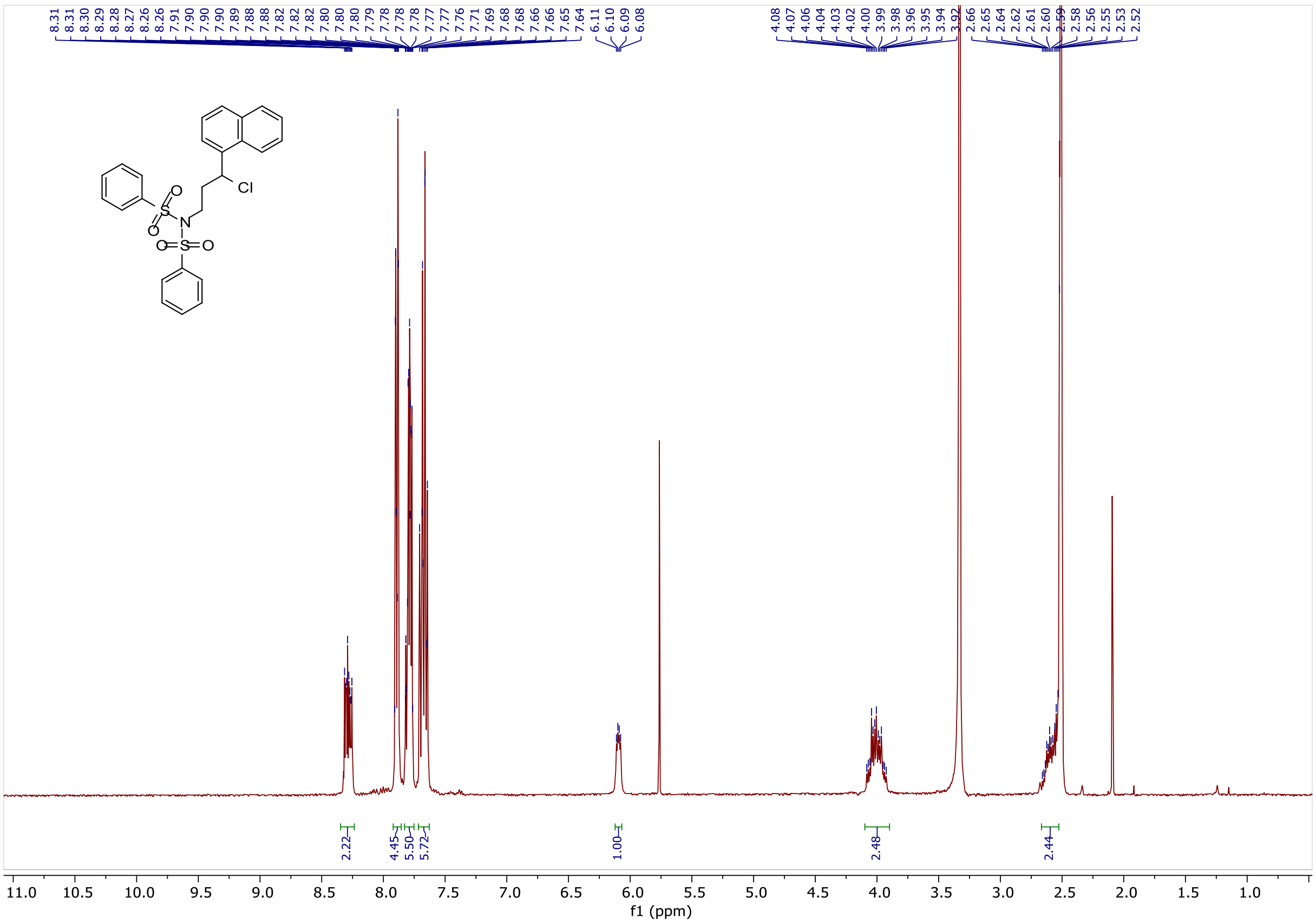

^{13}C NMR Spectrum of 6n (101 MHz, $CDCl_3$)

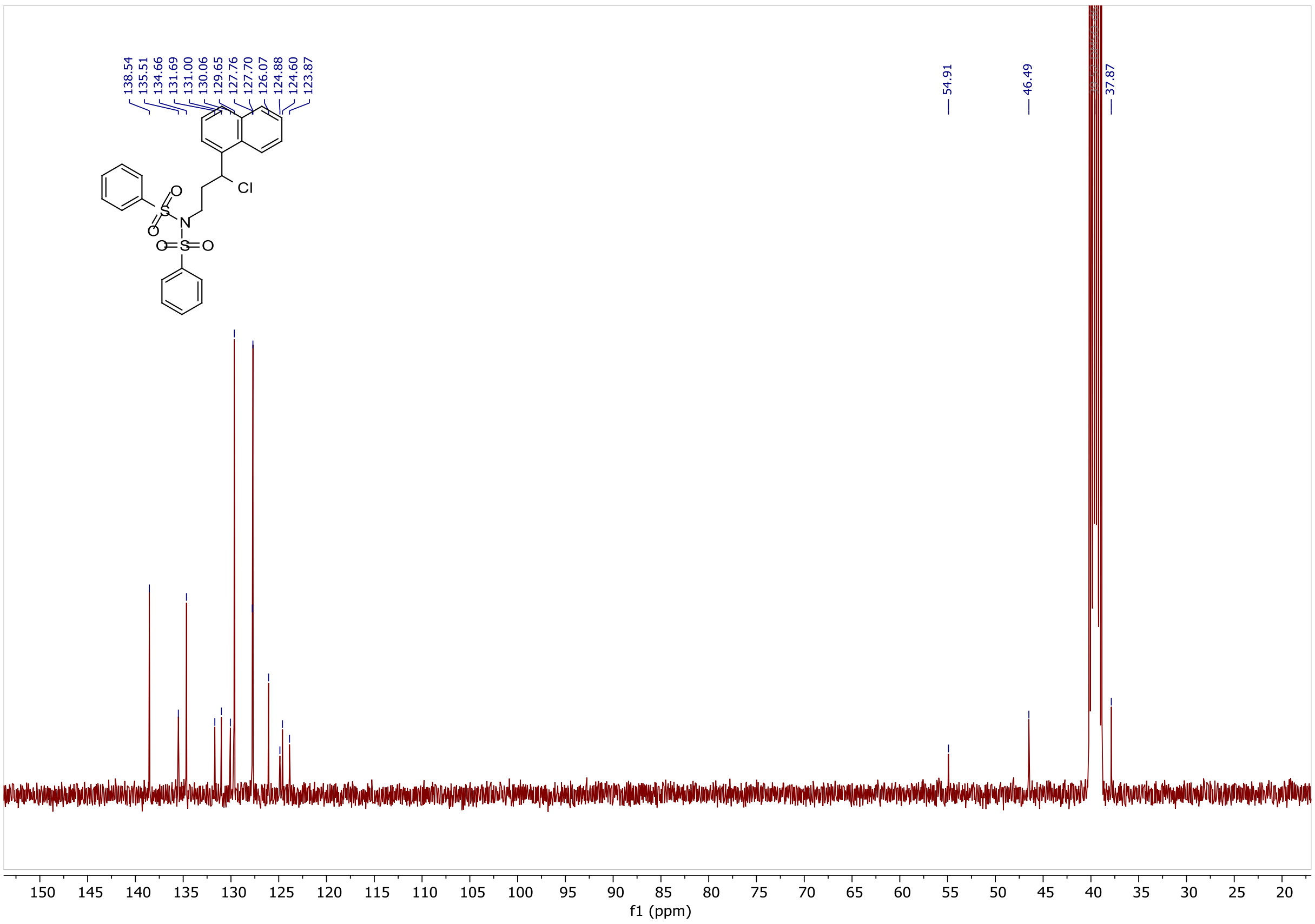

^{1}H **NMR Spectrum of 6o** (400 MHz, $CDCl_3$)

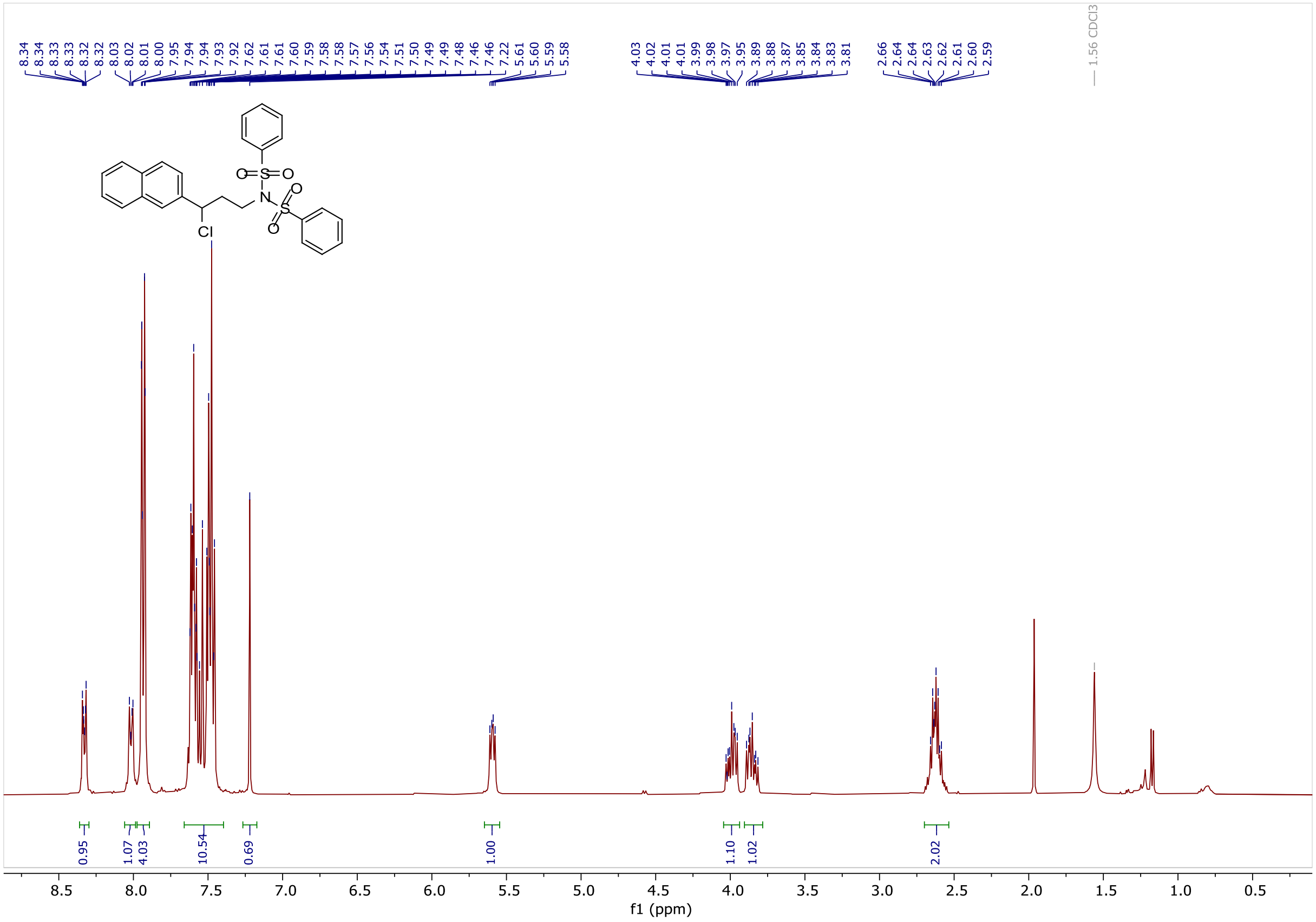

^{13}C **NMR Spectrum of 6o** (101 MHz, $CDCl_3$)

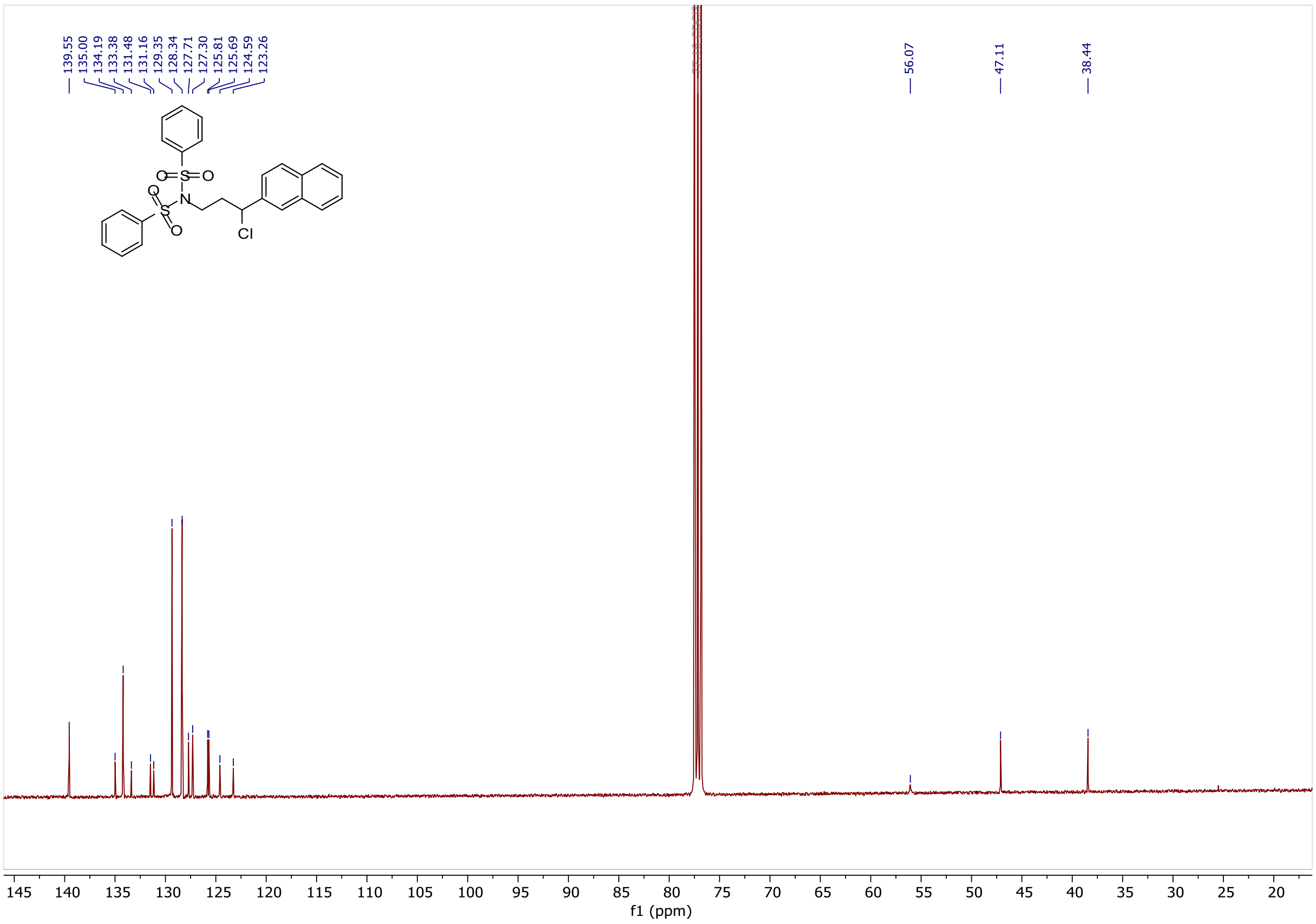

^{1}H NMR Spectrum of 6p (400 MHz, $CDCl_3$)

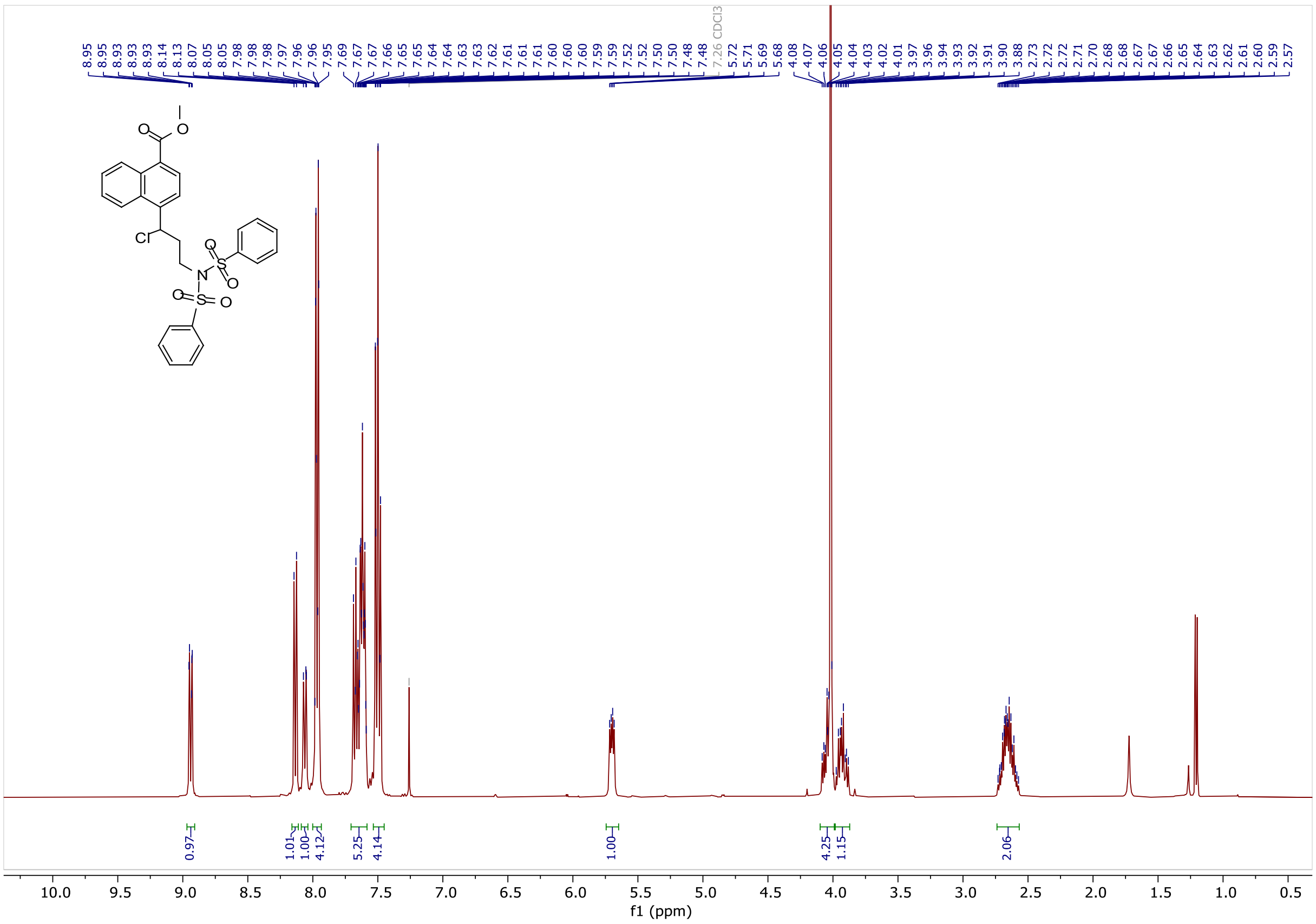

^{13}C NMR Spectrum of 6p (101 MHz, $CDCl_3$)

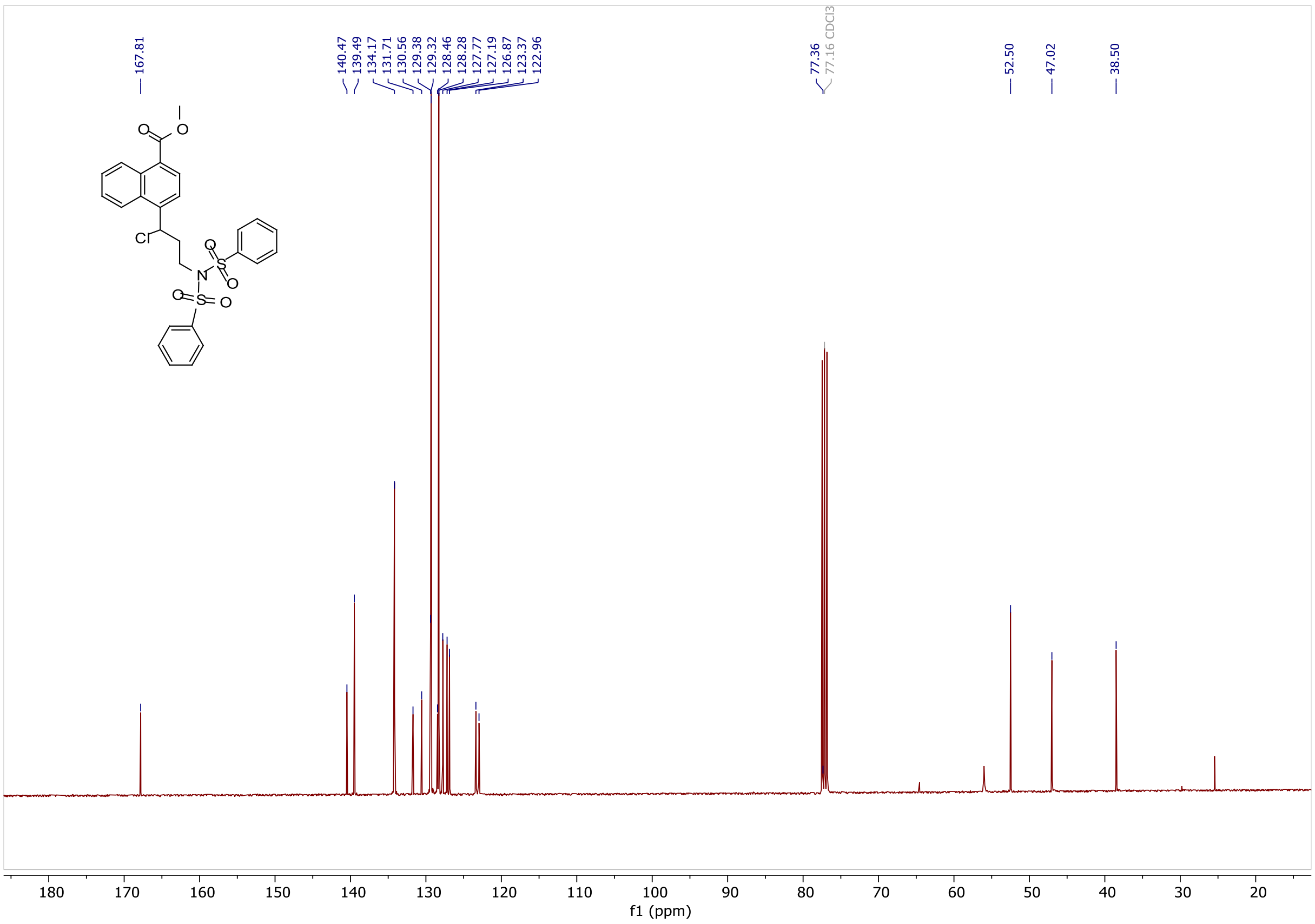

^{1}H NMR Spectrum of 6q (400 MHz, $CDCl_3$)

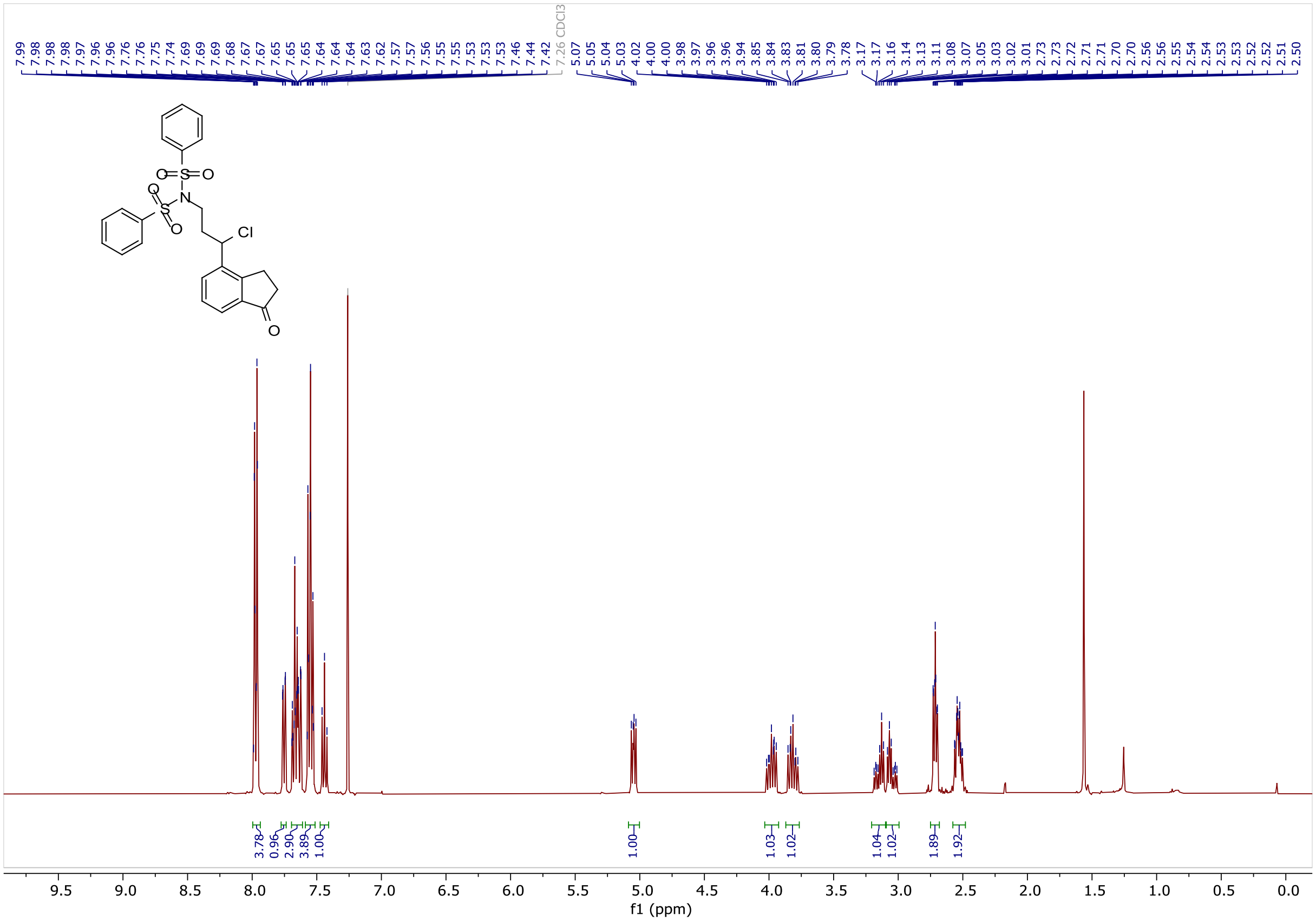

^{13}C NMR Spectrum of 6q (101 MHz, $CDCl_3$)

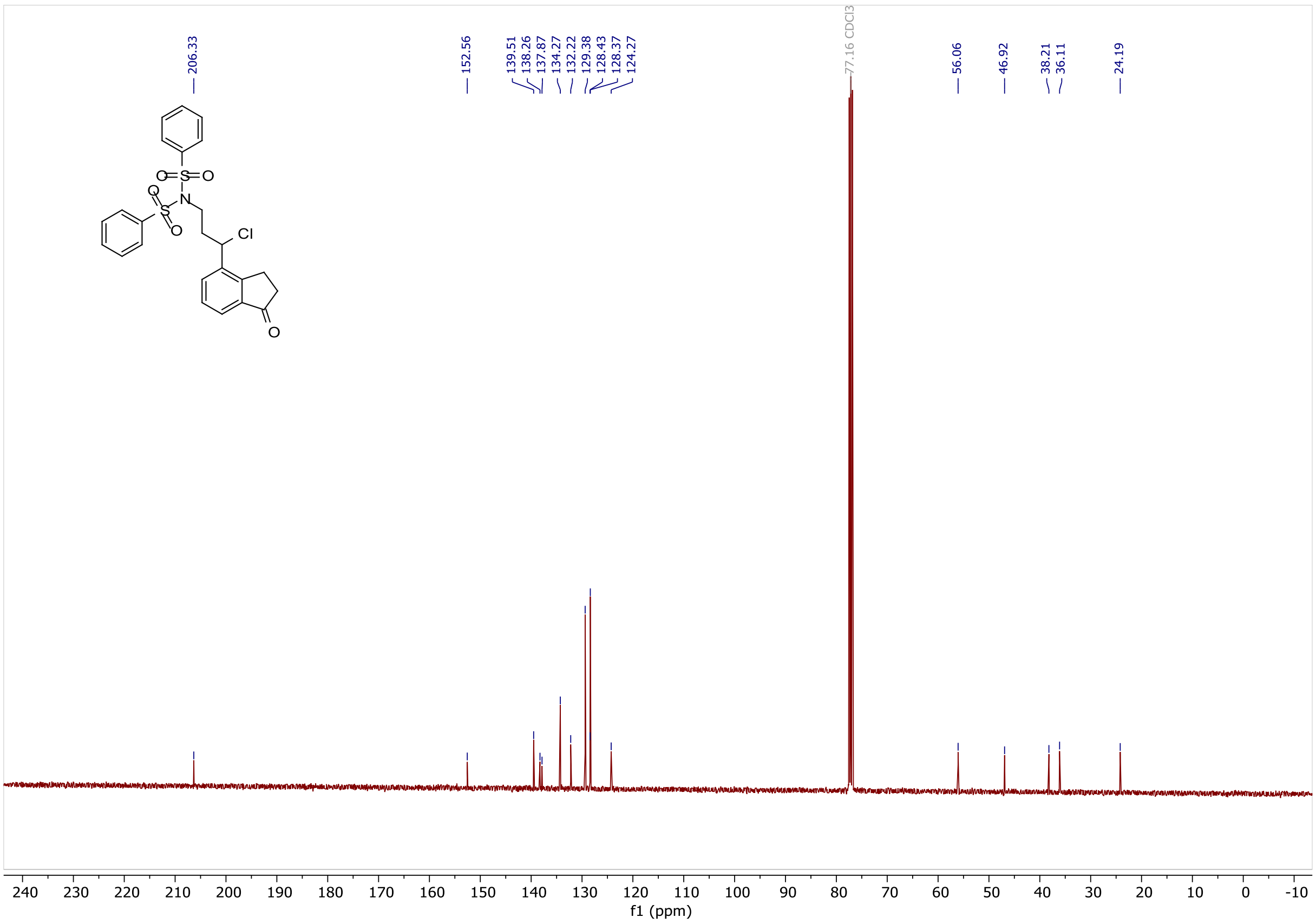

^{1}H NMR Spectrum of 6r (400 MHz, $CDCl_3$)

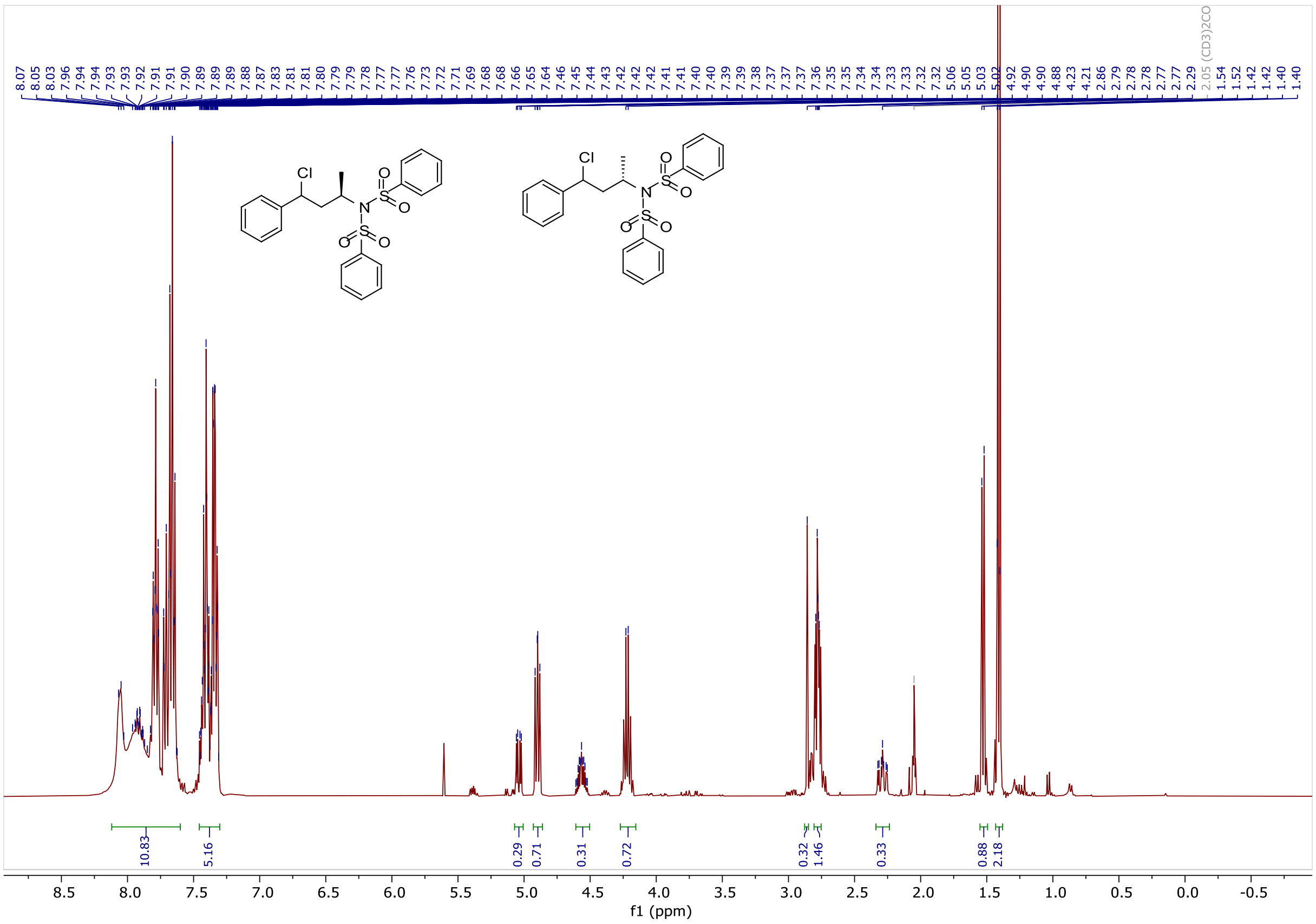

^{13}C NMR Spectrum of 6r (101 MHz, $CDCl_3$)

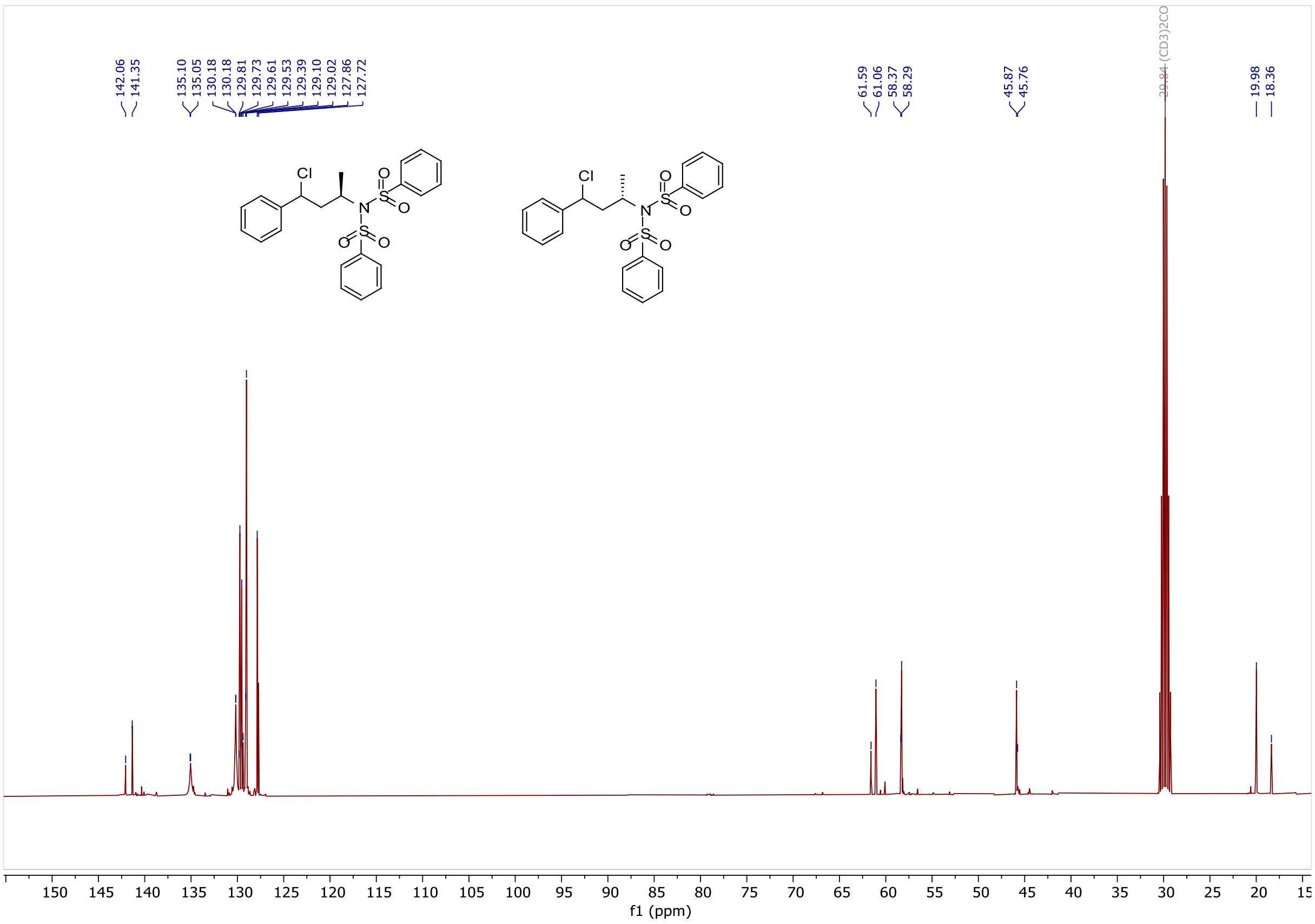

^{1}H NMR Spectrum of 6s (400 MHz, $CDCl_3$)

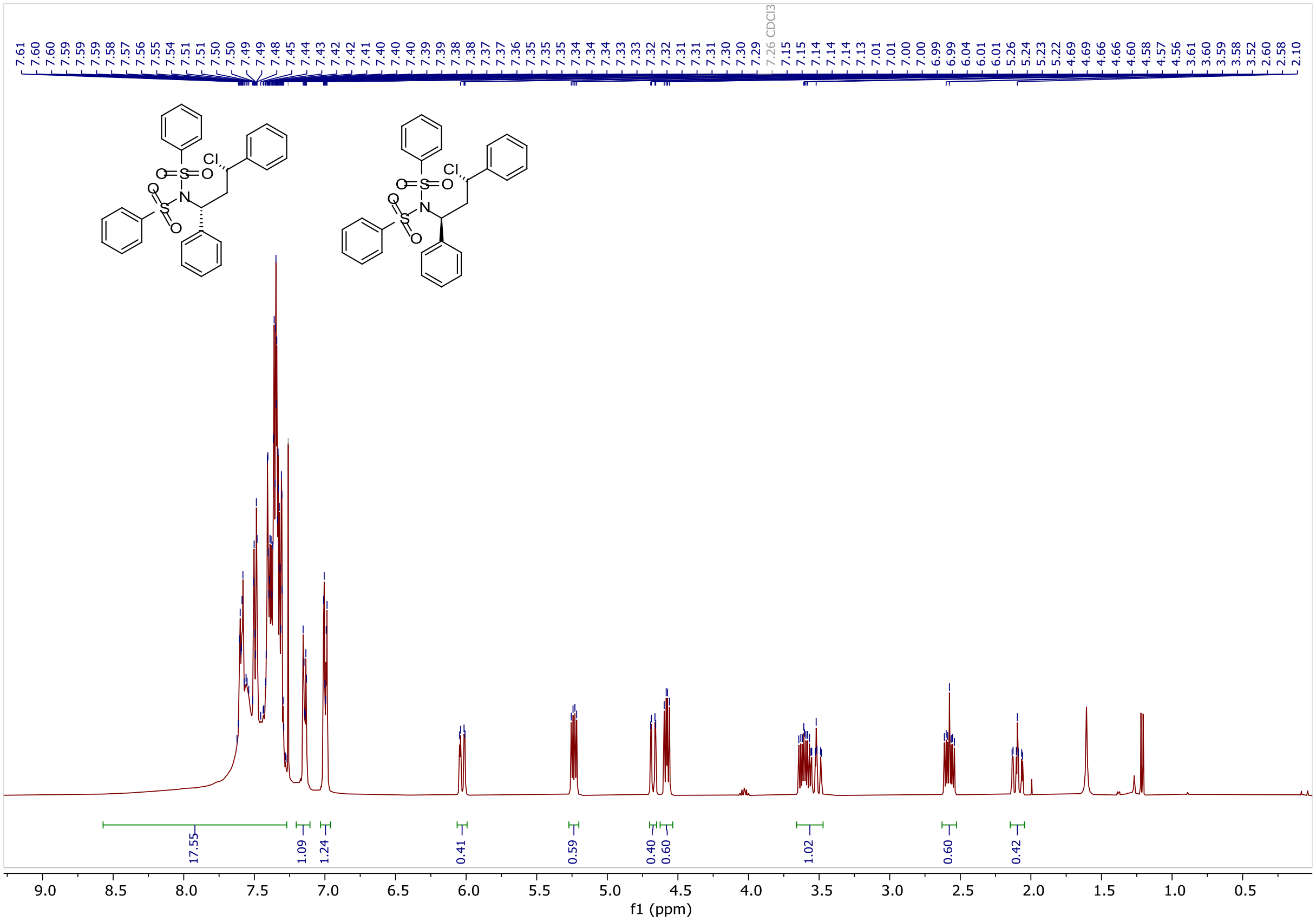

^{13}C NMR Spectrum of 6s (101 MHz, $CDCl_3$)

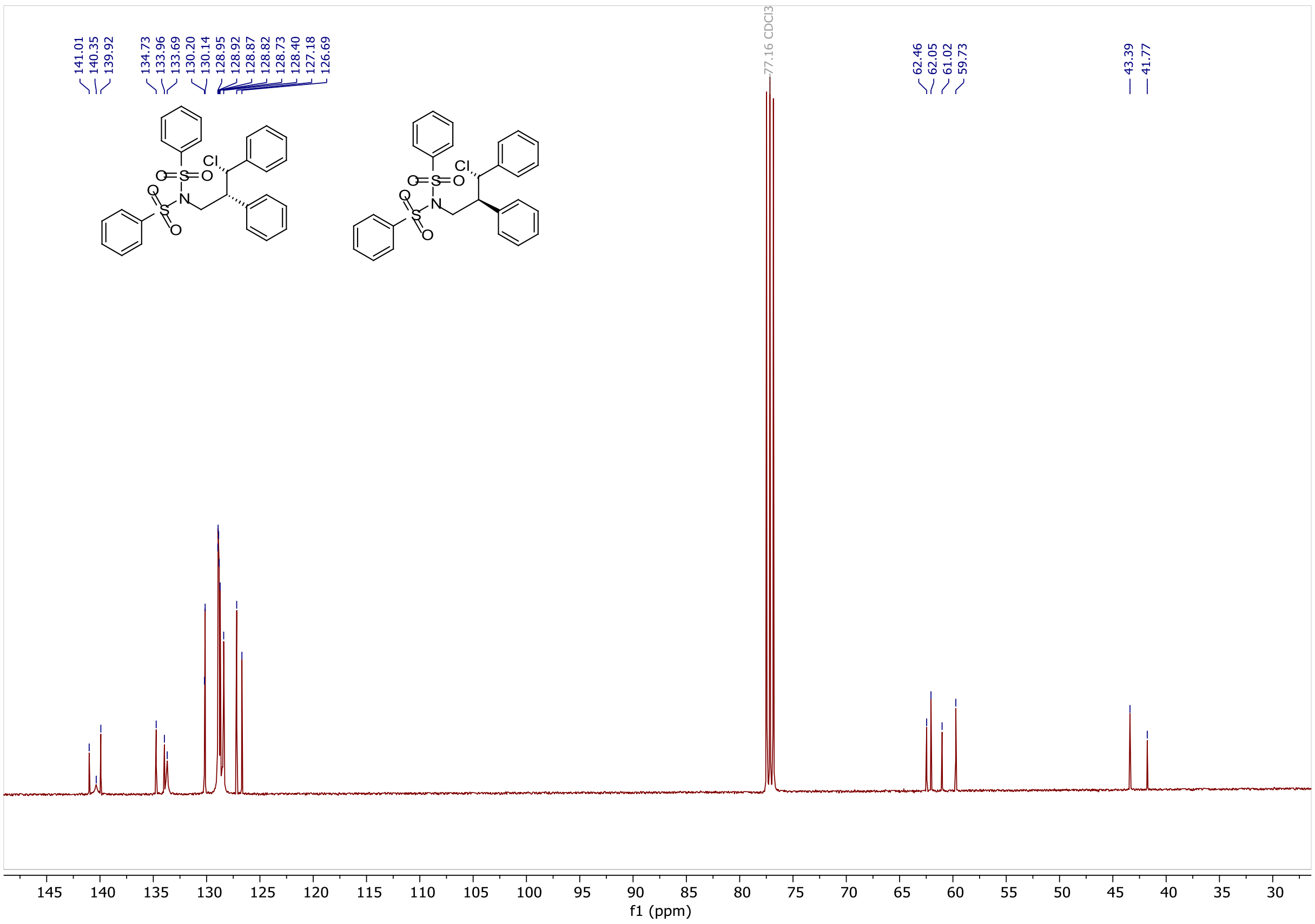

1,3-aminoarylated products **7**

^{1}H NMR Spectrum of *o*-7a (400 MHz, $CDCl_3$)

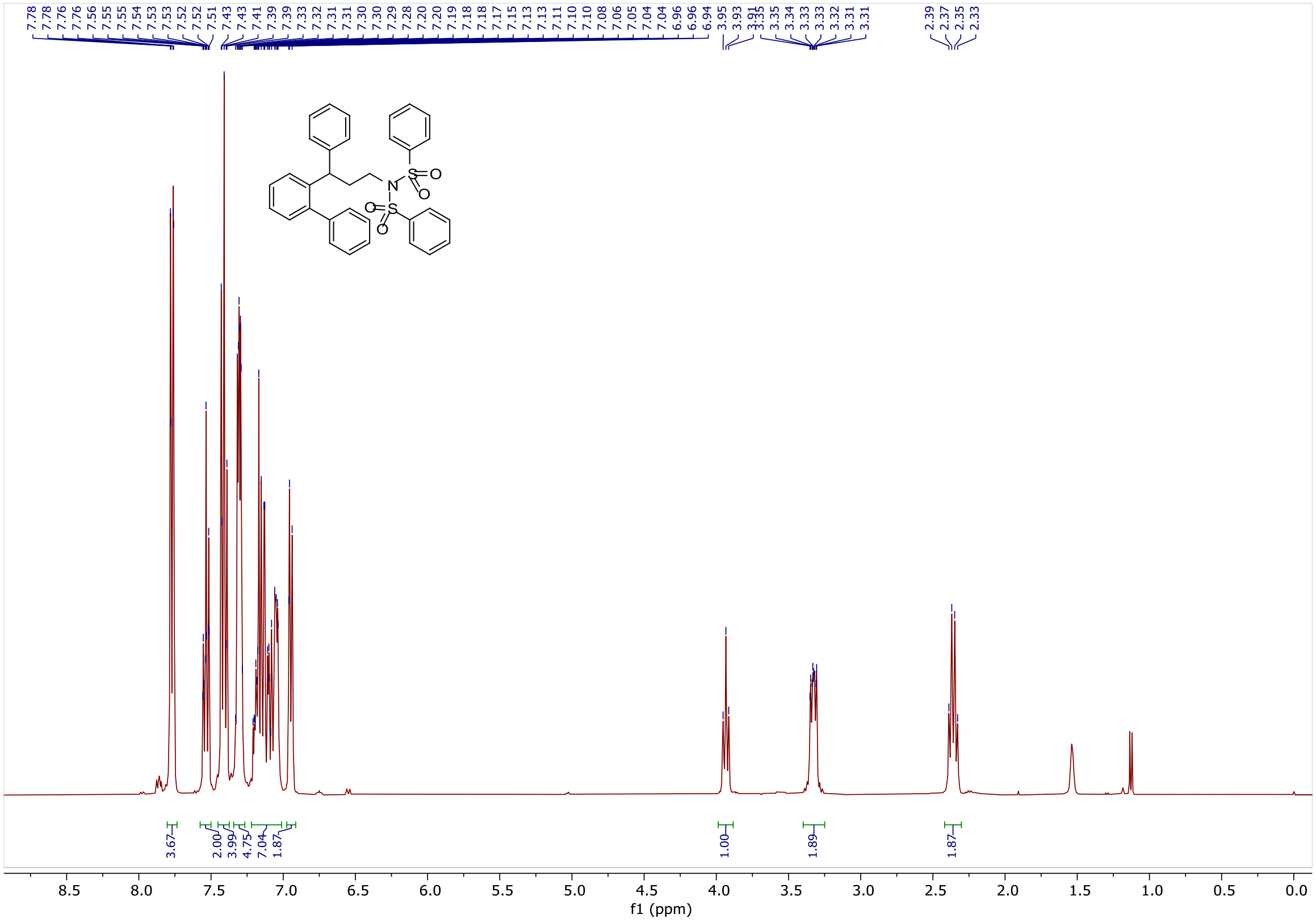

^{13}C NMR Spectrum of *o*-7a (101 MHz, $CDCl_3$)

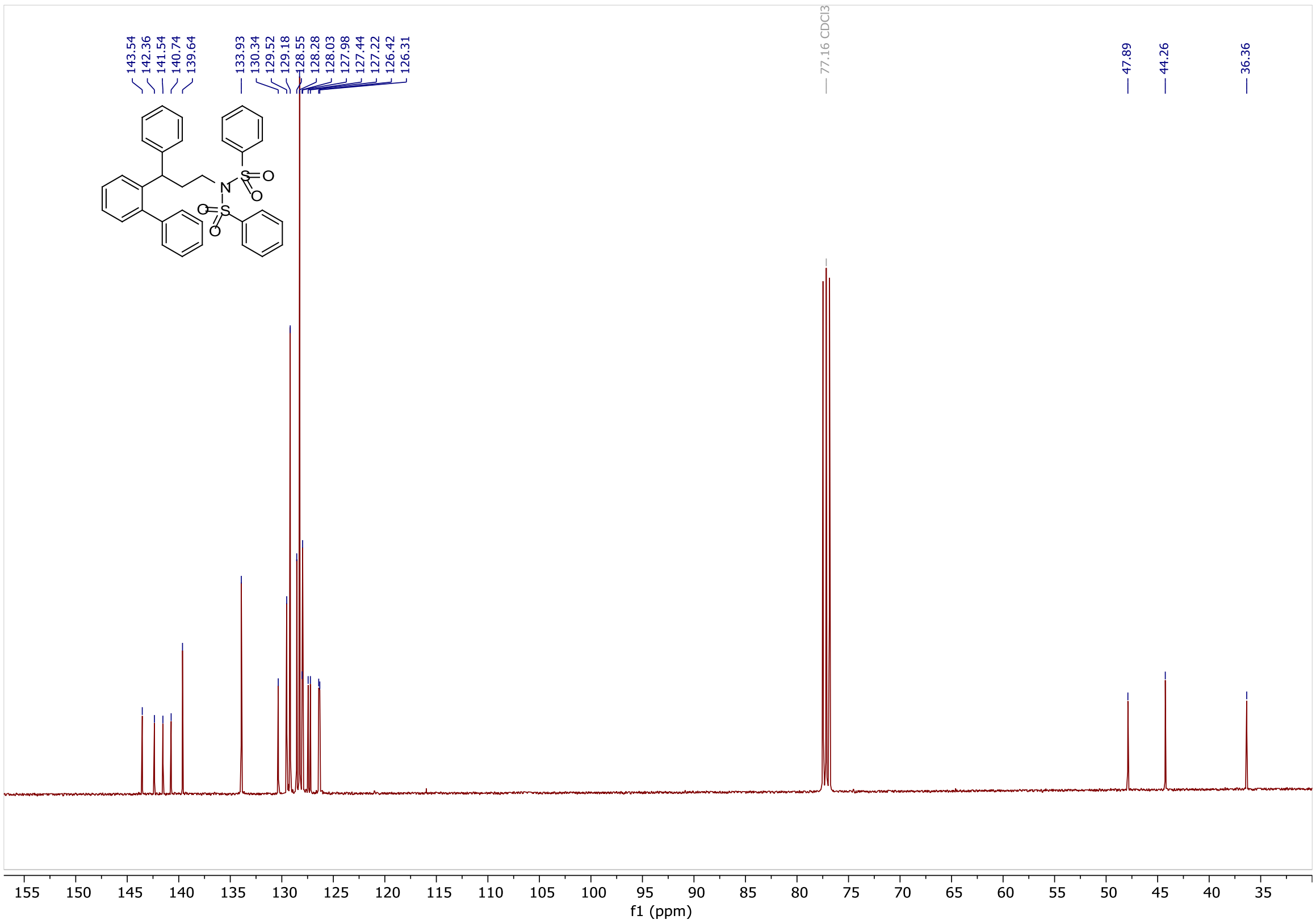

^{1}H NMR Spectrum of *m*-7a (400 MHz, $CDCl_3$)

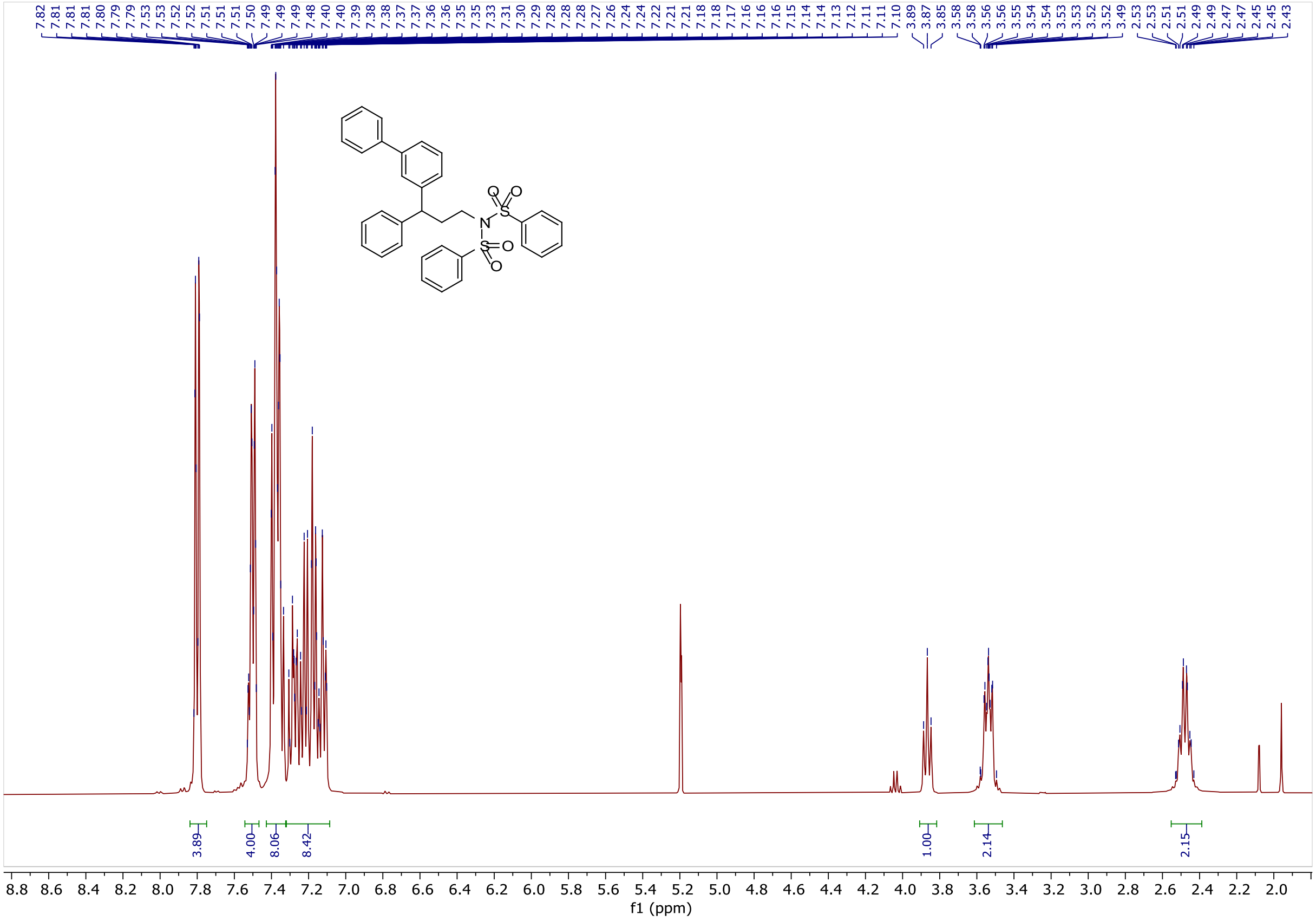

^{13}C NMR Spectrum of *m*-7a (101 MHz, $CDCl_3$)

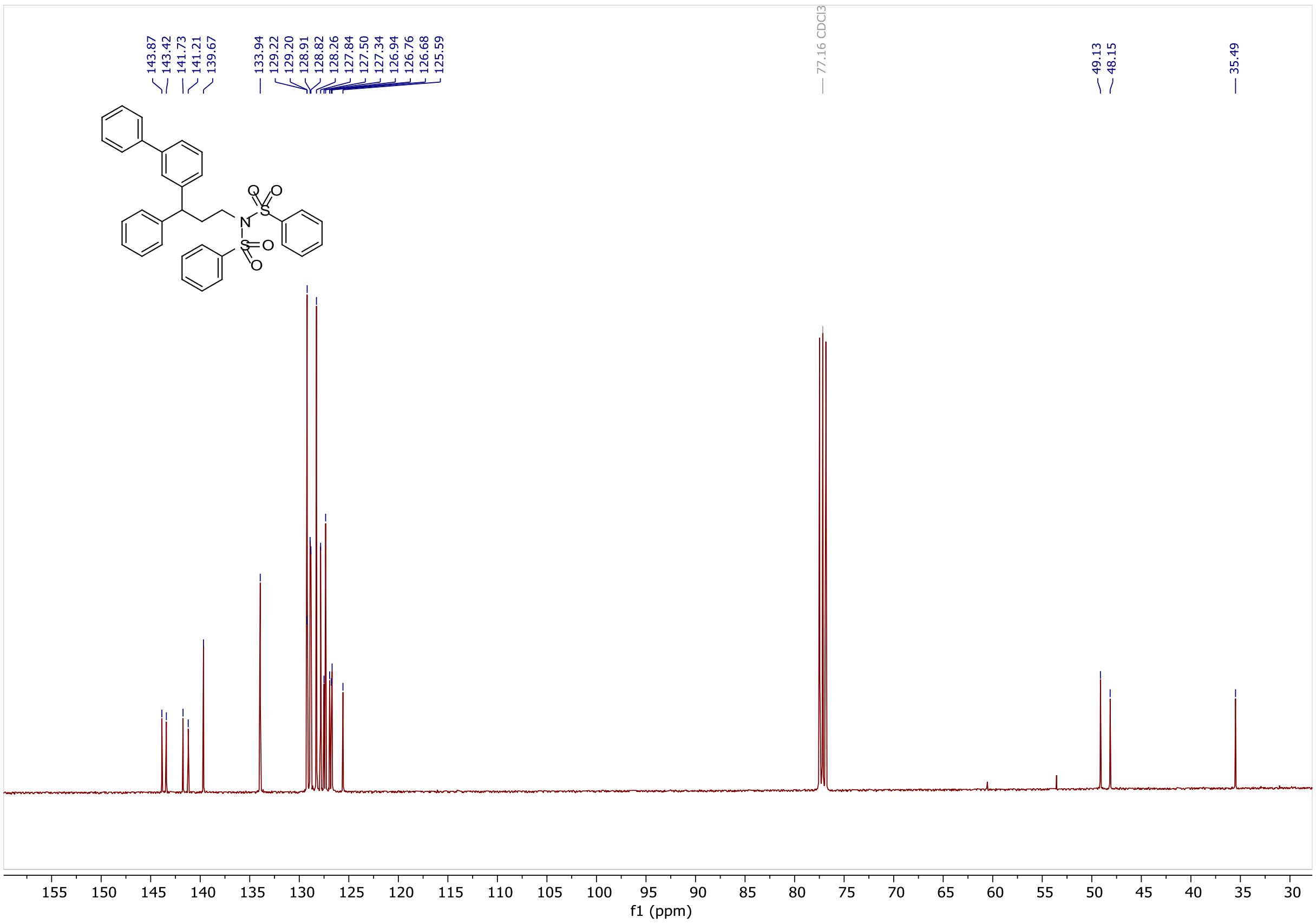

^{1}H NMR Spectrum of *p*-7a (400 MHz, $CDCl_3$)

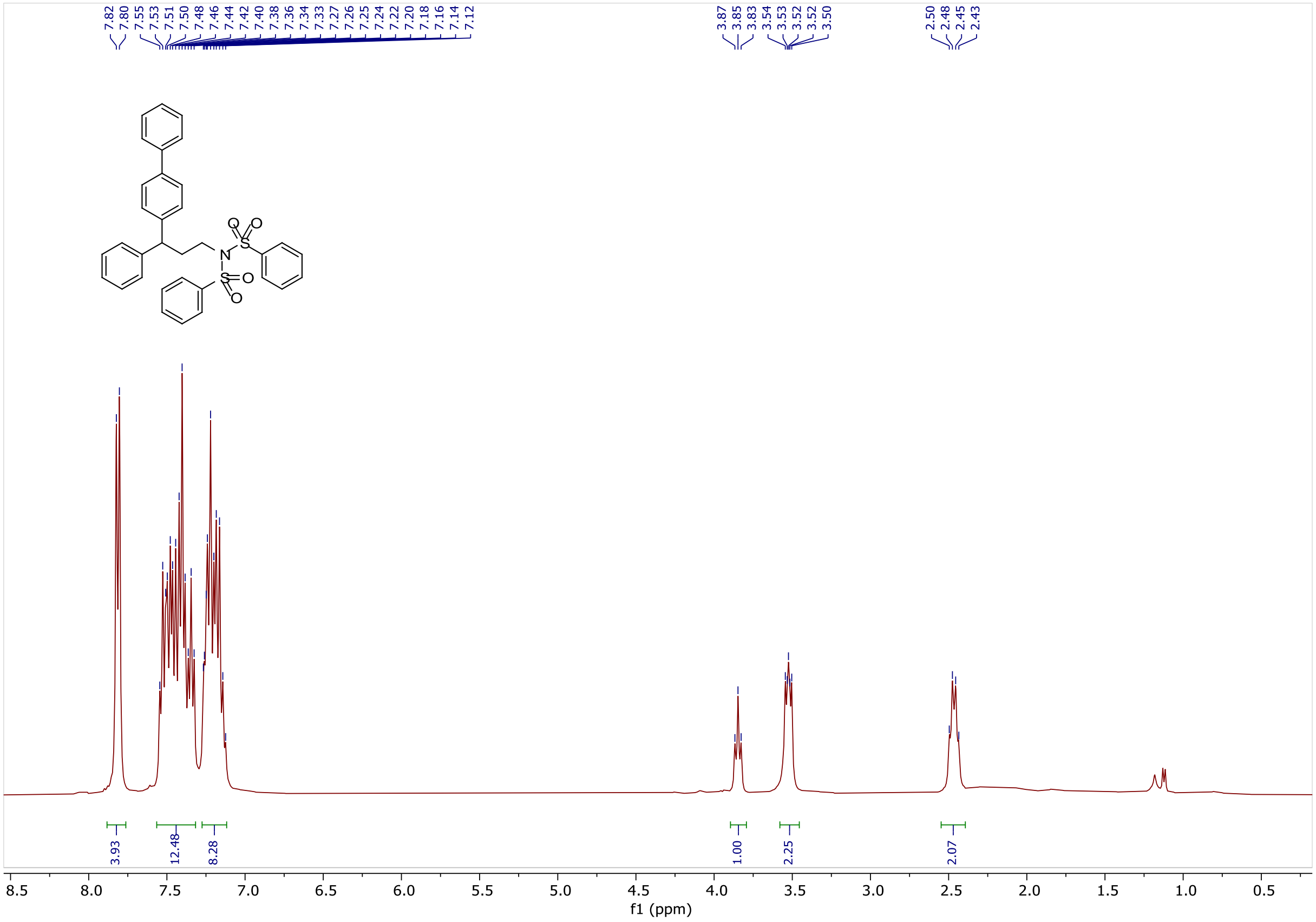

^{13}C NMR Spectrum of *p*-7a (101 MHz, $CDCl_3$)

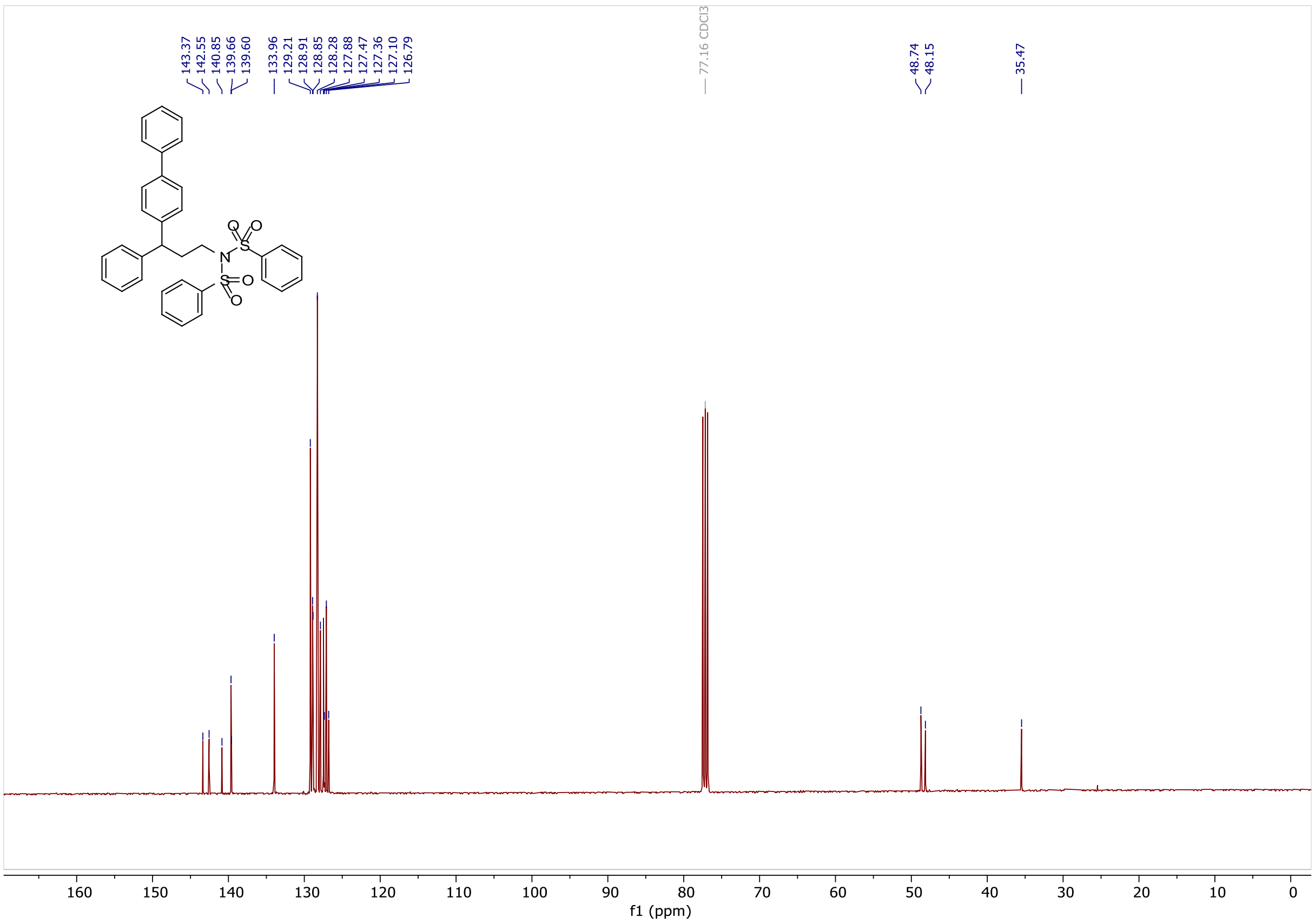

^{1}H NMR Spectrum of *o*-7b (400 MHz, $CDCl_3$)

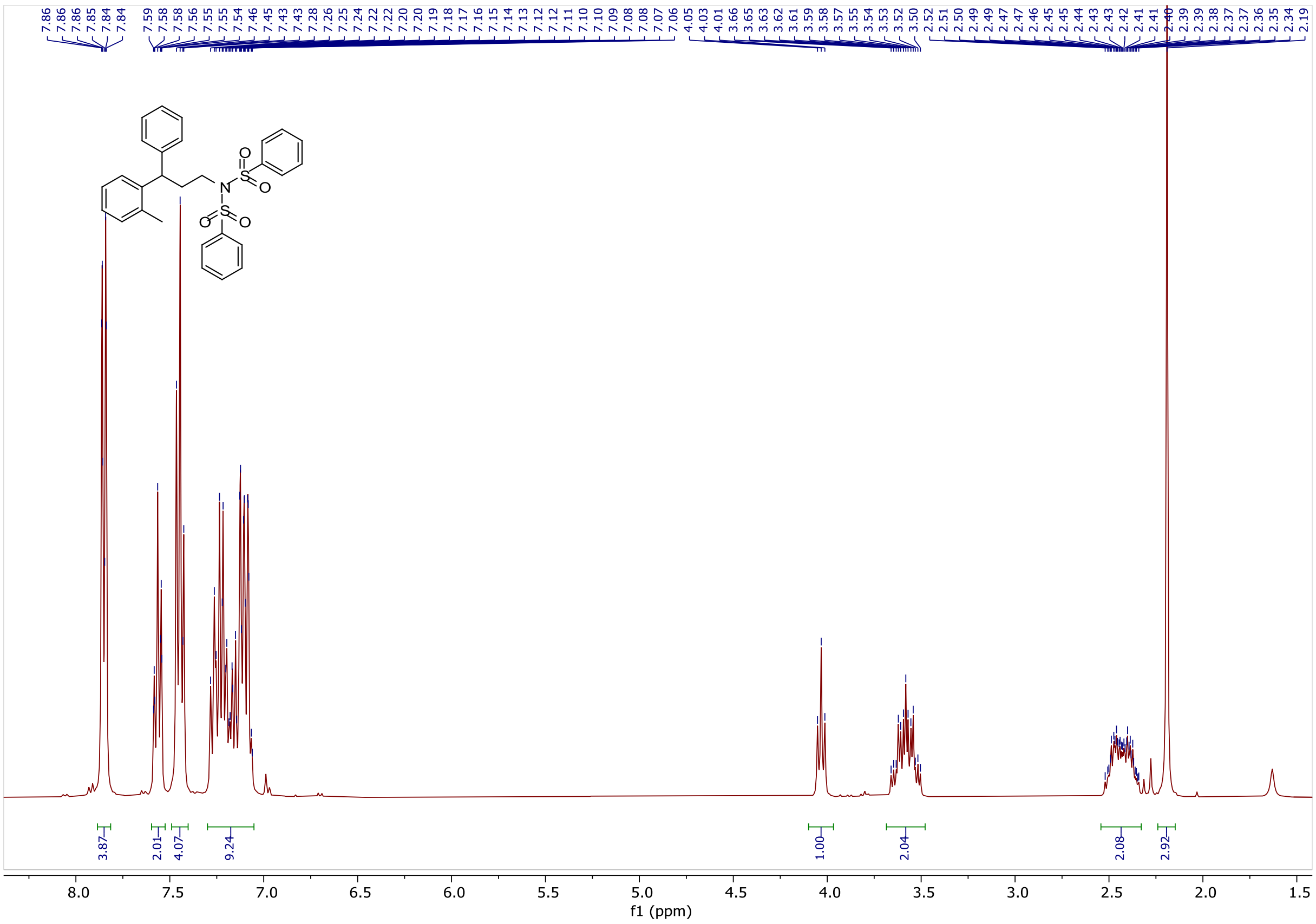

^{13}C NMR Spectrum of *o*-7b (101 MHz, $CDCl_3$)

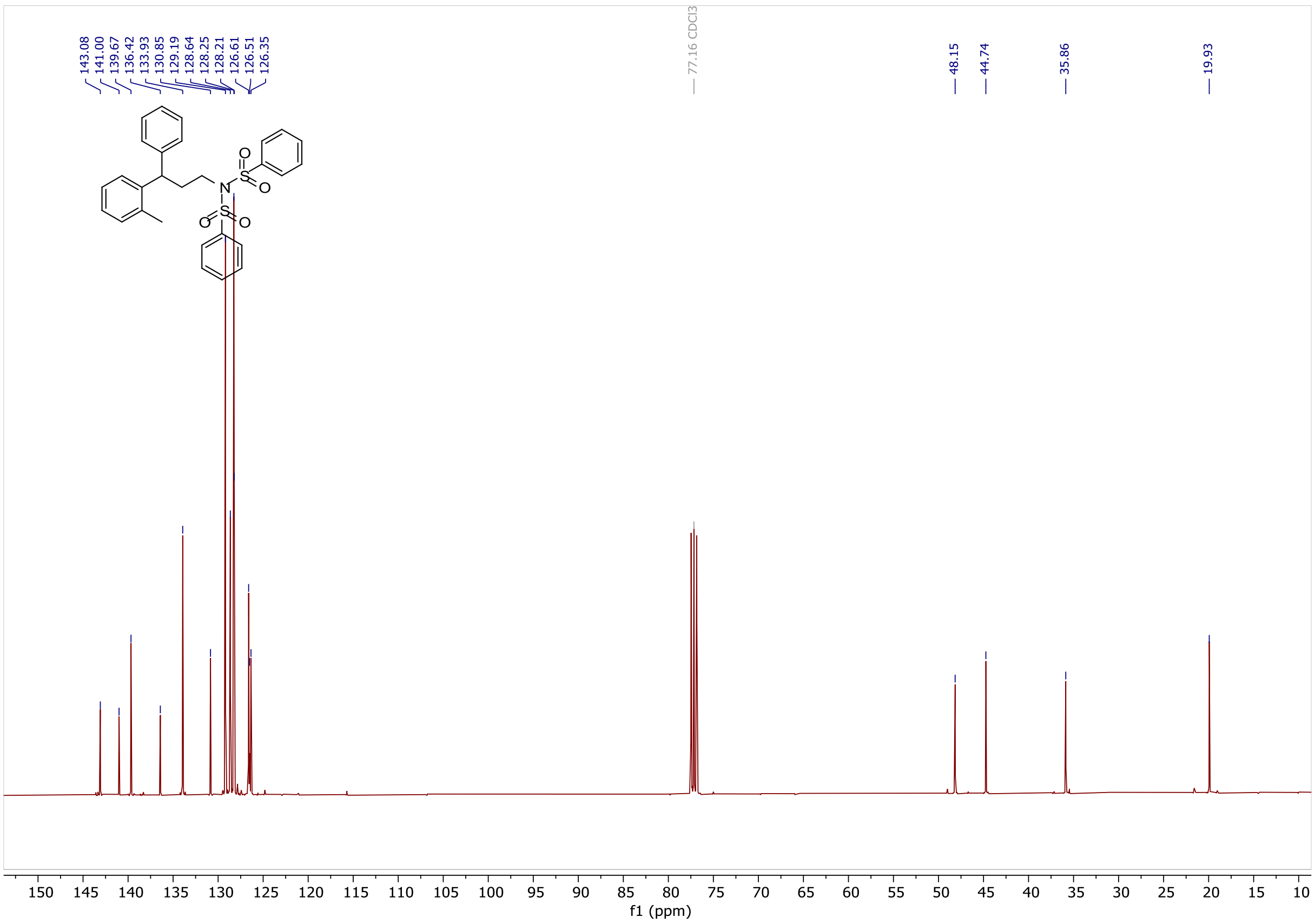

^{1}H NMR Spectrum of *m*-7b (400 MHz, $CDCl_3$)

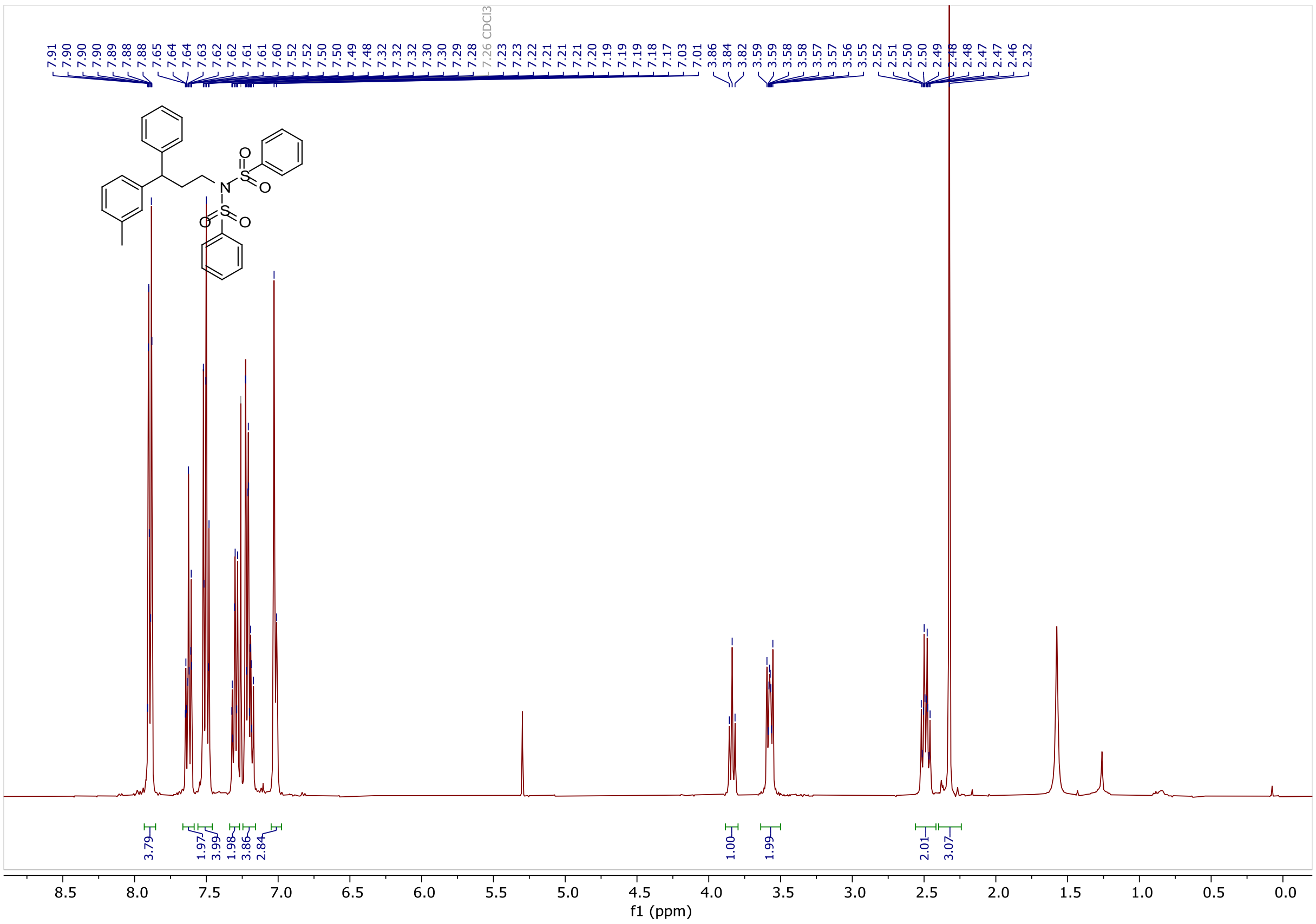

^{13}C NMR Spectrum of *m*-7b (101 MHz, $CDCl_3$)

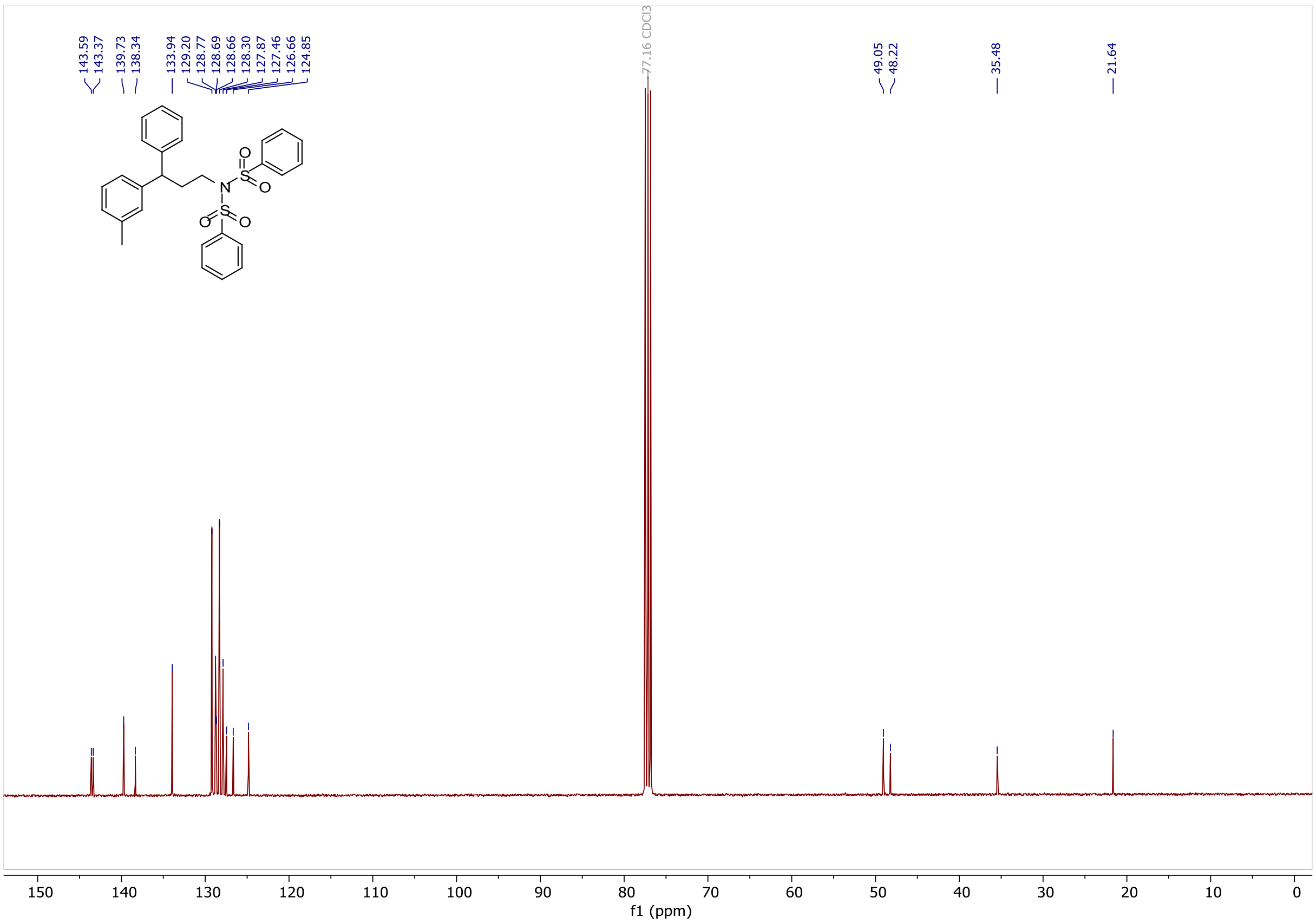

^{1}H NMR Spectrum of *p*-7b (400 MHz, $CDCl_3$)

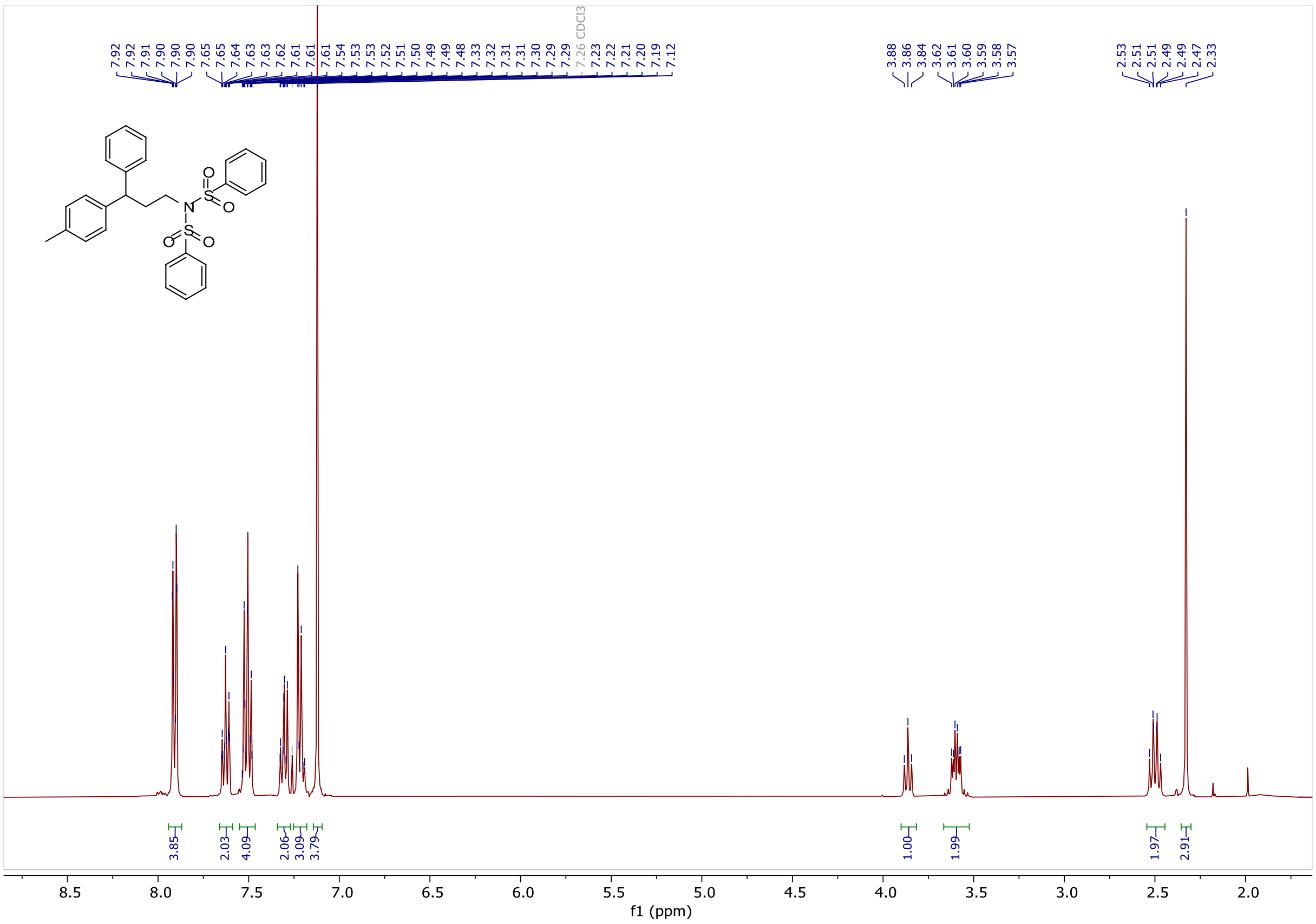

^{13}C NMR Spectrum of *p*-7b (101 MHz, $CDCl_3$)

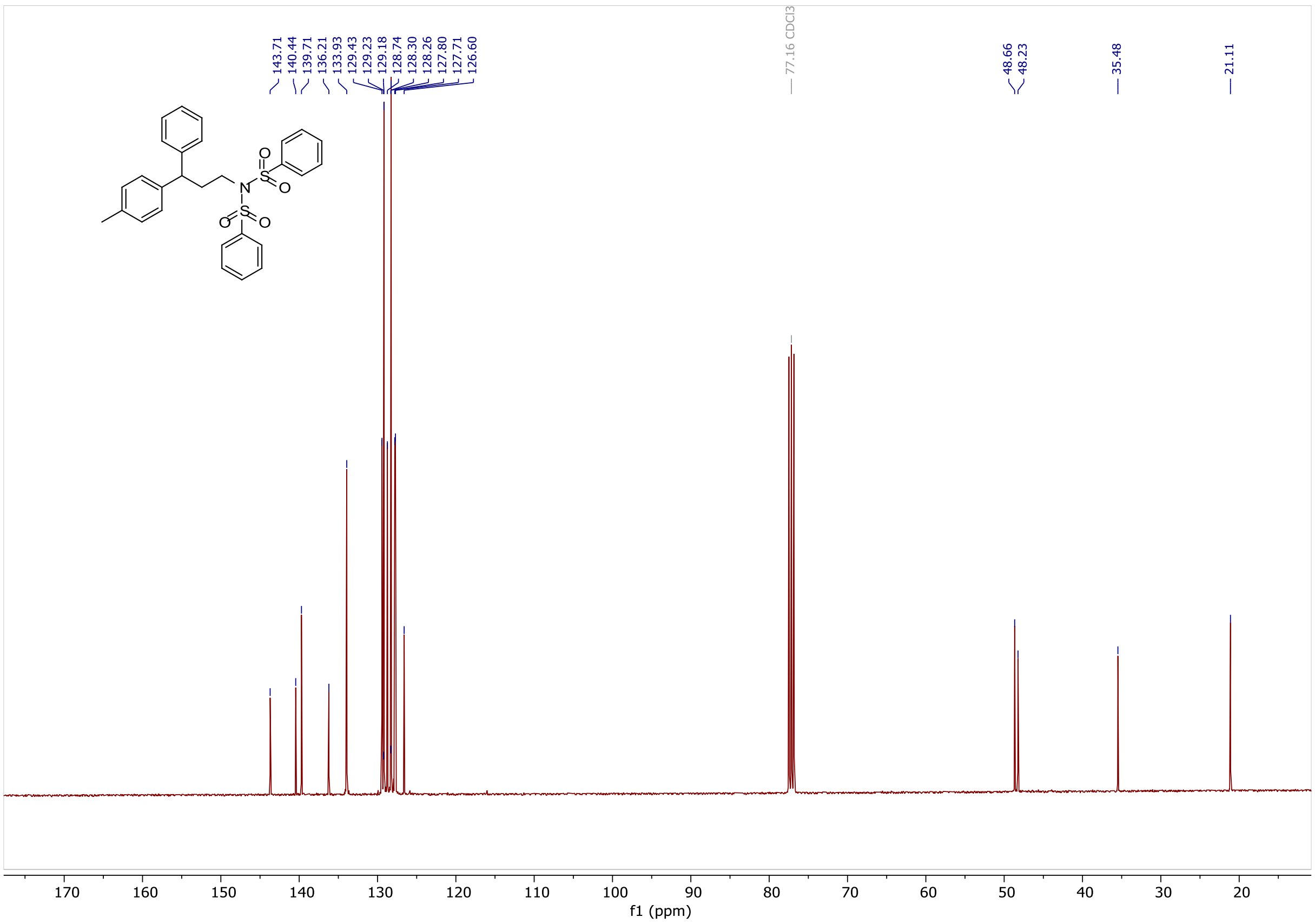

^{1}H NMR Spectrum of *o*-7c (400 MHz, $CDCl_3$)

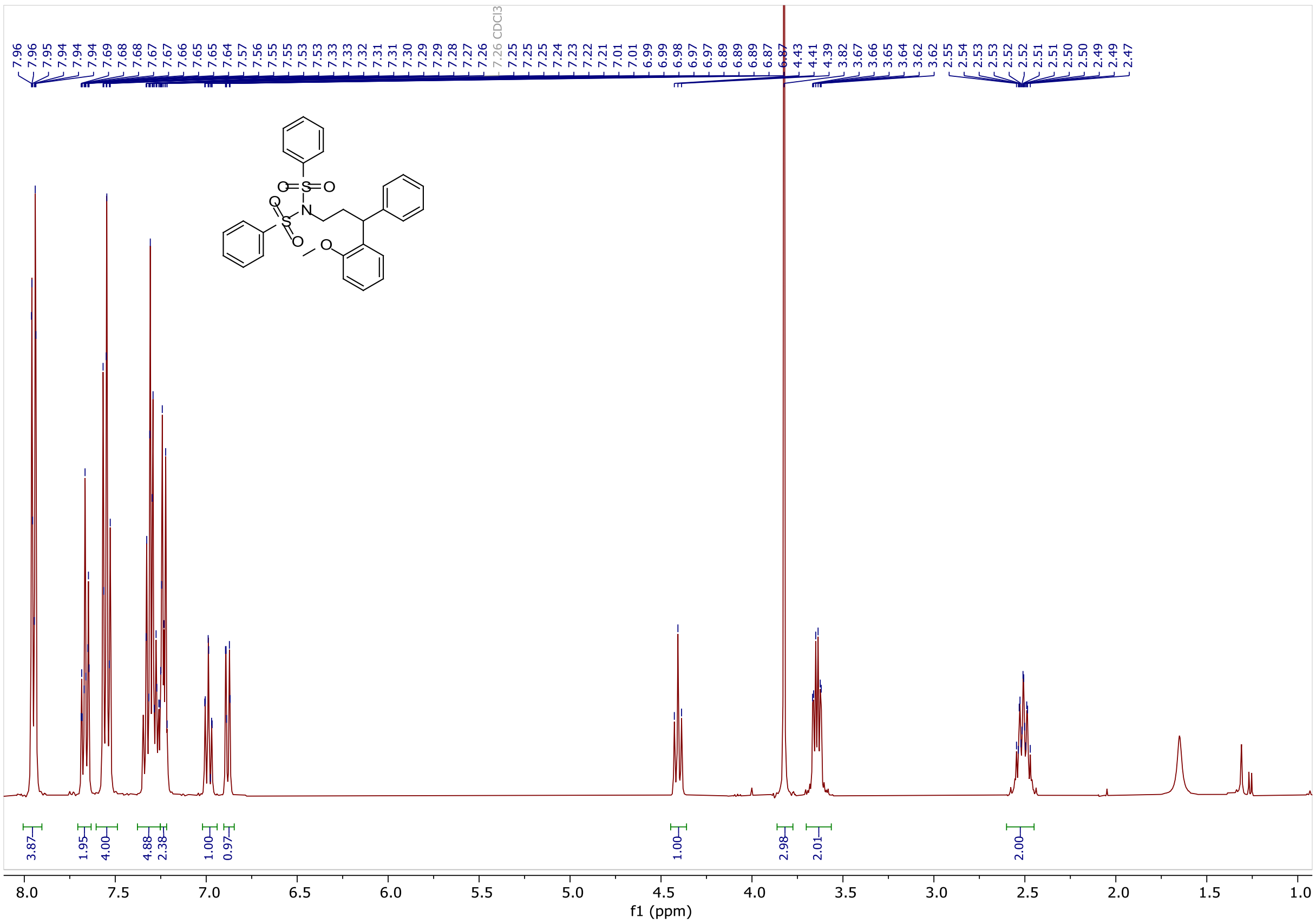

^{13}C NMR Spectrum of *o*-7c (101 MHz, $CDCl_3$)

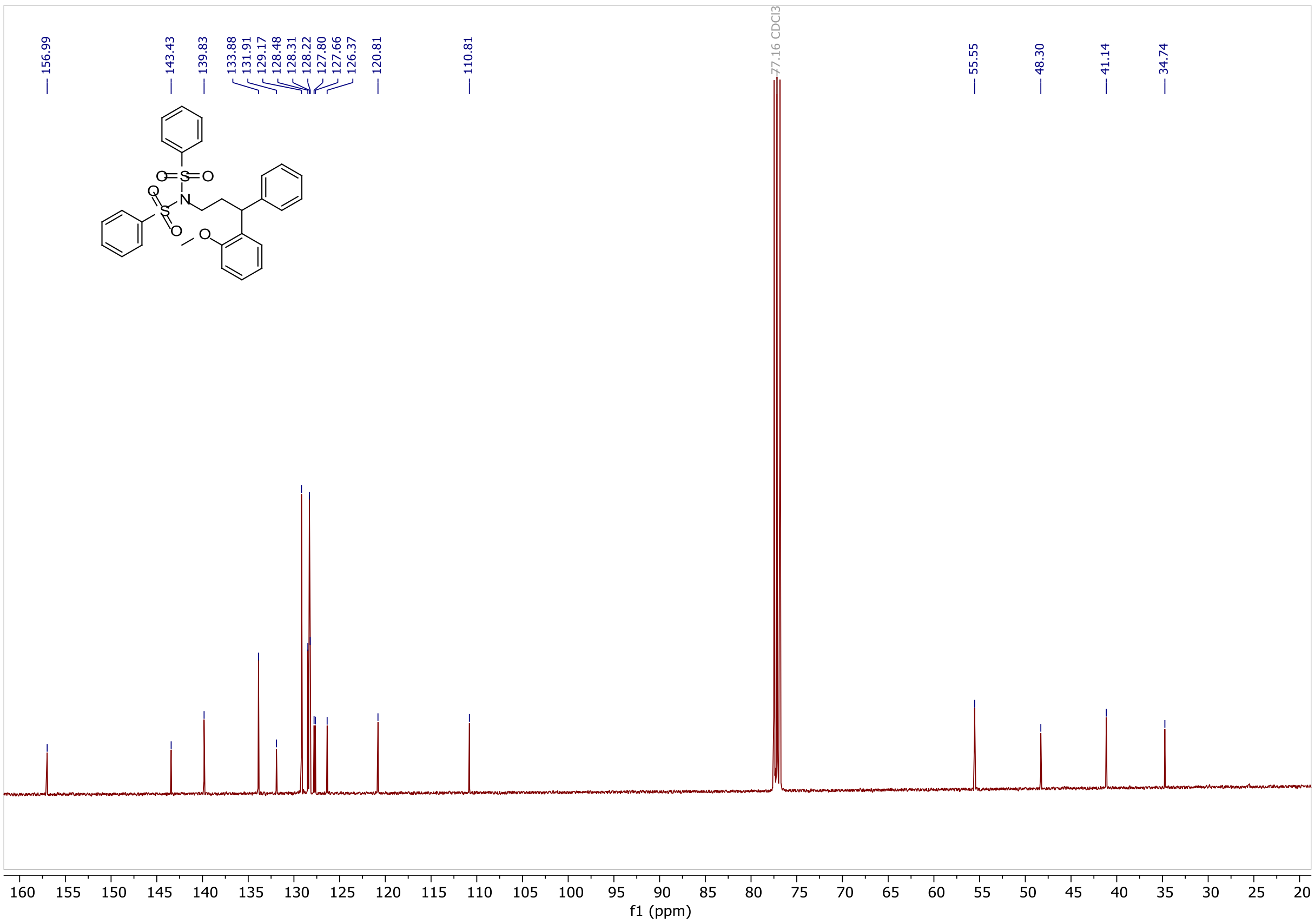

^{1}H NMR Spectrum of *p*-7c (400 MHz, $CDCl_3$)

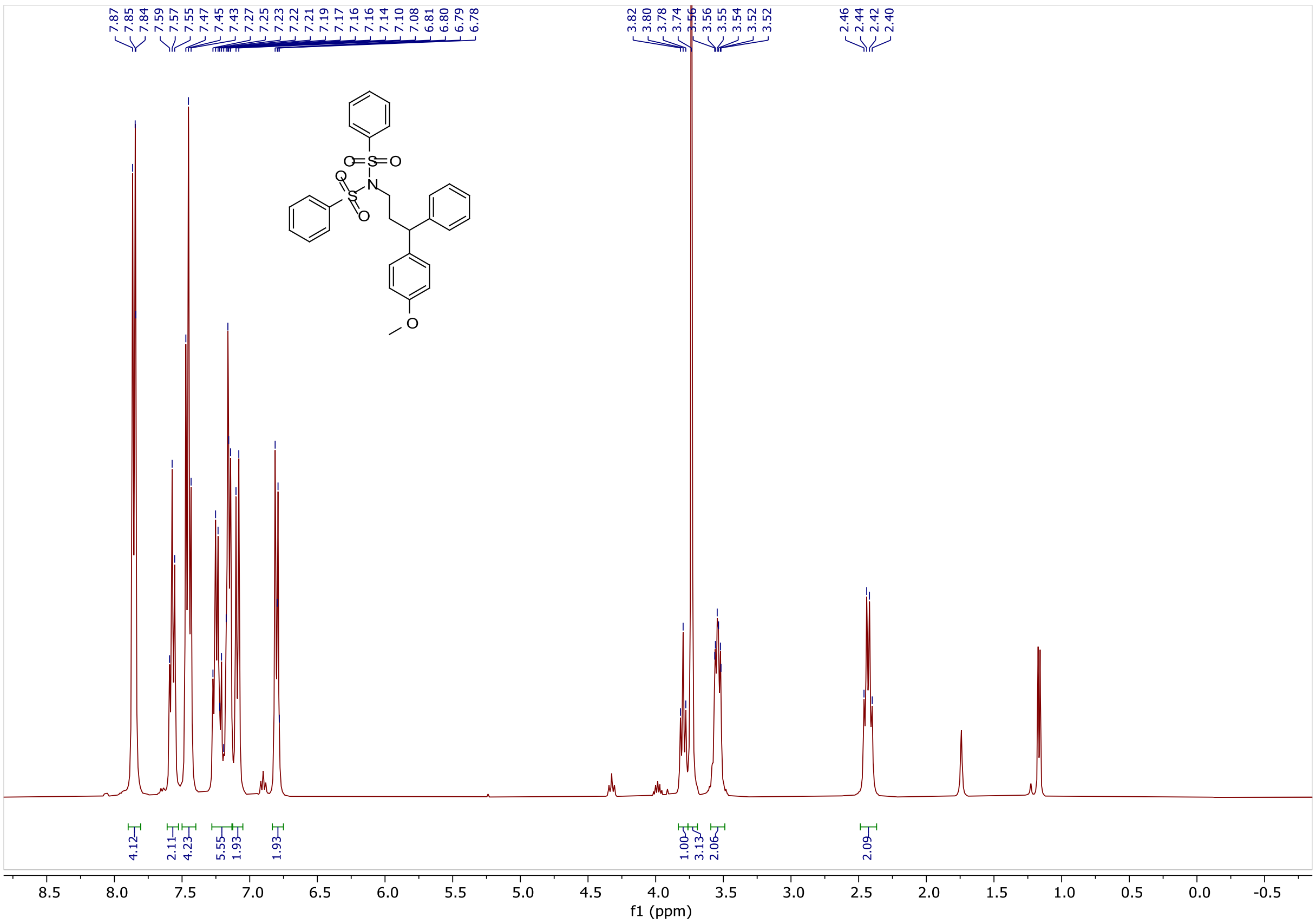

^{13}C NMR Spectrum of *p*-7c (101 MHz, $CDCl_3$)

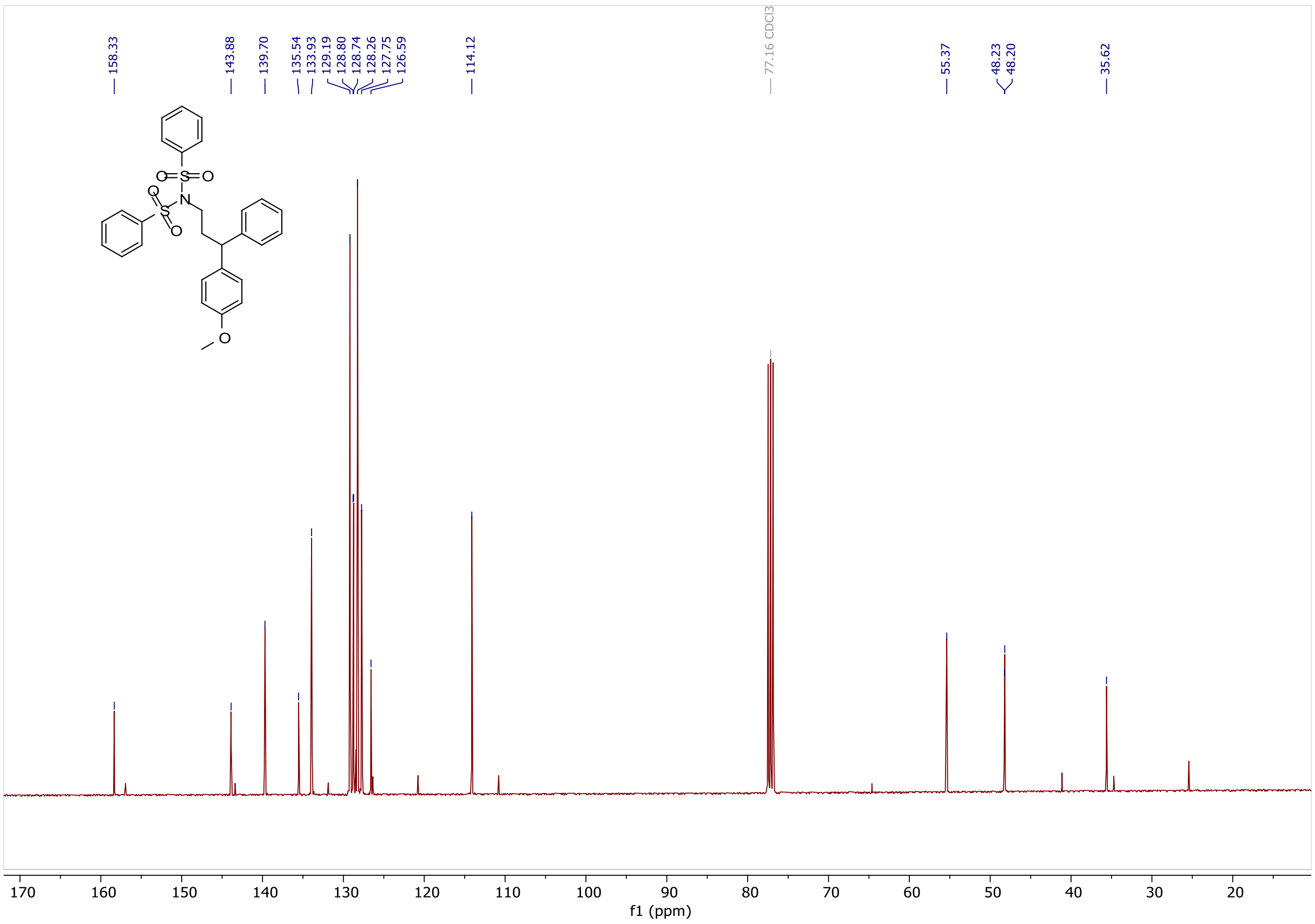

^{1}H NMR Spectrum of 7d (400 MHz, $CDCl_3$)

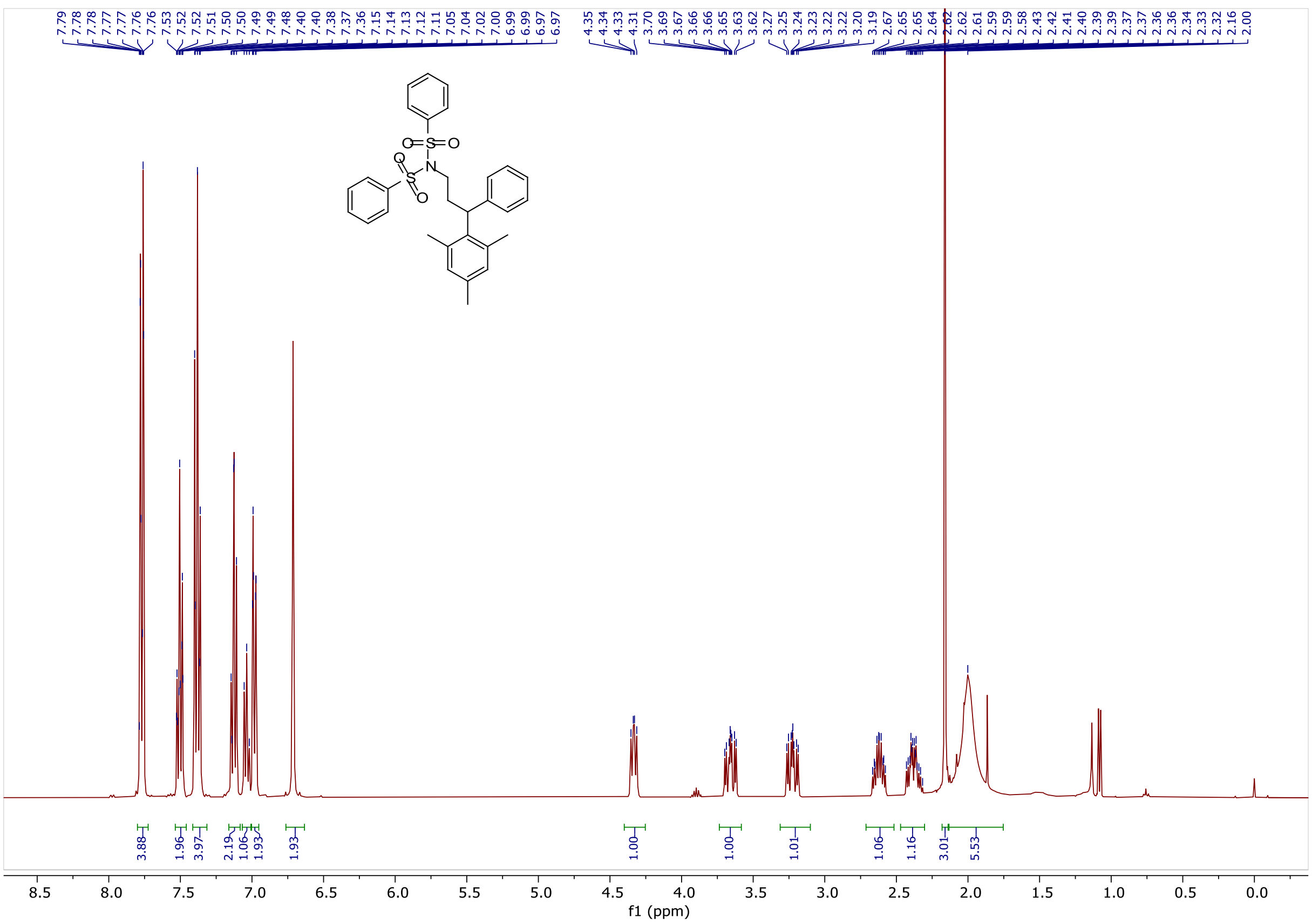

^{13}C NMR Spectrum of 7d (101 MHz, $CDCl_3$)

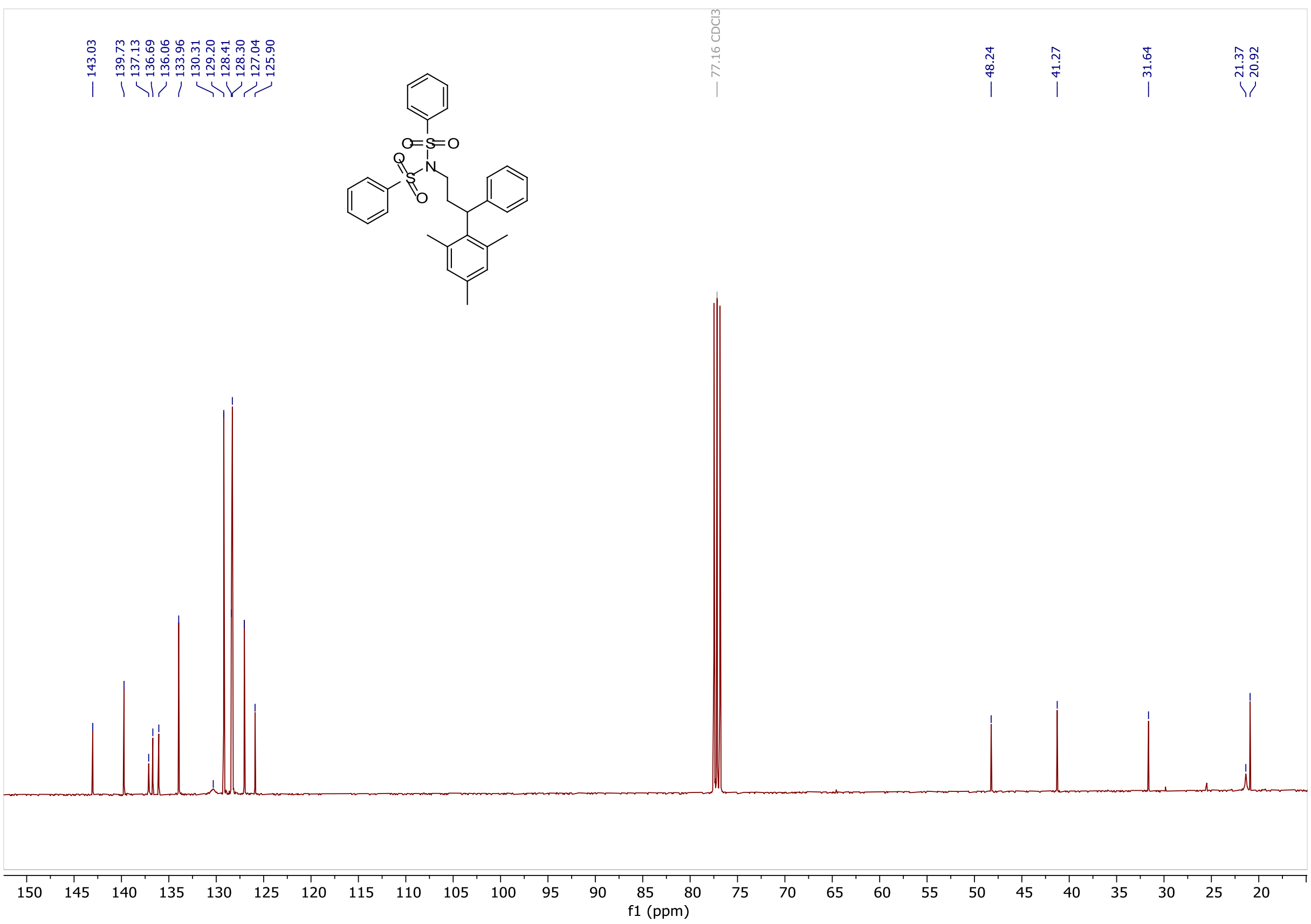

^{1}H NMR Spectrum of 7e (400 MHz, $CDCl_3$)

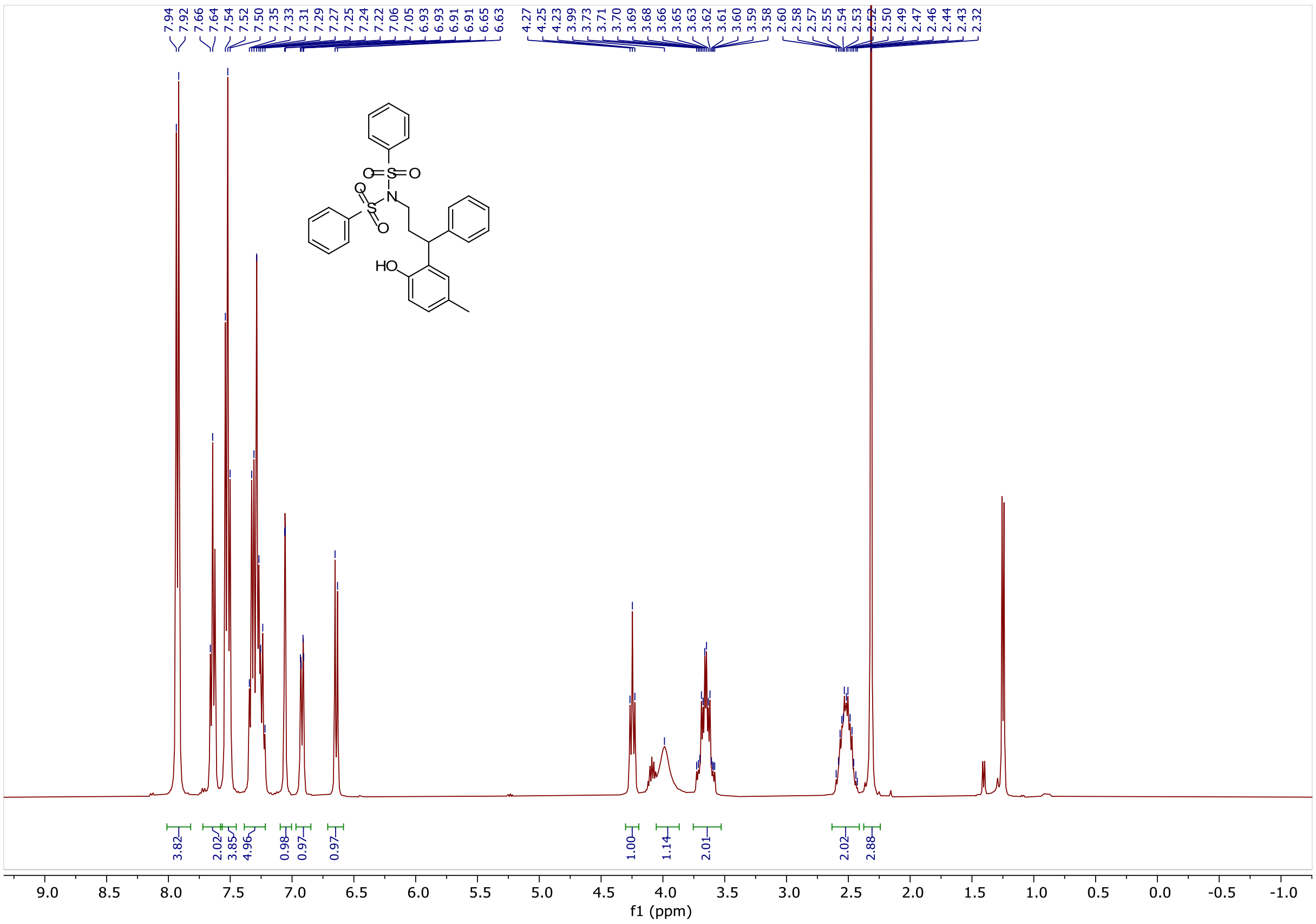

^{13}C NMR Spectrum of 7e (101 MHz, $CDCl_3$)

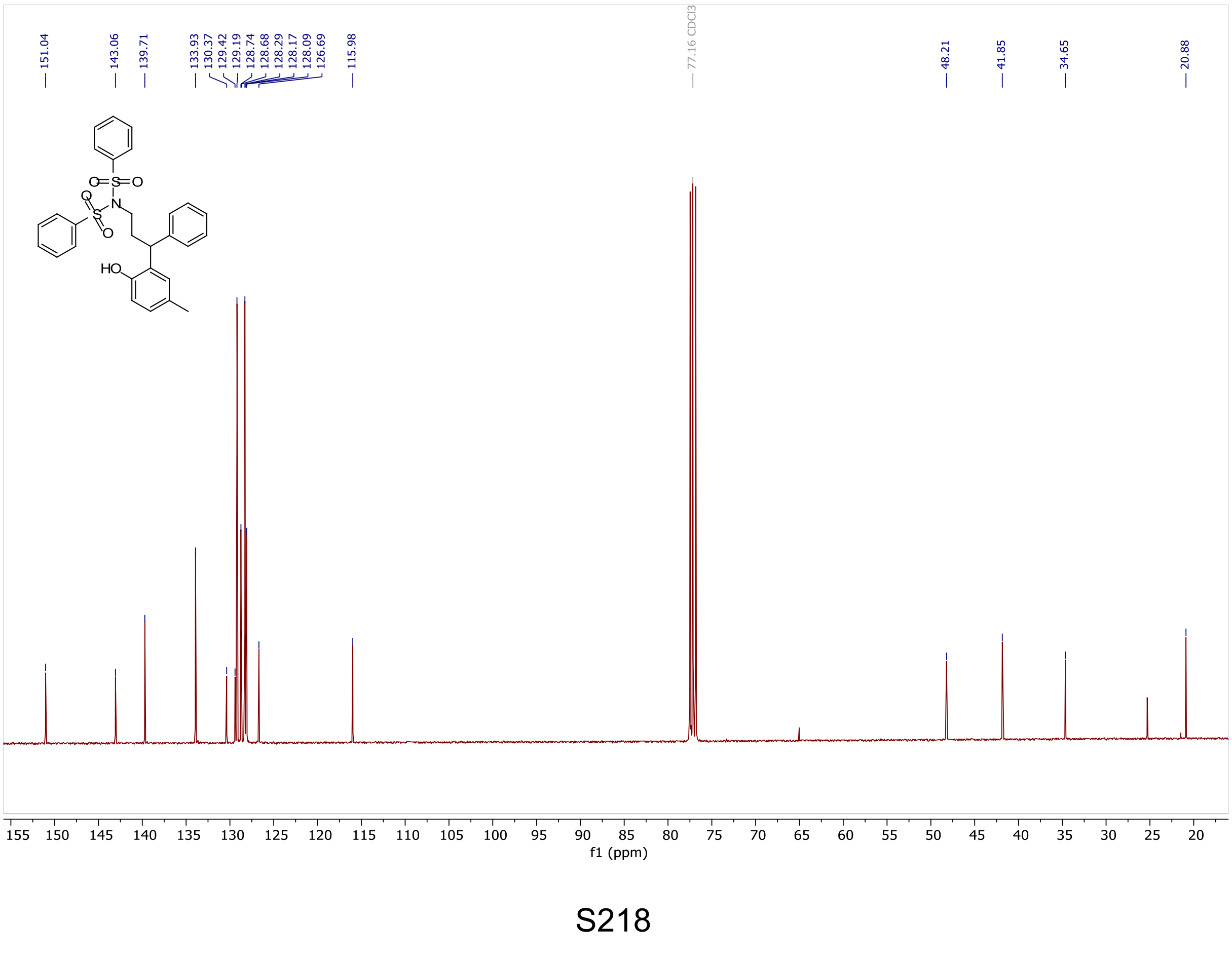

^{1}H NMR Spectrum of 7f (400 MHz, $CDCl_3$)

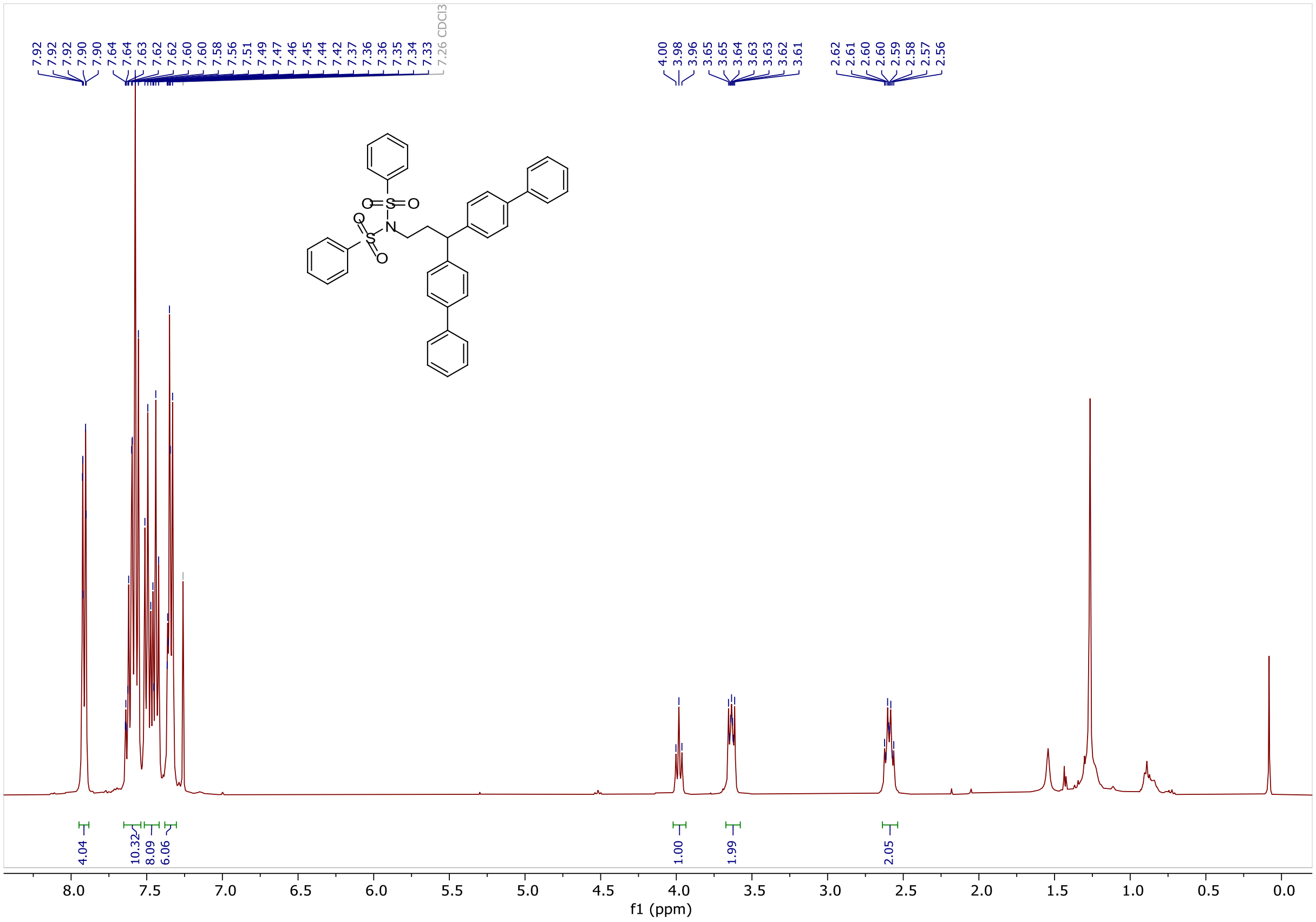

^{13}C NMR Spectrum of 7f (101 MHz, $CDCl_3$)

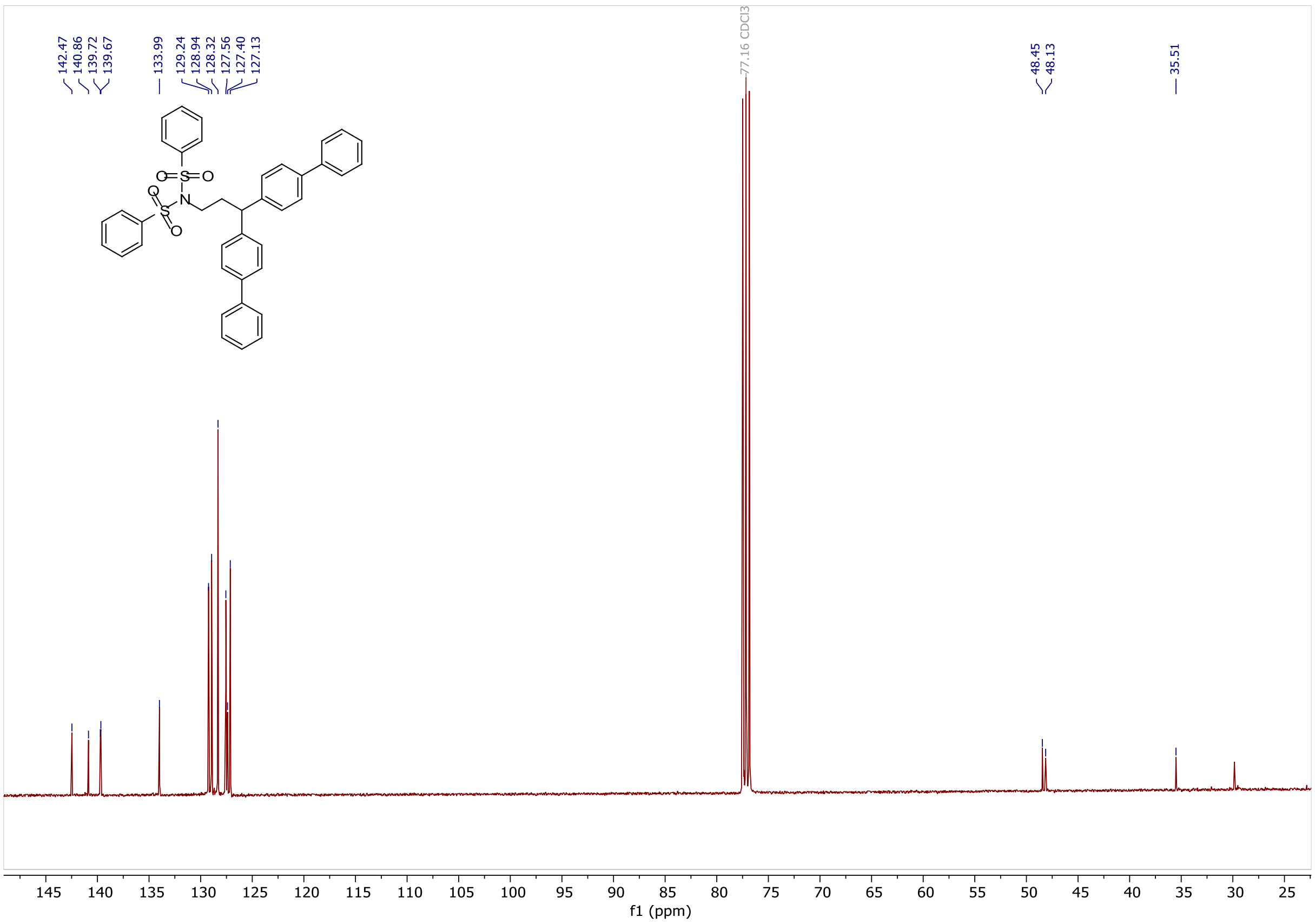

^{1}H NMR Spectrum of 7g (400 MHz, $CDCl_3$)

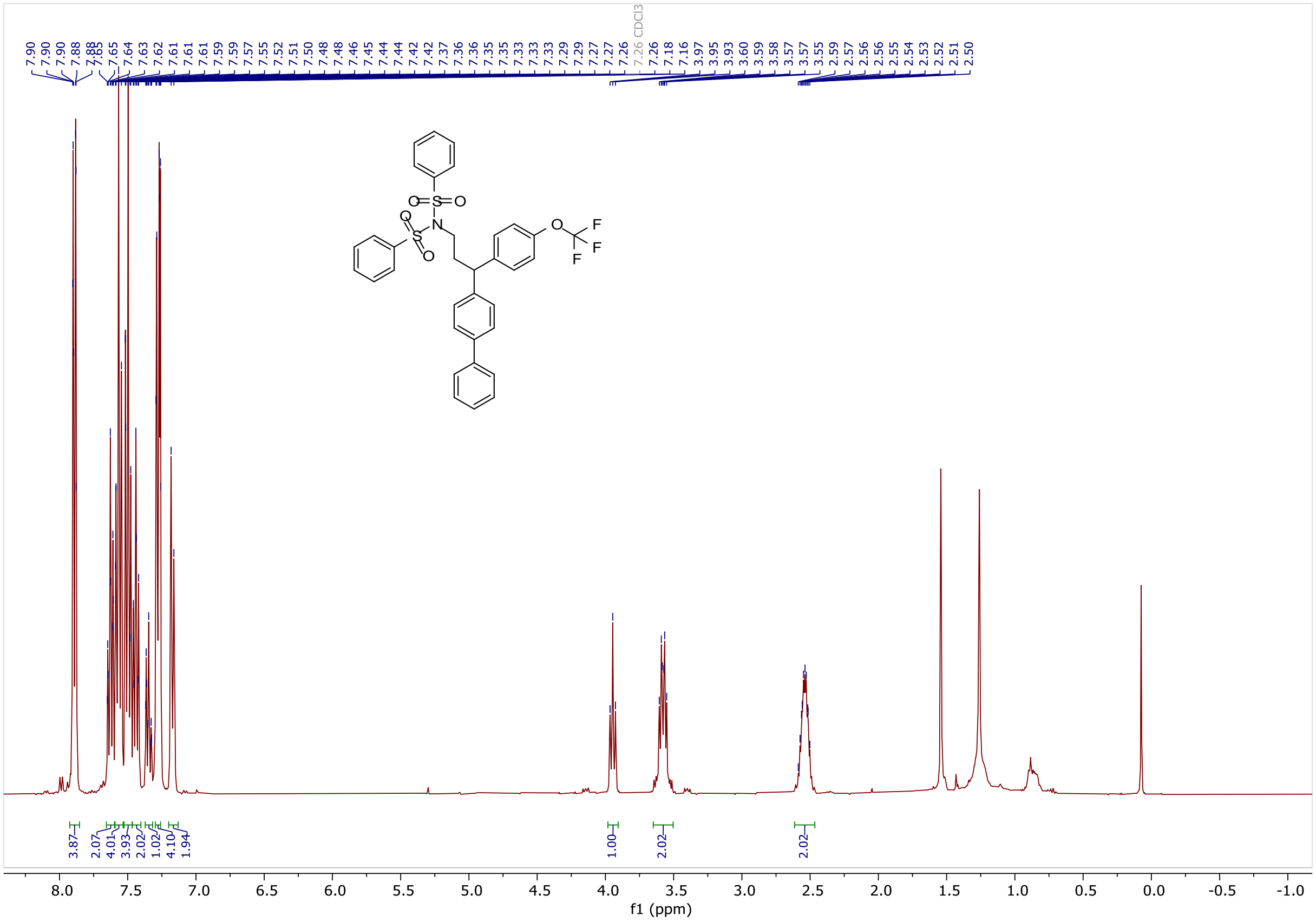

^{19}F NMR Spectrum of 7g (376 MHz, $CDCl_3$)

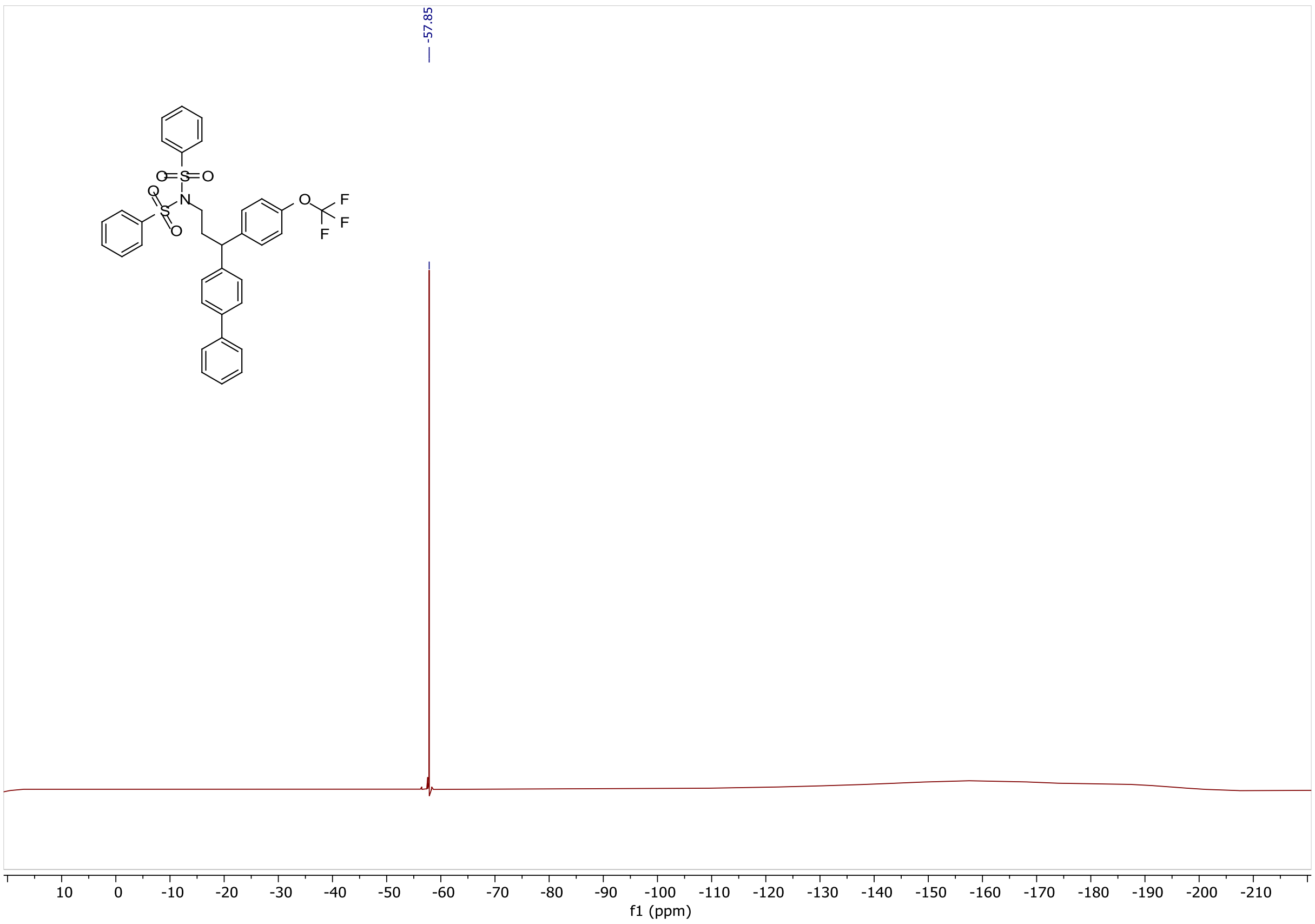

^{13}C NMR Spectrum of 7g (101 MHz, $CDCl_3$)

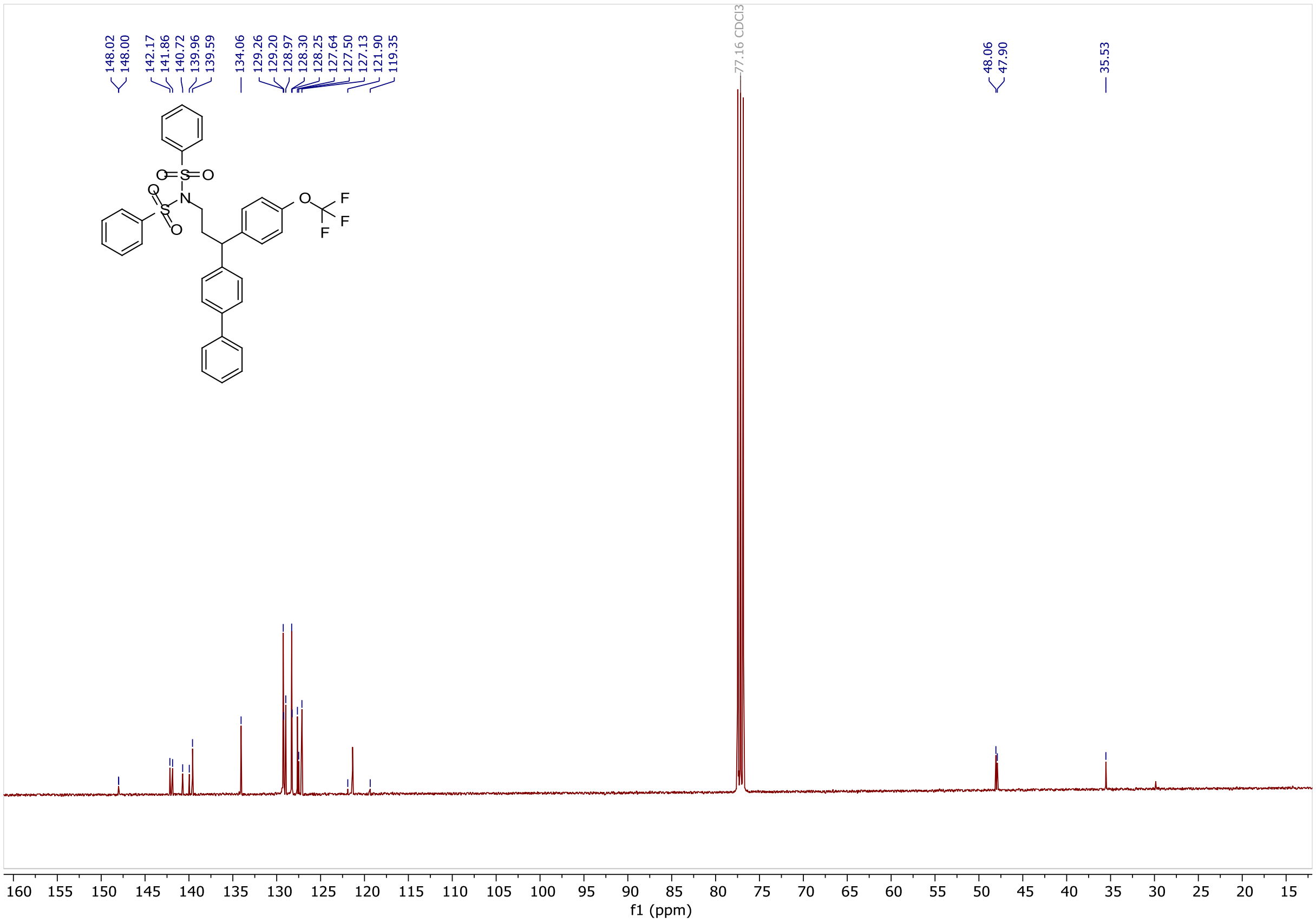

^{1}H NMR Spectrum of 7h (400 MHz, $CDCl_3$)

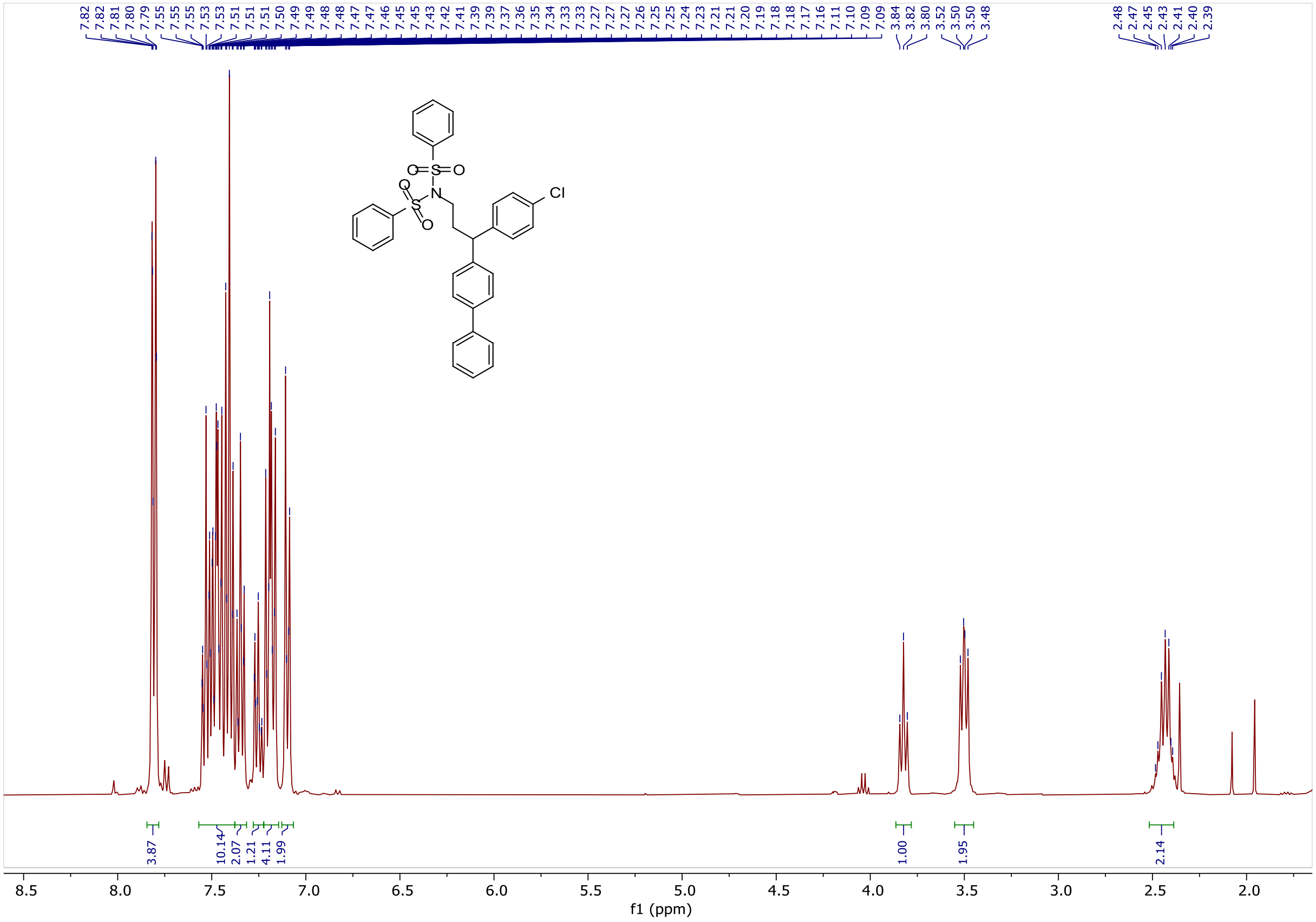

^{13}C NMR Spectrum of 7h (101 MHz, $CDCl_3$)

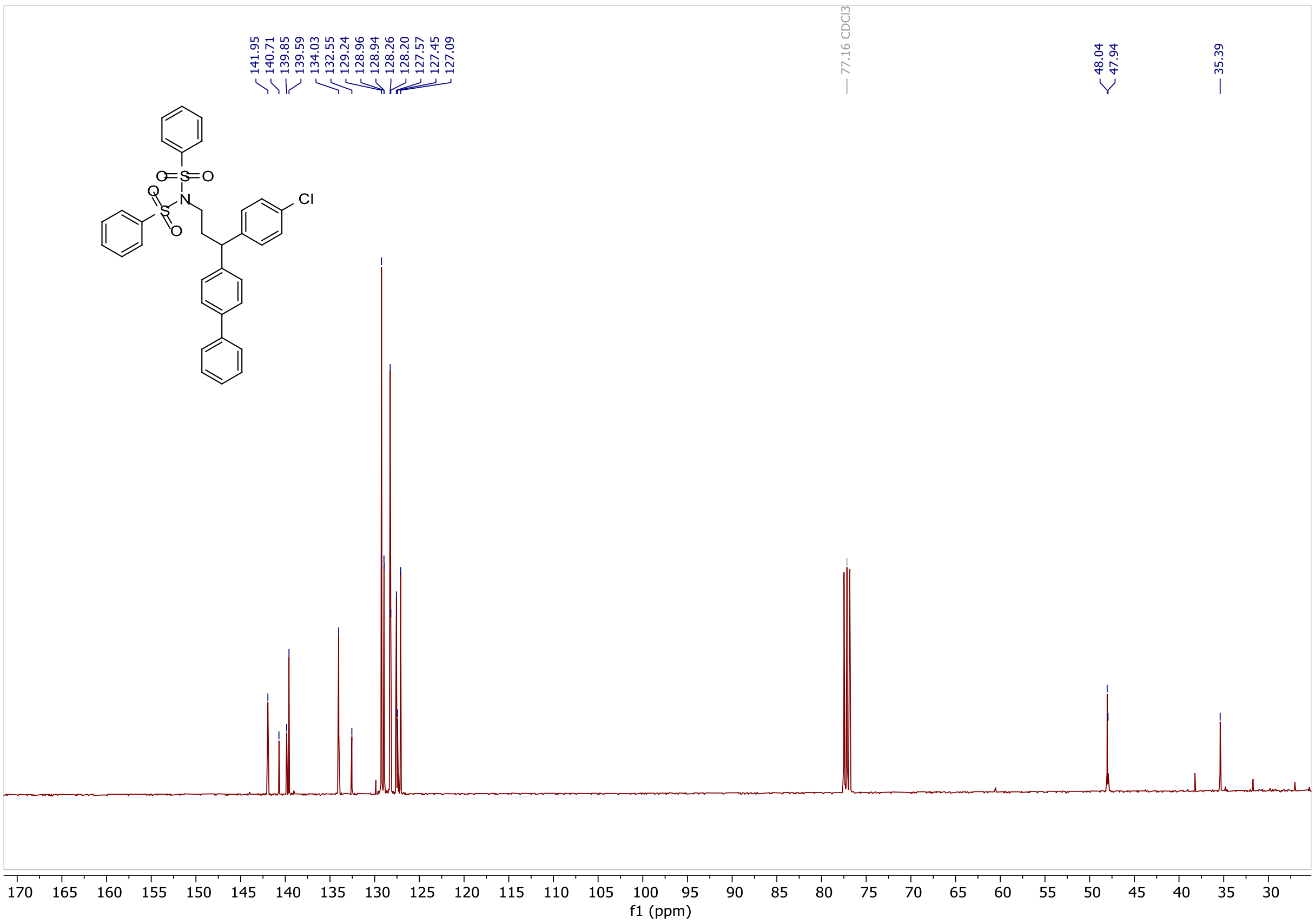

^{1}H NMR Spectrum of 7i (400 MHz, $CDCl_3$)

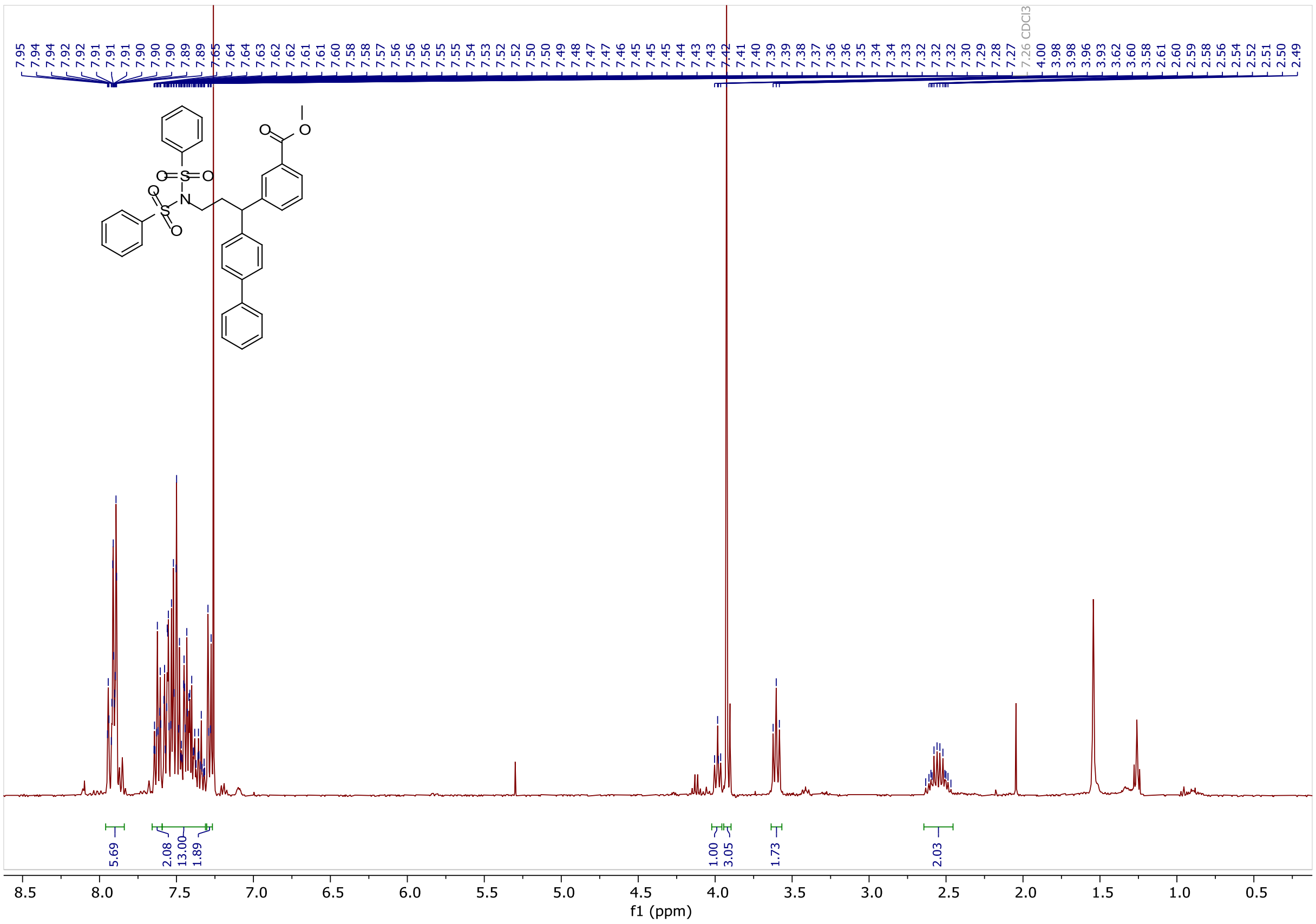

^{13}C NMR Spectrum of 7i (101 MHz, $CDCl_3$)

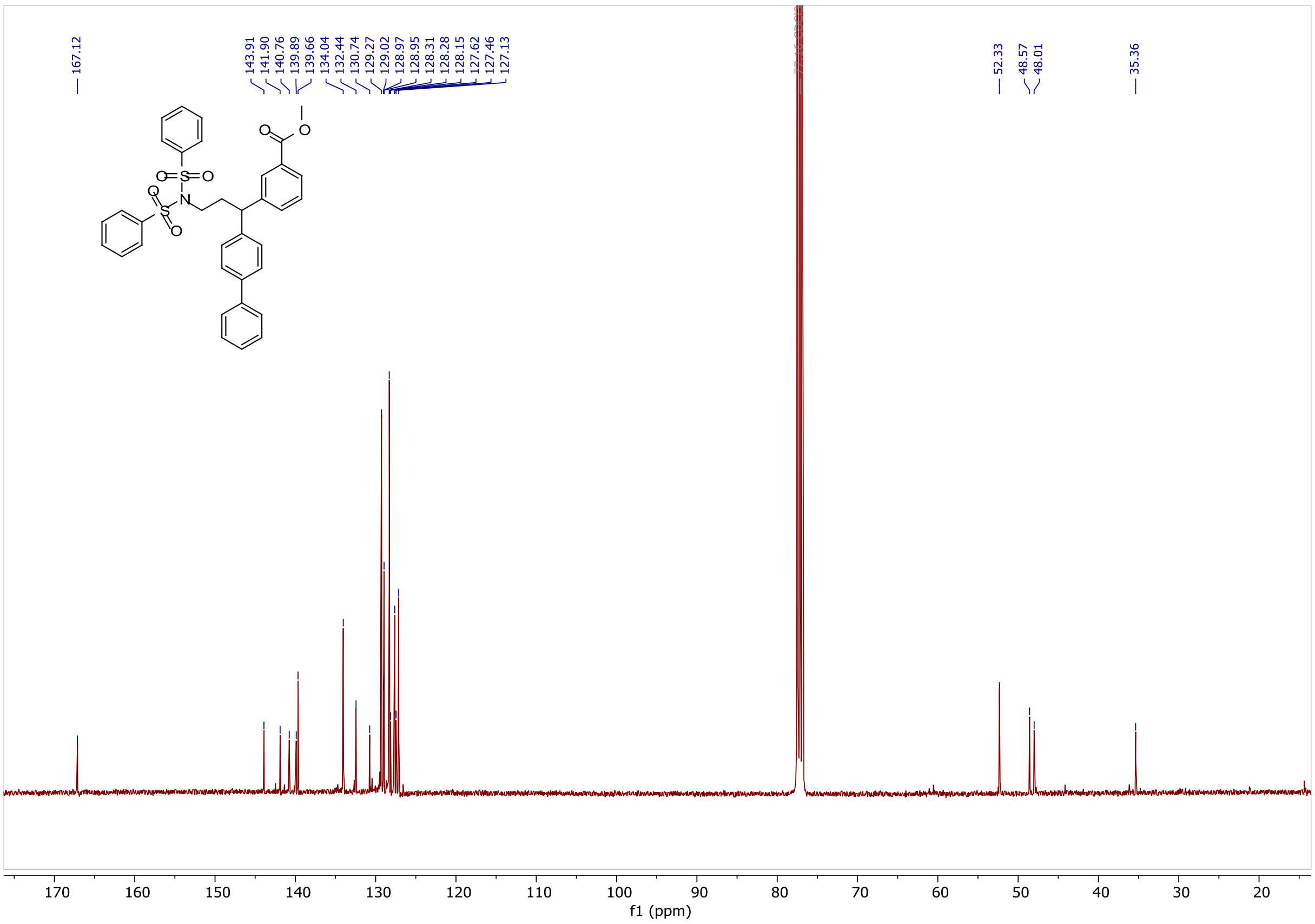

^{1}H NMR Spectrum of 7j (400 MHz, $CDCl_3$)

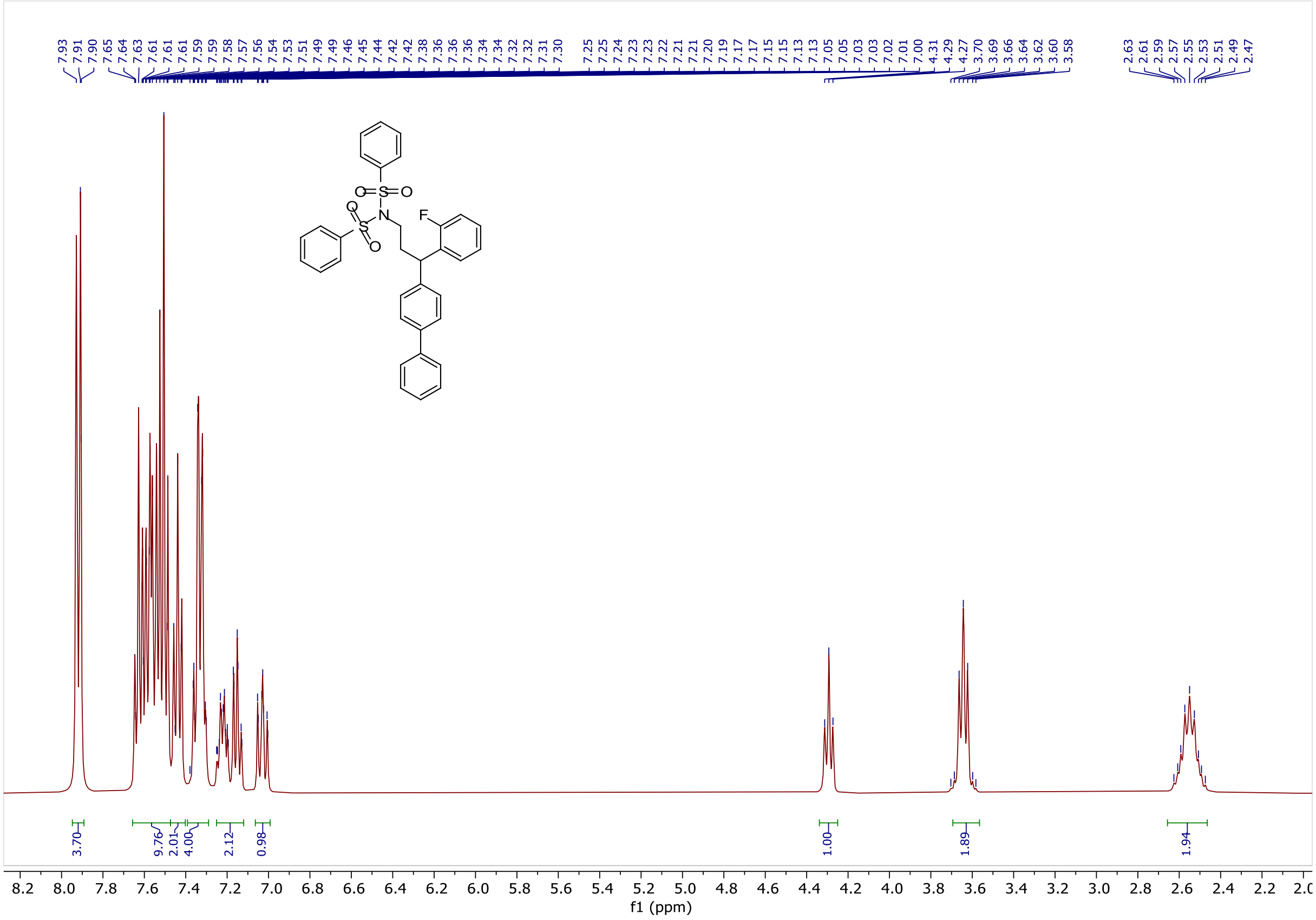

^{19}F NMR Spectrum of 7j (376 MHz, $CDCl_3$)

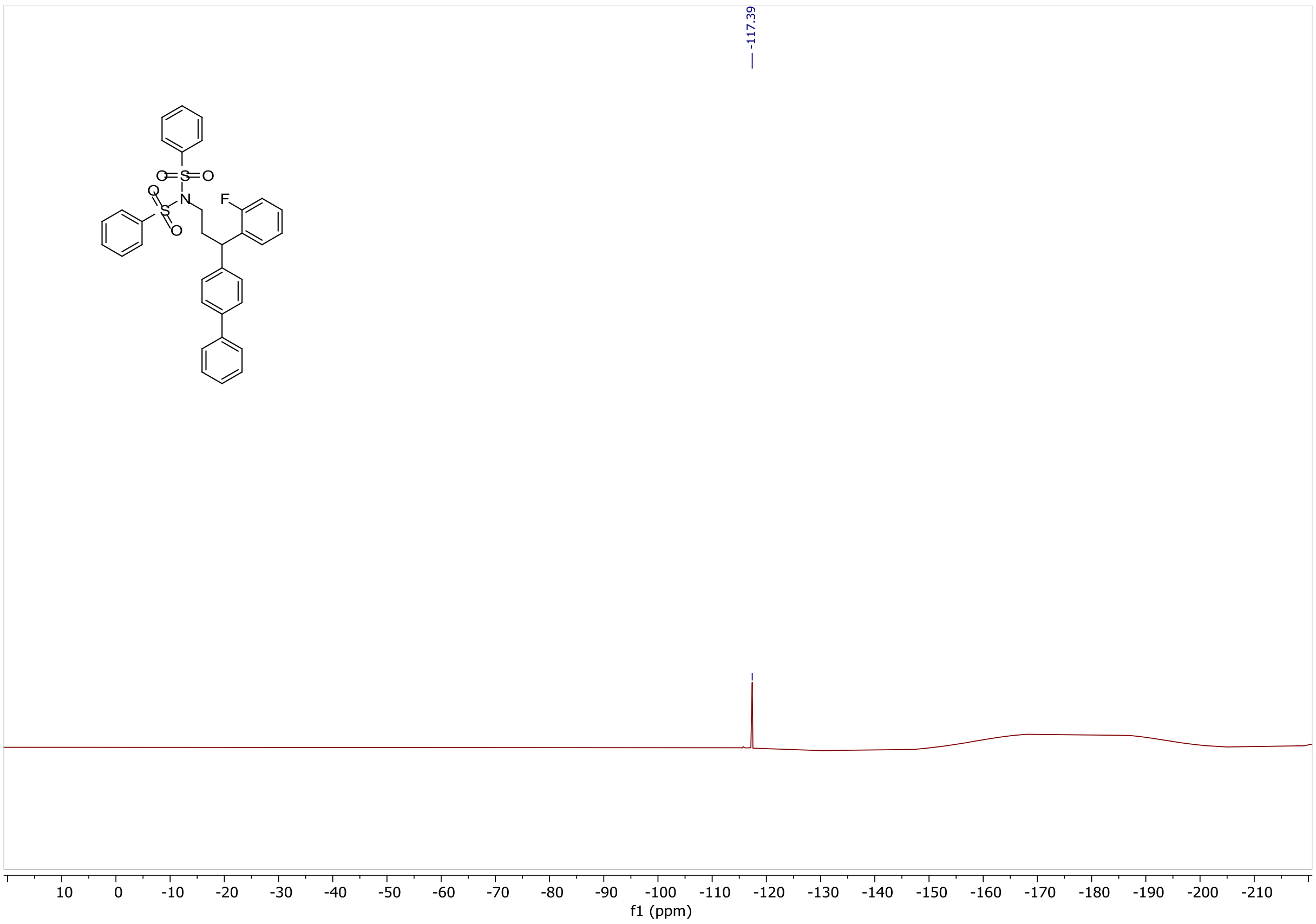

^{13}C NMR Spectrum of 7j (101 MHz, $CDCl_3$)

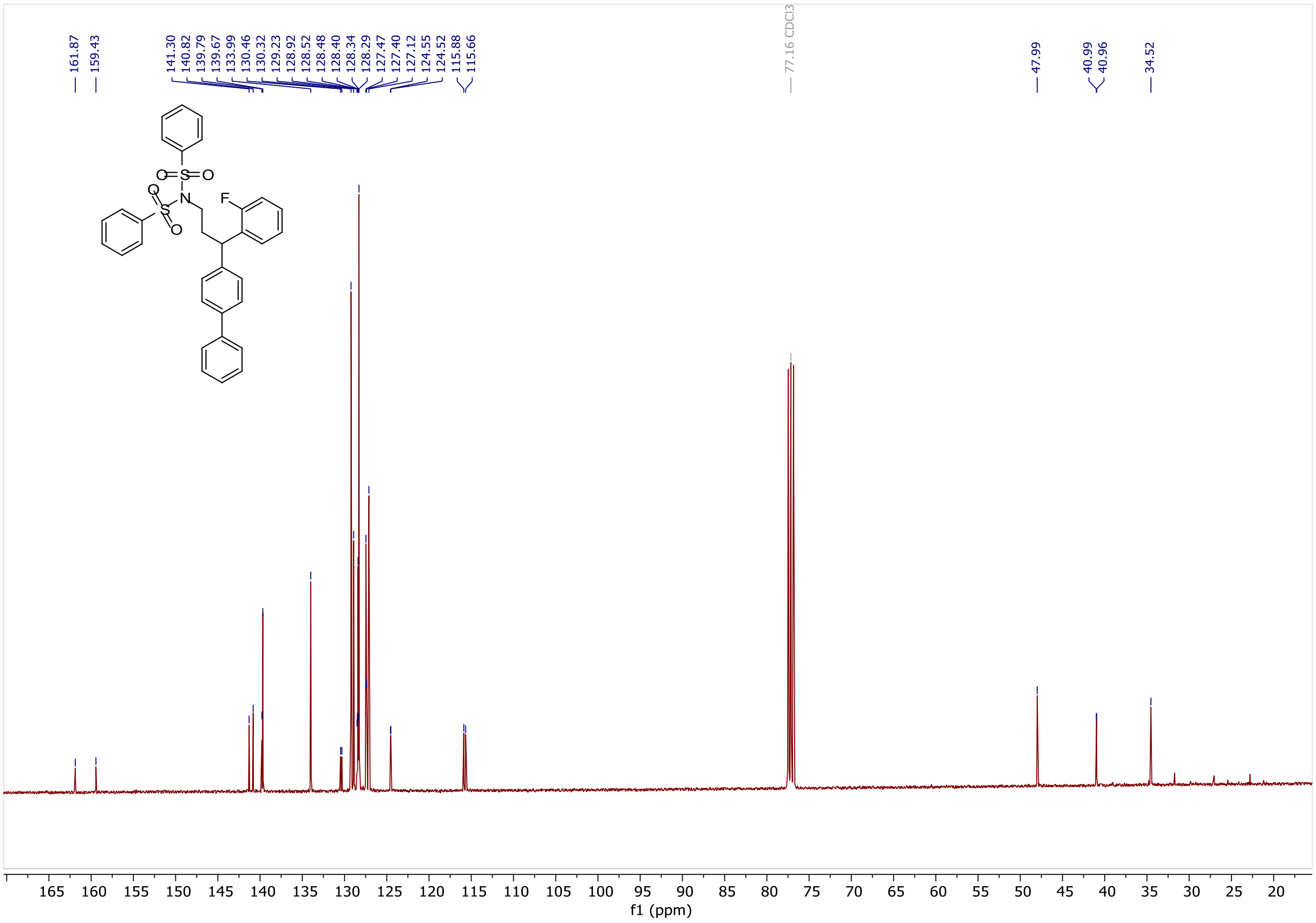

^{1}H NMR Spectrum of 7k (400 MHz, $CDCl_3$)

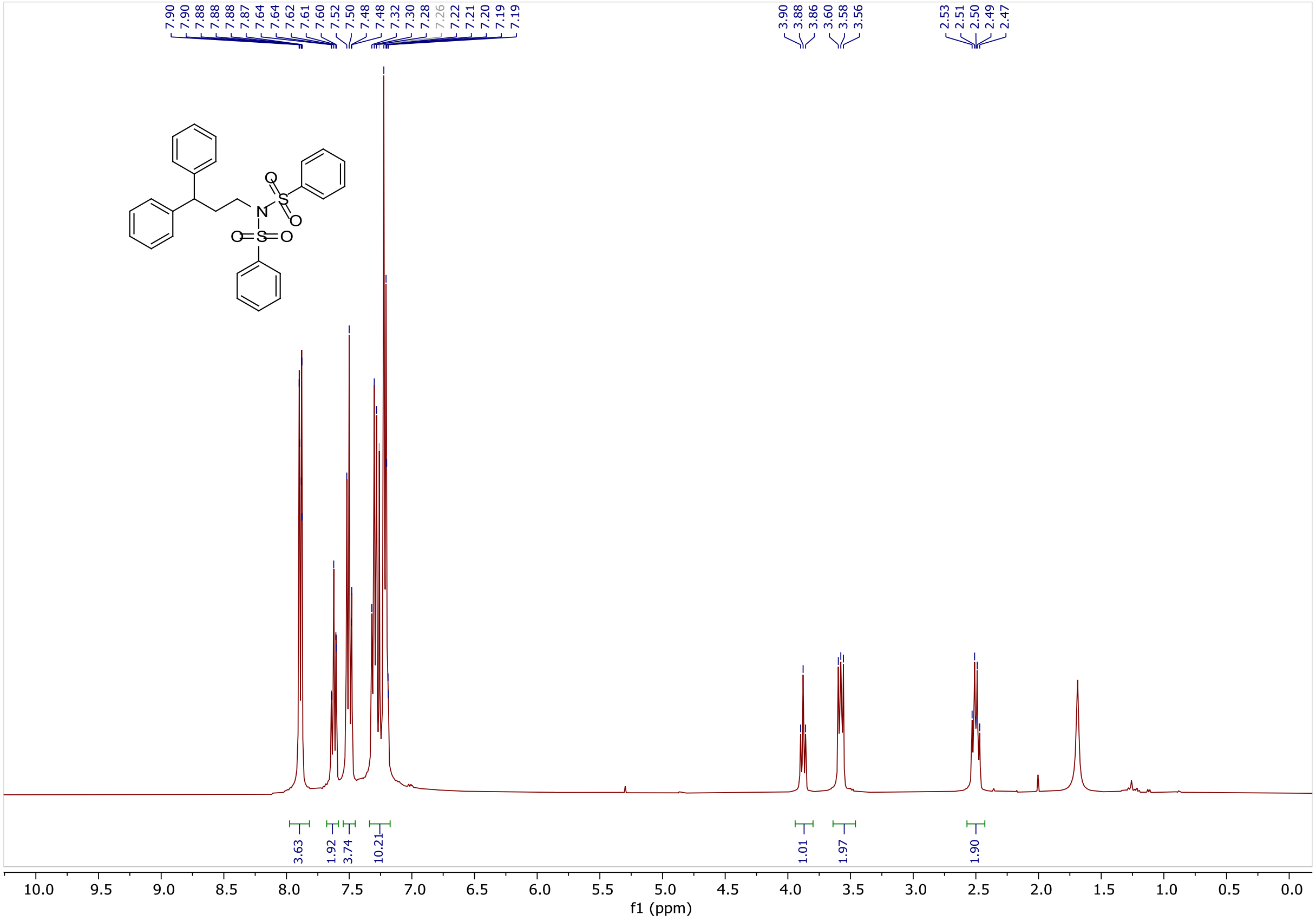

^{13}C NMR Spectrum of 7k (101 MHz, $CDCl_3$)

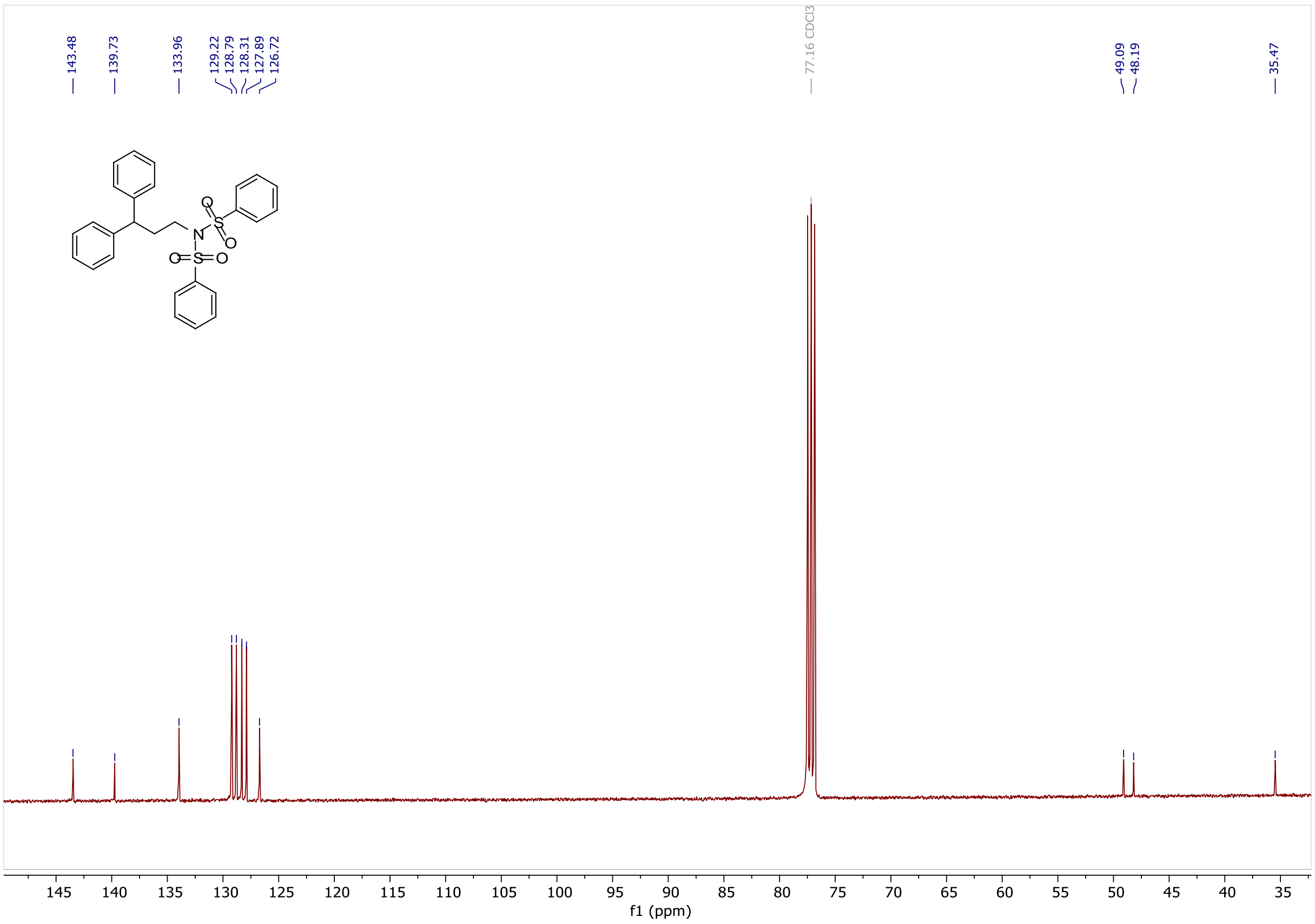

^{1}H NMR Spectrum of *m*-7l (400 MHz, CDCl$_3$)

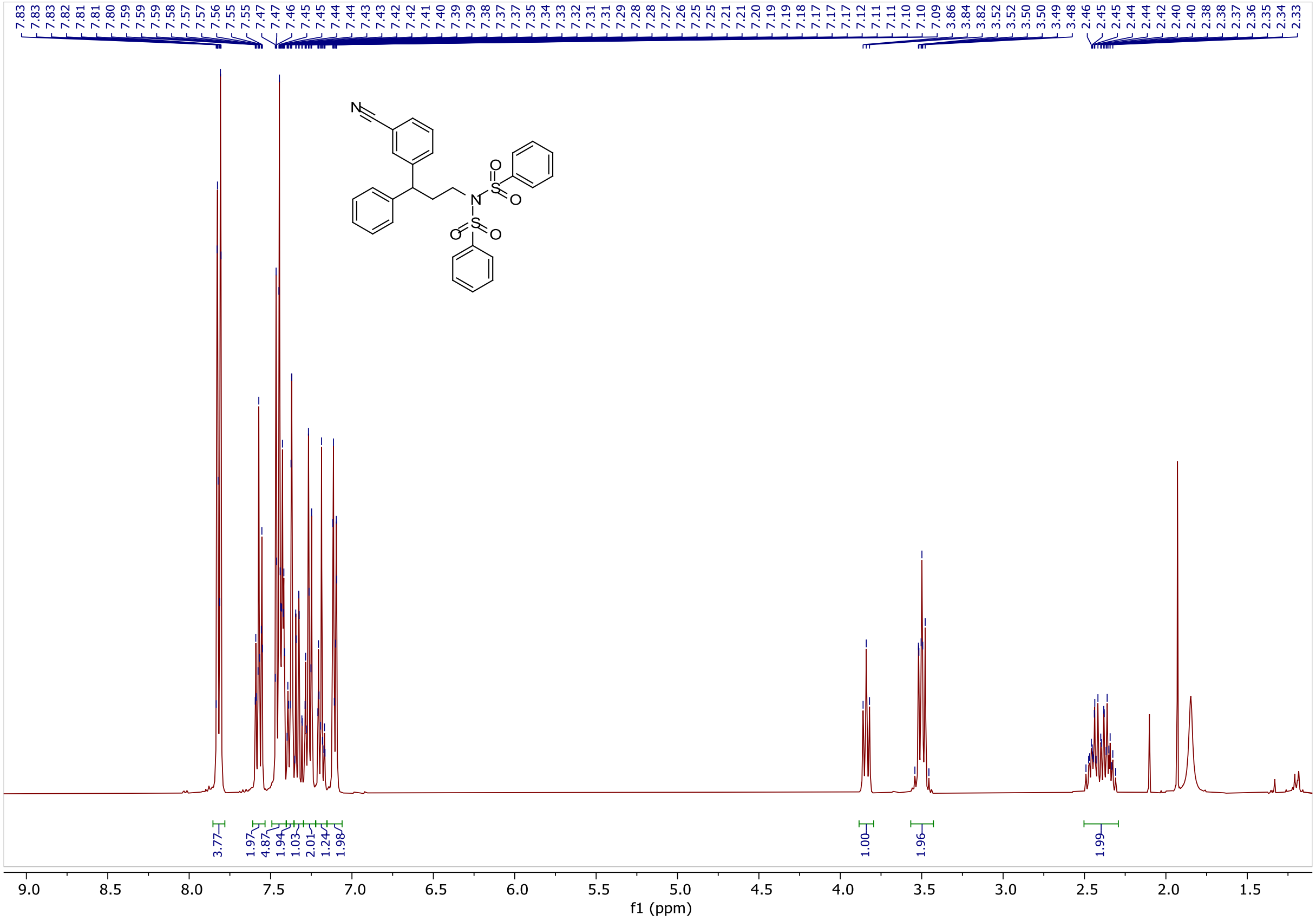

^{13}C NMR Spectrum of *m*-7l (101 MHz, CDCl$_3$)

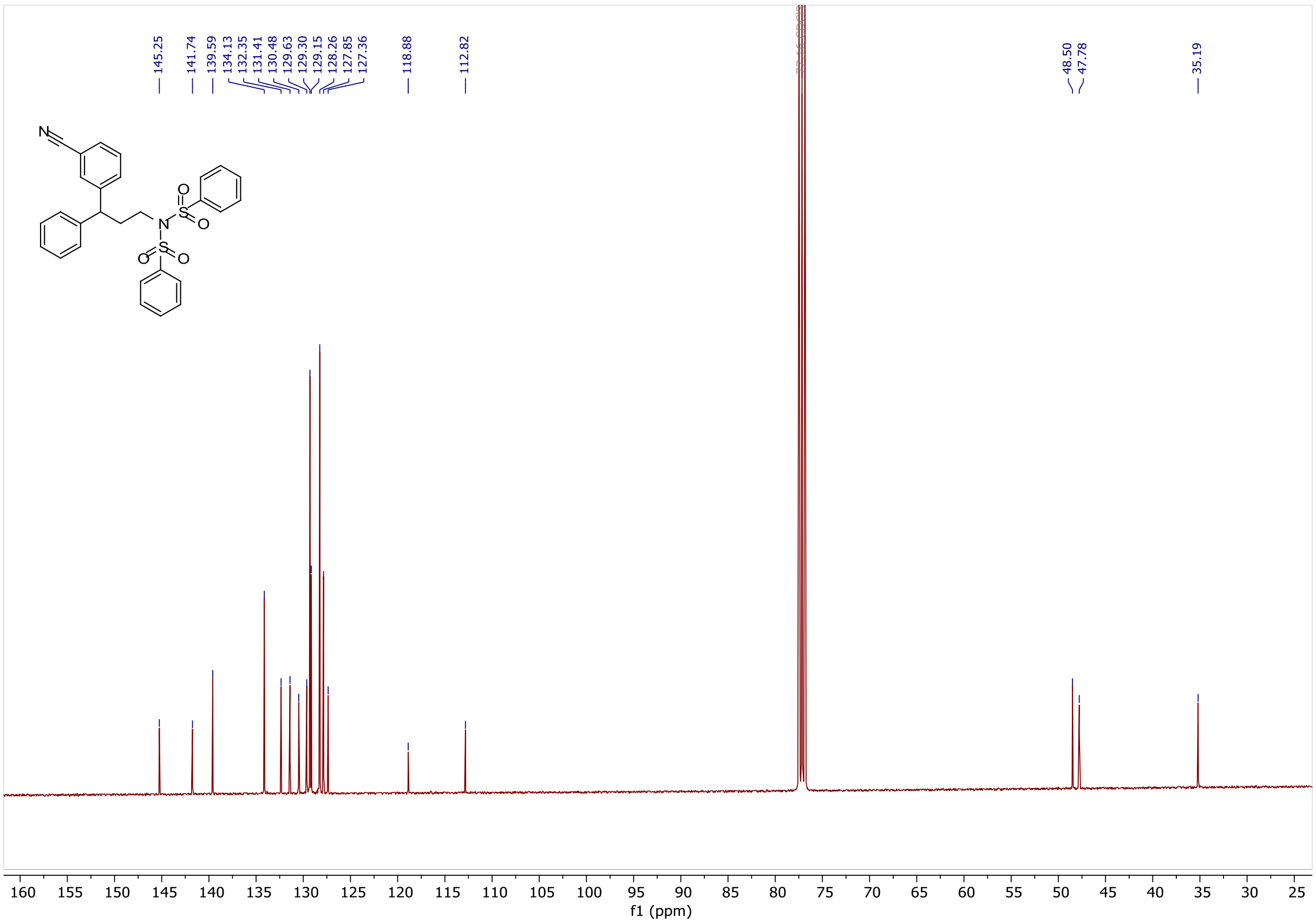

^{1}H NMR Spectrum of *p*-7l (400 MHz, $CDCl_3$)

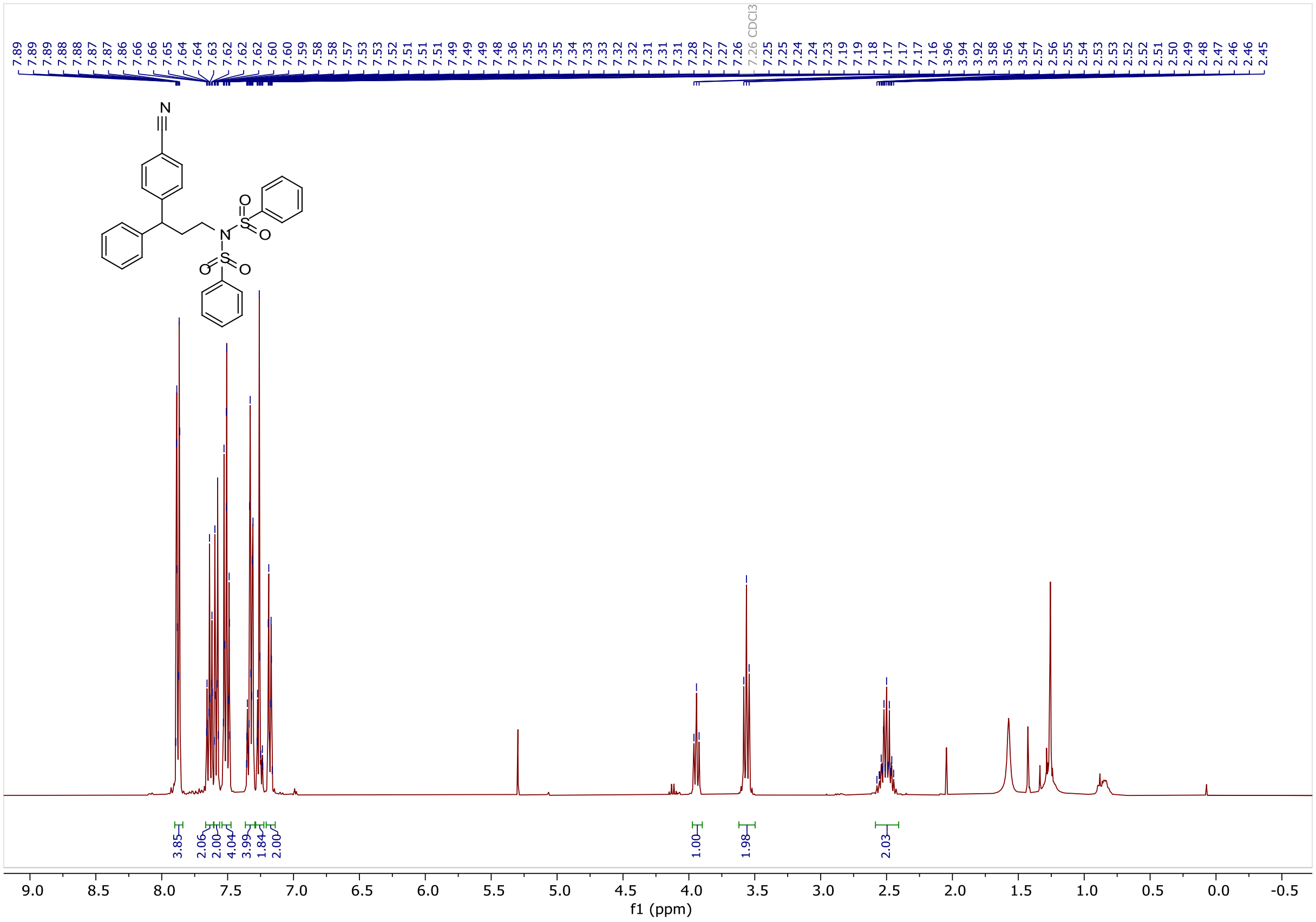

^{13}C NMR Spectrum of *p*-7l (101 MHz, $CDCl_3$)

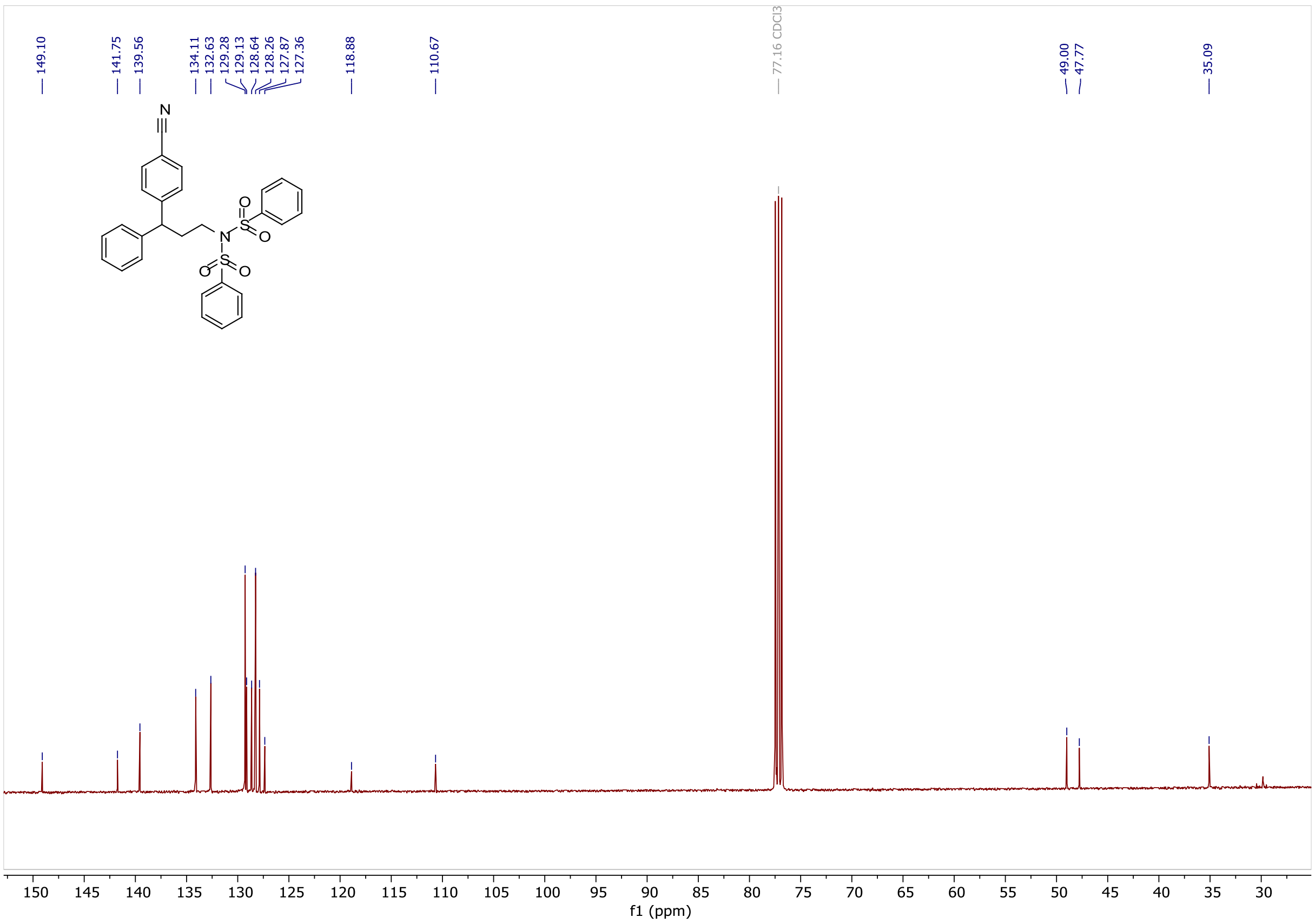

^{1}H NMR Spectrum of *o*-7m (400 MHz, $CDCl_3$)

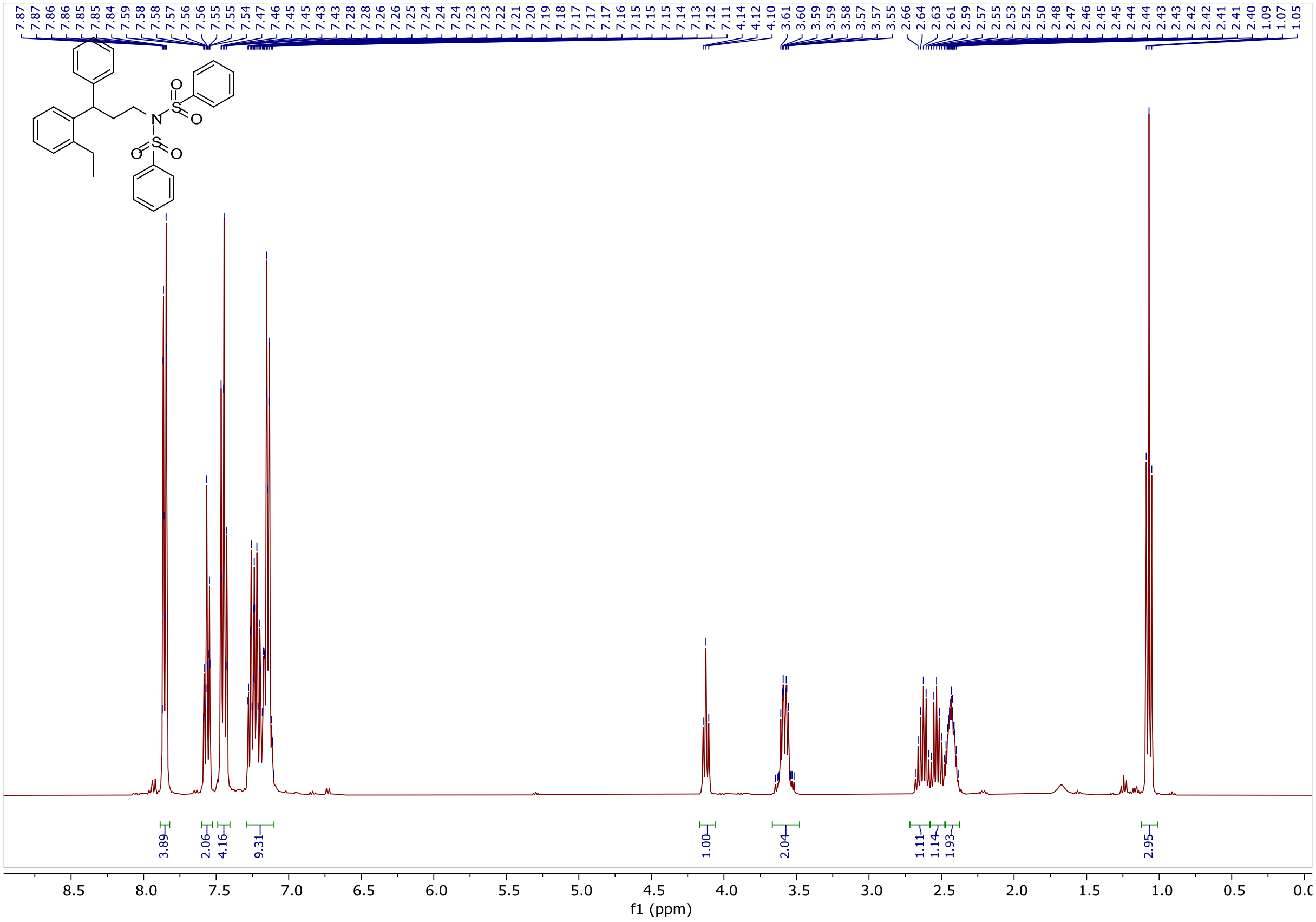

^{13}C NMR Spectrum of *o*-7m (101 MHz, $CDCl_3$)

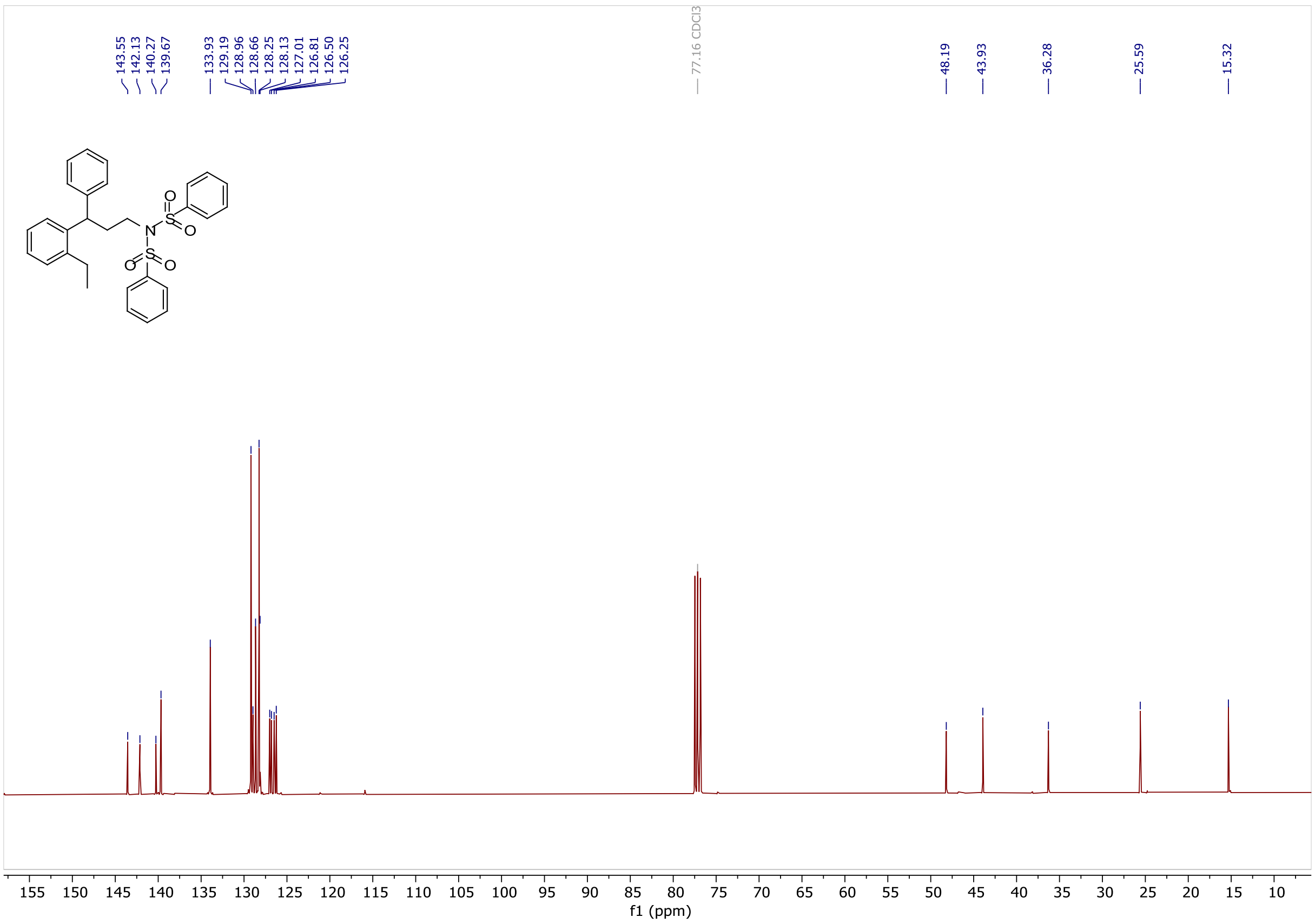

^{1}H NMR Spectrum of *m*-7m (400 MHz, CDCl$_3$)

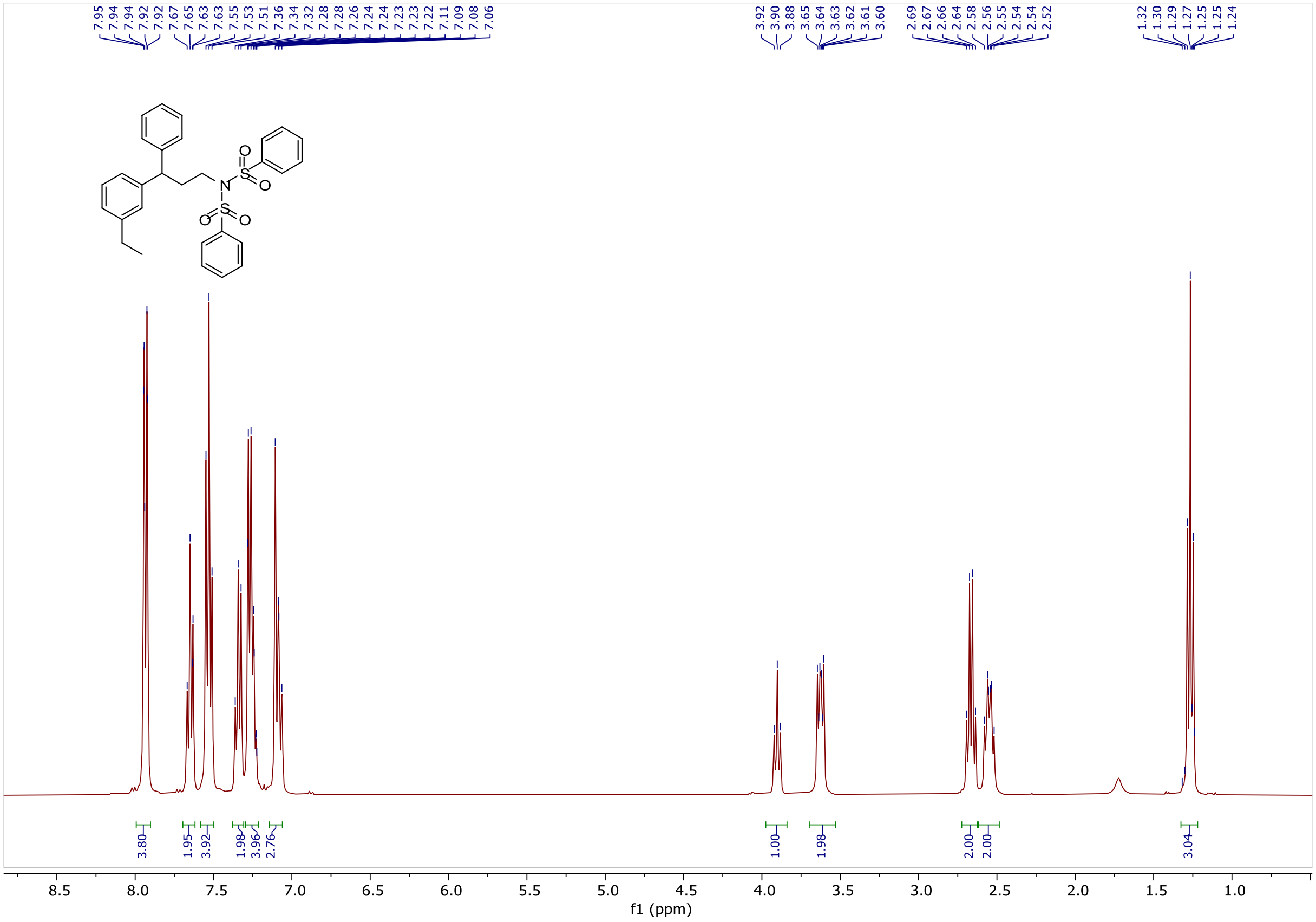

^{13}C NMR Spectrum of *m*-7m (101 MHz, CDCl$_3$)

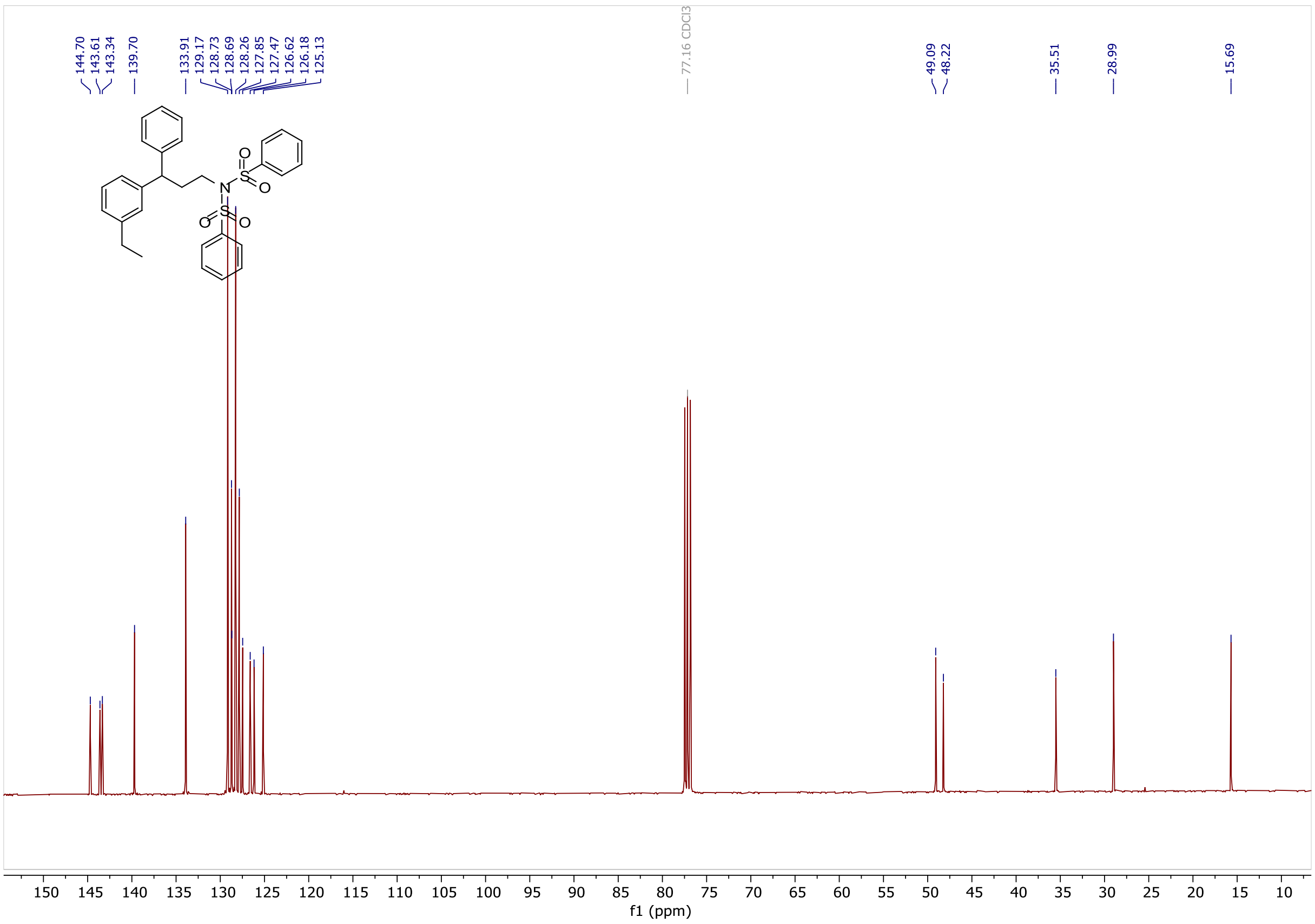

^{1}H NMR Spectrum of *p*-7m (400 MHz, $CDCl_3$)

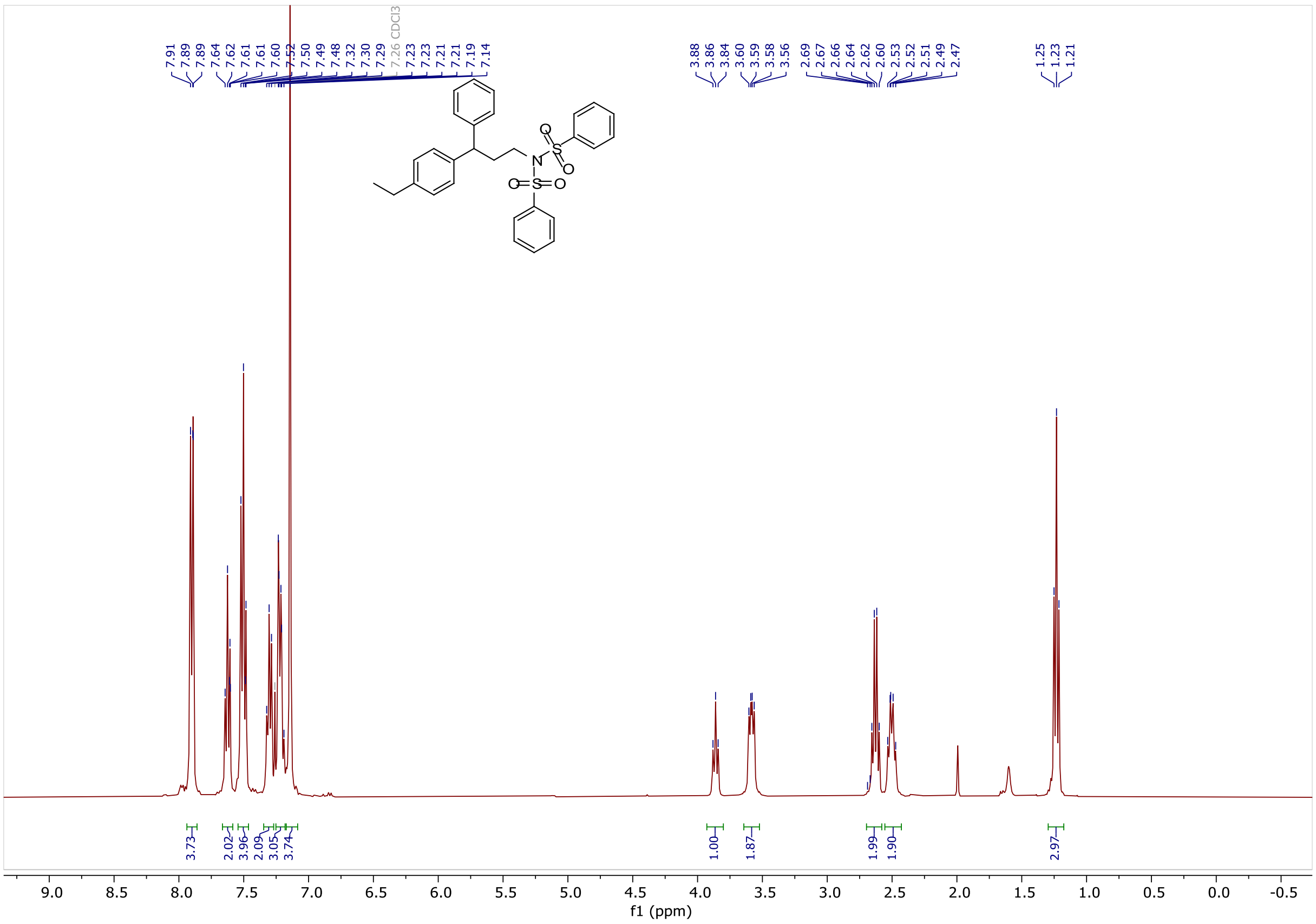

^{13}C NMR Spectrum of *p*-7m (101 MHz, $CDCl_3$)

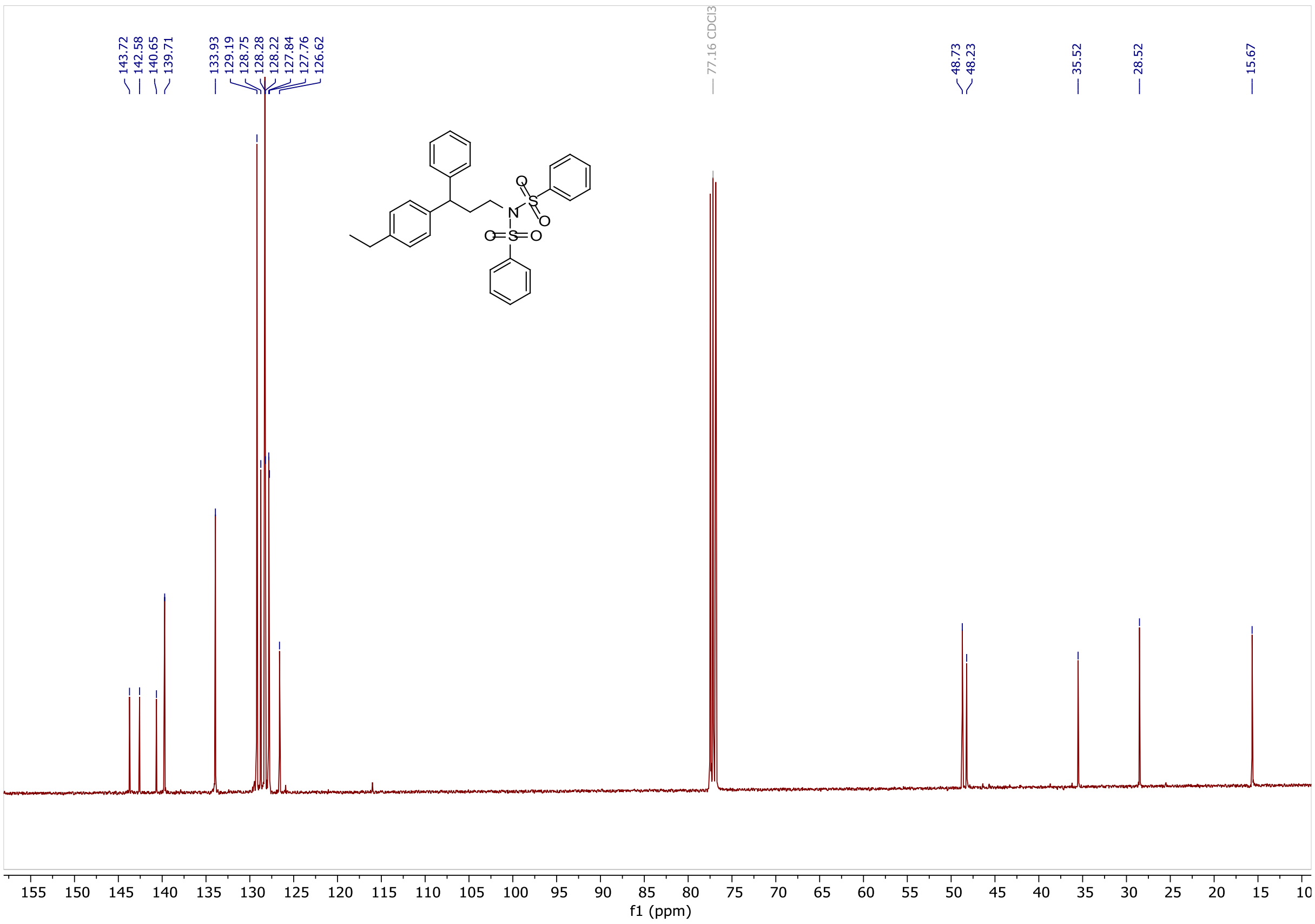

1H NMR Spectrum of *m*-7n (400 MHz, $CDCl_3$)

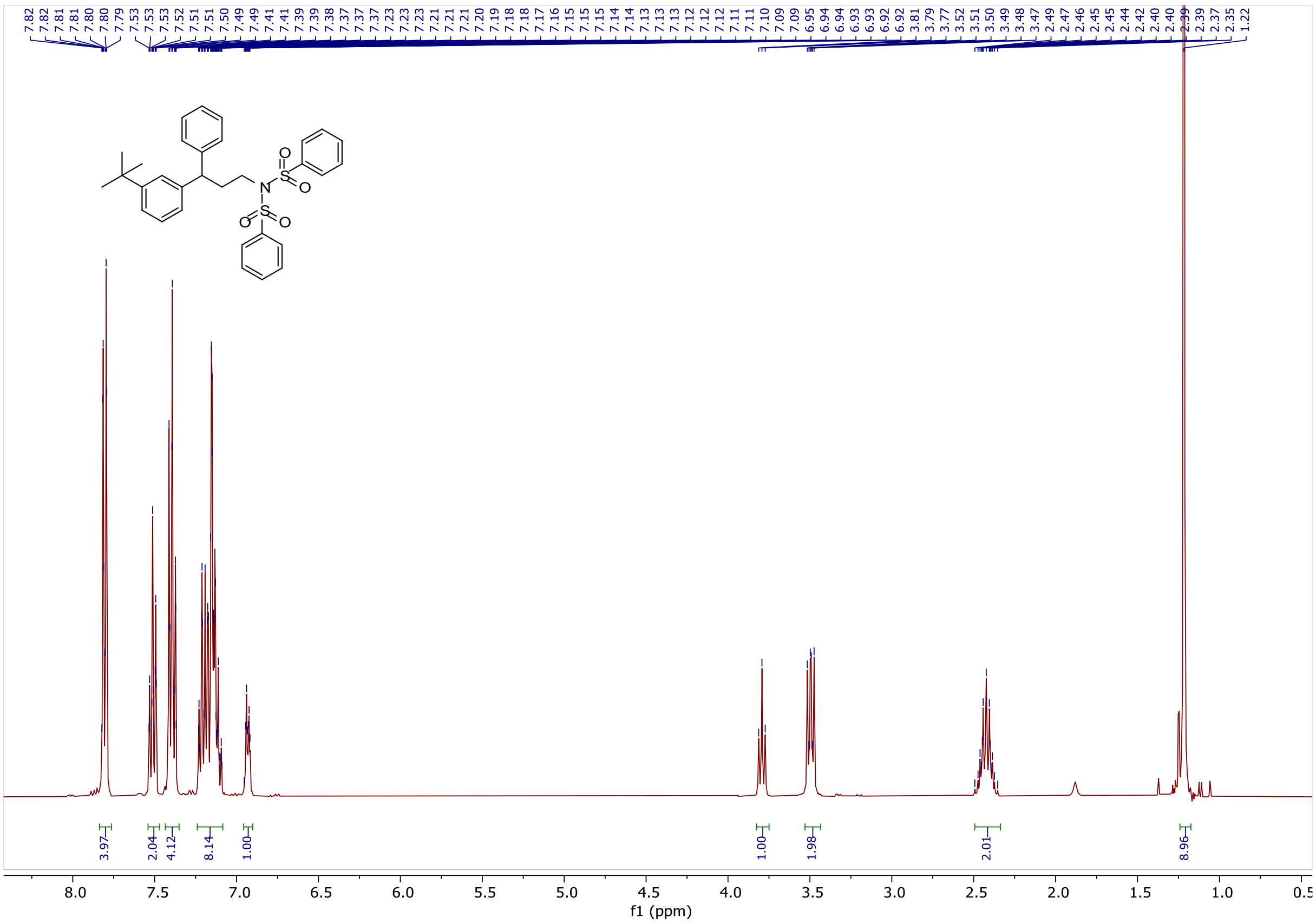

13C NMR Spectrum of *m*-7n (101 MHz, $CDCl_3$)

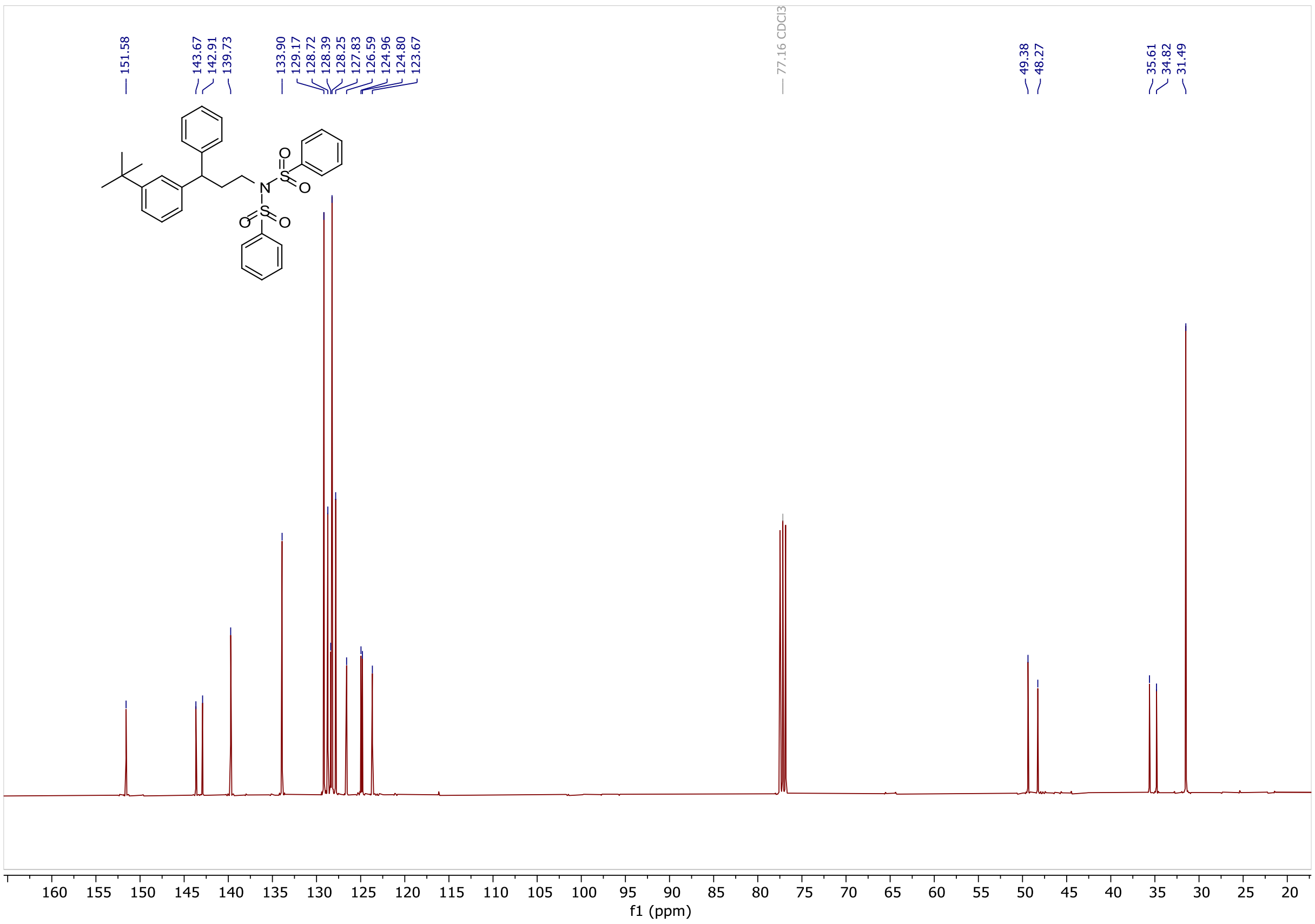

^{1}H NMR Spectrum of *p*-7n (400 MHz, $CDCl_3$)

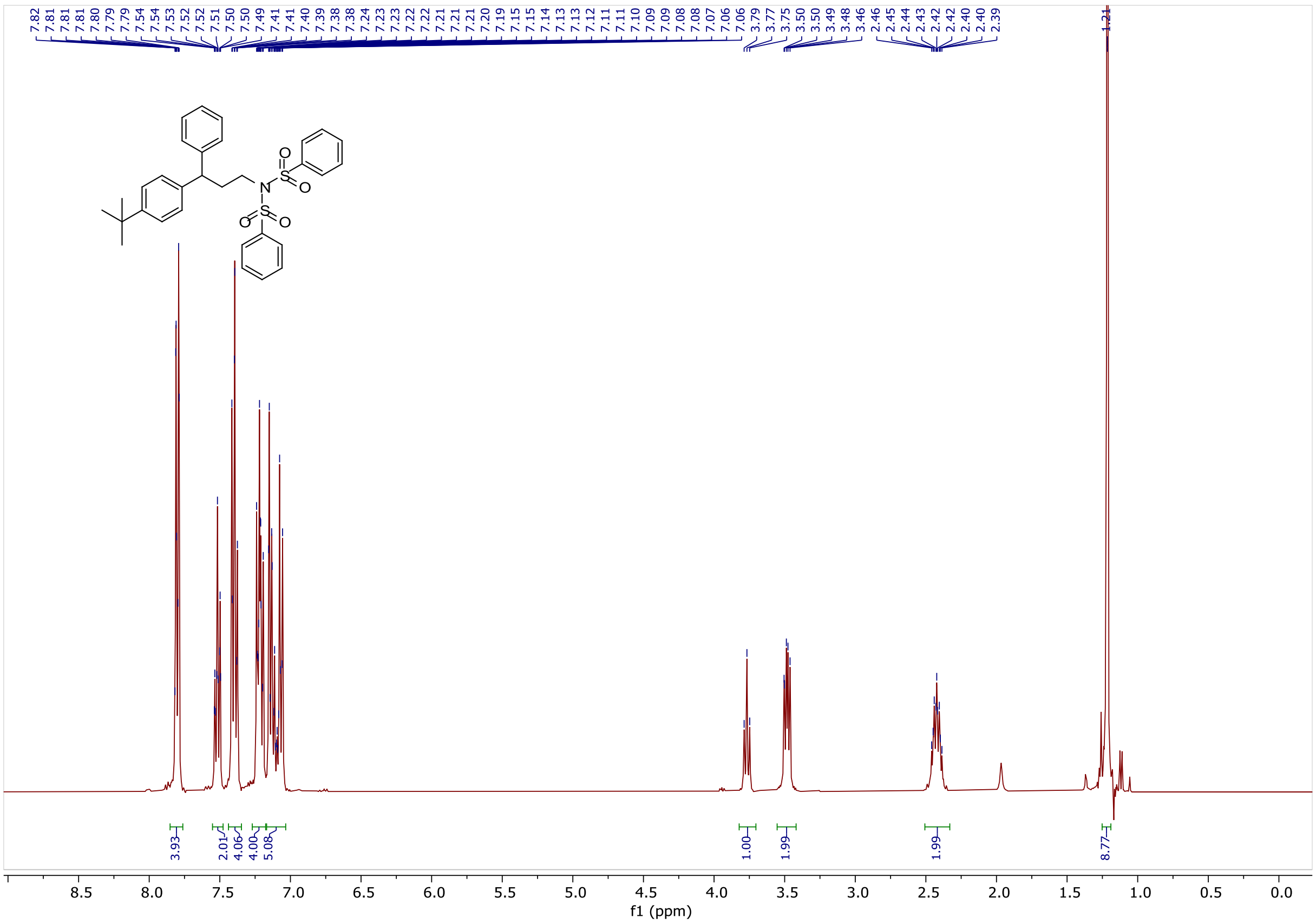

^{13}C NMR Spectrum of *p*-7n (101 MHz, $CDCl_3$)

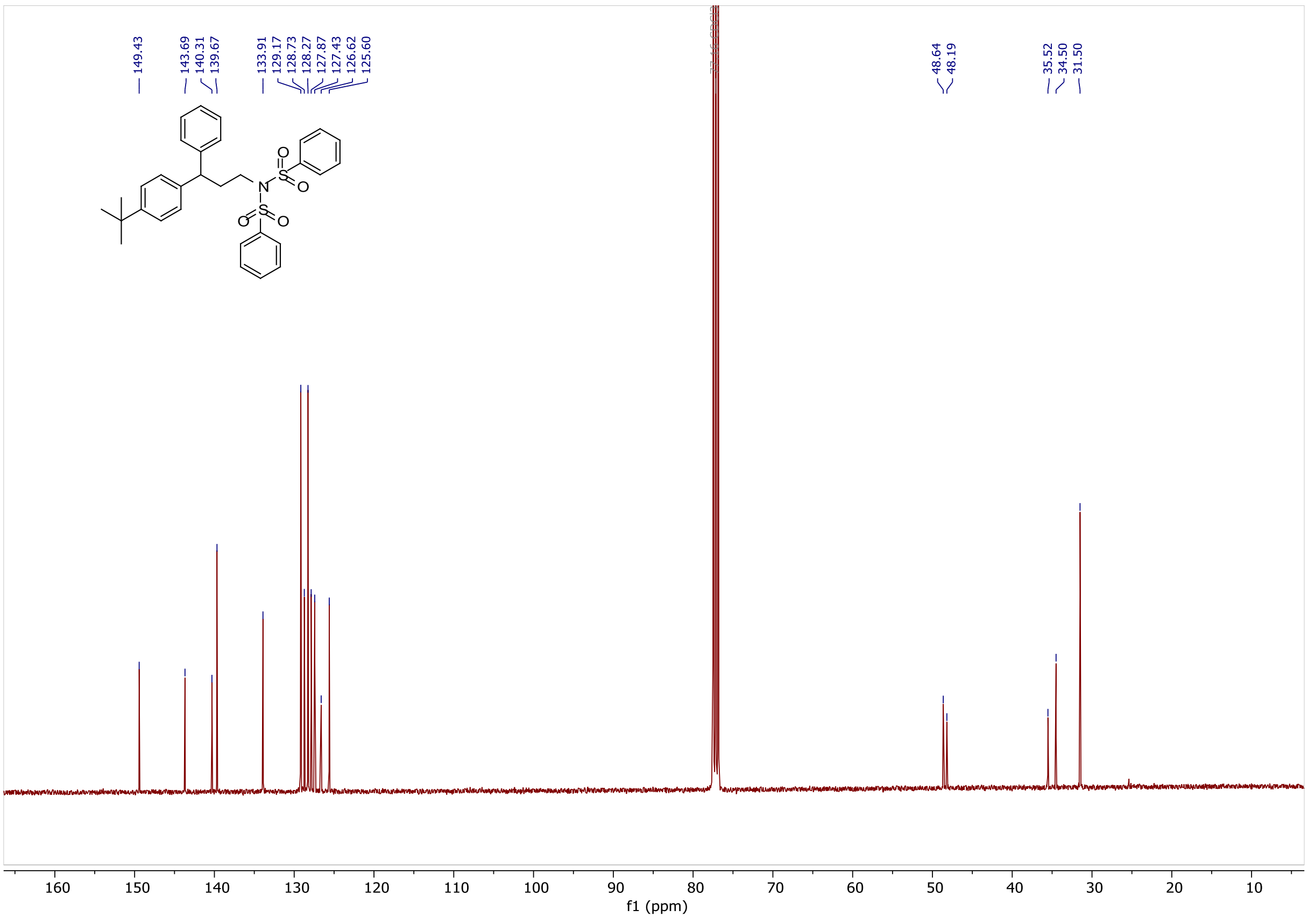

¹H NMR Spectrum of 7o (400 MHz, $CDCl_3$)

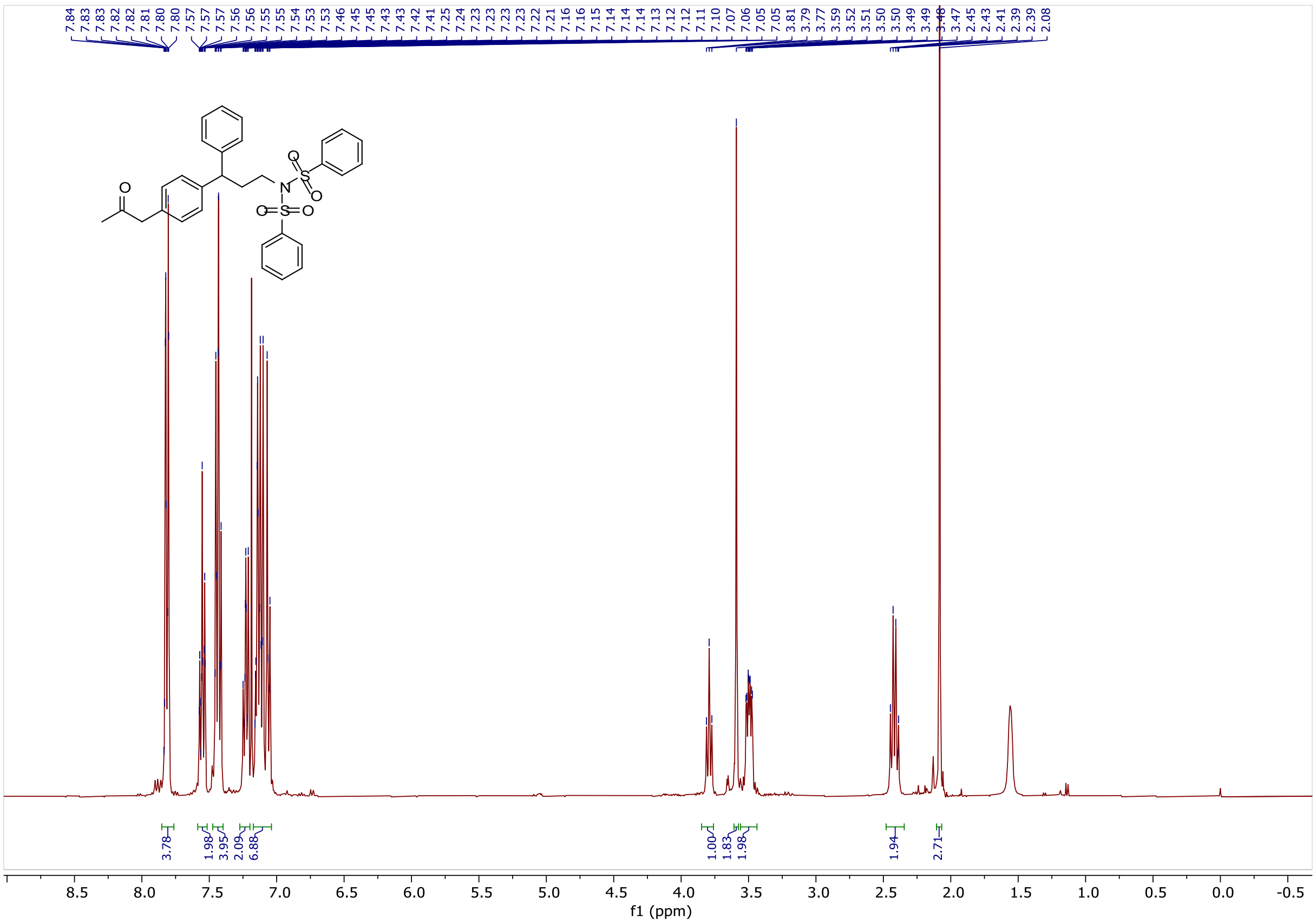

¹³C NMR Spectrum of 7o (101 MHz, $CDCl_3$)

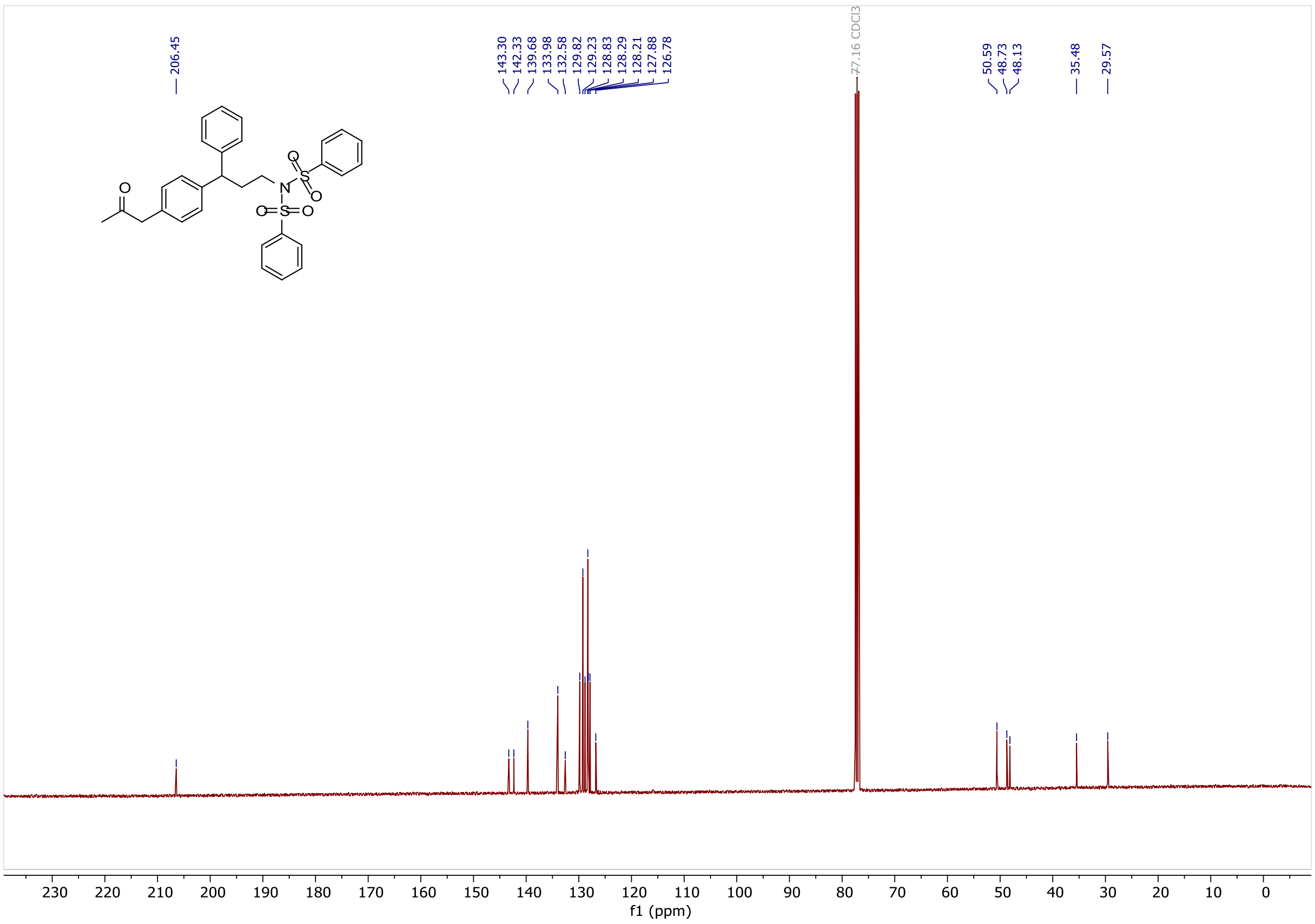

^{1}H NMR Spectrum of *o*-7p (400 MHz, $CDCl_3$)

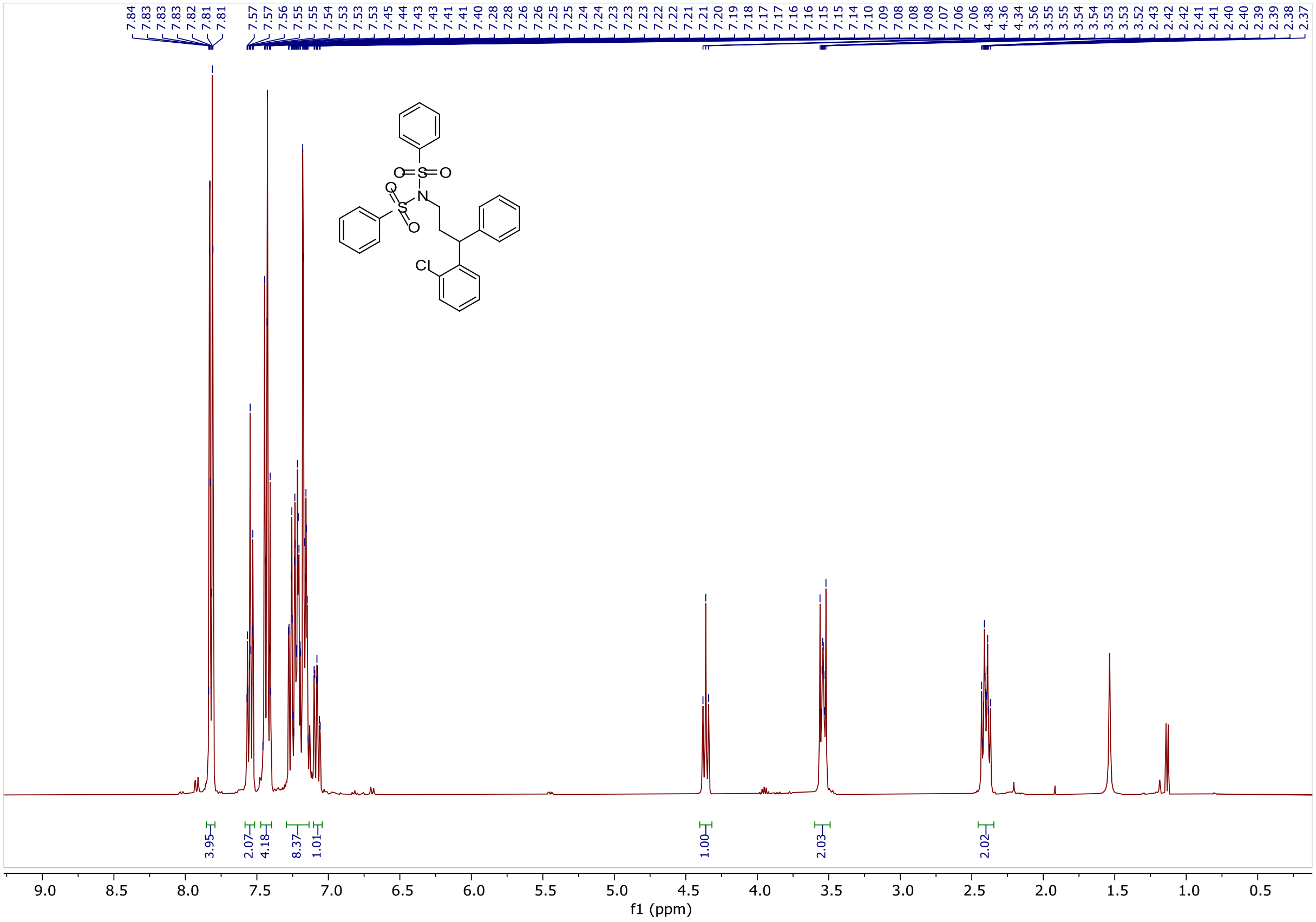

^{13}C NMR Spectrum of *o*-7p (101 MHz, $CDCl_3$)

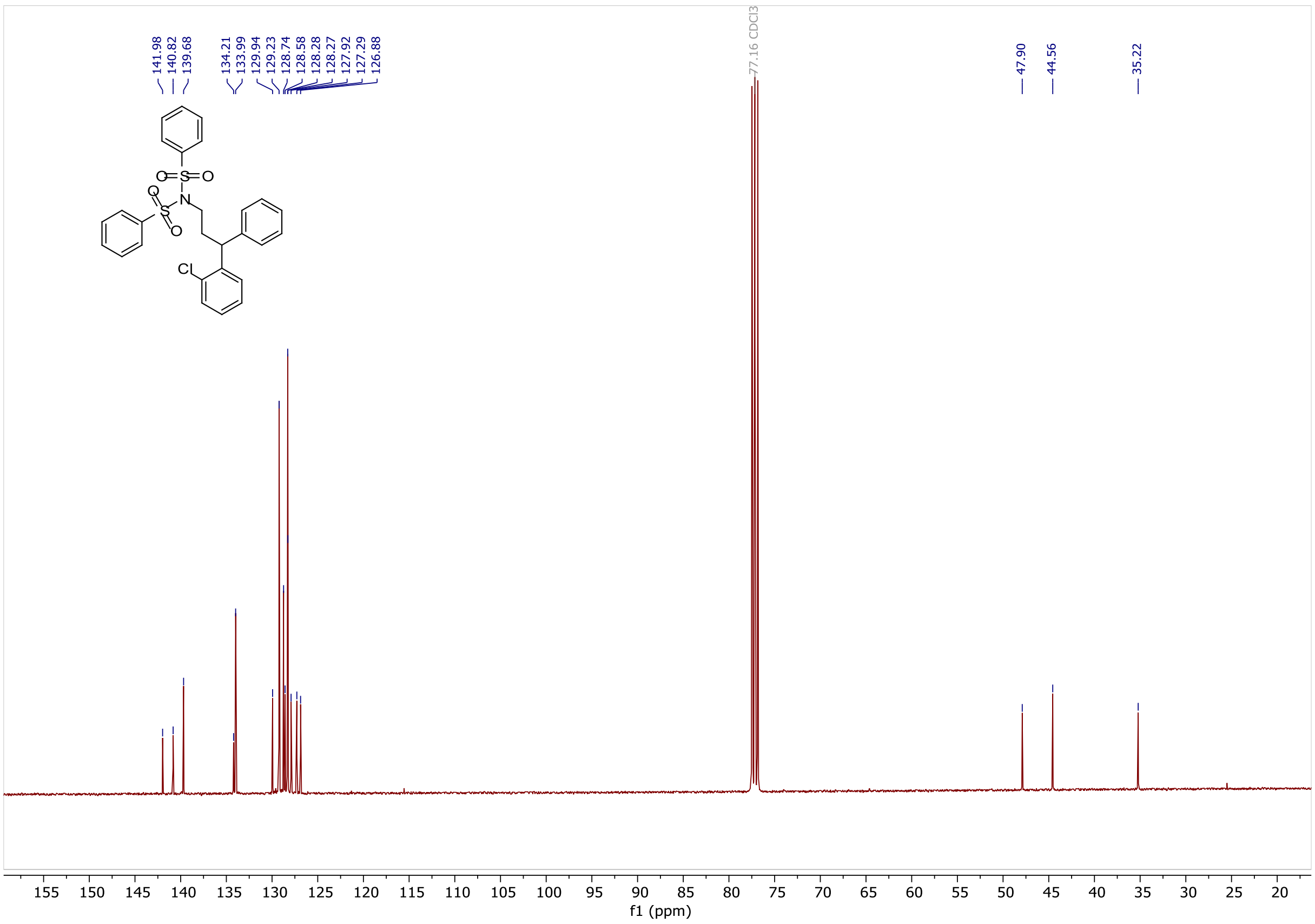

^{1}H NMR Spectrum of *m*-7p (400 MHz, CDCl3)

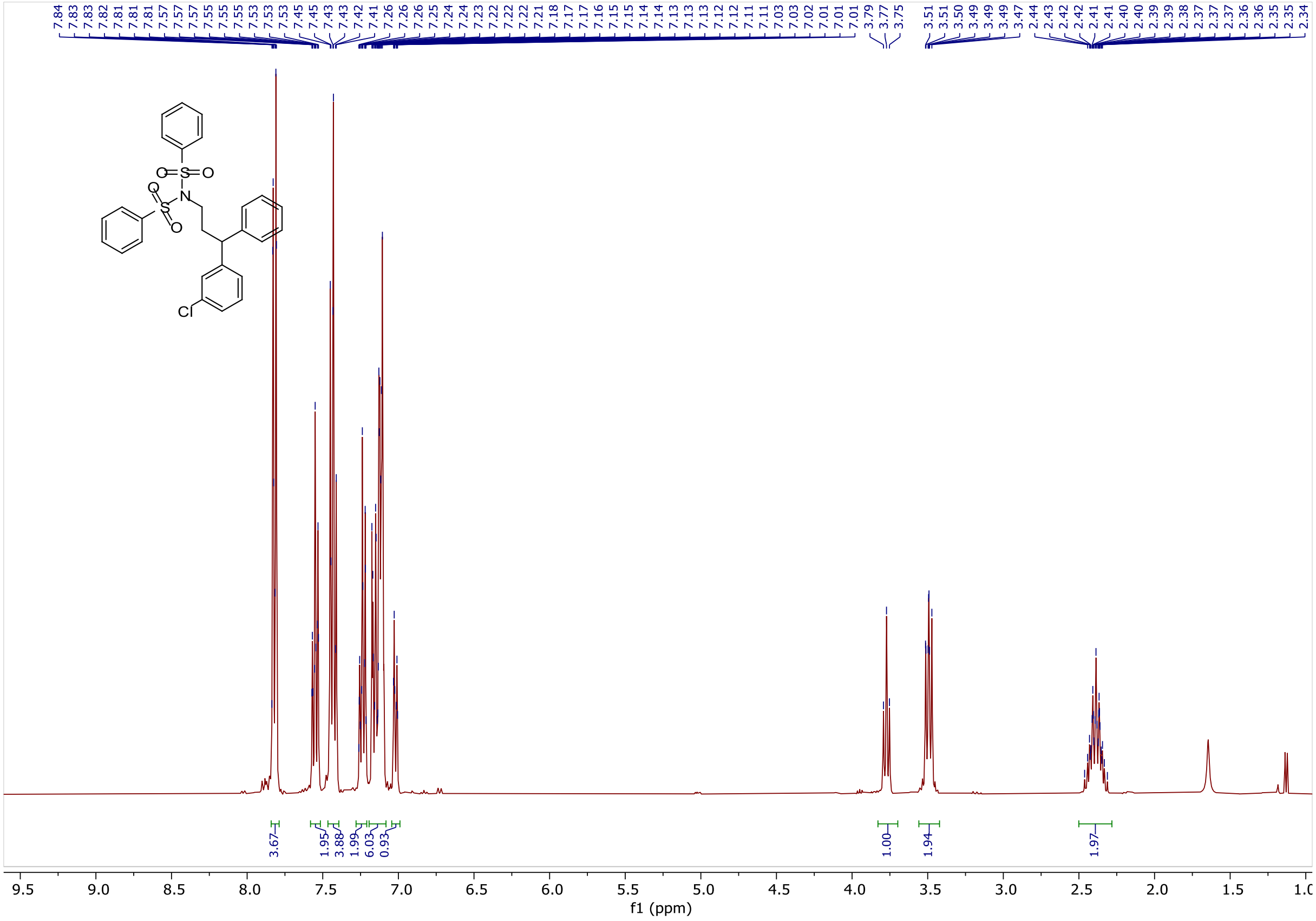

^{13}C NMR Spectrum of *m*-7p (101 MHz, CDCl3)

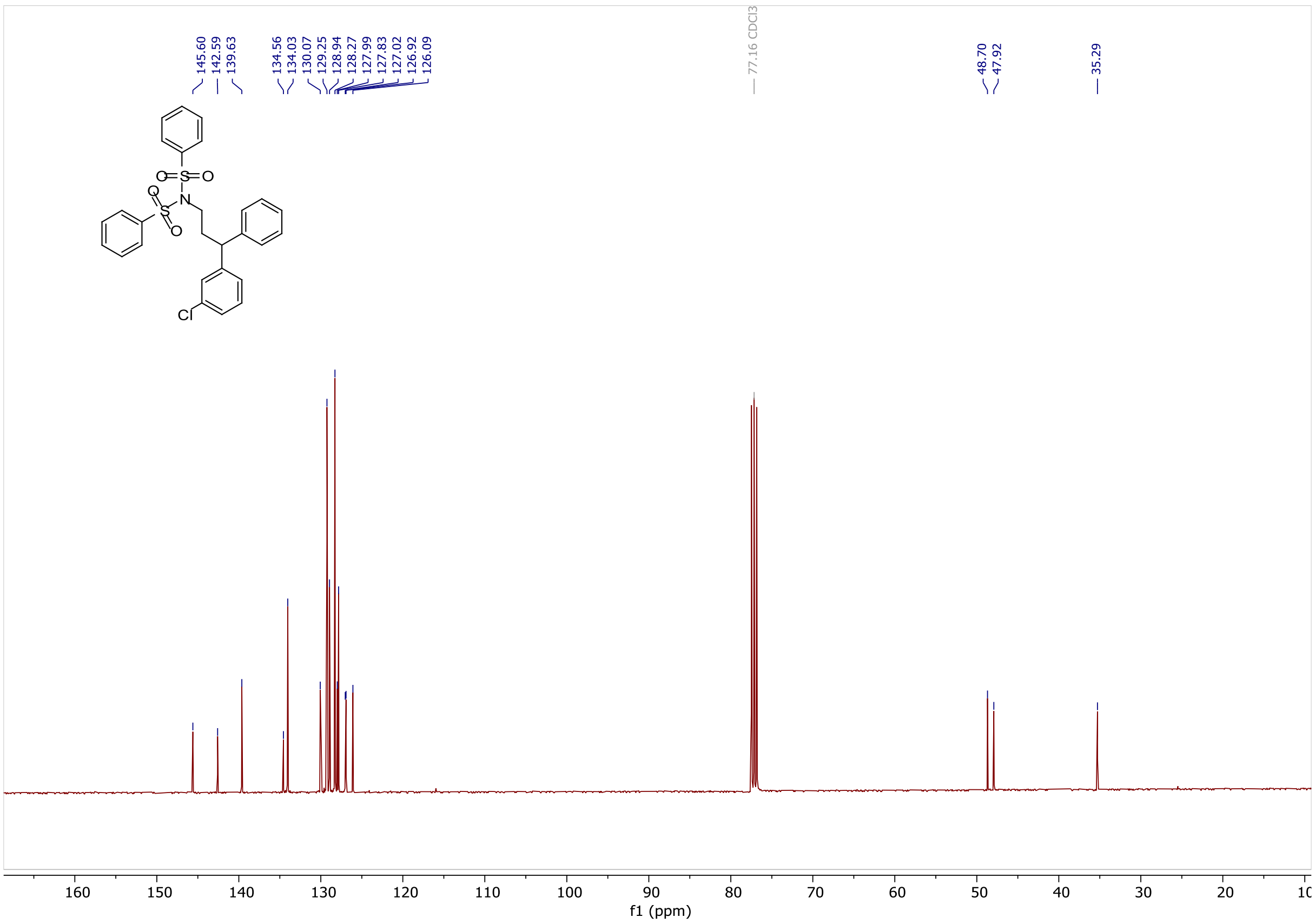

^{1}H NMR Spectrum of *p*-7p (400 MHz, $CDCl_3$)

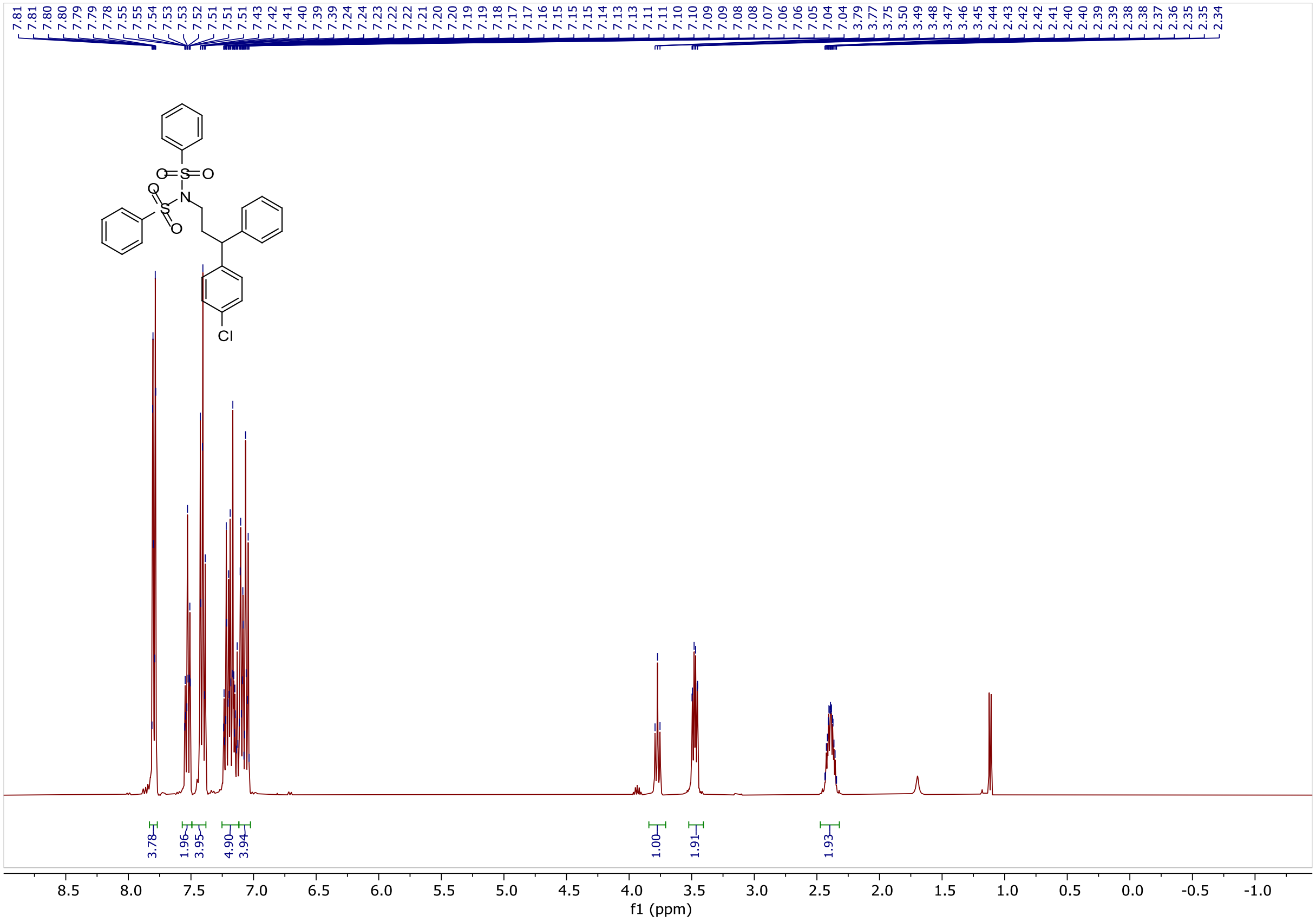

^{13}C NMR Spectrum of *p*-7p (101 MHz, $CDCl_3$)

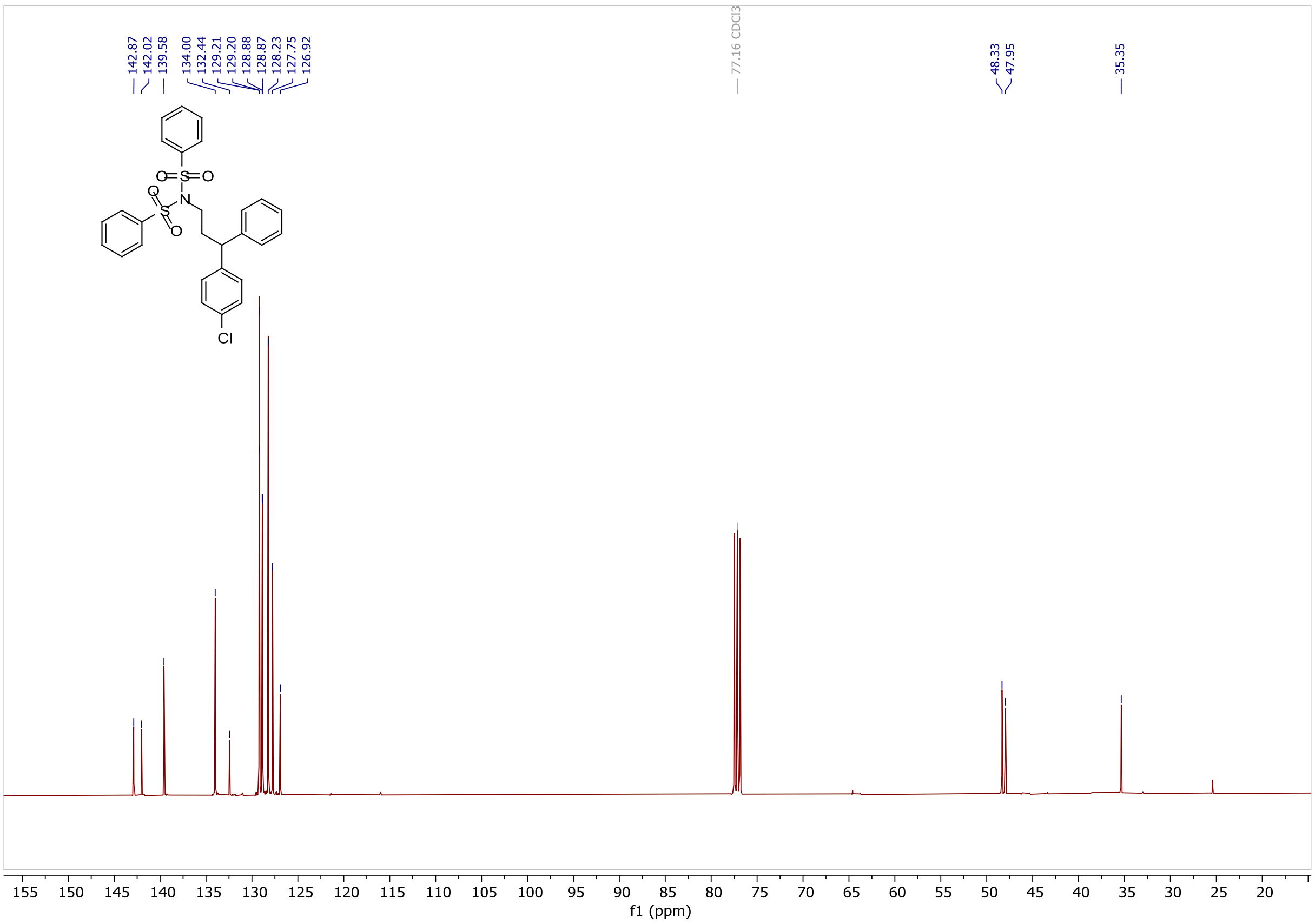

¹H NMR Spectrum of *o*-7q (400 MHz, CDCl3)

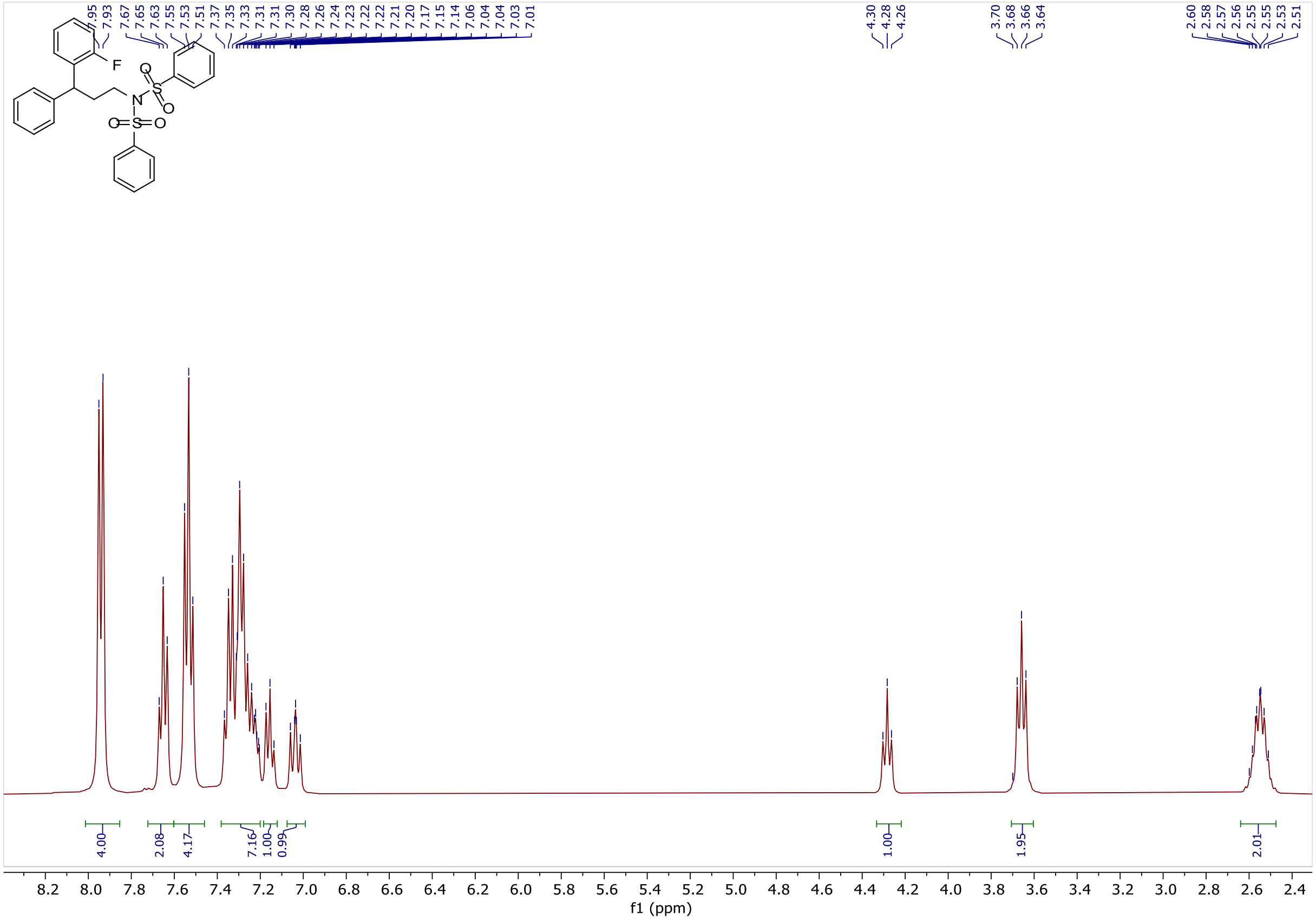

¹⁹F NMR Spectrum of *o*-7q (376 MHz, CDCl3)

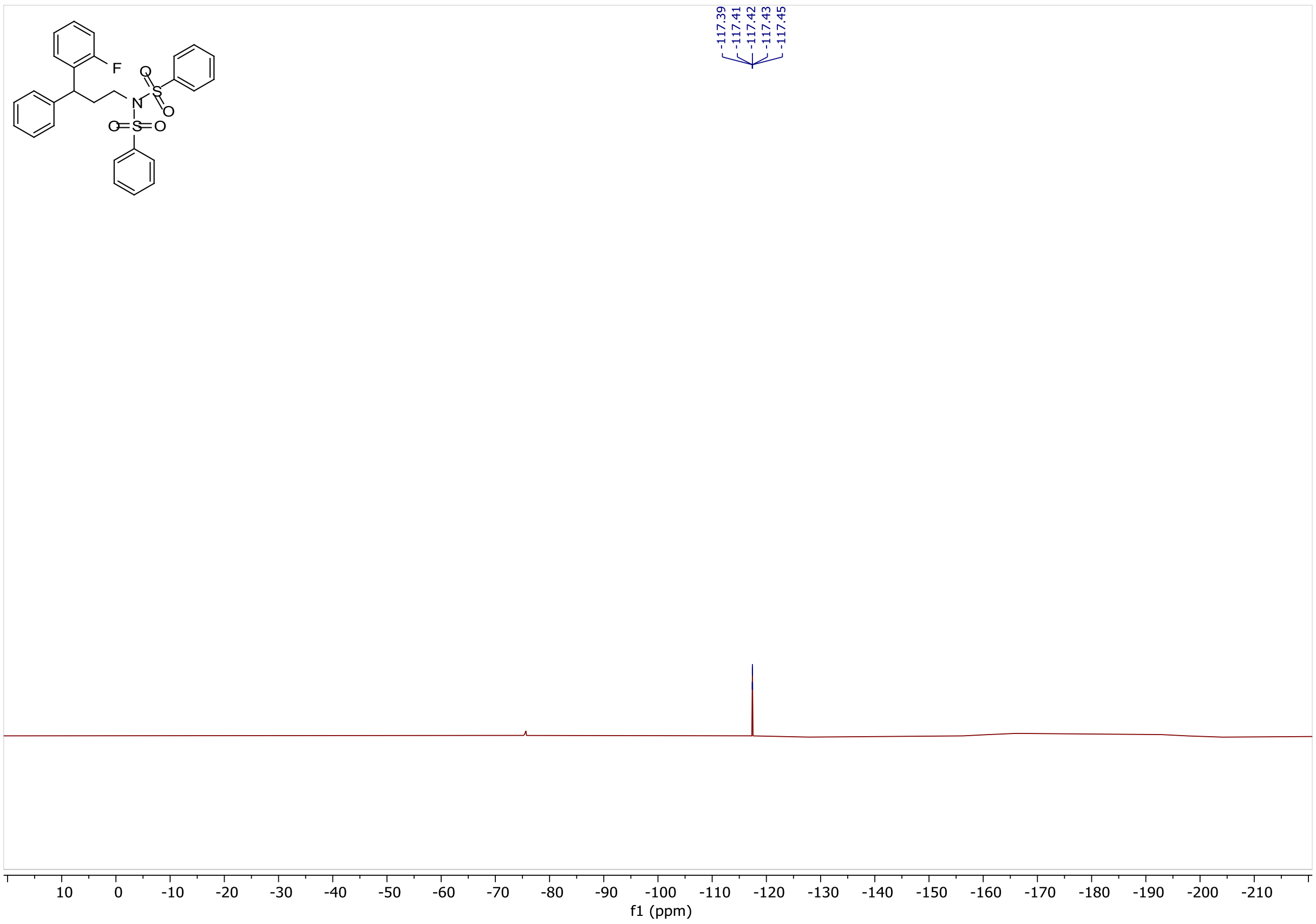

^{13}C NMR Spectrum of *o*-7q (101 MHz, $CDCl_3$)

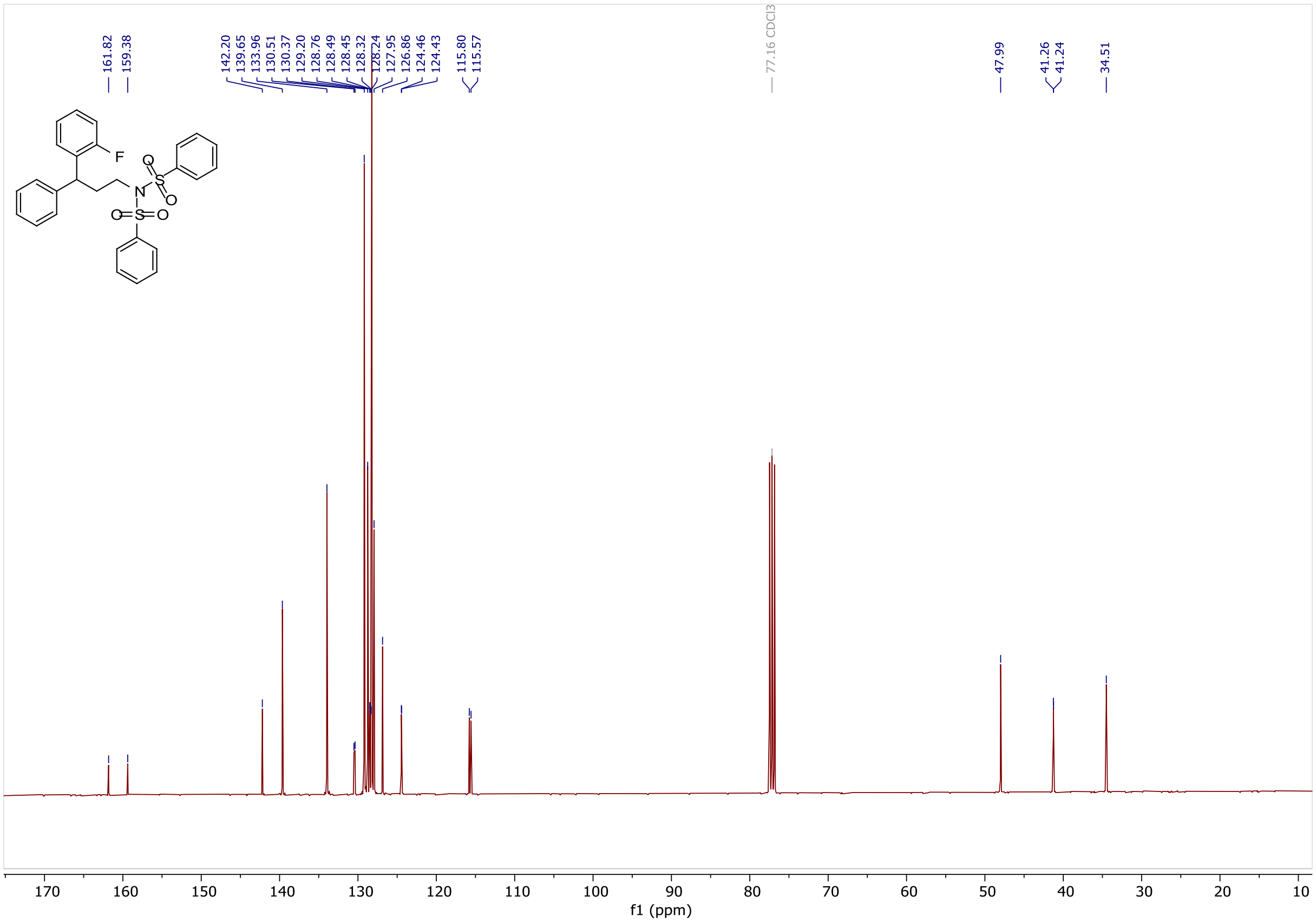

^{1}H NMR Spectrum of *m*-7q (400 MHz, $CDCl_3$)

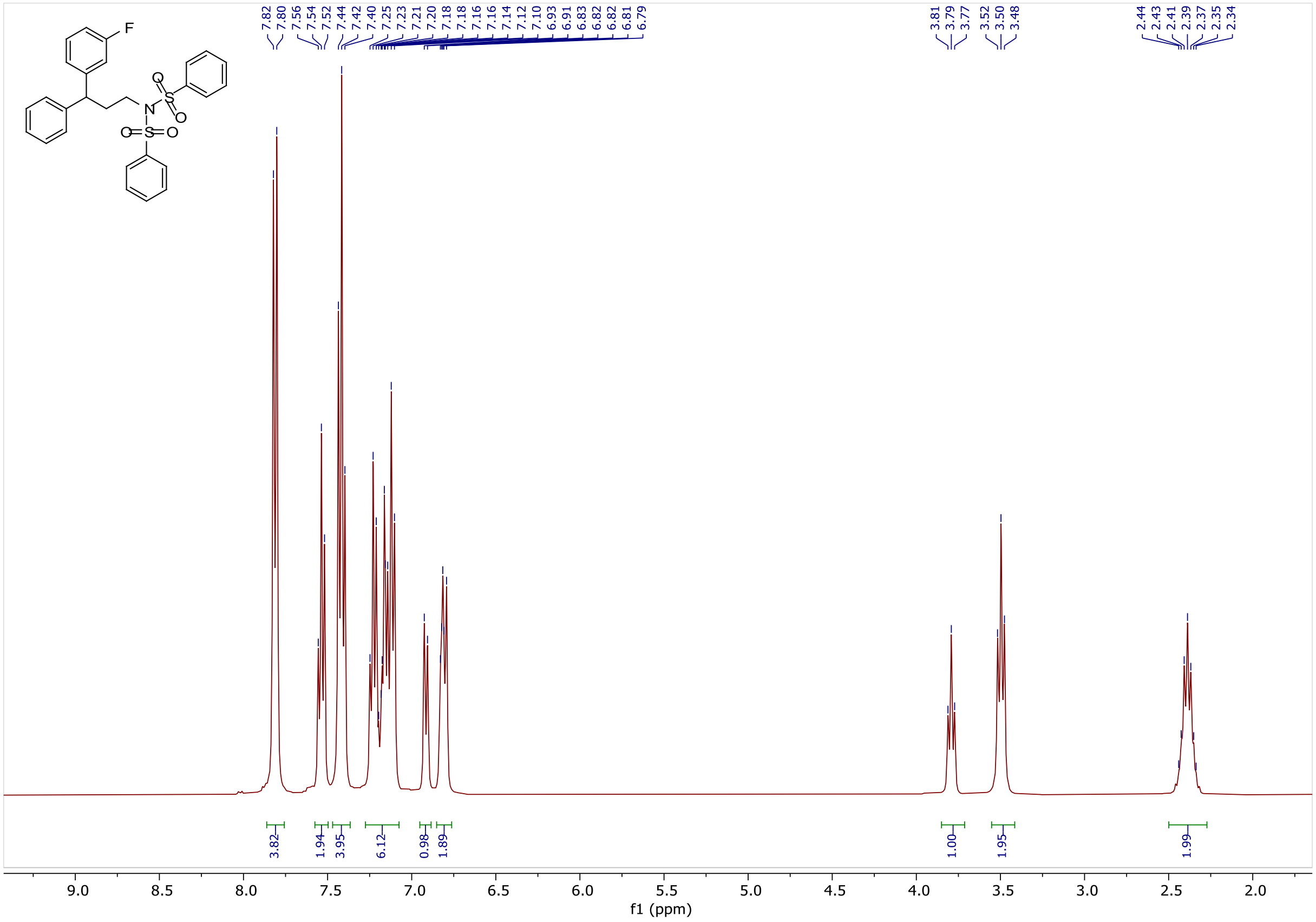

^{19}F NMR Spectrum of *m*-7q (376 MHz, $CDCl_3$)

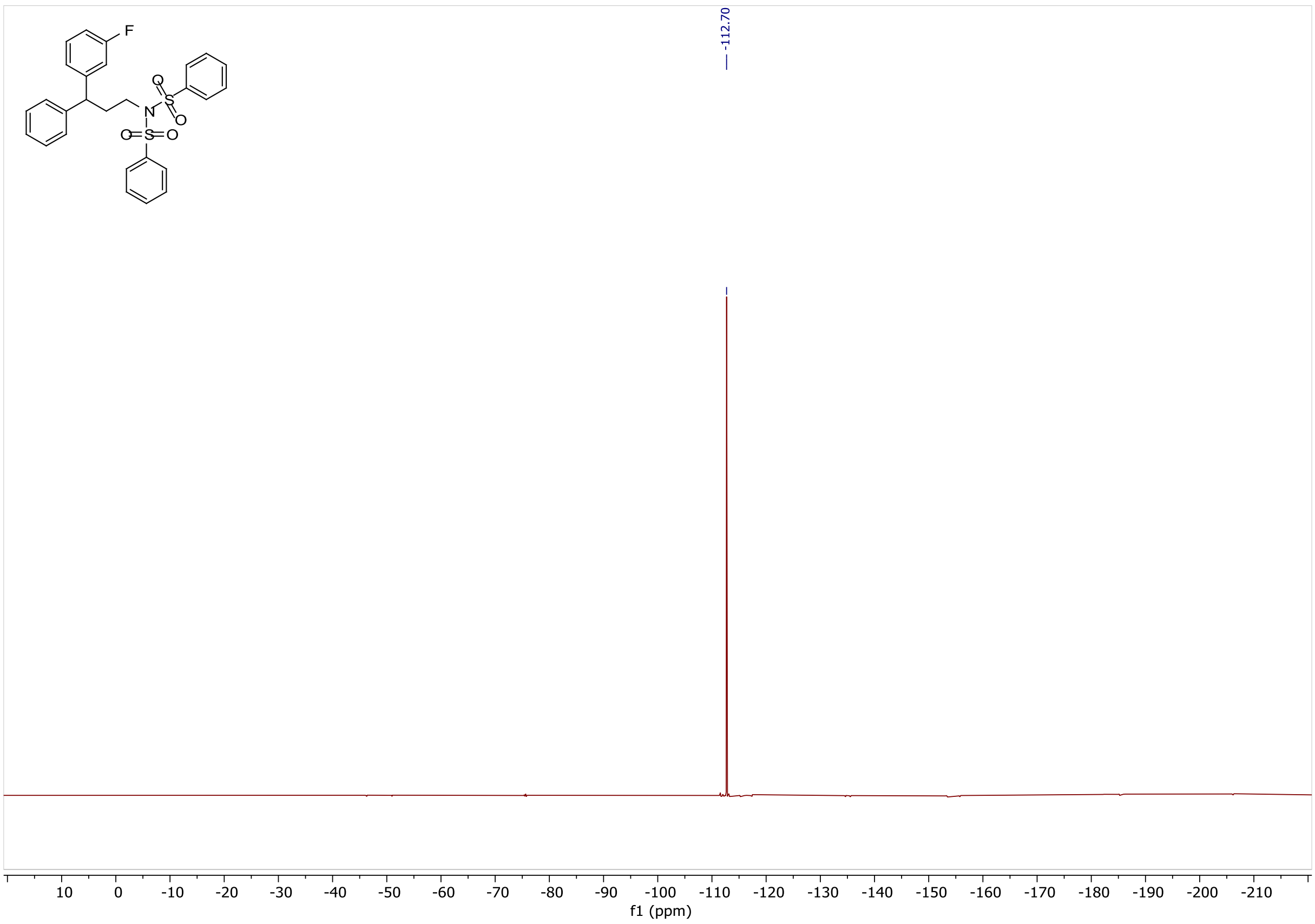

^{13}C NMR Spectrum of *m*-7q (101 MHz, $CDCl_3$)

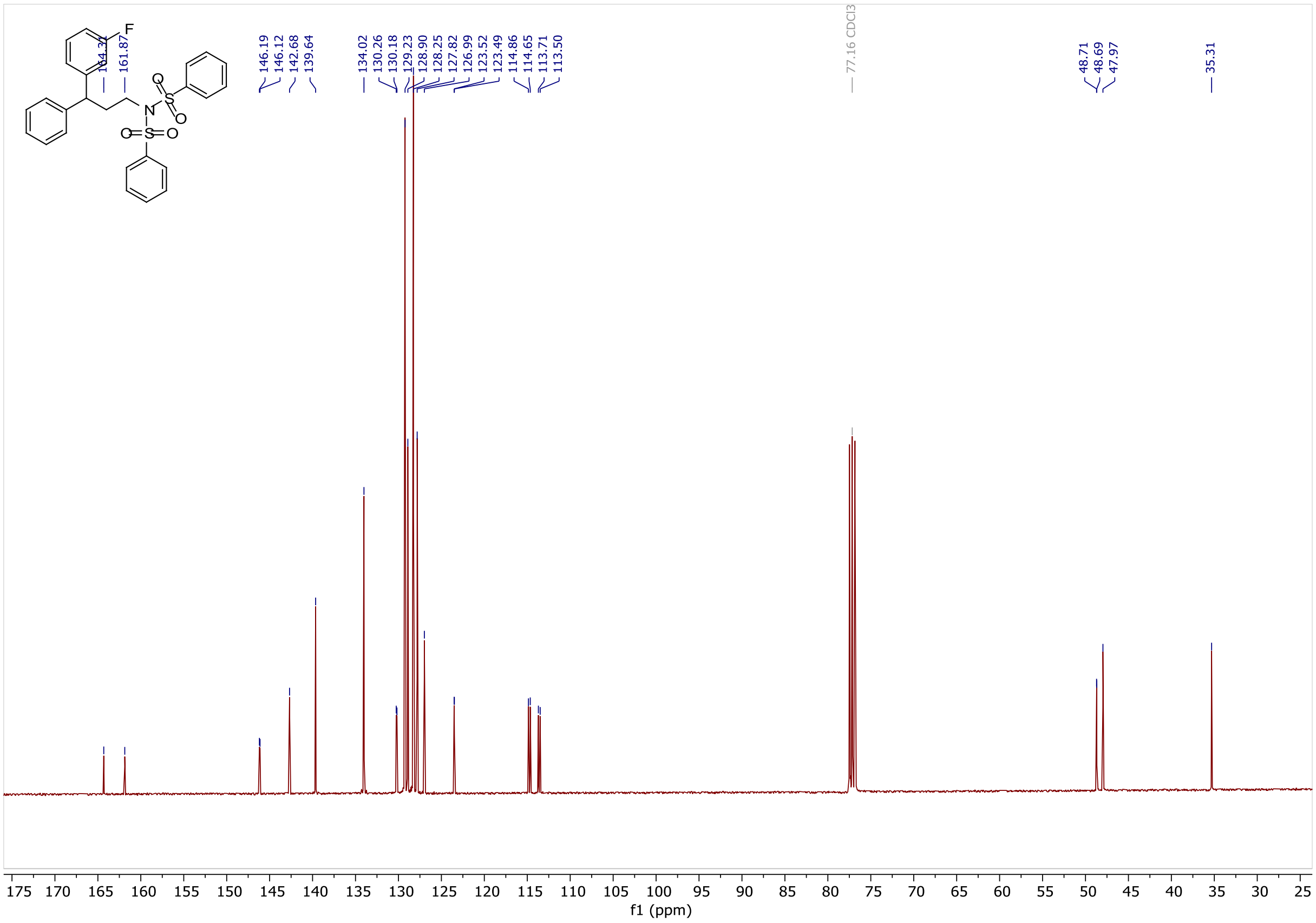

^{1}H NMR Spectrum of *p*-7q (400 MHz, $CDCl_3$)

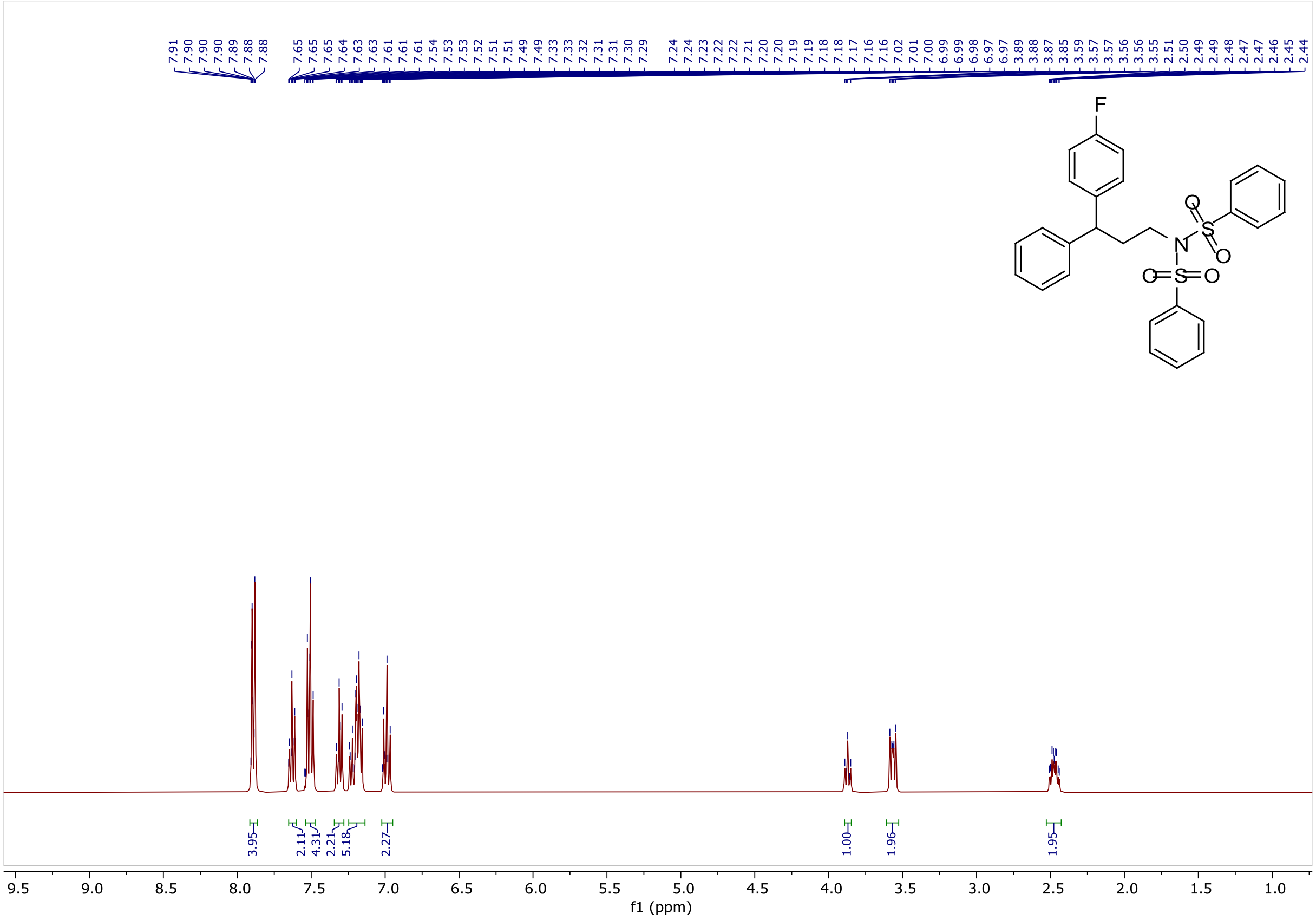

^{19}F NMR Spectrum of *p*-7q (376 MHz, $CDCl_3$)

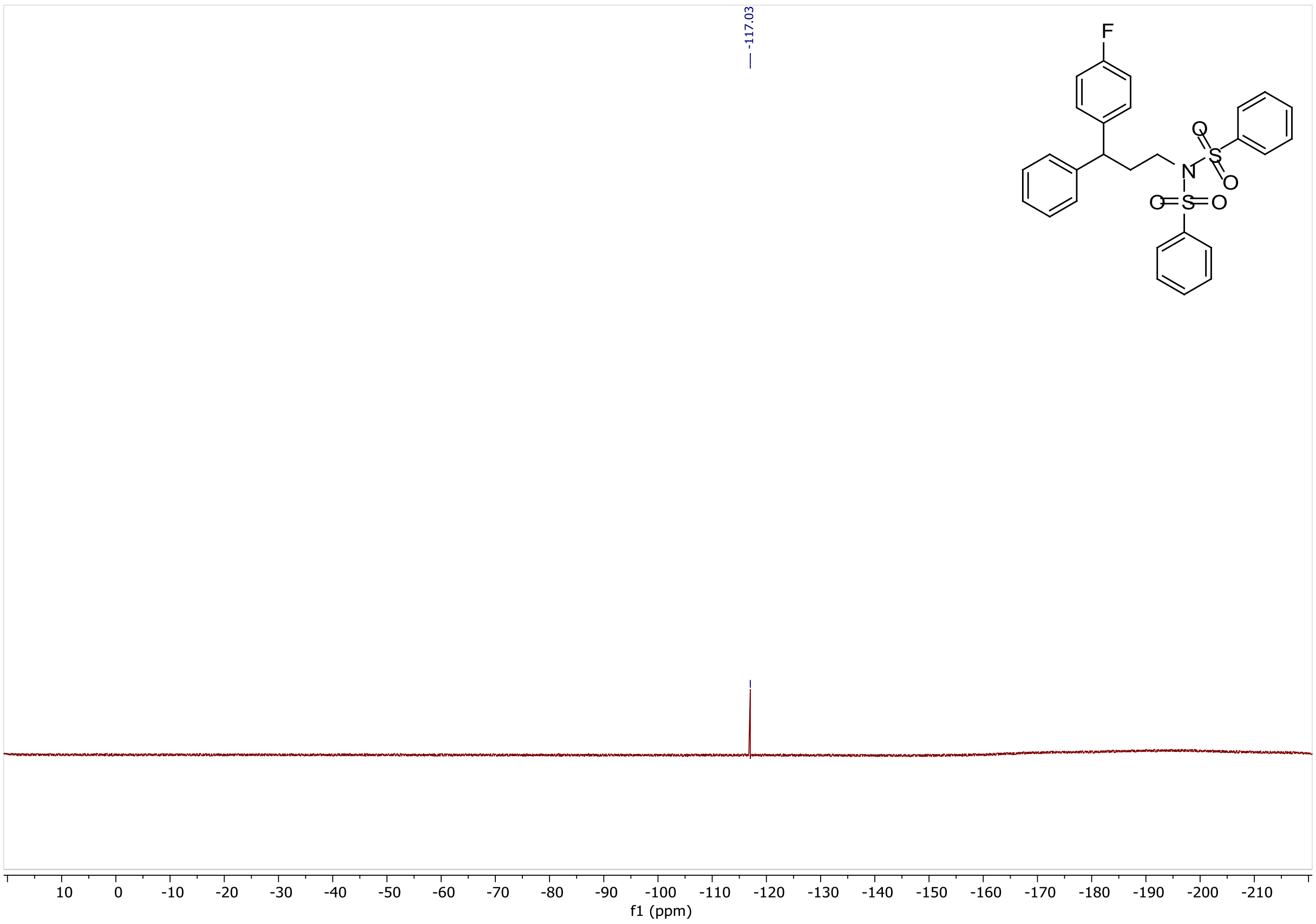

^{13}C NMR Spectrum of *p*-7q (101 MHz, $CDCl_3$)

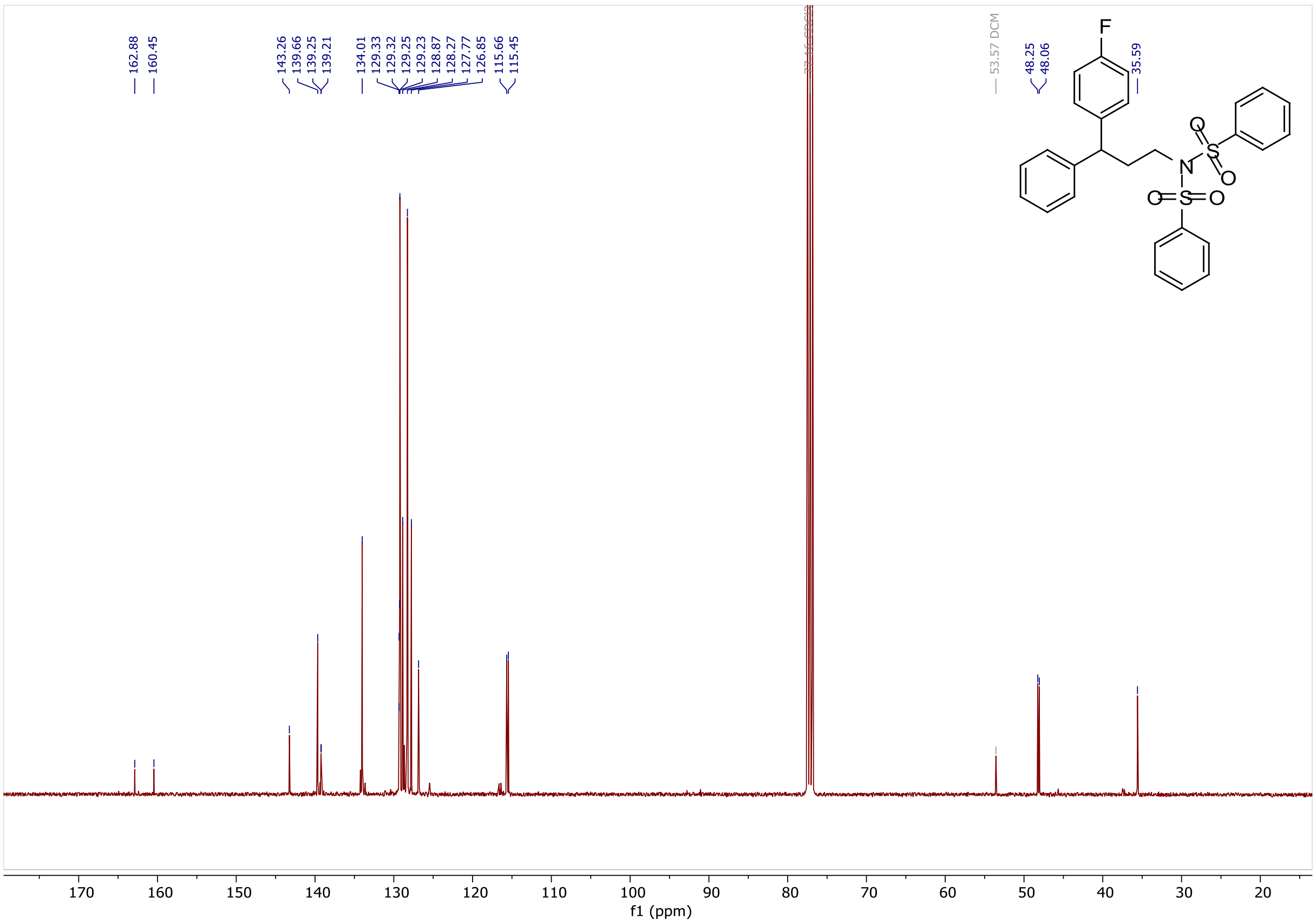

^{1}H NMR Spectrum of *p*-7r (400 MHz, $CDCl_3$)

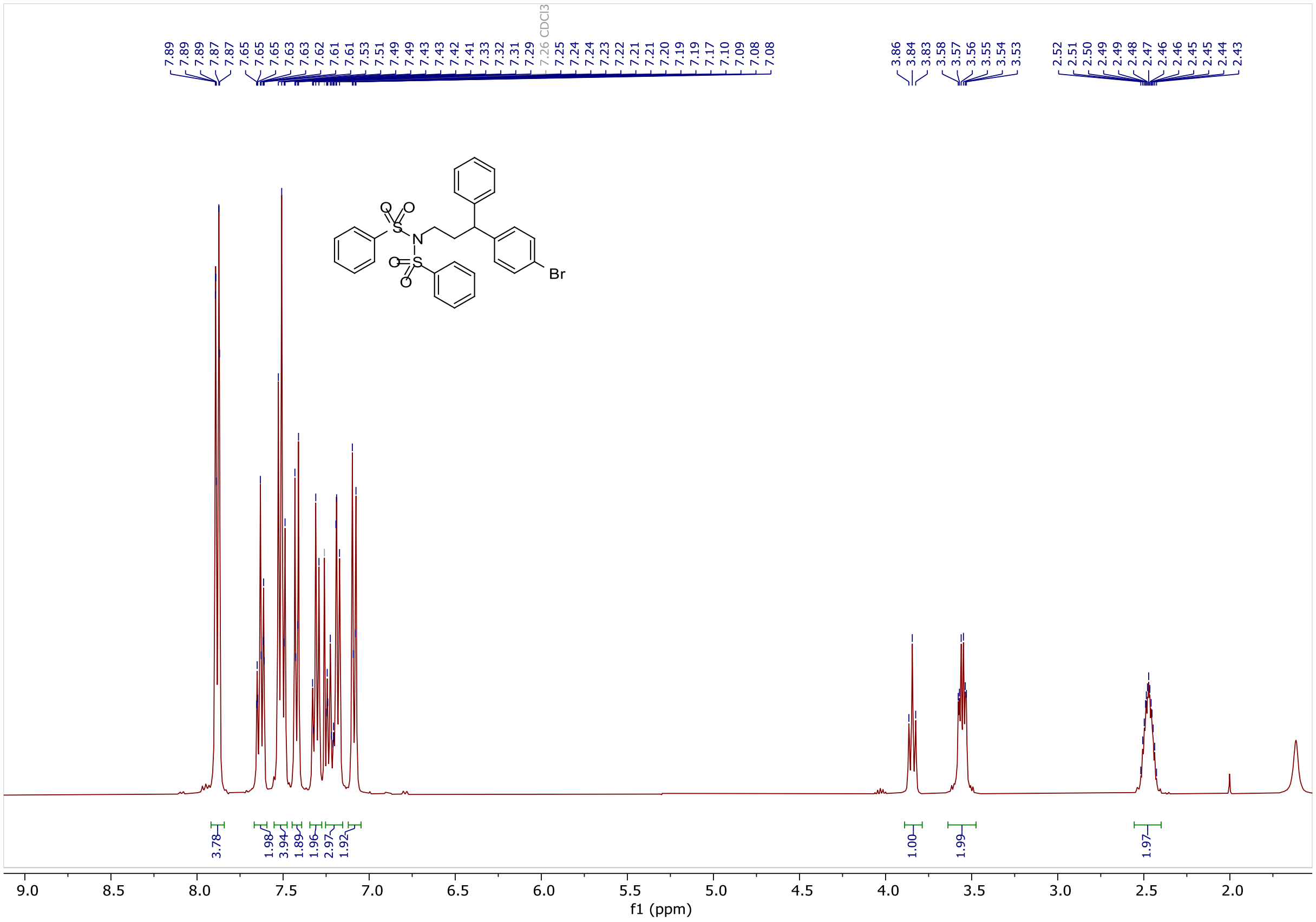

^{13}C NMR Spectrum of *p*-7r (101 MHz, $CDCl_3$)

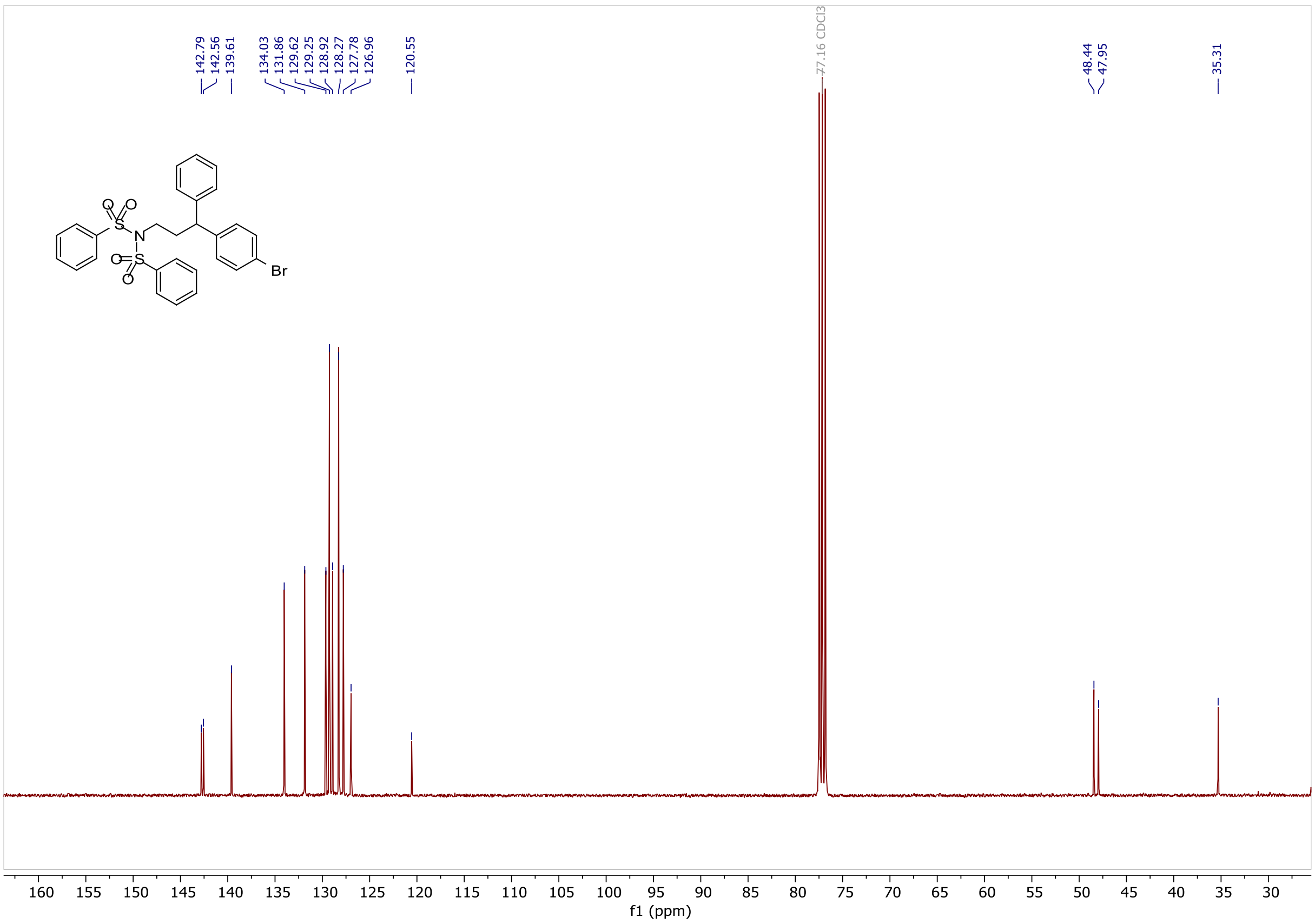

^{1}H NMR Spectrum of *o*-7s (400 MHz, $CDCl_3$)

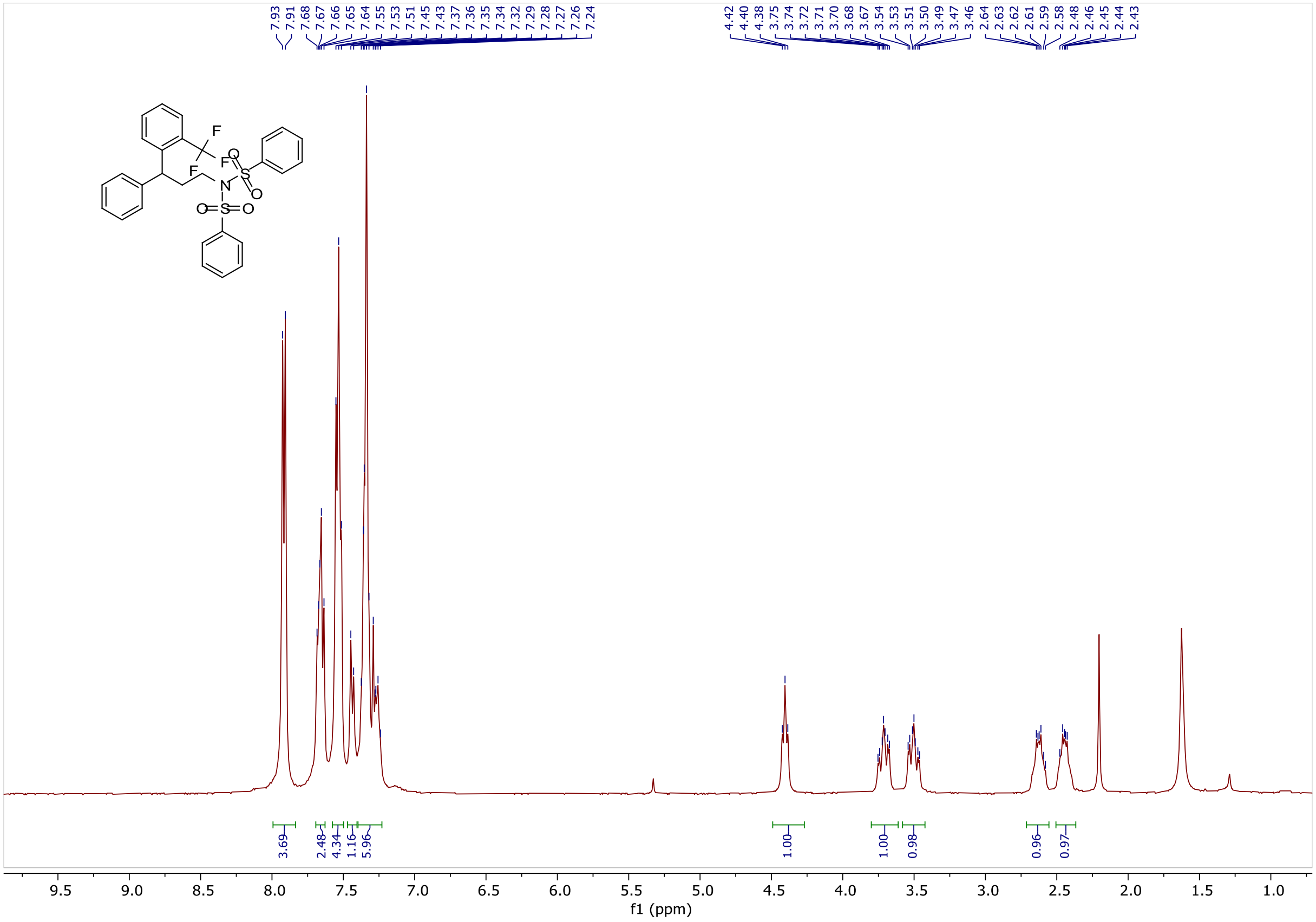

^{19}F NMR Spectrum of *o*-7s (376 MHz, $CDCl_3$)

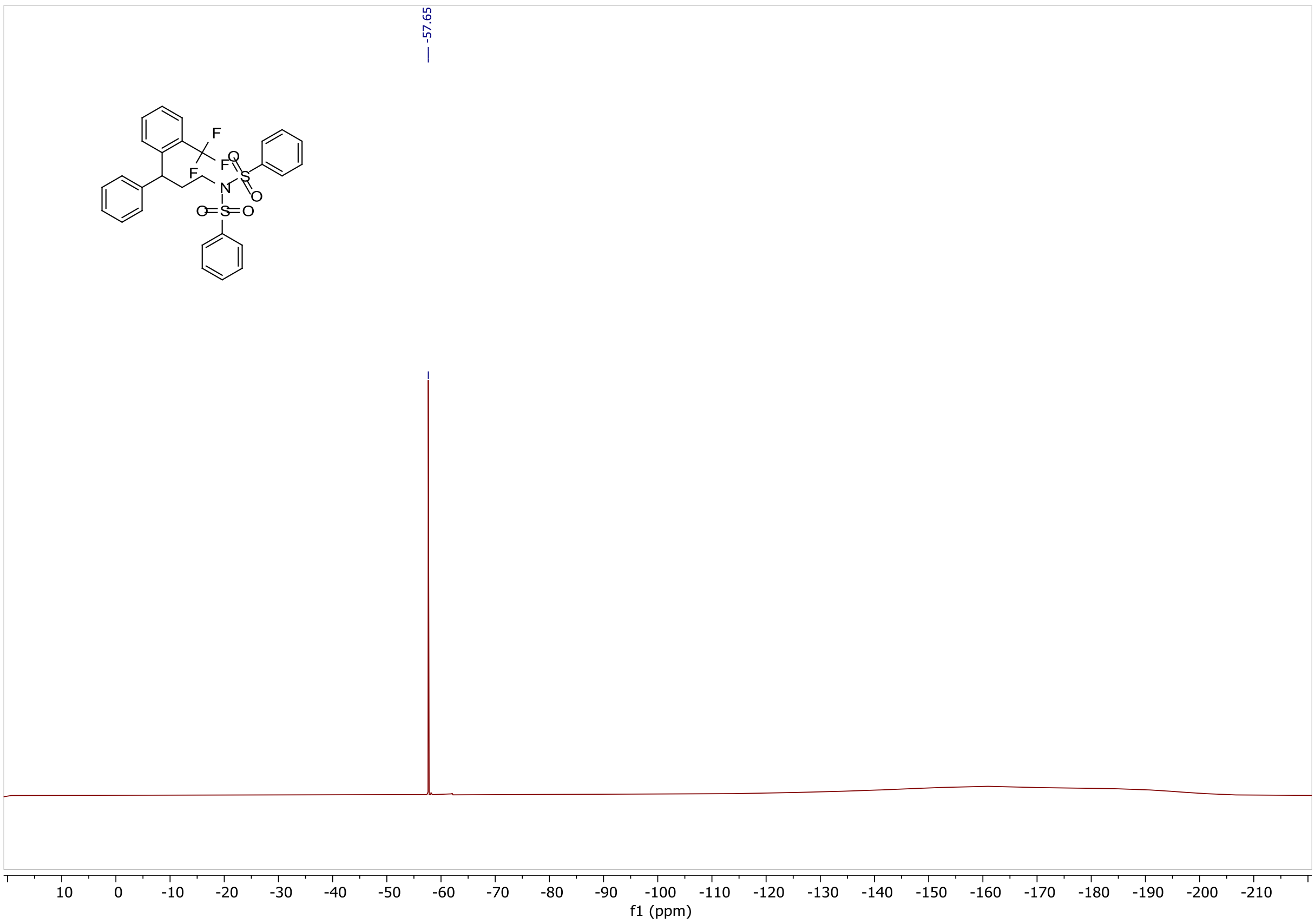

^{13}C NMR Spectrum of *o*-7s (101 MHz, $CDCl_3$)

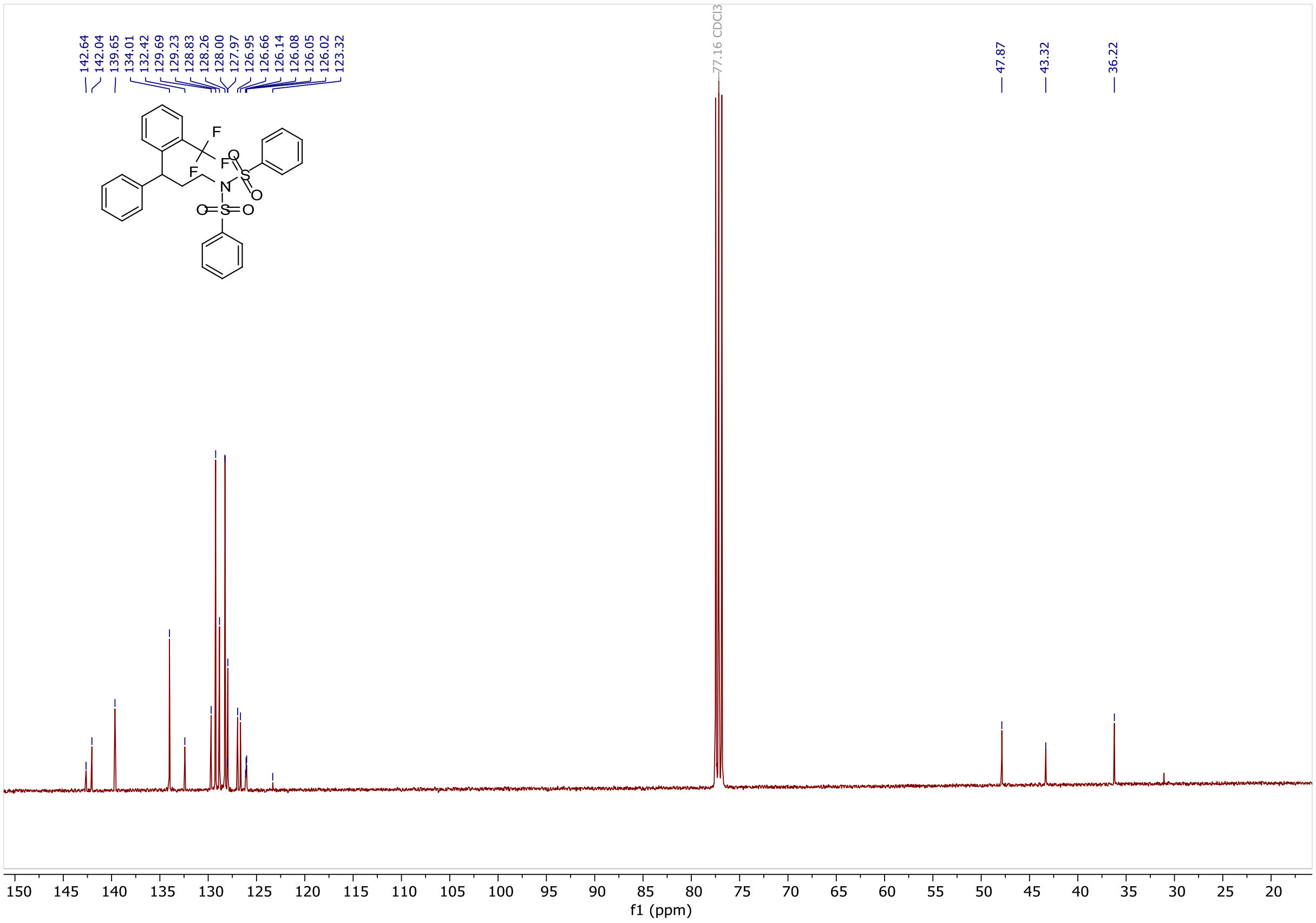

^{1}H NMR Spectrum of *m*-7s (400 MHz, $CDCl_3$)

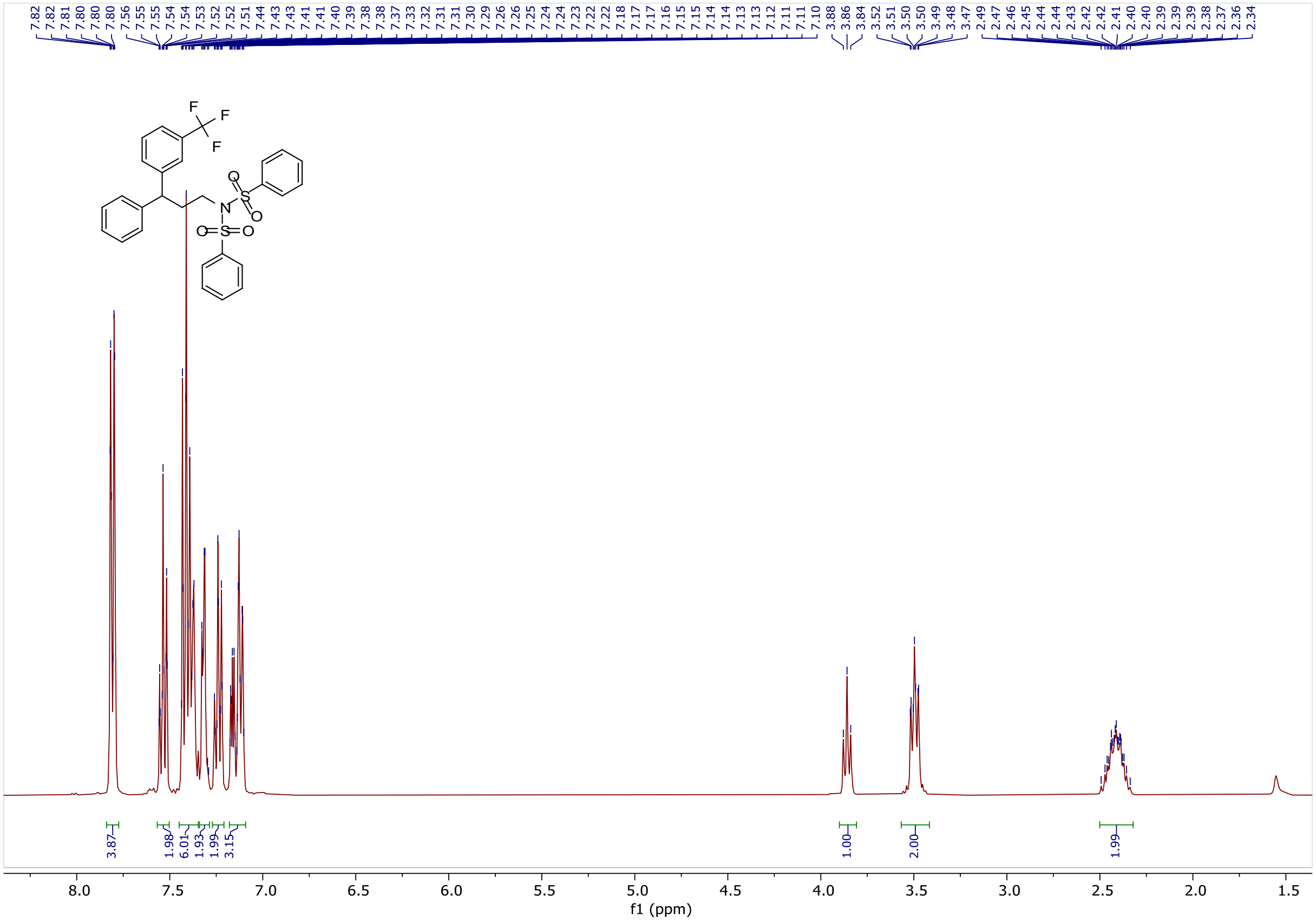

^{19}F NMR Spectrum of *m*-7s (376 MHz, $CDCl_3$)

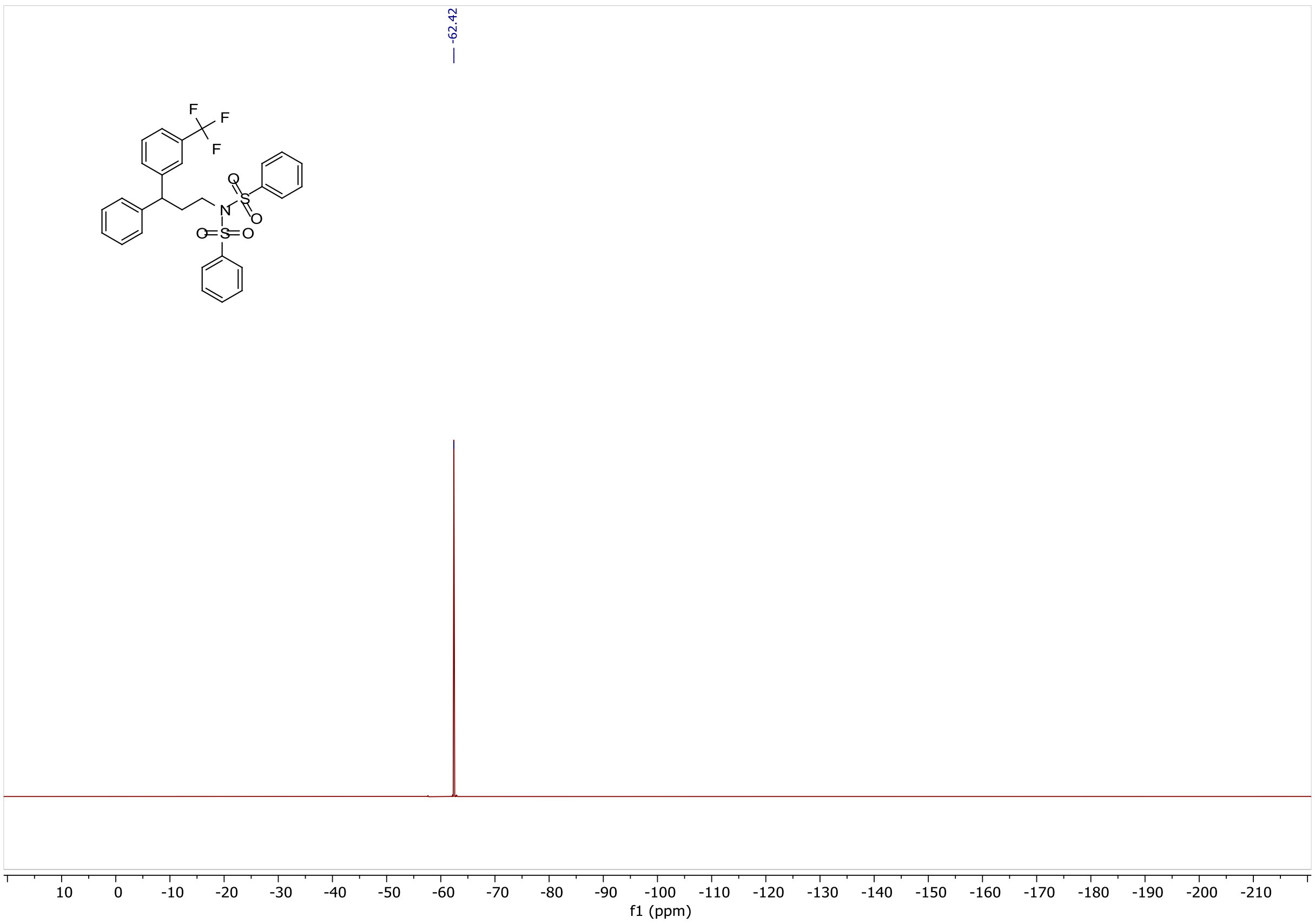

^{13}C NMR Spectrum of *m*-7s (101 MHz, $CDCl_3$)

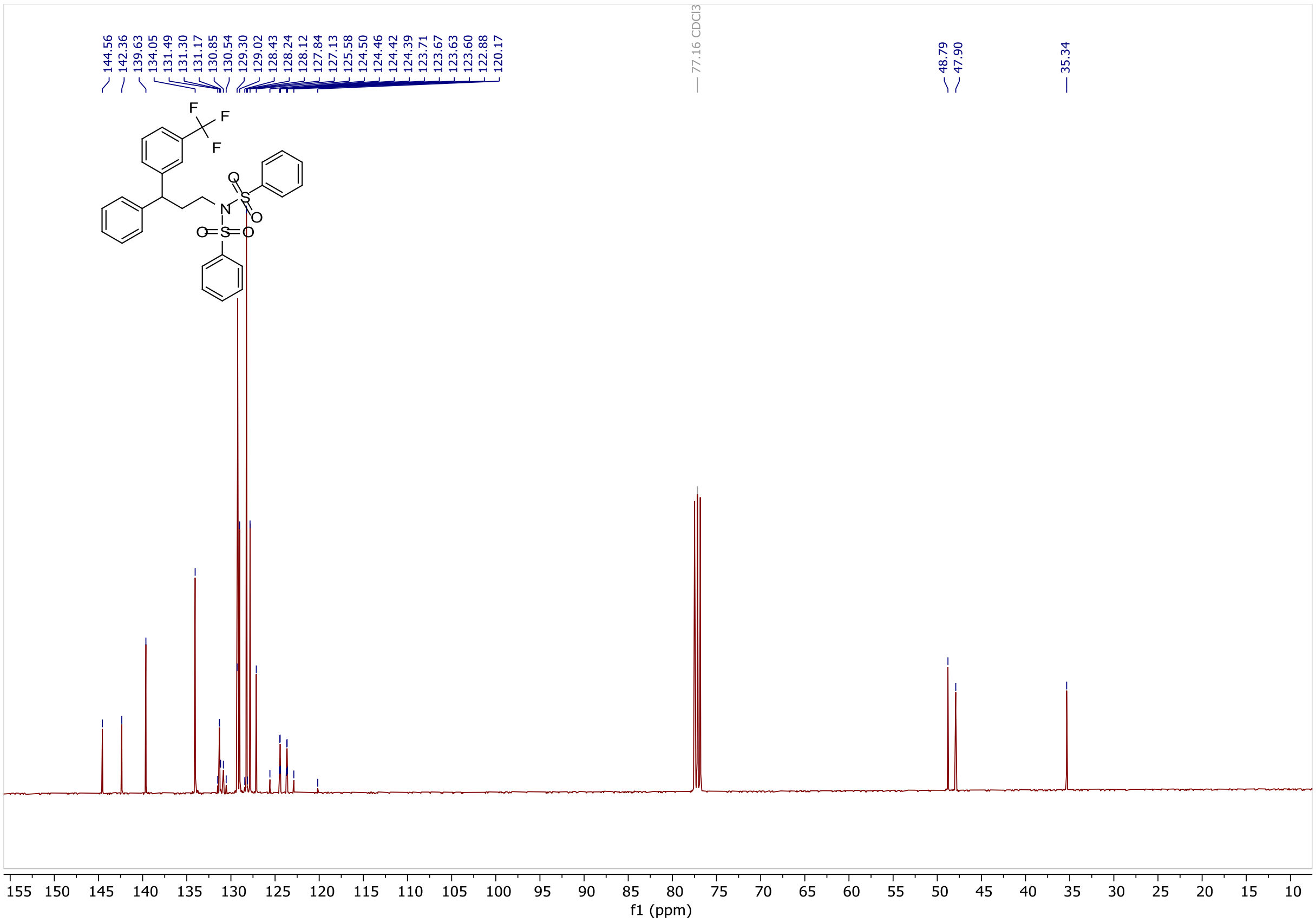

^{1}H NMR Spectrum of *p*-7s (400 MHz, $CDCl_3$)

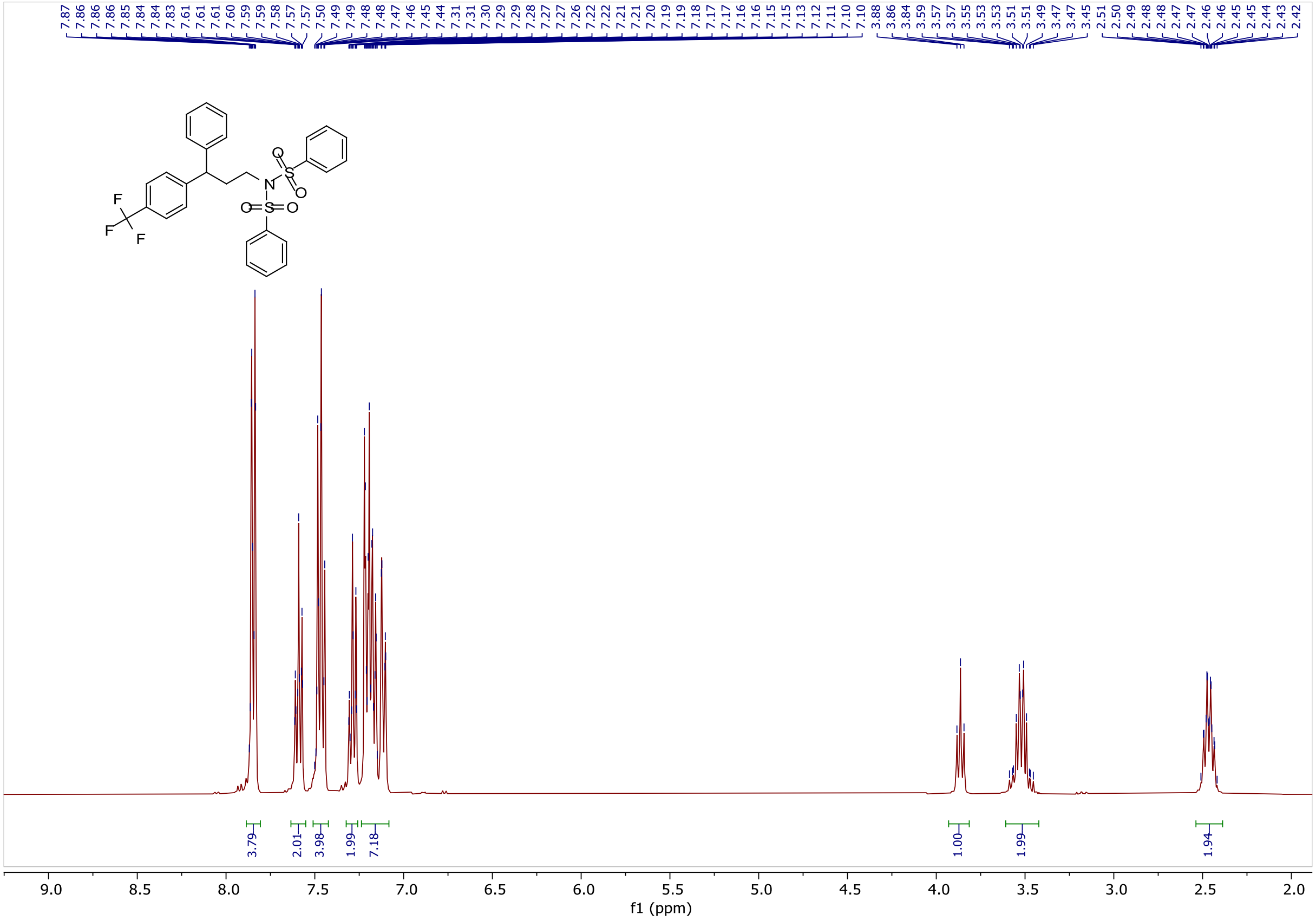

^{19}F NMR Spectrum of *p*-7s (376 MHz, $CDCl_3$)

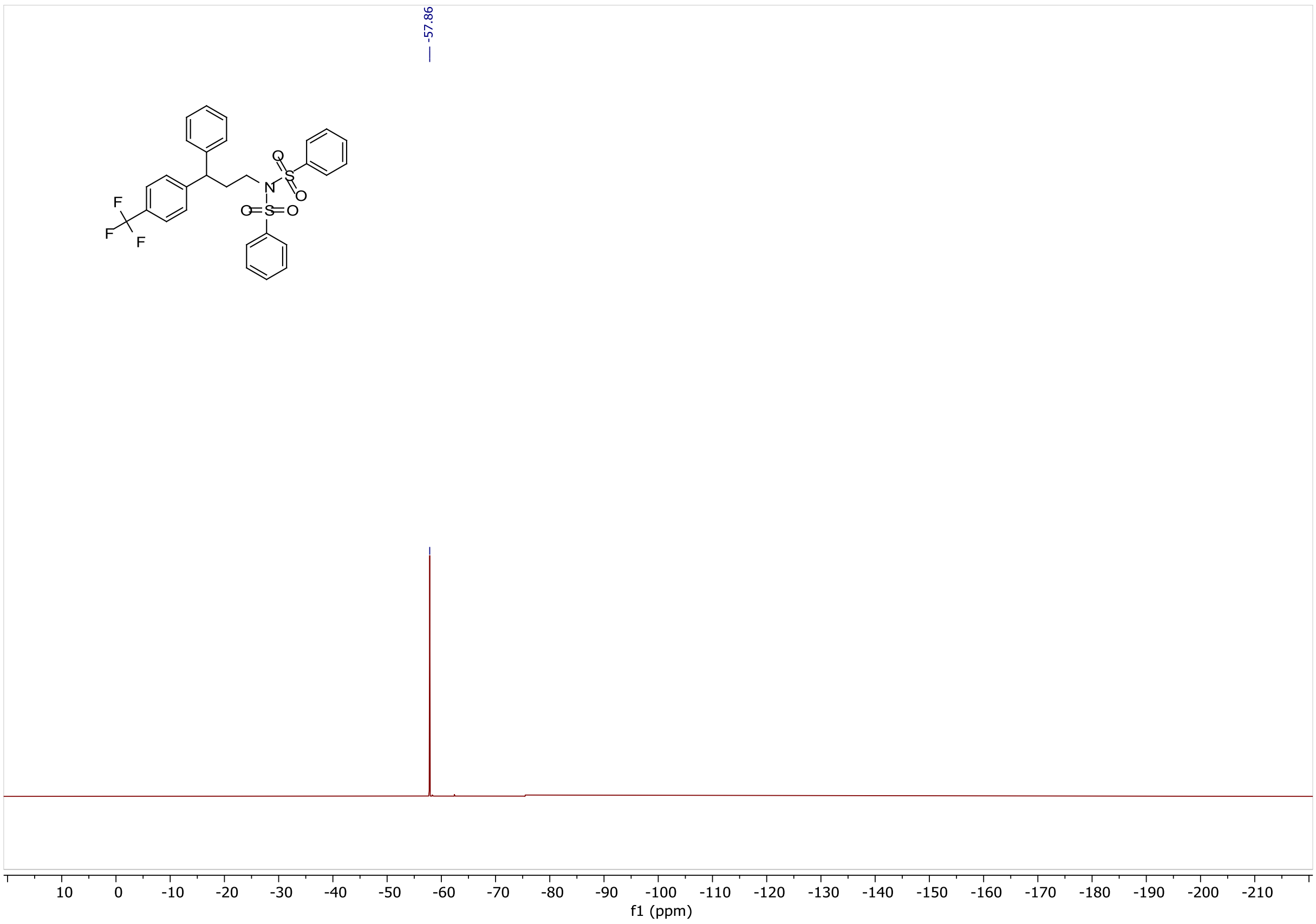

^{13}C NMR Spectrum of *p*-7s (101 MHz, CDCl$_3$)

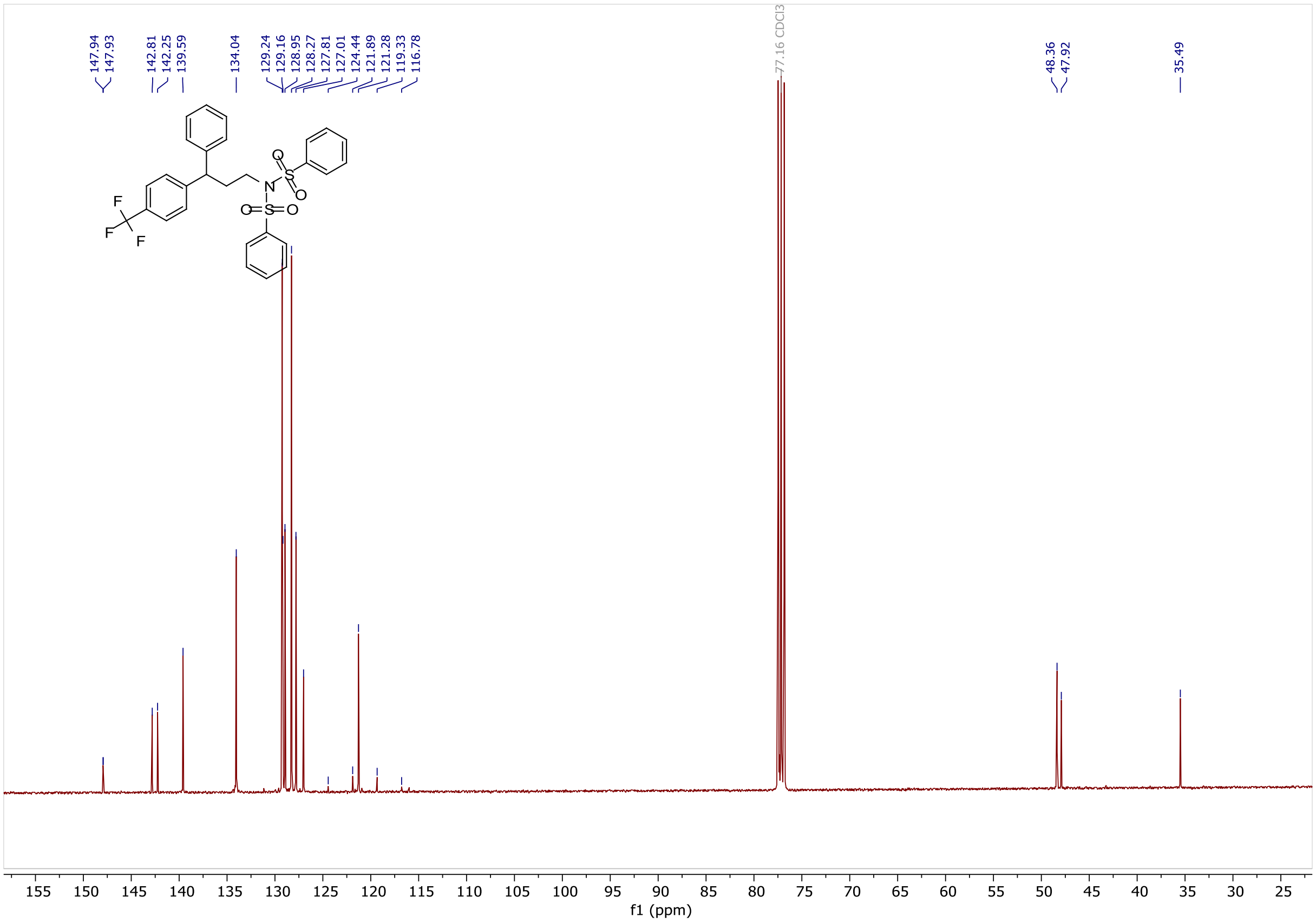

^{1}H NMR Spectrum of *o*-7t (400 MHz, $CDCl_3$)

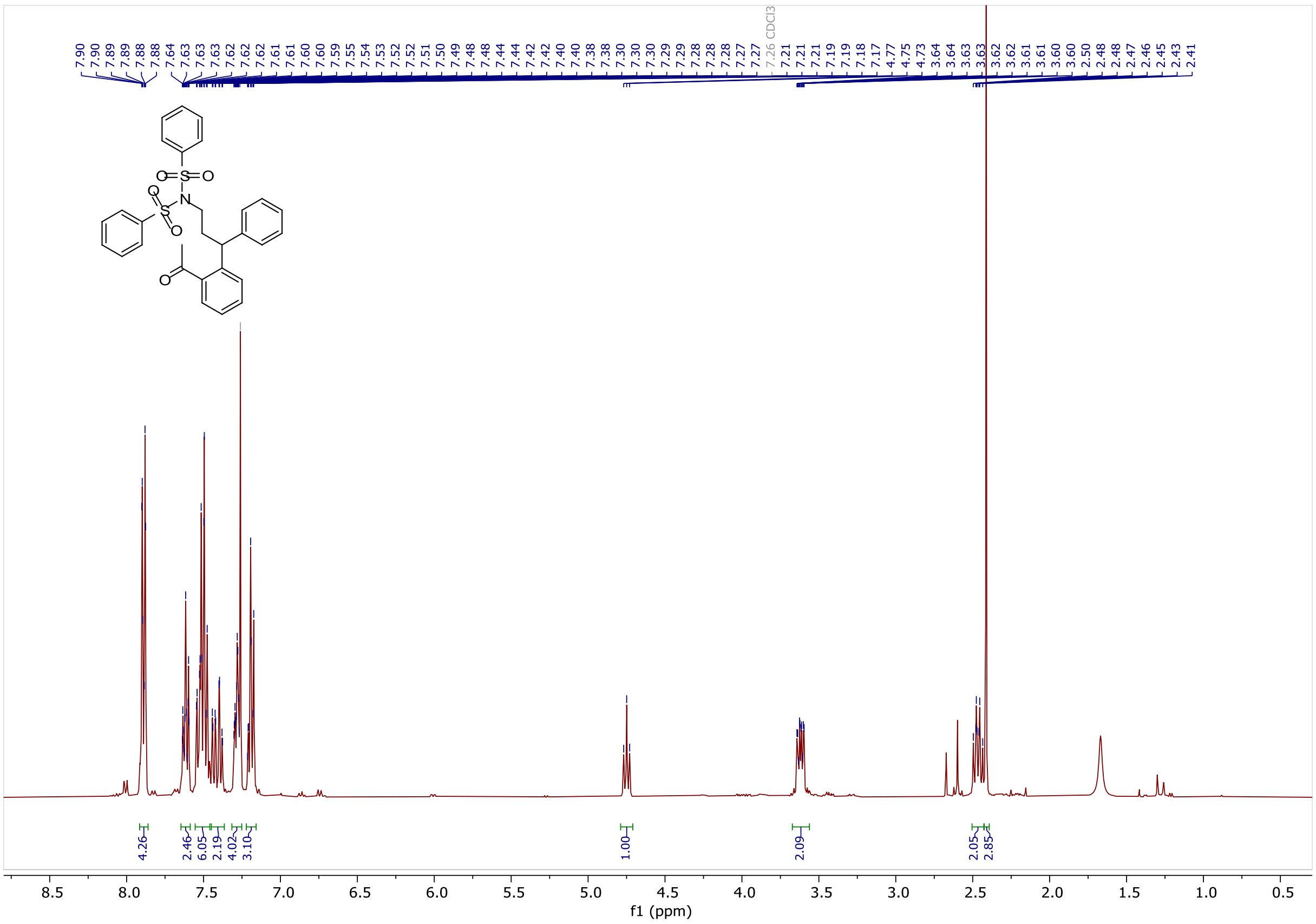

^{13}C NMR Spectrum of *o*-7t (101 MHz, $CDCl_3$)

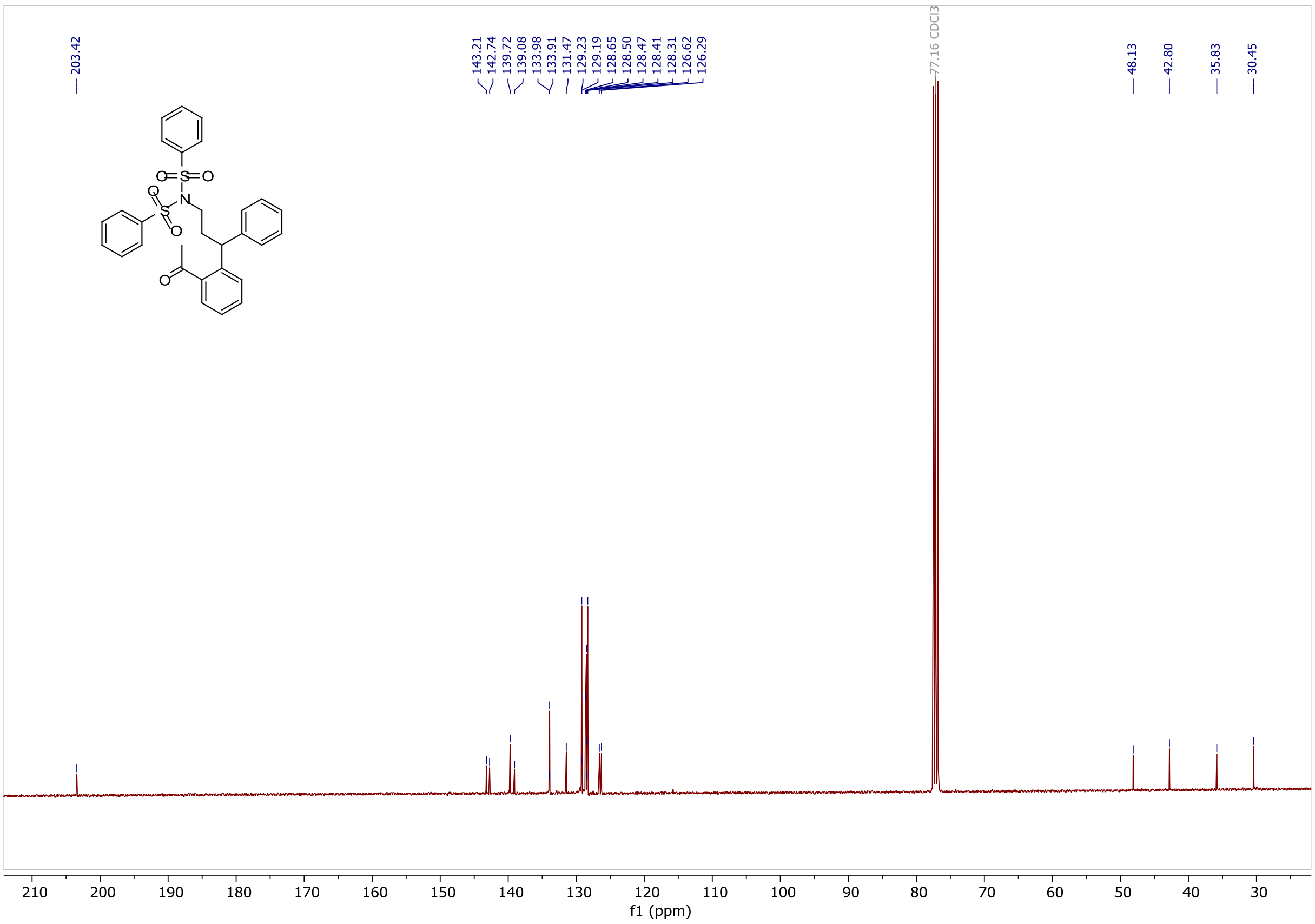

^{1}H NMR Spectrum of *p*-7t (400 MHz, $CDCl_3$)

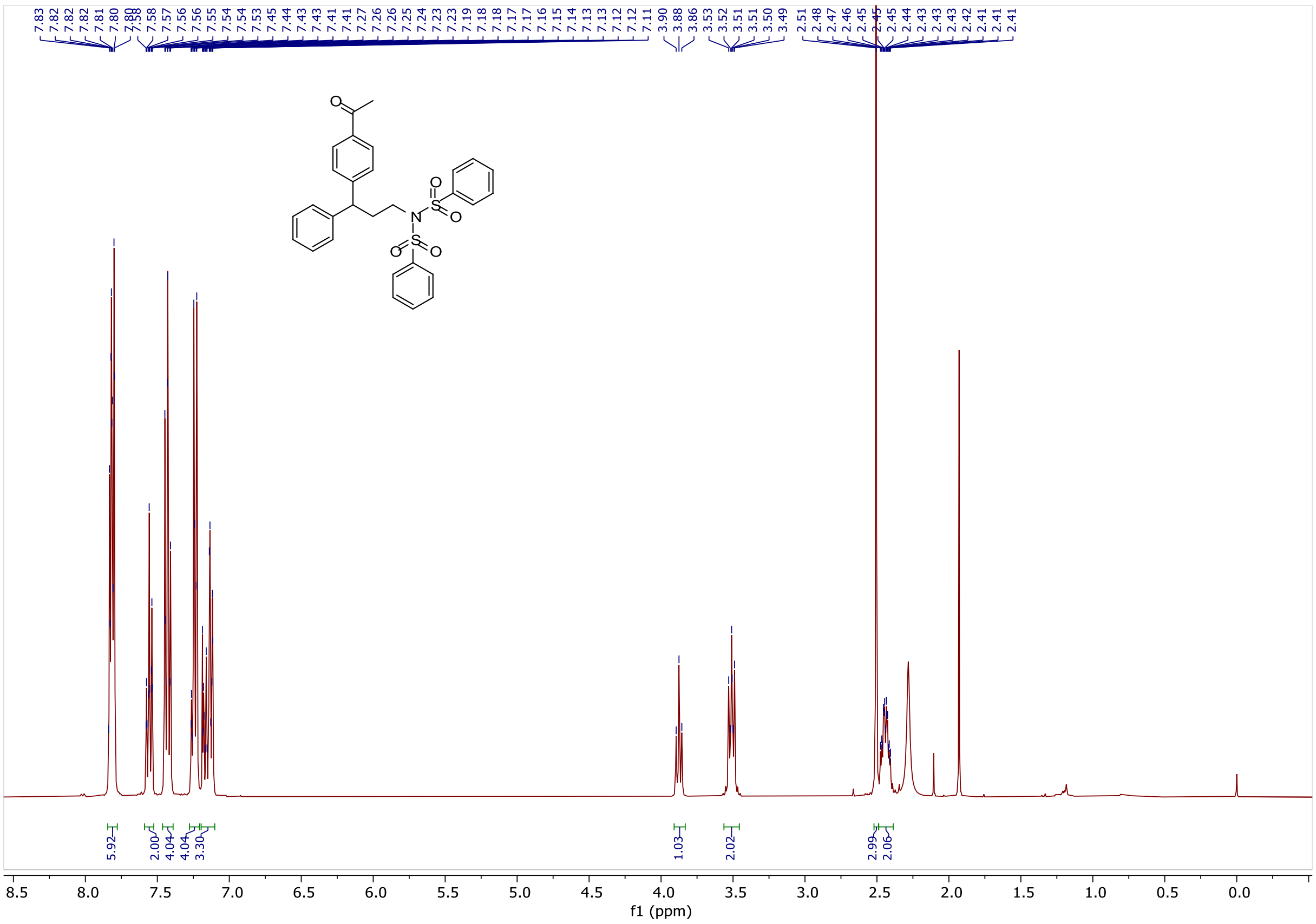

^{13}C NMR Spectrum of *p*-7t (101 MHz, $CDCl_3$)

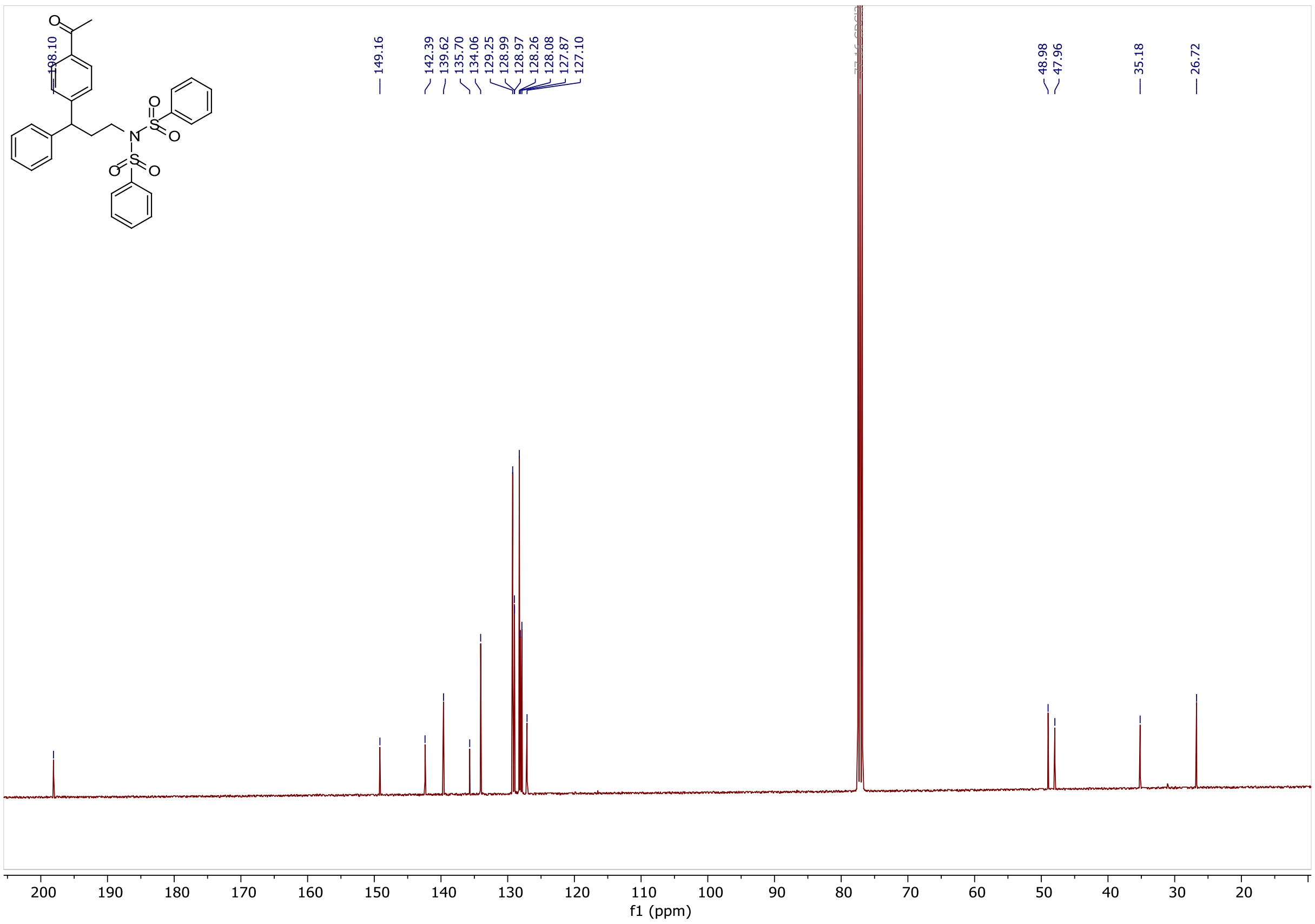

^{1}H NMR Spectrum of 7u (400 MHz, $CDCl_3$)

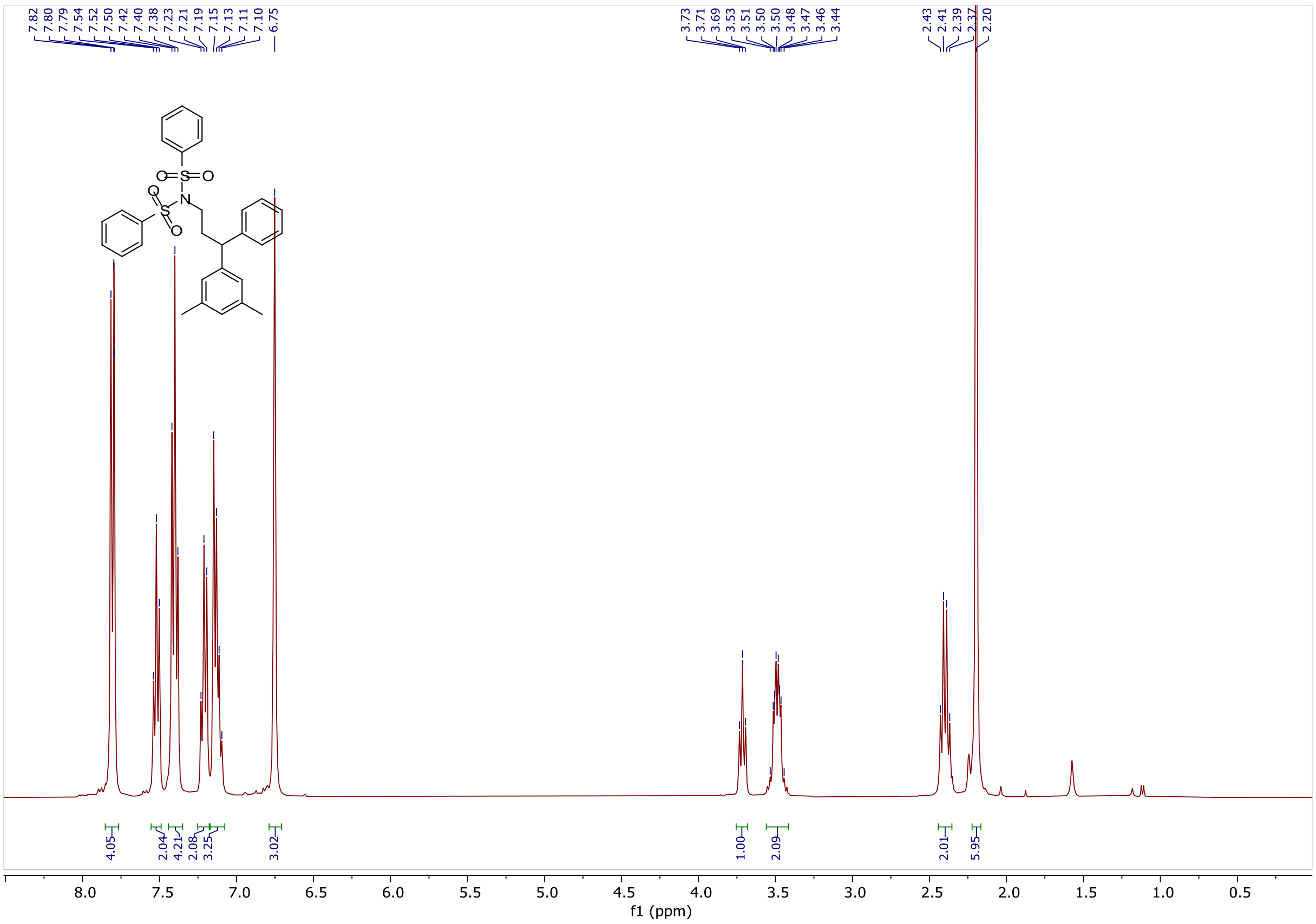

^{13}C NMR Spectrum of 7u (101 MHz, $CDCl_3$)

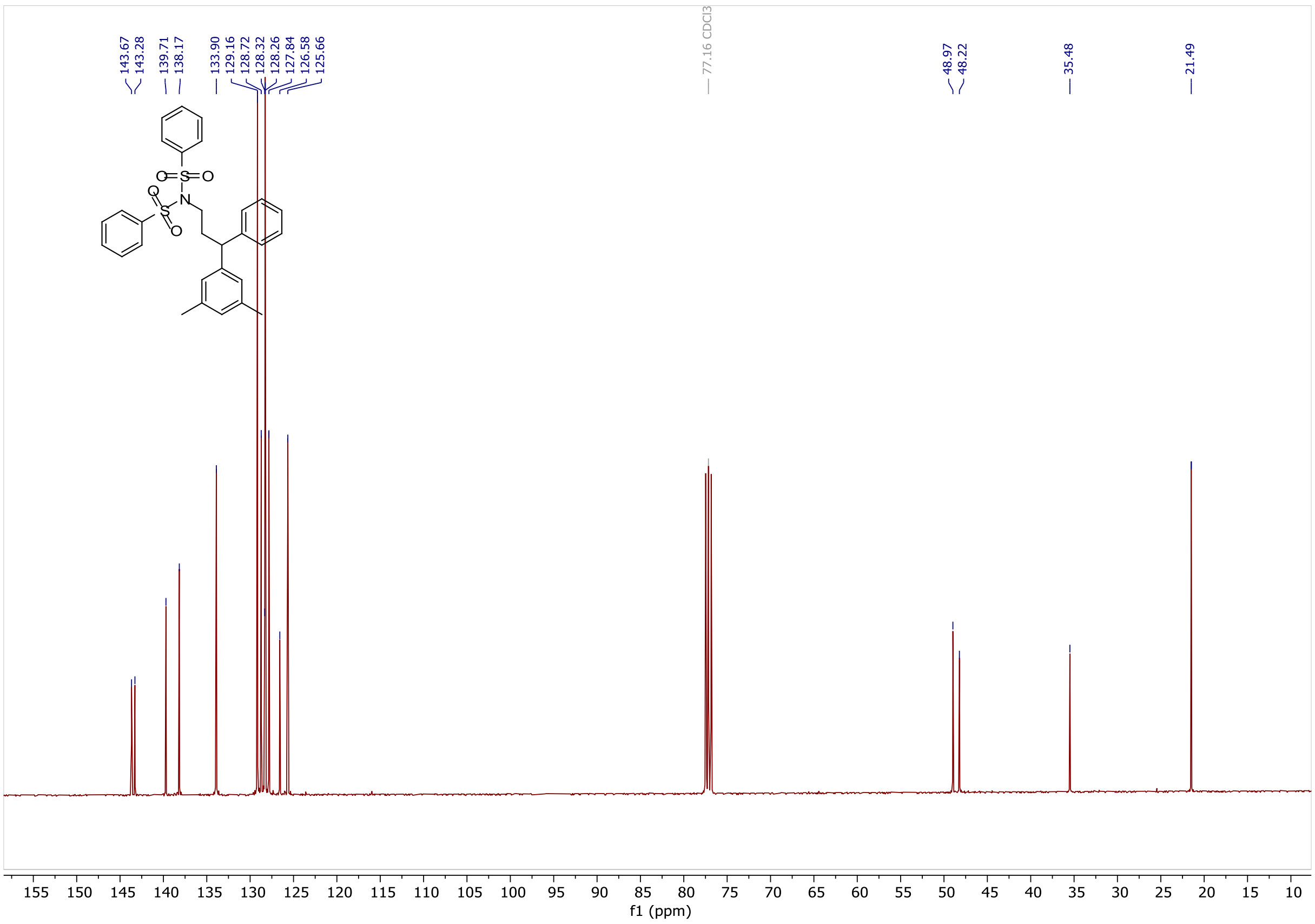

^{1}H NMR Spectrum of 7v (400 MHz, $CDCl_3$)

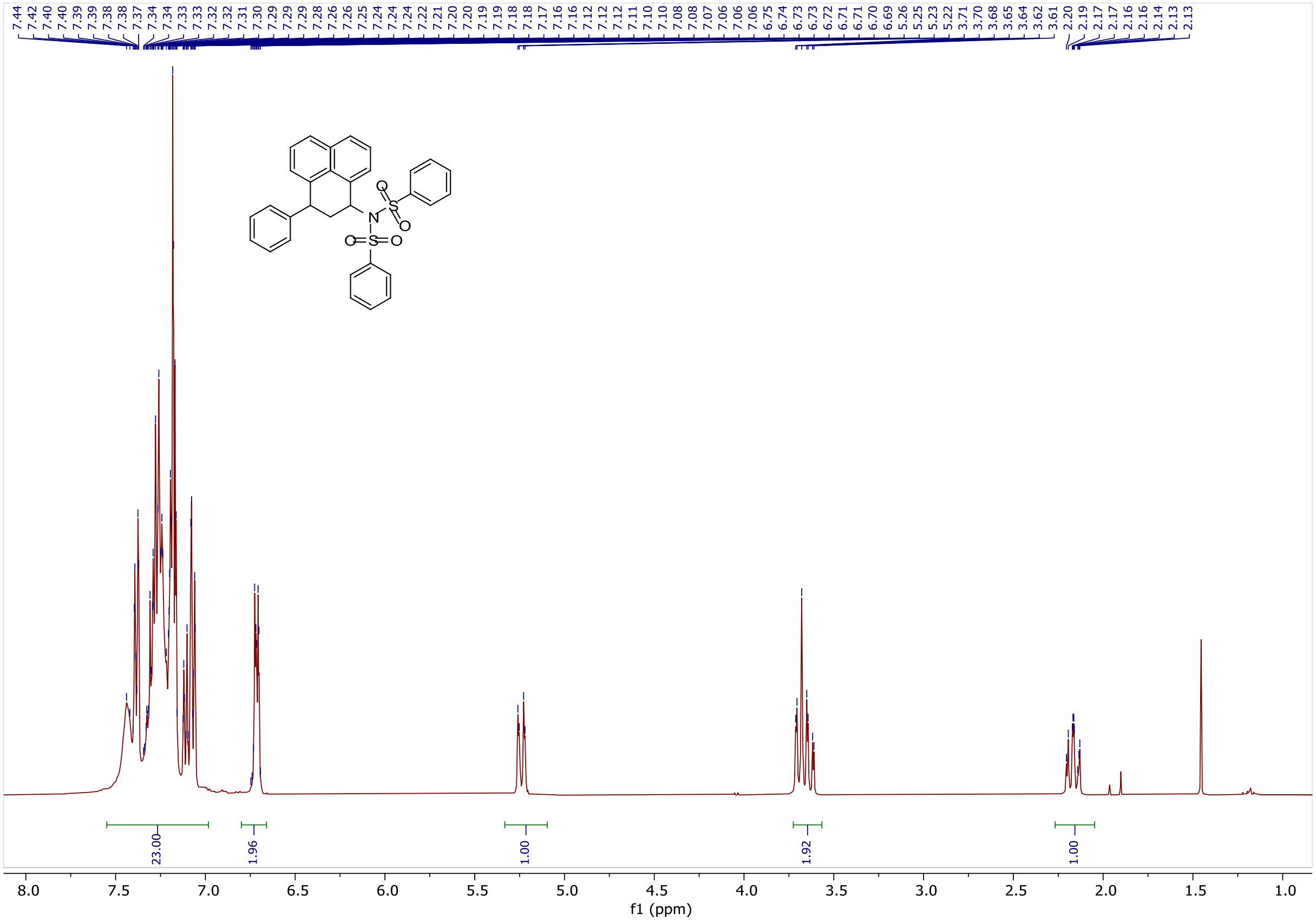

^{13}C NMR Spectrum of 7v (101 MHz, $CDCl_3$)

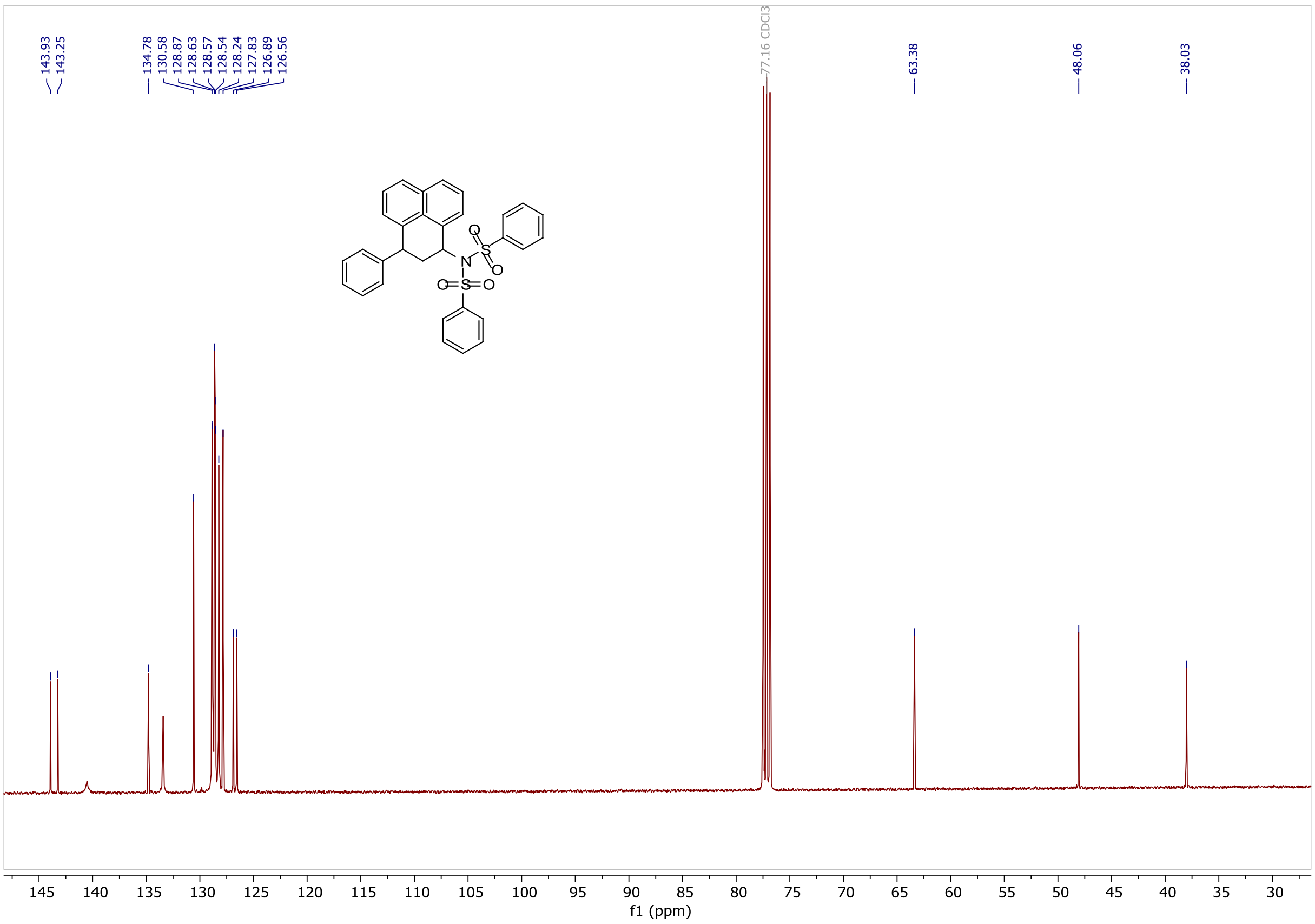

^{1}H NMR Spectrum of *o*-7w (400 MHz, CDCl$_3$)

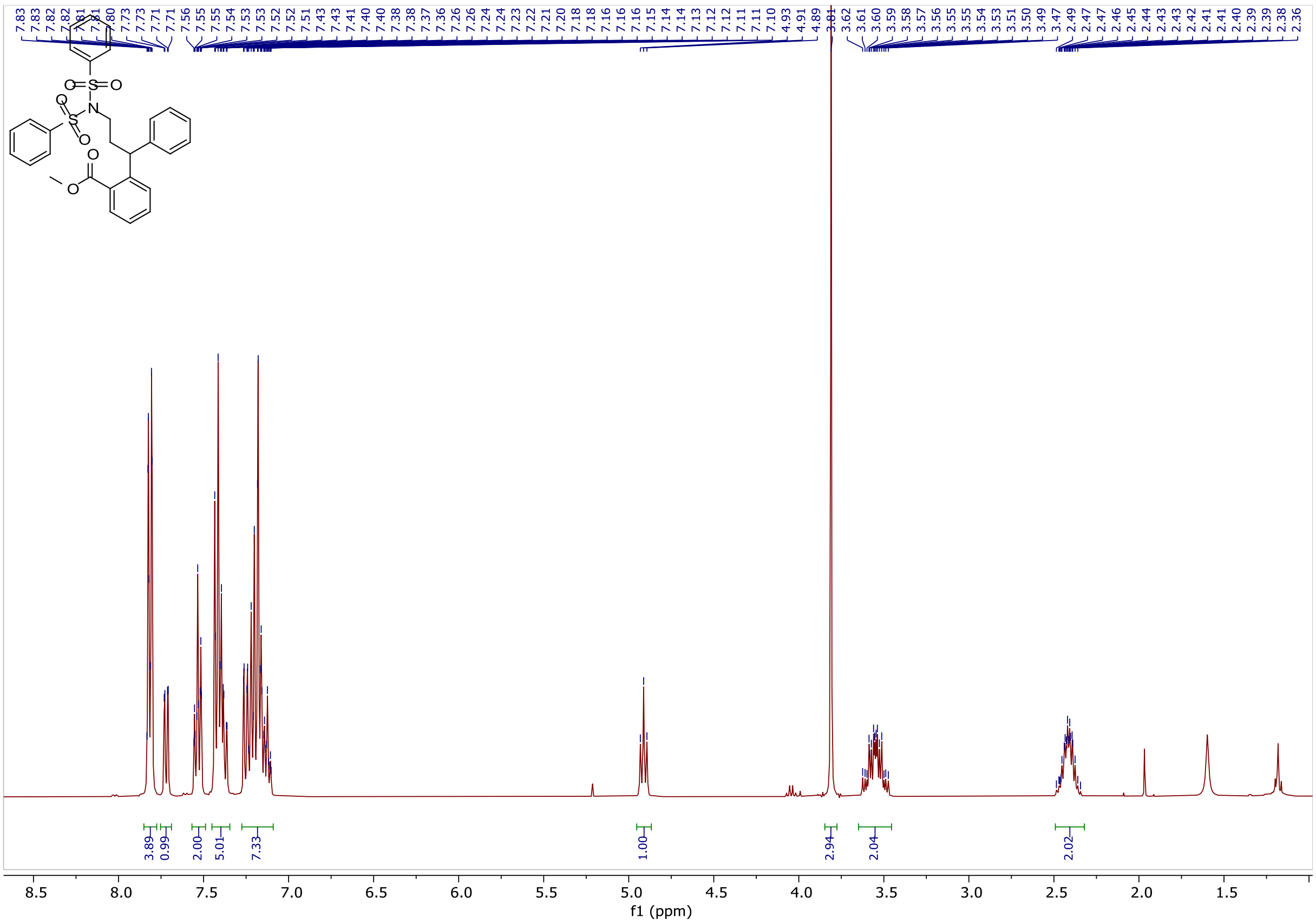

^{13}C NMR Spectrum of *o*-7w (101 MHz, CDCl$_3$)

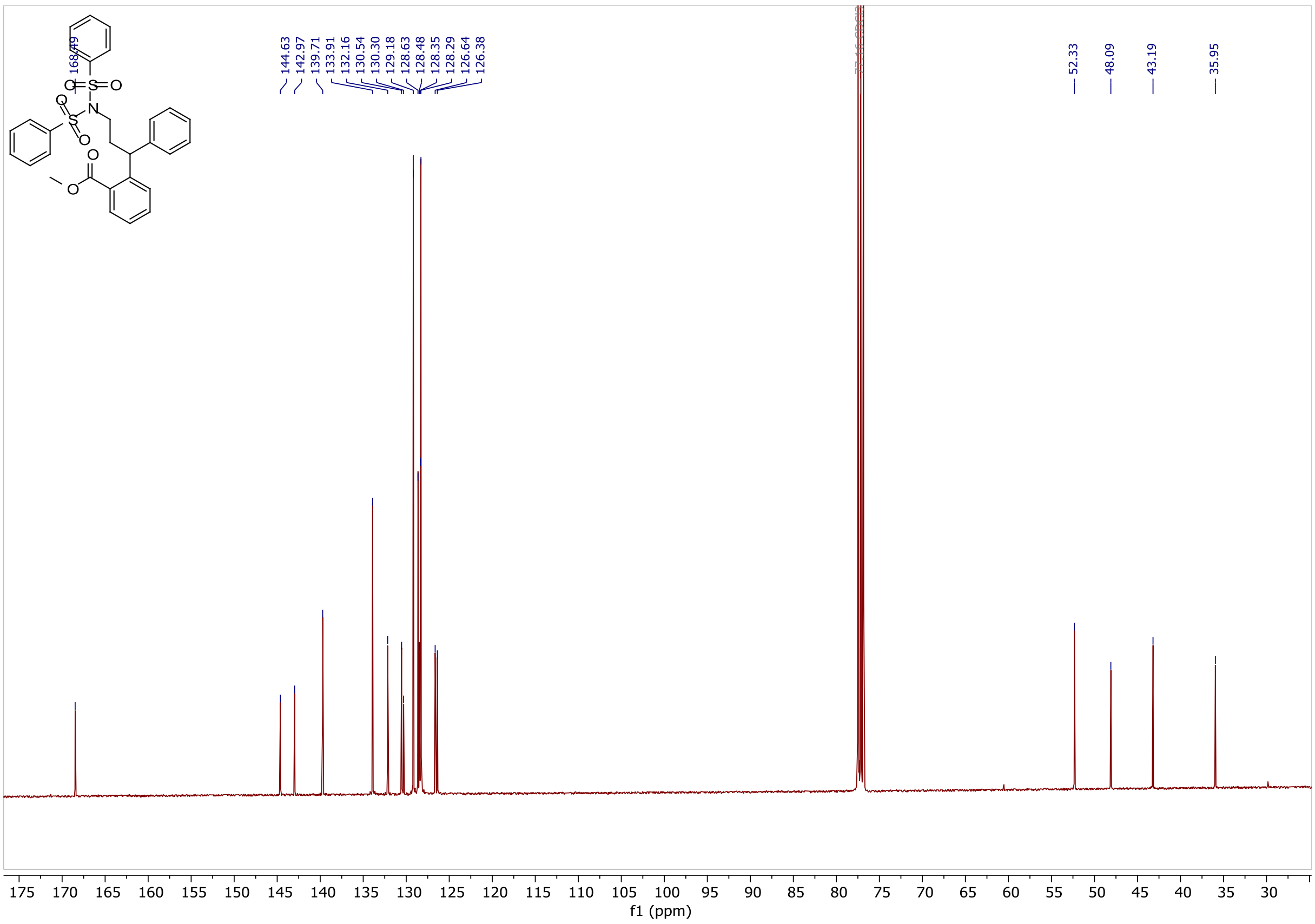

^{1}H NMR Spectrum of *m*-7w (400 MHz, $CDCl_3$)

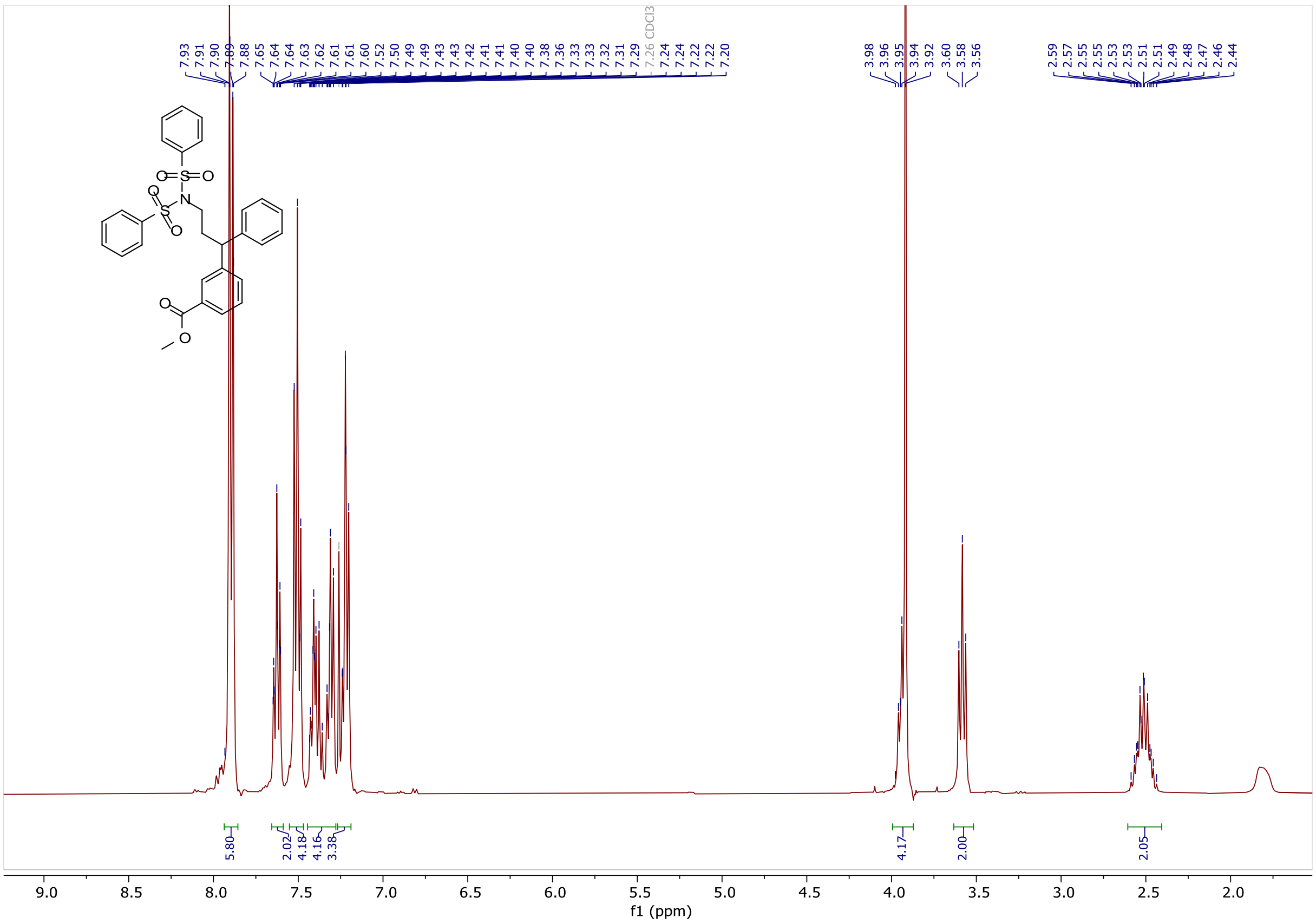

^{13}C NMR Spectrum of *m*-7w (101 MHz, $CDCl_3$)

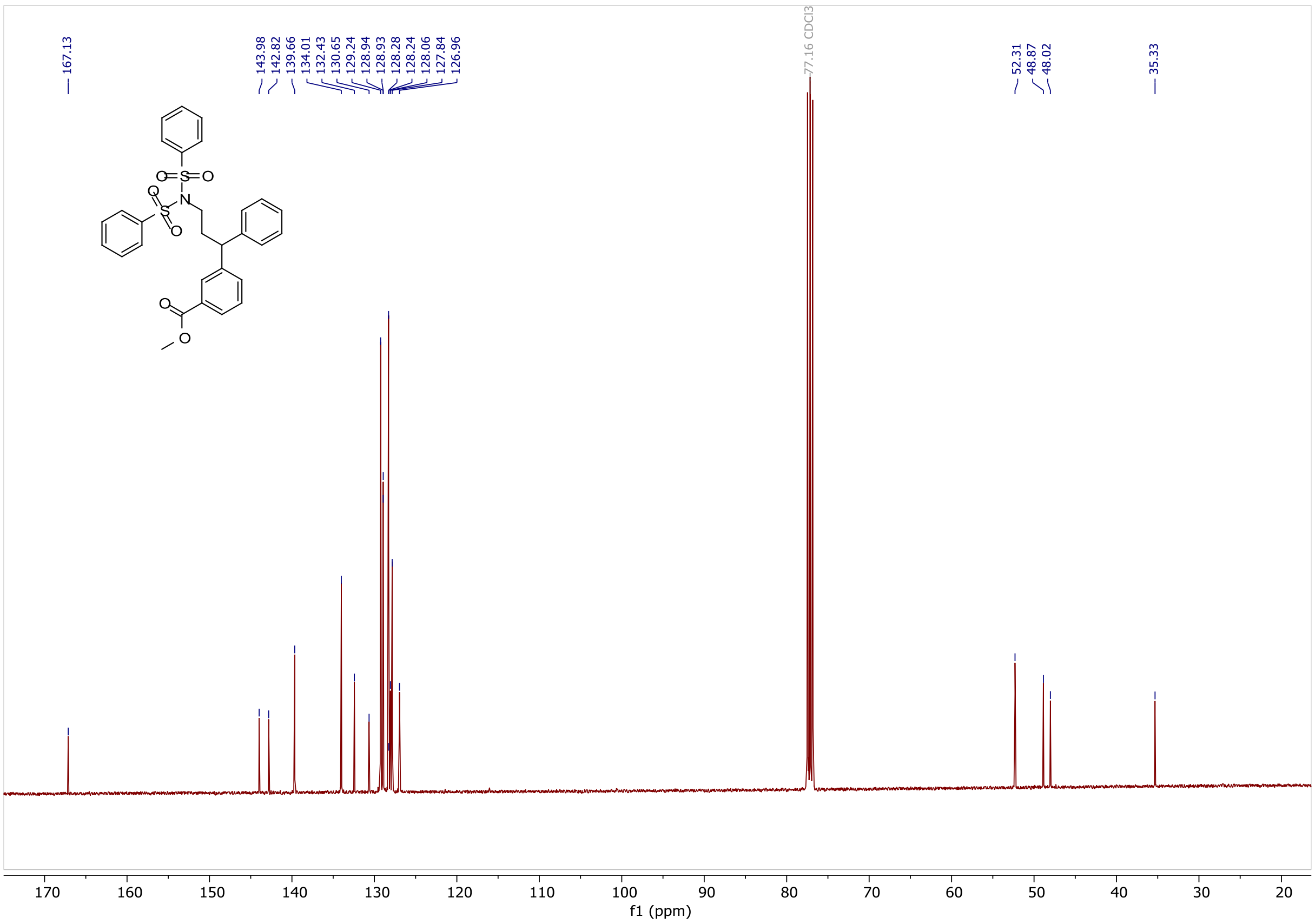

^{1}H NMR Spectrum of *p*-7w (400 MHz, CDCl3)

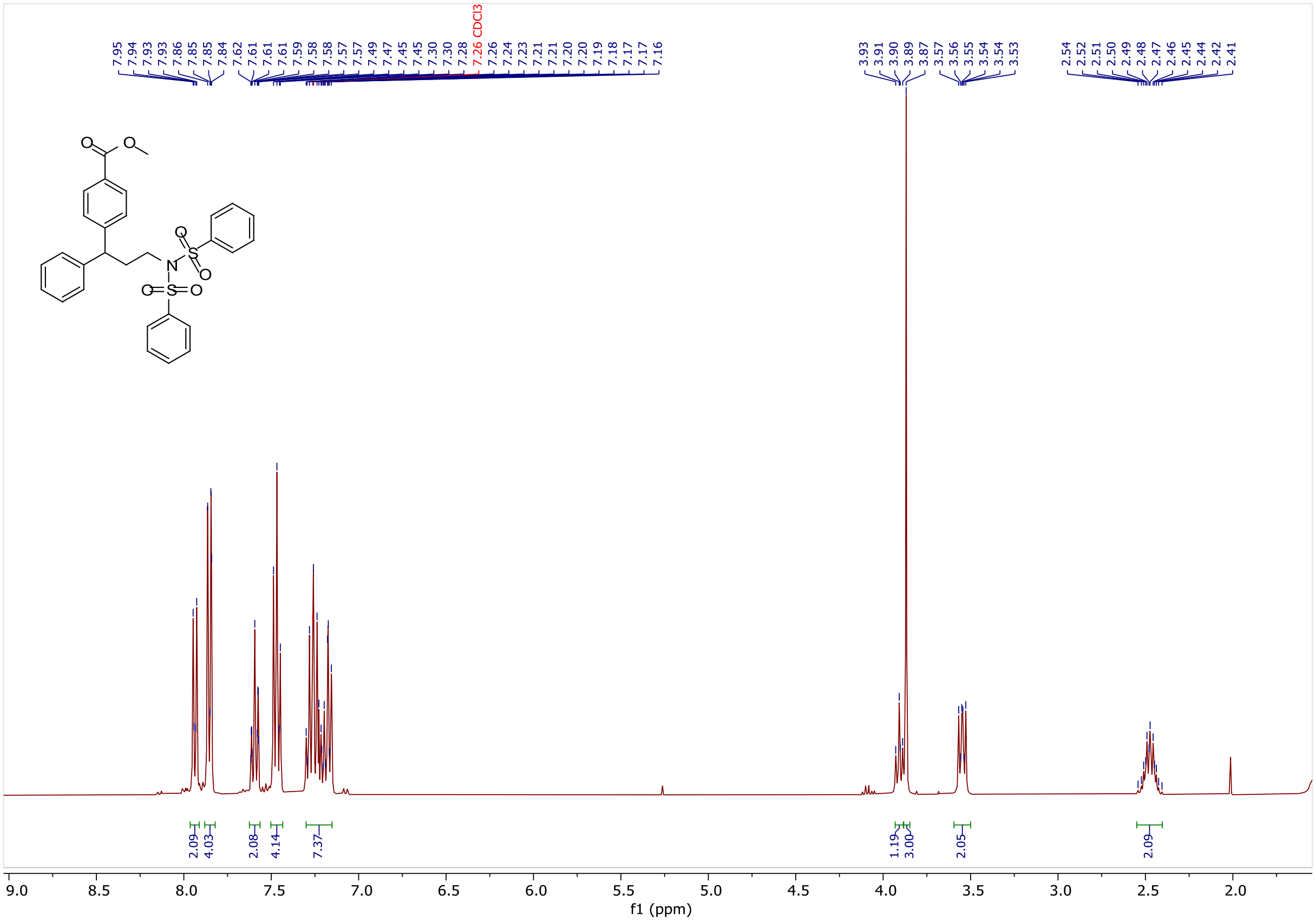

^{13}C NMR Spectrum of *p*-7w (101 MHz, CDCl3)

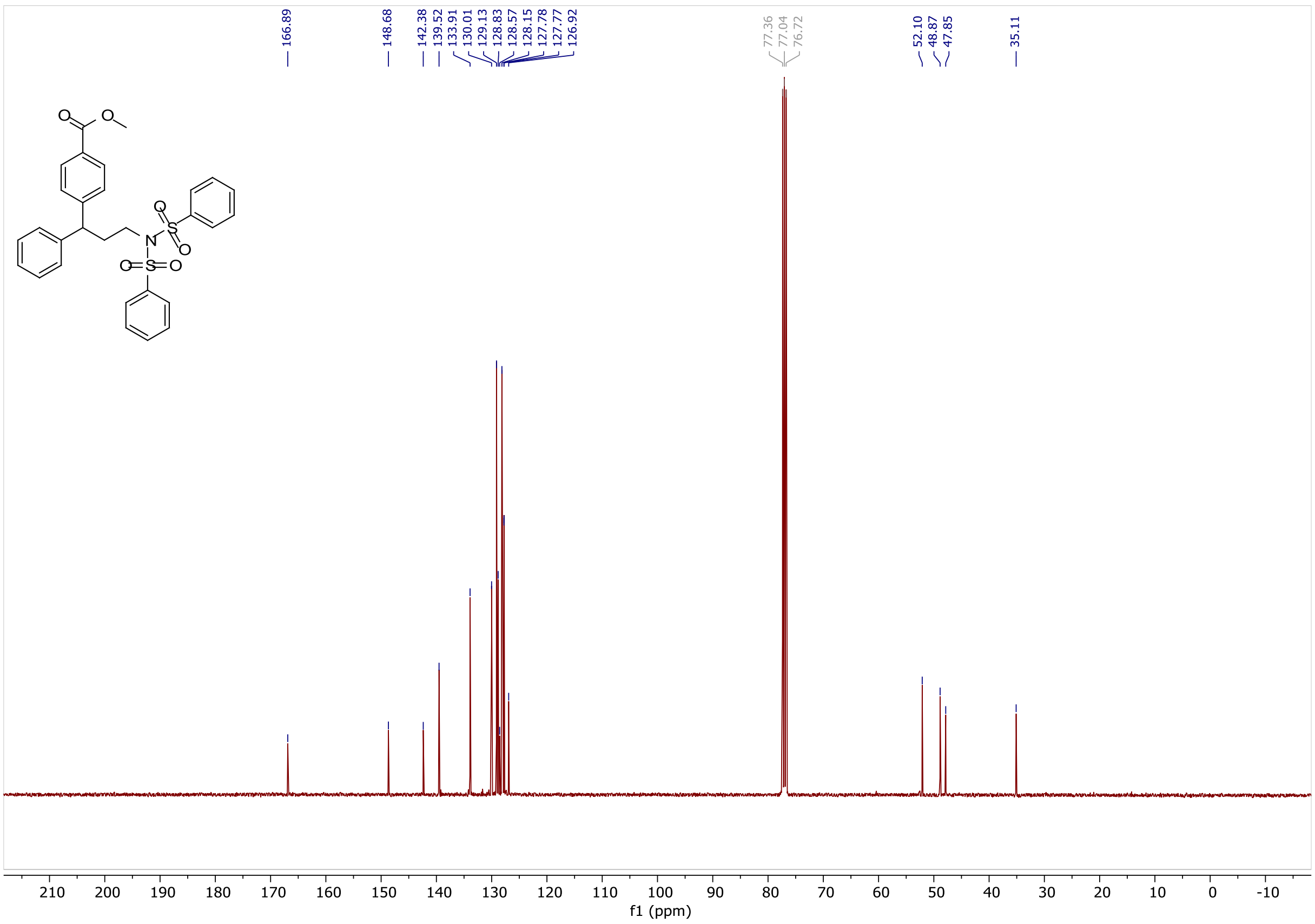

^{1}H NMR Spectrum of *o*-7x (400 MHz, $CDCl_3$)

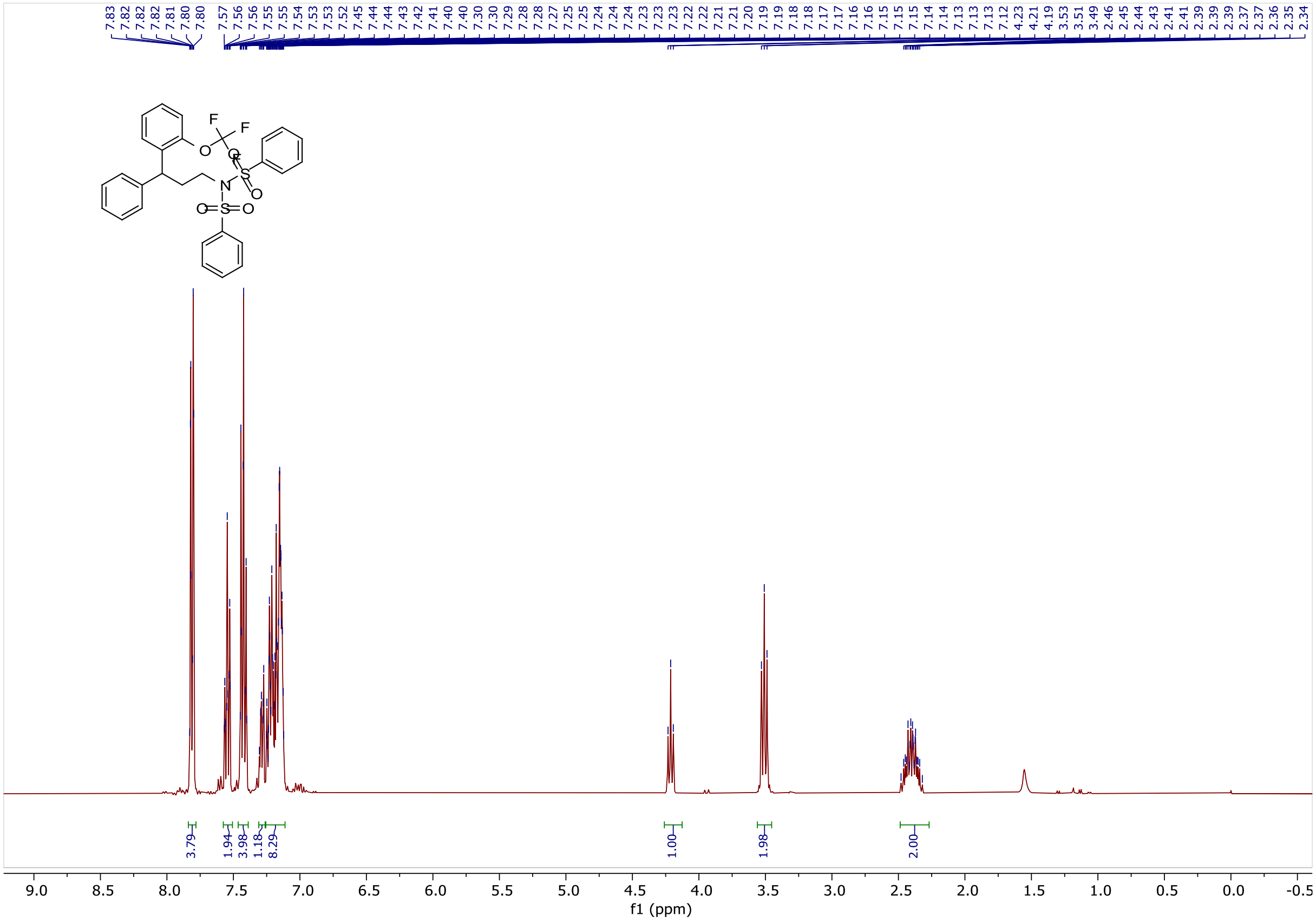

^{19}F NMR Spectrum of *o*-7x (376 MHz, $CDCl_3$)

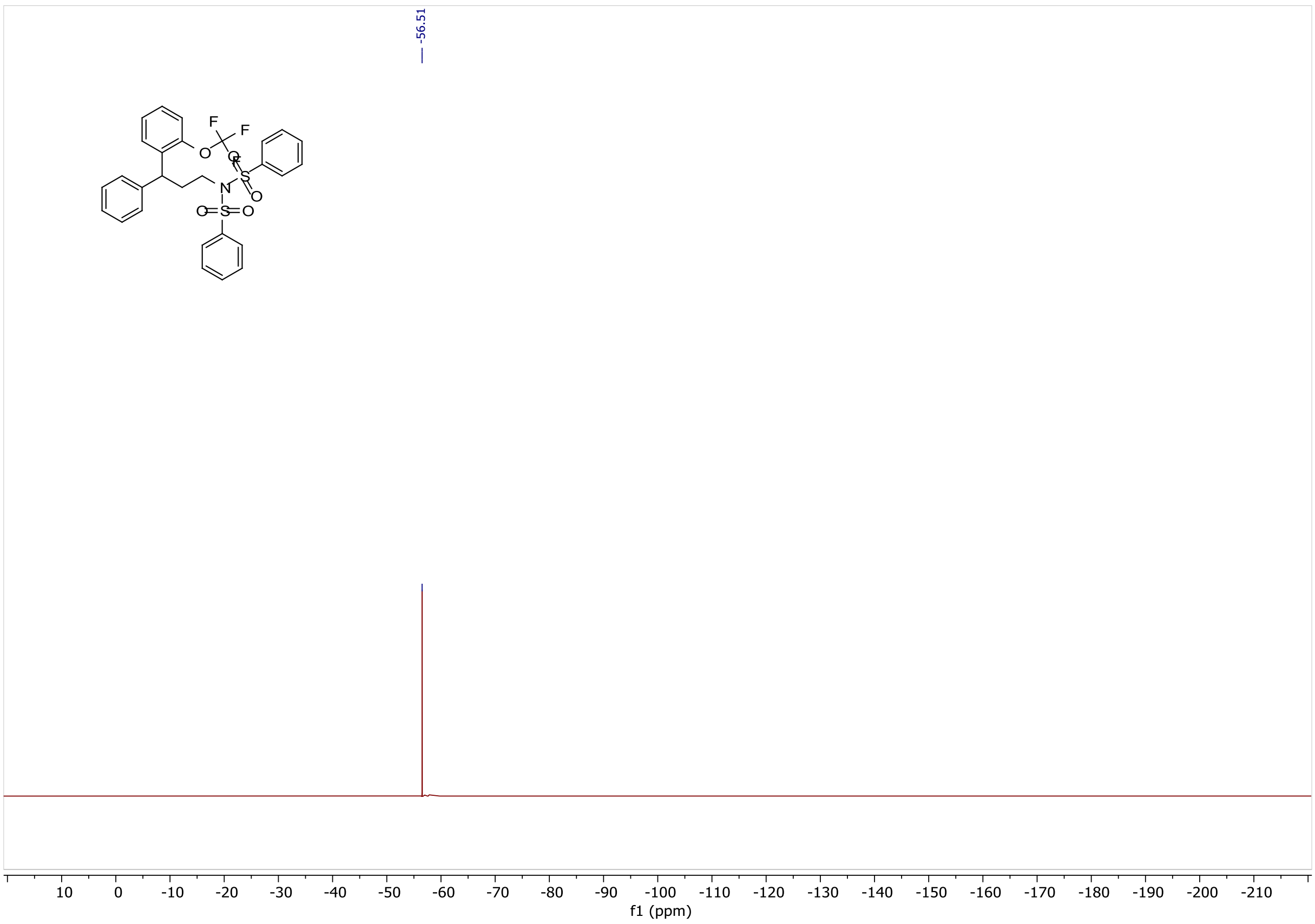

^{13}C NMR Spectrum of *o*-7x (101 MHz, $CDCl_3$)

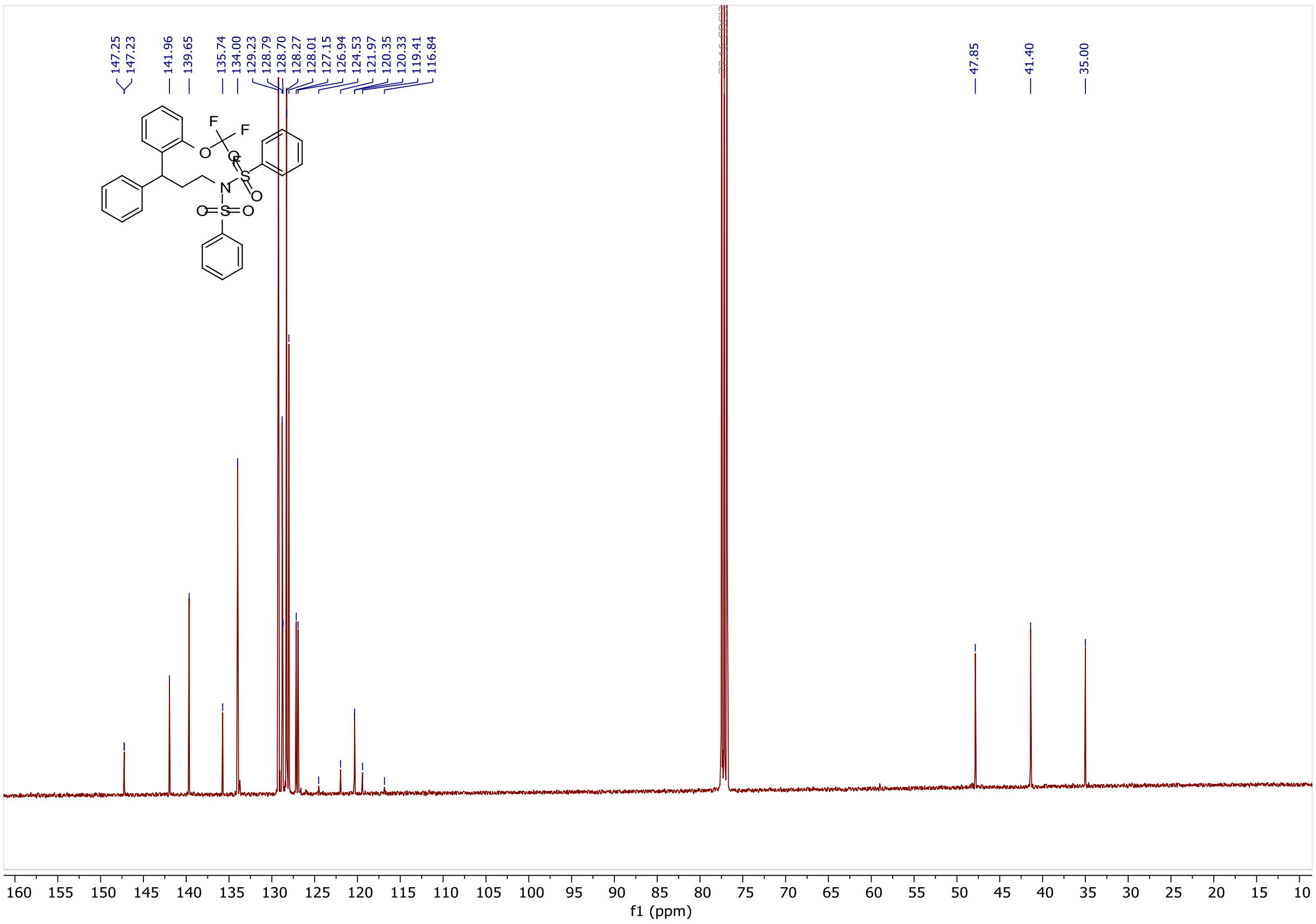

^{1}H NMR Spectrum of *m*-7x (400 MHz, $CDCl_3$)

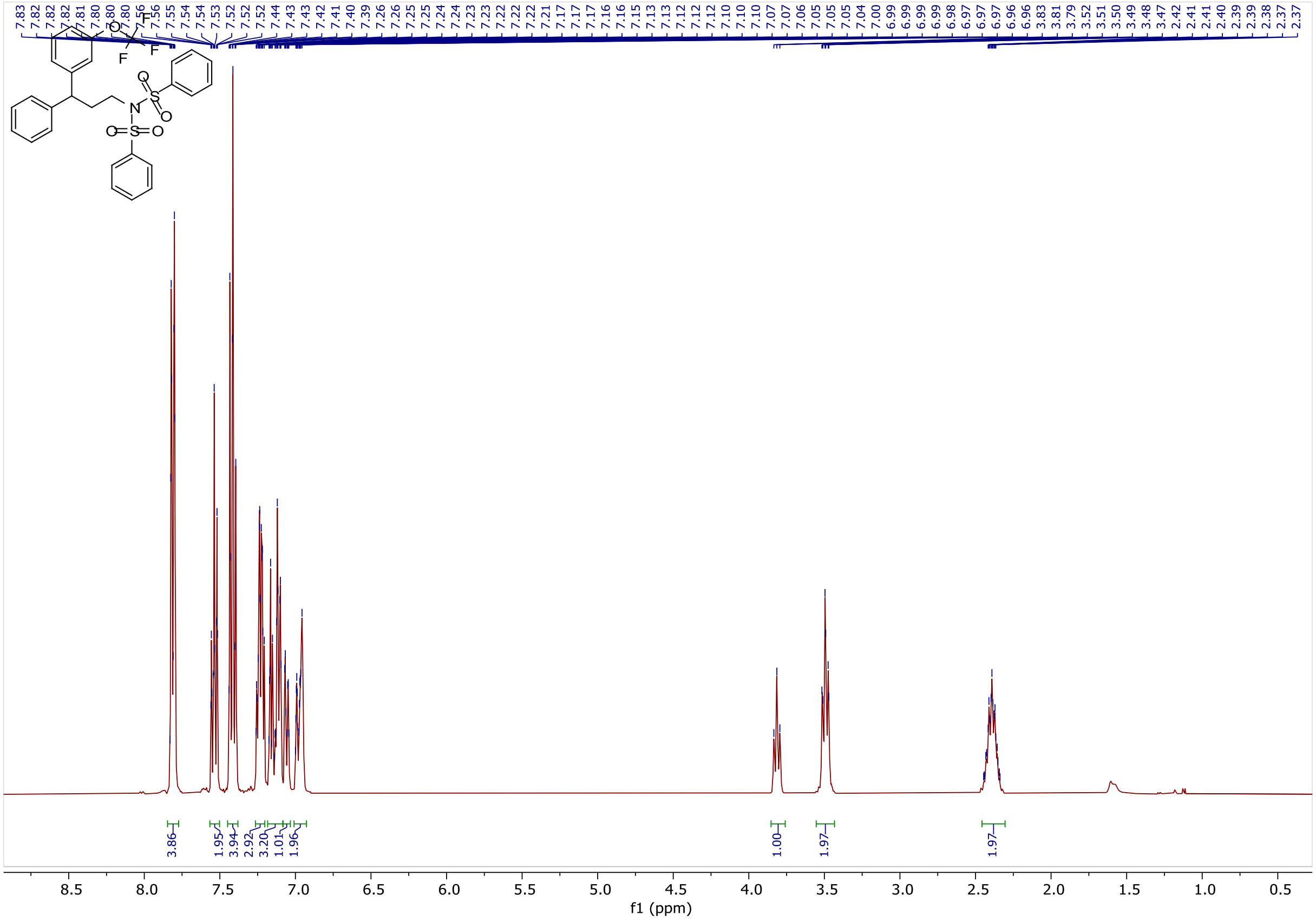

^{19}F NMR Spectrum of *m*-7x (376 MHz, $CDCl_3$)

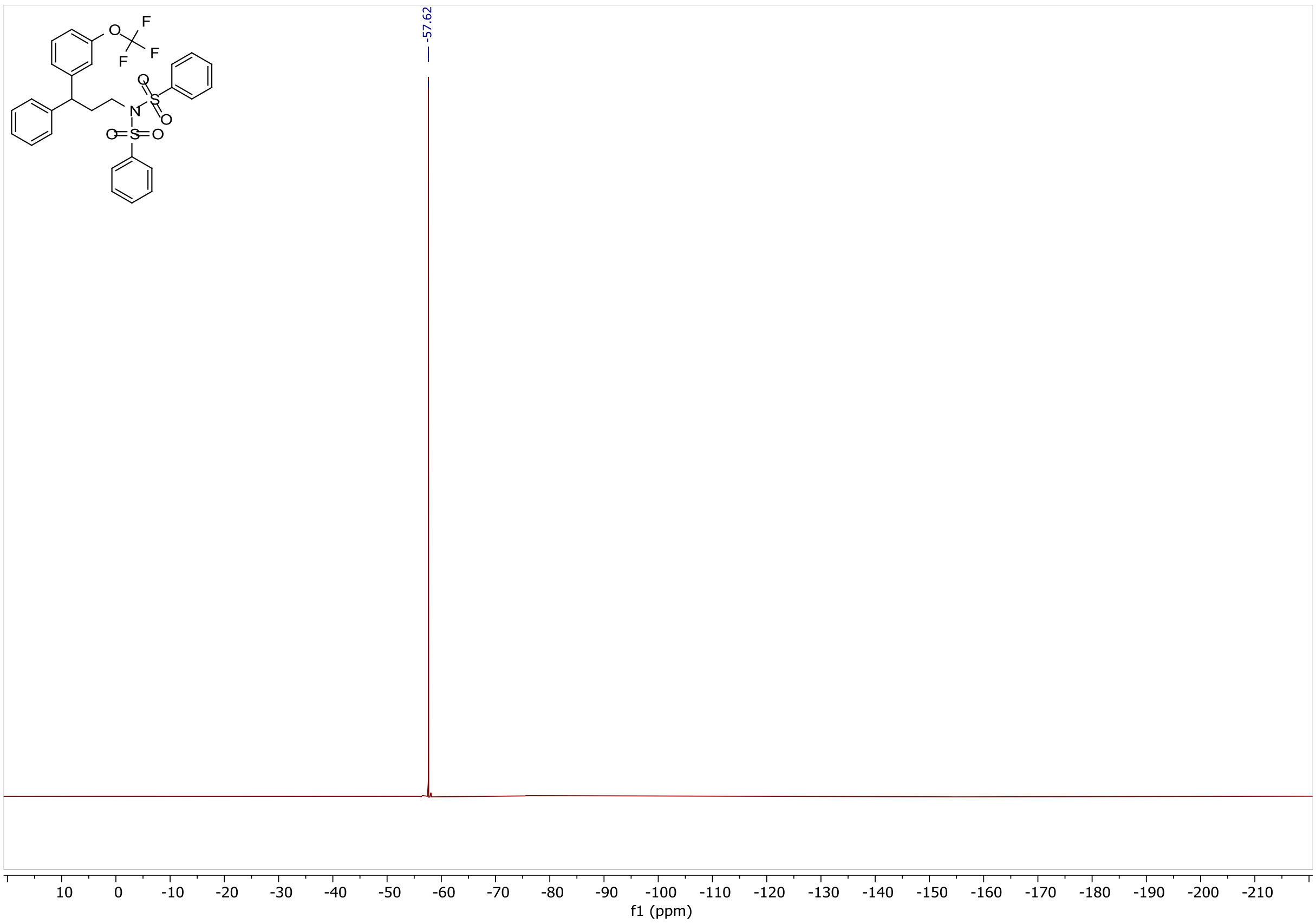

^{13}C NMR Spectrum of *m*-7x (101 MHz, $CDCl_3$)

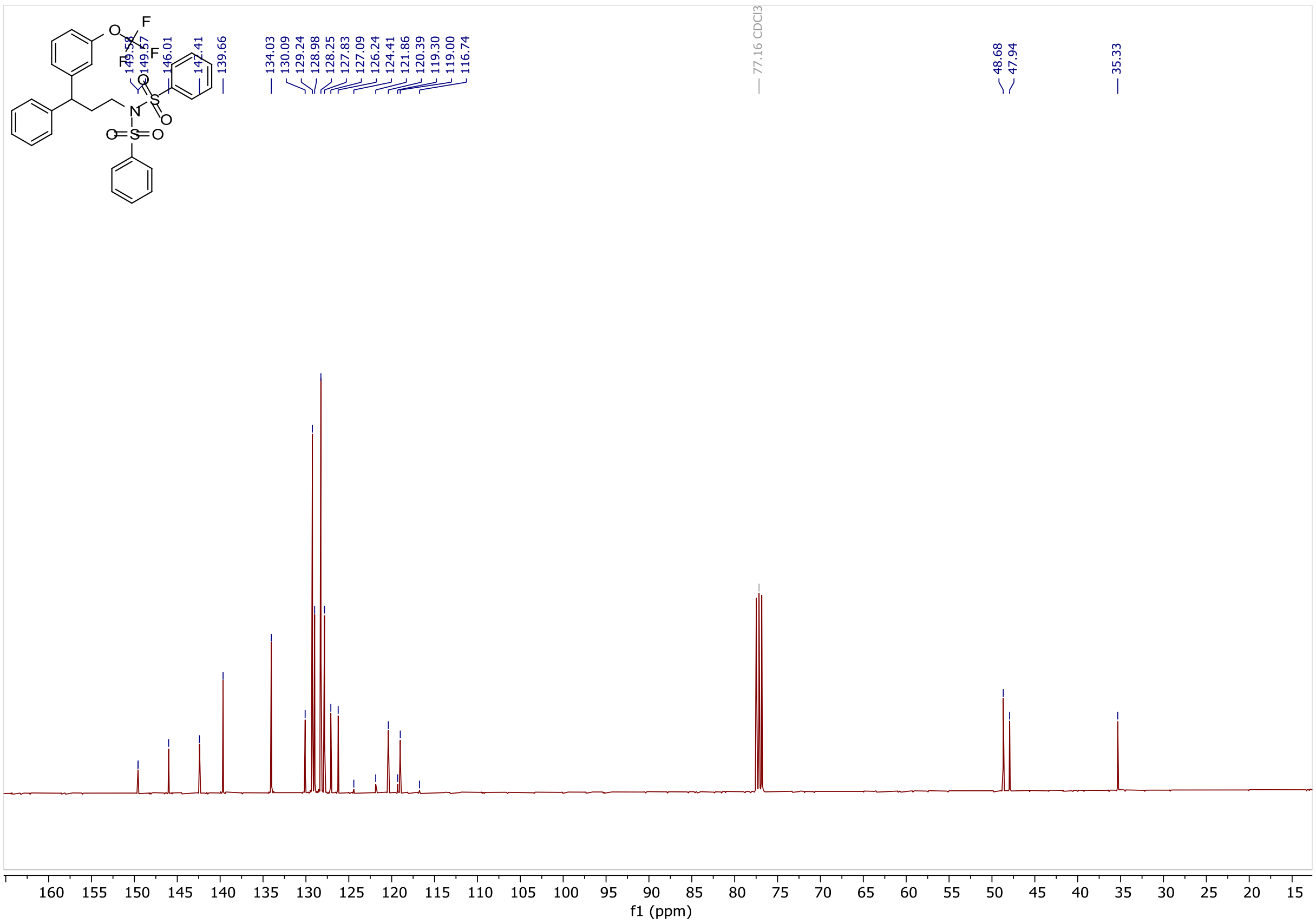

^{1}H NMR Spectrum of *p*-7x (400 MHz, $CDCl_3$)

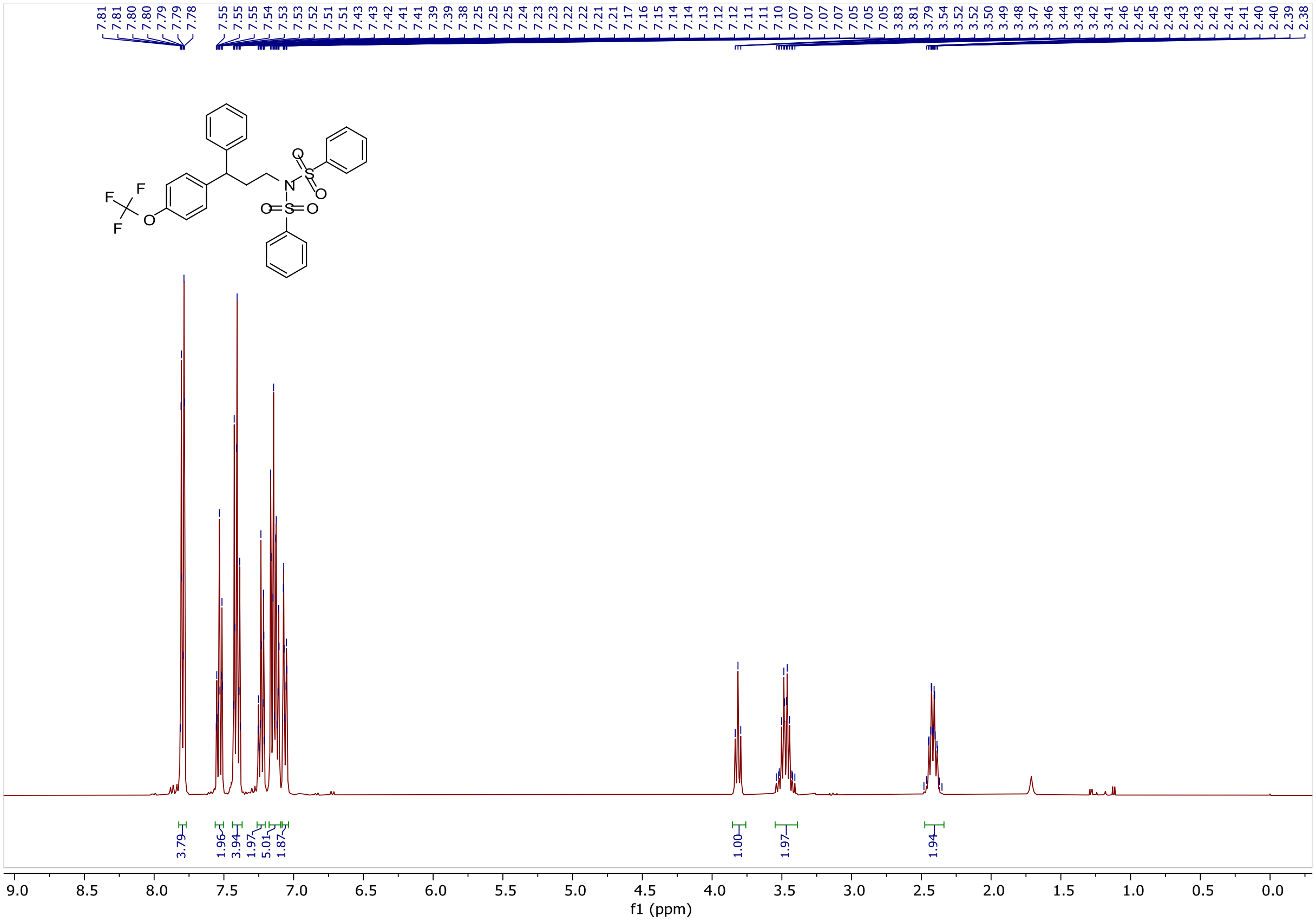

^{19}F NMR Spectrum of *p*-7x (376 MHz, $CDCl_3$)

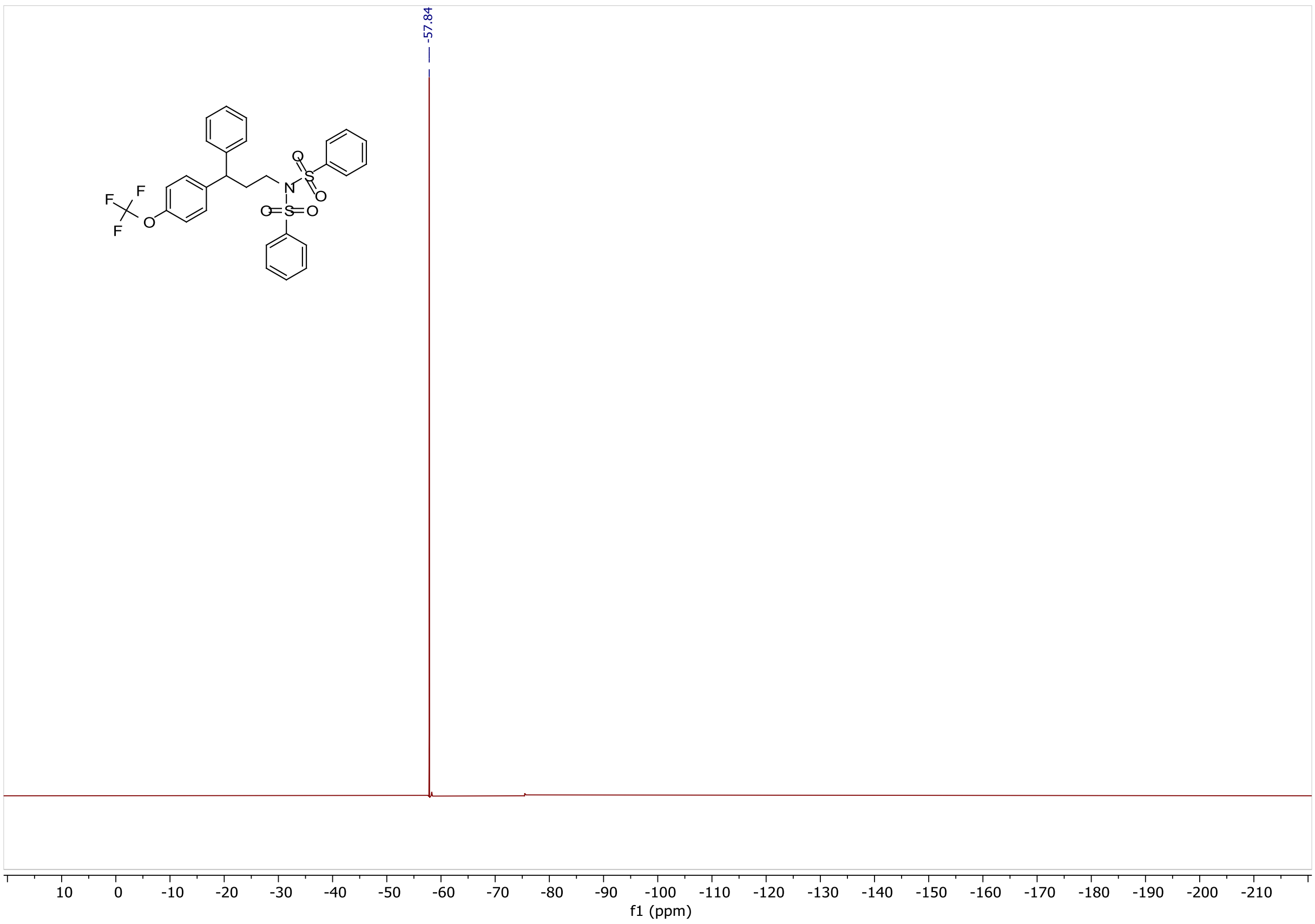

^{13}C NMR Spectrum of *p*-7x (101 MHz, $CDCl_3$)

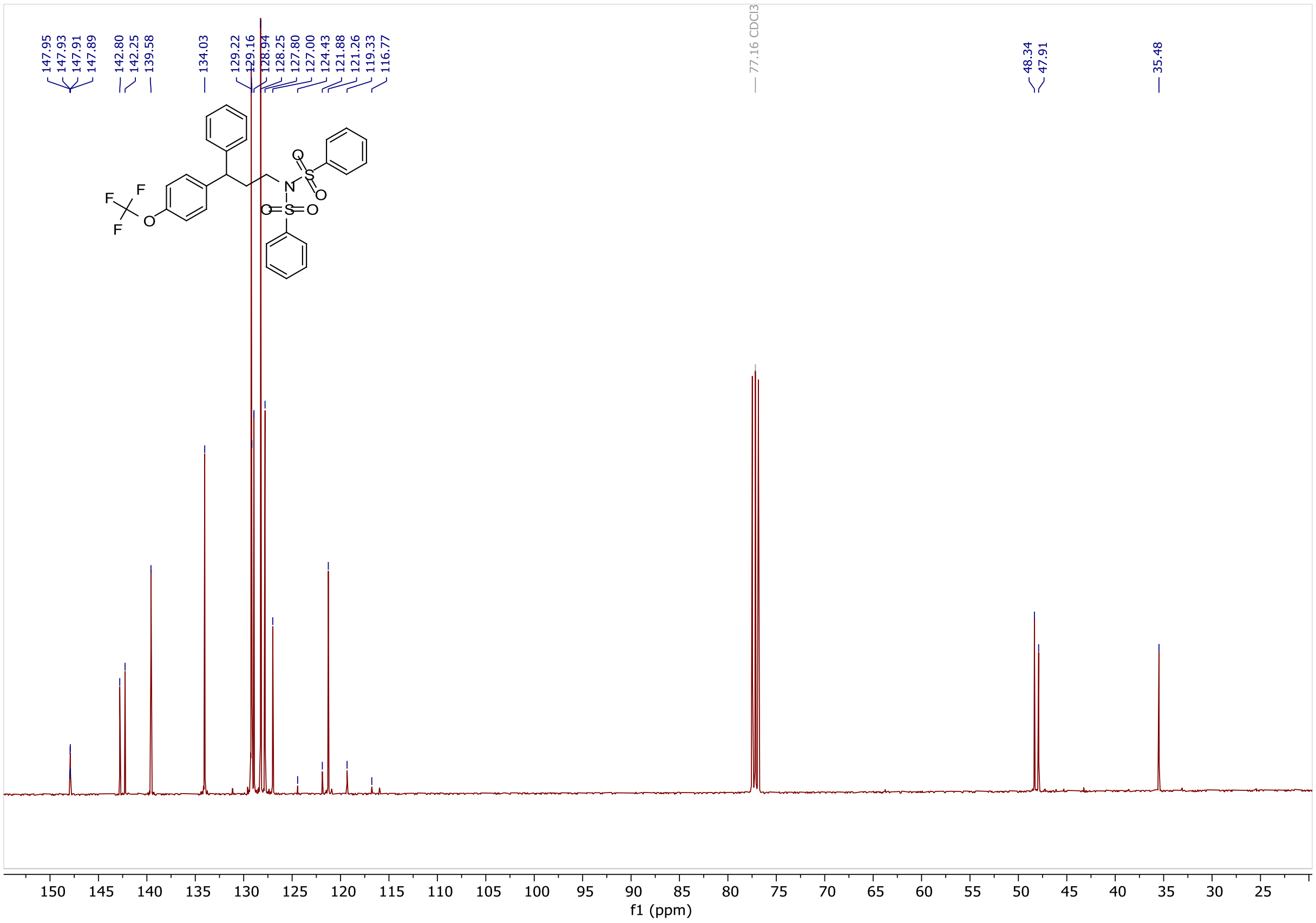

^{1}H NMR Spectrum of 7y (400 MHz, $CDCl_3$)

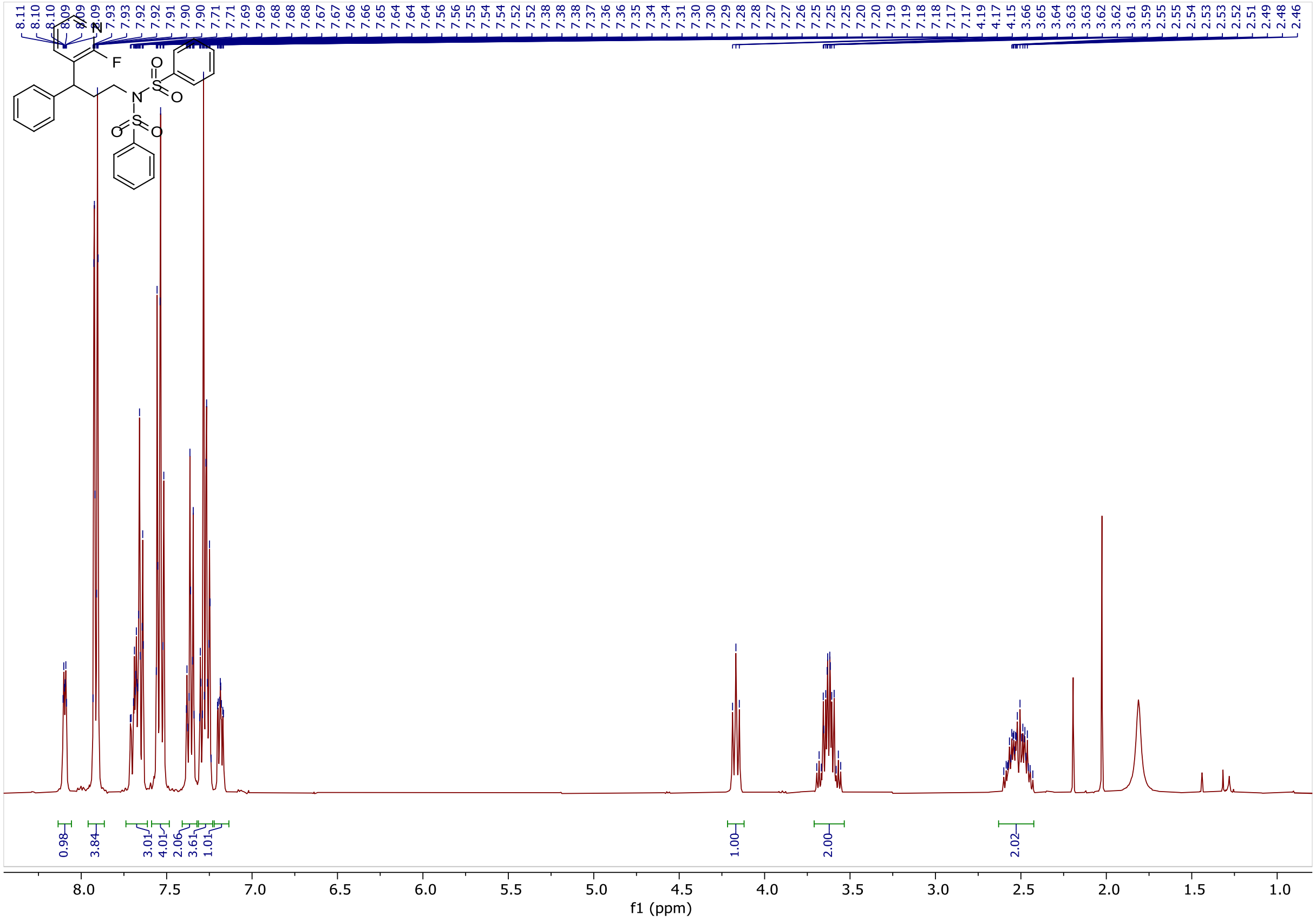

^{19}F NMR Spectrum of 7y (376 MHz, $CDCl_3$)

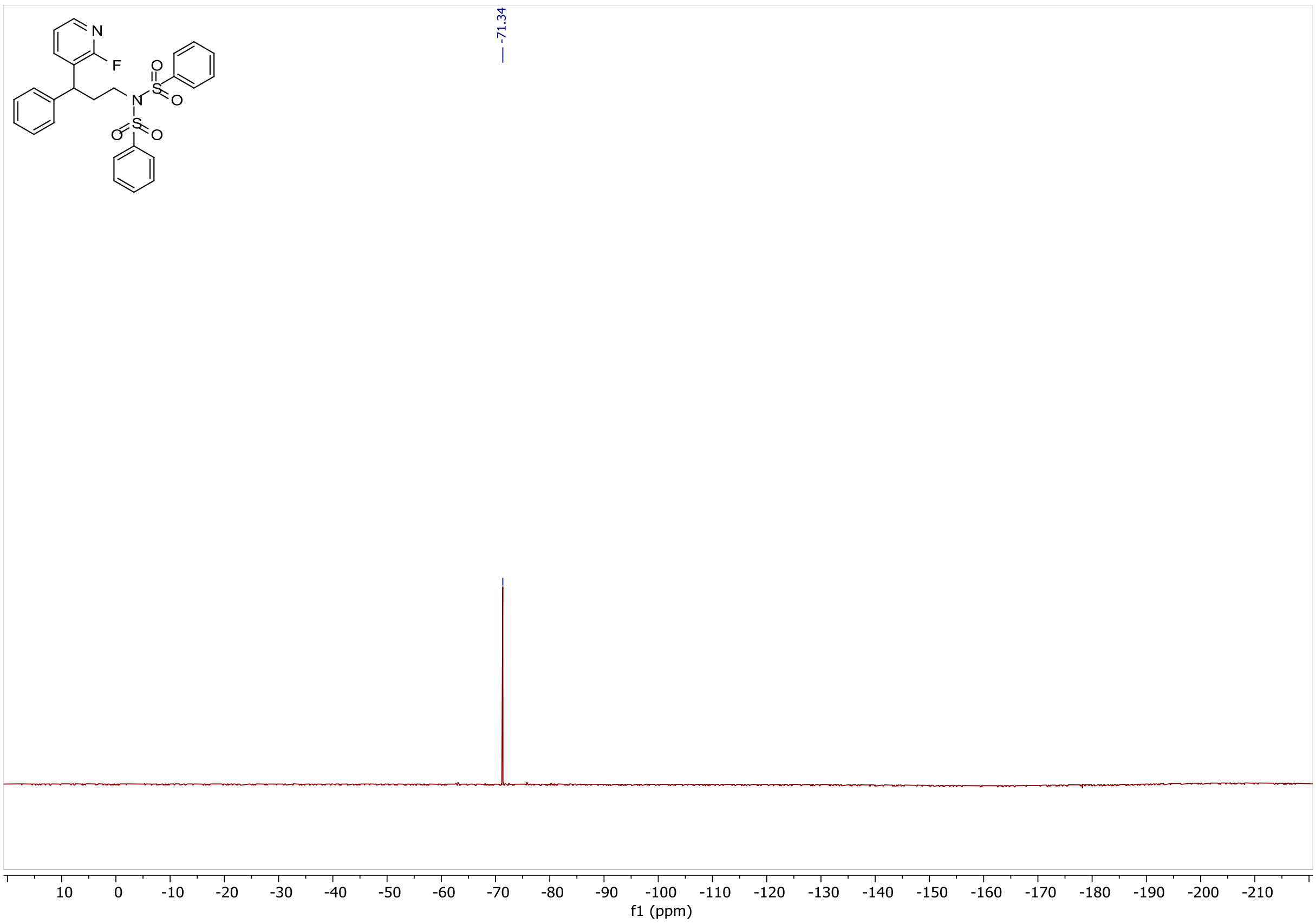

^{13}C NMR Spectrum of 7y (101 MHz, $CDCl_3$)

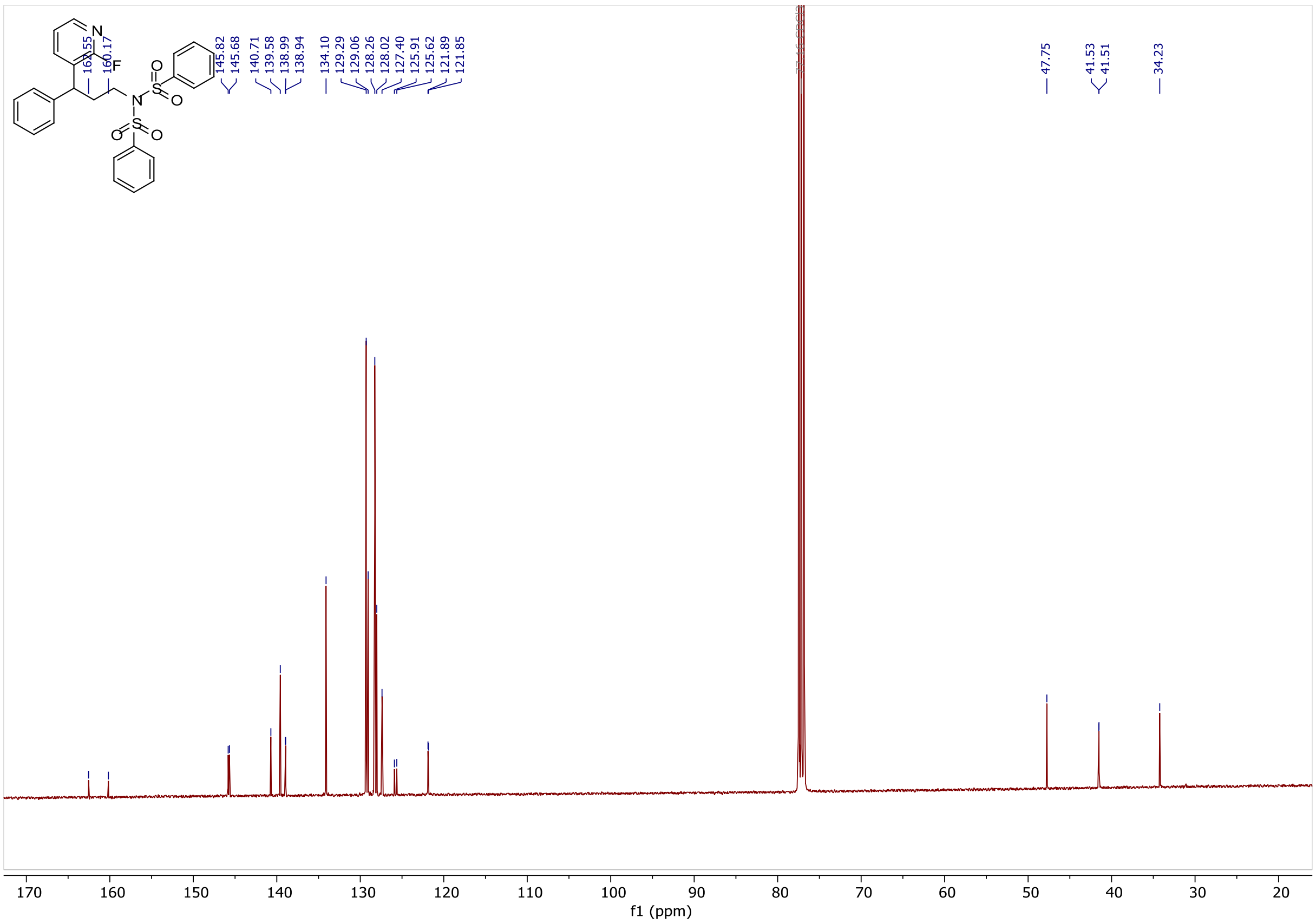

^{1}H NMR Spectrum of 7z (400 MHz, $CDCl_3$)

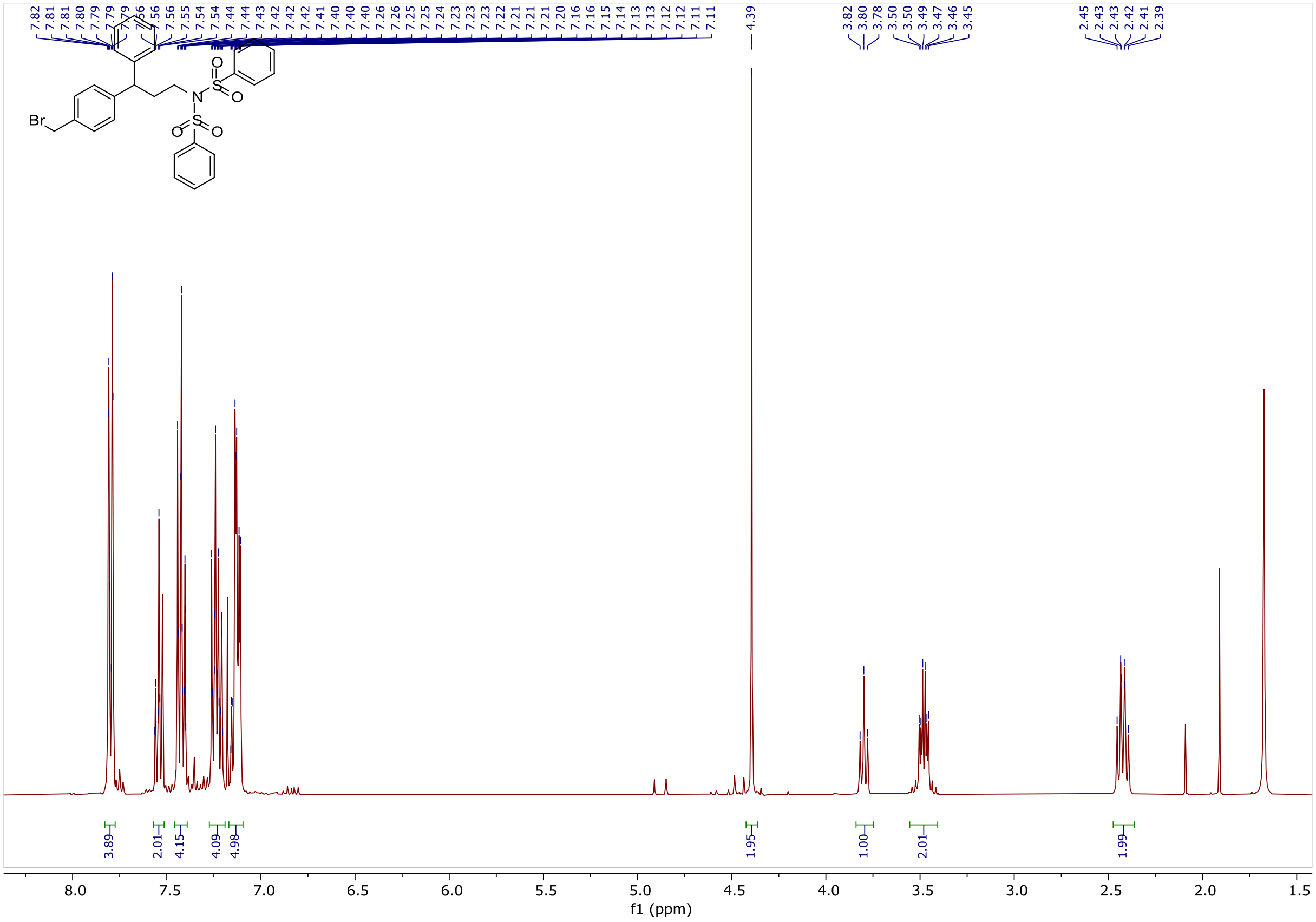

^{13}C NMR Spectrum of 7z (101 MHz, $CDCl_3$)

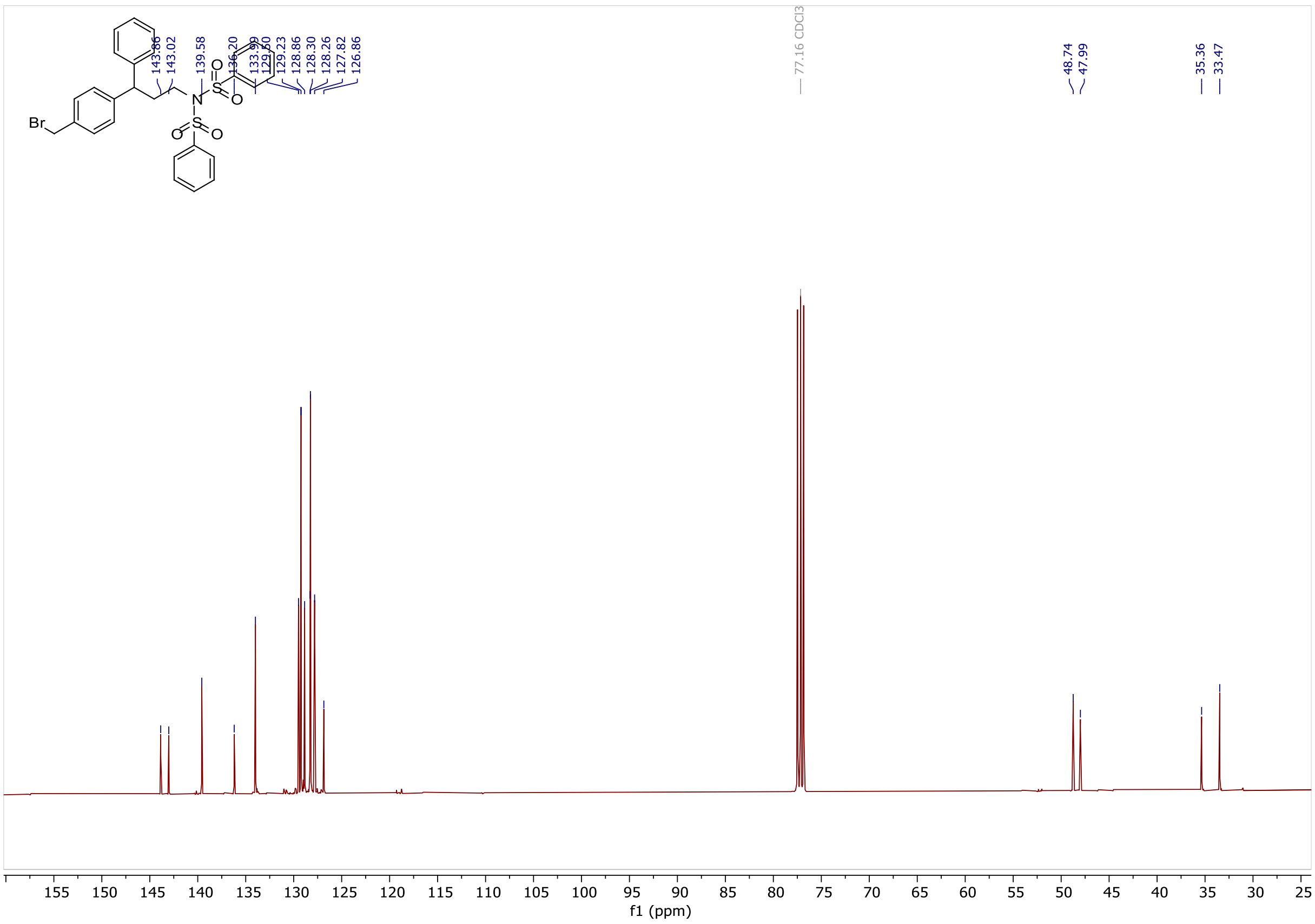

^{1}H NMR Spectrum of 7aa (400 MHz, $CDCl_3$)

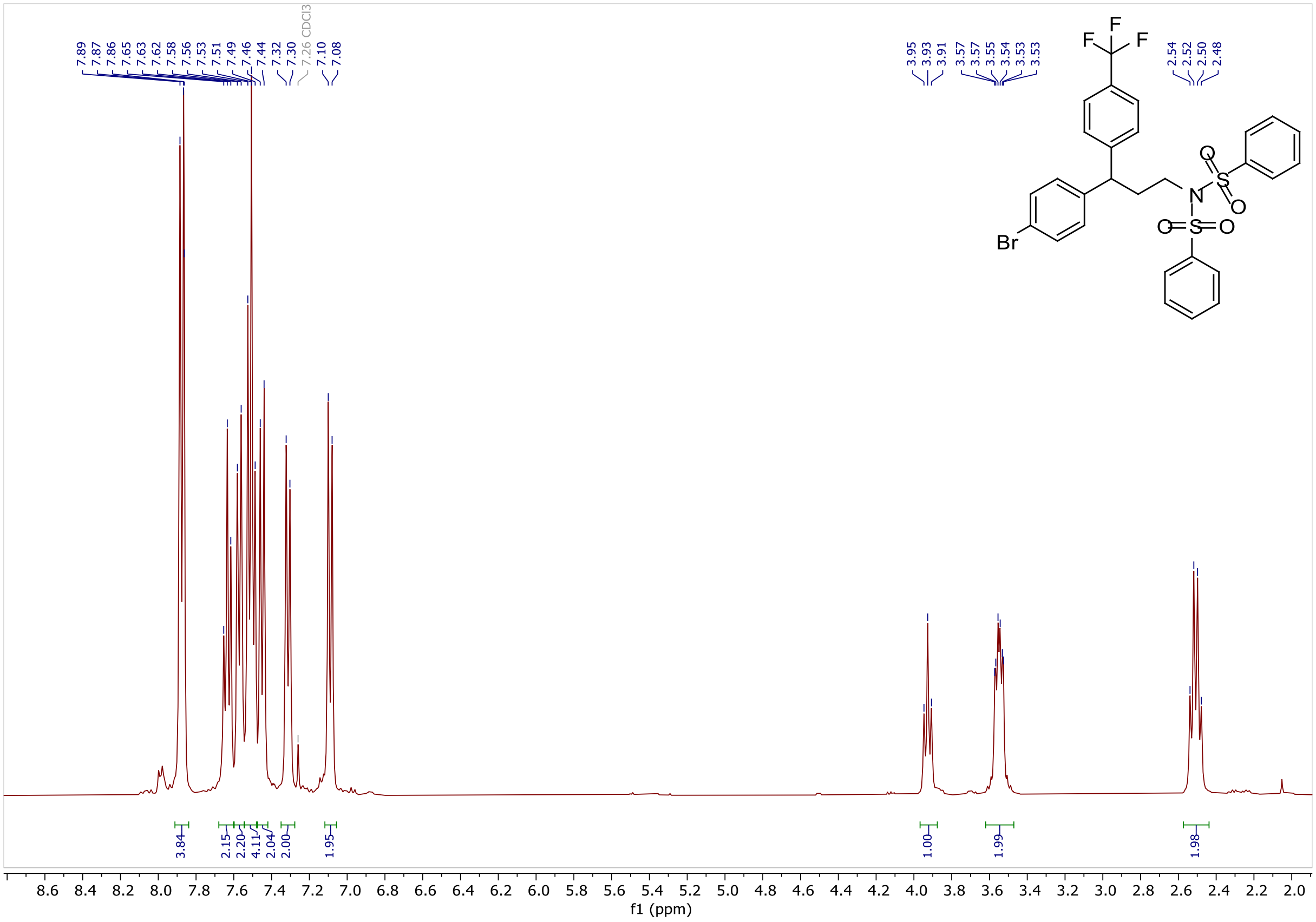

^{19}F NMR Spectrum of 7aa (376 MHz, $CDCl_3$)

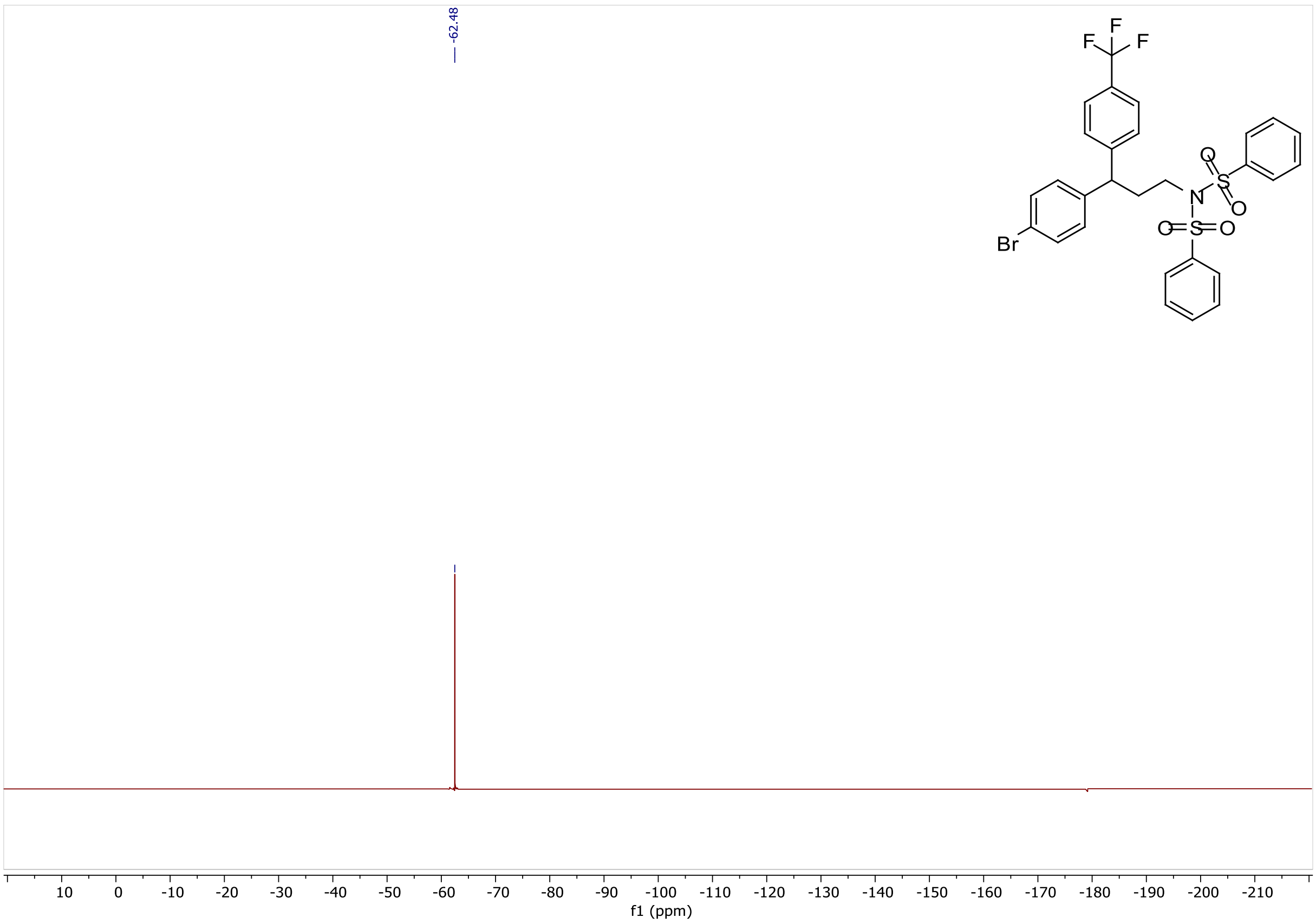

^{13}C NMR Spectrum of 7aa (101 MHz, $CDCl_3$)

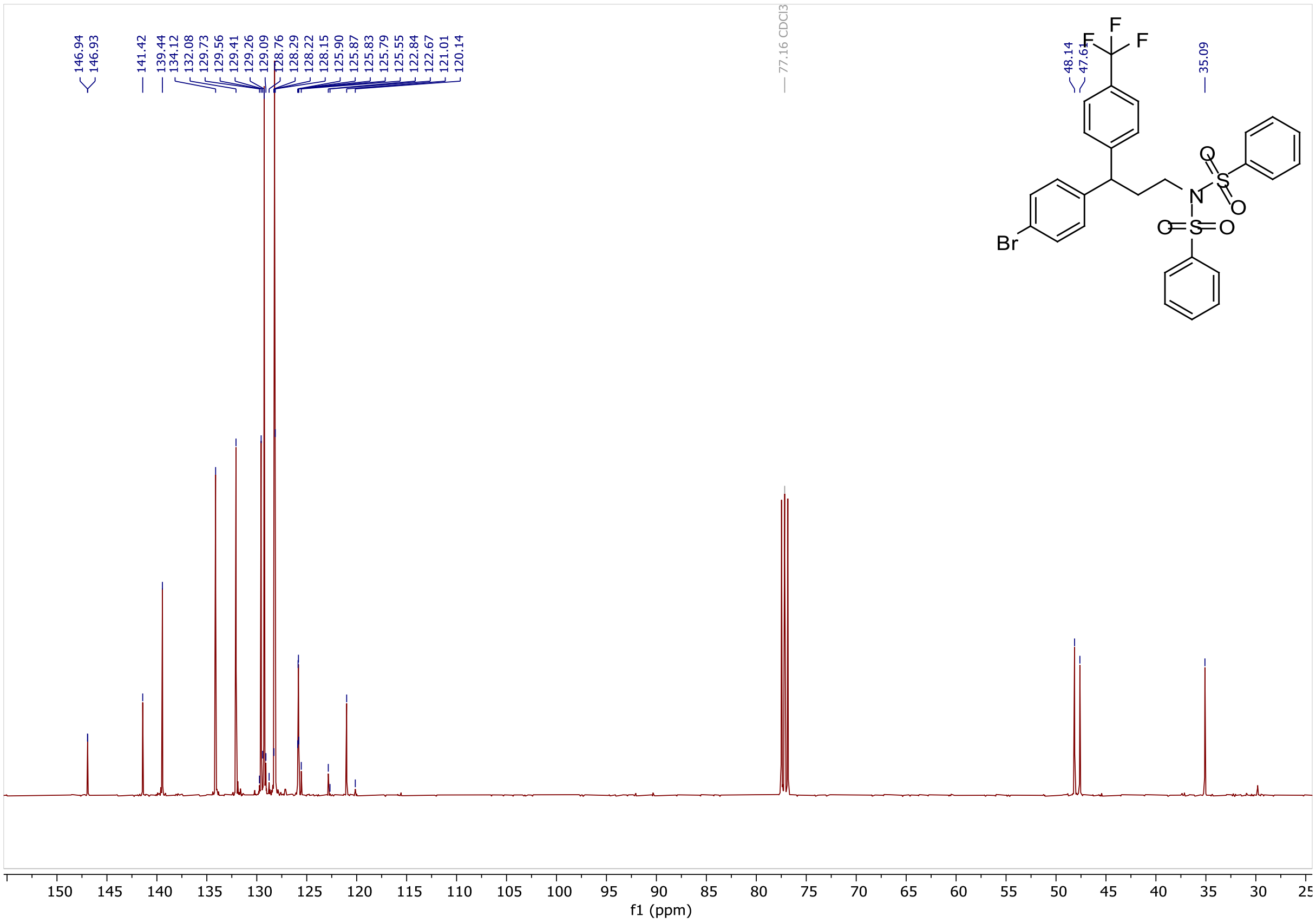

^{1}H NMR Spectrum of 7ab (400 MHz, $CDCl_3$)

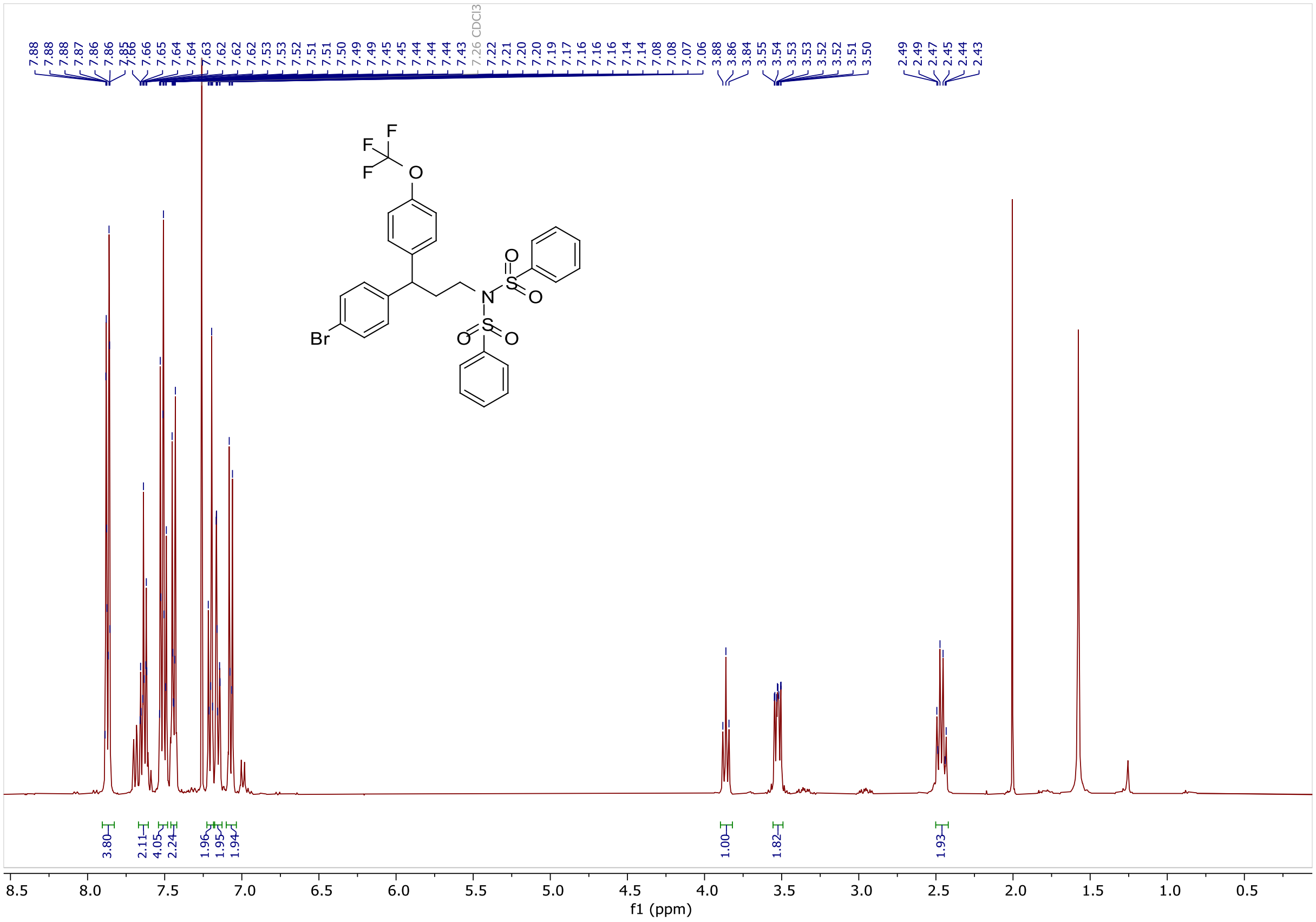

^{19}F NMR Spectrum of 7ab (376 MHz, $CDCl_3$)

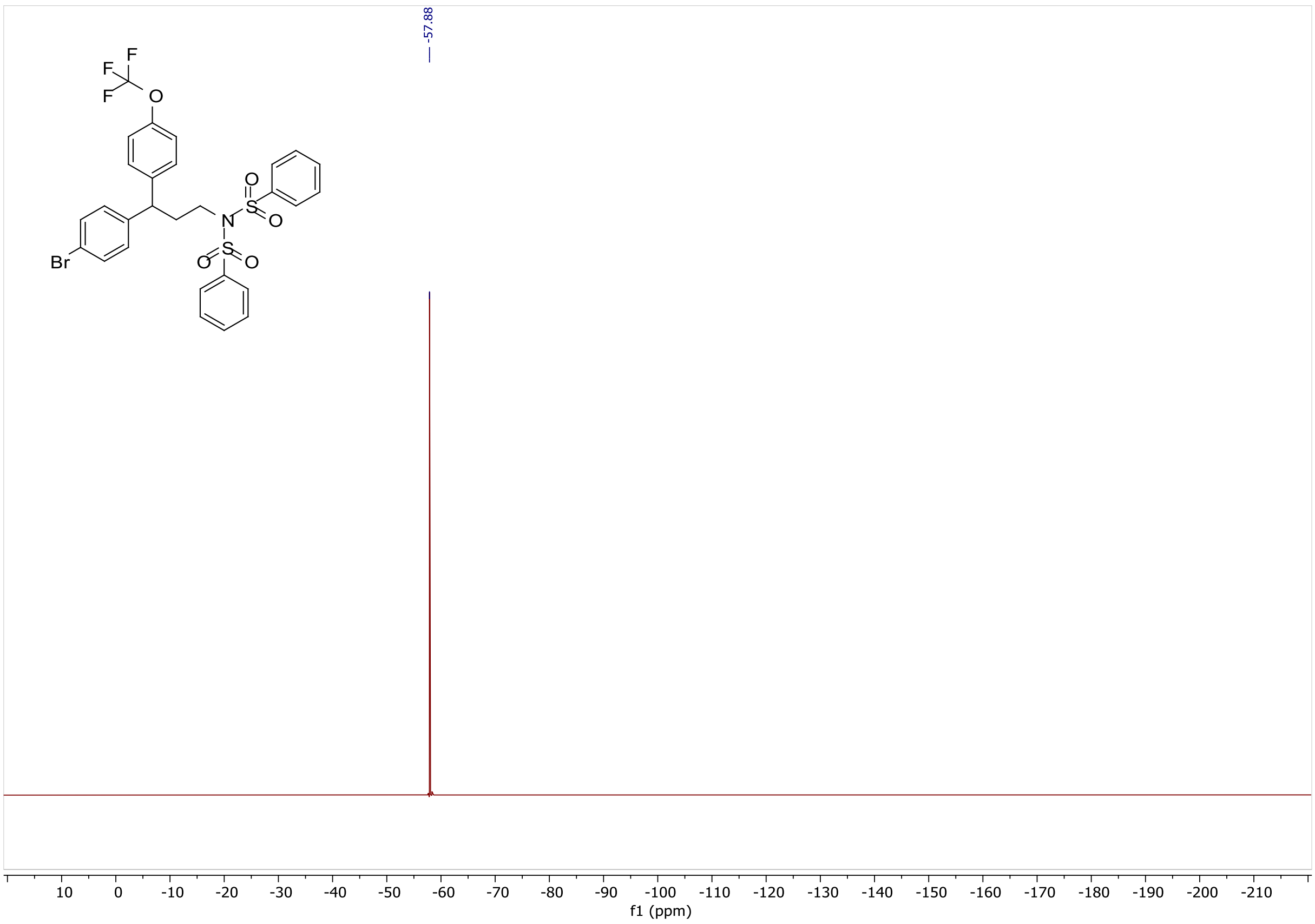

^{13}C NMR Spectrum of 7ab (101 MHz, CDCl$_3$)

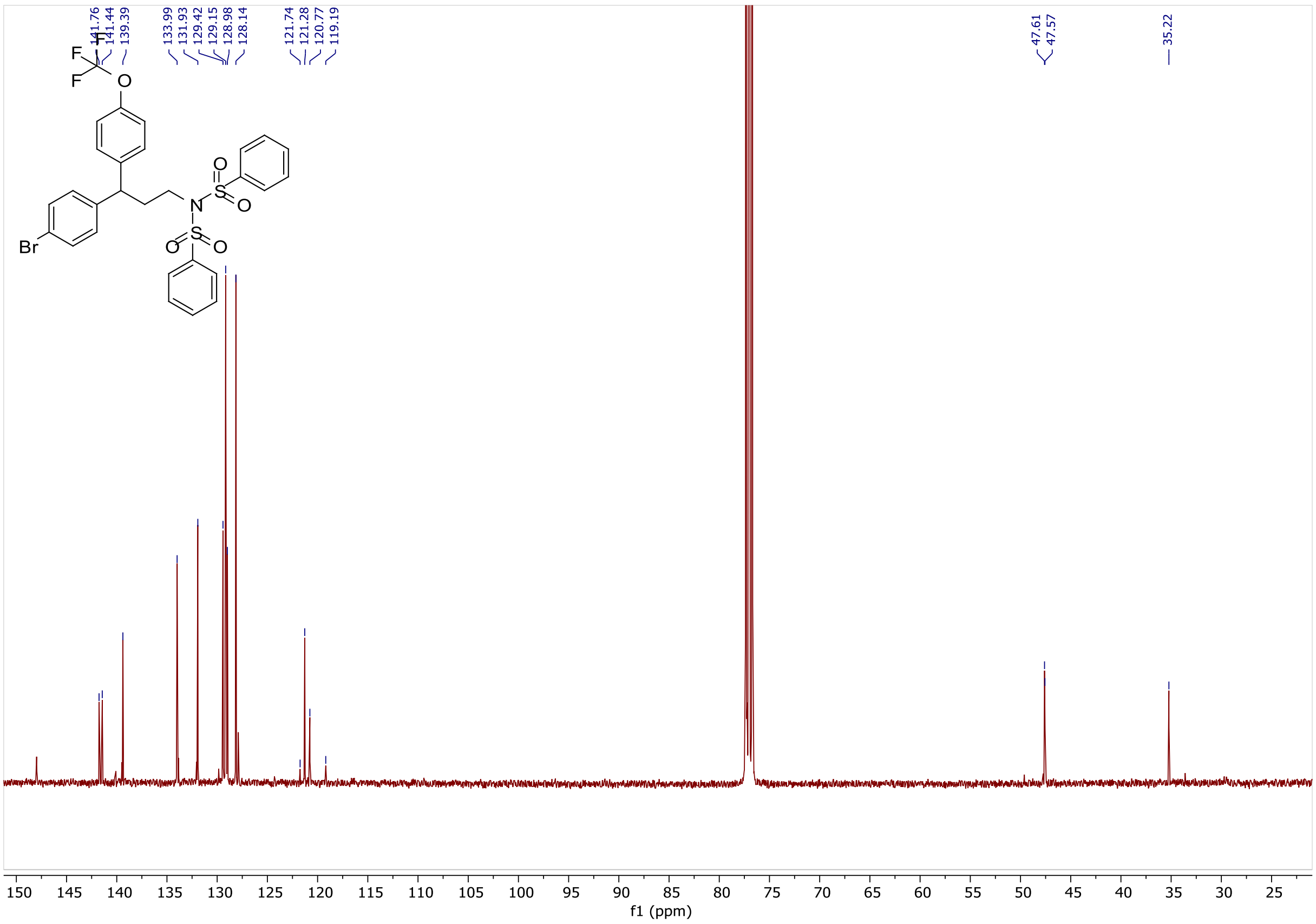

Other products

^{1}H NMR Spectrum of 8a (400 MHz, $CDCl_3$)

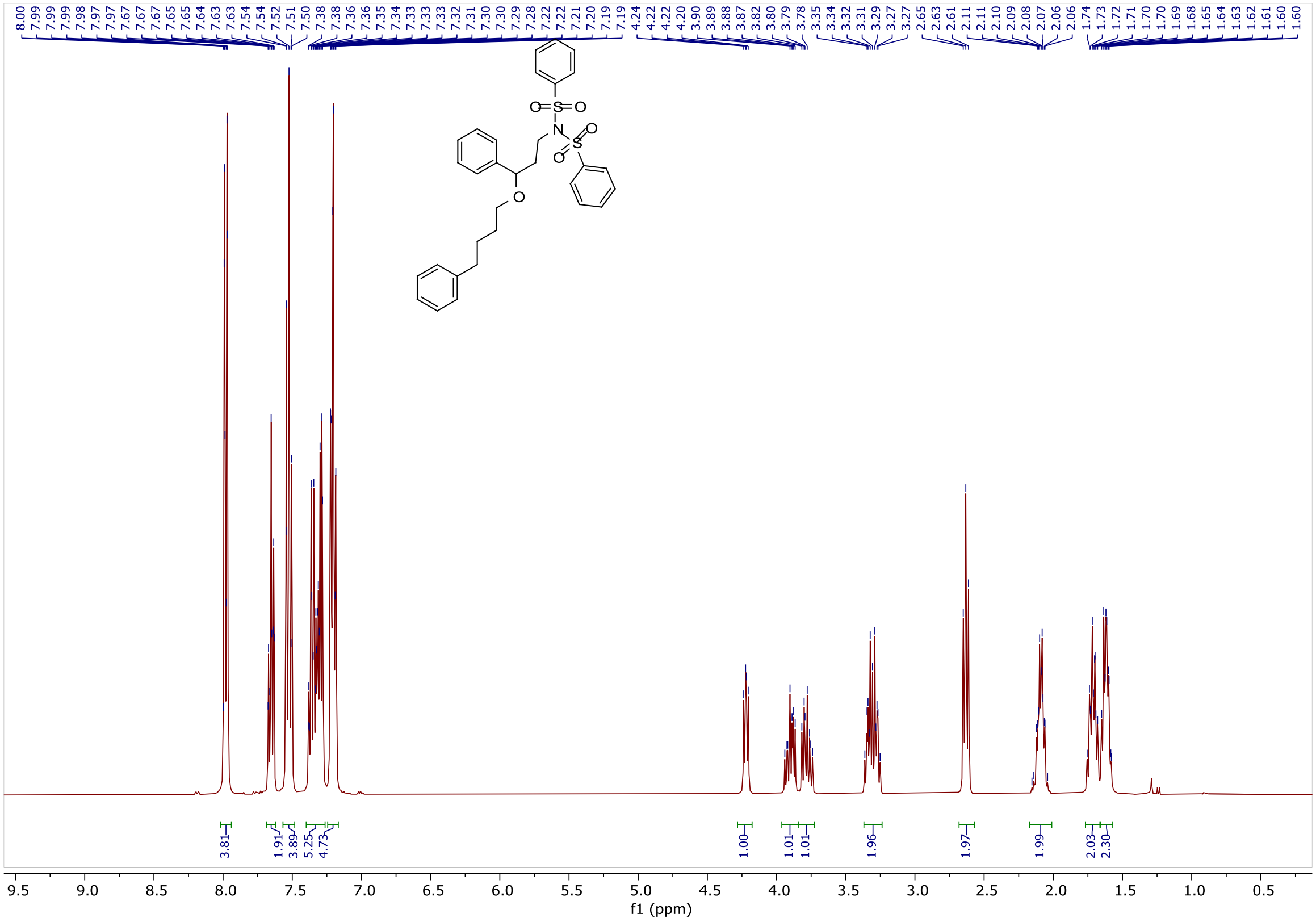

^{13}C NMR Spectrum of 8a (101 MHz, $CDCl_3$)

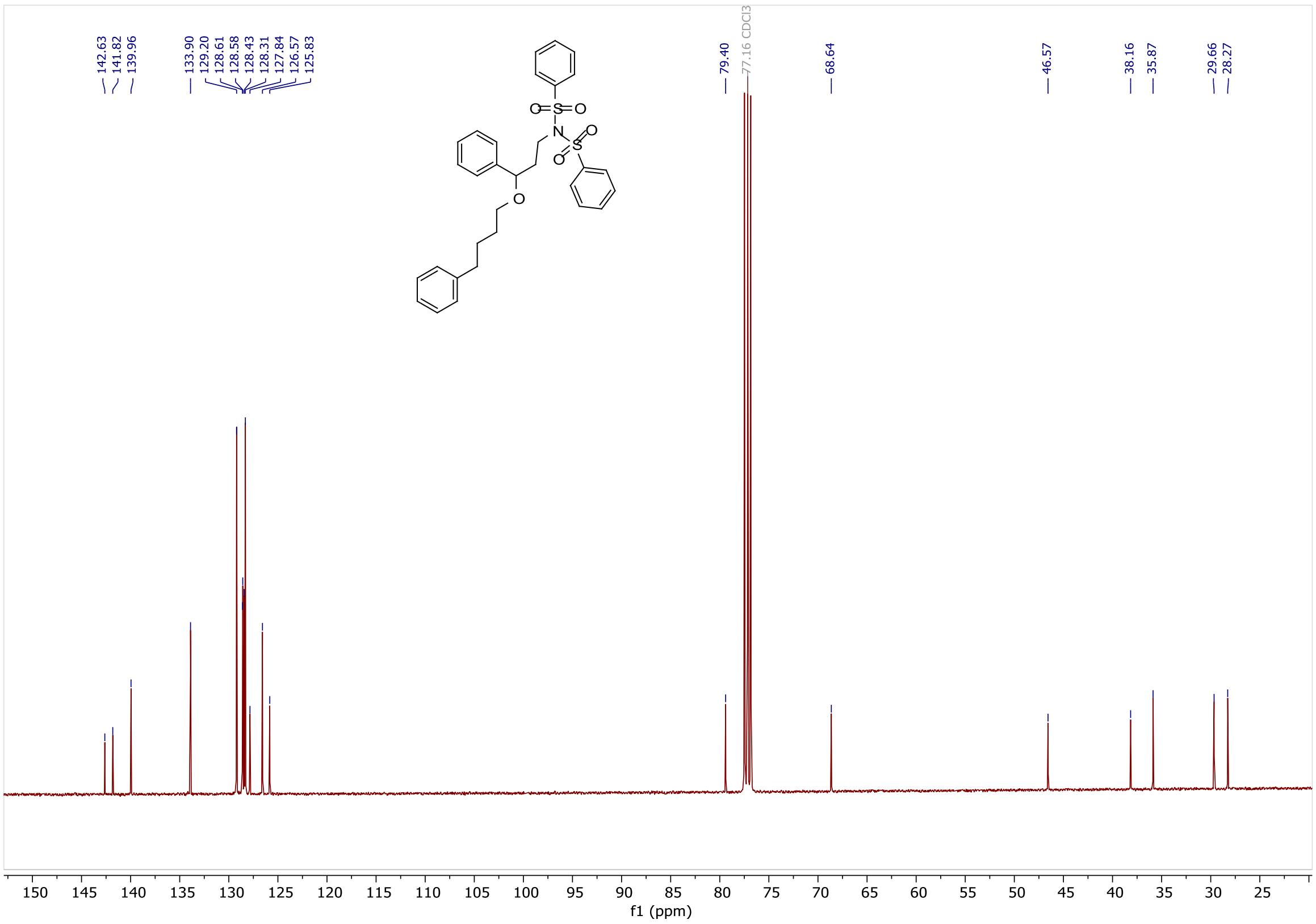

¹H NMR Spectrum of 8b (400 MHz, CDCl3)

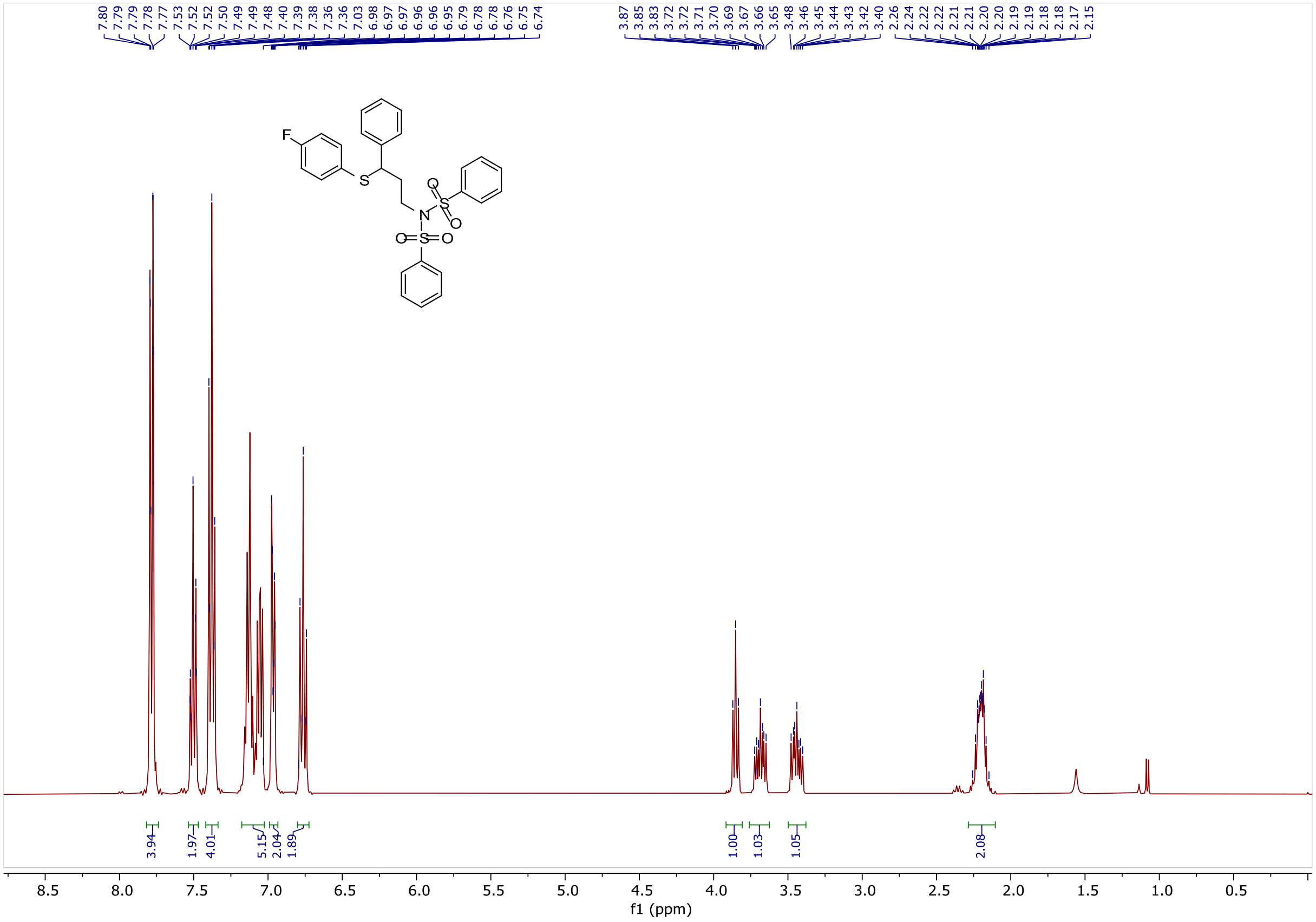

¹⁹F NMR Spectrum of 8b (376 MHz, CDCl3)

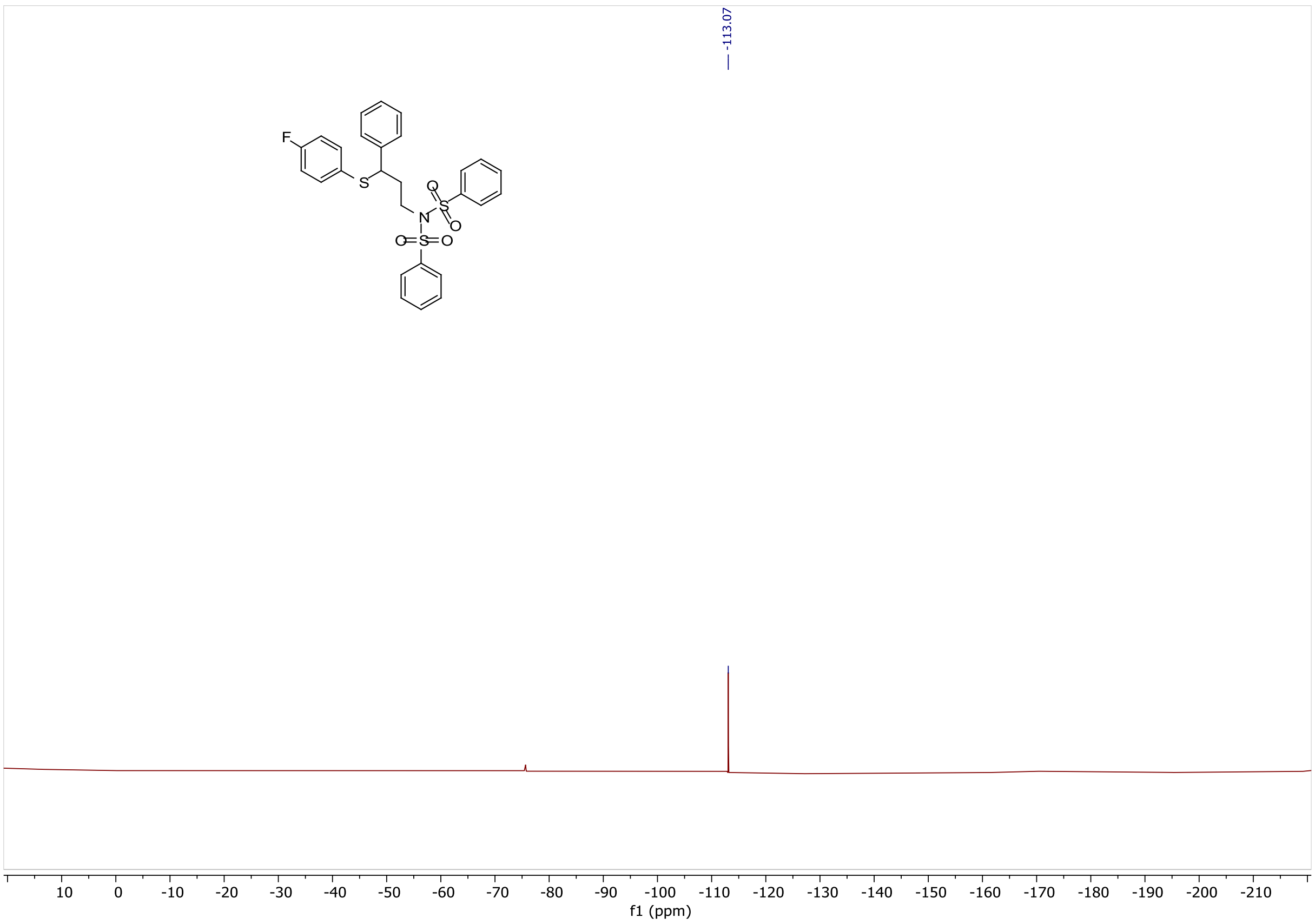

^{13}C NMR Spectrum of 8b (101 MHz, $CDCl_3$)

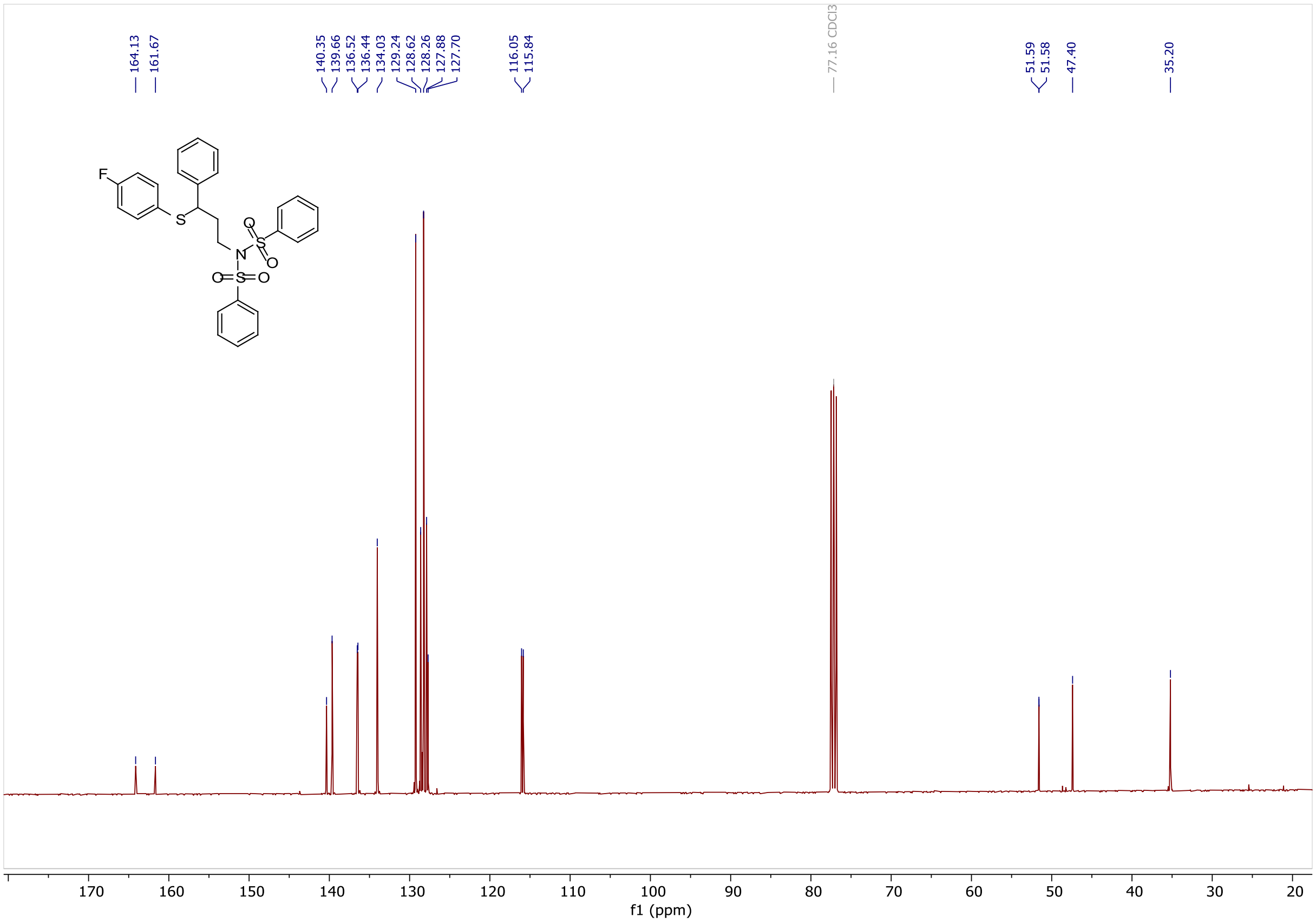

^{1}H NMR Spectrum of 10 (400 MHz, $CDCl_3$)

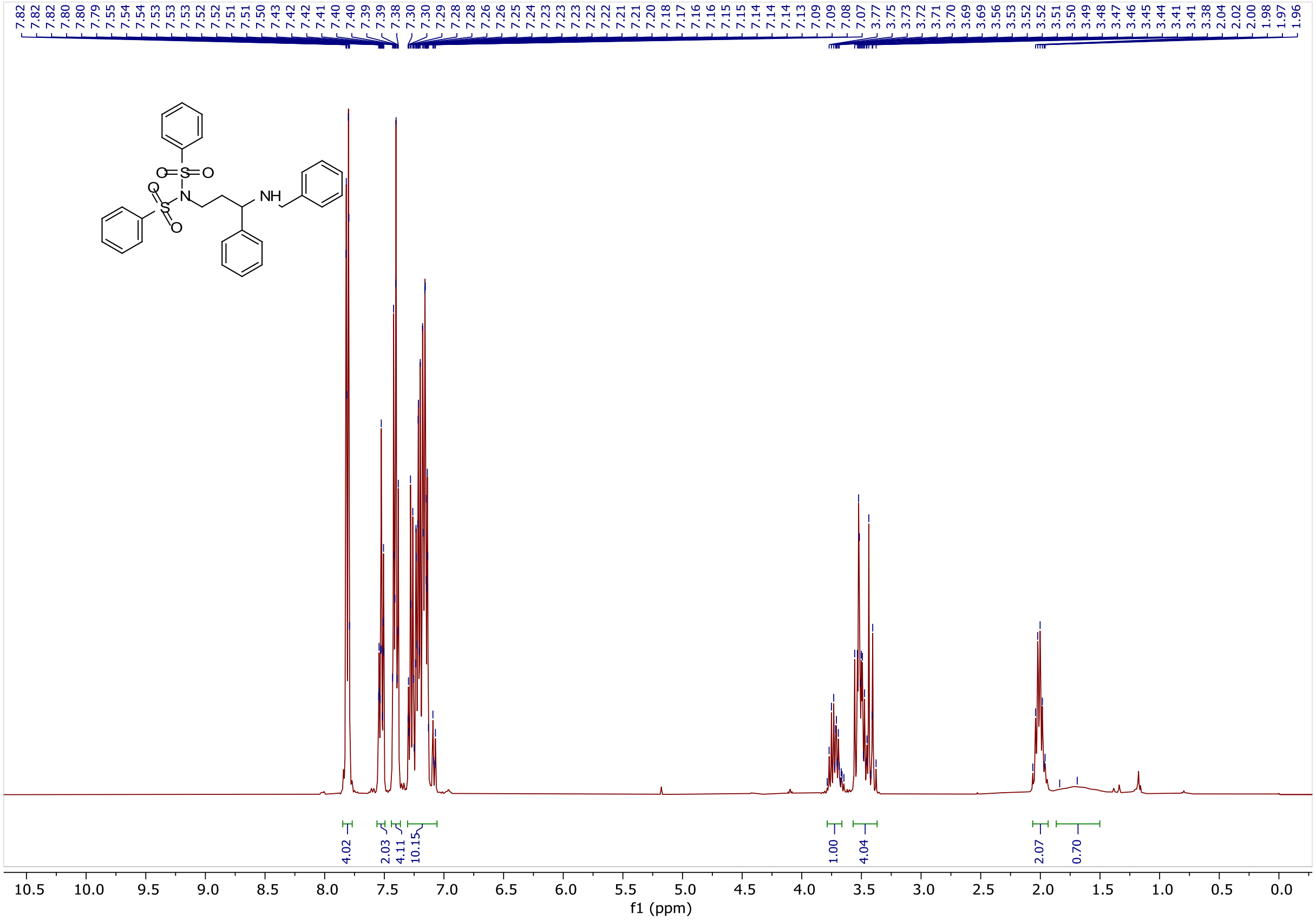

^{13}C NMR Spectrum of 10 (101 MHz, $CDCl_3$)

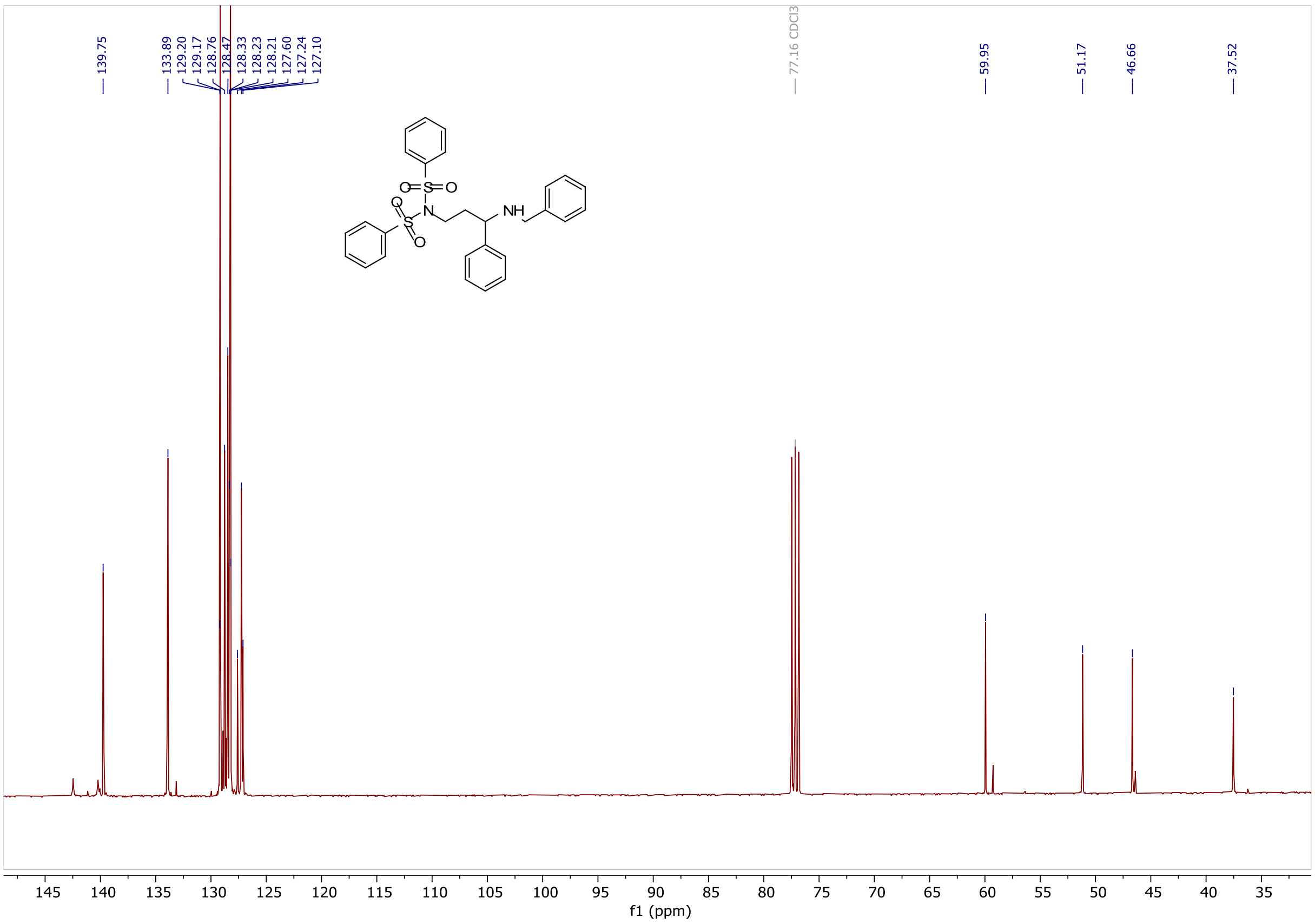

^{1}H NMR Spectrum of 12 (400 MHz, $CDCl_3$)

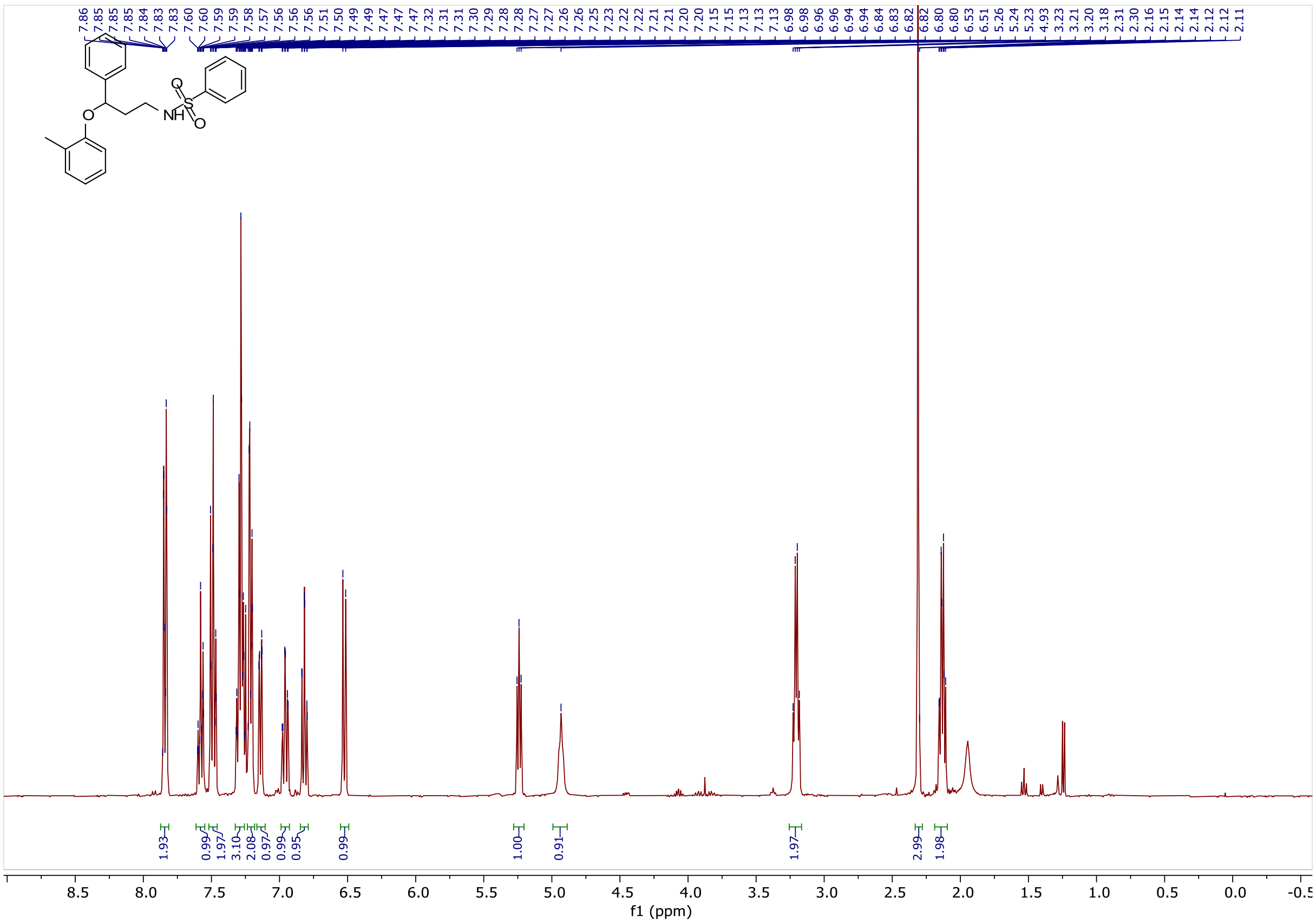

^{13}C NMR Spectrum of 12 (101 MHz, $CDCl_3$)

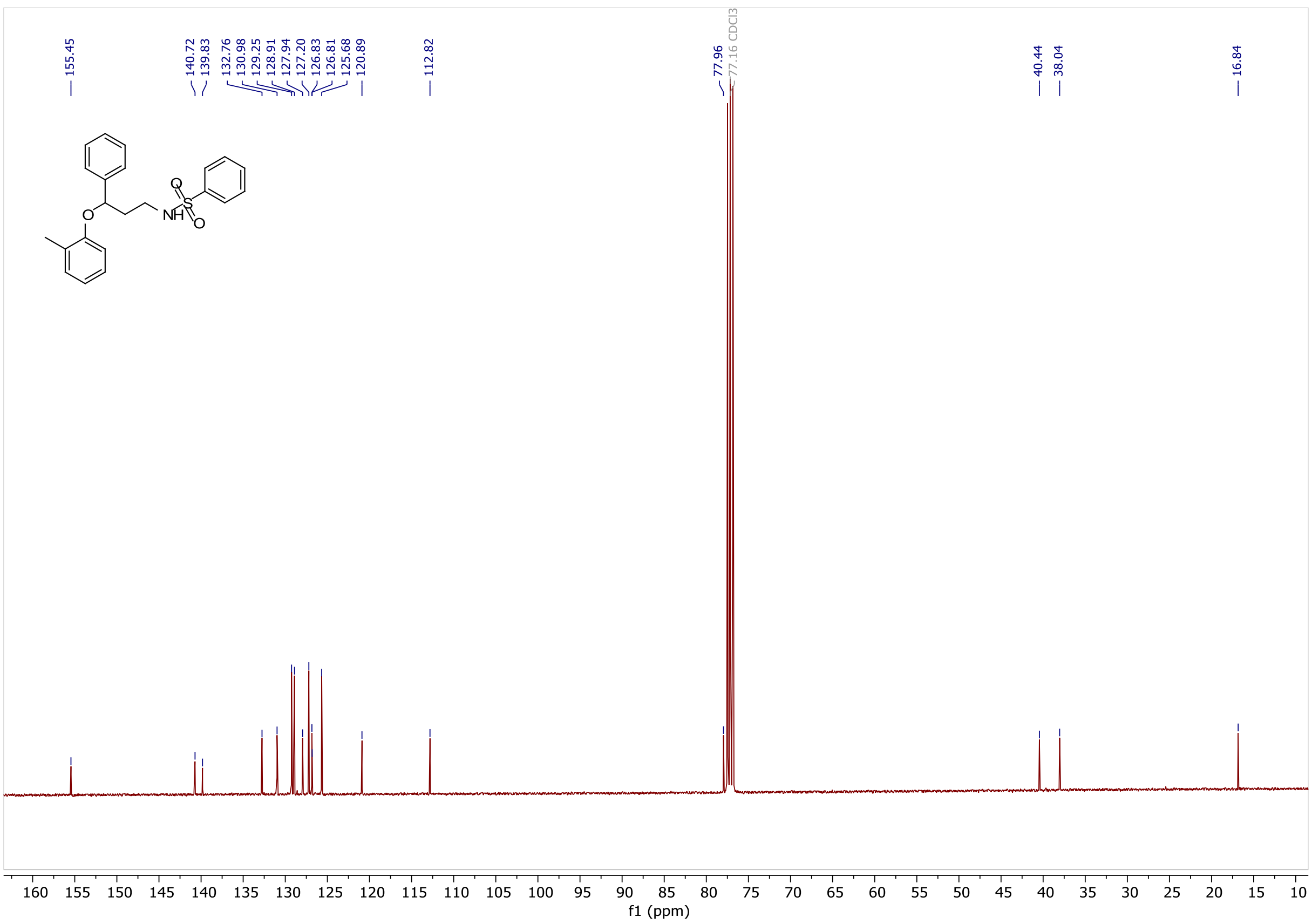

^{1}H NMR Spectrum of 15a (400 MHz, $CDCl_3$)

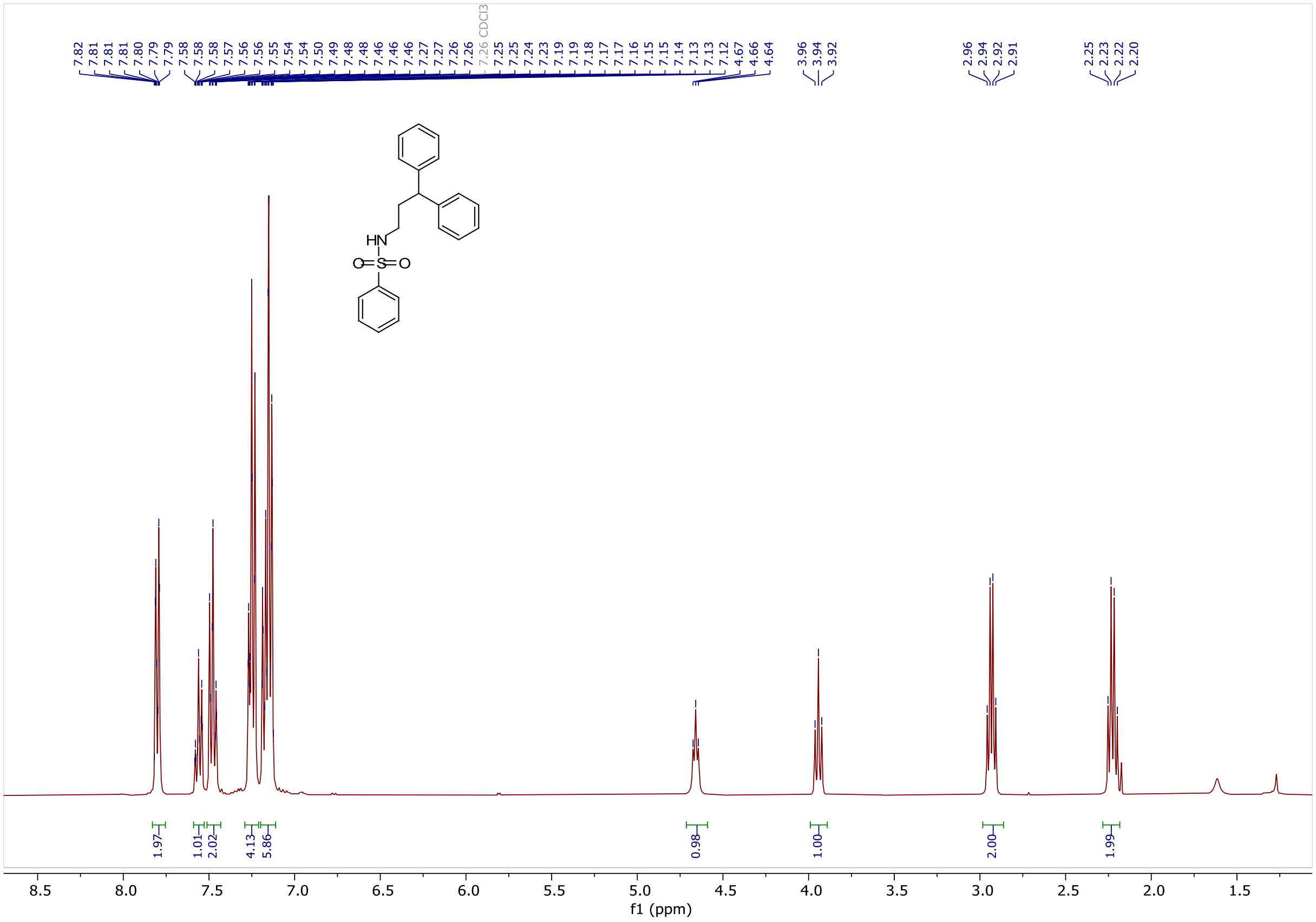

^{13}C NMR Spectrum of 15a (101 MHz, $CDCl_3$)

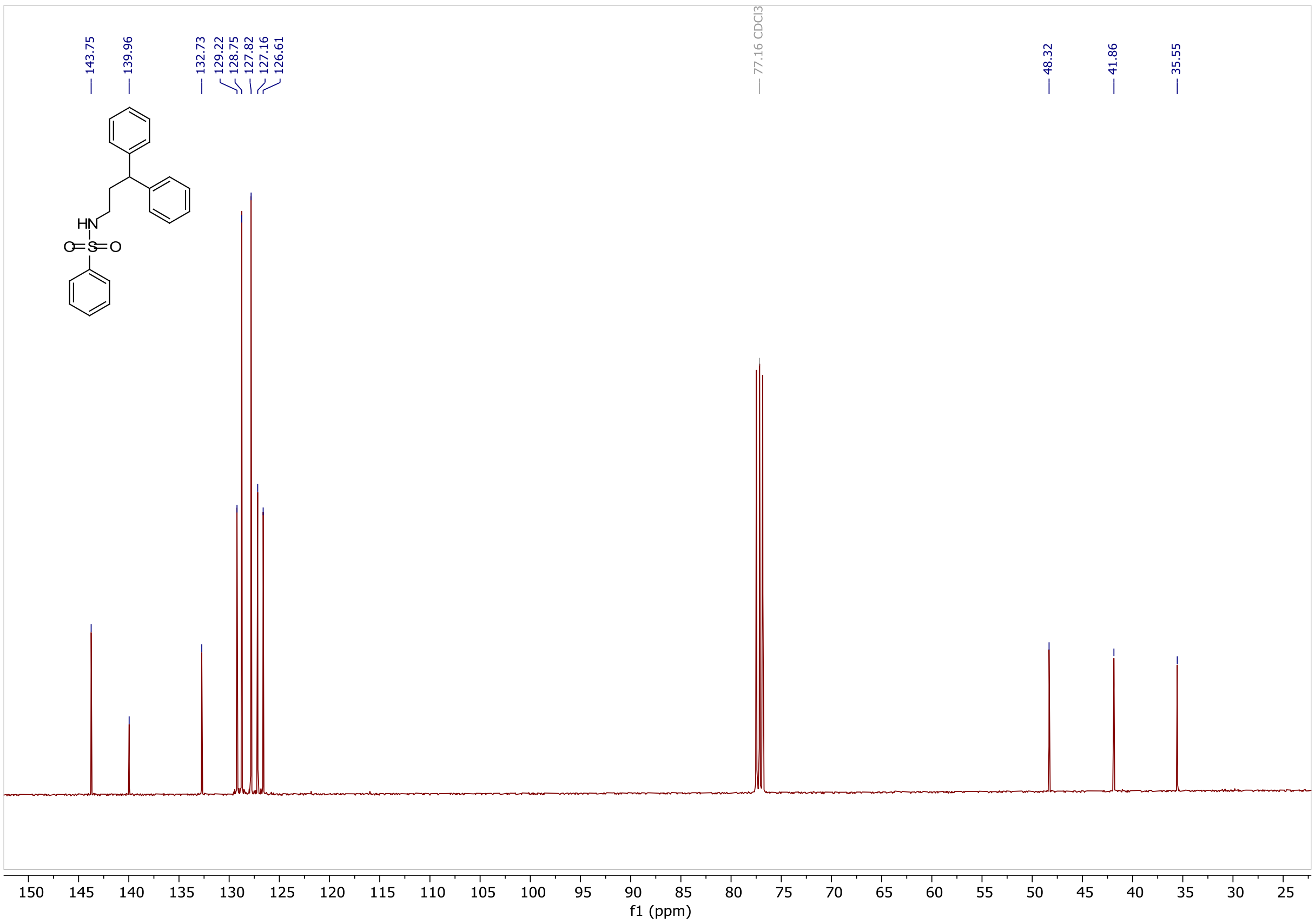

^{1}H NMR Spectrum of 15b (400 MHz, DMSO)

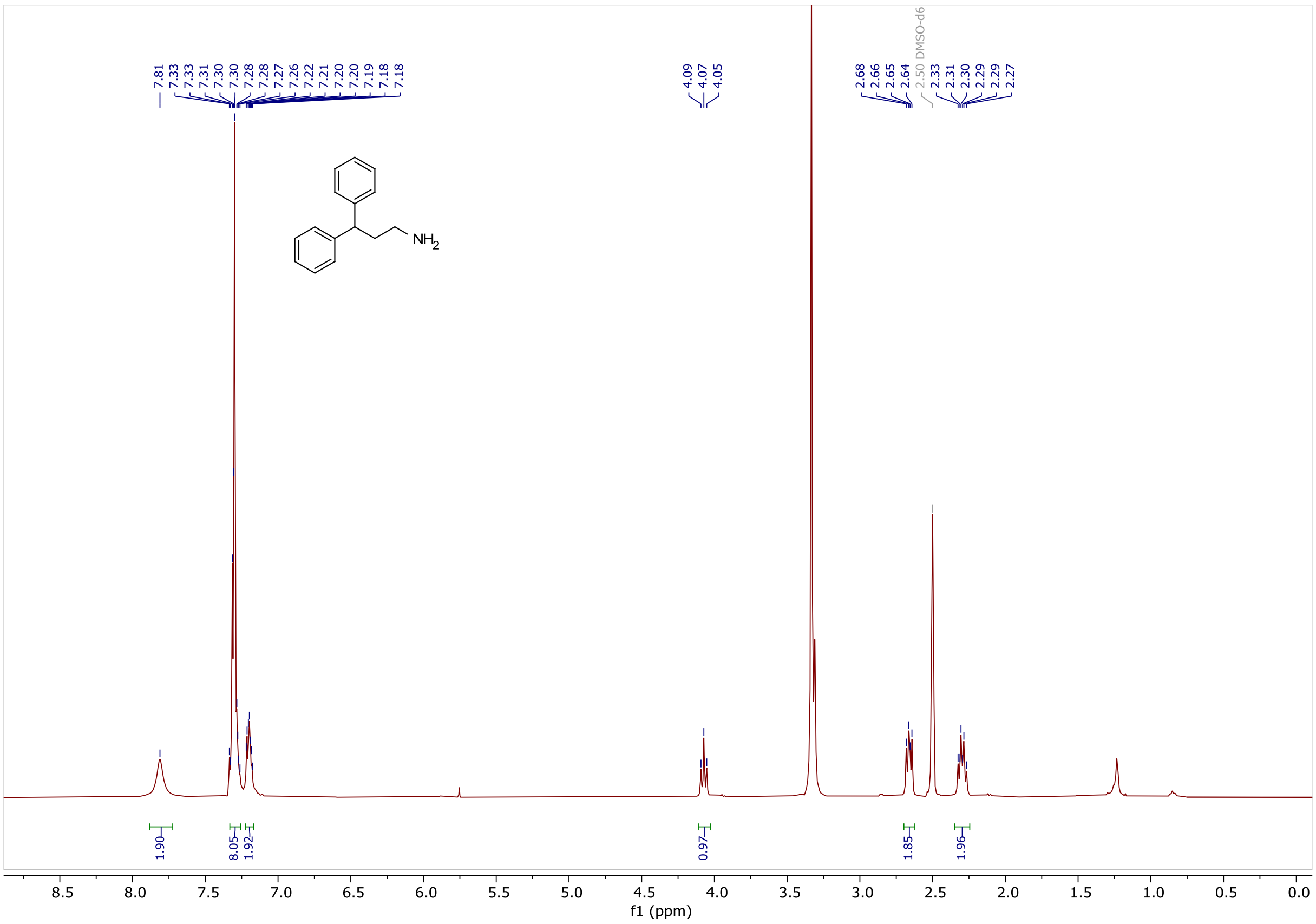

^{13}C NMR Spectrum of 15b (101 MHz, DMSO)

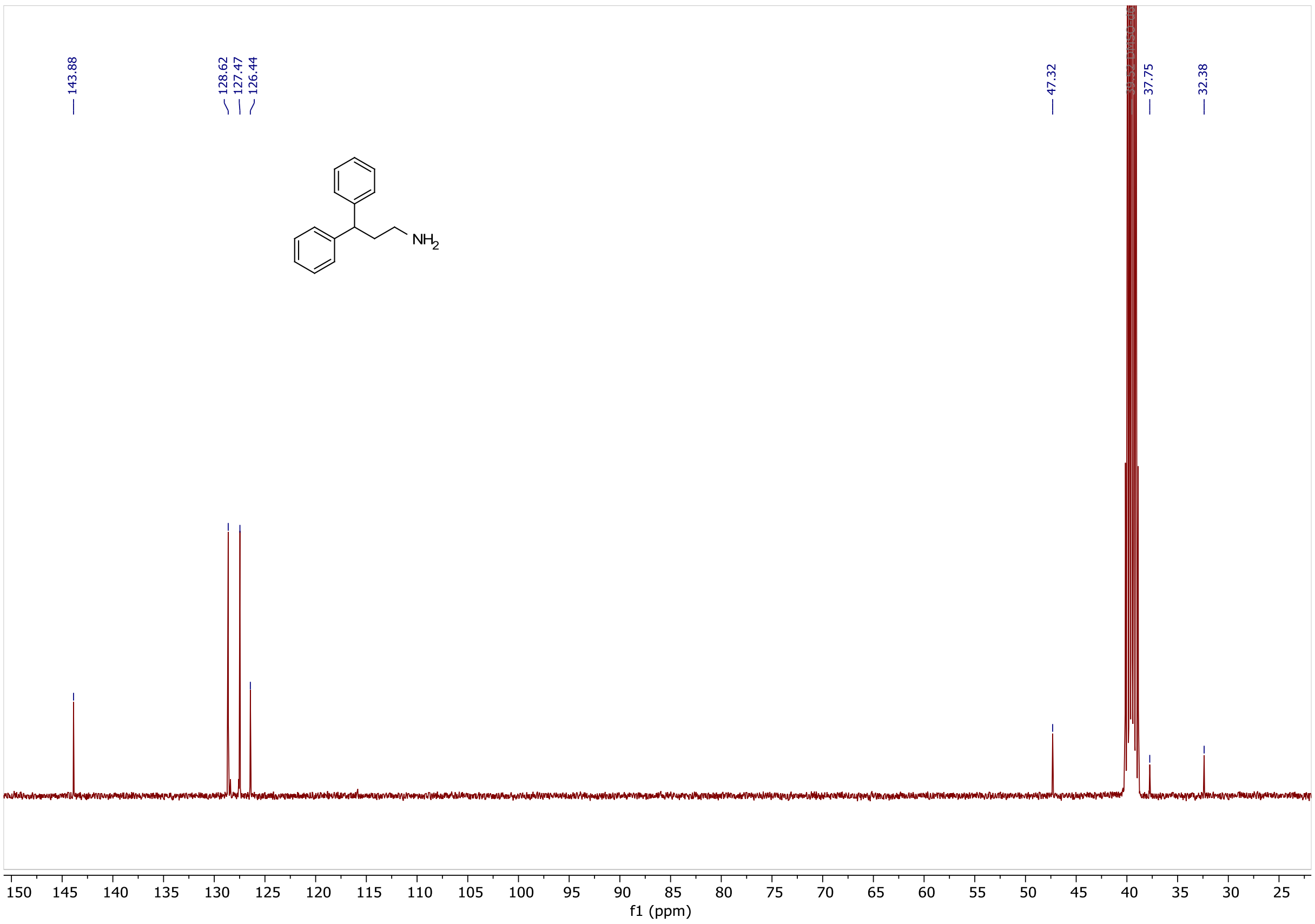

^{1}**H NMR Spectrum of 18** (400 MHz, CDCl$_3$)

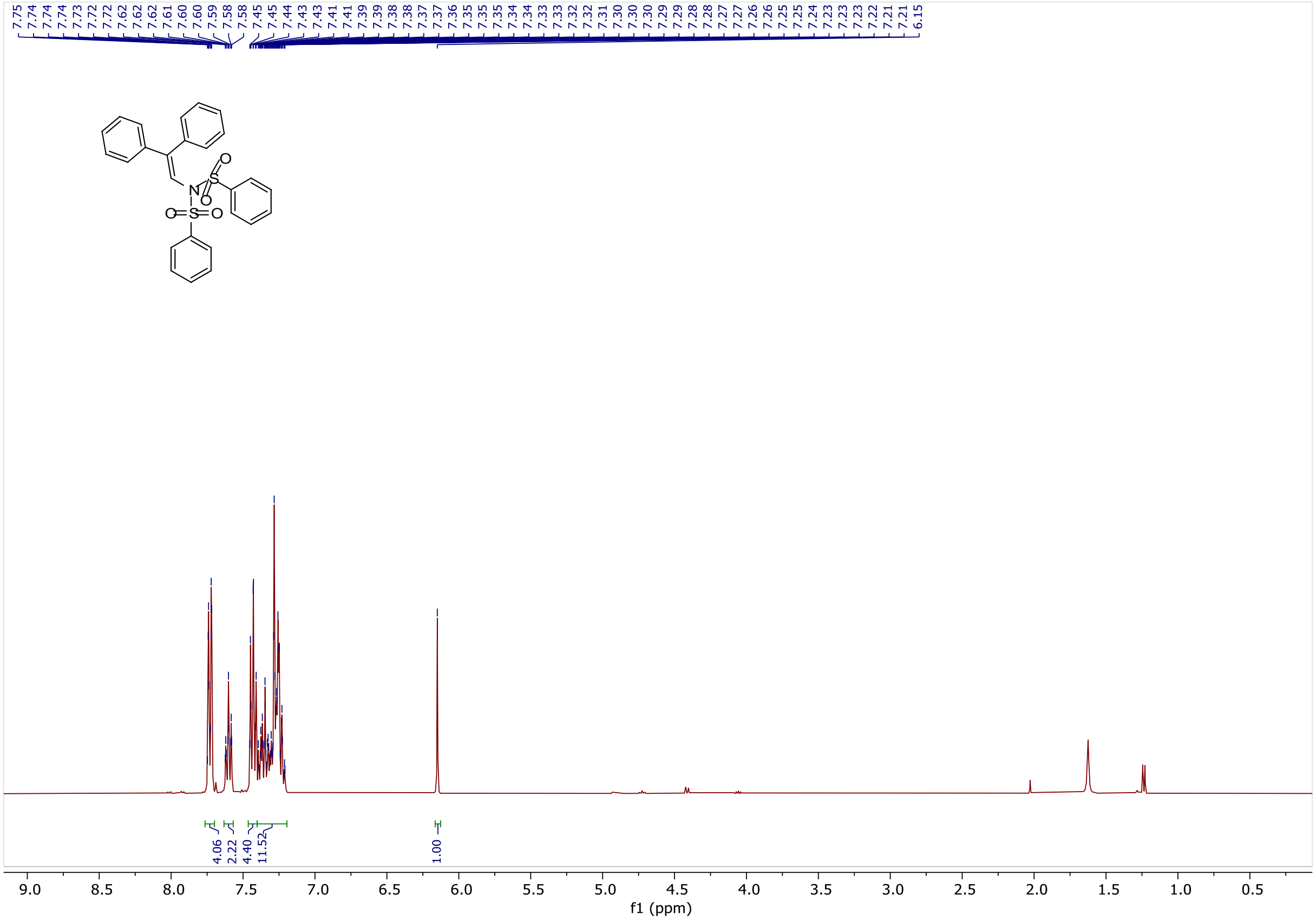

^{13}**C NMR Spectrum of 18** (101 MHz, CDCl$_3$)

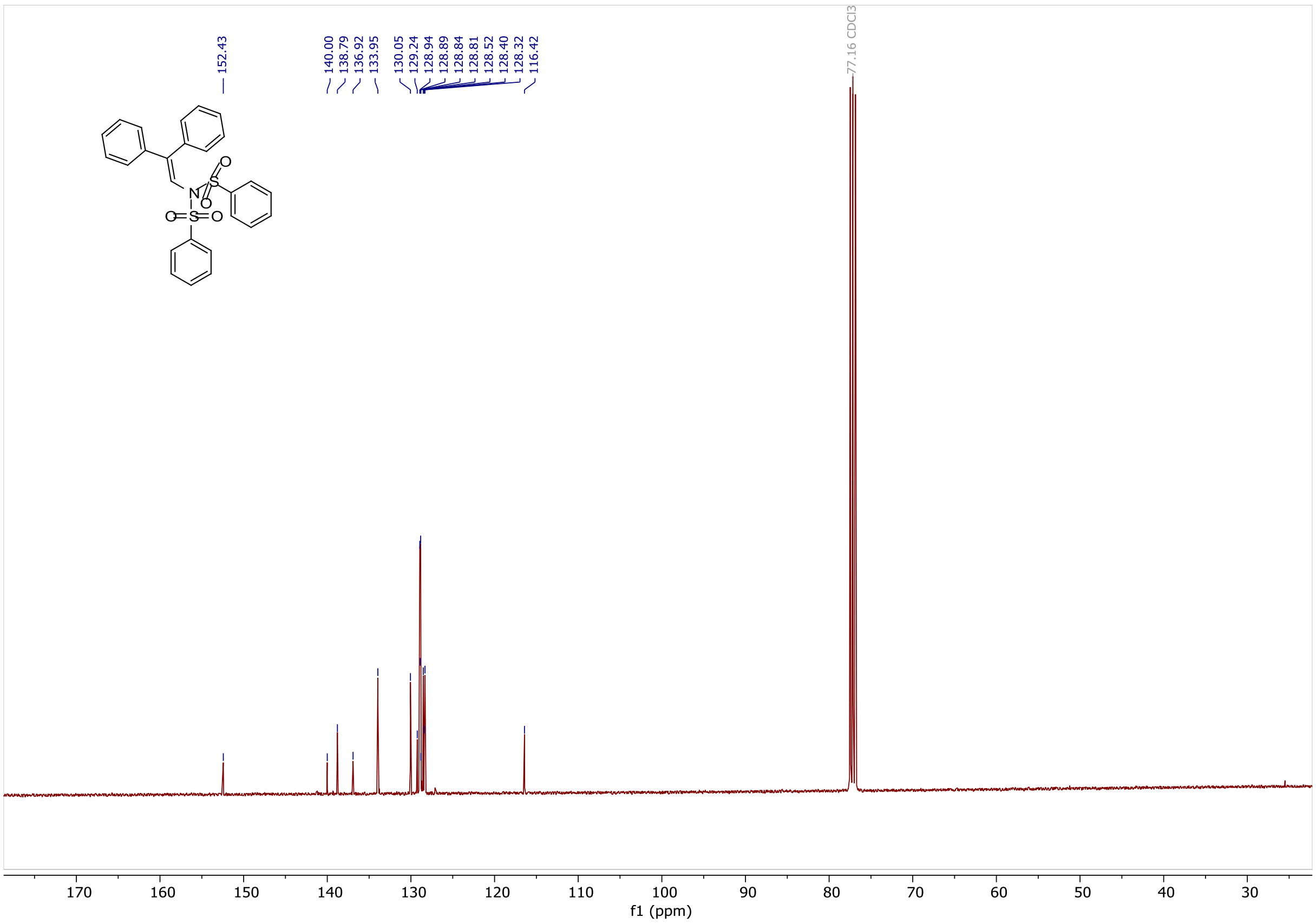

^{1}H NMR Spectrum of 19 (400 MHz, $CDCl_3$)

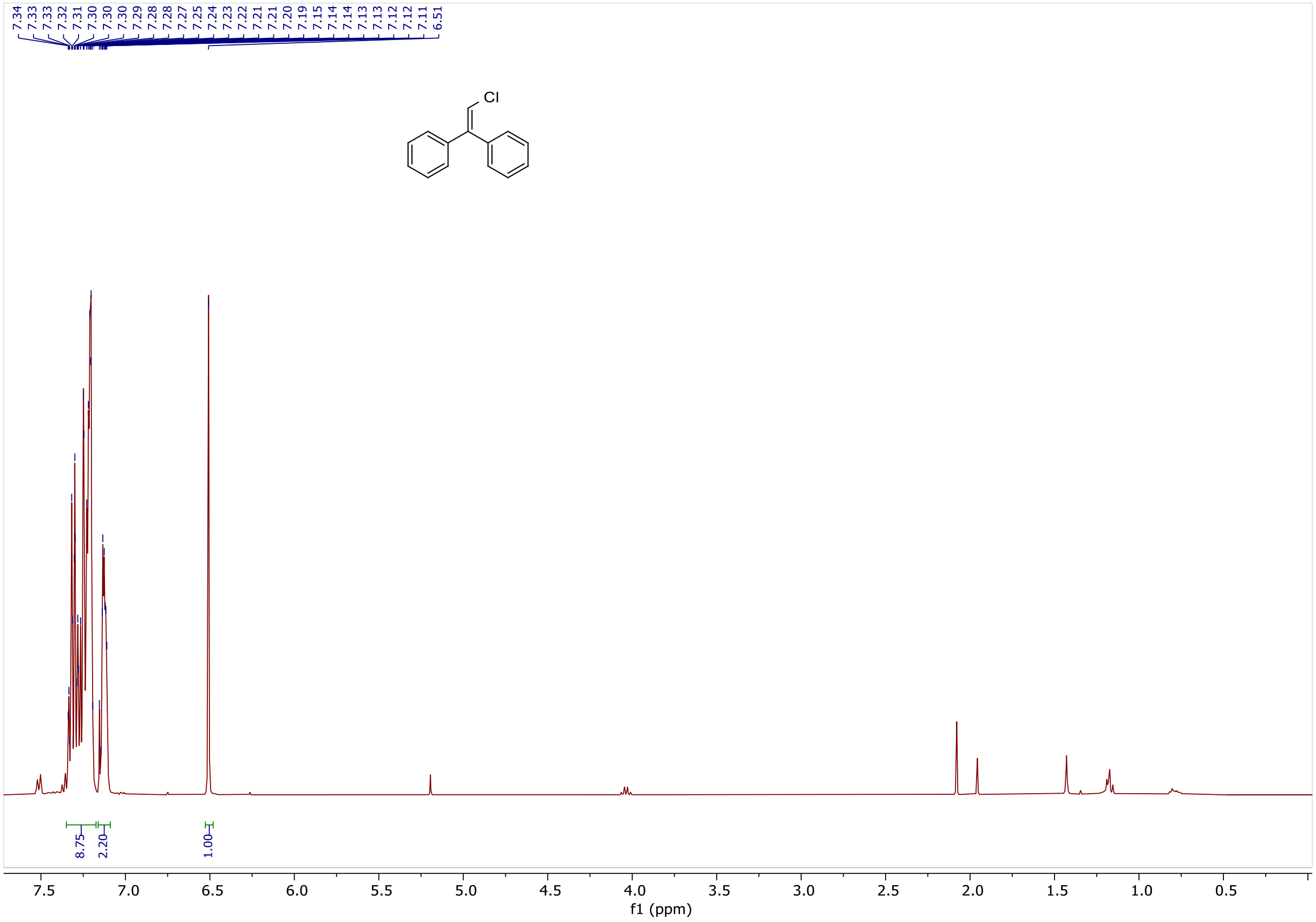

^{13}C NMR Spectrum of 19 (101 MHz, $CDCl_3$)

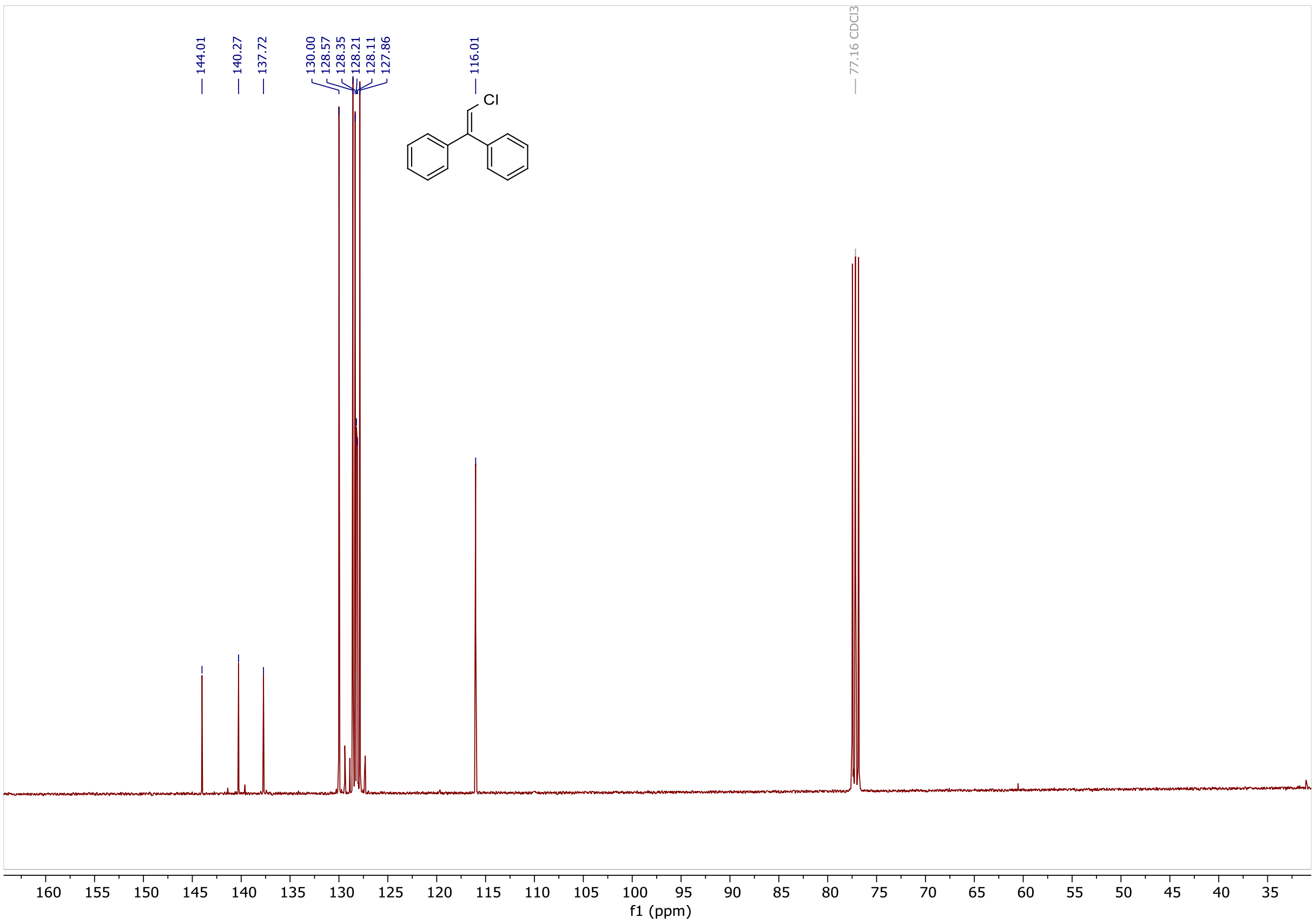

^{1}H NMR Spectrum of 20 (400 MHz, $CDCl_3$)

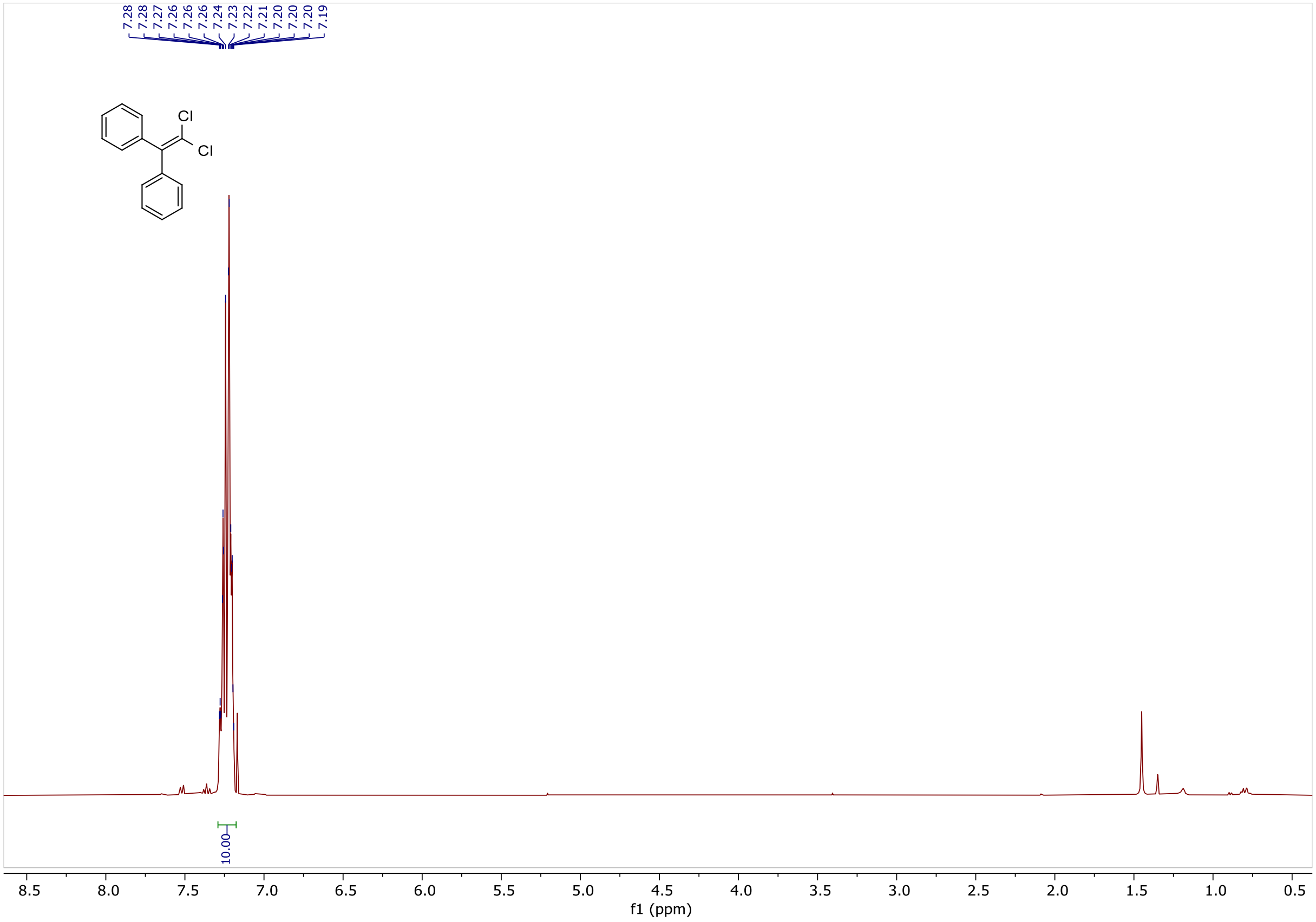

^{13}C NMR Spectrum of 20 (101 MHz, $CDCl_3$)

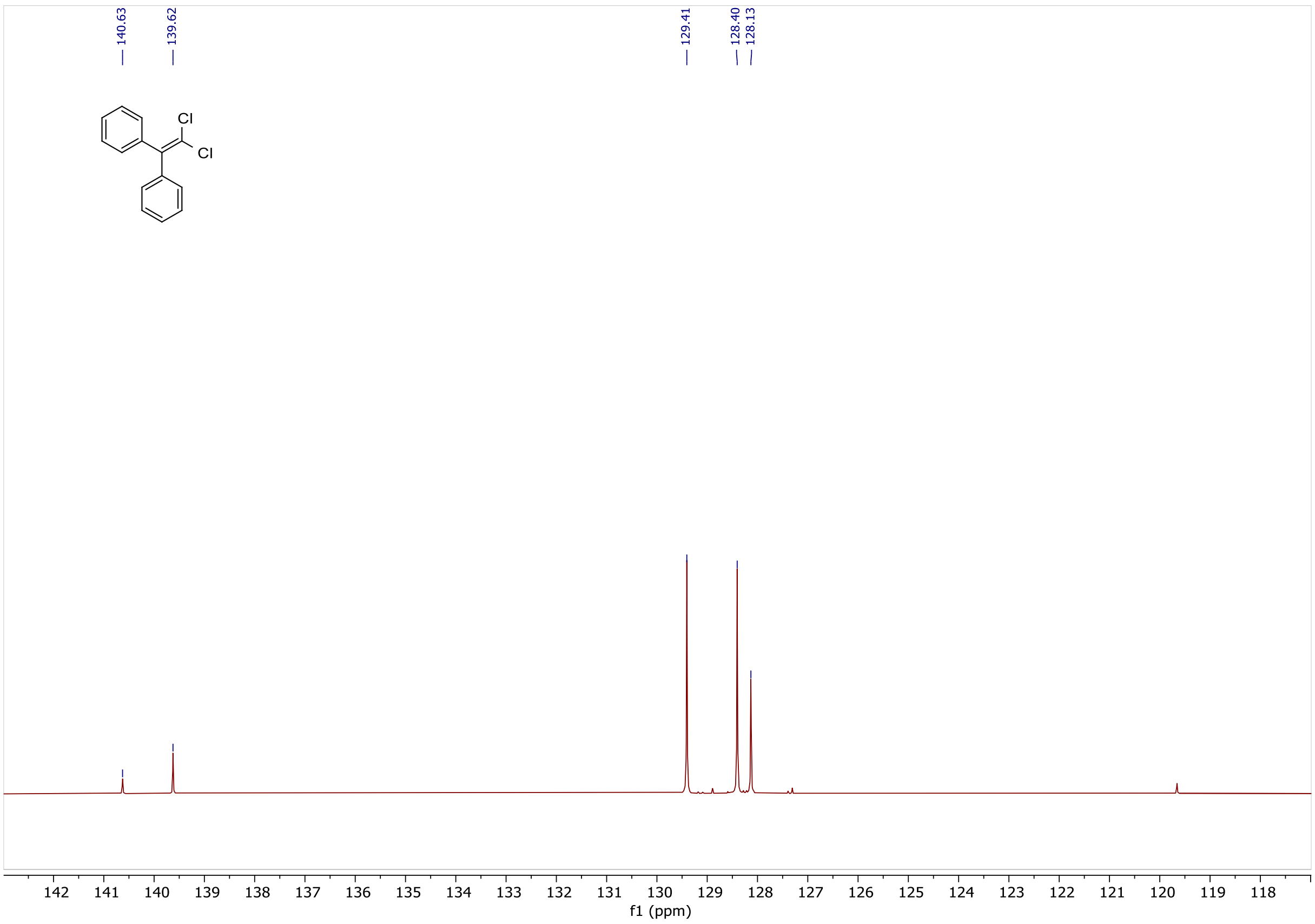

^{1}H NMR Spectrum of 21 (400 MHz, $CDCl_3$)

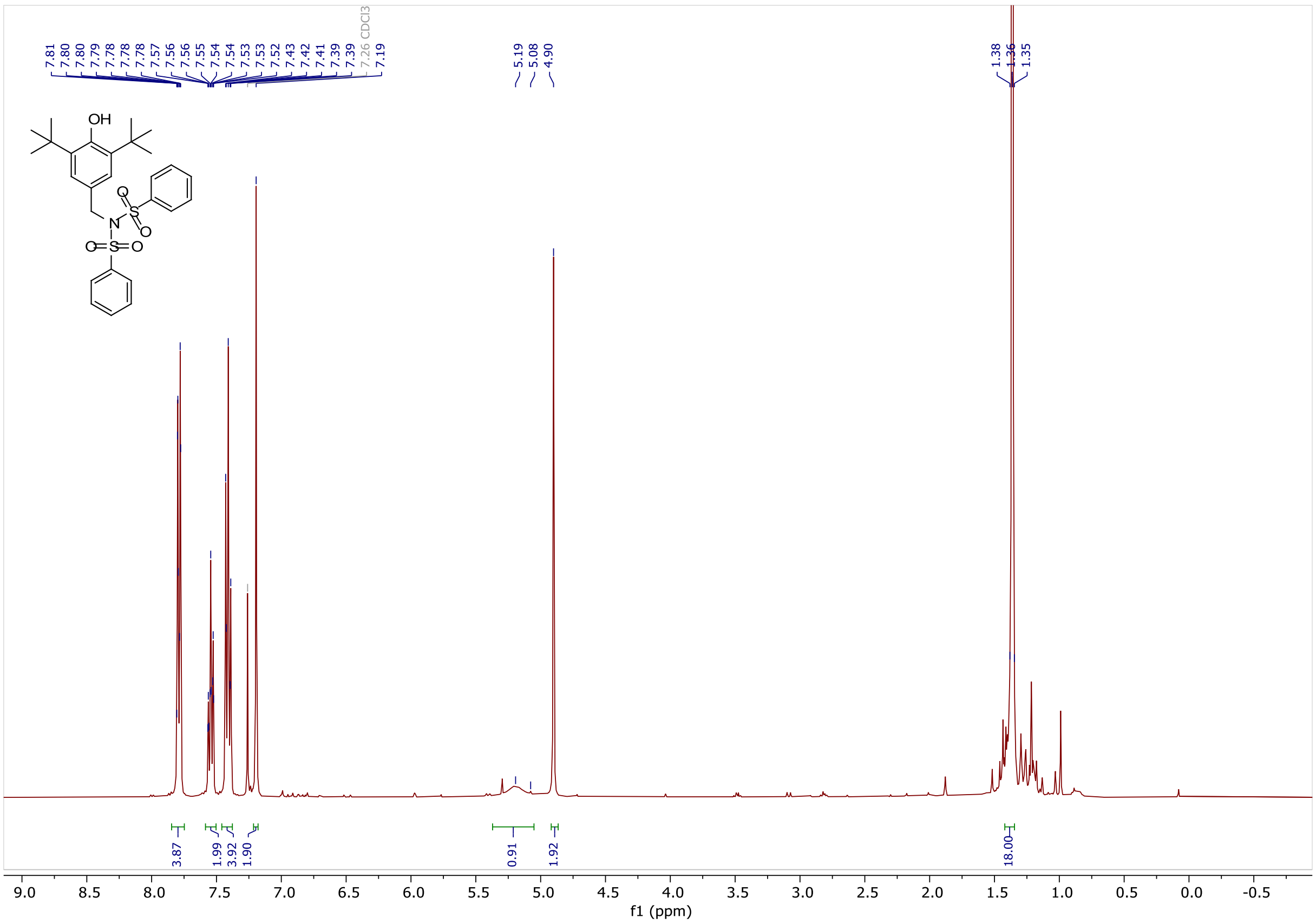

^{13}C NMR Spectrum of 21 (101 MHz, $CDCl_3$)

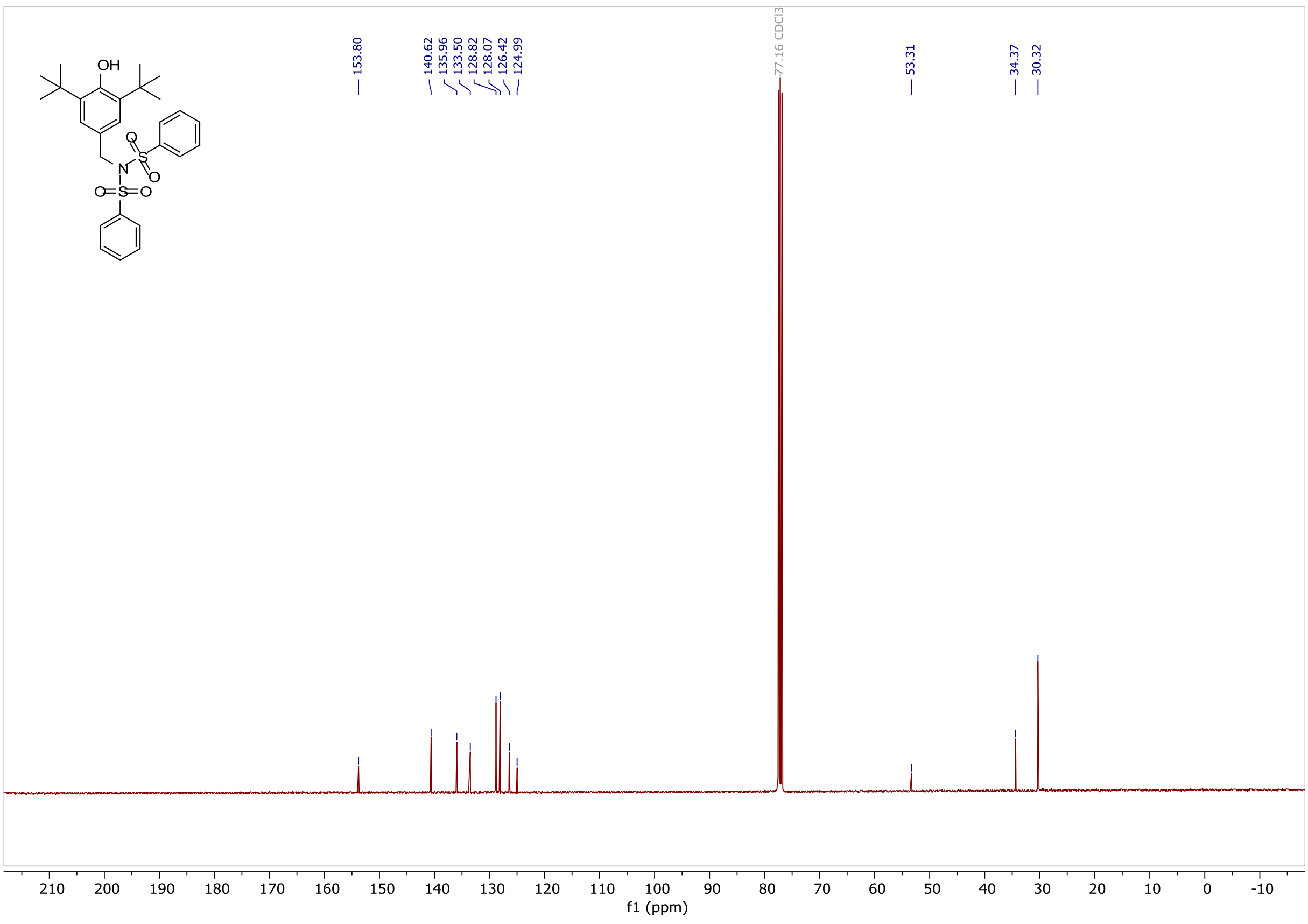

^{1}H NMR Spectrum of 22 (400 MHz, $CDCl_3$)

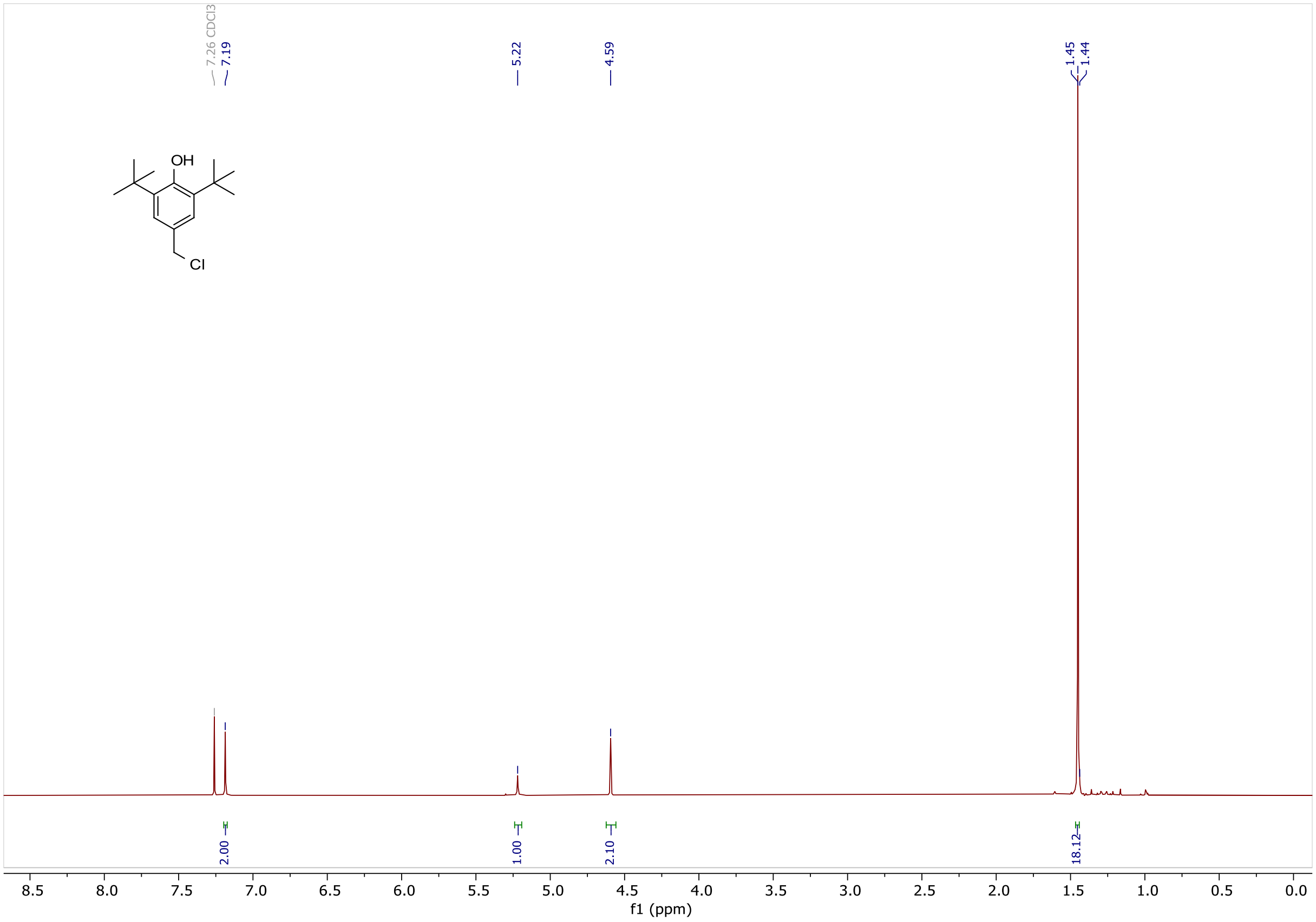

^{13}C NMR Spectrum of 22 (101 MHz, $CDCl_3$)

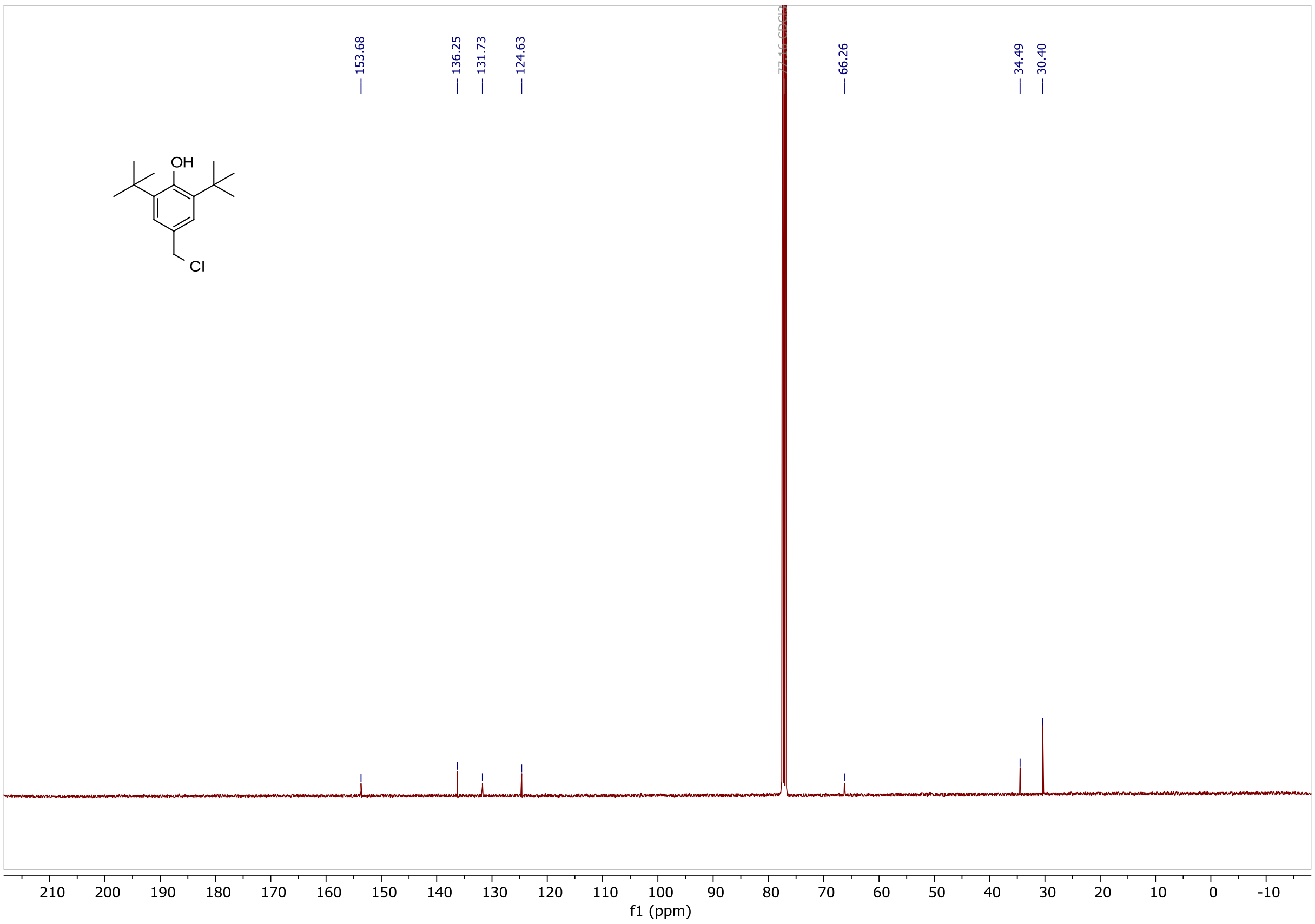

^{1}H NMR Spectrum of 23 (400 MHz, CDCl3)

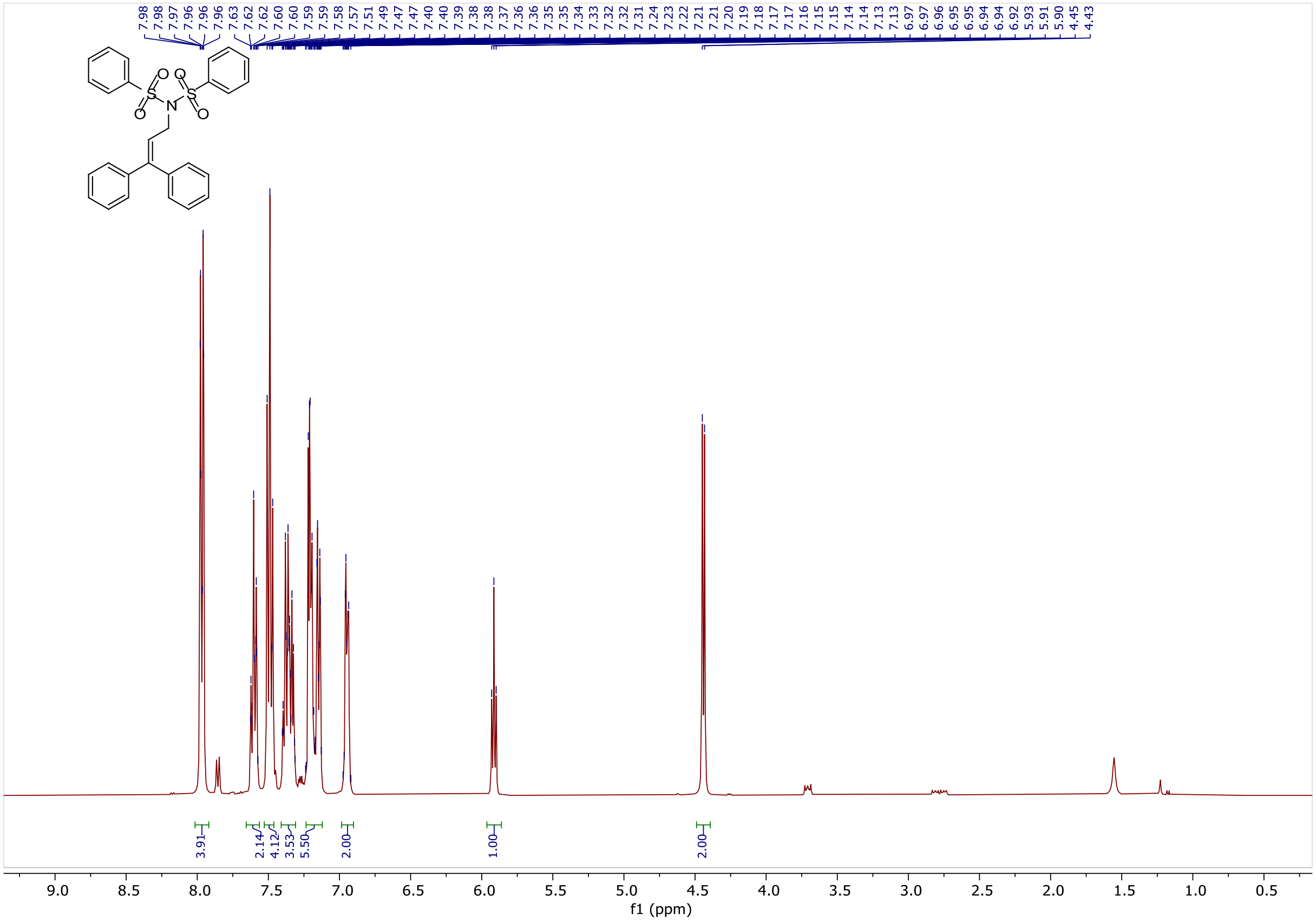

^{13}C NMR Spectrum of 23 (101 MHz, CDCl3)

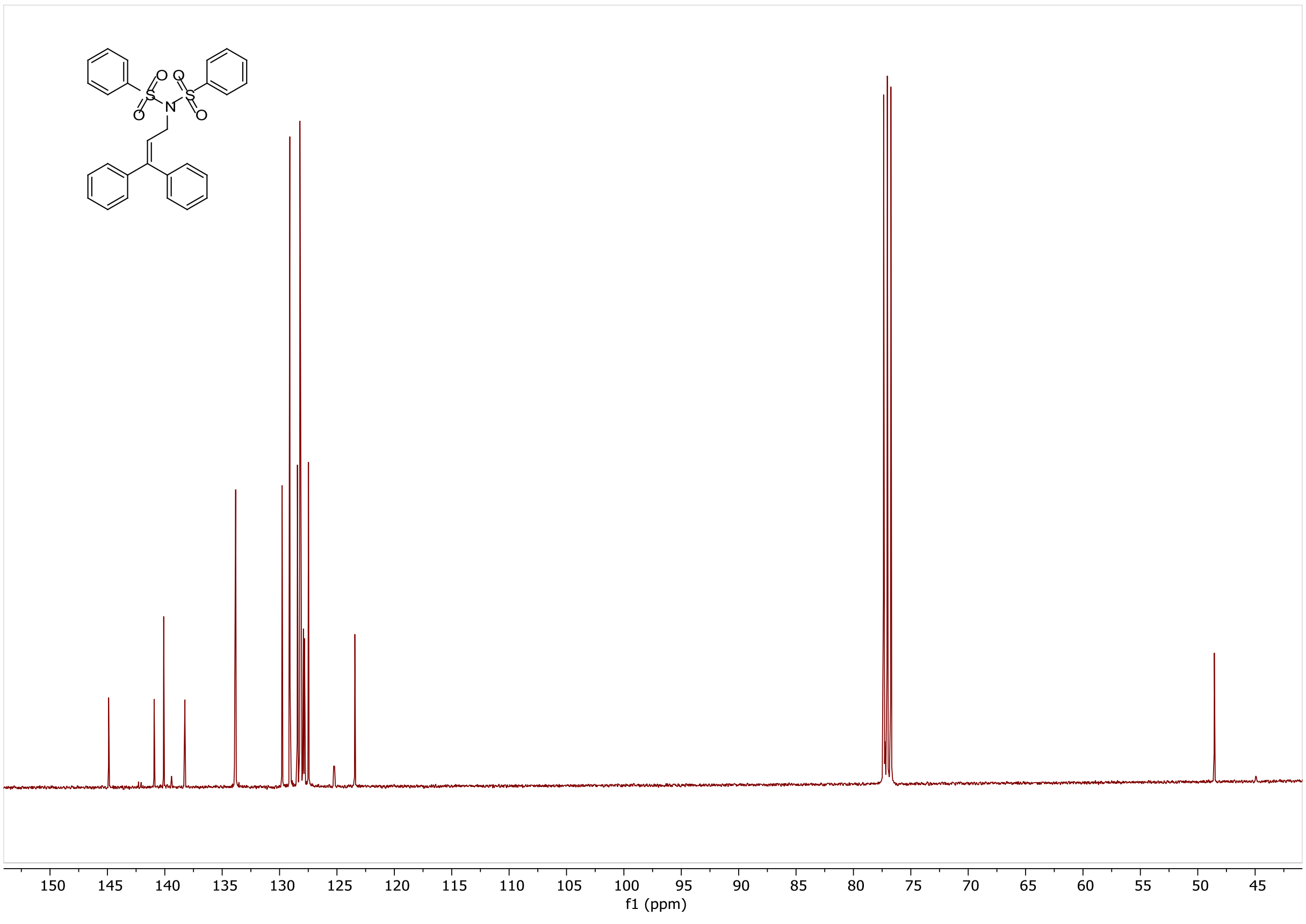

^{1}H NMR Spectrum of 25 (400 MHz, $CDCl_3$)

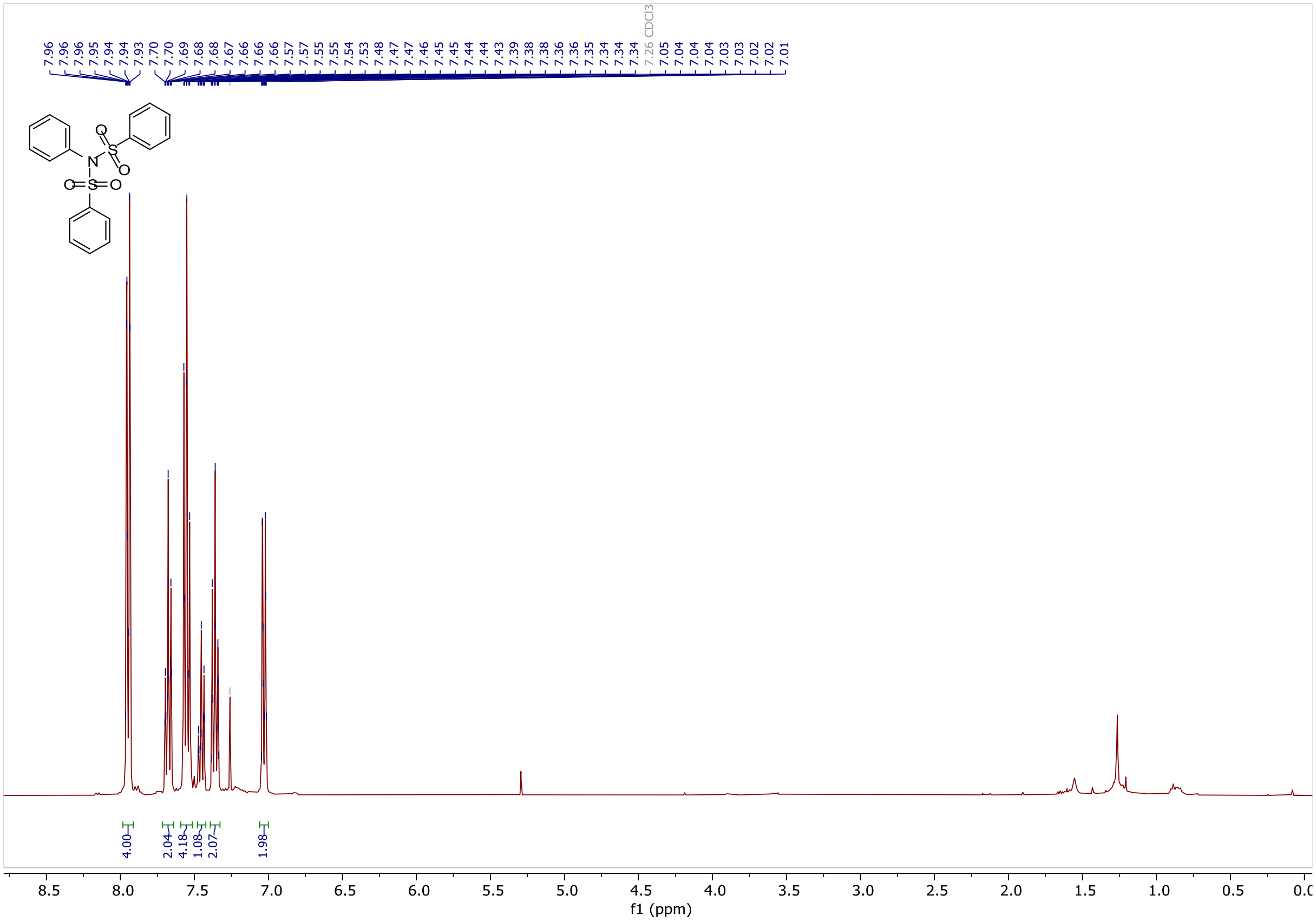

^{13}C NMR Spectrum of 25 (101 MHz, $CDCl_3$)

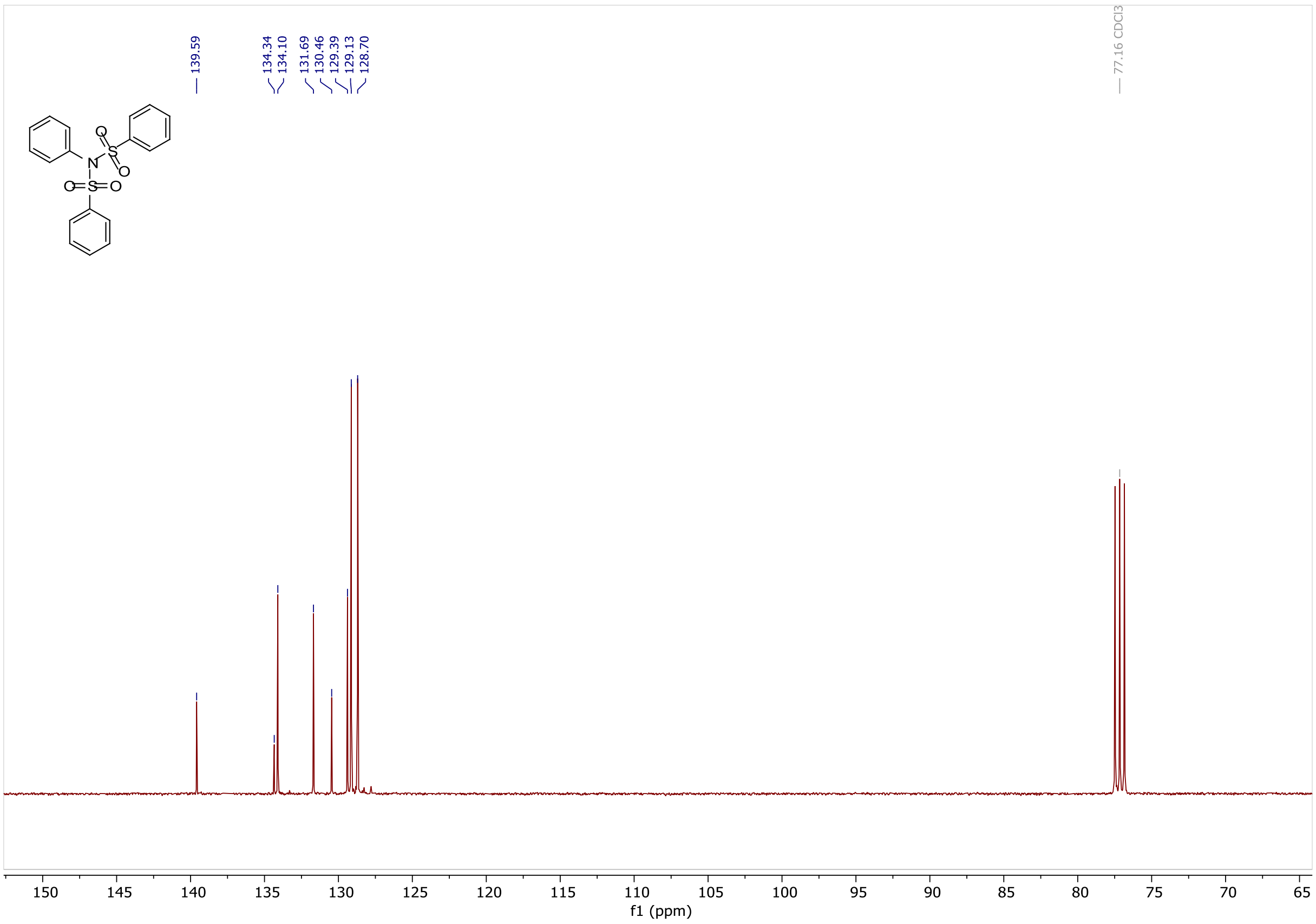

^{1}H NMR Spectrum of 26 (400 MHz, $CDCl_3$)

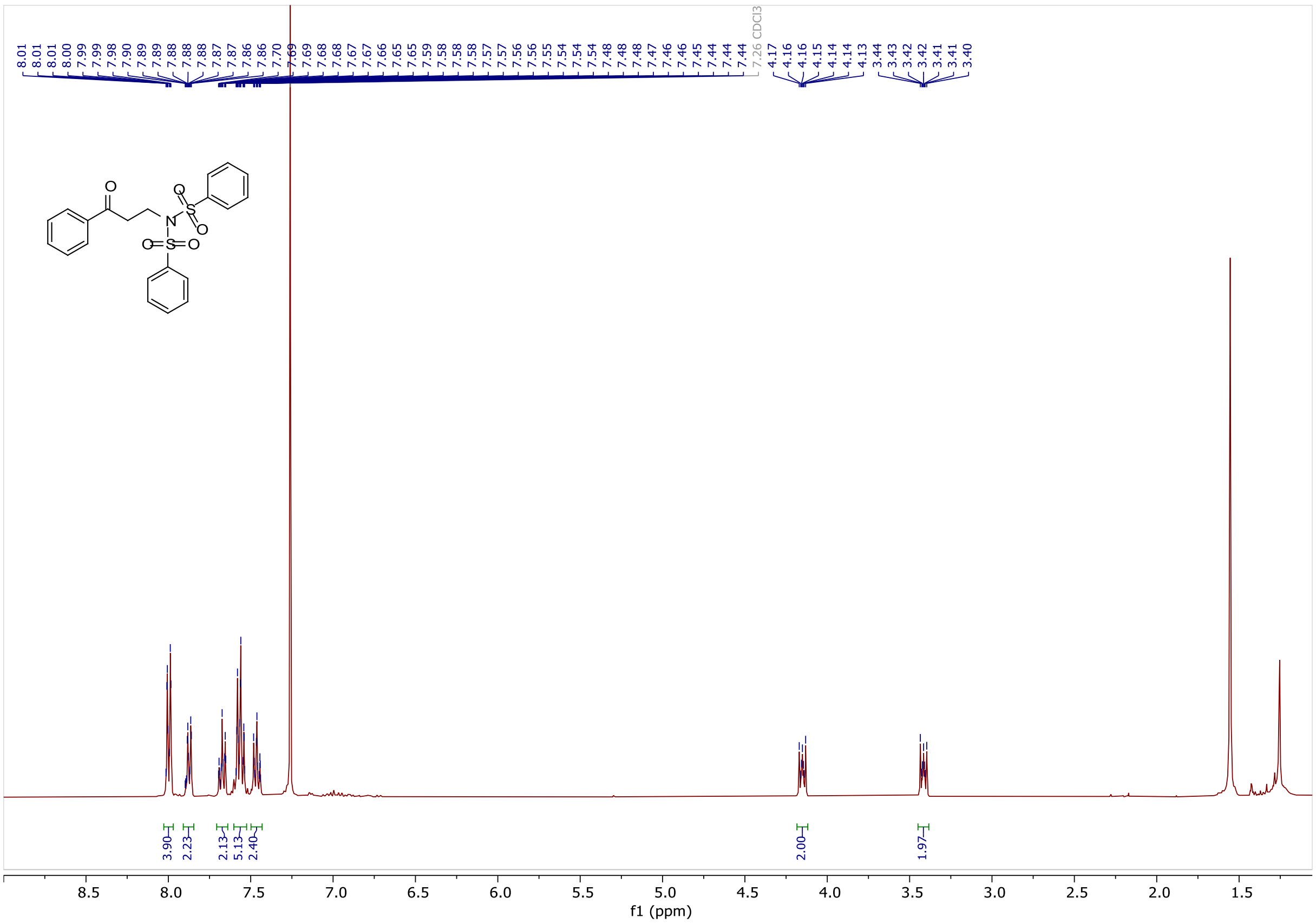

^{13}C NMR Spectrum of 26 (101 MHz, $CDCl_3$)

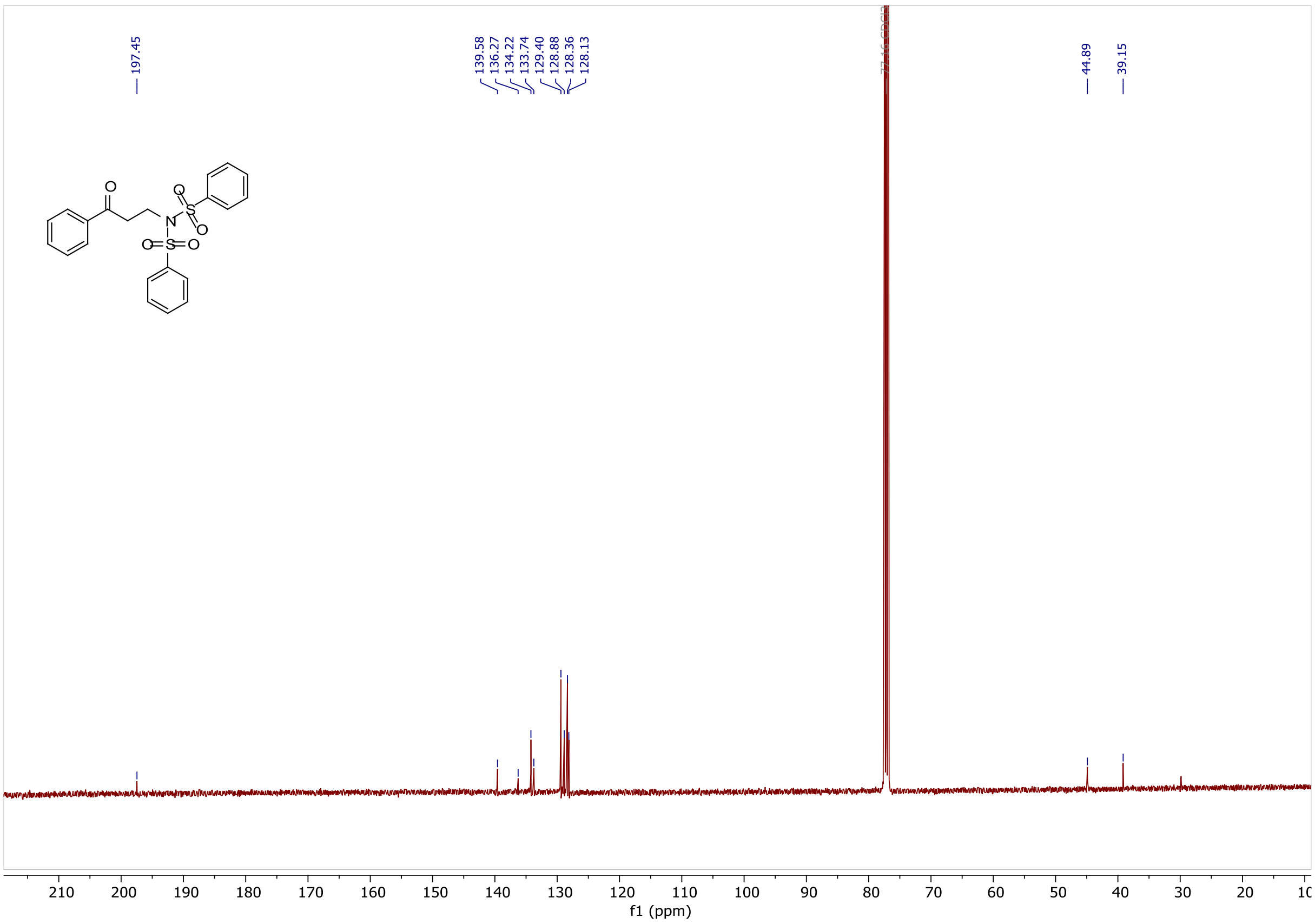

^{1}H NMR Spectrum of 27 (400 MHz, $CDCl_3$)

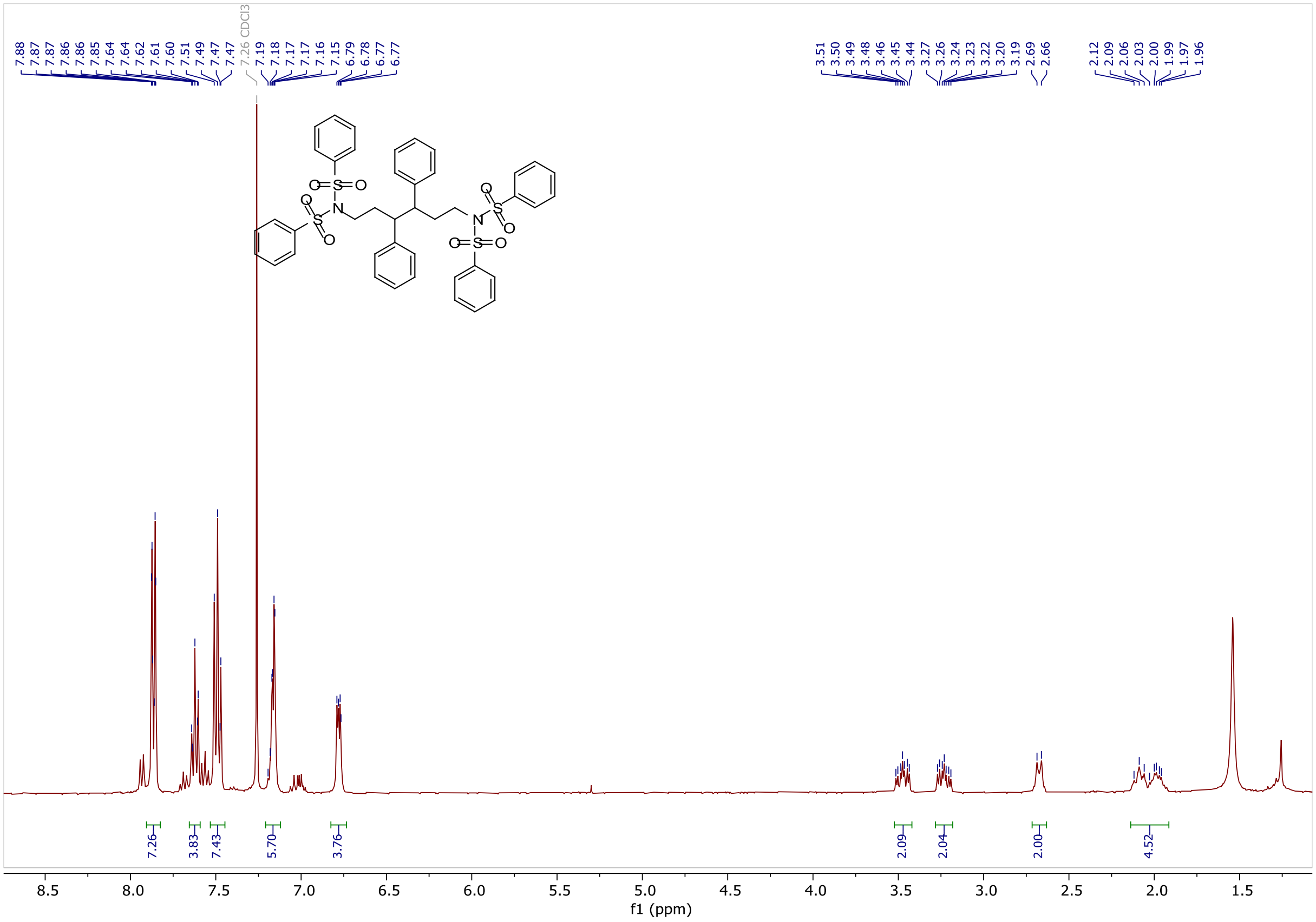

^{13}C NMR Spectrum of 27 (101 MHz, $CDCl_3$)

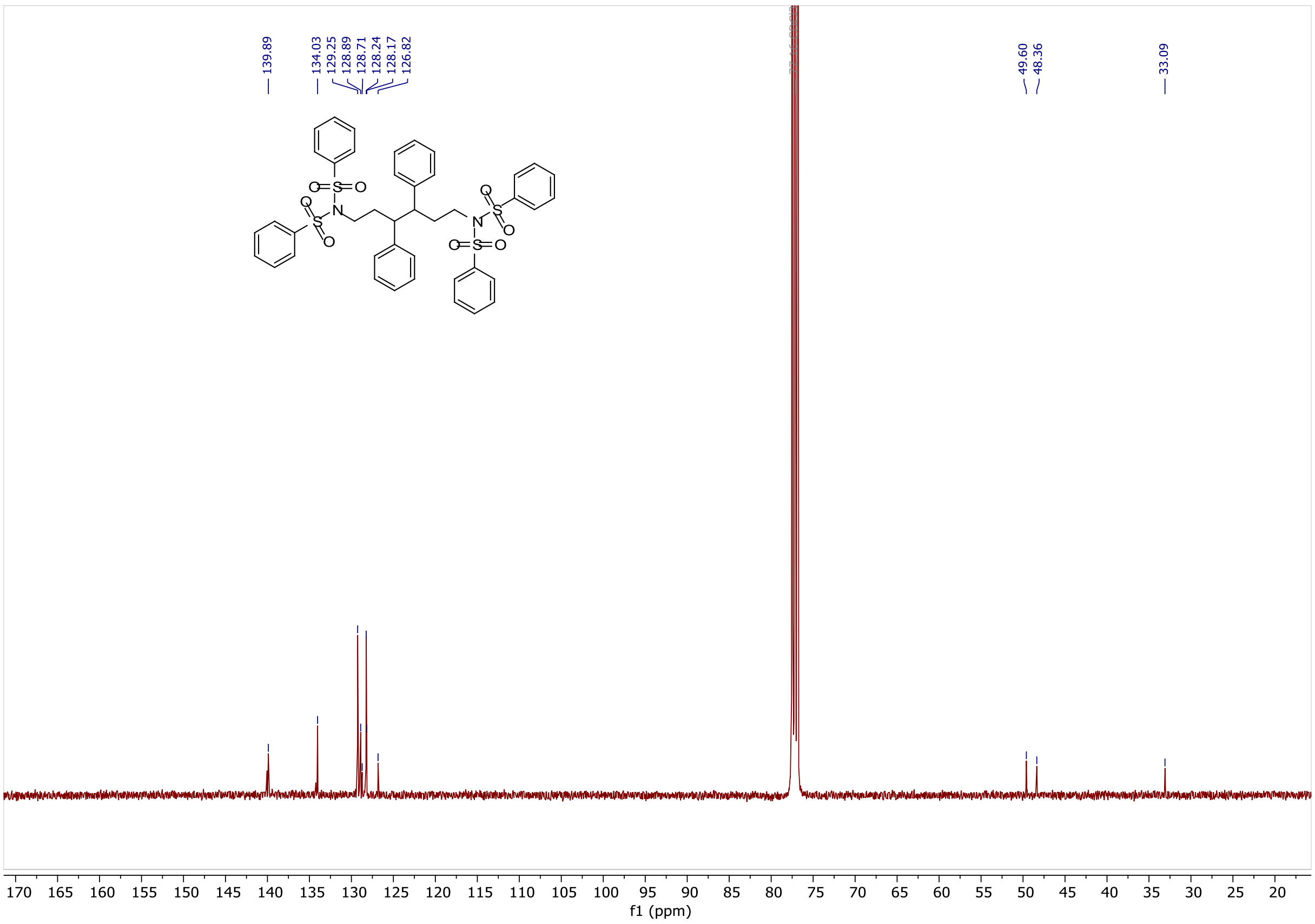

^{1}H NMR Spectrum of 28 (400 MHz, $CDCl_3$)

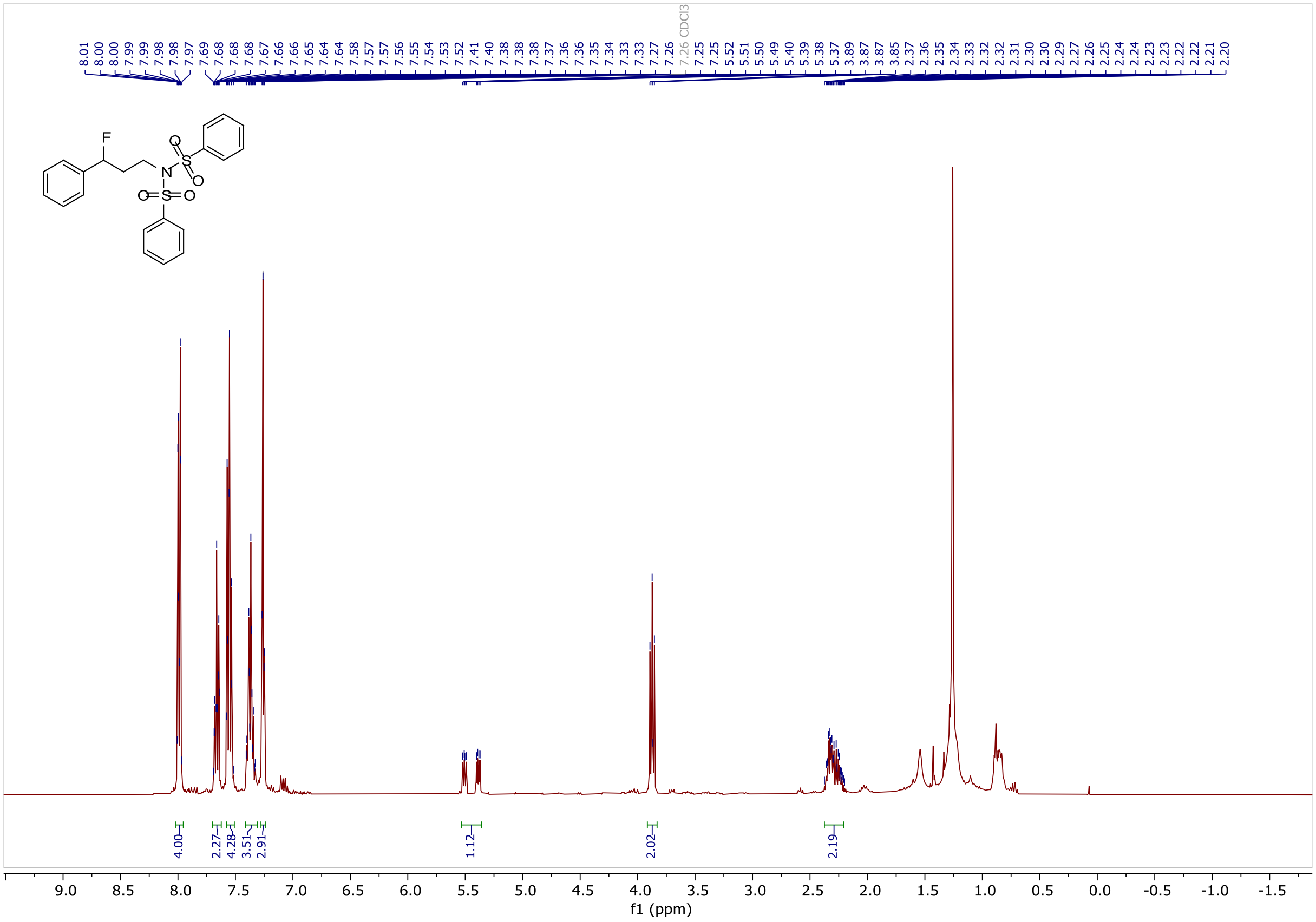

^{19}F NMR Spectrum of 28 (376 MHz, $CDCl_3$)

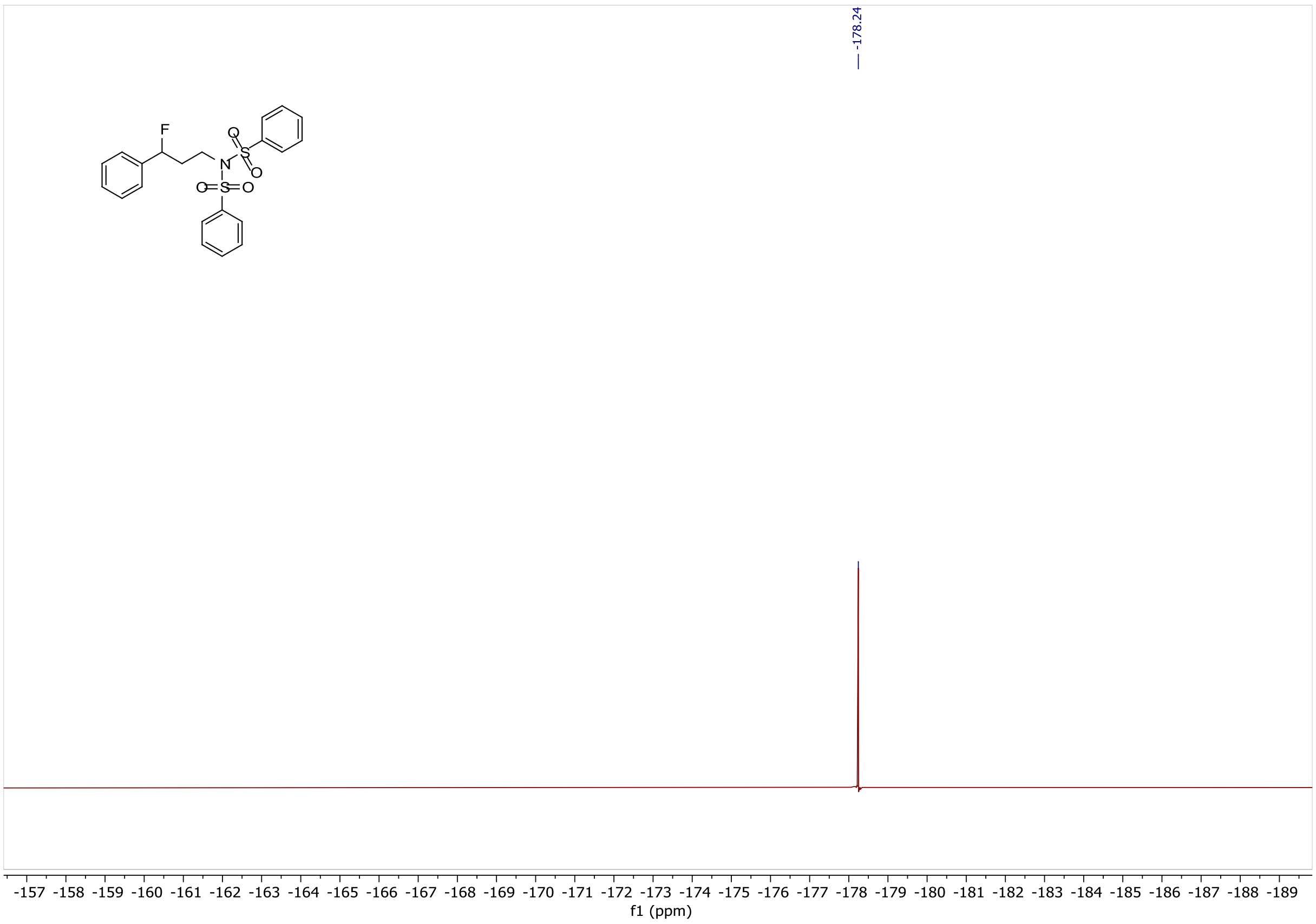

^{13}C NMR Spectrum of 28 (101 MHz, $CDCl_3$)

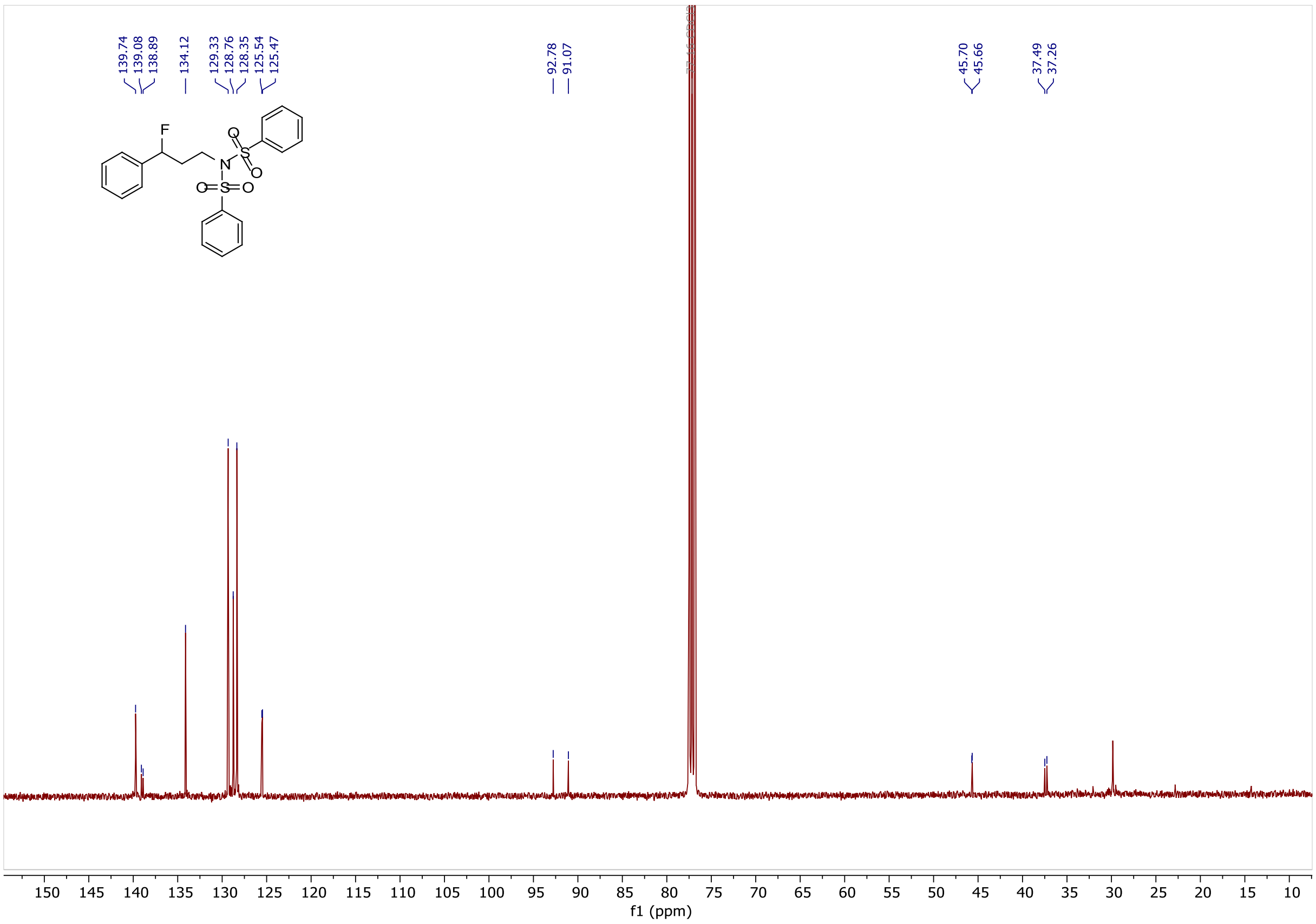

^{1}H NMR Spectrum of 29 (400 MHz, $CDCl_3$)

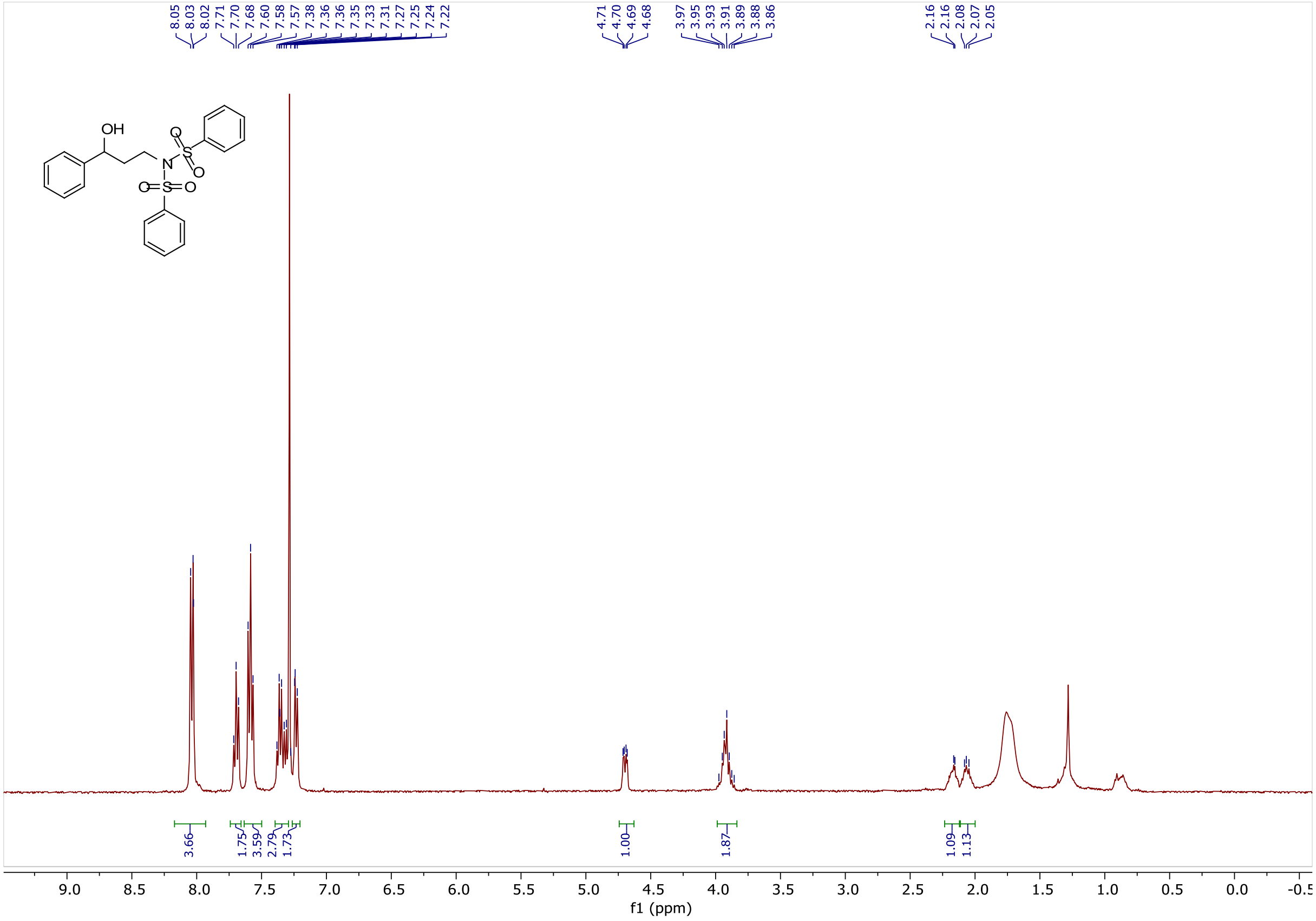

^{13}C NMR Spectrum of 29 (101 MHz, $CDCl_3$)

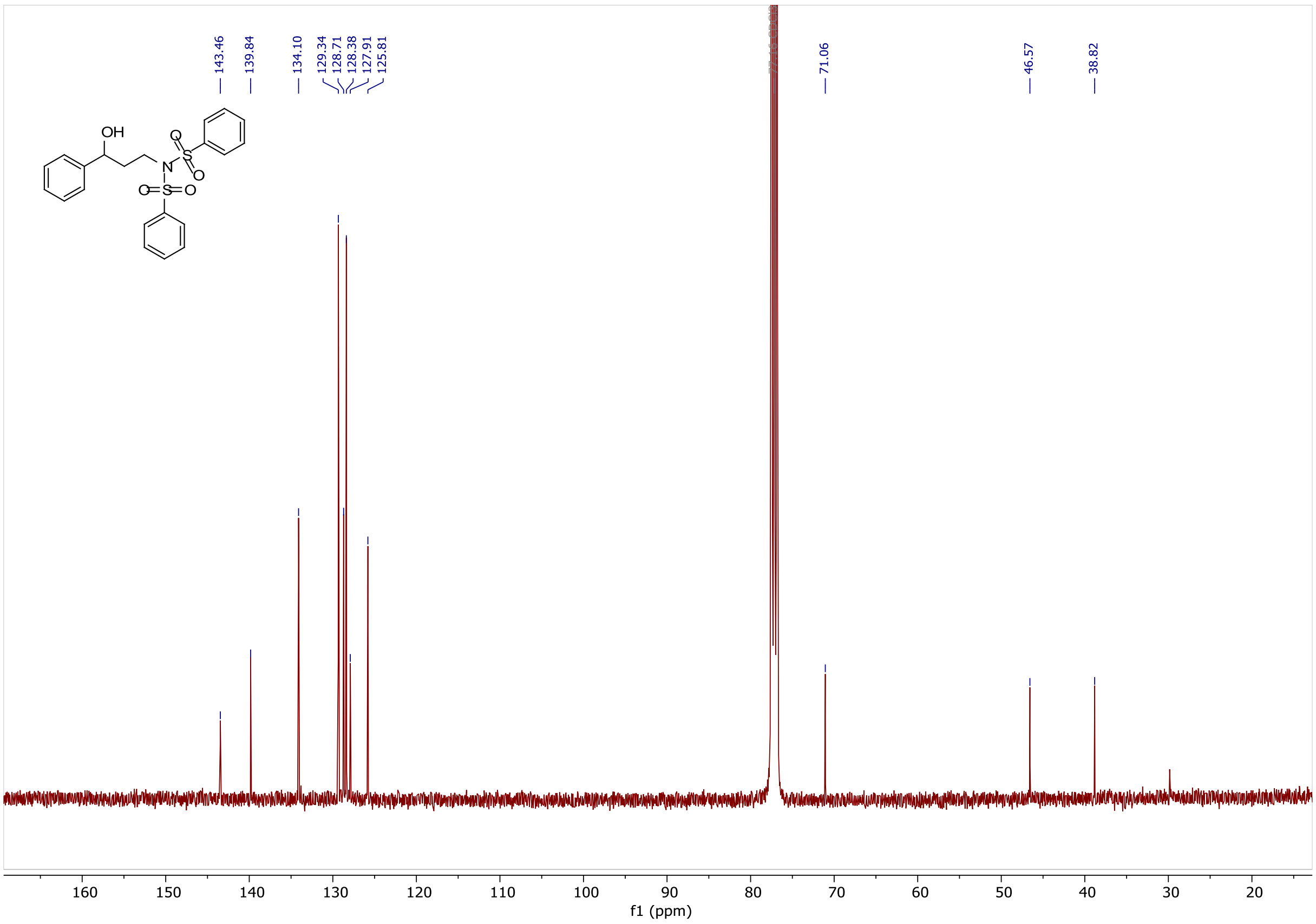

^{1}H NMR Spectrum of 30 (400 MHz, $CDCl_3$)

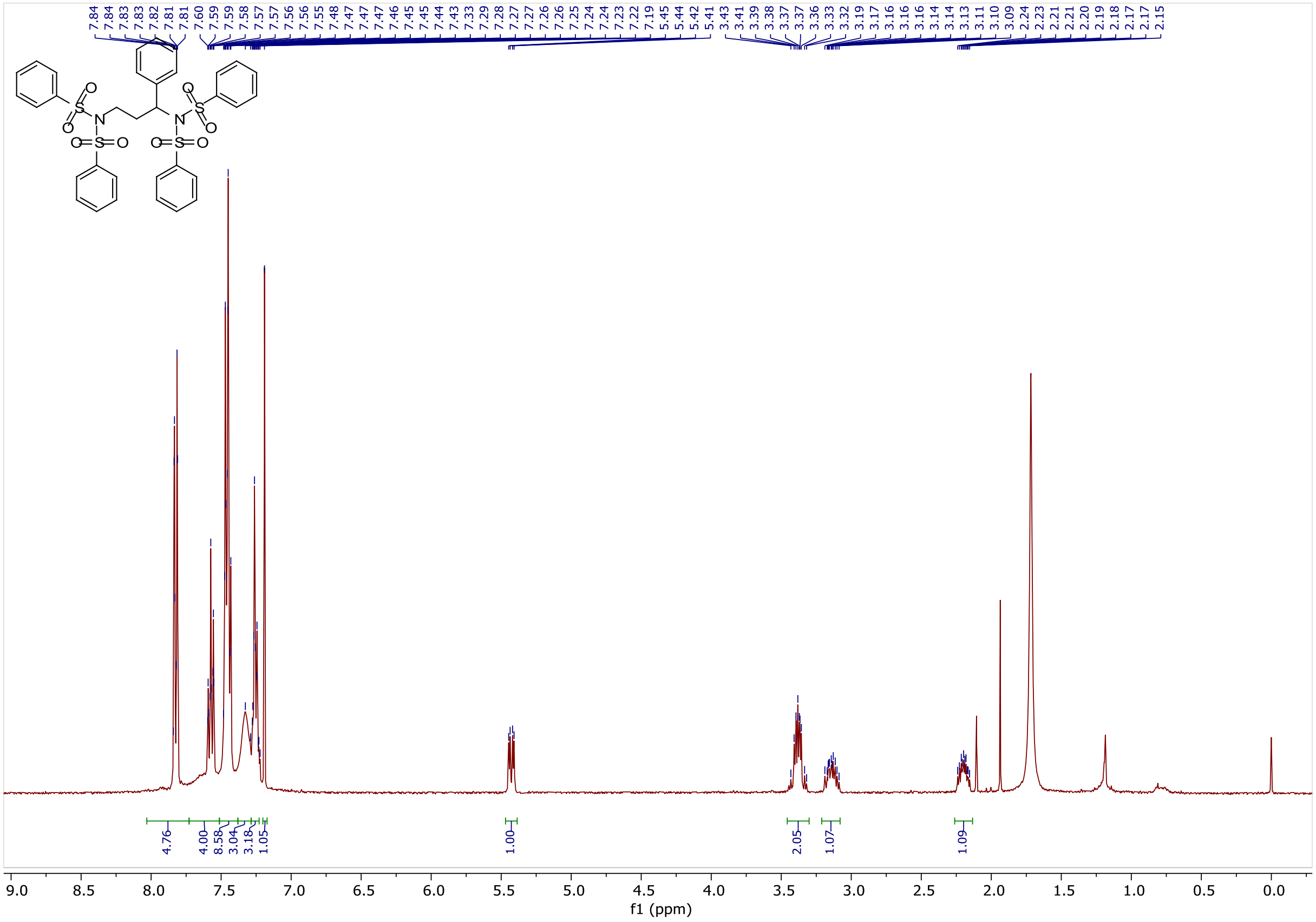

^{13}C NMR Spectrum of 30 (101 MHz, $CDCl_3$)

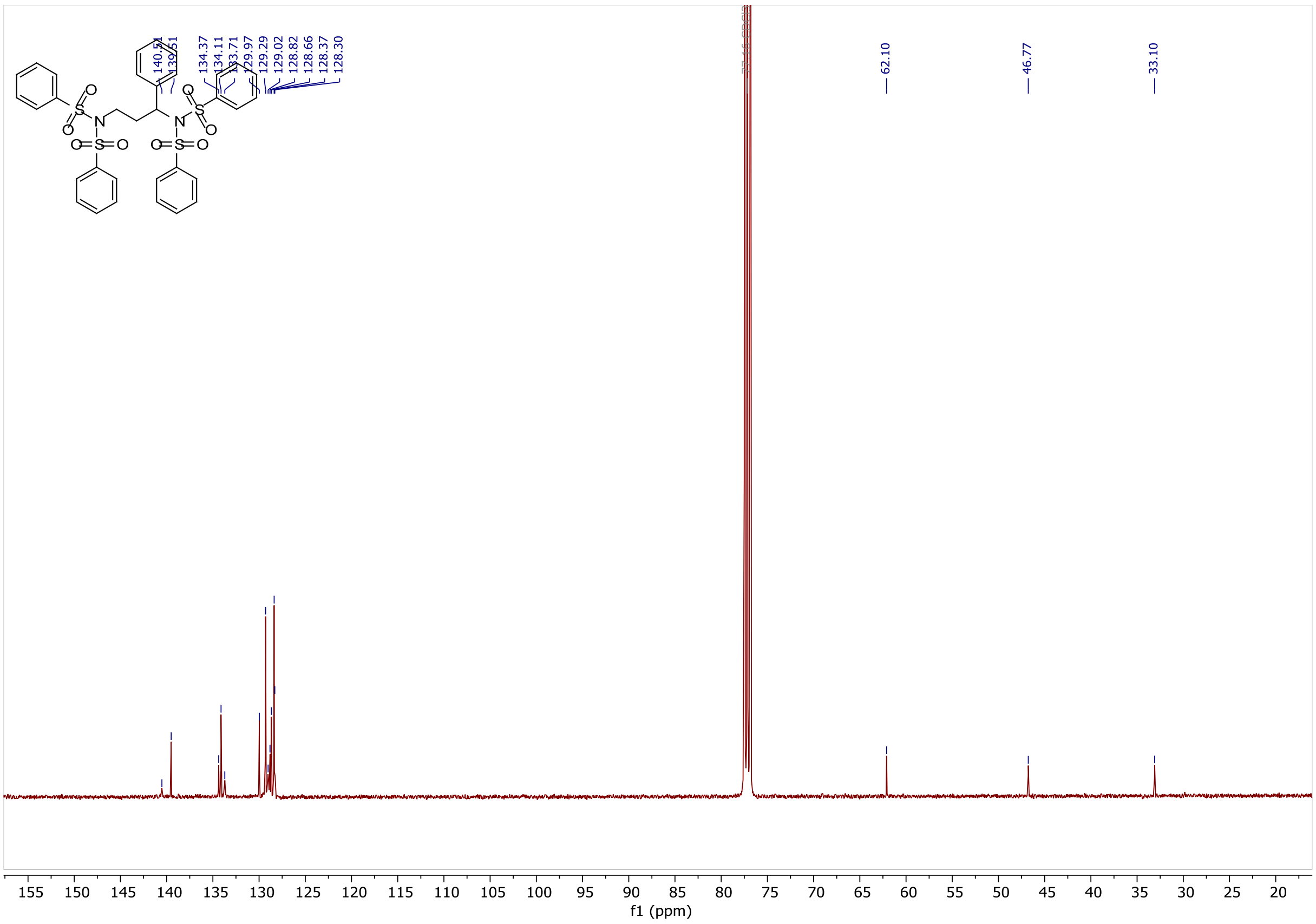

Supplement: Supplementary file 1 [file SC-OLF-D6SC06246F-s001.pdf]
